# Mechanism of the Aryl–F Bond-Forming Step from Bi(V) Fluorides

**DOI:** 10.1021/jacs.2c01072

**Published:** 2022-08-03

**Authors:** Oriol Planas, Vytautas Peciukenas, Markus Leutzsch, Nils Nöthling, Dimitrios A. Pantazis, Josep Cornella

**Affiliations:** Max-Planck-Institut für Kohlenforschung, Kaiser-Wilhelm-Platz 1, Mülheim an der Ruhr 45470, Germany

## Abstract

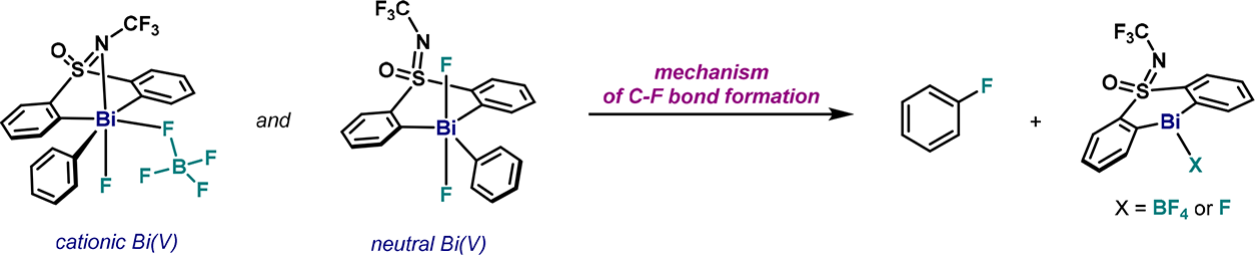

In this article, we describe a combined experimental and theoretical mechanistic investigation of the C(sp^2^)–F bond formation from neutral and cationic high-valent organobismuth(V) fluorides, featuring a dianionic bis-aryl sulfoximine ligand. An exhaustive assessment of the substitution pattern in the ligand, the sulfoximine, and the reactive aryl on neutral triarylbismuth(V) difluorides revealed that formation of dimeric structures in solution promotes facile Ar–F bond formation. Noteworthy, theoretical modeling of reductive elimination from neutral bismuth(V) difluorides agrees with the experimentally determined kinetic and thermodynamic parameters. Moreover, the addition of external fluoride sources leads to inactive octahedral anionic Bi(V) trifluoride salts, which decelerate reductive elimination. On the other hand, a parallel analysis for cationic bismuthonium fluorides revealed the crucial role of tetrafluoroborate anion as fluoride source. Both experimental and theoretical analyses conclude that C–F bond formation occurs through a low-energy five-membered transition-state pathway, where the F anion is delivered to a C(sp^2^) center, from a BF_4_ anion, reminiscent of the Balz–Schiemann reaction. The knowledge gathered throughout the investigation permitted a rational assessment of the key parameters of several ligands, identifying the simple sulfone-based ligand family as an improved system for the stoichiometric and catalytic fluorination of arylboronic acid derivatives.

## Introduction

The development of methodologies to forge C(sp^2^)–F bonds is of capital importance, as fluorine-containing molecules find applications as drugs,^1^ agrochemical products,^2^ organic materials,^3^ or [^18^F]Fluoride-labeled radiotracers for positron emission tomography (PET).^4^ In addition to the traditional nucleophilic aromatic substitution,^5^ fluorine has typically been anchored to aromatic substrates via the Balz–Schiemman reaction^6^ or through the Halex process.^7^ Despite numerous applications, these methods still suffer from harsh reaction conditions and a limited substrate scope. Attractive alternatives have recently emerged that facilitate C(sp^2^)–F bond formation under much milder reaction conditions which broaden the spectrum of compatible functional groups during C–F formation. These methods rely on the use of *d*-block elements, which have demonstrated to be excellent candidates for this purpose.^8^ Yet, metal-catalyzed C(sp^2^)–F bond-forming reactions are still arduous, due to the challenging reductive elimination from the small and highly electronegative fluoride anion. Therefore, the handful of examples reported successfully forged the C(sp^2^)–F bond mainly via high-valent metal centers or crafted ligands.

Early mechanistic studies by Grushin and Yandulov on late-transition-metal fluorides identified the main challenges to promote reductive elimination from Pd(II) centers.^9^ These seminal studies served as the stepping stone for the development of a groundbreaking nucleophilic fluorination process based on the Pd(0)/Pd(II) redox couple by Buchwald.^10^ In order to understand the challenges associated with the C(sp^2^)–F bond-forming step, the isolation and study of high-valent intermediates has been shown to serve as a valid strategy. In this context, Ritter reported the C(sp^2^)–F reductive elimination from well-defined σ-aryl Pd(IV)–F species.^11^ This work, together with subsequent studies from Sanford,^12^ revealed key structural features to guide the development of Pd-based fluorination methods, including relevant works on C(sp^2^)–H and C(sp^2^)–B functionalization.^13^ In addition to Pd, mechanistic investigations of σ-aryl Pt(IV)–F compounds by Gagné,^14^ Vigalok and Vedernikov,^15^ and Haghighi^16^ identified pathways to achieve smooth C(sp^2^)–F formation, overcoming unproductive side reactions. With the focus on more earth-abundant elements, aryl–F reductive elimination from Ni centers has been recently established. Ritter^17^ and Sanford,^18^ who independently identified C(sp^2^)–F reductive elimination pathways occurring from σ-aryl Ni(III)–F and Ni(IV)–F species, respectively, showed that aryl fluoride formation is feasible from different oxidation states. Along with group 10 metals, coinage metals have also shown promising results for both nucleophilic and electrophilic fluorination.^19^ Mechanistic studies on σ-aryl Cu–F complexes by Ribas demonstrated the intermediacy of Cu(III)–F species, which were further suggested by Hartwig.^19a,19d^ Silver has also received significant attention in electrophilic fluorination.^20^ Clues about its mode of action where reported by Ribas, who described σ-aryl Ag(III) species endowed with the ability to engage in C(sp^2^)–F bond-forming events via a putative aryl–Ag(III)–F intermediate.^20f^ Nevertheless, bimetallic Ag(II)–Ag(II) species have also been proposed to mediate aryl–F bond formation via one-electron participation of two Ag atoms, both in stoichiometric and catalytic fashion.^20b,20c^

**Figure 1:**
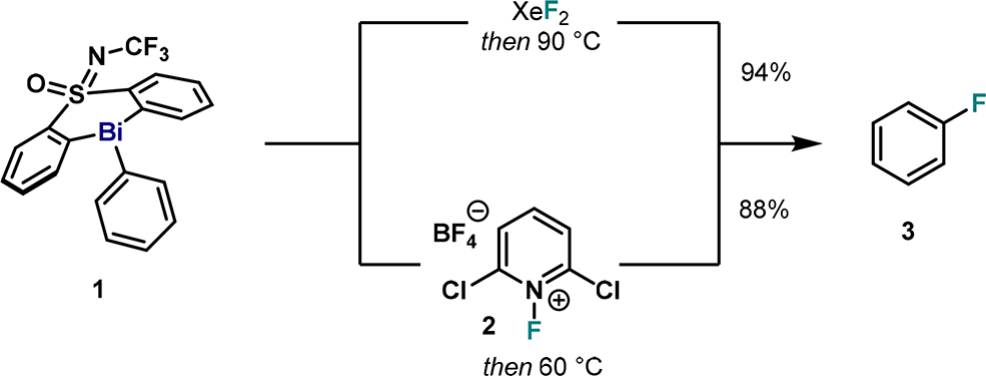
Fluorination protocol from **1** via an oxidation/C(sp^2^)–F bond formation sequence.

Beyond the need to find alternative solutions to the imminent threat posed by the availability of noble metals, it is desirable to explore unchartered territories beyond the *d*-block to seek new reactivity. Accordingly, certain main group elements have recently been identified as potential candidates to mimic organometallic transformations.^21^ In the context of aryl–F bond formation, however, only a privileged selection has been demonstrated to satisfactorily forge C–F bonds. In the early 1980s, Van Der Puy unveiled σ-aryl hypervalent iodine(III) compounds as powerful reagents to readily obtain fluoroarenes,^22^ and subsequent mechanistic studies were key to introduce [^18^F]Fluoride into organic molecules.^23^ Recent examples of aryl–F bond formation have also been reported among chalcogens by the use of sulfonium salts,^24^ which are proposed to undergo reductive elimination from hypervalent sulfurane intermediates.^25^ Thermal decomposition of certain organolead^26^ and organothallium^27^ compounds has also been shown to forge the corresponding C–F bond. Comparatively, a heavy element that received much less attention is bismuth (Bi).^28^ While simple halogen-containing Ph_3_Bi(V)X_2_ (X = Cl, Br, I) compounds can thermally decompose to forge aryl–X bonds, analogous studies using F as anion resulted in traces of Ar–F.^29^ In an isolated example, Akiba reported that aryl–F bond formation is feasible from octahedral Bi(V) difluorides;^30^ yet, no additional information on this particular step was reported. Inspired by these promising precedents, together with the well-known benign properties associated with Bi,^31^ we started a research program capitalizing on the organometallic properties of high- and low-valent Bi complexes, both in redox and nonredox catalysis.^32^ Inspired by the sulfone-based bismacyclic scaffolds by Suzuki,^33^ our group reported sulfoximine-based Bi compounds capable of forging C(sp^2^)–F bond formation.^32b^ Specifically, we provided conditions for the *stoichiometric* and *catalytic oxidative fluorination of arylboronic acid derivatives in a redox process*. In the former, aryl fluoride is formed upon oxidation of **1** with XeF_2_ and subsequent thermal decomposition at 90 °C (Figure 1). In the latter, 1-fluoro-2,6-dichloropyridinium tetrafluoroborate (**2**) acts as the sole oxidant to access a Bi(V) intermediate, which rapidly delivers fluorobenzene (**3**). Preliminary stoichiometric investigations led us to propose cationic σ-aryl Bi(V)–F intermediates; however, the genuine structure of the species promoting reductive elimination, together with the effect of exogenous additives such as fluoride, remained mysterious and needed further evaluation.

Herein, we report a mechanistic study aimed at providing a detailed analysis of the aryl–F reductive elimination event from σ-aryl Bi(V) fluoride species. To do so, we assess the role of electronic and geometric perturbations on the ligand scaffold in **1**, as well as on the pendant aryl moiety, with the aim of identifying the steric and electronic factors that govern the aryl–F bond formation. Theoretical investigations, kinetic studies, and an in-depth scrutiny of solvent effects and additives allowed us to fully identify the species involved in the aryl–F bond-forming event from neutral and cationic complexes. The outcome of this analysis led us to design a second generation of bismuth complexes that permit both stoichiometric and catalytic aryl–F bond formation with a wider substrate scope and milder reaction conditions.

## Results

### Solid-State Analysis of Bi(V) Difluoride **4**

**Figure 2:**
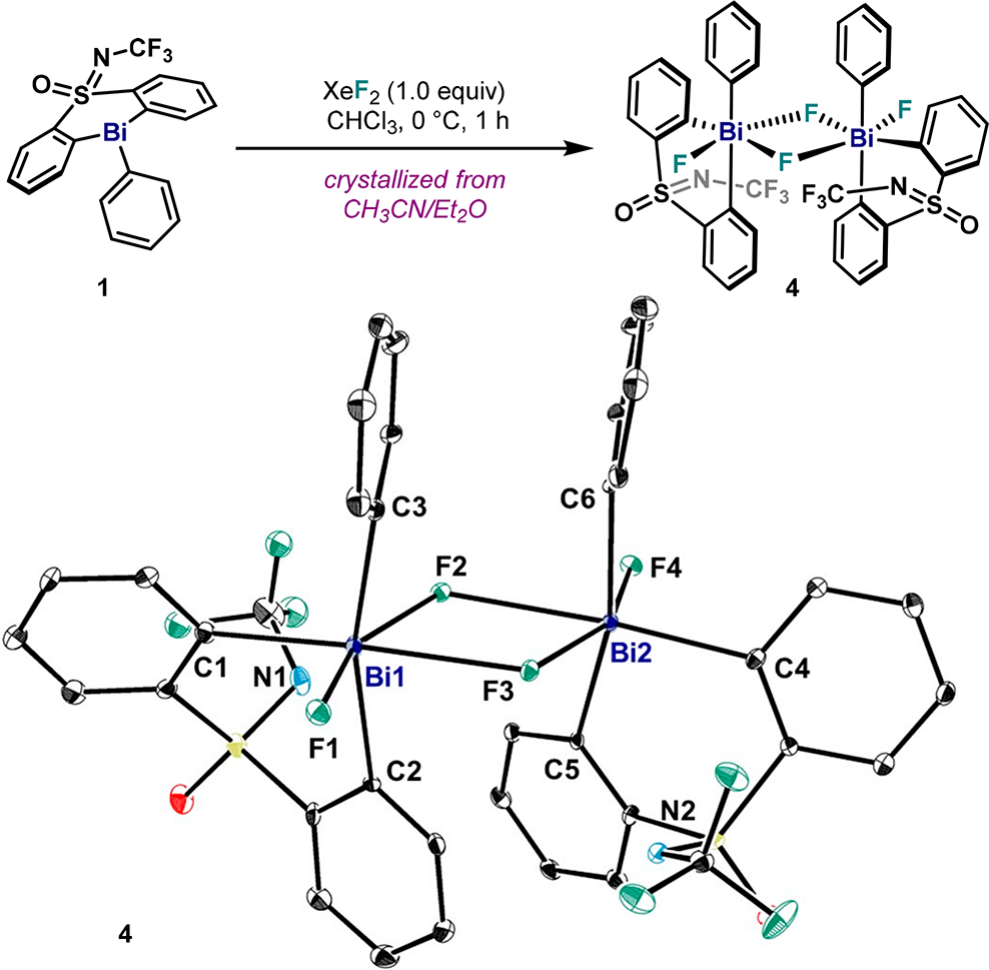
Synthesis of pentavalent complex **4** and XRD structure analysis. Hydrogen atoms, disordered parts, and solvent molecules omitted for clarity.

At the onset of our investigations, we focused on the structural characterization of pentavalent σ-aryl Bi(V) difluoride **4** (Figure 2), which served as a model complex during this study. When **1** is oxidized with 1.0 equiv of XeF_2_ in CHCl_3_ at 0 °C, a white solid corresponding to **4** is obtained after evaporation of the volatiles. Cooling a concentrated solution in MeCN at 4 °C led to suitable single-crystals to be analyzed by X-ray diffraction (XRD). As shown in Figure 2, **4** presents a quasi-symmetric dimeric structure with both Bi centers in oxidation state +5. Each Bi atom in **4** adopts a distorted octahedral geometry, with two fluorine atoms positioned *trans* to each other (F1–Bi1–F2, 157.40(13) ° and F3–Bi2–F4, 157.32(13) °). Interestingly, the pendant phenyl substituents are located *syn* to each other in close proximity (centroid–centroid distance of 3.766 Å), suggesting π–π attractive interactions. Additionally, one of the F atoms is shared with the other monomer, thus forming a four-membered ring with a μ-difluoro diamond-like core. The shared F atoms do not have equal distances to both Bi atoms (Bi1–F3, 2.585(3) Å and Bi2–F2, 2.582(3) Å are much longer compared to Bi1–F2, 2.185(3) Å and Bi2–F3, 2.178(3) Å). Importantly, the N atoms of the NCF_3_ moiety are in close proximity to the Bi centers (Bi1–N1, 3.535(6) Å and Bi2–N2, 3.665(4) Å). Overall, the solid-state structure of **4** resembles the dimer previously reported featuring a SO_2_ motif in place of the S(O)NCF_3_ in the ligand backbone;^32b^ yet, the distance between Bi–N is ca. 0.3 Å longer than Bi-O distance of the sulfone.

### Solution-State Analysis of Bi(V) Difluoride **4**

**Figure 3:**
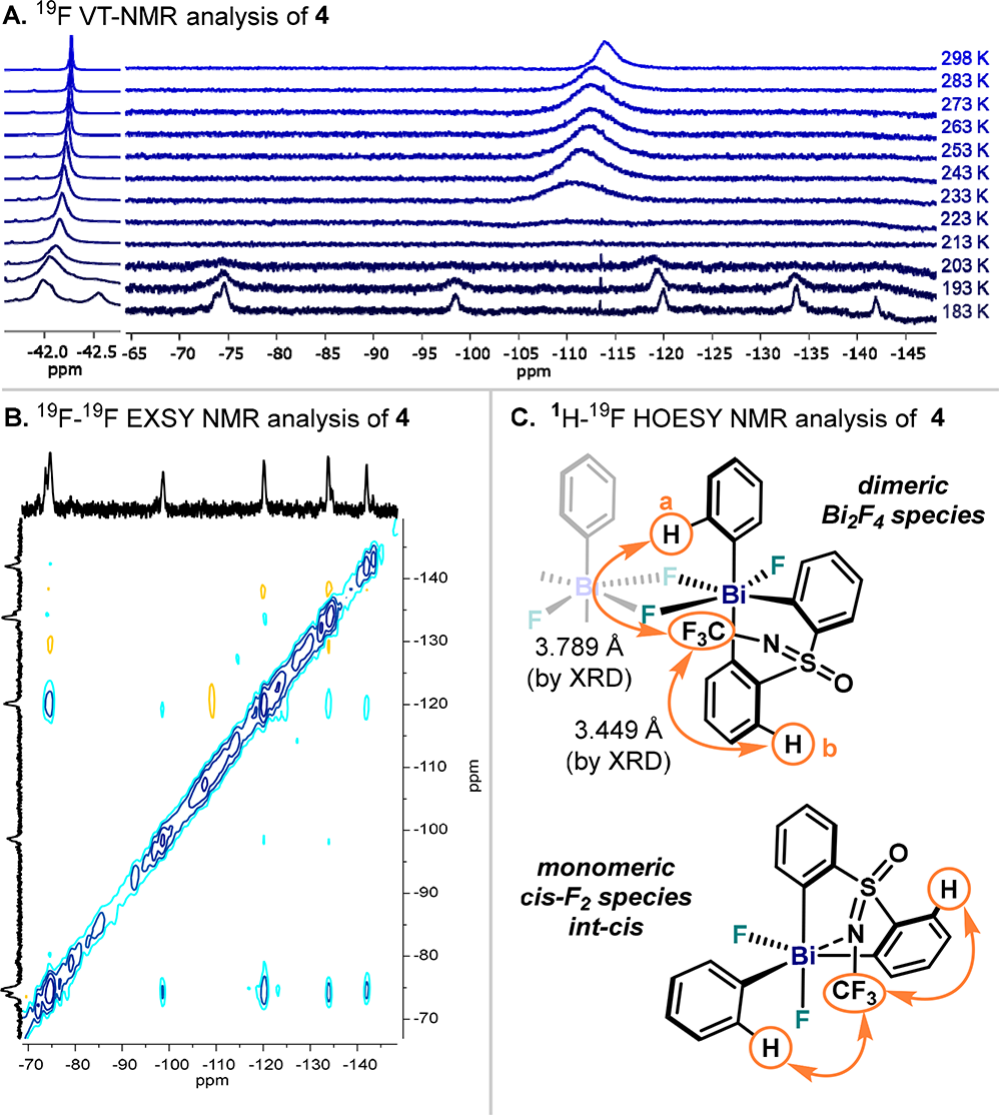
(A) VT ^19^F NMR measurements of **4** in CD_2_Cl_2_. (B) ^19^F–^19^F EXSY NMR spectrum at 183 K in CD_2_Cl_2_ showing chemical exchange. (C) ^1^H–^19^F HOESY cross peaks of **4** in CD_2_Cl_2_ at 298 K.

In our previous study, **4** was postulated to be a monomer in solution. To obtain more information, variable-temperature (VT) NMR was performed in CD_2_Cl_2_ (Figure 3A). At 298 K, ^1^H NMR measurements reveal **4** as a symmetric compound: both aryl groups of the sulfoximine are equivalent.^34^ Cooling down the sample to 183 K results in a significant broadening of all signals, which complicated interpretation. Similar results were obtained using other solvents, such as CD_3_CN at 233 K.^34^ The ^19^F NMR spectrum at 298 K (Figure 3A) showed a broad singlet at −112 ppm corresponding to the Bi–F unit. Interestingly, cooling the solution to 183 K caused the appearance of several broad signals at the region of δ = −70 to – 140 ppm as well as a new poorly defined peak around δ = −42.5 ppm, the region corresponding to the NCF_3_ unit. In order to discard the possibility of solubility issues when cooling down a solution of **4**, an analogous Bi(V) compound bearing ^*t*^Bu groups in the ligand scaffold (**5**) was synthesized and analyzed by VT-NMR.^34^ Indeed a parallel behavior was observed for **5**, which at 183 K also shows multiple species in solution. In addition, dilution experiments of **4** and **5** show peak broadening and movement at room temperature, pointing towards aggregation in solution. Additionally, ^19^F–^19^F COSY and EXSY NMR experiments of a solution of **4** in CD_2_Cl_2_ at 183 K (Figure 3B) unambiguously point at a chemical exchange between all F atoms (except CF_3_). These results manifest a complex dynamic behavior, showing a variety of species in solution undergoing rapid F exchange even at very low temperatures. Unfortunately, characterization of complex **4** at low temperature proved extremely difficult due to broad bands, partial precipitation, and low concentration of different species. However, warming the mixture to 298 K allowed the measurement of ^1^H–^19^F through-space interactions via HOESY NMR (Figure 3C). ^1^H–^19^F contacts between the NCF_3_ moiety with the *ortho*-H in the pendant aryl (H_(a)_, Figure 3C) and the *meta*-H in the ligand scaffold (in respect to Bi, H_(b)_, Figure 3C) were observed. This latter result is consistent with a dimeric species in solution such as the crystal structure of **4** in Figure 2. Yet, a monomeric *cis*-difluoride complex (***int-cis***) is predicted to possess similar spectroscopic features. As a result of such fast dynamic configurational processes, dimeric and monomeric species are proposed to coexist at higher temperatures, averaging the signals in ^1^H and ^19^F NMR and subsequently posing a severe challenge to identify the species responsible for aryl–F bond formation.

### Reductive Elimination from Pentavalent Bi(V) Difluoride **4**

**Figure 4:**
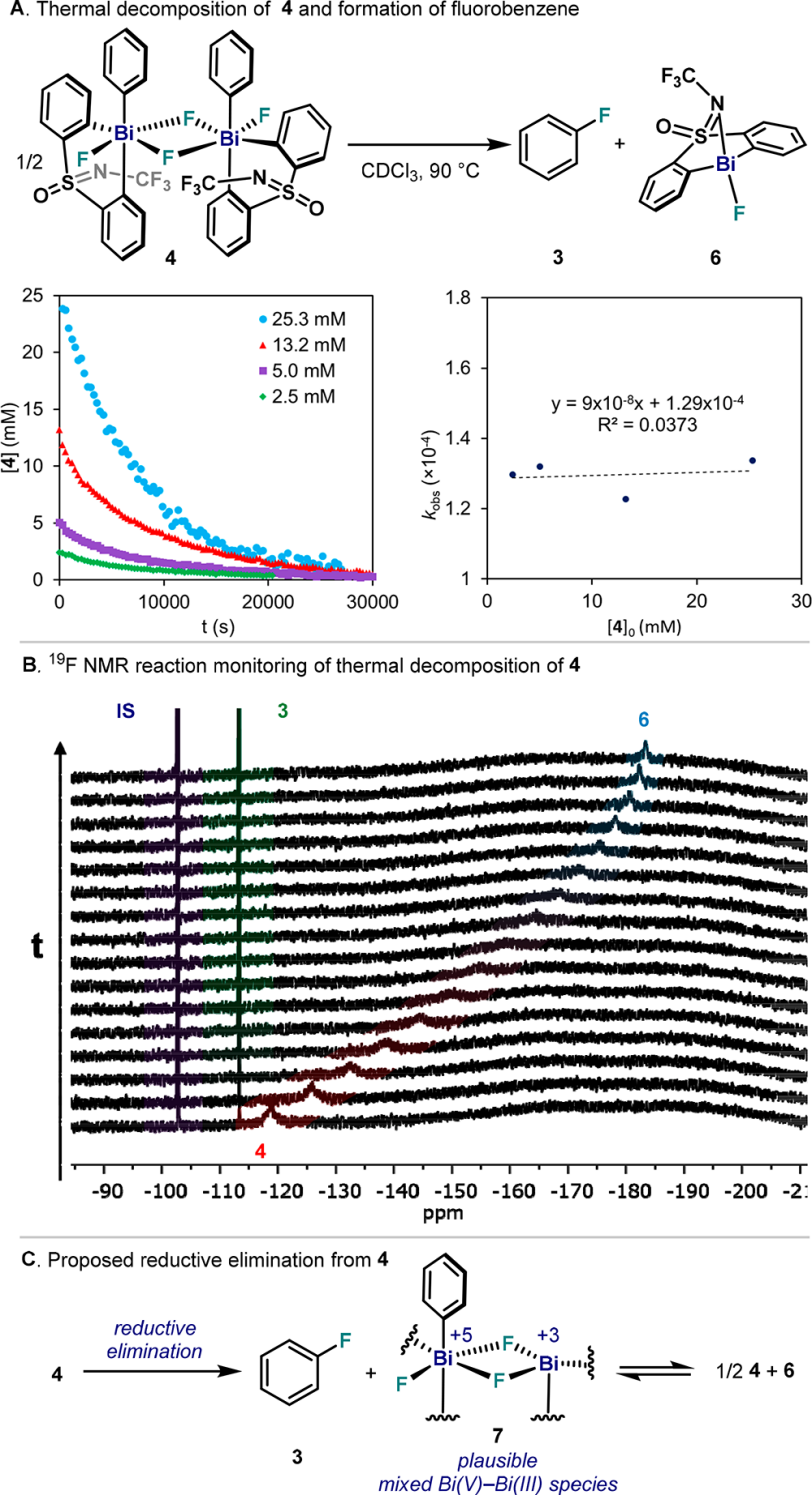
(A) Left, reaction profile of reductive elimination from **4** over a range of concentrations. Right, plot of *k*_obs_ vs [**4**]_0_. (B) ^19^F NMR reaction monitoring of reductive elimination from **4** (red) with 1-fluoro-4-nitrobenzene (purple) as internal standard, showing formation of fluorobenzene (**3**, green) and **6** (blue). *t* = time. (C) Putative mixed-valent Bi(V)–Bi(III) species after C–F formation from **4**.

First, thermal decomposition at 70 °C of **4** was attempted in solvents with diverse dielectric constants (ε), and reactions were monitored by ^19^F and ^1^H NMR spectroscopy. Interestingly, similar rates were observed in CDCl_3_ (ε = 4.8, *k*_obs_ = 3.09 ± 0.02 × 10^–5^ s^–1^), CD_2_Cl_2_ (ε = 8.9, *k*_obs_ = 3.05 ± 0.05 × 10^–5^ s^–1^), and CD_3_CN (ε = 37.5, *k*_obs_ = 3.33 ± 0.02 × 10^–5^ s^–1^, pointing to a nonionic pathway. Further mechanistic information was obtained from the study of thermal decay from species **4**, which was previously shown to follow first order kinetics.^32b^ This result was further validated when reactions over a range of concentrations showed an unchanged rate constant (*k*_obs_ ≈ 1.3 × 10^–4^ s^–1^, Figure 4A), indicating a unimolecular phenyl–F bond-forming event that is first order in **4**. Collectively, these data suggest that C–F bond formation proceeds from **4**, after rapid pre-equilibrium with monomeric species (*cis* and *trans*). Further information was obtained when the reductive elimination was monitored by ^19^F NMR (Figure 4B). Strikingly, the broad singlet at δ = −118 ppm corresponding to the Bi(V)–F_2_ unit in **4** did not fade away simultaneously with the appearance of a peak at δ = −182 ppm, which corresponds to Bi(III)–F byproduct **6**. Instead, the Bi(V)–F_2_ NMR signal gradually shifts toward the Bi(III)–F unit (**6**), indicating a fast exchange between fluorides from Bi(V) and Bi(III) species. Similar results were obtained when mixtures of **4** and **6** were analyzed by NMR, showing unchanged ^1^H NMR spectra but different ^19^F signals depending on the concentration of the components.^34^ Thus, reductive elimination from dimeric species **4** produces fluorobenzene together with the corresponding Bi(III)–F complex **6** and a monomeric Bi(V) species (Figure 4C). These compounds, which are released in close proximity, presumably exchange fluoride ligands in a mixed Bi(V)–Bi(III) complex such as **7**. This mixed-valence bimetallic compound is proposed to undergo complex downstream equilibria that will eventually lead back to **4**.

### Effect of Substitution on the Pendant Aryl Ring in the C–F Bond Formation from **4**

**Figure 5:**
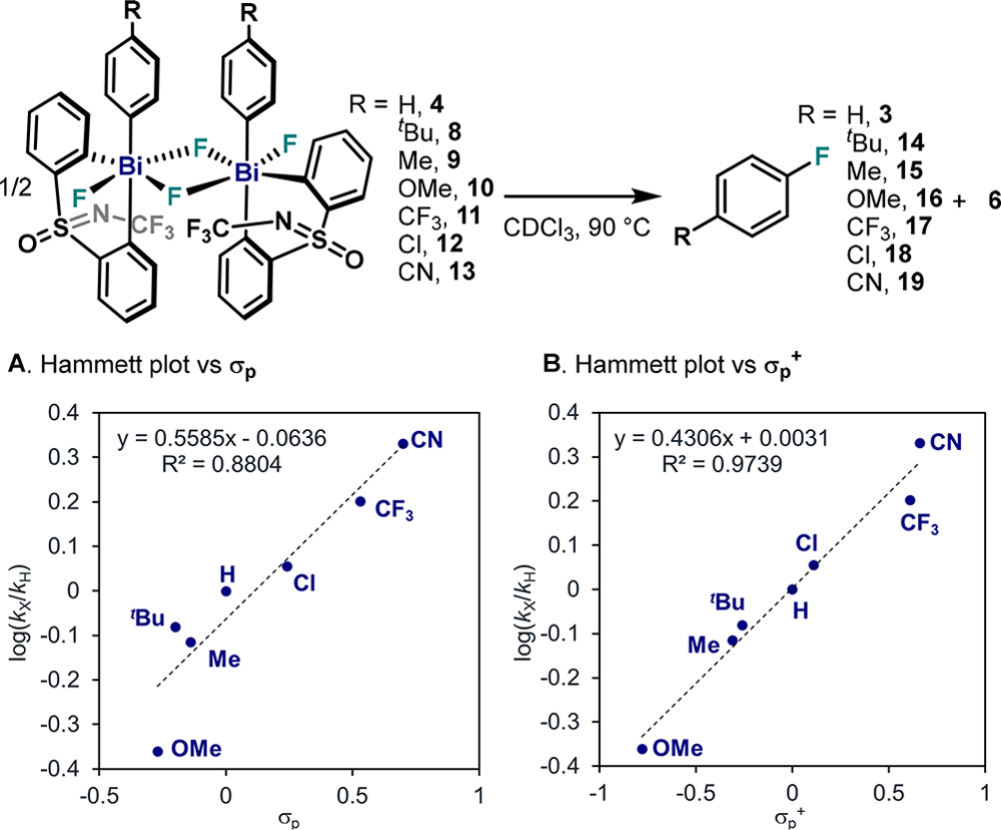
Electronic analysis of reductive elimination from **4**–**13**. (A) Hammett plot vs σ_p_.values. (B) Hammett plot vs σ_p_^+^ values.

In our previous study, we showed that the presence of electron-withdrawing groups at the *para*-position of the σ-aryl Bi(V) difluorides accelerates C–F bond formation.^32b^ This tendency was explored further by including additional *para*-substituted complexes (Figure 5). Deviation of linearity (*R*^2^ = 0.88) with standard σ_p_ parameters is mainly caused by *p*-OMe substituted complex **10** (Figure 5A), indicating the great influence of strong π-donating groups. As shown in Figure 5B, better linearity (*R*^2^ = 0.97) is obtained when including resonance effects using σ_p_^+^ values. These results indicate that there exists a buildup of negative charge around the C_ipso_ atom in the transition state (TS) compared to the ground state, consistent with the nucleophilic attack by a fluoride in the rate-determining step. These data can be interpreted as the C_ipso_ acting as an electrophile in the TS. When electronic effects at the *meta* position were evaluated, linearity became nonobvious when analyzed with various methods and using several Hammett parameters.^34^ However, a trend could be observed when comparing sterical bulkiness: Large substituents accelerate the reductive elimination. However, a model that accommodates all the observations could not be established and is currently under investigation.

It is important to mention that Bi(V) difluoride compounds bearing an *ortho* substituent were shown to undergo extremely fast reductive elimination. For example, a Bi(V) complex with a pending *o*-tolyl group (**20**) underwent fluorination ca. 22 times faster than model complex **4**.^34^ Larger groups at *ortho*-position such as −Et (**21**) or −^*i*^Pr (**22**) resulted in instantaneous reductive elimination at 90 °C, and formation of the corresponding fluoroarene was observed even at 25 °C. Although **20**–**22** could be characterized at low temperature by NMR, structural information through XRD or solution-state NMR was prevented by their intrinsic high instability. However, we believe that in **20**–**22**, the Bi–C_aryl_ distance in pentavalent Bi species bearing *ortho* substitution becomes larger compared to **4**, thus leading to a more electrophilic C center, a weaker C–Bi bond and a subsequently faster reductive elimination.

### Solid-State Analysis of Sterically Hindered Bi(V) Difluorides **25** and **26**

**Figure 6:**
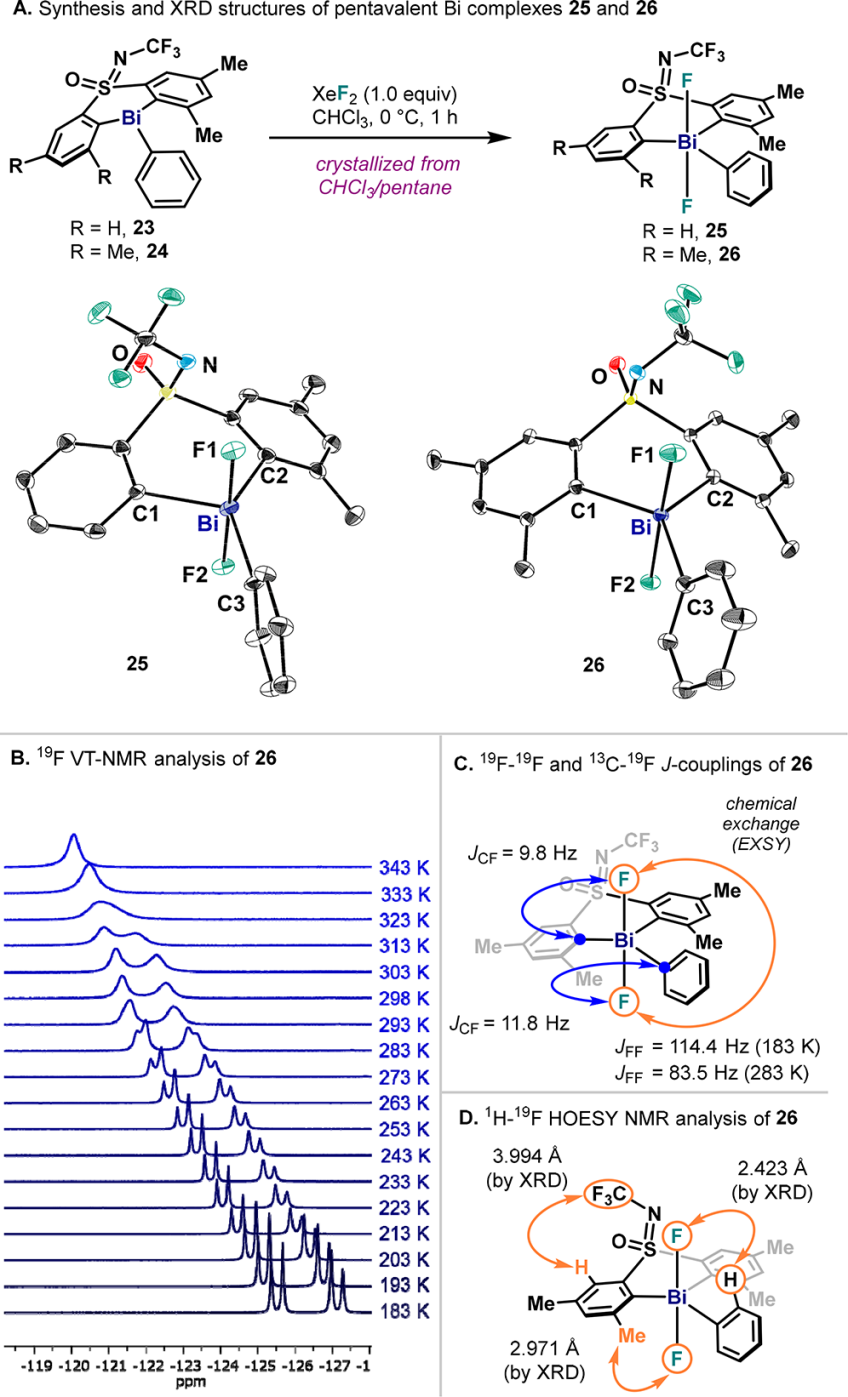
(A) Synthesis of monomeric pentavalent bismine fluoride complex **25** and **26** and XRD structure analysis. Hydrogen atoms and solvent molecules omitted for clarity. (B) VT ^19^F NMR measurements of **26** in CD_2_Cl_2_. (C) ^19^F–^19^F and ^13^C–^19^F *J*-coupling constants of **26** in CD_2_Cl_2_ and CDCl_3_ respectively, together with *J*_FF_ constants at different temperatures. (D) ^1^H–^19^F HOESY measurements of **26** in CD_2_Cl_2_. For simplicity, orange and blue arrows are not used to show all C–F and H–F interactions, but only to represent the distinct ones.

After an exhaustive assessment of several parameters influencing reductive elimination from model species **4**, our efforts focused on the study of σ-aryl Bi(V) complexes presenting steric congestion on the ligand backbone. As we demonstrated earlier,^32b^ the introduction of Me groups at the *ortho* position with respect to the Bi center allowed the synthesis of distorted trigonal bipyramidal (TBP) monomeric Bi(V) difluoride complex **25** upon oxidation of **23** with XeF_2_ at 0 °C, which was possible to characterize by XRD after it was crystallized from CHCl_3_/pentane mixture (Figure 6A, left). To provide additional evidence on the influence of the Me groups in the structure of the Bi(V) center, complex **26** bearing two additional Me groups was also synthesized and characterized by XRD (Figure 6A, right). This complex also presents a distorted trigonal bipyramidal geometry with similar structural features to **25**; in this case, however, the Bi center is flanked by two Me groups in both sides of the sulfoximine ligand.

### Solution-State Analysis of Sterically Hindered Bi(V) Difluorides **25** and **26**

Due to its highly symmetric structure and simplified spectroscopic features, complex **26** was analyzed in solution. VT ^19^F NMR of **26** in CD_2_Cl_2_ revealed a broad singlet with a chemical shift of δ = −120.1 ppm at 343 K, corresponding to the Bi–F_2_ unit. However, measurements at 183 K resulted in a separate set of two doublets with a chemical shift of δ = −125.6 ppm and a *J*_FF_ = 114.4 Hz, with a coalescence temperature of *T*_c_ = 323 K (Figure 6B). The appearance of these doublets with a *J*_FF_ = 114.4 Hz indicates two nonequivalent fluoride ligands, indicating a *trans*-difluoride Bi(V) configuration in solution. Indeed, comparable chemical shift values and coupling constants were described for previously reported triaryl-Sb(V) and triaryl-Bi(V) *trans*-difluoride complexes.^30,35^ Analogous splitting was observed for the previously reported complex **25**, with a *J*_FF_ = 115.1 Hz; in this case, the coalescence temperature of the doublets was significantly lower, with a value of *T*_c_ = 233 K.^34^ The reduced *J*_FF_ value of **26** at higher temperatures (*J*_FF_ = 112.1 Hz at 253 K; *J*_FF_ = 102.1 Hz at 273 K; *J*_FF_ = 83.5 Hz at 283 K) suggests a higher contribution of the *cis*-conformer to the NMR signal, where the time-averaged F–F angle is reduced. The coalescence of the Bi–F signals at temperatures above 323 K is a result of a chemical exchange, probably due to fast F–F interconversion through rotation processes such as Berry pseudorotation and turnstile rotation with the intermediacy of *cis*-difluoride species.^36^ Analysis by ^19^F–^19^F EXSY NMR of **26** in CD_2_Cl_2_ showed significant exchange between both F atoms even at 223 K (Figure 6C), similarly to complex **4**. Further confirmation of the *trans*-difluoride disposition was obtained by ^1^H–^19^F HOESY measurements in CD_2_Cl_2_ at 223 K (Figure 6D), which showed through-space H–F contacts consistent with this configuration in solution.^34^ Interestingly, dilution experiments of **25** and **26** showed no significant change in chemical shifts and peak broadening at room temperature, suggesting no aggregation in solution. Altogether, these results indicate that complexes **25** and **26** preserve the TBP geometry in solution with a *trans-*difluoride configuration. Installation of steric hindrance in the ligand certainly avoids dimerization and favors *trans*-difluoride monomers; yet fast F–F exchange still occurs even at low temperatures, thus highlighting the talent of pentavalent Bi complexes to undergo a collection of dynamic processes.

### Reductive Elimination from Sterically Hindered Pentavalent Bi(V) Difluorides **25** and **26**

**Figure 7:**
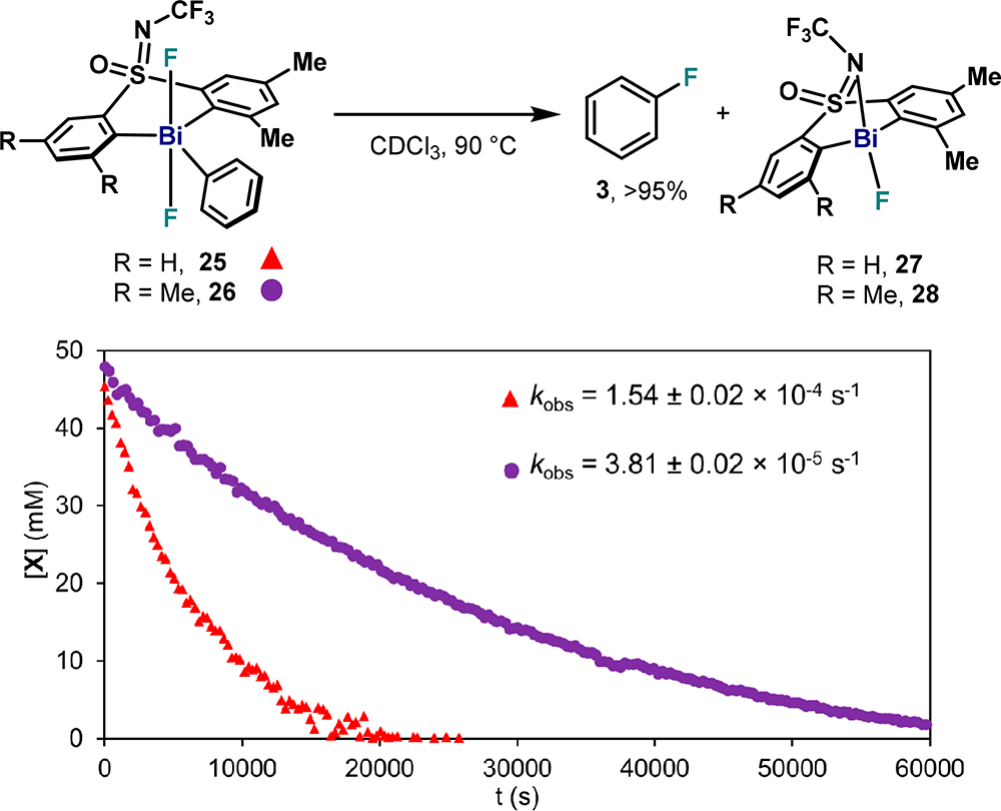
Reaction profile of reductive elimination from **25** (red) and **26** (purple) measured by ^19^F NMR with 1-fluoro-4-nitrobenzene as internal standard.

Complexes **25** and **26** were also subjected to thermal decomposition at 90 °C in CDCl_3_, and their kinetic profiles were measured (Figure 7). C(sp^2^)–F bond formation from **25** resulted in nearly identical kinetic profiles compared to **4** (*k*_obs_ = 1.54 ± 0.02 × 10^–4^ s^–1^), while sterically more crowded complex **26** showed a slower decay (*k*_obs_ = 3.81 ± 0.02 × 10^–5^ s^–1^) and formation of fluorobenzene (*k*_obs_ = 2.93 ± 0.02 × 10^–5^ s^–1^).^34^ Contrarily to **4**, ^19^F NMR revealed a clean conversion of **25** and **26** to the corresponding Bi(III)–F (**27** and **28**, respectively) without a gradual shift of the Bi–F signal.^34^ This behavior suggests no F–F exchange processes during aryl–F reductive elimination, presumably proceeding from the monomeric Bi(V).

### Effect of the Substitution on the Pendant Aryl Ring in the C(sp^2^)– – F Bond Formation from **25**

**Figure 8:**
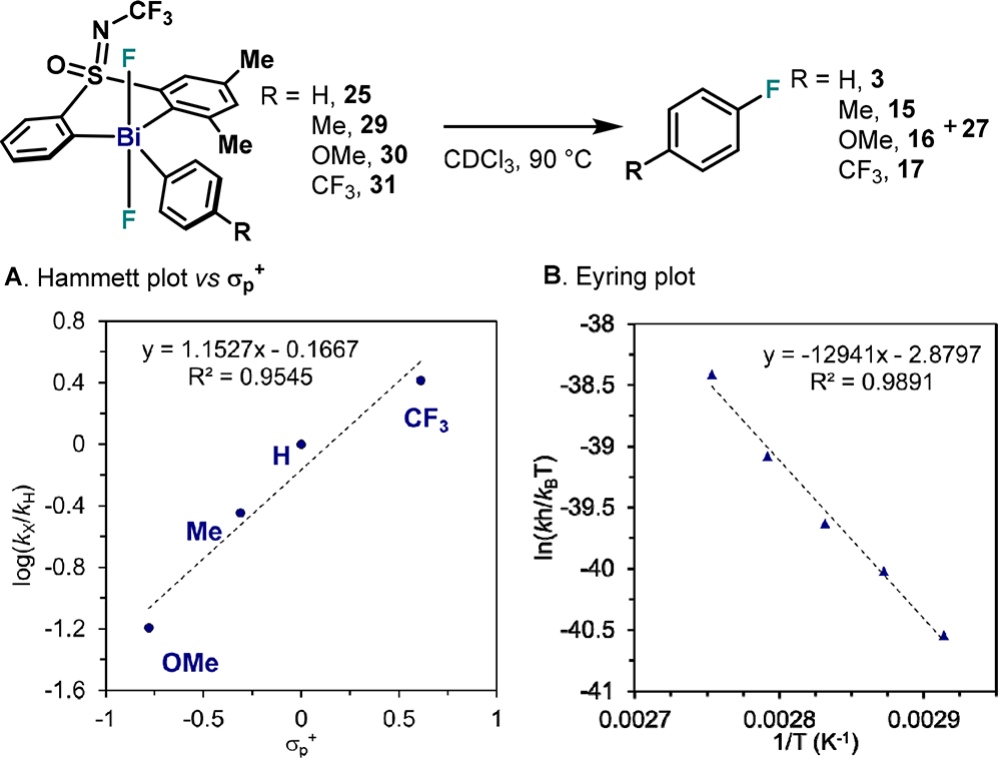
Electronic and thermodynamic analysis of reductive elimination from **25** and **29**–**31**. (A) Hammett plot for the reductive elimination of *para*-substituted σ-aryl-Bi(V) difluorides **25** and **29**–**31**. (B) Eyring analysis for **25**.

Electronic modulation of the pendant aryl ring was also assessed in monomeric Bi(V) difluoride complexes. Due to synthetic simplicity, we focused on the reductive elimination from **25**, containing a sole *ortho*-Me in the ligand scaffold. Thus, we synthesized several *para*-substituted σ-aryl Bi(V) difluoride complexes (**29**–**31**, Figure 8), and their thermal decomposition was evaluated in CDCl_3_ at 90 °C.^34^ Similarly to model complex **4**, the Hammett plot using σ_p_ parameters resulted in poor linearity (*R*^2^ = 0.77); yet, when plotting log(*k*_X_/*k*_H_) vs σ_p_^+^, a *R*^2^ = 0.9545 was obtained (Figure 8A). The ρ = 1.15 indicates a faster reductive elimination when electron-withdrawing groups (EWG) are present in the pendant aryl ring. In addition, this reaction presents higher sensitivity to *para* substitution compared to model complex **4** (ρ = 0.43). Evaluation of the thermodynamic parameters through Eyring analysis of **25** in CDCl_3_ (Figure 8B) revealed a Δ*H*^⧧^ = 25.7 ± 1.6 kcal·mol^–1^ and a Δ*S*^⧧^ = −5.7 ± 4.4 cal·mol^–1^·K^–1^, similar to values obtained for sterically crowded **26** (Δ*H*^⧧^ = 26.5 ± 1.5 kcal·mol^–1^ and Δ*S*^⧧^ = −6.1 ± 4.2 cal·mol^–1^·K^–1^).^34^ The rather small values on the entropic contribution are in stark contrast with that obtained for model complex **4** (Δ*S*^⧧^ = −34.7 ± 1.9 cal·mol^–1^·K^–1^). This latter value, combined with the structural analysis of **4**, suggests that dimerization processes prior to C(sp^2^)–F bond formation could play an important role in the reductive elimination from model complex **4**. Large entropic contributions have also been observed in other dimerization equilibrium in Bi(II) species.^37^ Hence, the small Δ*S*^⧧^ obtained for sterically congested **25** and **26** points to a reductive elimination from monomeric species, without prior dimerization.

### Effect of the Substitution on the Sulfoximine Scaffold in the C–F Reductive Elimination

**Figure 9:**
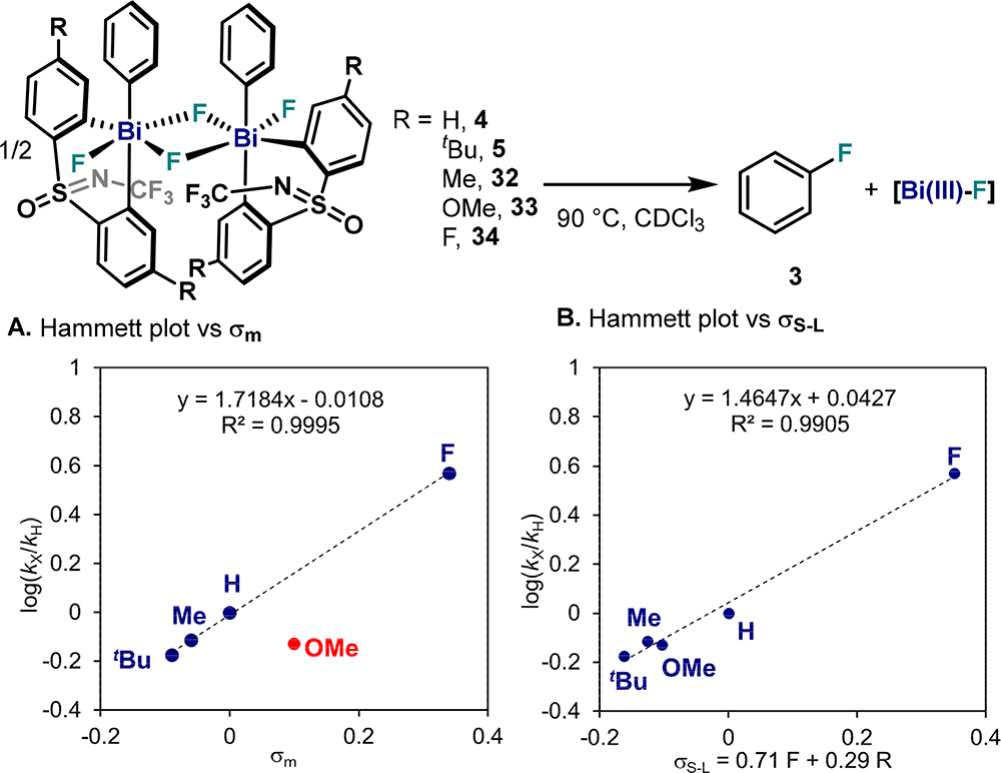
Electronic analysis of reductive elimination from **4**, **5**, and **32**–**34**. (A) Hammett plot vs σ_m_ values. (B) Hammett plot vs σ_S–L_ values.

Various σ-aryl Bi(V) difluoride complexes bearing ligands with substituents in *meta*-position with respect to the Bi center (**4**, **5**, **32**–**34**, Figure 9) were thermally decomposed at 90 °C, and the decay was monitored by ^19^F NMR together with formation of **3** and the corresponding Bi(III)–F. Arguably, a study employing ligands with *para*-substituents to the Bi center would have been more appropriate. However, due to synthetic limitations on the synthesis of the parent Bi(III)–Ph complexes, symmetric diphenyl sulfoximine scaffolds were utilized. As shown in Figure 9A, a Hammett analysis resulted in a value of ρ = 1.72 ± 0.03 when σ_m_ was used in the *x*-axis, excluding complex **33** bearing −OMe moieties, which followed a differing trend. Introducing resonance effects via the Swain–Lupton equation,^38^ a similar slope of ρ = 1.47 ± 0.08 was obtained (Figure 9B), now including **33**.^34^ These results indicate an important role of resonance contributions from strong π-donor substituents as well as a faster reductive elimination with ligands bearing *m*-EWG. We hypothesize that the Bi center is mainly affected by field, while the S(O)NCF_3_ unit is strongly affected by resonance, and for these reasons, pure σ_m_ and σ_p_ values could not be used.

### Evaluation of Fluoride Inhibition in the C(sp^2^)–F Bond Formation from **4**

**Figure 10:**
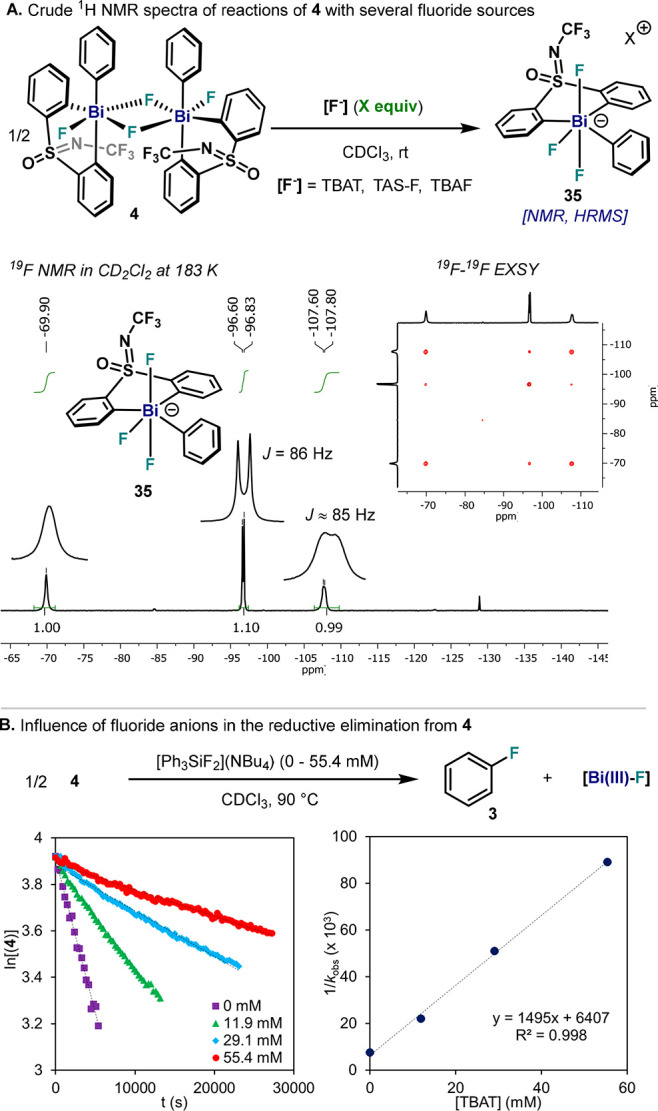
(A) Synthesis of anionic species **35** from **4** in the presence of several fluoride sources. (B) Left, reductive elimination of fluorobenzene from **4** in the presence of different amounts of TBAT. Right, decay of **4** dependence on TBAT concentration.

In our previous study, we noted that formation of fluorobenzene was prevented when 1.0 equiv of tetrabutylammonium fluoride (TBAF) was added, which led us to propose a cationic intermediate in equilibrium with neutral pentavalent Bi(V) species **4**.^32b^ Together with a slower rate, we also observed significant shifts in ^1^H NMR and partial decomposition of the initial Bi(V) difluoride, which we attributed to possible interactions with THF or even H_2_O, which is present in 5 wt % in commercial 1.0 M TBAF solutions. With the goal of elucidating the effect of fluoride anions in the aryl–F reductive elimination step, fluoride sources that present high solubility in common organic solvents were selected and mixed with complex **4** (Figure 10A). Addition of 1.0 equiv of tetrabutylammonium difluorotriphenylsilicate (TBAT) or tris(dimethylamino)sulfonium difluorotrimethylsilicate (TAS-F) to **4** resulted in the smooth formation of a new species together with concomitant formation of the corresponding R_3_Si–F species. These results encouraged us to re-evaluate our previous experiment using 1.0 equiv of TBAF from a 1.0 M solution in THF; indeed, the same species obtained with TBAT and TAS-F were observed. These experiments collectively suggest the formation of an anionic Bi(V) compound consisting of three fluorine atoms directly bound to the Bi center. Indeed, HRMS analysis of these samples confirmed the presence of anionic species **35** (experimental *m*/*z* = 626.0438; theoretical *m*/*z* = 626.0431). Although it was not possible to obtain a suitable single-crystal for XRD analysis, **35** (from reaction with TAS-F) was fully characterized spectroscopically by NMR in CD_2_Cl_2_.^34^ Initial proof of the presence of three F atoms directly bonded to the Bi center was collected measuring ^13^C NMR at 223 K, which revealed two reasonably resolved Bi–^13^C signals as quadruplets with a coupling constant of *J*_CF_ ≈ 20 Hz, which compares to the values obtained for monomeric complex **26**. This result points to three equivalent fluorine atoms directly bound to the Bi center experiencing fast dynamic processes. VT ^19^F NMR provided additional insight on the dynamics of this anionic Bi(V). Whereas at 298 K, a broad Bi–F signal appears at −88.9, at 183 K, the signal splits into three independent signals at −69.9, −96.7, and −107.7 ppm, with a coupling constant within the range of F–Bi(V)–F (*J*_FF_ ≈ 85 Hz). Noteworthy, ^19^F–^19^F COSY measurements confirmed F–Bi–F through-bond interactions,^34^ while ^19^F–^19^F EXSY studies (inset Figure 10A) revealed fast exchange between F atoms even at 183 K. Unfortunately, no ^19^F–^19^F through-space coupling was observed between Bi–F and the CF_3_ moiety, impeding a full assignment of the F signals. However, the presence of three ^19^F signals, two of them being doublets with *J*_FF_ = 85 Hz and similar chemical shift, suggests that **35** could adopt a pseudo-octahedral structure such as the one depicted in Figure 10A.

At this point, **4** was subjected to thermal decomposition in the presence of different amounts of TBAT, and the rate of reductive elimination was monitored. As depicted in Figure 10B (left), slower reaction rates were obtained with higher concentrations of TBAT, which indicate an inverse rate dependence as a function of TBAT concentration. Indeed, a positive, linear dependence of the reciprocal of the rate constant of Bi(V) decay (1/*k*_obs_) vs TBAT concentration was observed together with a nonzero intercept (Figure 10B, right). Furthermore, while the yield of fluorination was 94% for **4** without additional fluorides, it decreased to 71% in the presence of 1.1 equiv of TBAT. Altogether, these results unambiguously indicate that the slower rate in the presence of fluoride anions is a consequence of the formation of hexacoordinated anionic species **35**. This species is proposed to engage in aryl–F reductive elimination events via neutral pentavalent Bi(V) intermediates, a process that appears to be more feasible compared to the previously proposed cationic species.

### Theoretical Analysis of the Reductive Elimination Step from Neutral Bi(V) Difluoride **4**

**Figure 11:**
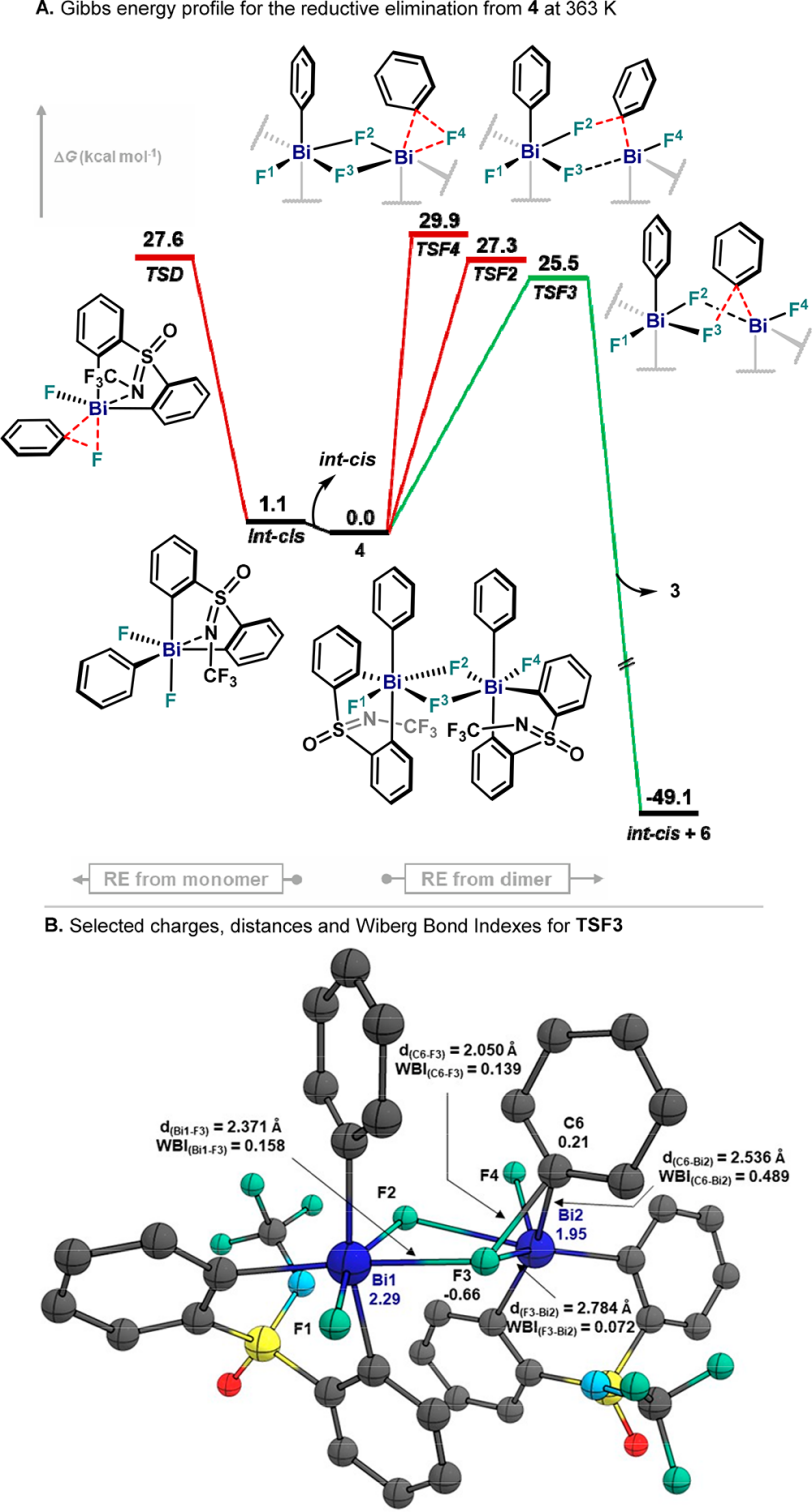
(A) Gibbs energy profile of the reductive elimination of fluorobenzene from species **4** at 363 K. (B) Selected structural and electronic parameters for **TSF3**. Relative Gibbs energy values are given in kcal·mol^–1^.

Intrigued by the experimental results obtained with neutral pentavalent σ-aryl Bi(V) difluoride complexes, we performed a collection of DFT calculations to support and fully understand the aryl–F bond-forming step. After a brief method evaluation,^34^ geometry optimizations and frequency calculations were carried out at the B3LYP-D3BJ level of theory^39^ with the def2-TZVP(-f) basis sets and matching auxiliary basis set (def2/J).^40^ The default small-core effective core potential was used for Bi,^41^ and solvent effects (chloroform) were incorporated using a conductor-like polarizable continuum model. Geometry optimization, normal-mode analysis, and single-point calculations were carried out with a development version of the ORCA 4.2 suite of programs.^42^ Natural bond orbital (NBO) analysis was performed at the same level of theory.^43^ Initially, the reductive elimination of fluorobenzene from species **4** was evaluated at 363 K (Figure 11A). Due to the symmetric nature of **4**, three different TSs for the reductive elimination from one Bi center (Bi2, see Figure 2) were identified, which involved fluoride ligands in pendant (F4, equivalent to F1) and μ-bridged positions (F3 and F2). Reductive elimination from the pendant fluoride ligand was highly energetic, with an activation barrier of Δ*G*^⧧^ = 29.9 kcal·mol^–1^ (**TSF4**), similar to the C–F bond formation from the shared fluoride ligand within the μ-bridge (Δ*G*^⧧^ = 27.3 kcal·mol^–1^, **TSF2**). Interestingly, a value of Δ*G*^⧧^ = 25.5 kcal·mol^–1^ was obtained when reductive elimination was computed from F3, releasing fluorobenzene and leading to ***int-cis***, which is the most stable monomeric **4** isomer (vide infra) together with **4**. This value is in agreement with the activation barrier obtained experimentally by Eyring analysis, Δ*G*^⧧^ = 25.9 ± 0.9 kcal·mol^–1^, which suggests the reductive elimination of fluorobenzene could proceed through **TSF3**. In addition, the monometallic aryl–F bond-forming event described in Figure 11 constitutes a rare example of μ-difluoride-bridged species, leading to fluorobenzene in synthetically relevant yields, as μ-difluoride-bridged dimers in transition-metal chemistry tend to inhibit further reactivity due to their high stability.^9e^ Characterization of structural and electronic parameters of **TSF3** through NBO analysis revealed a positive charge on C6 in the TS with a value of *q*_*C6*_ = +0.21 (Figure 11B). Meanwhile, the fluoride anion remained nucleophilic (*q*_F3_ = −0.66), in agreement with the Hammett plot obtained with *para*-substituted **4** and **8**–**13** (Figure 5). Interestingly, Bi2 presents a smaller positive charge (*q*_Bi1_ = +1.95) compared to Bi1 (*q*_Bi1_ = +2.25), which denotes its partial reduction to Bi(III). Furthermore, the Wiberg bond index (WBI) and bond distance analysis clearly show the cleavage of the Bi2–C6 (WBI_(Bi2–C6)_ = 0.49, *d* = 2.536 Å) and Bi2–F3 (WBI_(Bi2–F3)_ = 0.07, *d* = 2.784 Å) bonds occurs simultaneously with C6–F3 formation (WBI_(C6–F3)_ = 0.14, *d* = 2.050 Å), suggesting a concerted reductive elimination of fluorobenzene. It is important to note that dimeric species **4** is calculated to be ≥1.1 kcal·mol^–1^ more stable than two individual ***int-cis*** or ***int-trans*** monomers, indicating **4** as the lowest-energy species.^34^ Nonetheless, the aryl–F bond formation from monomeric species was also evaluated (Figure 11A). Results obtained for the reductive elimination of monomeric ***int-cis*** proceed through a concerted mechanism (**TSD)** and are higher in energy than **TSF3** (ΔΔ*G*^⧧^(**TSF3**–**TSD**) = −1.0 kcal·mol^–1^). In this case, however, additional 1.1 kcal·mol^–1^ would be required in the **TSD** to overcome the dissociation of **4**, resulting in an overall 27.6 kcal·mol^–1^. Pathways from ***int-trans*** were located >28 kcal·mol^–1^ and, hence, not considered.^34^ These results suggest that reductive elimination for model complex **4** takes place preferentially from a bimetallic Bi(V) species in solution, albeit the reductive elimination from ***int-cis*** is also feasible. This is consistent with the large negative Δ*S*^⧧^ value obtained experimentally for **4**, which can be explained by a highly ordered TS in bimetallic **TSF3** (Figure 11) or the possible monomer–dimer equilibriums previous to the aryl–F reductive elimination step.

### Theoretical Analysis of the Reductive Elimination Step from Neutral Bi(V) Difluoride **26**

**Figure 12:**
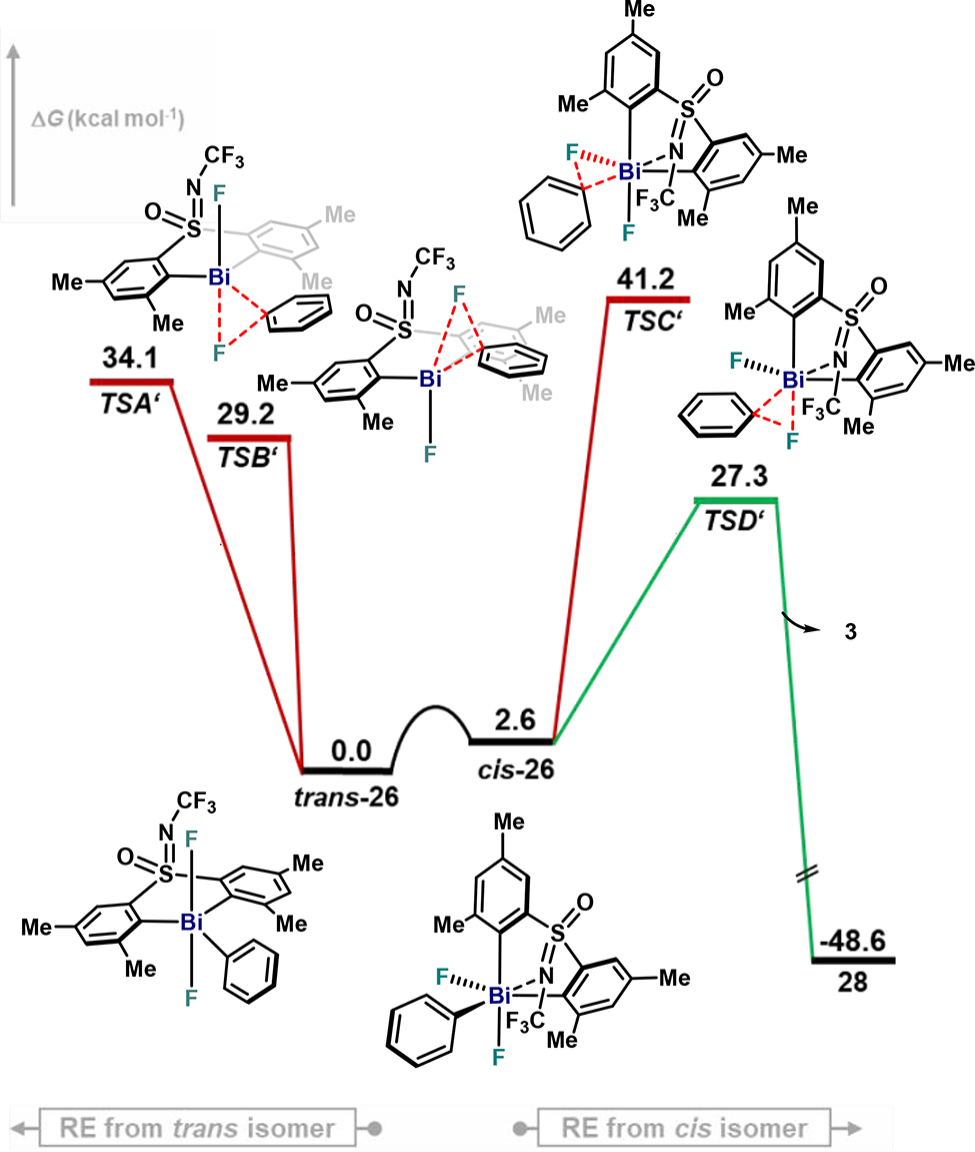
Gibbs energy profile of the reductive elimination of fluorobenzene from sterically congested monomeric species **26** at 363 K. Relative Gibbs energy values are given in kcal·mol^–1^.

As shown in Figure 6, sterically bulky **26** is characterized as a monomer, and no dimers were formed in solution or in the solid state. Yet, C–F bond formation is also possible from this complex, leading to good yields of **3**. To investigate the differences between complexes such as **26** and **4**, reductive elimination from symmetric monomeric compound **26** was evaluated at 363 K, and its possible pathways for fluorobenzene formation are depicted in Figure 12. Similarly to monomeric configurations of model complex **4** (Figure 11A), *trans* and *cis* isomers of **26** were studied. In this case, ***trans**-***26** resulted to be more stable than ***cis*-26**, which is consistent with the characterization of this compound in solution as well as in solid state (Figure 6). Fluorobenzene from monomeric ***trans**-***26** stems from a concerted C–F bond-forming event involving the bottom (**TSA′**) or the top (**TSB′**) fluoride ligand, with an activation energy of 34.1 kcal·mol^–1^ and 29.2 kcal·mol^–1^, respectively. Equatorial C–F bond formation occurs from ***cis*-26** through a highly energetic **TSC′**, with a value of Δ*G*^⧧^ = 41.2 kcal·mol^–1^. On the other hand, equatorial C and axial F in **TSD′** results in a more favorable pathway (27.3 kcal·mol^–1^) for C–F bond formation, in agreement with the experimental activation barrier obtained from the Eyring analysis for **26**, Δ*G*^⧧^ = 28.3 kcal·mol^–1^ ± 0.9 kcal·mol^–1^. Results depicted in Figure 12 show the feasibility of the aryl–F bond-forming event for monomeric σ-aryl Bi(V) difluoride species in solution. The activation energy for **26**, however, is still higher than that obtained for the lowest-energy pathway for model complex **4**, which is consistent with the slower kinetic profile obtained for sterically crowded σ-aryl Bi(V) difluoride **26** (Figure 7).

### Solid-State Analysis of a Fluorobismuthonium Bi(V)–F

**Figure 13:**
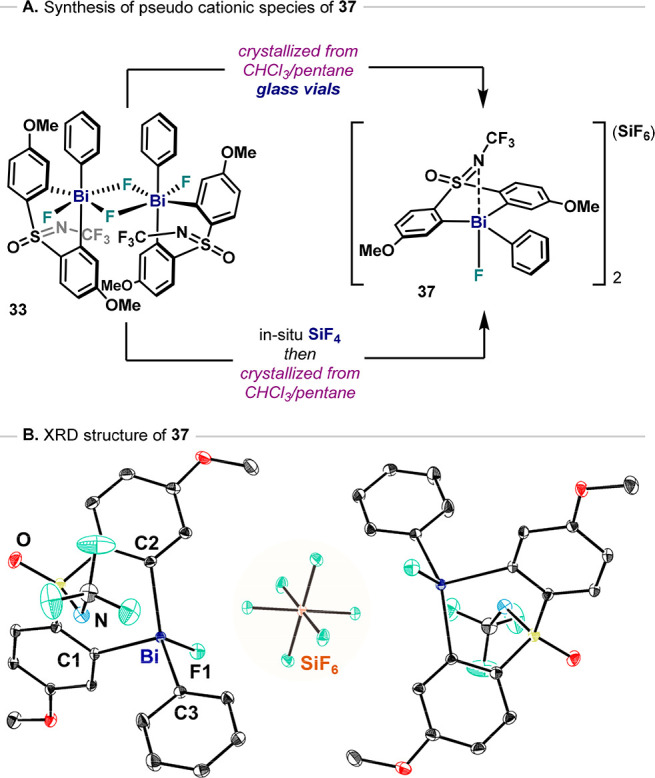
(A) Synthesis of fluorobismuthonium species **37** and (B) ORTEP representation of XRD structure of **37**. Hydrogen atoms and solvent molecules omitted for clarity.

The addition of Lewis acids such as BF_3_ to **4** could lead to the formation of fluorobismuthonium species in solution bearing BF_4_^–^ as counteranions.^32b^ While the compound **36** obtained from model complex **4** was originally characterized by NMR and HRMS, solid-state characterization was precluded due to the poor thermal stability and high hygroscopic properties of these complexes. Surprisingly, during crystallization attempts of neutral difluoride species **33**, we isolated hexafluorosilicate salt **37** instead (Figure 13A). It was speculated that the interaction of difluoride **33** with glass generated SiF_4_ in situ, leading to fluoride abstraction from the neutral difluoride **33**. To reproducibly obtain this species, a mixture of difluoride **33** in dry CH_2_Cl_2_ was bubbled with in situ generated SiF_4_ gas, leading to **37** in modest yields.^34^ Compound **37** crystallizes as a symmetric salt, with two monocationic Bi moieties sharing one SiF_6_^2–^ anion with a Bi···FSiF_5_ contact of 2.6607(13) Å (Figure 13B). The SiF_6_^2–^ anion shows elongated Si–F distances (*d* = 1.7215(13) Å) for the coordinating F atoms compared to noncoordinating Si–F bonds (*d* = 1.6701(14)–1.6809(14) Å). The cationic Bi fragment in **37** exhibits a distorted TBP geometry, with a single Bi–F bond (*d* = 2.0414(16) Å) and a short Bi–N interaction (*d* = 2.836(2) Å). Species **37** shows similar Bi–C1 (*d* = 2.224(2) Å) and Bi–C2 (*d* = 2.222(2) Å) distances and a slightly shorter Bi–C3 (*d* = 2.207(2) Å) bond, slightly bent toward C2 (C3–Bi–C2, 139.03(8) °; C1–Bi–C3, 110.95(8) °).

### Effect of the Substitution on the Sulfoximine Scaffold in the C–F Reductive Elimination from Fluorobismuthonium Bi(V)–F

**Figure 14:**
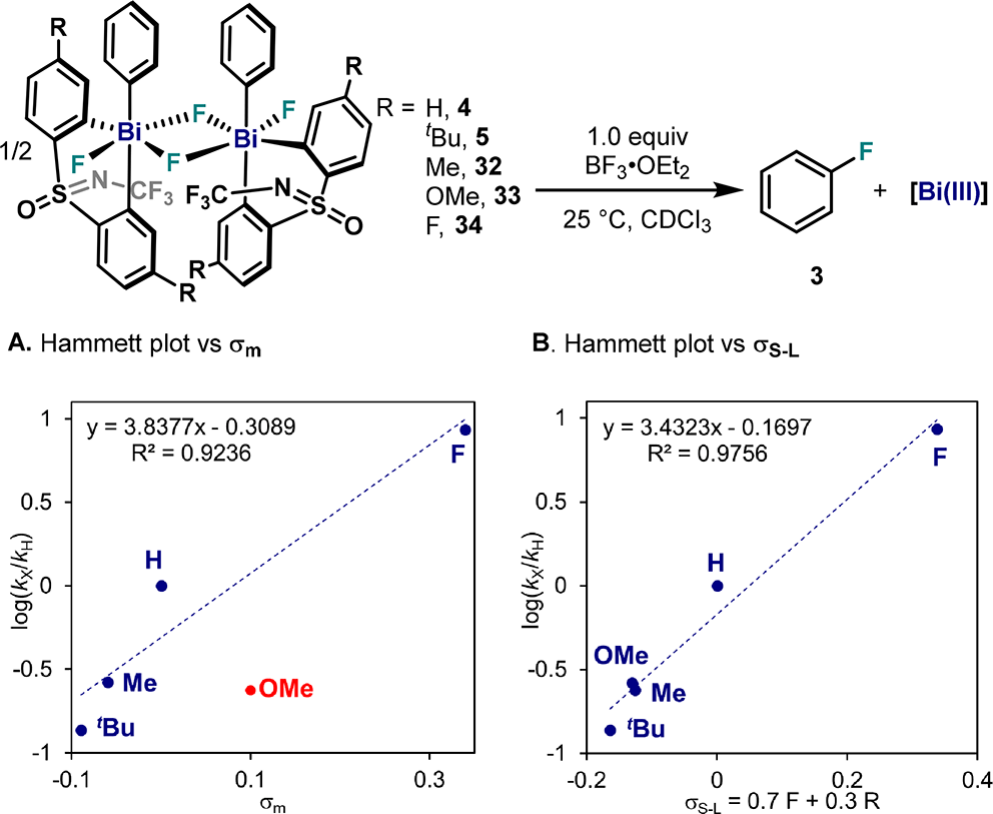
Electronic analysis of reductive elimination from **4**, **5**, and **32**–**34** in the presence of 1.0 equiv of BF_3_·OEt_2_. (A) Hammett plot vs σ_m_ values. (B) Hammett plot vs σ_S–L_ values.

Intriguingly, reductive elimination from **37** did not occur at 25 °C in CDCl_3_, and it was sluggish at 60 °C (31% of **3**). The slow reactivity for cation **37** was ascribed to the strong electron-releasing properties of −OMe groups in the ligand scaffold and prompted us to conduct an assessment on the effects of the substituents in the ligand on the reductive elimination. To this end, a variety of complexes (**4**, **5**, and **32**–**34**) were thermally decomposed at 25 °C in the presence of BF_3_ and the kinetics monitored by NMR (Figure 14). As shown in Figure 14A, a Hammett analysis of the reaction kinetics resulted in a value of ρ = 3.84 ± 0.78 when σ_m_ was plotted in the *x*-axis, excluding complex **33** bearing *m*-OMe moieties. Introducing resonance effects via the Swain–Lupton equation (Figure 14B),^38^ a similar ρ value was obtained (ρ = 3.43 ± 0.31) with improved linearity (*R*^2^ = 0.9756), now including −OMe groups (**33**). The trend obtained for this Hammett plot is similar to the results obtained for neutral σ-aryl Bi(V) difluorides (Figure 9), highlighting the increased nucleofuge character of the Bi center when EWGs are installed in the ligand backbone. Interestingly, thermal decomposition of **33** bearing *m*-OMe substituents produces a significant 92% yield of **3**. This result is in stark contrast with species **37** bearing a SiF_6_^2–^ counteranion, which produced fluorobenzene in <5% yield at 298 K after 48 h. Indeed, high yields of fluorobenzene when mixing species **33** with BF_3_ suggest a possible involvement of the BF_4_ anion in the C–F bond-forming step. Use of other Lewis acids to abstract a fluoride ligand, such as B(C_5_H_6_)_3_ and SbF_5_, resulted in similar outcomes to SiF_4_,^34^ presenting slower reaction rates or decomposition of starting material.

### Effect of Substitution on the Pendant Aryl Ring in the C(sp^2^)–F Bond Formation from Fluorobismuthonium Bi(V)–F

**Figure 15:**
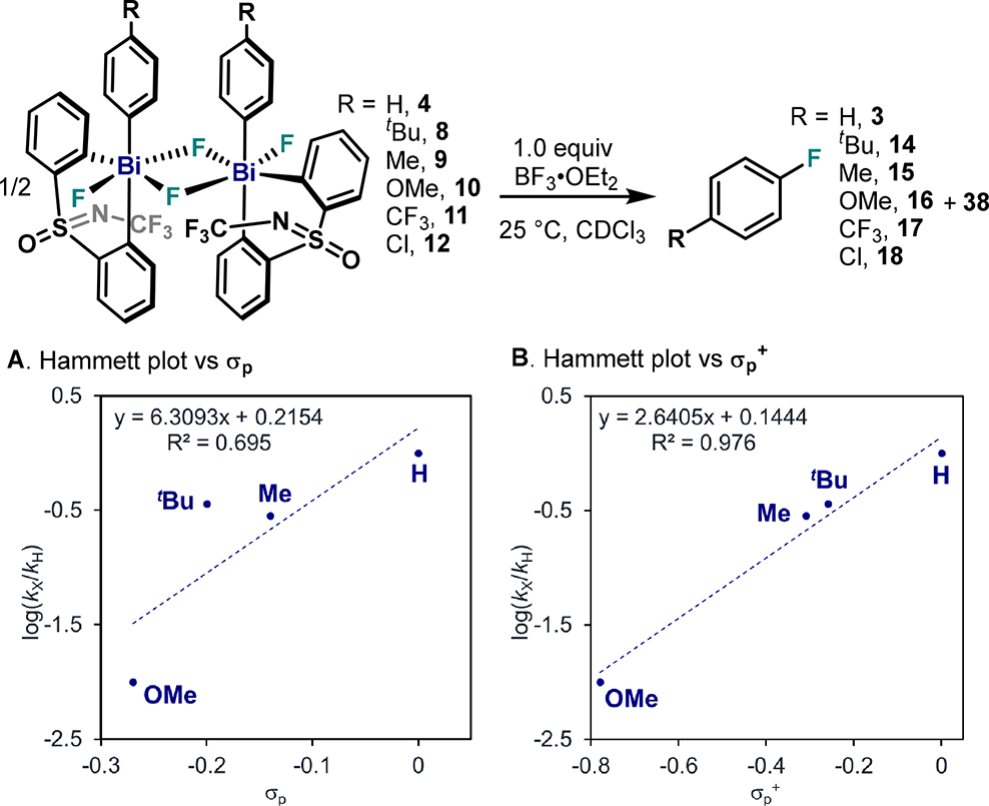
Electronic analysis of reductive elimination from **4** and **8**–**12** in the presence of 1.0 equiv of BF_3_·OEt_2_. (A) Hammett plot vs σ_p_. (B) Hammett plot vs σ_p_^+^ taking into account resonance contributions.

*p*-Substituted aryl Bi(V) difluorides were dissolved in CDCl_3_ at 25 °C together with 1.0 equiv of BF_3_·OEt_2_, and the decay of the *in situ* generated cationic complex was monitored by NMR spectroscopy (Figure 15). Despite the poor linearity observed in Figure 15A, a positive slope (ρ = 6.31 ± 2.96) was obtained for **4** and **8**–**12** (*R*^2^ = 0.695). An increased correlation (*R*^2^ = 0.976) was obtained when Hammett analysis was done using σ_p_^+^ values obtaining a ρ = 2.64 ± 0.29 (Figure 15B). Interestingly, this value corresponds to a 5-fold increase compared to neutral difluorides (Figure 5), pointing to a much larger change in electron density in the TS for these fluorobismuthonium species. Strikingly, no reaction was observed with *p*-EWG (*p*-CF_3_, **11** and *p*-Cl, **12**). Although formation of fluorobismuthonium complexes was confirmed by NMR and HRMS studies, fluoroarenes **17** and **18** were only obtained after warming the reaction mixture to 90 °C over 2 h, leading to large amounts of decomposition.^34^ This result is in bold contrast to the Hammett plot for neutral difluorides, which showed rapid reaction kinetics in the presence of *p*-CF_3_ or *p*-CN. We speculated that the complete inhibition of reductive elimination from fluorobismuthonium species bearing electron-deficient arenes could be connected to the need of elongation of the Bi–C_ipso_ bond, thus becoming a highly energetic rate-determining step prior to the nucleophilic attack of the BF_4_^–^ anion in the TS.^34^ In the Hammett plot, this change in the rate-determining step would be represented in a very sharp break with a large negative ρ value for EWGs.

### Theoretical Analysis of the Reductive Elimination Step from Fluorobismuthonium **36**

**Figure 16:**
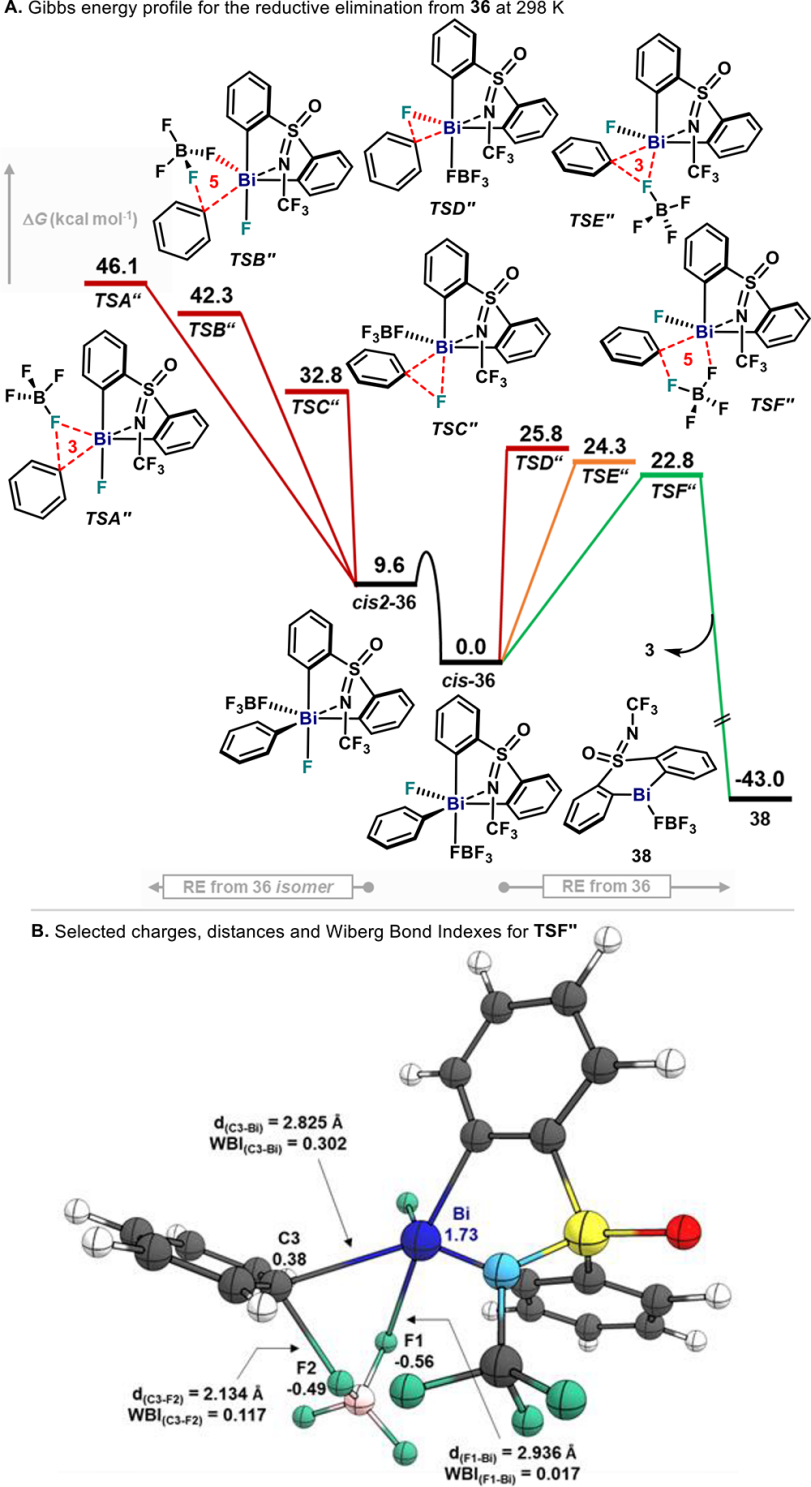
(A) Gibbs energy profile of the reductive elimination of fluorobenzene from fluorobismuthonium species **36** at 298 K. (B) Selected structural and electronic parameters for **TSF′′**. Relative Gibbs energy values are given in kcal·mol^–1^.

XRD studies of **37** and previously conducted NMR studies of **36**(32b) point to species ***cis*-36** being the most stable isomer; hence, different C–F bond formation pathways from ***cis*****-36** were evaluated at 298 K (Figure 16A). Although reductive elimination pathways from thermodynamically less stable ***cis*****2**–**36** species (Δ*G* = 9.6 kcal·mol^–1^) were also studied, energetic barriers resulted in prohibitive values (Δ*G*(**TSA′′**)^⧧^ = 46.1 kcal mol^–1^, Δ*G*(**TSB′′**)^⧧^ = 42.3 kcal·mol^–1^, and Δ*G*(**TSC′′**)^⧧^ = 32.8 kcal·mol^–1^). Therefore, reductive elimination from the most stable isomer, ***cis**-***36**, was studied in more detail. Three pathways for C–F bond formation with significantly distinct energy barriers were identified. On one hand, the direct C–F bond formation through a three-membered TS involving the Bi–F bond resulted in a barrier of Δ*G*(**TSD′′**)^⧧^ = 25.8 kcal·mol^–1^. On the other hand, lower values of Δ*G*^⧧^ were obtained when BF_4_ was used as the fluoride source. Indeed, a three-membered TS involving B–F cleavage delivered an activation energy of Δ*G*(**TSE′′**)^⧧^ = 24.3 kcal·mol^–1^, while a five-membered TS resulted in the energetically lowest pathway in Figure 16A, with a theoretical value of Δ*G*(**TSF′′**)^⧧^ = 22.8 kcal·mol^–1^, which is in agreement with the experimental value obtained (Δ*G*^⧧^ = 22.4 ± 2.2 kcal·mol^–1^).^32b^ Structural and electronic analyses of **TSF′′** by NBO analysis show a dramatic buildup of positive charge at C3 during the TS, with a value of *q*_C3_ = +0.38, which represents a 2-fold increase compared to neutral difluorides (see Figure 16B). The fluoride F2 in the BF_4_ unit remains nucleophilic (*q*_F2_ = −0.49), and the Bi center presents a smaller positive charge Bi (*q*_Bi_ = +1.79) compared to the neutral difluoride TSs previously analyzed. These results, together with the Hammett plot presented in Figure 15, suggest that the Bi center in **TSF′′** presents more Bi(III) character, and it can be regarded as a highly polarized, late TS. Indeed, the WBI and bond distance analysis clearly show an almost cleaved Bi–C3 bond (WBI_(Bi–C3)_ = 0.30, *d* = 2.825 Å) together with a partially formed C3–F2 bond (WBI_(C3–F2)_ = 0.12, *d* = 2.134 Å). This concerted-asynchronous ligand coupling event involves an initial elongation of the Bi–C3 bond, leading to a highly polarized TS for the final C3–F2 bond formation. The required elongation of the Bi–C3 in **TSF′′** results in an energetic penalty in the **TS** for fluorobismuthonium complexes with pendant aryl moieties bearing *para*-EWG, consistent with the absence of C–F bond formation from **11** and **12** at 25 °C.^34^ Indeed, activation barriers following **TSF′′** for fluorobismuthonium derivative of **11** resulted in a Δ*G*^⧧^ = 25.3 kcal·mol^–1^, significantly higher compared to **36**.^34^

Collectively, these results point to a preferred five-membered TS using BF_4_^–^ as a fluoride source, similar to previous reports with heavy main group metals such as Pb and Tl^26,27^ or recent examples using Bi and OTf, ONf^32c^ or phenols^44^ as ligands. Collectively, the data presented herein show that aryl–F bond formation from fluorobismuthonium σ-aryl Bi(V) fluoride species **36** proceeds through a different mechanism when compared to neutral σ-aryl Bi(V) difluorides.

### Identification of Cationic Species in the Oxidation of **1** with 1-Fluoro-2,6-dichloropyridinium Tetrafluoroborate (**2**)

**Figure 17:**
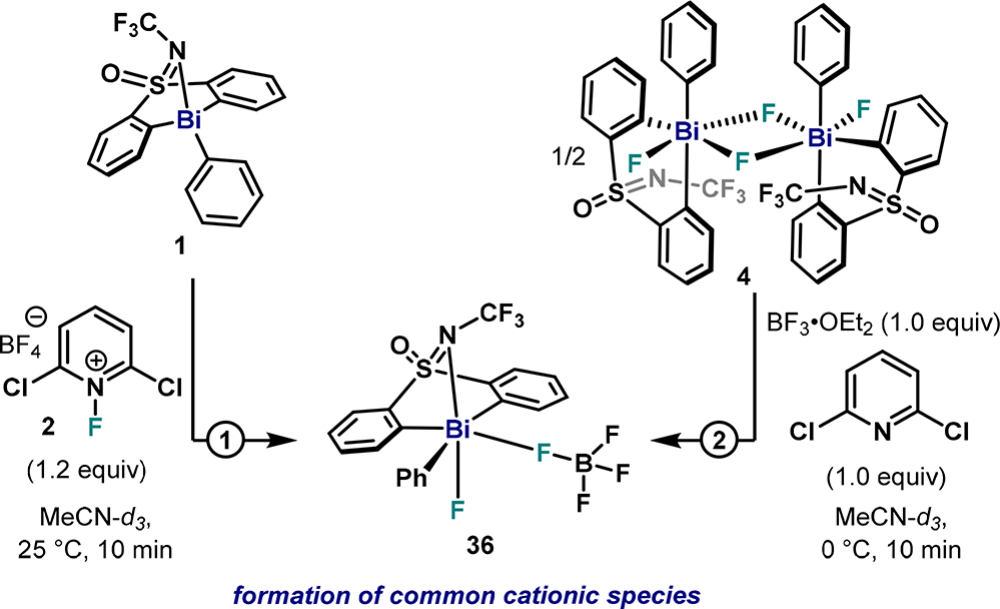
Reactivity of **1** with 1-fluoro-2,6-dichloropyridinium tetrafluoroborate **2** in MeCN-*d*_3_ (reaction 1) and **4** with BF_3_·OEt_2_ complex in the presence of 2,6-dichloropyridine in MeCN-*d*_3_ (reaction 2).

Due to the faster reaction rates for C–F bond formation from fluorobismuthonium species, **2** has been identified as a suitable electrophilic fluorinating agent for Bi(III). Despite the excellent yield of fluorobenzene after thermal decomposition, evidence of the intermediacy of similar fluorobismuthonium complexes such as **36** was eluded by the poor solubility of **2** salt in CDCl_3_. Hence, the eaction of **1** with 1.0 equiv of **2** was performed in MeCN-*d*_3_ (Figure 17, reaction 1) at 0 °C, and the reaction crude was analyzed by ^1^H and ^19^F NMR after 10 min. In parallel, with the aim of furnishing fluorobismuthonium intermediate **36**, pentavalent difluoride **4** was reacted with BF_3_·OEt_2_ in the presence of 1.0 equiv of 2,6-dichloropyridine in MeCN-*d*_3_ (Figure 17, reaction 2). Similar ^1^H and ^19^F NMR spectra were obtained in both cases, thus coinciding with the characterization data obtained for cationic species **36** in reaction 2 (Figure 17). Furthermore, HRMS crude analysis of reaction 1 showed a peak with *m*/*z* = 588.0456 corresponding to the [**36**-BF_4_]^+^ ion (theoretical *m*/*z* = 588.0456).^34^

### Evaluation of the Ligand Scaffold in the C–F Bond Formation from Bi(III)–Ph and 1-Fluoro-2,6-dichloropyridinium Tetrafluoroborate (**2**)

In order to gain insight on the features required to promote and inhibit formation of fluorobenzene, oxidation of various sulfone- and sulfoximine-based Bi complexes was examined using **2** (Table 1). Similar to neutral difluorides,^32b,34^ complexes with sulfone-based backbones without substituents (**39**) resulted in poor yields of **3**. Introduction of EWG in the flanking aryl rings resulted in a dramatic increase of yield (**40** and **41**), similar to the effect observed for fluorobismuthonium species **34** in Figure 9. Sulfoximine-based complexes varying the substituent on the N atom were also evaluated. Species bearing N–CF_3_ (**1**) and N–CF_2_CF_3_ (**42**) groups were demonstrated to be excellent platforms for the synthesis of **3**, while the installation of a N–Me group (**43**) failed to provide the desired product. Complexes bearing N–Ar units (**44**–**46**) were also tested, identifying N–Ar moieties with *p*-EWG as superior ligands. The results obtained in Table 1 are therefore in agreement with all the data collected up to now on the C–F bond formation: Electron-deficient ligand scaffolds promote aryl–F reductive elimination, making the Bi center a better nucleofuge toward the incoming F nucleophile.

**Table 1:**
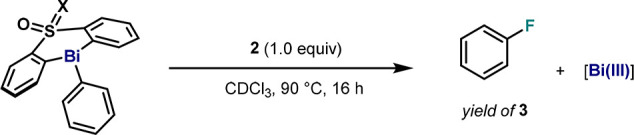

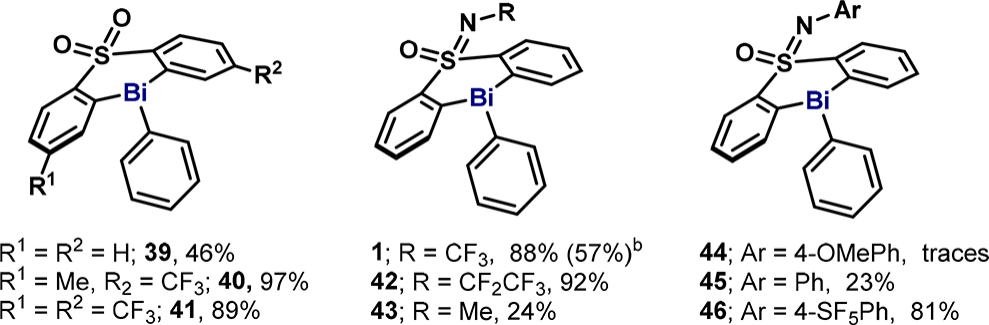
Substitution Effects on the Diphenyl Sulfone Scaffold and the Sulfoximine Moiety on the Oxidation/Reductive Elimination Sequence from Phenyl Bismine Species^a^

a Yields determined by ^19^F NMR using 1-fluoro-4-nitrobenzene as internal standard.

b Reaction performed using CD_3_CN as solvent.

### Improved Methods for the Fluorination of Boronic Acids

The mechanistic considerations inferred from data presented in Table 1 provide relevant information toward a more practical protocol for fluorination. Indeed, the excellent yield obtained with complex **40** evades the use of compounds bearing −S(O)NCF_3_– moieties (**1**), which are synthetically tedious, low yielding, and expensive compared to sulfone-based ligands. Thus, with the aim of developing an improved and easily accessible method, we evaluated compounds **40** and **41**, which can be easily synthesized and furnished fluorobenzene in excellent yields. First, we assessed the stoichiometric fluorination of boronic acids through a two-step method involving a transmetalation and a one-pot oxidation/reductive elimination. For the first step, we employed the Bi–OTs complex **47** (Table 2).^34^ Transmetalation with a variety of arylboronic acids was assessed using Ball’s conditions,^44c^ furnishing a variety of Bi–aryl compounds in excellent yields independently of the substitution pattern of the arylboronic acid (Table 2).^34^ It is important to note that this transmetalation protocol employs 1.0 equiv of arylboronic acid, while in our previous report, we were restricted to an excess of transmetallating reagent.^32b^ After transmetalation, oxidation of Bi–aryl compounds with 1.0 equiv of **2** in CDCl_3_ at 90 °C furnished the corresponding arylfluorides. It is worth mentioning that due to decomposition of **2** in the presence of water,^45^ it was not possible to perform a one-pot reaction without previous isolation of the corresponding Bi–aryl compounds. Interestingly, this system bodes well with a variety of *para*-substituents (**3**, **15**–**17**, **48**–**53**), including CF_3_ (**17**, 55%), halogens (R = Cl, **18**, 51%; R = F, **49**, 52%), TMS (**50**, 93%), and alkynyl (**52**, 22%) moieties. Despite the broader functional group tolerance, trace amounts of arylfluoride were obtained when ether substituents were evaluated (**16** and **53**, <5%), highlighting some limitations of the methodology. Alkyl chains (**54**–**56**) and silyl groups (**57**) in *meta*-position resulted in good yields, including compounds with large substituents (**58** and **63**), while the installation of *meta*-EWG (**59**–**62**) slightly decreased the efficiency of the oxidation/reductive elimination. The electrophilic fluorination could also be performed on Bi–aryl compounds bearing *ortho*-substituents, such as Me (**64**, 92%) and Br (**65**, 33%) groups, albeit with lower yields for the latter. Furthermore, vinyl groups (**66**, 63%) and polyaromatic arenes (**67**, 63%) could also be accommodated. Overall, the Bi-mediated fluorination of arylboronic acids developed herein shows a broader scope and uses a readily available Bi–OTs species (**47**), in contrast to our previous methodology, which is based on the use of ligand scaffolds incorporating the −S(O)NCF_3_– unit.

**Table 2:**
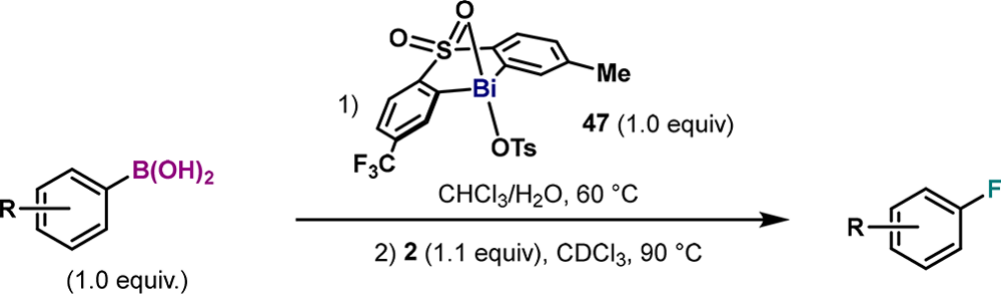

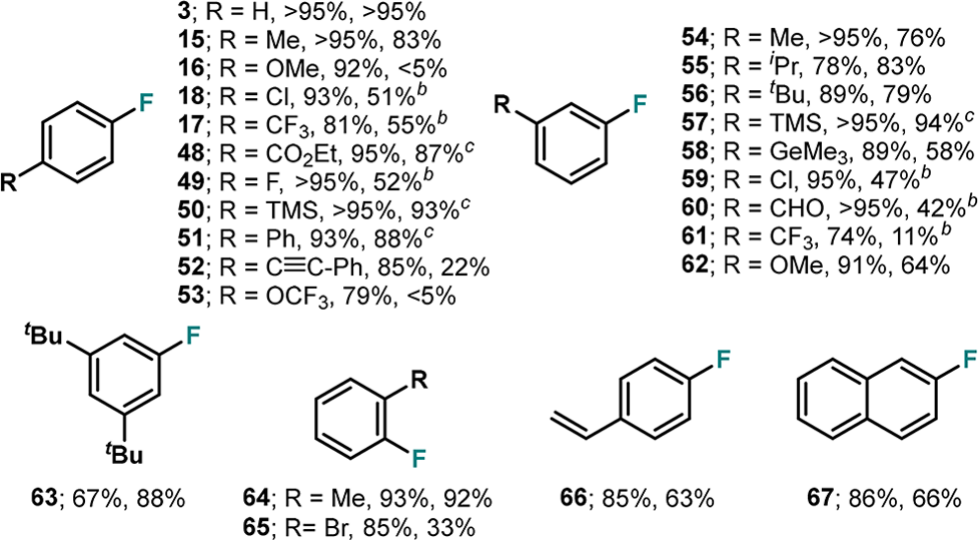
Bismuth-Mediated Two-Step Method for the Fluorination of Arylboronic Acids with **47**^a^

a Yields are given for step 1 (isolated) and step 2 (determined by ^19^F NMR using 1-fluoro-4-nitrobenzene as internal standard).

b Step 2 performed in the presence of 5.0 equiv of NaF at 110 °C.

c Isolated yield by preparative TLC. Products contain trace amounts (<5%) of protodeboronation byproducts.

After providing an improved stoichiometric method based on complex **47** for the fluorination of arylboronic acids, we focused our attention on transferring the benefits of sulfone-based complexes to high-valent Bi-catalyzed fluorination reactions. A major limitation of our previously reported method was the need of 3.0 equiv of arylboronic ester, making this transformation impractical for valuable substrates. Optimal conditions for catalytic fluorination from aryl boronic esters (76% of **3**) were found using sulfone-based catalyst **68** bearing two *meta*-CF_3_ groups, in combination with oxidant **2** (1.1 equiv) in CDCl_3_ (Table 3). Noteworthy, *arylboronic esters could be utilized as limiting reagents*, *and most importantly, the reaction worked smoothly in the absence of base*. Indeed, analysis of the reaction crude after fluorobenzene formation revealed the presence of BF_3_ in solution, as well as in the headspace,^34^ which suggests that the BF_4_^–^ anion also acts as a fluoride source during the catalytic transformation. Under the optimized conditions, a variety of *para*-substituents could be accommodated in good yields, including alkyl groups (**14**, 85%; **15**, 65%), Ph (**51**, 73%), and TMS (**50**, 77%). In addition, arylboronic esters bearing *para*-EWG groups such as halogen atoms required the use of a base (5.0 equiv of NaF) and higher reaction temperatures to yield arylfluorides in moderate yields (**18**, **69**, and **70**). Arylboronic esters containing *meta*-substituents were also tolerated (**54**, **59**, **62**, and **71**), although strong *meta*-EWG inhibited the formation of fluoroarenes (**61** and **72**) even in the presence of NaF. *ortho*-Substitution was also well accommodated (**64** and **73**), albeit low yields were obtained with *o*-Br (**65**) and *o*-CO_2_Me (**74**) groups. Interestingly, this catalytic system allowed the introduction of several strong *para*-EWG groups such as CF_3_ (**17**, 56%), CO_2_Me (**75**, 56%), Br (**76**, 69%), and SO_2_Me (**77**, 35%), which were previously shown to inhibit reactivity when using sulfoximine-based Bi-catalysts.^32b^ In fact, the use of sulfone-based catalyst **68** also boded well with sterically hindered substrates (**78**, 64%) and alkynyl groups (**79**, 49%), showing a wide scope and practicality. Although these protocols produce high yields of fluorinated arenes, isolation of these compounds in pure form without traces of Ar–H becomes tedious, requiring HPLC separations which, in some cases, result in low yields (**14** and **51**, Table 3).

**Table 3:**
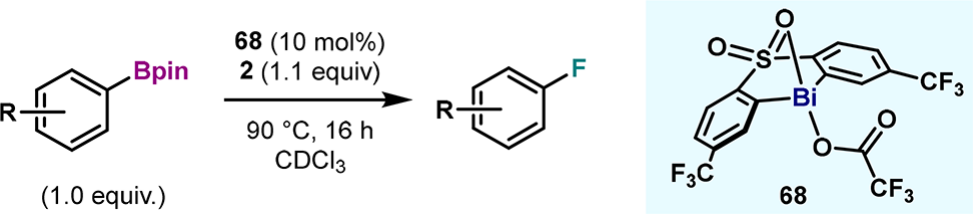

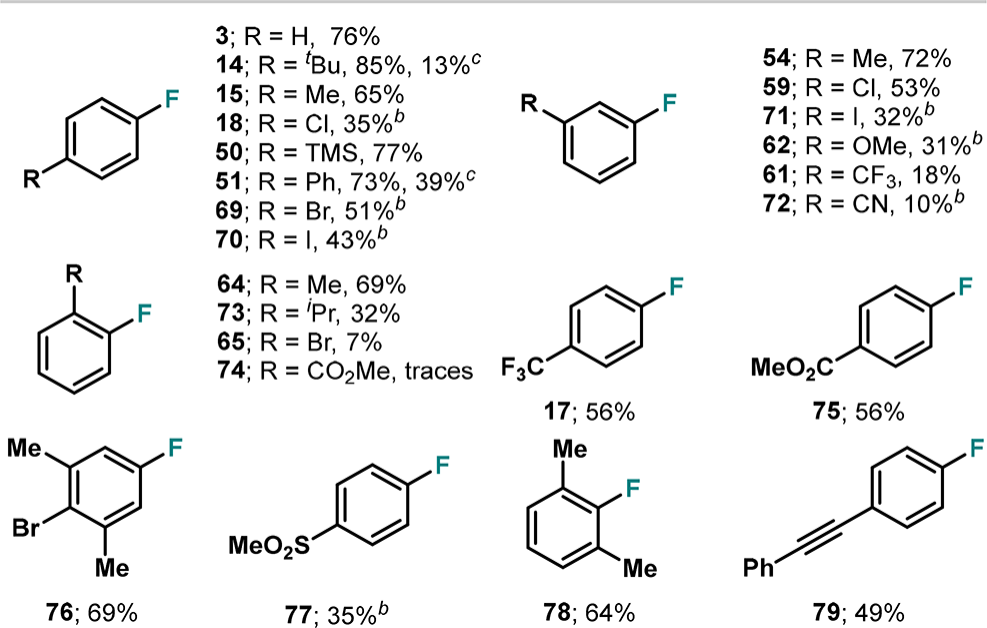
Bismuth-Catalyzed Fluorination of Arylboronic Esters with Sulfone-Based Catalyst **68**^a^

a Yields determined by ^19^F NMR using 1-fluoro-4-nitrobenzene as internal standard.

b Reaction performed in the presence of 5.0 equiv of NaF.

c Isolated yield by preparative HPLC.

## Conclusion

**Figure 18:**
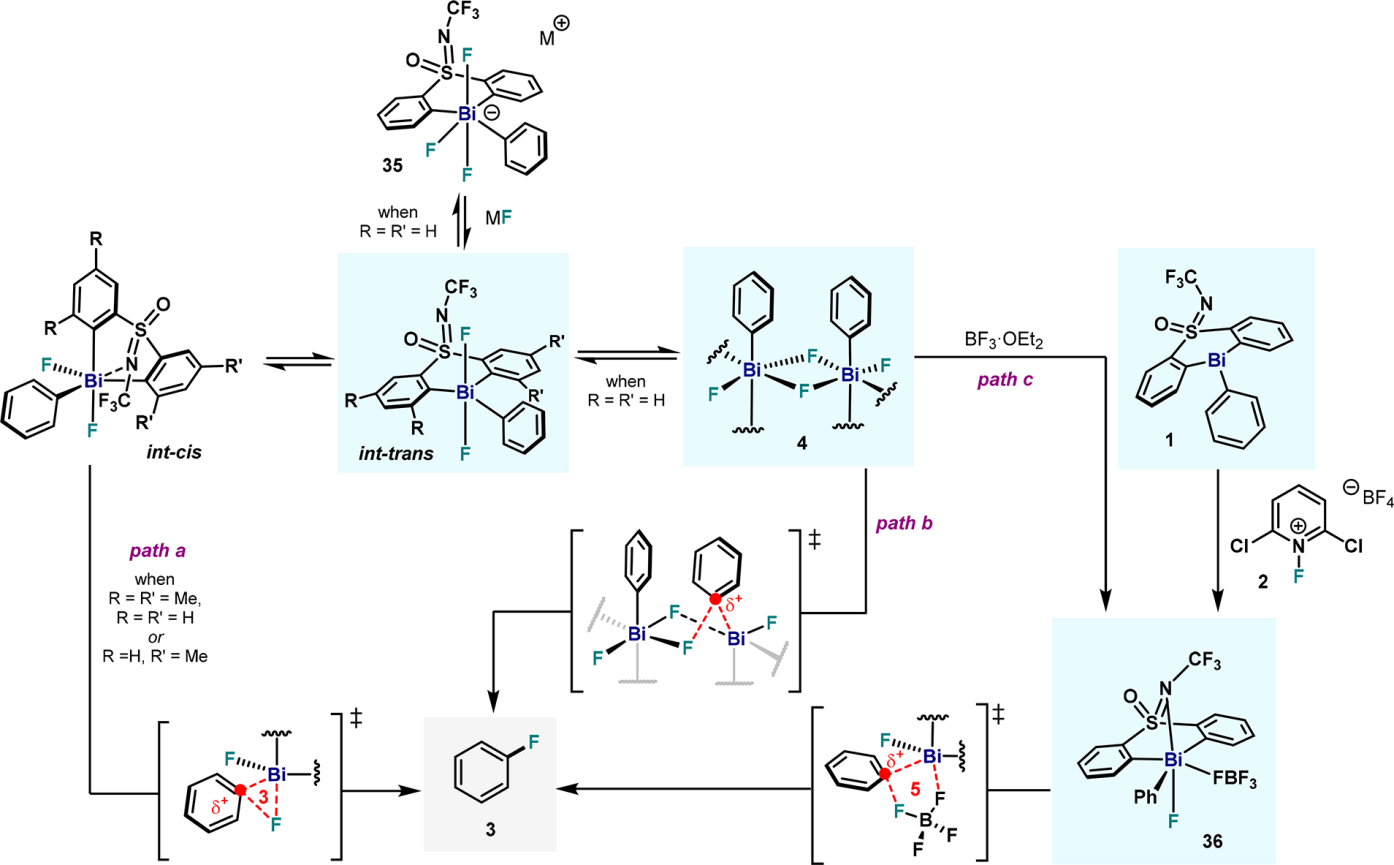
Overview of the C(sp^2^)–F reductive elimination from Bi(V).

We provide herein a mechanism of the reductive elimination of aryl–F bonds from neutral triarylbismuth difluorides as well as cationic fluorobismuthonium species (Figure 18). Solid-state (XRD) and spectroscopic characterization in solution (1D and 2D NMR) suggests that **4** presents a dimeric structure and undergoes fast dynamic processes in solution. Evaluation of the electronic and steric effects on the pendant aryl ligand revealed that *para*-EWG enhances the rate of fluorobenzene (**3**) formation. Installation of Me groups—*ortho* with respect to the Bi center—in the sulfoximine ligand scaffold permitted the synthesis and characterization of monomeric TBP aryl Bi(V) difluorides **25** and **26**. In contrast to model complex **4**, these compounds have been characterized as *trans*-difluoride monomers in solid state and in solution. Evaluation of electronic effects affecting the reductive elimination of fluorobenzene revealed analogous effects on the reactive aryl compared to **4**. Yet, the Eyring plot revealed a Δ*S*^⧧^ ≈ −6 cal·mol^–1^ K^–1^ for monomeric **25** and **26**, which is in stark contrast to the Δ*S*^⧧^ ≈ −34 cal·mol^–1^ K^–1^ for complex **4**. Evaluation of the effect of external fluoride anions in the reductive elimination of **4** revealed the formation of anionic species **35**, which has a detrimental effect on fluorobenzene formation. Theoretical studies of the C–F bond formation from dimeric and monomeric neutral difluorides showed kinetic barriers in agreement with experimentally determined parameters. Indeed, for species **4**, the aryl–F bond formation from neutral Bi(V) difluoride centers is postulated to proceed through a dimeric TS, albeit a reductive elimination event from monomeric species cannot be disregarded.

Evaluation of the C–F bond formation from cationic fluorobismuthonium species was also assessed. Isolation of hexafluorosilicate compound **37** allowed solid-state characterization of the fluorobismuhtonium species. Electronic modulations on the pendant aryl and the ligand scaffold suggested a highly polarized TS, consistent with the cationic nature of this complex. DFT studies of cationic species **36** unveiled the BF_4_^–^ anion as the true fluoride source, forging the C–F bond through a five-membered TS. Reaction of **1** with the milder 1-fluoro-2,6-pyridinium tetrafluoroborate (**2**) also delivered a high-valent Bi(V) species **36**, further supporting the involvement of fluorobismuthonium intermediates. With this mechanistic picture, re-evaluation of the ligand features led to the development of improved stoichiometric and catalytic fluorination reactions of arylboronic acid derivatives, using a simpler and easy to handle Bi catalyst. This second-generation fluorination has been successfully applied to >40 substrates, thus improving the yields over our previously reported methodologies. Overall, the detailed mechanistic investigation provided herein enabled the identification of the key parameters and limitations of the C–F bond-forming step from Bi(V) centers, revealing different pathways between neutral and cationic species. Finally, this article illustrates that by means of a mechanistic understanding, a rational design for an improved methodology for the fluorination of organic compounds based on Bi could be established.

## Supporting Information Available

Complete experimental details, NMR, computational details and crystallographic data (PDF)
