# Supplementary material for: Mechanism of the Aryl–F Bond-Forming Step from Bi(V) Fluorides
Source: J Am Chem Soc. 2022 Aug 3;144(32):14489–504. doi: 10.1021/jacs.2c01072 (PMC9394462; doi:10.1021/jacs.2c01072)
Supplement: Supplementary file 1 — ja2c01072_si_001.pdf [file ja2c01072_si_001.pdf]

Supporting Information

Mechanism of the Aryl–F Bond-Forming Step from Bi(V) Fluorides

Oriol Planas, Vytautas Peciukenas, Markus Leutzsch, Nils Nöthling, Dimitrios A. Pantazis and Josep Cornella*

Max-Planck-Institut für Kohlenforschung, Kaiser-Wilhelm-Platz 1, Mülheim an der Ruhr, 45470, Germany.

*corresponding author: cornella@kofo.mpg.de

Table of Contents

1. General methods 4
   1.1 Experimental methods 4
2. Synthesis of Bi(III) compounds 5
   2.1 Synthesis of arylbismines via dilithiation 5
   2.2 Synthesis of arylbismines via transmetalation with arylboronic acids 19
3. Synthesis of Bi(V) compounds 48
   3.1 General procedure for the synthesis of bismuth(V) species 49
   3.2 Aggregation studies of **4**, **5**, **25** and **26** 70
4. Reactivity studies of neutral σ-aryl Bi(V) fluorides 78
   4.1 Reactivity of model complex **4** 78
   4.2 Hammett analysis from Bi(V) complexes with *p*-substituted arenes 91
   4.3 Hammett analysis from Bi(V) complexes with *m*-substituted arenes 93
   4.4 Hammett analysis from Bi(V) complexes with *o*-substituted arenes 96
   4.5 Hammett analysis from Bi(V) complexes with substituted backbone 98
   4.6 Influence of ligand substitution 101
   4.7 Reductive elimination from monomeric complex **25** 104
   4.8 Reductive elimination from **26** 114
   4.9 Addition of fluoride sources 123
5. Reductive elimination from fluorobismuthonium σ-aryl Bi(V) fluoride complexes 134
   5.1 Synthesis and characterization of **37** 134
   5.2 Reductive elimination from **37** 135
   5.3 Reactivity of **4** with Lewis acids 136
   5.4 Hammett (ligand backbone) from cationic Bi(V) complexes 137
   5.5 Hammett (pendant aryl) from cationic Bi(V) complexes 140
   5.6 Reductive elimination from **180** 145
   5.7 Reductive elimination from **182** 149
6. Reactivity with 1-fluoro-2,6-dichloropyridinium tetrafluoroborate (**2**) 152
   6.1 Evidences for the formation of Bi(V) species with **2** 152
   6.2 Influence of the ligand scaffold in the oxidation/reductive elimination sequence 159
   6.3 Scope of the oxidation and reductive elimination of arylfluorides 160
7. Catalytic fluorination 167
   7.1 Role of base in catalytic fluorination with **38** as catalyst 167
   7.2 Optimization for Bi-catalyzed fluorination 169
   7.3 Crude mixture analysis after Bi-catalyzed fluorination of boron based nucleophiles 171
   7.4 Identification of byproducts of Bi-catalyzed fluorination of boron-based nucleophiles 175
   7.5 Scope of Bi-catalyzed fluorination of boron-based nucleophiles 182
8. Crystallographic data 190
   8.1 Single crystal structure analysis of **4·(CH_3CN)$_2$** 190
   8.2 Single crystal structure analysis of **24a** 196
   8.3 Single crystal structure analysis of **24b·$CHCl_3$** 201
   8.4 Single crystal structure analysis of **26·($CHCl_3$)$_2$** 209
   8.5 Single crystal structure analysis of **28·$CHCl_3$** 215
   8.6 Single crystal structure analysis of **37·$CHCl_3$** 221
   8.7 Single crystal structure analysis of **42** 227
   8.8 Single crystal structure analysis of **47·CH_2Cl_2** 234

8.9 Single crystal structure analysis of **82**....240
8.10 Single crystal structure analysis of **83**....247
8.11 Single crystal structure analysis of **84**....252
8.12 Single crystal structure analysis of **87**....257
8.13 Single crystal structure analysis of **94**....265
8.14 Single crystal structure analysis of **95**....270
8.15 Single crystal structure analysis of **96a**....276
8.16 Single crystal structure analysis of **96b**....284
8.17 Single crystal structure analysis of **97**....291
8.18 Single crystal structure analysis of **108**....296
8.19 Single crystal structure analysis of **110**....305
8.20 Single crystal structure analysis of **146**....310
8.21 Single crystal structure analysis of **161·$CHCl_3$**....317
8.22 Single crystal structure analysis of **162·$(CHCl_3)_2$**....322
8.23 Single crystal structure analysis of **175·$CHCl_3$**....328
9. Computational details....335
9.1 Benchmarking of DFT methods with XRD structure of dimeric species **4**....336
9.2 Reductive elimination from dimeric species **4**....337
9.3 Reductive elimination from monomeric species ***int-cis*** and ***int-trans***....341
9.4 Reductive elimination from monomeric species **26**....351
9.5 Reductive elimination from fluorobismuthonium species ***cis*-36**....358
9.6 XYZ coordinates of DFT optimized structures....371
10. NMR spectra of Bi(III) compounds....389
11. NMR spectra of Bi(V) compounds....595
12. Crude ^{19}F NMR spectra for stoichiometric fluorination of Bi(III) compounds....792
13. NMR spectra for isolated fluoroarenes from stoichiometric reactions....817
14. Crude ^{19}F NMR spectra for catalytic fluorination of arylboronic esters....829
15. NMR spectra for isolated fluoroarenes from catalytic reactions....855
16. References....861

1. General methods

1.1 Experimental methods

Unless otherwise stated, all manipulations were performed using standard Schlenk techniques under dry argon (Ar) in flame-dried glassware. Anhydrous solvents were distilled from appropriate drying agents and were transferred under Ar: tetrahydrofuran (Na/K), chloroform (CaH_2) and chloroform-*d* (MS), acetonitrile (SPS) and acetonitrile-d_3 (MS), acetone, toluene (Na/K), dichloromethane (CaH_2/P_4O_{10}) and dimethylformamide (MS). Commercially available xenon difluoride obtained from STREM, 1-fluoro-2,6-dichloropyridinium tetrafluoroborate, all the arylboronic acids and boronic esters utilized in this work were obtained from Sigma-Aldrich or were synthesized through reported procedures.[1] Sulfone and sulfoximine ligands were synthesized through reported procedures.[2-11] Flash chromatography: Merck silica gel 60 (40-63 µm). ESI-MS: ESQ 3000 (Bruker). Preparative TLC plates: PLC Silica gel 60 F_{254}, 1 mm, 20x20 cm (Sigma-Aldrich). High-resolution mass determinations: Bruker APEX III FT-MS (7 T magnet) or MAT 95 (Finnigan). Melting points were measured with an EZ-Melt Automated Melting Point Apparatus from Standford Research Systems. IR spectra were recorded using Bruker ALPHA FT-IR spectrometer under ambient conditions with 16 scans on a diamond ATR unit. NMR spectra were recorded using 300 MHz Bruker Avance III and 500 MHz Bruker Avance III NMR spectrometers. VT-NMR recordings were conducted using 600 MHz Bruker Avance III or 400 MHz Bruker Avance III HD NMR spectrometers. ^{1}H NMR spectra (300.13 MHz, 500.1 Hz) were referenced to the residual protons of the deuterated solvent,[12] and are reported to tetramethylsilane (δ_{TMS} = 0 ppm), chloroform-*d* (δ = 7.26 ppm) or acetonitrile-d_3 (δ = 1.94 ppm). ^{13}C{^{1}H} NMR spectra (75.47 MHz, 125 MHz) were referenced internally to the D-coupled ^{13}C resonances of the NMR solvent and are reported to tetramethylsilane (δ_{TMS} = 0 ppm), chloroform-*d* (δ = 77.16 ppm) or acetonitrile-d_3 (δ = 1.32 ppm). ^{19}F{^{1}H} NMR spectra (282 MHz, 471 MHz) are reported relative to the ^{19}F resonances of $CFCl_3$. Chemical shifts (δ) are given in ppm, relative to deuterated solvent residual peak, and coupling constants (*J*) provided in Hz. For bismuth complexes **42** and **164**, ^{13}C NMR spectra were acquired with a Bruker BB-1H/19F TBO Probe with inverse gated decoupling. For ^{1}H NMR waltz16 was used for decoupling. For ^{19}F the decoupling scheme bi_p5m4sp_4sp.2 with adiabatic chirp pulses was used to ensure the broadband decoupling on ^{19}F. Sometimes small artifacts can be seen in the spectra. ^{19}F-HMQCs were acquired to show which ^{19}F signals correlate with the corresponding ^{13}C nuclei.

2. Synthesis of Bi(III) compounds

2.1 Synthesis of arylbismines via dilithiation

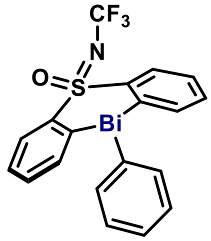

1

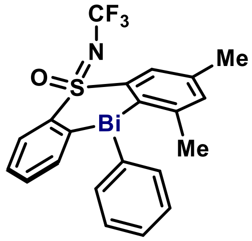

23

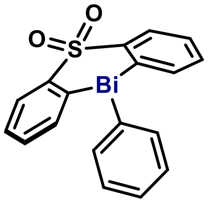

39

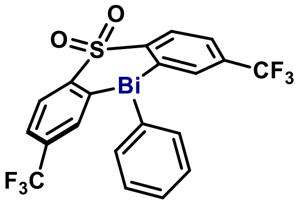

41

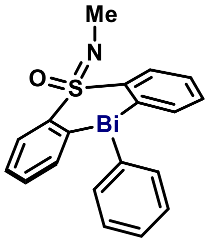

43

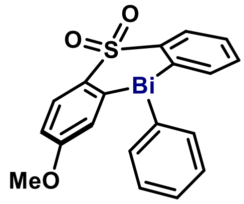

79

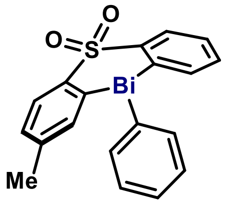

80

Phenylbismines **1**, **23**, **39**, **41**, **43**, **79** and **80** were synthesized according to previously reported protocols.[2-3] Arylbismines **24**, **40**, **42**, **44-46**, **81-96** were synthesized following the procedure described below.

General procedure for the synthesis of aryl bismines.[13]

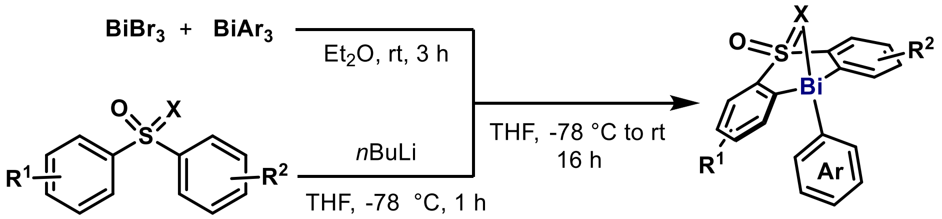

A Schlenk flask equipped with a stir bar was charged with $BiBr_3$ (0.66 equiv), $BiAr_3$ (0.33 equiv) and Et_2O (8 mL) under an Ar atmosphere and stirred at room temperature for 3 h. THF (10 mL) was added to completely dissolve the yellow precipitate, and this solution was added drop-wise to a –78 °C dilithiated ligand solution. The dilithiated ligand was prepared in a separate Schlenk flask from the corresponding ligand (1.0 equiv), dissolved in THF (30 mL), with *n*-butyl lithium (2.6 M in hexanes, 2.1 equiv) at –78 °C for 1 h. Following the addition of the bismuth compound to the dilithiated ligand, the reaction was stirred overnight, slowly warming to room temperature. The reaction was quenched by the addition of brine (20 mL), extracted with ethyl acetate (40 mL), and re-extracted with CH_2Cl_2 (2 × 50 mL). The combined organics were dried

over Na_2SO_4, filtered and concentrated to give a thick yellow residue. The crude material was purified by flash chromatography (silica gel, Hexanes/Ethyl acetate) to give the corresponding arylbismine as a white solid.

10-phenyl-5-((trifluoromethyl)imino)-5,10-dihydro-5λ^4-dibenzo[b,e][1,4]thiabismine 5-oxide (1): NMR, HRMS and m.p. characterization has been previously reported.[2]

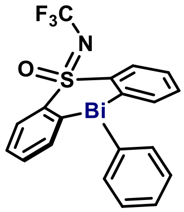

1

IR: 3046*w*, 1561*w*, 1474*w*, 1443*w*, 1430*w*, 1293*w*, 1267*m*, 1231*s*, 1142*m*, 1092*s*, 1083*s*, 1057*m*, 1029*w*, 1012*w*, 996*w*, 964*w*, 905*w*, 847*w*, 801*w*, 771*m*, 741*m*, 723*s*, 711*m*, 692*m*, 674*w*, 636*w*, 616*w*, 583*s*, 555*s*, 529*m*, 491*m*, 461*m*, 440*m*, 411*w*.

1,3-dimethyl-10-phenyl-5-((trifluoromethyl)imino)-5,10-dihydro-5λ^4-dibenzo[b,e][1,4]thiabismine 5-oxide (23): NMR, HRMS and m.p. characterization has been previously reported.[2]

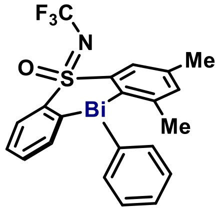

23

IR: 3059*w*, 3038*w*, 2981*w*, 2955*w*, 2922*w*, 1591*w*, 1567*w*, 1474*w*, 1445*w*, 1431*w*, 1382*w*, 1273*s*, 1232*s*, 1141*m*, 1136*m*, 1081*s*, 1057*m*, 1032*w*, 1012*w*, 995*w*, 870*w*, 851*w*, 788*w*, 757*m*, 740*w*, 727*s*, 707*w*, 694*m*, 673*w*, 623*w*, 594*s*, 568*m*, 533*m*, 505*w*, 491*w*, 481*w*, 447*w*, 438*w*, 418*w*, 408*w*.

1,3,7,9-tetramethyl-10-phenyl-5-((trifluoromethyl)imino)-5,10-dihydro-5λ^4-dibenzo[b,e][1,4]thiabismine 5-oxide (24): Following the general procedure, using 0.83 g of the corresponding sulfone ligand, **24** was obtained in 71% overall yield (Eluent: Hexanes/Ethyl acetate = 9/1, low pressure). **24a** was isolated as white solid in 52% yield and **24b** was isolated as white solid in 19% yield. Crystals suitable for XRD analysis were obtained by slow evaporation of a concentrated solution of **24a** in a mixture of CH_2Cl_2/*iso*-hexane (5:1) and by slow pentane diffusion into a concentrated solution of **24b** in $CHCl_3$ at 4 °C.

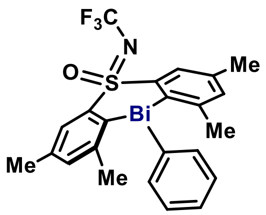

24a

^{1}H NMR - (300 MHz, $CDCl_3$): δ 8.19 (s, 2H), 7.70 – 7.67 (m, 2H), 7.26 – 7.24 (m, 3H), 7.14 (s, 2H), 2.38 (s, 6H), 1.89 (s, 6H).

^{13}C NMR - (75 MHz, $CDCl_3$): δ 161.2, 153.9, 145.1, 139.0, 138.9, 138.5, 136.2, 130.8, 127.8, 125.8, 121.7 (q, *J* = 272.2 Hz), 25.9, 21.1.

^{19}F NMR - (282 MHz, $CDCl_3$): δ –41.8 (s, 3F).

HRMS (ESI, m/z): calc'd for $C_{23}H_{21}BiF_3SNONa^+$ $[M+Na]^+$ 648.0992; found 648.0989.

m.p. (°C): 192.1 – 193.3.

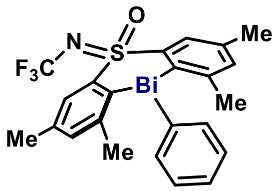

24b

^{1}H NMR - (300 MHz, $CDCl_3$): δ 8.23 (s, 2H), 7.64 (bs, 2H), 7.27 – 7.25 (m, 3H), 7.10 (s, 2H), 2.38 (s, 6H), 1. 88 (s, 6H).

^{13}C NMR - (75 MHz, $CDCl_3$): δ 161.6, 153.5, 145.1, 141.5, 138.7, 138.3, 135.9, 130.8, 127.9, 125.9, 123.2 (q, *J* = 272.1 Hz), 25.9, 21.2.

^{19}F NMR - (282 MHz, $CDCl_3$): δ –38.1 (s, 3F).

HRMS (ESI, m/z): calc'd for $C_{23}H_{22}BiF_3SNO^+$ $[M+H]^+$ 626.1173; found 626.1173.

m.p. (°C): 202.7 – 204.1.

IR: 3057*w*, 3036*w*, 3011*w*, 2964*w*, 2920*w*, 2859*w*, 1591*w*, 1569*w*, 1474*w*, 1442*w*, 1430*w*, 1379*w*, 1291*s*, 1254*m*, 1213*m*, 1202*m*, 1154*s*, 1137*m*, 1084*s*, 1063*m*, 1013*w*, 996*w*, 954*w*, 907*w*, 863*m*, 843*m*, 785*m*, 728*m*, 696*s*, 616*m*, 604*s*, 581*m*, 561*m*, 543*w*, 512*w*, 488*w*, 474*m*, 446*w*, 432*w*.

2-methyl-10-phenyl-8-(trifluoromethyl)-10H-dibenzo[b,e][1,4]thiabismine 5,5-dioxide (40):

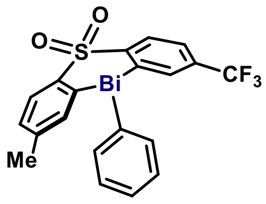

40

Following the general procedure, using 2.1 g of the corresponding sulfone ligand, **40** was obtained in 65% yield as a white solid (Eluent: Hexanes/Ethyl acetate = 4/1).

^{1}H NMR - (300 MHz, $CDCl_3$): δ 8.44 (d, *J* = 8.1 Hz, 1H), 8.29 (d, *J* = 7.9 Hz, 1H), 8.06 (s, 1H), 7.78 (dd, *J* = 7.8, 1.4 Hz, 2H), 7.72 (s, 1H), 7.66 (d, *J* = 8.1 Hz, 1H), 7.53 – 7.35 (m, 3H), 7.23 (d, *J* = 7.9 Hz, 1H), 2.28 (s, 3H).

^{13}C NMR - (75 MHz, $CDCl_3$): δ 166.4, 159.7, 159.2, 145.8, 144.8, 138.7, 138.4, 138.1, 134.7 (q, *J* = 32.9 Hz), 134.5 (q, *J* = 3.9 Hz), 131.3, 129.2, 129.1, 127.7, 126.9, 125.4 (q, *J* = 3.9 Hz), 123.4 (q, *J* = 272.5 Hz), 21.6.

^{19}F NMR - (282 MHz, $CDCl_3$): δ –62.9 (s, 3F).

HRMS (ESI, m/z): calc'd for $C_{20}H_{14}BiSO_2F_3Na^+$ [M+Na]$^+$ 607.0362; found 607.0363.

m.p. (°C): 146.8 – 148.2.

5-((perfluoroethyl)imino)-10-phenyl-5,10-dihydro-5l4-dibenzo[b,e][1,4]thiabismine 5-oxide (42). Following the general procedure, using 540 mg of the corresponding sulfone ligand, **42** was obtained in 69% yield as white solid (Eluent: Hexanes/Ethyl acetate = 4/1). Crystals suitable for XRD analysis were obtained by slow evaporation of a concentrated solution of **42** in a mixture of CH_2Cl_2/*iso*-hexane (5:1).

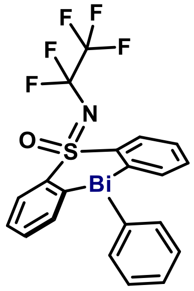

42

^{1}H NMR - (600 MHz, $CDCl_3$): δ 8.50 – 8.43 (m, 2H), 7.90 (dd, J = 7.3, 0.9 Hz, 2H), 7.76 (dd, J = 7.9, 1.4 Hz, 2H), 7.49 – 7.34 (m, 7H).

^{13}C NMR - (151 MHz, $CDCl_3$): δ 167.2, 158.3, 140.8, 138.9, 138.0, 134.0, 131.1, 128.8, 128.6, 127.8, 118.0 (qt, *J* = 283.1, 46.3 Hz), 114.0 (tq, *J* = 266.5, 40.7 Hz).

^{19}F NMR - (564 MHz, $CDCl_3$): δ –78.2 (s, 2F), –84.12 (s, 3F).

HRMS (ESI, m/z): calc'd for $C_{20}H_{13}BiF_5SNONa^+$ [M+Na]$^+$ 642.0334; found 642.0331.

m.p. (°C): 150.7– 152.0.

5-((4-methoxyphenyl)imino)-10-phenyl-5,10-dihydro-5λ⁴-dibenzo[b,e][1,4]thiabismine 5-oxide (44). Following the general procedure, using 327 mg of the corresponding sulfone ligand, **44** was obtained in 57% yield as white solid (Eluent: Hexanes/Ethyl acetate = 4/1).

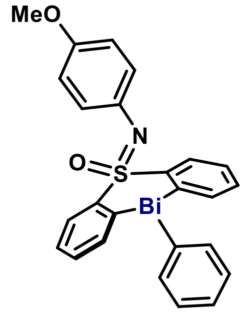

44

^{1}H NMR - (300 MHz, $CDCl_3$): δ 8.48 (dd, *J* = 7.8, 1.1 Hz, 2H), 7.82 (dd, *J* = 7.2, 1.0 Hz, 2H), 7.77 (dd, *J* = 7.7, 1.5 Hz, 2H), 7.49 – 7.28 (m, 7H), 7.08 – 6.96 (m, 2H), 6.71 – 6.60 (m, 2H), 3.68 (s, 3H).

^{13}C NMR - (75 MHz, $CDCl_3$): δ 168.4, 158.8, 155.0, 140.4, 138.8, 137.8, 137.1, 133.3, 130.9, 129.6, 128.4, 128.2, 123.9, 114.4, 55.4.

HRMS (ESI, m/z): calc'd for $C_{25}H_{21}BiSNO_2^+$ [M+H]$^+$ 608.1097; found 608.1096.

m.p. (°C): 106.2 decomposition

10-phenyl-5-(phenylimino)-5,10-dihydro-5λ⁴-dibenzo[b,e][1,4]thiabismine 5-oxide (45). Following the general procedure, using 439 mg of the corresponding sulfone ligand, **45** was obtained in 28% yield as white solid (Eluent: Hexanes/Ethyl acetate = 4/1).

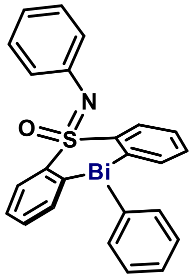

45

^{1}H NMR - (300 MHz, $CDCl_3$): δ 8.50 (dd, *J* = 7.8, 1.0 Hz, 2H), 7.83 (dd, *J* = 7.3, 0.9 Hz, 2H), 7.77 (dd, *J* = 7.9, 1.4 Hz, 2H), 7.47 – 7.30 (m, 7H), 7.10 (d, *J* = 6.5 Hz, 4H), 6.89 – 6.81 (m, 1H).

^{13}C NMR - (75 MHz, $CDCl_3$): δ 168.6, 158.9, 144.0, 140.3, 138.8, 137.9, 133.4, 130.9, 129.5, 129.0, 128.5, 128.2, 123.1, 122.1.

HRMS (ESI, m/z): calc'd for $C_{24}H_{19}BiSNO^+$ [M+H]$^+$ 578.0991; found 578.0989.

m.p. (°C): 209.0 – 211.4.

5-((4-(pentafluoro-λ^6-sulfanyl)phenyl)imino)-10-phenyl-5,10-dihydro-5λ^4-dibenzo[b,e][1,4]thiabismine 5-oxide (46). Following the general procedure, using 764 mg of the corresponding sulfone ligand, **46** was obtained in 61% yield as white solid (Eluent: Hexanes/Ethyl acetate = 4/1).

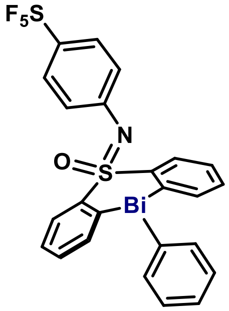

46

^{1}H NMR - (300 MHz, $CDCl_3$): δ 8.50 (dd, J = 7.8, 1.0 Hz, 2H), 7.86 (dd, J = 7.2, 1.0 Hz, 2H), 7.77 (dd, J = 7.8, 1.5 Hz, 2H), 7.51 – 7.33 (m, 9H), 7.07 (d, J = 9.0 Hz, 2H).

^{13}C NMR - (75 MHz, $CDCl_3$): δ 168.2, 158.6, 147.8, 147.5, 139.8, 138.8, 138.1, 133.7, 131.0, 129.3, 128.7, 128.4, 126.9 (quint, J = 32.6 Hz), 122.2.

^{19}F NMR - (282 MHz, $CDCl_3$): δ 86.6 (quint, J = 149.4 Hz, 1F), 64.03 (d, J = 149.4 Hz, 4F).

HRMS (ESI, m/z): calc'd for $C_{24}H_{18}BiF_5S_2NO^+$ $[M+H]^+$ 704.0554; found 704.0554.

m.p. (°C): 187.3 – 188.8.

2,8-di-tert-butyl-10-phenyl-10H-dibenzo[b,e][1,4]thiabismine 5,5-dioxide (81): Following the general procedure using 1.8 g of the corresponding sulfone ligand, **81** was obtained in 54% yield as a white solid (Eluent: Hexanes/Ethyl acetate = 4/1).

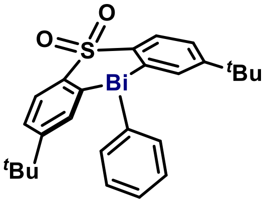

81

^{1}H NMR - (300 MHz, $CDCl_3$): δ 8.27 (d, J = 8.2 Hz, 2H), 7.89 (d, J = 1.8 Hz, 2H), 7.79 (dd, J = 7.8, 1.5 Hz, 2H), 7.46 – 7.31 (m, 5H), 1.16 (s, 18H).

^{13}C NMR - (75 MHz, $CDCl_3$): δ 167.0, 158.5, 156.4, 139.2, 138.7, 135.3, 130.6, 128.5, 126.8, 124.9, 35.2, 31.1.

HRMS (ESI, m/z): calc'd for $C_{26}H_{29}BiSO_2Na^+$ $[M+Na]^+$ 637.1584; found 637.1585.

m.p. (°C): 288.7– 289.9.

1,3-dimethyl-10-phenyl-10H-dibenzo[b,e][1,4]thiabismine 5,5-dioxide (82): Following the general procedure, using 0.7 g of the corresponding sulfone ligand, **82** was obtained in 61% yield as white solid (Eluent: Hexanes/Ethyl acetate = 4/1). Crystals suitable for XRD analysis were obtained by slow evaporation of a concentrated solution of **82** in a mixture of CH_2Cl_2/*iso*-hexane (5:1).

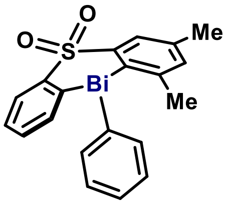

82

^{1}H NMR - (300 MHz, $CDCl_3$): δ 8.38 (dd, J = 7.6, 1.3 Hz, 1H), 8.12 (s, 1H), 7.80 – 7.68 (m, 3H), 7.41 – 7.22 (m, 5H), 7.15 (s, 1H), 2.36 (s, 3H), 1.95 (s, 3H).

^{13}C NMR - (75 MHz, $CDCl_3$): δ 163.2, 157.5, 155.4, 144.7, 141.9, 141.3, 138.7, 138.6, 138.1, 135.3, 133.5, 130.8, 128.3, 126.5, 125.8, 26.3, 21.0.

One carbon signal is not observed.

HRMS (ESI, m/z): calc'd for $C_{20}H_{17}BiSO_2Na^+$ $[M+Na]^+$ 553.0645; found 553.0645.

m.p. (°C): 185.2 – 186.6.

1,3,7,9-tetramethyl-10-phenyl-10H-dibenzo[b,e][1,4]thiabismine 5,5-dioxide (83): Following the general procedure, using 0.83 g of the corresponding sulfone ligand, **83** was obtained in 45% yield as white solid (Eluent: Hexanes/Ethyl acetate = 4/1). Crystals suitable for XRD analysis were obtained by slow evaporation of a concentrated solution of **83** in a mixture of CH_2Cl_2/*iso*-hexane (5:1).

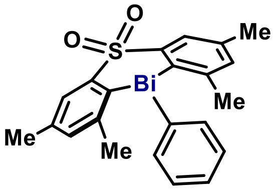

83

^{1}H NMR - (300 MHz, $CDCl_3$): δ 8.18 (s, 2H), 7.75 – 7.71 (m, 2H), 7.29 – 7.27 (m, 3H), 7.14 (s, 2H), 2.40 (s, 6H), 1.92 (s, 6H).

^{13}C NMR - (75 MHz, $CDCl_3$): δ 159.2, 154.1, 144.8, 141.2, 138.6, 138.5, 135.7, 130.7, 127.7, 125.0, 25.7, 21.0.

HRMS (ESI, m/z): calc'd for $C_{22}H_{21}BiSO_2Na^+$ $[M+Na]^+$ 581.0958; found 581.0963.

m.p. (°C): 269.7 – 271.2.

2,8-dimethyl-10-phenyl-5-((trifluoromethyl)imino)-5,10-dihydro-5λ^4-

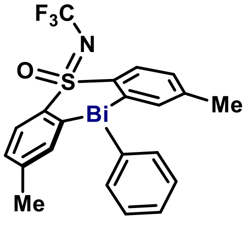

84

dibenzo[b,e][1,4]thiabismine 5-oxide (84): Following the general procedure, using 0.83 g of the corresponding sulfone ligand, **84** was obtained in 76% yield as white solid (Eluent: Hexanes/Ethyl acetate = 4/1). Crystals suitable for XRD analysis were obtained by slow evaporation of a concentrated solution of **84** in a mixture of CH_2Cl_2/*iso*-hexane (5:1).

^{1}H NMR - (300 MHz, $CDCl_3$): δ 8.30 (d, *J* = 8.0 Hz, 2H), 7.78 (dd, *J* = 7.8, 1.5 Hz, 2H), 7.68 (s, 2H), 7.49 – 7.34 (m, 3H), 7.28 – 7.20 (m, 2H), 2.26 (s, 6H).

^{13}C NMR - (75 MHz, $CDCl_3$): δ 167.1, 157.9, 144.7, 138.8, 138.5, 137.2, 131.0, 129.2, 128.7, 127.7, 121.9 (q, *J* = 272.1 Hz), 21.5.

^{19}F NMR - (282 MHz, $CDCl_3$): δ –41.8 (s, 3F).

HRMS (ESI, m/z): calc'd for $C_{21}H_{17}BiF_3SNONa^+$ $[M+Na]^+$ 620.0679; found 620.0680.

m.p. (°C): 194.7 – 196.0.

2,8-di-tert-butyl-10-phenyl-5-((trifluoromethyl)imino)-5,10-dihydro-5λ^4-

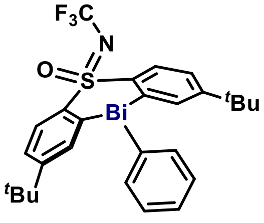

85

dibenzo[b,e][1,4]thiabismine 5-oxide (85): Following the general procedure, using 0.33 g of the corresponding sulfone ligand, **85** was obtained in 68% yield as white solid (Eluent: Hexanes/Ethyl acetate = 4/1).

^{1}H NMR - (300 MHz, $CDCl_3$): δ 8.33 (d, *J* = 8.3 Hz, 2H), 7.91 (d, *J* = 1.9 Hz, 2H), 7.84 – 7.74 (m, 2H), 7.50 – 7.31 (m, 5H), 1.16 (s, 18H).

^{13}C NMR - (75 MHz, $CDCl_3$): δ 167.6, 158.2, 157.1, 138.7, 137.2, 135.7, 130.8, 128.6, 127.6, 125.3, 121.8 (q, *J* = 272.1 Hz), 35.3, 31.1.

^{19}F NMR - (282 MHz, $CDCl_3$): δ –41.8 (s, 3F).

HRMS (ESI, m/z): calc'd for $C_{27}H_{29}BiF_3SNONa^+$ $[M+Na]^+$ 704.1618; found 704.1619.

m.p. (°C): 107.8 – 109.4.

IR: 3062*w*, 2962*w*, 2906*w*, 2868*w*, 1572*w*, 1554*w*, 1476*w*, 1430*w*, 1395*w*, 1379*w*, 1364*w*, 1294*m*, 1270*s*, 1237*s*, 1141*m*, 1090*s*, 1060*m*, 1014*w*, 997*w*, 897*w*, 849*w*, 833*w*, 797*m*, 775*w*, 725*m*, 695*m*, 687*m*, 656*w*, 629*m*, 613*s*, 580*w*, 563*w*, 529*m*, 520*m*, 495*w*, 474*w*, 436*w*, 411*w*.

2,8-difluoro-10-phenyl-5-((trifluoromethyl)imino)-5,10-dihydro-5λ⁴-

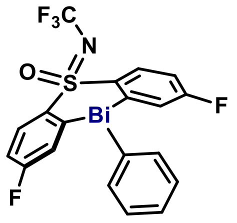

86

dibenzo[b,e][1,4]thiabismine 5-oxide (86): Following the general procedure, using 1.3 g of the corresponding sulfone ligand, **86** was obtained in 56% yield as white solid (Eluent: Hexanes/Ethyl acetate = 4/1).

^{1}H NMR - (300 MHz, $CDCl_3$): δ 8.44 (dd, *J* = 8.7, 4.6 Hz, 2H), 7.79 (dd, *J* = 7.9, 1.4 Hz, 2H), 7.62 (dd, *J* = 6.9, 2.5 Hz, 2H), 7.57 – 7.47 (m, 2H), 7.47 – 7.38 (m, 1H), 7.16 (td, *J* = 8.5, 2.5 Hz, 2H).

^{13}C NMR - (75 MHz, $CDCl_3$): δ 168.3, 164.9, 161.7, 138.7, 135.5 (d, *J* = 2.1 Hz), 131.6, 130.5 (d, *J* = 8.7 Hz), 129.3, 125.1 (d, *J* = 21.1 Hz), 121.6 (q, *J* = 272.1 Hz), 115.91 (d, *J* = 23.1 Hz).

^{19}F NMR - (282 MHz, $CDCl_3$): δ –42.1 (s, 3F), –105.1 (s, 2F).

HRMS (ESI, m/z): calc'd for $C_{19}H_{11}BiF_5SNONa^+$ $[M+Na]^+$ 628.0178; found 628.0180.

m.p. (°C): 142.3 – 143.7.

2,8-difluoro-10-phenyl-5-((trifluoromethyl)imino)-5,10-dihydro-5λ⁴-

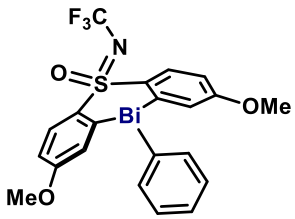

87

dibenzo[b,e][1,4]thiabismine 5-oxide (87): Following the general procedure, using 1.3 g of the corresponding sulfone ligand, **87** was obtained in 79% yield as white solid (Eluent: Hexanes/Ethyl acetate = 4/1). Crystals suitable for XRD analysis were obtained by slow evaporation of a concentrated solution of **87** in a mixture of CH_2Cl_2/*iso*-hexane (5:1).

^{1}H NMR - (300 MHz, $CDCl_3$): δ 8.34 (d, *J* = 8.7 Hz, 2H), 7.81 (dd, *J* = 7.9, 1.3 Hz, 2H), 7.53 – 7.31 (m, 5H), 6.90 (dd, *J* = 8.7, 2.5 Hz, 2H), 3.69 (s, 6H).

^{13}C NMR - (75 MHz, $CDCl_3$): δ 168.16, 163.99, 159.85, 138.77, 131.67, 131.07, 129.45, 128.79, 123.79, 121.8 (q, *J* = 272.1 Hz), 113.17, 55.72.

^{19}F NMR - (282 MHz, $CDCl_3$): δ –42.8 (s, 3F).

HRMS (ESI, m/z): calc'd for $C_{21}H_{17}BiF_3SNO_3Na^+$ $[M+Na]^+$ 652.0577; found 652.0579.

m.p. (°C): 133.1 – 134.6.

(S)-1,3-dimethyl-10-(p-tolyl)-5-((trifluoromethyl)imino)-5,10-dihydro-5λ⁴-dibenzo[b,e][1,4]thiabismine 5-oxide (88). Following the general procedure, using 470 mg of the corresponding sulfone ligand, **88** was obtained in 41% yield as white solid (Eluent: Hexanes/Ethyl acetate = 4/1).

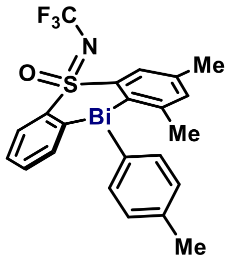

88

^{1}H NMR - (300 MHz, $CDCl_3$): δ 8.45 (dd, *J* = 7.8, 1.0 Hz, 1H), 8.18 (s, 1H), 7.79 (dd, *J* = 7.3, 0.9 Hz, 1H), 7.63 (d, *J* = 7.6 Hz, 2H), 7.44 (td, *J* = 7.6, 1.3 Hz, 1H), 7.34 (td, *J* = 7.4, 1.3 Hz, 1H), 7.21 (s, 1H), 7.17 (d, *J* = 7.4 Hz, 2H), 2.40 (s, 3H), 2.31 (s, 3H), 2.00 (s, 3H).

^{13}C NMR - (75 MHz, $CDCl_3$): δ 161.3, 157.2, 154.6, 145.2, 139.8, 139.4, 138.9, 138.8, 138.4, 138.2, 135.8, 133.8, 131.8, 128.6, 127.3, 126.4, 121.9 (q, *J* = 262.4 Hz), 26.5, 21.8, 21.1.

^{19}F NMR - (282 MHz, $CDCl_3$): δ –41.9 (s, 3F).

HRMS (ESI, m/z): calc'd for $C_{22}H_{19}BiF_3SNONa^+$ $[M+Na]^+$ 634.0836; found 634.0836.

m.p. (°C): 150.4 – 151.8.

(S)-1,3-dimethyl-5-((trifluoromethyl)imino)-10-(4-(trifluoromethyl)phenyl)-5,10-dihydro-5λ⁴-dibenzo[b,e][1,4]thiabismine 5-oxide (89). Following the general procedure, using 470 mg of the corresponding sulfone ligand, **89** was obtained in 45% yield as white solid (Eluent: Hexanes/Ethyl acetate = 4/1).

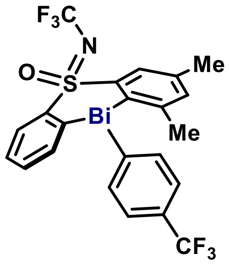

89

^{1}H NMR - (300 MHz, $CDCl_3$): δ 8.47 (dd, *J* = 7.8, 1.1 Hz, 1H), 8.20 (s, 1H), 7.88 (d, *J* = 7.5 Hz, 2H), 7.74 (dd, *J* = 7.3, 1.0 Hz, 1H), 7.56 (d, *J* = 7.8 Hz, 2H), 7.48 (td, *J* = 7.6, 1.2 Hz, 1H), 7.37 (td, *J* = 7.4, 1.3 Hz, 1H), 7.25 (s, 1H), 2.41 (s, 3H), 1.97 (s, 3H).

^{13}C NMR - (75 MHz, $CDCl_3$): δ 169.2, 157.5, 155.1, 144.8, 139.8, 139.5, 139.4, 139.1, 138.2, 136.2, 134.2, 130.4 (q, *J* = 32.5 Hz), 128.9, 127.6, 127.3 (q, *J* = 3.8 Hz), 126.7, 124.2 (q, *J* = 270.4 Hz), 121.6 (q, *J* = 269.1 Hz), 26.5, 21.1.

^{19}F NMR - (282 MHz, $CDCl_3$): δ –41.9 (s, 3F), –62.8 (s, 3F).

HRMS (ESI, m/z): calc'd for $C_{22}H_{16}BiF_6SNONa^+$ $[M+Na]^+$ 688.0553; found 688.0553.

m.p. (°C): 187.4 – 188.8.

10-(4-methoxyphenyl)-1,3-dimethyl-5-((trifluoromethyl)imino)-5,10-dihydro-5λ⁴-dibenzo[b,e][1,4]thiabismine 5-oxide (90). Following the general procedure, using 500 mg of the corresponding sulfone ligand, **90** was obtained in 53% yield as white solid (Eluent: Hexanes/Ethyl acetate = 4/1).

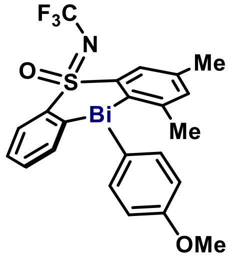

90

^{1}H NMR - (300 MHz, $CDCl_3$): δ 8.44 (dd, *J* = 7.8, 1.1 Hz, 1H), 8.17 (s, 1H), 7.80 (dd, *J* = 7.3, 1.0 Hz, 1H), 7.63 (d, *J* = 8.4 Hz, 2H), 7.44 (td, *J* = 7.6, 1.3 Hz, 1H), 7.34 (td, *J* = 7.3, 1.3 Hz, 1H), 7.20 (s, 1H), 6.87 (d, *J* = 8.5 Hz, 2H), 3.78 (s, 3H), 2.39 (s, 3H), 2.00 (s, 3H).

^{13}C NMR - (75 MHz, $CDCl_3$): δ 159.7, 157.2, 155.7, 154.4, 145.2, 140.3, 139.8, 139.4, 138.9, 138.4, 135.8, 133.8, 128.6, 127.2, 126.4, 121.6 (q, *J* = 269.1 Hz), 116.9, 55.1, 26.4, 21.1.

^{19}F NMR - (282 MHz, $CDCl_3$): δ –41.9 (s, 3F).

HRMS (ESI, m/z): calc'd for $C_{22}H_{20}BiF_3SNO_2^+$ $[M+H]^+$ 628.0965; found 628.0965.

m.p. (°C): 151.7 – 153.0.

(S)-1,3-dimethyl-10-(m-tolyl)-5-((trifluoromethyl)imino)-5,10-dihydro-5λ⁴-dibenzo[b,e][1,4]thiabismine 5-oxide (91). Following the general procedure, using 250 mg of the corresponding sulfone ligand, **91** was obtained in 63% yield as white solid (Eluent: Hexanes/Ethyl acetate = 4/1).

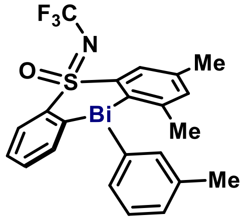

91

^{1}H NMR - (300 MHz, $CDCl_3$): δ 8.62 – 8.50 (m, 1H), 8.30 (s, 1H), 7.90 (dd, *J* = 7.3, 1.0 Hz, 1H), 7.73 (s, 1H), 7.63 – 7.53 (m, 2H), 7.45 (td, *J* = 7.4, 1.3 Hz, 1H), 7.39 – 7.31 (m, 2H), 7.25 (d, *J* = 7.5 Hz, 1H), 2.51 (s, 3H), 2.38 (s, 3H), 2.12 (s, 3H).

^{13}C NMR - (75 MHz, $CDCl_3$): δ 164.6, 157.3, 154.8, 145.2, 140.3, 139.8, 139.4, 139.0, 138.4, 135.9, 135.8, 133.8, 131.0, 129.2, 128.6, 127.4, 126.5, 125.6, 121.8 (q, *J* = 269.1 Hz), 26.5, 21.7, 21.1.

^{19}F NMR - (282 MHz, $CDCl_3$): δ –41.9 (s, 3F).

HRMS (ESI, m/z): calc'd for $C_{22}H_{20}BiF_3SNO^+$ $[M+H]^+$ 612.1016; found 612.1014.

m.p. (°C): 172.4 – 174.0.

(S)-1,3-dimethyl-5-((trifluoromethyl)imino)-10-(3-(trifluoromethyl)phenyl)-5,10-dihydro-5l4-dibenzo[b,e][1,4]thiabismine 5-oxide (92). Following the general procedure, using 500 mg of the corresponding sulfone ligand, **92** was obtained in 66% yield as white solid (Eluent: Hexanes/Ethyl acetate = 4/1).

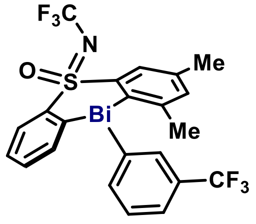

92

[1]**H NMR** - (300 MHz, $CDCl_3$): δ 8.47 (dd, *J* = 7.8, 1.1 Hz, 1H), 8.20 (s, 1H), 8.09 (s, 1H), 7.86 (d, *J* = 7.3 Hz, 1H), 7.74 (dd, *J* = 7.3, 1.0 Hz, 1H), 7.59 (d, *J* = 7.8 Hz, 1H), 7.52 – 7.33 (m, 3H), 7.25 (s, 1H), 2.41 (s, 3H), 1.97 (s, 3H).

^{13}**C NMR** - (75 MHz, $CDCl_3$): δ 165.3, 157.5, 155.0, 144.8, 142.5, 139.8, 139.4, 139.3, 138.2, 136.1, 134.9, 134.1, 132.5 (q, *J* = 32.2 Hz), 131.2, 129.0, 127.7, 126.8, 125.2 (q, *J* = 3.8 Hz), 124.3 (q, *J* = 284.1 Hz), 121.6 (q, *J* = 265.1 Hz), 26.5, 21.1.

^{19}**F NMR** - (282 MHz, $CDCl_3$): δ –41.9 (s, 3F), –62.5 (s, 3F).

HRMS (ESI, m/z): calc'd for $C_{22}H_{16}BiF_6SNONa^+$ $[M+Na]^+$ 688.0553; found 688.0550.

m.p. (°C): 179.9 – 181.6.

5-((4-(tert-butyl)phenyl)imino)-10-phenyl-5,10-dihydro-5λ^4-dibenzo[b,e][1,4]thiabismine 5-oxide (93). Following the general procedure, using 379 mg of the corresponding sulfone ligand, **93** was obtained in 57% yield as white solid (Eluent: Hexanes/Ethyl acetate = 4/1).

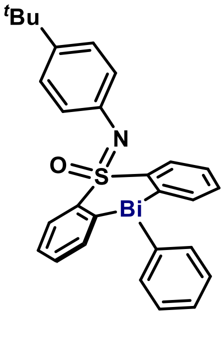

93

^{1}**H NMR** - (300 MHz, $CDCl_3$): δ 8.49 (dd, *J* = 7.8, 1.0 Hz, 2H), 7.83 (dd, *J* = 7.3, 0.9 Hz, 2H), 7.77 (dd, *J* = 7.9, 1.4 Hz, 2H), 7.45 – 7.29 (m, 7H), 7.14 – 7.07 (m, 2H), 7.03 – 6.94 (m, 2H), 1.21 (s, 9H).

^{13}**C NMR** - (75 MHz, $CDCl_3$): δ 168.7, 159.1, 144.6, 141.1, 140.7, 138.9, 137.9, 133.2, 130.9, 129.5, 128.4, 128.2, 125.9, 122.4, 34.2, 31.5.

HRMS (ESI, m/z): calc'd for $C_{28}H_{27}BiSNO^+$ $[M+H]^+$ 634.1617; found 634.1613.

m.p. (°C): 173.0 – 174.5.

10-phenyl-5-((4-(trifluoromethyl)phenyl)imino)-5,10-dihydro-5λ⁴-dibenzo[b,e][1,4]thiabismine 5-oxide (94). Following the general procedure, using 577 mg of the corresponding sulfone ligand, **94** was obtained in 57% yield as white solid (Eluent: Hexanes/Ethyl acetate = 4/1). Crystals suitable for XRD analysis were obtained by slow evaporation of a concentrated solution of **83** in a mixture of CH_2Cl_2/*iso*-hexane (5:1).

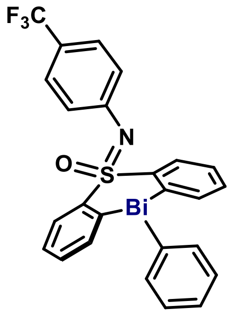

94

^{1}H NMR - (300 MHz, $CDCl_3$): δ 8.51 (d, *J* = 7.6 Hz, 2H), 7.85 (d, *J* = 7.1 Hz, 2H), 7.77 (d, *J* = 6.8 Hz, 2H), 7.51 – 7.30 (m, 9H), 7.14 (d, *J* = 8.3 Hz, 2H).

^{13}C NMR - (75 MHz, $CDCl_3$): δ 168.3, 158.6, 147.7, 139.9, 138.8, 138.0, 133.6, 131.0, 129.4, 128.6, 128.3, 126.2 (q, *J* = 3.8 Hz), 124.6 (q, *J* = 272.8 Hz), 123.8 (q, *J* = 32.6 Hz), 122.8.

^{19}F NMR - (282 MHz, $CDCl_3$): δ –61.7 (s, 3F).

HRMS (ESI, m/z): calc'd for $C_{25}H_{18}BiF_3SNO^+$ $[M+H]^+$ 646.0860; found 646.0865.

m.p. (°C): 176.7 – 178.2

5-((3,5-bis(trifluoromethyl)phenyl)imino)-10-phenyl-5,10-dihydro-5λ⁴-dibenzo[b,e][1,4]thiabismine 5-oxide (95). Following the general procedure, using 423 mg of the corresponding sulfone ligand, **95** was obtained in 32% yield as white solid (Eluent: Hexanes/Ethyl acetate = 4/1). Crystals suitable for XRD analysis were obtained by slow evaporation of a concentrated solution of **83** in a mixture of CH_2Cl_2/*iso*-hexane (5:1).

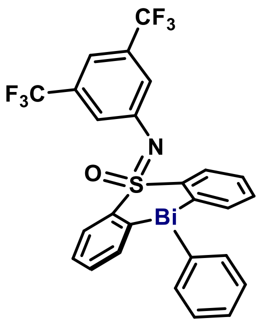

95

^{1}H NMR - (300 MHz, $CDCl_3$): δ 8.53 (dd, *J* = 7.7, 1.3 Hz, 2H), 7.86 (dd, *J* = 7.3, 1.2 Hz, 2H), 7.82 – 7.68 (m, 2H), 7.55 – 7.34 (m, 9H), 7.32 (s, 1H).

^{13}C NMR - (75 MHz, $CDCl_3$): δ 167.6, 158.2, 146.1, 139.3, 138.8, 138.2, 133.9, 132.1 (q, *J* = 32.6 Hz), 131.1, 129.4, 128.8, 128.5, 123.4 (q, *J* = 272.6 Hz), 122.6 (q, *J* = 3.6 Hz), 115.2 (quint, *J* = 32.6 Hz).

^{19}F NMR - (282 MHz, $CDCl_3$): δ –63.1 (s, 6F).

HRMS (ESI, m/z): calc'd for $C_{26}H_{17}BiF_6SNO^+$ $[M+H]^+$ 714.0739; found 714.0735.

m.p. (°C): 174.1 – 175.5.

10-(3,5-di-tert-butylphenyl)-1,3,7,9-tetramethyl-5-((trifluoromethyl)imino)-5,10-dihydro-5λ⁴-dibenzo[b,e][1,4]thiabismine 5-oxide (96): Following the general procedure, using 0.62 g of the corresponding sulfone ligand, **96** was obtained in 62% overall yield (Eluent: Hexanes/Ethyl acetate = 9/1, low pressure). **96a** was isolated as white solid in 40% yield and **96b** was isolated as white solid in 22% yield. Crystals suitable for XRD analysis were obtained by slow evaporation of a concentrated solution of **96a** and **96b** in a mixture of CH_2Cl_2/*iso*-hexane (5:1), respectively.

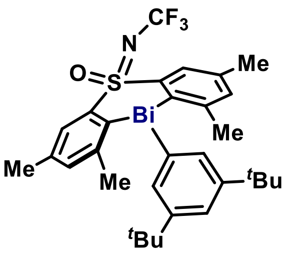

96a

^{1}H NMR - (300 MHz, $CDCl_3$): δ 8.19 (s, 2H), 7.58 – 7.42 (m, 2H), 7.22 (t, J = 1.8 Hz, 1H), 7.12 (s, 2H), 2.38 (s, 6H), 1.89 (s, 6H), 1.11 (s, 18H).

^{13}C NMR - (75 MHz, $CDCl_3$): δ 160.6, 154.3, 152.6, 145.3, 139.0, 138.7, 135.8, 133.0, 126.0, 121.7 (q, *J* = 272.2 Hz), 121.0, 35.1, 31.4, 25.8, 21.1.

^{19}F NMR - (282 MHz, $CDCl_3$): δ –41.8 (s, 3F).

HRMS (ESI, m/z): calc'd for $C_{31}H_{37}BiF_3SNONa^+$ $[M+Na]^+$ 760.2242; found 760.2242.

m.p. (°C): 267.7 – 269.1.

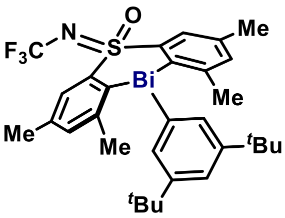

96b

^{1}H NMR - (300 MHz, $CDCl_3$): δ 8.23 (s, 2H), 7.44 (s, 2H), 7.21 (t, *J* = 1.8 Hz, 1H), 7.07 (s, 2H), 2.37 (s, 6H), 1.87 (s, 6H), 1.09 (s, 18H).

^{13}C NMR - (75 MHz, $CDCl_3$): δ 161.2, 154.0, 152.6, 145.3, 141.4, 138.5, 135.5, 132.8, 125.4, 123.2 (q, *J* = 257.1 Hz), 121.0, 35.1, 31.4, 25.8, 21.1.

^{19}F NMR - (282 MHz, $CDCl_3$): δ –38.1 (s, 3F).

HRMS (ESI, m/z): calc'd for $C_{31}H_{37}BiF_3SNONa^+$ $[M+Na]^+$ 760.2242; found 760.2240.

m.p. (°C): 207.0 – 208.4.

2.2 Synthesis of arylbismines via transmetalation with arylboronic acids

2.2.1 Synthesis of bismine tosylates

Bismine tosylate **99** was synthesized following a previously reported method.[14]

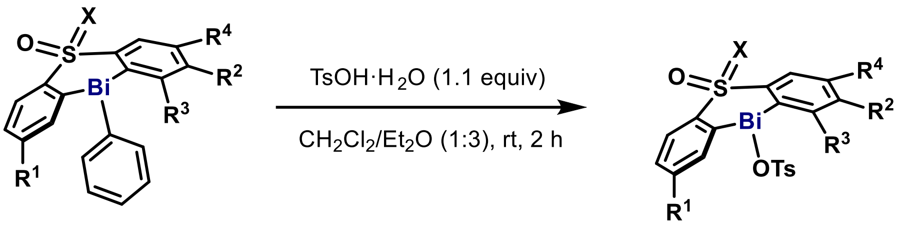

1; $R^1 = R^2 = R^3 = R^4 = H$; $X = NCF_3$
23; $R^1 = R^2 = H$; $R^3 = R^4 = Me$ $X = NCF_3$
39; $R^1 = R^2 = R^3 = R^4 = H$; $X = O$
40; $R^1 = Me$; $R^2 = CF_3$; $R^3 = R^4 = H$; $X = O$

97; $R^1 = R^2 = R^3 = R^4 = H$; $X = NCF_3$
98; $R^1 = R^2 = H$; $R^3 = R^4 = Me$ $X = NCF_3$
99; $R^1 = R^2 = R^3 = R^4 = H$; $X = O$
47; $R^1 = Me$; $R^2 = CF_3$; $R^3 = R^4 = H$; $X = O$

General procedure: Phenylbismine (1.0 equiv) was mixed with Et_2O and *p*-toluenesulfonic acid monohydride (1.1 equiv) and the reaction was stirred for 2 h at room temperature. Then, the solvent was removed under vacuum, and the remaining solid was washed with a CH_2Cl_2/pentane (1:5) solution. The white solid was dried under vacuum for 1 h.

2-methyl-5,5-dioxido-8-(trifluoromethyl)-10H-dibenzo[b,e][1,4]thiabismin-10-yl 4-methylbenzenesulfonate (47). Following the general procedure, using 1.5 g of bismine **40**, compound **47** was obtained in 98% yield as white solid. Crystals suitable for XRD analysis were obtained by slow evaporation of a concentrated solution of **47** in $CHCl_3$.

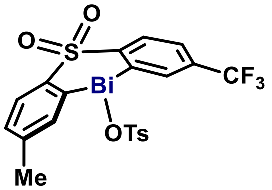

47

^{1}H NMR - (300 MHz, $CDCl_3$): δ 9.06 (s, 1H), 8.79 (s, 1H), 8.43 (d, *J* = 8.0 Hz, 1H), 8.31 (d, *J* = 7.8 Hz, 1H), 7.82 (d, *J* = 8.2 Hz, 2H), 7.70 (d, *J* = 8.0 Hz, 1H), 7.27 (d, *J* = 8.0 Hz, 3H), 2.40 (s, 3H), 2.38 (s, 3H).

^{13}C NMR - (75 MHz, $CDCl_3$): δ 191.4, 191.2, 148.2, 144.1, 142.8, 138.4, 137.4 (q, *J* = 32.6 Hz), 136.5, 135.8, 132.7 (q, *J* = 3.6 Hz), 130.1, 129.9, 129.6, 129.2, 126.5, 126.2 (q, *J* = 3.6 Hz), 123.8 (q, *J* = 273.6 Hz), 22.1, 21.6.

^{19}F NMR - (282 MHz, $CDCl_3$): δ –62.8 (s, 3F).

HRMS (ESI, m/z): calc'd for $C_{21}H_{16}BiF_3S_2O_5Na^+$ $[M+Na]^+$ 701.0087; found 701.0076.

m.p. (°C): 227.9 – 229.3.

5-oxido-5-((trifluoromethyl)imino)-5l4-dibenzo[b,e][1,4]thiabismin-10(5H)-yl 4-methylbenzenesulfonate (97). Following the general procedure, using 0.55 g of bismine **1**, compound **97** was obtained in 96% yield as white solid. Crystals suitable for XRD analysis were obtained by slow evaporation of a concentrated solution of **97** in $CHCl_3$.

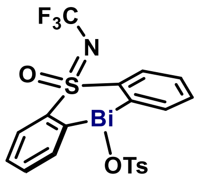

97

^{1}H NMR - (300 MHz, $CDCl_3$): δ 9.06 – 8.92 (m, 2H), 8.37 (*d*, J = 7.7 Hz, 2H), 7.91 – 7.74 (m, 4H), 7.56 (*td*, J = 7.7, 1.1 Hz, 2H), 7.28 (*d*, J = 8.0 Hz, 2H), 2.41 (s, 3H).

^{13}C NMR - (75 MHz, $CDCl_3$): δ 183.4, 142.4, 139.3, 138.9, 136.5, 136.0, 129.8, 129.5, 129.3, 126.5, 121.4 (q, *J* = 272.1 Hz), 21.61.

^{19}F NMR - (282 MHz, $CDCl_3$): δ –41.9 (s, 3F).

HRMS (ESI, m/z): calc'd for $C_{20}H_{15}BiNF_3S_2O_4Na^+$ $[M+Na]^+$ 686.0091; found 686.0088.

m.p. (°C): 219.0 – 220.1.

(S)-1,3-dimethyl-5-oxido-5-((trifluoromethyl)imino)-5l4-dibenzo[b,e][1,4]thiabismin-10(5H)-yl 4-methylbenzenesulfonate (98). Following the general procedure, using 0.55 g of bismine **23**, compound **98** was obtained in 34% yield as white solid.

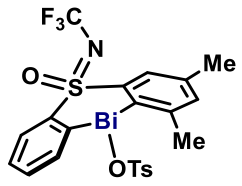

98

^{1}H NMR - (300 MHz, $CDCl_3$): δ 9.12 (d, J = 7.4 Hz, 1H), 8.39 (d, J = 7.7 Hz, 1H), 8.04 (s, 1H), 7.84 (t, J = 8.3 Hz, 3H), 7.58 (t, J = 7.6 Hz, 1H), 7.47 (s, 1H), 7.25 (d, J = 2.9 Hz, 2H), 2.43 (s, 3H), 2.41 (s, 3H), 2.35 (s, 3H).

^{13}C NMR - (75 MHz, $CDCl_3$): δ 182.9, 178.3, 145.4, 142.2, 140.1, 139.3, 138.8, 138.6, 138.5, 136.6, 136.3, 129.4, 129.3, 128.3, 126.5, 121.4 (q, *J* = 272.1 Hz), 24.4, 21.6, 21.4.

One carbon signal is not observed.

^{19}F NMR - (282 MHz, $CDCl_3$): δ –41.9 (s, 3F).

HRMS (ESI, m/z): calc'd for $C_{22}H_{19}BiNF_3S_2O_4Na^+$ $[M+Na]^+$ 714.0405; found 714.0404.

m.p. (°C): 189.1 – 190.4.

2.2.2 Synthesis of bismine trifluoroacetates

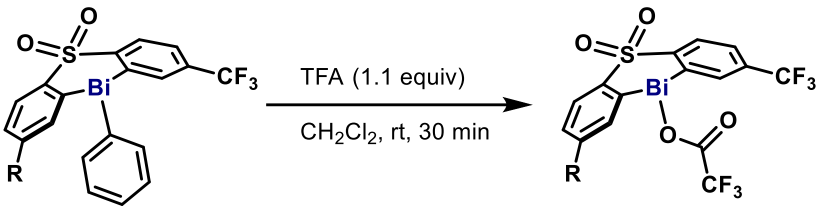

40; R = Me
41; R = CF_3

100; R = Me
68; R = CF_3

General procedure.[14] Phenylbismine (1.0 equiv) was mixed with anhydrous CH_2Cl_2 and trifluoroacetic acid (1.1 equiv) and the reaction was stirred for 30 min at room temperature. Then, solvent was removed under vacuum, and the remaining solid was dried under vacuum for 2 h

.

5,5-dioxido-2,8-bis(trifluoromethyl)-10H-dibenzo[b,e][1,4]thiabismin-10-yl 2,2,2-trifluoroacetate (68). Following the general procedure, using 260 mg of phenylbismine **41**, compound **68** was obtained in 99% yield as white solid.

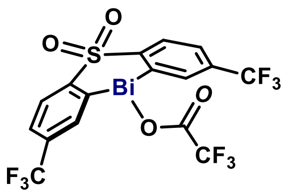

68

^{1}H NMR - (300 MHz, $CDCl_3$): δ 9.00 (s, 2H), 8.54 (d, J = 8.1 Hz, 2H), 7.81 (d, J = 8.1 Hz, 2H).

^{13}C NMR - (75 MHz, $CDCl_3$): δ 188.0, 164.2 (q, J = 40.0 Hz), 143.1, 138.1 (q, J = 33.0 Hz), 133.0 (q, J = 3.7 Hz), 130.0, 126.9 (q, J = 3.6 Hz), 123.5 (q, J = 274.1 Hz), 116.5 (q, J = 287.7 Hz).

^{19}F NMR - (282 MHz, $CDCl_3$): δ –63.1 (s, 6F), –73.9 (s, 3F).

HRMS (ESI, m/z): calc'd for $C_{16}H_6BiF_9SO_4Na^+$ $[M+Na]^+$ 696.9537; found 696.9539.

m.p. (°C): 230.5 (decomposition)

2-methyl-5,5-dioxido-8-(trifluoromethyl)-10H-dibenzo[b,e][1,4]thiabismin-10-yl 2,2,2-trifluoroacetate (100). Following the general procedure, using 100 mg of phenylbismine **40**, compound **100** was obtained in 99% yield as white solid.

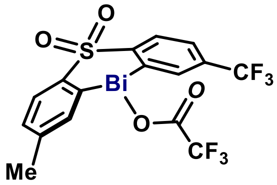

100

^{1}H NMR - (300 MHz, $CDCl_3$): δ 8.95 (s, 1H), 8.56 (s, 1H), 8.47 (d, *J* = 8.0 Hz, 1H), 8.34 (d, *J* = 7.9, 1H), 7.75 (d, *J* = 8.0, 1H), 7.32 (d, *J* = 7.9, 1H), 2.45 (s, 3H).

^{13}C NMR - (101 MHz, $CDCl_3$): δ 186.8, 186.7, 163.9 (q, *J* = 40.2 Hz), 148.2, 144.3, 137.4 (q, *J* = 33.1 Hz), 136.3, 136.3, 132.7 (q, *J* = 3.8 Hz), 130.1, 130.1, 129.1 126.5 (q, *J* = 3.7 Hz), 123.6 (q, *J* = 273.7 Hz), 116.3 (q, *J* = 288.1 Hz), 22.2.

^{19}F NMR - (282 MHz, $CDCl_3$): δ –62.9 (s, 3F), –74.0 (s, 3F).

HRMS (ESI+, m/z): calc'd for $C_{16}H_9BiF_6SO_4Na^+$ $[M+Na]^+$ 642.9822; found 642.9822.

m.p. (°C): 207.8 (decomposition)

2.2.3 Synthesis of aryl bismine species

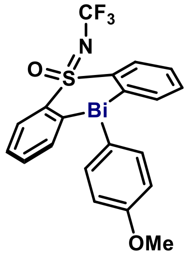
101

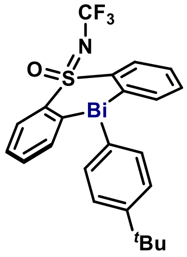
102

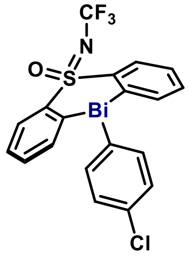
103

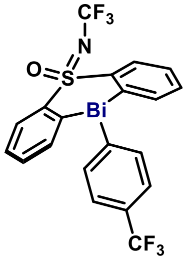
104

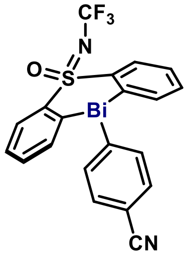
105

Arylbismines **101-105** were synthesized according to previously reported protocol.[2]

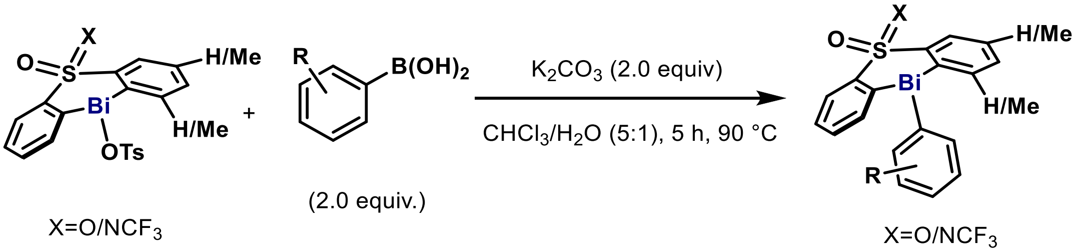

General procedure.[14] Bismine tosylate (1.0 equiv) was weighted in a pressure Schlenk together with the corresponding arylboronic acid (2.0 equiv) and K_2CO_3 (2.0 equiv). A mixture of $CHCl_3$ and water (5:1) was added, the Schlenk was sealed and the reaction was stirred at 90 °C for 5 h. Then, the mixture was cooled to room temperature, diluted with CH_2Cl_2 and washed with a saturated aqueous solution of $NaHCO_3$. The solvent was removed under reduced pressure and the crude was purified by flash chromatography (silica gel, Hexanes/Ethyl acetate) to give the corresponding phenylbismine as a white solid.

10-(p-tolyl)-5-((trifluoromethyl)imino)-5,10-dihydro-5l4-dibenzo[b,e][1,4]thiabismine 5-oxide (106). Following the general procedure, using 290 mg of bismine tosylate **97**, compound **106** was obtained in 98% yield as white solid (Ethyl acetate/Hexanes 1/4).

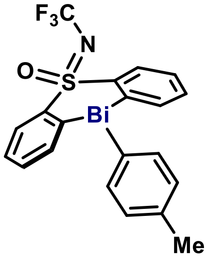

106

^{1}H NMR - (300 MHz, $CDCl_3$): δ 8.43 (dd, *J* = 7.6, 1.4 Hz, 2H), 7.89 (dd, *J* = 7.2, 1.4 Hz, 2H), 7.64 (d, *J* = 7.8 Hz, 2H), 7.45 (td, *J* = 7.6, 1.4 Hz, 2H), 7.38 (td, *J* = 7.3, 1.5 Hz, 2H), 7.29 – 7.18 (m, 2H), 2.34 (s, 3H).

^{13}C NMR - (75 MHz, $CDCl_3$): δ 164.0, 158.3, 140.0, 138.9, 138.8, 138.0, 133.9, 131.9, 128.6, 127.9, 121.8 (q, *J* = 272.3 Hz), 21.8.

^{19}F NMR - (282 MHz, $CDCl_3$): δ –41.9 (s, 3F).

HRMS (EI, m/z): calc'd for $C_{20}H_{15}BiF_3SNO^+$ $[M]^+$ 583.0625; found 583.0637.

m.p. (°C): 162.1 – 163.3.

10-(3,5-di-tert-butylphenyl)-5-((trifluoromethyl)imino)-5,10-dihydro-5λ^4-dibenzo[b,e][1,4]thiabismine 5-oxide (107). Following the general procedure, using 230 mg of bismine tosylate **97**, compound **107** was obtained in 93% yield as white solid (Ethyl acetate/Hexanes 1/4).

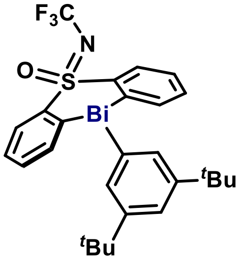

107

^{1}H NMR - (300 MHz, $CDCl_3$): δ 8.45 (d, *J* = 7.7 Hz, 2H), 7.95 (d, J = 7.1 Hz, 2H), 7.60 (d, *J* = 1.7 Hz, 2H), 7.47 (t, *J* = 7.5 Hz, 2H), 7.43 – 7.33 (m, 3H), 1.23 (s, 18H).

^{13}C NMR - (75 MHz, $CDCl_3$): δ 167.0, 158.8, 152.9, 140.0, 138.2, 133.5, 133.1, 128.5, 127.9, 122.5, 121.8 (q, J = 272.3 Hz), 35.4, 31.5.

^{19}F NMR - (282 MHz, $CDCl_3$): δ –41.8 (s, 3F).

HRMS (ESI, m/z): calc'd for $C_{27}H_{30}BiF_3SNO^+$ $[M+H]^+$ 682.1799; found 682.1793.

m.p. (°C): 227.7 – 228.9.

10-(2,6-dimethylphenyl)-5-((trifluoromethyl)imino)-5,10-dihydro-5λ⁴-dibenzo[b,e][1,4]thiabismine 5-oxide (108). Following the general procedure, using 230 mg of bismine tosylate **97**, compound **108** was obtained in 83% yield as white solid (Ethyl acetate/Hexanes 1/4). Crystals suitable for XRD analysis were obtained by slow evaporation of a concentrated solution of **108** in $CHCl_3$.

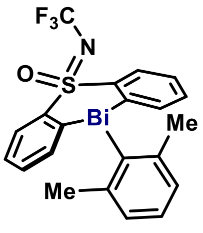

108

^{1}H NMR - (300 MHz, $CDCl_3$): δ 8.58 – 8.44 (m, 2H), 8.08 (d, *J* = 7.2 Hz, 2H), 7.50 (td, *J* = 7.7, 1.1 Hz, 2H), 7.40 (td, *J* = 7.3, 1.1 Hz, 2H), 7.23 (s, 3H), 2.89 (bs, 3H), 1.06 (bs, 3H).

^{13}C NMR - (75 MHz, $CDCl_3$): δ 170.5, 155.2, 140.4, 139.1, 138.0, 136.2, 133.7, 129.5, 128.9, 128.2, 121.8 (q, J = 272.3 Hz), 28.4.

^{19}F NMR - (282 MHz, $CDCl_3$): δ –41.9 (s, 3F).

HRMS (ESI, m/z): calc'd for $C_{21}H_{17}BiNF_3SONa^+$ $[M+Na]^+$ 620.0679; found 620.0681.

m.p. (°C): 185.1 – 186.6.

10-(3-methoxyphenyl)-5-((trifluoromethyl)imino)-5,10-dihydro-5λ⁴-dibenzo[b,e][1,4]thiabismine 5-oxide (109). Following the general procedure, using 310 mg of bismine tosylate **97**, compound **109** was obtained in 86% yield as white solid (Ethyl acetate/Hexanes 1/4). Characterization matches previously reported data.

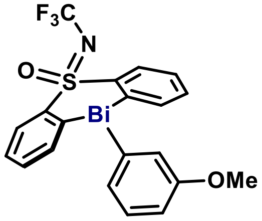

109

^{1}H NMR - (300 MHz, $CDCl_3$): δ 8.45 (dd, *J* = 7.4, 1.6 Hz, 2H), 7.93 (dd, *J* = 6.8, 1.5 Hz, 2H), 7.59 – 7.29 (m, 7H), 6.89 (ddd, *J* = 8.1, 2.6, 1.1 Hz, 1H), 3.71 (s, 3H).

^{13}C NMR - (75 MHz, $CDCl_3$): δ 168.1, 162.2, 158.5, 139.9, 138.0, 134.0, 132.1, 130.8, 128.7, 128.0, 124.5, 121.8 (q, *J* = 272.4 Hz), 114.1, 55.3.

^{19}F NMR - (282 MHz, $CDCl_3$): δ –41.9 (s, 3F).

HRMS (ESI, m/z): calc'd for $C_{20}H_{15}BiF_3SNO_2Na^+$ $[M+Na]^+$ 622.0472; found 622.0469.

10-(2,6-dimethylphenyl)-5-((trifluoromethyl)imino)-5,10-dihydro-5λ^4-dibenzo[b,e][1,4]thiabismine 5-oxide (110). Following the general procedure, using 230 mg of bismine tosylate **99**, compound **110** was obtained in 92% yield as white solid (Ethyl acetate/Hexanes 1/4). Crystals suitable for XRD analysis were obtained by slow evaporation of a concentrated solution of **110** in $CHCl_3$.

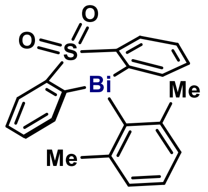

110

^{1}H NMR - (300 MHz, $CDCl_3$): δ 8.43 (dd, *J* = 7.7, 1.1 Hz, 2H), 8.04 (dd, *J* = 7.2, 1.0 Hz, 2H), 7.42 (td, *J* = 7.6, 1.3 Hz, 2H), 7.33 (td, *J* = 7.3, 1.3 Hz, 2H), 7.21 (s, 3H), 1.97 (s, 6H).

^{13}C NMR - (75 MHz, $CDCl_3$): δ 155.4, 146.5, 141.2, 137.9, 133.2, 129.4, 129.0, 128.9, 128.6, 127.4, 28.3.

HRMS (ESI, m/z): calc'd for $C_{20}H_{17}BiSO_2Na^+$ $[M+Na]^+$ 553.0645; found 553.0646.

m.p. (°C): 278.0 (decomposition).

10-(3-chlorophenyl)-5-((trifluoromethyl)imino)-5,10-dihydro-5λ^4-dibenzo[b,e][1,4]thiabismine 5-oxide (111). Following the general procedure, using 232 mg of bismine tosylate **97**, compound **111** was obtained in 90% yield as white solid (Ethyl acetate/Hexanes 1/4).

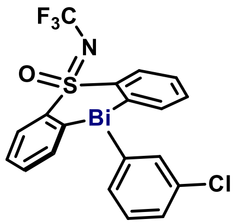

111

^{1}H NMR - (300 MHz, $CDCl_3$): δ 8.46 (dd, *J* = 7.6, 1.2 Hz, 2H), 7.90 (dd, *J* = 7.0, 1.1 Hz, 2H), 7.76 (s, 1H), 7.62 (d, J = 6.7 Hz, 1H), 7.53 – 7.31 (m, 6H).

^{13}C NMR - (75 MHz, $CDCl_3$): δ 168.6, 158.7, 139.9, 138.4, 137.9, 137.8, 136.7, 134.2, 132.4, 129.1, 128.9, 128.2, 121.7 (q, *J* = 272.4 Hz).

^{19}F NMR - (282 MHz, $CDCl_3$): δ –41.9 (s, 3F).

HRMS (ESI, m/z): calc'd for $C_{19}H_{12}BiF_3SNOClNa^+$ $[M+Na]^+$ 625.9973; found 625.9973.

m.p. (°C): 87.1 – 88.6.

10-(m-tolyl)-5-((trifluoromethyl)imino)-5,10-dihydro-5λ^4-dibenzo[b,e][1,4]thiabismine 5-oxide (112). Following the general procedure, using 232 mg of bismine tosylate **97**, compound **112** was obtained in 79% yield as white solid (Ethyl acetate/Hexanes 1/4).

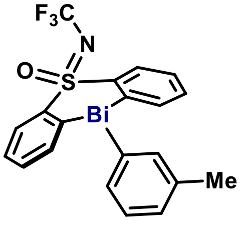

112

^{1}H NMR - (300 MHz, $CDCl_3$): δ 8.46 (d, *J* = 7.7 Hz, 2H), 7.92 (d, *J* = 7.2 Hz, 2H), 7.63 (s, 1H), 7.56 – 7.30 (m, 6H), 7.24 – 7.15 (m, 1H), 2.30 (s, 3H)

^{13}C NMR - (75 MHz, $CDCl_3$): δ 167.1, 158.3, 140.5, 139.9, 139.1, 138.0, 135.9, 133.9, 131.1, 129.6, 128.6, 128.0, 121.9 (q, *J* = 272.4 Hz), 21.7.

^{19}F NMR - (282 MHz, $CDCl_3$): δ –41.9 (s, 3F).

HRMS (ESI, m/z): calc'd for $C_{20}H_{15}BiF_3SNONa^+$ $[M+Na]^+$ 606.0518; found 606.0523.

m.p. (°C): 157.9 – 158.6.

5-((trifluoromethyl)imino)-10-(3-(trifluoromethyl)phenyl)-5,10-dihydro-5λ^4-dibenzo[b,e][1,4]thiabismine 5-oxide (113). Following the general procedure, using 230 mg of bismine tosylate **97**, compound **113** was obtained in 71% yield as white solid (Ethyl acetate/Hexanes 1/4).

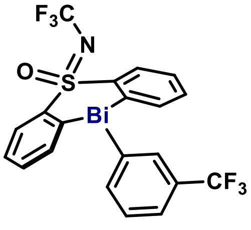

113

^{1}H NMR - (300 MHz, $CDCl_3$): δ 8.47 (dd, *J* = 7.6, 1.1 Hz, 2H), 8.10 (s, 1H), 7.97 – 7.84 (m, 3H), 7.63 (d, *J* = 7.8 Hz, 1H), 7.54 – 7.40 (m, 5H).

^{13}C NMR - (75 MHz, $CDCl_3$): δ 167.9, 158.7, 142.6, 140.0, 137.8, 135.1 (q, *J* = 3.8 Hz), 134.3, 132.7 (q, *J* = 31.8 Hz), 131.3, 128.9, 128.3, 125.6 (q, *J* = 3.9 Hz), 124.4 (q, *J* = 212.4 Hz), 121.9 (q, *J* = 272.4 Hz).

^{19}F NMR - (282 MHz, $CDCl_3$): δ –41.9 (s, 3F), –62.5 (s, 3F).

HRMS (ESI, m/z): calc'd for $C_{20}H_{12}BiF_6SNONa^+$ $[M+Na]^+$ 660.0236; found 660.0240.

m.p. (°C): 159.7 – 161.2.

10-(3-isopropylphenyl)-5-((trifluoromethyl)imino)-5,10-dihydro-5λ^4-

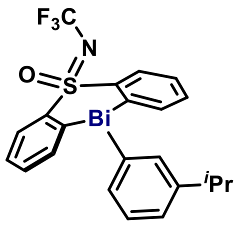

114

dibenzo[b,e][1,4]thiabismine 5-oxide (114). Following the general procedure, using 199 mg of bismine tosylate **97**, compound **114** was obtained in 97% yield as white solid (Ethyl acetate/Hexanes 1/4).

^{1}H NMR - (300 MHz, $CDCl_3$): δ 8.36 (d, *J* = 7.7 Hz, 2H), 7.83 (d, *J* = 7.1 Hz, 2H), 7.57 (s, 1H), 7.49 – 7.22 (m, 6H), 7.16 (d, *J* = 8.4 Hz, 2H), 2.76 (hept, *J* = 7.0 Hz, 1H), 1.12 (d, *J* = 6.9 Hz, 6H).

^{13}C NMR - (75 MHz, $CDCl_3$): δ 167.3, 158.5, 151.2, 140.0, 138.1, 136.9, 136.4, 133.8, 131.2, 128.6, 128.0, 126.8, 121.8 (q, *J* = 272.4 Hz), 34.4, 24.1.

^{19}F NMR - (282 MHz, $CDCl_3$): δ –41.9 (s, 3F).

HRMS (ESI, m/z): calc'd for $C_{22}H_{20}BiF_3SNO^+$ $[M+H]^+$ 612.1025; found 612.1016.

m.p. (°C): 130.7 – 132.1.

10-(3-(tert-butyl)phenyl)-5-((trifluoromethyl)imino)-5,10-dihydro-5λ^4-

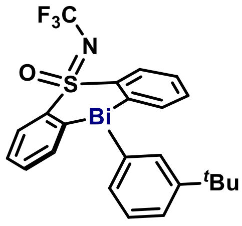

115

dibenzo[b,e][1,4]thiabismine 5-oxide (115). Following the general procedure, using 221 mg of bismine tosylate **97**, compound **115** was obtained in 80% yield as white solid (Ethyl acetate/Hexanes 1/4).

^{1}H NMR - (300 MHz, $CDCl_3$): δ 8.45 (d, *J* = 7.6 Hz, 2H), 7.92 (d, *J* = 7.0 Hz, 2H), 7.84 (s, 1H), 7.59 – 7.31 (m, 7H), 1.26 (s, 9H).

^{13}C NMR - (75 MHz, $CDCl_3$): δ 167.3, 158.6, 153.3, 139.9, 138.1, 136.0, 135.8, 133.8, 130.9, 128.6, 128.0, 125.6, 121.8 (q, *J* = 272.4 Hz), 35.2, 31.4.

^{19}F NMR - (282 MHz, $CDCl_3$): δ –41.9 (s, 3F).

HRMS (ESI, m/z): calc'd for $C_{23}H_{22}BiF_3SNO^+$ $[M+H]^+$ 626.1180; found 626.1173.

m.p. (°C): 178.1 – 179.7.

10-(o-tolyl)-5-((trifluoromethyl)imino)-5,10-dihydro-5λ^4-dibenzo[b,e][1,4]thiabismine 5-oxide (116). Following the general procedure, using 196 mg of bismine tosylate **97**, compound **116** was obtained in 70% yield as white solid (Ethyl acetate/Hexanes 1/4).

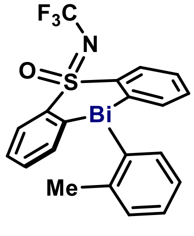

116

^{1}H NMR - (300 MHz, $CDCl_3$): δ 8.45 (dd, *J* = 7.8, 1.2 Hz, 2H), 7.91 (dd, *J* = 7.2, 1.2 Hz, 2H), 7.58 (d, *J* = 7.4 Hz, 1H), 7.48 (td, *J* = 7.6, 1.3 Hz, 3H), 7.39 (td, *J* = 7.3, 1.3 Hz, 2H), 7.31 (td, *J* = 7.5, 1.3 Hz, 1H), 7.07 (t, *J* = 7.3 Hz, 1H), 2.57 (s, 3H).

^{13}C NMR - (75 MHz, $CDCl_3$): δ 168.6, 157.5, 144.4, 141.2, 139.8, 138.0, 133.7, 130.4, 129.4, 128.9, 128.5, 127.8, 121.7 (q, *J* = 256.5 Hz), 26.3.

^{19}F NMR - (282 MHz, $CDCl_3$): δ –41.9 (s, 3F).

HRMS (ESI, m/z): calc'd for $C_{20}H_{15}BiF_3SNONa^+$ $[M+Na]^+$ 606.0518; found 606.0523.

m.p. (°C): 127.3 – 128.7.

10-(2-ethylphenyl)-5-((trifluoromethyl)imino)-5,10-dihydro-5λ^4-dibenzo[b,e][1,4]thiabismine 5-oxide (117). Following the general procedure, using 232 mg of bismine tosylate **97**, compound **117** was obtained in 72% yield as white solid (Ethyl acetate/Hexanes 1/4).

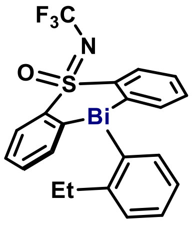

117

^{1}H NMR - (300 MHz, $CDCl_3$): δ 8.44 (dd, *J* = 7.8, 1.2 Hz, 2H), 7.86 (dd, *J* = 7.8, 1.2 Hz, 2H), 7.54 - 7.34 (m, 7H), 7.03 (td, J = 7.4, 1.3 Hz, 1H), 3.02 (q, J = 7.5 Hz, 2H), 1.44 (t, J = 7.6 Hz, 3H).

^{13}C NMR - (75 MHz, $CDCl_3$): δ 168.6, 157.9, 151.3, 143.4, 140.1, 138.3, 133.9, 130.0, 129.1, 129.0, 128.6, 127.9, 121.7 (q, *J* = 272.4 Hz), 33.2, 18.0.

^{19}F NMR - (282 MHz, $CDCl_3$): δ –41.9 (s, 3F).

HRMS (ESI, m/z): calc'd for $C_{21}H_{17}BiF_3SNONa^+$ $[M+Na]^+$ 620.0676; found 620.0679.

m.p. (°C): 169.1 – 170.4.

10-(2-isopropylphenyl)-5-((trifluoromethyl)imino)-5,10-dihydro-5λ⁴-dibenzo[b,e][1,4]thiabismine 5-oxide (118). Following the general procedure, using 232 mg of bismine tosylate **97**, compound **118** was obtained in 53% yield as white solid (Ethyl acetate/Hexanes 1/4).

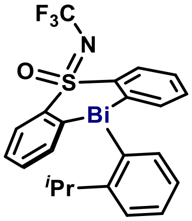

118

^{1}**H NMR** - (300 MHz, $CDCl_3$): δ 8.44 (dd, *J* = 7.8, 1.3 Hz, 2H), 7.89 (dd, *J* = 7.2, 1.2 Hz, 2H), 7.61 (d, *J* = 8.8 Hz, 1H), 7.57 – 7.35 (m, 6H), 7.00 (td, *J* = 7.4, 1.2 Hz, 1H), 3.47 (dq, *J* = 13.0, 6.3 Hz, 1H), 1.46 (d, *J* = 6.8 Hz, 6H).

^{13}**C NMR** - (75 MHz, $CDCl_3$): δ 169.0, 158.3, 155.8, 142.5, 140.1, 138.4, 133.9, 130.1, 129.2, 128.6, 127.9, 125.7, 121.7 (q, *J* = 272.4 Hz), 37.8, 25.0.

^{19}**F NMR** - (282 MHz, $CDCl_3$): δ –41.9 (s, 3F).

HRMS (ESI, m/z): calc'd for $C_{22}H_{19}BiF_3SNONa^+$ $[M+Na]^+$ 634.0830; found 634.0836.

m.p. (°C): 180.2 – 181.3.

5-((trifluoromethyl)imino)-10-(3-(trimethylgermyl)phenyl)-5,10-dihydro-5λ⁴-dibenzo[b,e][1,4]thiabismine 5-oxide (119). Following the general procedure, using 190 mg of bismine tosylate **97**, compound **119** was obtained in 67% yield as white solid (Ethyl acetate/Hexanes 1/4).

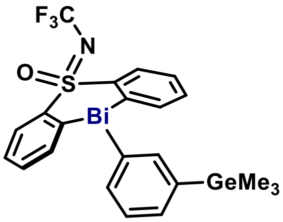

119

^{1}**H NMR** - (300 MHz, $CDCl_3$): δ 8.45 (dd, *J* = 7.8, 1.1 Hz, 2H), 7.92 (dd, *J* = 7.2, 1.1 Hz, 2H), 7.86 (s, 1H), 7.66 (d, *J* = 7.3 Hz, 1H), 7.56 – 7.43 (m, 3H), 7.39 (td, *J* = 7.4, 1.4 Hz, 3H), 0.34 (s, 9H).

^{13}**C NMR** - (75 MHz, $CDCl_3$): δ 166.9, 158.6, 145.1, 142.8, 140.0, 139.0, 138.1, 133.9, 133.3, 131.0, 128.6, 128.0, 121.8 (q, *J* = 272.4 Hz), –1.6.

^{19}**F NMR** - (282 MHz, $CDCl_3$): δ –41.9 (s, 3F).

HRMS (ESI, m/z): calc'd for $C_{22}H_{22}BiF_3SGeNO^+$ $[M+H]^+$ 688.0384; found 688.0388.

m.p. (°C): 175.3 – 176.3.

5-((trifluoromethyl)imino)-10-(3-(trimethylsilyl)phenyl)-5,10-dihydro-5λ⁴-

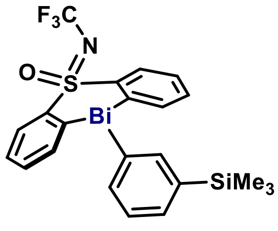

120

dibenzo[b,e][1,4]thiabismine 5-oxide (120). Following the general procedure, using 167 mg of bismine tosylate **97**, compound **120** was obtained in 71% yield as white solid (Ethyl acetate/Hexanes 1/4).

^{1}H NMR - (300 MHz, $CDCl_3$): δ 8.46 (dd, *J* = 7.7, 1.0 Hz, 2H), 7.92 (d, *J* = 5.7 Hz, 3H), 7.70 (d, *J* = 7.4 Hz, 1H), 7.56 (d, *J* = 7.3 Hz, 1H), 7.47 (td, *J* = 7.6, 1.2 Hz, 2H), 7.43 – 7.32 (m, 3H), 0.23 (s, 9H).

^{13}C NMR - (75 MHz, $CDCl_3$): δ 166.8, 158.6, 143.2, 142.8, 140.0, 139.6, 138.1, 133.8, 133.6, 130.8, 128.6, 128.0, 121.8 (q, *J* = 272.4 Hz), –1.0.

^{19}F NMR - (282 MHz, $CDCl_3$): δ –41.8 (s, 3F).

HRMS (ESI, m/z): calc'd for $C_{22}H_{21}BiF_3SSiNONa^+$ $[M+Na]^+$ 664.0761; found 664.0763.

m.p. (°C): 169.0 – 170.5.

(S)-10-(3,5-di-tert-butylphenyl)-1,3-dimethyl-5-((trifluoromethyl)imino)-5,10-dihydro-5l4-

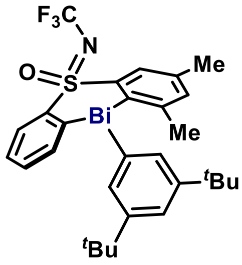

121

dibenzo[b,e][1,4]thiabismine 5-oxide (121). Following the general procedure, using 200 mg of bismine tosylate **98**, compound **121** was obtained in 35% yield as white solid (Ethyl acetate/Hexanes 1/4).

^{1}H NMR - (300 MHz, $CDCl_3$): δ 8.44 (dd, *J* = 7.8, 1.3 Hz, 1 H), 8.19 (s, 1H), 7.85 (dd, *J* = 7.8, 1.3 Hz, 1H), 7.59 (d, J = 1.7 Hz, 2H), 7.44 (td, J = 7.6, 1.2 Hz, 1H), 7.34 (d, J = 6.2 Hz, 2H), 7.19 (s, 1H), 2.39 (s, 3H), 1.96 (s, 3H), 1.20 (s, 18 H).

^{13}C NMR - (75 MHz, $CDCl_3$): δ 164.4, 157.8, 155.1, 152.8, 145.3, 139.8, 139.5, 138.7, 138.6, 135.5, 133.5, 133.1, 128.5, 127.3, 126.4, 121.8 (q, *J* = 272.4 Hz), 121.7, 35.3, 31.5, 26.4, 21.1.

^{19}F NMR - (282 MHz, $CDCl_3$): δ –41.9 (s, 3F).

HRMS (ESI, m/z): calc'd for $C_{29}H_{33}BiF_3SNONa^+$ $[M+Na]^+$ 732.1932; found 732.1932.

2.2.4 Synthesis of aryl bismine species from tosylate **47**

Aryl bismines **122-145** were synthesized following a previously reported method.[14]

<u>General procedure</u>. Bismine tosylate **47** (1.0 equiv) was weighted in a scintillation vial together with the corresponding arylboronic acid (1.0 equiv). A mixture of $CDCl_3$ and a saturated aqueous solution of Na_2CO_3 (9:1) was added, the vial was sealed and the reaction was stirred at 60 °C for 16 h. Then, the mixture was cooled to room temperature, the solvent was removed under reduced pressure and the crude was purified by flash chromatography (silica gel, Hexanes/Ethyl acetate) to give the corresponding phenylbismine as a white solid.

Table S1. Scope of the transmetalation reaction of arylboronic acids with bismine tosylate **47**. Yields of isolated products are reported.

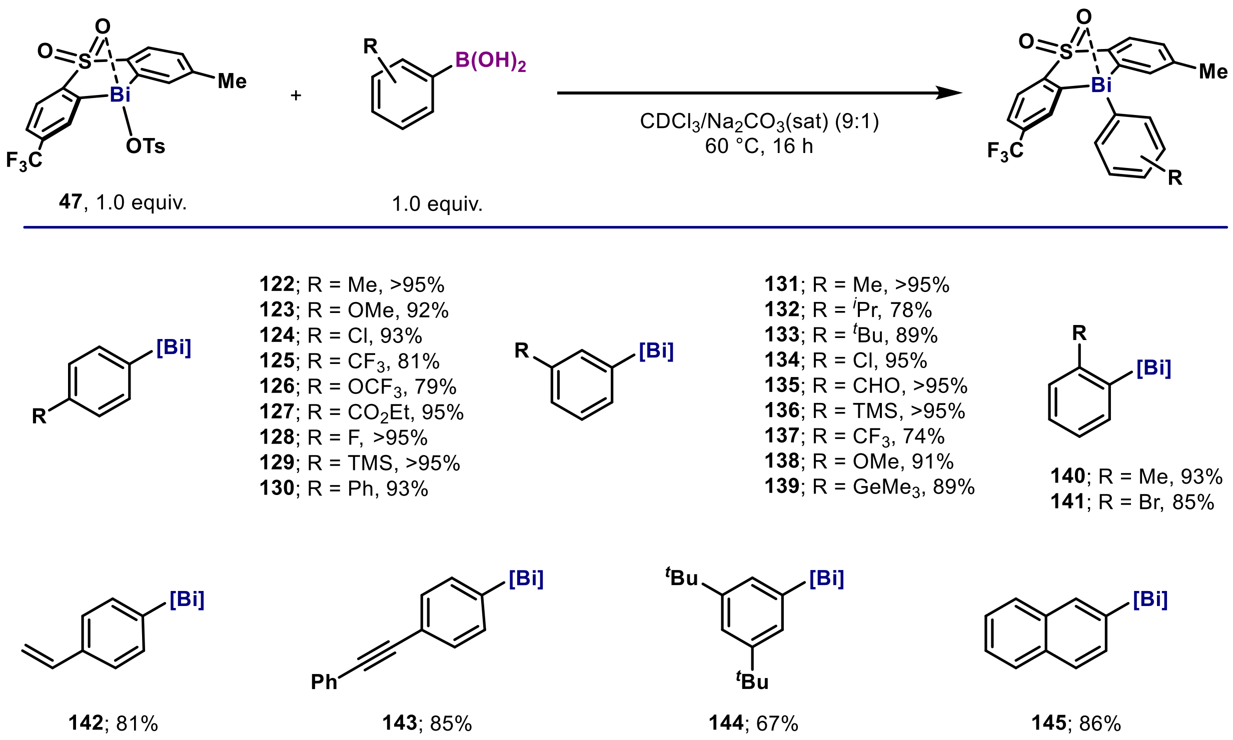

2-methyl-10-(p-tolyl)-8-(trifluoromethyl)-10H-dibenzo[b,e][1,4]thiabismine 5,5-dioxide (122). Following the general procedure, using 136 mg (0.2 mmol) of bismine tosylate **47**, compound **122** was obtained in 98% yield (117 mg, 0.196 mmol) as a white solid (Ethyl acetate/Hexanes 1/4).

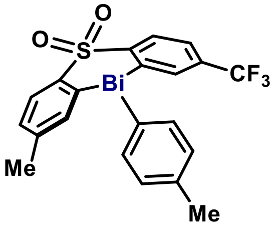

122

^{1}H NMR - (300 MHz, $CDCl_3$): δ 8.43 (d, J = 8.1 Hz, 1H), 8.28 (d, J = 7.9 Hz, 1H), 8.06 (s, 1H), 7.71 (s, 1H), 7.65 (d, J = 7.9 Hz, 3H), 7.28 (d, J = 7.9 Hz, 2H), 7.22 (d, J = 7.0 Hz, 1H), 2.36 (s, 3H), 2.28 (s, 3H).

^{13}C NMR - (75 MHz, $CDCl_3$): δ 162.9, 159.4, 158.9, 145.8 (q, *J* = 1.4 Hz), 144.7, 139.1, 138.7, 138.4, 138.1, 134.6 (q, *J* = 32.4 Hz), 134.5 (q, *J* = 3.8 Hz), 132.1, 129.2, 127.7, 126.8, 125.3 (q, *J* = 3.8 Hz), 123.6 (q, *J* = 272.8 Hz), 21.8, 21.6.

^{19}F NMR - (282 MHz, $CDCl_3$): δ –62.9 (s, 3F).

HRMS (ESI, m/z): calc'd for $C_{21}H_{16}BiF_3SO_2Na^+$ $[M+Na]^+$ 621.0519; found 621.0526.

m.p. (°C): 144.1 – 145.6.

10-(4-methoxyphenyl)-2-methyl-8-(trifluoromethyl)-10H-dibenzo[b,e][1,4]thiabismine 5,5-dioxide (123). Following the general procedure, using 136 mg (0.2 mmol) of bismine tosylate **47**, compound **123** was obtained in 92% yield (113 mg, 0.184 mmol) as a white solid (Ethyl acetate/Hexanes 1/4).

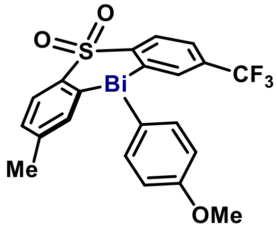

123

^{1}H NMR - (300 MHz, $CDCl_3$): δ 8.43 (d, *J* = 8.1 Hz, 1H), 8.27 (d, *J* = 7.9 Hz, 1H), 8.07 (s, 1H), 7.74 – 7.59 (m, 4H), 7.22 (d, *J* = 7.9 Hz, 1H), 6.97 (d, *J* = 8.6 Hz, 2H), 3.82 (s, 3H), 2.28 (s, 3H).

^{13}C NMR - (75 MHz, $CDCl_3$): δ 166.2, 159.4, 158.9, 157.4, 145.8, 144.7, 140.3, 138.4, 138.1, 134.6 (q, *J* = 32.7 Hz), 134.5 (q, *J* = 3.7 Hz), 129.2, 127.6, 126.8, 125.3 (q, *J* = 3.7 Hz), 123.4 (q, *J* = 272.7 Hz), 117.1, 55.2, 21.6.

^{19}F NMR - (282 MHz, $CDCl_3$): δ –62.8 (s, 3F).

HRMS (ESI, m/z): calc'd for $C_{21}H_{16}BiF_3SO_3Na^+$ $[M+Na]^+$ 637.0468; found 637.0471.

m.p. (°C): 131.6 – 133.0.

10-(4-chlorophenyl)-2-methyl-8-(trifluoromethyl)-10H-dibenzo[b,e][1,4]thiabismine 5,5-dioxide (124). Following the general procedure, using 136 mg (0.2 mmol) of bismine tosylate **47**, compound **124** was obtained in 93% yield (115 mg, 0.186 mmol) as a white solid (Ethyl acetate/Hexanes 1/4).

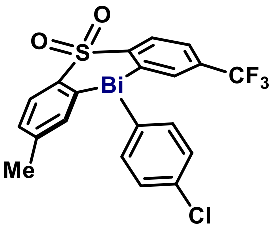

124

^{1}H NMR - (300 MHz, $CDCl_3$): δ 8.44 (d, *J* = 8.1 Hz, 1H), 8.28 (d, *J* = 7.9 Hz, 1H), 8.05 (s, 1H), 7.69 (d, *J* = 8.2 Hz, 4H), 7.40 (d, *J* = 8.2 Hz, 2H), 7.23 (d, *J* = 7.9 Hz, 1H), 2.29 (s, 3H).

^{13}C NMR - (75 MHz, $CDCl_3$): δ 164.3, 159.7, 159.2, 145.8, 145.1, 140.3, 138.3, 138.0, 135.4, 134.8 (q, *J* = 32.7 Hz), 134.3 (q, *J* = 3.6 Hz), 131.5, 129.4, 127.8, 127.0, 125.6 (q, *J* = 3.7 Hz), 123.4 (q, *J* = 273.0 Hz), 21.6.

^{19}F NMR - (282 MHz, $CDCl_3$): δ –62.9 (s, 3F).

HRMS (ESI, m/z): calc'd for $C_{20}H_{13}BiF_3SO_2ClNa^+$ $[M+Na]^+$ 640.9973; found 640.9973.

m.p. (°C): 140.1 – 141.4.

2-methyl-8-(trifluoromethyl)-10-(4-(trifluoromethyl)phenyl)-10H-dibenzo[b,e][1,4]thiabismine 5,5-dioxide (125). Following the general procedure, using 407 mg (0.6 mmol) of bismine tosylate **47**, compound **125** was obtained in 81% yield (317 mg, 0.486 mmol) as a white solid (Ethyl acetate/Hexanes 1/4).

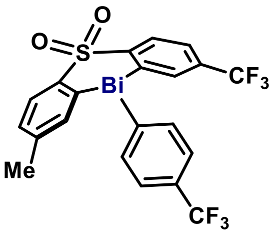

125

^{1}H NMR - (300 MHz, $CDCl_3$): δ 8.46 (d, *J* = 8.0 Hz, 1H), 8.30 (d, *J* = 7.9 Hz, 1H), 8.03 (s, 1H), 7.91 (d, *J* = 7.5 Hz, 2H), 7.67 (d, *J* = 8.0 Hz, 4H), 7.25 (d, *J* = 8.0 Hz, 1H), 2.29 (s, 3H).

^{13}C NMR - (75 MHz, $CDCl_3$): δ 170.1, 160.0, 159.4, 145.8 (q, *J* = 1.4 Hz), 145.2, 139.2, 138.2, 138.0, 135.0 (q, *J* = 32.5 Hz), 134.3 (q, *J* = 3.8 Hz), 131.2 (q, *J* = 32.5 Hz),129.5, 128.0, 127.7 (q, *J* = 3.7 Hz), 127.2, 125.7 (q, *J* = 3.5 Hz), 124.2 (q, *J* = 272.8 Hz), 123.3 (q, *J* = 272.5 Hz), 21.6.

^{19}F NMR - (282 MHz, $CDCl_3$): δ –62.90 (s, 3F), –62.93 (s, 3F).

HRMS (ESI, m/z): calc'd for $C_{21}H_{13}BiF_6SO_2Na^+$ $[M+Na]^+$ 675.0237; found 675.0246.

m.p. (°C): 171.5 – 172.8.

2-methyl-10-(4-(trifluoromethoxy)phenyl)-8-(trifluoromethyl)-10H-dibenzo[b,e][1,4]thiabismine 5,5-dioxide (126). Following the general procedure, using 136 mg (0.2 mmol) of bismine tosylate **47**, compound **126** was obtained in 79% yield (106 mg, 0.159 mmol) as a white solid (Ethyl acetate/Hexanes 1/4).

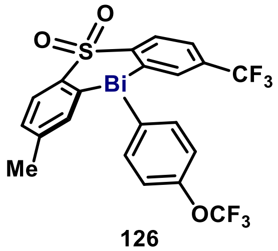

126

^{1}H NMR - (300 MHz, $CDCl_3$): δ 8.45 (d, J = 8.0 Hz, 1H), 8.30 (d, J = 7.9 Hz, 1H), 8.03 (s, 1H), 7.80 (d, J = 8.4 Hz, 2H), 7.68 (d, J = 8.5 Hz, 2H), 7.26 (t, J = 7.3 Hz, 3H), 2.30 (s, 3H).

^{13}C NMR - (75 MHz, $CDCl_3$): δ 164.3, 159.8, 159.3, 149.9 (q, J = 1.8 Hz), 145.8 (q, J = 1.8 Hz), 145.1, 140.5, 138.2, 138.1, 134.9 (q, J = 32.8 Hz), 134.4 (q, J = 3.8 Hz), 129.5, 127.9, 127.0, 125.6 (q, J = 3.8 Hz), 123.6, 123.3 (q, J = 273.8 Hz), 120.6 (q, J = 262.8 Hz), 21.6.

^{19}F NMR - (282 MHz, $CDCl_3$): δ –57.7 (s, 3F), –62.9 (s, 3F).

HRMS (ESI, m/z): calc'd for $C_{21}H_{13}BiF_6SO_3Na^+$ $[M+Na]^+$ 691.0186; found 691.0190.

m.p. (°C): 129.7 – 131.1.

Ethyl 4-(2-methyl-5,5-dioxido-8-(trifluoromethyl)-10H-dibenzo[b,e][1,4]thiabismin-10-yl)benzoate (127). Following the general procedure, using 136 mg (0.2 mmol) of bismine tosylate **47**, compound **127** was obtained in 95% yield (125 mg, 0.19 mmol) as a white solid (Ethyl acetate/Hexanes 1/4).

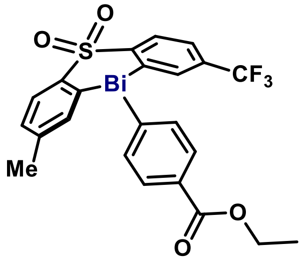

127

^{1}H NMR - (300 MHz, $CDCl_3$): δ 8.45 (d, J = 8.1 Hz, 1H), 8.29 (d, J = 7.9 Hz, 1H), 8.14 – 8.00 (m, 3H), 7.85 (d, J = 8.2 Hz, 2H), 7.67 (d, J = 8.5 Hz, 2H), 7.23 (d, J = 7.9 Hz, 1H), 4.38 (q, J = 7.1 Hz, 2H), 2.27 (s, 3H), 1.39 (t, J = 7.1 Hz, 3H).

^{13}C NMR - (75 MHz, $CDCl_3$): δ 172.2, 166.7, 159.8, 159.4, 145.8, 145.1, 138.8, 138.3, 138.0, 134.8 (q, J = 32.8 Hz), 134.4 (q, J = 3.8 Hz), 131.8, 131.0, 129.4, 127.9, 127.1, 125.6 (q, J = 3.8 Hz), 123.3 (q, J = 273.5 Hz), 61.3, 21.6, 14.4.

^{19}F NMR - (282 MHz, $CDCl_3$): δ –62.9 (s, 3F).

HRMS (ESI, m/z): calc'd for $C_{23}H_{18}BiF_3SO_4Na^+$ $[M+Na]^+$ 679.0574; found 679.0583.

m.p. (°C): 154.1 – 155.5.

10-(4-fluorophenyl)-2-methyl-8-(trifluoromethyl)-10H-dibenzo[b,e][1,4]thiabismine 5,5-dioxide (128). Following the general procedure, using 136 mg (0.2 mmol) of bismine tosylate **47**, compound **128** was obtained in 96% yield (116 mg, 0.193 mmol) as a white solid (Ethyl acetate/Hexanes 1/4).

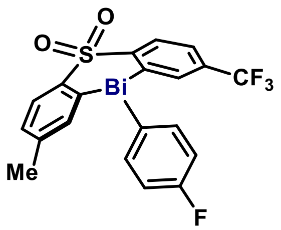

128

^{1}H NMR - (300 MHz, $CDCl_3$): δ 8.44 (d, *J* = 8.1 Hz, 1H), 8.28 (d, *J* = 7.9 Hz, 1H), 8.04 (s, 1H), 7.79 – 7.59 (m, 4H), 7.23 (d, *J* = 7.9 Hz, 1H), 7.19 – 7.05 (m, 2H), 2.29 (s, 3H).

^{13}C NMR - (75 MHz, $CDCl_3$): δ 163.2 (d, *J* = 249.8 Hz), 161.3, 159.7, 159.2, 145.8 (q, *J* = 1.6 Hz), 145.0, 140.9 (d, *J* = 7.8 Hz), 138.3, 138.1, 134.8 (q, *J* = 32.8 Hz), 134.4 (q, *J* = 3.8 Hz), 129.4, 127.8, 127.0, 125.5 (q, *J* = 3.8 Hz), 123.4 (q, *J* = 272.7 Hz), 118.7 (d, *J* = 19.8 Hz), 21.6.

^{19}F NMR - (282 MHz, $CDCl_3$): δ –62.9 (s, 3F), –110.4 (s, 1F).

HRMS (ESI, m/z): calc'd for $C_{20}H_{13}BiF_4SO_2Na^+$ $[M+Na]^+$ 625.0268; found 625.0271.

m.p. (°C): 171.3 – 172.7.

2-methyl-8-(trifluoromethyl)-10-(4-(trimethylsilyl)phenyl)-10H-dibenzo[b,e][1,4]thiabismine 5,5-dioxide (129). Following the general procedure, using 136 mg (0.2 mmol) of bismine tosylate **47**, compound **129** was obtained in 98% yield (129 mg, 0.196 mmol) as a white solid (Ethyl acetate/Hexanes 1/4).

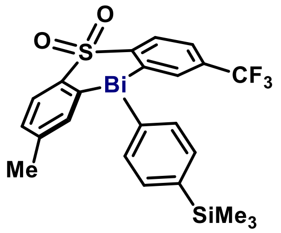

129

^{1}H NMR - (300 MHz, $CDCl_3$): δ 8.44 (d, *J* = 8.1 Hz, 1H), 8.29 (d, *J* = 7.9 Hz, 1H), 8.03 (s, 1H), 7.75 (d, *J* = 7.8 Hz, 3H), 7.69 – 7.58 (m, 3H), 7.23 (d, *J* = 7.9 Hz, 1H), 2.29 (s, 3H), 0.28 (s, 9H).

^{13}C NMR - (75 MHz, $CDCl_3$): δ 166.9, 159.5, 159.0, 145.8, 144.8, 141.9, 138.4, 138.1, 137.8, 135.9, 134.6 (q, *J* = 32.5 Hz), 134.4 (q, *J* = 3.8 Hz), 129.2, 127.8, 126.9, 125.4 (q, *J* = 3.8 Hz), 123.3 (q, *J* = 272.5 Hz), 21.6, –1.1.

^{19}F NMR - (282 MHz, $CDCl_3$): δ –62.9 (s, 3F).

HRMS (ESI, m/z): calc'd for $C_{23}H_{22}BiF_3SO_2SiNa^+$ $[M+Na]^+$ 679.0758; found 679.0767.

m.p. (°C): 194.6 – 196.1.

10-([1,1'-biphenyl]-4-yl)-2-methyl-8-(trifluoromethyl)-10H-dibenzo[b,e][1,4]thiabismine 5,5-dioxide (130). Following the general procedure, using 136 mg (0.2 mmol) of bismine tosylate **47**, compound **130** was obtained in 93% yield (123 mg, 0.186 mmol) as a white solid (Ethyl acetate/Hexanes 1/4).

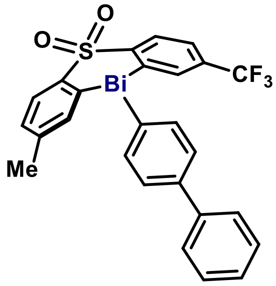

130

^{1}**H NMR** - (300 MHz, $CDCl_3$): δ 8.36 (d, *J* = 8.0 Hz, 1H), 8.20 (d, *J* = 7.9 Hz, 1H), 8.02 (s, 1H), 7.74 (d, *J* = 7.6 Hz, 2H), 7.66 (s, 1H), 7.63 – 7.54 (m, 3H), 7.51 (d, *J* = 7.4 Hz, 2H), 7.35 (t, *J* = 7.3 Hz, 2H), 7.30 – 7.20 (m, 1H), 7.13 (d, *J* = 8.2 Hz, 1H), 2.19 (s, 3H).

^{13}**C NMR** - (75 MHz, $CDCl_3$): δ 165.2, 159.6, 159.1, 145.8, 144.9, 141.8, 140.7, 139.2, 138.4, 138.1, 134.7 (q, *J* = 32.7 Hz), 134.5 (q, *J* = 3.7 Hz), 129.8, 129.3, 128.9, 127.8, 127.7, 127.2, 126.9, 125.4 (q, *J* = 3.7 Hz), 123.4 (q, *J* = 272.7 Hz), 21.6.

^{19}**F NMR** - (282 MHz, $CDCl_3$): δ –62.8 (s, 3F).

HRMS (ESI, m/z): calc'd for $C_{26}H_{18}BiF_3SO_2Na^+$ $[M+Na]^+$ 683.0681; found 683.0684.

m.p. (°C): 188.3 – 189.8.

2-methyl-10-(m-tolyl)-8-(trifluoromethyl)-10H-dibenzo[b,e][1,4]thiabismine 5,5-dioxide (131). Following the general procedure, using 136 mg (0.2 mmol) of bismine tosylate **47**, compound **131** was obtained in 97% yield (116 mg, 0.194 mmol) as a white solid (Ethyl acetate/Hexanes 1/4).

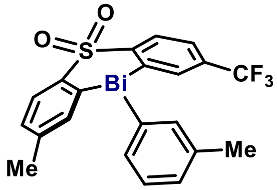

131

^{1}**H NMR** - (300 MHz, $CDCl_3$): δ 8.44 (d, *J* = 8.1 Hz, 1H), 8.29 (d, *J* = 7.9 Hz, 1H), 8.07 (s, 1H), 7.72 (s, 1H), 7.66 (d, *J* = 8.1 Hz, 1H), 7.62 (s, 1H), 7.53 (d, *J* = 7.3 Hz, 1H), 7.37 (t, *J* = 7.4 Hz, 1H), 7.22 (d, *J* = 7.3 Hz, 2H), 2.31 (s, 3H), 2.28 (s, 3H).

^{13}**C NMR** - (75 MHz, $CDCl_3$): δ 166.1, 159.5, 159.1, 145.8, 144.8, 140.7, 139.0, 138.4, 138.1, 135.7, 134.6 (q, *J* = 32.8 Hz), 134.5 (q, *J* = 3.8 Hz), 131.2, 129.9, 129.2, 127.7, 126.9, 125.3 (q, *J* = 32.8 Hz), 123.4 (q, *J* = 272.7 Hz), 21.6, 21.5.

^{19}**F NMR** - (282 MHz, $CDCl_3$): δ –62.9 (s, 3F).

HRMS (ESI, m/z): calc'd for $C_{21}H_{16}BiF_3SO_2Na^+$ $[M+Na]^+$ 621.0519; found 621.0524.

m.p. (°C): 86.0 – 87.5.

10-(3-isopropylphenyl)-2-methyl-8-(trifluoromethyl)-10H-dibenzo[b,e][1,4]thiabismine 5,5-dioxide (132). Following the general procedure, using 136 mg (0.2 mmol) of bismine tosylate **47**, compound **132** was obtained in 78% yield (98 mg, 0.156 mmol) as a white solid (Ethyl acetate/Hexanes 1/4).

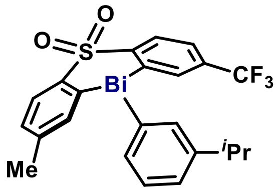

132

^{1}H NMR - (300 MHz, $CDCl_3$): δ 8.44 (d, *J* = 8.1 Hz, 1H), 8.29 (d, *J* = 7.9 Hz, 1H), 8.08 (s, 1H), 7.73 (s, 1H), 7.69 – 7.56 (m, 3H), 7.43 (t, *J* = 7.4 Hz, 1H), 7.31 – 7.16 (m, 2H), 2.83 (hept, *J* = 7.0 Hz, 1H), 2.27 (s, 3H), 1.18 (d, *J* = 1.0 Hz, 3H), 1.15 (d, *J* = 1.0 Hz, 3H).

^{13}C NMR - (75 MHz, $CDCl_3$): δ 166.3, 159.8, 159.3, 151.8, 145.8, 144.6, 138.4, 138.1, 136.9, 135.9, 134.5 (q, *J* = 33.0 Hz), 134.5 (q, *J* = 3.8 Hz), 131.0, 129.2, 127.8, 127.2, 126.9, 125.3, 123.4 (q, *J* = 273.0 Hz), 34.4, 24.0, 21.5.

^{19}F NMR - (282 MHz, $CDCl_3$): δ –62.9 (s, 3F).

HRMS (ESI, m/z): calc'd for $C_{23}H_{20}BiF_3SO_2Na^+$ $[M+Na]^+$ 649.0838; found 649.0838.

m.p. (°C): 85.8 – 87.3.

10-(3-(tert-butyl)phenyl)-2-methyl-8-(trifluoromethyl)-10H-dibenzo[b,e][1,4]thiabismine 5,5-dioxide (133). Following the general procedure, using 136 mg (0.2 mmol) of bismine tosylate **47**, compound **133** was obtained in 89% yield (114 mg, 0.178 mmol) as a white solid (Ethyl acetate/Hexanes 1/4).

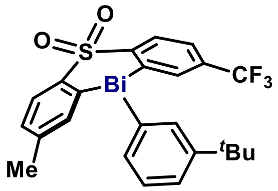

133

^{1}H NMR - (300 MHz, $CDCl_3$): δ 8.44 (d, *J* = 8.1 Hz, 1H), 8.29 (d, *J* = 7.9 Hz, 1H), 8.07 (s, 1H), 7.74 (d, *J* = 5.2 Hz, 2H), 7.68 – 7.59 (m, 2H), 7.48 – 7.40 (m, 2H), 7.22 (d, *J* = 8.8 Hz, 1H), 2.27 (s, 3H), 1.20 (s, 9H).

^{13}C NMR - (75 MHz, $CDCl_3$): δ 166.3, 160.0, 159.4, 154.0, 145.8, 144.6, 138.4, 138.1, 136.1, 135.4, 134.5 (q, *J* = 33.0 Hz), 134.5 (q, *J* = 3.8 Hz), 130.7, 129.2, 127.8, 126.9, 125.9, 125.3 (q, *J* = 3.8 Hz), 123.4 (q, *J* = 273.0 Hz), 35.2, 31.3, 21.5.

^{19}F NMR - (282 MHz, $CDCl_3$): δ –62.9 (s, 3F).

HRMS (ESI, m/z): calc'd for $C_{24}H_{22}BiF_3SO_2Na^+$ $[M+Na]^+$ 663.0994; found 663.0992.

m.p. (°C): 147.7 – 149.2.

10-(3-chlorophenyl)-2-methyl-8-(trifluoromethyl)-10H-dibenzo[b,e][1,4]thiabismine 5,5-dioxide (134). Following the general procedure, using 136 mg (0.2 mmol) of bismine tosylate **47**, compound **134** was obtained in 95% yield (118 mg, 0.191 mmol) as a white solid (Ethyl acetate/Hexanes 1/4).

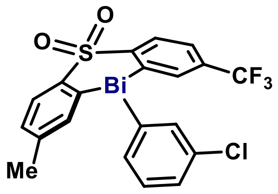

134

^{1}H NMR - (300 MHz, $CDCl_3$): δ 8.45 (d, *J* = 8.1 Hz, 1H), 8.29 (d, *J* = 7.9 Hz, 1H), 8.06 (s, 1H), 7.80 (s, 1H), 7.74 – 7.65 (m, 2H), 7.63 – 7.56 (m, 1H), 7.46 – 7.29 (m, 2H), 7.24 (d, *J* = 7.9 Hz, 1H), 2.29 (s, 3H).

^{13}C NMR - (75 MHz, $CDCl_3$): δ 167.5, 160.1, 159.6, 145.8, 145.1, 138.3, 138.2, 138.0, 137.9, 136.7, 134.9 (q, *J* = 32.7 Hz), 134.3 (q, *J* = 3.7 Hz), 132.6, 129.4, 129.3, 127.9, 127.1, 125.6 (q, *J* = 3.7 Hz), 123.5 (q, *J* = 272.7 Hz), 21.6.

^{19}F NMR - (282 MHz, $CDCl_3$): δ –62.9 (s, 3F).

HRMS (ESI, m/z): calc'd for $C_{20}H_{13}BiF_3SClO_2Na^+$ $[M+Na]^+$ 640.9973; found 640.9979.

m.p. (°C): 87.1 – 88.6.

3-(2-methyl-5,5-dioxido-8-(trifluoromethyl)-10H-dibenzo[b,e][1,4]thiabismin-10-yl)benzaldehyde (135). Following the general procedure, using 136 mg (0.2 mmol) of bismine tosylate **47**, compound **135** was obtained in 98% yield (120 mg, 0.196 mmol) as a white solid (Ethyl acetate/Hexanes 1/4).

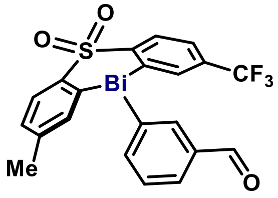

135

^{1}H NMR - (300 MHz, $CDCl_3$): δ 10.01 (s, 1H), 8.46 (d, *J* = 8.0 Hz, 1H), 8.36 (s, 1H), 8.30 (d, *J* = 7.9 Hz, 1H), 8.03 (s, 1H), 7.97 (d, *J* = 7.3 Hz, 1H), 7.91 (d, *J* = 7.7 Hz, 1H), 7.68 (d, *J* = 7.2 Hz, 2H), 7.59 (t, *J* = 7.5 Hz, 1H), 7.25 (d, *J* = 8.7 Hz, 1H), 2.27 (s, 3H).

^{13}C NMR - (75 MHz, $CDCl_3$): δ 192.5, 167.1, 159.9, 159.4, 145.8, 145.2, 145.1, 139.2, 138.2, 138.1, 138.0, 134.8 (q, *J* = 32.8 Hz), 134.3 (q, *J* = 3.6 Hz), 131.9, 129.6, 129.5, 128.0, 127.2, 125.7 (q, *J* = 3.8 Hz), 123.3 (q, *J* = 272.8 Hz), 21.6.

^{19}F NMR - (282 MHz, $CDCl_3$): δ –62.9 (s, 3F).

HRMS (ESI, m/z): calc'd for $C_{21}H_{14}BiF_3SO_3Na^+$ $[M+Na]^+$ 635.0311; found 635.0317.

m.p. (°C): 84.3 – 85.8.

2-methyl-8-(trifluoromethyl)-10-(3-(trimethylsilyl)phenyl)-10H-dibenzo[b,e][1,4]thiabismine 5,5-dioxide (136). Following the general procedure, using 136 mg (0.2 mmol) of bismine tosylate **47**, compound **136** was obtained in 95% yield (125 mg, 0.19 mmol) as a white solid (Ethyl acetate/Hexanes 1/4).

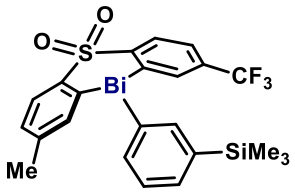

136

^{1}H NMR - (300 MHz, $CDCl_3$): δ 8.44 (d, *J* = 8.1 Hz, 1H), 8.29 (d, *J* = 7.9 Hz, 1H), 8.06 (s, 1H), 7.84 (s, 1H), 7.76 (dt, *J* = 7.3, 1.3 Hz, 1H), 7.72 (s, 1H), 7.66 (d, *J* = 8.7 Hz, 1H), 7.58 (dt, *J* = 7.3, 1.3 Hz, 1H), 7.44 (t, *J* = 7.3 Hz, 1H), 7.23 (d, *J* = 7.9 Hz, 1H), 2.27 (s, 3H), 0.18 (s, 9H).

^{13}C NMR - (75 MHz, $CDCl_3$): δ 165.7, 159.8, 159.3, 145.8, 144.7, 143.4, 143.3, 139.0, 138.4, 138.1, 134.6 (q, *J* = 32.7 Hz), 134.5 (q, *J* = 3.7 Hz), 133.9, 130.6, 129.2, 127.8, 126.9, 125.3 (q, *J* = 3.7 Hz), 123.4 (q, *J* = 272.3 Hz), 21.5, –1.1.

^{19}F NMR - (282 MHz, $CDCl_3$): δ –62.9 (s, 3F).

HRMS (ESI, m/z): calc'd for $C_{23}H_{22}BiF_3SSiO_2Na^+$ $[M+Na]^+$ 679.0758; found 679.0761.

m.p. (°C): 141.6 – 143.1.

2-methyl-8-(trifluoromethyl)-10-(3-(trifluoromethyl)phenyl)-10H-dibenzo[b,e][1,4]thiabismine 5,5-dioxide (137). Following the general procedure, using 136 mg (0.2 mmol) of bismine tosylate **47**, compound **137** was obtained in 74% yield (97 mg, 0.149 mmol) as a white solid (Ethyl acetate/Hexanes 1/4).

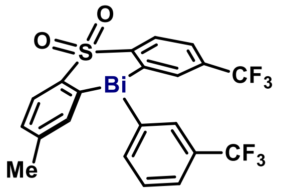

137

^{1}H NMR - (300 MHz, $CDCl_3$): δ 8.46 (d, *J* = 8.1 Hz, 1H), 8.30 (d, *J* = 7.9 Hz, 1H), 8.06 (d, *J* = 13.3 Hz, 2H), 7.92 (d, *J* = 7.3 Hz, 1H), 7.74 – 7.63 (m, 3H), 7.55 (t, *J* = 7.6 Hz, 1H), 7.25 (d, *J* = 8.0 Hz, 1H), 2.28 (s, 3H).

^{13}C NMR - (75 MHz, $CDCl_3$): δ 166.9, 160.0, 159.6, 145.8, 145.2, 142.4, 138.2, 138.0, 135.2 (q, *J* = 3.8 Hz), 134.9 (q, *J* = 32.8 Hz), 134.3 (q, *J* = 3.8 Hz), 133.0 (q, *J* = 33.0 Hz), 131.4, 129.5, 128.0, 127.2, 125.9 (q, *J* = 3.8 Hz), 125.7 (q, *J* = 3.8 Hz), 124.4 (q, *J* = 273.2 Hz), 123.3 (q, *J* = 272.8 Hz), 21.5.

^{19}F NMR - (282 MHz, $CDCl_3$): δ –62.6 (s, 3F), –63.0 (s, 3F).

HRMS (ESI, m/z): calc'd for $C_{21}H_{13}BiF_6SO_2Na^+$ $[M+Na]^+$ 675.0237; found 675.0237.

m.p. (°C): 71.2 – 72.6.

10-(3-methoxyphenyl)-2-methyl-8-(trifluoromethyl)-10H-dibenzo[b,e][1,4]thiabismine 5,5-dioxide (138). Following the general procedure, using 136 mg (0.2 mmol) of bismine tosylate **47**, compound **138** was obtained in 91% yield (112 mg, 0.182 mmol) as a white solid (Ethyl acetate/Hexanes 1/4).

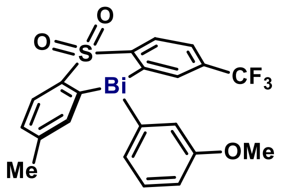

138

^{1}H NMR - (300 MHz, $CDCl_3$): δ 8.44 (d, *J* = 8.1 Hz, 1H), 8.28 (d, *J* = 7.9 Hz, 1H), 8.09 (s, 1H), 7.73 (s, 1H), 7.66 (d, *J* = 8.1 Hz, 1H), 7.49 – 7.39 (m, 1H), 7.37 (d, *J* = 2.6 Hz, 1H), 7.32 (d, *J* = 7.2 Hz, 1H), 7.22 (d, *J* = 7.9 Hz, 1H), 6.92 (ddd, *J* = 8.2, 2.6, 0.9 Hz, 1H), 3.71 (s, 3H), 2.28 (s, 3H).

^{13}C NMR - (75 MHz, $CDCl_3$): δ 167.0, 162.3, 159.7, 159.3, 145.8, 144.8, 138.3, 138.0, 134.6 (q, *J* = 32.6 Hz), 134.5 (q, *J* = 3.8 Hz), 132.3, 130.7, 129.3, 127.8, 126.9, 125.4 (q, *J* = 3.8 Hz), 124.1, 123.3 (q, *J* = 272.5 Hz), 144.8, 55.4, 21.6.

^{19}F NMR - (282 MHz, $CDCl_3$): δ –62.9 (s, 3F).

HRMS (ESI, m/z): calc'd for $C_{21}H_{16}BiF_3SO_3Na^+$ $[M+Na]^+$ 637.0468; found 637.0477.

m.p. (°C): 131.4 – 133.0.

10-(2-bromophenyl)-2-methyl-8-(trifluoromethyl)-10H-dibenzo[b,e][1,4]thiabismine 5,5-dioxide (139). Following the general procedure, using 136 mg (0.2 mmol) of bismine tosylate **47**, compound **139** was obtained in 89% yield (125 mg, 0.178 mmol) as a white solid (Ethyl acetate/Hexanes 1/4).

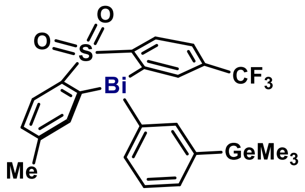

139

^{1}H NMR - (300 MHz, $CDCl_3$): δ 8.45 (d, *J* = 8.0 Hz, 1H), 8.29 (d, *J* = 7.9 Hz, 1H), 8.07 (s, 1H), 7.79 (s, 1H), 7.76 – 7.71 (m, 2H), 7.66 (d, *J* = 7.0 Hz, 1H), 7.53 (dt, *J* = 7.3, 1.3 Hz, 1H), 7.48 – 7.39 (m, 1H), 7.23 (d, *J* = 7.9 Hz, 1H), 2.28 (s, 3H), 0.31 (s, 9H).

^{13}C NMR - (75 MHz, $CDCl_3$): δ 165.8, 159.8, 159.3, 145.8, 145.7, 144.7, 142.9, 138.4, 138.3, 138.1, 134.6 (q, *J* = 32.7 Hz), 134.4 (q, *J* = 3.7 Hz), 133.5, 130.8, 129.2, 127.8, 126.9, 125.3 (q, *J* = 3.7 Hz), 123.4 (q, *J* = 272.8 Hz), 21.5, –1.7.

^{19}F NMR - (282 MHz, $CDCl_3$): δ –62.9 (s, 3F).

HRMS (ESI, m/z): calc'd for $C_{23}H_{22}BiF_3SGeO_2Na^+$ $[M+Na]^+$ 725.0200; found 725.0196.

m.p. (°C): 135.9 – 137.2.

2-methyl-10-(o-tolyl)-8-(trifluoromethyl)-10H-dibenzo[b,e][1,4]thiabismine 5,5-dioxide (140). Following the general procedure, using 136 mg (0.2 mmol) of bismine tosylate **47**, compound **140** was obtained in 93% yield (111 mg, 0.185 mmol) as a white solid (Ethyl acetate/Hexanes 1/4).

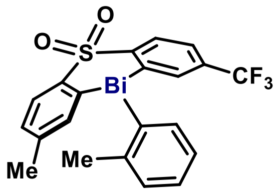

140

^{1}H NMR - (300 MHz, $CDCl_3$): δ 8.44 (d, *J* = 8.1 Hz, 1H), 8.27 (d, *J* = 7.9 Hz, 1H), 8.05 (s, 1H), 7.71 (s, 1H), 7.66 (d, *J* = 8.1 Hz, 1H), 7.57 (d, *J* = 7.4 Hz, 1H), 7.48 (d, *J* = 7.6 Hz, 1H), 7.33 (td, *J* = 7.5, 1.3 Hz, 1H), 7.22 (d, *J* = 7.9 Hz, 1H), 7.11 (t, *J* = 7.4 Hz, 1H), 2.57 (s, 3H), 2.27 (s, 3H).

^{13}C NMR - (75 MHz, $CDCl_3$): δ 167.8, 158.9, 158.5, 145.9, 144.7, 144.5, 141.1, 138.6, 138.1, 134.7 (q, *J* = 3.7 Hz), 134.5 (q, *J* = 32.7 Hz), 130.8, 129.7, 129.4, 129.2, 127.7, 126.8, 125.4 (q, *J* = 3.7 Hz), 123.4 (q, *J* = 272.6 Hz), 26.4, 21.6.

^{19}F NMR - (282 MHz, $CDCl_3$): δ –62.9 (s, 3F).

HRMS (ESI, m/z): calc'd for $C_{21}H_{16}BiF_3SO_2Na^+$ $[M+Na]^+$ 621.0525; found 621.0525.

m.p. (°C): 167.5 – 168.8.

10-(2-bromophenyl)-2-methyl-8-(trifluoromethyl)-10H-dibenzo[b,e][1,4]thiabismine 5,5-dioxide (141). Following the general procedure, using 136 mg (0.2 mmol) of bismine tosylate **47**, compound **141** was obtained in 85% yield (113 mg, 0.17 mmol) as a white solid (Ethyl acetate/Hexanes 1/4).

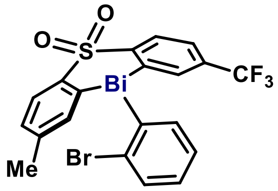

141

^{1}H NMR - (300 MHz, $CDCl_3$): δ 8.46 (d, *J* = 8.0 Hz, 1H), 8.30 (d, *J* = 7.9 Hz, 1H), 8.18 (s, 1H), 7.81 (s, 1H), 7.76 (dd, *J* = 7.8, 1.3 Hz, 1H), 7.68 (d, *J* = 8.1 Hz, 1H), 7.51 (dd, *J* = 7.3, 1.8 Hz, 1H), 7.29 – 7.21 (m, 2H), 7.17 (td, *J* = 7.3, 1.3 Hz, 1H), 2.31 (s, 3H).

^{13}C NMR - (75 MHz, $CDCl_3$): δ 169.5, 163.2, 162.9, 145.8, 145.2, 140.9, 138.3, 138.0, 134.9 (q, *J* = 32.7 Hz), 134.4 (q, *J* = 3.7 Hz), 132.7, 132.0, 131.1, 131.0, 129.3, 127.9, 127.1, 125.4 (q, *J* = 3.7 Hz), 123.4 (q, *J* = 272.6 Hz), 21.7.

^{19}F NMR - (282 MHz, $CDCl_3$): δ –62.9 (s, 3F).

HRMS (ESI, m/z): calc'd for $C_{20}H_{13}BiBrF_3SO_2Na^+$ $[M+Na]^+$ 684.9472; found 684.9468.

m.p. (°C): 123.3 – 124.7.

2-methyl-8-(trifluoromethyl)-10-(4-vinylphenyl)-10H-dibenzo[b,e][1,4]thiabismine 5,5-dioxide (142). Following the general procedure, using 136 mg (0.2 mmol) of bismine tosylate **47**, compound **142** was obtained in 81% yield (99 mg, 0.162 mmol) as a white solid (Ethyl acetate/Hexanes 1/4).

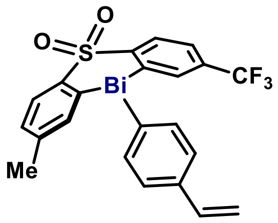

142

^{1}H NMR - (300 MHz, $CDCl_3$): δ 8.44 (d, *J* = 8.1 Hz, 1H), 8.28 (d, *J* = 7.9 Hz, 1H), 8.08 (s, 1H), 7.81 – 7.62 (m, 4H), 7.49 (d, *J* = 8.0 Hz, 2H), 7.22 (d, *J* = 8.8 Hz, 1H), 6.70 (dd, *J* = 17.6, 10.9 Hz, 1H), 5.79 (d, *J* = 18.3 Hz, 1H), 5.29 (d, *J* = 11.5 Hz, 1H), 2.28 (s, 3H).

^{13}C NMR - (75 MHz, $CDCl_3$): δ 166.1, 159.5, 159.1, 145.8, 144.9, 139.0, 138.4, 138.2, 138.1, 136.8, 134.7 (q, *J* = 32.7 Hz), 134.5 (q, *J* = 3.7 Hz), 129.3, 129.0, 127.7, 126.9, 125.4 (q, *J* = 3.7 Hz), 123.4 (q, *J* = 272.7 Hz), 115.0, 21.6.

^{19}F NMR - (282 MHz, $CDCl_3$): δ –62.8 (s, 3F).

HRMS (ESI, m/z): calc’d for $C_{22}H_{16}BiF_3SO_2Na^+$ [M+Na]$^+$ 633.0525; found 633.0523.

m.p. (°C): 205.4 – 206.8.

2-methyl-10-(4-(phenylethynyl)phenyl)-8-(trifluoromethyl)-10H-dibenzo[b,e][1,4]thiabismine 5,5-dioxide (143). Following the general procedure, using 136 mg (0.2 mmol) of bismine tosylate **47**, compound **143** was obtained in 85% yield (116 mg, 0.169 mmol) as a white solid (Ethyl acetate/Hexanes 1/4).

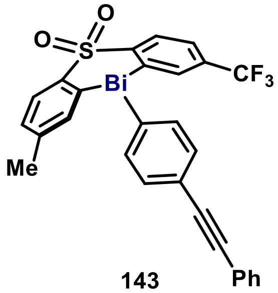

143

^{1}H NMR - (300 MHz, $CDCl_3$): δ 8.45 (d, *J* = 8.0 Hz, 1H), 8.29 (d, *J* = 7.9 Hz, 1H), 8.09 (s, 1H), 7.77 (d, *J* = 8.0 Hz, 2H), 7.73 – 7.65 (m, 2H), 7.61 (d, *J* = 8.0 Hz, 2H), 7.54 (dd, *J* = 6.7, 3.0 Hz, 2H), 7.40 – 7.31 (m, 3H), 7.23 (d, *J* = 7.9 Hz, 1H), 2.29 (s, 3H).

^{13}C NMR - (75 MHz, $CDCl_3$): δ 166.9, 159.8, 159.3, 145.8, 145.0, 138.8, 138.3, 138.0, 134.7 (q, *J* = 32.5 Hz), 134.4 (q, *J* = 3.8 Hz), 134.0, 131.8, 129.3, 128.7, 128.5, 127.8, 127.0, 125.5 (q, *J* = 3.8 Hz), 124.0, 123.3 (q, *J* = 272.5 Hz), 123.1, 91.0, 89.2, 21.6.

^{19}F NMR - (282 MHz, $CDCl_3$): δ –62.8 (s, 3F).

HRMS (ESI, m/z): calc’d for $C_{28}H_{18}BiF_3SO_2Na^+$ [M+Na]$^+$ 707.0676; found 707.0677.

m.p. (°C): 197.7 – 199.2.

10-(3,5-di-tert-butylphenyl)-2-methyl-8-(trifluoromethyl)-10H-dibenzo[b,e][1,4]thiabismine 5,5-dioxide (144). Following the general procedure, using 136 mg (0.2 mmol) of bismine tosylate **47**, compound **144** was obtained in 67% yield (93 mg, 0.134 mmol) as a white solid (Ethyl acetate/Hexanes 1/4).

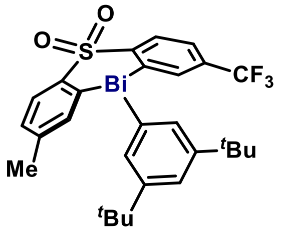

144

^{1}H NMR - (300 MHz, $CDCl_3$): δ 8.44 (d, *J* = 8.1 Hz, 1H), 8.28 (d, *J* = 7.9 Hz, 1H), 8.08 (s, 1H), 7.75 (s, 1H), 7.64 (d, *J* = 8.1 Hz, 1H), 7.59 (d, *J* = 1.8 Hz, 2H), 7.42 (t, *J* = 1.8 Hz, 1H), 7.25 – 7.18 (m, 1H), 2.27 (s, 3H), 1.24 (s, 18H).

^{13}C NMR - (75 MHz, $CDCl_3$): δ 165.9, 160.0, 159.5, 153.2, 145.9, 144.4, 138.5, 138.2, 134.6 (q, *J* = 3.8 Hz), 134.3 (q, *J* = 32.3 Hz), 132.7, 129.1, 127.8, 126.9, 125.2 (q, *J* = 3.8 Hz), 123.4 (q, *J* = 273.8 Hz), 122.8, 35.4, 31.5, 21.5.

^{19}F NMR - (282 MHz, $CDCl_3$): δ –62.9 (s, 3F).

HRMS (ESI, m/z): calc'd for $C_{28}H_{30}BiF_3SO_2Na^+$ $[M+Na]^+$ 719.1620; found 719.1620.

m.p. (°C): 166.4 – 167.8.

2-methyl-10-(naphthalen-2-yl)-8-(trifluoromethyl)-10H-dibenzo[b,e][1,4]thiabismine 5,5-dioxide (145). Following the general procedure, using 136 mg (0.2 mmol) of bismine tosylate **47**, compound **145** was obtained in 86% yield (109 mg, 0.172 mmol) as a white solid (Ethyl acetate/Hexanes 1/4) with an unidentified impurity which was not possible to separate.

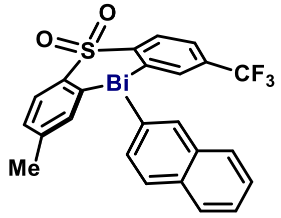

145

^{1}H NMR - (300 MHz, $CDCl_3$): δ 8.48 (d, *J* = 8.7 Hz, 2H), 8.32 (d, *J* = 7.9 Hz, 1H), 8.10 (s, 1H), 7.89 – 7.81 (m, 3H), 7.73 – 7.53 (m, 5H), 7.23 (d, *J* = 7.9 Hz, 1H), 2.23 (s, 3H).

^{13}C NMR - (75 MHz, $CDCl_3$): δ 163.5, 159.5, 159.1, 145.9, 144.9, 138.7, 138.4, 138.1, 135.6, 135.1, 134.6 (q, *J* = 32.8 Hz), 134.5 (q, *J* = 3.7 Hz), 133.4, 131.2, 130.3, 129.3, 128.2, 127.9, 127.8, 127.0, 126.9, 126.7, 125.5 (q, *J* = 3.8 Hz), 123.2 (q, *J* = 272.8 Hz), 21.5.

^{19}F NMR - (282 MHz, $CDCl_3$): δ –62.9 (s, 3F).

HRMS (ESI, m/z): calc'd for $C_{24}H_{16}BiF_3SO_2Na^+$ $[M+Na]^+$ 657.0519; found 657.05222.

m.p. (°C): 174.4 – 175.8.

2.2.5 Synthesis of fluorobismine species via reductive elimination

Fluorobismine **6** was synthesized following a previously reported protocotol.[2]

A similar method was employed for the synthesis of fluorobismines **28** and **146**, as shown below.

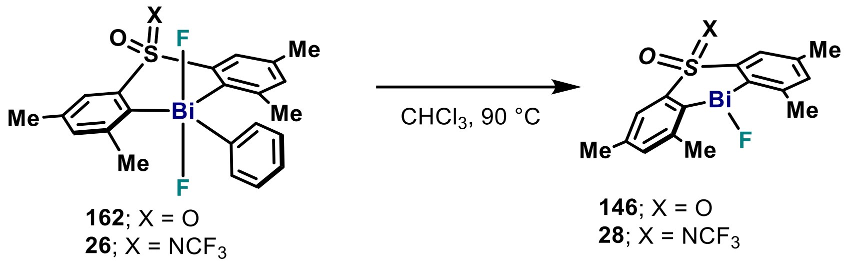

General procedure. The corresponding pentavalent bismine (0.2 mmol) was mixed with anhydrous $CHCl_3$ (1 mL). Then, the mixture was stirred at 90 °C (48 h for **162**; 16 h for **26**). The solvent was evaporated and the residue was washed with pentane (2 x 2 mL). The remaining white solid was dried under vacuum for 1 h. Single crystals suitable for XRD were obtained by slow diffusion of pentane in a concentrated solution of the corresponding fluorobismine in anhydrous $CHCl_3$ at 4 °C overnight.

10-fluoro-1,3,7,9-tetramethyl-5-((trifluoromethyl)imino)-5,10-dihydro-5λ⁴-dibenzo[b,e][1,4]thiabismine 5-oxide (28). Following the general procedure, compound **28** was obtained in 95% yield as off-white solid. Crystals suitable for XRD analysis were obtained by slow pentane diffusion into a concentrated solution of **28** in $CHCl_3$ at 4 °C.

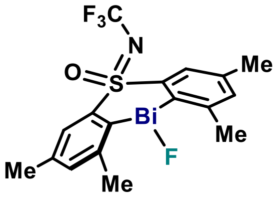

28

^{1}H NMR - (400 MHz, $CDCl_3$): δ 8.05 (s, 2H), 7.44 (s, 2H), 2.64 (s, 6H), 2.36 (s, 6H).

^{13}C NMR – (101 MHz, $CDCl_3$): δ 178.3 (q, J = 12.1 Hz), 145.4, 139.6, 139.1, 138.5, 127.3, 121.7 (q, J = 272.3 Hz), 22.8, 21.3.

^{19}F NMR - (377 MHz, $CDCl_3$): δ –41.6 (s, 3F), –184.0 (s, 1F).

HRMS (ESI, m/z): calc'd for $C_{17}H_{16}BiF_4SNONa^+$ $[M+Na]^+$ 590.0588; found 590.0585.

m.p. (°C): 244.9 – 246.2.

10-fluoro-1,3,7,9-tetramethyl-10H-dibenzo[b,e][1,4]thiabismine 5,5-dioxide (146). Following the general procedure, compound **146** was obtained in 78% yield as off-white solid. Crystals suitable for XRD analysis were obtained by slow pentane diffusion into a concentrated solution of **146** in $CHCl_3$ at 4 °C.

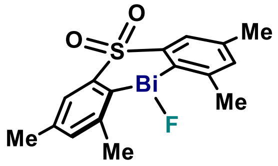

146

^{1}H NMR - (400 MHz, $CDCl_3$): δ 8.05 (s, 2H), 7.39 (s, 2H), 2.60 (s, 6H), 2.34 (s, 6H).

^{13}C NMR – (101 MHz, $CDCl_3$): δ 183.2 (q, *J* = 12.1 Hz), 145.1, 140.0, 139.3, 138.2, 126.9, 22.5, 21.2.

^{19}F NMR - (377 MHz, $CDCl_3$): δ –193.8 (s, 1F).

HRMS (ESI, m/z): calc'd for $C_{16}H_{16}BiFSO_2Na^+$ $[M+Na]^+$ 523.0551; found 523.0552.

m.p. (°C): 249.9 – 251.4.

3. Synthesis of Bi(V) compounds

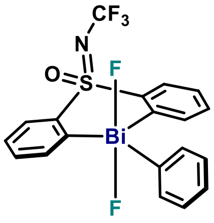

4

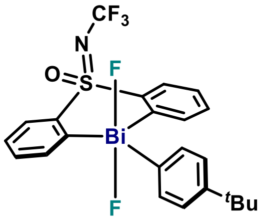

8

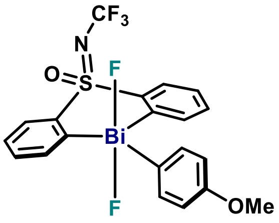

10

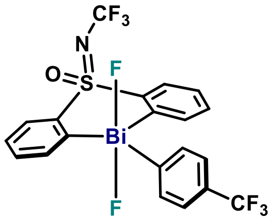

11

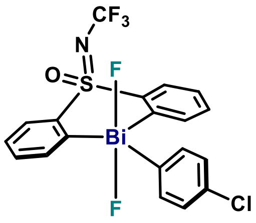

12

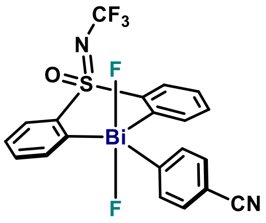

13

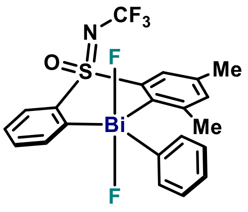

25

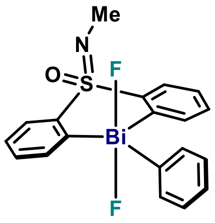

163

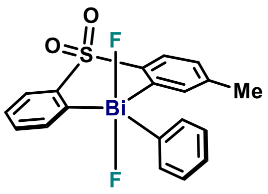

156

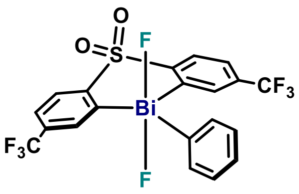

160

Aryldifluorobismuth(V) compounds **4**, **8**, **10-13**, **25**, **156**, **160** and **163** were synthesized according to previously reported protocols.[2-3] Compound **4** was recrystallized as a dimer from a concentrated MeCN solution at 4°C over 2 days.

3.1 General procedure for the synthesis of bismuth(V) species

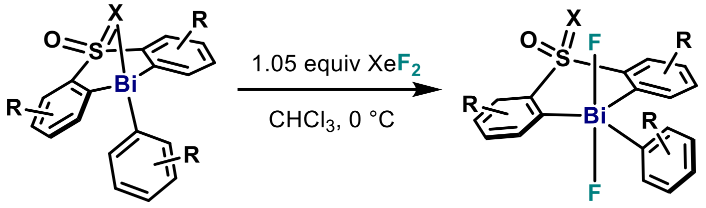

The corresponding arylbismine (1.0 equiv) was mixed with anhydrous $CHCl_3$ (2 mL) and the mixture was cooled with a water/ice bath. Then, XeF_2 (1.05 equiv) was added as a solid at once. The reaction was stirred over 1 h at 0 °C. Then, the solvent was evaporated and the residue was dried under vacuum for 1 h at room temperature. After oxidation, aryldifluorobismuth(V) compounds were obtained in quantitative yields.

*Note: complexes are depicted as monomers for simplicity, albeit they are expected to experience dynamic processes leading to monomeric and dimeric species in solution, similarly to species **4**.*

10-phenyl-5-((trifluoromethyl)imino)-5,10-dihydro-5λ^4-dibenzo[b,e][1,4]thiabismine 5-oxide (1): NMR, HRMS and m.p. characterization has been previously reported.[2]

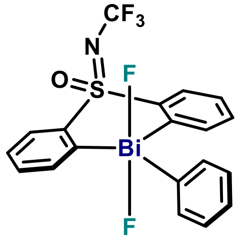

4

IR: 3064*w*, 1557*w*, 1468*w*, 1439*w*, 1429*w*, 1295*m*, 1239*s*, 1155*m*, 1086*s*, 1065*m*, 1033*w*, 1006*w*, 985*m*, 926*w*, 910*w*, 797*w*, 760*m*, 727*s*, 708*m*, 676*w*, 647*w*, 638*w*, 622*w*, 586*s*, 553*s*, 528*m*, 492*w*, 461*w*, 442*m*, 417*m*.

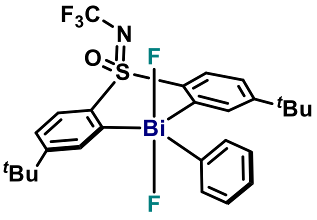

5

2,8-di-tert-butyl-10,10-difluoro-10-phenyl-5-((trifluoromethyl)imino)-5,10-dihydro-5λ^4,10λ^5-dibenzo[b,e][1,4]thiabismine 5-oxide (5). Following the general procedure, using 136 mg of bismine **85**, compound **5** was obtained in >95% yield as pale yellow solid.

^{1}H NMR - (300 MHz, $CDCl_3$): δ 8.53 (d, *J* = 1.7 Hz, 2H), 8.24 (d, *J* = 8.2 Hz, 2H), 8.23 (d, *J* = 8.3 Hz, 2H), 7.68 (t, *J* = 7.7 Hz, 2H), 7.63 – 7.56 (m, 3H), 1.35 (s, 18H).

^{13}C NMR - (75 MHz, $CDCl_3$): δ 166.8, 159.3, 156.7, 140.8, 133.2, 132.3, 131.8, 130.8, 127.1, 120.2 (q, *J* = 272.2 Hz), 35.1, 30.0.

^{19}F NMR - (282 MHz, $CDCl_3$): δ –41.9 (s, 3F), –120.9 (bs, 2F).

HRMS (ESI, m/z): calc'd for $C_{27}H_{29}BiF_5SNONa^+$ $[M+Na]^+$ 742.1586; found 742.1579.

IR: 3063*w*, 2964*w*, 2910*w*, 2869*w*, 1572*w*, 1552*w*, 1467*w*, 1440*w*, 1383*w*, 1365*w*, 1294*m*, 1270*m*, 1240*s*, 1157*m*, 1092*s*, 1062*m*, 1009*w*, 987*m*, 909*w*, 840*w*, 798*m*, 775*w*, 729*s*, 686*m*, 629*m*, 613*s*, 582*w*, 563*w*, 521*m*, 497*m*, 476*w*, 431*m*, 413*m*.

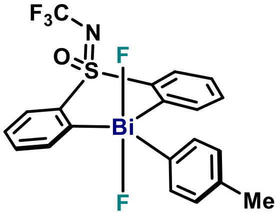

9

10,10-difluoro-10-(p-tolyl)-5-((trifluoromethyl)imino)-5,10-dihydro-5λ⁴,10λ⁵-dibenzo[b,e][1,4]thiabismine 5-oxide (9). Following the general procedure, using 156 mg of bismine **106**, compound **9** was obtained in >95% yield as yellow solid.

^{1}H NMR - (300 MHz, $CDCl_3$): δ 8.49 (d, J = 7.5 Hz, 2H), 8.34 (d, J = 7.8 Hz, 2H), 8.04 (d, J = 8.3 Hz, 2H), 7.82 (t, J = 7.6 Hz, 2H), 7.60 (t, J = 7.7 Hz, 2H), 7.43 (d, J = 8.2 Hz, 2H), 2.42 (s, 3H).

^{13}C NMR - (75 MHz, $CDCl_3$): δ 168.0, 155.1, 144.9, 143.8, 136.3, 135.9, 133.9, 132.6, 131.2, 128.4, 121.1 (q, *J* = 272.2 Hz), 21.5.

^{19}F NMR - (282 MHz, $CDCl_3$): δ –42.0 (s, 3F), –115.5 (bs, 2F).

HRMS (ESI, m/z): calc'd for $C_{20}H_{15}BiF_5SNONa^+$ $[M+Na]^+$ 644.0491; found 644.0490.

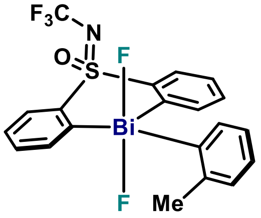

20

10,10-difluoro-10-(o-tolyl)-5-((trifluoromethyl)imino)-5,10-dihydro-5λ⁴,10λ⁵-dibenzo[b,e][1,4]thiabismine 5-oxide (20). Following the general procedure, using 117 mg of bismine **116**, compound **20** was obtained in >95% yield as yellow solid.

^{1}H NMR - (300 MHz, $CDCl_3$): δ 8.66 (d, *J* = 6.8 Hz, 2H), 8.30 (dd, *J* = 7.8, 1.3 Hz, 2H), 7.90 (d, *J* = 7.7 Hz, 1H), 7.78 (t, *J* = 7.4 Hz, 2H), 7.55 (t, *J* = 7.6 Hz, 2H), 7.46 – 7.32 (m, 3H), 2.66 (s, 3H).

^{13}C NMR - (75 MHz, $CDCl_3$): δ 170.7, 163.1, 144.6, 141.4, 136.7, 135.8, 133.4, 133.3, 132.2, 131.0, 128.7, 128.4, 121.3 (q, *J* = 262.7 Hz), 23.6.

^{19}F NMR - (282 MHz, $CDCl_3$): δ –42.7 (s, 3F), –102.5 (bs, 2F).

HRMS (ESI, m/z): calc'd for $C_{20}H_{15}BiF_5SNONa^+$ $[M+Na]^+$ 644.0491; found 644.0489.

10-(2-ethylphenyl)-10,10-difluoro-5-((trifluoromethyl)imino)-5,10-dihydro-5λ^4,10λ^5-dibenzo[b,e][1,4]thiabismine 5-oxide (21) and 10,10-difluoro-10-(2-isopropylphenyl)-5-((trifluoromethyl)imino)-5,10-dihydro-5l4,10l5-dibenzo[b,e][1,4]thiabismine 5-oxide (22).

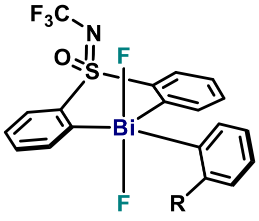

21, R = Et
22, R = ^{i}Pr

Following the general procedure, using 119 mg of bismine **117**, compound **21** could not be characterized by NMR techniques due to fast decomposition after oxidation with XeF_2, leading to fluorobismine **6** and the corresponding *ortho*-substituted fluoroarenes even at low temperatures (see Figures S1 and S2). Similar results were obtained with compound **22**, which was synthesized using 60 mg of compound **118**. However, formation of these compounds was confirmed by HRMS after oxidizing the corresponding aryl bismines. For **21**, **HRMS (ESI, m/z):** calc'd for $C_{21}H_{17}BiF_5SNONa^+$ $[M+Na]^+$ 658.0647; found 658.0645. For **22**, **HRMS (ESI, m/z):** calc'd for $C_{22}H_{19}BiF_5SNONa^+$ $[M+Na]^+$ 672.0804; found 672.0802.

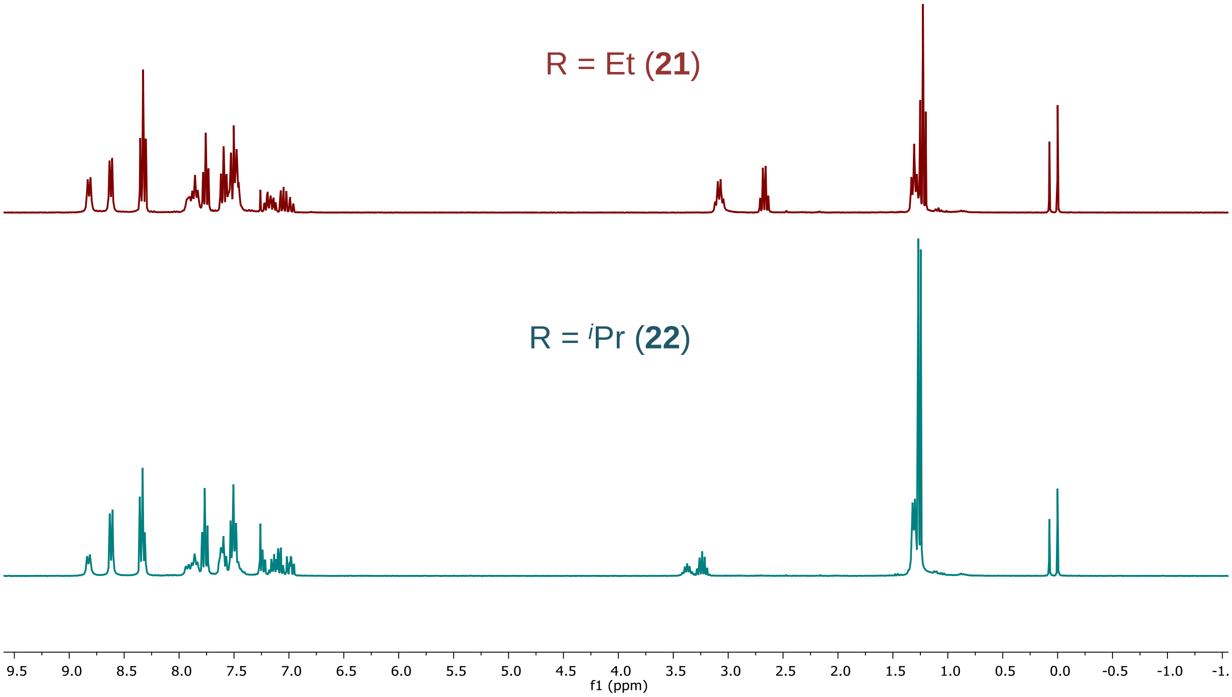

Figure S1. ^{1}H NMR of species **21** and **22** after oxidation with XeF_2 showing decomposition to trivalent bismine (δ ≈ 8.8, 8.6, 7.7, 7.5 ppm) species and fluoroarenes (δ ≈ 7.1 ppm).

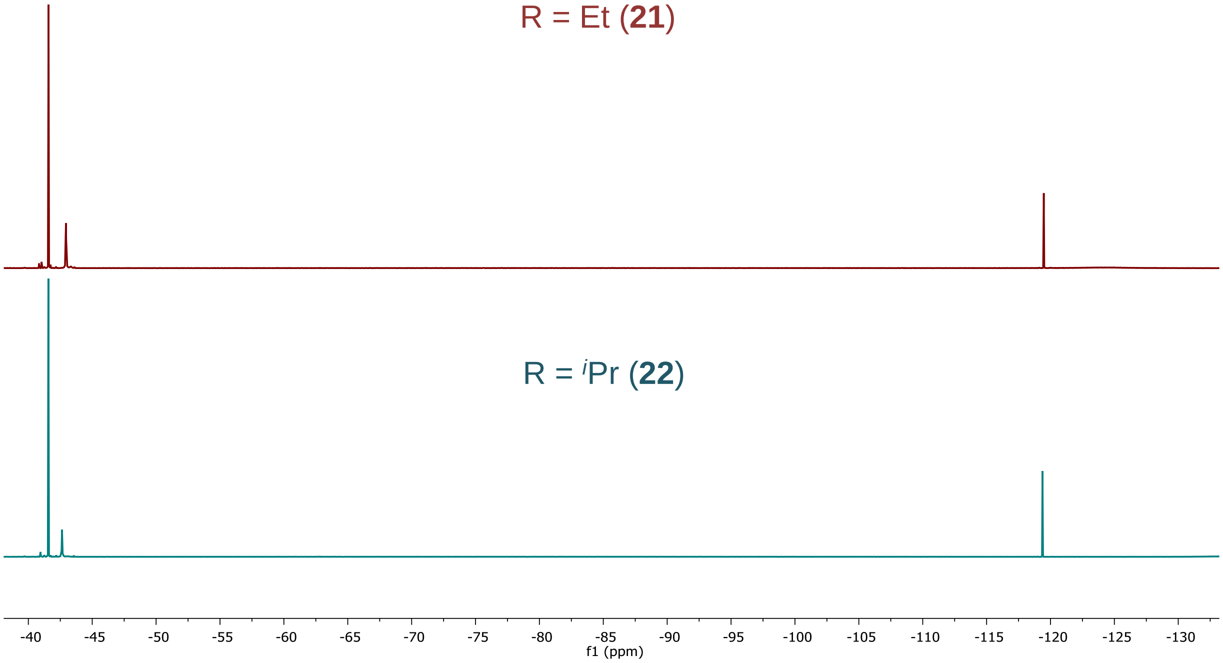

Figure S2. ^{19}F NMR of species **21** and **22** after oxidation with XeF_2 showing decomposition to trivalent bismine species ($\delta \approx -41$ ppm) and fluoroarenes ($\delta \approx -120$ ppm).

1,3-dimethyl-10-phenyl-5-((trifluoromethyl)imino)-5,10-dihydro-5λ^4-dibenzo[b,e][1,4]thiabismine 5-oxide (23): NMR, HRMS and m.p. characterization has been previously reported.[2]

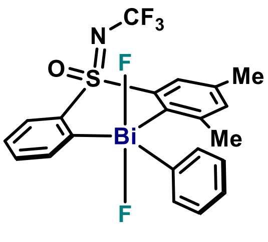

25

IR: 3077*w*, 3053*w*, 2965*w*, 2923*w*, 2863*w*, 1588*w*, 1557*w*, 1464*w*, 1440*w*, 1382*w*, 1288*m*, 1237*s*, 1158*m*, 1144*m*, 1091*s*, 1068*m*, 1030*w*, 1009*w*, 984*m*, 847*w*, 794*w*, 760*w*, 733*m*, 700*w*, 677*w*, 645*m*, 624*w*, 595*m*, 568*m*, 532*m*, 507*w*, 487*w*, 437*m*.

10,10-difluoro-1,3,7,9-tetramethyl-10-phenyl-5-((trifluoromethyl)imino)-5,10-dihydro-5λ[4],10λ[5]-dibenzo[b,e][1,4]thiabismine 5-oxide (26). Following the general procedure, using 150 mg of bismine **24a** or **24b**, compound **26** was obtained in >95% yield as pale yellow solid. Crystals suitable for XRD analysis were obtained by slow pentane diffusion into a concentrated solution of **26** in $CHCl_3$ at 4 °C.

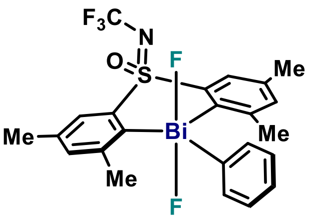

26

^{1}H NMR - (400 MHz, $CDCl_3$, 273 K): δ 8.53 (d, *J* = 8.0 Hz, 2H), 8.30 (s, 2H), 7.77 (t, *J* = 7.4 Hz, 2H), 7.62 (t, *J* = 7.1 Hz, 1H), 7.54 (s, 2H), 2.52 (s, 6H), 2.48 (s, 6H).

^{13}C NMR - (101 MHz, $CDCl_3$, 273 K): δ 156.0 (t, *J* = 12.0 Hz), 153.6 (t, *J* = 10.1 Hz), 144.3, 143.3, 143.1, 137.7, 134.1 (t, *J* = 5.3 Hz), 132.9, 132.7, 127.3, 120.8 (q, *J* = 272.3 Hz), 24.8, 21.7.

^{19}F NMR - (376 MHz, $CDCl_3$, 273 K): δ –43.3 (s, 3F), –127.6 (s, 2F).

HRMS (ESI, m/z): calc'd for $C_{23}H_{21}BiF_5SNONa^+$ $[M+Na]^+$ 686.0960; found 686.0956.

IR: 3090*w*, 3054*w*, 2919*w*, 1590*w*, 1560*w*, 1463*w*, 1449*w*, 1440*w*, 14321*w*, 1382*w*, 1290*s*, 1238*s*, 1156*m*, 1143*m*, 1096*s*, 1072*s*, 1038*w*, 1009*w*, 985*m*, 859*m*, 837*m*, 787*w*, 742*m*, 730*m*, 694*s*, 679*m*, 626*m*, 605*m*, 576*s*, 534*m*, 508*w*, 489*w*, 473*m*, 455*w*, 440*w*, 420*s*.

10,10-difluoro-1,3-dimethyl-10-(p-tolyl)-5-((trifluoromethyl)imino)-5,10-dihydro-5λ[4],10λ[5]-dibenzo[b,e][1,4]thiabismine 5-oxide (29). Following the general procedure, using 150 mg of bismine **88**, compound **29** was obtained in >95% yield as pale yellow solid.

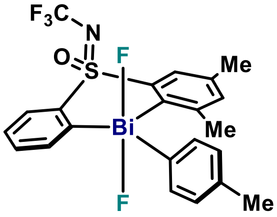

29

^{1}H NMR - (300 MHz, $CDCl_3$): δ 8.57 (dd, *J* = 7.9, 1.2 Hz, 1H), 8.33 (d, *J* = 8.5 Hz, 2H), 8.29 (s, 1H), 7.99 – 7.91 (m, 1H), 7.82 (td, *J* = 7.5, 1.3 Hz, 1H), 7.69 (td, *J* = 7.8, 1.2 Hz, 1H), 7.58 (d, *J* = 8.3 Hz, 2H), 7.53 (s, 1H), 2.61 (s, 3H), 2.50 (s, 3H), 2.48 (s, 3H).

^{13}C NMR - (75 MHz, $CDCl_3$): δ 157.9, 156.6, 149.6, 145.7, 145.0, 144.2, 143.1, 143.0, 137.6, 135.9, 134.9, 134.8, 133.2, 132.4, 129.1, 127.5, 121.0 (q, *J* = 262.3 Hz), 25.2, 21.6, 21.5.

^{19}F NMR - (282 MHz, $CDCl_3$): δ –43.0 (s, 3F), –119.5 (s, 2F).

HRMS (ESI, m/z): calc'd for $C_{22}H_{19}BiF_5SNONa^+$ $[M+Na]^+$ 672.0804; found 672.0796.

10,10-difluoro-10-(4-methoxyphenyl)-1,3-dimethyl-5-((trifluoromethyl)imino)-5,10-dihydro-5λ^4,10λ^5-dibenzo[b,e][1,4]thiabismine 5-oxide (30).

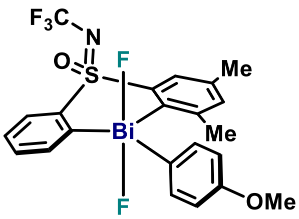

30

Following the general procedure, using 125 mg of bismine **90**, compound **30** was obtained in >95% yield as pale yellow solid.

^{1}H NMR - (300 MHz, $CDCl_3$): δ 8.57 (dd, *J* = 7.9, 1.3 Hz, 1H), 8.37 (d, *J* = 9.2 Hz, 2H), 8.29 (s, 1H), 7.96 (dd, *J* = 7.5, 1.2 Hz, 1H), 7.83 (td, *J* = 7.5, 1.4 Hz, 1H), 7.69 (td, *J* = 7.8, 1.3 Hz, 1H), 7.53 (s, 1H), 7.30 – 7.18 (m, 2H), 3.91 (s, 3H), 2.61 (s, 3H), 2.48 (s, 3H).

^{13}C NMR - (75 MHz, $CDCl_3$): δ 163.5, 157.7, 156.4, 145.8, 145.1, 143.1, 143.0, 142.4, 137.6, 136.6, 135.8, 134.7, 132.4, 129.1, 127.5, 121.1 (q, *J* = 261.3 Hz), 117.7, 55.9, 25.2, 21.6.

^{19}F NMR - (282 MHz, $CDCl_3$): δ –43.1 (s, 3F), –117.8 (s, 2F).

HRMS (ESI, m/z): calc'd for $C_{22}H_{19}BiF_5SNO_2Na^+$ $[M+Na]^+$ 688.0753; found 688.0754.

10,10-difluoro-1,3-dimethyl-5-((trifluoromethyl)imino)-10-(4-(trifluoromethyl)phenyl)-5,10-dihydro-5λ^4,10λ^5-dibenzo[b,e][1,4]thiabismine 5-oxide (31).

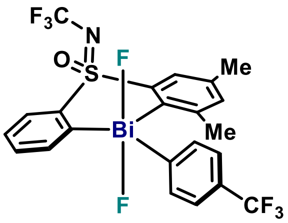

31

Following the general procedure, using 133 mg of bismine **89**, compound **31** was obtained in >95% yield as pale yellow solid.

^{1}H NMR - (300 MHz, $CDCl_3$): δ 8.65 (d, *J* = 8.4 Hz, 2H), 8.56 (dd, *J* = 7.9, 1.3 Hz, 1H), 8.27 (s, 1H), 8.04 – 7.93 (m, 3H), 7.85 (td, *J* = 7.5, 1.3 Hz, 1H), 7.71 (td, *J* = 7.7, 1.2 Hz, 1H), 7.56 (s, 1H), 2.60 (s, 3H), 2.49 (s, 3H).

^{13}C NMR - (75 MHz, $CDCl_3$): δ 158.4, 157.4, 157.2, 145.8, 145.1, 143.4, 143.1, 137.9, 136.1, 135.9, 135.2 (q, *J* = 33.7 Hz), 134.9, 132.6, 129.4, 128.9 (q, *J* = 3.6 Hz), 127.7, 123.0 (q, *J* = 273.3 Hz), 120.9 (q, *J* = 263.3 Hz), 25.2, 21.6.

^{19}F NMR - (282 MHz, $CDCl_3$): δ –43.1 (s, 3F), –63.1 (s, 3F), –115.6 (s, 2F).

HRMS (ESI, m/z): calc'd for $C_{22}H_{16}BiF_8SNONa^+$ $[M+Na]^+$ 726.0521; found 726.0514.

10,10-difluoro-2,8-dimethyl-10-phenyl-5-((trifluoromethyl)imino)-5,10-dihydro-5λ^{4},10λ^{5}-dibenzo[b,e][1,4]thiabismine 5-oxide (32). Following the general procedure, using 180 mg of bismine **84**, compound **32** was obtained in >95% yield as pale yellow solid.

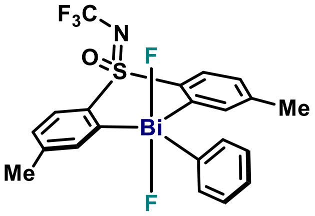

32

^{1}H NMR - (400 MHz, $CDCl_3$): δ 8.31 (s, 2H), 8.19 (d, *J* = 7.9 Hz, 4H), 7.65 (t, *J* = 7.6 Hz, 2H), 7.55 (t, *J* = 7.2 Hz, 1H), 7.38 (d, *J* = 8.6 Hz, 2H), 2.46 (s, 6H).

^{13}C NMR - (101 MHz, $CDCl_3$): δ 167.5, 158.2, 147.7, 142.0, 136.7, 134.2, 132.9, 131.9, 131.68, 128.2, 121.3 (q, *J* = 272.2 Hz), 22.1.

^{19}F NMR - (376 MHz, $CDCl_3$): δ –42.0 (s, 3F), –117.2 (bs, 2F).

HRMS (ESI, m/z): calc'd for $C_{21}H_{17}BiF_5SNONa^+$ $[M+Na]^+$ 658.0647; found 658.0646.

10,10-difluoro-2,8-dimethoxy-10-phenyl-5-((trifluoromethyl)imino)-5,10-dihydro-5l4,10l5-dibenzo[b,e][1,4]thiabismine 5-oxide (33). Following the general procedure, using 188 mg of bismine **87**, compound **33** was obtained in >95% yield as yellow solid.

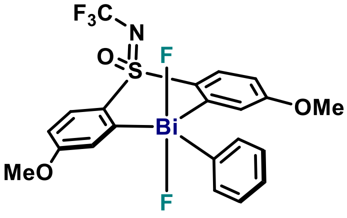

33

^{1}H NMR - (400 MHz, $CDCl_3$): δ 8.19 (d, *J* = 8.7 Hz, 4H), 8.09 (s, 2H), 7.65 (t, *J* = 7.0 Hz, 2H), 7.59 – 7.50 (m, 1H), 7.01 (dd, *J* = 8.7, 2.3 Hz, 2H), 3.90 (s, 6H).

^{13}C NMR - (101 MHz, $CDCl_3$): δ 170.7, 165.4, 159.0, 136.9, 134.0, 132.8, 132.0, 129.6, 121.2, 121.1 (q, *J* = 272.2 Hz), 116.7, 56.5.

^{19}F NMR - (376 MHz, $CDCl_3$): δ –41.8 (s, 3F), –117.5 (bs, 2F).

HRMS (ESI, m/z): calc'd for $C_{21}H_{17}BiF_5SNO_3Na^+$ $[M+Na]^+$ 690.0545; found 690.0540.

2,8,10,10-tetrafluoro-10-phenyl-5-((trifluoromethyl)imino)-5,10-dihydro-5λ^4,10λ^5-

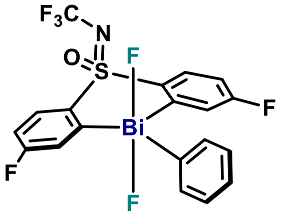

34

dibenzo[b,e][1,4]thiabismine 5-oxide (34). Following the general procedure, using 121 mg of bismine **86**, compound **34** was obtained in >95% yield as yellow solid. The sample is slightly contaminated with 5% of starting material **86**.

^{1}H NMR - (400 MHz, $CDCl_3$): δ 8.34 (dd, *J* = 8.6, 4.5 Hz, 2H), 8.21 (dd, *J* = 6.5, 2.1 Hz, 2H), 8.07 (d, *J* = 7.8 Hz, 2H), 7.60 (t, *J* = 7.6 Hz, 2H), 7.50 (t, *J* = 7.2 Hz, 1H), 7.24 (td, *J* = 8.3, 2.5 Hz, 2H).

^{13}C NMR - (101 MHz, $CDCl_3$): δ 172.3, 167.3 (d, *J* = 270 Hz), 160.0, 140.3, 133.4, 133.0, 132.2, 130.7 (d, *J* = 8.8 Hz), 124.3 (d, *J* = 24.2 Hz), 121.1 (q, *J* = 272.2 Hz), 118.3 (d, *J* = 24.2 Hz).

^{19}F NMR - (376 MHz, $CDCl_3$): δ –42.1 (s, 3F), –98.7 (s, 2F). Peaks corresponding to Bi–**F** not observed.

HRMS (ESI, m/z): calc'd for $C_{19}H_{11}BiF_7SNONa^+$ $[M+Na]^+$ 666.0146; found 666.0140.

10,10-difluoro-5-((trifluoromethyl)imino)-10-(3-(trimethylsilyl)phenyl)-5,10-dihydro-

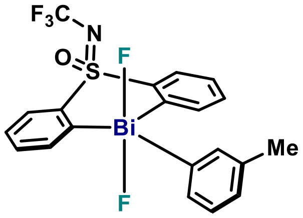

147

5l4,10l5-dibenzo[b,e][1,4]thiabismine 5-oxide (147). Following the general procedure, using 120 mg of bismine **112**, compound **147** was obtained in >95% yield as yellow solid. The high insolubility of this species prevented its characterization by ^{13}C NMR due to the appearance of broad bands, even at 363 K.

^{1}H NMR - (400 MHz, $CDCl_3$, 363 K): δ 8.57 (d, J = 7.5 Hz, 2H), 8.35 (d, J = 7.7 Hz, 2H), 8.00 (d, J = 31.1 Hz, 1H), 7.89 (d, J = 19.0 Hz, 1H), 7.82 (d, J = 7.6 Hz, 2H), 7.56 (dt, J = 21.8, 6.2 Hz, 3H), 7.38 (d, J = 7.7 Hz, 1H), 2.43 (s, 3H).

^{19}F NMR - (376 MHz, $CDCl_3$, 363 K): δ –42.0 (s, 3F), –120.1 (bs, 2F).

HRMS (ESI, m/z): calc'd for $C_{20}H_{15}BiF_5SNONa^+$ $[M+Na]^+$ 644.0491; found 644.0494.

10,10-difluoro-5-((trifluoromethyl)imino)-10-(3-(trimethylsilyl)phenyl)-5,10-dihydro-5λ⁴,10λ⁵-dibenzo[b,e][1,4]thiabismine 5-oxide (148). Following the general procedure, using 113 mg of bismine **114**, compound **148** was obtained in >95% yield as yellow solid.

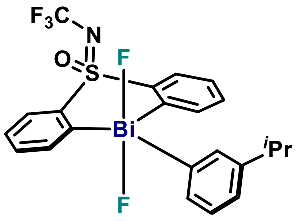

148

¹H NMR - (400 MHz, $CDCl_3$): δ 8.53 (dd, *J* = 7.8, 1.3 Hz, 2H), 8.34 (dd, *J* = 7.8, 1.3 Hz, 2H), 8.08 (s, 1H), 7.93 (d, *J* = 8.1 Hz, 1H), 7.84 (td, *J* = 7.6, 1.3 Hz, 2H), 7.65 – 7.55 (m, 3H), 7.42 (d, J = 7.6 Hz, 1H), 2.98 (hept, *J* = 6.9 Hz, 1H), 1.24 (d, *J* = 6.9 Hz, 6H).

¹³C NMR - (101 MHz, $CDCl_3$): δ 168.3, 157.7, 153.7, 145.0, 136.4, 135.9, 132.1, 131.8, 131.2(br)*, 131.1, 128.5, 121.2 (q, *J* = 261.4 Hz), 34.9, 23.8.

**Peak at 131.2 ppm is broad. Considering that we observe one signal less than expected, this peak might be an overlap of two signals.*

¹⁹F NMR - (376 MHz, $CDCl_3$): δ –42.0 (s, 3F), –118.0 (bs, 2F).

HRMS (ESI, m/z): calc'd for $C_{22}H_{19}BiF_5SNONa^+$ $[M+Na]^+$ 672.0804; found 672.0803.

10,10-difluoro-5-((trifluoromethyl)imino)-10-(3-(trimethylsilyl)phenyl)-5,10-dihydro-5λ⁴,10λ⁵-dibenzo[b,e][1,4]thiabismine 5-oxide (149). Following the general procedure, using 128 mg of bismine **120**, compound **149** was obtained in >95% yield as yellow solid.

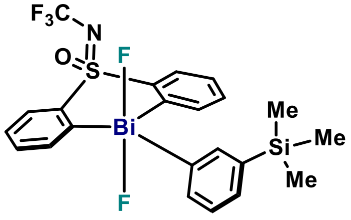

149

¹H NMR - (300 MHz, $CDCl_3$): δ 8.55 (d, *J* = 7.3 Hz, 2H), 8.35 (dd, *J* = 7.8, 1.2 Hz, 2H), 8.26 (s, 1H), 8.11 (d, *J* = 7.4 Hz, 1H), 7.86 (t, *J* = 7.5 Hz, 2H), 7.69 – 7.61(m, 4H), 0.26 (s, 9H).

¹³C NMR - (75 MHz, $CDCl_3$): δ 168.5, 158.4, 147.0, 144.9, 138.2, 137.7, 136.4, 135.9, 135.8, 134.0, 132.1, 131.3, 128.5, 121.3 (q, *J* = 262.1 Hz), –1.2.

*Partial decomposition of the compound was observed. The signals of decomposition products are not distinguishable from signals of **149**. Therefore, the number of signals is higher than expected.*

¹⁹F NMR - (282 MHz, $CDCl_3$): δ –42.0 (s, 3F), –120.7 (bs, 2F).

HRMS (ESI, m/z): calc'd for $C_{22}H_{21}BiF_5SSiNONa^+$ $[M+Na]^+$ 702.0729; found 702.0730.

10,10-difluoro-5-((trifluoromethyl)imino)-10-(3-(trimethylgermyl)phenyl)-5,10-dihydro-5λ^4,10λ^5-dibenzo[b,e][1,4]thiabismine 5-oxide (150). Following the general procedure, using 82 mg of bismine **119**, compound **150** was obtained in >95% yield as yellow solid.

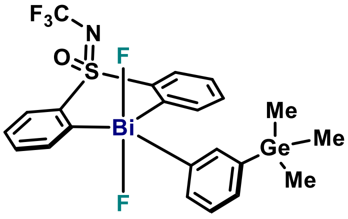

150

^{1}H NMR - (300 MHz, $CDCl_3$): δ 8.55 (d, *J* = 7.5 Hz, 2H), 8.34 (dd, *J* = 7.8, 1.3 Hz, 2H), 8.23 (s, 1H), 8.07 (d, *J* = 7.5 Hz, 1H), 7.85 (td, *J* = 7.6, 1.2 Hz, 2H), 7.69 – 7.57 (m, 4H), 0.39 (s, 9H).

^{13}C NMR - (75 MHz, $CDCl_3$): δ 168.7, 158.4, 149.2, 145.0, 137.8, 137.4, 136.4, 135.9, 133.5, 132.3, 131.2, 128.4, 121.0 (q, *J* = 262.1 Hz), –1.6.

^{19}F NMR - (282 MHz, $CDCl_3$): δ –41.9 (s, 3F), –118.9 (bs, 2F).

HRMS (ESI, m/z): calc'd for $C_{22}H_{21}BiF_5SGeNONa^+$ $[M+Na]^+$ 748.0172; found 748.0170.

10-(3-(tert-butyl)phenyl)-10,10-difluoro-5-((trifluoromethyl)imino)-5,10-dihydro-5λ^4,10λ^5-dibenzo[b,e][1,4]thiabismine 5-oxide (151). Following the general procedure, using 125 mg of bismine **115**, compound **151** was obtained in >95% yield as yellow solid.

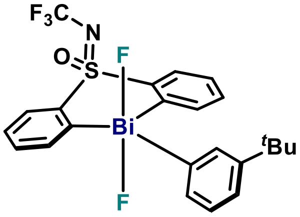

151

^{1}H NMR - (300 MHz, $CDCl_3$): δ 8.55 (d, *J* = 7.5 Hz, 2H), 8.34 (dd, *J* = 7.8, 1.3 Hz, 2H), 8.21 (s, 1H), 7.94 (dd, *J* = 6.7, 3.8 Hz, 1H), 7.85 (td, *J* = 7.6, 1.2 Hz, 2H), 7.70 – 7.55 (m, 4H), 1.30 (s, 9H).

^{13}C NMR - (75 MHz, $CDCl_3$): δ 168.5, 158.0, 156.1, 144.9, 136.4, 135.9, 131.6, 131.4, 131.2, 130.9, 130.1, 128.4, 121.3 (q, *J* = 272.2 Hz), 36.0, 31.2.

^{19}F NMR - (282 MHz, $CDCl_3$): δ –42.0 (s, 3F), –118.9 (bs, 2F).

HRMS (ESI, m/z): calc'd for $C_{23}H_{21}BiF_5SNONa^+$ $[M+Na]^+$ 686.0960; found 686.0958.

10,10-difluoro-5-((trifluoromethyl)imino)-10-(3-(trifluoromethyl)phenyl)-5,10-dihydro-5λ⁴,10λ⁵-dibenzo[b,e][1,4]thiabismine 5-oxide (152). Following the general procedure, using 127 mg of bismine **113**, compound **152** was obtained in >95% yield as yellow solid.

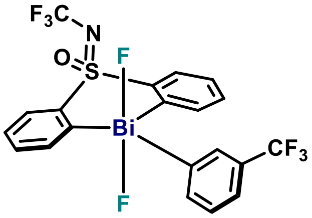

152

^{1}H NMR - (300 MHz, $CDCl_3$): δ 8.46 (s, 2H), 8.35 (d, *J* = 7.6 Hz, 3H), 7.88 – 7.69 (m, 4H), 7.63 (t, *J* = 7.7 Hz, 3H).

^{13}C NMR - (75 MHz, $CDCl_3$): δ 168.1, 160.1, 145.1, 137.3, 136.3, 136.1, 133.7 (q, *J* = 33.2 Hz), 131.9, 131.5, 129.2, 129.1, 128.8, 123.3 (q, *J* = 272.2 Hz), 121.4 (q, *J* = 262.1 Hz).

^{19}F NMR - (282 MHz, $CDCl_3$): δ –42.1 (s, 3F), –62.6 (s, 3F), –107.2 (bs, 2F).

HRMS (ESI, m/z): calc'd for $C_{20}H_{12}BiF_8SNONa^+$ $[M+Na]^+$ 698.0208; found 698.0206.

10-(3-chlorophenyl)-10,10-difluoro-5-((trifluoromethyl)imino)-5,10-dihydro-5λ⁴,10λ⁵-dibenzo[b,e][1,4]thiabismine 5-oxide (153). Following the general procedure, using 120 mg of bismine **111**, compound **153** was obtained in >95% yield as yellow solid.

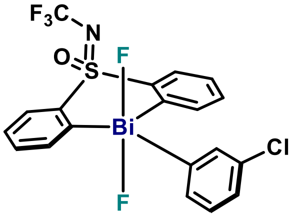

153

^{1}H NMR - (300 MHz, $CDCl_3$): δ 8.55 (d, *J* = 7.0 Hz, 2H), 8.35 (d, *J* = 6.7 Hz, 2H), 8.26 (s, 1H), 8.09 (d, *J* = 4.9 Hz, 1H), 7.85 (t, *J* = 6.6 Hz, 2H), 7.61 (dd, *J* = 16.6, 9.3 Hz, 4H).

^{13}C NMR - (75 MHz, $CDCl_3$): δ 168.3, 159.6, 145.1, 137.9, 136.3, 136.1, 133.5, 133.0, 132.2, 131.9, 131.4, 128.6, 121.2 (q, *J* = 272.2 Hz).

^{19}F NMR - (282 MHz, $CDCl_3$): δ –42.0 (s, 3F), –113.6 (bs, 2F).

HRMS (ESI, m/z): calc'd for $C_{19}H_{12}BiF_5SNClONa^+$ $[M+Na]^+$ 663.9944; found 663.9946.

10,10-difluoro-10-(3-methoxyphenyl)-5-((trifluoromethyl)imino)-5,10-dihydro-5λ^4,10λ^5-dibenzo[b,e][1,4]thiabismine 5-oxide (154). Following the general procedure, using 119 mg of bismine **109**, compound **154** was obtained in >95% yield as yellow solid.

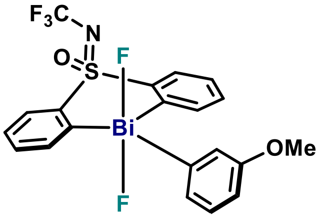

154

^{1}H NMR - (300 MHz, $CDCl_3$): δ 8.49 (dd, *J* = 7.6, 0.9 Hz, 2H), 8.34 (dd, *J* = 7.8, 1.3 Hz, 2H), 7.87 – 7.76 (m, 3H), 7.62 (qd, *J* = 8.3, 7.7, 0.8 Hz, 3H), 7.52 (t, *J* = 8.1 Hz, 1H), 7.06 (ddd, *J* = 8.2, 2.4, 0.8 Hz, 1H), 3.82 (s, 3H).

^{13}C NMR - (75 MHz, $CDCl_3$): δ 168.4, 162.2, 158.5, 145.0, 136.3, 136.0, 131.8, 131.3, 128.4, 125.7, 121.2 (q, *J* = 272.2 Hz), 119.7, 118.4, 56.0.

^{19}F NMR - (282 MHz, $CDCl_3$): δ –41.9 (s, 3F), –116.2 (bs, 2F).

HRMS (ESI, m/z): calc'd for $C_{20}H_{15}BiF_5SNO_2Na^+$ $[M+Na]^+$ 660.0440; found 660.0439.

10,10-difluoro-10-phenyl-10H-10λ^5-dibenzo[b,e][1,4]thiabismine 5,5-dioxide (155). Following the general procedure, using 151 mg of bismine **39**, compound **155** was obtained in >95% yield as pale yellow solid.

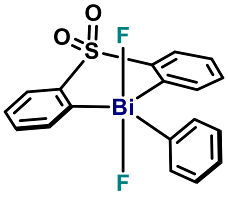

155

^{1}H NMR - (400 MHz, $CDCl_3$): δ 8.34 (d, *J* = 8.2 Hz, 2H), 8.30 (d, *J* = 7.8 Hz, 2H), 8.15 (d, *J* = 6.6 Hz, 2H), 7.75 (t, *J* = 7.3 Hz, 2H), 7.64 – 7.52 (m, 5H).

^{13}C NMR - (75 MHz, $CDCl_3$): δ 167.5, 156.9, 147.1, 135.9, 135.6, 134.3, 132.7, 132.1, 131.4, 128.3.

^{19}F NMR - (282 MHz, $CDCl_3$): δ –113.1 (bs, 2F).

HRMS (ESI, m/z): calc'd for $C_{18}H_{13}BiF_2SO_2Na^+$ $[M+Na]^+$ 563.0300; found 563.0299.

10,10-difluoro-2-methoxy-10-phenyl-10H-10l5-dibenzo[b,e][1,4]thiabismine 5,5-dioxide

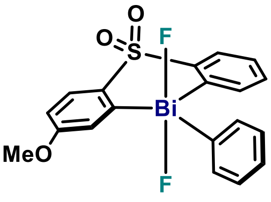

157

(157). Following the general procedure, using 160 mg of bismine **79**, compound **157** was obtained in >95% yield as pale yellow solid.

^{1}H NMR - (400 MHz, $CDCl_3$): δ 8.33 (d, *J* = 7.5 Hz, 1H), 8.27 (d, *J* = 7.1 Hz, 1H), 8.23 (d, *J* = 8.7 Hz, 1H), 8.15 (d, *J* = 7.6 Hz, 2H), 7.93 (s, 1H), 7.72 (t, *J* = 7.2 Hz, 1H), 7.62 (t, *J* = 7.4 Hz, 2H), 7.53 (dt, *J* = 7.1, 3.7 Hz, 2H), 6.97 (dd, *J* = 8.7, 2.4 Hz, 1H), 3.88 (s, 3H).

^{13}C NMR - (75 MHz, $CDCl_3$): δ 170.3, 168.8, 165.2, 157.6, 147.7, 137.9, 135.9, 135.1, 134.2, 132.7, 132.1, 131.2, 129.9, 127.7, 120.9, 116.6, 56.5.

^{19}F NMR - (282 MHz, $CDCl_3$): δ –113.8 (bs, 2F).

HRMS (ESI, m/z): calc'd for $C_{19}H_{15}BiF_2SO_3Na^+$ $[M+Na]^+$ 593.0406; found 593.0403.

2,8-di-tert-butyl-10,10-difluoro-10-phenyl-10H-10λ^5-dibenzo[b,e][1,4]thiabismine 5,5-

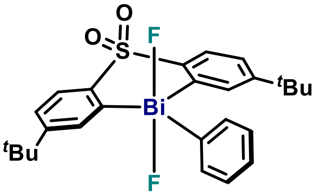

158

dioxide (158). Following the general procedure, using 184 mg of bismine **81**, compound **158** was obtained in >95% yield as pale yellow solid.

^{1}H NMR - (300 MHz, $CDCl_3$): δ 8.34 (s, 2H), 8.27 (dd, *J* = 8.0, 3.7 Hz, 4H), 7.70 (t, *J* = 7.7 Hz, 2H), 7.57 (dd, *J* = 8.2, 1.8 Hz, 3H), 1.33 (s, 18H).

^{13}C NMR - (75 MHz, $CDCl_3$): δ 167.1, 159.7, 155.5, 144.3, 134.8, 132.8, 132.2, 128.3, 128.1, 126.3, 36.2, 31.1.

^{19}F NMR - (282 MHz, $CDCl_3$): δ –120.4 (bs, 2F).

HRMS (ESI, m/z): calc'd for $C_{26}H_{29}BiF_2SO_2Na^+$ $[M+Na]^+$ 675.1552; found 675.1549.

10,10-difluoro-2-methyl-10-phenyl-8-(trifluoromethyl)-10H-10λ5-dibenzo[b,e][1,4]thiabismine 5,5-dioxide (159). Following the general procedure, using 175 mg of bismine **40**, compound **159** was obtained in >95% yield as pale yellow solid. The high insolubility of this species prevented its characterization by ^{13}C NMR due to the appearance of broad bands, similarly to compound **160**.[3]

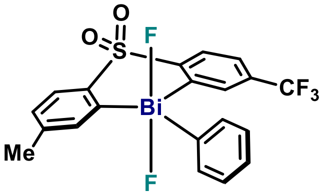

159

^{1}H NMR - (400 MHz, $CDCl_3$): δ 8.56 (s, 1H), 8.38 (s, 2H), 8.21 – 8.11 (m, 2H), 7.75 – 7.64 (m, 4H), 7.33 – 7.27 (m, 2H), 2.48 (s, 3H).

^{19}F NMR - (376 MHz, $CDCl_3$): δ –62.9 (s, 3F), –114.4 (bs, 2F).

HRMS (ESI, m/z): calc'd for $C_{20}H_{14}BiF_5SO_2Na^+$ $[M+Na]^+$ 645.0331; found 645.0328.

10,10-difluoro-1,3-dimethyl-10-phenyl-10H-10λ5-dibenzo[b,e][1,4]thiabismine 5,5-dioxide (161). Following the general procedure, using 55 mg of bismine **82**, compound **161** was obtained in >95% yield as pale yellow solid. Crystals suitable for XRD analysis were obtained by slow pentane diffusion into a concentrated solution of **161** in $CHCl_3$ at 4 °C.

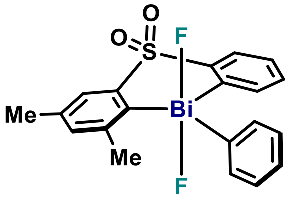

161

^{1}H NMR - (300 MHz, $CDCl_3$): δ 8.55 (dd, J = 7.8, 1.1 Hz, 1H), 8.44 (d, J = 7.9 Hz, 2H), 8.28 (s, 1H), 7.86 (d, J = 7.5 Hz, 1H), 7.77 (q, J = 7.8 Hz, 3H), 7.64 (t, J = 7.6 Hz, 2H), 7.49 (s, 1H), 2.60 (s, 3H), 2.45 (s, 3H).

^{13}C NMR - (75 MHz, $CDCl_3$): δ 157.9, 156.1, 151.7, 147.8, 147.3, 143.2, 142.3, 137.0, 135.3, 135.2, 134.5, 133.2, 132.6, 132.4, 128.4, 126.9, 25.3, 21.5.

^{19}F NMR - (282 MHz, $CDCl_3$): δ –122.3 (s, 2F).

HRMS (ESI, m/z): calc'd for $C_{20}H_{17}BiF_2SO_2Na^+$ $[M+Na]^+$ 591.0613; found 591.0609.

10,10-difluoro-1,3,7,9-tetramethyl-10-phenyl-10H-10λ5-dibenzo[b,e][1,4]thiabismine 5,5-dioxide (162). Following the general procedure, using 172 mg of bismine **83**, compound **162** was obtained in >95% yield as pale yellow solid. Crystals suitable for XRD analysis were obtained by slow pentane diffusion into a concentrated solution of **162** in $CHCl_3$ at 4 °C.

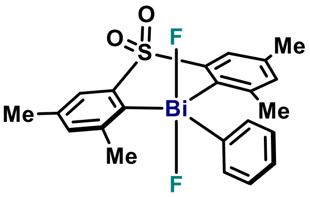

162

^{1}H NMR - (400 MHz, $CDCl_3$, 223 K): δ 8.50 (d, J = 8.1 Hz, 2H), 8.26 (s, 2H), 7.79 (t, J = 7.6 Hz, 2H), 7.63 (t, J = 7.2 Hz, 1H), 7.49 (s, 2H), 2.51 (s, 6H), 2.45 (s, 6H).

^{13}C NMR - (101 MHz, $CDCl_3$, 223 K): δ 155.0 (t, J = 11.4 Hz), 154.2 (t, J = 10.1 Hz), 146.7, 143.1, 142.81, 137.1, 133.9 (t, J = 5.3 Hz), 132.9, 132.7, 126.5, 24.8, 21.7.

^{19}F NMR - (376 MHz, $CDCl_3$, 223 K): δ –122.3 (s, 2F).

HRMS (ESI, m/z): calc'd for $C_{22}H_{21}BiF_2SO_2Na^+$ $[M+Na]^+$ 619.0926; found 619.0923.

10,10-difluoro-5-((perfluoroethyl)imino)-10-phenyl-5,10-dihydro-5λ4,10λ5-dibenzo[b,e][1,4]thiabismine 5-oxide (164). Following the general procedure, using 185 mg of bismine **42**, compound **164** was obtained in >95% yield as pale yellow solid.

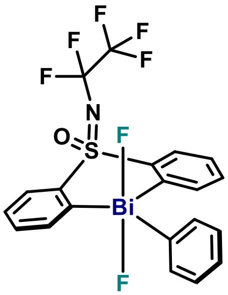

164

^{1}H NMR - (600 MHz, $CDCl_3$): δ 8.49 (d, J = 7.5 Hz, 2H), 8.36 (dd, J = 7.8, 1.3 Hz, 2H), 8.14 (d, J = 7.9 Hz, 2H), 7.86 – 7.79 (m, 2H), 7.67 – 7.58 (m, 4H), 7.56 (t, J = 7.3 Hz, 1H).

^{13}C NMR - (151 MHz, $CDCl_3$): δ 168.2, 158.3, 145.9, 136.4, 135.9, 134.0, 133.0, 132.0, 131.3, 128.4, 117.5 (qt, J = 283.1, 46.3 Hz), 113.9 (tq, J = 266.5, 40.7 Hz).

^{19}F NMR - (564 MHz, $CDCl_3$): δ –77.7 (s, 2F), –84.6 (s, 3F), –114.8 (bs, 2F).

HRMS (ESI, m/z): calc'd for $C_{20}H_{13}BiF_7SNONa^+$ $[M+Na]^+$ 680.0302; found 680.0306.

10,10-difluoro-5-((4-methoxyphenyl)imino)-10-phenyl-5,10-dihydro-5λ⁴,10λ⁵-dibenzo[b,e][1,4]thiabismine 5-oxide (165). Following the general procedure, using 84 mg of bismine **44**, compound **165** was obtained in ca. 90% yield as yellow solid, and the NMR analysis shows partial decomposition.

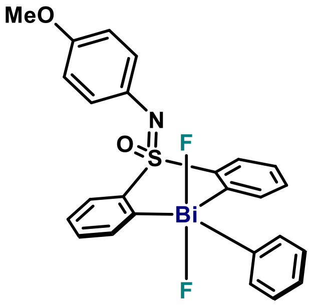

165

^{1}H NMR - (300 MHz, $CDCl_3$): δ 8.65 – 8.51 (m, 2H), 8.35 – 8.24 (m, 2H), 8.15 – 8.04 (m, 2H), 7.89 – 7.80 (m, 2H), 7.64 – 7.49 (m, 5H), 6.60 – 6.50 (m, 2H), 6.50 – 6.39 (m, 2H), 3.61 (s, 3H).

^{13}C NMR - (75 MHz, $CDCl_3$): δ 170.2, 159.2, 156.3, 144.9, 135.9, 135.3, 134.0, 132.9, 132.6, 131.9, 130.9, 128.7, 124.7, 114.6, 55.4.

^{19}F NMR - (282 MHz, $CDCl_3$): δ –122.0 (bs, 2F).

HRMS (ESI, m/z): calc'd for $C_{25}H_{20}BiF_2SNO_2Na^+$ $[M+Na]^+$ 668.0879; found 668.0883.

5-((4-(tert-butyl)phenyl)imino)-10,10-difluoro-10-phenyl-5,10-dihydro-5λ⁴,10λ⁵-dibenzo[b,e][1,4]thiabismine 5-oxide (166). Following the general procedure, using 101 mg of bismine **93**, compound **166** was obtained in >95% yield as yellow solid.

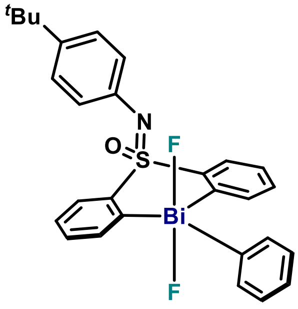

166

^{1}H NMR - (300 MHz, $CDCl_3$): δ 8.53 (dd, *J* = 7.6, 0.9 Hz, 2H), 8.31 (dd, *J* = 7.7, 1.3 Hz, 2H), 8.02 (dd, *J* = 7.7, 1.8 Hz, 2H), 7.81 (td, *J* = 7.5, 1.3 Hz, 2H), 7.60 (td, *J* = 7.6, 1.1 Hz, 2H), 7.55 – 7.46 (m, 3H), 6.89 – 6.82 (m, 2H), 6.49 – 6.40 (m, 2H), 1.13 (s, 9H).

^{13}C NMR - (75 MHz, $CDCl_3$): δ 170.3, 158.8, 146.5, 145.2, 137.5, 136.0, 135.2, 134.0, 132.5, 131.8, 130.8, 128.6, 126.1, 122.7, 34.2, 31.4.

^{19}F NMR - (282 MHz, $CDCl_3$): δ –117.4 (bs, 2F).

HRMS (ESI, m/z): calc'd for $C_{28}H_{26}BiF_2SNONa^+$ $[M+Na]^+$ 694.1399; found 694.1403.

10,10-difluoro-10-phenyl-5-(phenylimino)-5,10-dihydro-5λ[4],10λ[5]-dibenzo[b,e][1,4]thiabismine 5-oxide (167). Following the general procedure, using 93 mg of bismine **45**, compound **167** was obtained in >95% yield as yellow solid.

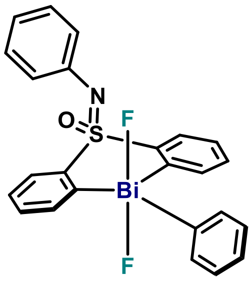

167

^{1}H NMR - (300 MHz, $CDCl_3$): δ 8.54 (dd, *J* = 7.6, 0.9 Hz, 2H), 8.31 (dd, *J* = 7.7, 1.3 Hz, 2H), 8.07 (dd, *J* = 8.0, 1.4 Hz, 2H), 7.82 (td, *J* = 7.5, 1.3 Hz, 2H), 7.60 (td, *J* = 7.6, 1.1 Hz, 2H), 7.57 – 7.48 (m, 3H), 6.94 – 6.85 (m, 2H), 6.85 – 6.78 (m, 1H), 6.55 (d, *J* = 8.1 Hz, 2H).

^{13}C NMR - (75 MHz, $CDCl_3$): δ 170.0, 158.8, 145.2, 140.4, 136.1, 135.3, 134.0, 132.7, 131.9, 130.9, 129.2, 128.7, 123.8, 123.4.

^{19}F NMR - (282 MHz, $CDCl_3$): –116.7 (bs, 2F).

HRMS (ESI, m/z): calc'd for $C_{24}H_{18}BiF_2SNONa^+$ $[M+Na]^+$ 638.0773; found 638.0777.

10,10-difluoro-10-phenyl-5-((4-(trifluoromethyl)phenyl)imino)-5,10-dihydro-5λ[4],10λ[5]-dibenzo[b,e][1,4]thiabismine 5-oxide (168). Following the general procedure, using 55 mg of bismine **94**, compound **168** was obtained in >95% yield as yellow solid.

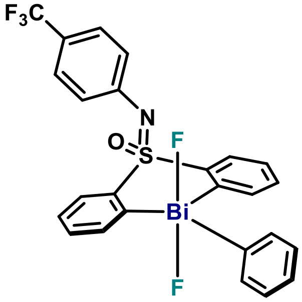

168

^{1}H NMR - (300 MHz, $CDCl_3$): δ 8.53 (d, *J* = 7.3 Hz, 2H), 8.36 (dd, *J* = 7.7, 1.1 Hz, 2H), 8.01 (dd, *J* = 6.3, 2.9 Hz, 2H), 7.88 – 7.78 (m, 2H), 7.63 (t, *J* = 7.6 Hz, 2H), 7.54 (d, *J* = 5.3 Hz, 3H), 7.11 (d, *J* = 8.5 Hz, 2H), 6.59 (d, *J* = 8.4 Hz, 2H).

^{13}C NMR - (75 MHz, $CDCl_3$): δ 169.3, 157.9, 144.9, 144.4, 136.3, 135.5, 134.0, 132.9, 132.0, 131.1, 128.8, 126.3 (q, *J* = 3.8 Hz), 125.3 (q, *J* = 33.4 Hz), 124.1 (q, *J* = 272.2 Hz), 122.9

^{19}F NMR - (282 MHz, $CDCl_3$): δ –62.2 (s, 3F), –116.2 (bs, 2F).

HRMS (ESI, m/z): calc'd for $C_{25}H_{17}BiF_5SNONa^+$ $[M+Na]^+$ 706.0647; found 706.0651.

5-((3,5-bis(trifluoromethyl)phenyl)imino)-10,10-difluoro-10-phenyl-5,10-dihydro-5λ^4,10λ^5-dibenzo[b,e][1,4]thiabismine 5-oxide (169). Following the general procedure, using 79 mg of bismine **95**, compound **169** was obtained in >95% yield as yellow solid.

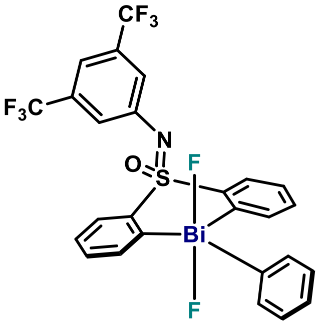

169

^{1}H NMR - (300 MHz, $CDCl_3$): δ 8.60 – 8.46 (m, 2H), 8.42 (dd, *J* = 7.8, 1.2 Hz, 2H), 8.10 – 7.95 (m, 2H), 7.85 (td, *J* = 7.6, 1.2 Hz, 2H), 7.66 (td, *J* = 7.7, 1.0 Hz, 2H), 7.58 (qd, *J* = 8.3, 7.8, 4.9 Hz, 3H), 7.29 (s, 1H), 6.91 (s, 2H).

^{13}C NMR - (75 MHz, $CDCl_3$): δ 168.2, 157.3, 144.7, 143.4, 136.6, 135.8, 134.0, 133.3, 132.3 (q, *J* = 7.6, 133.0 Hz), 132.2, 131.3, 129.1, 122.9 (q, *J* = 272.6 Hz), 122.5, 116.6 (quint, *J* = 3.7 Hz).

^{19}F NMR - (282 MHz, $CDCl_3$): δ –63.2 (s, 6F), –114.0 (bs, 2F).

HRMS (ESI, m/z): calc'd for $C_{26}H_{16}BiF_8SNONa^+$ $[M+Na]^+$ 774.0521; found 774.0514.

10,10-difluoro-5-((4-(pentafluoro-l6-sulfanyl)phenyl)imino)-10-phenyl-5,10-dihydro-5λ^4,10λ^5-dibenzo[b,e][1,4]thiabismine 5-oxide (170). Following the general procedure, using 91 mg of bismine **46**, compound **170** was obtained in >95% yield as yellow solid.

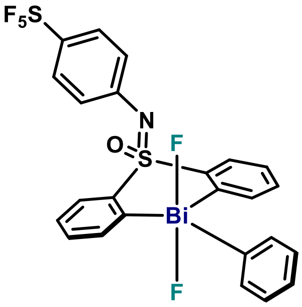

170

^{1}H NMR - (300 MHz, $CDCl_3$): δ 8.53 (dd, *J* = 7.6, 0.9 Hz, 2H), 8.37 (dd, *J* = 7.7, 1.3 Hz, 2H), 7.97 (dd, *J* = 6.3, 3.2 Hz, 2H), 7.85 (td, *J* = 7.6, 1.3 Hz, 2H), 7.64 (td, *J* = 7.6, 1.1 Hz, 2H), 7.54 (dd, *J* = 5.0, 1.7 Hz, 3H), 7.25 – 7.14 (m, 2H), 6.52 (d, *J* = 8.8 Hz, 2H).

^{13}C NMR - (75 MHz, $CDCl_3$): δ 169.1, 157.5, 148.8 (t, *J* = 18.4 Hz), 144.8, 144.2, 136.3, 135.6, 134.0, 133.1, 132.0, 131.2, 128.9, 126.9 (quint, *J* = 4.4 Hz), 122.3.

^{19}F NMR - (282 MHz, $CDCl_3$): δ 85.3 (quint, *J* = 150.1 Hz, 1F), 63.54 (d, *J* = 149.7 Hz, 4F), –115.94 (bs, 2F).

HRMS (ESI, m/z): calc'd for $C_{24}H_{17}BiF_7S_2NONa^+$ $[M+Na]^+$ 764.0336; found 764.0339.

(R)-10,10-difluoro-1,3-dimethyl-10-(m-tolyl)-5-((trifluoromethyl)imino)-5,10-dihydro-5λ^{4},10λ^{5}-dibenzo[b,e][1,4]thiabismine 5-oxide (171). Following the general procedure, using 133 mg of bismine **91**, compound **171** was obtained in >95% yield as pale yellow solid.

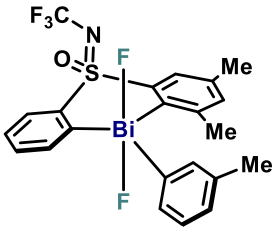

171

^{1}H NMR - (300 MHz, $CDCl_3$): δ 8.57 (d, *J* = 7.7 Hz, 1H), 8.29 (s, 2H), 8.24 (d, *J* = 8.0 Hz, 1H), 7.96 (d, J = 7.5 Hz, 1H), 7.83 (t, *J* = 7.5 Hz, 1H), 7.68 (q, *J* = 7.8 Hz, 2H), 7.53 (s, 1H), 7.44 (d, *J* = 7.5 Hz, 1H), 2.62 (s, 3H), 2.49 (s, 3H), 2.48 (s, 3H).

^{13}C NMR - (75 MHz, $CDCl_3$): δ 158.1, 156.6, 152.5, 145.7, 145.0, 143.4, 143.1, 142.9, 137.6, 135.9, 135.2, 134.8, 134.1, 132.4, 132.1, 132.0, 129.2, 127.5, 121.0 (q, *J* = 264.3 Hz), 25.3, 22.0, 21.6.

^{19}F NMR - (282 MHz, $CDCl_3$): δ –43.0 (s, 3F), –119.5 (bs, 2F).

HRMS (ESI, m/z): calc'd for $C_{22}H_{19}BiF_4SNO^+$ [M-F]$^+$ 630.0925; found 630.0922.

10,10-difluoro-1,3-dimethyl-5-((trifluoromethyl)imino)-10-(3-(trifluoromethyl)phenyl)-5,10-dihydro-5λ^{4},10λ^{5}-dibenzo[b,e][1,4]thiabismine 5-oxide (172). Following the general procedure, using 133 mg of bismine **92**, compound **172** was obtained in >95% yield as pale yellow solid.

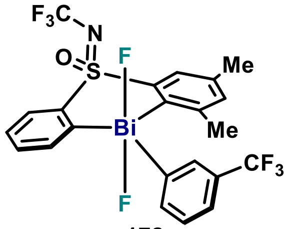

172

^{1}H NMR - (300 MHz, $CDCl_3$): δ 8.82 (s, 1H), 8.76 – 8.67 (m, 1H), 8.60 – 8.52 (m, 1H), 8.28 (s, 1H), 8.04 – 7.90 (m, 3H), 7.86 (td, *J* = 7.6, 1.3 Hz, 1H), 7.72 (td, *J* = 7.8, 1.1 Hz, 1H), 7.56 (s, 1H), 2.61 (s, 3H), 2.50 (s, 3H).

^{13}C NMR - (75 MHz, $CDCl_3$): δ 158.2, 156.9, 154.2, 145.9, 145.2, 143.5, 143.1, 138.8, 137.9, 136.2, 134.9, 134.8 (q, *J* = 33.7 Hz), 132.7, 132.6, 132.5 (q, *J* = 3.5 Hz), 130.1 (q, *J* = 3.5 Hz), 129.3, 127.7, 123.2 (q, *J* = 273.3 Hz), 120.9 (q, *J* = 263.3 Hz), 25.2, 21.6.

^{19}F NMR - (282 MHz, $CDCl_3$): δ –43.1 (s, 3F), –62.4 (s, 3F), –116.1 (bs, 2F).

HRMS (ESI, m/z): calc'd for $C_{22}H_{16}BiF_7SNO^+$ [M-F]$^+$ 684.0638; found 684.0639.

10-(3,5-di-tert-butylphenyl)-10,10-difluoro-1,3-dimethyl-5-((trifluoromethyl)imino)-5,10-dihydro-5λ^4,10λ^5-dibenzo[b,e][1,4]thiabismine 5-oxide (173). Following the general procedure, using 100 mg of bismine **121** compound **173** was obtained in >95% yield as pale yellow solid.

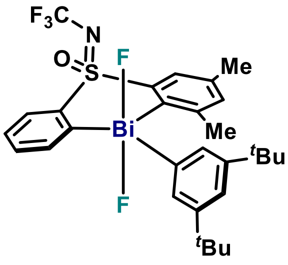

173

^{1}H NMR - (300 MHz, $CDCl_3$): δ 8.56 (d, *J* = 7.8 Hz, 1H), 8.28 (s, 1H), 8.22 (s, 2H), 8.06 (d, *J* = 7.5 Hz, 1H), 7.84 (t, *J* = 7.5 Hz, 1H), 7.69 (d, *J* = 7.9 Hz, 1H), 7.66 (s, 1H), 7.52 (s, 1H), 2.63 (s, 3H), 2.48 (s, 3H), 1.39 (s, 18H).

^{13}C NMR - (75 MHz, $CDCl_3$): δ 159.2, 158.0, 156.0, 153.7, 145.6, 145.1, 143.2, 142.7, 137.6, 135.9, 135.0, 132.2, 128.9, 128.6, 127.3, 126.2, 121.1 (q, *J* = 261.7 Hz), 36.4, 31.5, 25.1, 21.6.

^{19}F NMR - (282 MHz, $CDCl_3$): δ –42.9 (s, 3F), –120.4 (s, 2F).

HRMS (ESI, m/z): calc'd for $C_{29}H_{33}BiF_5SNONa^+$ [M+Na]$^+$ 770.1899; found 770.1888.

10-(3,5-di-tert-butylphenyl)-10,10-difluoro-5-((trifluoromethyl)imino)-5,10-dihydro-5l4,10l5-dibenzo[b,e][1,4]thiabismine 5-oxide (174). Following the general procedure, using 182 mg of bismine **107**, compound **174** was obtained in >95% yield as yellow solid.

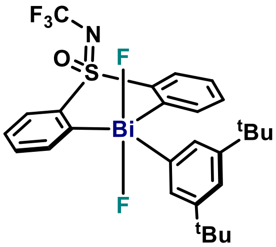

174

^{1}H NMR - (400 MHz, $CDCl_3$): δ 8.58 (d, *J* = 7.6 Hz, 2H), 8.34 (dd, *J* = 7.8, 1.3 Hz, 2H), 7.97 (d, *J* = 1.5 Hz, 2H), 7.86 (td, *J* = 7.6, 1.3 Hz, 2H), 7.68 – 7.55 (m, 3H), 1.30 (s, 18H).

^{13}C NMR - (75 MHz, $CDCl_3$): δ 169.0, 157.6, 155.5, 144.9, 136.4, 135.8, 131.2, 128.3, 127.9, 127.2, 121.3 (q, *J* = 272.2 Hz), 36.2, 31.3.

^{19}F NMR - (282 MHz, $CDCl_3$): δ –42.0 (s, 3F), –120.7 (bs, 2F).

HRMS (ESI, m/z): calc'd for $C_{27}H_{29}BiF_5SNONa^+$ [M+Na]$^+$ 742.1586; found 742.1578.

10,10-difluoro-10-(4-methoxyphenyl)-1,3-dimethyl-5-((trifluoromethyl)imino)-5,10-dihydro-5λ⁴,10λ⁵-dibenzo[b,e][1,4]thiabismine 5-oxide (175). Following the general procedure, using 100 mg of bismine **96a** or **96b**, compound **175** was obtained in >95% yield as pale yellow solid. Crystals suitable for XRD analysis were obtained by slow pentane diffusion into a concentrated solution of **175** in $CHCl_3$ at 4 °C.

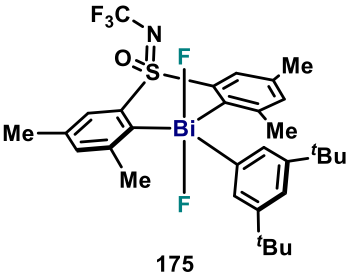

175

^{1}H NMR - (300 MHz, $CDCl_3$): δ 8.36 (d, *J* = 1.5 Hz, 2H), 8.31 (s, 2H), 7.61 (t, *J* = 1.5 Hz, 1H), 7.48 (s, 2H), 2.54 (s, 6H), 2.47 (s, 6H), 1.39 (s, 18H).

^{13}C NMR - (75 MHz, $CDCl_3$): δ 157.7, 156.0, 155.2, 145.5, 143.2, 142.9, 137.4, 127.9, 127.3, 126.6, 121.0 (q, *J* = 261.7 Hz), 36.4, 31.5, 24.9, 21.6.

^{19}F NMR - (282 MHz, $CDCl_3$): δ –43.0 (s, 3F), –124.1 (bs, 1F), –125.4 (bs, 1F).

HRMS (EI, m/z): calc'd for $C_{31}H_{37}BiF_4SNO^+$ $[M–F]^+$ 756.2330; found 756.2326.

10,10-difluoro-10-(p-tolyl)-5-((trifluoromethyl)imino)-5,10-dihydro-5λ⁴,10λ⁵-dibenzo[b,e][1,4]thiabismine 5-oxide (176). Following the general procedure, using 50 mg of bismine **108**, the synthesis of compound **176** was attempted but full decomposition was observed (to the corresponding Bi(III)–F and aryl fluoride), even at low temperatures.

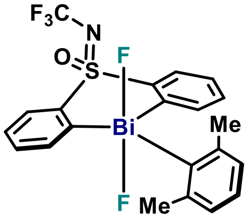

176

10-(2,6-dimethylphenyl)-10,10-difluoro-10H-10l5-dibenzo[b,e][1,4]thiabismine 5,5-dioxide (177). Following the general procedure, using 106 mg of bismine **110**, compound **177** was obtained in 90% yield as yellow solid. When analyzed by NMR, traces of decomposition product (Bi-F byproduct) were observed even at low temperatures, which prevented its characterization by ^{13}C NMR.

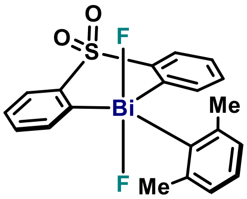

177

^{1}H NMR - (400 MHz, $CDCl_3$): δ 8.78 (d, J = 7.7 Hz, 2H), 8.20 (d, J = 7.6 Hz, 2H), 7.90 – 7.74 (m, 2H), 7.54 (t, J = 7.5 Hz, 2H), 7.30 (s, 3H), 2.80 (s, 6H).

^{19}F NMR - (282 MHz, $CDCl_3$): δ –106.3 (bs, 2F).

HRMS (ESI, m/z): calc'd for $C_{20}H_{17}BiF_2SO_2Na^+$ $[M+Na]^+$ 591.0613; found 591.0607.

3.2 Aggregation studies of **4**, **5**, **25** and **26**

3.2.1 Dilution experiments

In a NMR tube, pentavalent bismine **4** (0.0013-0.019 mmol, *dimer*), **5** (0.0015-0.061 mmol, *dimer*), **25** (0.0025-0.125 mmol, *monomer*) or **26** (0.0029-0.125 mmol, *monomer*) was mixed with anhydrous CD_2Cl_2 (0.5 mL). NMR samples were placed at a preheated NMR machine (25 °C) and the ^{1}H and ^{19}F NMR spectra were recorded. As shown in Figures S3-S6, bismines **4** and **5** show remarkable peak movement at different concentrations as well as significant peak broadening in ^{1}H NMR spectra, indicating a higher tendency for aggregation. However, **25** and **26** show much smaller peak movement as well as minimal peak broadening in ^{1}H NMR spectra, suggesting their preference for the monomeric forms in solution at room temperature.

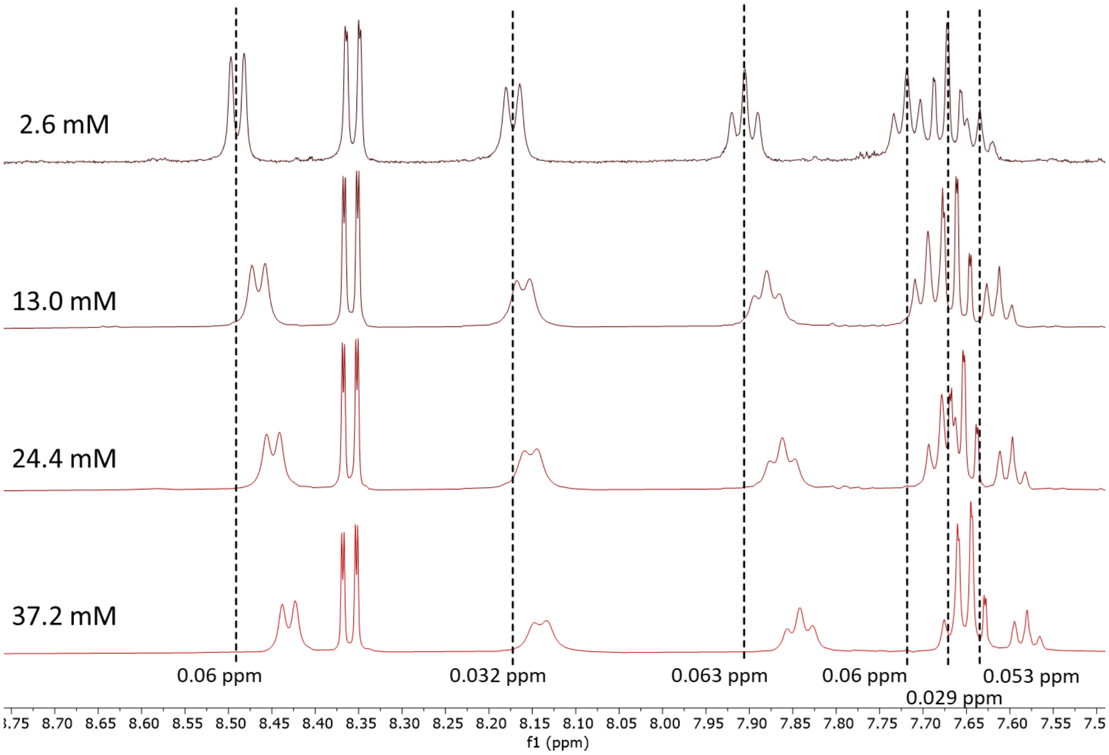

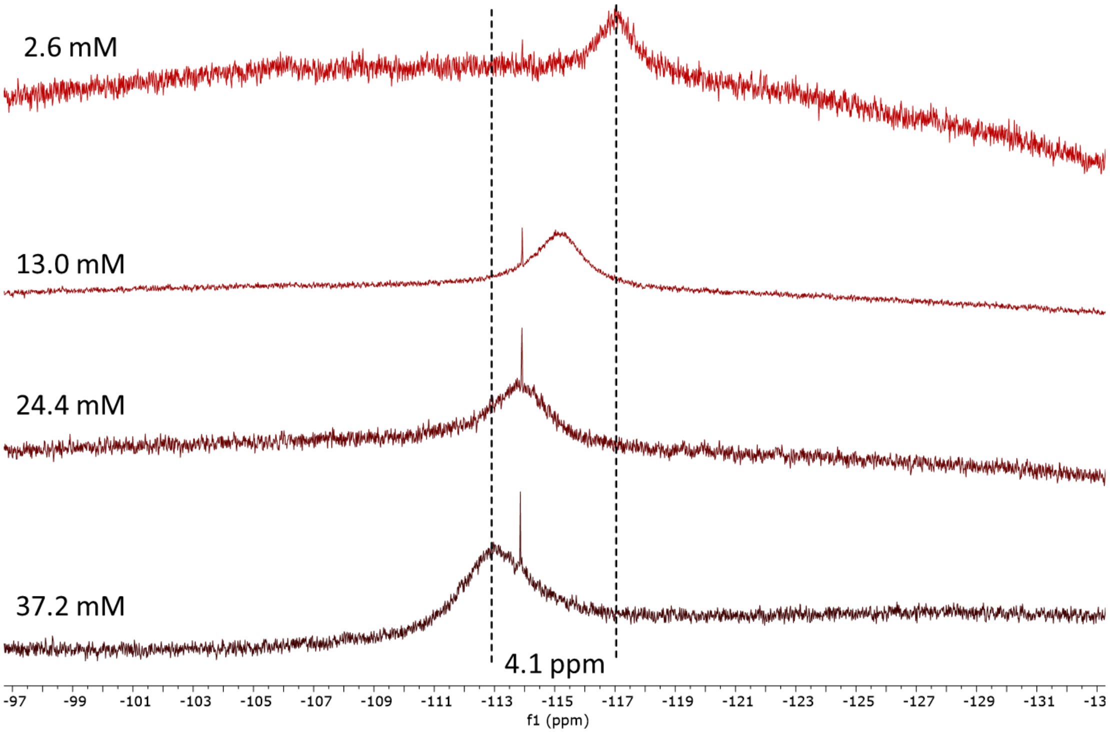

Figure S3. ^{1}H NMR aromatic region (top) and ^{19}F NMR (bottom) of **4** at different concentrations in CD_2Cl_2. Significant peak movement and broadening are observed when the concentration of **4** increases, indicating dimerization in solution at 25 °C.

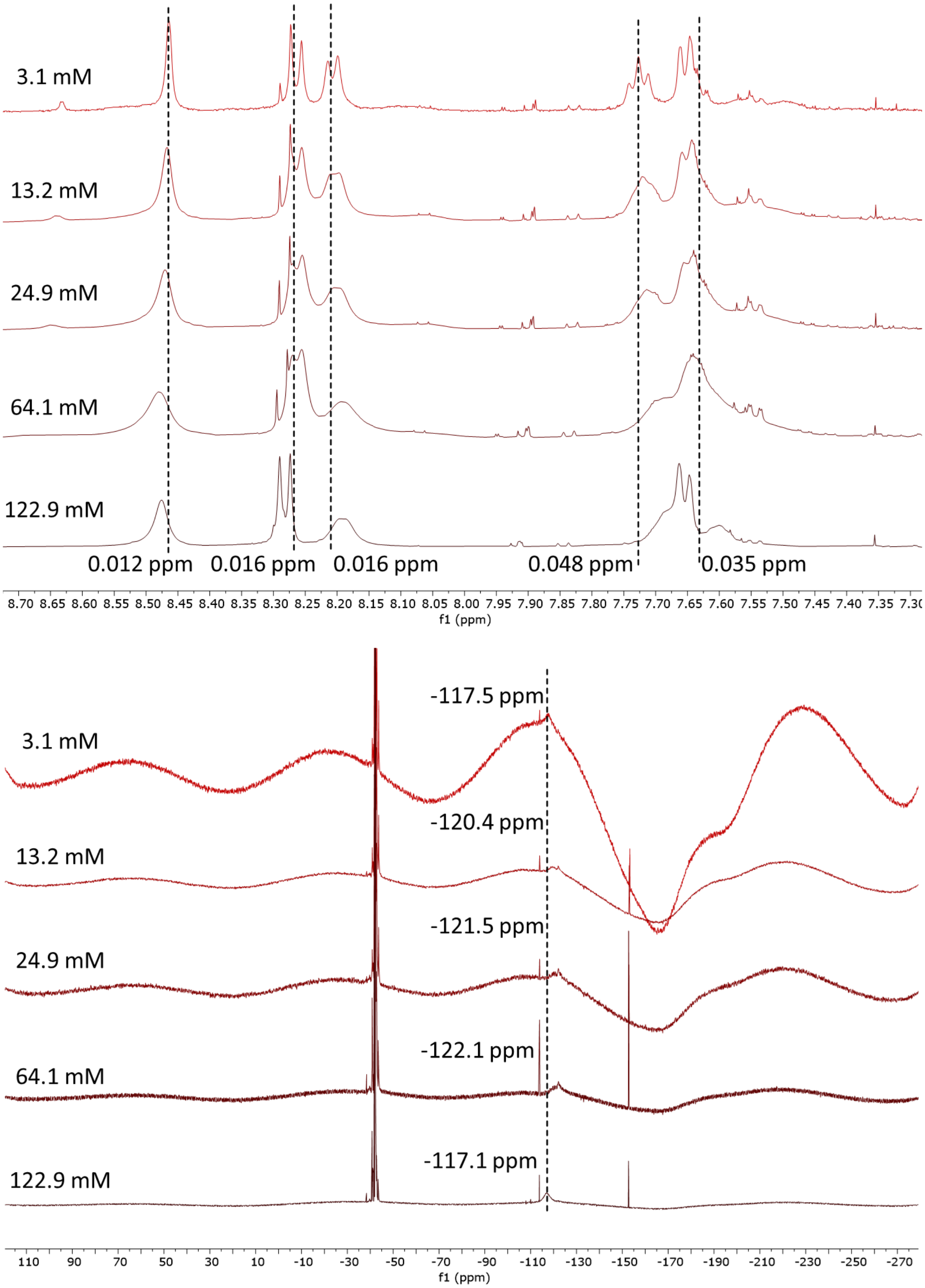

Figure S4. ^{1}H NMR aromatic region (top) and ^{19}F NMR (bottom) of **5** at different concentrations in CD_2Cl_2. Significant peak movement and broadening are observed when the concentration of **5** increases, indicating dimerization in solution at 25 °C.

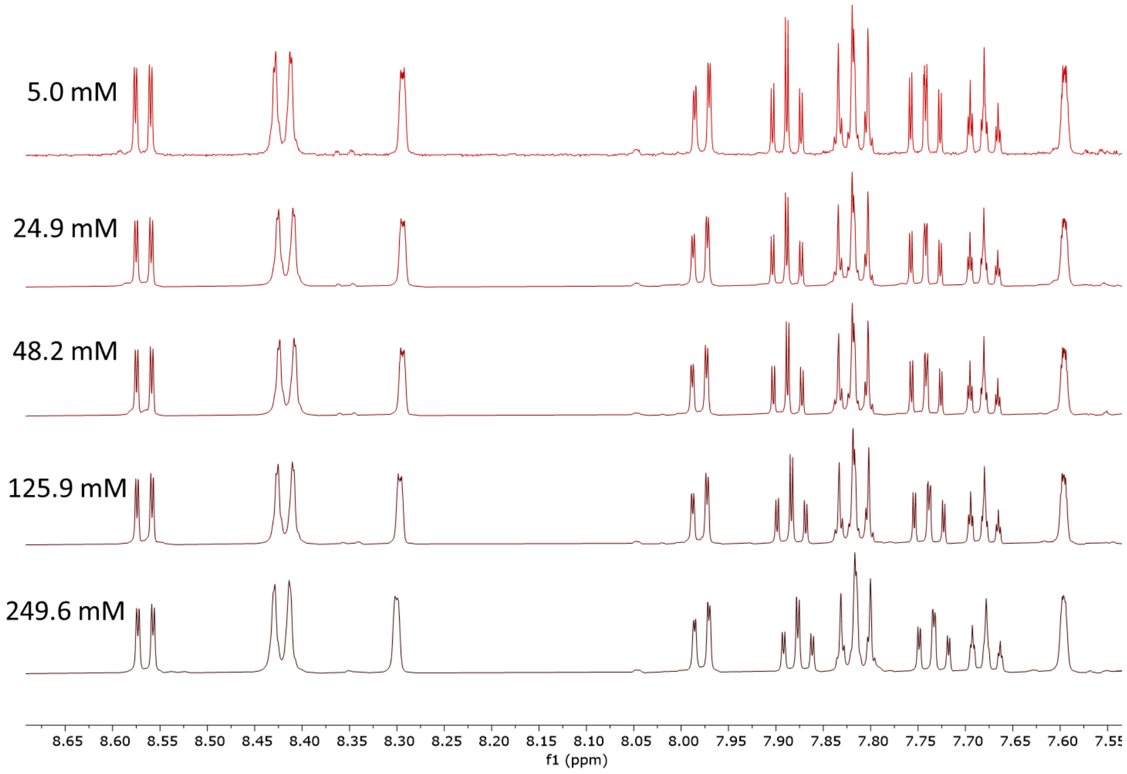
5.0 mM
24.9 mM
48.2 mM
125.9 mM
249.6 mM
8.65 8.60 8.55 8.50 8.45 8.40 8.35 8.30 8.25 8.20 8.15 8.10 8.05 8.00 7.95 7.90 7.85 7.80 7.75 7.70 7.65 7.60
f1 (ppm)

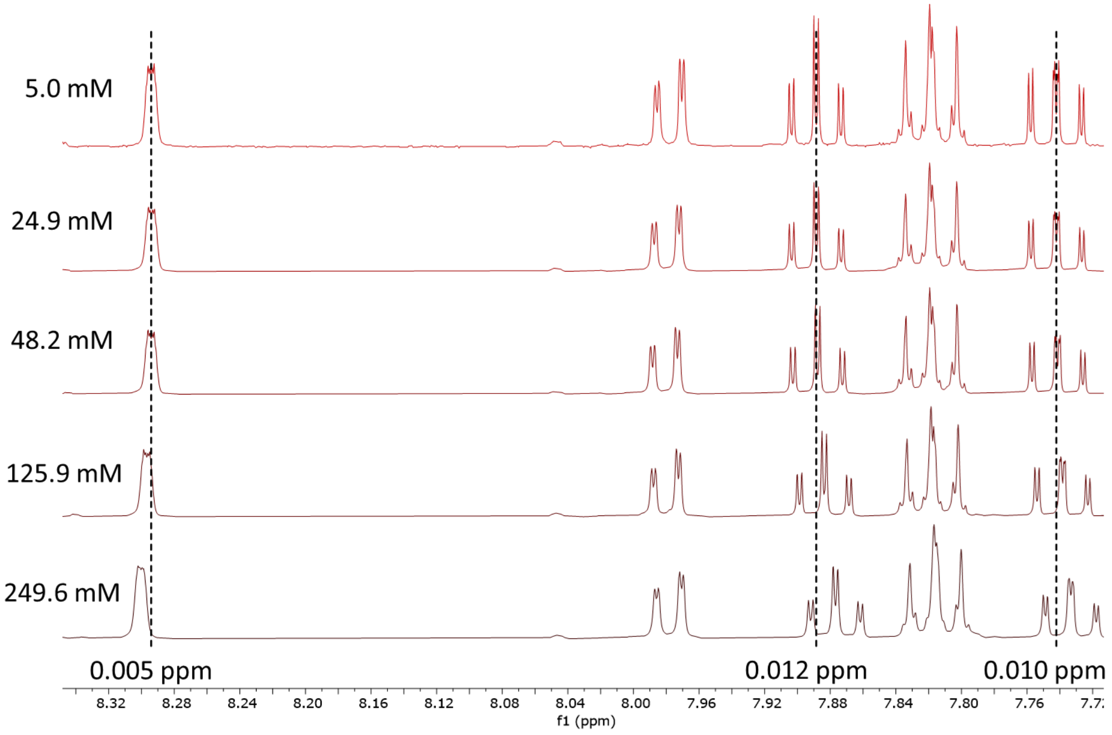
5.0 mM
24.9 mM
48.2 mM
125.9 mM
249.6 mM
0.005 ppm
0.012 ppm
0.010 ppm
8.32 8.28 8.24 8.20 8.16 8.12 8.08 8.04 8.00 7.96 7.92 7.88 7.84 7.80 7.76
f1 (ppm)

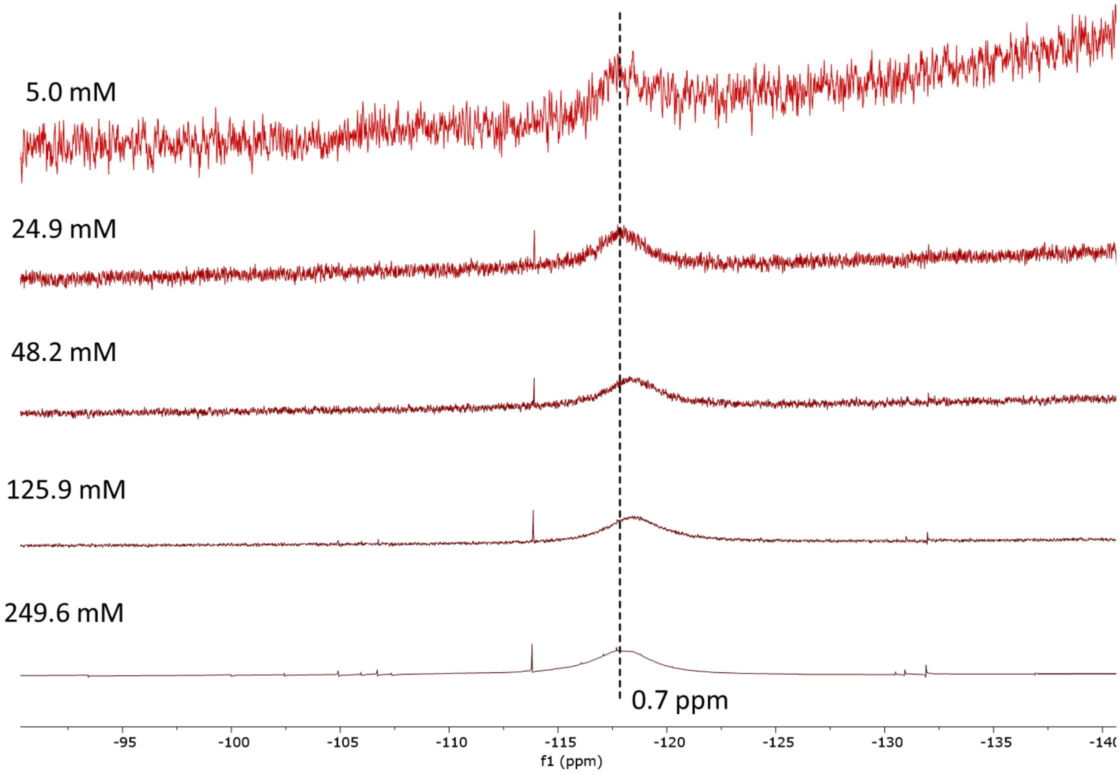

Figure S5. ^{1}H NMR aromatic region (top and middle) and ^{19}F NMR (bottom) of **25** at different concentrations in CD_2Cl_2. Minor peak movement and broadening are observed when the concentration of **25** increases, indicating very small chance of dimerization in solution at 25 °C.

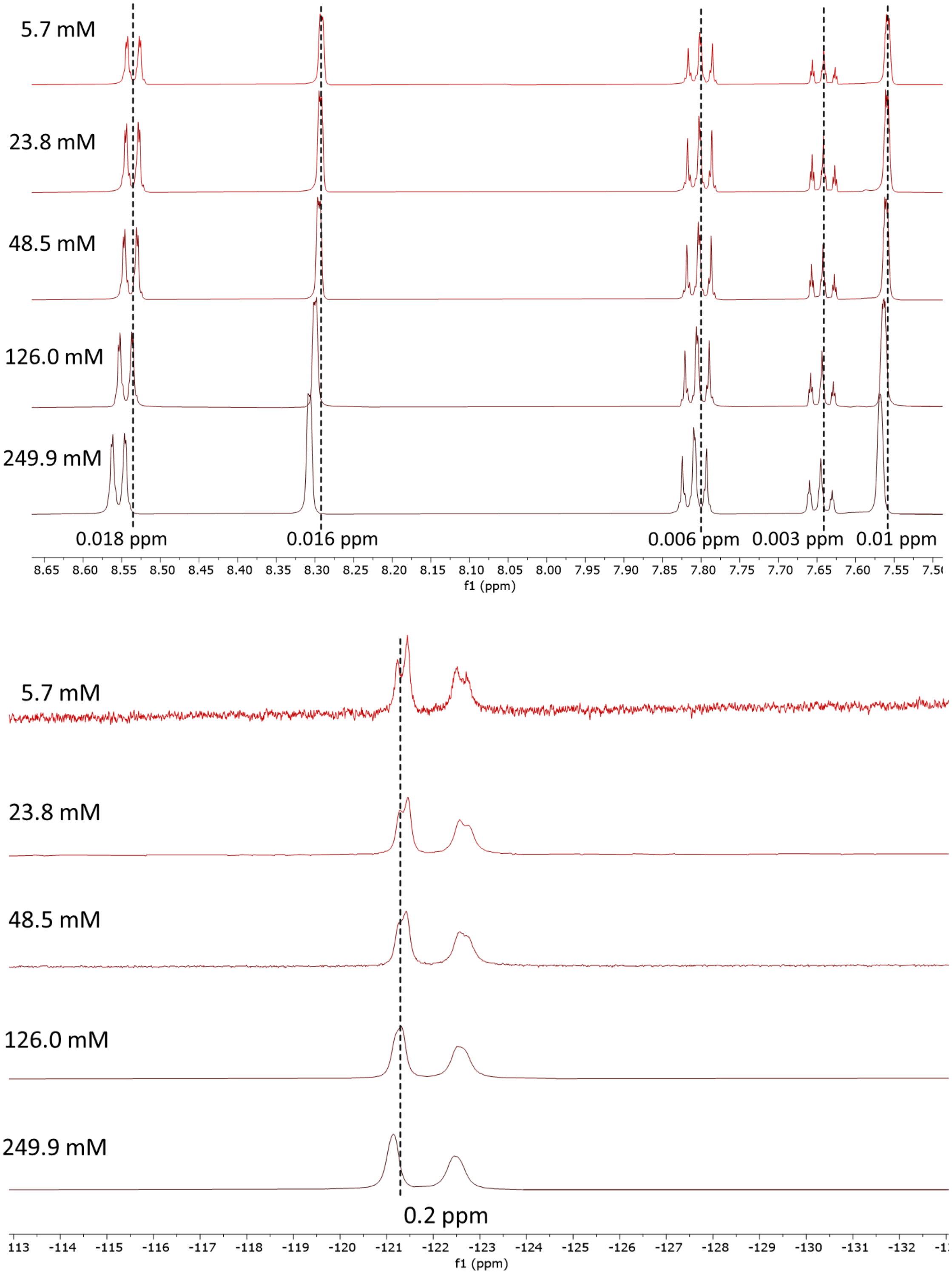

Figure S6. ^{1}H NMR aromatic region (top and middle) and ^{19}F NMR (bottom) of **26** at different concentrations in CD_2Cl_2. Minor peak movement and broadening are observed when the concentration of **26** increases, indicating very small chance of dimerization in solution at 25 °C.

3.2.2 Diffusion-ordered spectroscopy (DOSY) experiments

Diffusion coefficients were obtained from a double stimulated echo sequence with bipolar gradient pulses, convection compensation, longitudinal eddy current delay (LED) and three spoiler gradients (Bruker sequence: dstebpgp3s). The gradient pulse strength G was incremented from 2% to 98% of the maximum G_{max} with a squared gradient ramp in 40 steps. The diffusion time (Δ) used was 50 ms and the length of a gradient pulse gradient pulse ($\delta/2$) of the encoding gradient was 1.0 ms. The maximum gradient strength G_{max} of the NMR probe (PA BBO 500S1 BBF-H-D Z PLUS) was 53.5 $G\cdot cm^{-1}$. Diffusion coefficients were obtained by averaging three diffusion coefficients obtained from fitting the signal decay of three different resonance integrals to the Stejskal-Tanner equation (I) in the Bruker TOPSPIN T1T2 relaxation module.

$$I(G) = I_0 e^{-D(\gamma G \delta)^2(\Delta - \delta/3)} \qquad \text{(I)}$$

The self-diffusion values *D* of **4**, **5**, **25** and **26** were measured at different concentrations. The PFG self-diffusion value, where lower values can be associated with an increased average molecular weight of a compound[15-17] is indeed decreasing stronger for **4**. However, **5** shows similar behavior to monomeric **25** and **26** (see Figure S7). This result suggests that the average molecular weight due to (reversible) dimerization or other kinds of aggregation (i.e. π-stacking) of the compounds increases stronger for **4** than for **5**, **25** and **26**, which is in line with the observed changes in ^1H and ^{19}F NMR shifts.

Table S2. Self-diffusion coefficients obtained from PFG-NMR of **4**, **5**, **25** and **26** obtained at different sample concentrations (assuming purely monomeric forms) in CD_2Cl_2 at 298K.

Compound	[C] (mM)	10^9 D_{exp} (m^2 s^{-1})				SD	D_C/D_{5mM}
		D_1	D_2	D_3	$D_{average}$		
**4**	5.3	1.31	1.32	1.31	1.31	0.00	1.00
	24.7	1.26	1.28	1.28	1.27	0.01	0.97
	74.7	1.21	1.23	1.20	1.21	0.01	0.93
**5**	5.3	1.19	1.17	1.21	1.19	0.02	1.00
	49.8	1.16	1.15	1.16	1.16	0.01	0.97
	249.9	1.02	1.00	1.08	1.03	0.04	0.87
**25**	5.4	1.32	1.30	1.26	1.29	0.03	1.00
	49.7	1.23	1.23	1.21	1.23	0.01	0.95
	250.5	1.06	1.08	1.09	1.08	0.01	0.83
**26**	5.1	1.21	1.28	1.26	1.25	0.03	1.00
	50.0	1.19	1.23	1.21	1.21	0.02	0.97
	249.9	1.07	1.08	1.07	1.08	0.01	0.86

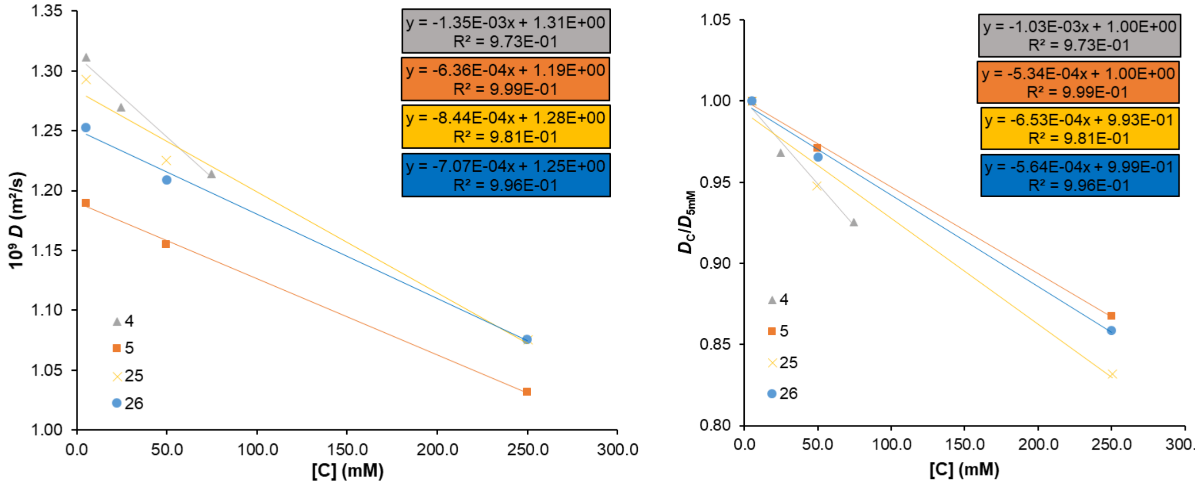

Figure S7. Absolute (left) and relative (right) self-diffusion values *D* of **4** (grey), **5** (orange), **25** (yellow) and **26** (blue) measured at different concentrations at 25 °C.

4. Reactivity studies of neutral σ-aryl Bi(V) fluorides

4.1 Reactivity of model complex **4**

4.1.1 Dependence of concentration for decay of **4**

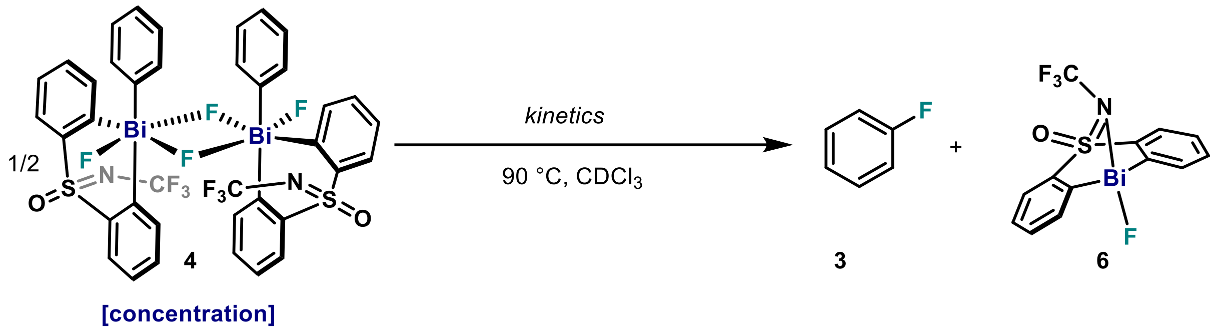

In a J-Young NMR tube, pentavalent bismine **4** (0.0013-0.013 mmol, *dimer*) was mixed with anhydrous $CDCl_3$ (0.5 mL) and 1-fluoro-4-nitrobenzene was added as internal standard (1.0 equiv respect to the monomeric form, 0.025 mmol, 2.65 μL). NMR samples were placed at a preheated NMR machine (90 °C) and the reaction was monitored by ^{1}H and ^{19}F NMR. Kinetic constants were obtained by plotting the Napierian logarithm (ln) of concentrations of the corresponding pentavalent bismine species versus time (at least 20% conversion, initial rates).

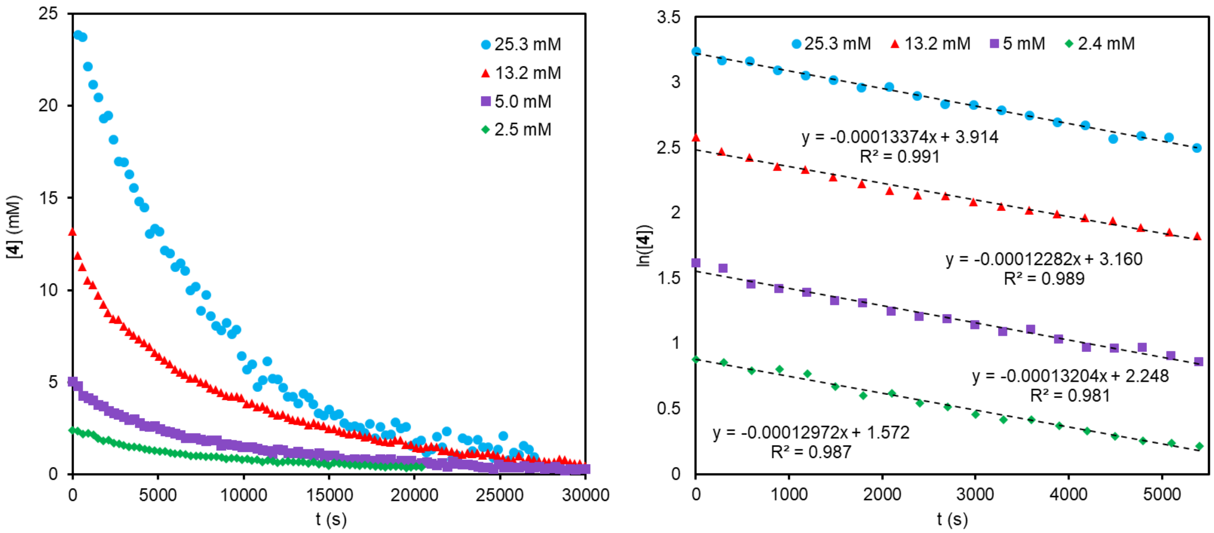

Figure S8. *Left*: Kinetic profile of the thermal decay of **4** in $CDCl_3$ at 90 °C. Concentrations: blue – 25.3 mM; red – 13.2 mM; purple – 5.0 mM; green – 2.4 mM.

[**4**]$_0$ (mM)	k_{obs}
25.3	0.00013374
13.2	0.00012282
5.0	0.00013204
2.4	0.00012972

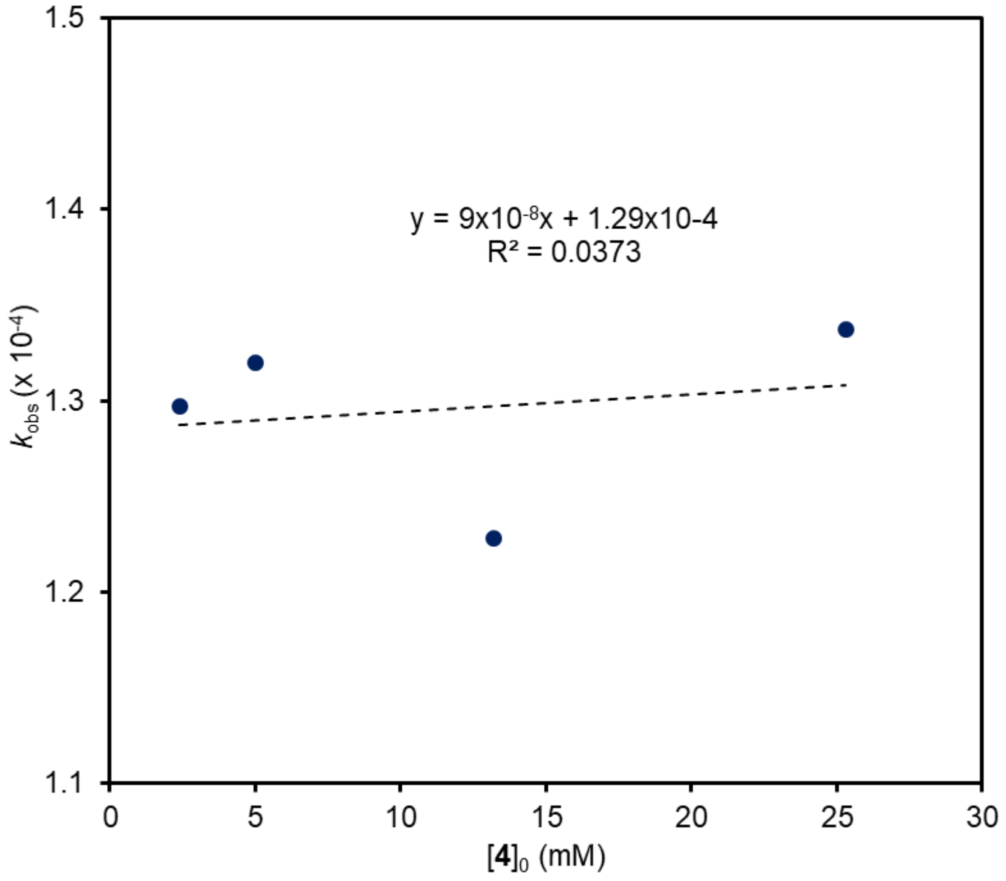

Figure S9. Dependence of k_{obs} vs. initial concentration of **4** (mM).

4.1.2 Model for fluorobenzene formation from species **4**

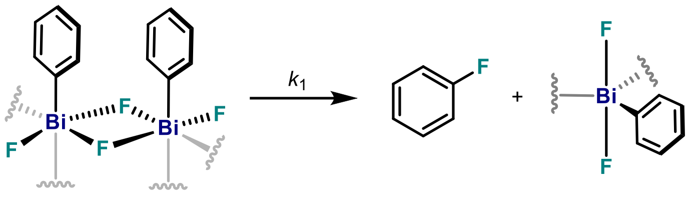

D **P** **M**

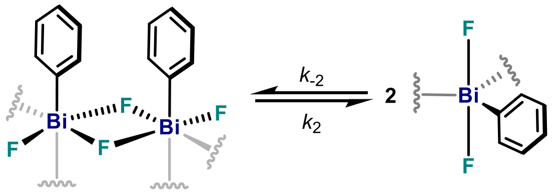

D **M**

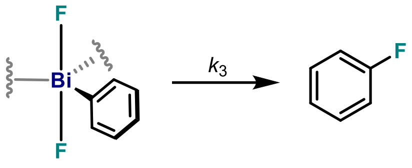

M **P**

The rate law for reductive elimination from **D** via aryl–F bond formation with direct mechanism and from a pentacoordinate complex **M** with pre-equilibrium dissociation from dimeric species is as follows (Bi(III) byproduct species are omitted for clarity):

k_1: rate of aryl–F bond formation from dimeric species **D**

k_2: rate of dimer dissociation to form monomeric species **M**

k_{-2}: rate of monomer association to form dimeric species **D**

k_3: rate of aryl–F bond formation from monomeric species **M**

Reaction rate is given in equation 1

$$\frac{d[P]}{dt} = k_1[D] + k_3[M] \qquad (1)$$

As **D** and **M** are in a fast equilibrium compared to the reductive elimination rates, pre-equilibrium approximation could be applied, yielding equation 2:

$$[D] = \frac{k_{-2}}{k_2}[M]^2$$

$$\frac{d[P]}{dt} = \frac{k_1 k_{-2}}{k_2}[M]^2 + k_3[M] \qquad (2)$$

Experimental kinetic data obtained shows good fitting to the first order kinetics. However, the quadratic polynomial fitting produces worse results, suggesting the actual rate law to be different to those depicted in equations 1 or 2.

In order to decipher the model for the reaction rate law, DFT calculation results (see section 9) were taken into consideration. At 363 K, $\Delta G^{\ddagger}$ for the reductive elimination from **D** was calculated to be 25.5 kcal·mol^{-1} (Figure S126). However, the smallest $\Delta G^{\ddagger}$ for the reductive elimination from lowest energy monomer **M** (called ***int-cis***) was calculated to be higher – 26.5 kcal·mol^{-1} (Figure S137). Using the Eyring–Polanyi equation (as usual, assuming the transmission coefficient to be 1, as the species passing through the transition state proceed directly to products and not back to the reactants) to compare the rate constants for these reductive eliminations, we obtain the following ratio:

$$\frac{k_3}{k_1} = e^{\frac{\Delta G^{\ddagger}{}_1 - \Delta G^{\ddagger}{}_3}{RT}} = e^{\frac{25.511-26.486}{0.72135}} = 0.2589$$

Furthermore, the dimeric species **D** are 1.149 kcal·mol^{-1} more stable than two lowest energy **M** (***int-cis***) molecules. This results in an equilibrium constant value of:

$$\Delta G = -RT ln K_{eq}$$
$$K_{eq} = e^{\frac{-\Delta G}{RT}} = e^{\frac{-1.149}{0.72135}} = 0.2034$$

Having this and knowing that one **D** produces two **M** molecules, we can calculate the ratio between monomer and dimer concentrations at different initial dimer (as **4** exists as a dimer in the solid state) concentrations:

$$K_{eq} = \frac{[M]^2}{[D]}$$
$$[D]_0 = [D] + 0.5[M]$$
$$[D] = [D]_0 - 0.5[M]$$
$$K_{eq} = \frac{[M]^2}{[D]_0 - 0.5[M]}$$
$$[M]^2 + 0.5 * K_{eq}[M] - [D]_0 K_{eq} = 0$$

Table S3. Calculation of monomer/dimer and reductive elimination rate ratios at different initial concentrations.

$[D]_0$ (mM)	[M] (mM)	[D] (mM)	[M]/[D]	$k_3[M]/k_1[D]$, %
25.0	2.205	23.898	0.09227	2.389
20.0	1.967	19.017	0.1034	2.678
15.0	1.697	14.152	0.1199	3.104
12.5	1.545	11.728	0.1317	3.410
10.0	1.376	9.312	0.1478	3.827
5.0	0.959	4.521	0.2121	5.492
2.5	0.664	2.168	0.3063	7.931

In the last column we multiplied the rate constant ratio obtained above with the monomer/dimer ratio to directly compare the two terms in the rate law depicted in the equation 1. As it is shown, having an initial concentration of dimer higher than 5 mM, the k_3[M] term is less than 5% of the k_1[D] term. Therefore, if we approximate the equation 1 to a first order rate law:

$$\frac{d[P]}{dt} = k_1[D] + k_3[M] \approx k_1[D]$$

we introduce a small error that is of the similar magnitude as experimental errors (errors in measuring exact sample weights, measuring the amount of internal standard, integrations of the NMR signals, etc.) in the obtained kinetic data. Such approximation and hence the kinetic model described above should suffice in explaining the observed data, and the interpretation of the first order kinetics observed. Especially, when considering data obtained at concentrations higher than 5 mM, which adjust sufficiently to justify the obtained kinetic parameters and validate the derived interpretations and discussions.

4.1.3 Solvent effects in the reductive elimination from **4**

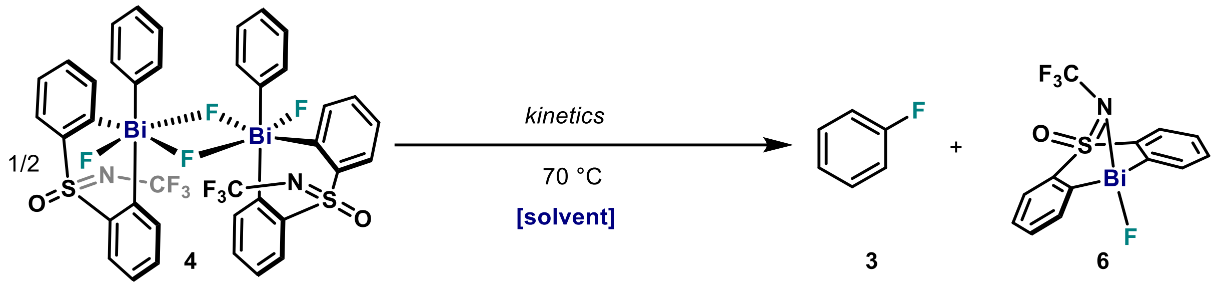

In a J-Young NMR tube, pentavalent bismine **4** (15.2 mg, 0.013 mmol, *dimer*) was mixed with anhydrous *solvent* (0.5 mL) and 1-fluoro-4-nitrobenzene was added as internal standard (1.0 equiv respect to the monomeric form, 0.025 mmol, 2.65 µL). NMR samples were placed at a preheated NMR machine (70 °C) and the reaction was monitored by ^{1}H and ^{19}F NMR. Kinetic constants were obtained by plotting the Napierian logarithm (ln) of concentrations of species **4** versus time (at least 20% conversion, initial rates).

solvent	ε	k_{obs}	k_{rel}
$CDCl_3$	4.81	0.00003087	1
CD_2Cl_2	8.93	0.00003049	0.98769
CD_3CN	37.5	0.00003333	1.07969

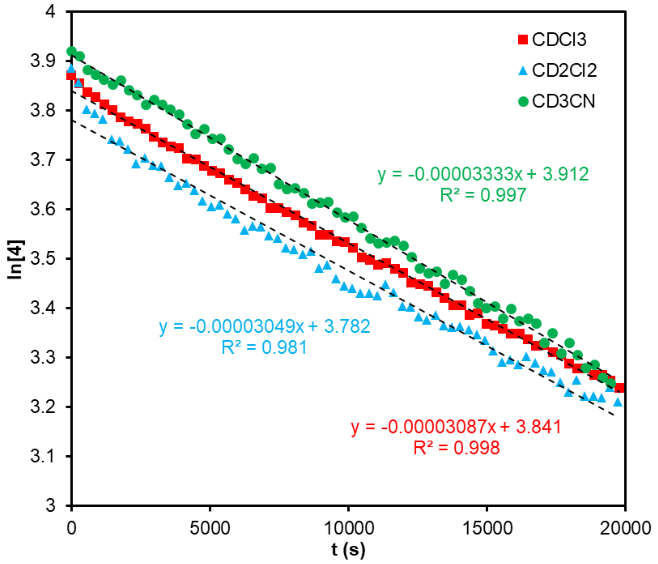

Figure S10. Relationship between rate (k_{obs}) of decay of **4** and solvent (ln([**4**]) based on monomer concentration).

Solvents such as THF, toluene, 1,4-dioxane or DMF were not tolerated and the pentavalent bismine **4** complex decomposed, preventing the measurement of kinetic constants.

4.1.4 Reaction monitoring for decay of **4**

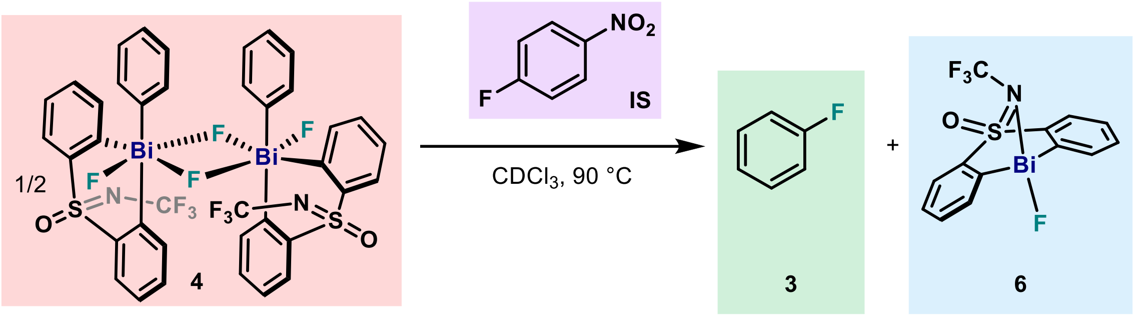

In a J-Young NMR tube, pentavalent bismine **4** (15.2 mg, 0.013 mmol, *dimer*) was mixed with anhydrous $CDCl_3$ (0.5 mL) and 1-fluoro-4-nitrobenzene was added as internal standard (1.0 equiv respect to the monomeric form, 0.025 mmol, 2.65 μL). The mixture was heated to 90 °C inside the NMR probe, and the reaction was monitored by ^1H and ^{19}F NMR over 10 h.

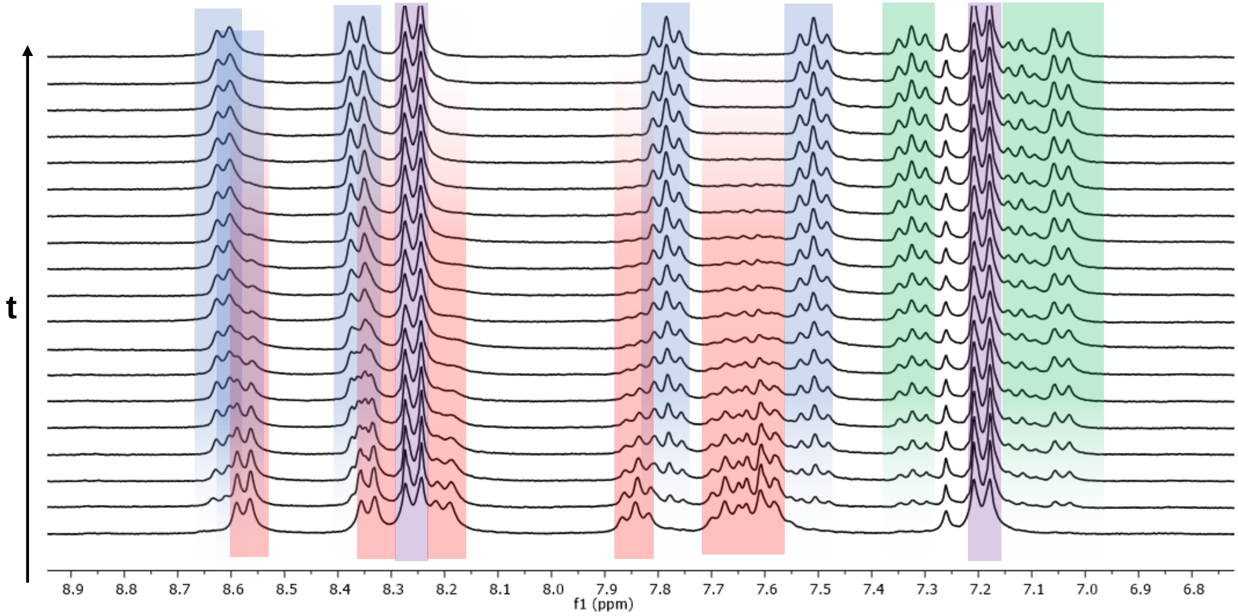

Figure S11. Crude ^1H NMR monitoring (aromatic region) of the thermal decay of species **4** (red) in $CDCl_3$ at 90 °C, furnishing fluorobismine **6** (blue) and fluorobenzene (green) in presence of 1-fluoro-4-nitrobenzene as internal standard (purple).

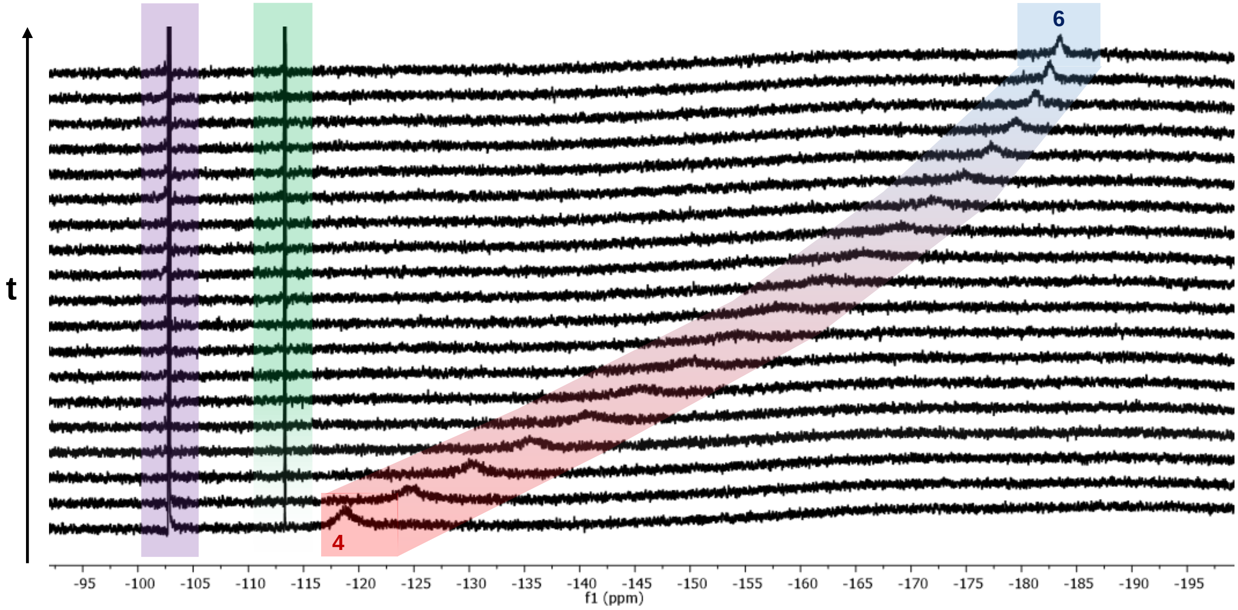

Figure S12. Crude ^{19}F NMR monitoring of the thermal decay of species **4** (red) in $CDCl_3$ at 90 °C, furnishing fluorobismine **6** (blue) and fluorobenzene (green) in presence of 1-fluoro-4-nitrobenzene as internal standard (purple).

4.1.5 Reaction analysis after partial decomposition of **4**

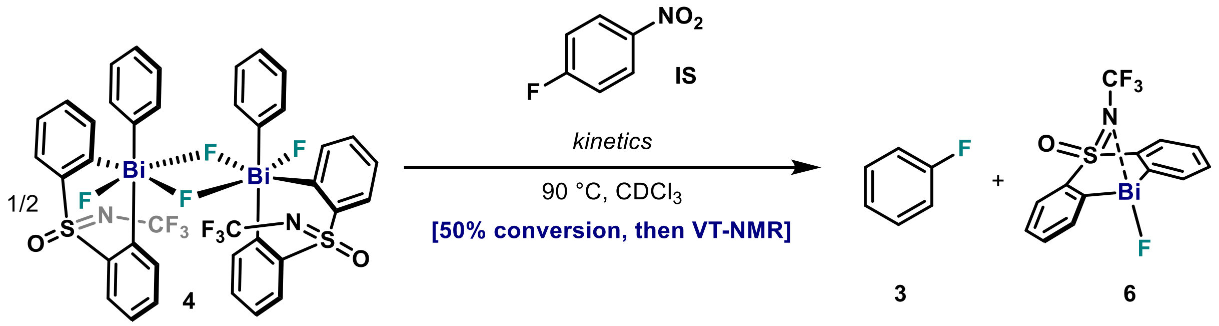

In a J-Young NMR tube, pentavalent bismine **4** (15.2 mg, 0.013 mmol, *dimer*) was mixed with anhydrous $CDCl_3$ (0.5 mL) and 1-fluoro-4-nitrobenzene was added as internal standard (1.0 equiv respect to the monomeric form, 0.025 mmol, 2.65 μL). The mixture was heated to 90 °C inside the NMR probe, and the reaction was monitored by ^1H and ^{19}F NMR until it reached 50% conversion. After 50% conversion was reached, the sample was analyzed by VT-NMR (See Figures S13 and S14).

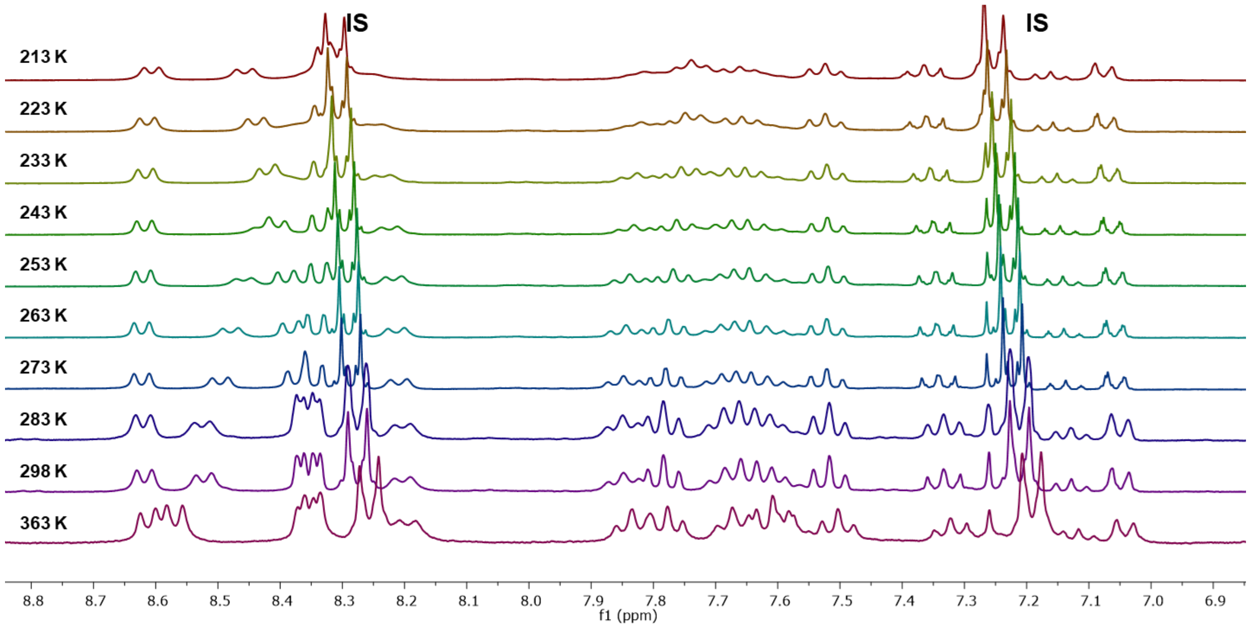

Figure S13. Decomposition of species **4** at 90 °C until 50% conversion is reached, obtaining **3** and **6** in approximately 50% yield. Then, VT ^1H NMR analysis of the crude reaction was done.

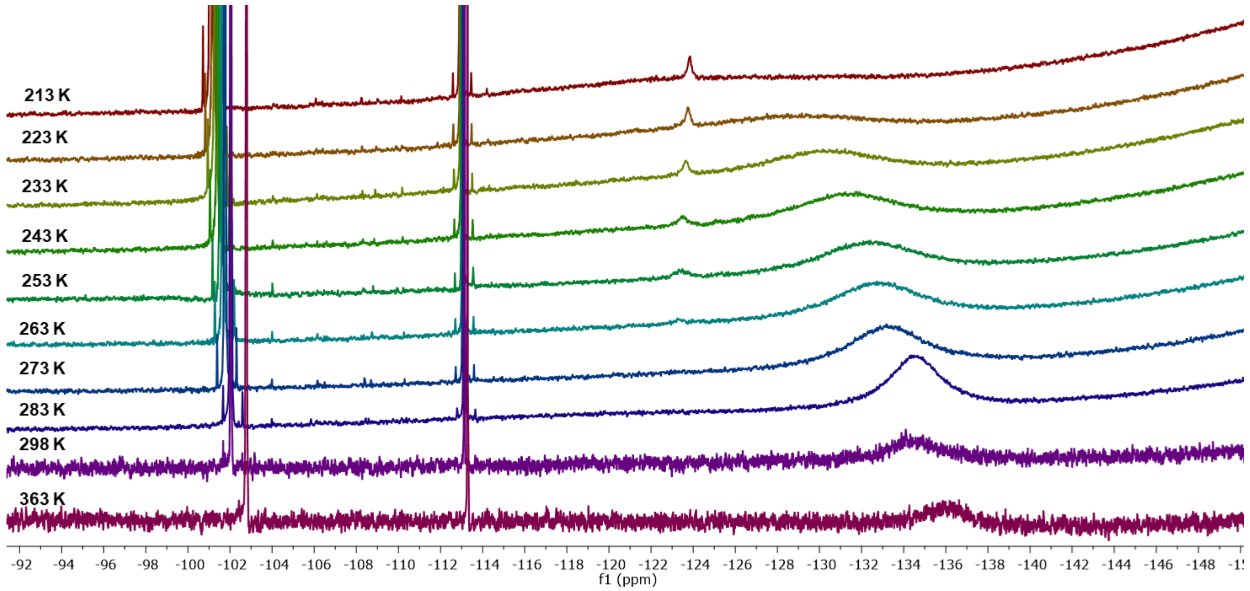

Figure S14. Decomposition of species **4** at 90 °C until 50% conversion is reached, obtaining **3** and **6** in approximately 50% yield. Then, VT ^{19}F NMR analysis of the crude reaction was done.

4.1.6 Reaction of **4** with trivalent fluorobismine **6**

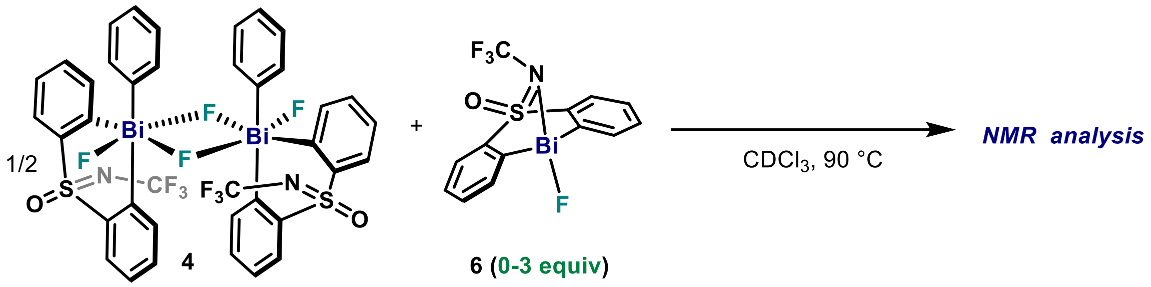

In a standard NMR tube, pentavalent bismine **4** (15.2 mg, 0.013 mmol, *dimer*) was mixed with fluorobismine **6** (0-3.0 equiv, respect to the monomeric form). Then, anhydrous $CDCl_3$ (0.5 mL) was added. The mixture was vigorously shaken and analyzed by ^1H and ^{19}F NMR. Results obtained are shown in Figures S15 and S16 (in parenthesis ratio of species **4**:**6**).

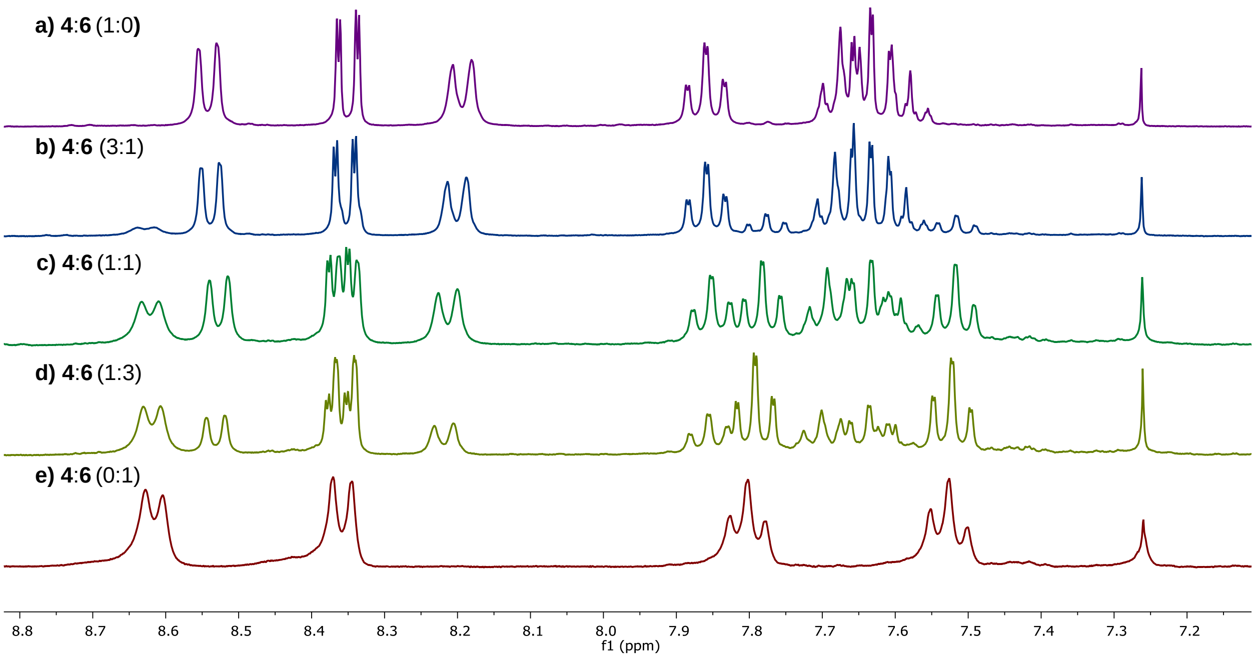

Figure S15. ^{1}H NMR measurements (aromatic region) of mixtures **4**:**6** in different ratios ($CDCl_3$, 298 K).

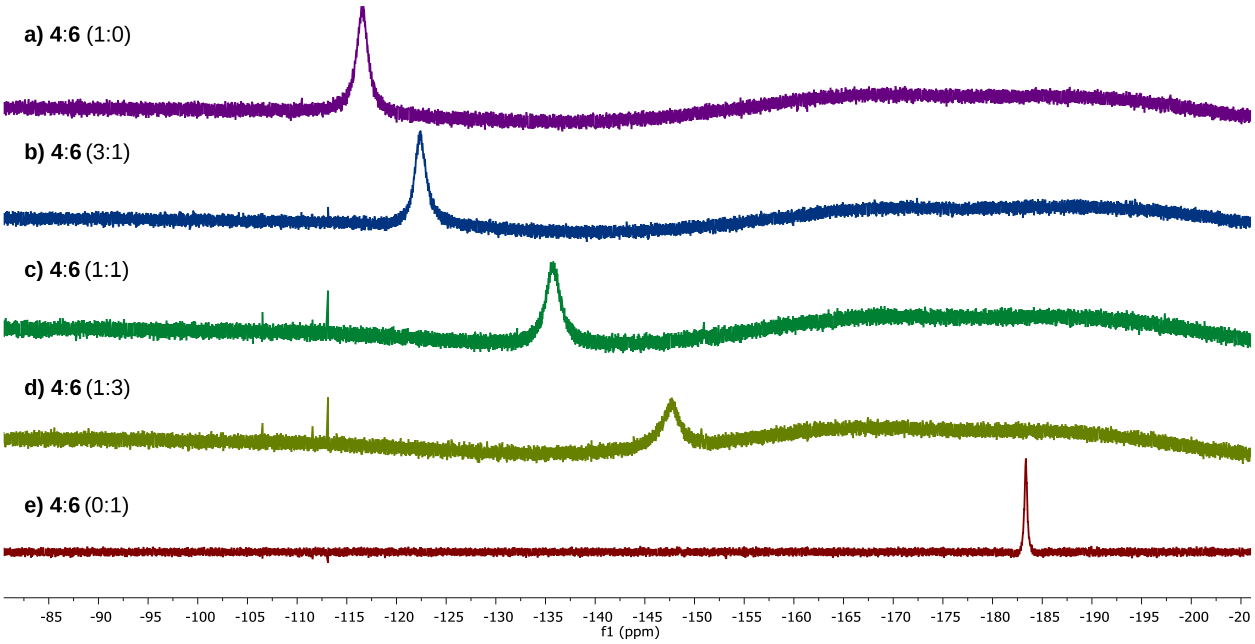

Figure S16. ^{19}F NMR measurements of mixtures **4**:**6** in different ratios ($CDCl_3$, 298 K).

4.1.7 Reaction profile of **4** decay in presence of **6**

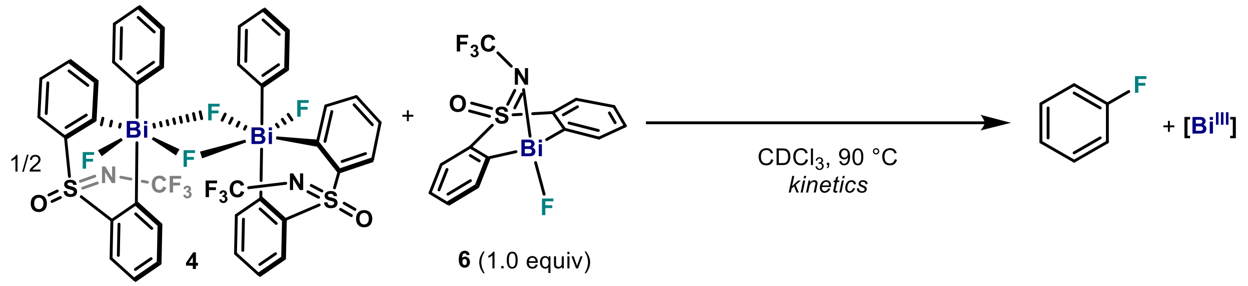

In a J-Young NMR tube, pentavalent bismine **4** (15.2 mg, 0.013 mmol, *dimer*) was mixed with fluorobismine **6** (1.0 equiv respect the monomeric form, 0.025 mmol, 12.8 mg), anhydrous $CDCl_3$ (0.5 mL) and 1-fluoro-4-nitrobenzene was added as internal standard (1.0 equiv respect the monomeric form, 0.025 mmol, 2.65 μL). The NMR sample was placed at a preheated NMR machine (90 °C) and the reaction was monitored by ^{1}H and ^{19}F NMR. Kinetic constants were obtained by plotting the Napierian logarithm (ln) of concentrations of the corresponding pentavalent bismine species versus time (at least 20% conversion, initial rates).

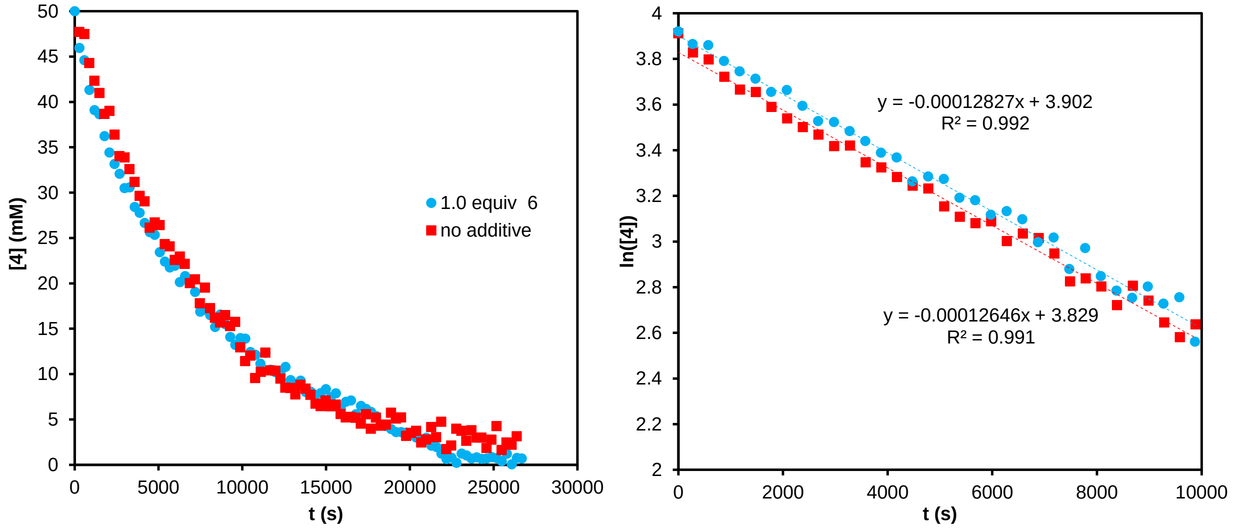

Figure S17. *Left*: Kinetic profile of the thermal decay of **4** in $CDCl_3$ at 90 °C (50 mM) in presence of **6** (blue, [**6**] = 51.1 mM, 1.03 equiv) or in absence of it (red). *Right:* Determination of k_{obs} for the thermal decay of **4** (ln([**4**]) vs t (s)) for the reactions in presence (blue) and in absence (red) of **6**.

4.2 Hammett analysis from Bi(V) complexes with *p*-substituted arenes

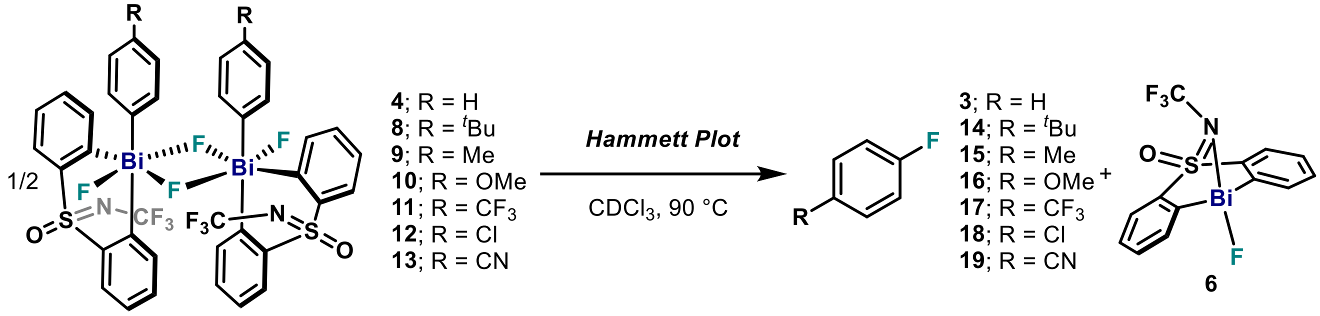

In a J-Young NMR tube, pentavalent bismine **4**, **8-13** (0.013 mmol, *dimer*) was mixed with anhydrous $CDCl_3$ (0.5 mL) and 1-fluoro-4-nitrobenzene was added as internal standard (1.0 equiv respect to monomeric form, 0.025 mmol, 2.65 μL). The mixture was heated to 90 °C inside the NMR probe, and the reaction was monitored by ^{1}H and ^{19}F NMR over 10 h. Kinetic constants were obtained by plotting the Napierian logarithm (ln) of concentrations of the corresponding pentavalent bismine species versus time (at least 20% conversion).

Table S4. Data corresponding to the Hammett plot for the thermal decomposition of **4**, **8-13**.

p-X (bismine)	k_X	k_X/k_H	$\log(k_X/k_H)$	σ_p	σ_p^+
CN (**13**)	0.0002755	2.1476	0.331956	0.7	0.66
CF_3 (**11**)	0.0002043	1.5924	0.202042	0.53	0.61
Cl (**12**)	0.0001458	1.1363	0.055501	0.24	0.11
H (**4**)	0.0001283	1	0	0	0
Me (**9**)	0.0001067	0.8316	–0.08006	–0.17	–0.31
^{t}Bu (**8**)	0.0001067	0.8316	–0.08006	–0.2	–0.26
OMe (**10**)	0.0000561	0.4365	–0.36004	–0.27	–0.78

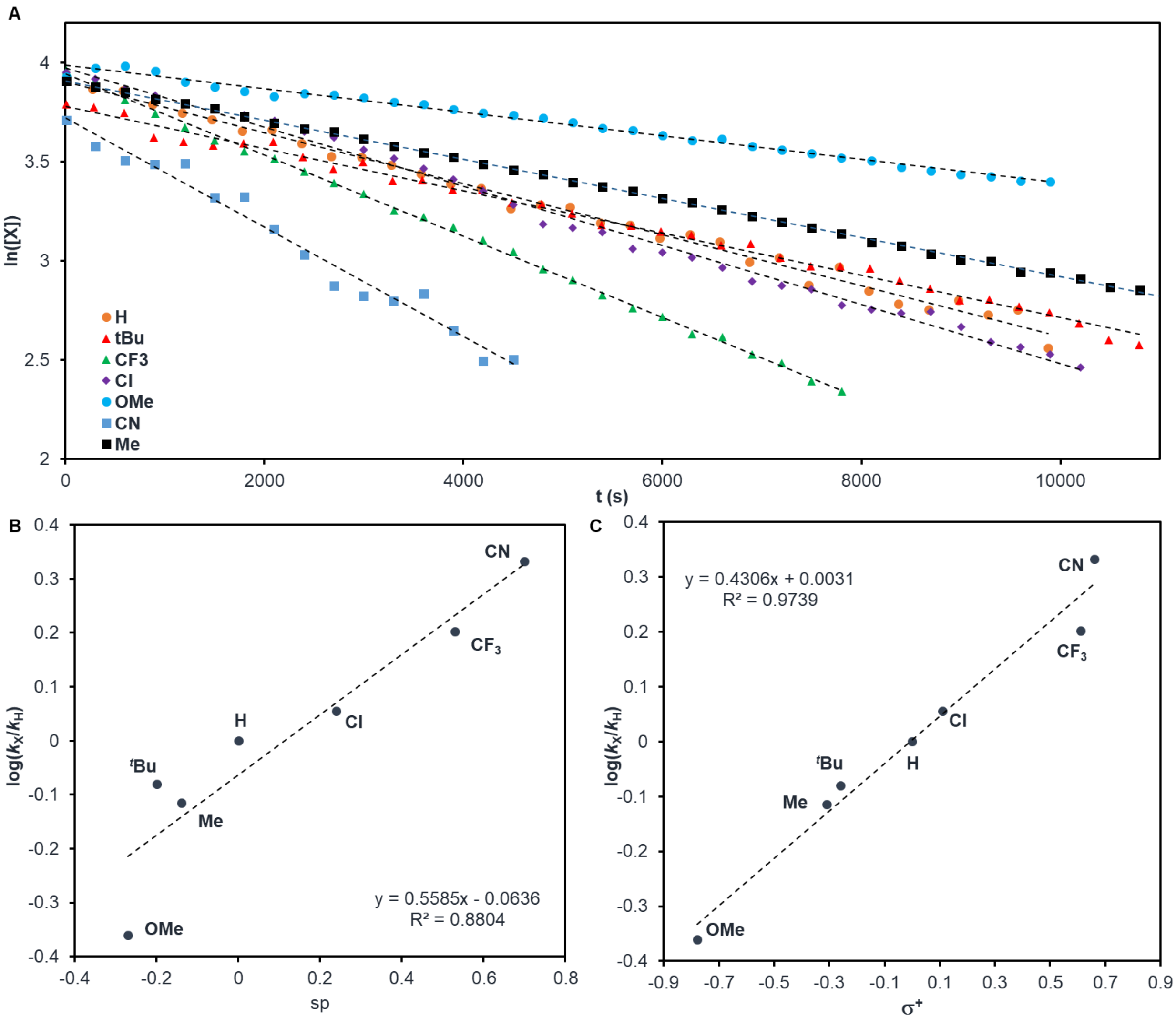

Figure S18. A. Determination of k_{obs} for the thermal decay of *para*-substituted arylbismines (**4**, **8-13**) at 90 °C in $CDCl_3$ (50 mM solution). **B.** Hammett plot for **4**, **8-13** (ρ = 0.56, R^2 = 0.88). **C.** Hammett plot for **4**, **8-13** considering resonance effects (ρ = 0.43, R^2 = 0.97).

4.3 Hammett analysis from Bi(V) complexes with *m*-substituted arenes

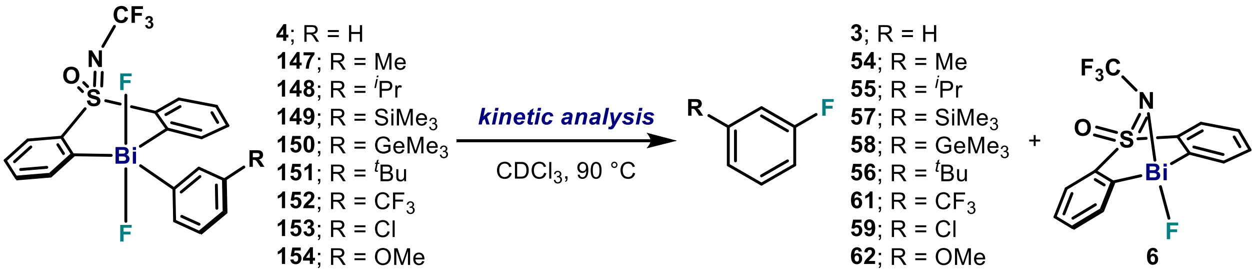

Note: Species **147-154** are depicted as monomers, however their behavior in solution is expected to be similar to complex 4, which is a dimeric species in solid state and has a complex behavior in solution.

In a J-Young NMR tube, pentavalent bismine **4**, **147-154** (0.025 mmol, *monomer*) was mixed with anhydrous $CDCl_3$ (0.5 mL) and 1-fluoro-4-nitrobenzene was added as internal standard (1.0 equiv, 0.025 mmol, 2.65 μL). The mixture was heated to 90 °C inside the NMR probe, and the reaction was monitored by ^{1}H and ^{19}F NMR over 10 h. Kinetic constants were obtained by plotting the Napierian logarithm (ln) of concentrations of the corresponding pentavalent bismine species versus time (at least 20% conversion). As shown in Figure S19A, no clear trend was observed albeit reasonable linearity was observed when using the equation developed by Taft (Figure S19C).

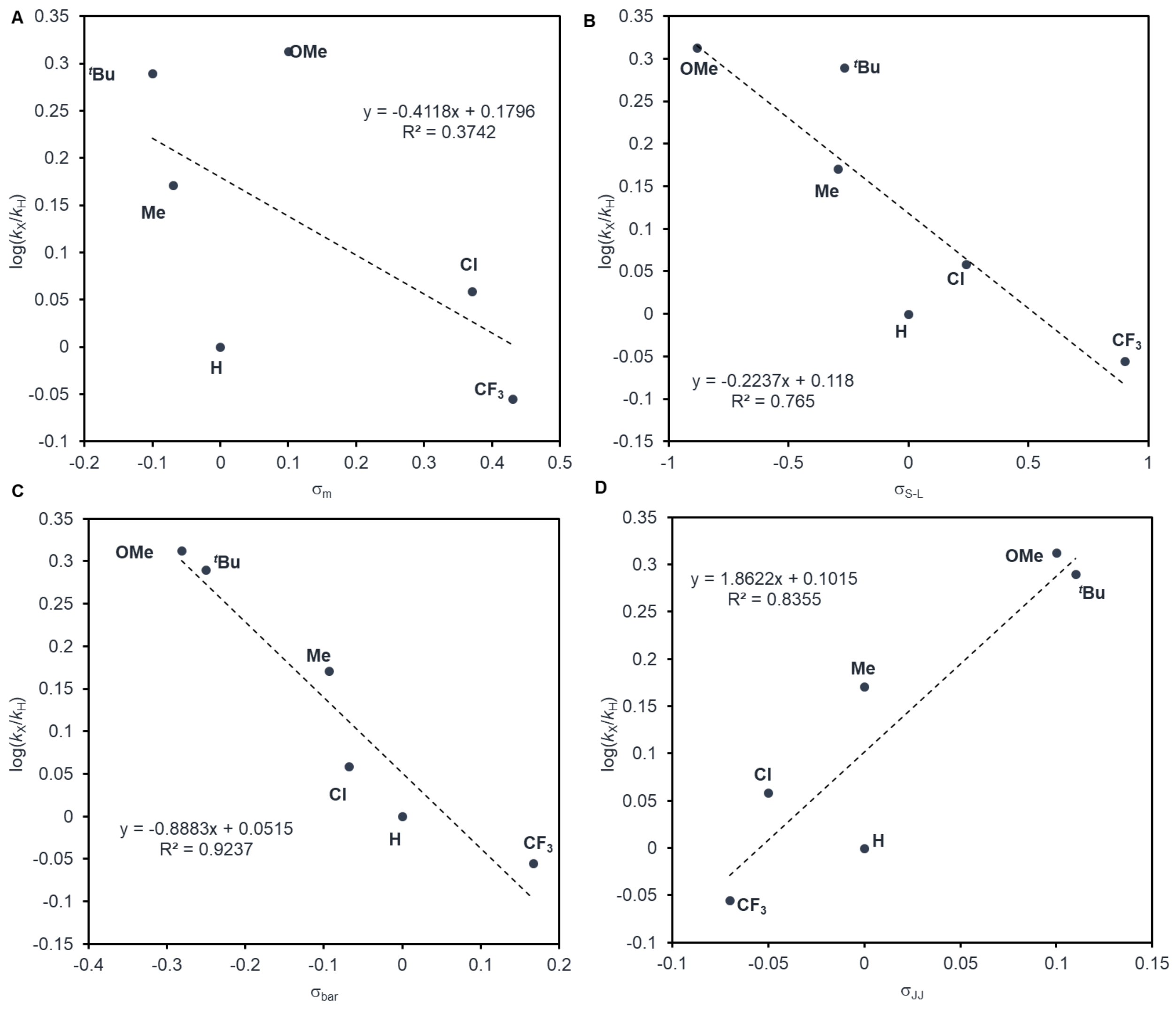

Figure S19. A. Hammett plot for **4**, **147**, **151-154** (ρ = –0.41, R^2 = 0.37).vs σ_m. **B.** Hammett plot for **4**, **147**, **151-154** (ρ = –0.22, R^2 = 0.77) considering resonance effects with the equation developed by Swain-Lupton.[18-19] **C.** Hammett plot for **4**, **147**, **151-154** (ρ = –0.88, R^2 = 0.92) considering resonance effects with the equation developed by Taft.[20-22] **D.** Hammett plot for **4**, **147**, **151-154** (ρ = 1.8, R^2 = 0.84) considering possible radical intermediates with the equation developed by Jiang.[23]

Comparison of electronic effects is further complicated by the rate enhancement observed when bulky substituents are place in *meta*-position. As shown in Table S5, increasing the volume of the substituent in *meta*-position accelerates the rate of Bi(V) decay and subsequently, the rate of formation of fluorobenzene. This is more obvious when moving down the group ($GeMe_3$ (**150**) >>> $SiMe_3$ (**149**) >>> CMe_3, (**151**) > H (**4**)) and when a second substituent is placed in *meta*-position (**174**).

Table S5. Kinetic data corresponding to the thermal decomposition of *meta*-substituted arylbismines comparing bulky substituents.

m-X (bismine)	k_X	k_X/k_H
H (**4**)	0.0001283	1
Me (**147**)	0.0001901	1.48
^{i}Pr (**148**)	0.0001994	1.55
^{t}Bu (**151**)	0.0002501	1.95
^{t}Bu$_2$ (**174**)	0.0004911	3.83
$SiMe_3$ (**149**)	0.0058501	45.6
$GeMe_3$ (**150**)	*instantaneous*	>100

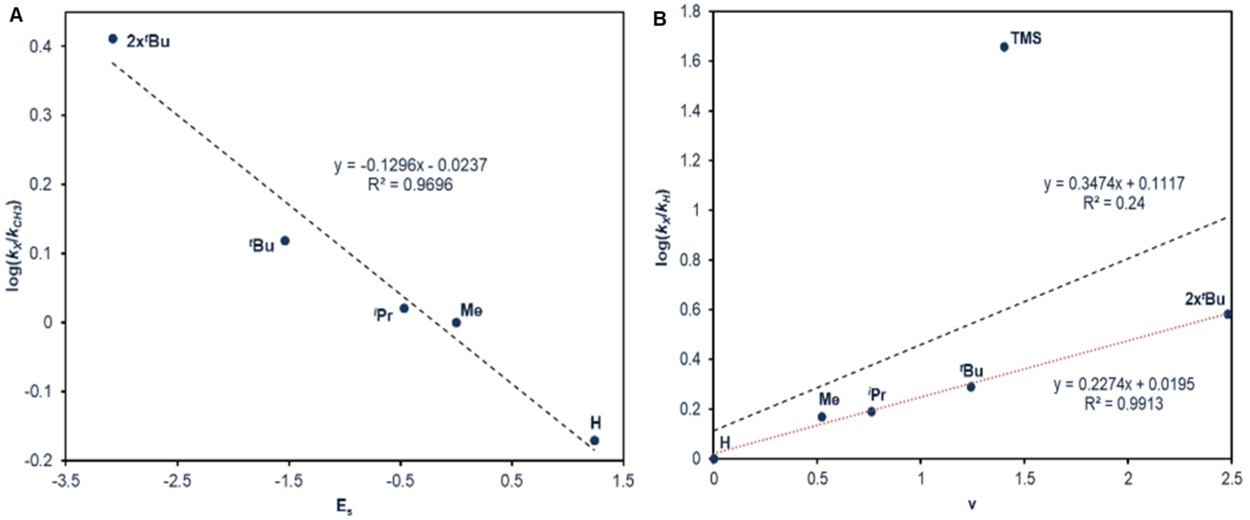

Figure S20. A. Hammett plot for **4**, **147**, **148**, **151**, **174** (ρ = –0.13, R^2 = 0.97).vs Taft steric parameter E_s. **B.** Hammett plot for **4**, **147-149**, **151**, **174** (ρ = 0.35, R^2 = 0.24, blue) and for **4**, **147**, **148**, **151**, **174** (ρ = 0.22, R^2 = 0.99, red) vs Charton values ν.

4.4 Hammett analysis from Bi(V) complexes with *o*-substituted arenes

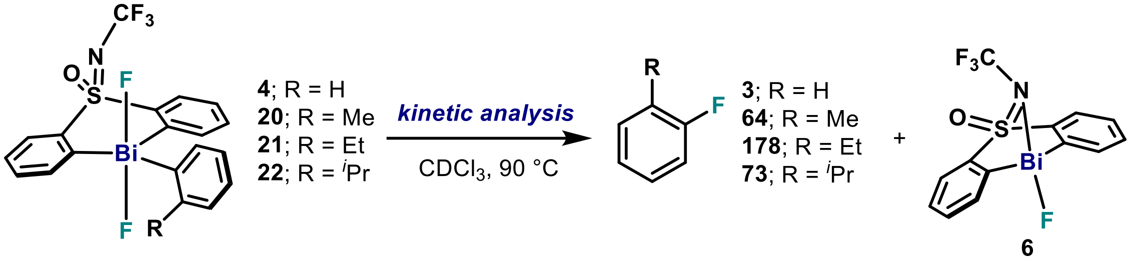

Note: Species **20-22** are depicted as monomers, however their behavior in solution is expected to be similar to complex **4**. In this case, however, steric pressure around the Bi center is higher, which plausibly makes the monomeric form more stable.

In a J-Young NMR tube, pentavalent bismine **4**, **20-22** (0.025 mmol, *monomer*) was mixed with anhydrous $CDCl_3$ (0.5 mL) and 1-fluoro-4-nitrobenzene was added as internal standard (1.0 equiv, 0.025 mmol, 2.65 μL). The mixture was heated to 90 °C inside the NMR probe, and the reaction was monitored by ^{1}H and ^{19}F NMR over 10 h. Kinetic constants were obtained by plotting the Napierian logarithm (ln) of concentrations of the corresponding pentavalent bismine species versus time (at least 20% conversion).

Table S6. Data corresponding to the thermal decomposition of *ortho*-substituted arylbismines **4**, **20-22**.

p-X (bismine)	k_X	k_X/k_H
H (**4**)	0.0001283	1
Me (**20**)	0.0029223	22.8
Et (**21**)	*instantaneous*	>100
^{i}Pr (**22**)	*instantaneous*	>100

Table S7. Data corresponding to the thermal decomposition of *ortho*-, *para*- and *meta*-methyl substituted arylbismines substituted arylbismines **9**, **20** and **147**.

x-Me (bismine)	k_X	k_X/k_H
para (**9**)	0.0000989	1
meta (**147**)	0.0001908	1.9
ortho (**20**)	0.0029223	29.6

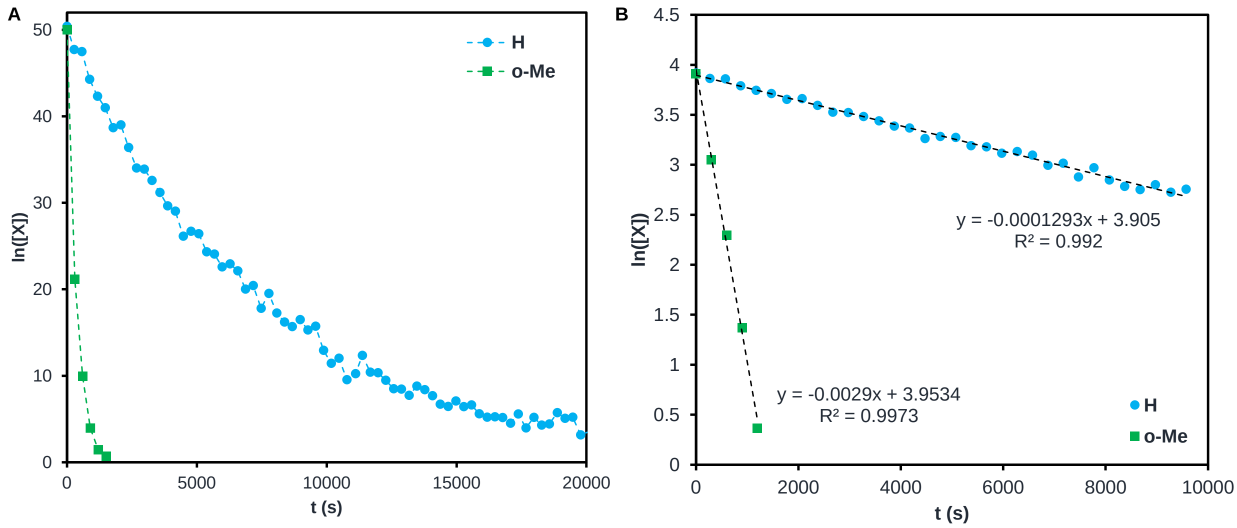

Figure S21. A. Reaction trace the thermal decay of *ortho*-substituted arylbismines (**4** and **20**) at 90 °C in $CDCl_3$ (50 mM solution). **B.** Determination of k_{obs} for the thermal decay of *ortho*-substituted arylbismines (**4** and **20**) at 90 °C in $CDCl_3$ (50 mM solution).

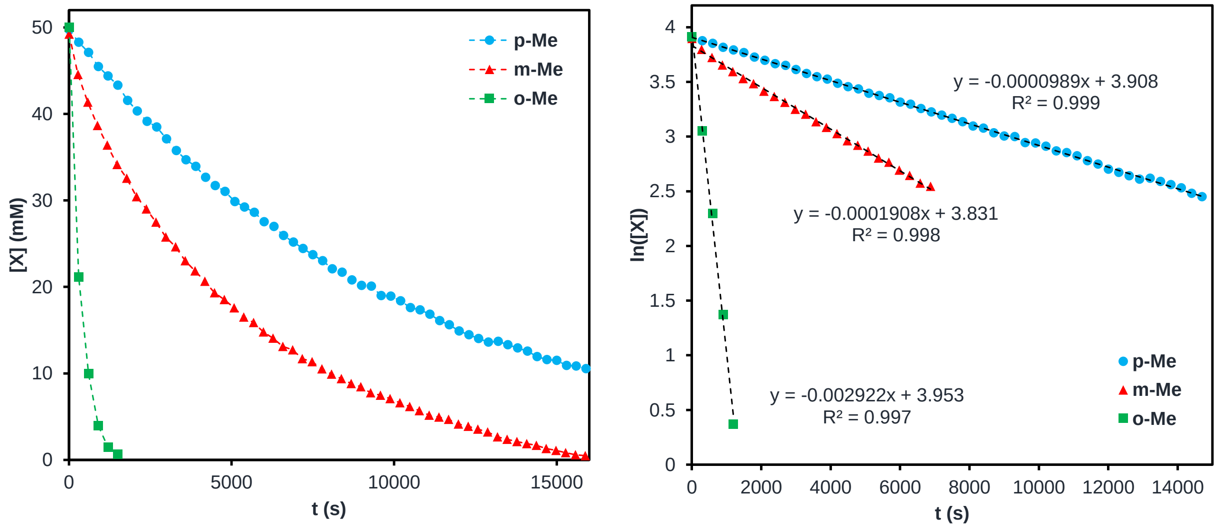

Figure S22 A. Reaction trace the thermal decay of *ortho*-, *para*- and *meta*-methyl substituted arylbismines (**9**, **20** and **147**) at 90 °C in $CDCl_3$ (50 mM solution). **B.** Determination of k_{obs} for the thermal decay of *ortho*-, *para*- and *meta*-methyl substituted arylbismines (**9**, **20** and **147**) at 90 °C in $CDCl_3$ (50 mM solution).

4.5 Hammett analysis from Bi(V) complexes with substituted backbone

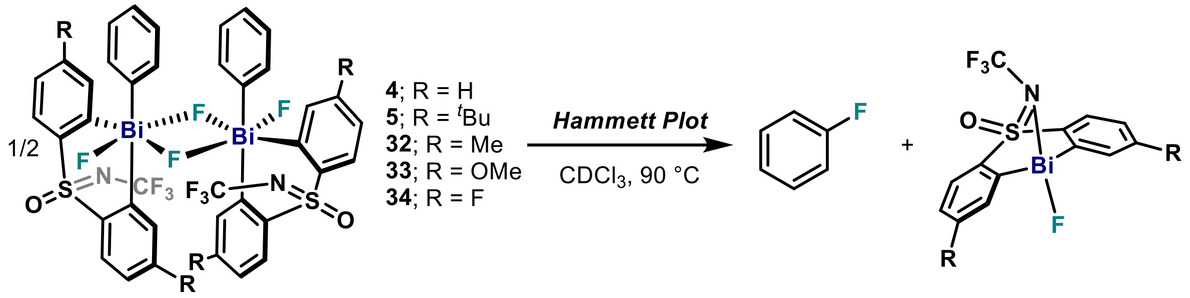

Note: Species **5, 32-34** are depicted as dimers, however their behavior in solution is expected to be similar to complex **4**.

In a J-Young NMR tube, pentavalent bismine **4**, **5**, **32-34** (0.013 mmol, *dimer*) was mixed with anhydrous $CDCl_3$ (0.5 mL) and 1-fluoro-4-nitrobenzene was added as internal standard (1.0 equiv respect to the monomeric form, 0.025 mmol, 2.65 μL). The mixture was heated to 90 °C inside the NMR probe, and the reaction was monitored by ^{1}H and ^{19}F NMR over 10 h. Kinetic constants were obtained by plotting the Napierian logarithm (ln) of concentrations of the corresponding pentavalent bismine species versus time (at least 20% conversion).

Table S8. Data corresponding to the Hammett plot for the thermal decomposition of **4**, **5**, **32-34** vs σ_m.

m-X (bismine)	k_X	k_X/k_H	$\log(k_X/k_H)$	σ_m
F (**34**)	0.00048177	3.7259	0.5712	0.34
H (**4**)	0.0001283	1	0	0
Me (**32**)	0.00009942	0.7689	–0.1141	–0.06
^{t}Bu (**5**)	0.00008667	0.6703	–0.1737	–0.10
OMe (**33**)	0.00009602	0.7426	–0.1292	0.1

Table S9. Data corresponding to the Hammett plot for the thermal decomposition of **4**, **5**, **32-34** considering resonance effects with the equation developed by Taft with $\lambda = 0.82$, $\rho_I = 1.92$ and $\rho_R = 1.58$.[20-22]

p-X (bismine)	k_X	k_X/k_H	$\log(k_X/k_H)$	σ_I	σ_R^0	σ_{bar}
F (**34**)	0.00048177	3.7259	0.5712	0.51	–0.34	0.126
H (**4**)	0.0001283	1	0	0	0	0
Me (**32**)	0.00009942	0.7689	–0.1141	–0.05	–0.10	–0.073
^{t}Bu (**5**)	0.00008667	0.6703	–0.1737	–0.06	–0.12	–0.087
OMe (**33**)	0.00009602	0.7426	–0.1292	0.26	–0.41	–0.042

Table S10. Data corresponding to the Hammett plot for the thermal decomposition of **4**, **5**, **32-34** considering resonance effects with the equation developed by Swain and Lupton with $f = 0.71$ and $r = 0.29$.[18-19]

p-X (bismine)	k_X	k_X/k_H	$\log(k_X/k_H)$	F	R	$\sigma_{S\text{-}L}$
F (**34**)	0.00048177	3.7259	0.5712	0.74	–0.60	0.3516
H (**4**)	0.0001283	1	0	0	0	0
Me (**32**)	0.00009942	0.7689	–0.1141	–0.01	–0.41	–0.126
^{t}Bu (**5**)	0.00008667	0.6703	–0.1737	–0.11	–0.29	–0.162
OMe (**33**)	0.00009602	0.7426	–0.1292	0.54	–1.68	–0.104

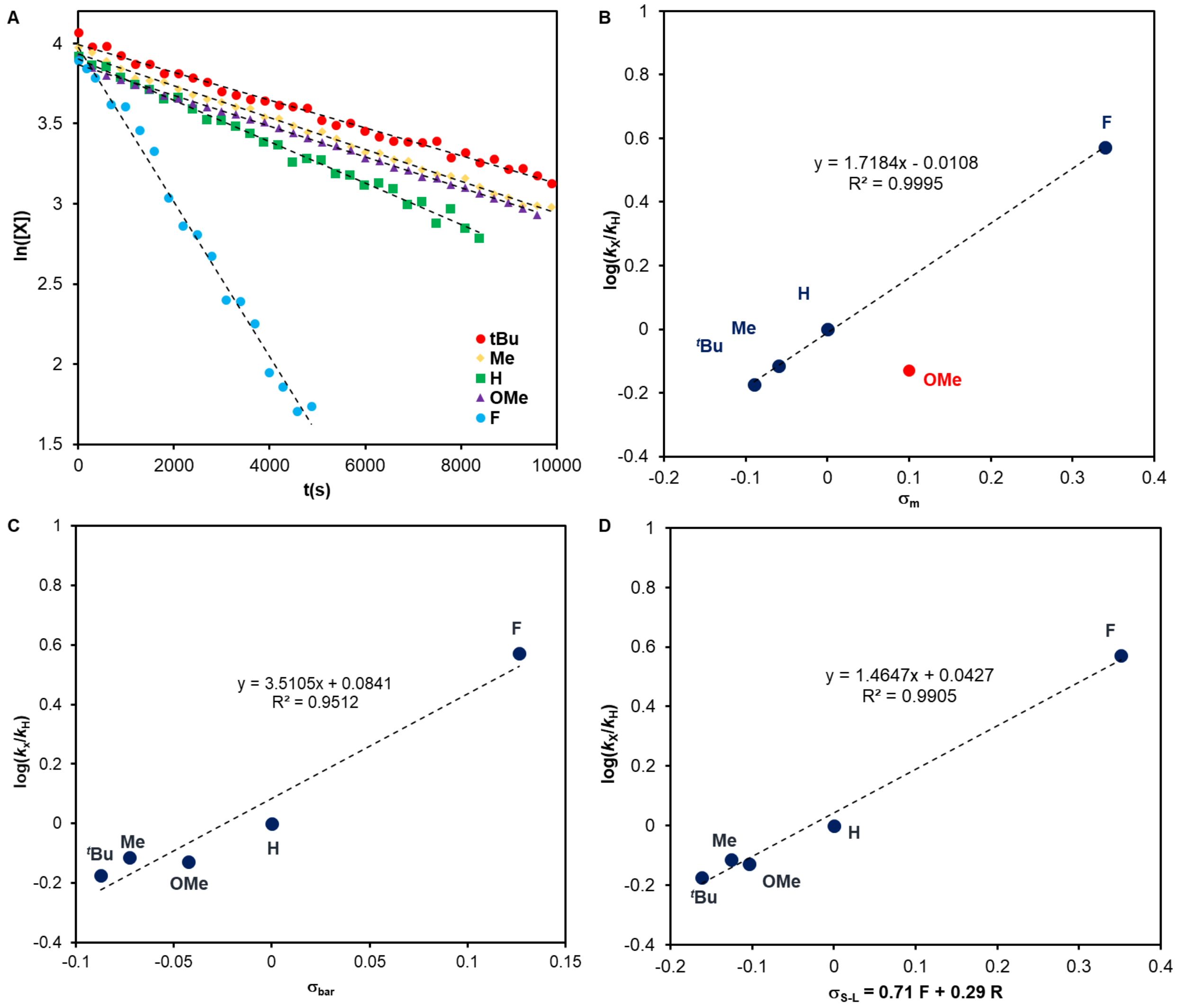

Figure S23. A. Determination of k_{obs} for the thermal decay of *para*-substituted arylbismines (**4**, **5**, **32-34**) at 90 °C in $CDCl_3$ (50 mM solution). **B.** Hammett plot for **4**, **5**, **32**, **34** (ρ = 1.72, R^2 = 0.99) *vs* σ_m, excluding pentavalent bismine **33** (X = OMe, red). **C.** Hammett plot for **4**, **5**, **32-34** (ρ = 3.51, R^2 = 0.95) considering resonance effects with the equation developed by Taft.[18-19] **D.** Hammett plot for **4**, **5**, **32-34** (ρ = 1.47, R^2 = 0.99) considering resonance effects with the equation developed by Swain-Lupton.[20-22]

4.6 Influence of ligand substitution

4.6.1 Effect of aryl backbone substitution on the reaction yield

Note: Bi(V) species shown in Table S11 are depicted as monomers to simplify the picture, however, their behavior in solution is hypothesized to be similar to species **4**.

In a scintillation vial, the corresponding pentavalent difluorophenylbismine (0.025 mmol, *monomer*) was mixed with $CDCl_3$ (1.0 mL) under an Ar atmosphere. The vial was sealed and the reaction was stirred for 16 h at 90 °C. Then, 1-fluoro-4-nitrobenzene was added as internal standard (0.025 mmol, addition by weight) and the crude reactions were analyzed by ^{19}F NMR to determine the yield of fluorobenzene.

Table S11. Influence of the ligand substitution in the reductive elimination from pentavalent difluorophenylbismine species. In parenthesis, reaction performed over 24 h at 110 °C.

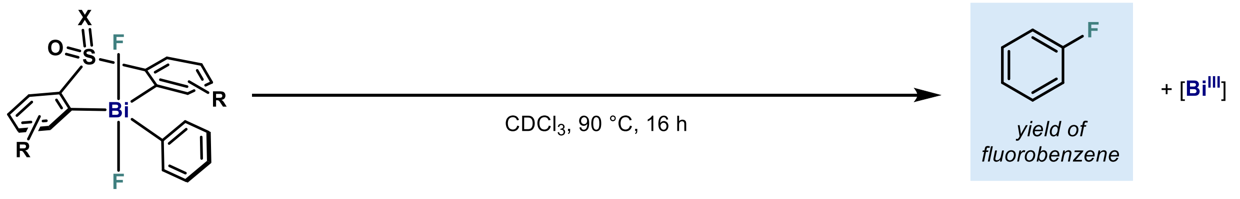

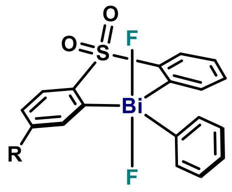

155; R = H, 22%
156; R = Me, 45%
157; R = OMe, 42%

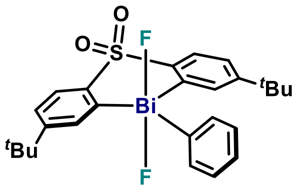

158
59%

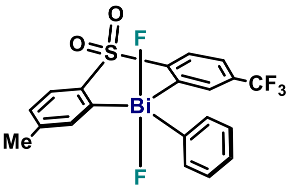

159
80%

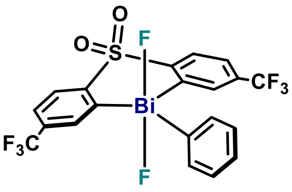

160
81%

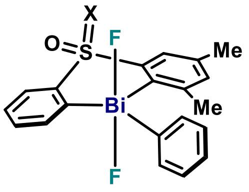

161; X = O, 58%
25; X = NCF_3, 94%

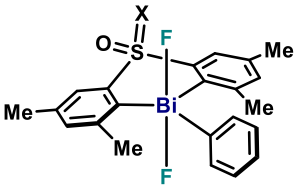

162; X = O, 70% (89%)
26; X = NCF_3, 91% (93%)

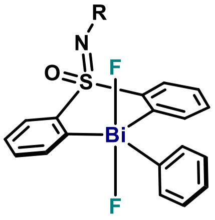

4; R = CF_3, 94%
163; R = Me, 28%
164; R = CF_2CF_3, >95%

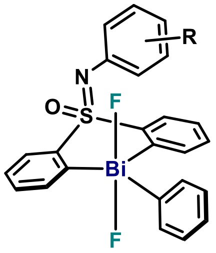

165; R = *4*-OMe, 49%
166; R = *4*-^{t}Bu, 41%
167; R = *4*-H, 47%
168; R = *4*-CF_3, 67%
169; R = *3,5-CF$_3$*, 82%
170; R = *4*-SF_5, 84%

4.6.2 Effect of sulfoximine substitution on the reaction rate

Note: Species **164**, **169** and **170** are depicted as monomers to simplify the picture, however, their behavior in solution is hypothesized to be similar to species **4**.

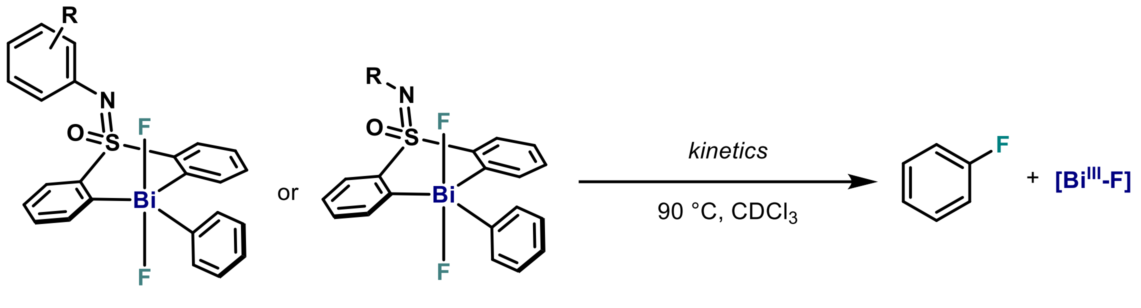

169; R = *3,5*-CF_3
170; R = *4*-SF_5

4; R = CF_3
164; R = CF_2CF_3

In a J-Young NMR tube, pentavalent bismine **4**, **164**, **169-170** (0.025 mmol, *monomer*) was mixed with anhydrous $CDCl_3$ (0.5 mL) and 1-fluoro-4-nitrobenzene was added as internal standard (1.0 equiv, 0.025 mmol, 2.65 µL). NMR samples were placed at a preheated NMR machine (90 °C) and the reaction was monitored by ^1H and ^{19}F NMR. Kinetic constants were obtained by plotting the Napierian logarithm (ln) of concentrations of the corresponding pentavalent bismine species versus time (at least 20% conversion, initial rates).

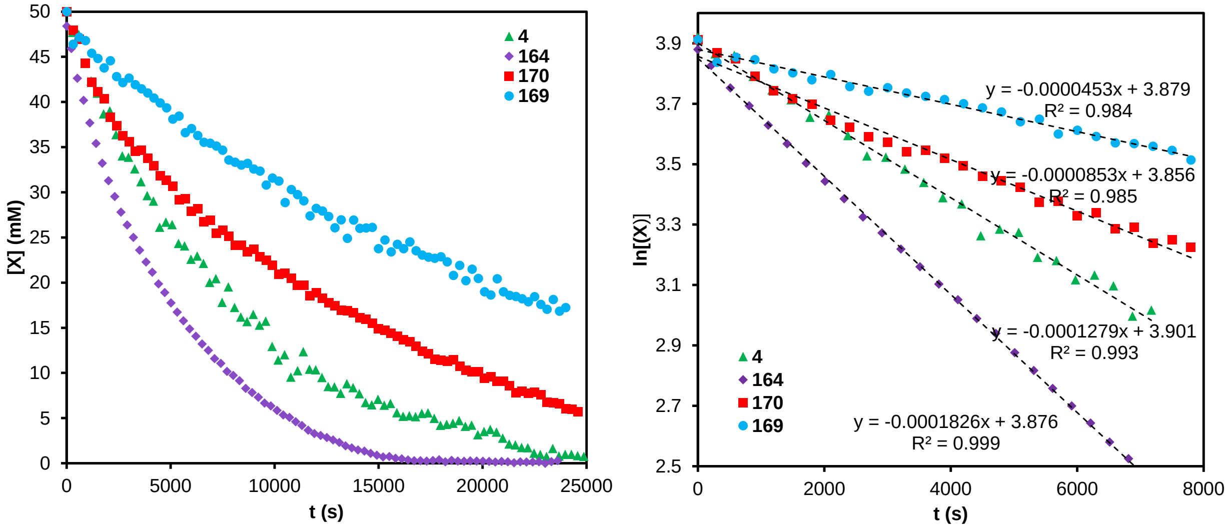

Figure S24. *Left*: Kinetic profile of the thermal decay of pentavalent bismine difluorides **4**, **164**, **169-170** in $CDCl_3$ at 90 °C (50 mM). *Right*: Determination of k_{obs} for the thermal decay of pentavalent bismine difluorides **4**, **164**, **169-170** (ln([**4**, **164**, **169-170**]) vs t (s)).

4.6.3 Sulfone versus N-trifluoromethyl sulfoximine

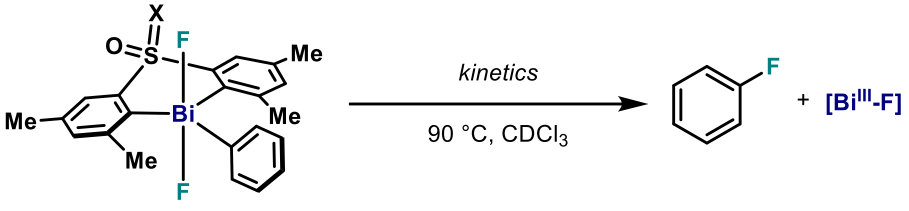

162; X = O
26; X = NCF_3

In a J-Young NMR tube, pentavalent bismine **26** or **162** (0.025 mmol) was mixed with anhydrous $CDCl_3$ (0.5 mL) and 1-fluoro-4-nitrobenzene was added as internal standard (1.0 equiv, 0.025 mmol, 2.65 μL). NMR samples were placed at a preheated NMR machine (90 °C) and the reaction was monitored by ^{1}H and ^{19}F NMR. Kinetic constants were obtained by plotting the Napierian logarithm (ln) of concentrations of the corresponding pentavalent bismine species versus time (at least 20% conversion, initial rates).

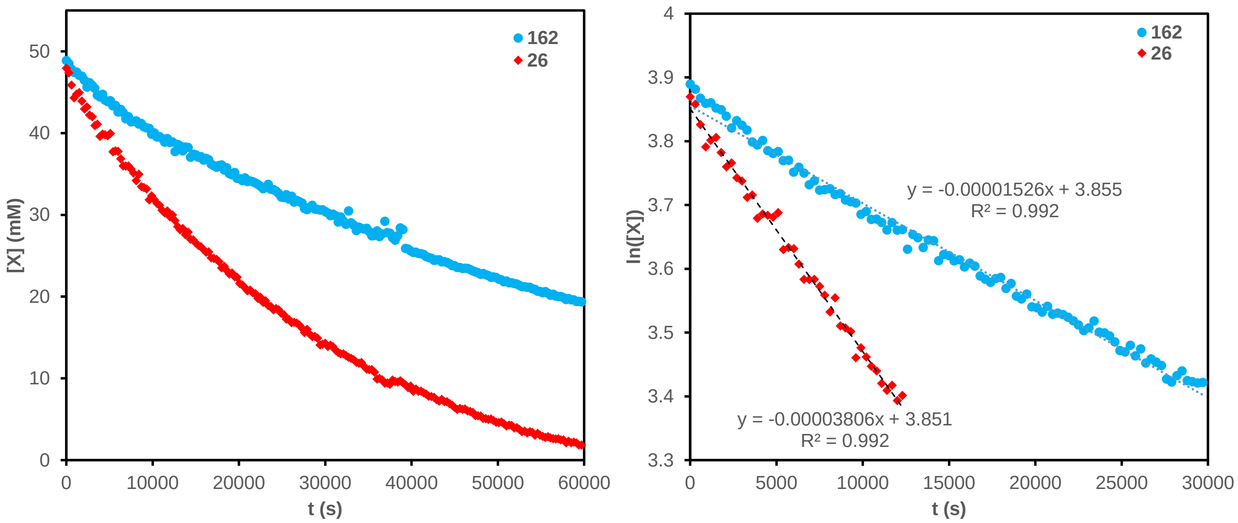

Figure S25. *Left*: Kinetic profile of the thermal decay of pentavalent bismine difluorides **26** and **162** in $CDCl_3$ at 90 °C (50 mM). *Right*: Determination of k_{obs} for the thermal decay of pentavalent bismine difluorides **26** and **162** (ln([**26** or **162**]) vs t (s)).

4.7 Reductive elimination from monomeric complex **25**

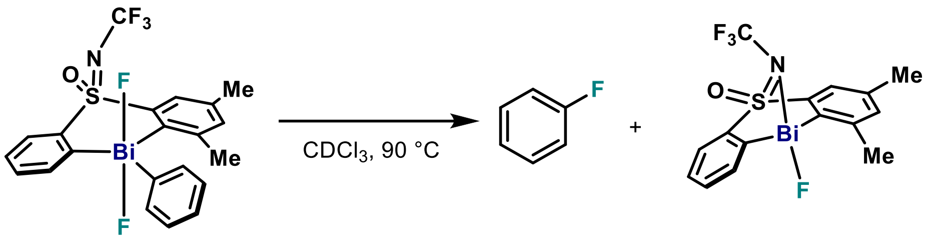

25 **3** **27**

In a J-Young NMR tube, pentavalent bismine **25** (15.7 mg, 0.025 mmol) was mixed with anhydrous $CDCl_3$ (0.5 mL) and 1-fluoro-4-nitrobenzene was added as internal standard (1.0 equiv, 0.025 mmol, 2.65 μL). The mixture was heated to 90 °C inside the NMR probe, and the reaction was monitored by ^{1}H and ^{19}F NMR over 10 h. After completion, fluorobenzene (**3**) was obtained in 93% yield and formation of fluorobismine **27**, which was not characterized, was quantitative.

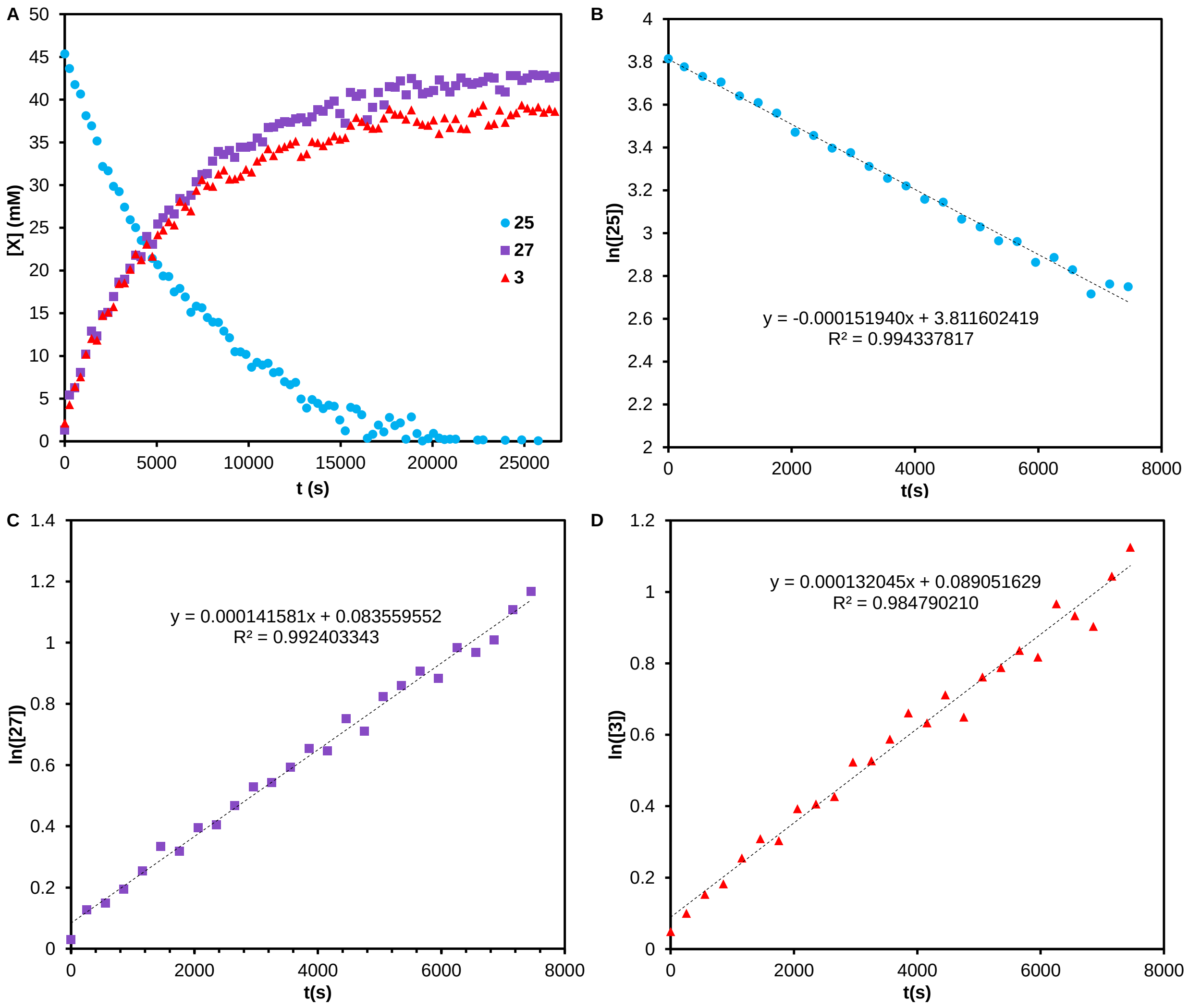

Figure S26. **A.** Kinetic profile of the thermal decay of **25** in $CDCl_3$ at 90 °C (50 mM). **B.** Determination of k_{obs} for the thermal decay of **25** (ln([**25**]) vs t (s)). **C.** Determination of k_{obs} for the formation of **27** (ln([**25**]$_o$/[**25**]$_o$–[**27**]) vs t (s)). **D.** Determination of k_{obs} for the formation of **6** (ln([**25**]$_o$/[**25**]$_o$–[**3**]) vs t (s)).

4.7.1 Reaction monitoring for decay of **25**

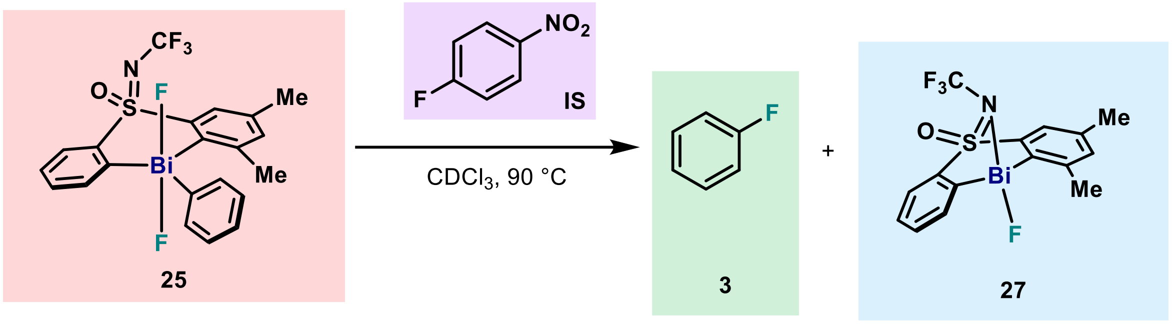

In a J-Young NMR tube, pentavalent bismine **25** (15.9 mg, 0.025 mmol) was mixed with anhydrous $CDCl_3$ (0.5 mL) and 1-fluoro-4-nitrobenzene was added as internal standard (1.0 equiv, 0.025 mmol, 2.65 μL). The mixture was heated to 90 °C inside the NMR probe, and the reaction was monitored by ^{1}H and ^{19}F NMR over 10 h.

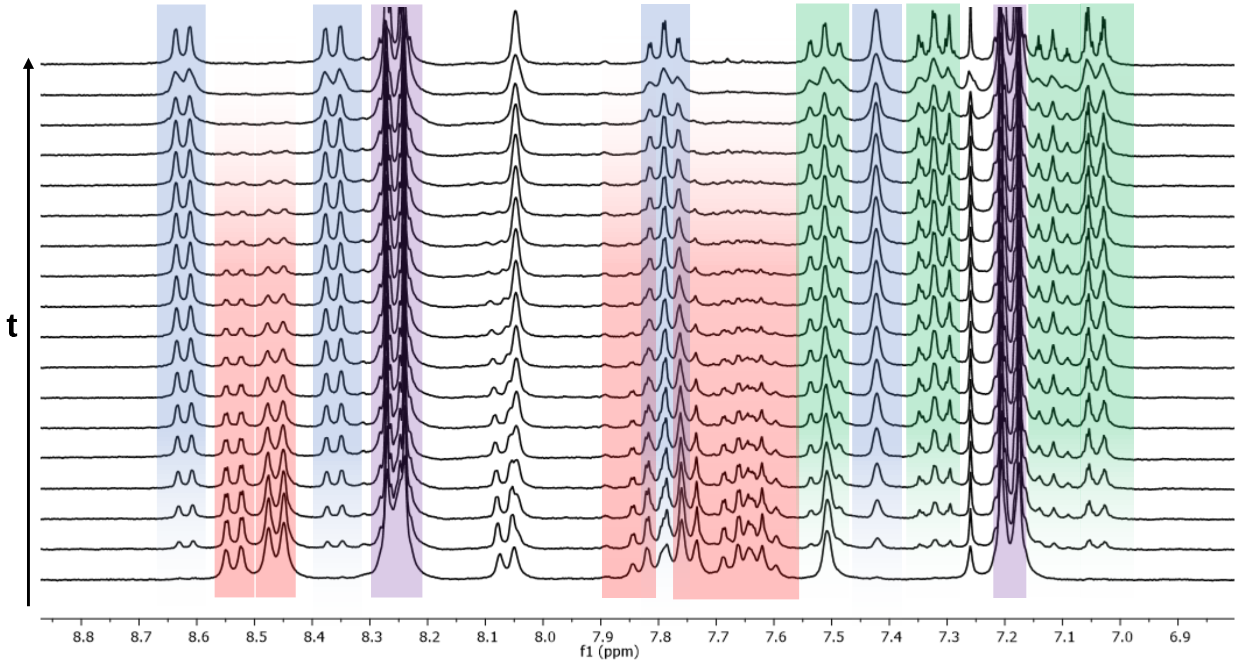

Figure S27. Crude ^{1}H NMR monitoring (aromatic region) of the thermal decay of species **25** (red) in $CDCl_3$ at 90 °C, furnishing fluorobismine **27** (blue) and fluorobenzene (green) in presence of 1-fluoro-4-nitrobenzene as internal standard (purple).

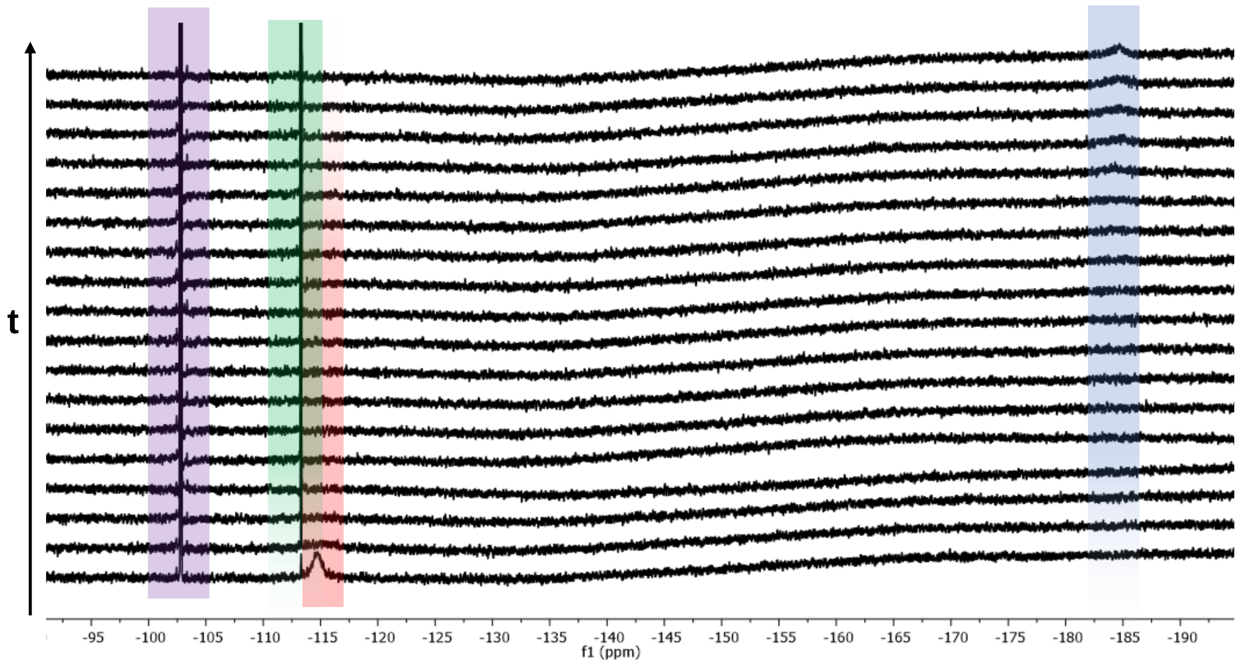

Figure S28. Crude ^{19}F NMR monitoring (aromatic region) of the thermal decay of species **25** (red) in $CDCl_3$ at 90 °C, furnishing fluorobismine **27** (blue) and fluorobenzene (green) in presence of 1-fluoro-4-nitrobenzene as internal standard (purple).

4.7.2 Hammett for the reductive elimination from pentavalent bismines with *p*-substituted arenes

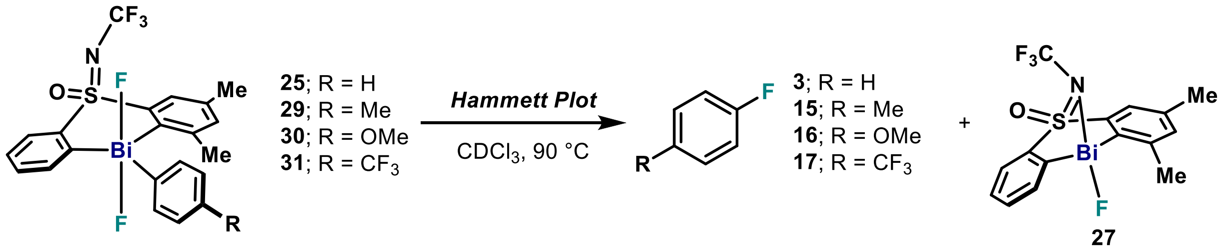

In a J-Young NMR tube, pentavalent bismine **25**, **29-31** (0.025 mmol) was mixed with anhydrous $CDCl_3$ (0.5 mL) and 1-fluoro-4-nitrobenzene was added as internal standard (1.0 equiv, 0.025 mmol, 2.65 μL). The mixture was heated to 90 °C inside the NMR probe, and the reaction was monitored by ^{1}H and ^{19}F NMR over 10 h. Kinetic constants were obtained by plotting the Napierian logarithm (ln) of concentrations of the corresponding pentavalent bismine species versus time (at least 20% conversion).

Table S12. Data corresponding to the Hammett plot for the thermal decomposition of **25**, **29-31**.

p-X (bismine)	k_X	k_X/k_H	$\log(k_X/k_H)$	σ_p	σ_p^+
CF_3 (**31**)	0.0004080	5.124	0.415955	0.53	0.61
H (**25**)	0.0001566	1	0	0	0
Me (**29**)	0.0000563	0.359	–0.444459	–0.17	–0.31
OMe (**30**)	0.0000101	0.064	–1.191707	–0.27	–0.78

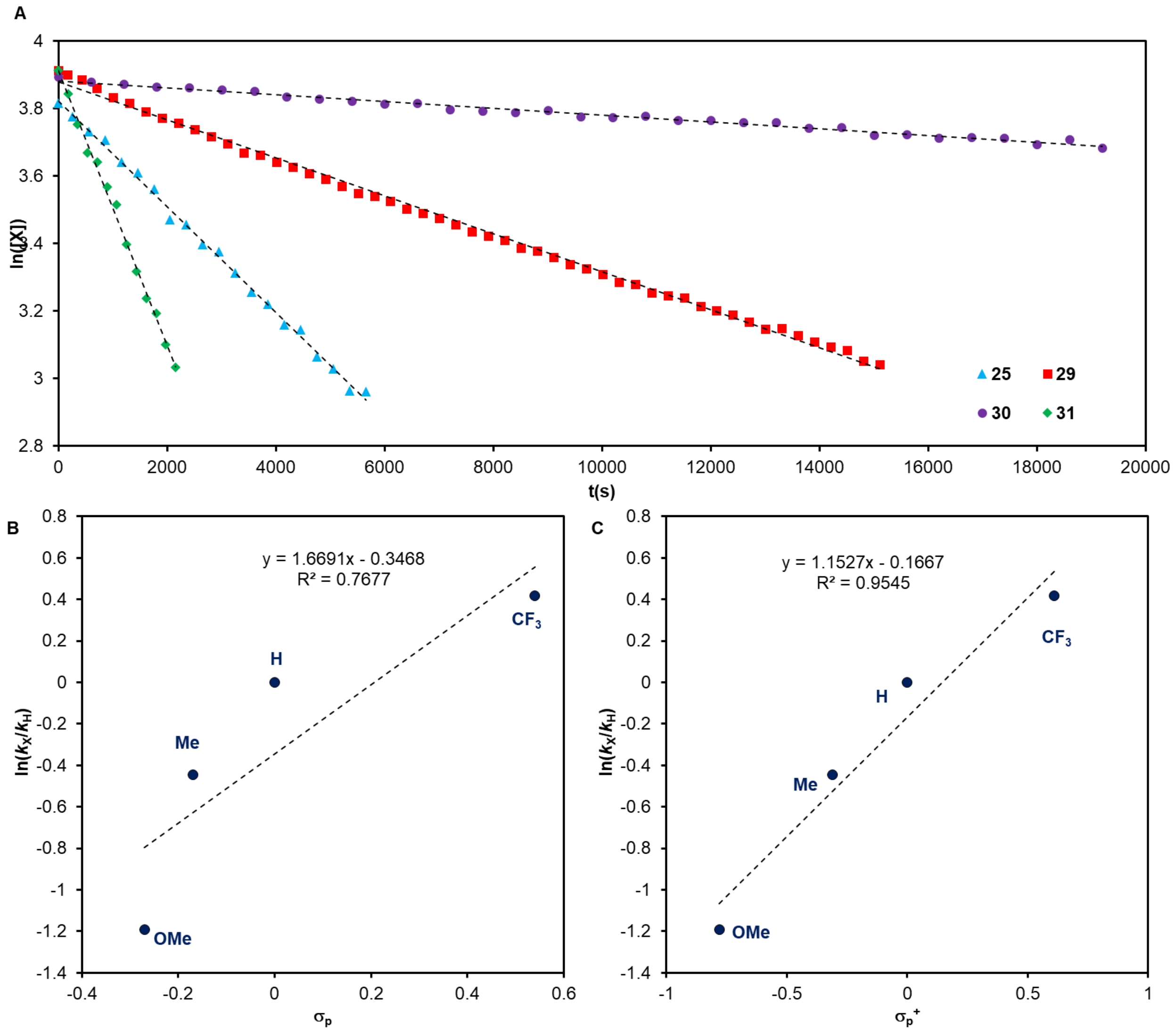

Figure S29. A. Determination of k_{obs} for the thermal decay of *para*-substituted arylbismines (**25**, **29**-**31**) at 90 °C in $CDCl_3$ (50 mM solution). **B.** Hammett plot for **25**, **29**-**31** (ρ = 1.67, R^2 = 0.77). **C.** Hammett plot for **25**, **29**-**31** considering resonance effects (ρ = 1.15, R^2 = 0.95).

4.7.3 Hammett for the reductive elimination from pentavalent bismines with *p*-substituted arenes

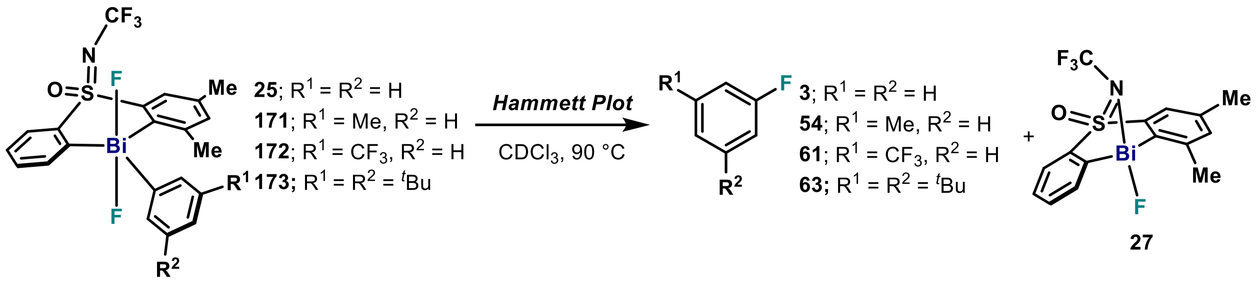

In a J-Young NMR tube, pentavalent bismine **25**, **171-173** (0.025 mmol) was mixed with anhydrous $CDCl_3$ (0.5 mL) and 1-fluoro-4-nitrobenzene was added as internal standard (1.0 equiv, 0.025 mmol, 2.65 μL). The mixture was heated to 90 °C inside the NMR probe, and the reaction was monitored by ^1H and ^{19}F NMR over 10 h. Kinetic constants were obtained by plotting the Napierian logarithm (ln) of concentrations of the corresponding pentavalent bismine species versus time (at least 20% conversion).

Table S13. Data corresponding to the Hammett plot for the thermal decomposition of **25**, **171-173**.

m-X (bismine)	k_X	k_{rel}
H (**25**)	0.00015658	1
Me (**171**)	0.00017603	1.124218
CF_3 (**172**)	0.0001397	0.892196
$^tBu_2$ (**173**)	0.00080231	5.123962

4.7.4 Eyring plot for the reductive elimination from **25**

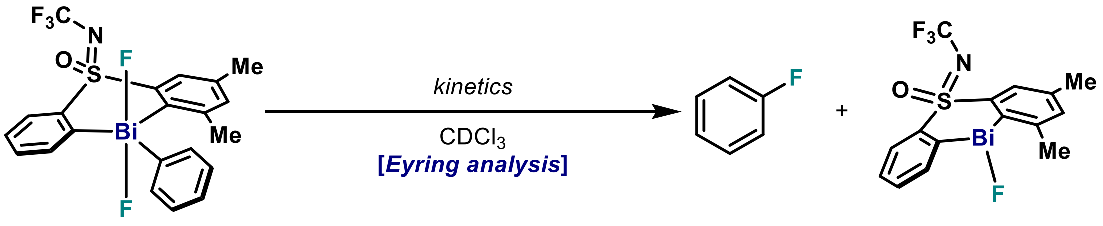

25 **3** **27**

In a J-Young NMR tube, pentavalent bismine **25** (15.9 mg, 0.025 mmol) was mixed with anhydrous $CDCl_3$ (0.5 mL) and 1-fluoro-4-nitrobenzene was added as internal standard (1.0 equiv, 0.025 mmol, 2.65 μL). The mixture was heated to different temperatures (70-90 °C) inside the NMR probe, and the reaction was monitored by ^{1}H and ^{19}F NMR over 10 h. Kinetic constants were obtained by plotting the Napierian logarithm (ln) of concentrations of the corresponding pentavalent bismine species versus time (at least 20% conversion).

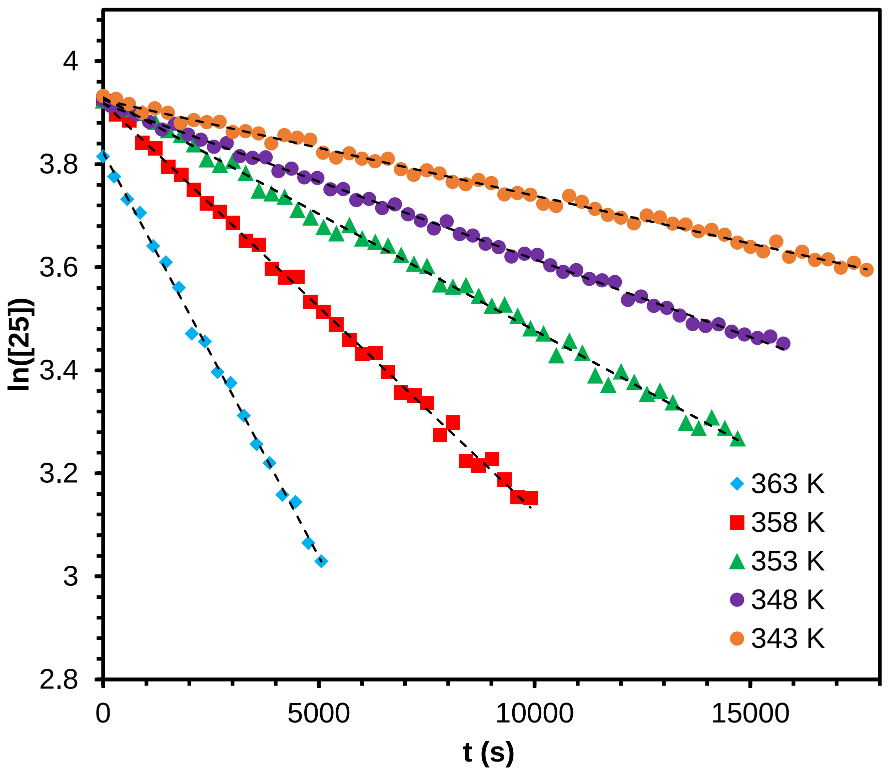

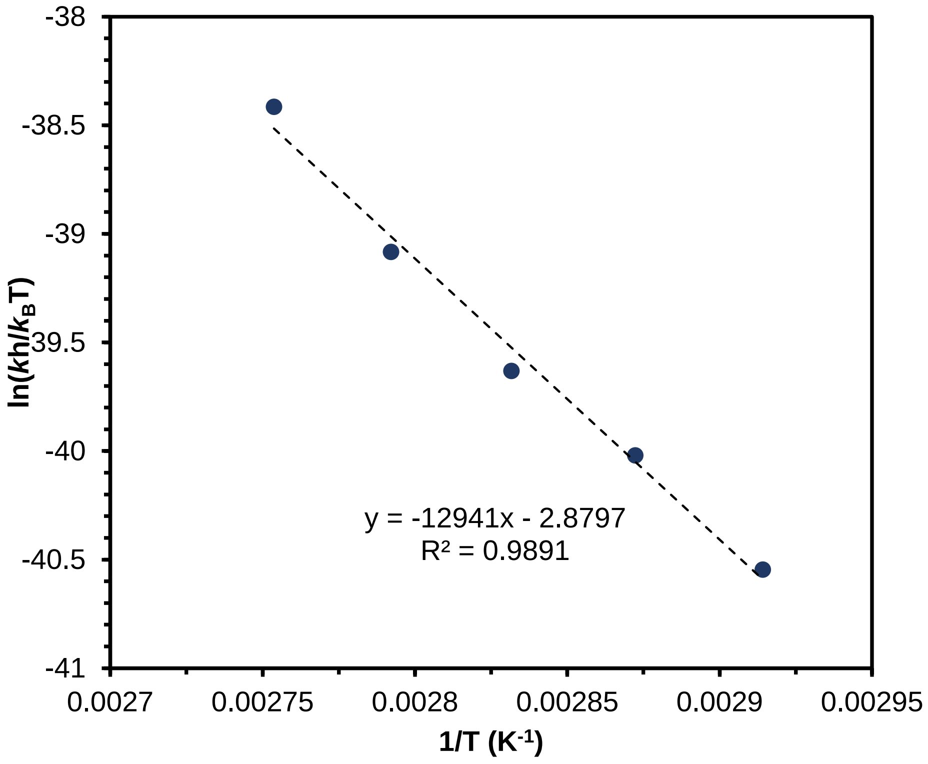

Figure S30. A. Determination of k_{obs} for the thermal decay of **25** at different temperatures (ln([**25**]) vs t (s)) in $CDCl_3$ (50 mM). **B.** Eyring plot for **25**.

Table S14. Data corresponding to the Eyring plot for the thermal decomposition of **25**.

T(° C)	T(K)	1/T (K^{-1})	k_{obs} (s^{-1})	$k_{obs}h/k_BT$	ln ($k_{obs}h/k_BT$)
90.0	363.15	0.002753683	1.57×10^{-4}	2.07485×10^{-17}	–38.41405821
85.0	358.15	0.002792126	7.94×10^{-5}	1.06343×10^{-17}	–39.08244547
80.0	353.15	0.002831658	4.52×10^{-5}	6.1426×10^{-18}	–39.63128385
75.0	348.15	0.002872325	3.02×10^{-5}	4.16582×10^{-18}	–40.01961751
70.0	343.15	0.002914177	1.76×10^{-5}	2.46291×10^{-18}	–40.54518879

Table S15. Activation parameters for the thermal decomposition of **25**.

	$\Delta H^{\ddagger}$ (kcal mol^{-1})	$\Delta S^{\ddagger}$ (cal K^{-1} mol^{-1})	$\Delta G_{298}^{\ddagger}$ (kcal mol^{-1})
**Calculated**	25.7	–5.7	27.4
**Error**	± 1.6	± 4.4	± 0.9

4.8 Reductive elimination from **26**

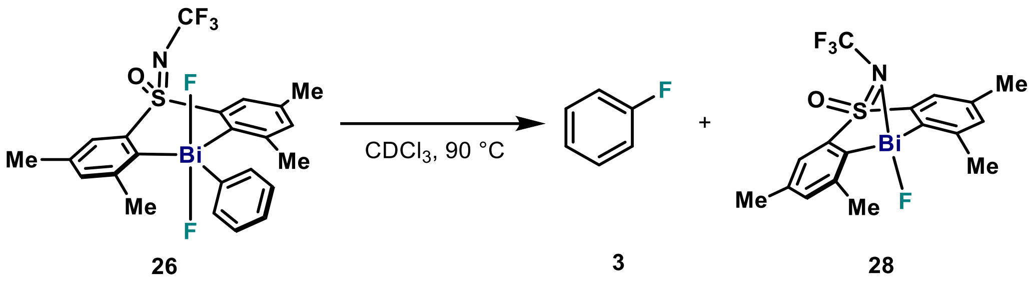

In a J-Young NMR tube, pentavalent bismine **26** (15.7 mg, 0.025 mmol) was mixed with anhydrous $CDCl_3$ (0.5 mL) and 1-fluoro-4-nitrobenzene was added as internal standard (1.0 equiv, 0.025 mmol, 2.65 μL). The mixture was heated to 90 °C inside the NMR probe, and the reaction was monitored by ^{1}H and ^{19}F NMR over 10 h. After completion, fluorobenzene (**3**) was obtained in 93% yield and formation of fluorobismine **28** was quantitative.

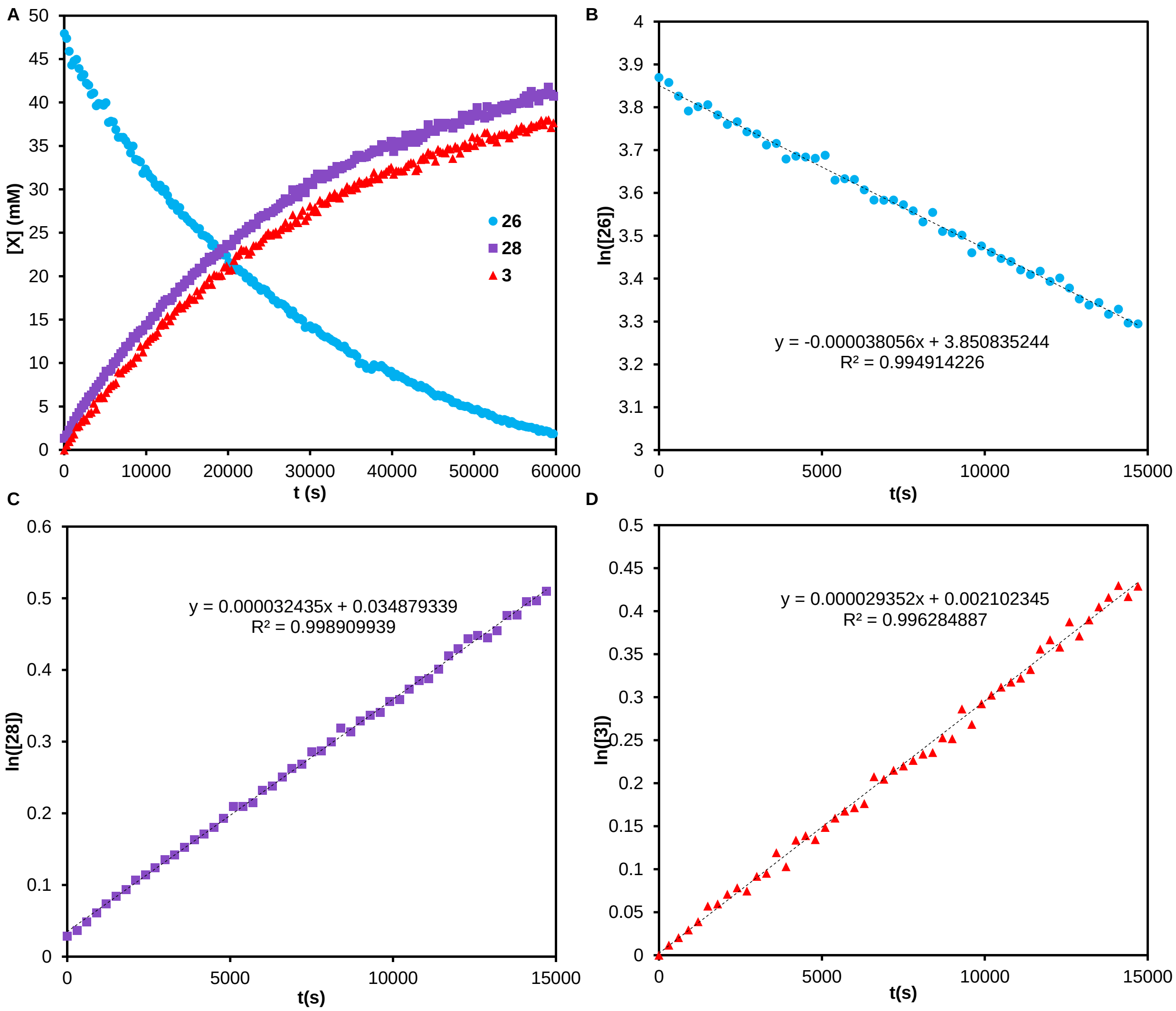

Figure S31. A. Kinetic profile of the thermal decay of **26** in $CDCl_3$ at 90 °C (50 mM). **B.** Determination of k_{obs} for the thermal decay of **26** (ln([**26**]) vs t (s)). **C.** Determination of k_{obs} for the formation of **3** (ln([**26**]$_o$/[**26**]$_o$–[**28**]) vs t (s)). **D.** Determination of k_{obs} for the formation of **6** (ln([**26**]$_o$/[**26**]$_o$–[**3**]) vs t (s)).

4.8.1 Reaction monitoring for decay of **26**

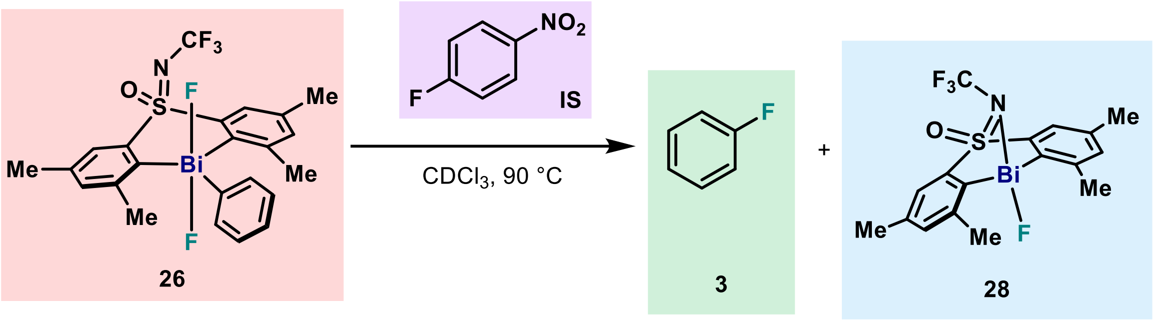

In a J-Young NMR tube, pentavalent bismine **26** (0.025 mmol) was mixed with anhydrous $CDCl_3$ (0.5 mL) and 1-fluoro-4-nitrobenzene was added as internal standard (1.0 equiv, 0.025 mmol, 2.65 μL). The mixture was heated to 90 °C inside the NMR probe, and the reaction was monitored by ^{1}H and ^{19}F NMR over 10 h.

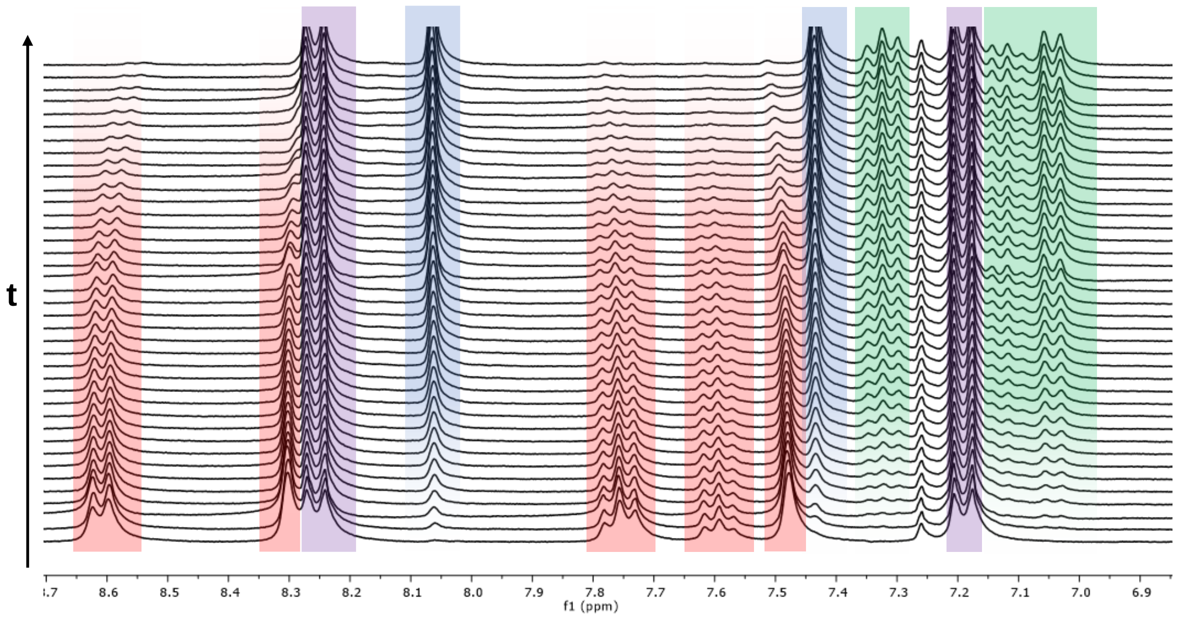

Figure S32. Crude ^{1}H NMR monitoring (aromatic region) of the thermal decay of species **26** (red) in $CDCl_3$ at 90 °C, furnishing fluorobismine **28** (blue) and fluorobenzene (green) in presence of 1-fluoro-4-nitrobenzene as internal standard (purple).

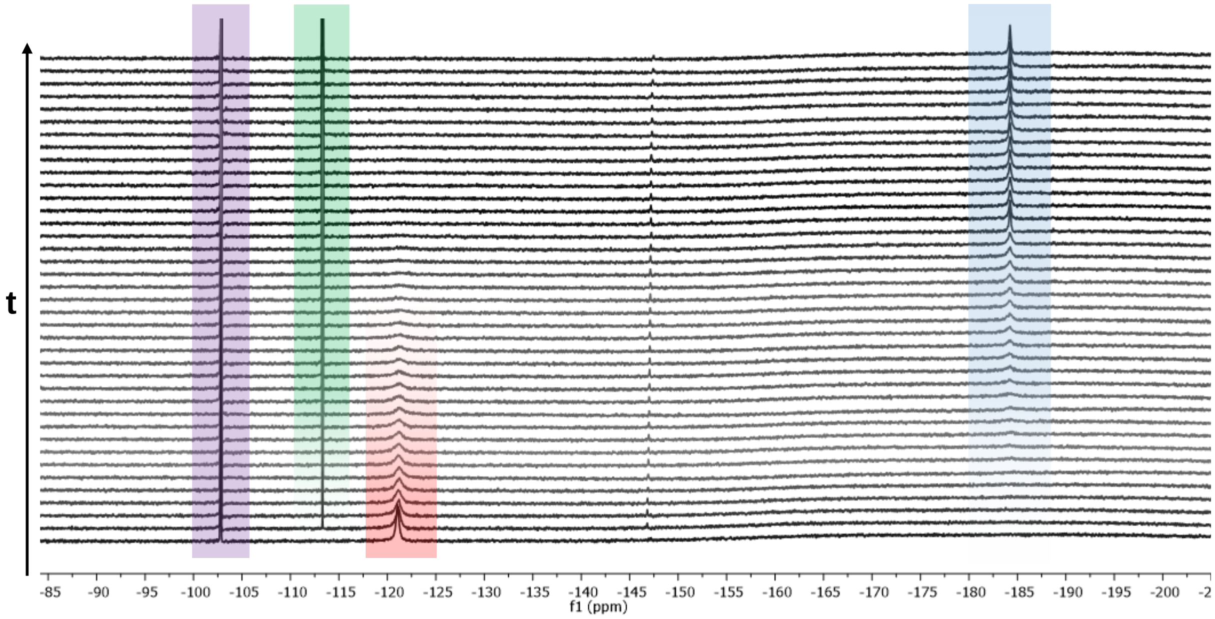

Figure S33. Crude ^{19}F NMR monitoring (aromatic region) of the thermal decay of species **26** (red) in $CDCl_3$ at 90 °C, furnishing fluorobismine **28** (blue) and fluorobenzene (green) in presence of 1-fluoro-4-nitrobenzene as internal standard (purple).

4.8.2 Reaction of **4** with trivalent fluorobismine **28**

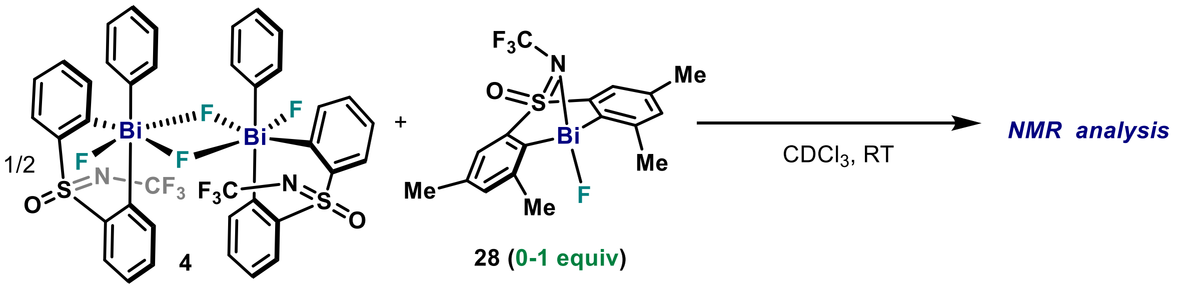

In a standard NMR tube, pentavalent bismine **4** (0.013 mmol, *dimer*) was mixed with fluorobismine **28** (0-1.0 equiv respect to monomer). Then, anhydrous $CDCl_3$ (0.5 mL) was added. The mixture was vigorously shaken and analyzed by ^1H and ^{19}F NMR.

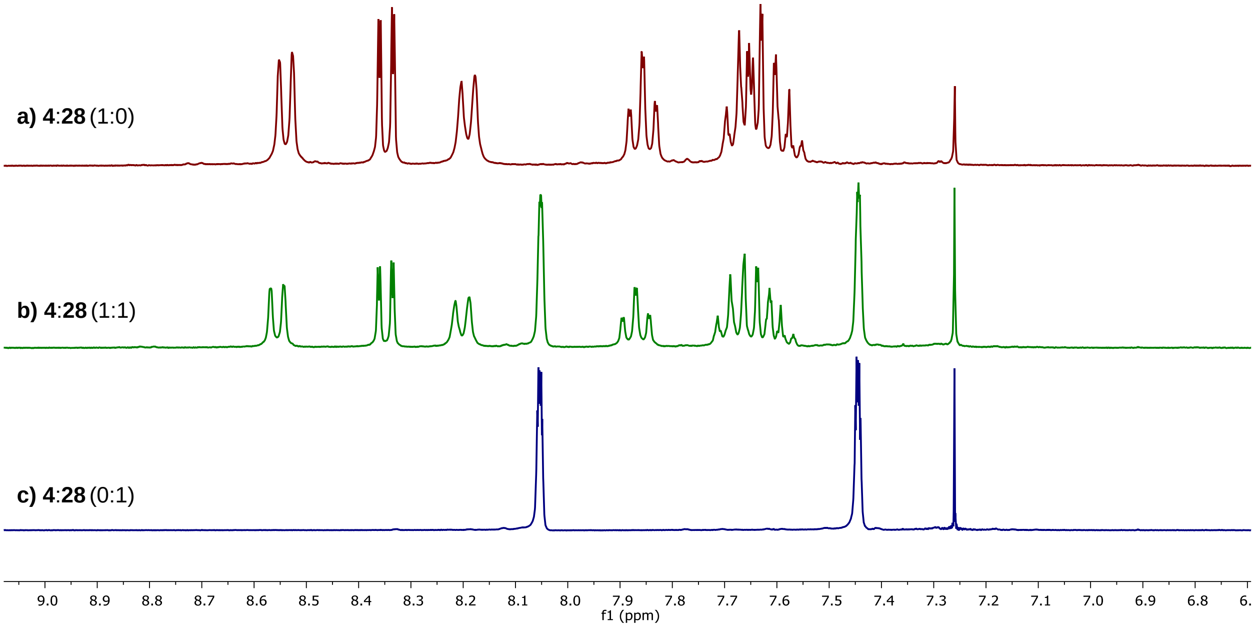

Figure S34. ^{1}H NMR measurements (aromatic region) of mixtures **4** and **28** in different ratios ($CDCl_3$, 298 K).

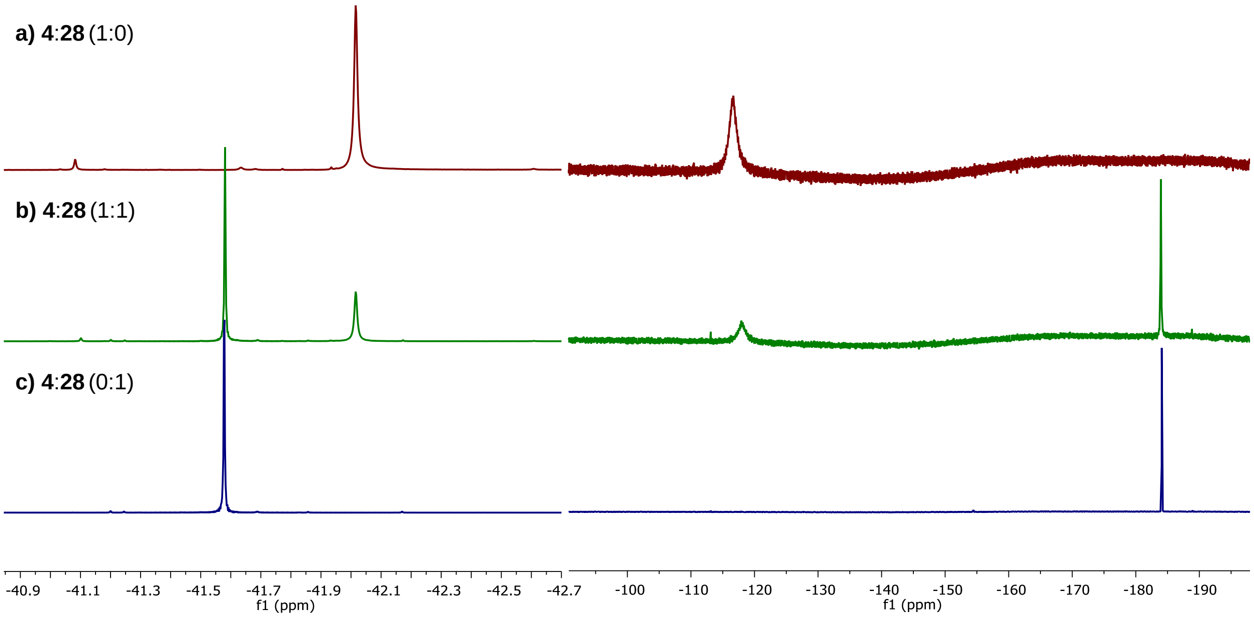

Figure S35. ^{19}F NMR measurements of mixtures **4** and **28** in different ratios ($CDCl_3$, 298 K).

4.8.3 Eyring plot for the reductive elimination from **26**

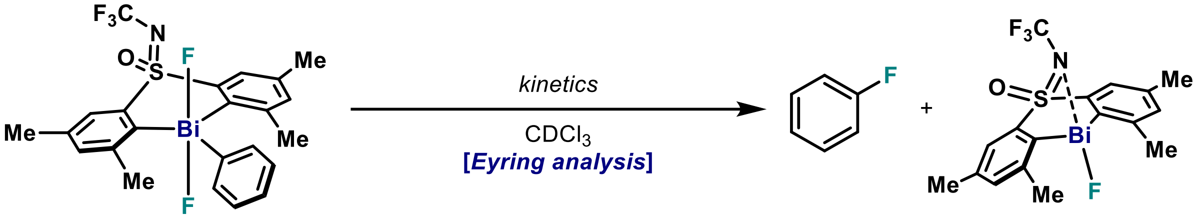

In a J-Young NMR tube, pentavalent bismine **26** (16.7 mg, 0.025 mmol) was mixed with anhydrous $CDCl_3$ (0.5 mL) and 1-fluoro-4-nitrobenzene was added as internal standard (1.0 equiv, 0.025 mmol, 2.65 μL). The mixture was heated to different temperatures (70-100 °C) inside the NMR probe, and the reaction was monitored by ^1H and ^{19}F NMR. Kinetic constants were obtained by plotting the Napierian logarithm (ln) of concentrations of the corresponding pentavalent bismine species versus time (at least 20% conversion).

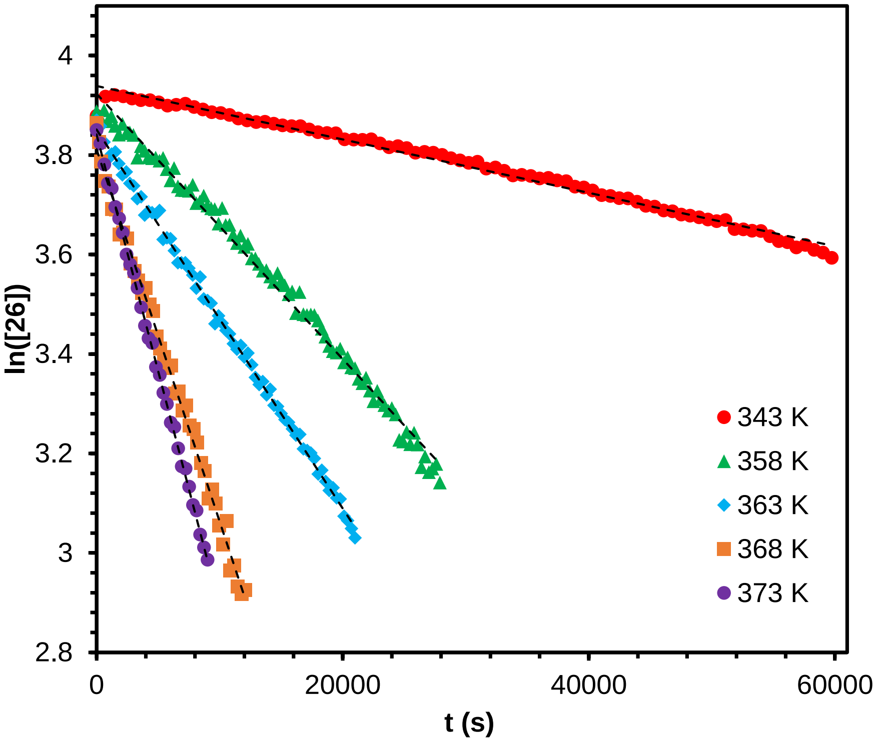

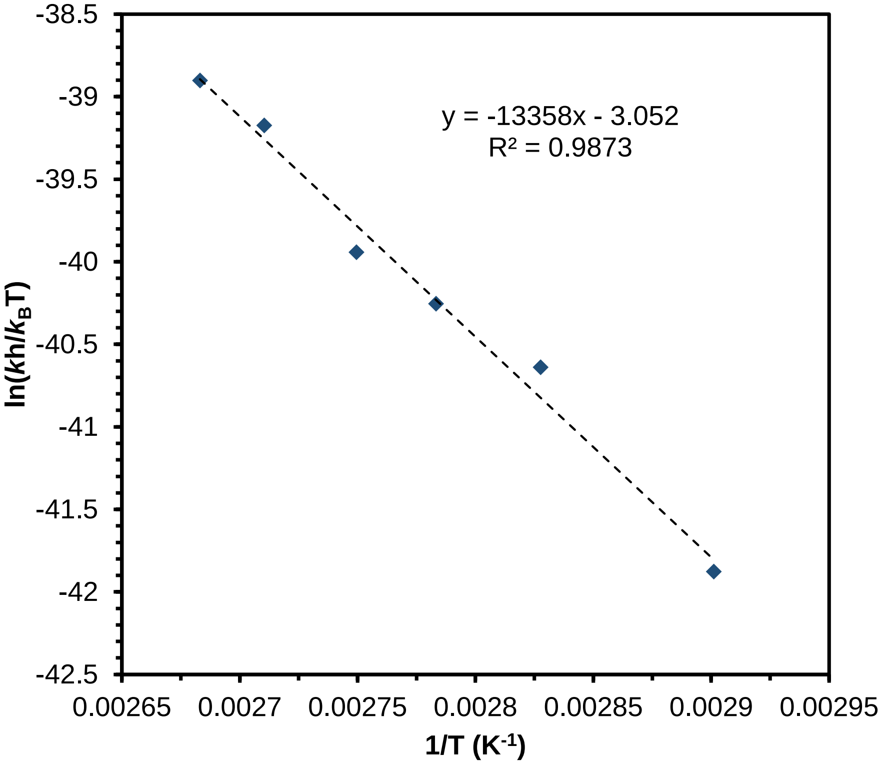

Figure S36. _Top:_ Determination of k_{obs} for the thermal decay of **26** at different temperatures (ln([**26**]) vs t (s)) in $CDCl_3$ (50 mM). _Bottom:_ Eyring plot for **26**.

Table S16. Data corresponding to the Eyring plot for the thermal decomposition of **26**

T(° C)	T(K)	1/T (K^{-1})	k_{obs} (s^{-1})	$k_{obs}h/k_BT$	ln ($k_{obs}h/k_BT$)
100	372.70	0.00268312	9.89×10^{-5}	1.27315×10^{-17}	–38.90245599
95	368.90	0.00270278	7.46×10^{-5}	9.67787×10^{-18}	–39.17668948
90	363.70	0.00274952	3.41×10^{-5}	5.02357×10^{-18}	–39.83239083
85	359.30	0.00278319	2.47×10^{-5}	3.29255×10^{-18}	–40.25486884
80	353.50	0.000340098	1.65×10^{-5}	2.24186×10^{-18}	–40.63922400
70	344.70	0.00290107	4.67×10^{-6}	7.47663×10^{-19}	–41.73733461

Table S17. Activation parameters for the thermal decomposition of **26**.

	$\Delta H^{\ddagger}$ (kcal mol^{-1})	$\Delta S^{\ddagger}$ (cal K^{-1} mol^{-1})	$\Delta G_{298}{}^{\ddagger}$ (kcal mol^{-1})
**Calculated**	26.5	–6.1	28.3
**Error**	± 1.5	±4.2	± 1.1

4.9 Addition of fluoride sources

4.9.1 Reaction of diverse fluoride salts with pentavalent bismine **4**

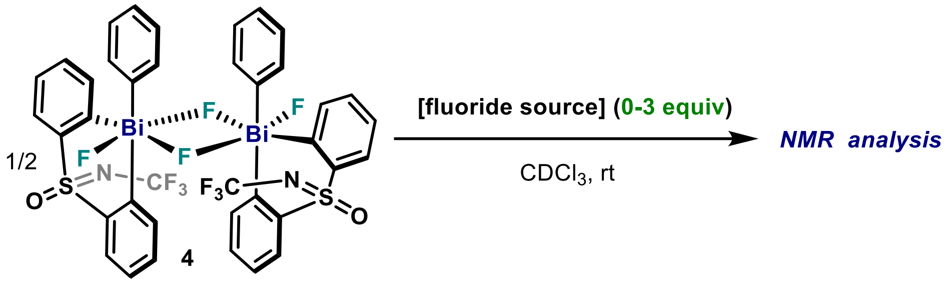

In an NMR tube, difluorophenylbismine **4** (0.02 mmol) was mixed with the corresponding fluoride source (0-3 equiv respect to monomer) and then $CDCl_3$ (1 mL) was added under an Ar atmosphere. The NMR tube was vigorously shaken and the crude was directly analyzed by ^1H and ^{19}F NMR (see Figures S37-S43).

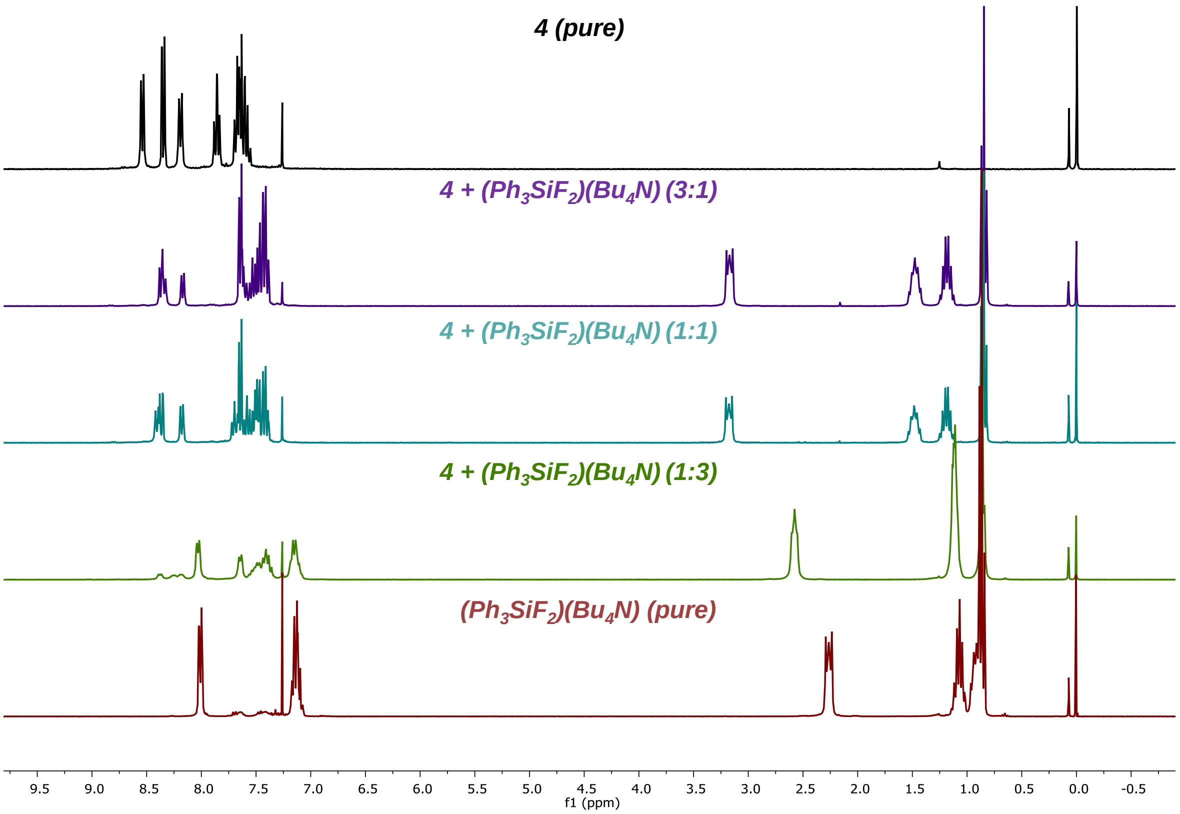

Figure S37. Crude ^1H NMR of pure **4** (black), mixtures of **4** and TBAT (purple, 3:1; blue, 1:1; green 1:3) and commercially available TBAT (red).

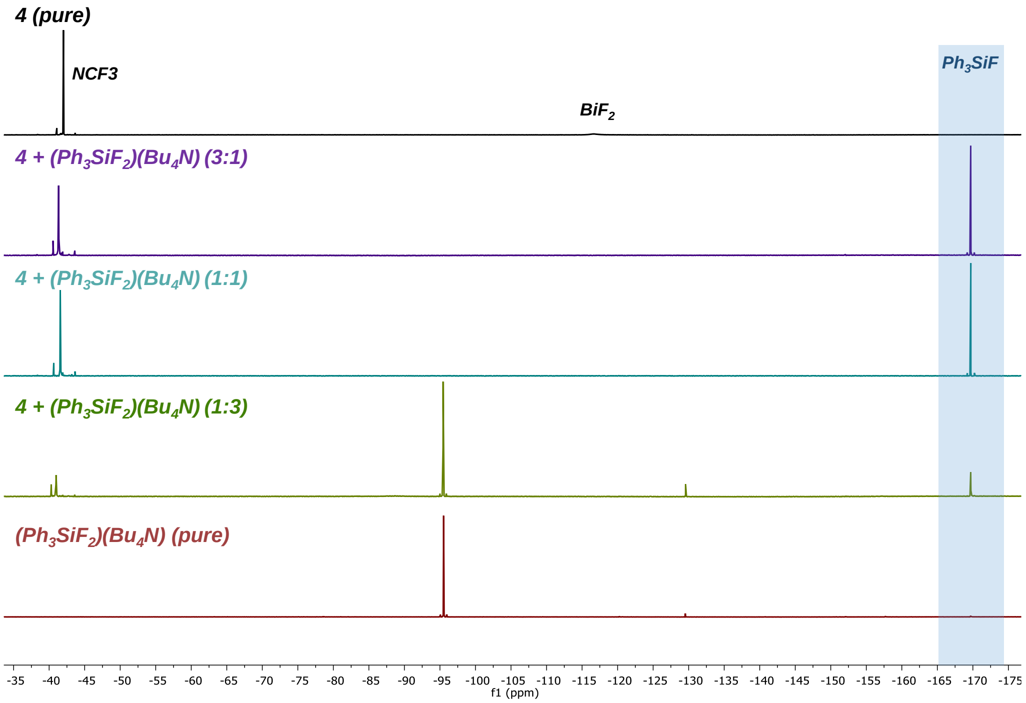

Figure S38. Crude ^{19}F NMR of pure **4** (black), mixtures of **4** and TBAT (purple, 3:1; blue, 1:1; green 1:3) and commercially available TBAT (red).

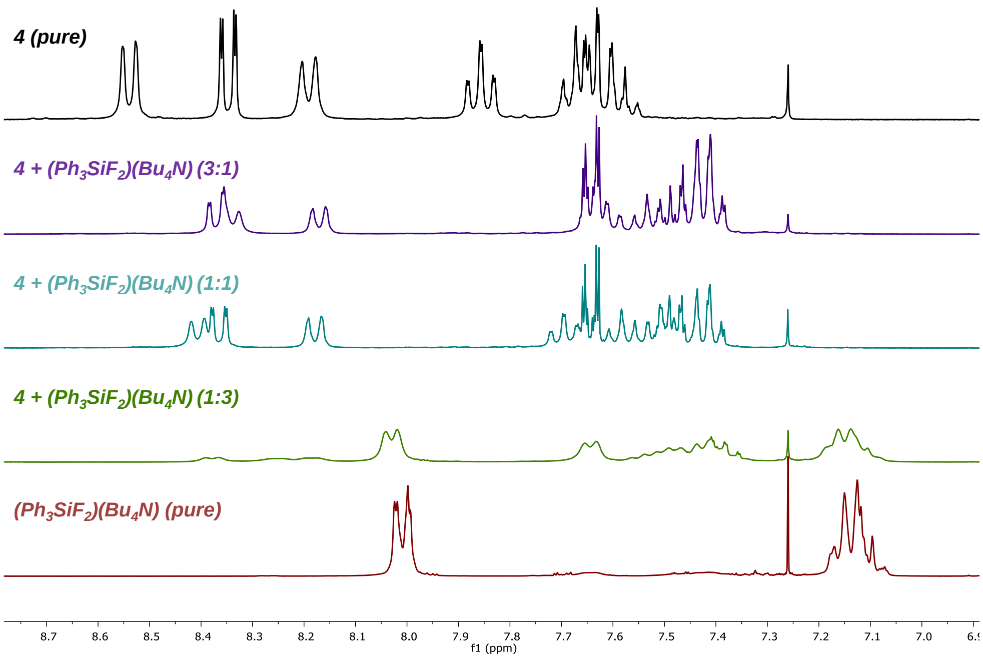

Figure S39. Crude ^{1}H NMR (*aromatic region*) of pure **4** (black), mixtures of **4** and TBAT (purple, 3:1; blue, 1:1; green 1:3) and commercially available TBAT (red).

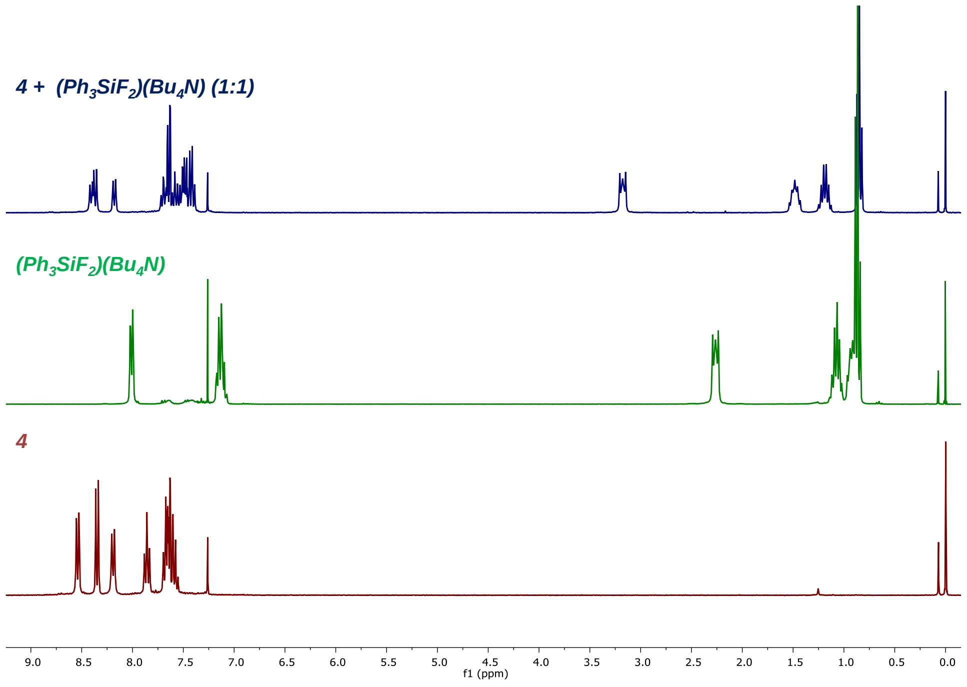

Figure S40. Crude ^{1}H NMR of a mixture of TBAT and **4** (blue) compared to commercially available TBAT (green) and pure pentavalent bismine **4** (red).

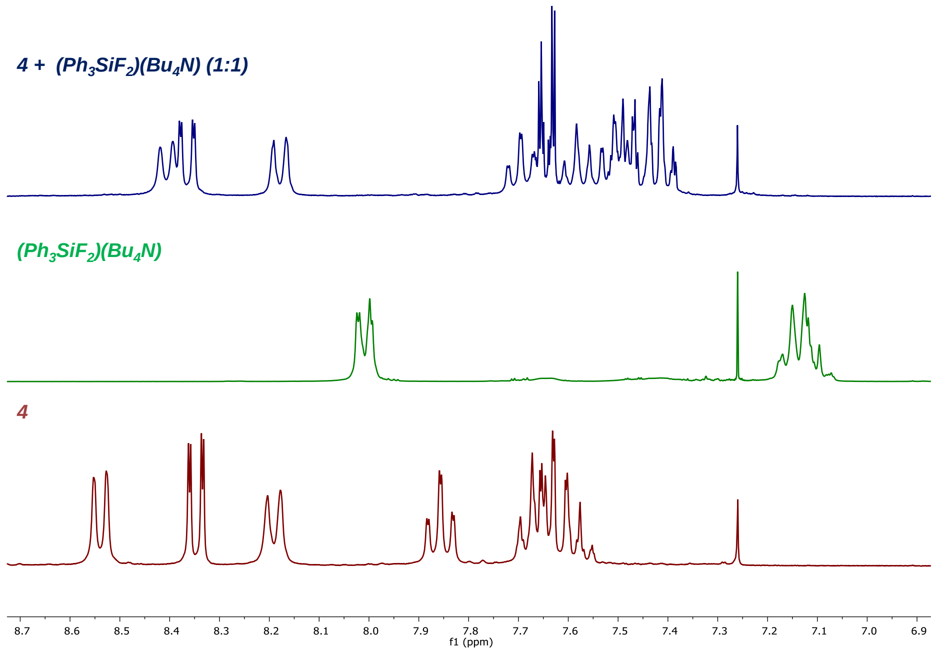

Figure S41. Crude ^{1}H NMR (*aromatic region*) of a mixture of TBAT and **4** (blue) compared to commercially available TBAT (green) and pure pentavalent bismine **4** (red).

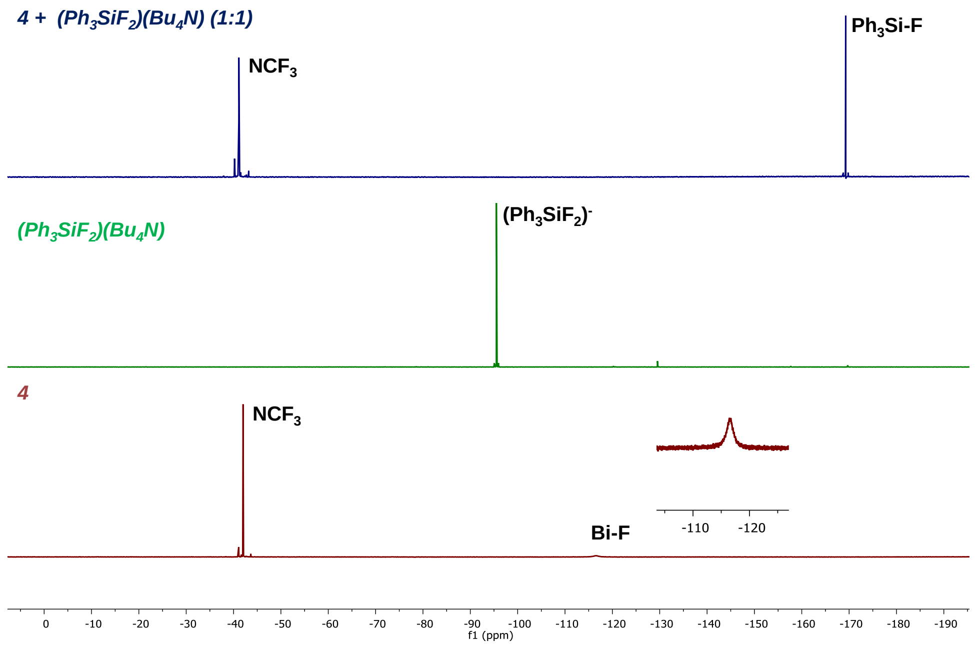

Figure S42. Crude ^{19}F NMR of a mixture of TBAT and **4** (blue) compared to commercially available TBAT (green) and pure pentavalent bismine **4** (red).

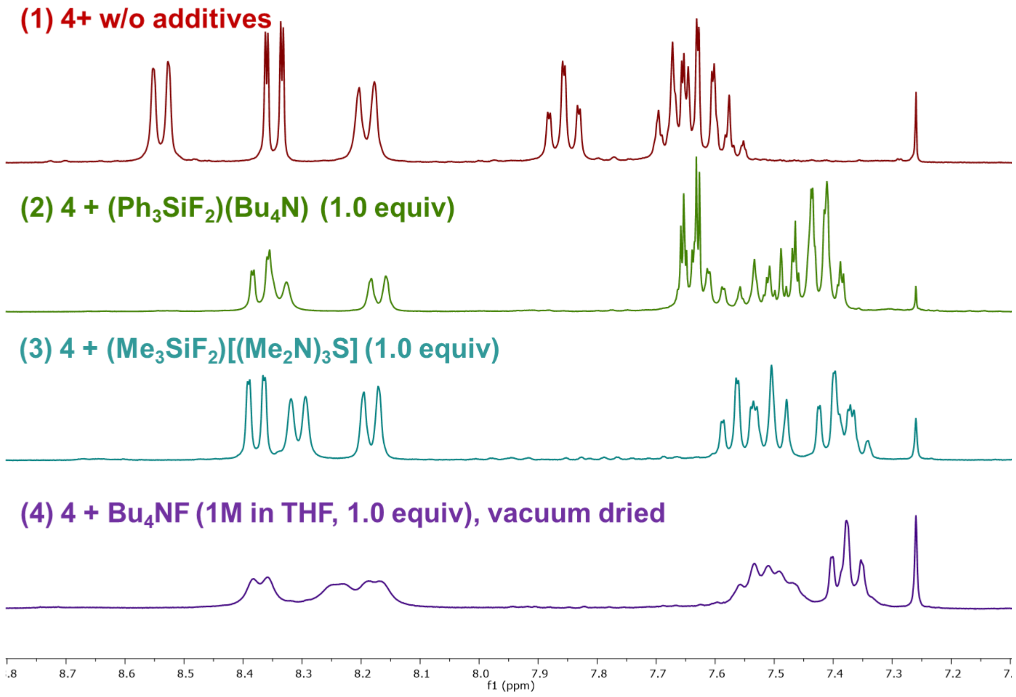

Figure S43. Crude ^{1}H NMR of a mixture of TBAT and **4** (blue) compared to commercially available TBAT (green) and pure pentavalent bismine **4** (red).

4.9.2 Kinetics in presence of TBAT

*4.9.2.1 Reaction profile of **4** decay in presence of TBAT*

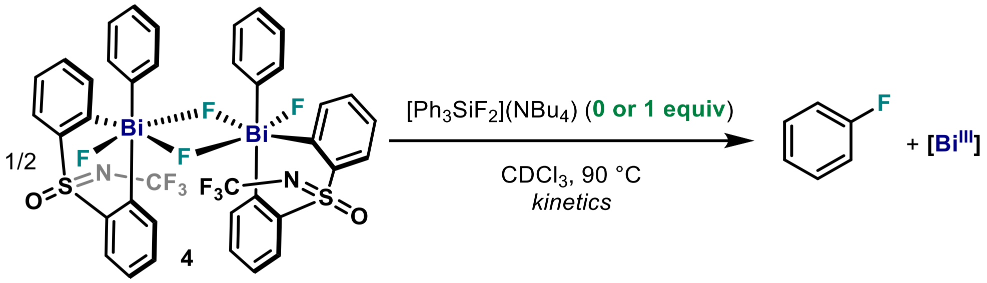

In a J-Young NMR tube, pentavalent bismine **4** (15.2 mg, 0.013 mmol, *dimer*) was mixed with TBAT (0-1 equiv respect to monomeric species), anhydrous $CDCl_3$ (0.5 mL) and 1-fluoro-4-nitrobenzene was added as internal standard (1.0 equiv, 0.025 mmol, 2.65 μL). NMR samples were placed at a preheated NMR machine (90 °C) and the reaction was monitored by ^1H and ^{19}F NMR. Kinetic constants were obtained by plotting the Napierian logarithm (ln) of concentrations of the corresponding pentavalent bismine species versus time (at least 20% conversion, initial rates).

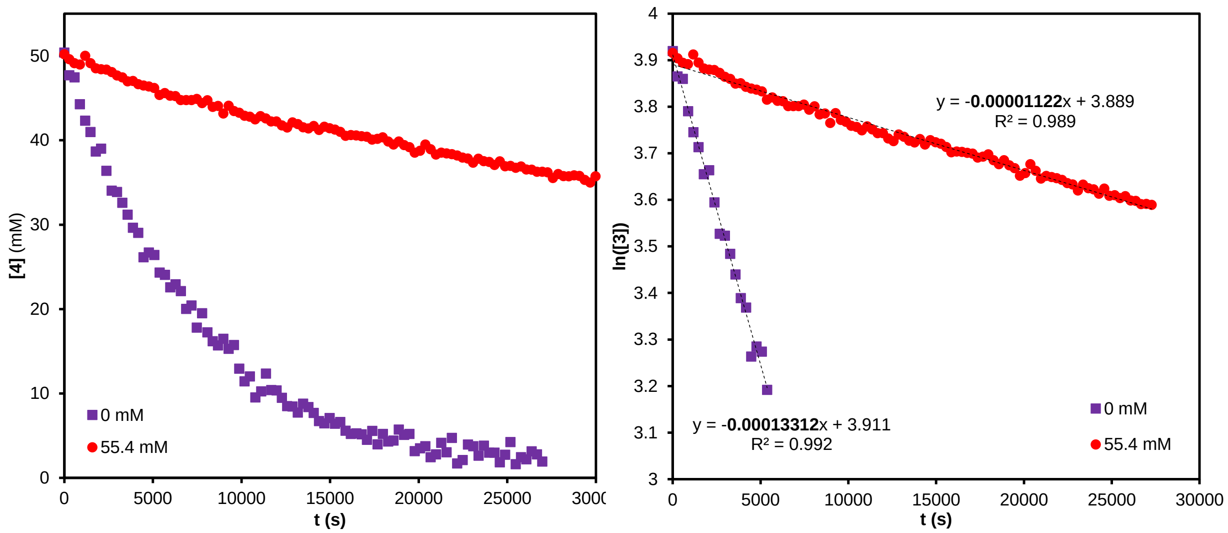

Figure S44. Left: Kinetic profile of the thermal decay of **4** in $CDCl_3$ at 90 °C (50 mM) in presence of TBAT (red, [TBAT] = 55.4 mM, 1.1 equiv) or in absence of it (purple). *Right:* Determination of k_{obs} for the thermal decay of **4** (ln([**4**]) vs t (s)) for the reactions in presence (red) and in absence (purple) of TBAT.

*4.9.2.2 Dependence of fluoride concentration for decay of **4***

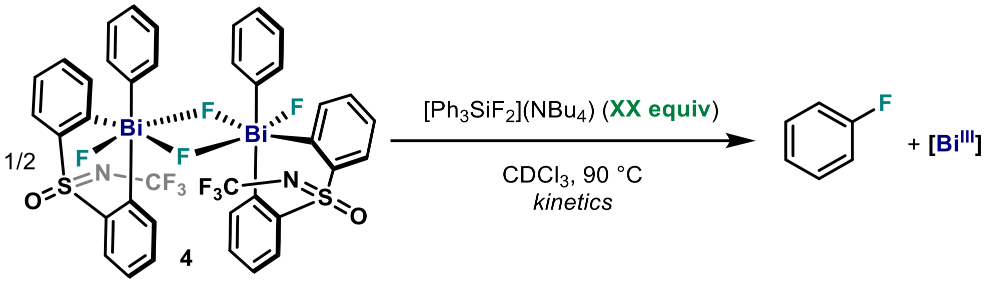

In a J-Young NMR tube, pentavalent bismine **4** (15.2 mg, 0.013 mmol, *dimers*) was mixed with TBAT (0-1.1 equiv respect to monomeric species), anhydrous $CDCl_3$ (0.5 mL) and 1-fluoro-4-nitrobenzene was added as internal standard (1.0 equiv respect to monomeric species, 0.025 mmol, 2.65 µL). NMR samples were placed at a preheated NMR machine (90 °C) and the reaction was monitored by ^1H and ^{19}F NMR. Kinetic constants were obtained by plotting the Napierian logarithm (ln) of concentrations of the corresponding pentavalent bismine species versus time (at least 20% conversion, initial rates).

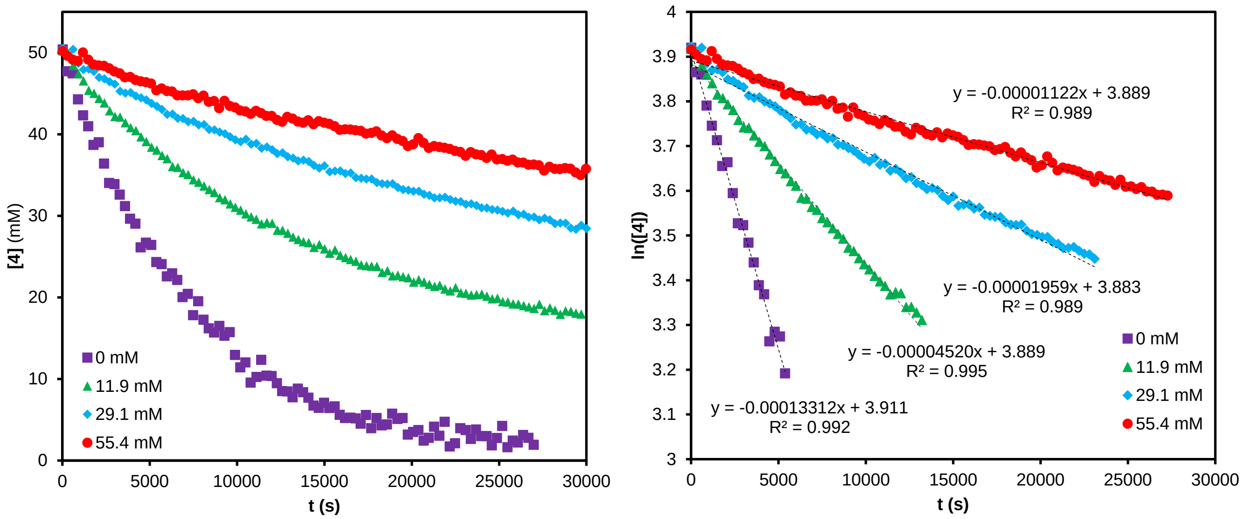

Figure S45. Left: Kinetic profile of the thermal decay of **4** in $CDCl_3$ at 90 °C (50 mM) in presence of different concentrations of TBAT (red, [TBAT] = 55.4 mM, 1.1 equiv; blue, [TBAT] = 29.1 mM, 0.58 equiv; green, [TBAT] = 11.9 mM, 0.238 equiv) and in absence of it (purple). Right: Determination of k_{obs} for the thermal decay of **4** (ln([**4**]) vs t (s)) for the reactions in presence and in absence of TBAT.

[TBAT] (mM)	k_{obs}	$1/k_{obs}$	Yield of PhF[a]
55.4	0.00001122	89126.55971	71%
29.1	0.00001959	51046.45227	83%
11.9	0.0000452	22123.89381	93%
0	0.00013312	7512.019231	94%

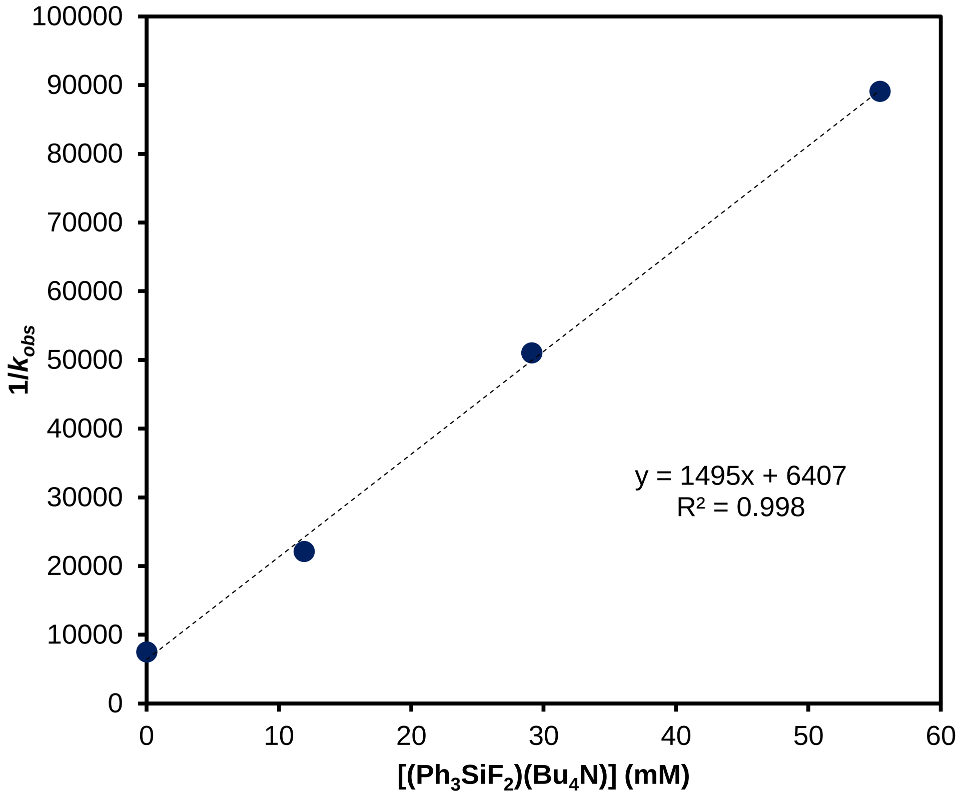

Figure S46. Relationship between rate (k_{obs}) of decomposition of **4** and TBAT concentration. [a]Yield of fluorobenzene after full conversion of **4**. Yield calculated by ^{19}F NMR using and 1-fluoro-4-nitrobenzene as internal standard.

4.9.4 Reaction of diverse fluoride salts with pentavalent bismine **26**

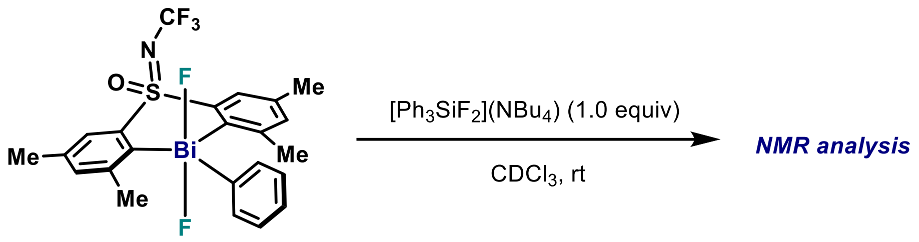

26

In an NMR tube, difluorophenylbismine **26** (19.9 mg, 0.03 mmol) was mixed with TBAT (1.0 equiv) and then $CDCl_3$ (1 mL) was added under an Ar atmosphere. The NMR tube was vigorously shaken and the crude was directly analyzed by ^{1}H and ^{19}F NMR (see Figures S47-S49).

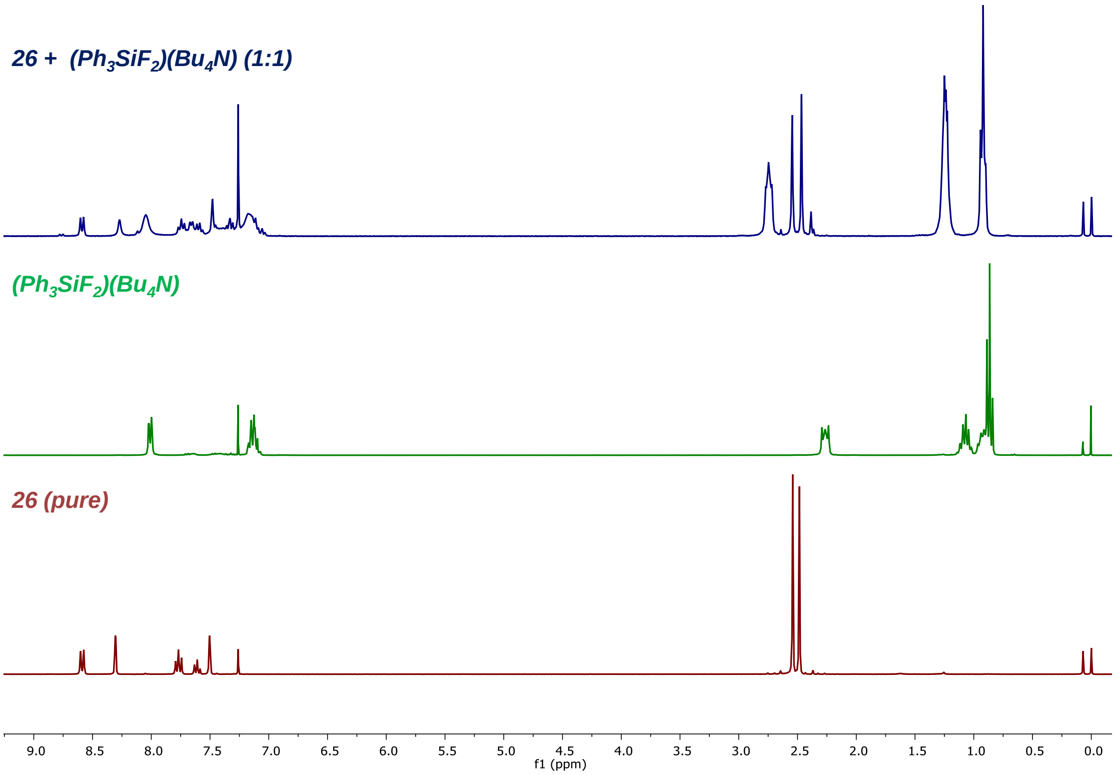

Figure S47. Crude ^{1}H NMR of a 1:1 mixture of TBAT and **26** (blue) compared to commercially available TBAT (green) and pure pentavalent bismine **26** (red).

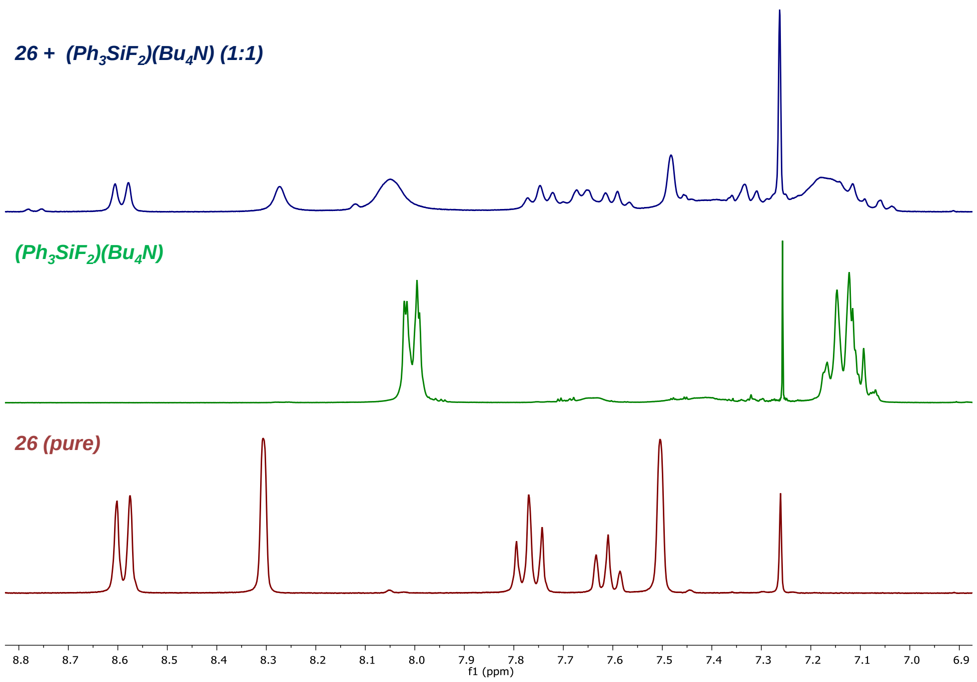

Figure S48. Crude ^{1}H NMR (aromatic region) of a 1:1 mixture of TBAT and **26** (blue) compared to commercially available TBAT (green) and pure pentavalent bismine **26** (red).

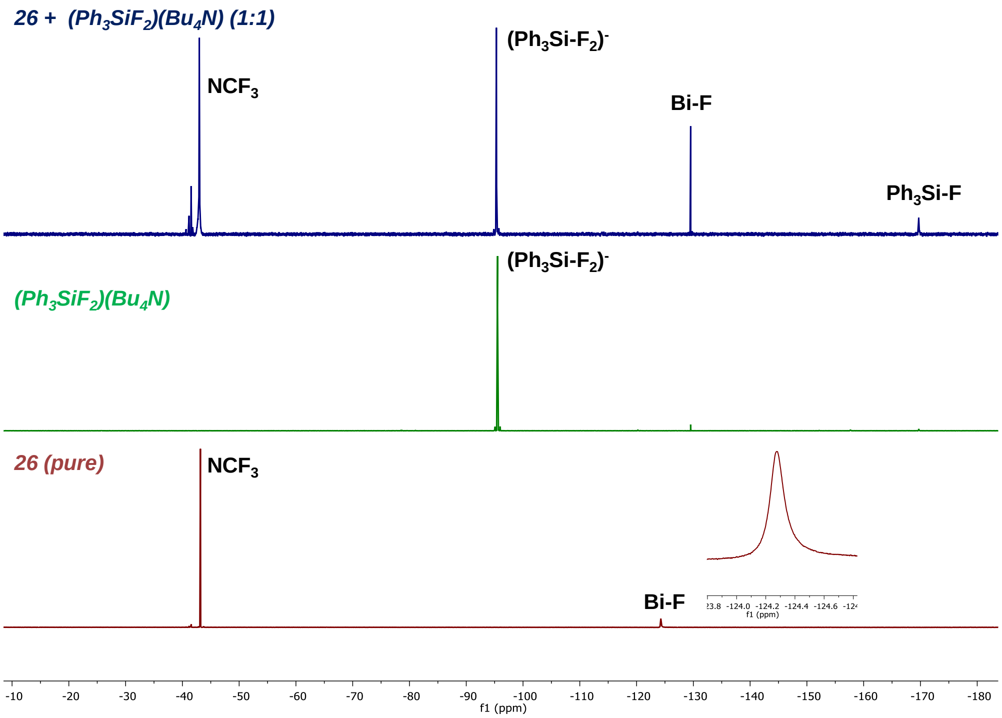

Figure S49. Crude ^{19}F NMR of a 1:1 mixture of TBAT and **26** (blue) compared to commercially available TBAT (green) and pure pentavalent bismine **26** (red).

*4.9.4.1 Reaction profile of **26** decay in presence of TBAT*

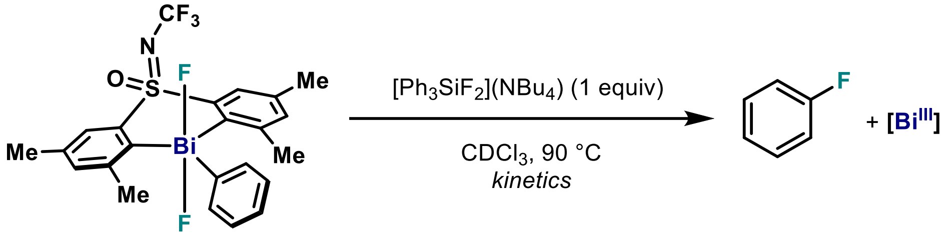

26

In a J-Young NMR tube, pentavalent bismine **26** (16.7 mg, 0.025 mmol) was mixed with TBAT (0-1 equiv), anhydrous $CDCl_3$ (0.5 mL) and 1-fluoro-4-nitrobenzene was added as internal standard (1.0 equiv, 0.025 mmol, 2.65 μL). NMR samples were placed at a preheated NMR machine (90 °C) and the reaction was monitored by ^1H and ^{19}F NMR. Kinetic constants were obtained by plotting the Napierian logarithm (ln) of concentrations of the corresponding pentavalent bismine species versus time (at least 20% conversion, initial rates).

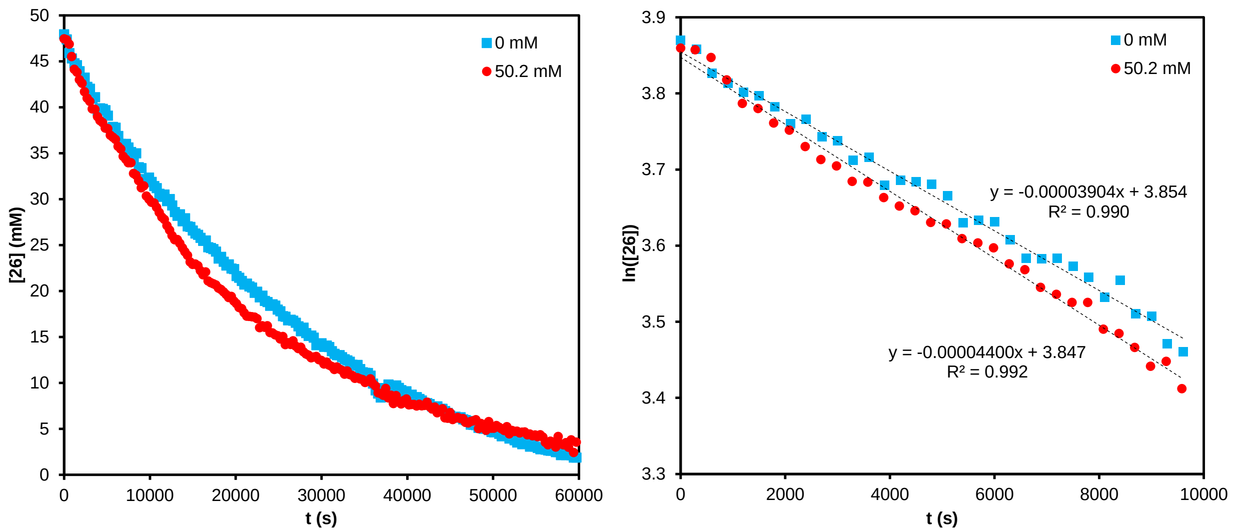

Figure S50. *Left*: Kinetic profile of the thermal decay of **26** in $CDCl_3$ at 90 °C (50 mM) in presence of TBAT (red, [TBAT] = 55.4 mM, 1.1 equiv) or in absence of it (blue). *Right:* Determination of k_{obs} for the thermal decay of **26** (ln([**26**]) vs t (s)) for the reactions in presence (red) and in absence (blue) of TBAT.

4.9.5 Synthesis and characterization of anionic pentavalent bismine **35-TAS**

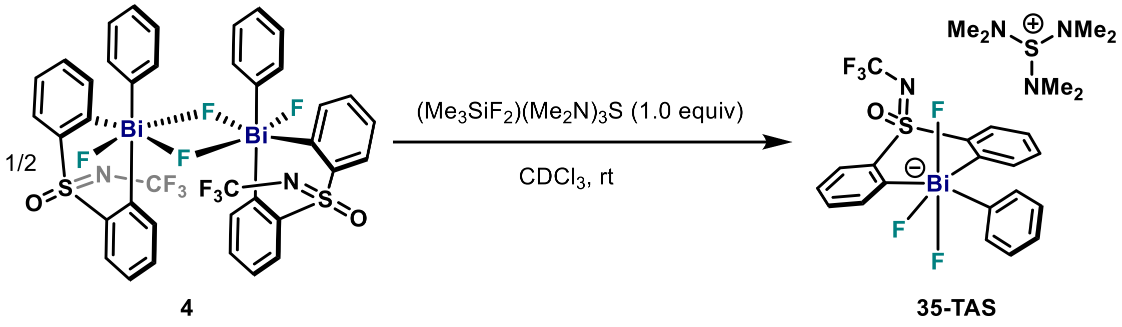

In scintillation vial, difluorophenylbismine **4** (30.4 mg, 0.025 mmol, *dimer*) was mixed with tris(dimethylamino)sulfonium difluorotrimethylsilicate (TASF, 13.8 mg, 1.0 equiv respect to monomeric species). The vial was capped and after evacuation and refilling with argon, dry CH_2Cl_2 (1 mL) was added. The mixture was stirred for 5 minutes, and the solvent was removed at the Schlenk line. The resulting solid was dried under high vacuum for 2 h, and the crude, which contained pure **35-TAS** after quantitative conversion of **4,** was analyzed by NMR and HRMS.

^{1}H NMR - (400 MHz, CD_2Cl_2, 213 K): δ 8.33 (d, *J* = 7.4 Hz, 2H), 8.12 (s, 2H), 8.02 (d, *J* = 7.5 Hz, 2H), 7.60 (t, *J* = 7.7 Hz, 2H), 7.56 (t, *J* = 7.7 Hz, 2H), 7.48 – 7.41 (m, 3H), 2.76 (s, 18H).

^{13}C NMR - (101 MHz, CD_2Cl_2, 213 K): δ 172.9 (q, *J* = 20.1 Hz), 164.9 (q, *J* = 21.4 Hz), 146.0, 135.4, 134.3, 133.0, 130.0, 129.9, 129.5, 127.2, 121.7 (q, *J* = 252.4 Hz), 38.7.

^{19}F NMR - (376 MHz, CD_2Cl_2, 183 K): δ –41.6 (s, 3F), –69.9 (bs, 1F), –96.7 (d, *J* = 86.2 Hz, 1F), –107.7 (d, *J* = 84.2 Hz, 1F).

HRMS (ESIneg, m/z): calc'd for $C_{19}H_{13}BiF_6SON^-$ [M–$(Me_2N)_3S$]$^-$ 626.0431 found 626.0438.

5. Reductive elimination from fluorobismuthonium σ-aryl Bi(V) fluoride complexes

5.1 Synthesis and characterization of **37**

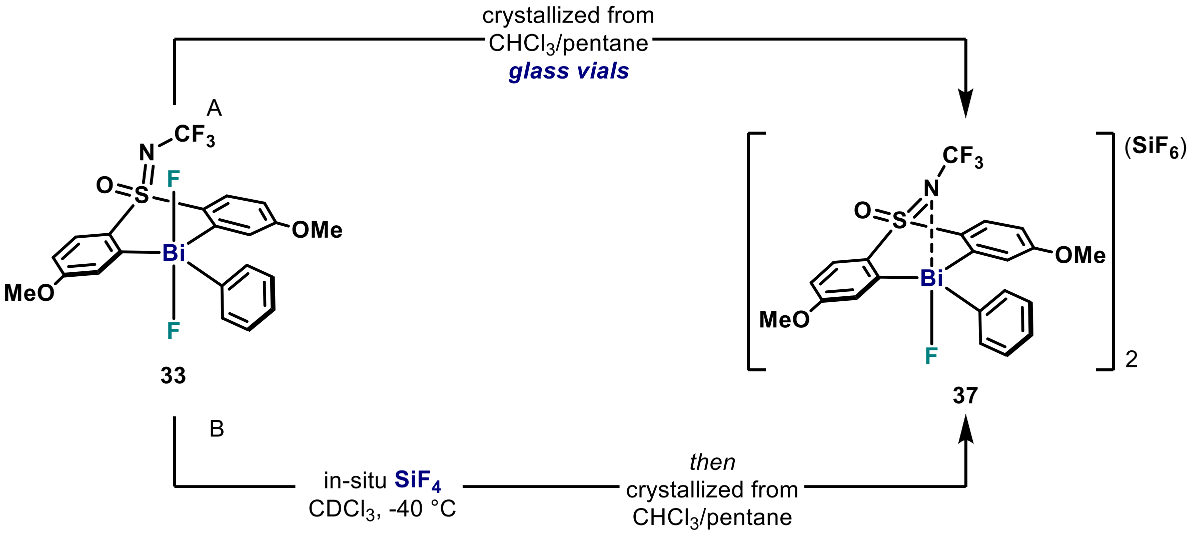

As mentioned in the main text, compound **37** was obtained via two routes. On one hand, *Route A* furnished fluorobismuthonium compound **37** in a serendipitous way, which consists on the crystallization of **33** in a $CDCl_3$/pentane solution over a week in glass vials. On the other hand, *route B* was developed to reproduce the results obtained through route A. Thus, **33** (70 mg, 0.11 mmol) was dissolved in a $CDCl_3$ solution and the mixture was cooled to –40 °C with a MeCN/N_2(l) bath. In a separate vial, dry $BaSiF_6$ was heated with a flame (ca. 400 °C), and the vapors were bubbled through the mixture of **33** in $CDCl_3$ during 10 min, keeping the mixture at –40 °C. Then, the crude was filtered and crystallized by slow evaporation of pentane at the freezer (–20 °C) during two days, obtaining pale yellow crystals corresponding to **37** (21 mg, 0.015 mmol, 27%).

^{1}H NMR - (500 MHz, CD_3CN, 273 K): δ 8.31 (d, *J* = 8.7 Hz, 2H), 7.97 (d, *J* = 8.9 Hz, 4H), 7.65 (q, *J* = 7.0, 6.6 Hz, 3H), 7.21 (dd, *J* = 8.8, 2.3 Hz, 2H), 3.88 (s, 6H).

^{13}C NMR - (125 MHz, CD_2Cl_2, 273 K): δ 166.4, 162.1, 157.8, 135.2, 134.3, 134.1, 133.4, 131.8, 124.8, 121.8 (q, *J* = 252.4 Hz), 116.3, 57.2.

^{19}F NMR - (470 MHz, CD_2Cl_2, 273 K): δ –41.6 (s, 3F). Signals corresponding to the SiF_6^{2-} anion and the Bi–F moiety were not detected.

HRMS (ESI, m/z): calc’d for $C_{21}H_{17}BiF_4SO_3N^-$ [M–($MSiF_6$)]$^-$ 648.0664 found 648.0662.

5.2 Reductive elimination from **37**

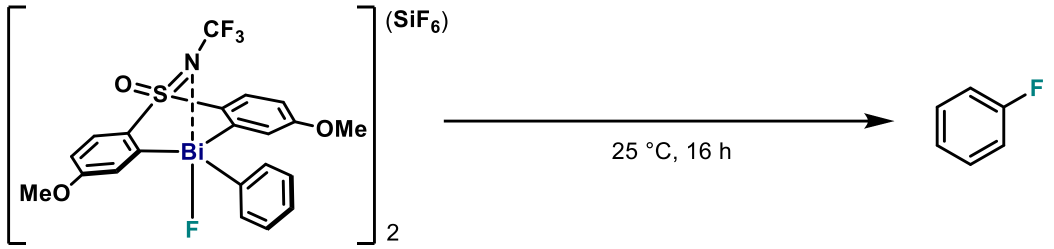

In a scintillation vial, **37** (14 mg, 0.01 mmol) was mixed with anhydrous $CDCl_3$ (0.5 mL) and the mixture was stirred 16 h at room temperature. Then, 1-fluoro-4-nitrobenzene was added as internal standard (1.0 equiv, 0.025 mmol, 2.65 μL) and the reaction crude was analyzed by ^{19}F NMR to obtain <5% yield of fluorobenzene. The same reaction was performed under the same conditions at 60 °C, obtaining 31 % of fluorobenzene together with decomposition byproducts presumably coming from **37**. When the reaction was performed in CD_3CN, a more suitable solvent for this ionic complexes, <5% fluorobenzene was detected both at rt or 60 °C due to possible hydrolysis caused by adventitious water or by strong coordination of CD_3CN, leading to deactivation of the reductive elimination event.

5.3 Reactivity of **4** with Lewis acids

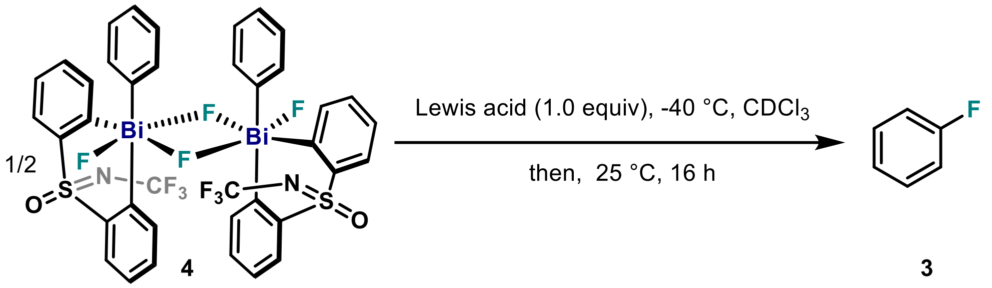

4 3

In a scintillation vial, pentavalent bismine **4** (15.2 mg, 0.013 mmol, *dimer*) was mixed with anhydrous $CDCl_3$ (0.5 mL) and the mixture was cooled to –40 °C in a MeCN/N_2(l) bath. Then, the corresponding Lewis acid (0.025 mmol, 1.0 equiv respect to monomeric species) was added, the reaction was warmed to room temperature and stirred overnight. Then, 1-fluoro-4-nitrobenzene was added as internal standard (1.0 equiv, 0.025 mmol, 2.65 μL) and the reaction crude was analyzed by ^{19}F NMR to obtain the yield of fluorobenzene.

Table S18. Reductive elimination from **4** in presence of a variety of Lewis acids.

Lewis acid	Yield of fluorobenzene[a]
$BF_3 \cdot OEt_2$	89%
$B(C_6F_5)_3$	43% + decomposition
SbF_5	<5% + decomposition
$(SiEt_3)(B(C_6F_5)_4)$	<5% + decomposition

[a]Yield determined by ^{19}F NMR using 1-Fluoro-4-nitrobenzene as internal standard.

5.4 Hammett (ligand backbone) from cationic Bi(V) complexes

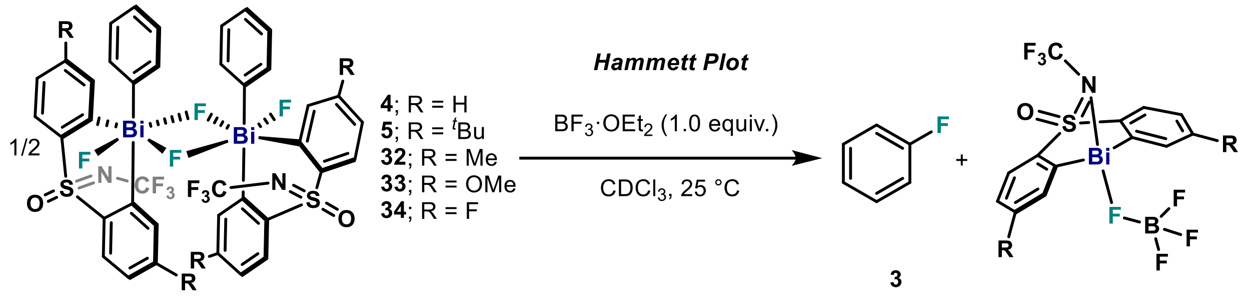

In a J-Young NMR tube, pentavalent bismine **4**, **5**, **32-34** (0.013 mmol, *dimer*) was mixed with anhydrous $CDCl_3$ (0.5 mL) and 1-fluoro-4-nitrobenzene was added as internal standard (1.0 equiv respect to monomeric species, 0.025 mmol, 2.65 μL). The mixture was heated to 25 °C inside the NMR probe, and the reaction was monitored by ^{1}H and ^{19}F NMR over 16 h. Kinetic constants were obtained by plotting the Napierian logarithm (ln) of concentrations of the corresponding pentavalent bismine species versus time (at least 20% conversion). The corresponding tetrafluoroboratebismines after reductive elimination were not characterized/isolated.

Table S19. Data corresponding to the Hammett plot for the thermal decomposition of **4**, **5**, **32-34** vs σ_m.

m-X (bismine)	k_X	k_X/k_H	$\log(k_X/k_H)$	σ_m
F (**34**)	0.0029900	8.5899	0.9339	0.34
H (**4**)	0.0003481	1	0	0
Me (**32**)	0.0000920	0.2643	–0.5779	–0.06
^{t}Bu (**5**)	0.0000478	0.1373	–0.8624	–0.10
OMe (**33**)	0.0000827	0.2378	–0.6238	0.1

Table S20. Data corresponding to the Hammett plot for the thermal decomposition of **4**, **5**, **32-34** considering resonance effects with the equation developed by Taft with $\lambda = 0.96$, $\rho_I = 4.82$ and $\rho_R = 4.62$.[20-22]

p-X (bismine)	k_X	k_X/k_H	$\log(k_X/k_H)$	σ_I	σ_R^0	σ_{bar}
F (**34**)	0.0029900	8.5899	0.9339	0.51	–0.34	0.188
H (**4**)	0.0003481	1	0	0	0	0
Me (**32**)	0.0000920	0.2643	–0.5779	–0.05	–0.10	–0.149
^{t}Bu (**5**)	0.0000478	0.1373	–0.8624	–0.06	–0.12	–0.178
OMe (**33**)	0.0000827	0.2378	–0.6238	0.26	–0.41	–0.136

Table S21. Data corresponding to the Hammett plot for the thermal decomposition of **4**, **5**, **32-34** considering resonance effects with the equation developed by Swain and Lupton with $f = 0.7$ and $r = 0.3$.[18-19]

p-X (bismine)	k_X	k_X/k_H	$\log(k_X/k_H)$	F	R	$\sigma_{S\text{-}L}$
F (**34**)	0.0029900	8.5899	0.9339	0.74	–0.60	0.338
H (**4**)	0.0003481	1	0	0	0	0
Me (**32**)	0.0000920	0.2643	–0.5779	–0.01	–0.41	–0.130
^{t}Bu (**5**)	0.0000478	0.1373	–0.8624	–0.11	–0.29	–0.164
OMe (**33**)	0.0000827	0.2378	–0.6238	0.54	–1.68	–0.126

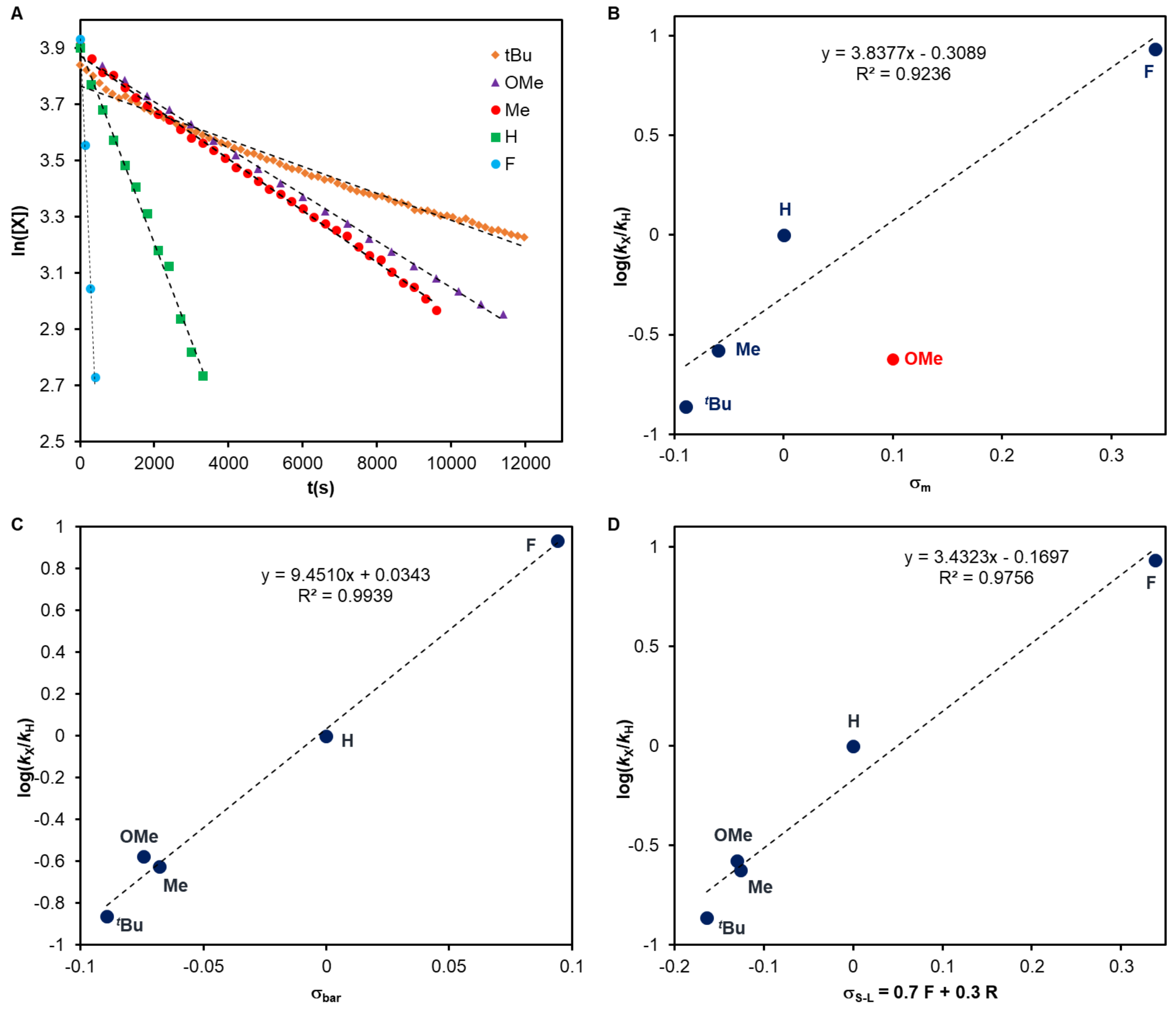

Figure S51. A. Determination of k_{obs} for the thermal decay of *para*-substituted arylbismines (**4**, **5**, **32-34**) at 25 °C after addition of $BF_3{\cdot}OEt_2$ in $CDCl_3$ (50 mM solution). **B.** Hammett plot for **4**, **5**, **32** and **34** ($\rho = 3.84$, $R^2 = 0.92$) *vs* σ_m, excluding pentavalent bismine **33** (X = OMe, red). **C.** Hammett plot for **4**, **5**, **32-34** ($\rho = 9.45$, $R^2 = 0.99$) considering resonance effects with the equation developed by Taft.[20-22] **D.** Hammett plot for **4**, **5**, **32-34** ($\rho = 3.43$, $R^2 = 0.98$) considering resonance effects with the equation developed by Swain-Lupton.[18-19]

5.5 Hammett (pendant aryl) from cationic Bi(V) complexes

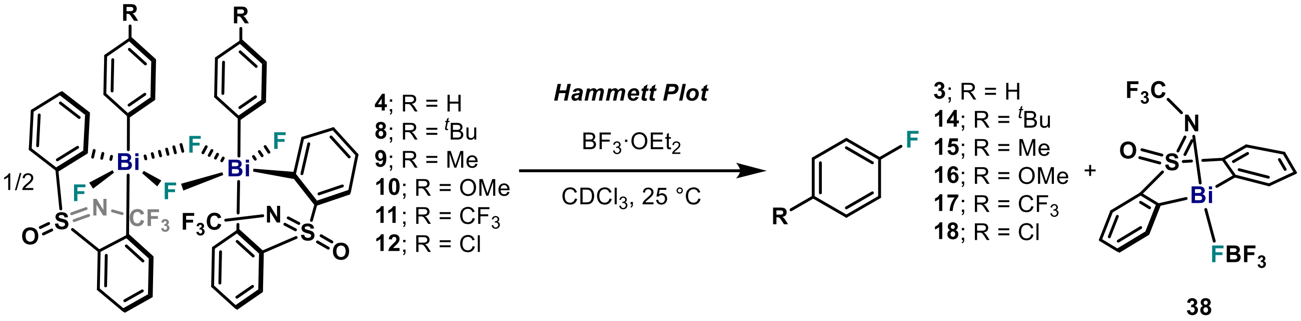

In a J-Young NMR tube, pentavalent bismine **4**, **8-12** (0.025 mmol) was mixed with anhydrous $CDCl_3$ (0.5 mL) and 1-fluoro-4-nitrobenzene was added as internal standard (1.0 equiv, 0.025 mmol, 2.65 μL). Then, $BF_3{\cdot}OEt_2$ (1.0 equiv respect to monomeric species, 0.025 mmol, 3.10 μL) was added at 0 °C and stirred for 10 min. The mixture was heated to 25 °C inside the NMR probe, and the reaction was monitored by ^1H and ^{19}F NMR over 10 h. Kinetic constants were obtained by plotting the Napierian logarithm (ln) of concentrations of the corresponding pentavalent bismine species versus time (at least 20% conversion). When *p*-EWG were introduced in the aryl ring, no reaction was observed at 25 °C, even though formation of the corresponding fluorobismuthonium complexes was confirmed by NMR spectroscopy. Only moderate amounts of fluoroarenes (23% *p*-CF_3, 39% *p*-Cl) were detected after heating at 90 °C for 16 h.

Table S22. Data corresponding to the Hammett plot for the thermal decomposition of **4**, **8-12**.

p-X (bismine)	k_X	k_X/k_H	$\log(k_X/k_H)$	σ_p	σ_p^+
CF_3 (**11**)	No reaction	-	-	0.53	0.61
Cl (**12**)	No reaction	-	-	0.24	0.11
H (**4**)	0.0003068	1	0	0	0
Me (**9**)	0.0000871	0.2839	–0.08006	–0.17	–0.31
^{t}Bu (**8**)	0.0001112	0.3625	–0.08006	–0.2	–0.26
OMe (**10**)	0.0000031	0.0100	–0.36004	–0.27	–0.78

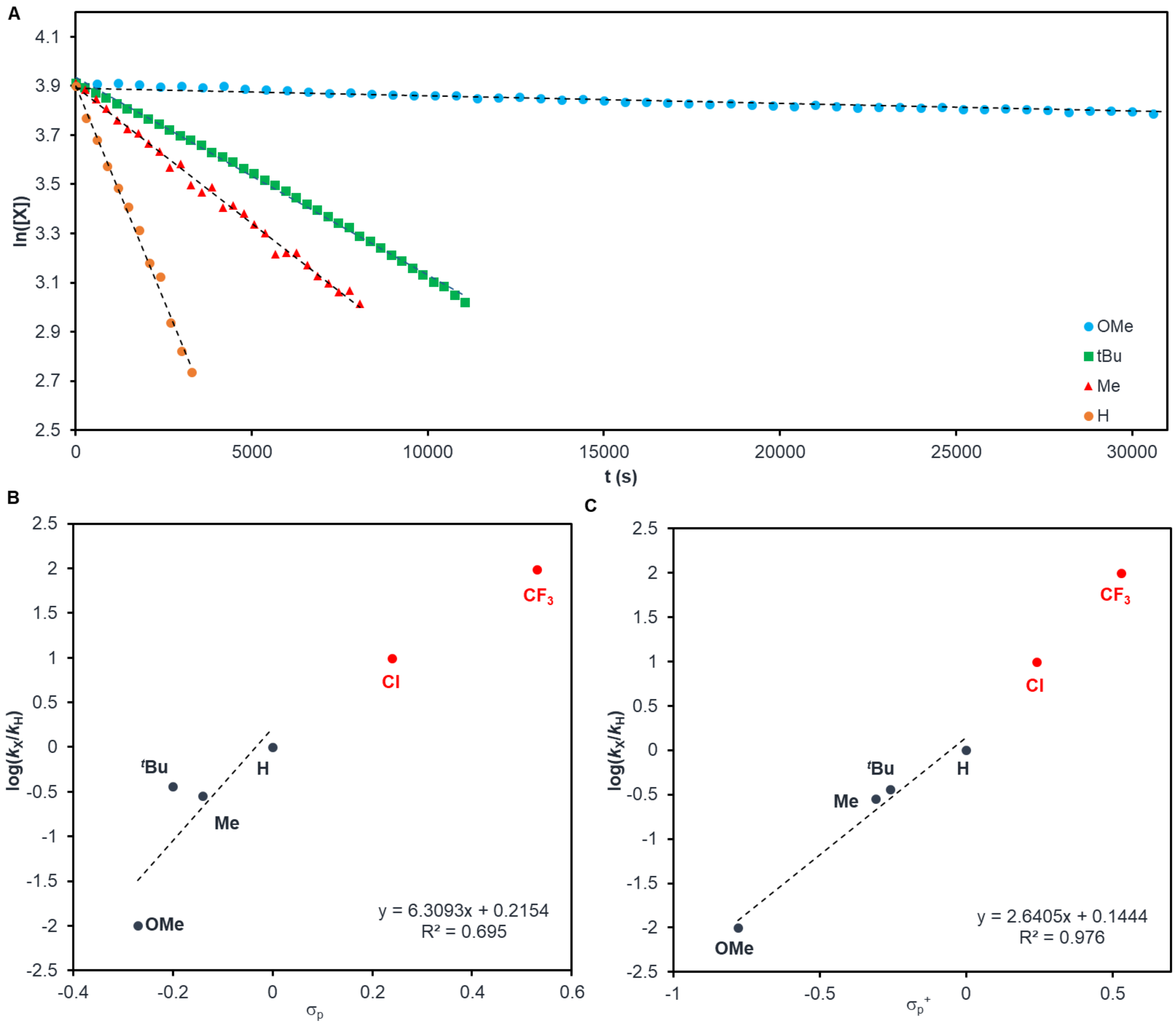

Figure S52. A. Determination of k_{obs} for the thermal decay of *para*-substituted arylbismines (**4**, **8-12**) at 25 °C after addition of $BF_3{\cdot}OEt_2$ in $CDCl_3$ (50 mM solution). **B.** Hammett plot for **4**, **8-12** (ρ = 6.30, R^2 = 0.70). **C.** Hammett plot for **4**, **8-12** considering resonance effects (ρ = 2.64, R^2 = 0.98).

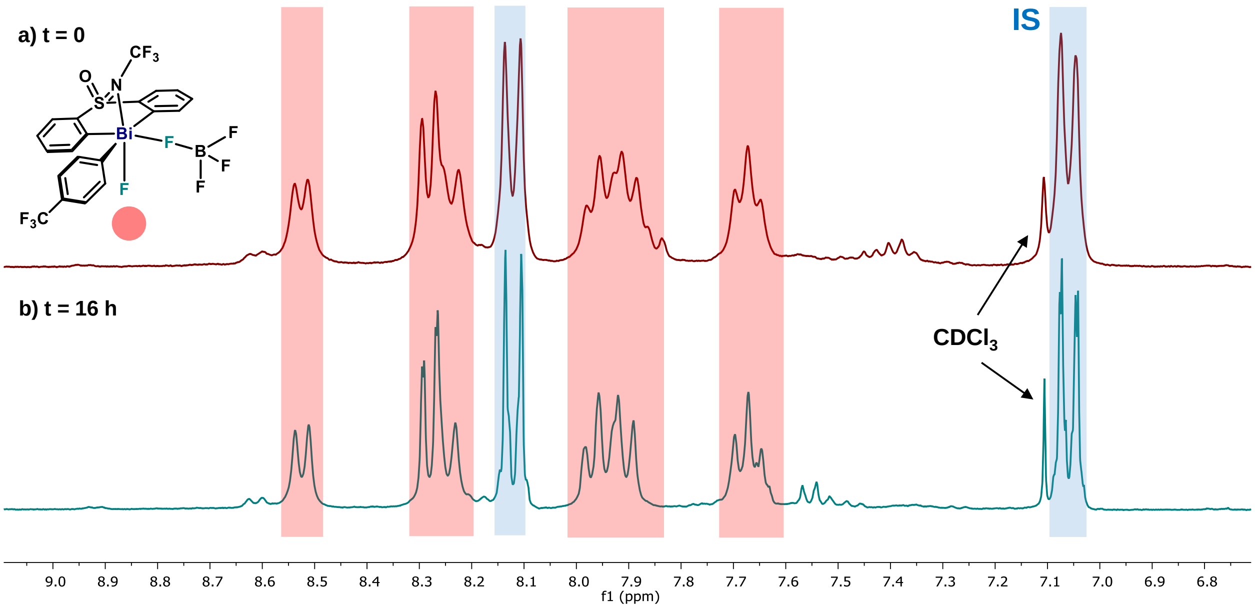

Figure S53. ^{1}H NMR measurements (aromatic region) of crude reactions of species **11** with $BF_3 \cdot OEt_2$ in $CDCl_3$ at 25 ºC after addition (top, t = 0) and after 16 h of measurements (bottom).

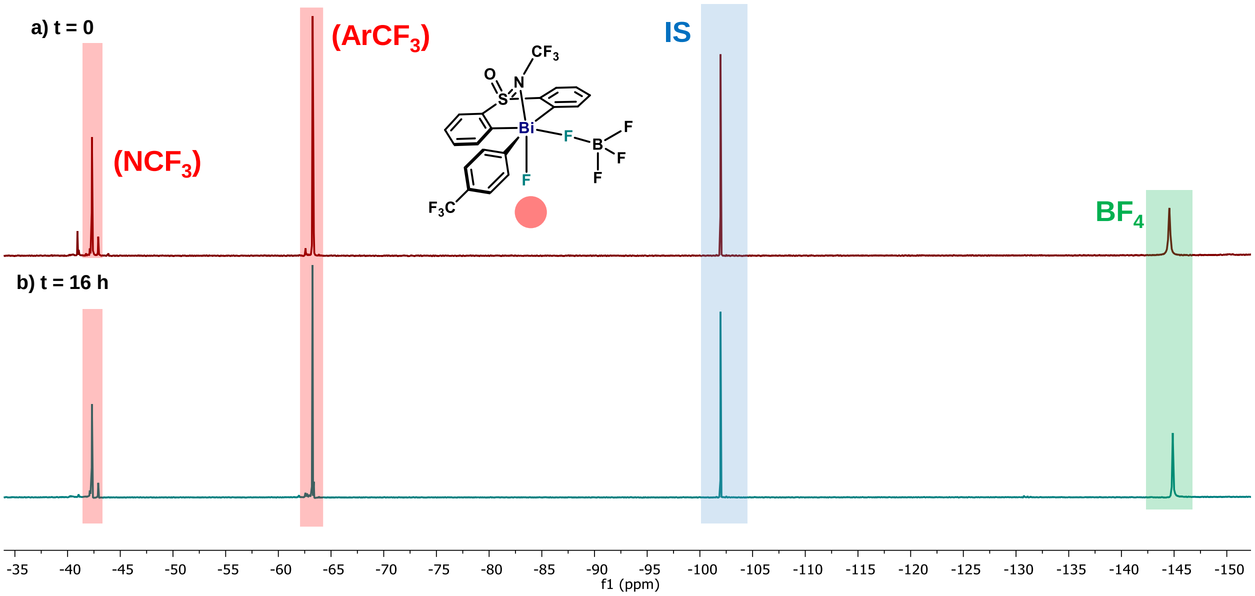

Figure S54. ^{19}F NMR measurements (aromatic region) of crude reactions of species **11** with $BF_3 \cdot OEt_2$ in $CDCl_3$ at 25 ºC after addition (top, t = 0) and after 16 h of measurements (bottom).

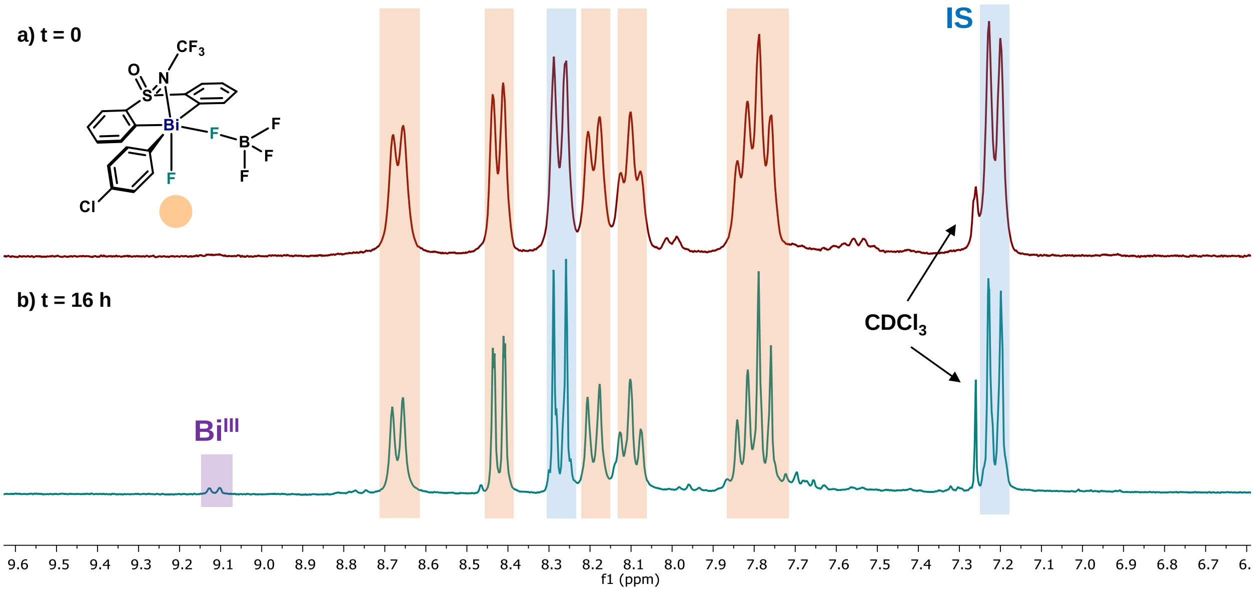

Figure S55. ^{1}H NMR measurements (aromatic region) of crude reactions of species **12** with $BF_3 \cdot OEt_2$ in $CDCl_3$ at 25 ºC after addition (top, t = 0) and after 16 h of measurements (bottom).

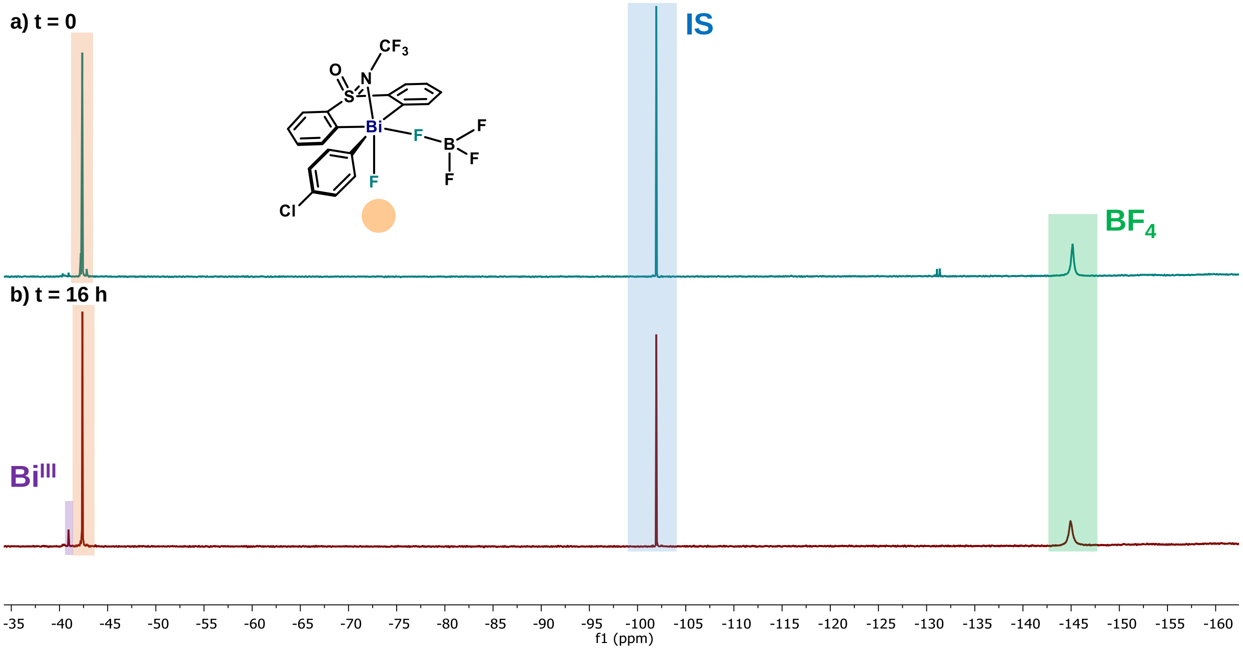

Figure S56. ^{19}F NMR measurements (aromatic region) of crude reactions of species **12** with $BF_3 \cdot OEt_2$ in $CDCl_3$ at 25 ºC after addition (top, t = 0) and after 16 h of measurements (bottom).

5.5.1 Synthesis of *cationic* pentavalent bismine **179**

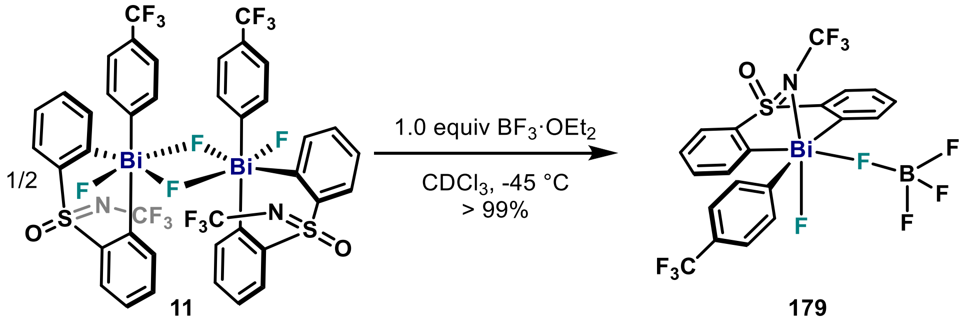

Pentavalent bismine **11** (33 mg, 0.025 mmol, *dimer*) was mixed with anhydrous $CDCl_3$ (2 mL) at –45°C (MeCN/N_2(l) bath). To the suspension, BF_3·OEt_2 complex (6.2 µL, 0.05 mmol, 1.0 equiv to monomeric species) was added and a clear solution was obtained. The solution was stirred for 10 min at –45 °C and **179** was fully characterized by NMR (at –40 °C) and HRMS.

^{1}H NMR - (500 MHz, $CDCl_3$, 233.15 K): δ 8.64 (d, *J* = 7.7 Hz, 2H), 8.44 (d, *J* = 7.7 Hz, 2H), 8.40 (d, *J* = 8.0 Hz, 2H), 8.12 (t, *J* = 7.5 Hz, 2H), 8.07 (d, *J* = 7.9 Hz, 2H), 7.85 (t, *J* = 7.6 Hz, 2H). Peaks at 4.23, 3.46, 1.41 and 1.22 ppm correspond to residual Et_2O and BF_3·OEt_2 in excess.

^{11}B NMR - (160 MHz, $CDCl_3$, 233.15 K): δ –0.87 (**B**F_4). Peak at δ = 0.01 ppm corresponds to excess BF_3·OEt_2.

^{19}F NMR - (470 MHz, $CDCl_3$, 233.15 K): δ –42.3 (s, 3F, NC**F**$_3$), –62.9 (s, 3F, C**F**$_3$), –144.6 (bs, 4F, B**F**$_4$), –162.2 (brs, 1F, Bi-**F**). Peak at –153.4 ppm correspond to residual BF_3/BF_3·OEt_2 species.

^{13}C NMR - (126 MHz, $CDCl_3$, 233.15 K): δ 163.8, 161.1, 141.5, 138.5, 135.4, 134.0, 133.2, 130.5, 129.3, 127.7, 122.7 (q, *J* = 272.1 Hz, **C**F_3), 120.6 (q, *J* = 269.0 Hz, **C**F_3).

HRMS (ESI, m/z): calc'd for $C_{20}H_{12}BiF_7NSO^+$ [M–(BF_4)]$^+$ 656.0326; found 656.0318.

5.6 Reductive elimination from **180**

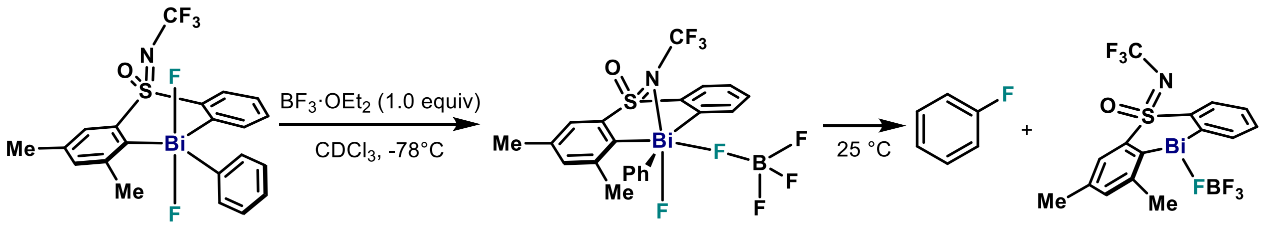

25 **180** **3** **181**

In a J-Young NMR tube, pentavalent bismine **25** (15.7 mg, 0.025 mmol) was mixed with anhydrous $CDCl_3$ (0.5 mL) and 1-fluoro-4-nitrobenzene was added as internal standard (1.0 equiv, 0.025 mmol, 2.65 μL). The mixture was cooled down to –78 °C and $BF_3{\cdot}OEt_2$ (2.65 μL, 0.025 mmol) was added with a Hamilton syringe, thus forming compound **180** in situ. Then, the reaction was shaken and inserted into the NMR probe, the temperature of which was set at 25 °C. The reaction was monitored by ^{1}H and ^{19}F NMR over 10 h. Kinetic constants were obtained by plotting the Napierian logarithm (ln) of initial concentrations of the corresponding pentavalent bismine species after reaching (at least) 50% conversion versus time. After completion, fluorobenzene (**3**) was obtained in 91% yield, together with signals corresponding to tetrafluoroboratebismine **181**, which was not further characterized.

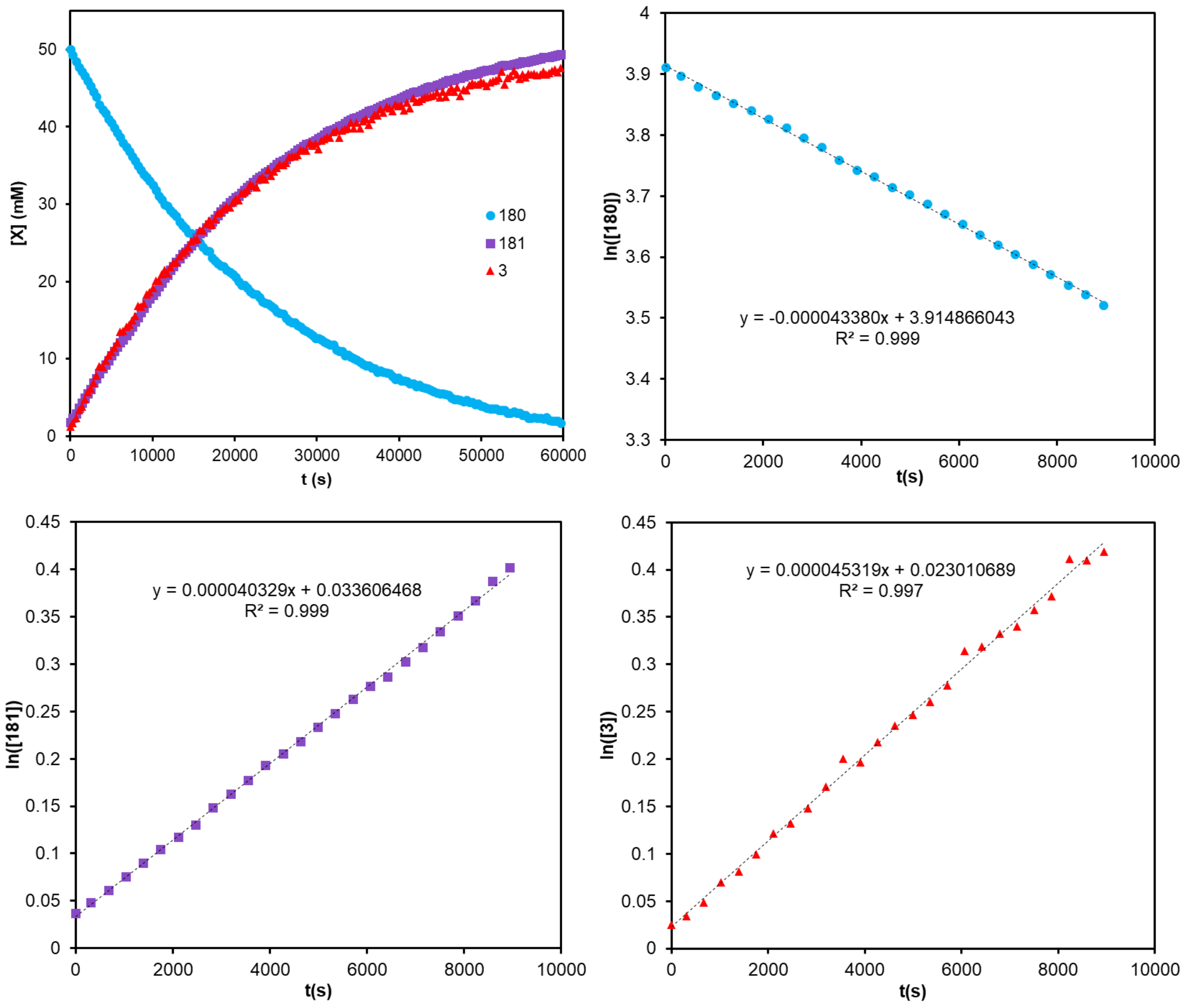

Figure S57. A. Kinetic profile of the thermal decay of in situ generated **180** in $CDCl_3$ at 25 °C (50 mM). **B.** Determination of k_{obs} for the thermal decay of **180** (ln([**180**]) vs t (s)). **C.** Determination of k_{obs} for the formation of **181** vs t (s)). **D.** Determination of k_{obs} for the formation of **3** vs t (s)).

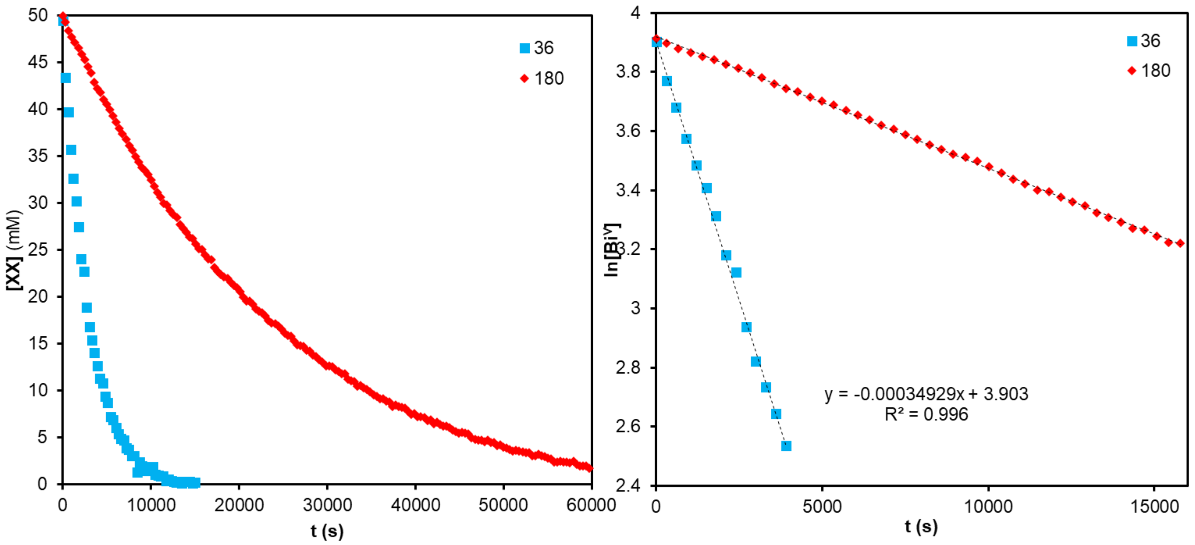

Figure S58. *Left*: Kinetic profile of the thermal decay of in situ generated **36** (blue) and **180** (red) in $CDCl_3$ at 25 °C (50 mM). *Right:* Determination of k_{obs} for the thermal decay of **36** (blue) and **180** (red) (ln([**36** or **180**]) vs t (s)).

5.6.1 Synthesis of *cationic* pentavalent bismine **180**

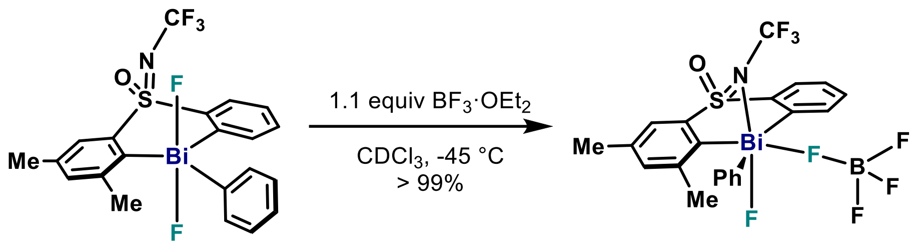

25 **180**

Pentavalent bismine **25** (32 mg, 0.05 mmol) was mixed with anhydrous $CDCl_3$ (1 mL) at –45°C (MeCN/N_2(l) bath). To the suspension, BF_3·OEt_2 complex (6.2 µL, 0.05 mmol) was added and a clear solution was obtained. The solution was stirred for 10 min at –45 °C and **180** was fully characterized by NMR (at –40 °C) and HRMS.

^{1}H NMR - (400 MHz, $CDCl_3$, 223.15 K): δ 8.92 (d, *J* = 8.1 Hz, 1H), 8.40 (d, *J* = 7.6 Hz, 1H), 8.21 (d, *J* = 8.3 Hz, 2H), 8.13 (t, *J* = 7.8 Hz, 1H), 8.09 (s, 1H), 7.84 (q, *J* = 7.2 Hz, 3H), 7.72 (t, *J* = 7.2 Hz, 1H), 7.63 (s, 1H), 2.49 (s, 3H), 2.44 (s, 3H). Peaks at 4.23, 3.46, 1.41 and 1.22 ppm correspond to residual Et_2O and BF_3·OEt_2 in excess.

^{11}B NMR - (128 MHz, $CDCl_3$, 223.15 K): δ –0.92 (**B**F_4). Peak at –0.1 ppm correspond to residual BF_3·OEt_2 in excess.

^{19}F NMR - (376 MHz, $CDCl_3$, 223.15 K): δ –42.4 (s, 3F, NC**F**$_3$), –146.5 (bs, 4F, B**F**$_4$), –161.1 (brs, 1F, Bi–**F**). Peak at –153.1 ppm corresponds to residual BF_3/BF_3·OEt_2 species.

^{13}C NMR - (100 MHz, $CDCl_3$, 223.15 K): δ 166.0 (d, *J* = 10.3 Hz), 158.0 (d, *J* = 6.2 Hz), 156.2 (d, *J* = 10.1 Hz), 146.6, 143.6, 141.6, 140.7, 139.5, 139.0, 135.1, 134.4, 134.2, 133.3, 133.0, 129.4, 126.7, 120.4 (t, *J* = 269.0 Hz, **C**F_3), 22.6, 21.7

HRMS (ESI, m/z): calc'd for $C_{21}H_{17}BiF_4NSO^+$ [M–(BF_4)]$^+$ 616.0765; found 616.0761.

5.7 Reductive elimination from **182**

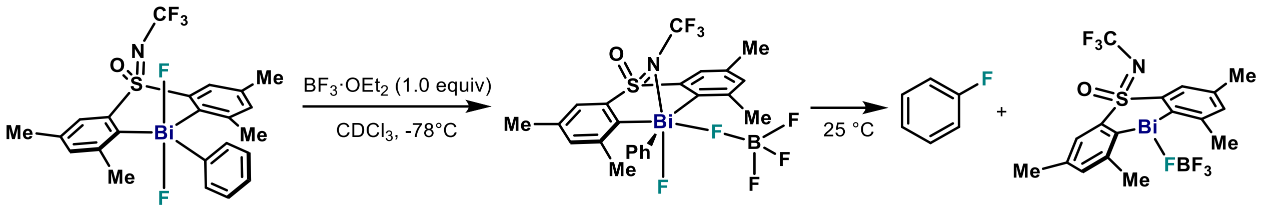

In a J-Young NMR tube, pentavalent bismine **26** (16.5 mg, 0.025 mmol) was mixed with anhydrous $CDCl_3$ (0.5 mL) and 1-fluoro-4-nitrobenzene was added as internal standard (1.0 equiv, 0.025 mmol, 2.65 µL). The mixture was cooled down to –78 °C and $BF_3·OEt_2$ (3.1 µL, 0.025 mmol) was added with a Hamilton syringe, thus forming compound **182** in situ. Then, the reaction was shaken and inserted into the NMR probe, the temperature of which was set at 25 °C. The reaction was monitored by ^{1}H and ^{19}F NMR over 10 h. After full conversion of species **182**, fluorobenzene (**3**) was obtained in 44% yield and decomposition was observed (see Figures S59 and S60).

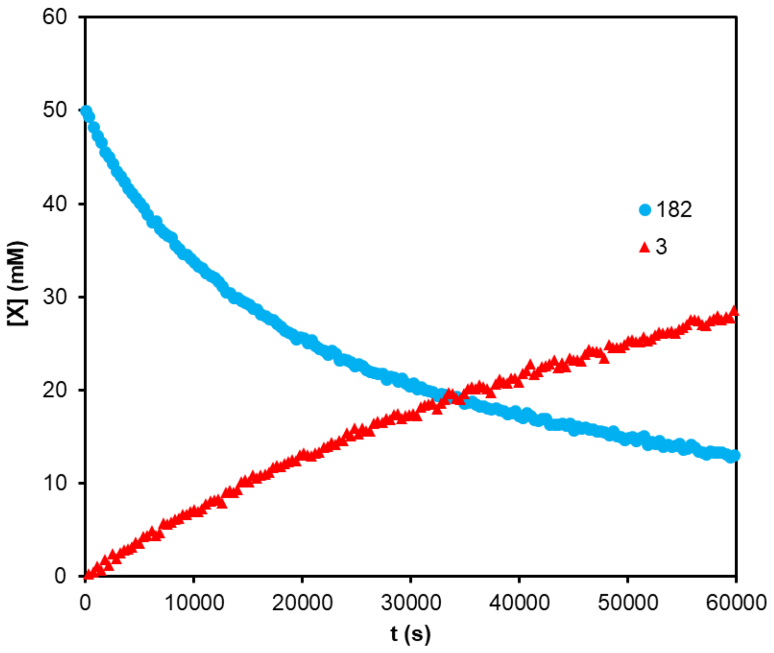

Figure S59. Kinetic profile of the thermal decay of in situ generated **182** in $CDCl_3$ at 25 °C (50 mM). Rate of decay of **182** does not match the rate of fluorobenzene (**3**) formation due to decomposition.

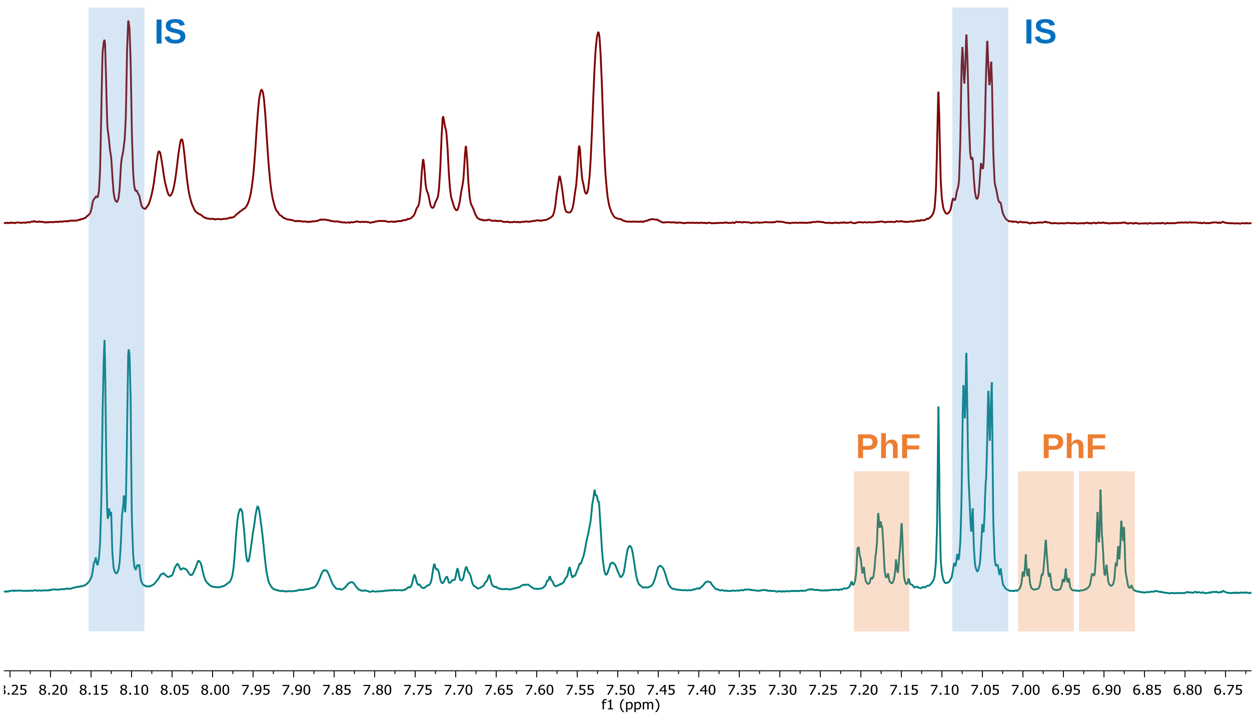

Figure S60. ^{1}H NMR measurements (aromatic region) of crude reactions of species **26** with $BF_3·OEt_2$ in $CDCl_3$ at 25 ºC after addition (top, t = 0) and after 16 h of measurements (bottom). After reaction, clear signs of decomposition can be observed together with the appearance of signals corresponding to fluorobenzene (orange).

5.7.1 Synthesis of *cationic* pentavalent bismine **182**

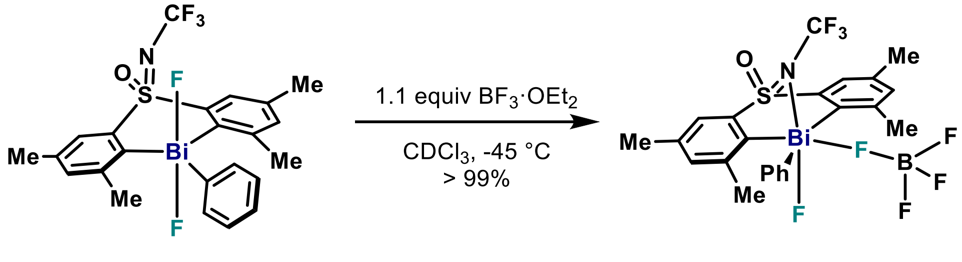

26 **182**

Pentavalent bismine **26** (33 mg, 0.05 mmol) was mixed with anhydrous $CDCl_3$ (1 mL) at –45°C (MeCN/N_2(l) bath). To the suspension, BF_3·OEt_2 complex (6.2 μL, 0.05 mmol) was added and a clear solution was obtained. The solution was stirred for 10 min at –45 °C and **182** was fully characterized by NMR (at –40 °C) and HRMS.

^{1}H NMR - (400 MHz, $CDCl_3$, 223.15 K): δ 8.11 (d, J = 8.2 Hz, 2H), 8.06 (s, 2H), 7.84 (t, J = 7.7 Hz, 2H), 7.68 (s, 3H), 2.66 (s, 6H), 2.48 (s, 6H). Peaks at 4.23, 3.46, 1.41 and 1.22 ppm correspond to residual Et_2O and BF_3·OEt_2 in excess.

^{11}B NMR - (128 MHz, $CDCl_3$, 223.15 K): δ –1.28 (**B**F_4). Peak at –0.1 ppm correspond to residual BF_3·OEt_2 in excess.

^{19}F NMR - (376 MHz, $CDCl_3$, 223.15 K): δ –43.2 (s, 3F, NC**F**$_3$), –146.9 (bs, 4F, B**F**$_4$), –165.6 (brs, 1F, Bi–**F**). Peaks at –150 - –155 ppm correspond to residual BF_3/BF_3·OEt_2 species.

^{13}C NMR - (101 MHz, $CDCl_3$, 223.15 K): δ 160.4 (d, *J* = 6.2 Hz), 159.4 (d, *J* = 10.2 Hz), 145.9, 143.8, 140.8, 140.6, 134.1, 133.9, 132.6, 127.0, 120.1 (q, *J* = 269.0 Hz, **C**F_3), 22.5 (d, *J* = 6.2 Hz), 21.6.

HRMS (ESI, m/z): calc'd for $C_{23}H_{21}BiF_4NSO^+$ [M–(BF_4)]$^+$ 644.1078; found 644.1082.

6. Reactivity with 1-fluoro-2,6-dichloropyridinium tetrafluoroborate (2)

6.1 Evidences for the formation of Bi(V) species with **2**

In our previous study,[2] formation of cationic intermediates after reaction of **1** with electrophilic fluoride source **2** was assessed by spectroscopic analysis of a 1:1 mixture in $CDCl_3$ at 60 ºC. Due to the low solubility of **2** in $CDCl_3$, conversion to the corresponding cationic species was very low. Due to this fact, small amounts of intermediate **36** or **184** were detected. This experiment was performed following the subsequent procedure.

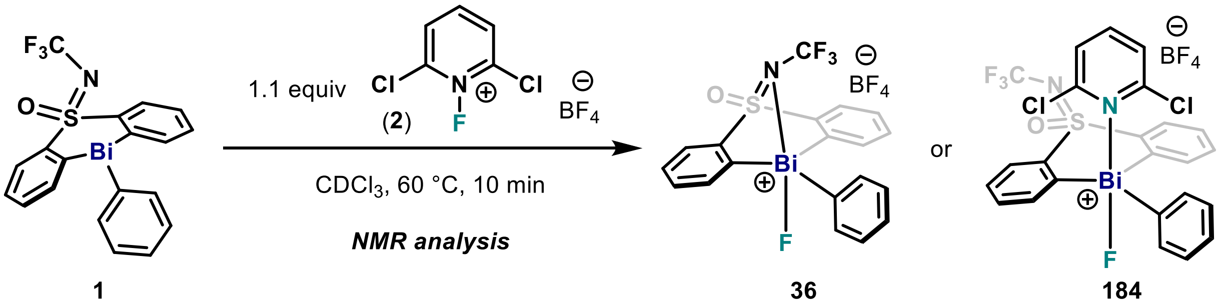

Phenylbismine **1** (14.2 mg, 0.025 mmol) and 1-fluoro-2,6-pyridinium tetrafluoroborate **2** (7.2 mg, 0.0275 mmol) were mixed in $CDCl_3$ under an Ar atmosphere and the reaction was stirred for 10 min at 60 °C. Then, the crude reaction mixture was analyzed by NMR. As shown in Figures S61 and S62, when **1** is reacted with **2** at 60 °C, peaks similar to species **36** can be detected (synthesized through reaction of species **4** with $BF_3{\cdot}OEt_2$), thus confirming the oxidation of the corresponding Bi(III) complex to a Bi(V) species similar to **36** or **184**.

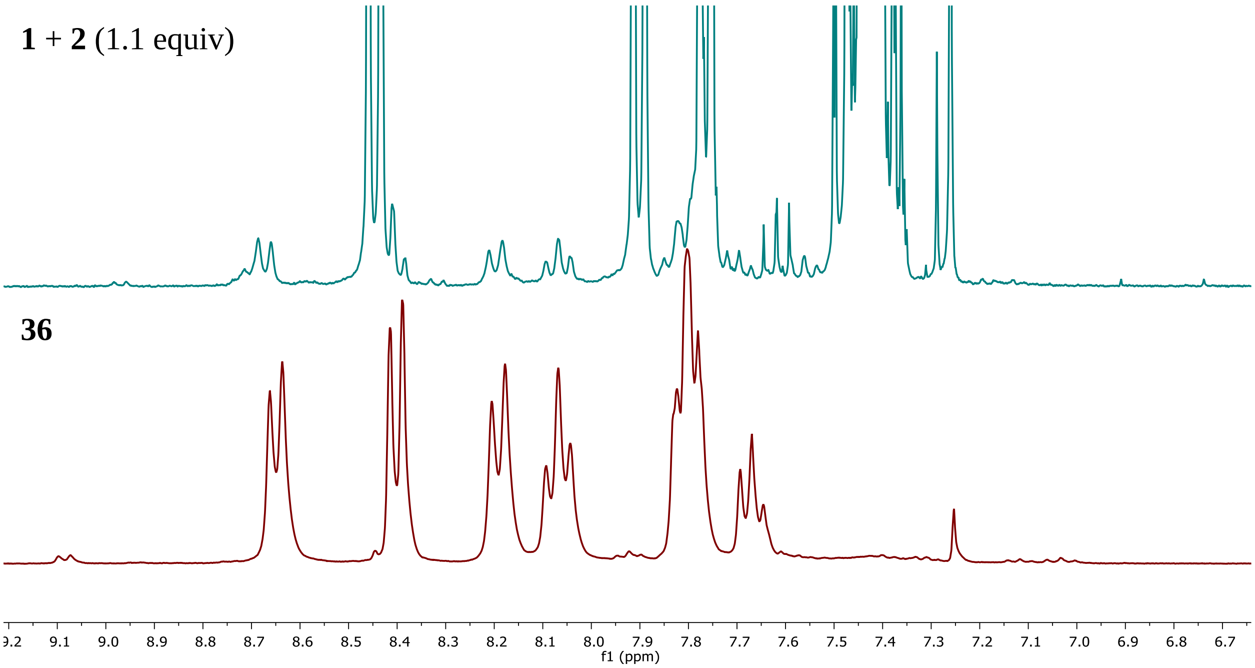

Figure S61. ^{1}H NMR of a mixture of **1** and **2** (1.1 equiv) after stirring at 60 °C for 10 min in $CDCl_3$ (top). ^{1}H NMR of pure **36** in $CDCl_3$ (bottom).

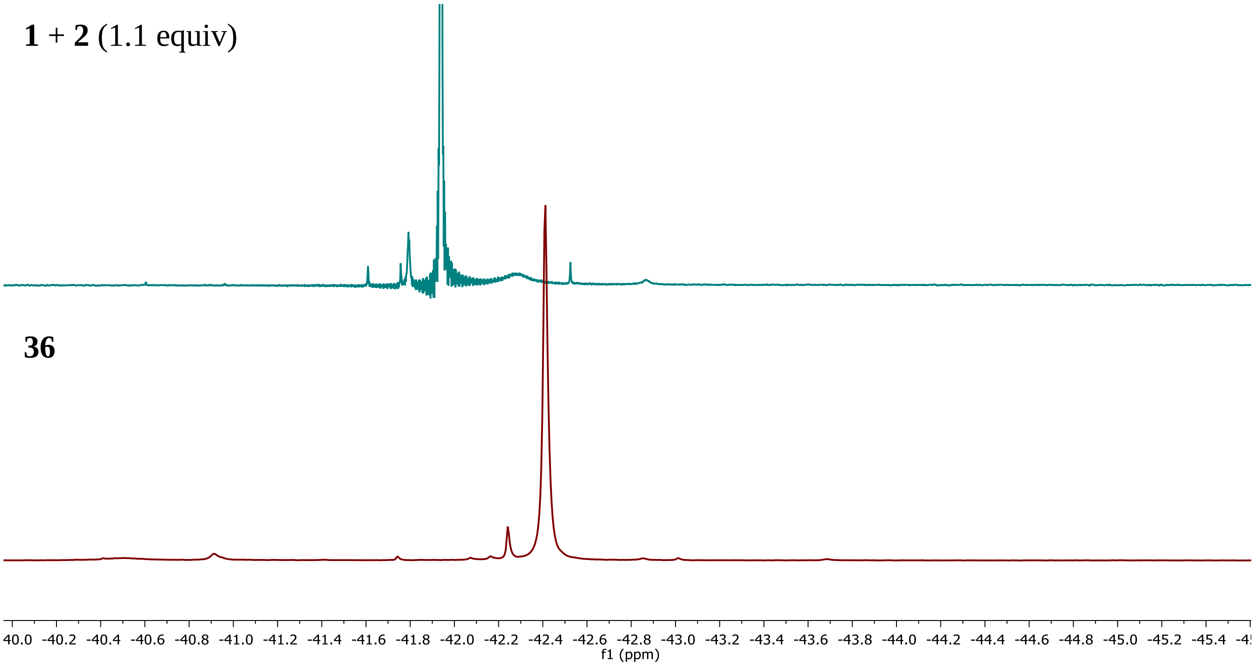

Figure S62. ^{19}F NMR of a mixture of **1** and **2** (1.1 equiv) after stirring at 60 °C for 10 min in $CDCl_3$ (top). ^{19}F NMR of **36** in $CDCl_3$ (bottom).

To solve the low solubility issue observed when using $CDCl_3$ as solvent and obtain further evidence of oxidation of **1** to a pentavalent Bi intermediate, we performed a set of experiments using CD_3CN. In this solvent, conversion to the corresponding cationic species was complete, and comparison with cationic intermediate **36** can be performed directly. These experiments were performed following the subsequent procedures.

(a) Reaction of **1** with electrophilic fluorinating agent **2** in CD_3CN

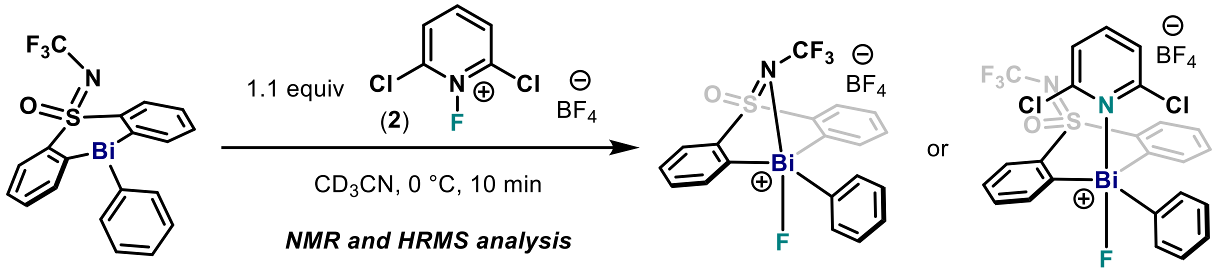

Phenylbismine **1** (14.2 mg, 0.025 mmol) and 1-fluoro-2,6-pyridinium tetrafluoroborate **2** (7.2 mg, 0.0275 mmol) were mixed in CD_3CN under an Ar atmosphere and the reaction was stirred for 10 min at 0 °C. Then, the crude reaction mixture was analyzed by NMR and HRMS (See Figures S63-S65). After analysis, the reaction mixture was heated to 60 °C and analyzed by NMR in presence of 1-fluoro-4-nitrobenzene, resulting in the formation of fluorobenzene in 59% yield.

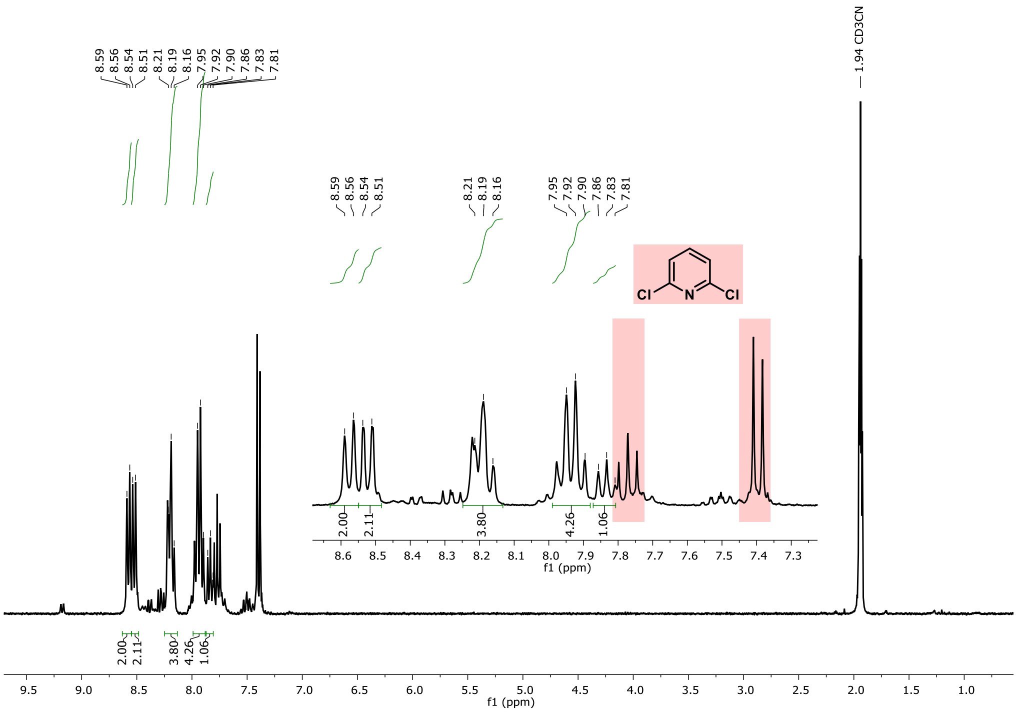

Figure S63. ^{1}H NMR of a mixture of **1** and **2** (1.1 equiv) after stirring at 0 °C for 10 min in CD_3CN.

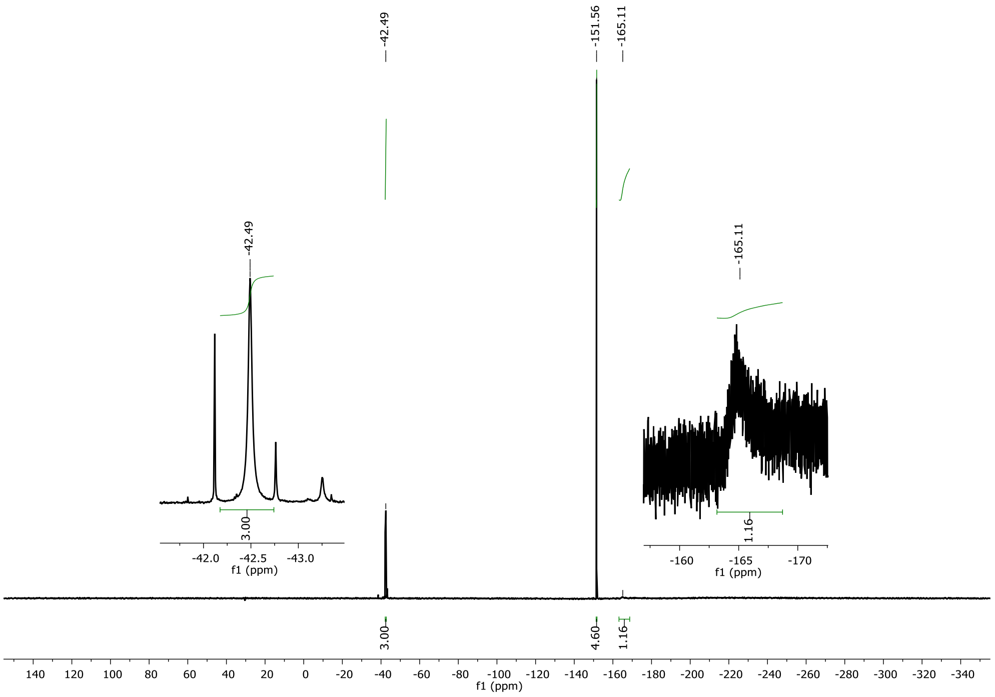

Figure S64. ^{19}F NMR of a mixture of **1** and **2** (1.1 equiv) after stirring at 0 °C for 10 min in CD_3CN.

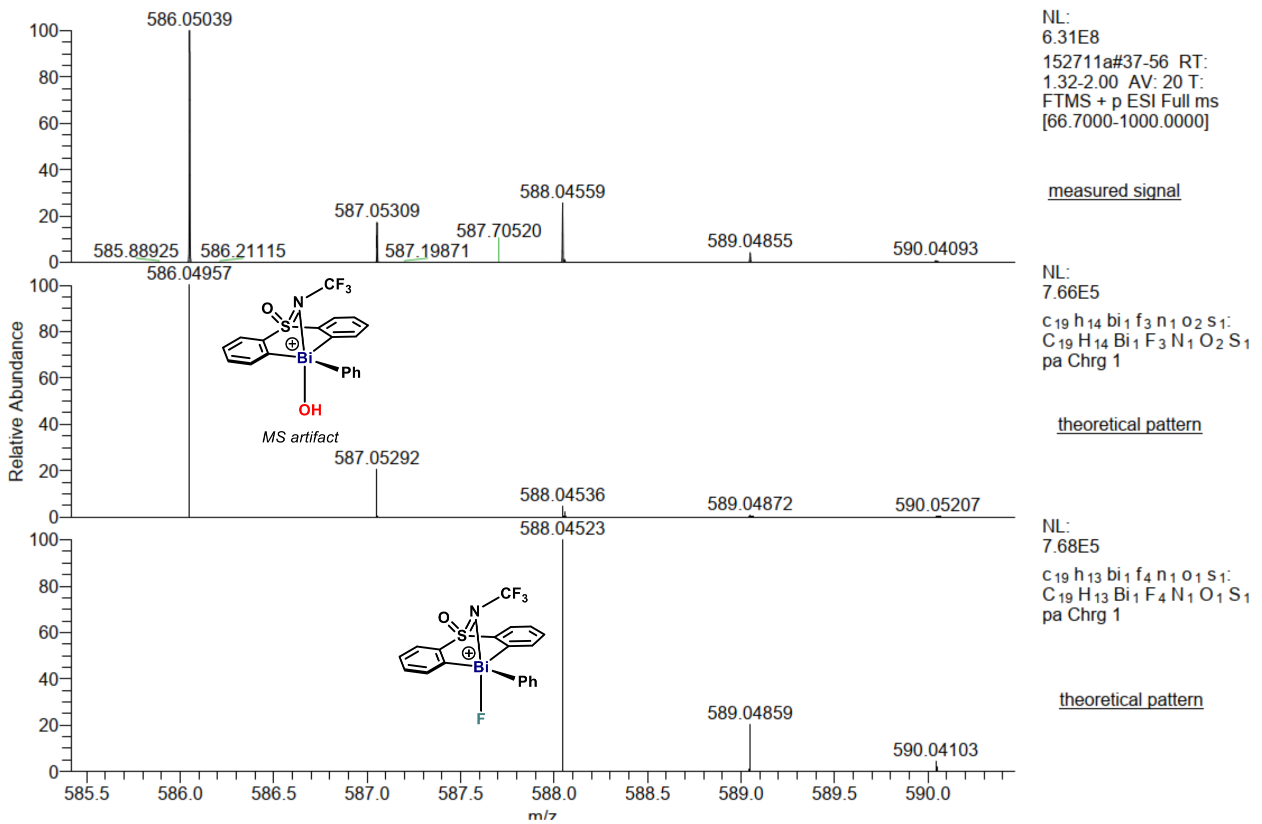

Figure S65. HRMS of a mixture of **1** and **2** (1.1 equiv) after stirring at 0 °C for 10 min in CD_3CN showing formation of cationic species.

(b) Reaction of **4** with $BF_3 \cdot OEt_2$ in presence of 2,6-dichloropyridine in CD_3CN

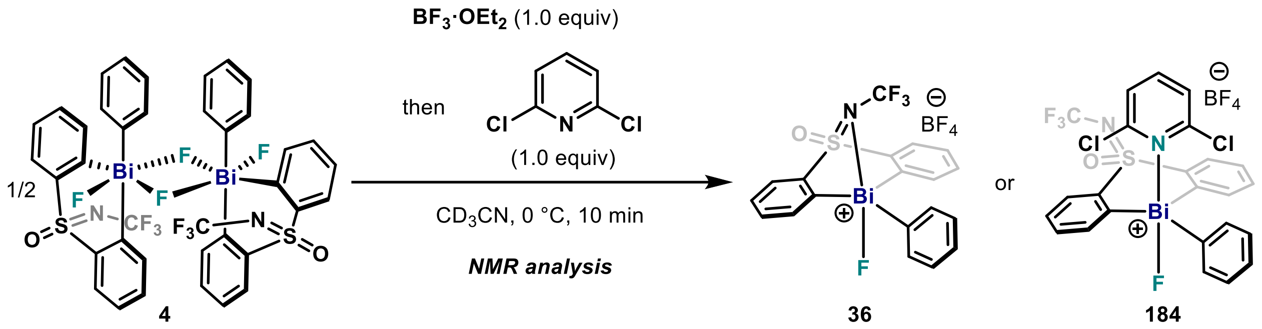

In a scintillation vial, pentavalent bismine **4** (15.2 mg, 0.013 mmol, *dimer*) was dissolved in CD_3CN under an Ar atmosphere and the mixture was cooled to 0 °C. Then, $BF_3 \cdot OEt_2$ (1.0 equiv respect to monomeric species, 3.1 μl, 0.025 mmol) was added together with 2,6-dichloropyridine (0.025 mmol, 3.7 mg) and the reaction was stirred for 5 min at 0 °C. Then, the crude was analyzed by NMR. As shown in Figures S66 and S67, when **4** is reacted with $BF_3 \cdot OEt_2$ and 2,6-dichloropyridine at 0 °C, peaks similar to species **36** can be detected (Figures S63 and S64), thus confirming the oxidation of the corresponding Bi(III) complex to a Bi(V) species similar to **36** or **184**.

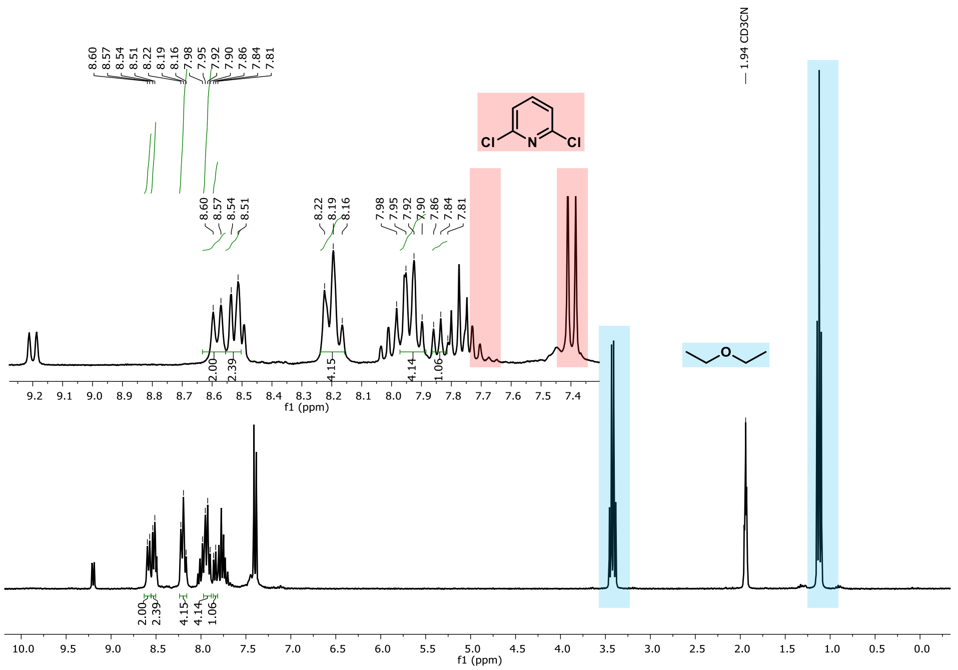

Figure S66. ^1H NMR of a mixture of **4**, $BF_3 \cdot OEt_2$ (1.0 equiv) and 2,6-dichloropyridine (1.0 equiv) after stirring at 0 °C for 10 min in CD_3CN.

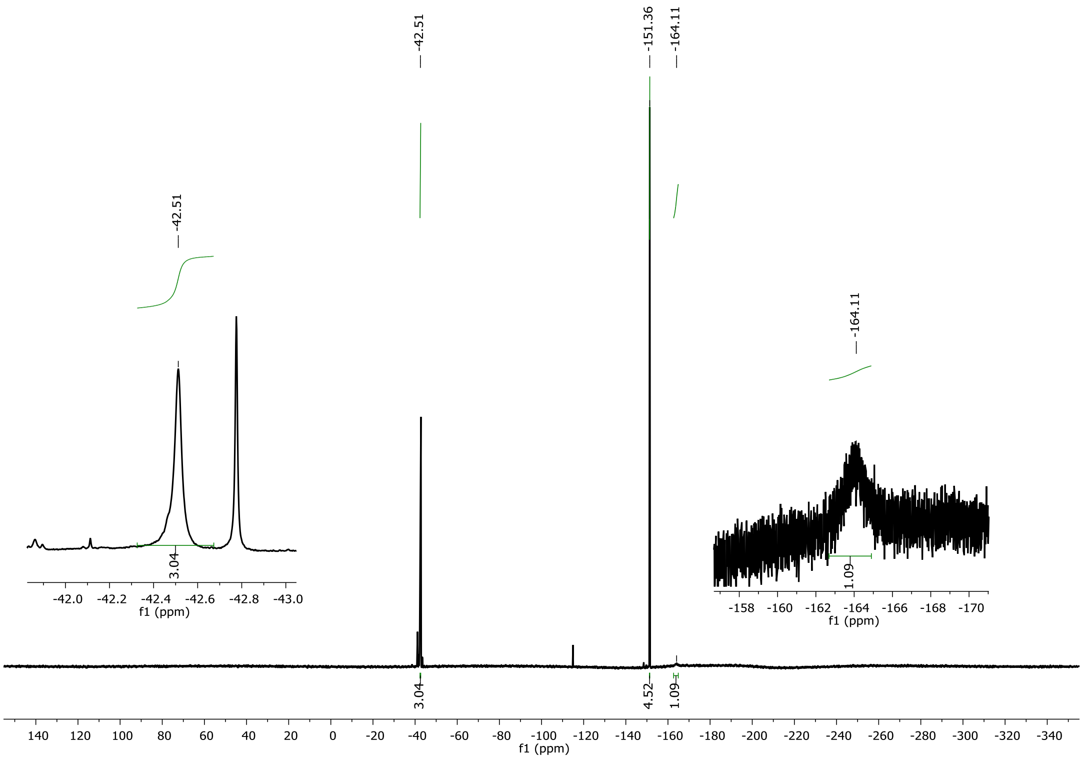

Figure S67. ^{19}F NMR of a mixture of **4**, $BF_3 \cdot OEt_2$ (1.0 equiv) and 2,6-dichloropyridine (1.0 equiv) after stirring at 0 °C for 10 min in CD_3CN. Signal at $\delta = -164.1$ ppm corresponds to the Bi–F unit.

6.2 Influence of the ligand scaffold in the oxidation/reductive elimination sequence

Under an Ar atmosphere, the corresponding trivalent phenylbismine (0.025 mmol) was weighted with **2** (1.1 equiv) in a scintillation vial, which was subsequently removed from the glovebox. Then, $CDCl_3$ (1 mL) was added under an Ar atmosphere. The vial was sealed and the reaction was stirred for 16 h at 90 °C. Then, 1-fluoro-4-nitrobenzene was added as internal standard (0.025 mmol, addition by weight) and the crude reactions were analyzed by ^{19}F NMR to determine the yield of fluorobenzene.

Table S23. Influence of the ligand substitution on the oxidation/reductive elimination sequence. In parenthesis, reaction performed in MeCN-d$_3$. Yields determined by ^{19}F NMR.

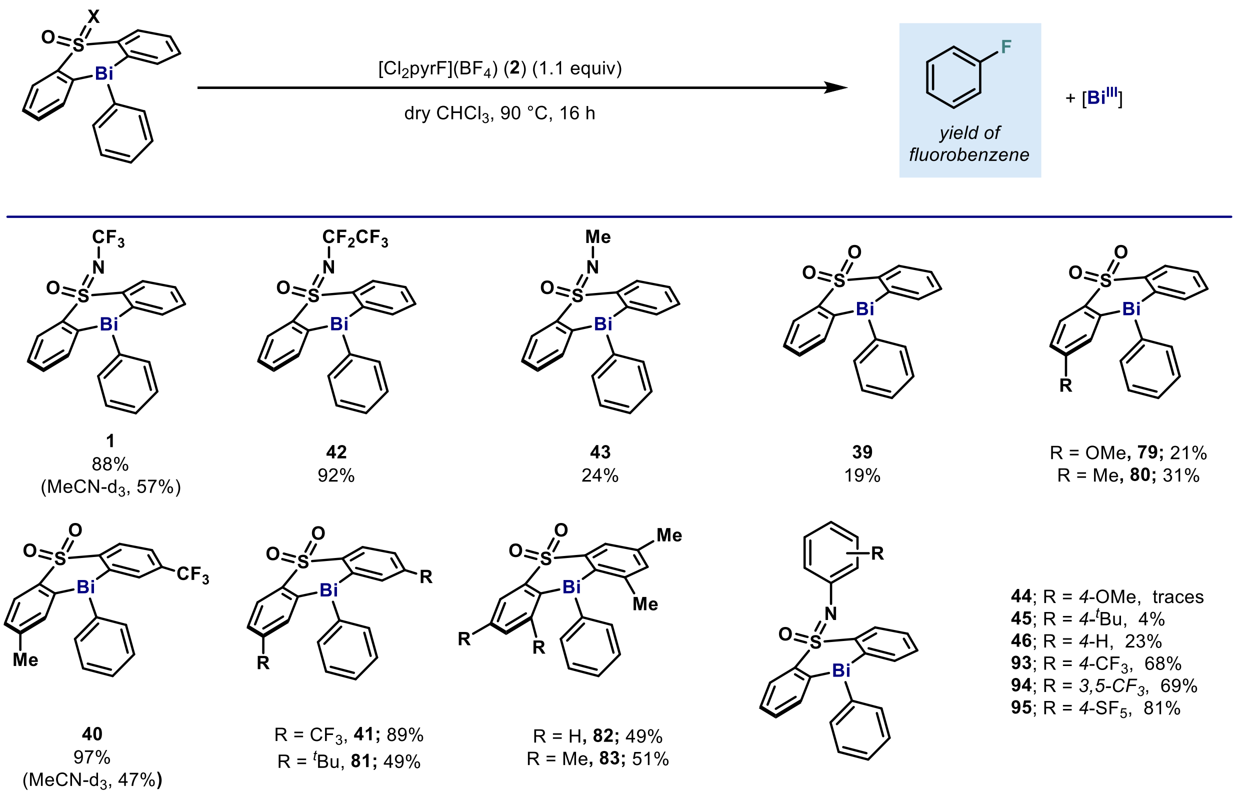

6.3 Scope of the oxidation and reductive elimination of arylfluorides

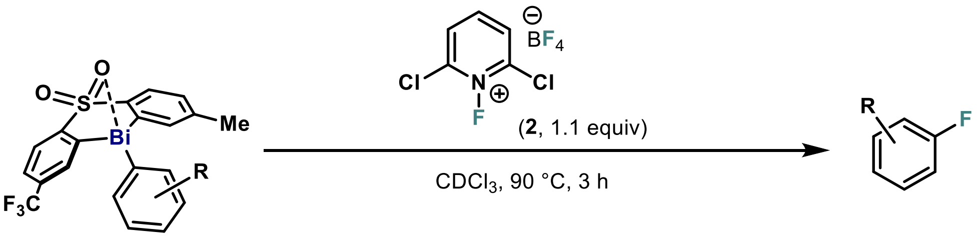

General procedure: The corresponding arylbismine (0.025 mmol) and 1-Fluoro-2,6-chloropyridinium tetrafluoroborate (**2**) were mixed with anhydrous $CHCl_3$ (0.5 mL) under an Ar atmosphere and the reaction was stirred for 3 h at 90 °C. Then, 1-Fluoro-4-nitrobenzene was added as internal standard (addition by weight) together with drops of $CDCl_3$ and the crude reactions were analyzed by ^{19}F NMR to determine the yield. Addition of an external fluoride source resulted in enhanced yields for *para*-CF_3 substituted aryl compounds (see Table S24). For this reason, fluorination of arylbismine compounds featuring electron withdrawing groups in the arene moiety were performed adding 5.0 equiv of NaF at 110 °C in dry chloroform over 16 h.

Table S24. Brief optimization of reaction conditions for *p*-CF_3 substituted arylbisimine **126**.

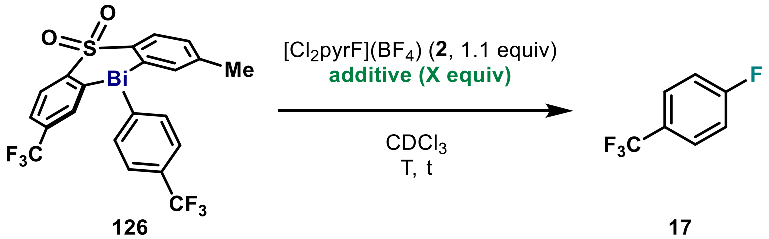

126 **17**

Entry	Additve (equiv)	Temperature, time	Yield of 17 (%)[a]
**1**	None	90 °C, 3 h	23%
**2**	NaF (1)	90 °C, 3 h	44%
**3**	CsF (1)	90 °C, 3 h	28%
**4**	NaF (3)	90 °C, 3 h	49%
**5**	NaF (3)	110 °C, 16 h	52%
**6**	NaF (5)	110 °C, 16 h	**55%**
**7**	NaF (10)	110 °C, 16 h	54%

[a]Yield determined by ^{19}F NMR using 1-Fluoro-4-nitrobenzene as internal standard.

Fluorobenzene (3): Compound **3** was obtained in >95% yield by ^{19}F NMR using 1-fluoro-4-nitrobenzene as internal standard (0.0368 mmol, integration = 1.47) and the peak (^{19}F NMR, δ = –113.1 ppm) was compared with a pure commercial sample from Sigma-Aldrich [CAS:462-06-6] (^{19}F NMR, δ = –113.1 ppm).

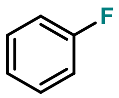

3

1-Fluoro-4-methylbenzene (15): Compound **15** was obtained in 83% yield by ^{19}F NMR using 1-fluoro-4-nitrobenzene as internal standard (0.037 mmol, integration = 1.58) and the peak (^{19}F NMR, δ = –118.7 ppm) was compared with a pure commercial sample from Sigma-Aldrich [CAS: 352-32-9] (^{19}F NMR, δ = –118.7 ppm).

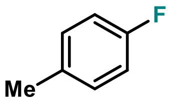

15

1-Fluoro-4-methoxybenzene (16): Compound **16** was obtained in 6% yield by ^{19}F NMR using 1-fluoro-4-nitrobenzene as internal standard (0.038 mmol, integration = 1.51) and the peak (^{19}F NMR, δ = –124.4 ppm) was compared with a pure commercial sample from Across Organics [CAS:459-60-9] (^{19}F NMR, δ = –124.3 ppm).

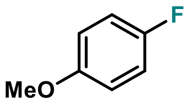

16

1-Fluoro-4-(trifluoromethyl)benzene (17): Compound **17** was obtained in 55% (addition of 5 equiv of NaF and reaction run at 110 °C over 16h) yield by ^{19}F NMR using 1-fluoro-4-nitrobenzene as internal standard (0.040 mmol, integration = 1.61) and the peak (^{19}F NMR, δ = –107.6 ppm) was compared with a previously reported sample.[24]

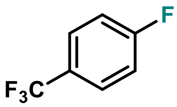

17

1-Chloro-4-fluorobenzene (18): Compound **18** was obtained in 51% (addition of 5 equiv of NaF and reaction run at 110 °C over 16 h) yield by ^{19}F NMR using 1-fluoro-4-nitrobenzene as internal standard (0.048 mmol, integration = 1.92) and the peak (^{19}F NMR, δ = –116.0 ppm) was compared was compared with a previously reported sample.[25]

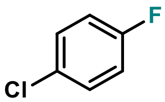

18

Ethyl 4-fluorobenzoate (48): Compound **48** was obtained in >95% yield by ^{19}F NMR using 1-fluoro-4-nitrobenzene as internal standard (0.023 mmol, integration = 0.87) and the peak (^{19}F NMR, δ = –106.1 ppm) was compared with a previously reported sample.[26]

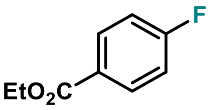

48

1,4-Difluorobenzene (49): Compound **49** was obtained in 52% yield (addition of 5 equiv of NaF and reaction run at 110 °C over 16 h) by ^{19}F NMR using 1-fluoro-4-nitrobenzene as internal standard (0.020 mmol, integration = 0.81) and the peak (^{19}F NMR, δ = –119.7 ppm) was compared with a pure commercial sample from Chempur Feinchemikalien [CAS:540-36-3] (^{19}F NMR, δ = –119.7 ppm).

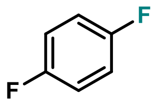
49

(4-Fluorophenyl)trimethylsilane (50): Compound **50** was obtained in >95% yield by ^{19}F NMR using 1-fluoro-4-nitrobenzene as internal standard (0.042 mmol, integration = 1.69) and the peak (^{19}F NMR, δ = –112.7 ppm) was compared with a previously reported sample.[26]

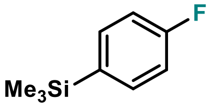
50

4-Fluoro-1,1'-biphenyl (51): Compound **51** was obtained in >95% yield by ^{19}F NMR using 1-fluoro-4-nitrobenzene as internal standard (0.0251 mmol, integration = 1.04) and the peak (^{19}F NMR, δ = –115.9 ppm) was compared with a pure commercial sample from Sigma-Aldrich [CAS: 324-74-3] (^{19}F NMR, δ = –115.9 ppm).

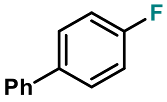
51

1-Fluoro-4-(phenylethynyl)benzene (52): Compound **52** was obtained in 22% yield by ^{19}F NMR using 1-fluoro-4-nitrobenzene as internal standard (0.042 mmol, integration = 1.69) and the peak (^{19}F NMR, δ = –111.0 ppm) was compared was compared with a previously reported sample.[27]

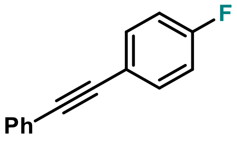
52

1-Fluoro-4-(trifluoromethoxy)benzene (53): Compound **53** was obtained in 6% yield by ^{19}F NMR using 1-fluoro-4-nitrobenzene as internal standard (0.026 mmol, integration = 1.02) and the peak (^{19}F NMR, δ = –115.1 ppm) was compared was compared with a previously reported sample.[28]

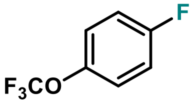
53

1-Fluoro-3-methylbenzene (54): Compound **54** was obtained in 73% yield by ^{19}F NMR using 1-fluoro-4-nitrobenzene as internal standard (0.038 mmol, integration = 1.51) and the peak (^{19}F NMR, δ = –114.3 ppm) was compared with a previously reported sample.[29]

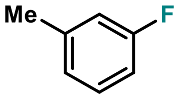
54

1-Fluoro-3-isopropylbenzene (55): Compound **55** was obtained in 83% yield by ^{19}F NMR using 1-fluoro-4-nitrobenzene as internal standard (0.029 mmol, integration = 1.16) and the peak (^{19}F NMR, δ = –113.9 ppm) was compared was compared with a previously reported sample.[25]

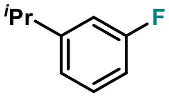
55

1-(tert-butyl)-3-fluorobenzene (56): Compound **56** was obtained in 79% yield by ^{19}F NMR using 1-fluoro-4-nitrobenzene as internal standard (0.031 mmol, integration = 1.24) and the peak (^{19}F NMR, δ = –113.8 ppm) was compared was compared with a previously reported sample.[30]

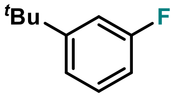

56

(3-Fluorophenyl)trimethylsilane (57): Compound **57** was obtained in 94% yield by ^{19}F NMR using 1-fluoro-4-nitrobenzene as internal standard (0.031 mmol, integration = 1.23) and the peak (^{19}F NMR, δ = –114.1 ppm) was compared was compared with a previously reported sample.[31]

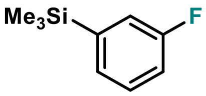

57

(3-Fluorophenyl)trimethylgermane (58): Compound **58** was obtained in 58% yield by ^{19}F NMR using 1-fluoro-4-nitrobenzene as internal standard (0.029 mmol, integration = 1.14) and the peak (^{19}F NMR, δ = –113.9 ppm) was compared was compared with a sample synthesized by lithiation of 1-bromo-3-fluorobenzene and quench by addition of chlorotrimethylgermane (^{19}F NMR, δ = –113.9 ppm).

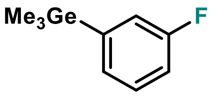

58

1-Chloro-3-fluorobenzene (59): Compound **59** was obtained in 47% (addition of 5 equiv of NaF and reaction run at 110 °C over 16 h) yield by ^{19}F NMR using 1-fluoro-4-nitrobenzene as internal standard (0.043 mmol, integration = 1.73) and the peak (^{19}F NMR, δ = –111.0 ppm) was compared with a pure commercial sample from Apollo Scientific [CAS: 625-98-9] (^{19}F NMR, δ = –110.9 ppm).

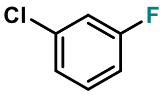

59

4-Fluorobenzaldehyde (60): Compound **60** was obtained in 42% yield (addition of 5 equiv of NaF and reaction run at 110 °C over 16h) by ^{19}F NMR using 1-fluoro-4-nitrobenzene as internal standard (0.046 mmol, integration = 1.85) and the peak (^{19}F NMR, δ = –111.4 ppm) was compared with a pure commercial sample from Sigma-Aldrich [CAS: 456-48-4] (^{19}F NMR, δ = –111.4 ppm).

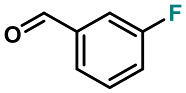

60

1-Fluoro-3-methoxybenzene (61): Compound **61** was obtained in 11% yield (addition of 5 equiv of NaF and reaction run at 110 °C over 16 h) by ^{19}F NMR using 1-fluoro-4-nitrobenzene as internal standard (0.031 mmol, integration = 1.22) and the peak (^{19}F NMR, δ = –110.8 ppm) was compared was compared with a previously reported sample.[32]

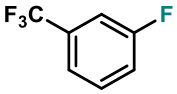

61

1-Fluoro-3-methoxybenzene (62): Compound **62** was obtained in 64% yield by ^{19}F NMR using 1-fluoro-4-nitrobenzene as internal standard (0.035 mmol, integration = 1.41) and the peak (^{19}F NMR, δ = –111.8 ppm) was compared with a pure commercial sample from Sigma-Aldrich [CAS:456-49-5] (^{19}F NMR, δ = –111.9 ppm).

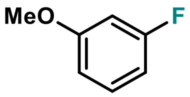

62

4-Fluoro-1,1'-biphenyl (63): Compound **63** was obtained in 88% yield by ^{19}F NMR using 1-fluoro-4-nitrobenzene as internal standard (0.030 mmol, integration = 1.21) and the peak (^{19}F NMR, δ = –114.6 ppm) was compared was compared with a previously reported sample.[33]

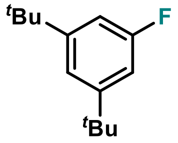

63

1-Fluoro-2-methylbenzene (64): Compound **64** was obtained in 92% yield by ^{19}F NMR using 1-fluoro-4-nitrobenzene as internal standard (0.033 mmol, integration = 1.31) and the peak (^{19}F NMR, δ = –117.7 ppm) was compared with a pure commercial sample from TCI Deutschland [CAS: 95-52-3] (^{19}F NMR, δ = –117.6 ppm).

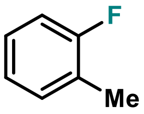

64

1-Bromo-2-fluorobenzene (65): Compound **65** was obtained in 33% yield by ^{19}F NMR using 1-fluoro-4-nitrobenzene as internal standard (0.046 mmol, integration = 1.82) and the peak (^{19}F NMR, δ = –107.2 ppm) was compared with a pure commercial sample from Sigma-Aldrich [CAS:1072-85-*1*] (^{19}F NMR, δ = –107.2 ppm).

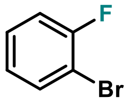

65

4-Fluorobenzaldehyde (66): Compound **66** was obtained in 63% yield by ^{19}F NMR using 1-fluoro-4-nitrobenzene as internal standard (0.028 mmol, integration = 1.11) and the peak (^{19}F NMR, δ = –114.4 ppm) was compared with a pure commercial sample from Sigma-Aldrich [CAS: 405-99-2] (^{19}F NMR, δ = –114.5 ppm).

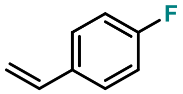

66

2-Fluoronaphthalene (67): Compound **67** was obtained in 66% yield by ^{19}F NMR using 1-fluoro-4-nitrobenzene as internal standard (0.040 mmol, integration = 1.61) and the peak (^{19}F NMR, δ = –114.9 ppm) was compared with a previously reported sample.[34]

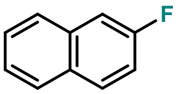

67

6.3.1 Isolation several aryl fluorides representative examples

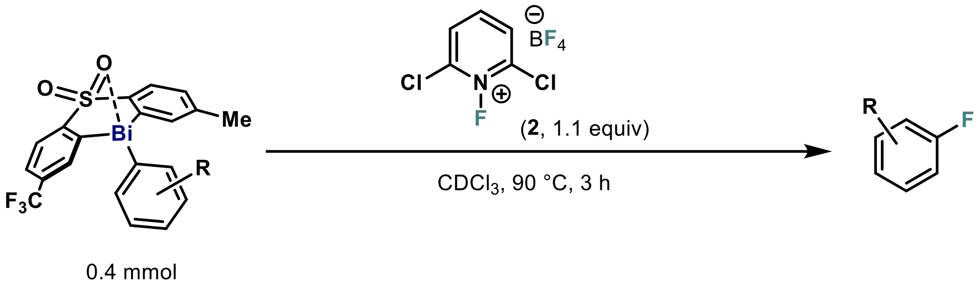

The corresponding arylbismine (0.4 mmol, 1.0 equiv) and 1-fluoro-2,6-chloropyridinium tetrafluoroborate (**2**; 112 mg, 0.44 mmol, 1.1 equiv) were mixed with anhydrous $CHCl_3$ (8.0 mL) under an Ar atmosphere and the reaction was stirred for 3 h at 90 °C. Then, the solvent was removed at room temperature and the crude was purified by silica column chromatography. After evaporating the solvent at room temperature, the title compound was obtained.

Ethyl 4-fluorobenzoate (48): Compound **48** was obtained in 87% after column chromatography using a mixture of pentane and CH_2Cl_2 (5:1) as eluent. Characterization data matched with previously reported data.[26] The product was isolated with residual amounts of protodeboronation product (<5%), which cannot be separated.

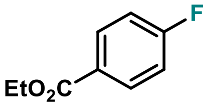

48

^{1}H NMR - (300 MHz, $CDCl_3$): δ 8.04 (dd, *J* = 8.5, 5.7 Hz, 2H), 7.08 (t, *J* = 8.7 Hz, 2H), 4.35 (q, *J* = 7.1 Hz, 2H), 1.37 (t, *J* = 7.1 Hz, 3H).

^{13}C NMR - (75 MHz, $CDCl_3$): δ 165.8 (d, *J* = 254.4 Hz), 165.7, 132.1 (d, *J* = 9.4 Hz), 126.9 (d, *J* = 2.9 Hz), 115.5 (d, *J* = 22.2 Hz), 61.1, 14.3.

^{19}F NMR - (282 MHz, $CDCl_3$): δ –106.2.

(4-Fluorophenyl)trimethylsilane (50): Compound **50** was obtained in 93% after column chromatography using a mixture of pentane and CH_2Cl_2 (5:1) as eluent. Characterization data matched with previously reported data.[26] The product was isolated with residual amounts of protodeboronation product (<5%), which cannot be separated.

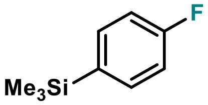

50

^{1}H NMR - (300 MHz, $CDCl_3$): δ 7.58-7.52 (m, 2H), 7.14-7.07 (m, 2H), 0.33 (s, 9H).

^{13}C NMR - (75 MHz, $CDCl_3$): δ 163.8 (d, J = 272 Hz), 136.0 (d, J = 3.9 Hz), 135.3 (d, J = 7.9 Hz), 114.9 (d, J = 19.7 Hz), –0.88.

^{19}F NMR - (282 MHz, $CDCl_3$): δ –112.5.

4-Fluoro-1,1'-biphenyl (51): Compound **51** was obtained in 88% after column chromatography using a mixture of pentane and CH_2Cl_2 (5:1) as eluent. Characterization data matched with previously reported data and pure commercial sample from Sigma-Aldrich [CAS: 324-74-3] (^{19}F NMR, δ = –115.9 ppm).[35]

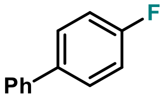

51

^{1}H NMR - (300 MHz, $CDCl_3$): δ 7.71 – 7.54 (m, 4H), 7.54 – 7.44 (m, 2H), 7.44 – 7.35 (m, 1H), 7.24 – 7.11 (m, 2H).

^{13}C NMR - (75 MHz, $CDCl_3$): δ 162.7 (d, J = 244 Hz), 140.5, 137.6, 129.0, 128.9 (d, J = 8.5 Hz), 127.5, 127.3, 115.8 (d, J = 21 Hz)

^{19}F NMR - (282 MHz, $CDCl_3$): δ –115.7.

(3-Fluorophenyl)trimethylsilane (57): Compound **57** was obtained in 94% after column chromatography using a mixture of pentane and CH_2Cl_2 (5:1) as eluent. Characterization data matched with previously reported data.[31] The product was isolated with trace amounts of unidentified byproducts.

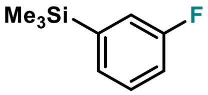

57

^{1}H NMR - (300 MHz, $CDCl_3$): δ 7.41 – 7.26 (m, 2H), 7.23 – 7.16 (m, 1H), 7.03 (tdd, J = 7.8, 2.7, 1.4 Hz, 1H), 0.28 (s, 9H).

^{13}C NMR - (75 MHz, $CDCl_3$): δ 162.6 (d, J = 248.3 Hz), 143.7 (d, J = 3.7 Hz), 129.5 (d, J = 6.9 Hz), 128.7 (d, J = 2.9 Hz), 119.6 (d, J = 18.2 Hz), 115.6 (d, J = 21.0 Hz), −1.3.

^{19}F NMR - (282 MHz, $CDCl_3$): δ –114.0.

7. Catalytic fluorination

7.1 Role of base in catalytic fluorination with **38** as catalyst

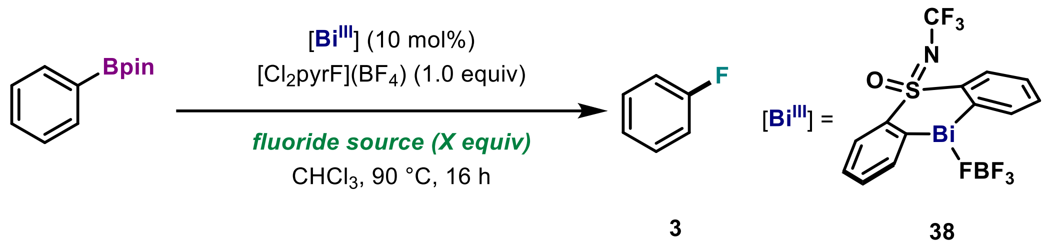

A culture tube equipped with a stir bar and a cap with a plastic seal was charged with phenylboronic acid pinacol ester (0.075 mmol, 3 equiv) and **38** (10 mol%). The tube was transferred to a glove box, 1-fluoro-2,6-dichloropyridinium tetrafluoroborate **2** (1.0 equiv, 0.025 mmol) and *fluoride source* (Y equiv) were added. The tube was removed from the glove box and subjected to a positive pressure of argon. Dry $CDCl_3$ (1.0 mL) was added without piercing the plastic seal and the reaction was then stirred overnight at the corresponding temperature. After the indicated time, the yield was calculated by ^{19}F NMR using 1-fluoro-4-nitrobenzene as an internal standard (addition by weight).

Table S25. Fluorination of phenylboronic esters catalyzed by **38** in presence of a variety of fluoride sources. Yield determined by ^{19}F NMR.

Entry	Additive (equiv)	Yield of 3 (%)
**1**	None	43%
**2**	NaF (1)	67%
**3**	NaF (5)	90%
**4**	TBAF (1)	<5%
**5**	TBAT (1)	<5%
**6**	TAS-F (1)	<5%
**7**	CsF (1)	<5%

In our previous study[2] we observed that fluorides such as sodium fluoride (NaF) have a critical role in the Bi-catalyzed fluorination of arylboronic esters, enhancing the yield of fluorobenzene (Table S25, entries 1-3). These observations are in stark contrast with the formation of hexacoordinated species **35** (Figure 10, main text) in presence of soluble fluoride sources such as TBAT and TAS-F, as well as the subsequent inhibition of aryl–F reductive elimination. Indeed, if NaF is substituted for soluble fluoride sources the catalytic reaction yield also drops dramatically (entries 4-6).

Soluble inorganic salts such as CsF (entry 7) cause a similar inhibition. These results suggest that low concentrations of fluoride anions yield a beneficial effect in catalytic reactions, most likely in the transmetalation reaction, which is thought to be the rate determining step based on the fast oxidation and reductive elimination sequence in the stoichiometric fluorination of phenyl bismines. However, a high concentration of F^- given by the use of soluble fluoride sources results in the formation of hexacoordinated anionic species **35**, hence hampering the reductive elimination step and inhibiting catalysis.

7.2 Optimization for Bi-catalyzed fluorination

General Procedure for Optimization for Bi-catalyzed fluorination of boron-based nucleophiles: A culture tube equipped with a stir bar and a cap with a plastic seal was charged with phenylboronic acid pinacol ester (0.05 mmol) and **68** (10 mol%). The tube was transferred to a glove box, 1-fluoro-2,6-dichloropyridinium tetrafluoroborate **2** (X equiv) and base (Y equiv) were added. The tube was removed from the glove box and subjected to a positive pressure of Ar. Dry $CDCl_3$ (1.0 mL) was added without piercing the plastic seal and the reaction was then stirred overnight at the corresponding temperature. After the indicated time, the yield was calculated by ^{19}F NMR using 1-fluoro-4-nitrobenzene as an internal standard (addition by weight).

Table S26. Optimization of reaction conditions for the Bi-catalyzed formation of fluorobenzene **3** with **68**.

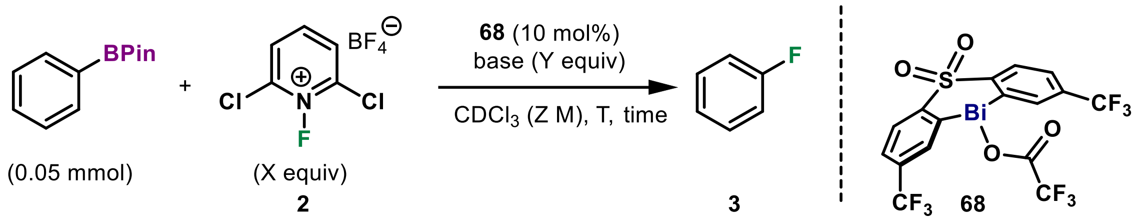

entry	X	Base	Y	Z	Time (h)	T (°C)	yield of 3 (%)[a]
1	1.1	NaF	1.0	0.05	16	90	74
2	1.1	NaF	3.0	0.05	16	90	71
3	1.1	NaF	6.0	0.05	16	90	75
4	1.1	NaF	10.0	0.05	16	90	71
5	1.1	NaF	0.5	0.05	16	90	75
6	1.1	$NaBF_4$	1.0	0.05	16	90	73
7	1.1	$NaBF_4$	3.0	0.05	16	90	74
8	1.1	$NaBF_4$	5.0	0.05	16	90	65
9	1.1	-	-	0.05	16	90	76
10	1.5	-	-	0.05	16	90	73
11	2.0	-	-	0.05	16	90	73
12	3.0	-	-	0.05	16	90	73
13	1.1	-	-	0.025	16	90	65
14	1.1	-	-	0.04	16	90	71
15	1.1	-	-	0.075	16	90	72
16	1.1	-	-	0.05	1	90	Traces
17	1.1	-	-	0.05	2	90	19
18	1.1	-	-	0.05	4	90	42
19	1.1	-	-	0.05	6	90	57
20	1.1	-	-	0.05	8	90	70
21	1.1	-	-	0.05	16	60	31
22	1.1	-	-	0.05	16	70	40
23	1.1	-	-	0.05	16	80	74
24	1.1	-	-	0.05	16	100	76
25	1.1	-	-	0.05	16	110	71
26[b]	1.1	-	-	0.05	16	90	n.r.
27[c]	1.1	-	-	0.05	16	90	59

[a] ^{19}F NMR yield using 1-fluoro-4-nitrobenzene as internal standard; [b] Reaction run without catalyst; [c] Reaction run using catalyst **100** (10 mol%) instead of **68**.

7.3 Crude mixture analysis after Bi-catalyzed fluorination of boron based nucleophiles

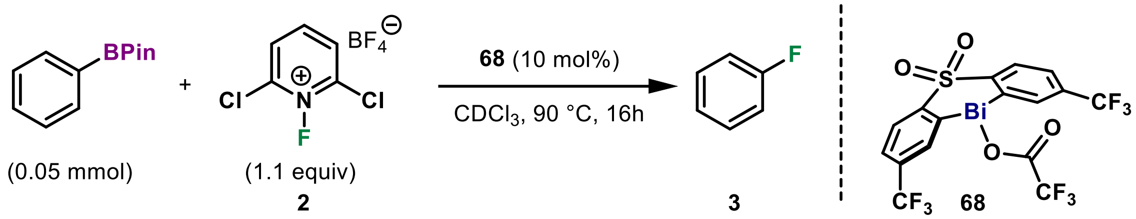

A culture tube equipped with a stir bar and a cap with a plastic seal was charged with phenylboronic acid pinacol ester (0.05 mmol) and **68** (10 mol%). The tube was transferred to a glove box, 1-fluoro-2,6-dichloropyridinium tetrafluoroborate **2** (1.1 equiv) was added. The tube was removed from the glove box and subjected to a positive pressure of Ar. Dry $CDCl_3$ (1.0 mL) was added without piercing the plastic seal and the reaction was then stirred overnight at 90 °C. After 16 h, the crude was analyzed by ^{1}H NMR, ^{11}B NMR and ^{19}F NMR to determine the species in solution. As shown in Figure S68, fluorobenzene was detected in 76% (in blue). Around 80% of **68** was recovered (in black) as well as 83% of the corresponding 2,6-dichloropyridine byproduct (0.83 equiv, in green) was produced. Furthermore, benzene was also detected by ^{1}H NMR (approximately 8% yield, signal mixed with fluorobenzene, in red) due to protodemetalation. Besides the species in solution, evolution of white fumes was observed after allowing the atmosphere of the reaction media mix with air.

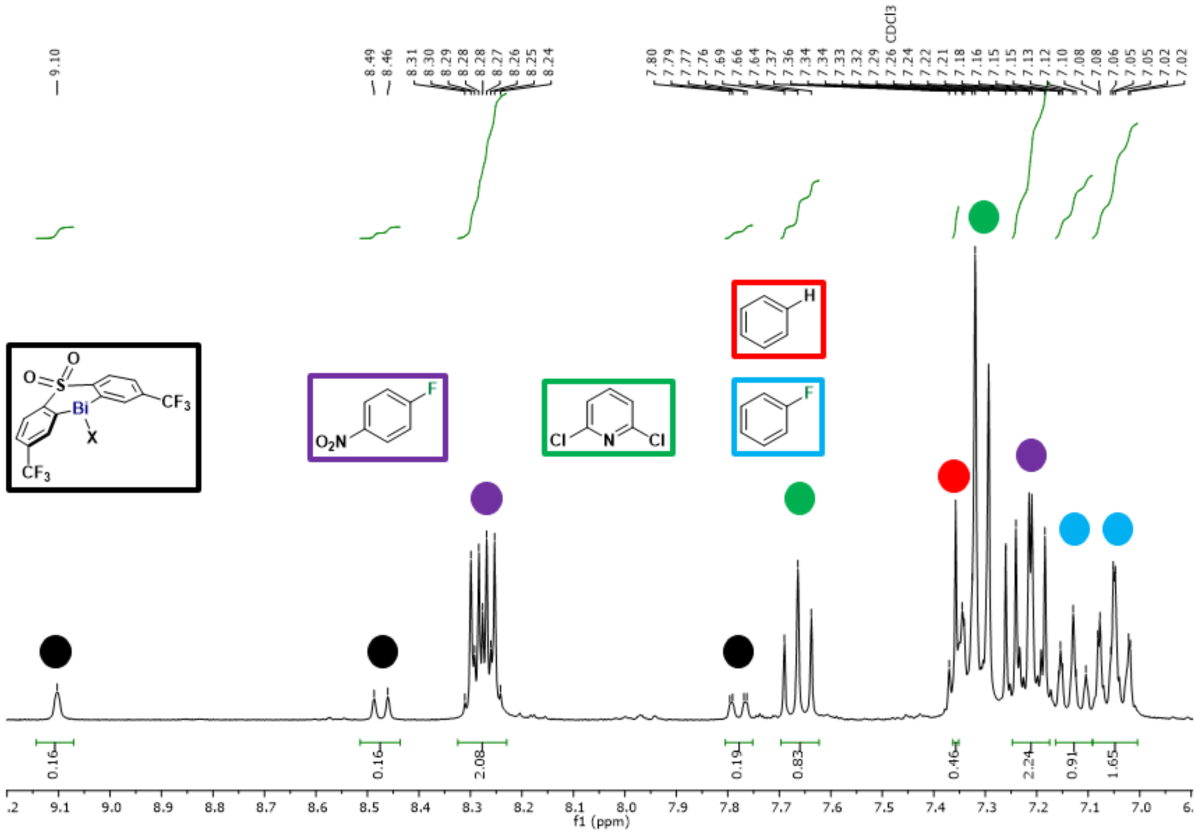

Figure S68. Aromatic region of ^{1}H NMR of the crude mixture after Bi-catalyzed fluorination of phenylboronic acid pinacol ester with 1-fluoro-2,6-chloropyridinium tetrafluoroborate **2** in $CDCl_3$ at 90 °C.

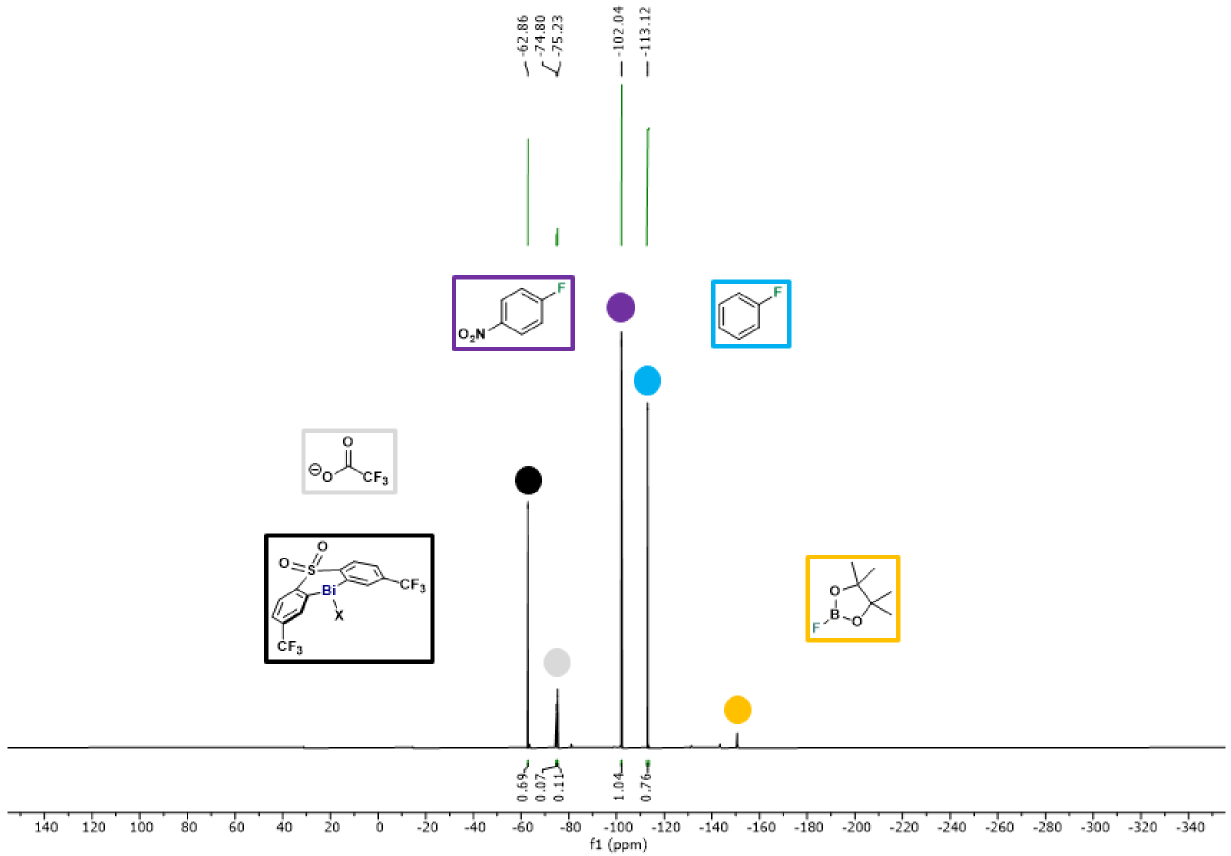

Figure S69. ^{19}F NMR of the crude mixture after Bi-catalyzed fluorination of phenylboronic acid pinacol ester with 1-fluoro-2,6-chloropyridinium tetrafluoroborate **2** in $CDCl_3$ at 90 °C.

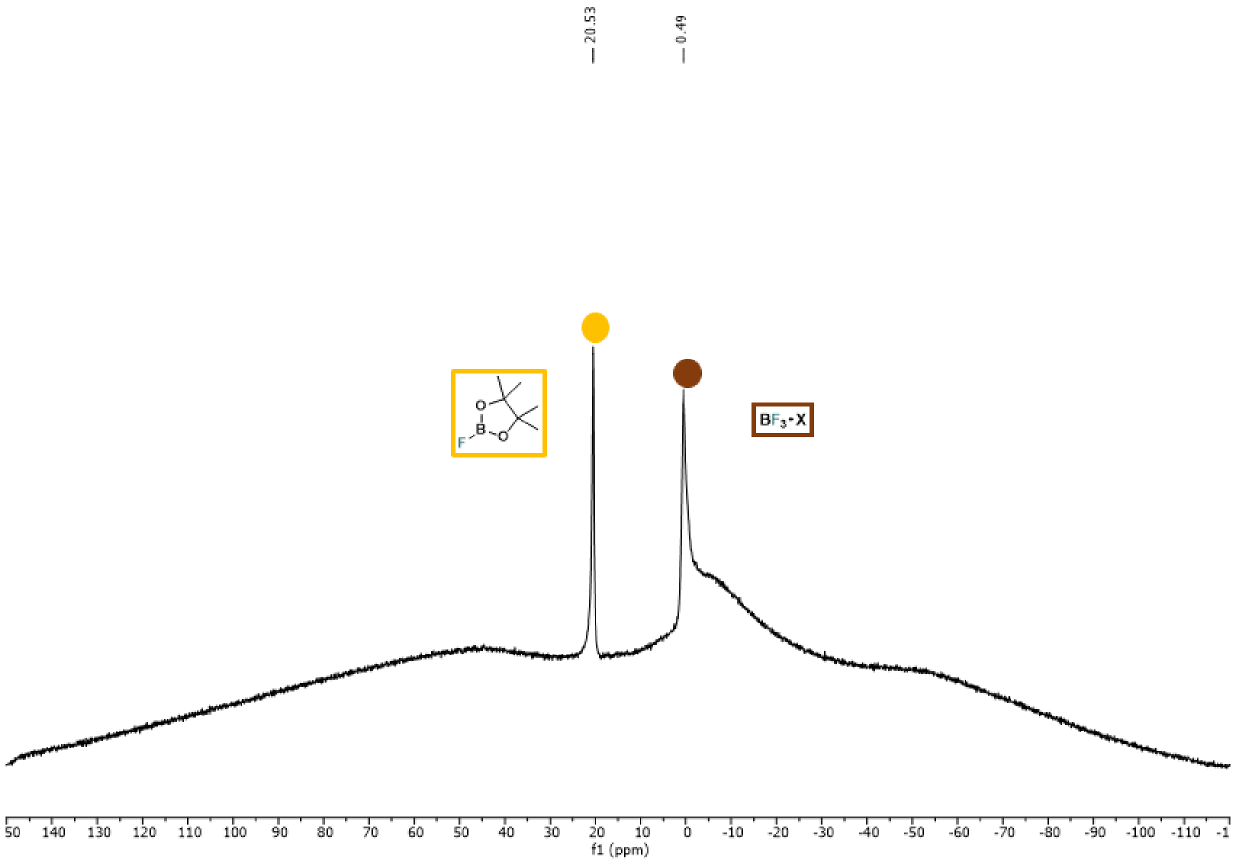

Figure S70. ^{11}B NMR of the crude mixture after Bi-catalyzed fluorination of phenylboronic acid pinacol ester with 1-fluoro-2,6-chloropyridinium tetrafluoroborate **2** in $CDCl_3$ at 90 °C.

7.4 Identification of byproducts of Bi-catalyzed fluorination of boron-based nucleophiles

The presence of FBpin (2-fluoro-4,4,5,5-tetramethyl-1,3,2-dioxaborolane) in the reaction media after the catalysis was proven by synthesizing FBpin and comparing its ^{1}H, ^{19}F and ^{11}B NMR spectra with the crude mixture of catalysis.[36]

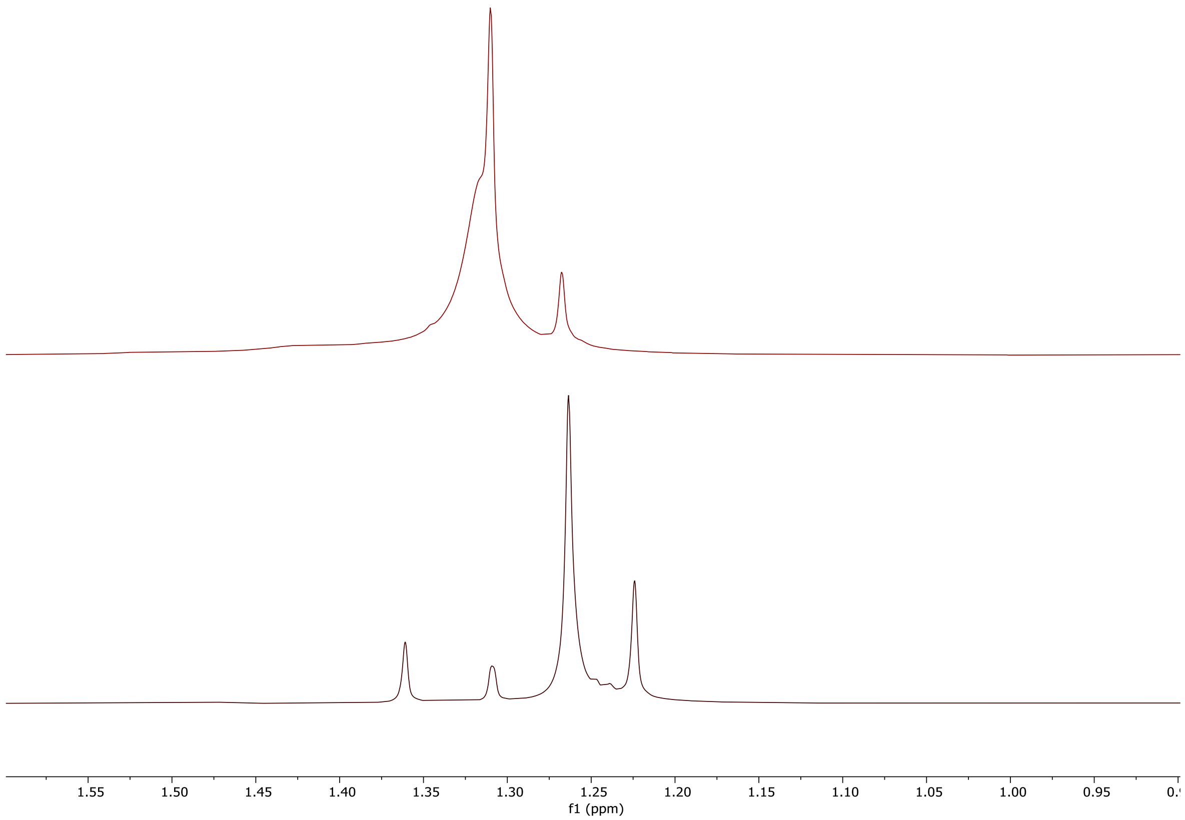

Figure S71. ^{1}H NMR signal of FBpin of the crude mixture after Bi-catalyzed fluorination of phenylboronic acid pinacol ester with 1-fluoro-2,6-chloropyridinium tetrafluoroborate **2** in $CDCl_3$ at 90 °C (top) and synthesized material (bottom).

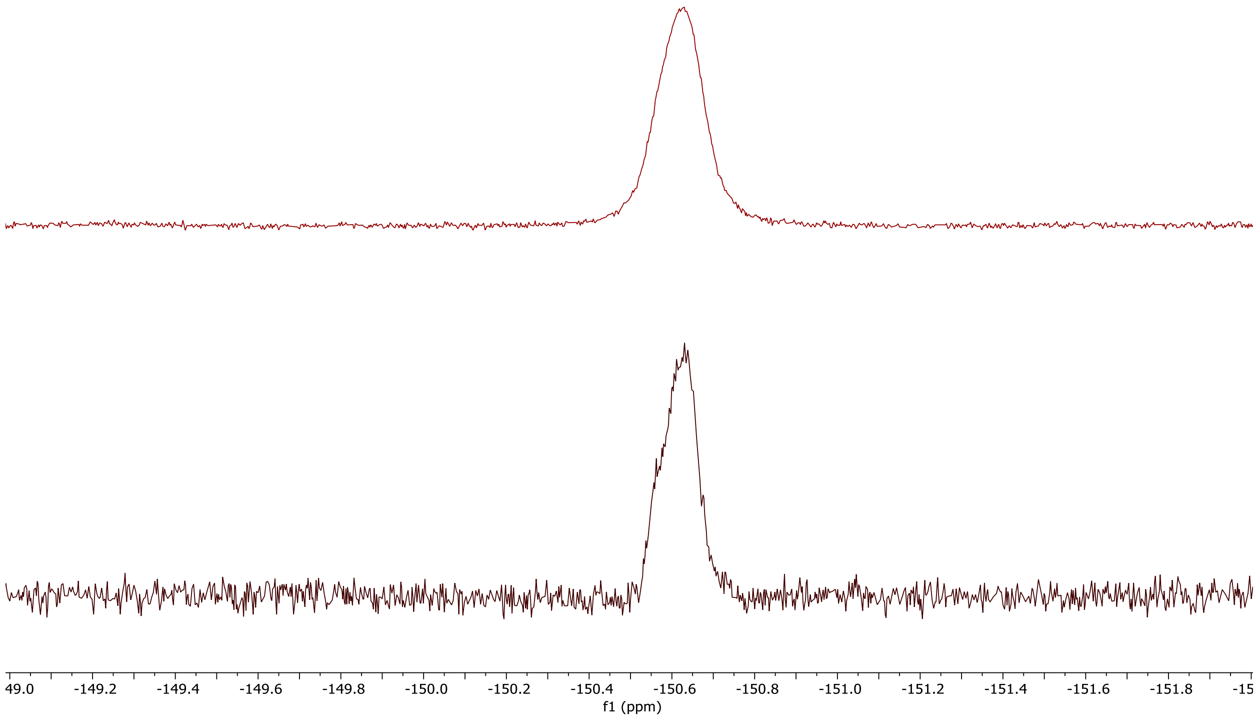

Figure S72. ^{19}F NMR signal of FBpin of the crude mixture after Bi-catalyzed fluorination of phenylboronic acid pinacol ester with 1-fluoro-2,6-chloropyridinium tetrafluoroborate **2** in $CDCl_3$ at 90 °C (top) and synthesized material (bottom).

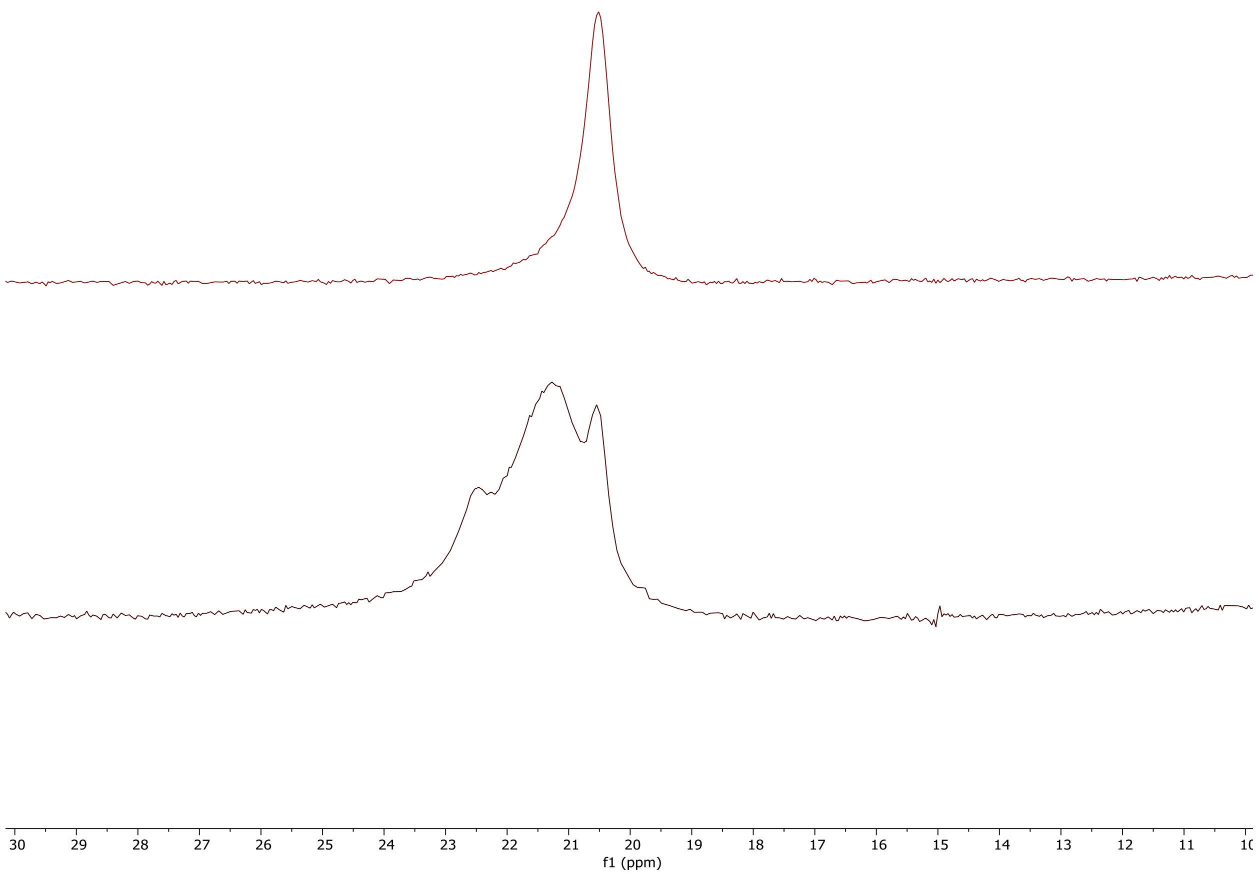

Figure S73. ^{11}B NMR signal of FBpin of the crude mixture after Bi-catalyzed fluorination of phenylboronic acid pinacol ester with 1-fluoro-2,6-chloropyridinium tetrafluoroborate **2** in $CDCl_3$ at 90 °C (top) and synthesized material (bottom).

The observation of formation of white fumes after opening the screw cap of the culture tube after reaction completion suggested the presence of BF_3 as byproduct of the catalysis. Colorless BF_3 gas is known to hydrolyze in contact with moist air producing white fumes. The presence of BF_3 in the atmosphere of the reaction was proved by bubbling the gas at the headspace through deuterated acetonitrile, or a pyridine solution in deuterated chloroform. Then, ^{19}F and ^{11}B NMR spectra of these solutions were compared with respective NMR spectra of a CD_3CN solution of $BF_3{\cdot}OEt_2$ (Figures S74 and S75) and pyridine–BF_3 complex NMR values reported in literature (Figures S76 and S77).

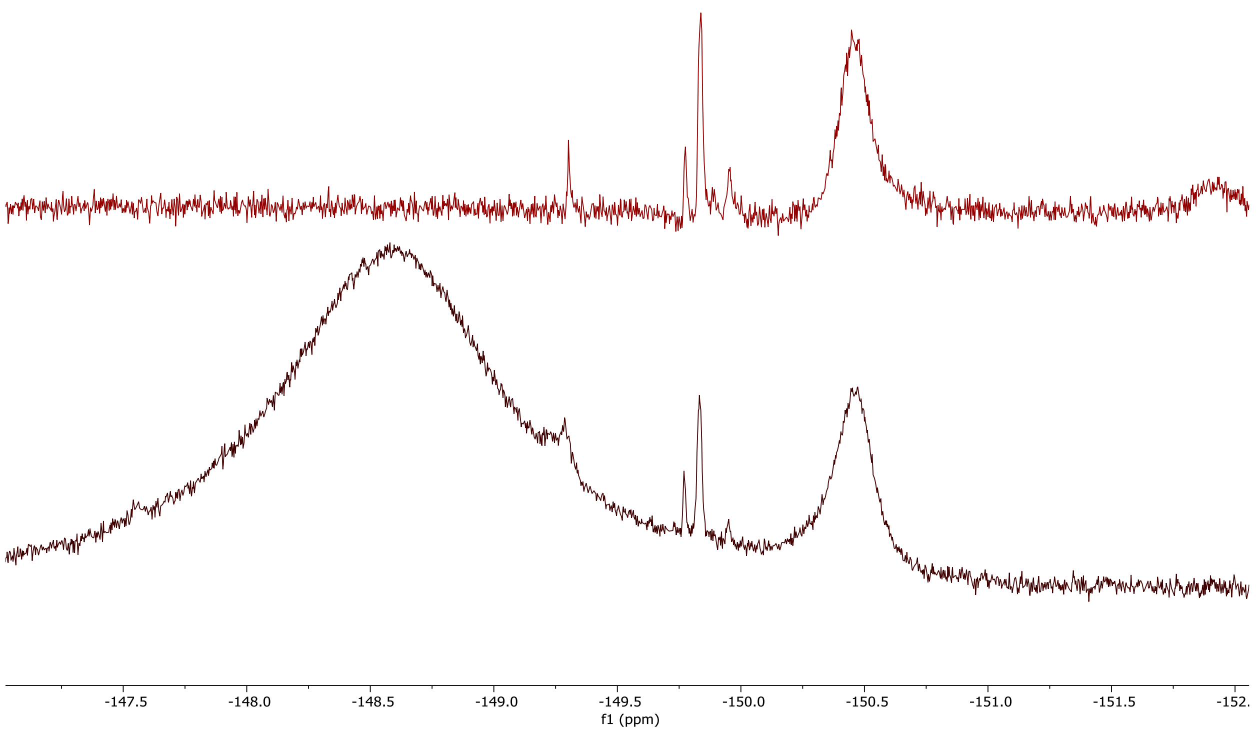

Figure S74. ^{19}F NMR of CD_3CN after bubbling the gases in the headspace after reaction completion (top) and a CD_3CN solution of $BF_3{\cdot}OEt_2$ (bottom).

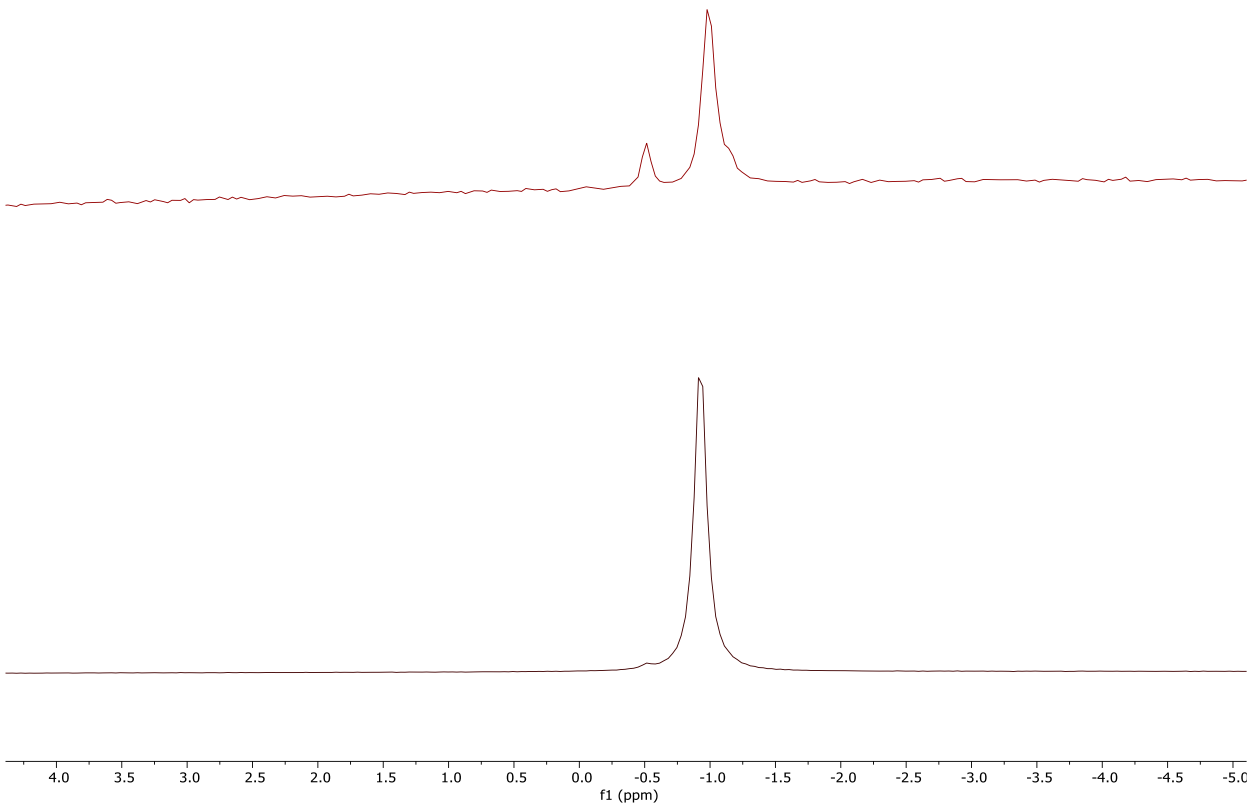

Figure S75. ^{11}B NMR after bubbling the gases in the headspace after reaction completion (top) and of CD_3CN solution of $BF_3{\cdot}OEt_2$ (bottom).

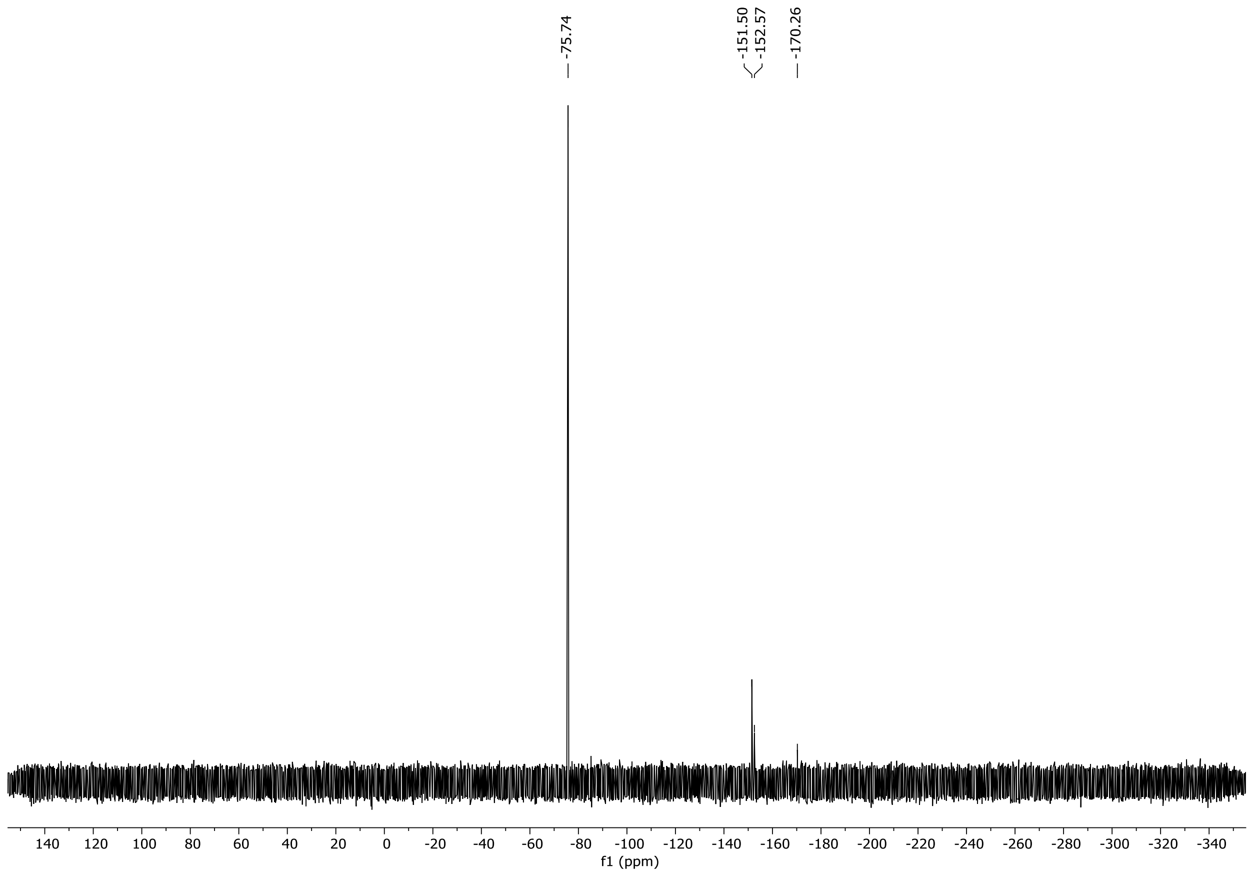

Figure S76. ^{19}F NMR of the formed $CDCl_3$ solution of the pyridine–BF_3 complex. Literature value –151.8 - –152.0 ppm.[37] Species at –75.7 ppm correspond to the fluorine shift of trifluoroacetic acid.

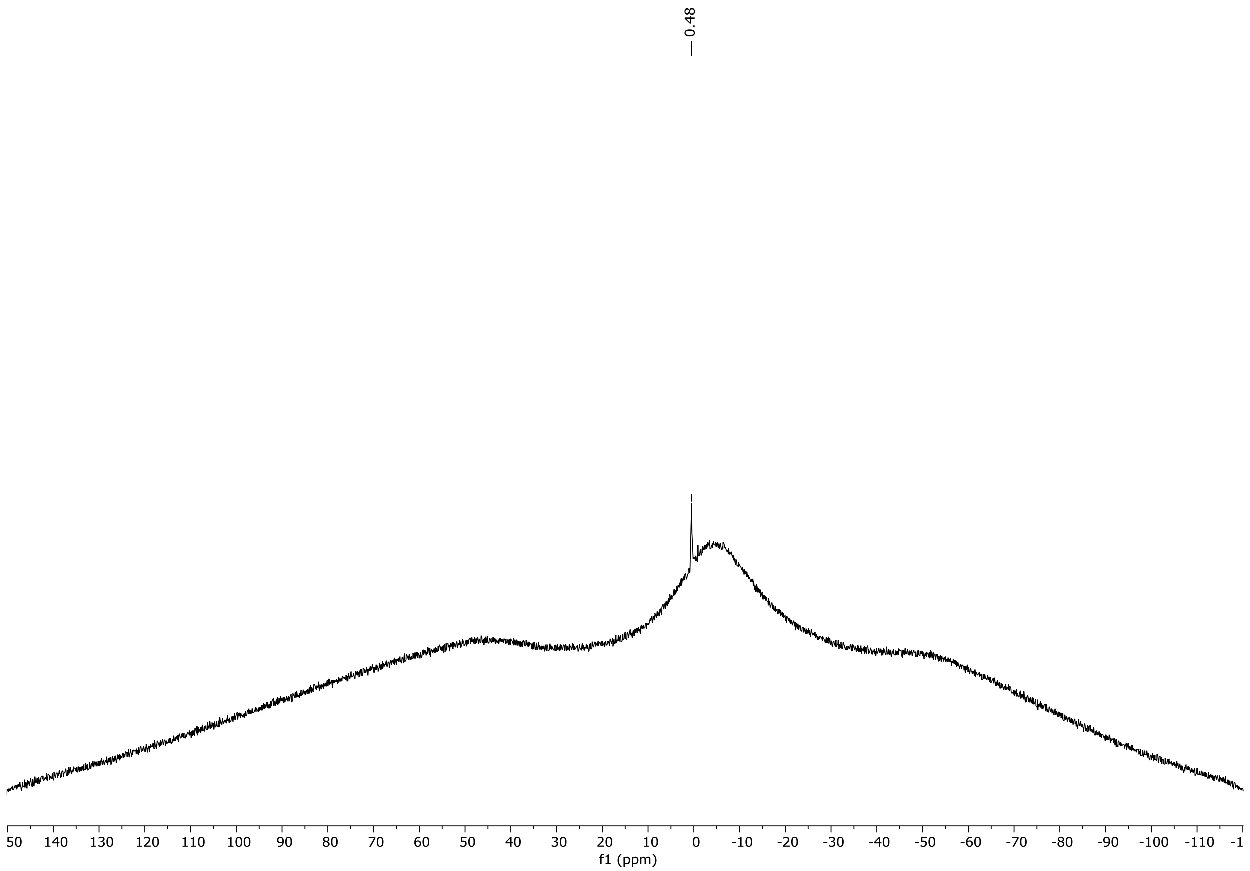

Figure S77. ^{11}B NMR of the formed $CDCl_3$ solution of the pyridine–BF_3 complex. Literature value 0.62 ppm.[37]

7.5 Scope of Bi-catalyzed fluorination of boron-based nucleophiles

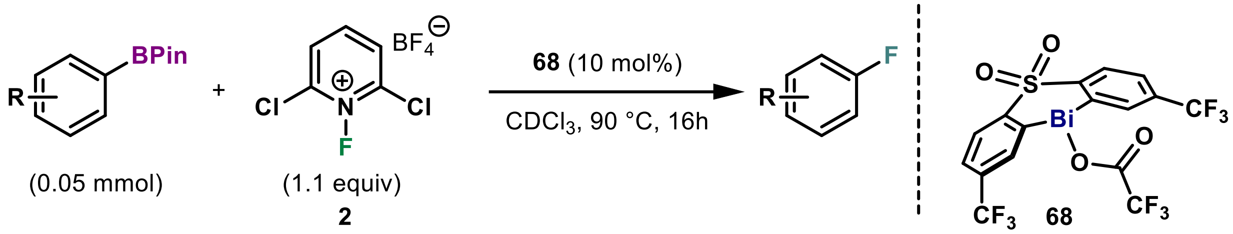

General Procedure of Bi-catalyzed fluorination of boron-based nucleophiles: A culture tube equipped with a stir bar and a cap with a plastic seal was charged with arylboronic acid pinacol ester (0.05 mmol) and **68** (10 mol%). The tube was transferred to a glove box, 1-fluoro-2,6-dichloropyridinium tetrafluoroborate **2** (1.1 equiv) was added. The tube was removed from the glove box and subjected to a positive pressure of argon. Dry $CDCl_3$ (1.0 mL) was added without piercing the plastic seal and the reaction was then stirred overnight at 90 °C. After 16 h, the crude was analyzed by ^{19}F NMR to determine the yield using 1-fluoro-4-nitrobenzene as internal standard.

Fluorobenzene (3): Compound **3** was obtained in 76% yield (7.6 TON) by ^{19}F NMR using 1-fluoro-4-nitrobenzene as internal standard (0.0524 mmol, integration = 1.04) and the peak (^{19}F NMR, δ = –113.1 ppm) was compared with a pure commercial sample from Sigma-Aldrich [CAS:462-06-6] (^{19}F NMR, δ = –113.1 ppm).

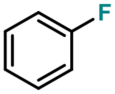

3

1-(tert-butyl)-4-fluorobenzene (14): Compound **14** was obtained in 85% yield (8.5 TON) by ^{19}F NMR using 1-fluoro-4-nitrobenzene as internal standard (0.0321 mmol, integration = 1.28) and the peak (^{19}F NMR, δ = –118.8 ppm) was compared with a previously reported sample.[34]

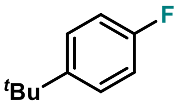

14

1-fluoro-4-methylbenzene (15): Compound **15** was obtained in 65% yield (6.5 TON) by ^{19}F NMR using 1-fluoro-4-nitrobenzene as internal standard (0.0539 mmol, integration = 1.05) and the peak (^{19}F NMR, δ = –118.7 ppm) was compared with a previously reported sample.[29]

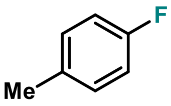

15

1-fluoro-4-(trifluoromethyl)benzene (17): Compound **17** was obtained in 56% yield (5.6 TON) by ^{19}F NMR using 1-fluoro-4-nitrobenzene as internal standard (0.0496 mmol, integration = 1.01) and the peak (^{19}F NMR, δ = –107.6 ppm) was compared with a previously reported sample.[24]

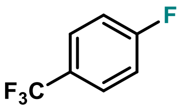

17

1-chloro-4-fluorobenzene (18): Compound **18** was synthesized by adding additional 5.0 equiv of NaF to general procedure and was obtained in 35% yield (3.5 TON) by ^{19}F NMR using 1-fluoro-4-nitrobenzene as internal standard (0.0363 mmol, integration = 1.45) and the peak (^{19}F NMR, δ = –116.0 ppm) was compared was compared with a previously reported sample.[25]

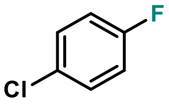

18

1,4-difluorobenzene (49): Compound **49** was synthesized by adding additional 5.0 equiv of NaF to general procedure and was obtained in 19% yield (1.9 TON) by ^{19}F NMR using 1-fluoro-4-nitrobenzene as internal standard (0.019 mmol, integration = 1.39) and the peak (^{19}F NMR, δ = –119.7 ppm) was compared was compared with a previously reported sample.[25]

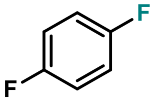

49

(4-fluorophenyl)trimethylsilane (50): Compound **50** was obtained in 77% yield (7.7 TON) by ^{19}F NMR using 1-fluoro-4-nitrobenzene as internal standard (0.0524 mmol, integration = 1.07) and the peak (^{19}F NMR, δ = –112.7 ppm) was compared with a previously reported sample.[38]

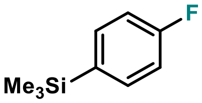

50

4-fluoro-1,1'-biphenyl (51): Compound **51** was obtained in 73% yield (7.3 TON) by ^{19}F NMR using 1-fluoro-4-nitrobenzene as internal standard (0.051 mmol, integration = 1.02) and the peak (^{19}F NMR, δ = –115.9 ppm) was compared with a previously reported sample.[39]

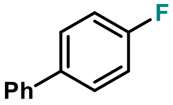

51

1-fluoro-4-(phenylethynyl)benzene (52): Compound **52** was obtained in 49% yield (4.9 TON) by ^{19}F NMR using 1-fluoro-4-nitrobenzene as internal standard (0.051 mmol, integration = 1.04) and the peak (^{19}F NMR, δ = –111.0 ppm) was compared with a previously reported sample.[40]

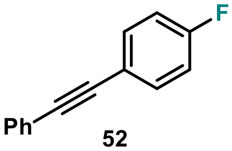

52

1-fluoro-4-(trifluoromethoxy)benzene (53): Compound **53** was obtained in 18% yield (1.8 TON) by ^{19}F NMR using 1-fluoro-4-nitrobenzene as internal standard (0.0517 mmol, integration = 1.04) and the peak (^{19}F NMR, δ = –115.1 ppm) was compared with a previously reported sample.[28]

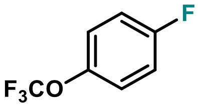

53

1-fluoro-3-methylbenzene (54): Compound **54** was obtained in 72% yield (7.2 TON) by ^{19}F NMR

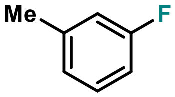

54

using 1-fluoro-4-nitrobenzene as internal standard (0.0496 mmol, integration = 0.96) and the peak (^{19}F NMR, δ = –114.3 ppm) was compared with a previously reported sample.[29]

1-chloro-3-fluorobenzene (59): Compound **59** was obtained in 53% yield (5.3 TON) by ^{19}F NMR

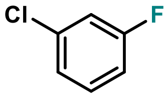

59

using 1-fluoro-4-nitrobenzene as internal standard (0.051 mmol, integration = 1.06) and the peak (^{19}F NMR, δ = –111.0 ppm) was compared with a pure sample from Apollo Scientific [CAS: 625-98-9] (^{19}F NMR, δ = –110.9 ppm).

1-fluoro-3-(trifluoromethyl)benzene (61): Compound **61** was obtained in 18% yield (1.8 TON)

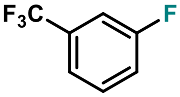

61

by ^{19}F NMR using 1-fluoro-4-nitrobenzene as internal standard (0.0524 mmol, integration = 1.12) and the peak (^{19}F NMR, δ = –110.9 ppm) was compared with a previously reported sample.[41]

1-fluoro-3-methoxybenzene (62): Compound **62** was synthesized by adding additional 5.0 equiv

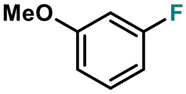

62

of NaF to general procedure and was obtained in 31% yield (3.1 TON) by ^{19}F NMR using 1-fluoro-4-nitrobenzene as internal standard (0.0318 mmol, integration = 1.27) and the peak (^{19}F NMR, δ = –111.8 ppm) was compared with a pure commercial sample from Sigma-Aldrich [CAS:456-49-5] (^{19}F NMR, δ = –111.9 ppm).

1-fluoro-2-methylbenzene (64): Compound **64** was obtained in 69% yield (6.9 TON) by ^{19}F NMR

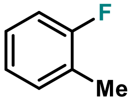

64

using 1-fluoro-4-nitrobenzene as internal standard (0.0517 mmol, integration = 1.10) and the peak (^{19}F NMR, δ = –117.7 ppm) was compared with a pure commercial sample from TCI Deutschland [CAS: 95-52-3] (^{19}F NMR, δ = –117.6 ppm).

1-bromo-2-fluorobenzene (65): Compound **65** was obtained in 7% yield (0.7 TON) by ^{19}F NMR

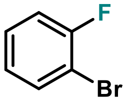

65

using 1-fluoro-4-nitrobenzene as internal standard (0.051 mmol, integration = 0.98) and the peak (^{19}F NMR, δ = –107.2 ppm) was compared with a pure commercial sample from Sigma-Aldrich [CAS:1072-85-1] (^{19}F NMR, δ = –107.2 ppm).

2-fluoronaphthalene (67): Compound **67** was obtained in 23% yield (2.3 TON) by ^{19}F NMR using

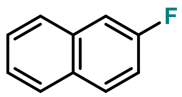

67

1-fluoro-4-nitrobenzene as internal standard (0.0524 mmol, integration = 1.05) and the peak (^{19}F NMR, δ = –114.9 ppm) was compared with a previously reported sample.[34]

1-bromo-4-fluorobenzene (69): Compound **69** was synthesized by adding additional 5.0 equiv of

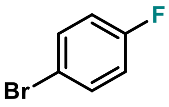

69

NaF to general procedure and was obtained in 51% yield (5.1 TON) by ^{19}F NMR using 1-fluoro-4-nitrobenzene as internal standard (0.029 mmol, integration = 1.19) and the peak (^{19}F NMR, δ = –115.4 ppm) was compared with a previously reported sample.[42]

1-fluoro-4-iodobenzene (70): Compound **70** was synthesized by adding additional 5.0 equiv of

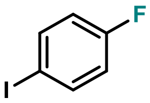

70

NaF to general procedure and was obtained in 43% yield (4.3 TON) by ^{19}F NMR using 1-fluoro-4-nitrobenzene as internal standard (0.022 mmol, integration = 1.55) and the peak (^{19}F NMR, δ = –114.4 ppm) was compared with a pure commercial sample from Alfa Aesar [CAS:352-34-1] (^{19}F NMR, δ = –114.4 ppm).

1-fluoro-3-iodobenzene (71): Compound **71** was synthesized by adding additional 5.0 equiv of

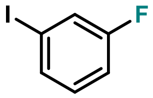

71

NaF to general procedure and was obtained in 32% yield (3.2 TON) by ^{19}F NMR using 1-fluoro-4-nitrobenzene as internal standard (0.034 mmol, integration = 1.36) and the peak (^{19}F NMR, δ = –110.6 ppm) was compared with a previously reported sample.[43]

3-fluorobenzonitrile (72): Compound **72** was synthesized by adding additional 5.0 equiv of NaF

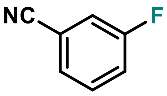

72

to general procedure and was obtained in 10% yield (1.0 TON) by ^{19}F NMR using 1-fluoro-4-nitrobenzene as internal standard (0.0305 mmol, integration = 1.22) and the peak (^{19}F NMR, δ = –109.6 ppm) was compared with a pure commercial sample from Alfa Aesar [CAS: 403-54-3] (^{19}F NMR, δ = –109.7 ppm).

1-fluoro-2-isopropylbenzene (73): Compound **73** was obtained in 32% yield (3.2 TON) by ^{19}F

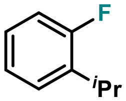

73

NMR using 1-fluoro-4-nitrobenzene as internal standard (0.0503 mmol, integration = 1.01) and the peak (^{19}F NMR, δ = –119.4 ppm) was compared with a previously reported sample.[44]

Methyl 4-fluorobenzoate (75): Compound **75** was obtained in 56% yield (5.6 TON) by ^{19}F NMR using 1-fluoro-4-nitrobenzene as internal standard (0.0532 mmol, integration = 1.06) and the peak (^{19}F NMR, δ = –105.8 ppm) was compared with a previously reported sample.[45]

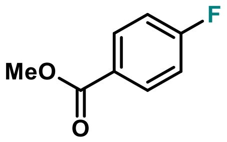

75

2-bromo-5-fluoro-1,3-dimethylbenzene (76): Compound **76** was obtained in 69% yield (6.9 TON) by ^{19}F NMR using 1-fluoro-4-nitrobenzene as internal standard (0.0539 mmol, integration = 1.12) and the peak (^{19}F NMR, δ = –117.3 ppm) was compared with a previously reported sample.[46]

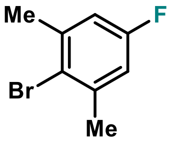

76

1-fluoro-4-(methylsulfonyl)benzene (77): Compound **77** was synthesized by adding additional 5.0 equiv of NaF to general procedure and was obtained in 35% yield (3.5 TON) by ^{19}F NMR using 1-fluoro-4-nitrobenzene as internal standard (0.0395 mmol, integration = 1.58) and the peak (^{19}F NMR, δ = –103.5 ppm) was compared with a previously reported sample.[47]

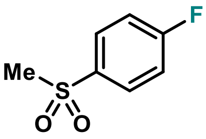

77

2-fluoro-1,3-dimethylbenzene (78): Compound **78** was obtained in 63% yield (6.3 TON) by ^{19}F NMR using 1-fluoro-4-nitrobenzene as internal standard (0.0318 mmol, integration = 1.27) and the peak (^{19}F NMR, δ = –122.7 ppm) was compared with a previously reported sample.[44]

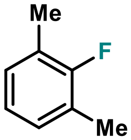

78

Although the reaction proved to be general with a variety of arylboronic pinacol esters, when electron-withdrawing groups were installed in the arene ring the reaction yields decreased dramatically (Figure S78). Similarly, no fluoroarenes were detected when heteroaromatic arylboronic esters were utilized as starting materials. These facts constitute the main limitations of this proof-of-concept bismuth-catalyzed fluorination protocol.

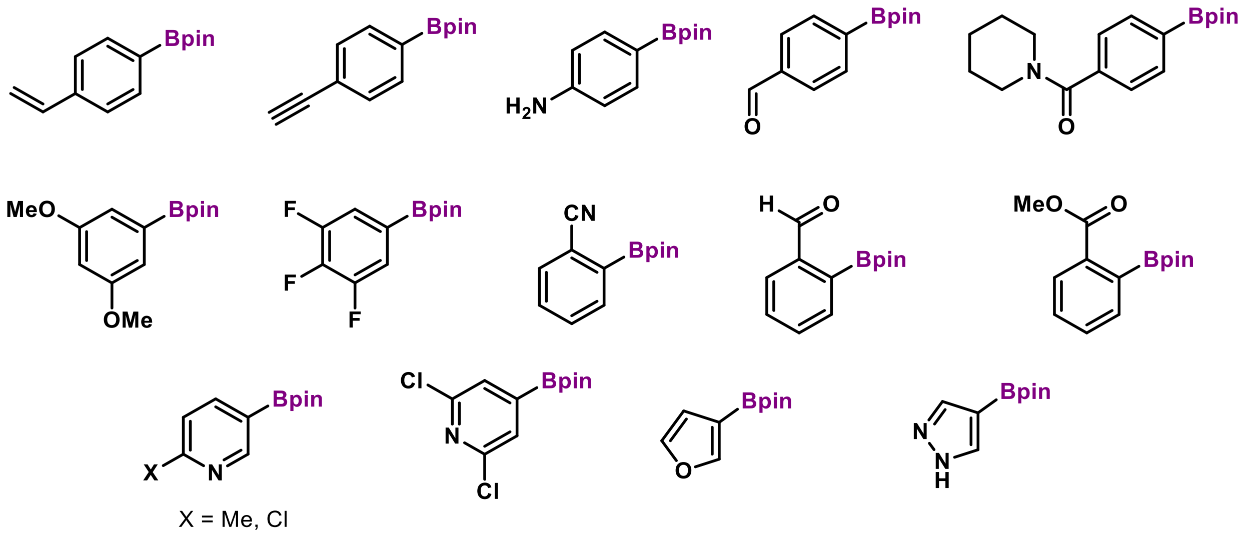

Figure S78. Unsuccessful substrates in the catalytic fluorination reaction.

7.5.1 Isolation several aryl fluorides representative examples

1-(tert-butyl)-4-fluorobenzene (**14**)

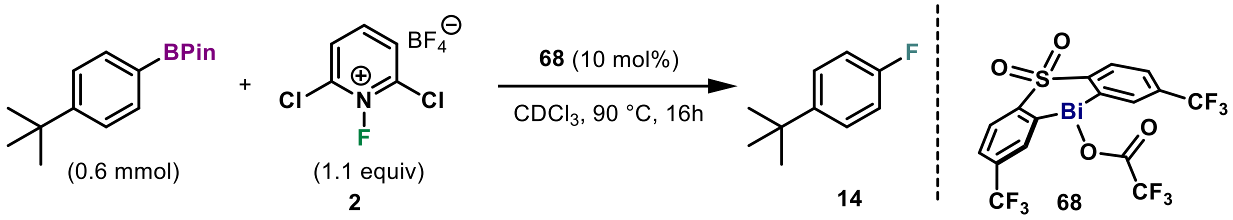

A culture tube equipped with a stir bar and a cap with a plastic seal was charged with 4-(tert-butyl)-phenylboronic acid pinacol ester (0.6 mmol) and **68** (10 mol%). The tube was transferred to a glove box, where 1-fluoro-2,6-dichloropyridinium tetrafluoroborate (1.1 equiv) was added. The tube was removed from the glove box and subjected to a positive pressure of argon. Dry $CDCl_3$ (12.0 mL) was added without piercing the plastic seal and the reaction was then stirred overnight at 90 °C. After that, the mixture was cooled down and concentrated at room temperature. The crude mixture was purified by preparative TLC using hexanes as eluent. The collected product contained protodeboronated impurity, hence the mixture was purified by preparative HPLC (Nucleodur PFP (20×250 mm: 5 µm), MeOH/water (70/30, v/v), flow rate = 20.0 mL/min, 35 ºC) to afford the title compound as a colorless liquid in 13% yield (11.8 mg, 0.078 mmol). Characterization data matched with previously a reported example.[34]

^{1}H NMR - (300 MHz, $CDCl_3$): δ 7.38-7.28 (m, 2H), 7.02-6.92 (m, 2H), 1.31 (s, 9H).

^{13}C NMR - (75 MHz, $CDCl_3$): δ 161.0 (d, J = 243.3 Hz), 146.9, 126.7 (d, J = 7.6 Hz), 114.7 (d, J = 20.8 Hz), 34.5, 31.6.

^{19}F NMR - (282 MHz, $CDCl_3$): δ –118.7.

4-fluoro-1,1'-biphenyl (51)

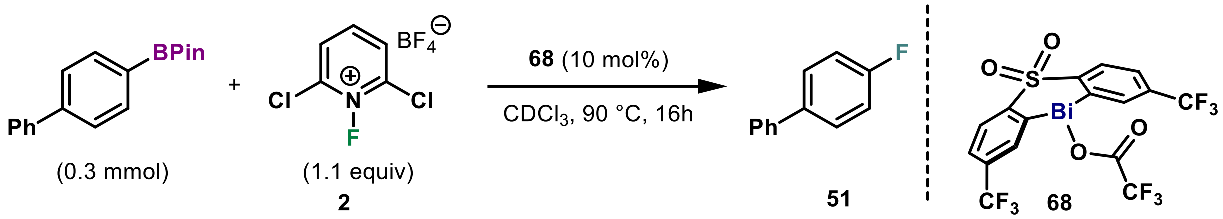

A culture tube equipped with a stir bar and a cap with a plastic seal was charged with 4-biphenylboronic acid pinacol ester (0.3 mmol) and **68** (10 mol%). The tube was transferred to a glove box, where 1-fluoro-2,6-dichloropyridinium tetrafluoroborate (1.1 equiv) was added. The tube was removed from the glove box and subjected to a positive pressure of argon. Dry $CDCl_3$ (6.0 mL) was added without piercing the plastic seal and the reaction was then stirred overnight at 90 °C. After that, the mixture was cooled down and concentrated at room temperature under slightly reduced pressure. The crude mixture was purified by preparative TLC using hexanes as eluent. The collected product contained protodeboronated impurity, hence the mixture was purified by preparative HPLC (Nucleodur PFP (20×250 mm: 5 µm), MeOH/water (60/40, v/v), flow rate = 20.0 mL/min, 35 ºC) to afford the title compound as a white solid in 39% yield (20.1 mg, 0.12 mmol). Characterization data matched with previously a reported example.[35]

^{1}H NMR - (300 MHz, $CDCl_3$): δ 7.59-7.51 (m, 4H), 7.48-7.41 (m, 2H), 7.39-7.31 (m, 1H), 7.19-7.09 (m, 2H).

^{13}C NMR - (75 MHz, $CDCl_3$): δ 162.6 (d, J = 246.3 Hz), 140.4, 137.5 (d, J = 3.3 Hz), 129.0, 128.8 (d, J = 8.0 Hz), 127.4, 127.2, 115.8 (d, J = 21.3 Hz).

^{19}F NMR - (282 MHz, $CDCl_3$): δ –115.9.

8. Crystallographic data

8.1 Single crystal structure analysis of **4·(CH_3CN)$_2$**

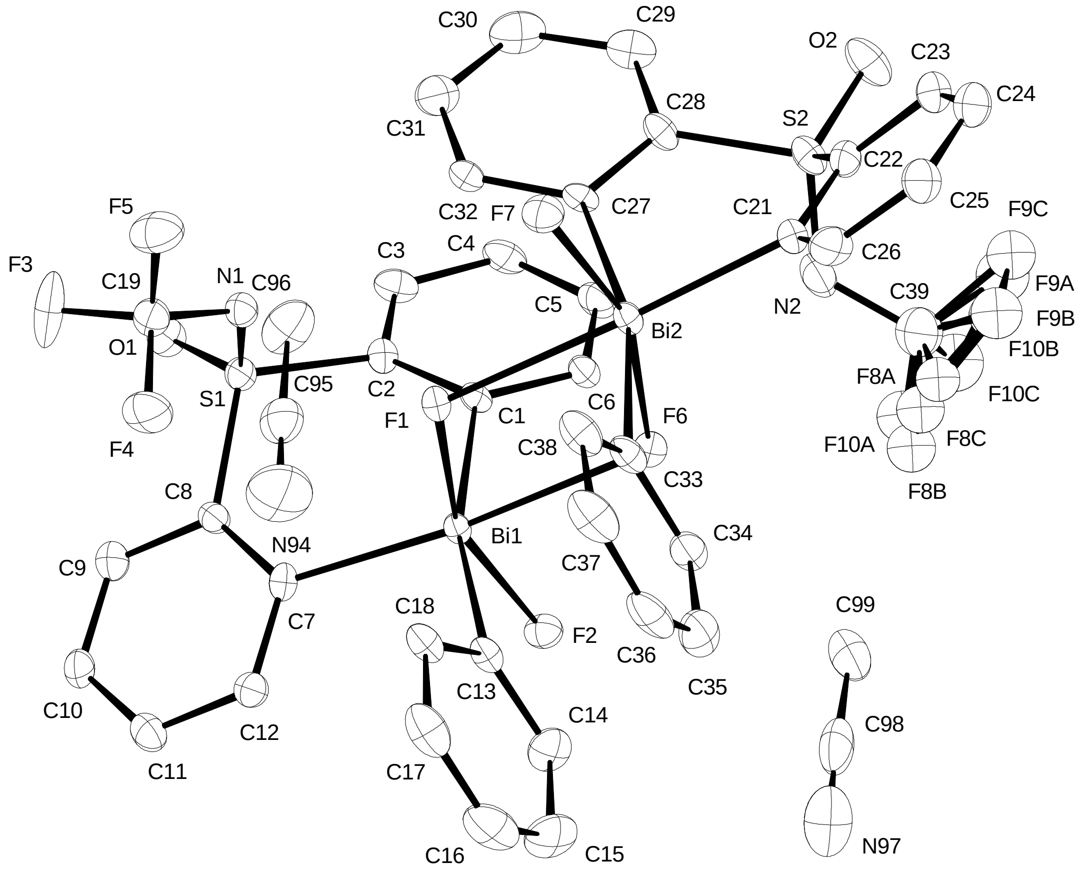

Figure S79. The molecular structure of complex **4·(CH_3CN)$_2$**. H atoms have been removed for clarity.

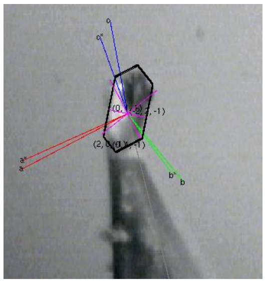

	h	k	l	distance (mm)
1.	0	-1	-1	0.04
2.	0	1	1	0.03
3.	0	-1	1	0.08
4.	0	1	-1	0.06
5.	-2	2	-1	0.04
6.	2	-2	1	0.04
7.	-2	0	1	0.08
8.	2	0	-1	0.07

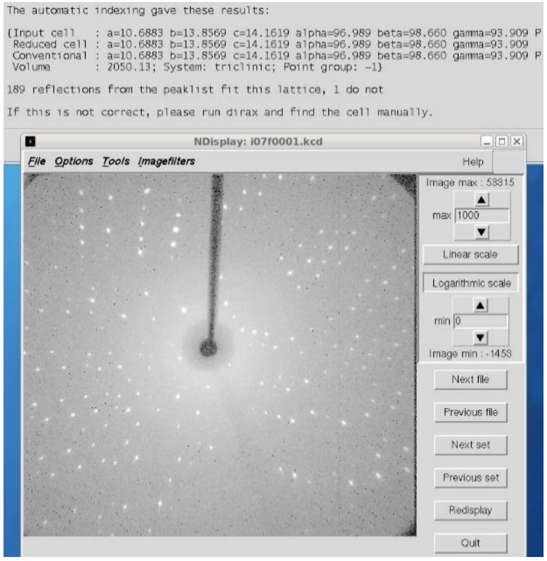

Figure S80. Crystal faces and unit cell determination of **4·(CH_3CN)$_2$**.

INTENSITY STATISTICS FOR DATASET

Resolution	#Data	#Theory	%Complete	Redundancy	Mean I	Mean I/s	Rmerge	Rsigma
Inf - 2.42	292	302	96.7	2.62	96.93	24.42	0.0434	0.0361
2.42 - 1.63	685	687	99.7	2.54	77.93	21.24	0.0354	0.0378
1.63 - 1.29	995	998	99.7	2.49	51.39	19.04	0.0374	0.0410
1.29 - 1.13	977	978	99.9	2.47	40.28	17.32	0.0374	0.0432
1.13 - 1.03	947	948	99.9	2.43	31.58	15.51	0.0425	0.0472
1.03 - 0.95	1064	1067	99.7	2.33	27.86	14.10	0.0434	0.0510
0.95 - 0.90	869	871	99.8	2.20	24.18	13.22	0.0449	0.0561
0.90 - 0.85	1114	1116	99.8	2.10	19.96	11.46	0.0515	0.0630
0.85 - 0.81	1060	1062	99.8	2.01	16.84	10.11	0.0547	0.0717
0.81 - 0.78	960	962	99.8	1.94	15.11	9.04	0.0688	0.0803
0.78 - 0.76	729	730	99.9	1.88	13.70	8.42	0.0769	0.0900
0.76 - 0.73	1247	1250	99.8	1.77	12.21	7.41	0.0822	0.1035
0.73 - 0.71	949	951	99.8	1.71	11.84	6.83	0.0921	0.1155
0.71 - 0.69	1092	1093	99.9	1.66	9.68	5.59	0.1108	0.1475
0.69 - 0.67	1168	1170	99.8	1.57	8.62	4.79	0.1247	0.1811
0.67 - 0.66	647	650	99.5	1.54	8.06	4.38	0.1353	0.2100
0.66 - 0.65	702	703	99.9	1.49	7.12	3.71	0.1627	0.2516
0.65 - 0.63	1529	1529	100.0	1.45	6.51	3.03	0.1737	0.3264
0.63 - 0.62	831	835	99.5	1.36	6.12	2.51	0.1864	0.4053
0.62 - 0.61	915	916	99.9	1.33	5.12	1.99	0.2439	0.5306
0.61 - 0.60	599	678	88.3	1.16	5.17	1.83	0.2352	0.5703
0.70 - 0.60	6960	7051	98.7	1.44	6.91	3.40	0.1576	0.3025
Inf - 0.60	19371	19496	99.4	1.88	20.52	9.27	0.0513	0.0852

One CF_3 group shows a rotational disorder over three positions with 40:40:20 occupancy. Disordered atoms are described by isotropic atomic displacement parameters. The structure contains additionally a disorder solute molecule (acetonitrile) on a crystallographic special position (inversion center). Some reflections are effected by the beam stop. Reflections are counted for which theta > 3 degrees and (Fo**2 - Fc**2) / sqrt(weight) < - 10.0. These reflections are not removed from the final refinement. Anharmonic displacements of the heavy atoms can possibly be the reason for the high residual electron density in the vicinity of the Bi atoms.

Complete .cif-data of the compound are available under the CCDC number **CCDC-2125778.**

Table S27. Crystal data and structure refinement of **4·(CH_3CN)$_2$**.

Identification code	12525sadabs	
Empirical formula	$C_{41} H_{30.50} Bi_2 F_{10} N_{3.50} O_2 S_2$	
Color	colourless	
Formula weight	1276.27 g·mol^{-1}	
Temperature	100(2) K	
Wavelength	0.71073 Å	
Crystal system	Triclinic	
Space group	*P*-1, (no. 2)	
Unit cell dimensions	a = 10.6553(7) Å	α= 96.975(5)°.
	b = 13.8429(14) Å	β= 98.559(5)°.
	c = 14.1333(4) Å	γ = 93.960(5)°.
Volume	2038.1(3) Å^3	
Z	2	
Density (calculated)	2.080 Mg·m^{-3}	
Absorption coefficient	8.812 mm^{-1}	
F(000)	1210 e	
Crystal size	0.15 x 0.14 x 0.07 mm^3	
θ range for data collection	2.677 to 27.499°.	
Index ranges	-12 ≤ h ≤ 13, -17≤ k ≤ 17, -18 ≤ l ≤ 18	
Reflections collected	21135	
Independent reflections	9320 [R_{int} = 0.0425]	
Reflections with I>2σ(I)	7937	
Completeness to θ = 25.242°	99.6 %	
Absorption correction	Gaussian	
Max. and min. transmission	0.58607 and 0.28298	
Refinement method	Full-matrix least-squares on F^2	
Data / restraints / parameters	9320 / 0 / 570	
Goodness-of-fit on F^2	1.050	
Final R indices [I>2σ(I)]	R_1 = 0.0359	wR^2 = 0.0918
R indices (all data)	R_1 = 0.0453	wR^2 = 0.0989
Extinction coefficient	n/a	
Largest diff. peak and hole	2.900 and -3.361 e·Å^{-3}	

Table S28. Bond lengths [Å] and angles [°] of **4·(CH$_3$CN)$_2$**.

Bi(1)-F(1)	2.178(3)	Bi(1)-F(2)	2.093(3)
Bi(1)-F(6)	2.582(3)	Bi(1)-C(1)	2.196(5)
Bi(1)-C(7)	2.234(6)	Bi(1)-C(13)	2.189(5)
Bi(2)-F(1)	2.585(3)	Bi(2)-F(6)	2.185(3)
Bi(2)-F(7)	2.078(3)	Bi(2)-C(21)	2.234(6)
Bi(2)-C(27)	2.201(5)	Bi(2)-C(33)	2.195(6)
S(1)-O(1)	1.447(4)	S(1)-N(1)	1.540(5)
S(1)-C(2)	1.782(6)	S(1)-C(8)	1.768(6)
S(2)-O(2)	1.445(5)	S(2)-N(2)	1.546(5)
S(2)-C(22)	1.776(7)	S(2)-C(28)	1.786(6)
F(3)-C(19)	1.345(7)	F(4)-C(19)	1.353(7)
F(5)-C(19)	1.354(7)	F(8A)-C(39)	1.217(14)
F(8B)-C(39)	1.391(13)	F(8C)-C(39)	1.22(3)
F(9A)-C(39)	1.298(14)	F(9B)-C(39)	1.507(14)
F(9C)-C(39)	1.46(2)	F(10A)-C(39)	1.467(14)
F(10B)-C(39)	1.324(13)	F(10C)-C(39)	1.25(3)
N(1)-C(19)	1.377(7)	N(2)-C(39)	1.375(9)
C(1)-C(2)	1.400(7)	C(1)-C(6)	1.398(8)
C(2)-C(3)	1.382(8)	C(3)-C(4)	1.398(9)
C(4)-C(5)	1.380(9)	C(5)-C(6)	1.387(8)
C(7)-C(8)	1.391(8)	C(7)-C(12)	1.383(8)
C(8)-C(9)	1.395(8)	C(9)-C(10)	1.389(9)
C(10)-C(11)	1.378(9)	C(11)-C(12)	1.396(8)
C(13)-C(14)	1.384(9)	C(13)-C(18)	1.396(8)
C(14)-C(15)	1.382(9)	C(15)-C(16)	1.387(11)
C(16)-C(17)	1.397(11)	C(17)-C(18)	1.379(9)
C(21)-C(22)	1.398(8)	C(21)-C(26)	1.376(9)
C(22)-C(23)	1.384(9)	C(23)-C(24)	1.377(10)
C(24)-C(25)	1.372(10)	C(25)-C(26)	1.391(9)
C(27)-C(28)	1.396(8)	C(27)-C(32)	1.397(8)
C(28)-C(29)	1.373(9)	C(29)-C(30)	1.396(10)
C(30)-C(31)	1.368(10)	C(31)-C(32)	1.399(9)
C(33)-C(34)	1.381(9)	C(33)-C(38)	1.391(9)
C(34)-C(35)	1.389(9)	C(35)-C(36)	1.411(11)
C(36)-C(37)	1.374(11)	C(37)-C(38)	1.394(9)

N(94)-C(95)	1.111(10)	C(95)-C(96)	1.456(10)
N(97)-C(98)	1.14(2)	C(98)-C(99)	1.44(2)
F(1)-Bi(1)-F(6)	69.46(11)	F(1)-Bi(1)-C(1)	92.16(16)
F(1)-Bi(1)-C(7)	105.52(17)	F(1)-Bi(1)-C(13)	88.11(18)
F(2)-Bi(1)-F(1)	157.32(13)	F(2)-Bi(1)-F(6)	87.87(12)
F(2)-Bi(1)-C(1)	84.80(17)	F(2)-Bi(1)-C(7)	97.15(17)
F(2)-Bi(1)-C(13)	89.18(19)	C(1)-Bi(1)-F(6)	81.45(15)
C(1)-Bi(1)-C(7)	96.9(2)	C(7)-Bi(1)-F(6)	174.58(16)
C(13)-Bi(1)-F(6)	84.63(16)	C(13)-Bi(1)-C(1)	165.0(2)
C(13)-Bi(1)-C(7)	97.4(2)	F(6)-Bi(2)-F(1)	69.32(11)
F(6)-Bi(2)-C(21)	111.01(18)	F(6)-Bi(2)-C(27)	90.10(17)
F(6)-Bi(2)-C(33)	86.30(18)	F(7)-Bi(2)-F(1)	88.09(12)
F(7)-Bi(2)-F(6)	157.40(13)	F(7)-Bi(2)-C(21)	91.57(18)
F(7)-Bi(2)-C(27)	86.56(18)	F(7)-Bi(2)-C(33)	90.8(2)
C(21)-Bi(2)-F(1)	176.89(17)	C(27)-Bi(2)-F(1)	81.08(16)
C(27)-Bi(2)-C(21)	95.8(2)	C(33)-Bi(2)-F(1)	83.13(17)
C(33)-Bi(2)-C(21)	100.0(2)	C(33)-Bi(2)-C(27)	164.1(2)
O(1)-S(1)-N(1)	119.6(3)	O(1)-S(1)-C(2)	108.6(3)
O(1)-S(1)-C(8)	108.3(3)	N(1)-S(1)-C(2)	101.3(3)
N(1)-S(1)-C(8)	109.0(3)	C(8)-S(1)-C(2)	109.7(3)
O(2)-S(2)-N(2)	120.8(3)	O(2)-S(2)-C(22)	107.6(3)
O(2)-S(2)-C(28)	108.9(3)	N(2)-S(2)-C(22)	109.6(3)
N(2)-S(2)-C(28)	100.7(3)	C(22)-S(2)-C(28)	108.6(3)
Bi(1)-F(1)-Bi(2)	110.60(12)	Bi(2)-F(6)-Bi(1)	110.49(13)
C(19)-N(1)-S(1)	120.6(4)	C(39)-N(2)-S(2)	120.4(5)
C(2)-C(1)-Bi(1)	121.1(4)	C(6)-C(1)-Bi(1)	118.6(4)
C(6)-C(1)-C(2)	119.3(5)	C(1)-C(2)-S(1)	124.0(4)
C(3)-C(2)-S(1)	114.8(4)	C(3)-C(2)-C(1)	120.8(5)
C(2)-C(3)-C(4)	119.4(6)	C(5)-C(4)-C(3)	120.0(6)
C(4)-C(5)-C(6)	120.9(5)	C(5)-C(6)-C(1)	119.5(5)
C(8)-C(7)-Bi(1)	122.6(4)	C(12)-C(7)-Bi(1)	117.0(4)
C(12)-C(7)-C(8)	119.8(5)	C(7)-C(8)-S(1)	121.8(4)
C(7)-C(8)-C(9)	120.8(5)	C(9)-C(8)-S(1)	116.9(4)
C(10)-C(9)-C(8)	118.9(6)	C(11)-C(10)-C(9)	120.3(6)
C(10)-C(11)-C(12)	120.8(6)	C(7)-C(12)-C(11)	119.3(5)

C(14)-C(13)-Bi(1)	119.5(4)	C(14)-C(13)-C(18)	122.7(5)
C(18)-C(13)-Bi(1)	117.0(4)	C(15)-C(14)-C(13)	117.9(6)
C(14)-C(15)-C(16)	120.8(7)	C(15)-C(16)-C(17)	120.2(6)
C(18)-C(17)-C(16)	120.1(7)	C(17)-C(18)-C(13)	118.2(6)
F(3)-C(19)-F(4)	105.0(5)	F(3)-C(19)-F(5)	105.8(5)
F(3)-C(19)-N(1)	115.7(5)	F(4)-C(19)-F(5)	104.7(5)
F(4)-C(19)-N(1)	115.2(5)	F(5)-C(19)-N(1)	109.6(5)
C(22)-C(21)-Bi(2)	122.9(4)	C(26)-C(21)-Bi(2)	117.3(5)
C(26)-C(21)-C(22)	118.5(6)	C(21)-C(22)-S(2)	119.6(5)
C(23)-C(22)-S(2)	118.4(5)	C(23)-C(22)-C(21)	121.7(6)
C(24)-C(23)-C(22)	119.1(6)	C(25)-C(24)-C(23)	119.6(6)
C(24)-C(25)-C(26)	121.5(6)	C(21)-C(26)-C(25)	119.5(6)
C(28)-C(27)-Bi(2)	121.1(4)	C(28)-C(27)-C(32)	119.1(5)
C(32)-C(27)-Bi(2)	119.2(4)	C(27)-C(28)-S(2)	123.4(5)
C(29)-C(28)-S(2)	114.8(5)	C(29)-C(28)-C(27)	121.5(6)
C(28)-C(29)-C(30)	119.0(6)	C(31)-C(30)-C(29)	120.3(6)
C(30)-C(31)-C(32)	121.0(6)	C(27)-C(32)-C(31)	118.8(6)
C(34)-C(33)-Bi(2)	118.3(5)	C(34)-C(33)-C(38)	123.2(6)
C(38)-C(33)-Bi(2)	117.9(5)	C(33)-C(34)-C(35)	118.7(6)
C(34)-C(35)-C(36)	119.4(7)	C(37)-C(36)-C(35)	120.1(6)
C(36)-C(37)-C(38)	121.5(7)	C(33)-C(38)-C(37)	117.1(7)
F(8A)-C(39)-F(9A)	115.9(11)	F(8A)-C(39)-F(10A)	109.5(10)
F(8A)-C(39)-N(2)	113.7(8)	F(8B)-C(39)-F(9B)	95.4(9)
F(8C)-C(39)-F(9C)	108.6(19)	F(8C)-C(39)-F(10C)	112(2)
F(8C)-C(39)-N(2)	113.3(13)	F(9A)-C(39)-F(10A)	100.3(10)
F(9A)-C(39)-N(2)	113.7(8)	F(10B)-C(39)-F(8B)	106.7(9)
F(10B)-C(39)-F(9B)	99.9(9)	F(10B)-C(39)-N(2)	117.9(8)
F(10C)-C(39)-F(9C)	97.1(18)	F(10C)-C(39)-N(2)	114.6(14)
N(2)-C(39)-F(8B)	115.5(7)	N(2)-C(39)-F(9B)	118.2(7)
N(2)-C(39)-F(9C)	110.3(10)	N(2)-C(39)-F(10A)	101.8(8)
N(94)-C(95)-C(96)	178.4(8)	N(97)-C(98)-C(99)	177.6(18)

8.2 Single crystal structure analysis of **24a**

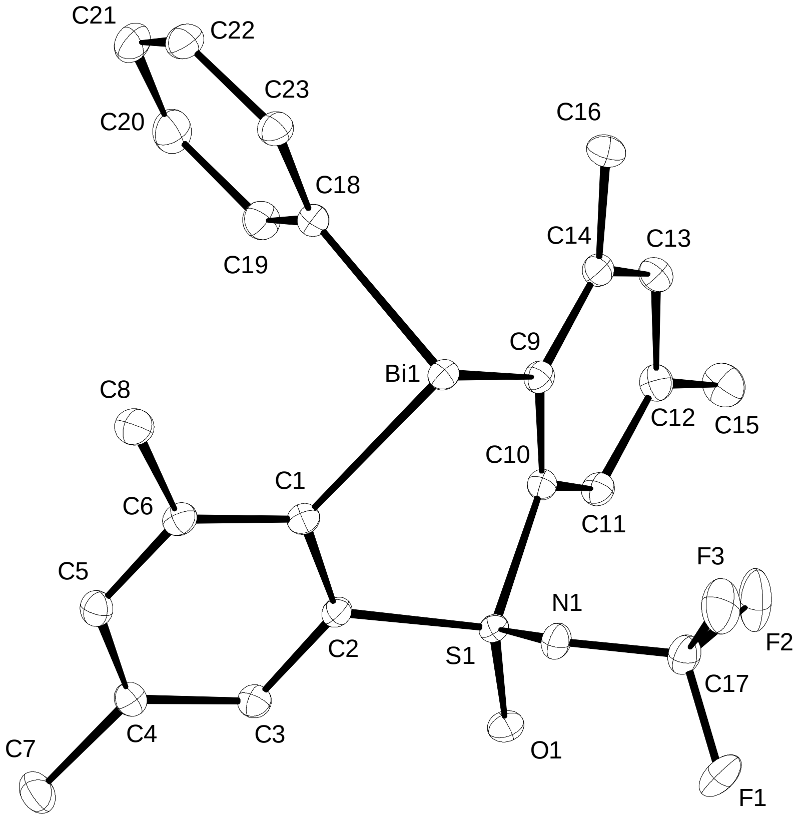

Figure S81. The molecular structure of complex **24a**. H atoms have been removed for clarity.

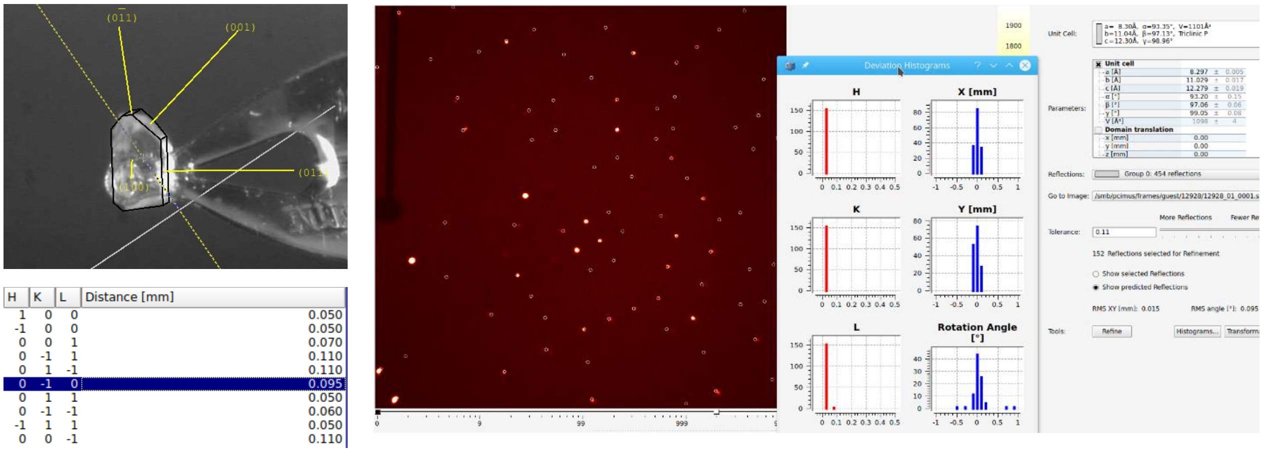

Figure S82. Crystal faces and unit cell determination of **24a**.

INTENSITY STATISTICS FOR DATASET

Resolution	#Data	#Theory	%Complete	Redundancy	Mean I	Mean I/s	Rmerge	Rsigma
Inf - 2.67	229	230	99.6	5.58	214.01	79.64	0.0301	0.0125
2.67 - 1.79	538	544	98.9	6.30	150.96	81.91	0.0258	0.0117
1.79 - 1.42	762	778	97.9	6.47	119.71	80.15	0.0226	0.0115
1.42 - 1.24	775	788	98.4	6.55	92.31	77.20	0.0212	0.0117
1.24 - 1.13	750	758	98.9	6.25	74.28	71.39	0.0224	0.0124
1.13 - 1.05	738	746	98.9	5.28	71.42	65.57	0.0202	0.0139
1.05 - 0.98	879	888	99.0	4.48	59.94	57.39	0.0199	0.0156
0.98 - 0.94	646	650	99.4	4.02	55.63	55.43	0.0194	0.0166
0.94 - 0.89	956	962	99.4	3.65	45.61	49.69	0.0194	0.0179
0.89 - 0.86	668	676	98.8	3.41	39.18	45.71	0.0188	0.0192
0.86 - 0.83	755	762	99.1	3.33	38.82	45.49	0.0193	0.0194
0.83 - 0.80	923	940	98.2	3.22	37.51	43.56	0.0191	0.0202
0.80 - 0.78	642	652	98.5	3.11	33.60	40.86	0.0213	0.0215
0.78 - 0.76	766	780	98.2	3.03	31.35	40.21	0.0210	0.0221
0.76 - 0.74	813	838	97.0	2.92	30.58	38.72	0.0212	0.0225
0.74 - 0.72	916	948	96.6	2.83	25.99	35.16	0.0231	0.0245
0.72 - 0.71	528	542	97.4	2.77	25.07	34.33	0.0224	0.0253
0.71 - 0.69	1035	1074	96.4	2.73	24.45	33.83	0.0234	0.0259
0.69 - 0.68	565	600	94.2	2.57	22.09	30.84	0.0252	0.0285
0.68 - 0.67	627	658	95.3	2.55	20.59	30.44	0.0268	0.0290
0.67 - 0.66	663	702	94.4	2.49	20.25	29.66	0.0266	0.0296
0.76 - 0.66	5147	5362	96.0	2.71	24.48	33.61	0.0236	0.0259
Inf - 0.66	15174	15516	97.8	3.90	52.41	49.69	0.0226	0.0165

Complete .cif-data of the compound are available under the CCDC number **CCDC-2125769.**

Table S29. Crystal data and structure refinement of **24a**.

Identification code	12928sadabs	
Empirical formula	$C_{23} H_{21} Bi F_3 N O S$	
Color	colourless	
Formula weight	625.45 g·mol^{-1}	
Temperature	100(2) K	
Wavelength	0.71073 Å	
Crystal system	Triclinic	
Space group	*P*-1, (no. 2)	
Unit cell dimensions	a = 8.2477(6) Å	α= 92.868(2)°.
	b = 10.9024(7) Å	β= 97.083(2)°.
	c = 12.1136(8) Å	γ = 99.113(2)°.
Volume	1064.60(13) Å^3	
Z	2	
Density (calculated)	1.951 Mg·m^{-3}	
Absorption coefficient	8.419 mm^{-1}	
F(000)	600 e	
Crystal size	0.233 x 0.148 x 0.100 mm^3	
θ range for data collection	1.698 to 32.575°.	
Index ranges	$-12 \le h \le 12$, $-16 \le k \le 16$, $-18 \le l \le 18$	
Reflections collected	60487	
Independent reflections	7754 [R_{int} = 0.0417]	
Reflections with I>2σ(I)	7680	
Completeness to θ = 25.242°	100.0 %	
Absorption correction	Gaussian	
Max. and min. transmission	0.48944 and 0.20785	
Refinement method	Full-matrix least-squares on F^2	
Data / restraints / parameters	7754 / 0 / 275	
Goodness-of-fit on F^2	1.141	
Final R indices [I>2σ(I)]	R_1 = 0.0156	wR^2 = 0.0398
R indices (all data)	R_1 = 0.0158	wR^2 = 0.0399
Extinction coefficient	n/a	
Largest diff. peak and hole	1.728 and -1.687 e·Å^{-3}	

Table S30. Bond lengths [Å] and angles [°] of **24a**.

Bi(1)-C(1)	2.2682(16)	Bi(1)-C(9)	2.2705(16)
Bi(1)-C(18)	2.2722(17)	S(1)-O(1)	1.4487(13)
S(1)-N(1)	1.5465(15)	S(1)-C(2)	1.7656(15)
S(1)-C(10)	1.7736(17)	F(1)-C(17)	1.349(2)
F(2)-C(17)	1.338(2)	F(3)-C(17)	1.351(2)
N(1)-C(17)	1.388(2)	C(1)-C(2)	1.398(2)
C(1)-C(6)	1.406(2)	C(2)-C(3)	1.390(2)
C(3)-C(4)	1.399(2)	C(4)-C(5)	1.397(2)
C(4)-C(7)	1.505(2)	C(5)-C(6)	1.398(2)
C(6)-C(8)	1.509(2)	C(9)-C(10)	1.398(2)
C(9)-C(14)	1.396(2)	C(10)-C(11)	1.394(2)
C(11)-C(12)	1.391(2)	C(12)-C(13)	1.400(2)
C(12)-C(15)	1.509(2)	C(13)-C(14)	1.400(2)
C(14)-C(16)	1.508(2)	C(18)-C(19)	1.395(2)
C(18)-C(23)	1.397(2)	C(19)-C(20)	1.397(3)
C(20)-C(21)	1.389(3)	C(21)-C(22)	1.393(3)
C(22)-C(23)	1.393(2)		
C(1)-Bi(1)-C(9)	91.08(6)	C(1)-Bi(1)-C(18)	89.45(6)
C(9)-Bi(1)-C(18)	90.93(6)	O(1)-S(1)-N(1)	120.83(8)
O(1)-S(1)-C(2)	110.10(8)	O(1)-S(1)-C(10)	108.76(8)
N(1)-S(1)-C(2)	100.08(8)	N(1)-S(1)-C(10)	108.34(8)
C(2)-S(1)-C(10)	107.93(7)	C(17)-N(1)-S(1)	121.13(12)
C(2)-C(1)-Bi(1)	121.00(11)	C(2)-C(1)-C(6)	117.12(15)
C(6)-C(1)-Bi(1)	121.89(12)	C(1)-C(2)-S(1)	119.68(12)
C(3)-C(2)-S(1)	116.36(12)	C(3)-C(2)-C(1)	123.81(14)
C(2)-C(3)-C(4)	118.90(15)	C(3)-C(4)-C(7)	120.98(15)
C(5)-C(4)-C(3)	117.96(15)	C(5)-C(4)-C(7)	121.06(15)
C(4)-C(5)-C(6)	122.95(15)	C(1)-C(6)-C(8)	121.88(15)
C(5)-C(6)-C(1)	119.22(15)	C(5)-C(6)-C(8)	118.90(14)
C(10)-C(9)-Bi(1)	121.63(12)	C(14)-C(9)-Bi(1)	120.68(12)
C(14)-C(9)-C(10)	117.68(15)	C(9)-C(10)-S(1)	118.48(12)
C(11)-C(10)-S(1)	117.49(13)	C(11)-C(10)-C(9)	123.36(15)
C(12)-C(11)-C(10)	118.89(15)	C(11)-C(12)-C(13)	118.28(15)
C(11)-C(12)-C(15)	120.81(16)	C(13)-C(12)-C(15)	120.86(16)

C(12)-C(13)-C(14)	122.63(16)	C(9)-C(14)-C(13)	119.14(15)
C(9)-C(14)-C(16)	121.83(15)	C(13)-C(14)-C(16)	118.98(15)
F(1)-C(17)-F(3)	105.03(15)	F(1)-C(17)-N(1)	112.64(16)
F(2)-C(17)-F(1)	105.89(15)	F(2)-C(17)-F(3)	106.31(15)
F(2)-C(17)-N(1)	117.11(15)	F(3)-C(17)-N(1)	109.02(14)
C(19)-C(18)-Bi(1)	121.58(12)	C(19)-C(18)-C(23)	118.86(16)
C(23)-C(18)-Bi(1)	119.34(12)	C(18)-C(19)-C(20)	120.42(17)
C(21)-C(20)-C(19)	120.36(18)	C(20)-C(21)-C(22)	119.50(17)
C(23)-C(22)-C(21)	120.15(17)	C(22)-C(23)-C(18)	120.67(17)

8.3 Single crystal structure analysis of **24b·$CHCl_3$**

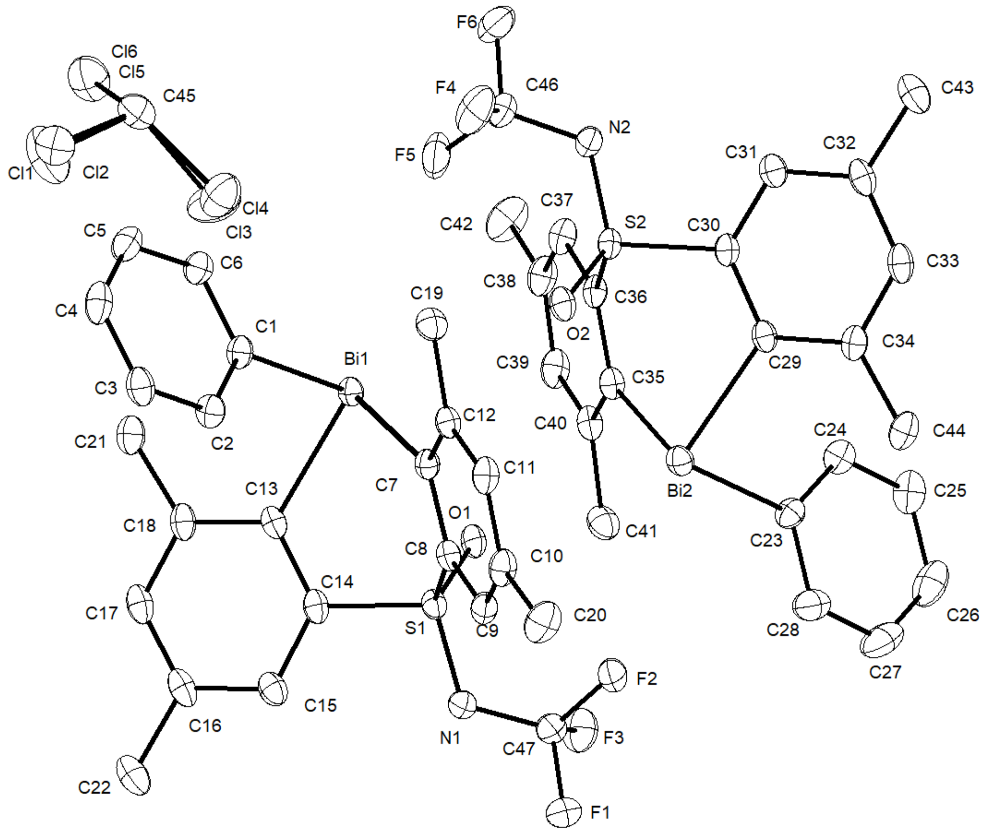

Figure S83. The molecular structure of complex **24b·$CHCl_3$**. H atoms have been removed for clarity.

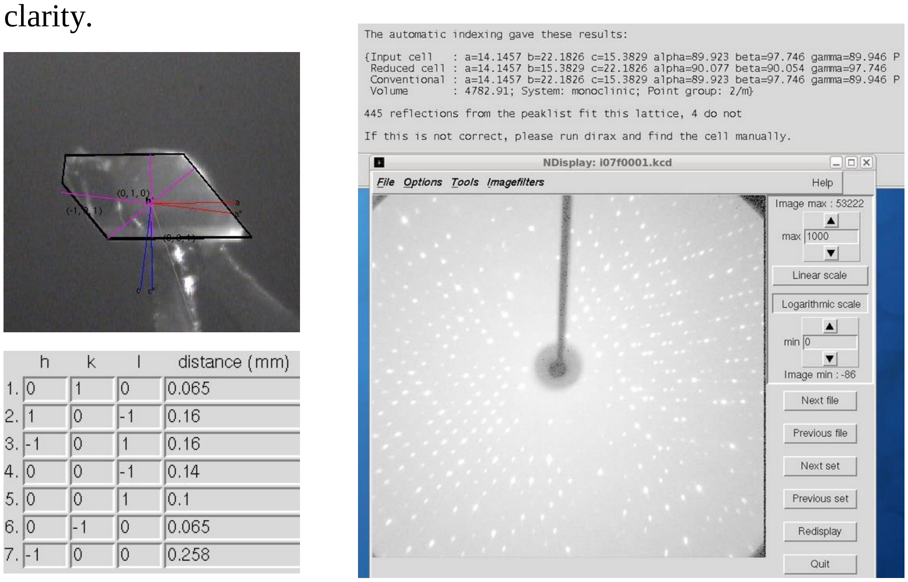

	h	k	l	distance (mm)
1.	0	1	0	0.065
2.	1	0	-1	0.16
3.	-1	0	1	0.16
4.	0	0	-1	0.14
5.	0	0	1	0.1
6.	0	-1	0	0.065
7.	-1	0	0	0.258

Figure S84. Crystal faces and unit cell determination of **24b·$CHCl_3$**.

INTENSITY STATISTICS FOR DATASET

Resolution	#Data	#Theory	%Complete	Redundancy	Mean I	Mean I/s	Rmerge	Rsigma
Inf - 1.96	693	712	97.3	18.78	79.93	117.92	0.0221	0.0103
1.96 - 1.31	1605	1605	100.0	20.97	50.73	112.88	0.0224	0.0074
1.31 - 1.04	2299	2299	100.0	19.38	29.33	94.91	0.0248	0.0080
1.04 - 0.90	2454	2455	100.0	10.14	19.71	59.11	0.0290	0.0130
0.90 - 0.82	2247	2247	100.0	8.40	13.14	42.27	0.0403	0.0174
0.82 - 0.76	2380	2380	100.0	9.75	10.81	38.60	0.0462	0.0191
0.76 - 0.71	2600	2600	100.0	11.08	8.34	34.36	0.0556	0.0222
0.71 - 0.68	1981	1981	100.0	11.50	7.26	31.28	0.0639	0.0256
0.68 - 0.65	2341	2341	100.0	11.35	5.74	25.94	0.0793	0.0324
0.65 - 0.62	2802	2802	100.0	11.04	4.94	21.53	0.0954	0.0397
0.62 - 0.60	2199	2199	100.0	10.79	4.41	18.75	0.1120	0.0469
0.60 - 0.58	2533	2533	100.0	10.43	3.38	14.46	0.1416	0.0641
0.58 - 0.56	2870	2870	100.0	10.13	3.05	12.30	0.1650	0.0756
0.56 - 0.55	1580	1580	100.0	9.88	2.67	10.54	0.1899	0.0896
0.55 - 0.54	1723	1723	100.0	9.62	2.36	9.14	0.2143	0.1039
0.54 - 0.52	3841	3842	100.0	9.03	2.09	7.59	0.2411	0.1248
0.52 - 0.51	2170	2170	100.0	8.67	1.65	5.84	0.2987	0.1651
0.51 - 0.50	2327	2327	100.0	8.46	1.41	4.83	0.3394	0.1992
0.50 - 0.49	2542	2542	100.0	8.25	1.25	4.14	0.3800	0.2310
0.49 - 0.48	2712	2712	100.0	7.96	1.05	3.36	0.4393	0.2838
0.58 - 0.48	19765	19766	100.0	8.96	1.92	7.10	0.2496	0.1384
Inf - 0.48	45899	45920	100.0	10.78	9.31	28.40	0.0479	0.0268

Several low angle reflections have likely been affected by the beam stop and were removed from dataset before final refinement cycles. A resolution cutoff (SHEL 99 0.7) was applied to exclude high-resolution reflections with poorly determined intensities.

Complete .cif-data of the compound are available under the CCDC number **CCDC-2125779.**

Table S31. Crystal data and structure refinement of **24b·CHCl$_3$**.

Identification code	12861sadabs	
Empirical formula	$C_{47} H_{43} Bi_2 Cl_3 F_6 N_2 O_2 S_2$	
Color	colourless	
Formula weight	1370.26 g·mol^{-1}	
Temperature	100(2) K	
Wavelength	0.71073 Å	
Crystal system	Monoclinic	
Space group	$P2_1$/n, (no. 14)	
Unit cell dimensions	a = 14.1325(19) Å	α= 90°.
	b = 22.170(3) Å	β= 97.783(10)°.
	c = 15.3665(14) Å	γ = 90°.
Volume	4770.2(10) Å^3	
Z	4	
Density (calculated)	1.908 Mg·m^{-3}	
Absorption coefficient	7.688 mm^{-1}	
F(000)	2632 e	
Crystal size	0.32 x 0.24 x 0.13 mm^3	
θ range for data collection	3.011 to 30.508°.	
Index ranges	$-20 \le h \le 20$, $-31 \le k \le 31$, $-21 \le l \le 21$	
Reflections collected	192075	
Independent reflections	14549 [R_{int} = 0.0279]	
Reflections with $I>2\sigma(I)$	13843	
Completeness to θ = 25.242°	99.8 %	
Absorption correction	Gaussian	
Max. and min. transmission	0.43629 and 0.09574	
Refinement method	Full-matrix least-squares on F^2	
Data / restraints / parameters	14549 / 0 / 617	
Goodness-of-fit on F^2	1.192	
Final R indices [$I>2\sigma(I)$]	R_1 = 0.0205	wR^2 = 0.0430
R indices (all data)	R_1 = 0.0225	wR^2 = 0.0436
Extinction coefficient	n/a	
Largest diff. peak and hole	0.786 and -0.675 e·Å^{-3}	

Table S32. Bond lengths [Å] and angles [°] of **24b·CHCl₃**.

Bi(1)-C(13)	2.298(2)	Bi(1)-C(7)	2.272(2)
Bi(1)-C(1)	2.265(2)	Bi(2)-C(35)	2.271(2)
Bi(2)-C(23)	2.257(2)	Bi(2)-C(29)	2.288(2)
S(1)-O(1)	1.4563(16)	S(1)-N(1)	1.536(2)
S(1)-C(14)	1.764(2)	S(1)-C(8)	1.772(2)
S(2)-O(2)	1.4550(16)	S(2)-N(2)	1.5358(19)
S(2)-C(36)	1.777(2)	S(2)-C(30)	1.767(2)
Cl(6)-C(45)	1.772(9)	Cl(1)-C(45)	1.83(2)
Cl(3)-C(45)	1.81(2)	F(3)-C(47)	1.352(3)
F(2)-C(47)	1.355(3)	F(1)-C(47)	1.340(3)
F(6)-C(46)	1.336(3)	F(4)-C(46)	1.349(3)
F(5)-C(46)	1.354(3)	C(13)-C(14)	1.388(3)
C(13)-C(18)	1.406(3)	C(35)-C(36)	1.392(3)
C(35)-C(40)	1.402(3)	N(2)-C(46)	1.378(3)
N(1)-C(47)	1.381(3)	C(14)-C(15)	1.395(3)
C(18)-C(17)	1.398(3)	C(18)-C(21)	1.507(3)
C(36)-C(37)	1.393(3)	C(23)-C(24)	1.392(3)
C(23)-C(28)	1.392(3)	C(15)-H(15)	0.9500
C(15)-C(16)	1.394(3)	C(7)-C(8)	1.394(3)
C(7)-C(12)	1.403(3)	C(17)-H(17)	0.9500
C(17)-C(16)	1.392(4)	C(21)-H(21A)	0.9800
C(21)-H(21B)	0.9800	C(21)-H(21C)	0.9800
C(41)-H(41A)	0.9800	C(41)-H(41B)	0.9800
C(41)-H(41C)	0.9800	C(41)-C(40)	1.506(3)
C(16)-C(22)	1.507(3)	C(8)-C(9)	1.390(3)
C(40)-C(39)	1.402(3)	C(11)-H(11)	0.9500
C(11)-C(10)	1.393(3)	C(11)-C(12)	1.399(3)
C(37)-H(37)	0.9500	C(37)-C(38)	1.393(3)
C(39)-H(39)	0.9500	C(39)-C(38)	1.389(3)
C(10)-C(9)	1.391(3)	C(10)-C(20)	1.506(3)
C(9)-H(9)	0.9500	C(38)-C(42)	1.510(3)
C(19)-H(19A)	0.9800	C(19)-H(19B)	0.9800
C(19)-H(19C)	0.9800	C(19)-C(12)	1.504(3)
C(24)-H(24)	0.9500	C(24)-C(25)	1.392(3)
C(28)-H(28)	0.9500	C(28)-C(27)	1.389(4)

C(3)-H(3)	0.9500	C(3)-C(4)	1.389(4)
C(3)-C(2)	1.393(3)	C(29)-C(30)	1.391(3)
C(29)-C(34)	1.409(3)	C(1)-C(6)	1.389(3)
C(1)-C(2)	1.388(3)	C(44)-H(44A)	0.9800
C(44)-H(44B)	0.9800	C(44)-H(44C)	0.9800
C(44)-C(34)	1.506(3)	C(30)-C(31)	1.390(3)
C(25)-H(25)	0.9500	C(25)-C(26)	1.388(4)
C(5)-H(5)	0.9500	C(5)-C(4)	1.384(3)
C(5)-C(6)	1.398(3)	C(33)-H(33)	0.9500
C(33)-C(34)	1.399(3)	C(33)-C(32)	1.393(3)
C(4)-H(4)	0.9500	C(6)-H(6)	0.9500
C(2)-H(2)	0.9500	C(22)-H(22A)	0.9800
C(22)-H(22B)	0.9800	C(22)-H(22C)	0.9800
C(31)-H(31)	0.9500	C(31)-C(32)	1.392(3)
C(20)-H(20A)	0.9800	C(20)-H(20B)	0.9800
C(20)-H(20C)	0.9800	C(32)-C(43)	1.507(3)
C(45)-Cl(5)	1.743(5)	C(45)-Cl(2)	1.739(6)
C(45)-Cl(4)	1.759(6)	C(45)-H(45)	0.94(3)
C(42)-H(42A)	0.9800	C(42)-H(42B)	0.9800
C(42)-H(42C)	0.9800	C(27)-H(27)	0.9500
C(27)-C(26)	1.386(4)	C(43)-H(43A)	0.9800
C(43)-H(43B)	0.9800	C(43)-H(43C)	0.9800
C(26)-H(26)	0.9500		

C(7)-Bi(1)-C(13)	89.88(7)	C(1)-Bi(1)-C(13)	89.97(7)
C(1)-Bi(1)-C(7)	90.78(7)	C(35)-Bi(2)-C(29)	89.99(7)
C(23)-Bi(2)-C(35)	92.58(7)	C(23)-Bi(2)-C(29)	91.29(8)
O(1)-S(1)-N(1)	120.22(10)	O(1)-S(1)-C(14)	107.80(10)
O(1)-S(1)-C(8)	106.31(9)	N(1)-S(1)-C(14)	104.53(10)
N(1)-S(1)-C(8)	111.34(11)	C(14)-S(1)-C(8)	105.72(10)
O(2)-S(2)-N(2)	120.24(10)	O(2)-S(2)-C(36)	106.07(10)
O(2)-S(2)-C(30)	107.83(9)	N(2)-S(2)-C(36)	111.30(10)
N(2)-S(2)-C(30)	104.21(10)	C(30)-S(2)-C(36)	106.39(9)
C(14)-C(13)-Bi(1)	121.15(14)	C(14)-C(13)-C(18)	117.00(19)
C(18)-C(13)-Bi(1)	121.85(16)	C(36)-C(35)-Bi(2)	119.51(14)
C(36)-C(35)-C(40)	117.96(19)	C(40)-C(35)-Bi(2)	122.45(16)

C(46)-N(2)-S(2)	121.06(16)	C(47)-N(1)-S(1)	120.51(16)
C(13)-C(14)-S(1)	115.91(16)	C(13)-C(14)-C(15)	124.22(19)
C(15)-C(14)-S(1)	119.52(17)	C(13)-C(18)-C(21)	122.1(2)
C(17)-C(18)-C(13)	119.0(2)	C(17)-C(18)-C(21)	118.84(19)
C(35)-C(36)-S(2)	117.68(15)	C(35)-C(36)-C(37)	123.47(19)
C(37)-C(36)-S(2)	118.75(17)	C(24)-C(23)-Bi(2)	122.54(16)
C(28)-C(23)-Bi(2)	118.08(17)	C(28)-C(23)-C(24)	119.3(2)
C(14)-C(15)-H(15)	120.8	C(16)-C(15)-C(14)	118.4(2)
C(16)-C(15)-H(15)	120.8	C(8)-C(7)-Bi(1)	119.50(14)
C(8)-C(7)-C(12)	118.00(18)	C(12)-C(7)-Bi(1)	122.33(15)
C(18)-C(17)-H(17)	118.5	C(16)-C(17)-C(18)	123.1(2)
C(16)-C(17)-H(17)	118.5	C(18)-C(21)-H(21A)	109.5
C(18)-C(21)-H(21B)	109.5	C(18)-C(21)-H(21C)	109.5
H(21A)-C(21)-H(21B)	109.5	H(21A)-C(21)-H(21C)	109.5
H(21B)-C(21)-H(21C)	109.5	H(41A)-C(41)-H(41B)	109.5
H(41A)-C(41)-H(41C)	109.5	H(41B)-C(41)-H(41C)	109.5
C(40)-C(41)-H(41A)	109.5	C(40)-C(41)-H(41B)	109.5
C(40)-C(41)-H(41C)	109.5	C(15)-C(16)-C(22)	120.3(2)
C(17)-C(16)-C(15)	118.2(2)	C(17)-C(16)-C(22)	121.5(2)
C(7)-C(8)-S(1)	117.73(15)	C(9)-C(8)-S(1)	119.14(17)
C(9)-C(8)-C(7)	123.07(19)	C(35)-C(40)-C(41)	122.5(2)
C(39)-C(40)-C(35)	118.2(2)	C(39)-C(40)-C(41)	119.34(19)
C(10)-C(11)-H(11)	118.4	C(10)-C(11)-C(12)	123.2(2)
C(12)-C(11)-H(11)	118.4	C(36)-C(37)-H(37)	120.7
C(38)-C(37)-C(36)	118.6(2)	C(38)-C(37)-H(37)	120.7
F(3)-C(47)-F(2)	104.55(18)	F(3)-C(47)-N(1)	113.7(2)
F(2)-C(47)-N(1)	116.28(19)	F(1)-C(47)-F(3)	106.13(18)
F(1)-C(47)-F(2)	106.31(19)	F(1)-C(47)-N(1)	109.20(19)
C(40)-C(39)-H(39)	118.3	C(38)-C(39)-C(40)	123.4(2)
C(38)-C(39)-H(39)	118.3	C(11)-C(10)-C(20)	121.3(2)
C(9)-C(10)-C(11)	118.0(2)	C(9)-C(10)-C(20)	120.6(2)
C(8)-C(9)-C(10)	119.2(2)	C(8)-C(9)-H(9)	120.4
C(10)-C(9)-H(9)	120.4	C(37)-C(38)-C(42)	120.8(2)
C(39)-C(38)-C(37)	118.3(2)	C(39)-C(38)-C(42)	120.9(2)
H(19A)-C(19)-H(19B)	109.5	H(19A)-C(19)-H(19C)	109.5
H(19B)-C(19)-H(19C)	109.5	C(12)-C(19)-H(19A)	109.5

C(12)-C(19)-H(19B)	109.5	C(12)-C(19)-H(19C)	109.5
C(23)-C(24)-H(24)	119.8	C(25)-C(24)-C(23)	120.3(2)
C(25)-C(24)-H(24)	119.8	C(23)-C(28)-H(28)	119.8
C(27)-C(28)-C(23)	120.4(2)	C(27)-C(28)-H(28)	119.8
C(7)-C(12)-C(19)	122.66(19)	C(11)-C(12)-C(7)	118.4(2)
C(11)-C(12)-C(19)	118.92(19)	C(4)-C(3)-H(3)	120.1
C(4)-C(3)-C(2)	119.9(2)	C(2)-C(3)-H(3)	120.1
C(30)-C(29)-Bi(2)	120.72(14)	C(30)-C(29)-C(34)	117.09(19)
C(34)-C(29)-Bi(2)	122.13(16)	C(6)-C(1)-Bi(1)	119.42(15)
C(2)-C(1)-Bi(1)	121.19(16)	C(2)-C(1)-C(6)	119.34(19)
H(44A)-C(44)-H(44B)	109.5	H(44A)-C(44)-H(44C)	109.5
H(44B)-C(44)-H(44C)	109.5	C(34)-C(44)-H(44A)	109.5
C(34)-C(44)-H(44B)	109.5	C(34)-C(44)-H(44C)	109.5
C(29)-C(30)-S(2)	116.56(15)	C(31)-C(30)-S(2)	118.99(16)
C(31)-C(30)-C(29)	124.11(19)	C(24)-C(25)-H(25)	120.0
C(26)-C(25)-C(24)	119.9(2)	C(26)-C(25)-H(25)	120.0
C(4)-C(5)-H(5)	120.0	C(4)-C(5)-C(6)	120.0(2)
C(6)-C(5)-H(5)	120.0	F(6)-C(46)-F(4)	105.62(19)
F(6)-C(46)-F(5)	105.95(19)	F(6)-C(46)-N(2)	109.71(19)
F(4)-C(46)-F(5)	104.8(2)	F(4)-C(46)-N(2)	113.88(19)
F(5)-C(46)-N(2)	116.12(19)	C(34)-C(33)-H(33)	118.5
C(32)-C(33)-H(33)	118.5	C(32)-C(33)-C(34)	123.0(2)
C(3)-C(4)-H(4)	120.0	C(5)-C(4)-C(3)	120.0(2)
C(5)-C(4)-H(4)	120.0	C(1)-C(6)-C(5)	120.3(2)
C(1)-C(6)-H(6)	119.8	C(5)-C(6)-H(6)	119.8
C(3)-C(2)-H(2)	119.8	C(1)-C(2)-C(3)	120.5(2)
C(1)-C(2)-H(2)	119.8	C(16)-C(22)-H(22A)	109.5
C(16)-C(22)-H(22B)	109.5	C(16)-C(22)-H(22C)	109.5
H(22A)-C(22)-H(22B)	109.5	H(22A)-C(22)-H(22C)	109.5
H(22B)-C(22)-H(22C)	109.5	C(30)-C(31)-H(31)	120.7
C(30)-C(31)-C(32)	118.6(2)	C(32)-C(31)-H(31)	120.7
C(29)-C(34)-C(44)	123.3(2)	C(33)-C(34)-C(29)	118.9(2)
C(33)-C(34)-C(44)	117.86(19)	C(10)-C(20)-H(20A)	109.5
C(10)-C(20)-H(20B)	109.5	C(10)-C(20)-H(20C)	109.5
H(20A)-C(20)-H(20B)	109.5	H(20A)-C(20)-H(20C)	109.5
H(20B)-C(20)-H(20C)	109.5	C(33)-C(32)-C(43)	121.1(2)

C(31)-C(32)-C(33)	118.3(2)	C(31)-C(32)-C(43)	120.6(2)
Cl(6)-C(45)-Cl(1)	110.3(7)	Cl(6)-C(45)-Cl(3)	105.6(12)
Cl(6)-C(45)-H(45)	108.7(18)	Cl(1)-C(45)-H(45)	112(2)
Cl(3)-C(45)-Cl(1)	108.0(10)	Cl(3)-C(45)-H(45)	112(2)
Cl(5)-C(45)-Cl(4)	112.5(4)	Cl(5)-C(45)-H(45)	108.1(18)
Cl(2)-C(45)-Cl(5)	112.3(2)	Cl(2)-C(45)-Cl(4)	110.9(2)
Cl(2)-C(45)-H(45)	107.3(18)	Cl(4)-C(45)-H(45)	105.4(18)
C(38)-C(42)-H(42A)	109.5	C(38)-C(42)-H(42B)	109.5
C(38)-C(42)-H(42C)	109.5	H(42A)-C(42)-H(42B)	109.5
H(42A)-C(42)-H(42C)	109.5	H(42B)-C(42)-H(42C)	109.5
C(28)-C(27)-H(27)	120.0	C(26)-C(27)-C(28)	120.0(2)
C(26)-C(27)-H(27)	120.0	C(32)-C(43)-H(43A)	109.5
C(32)-C(43)-H(43B)	109.5	C(32)-C(43)-H(43C)	109.5
H(43A)-C(43)-H(43B)	109.5	H(43A)-C(43)-H(43C)	109.5
H(43B)-C(43)-H(43C)	109.5	C(25)-C(26)-H(26)	120.0
C(27)-C(26)-C(25)	120.0(2)	C(27)-C(26)-H(26)	120.0

8.4 Single crystal structure analysis of **26·($CHCl_3$)$_2$**

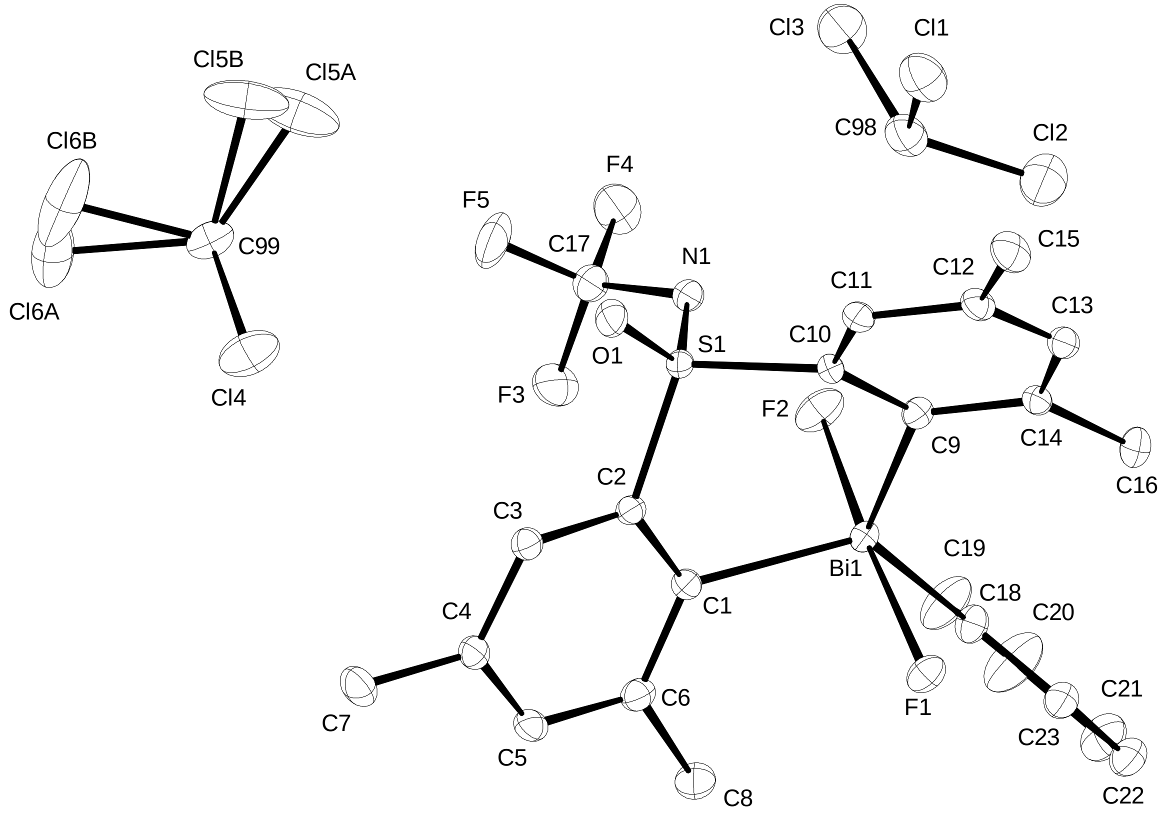

Figure S85. The molecular structure of complex **26·($CHCl_3$)$_2$**. H atoms have been removed for clarity.

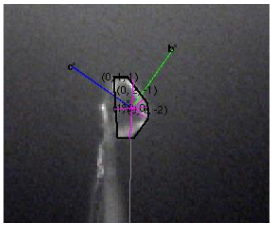

	h	k	l	distance (mm)
1.	-1	0	0	0.055
2.	1	0	0	0.055
3.	0	-1	2	0.055
4.	0	1	-2	0.055
5.	0	-1	-1	0.105
6.	0	1	1	0.105
7.	0	2	-1	0.055
8.	0	0	-1	0.065

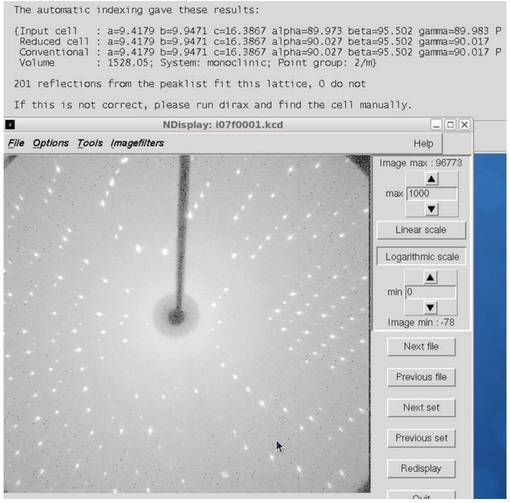

Figure S86. Crystal faces and unit cell determination of **26·($CHCl_3$)$_2$**.

INTENSITY STATISTICS FOR DATASET

Resolution	#Data	#Theory	%Complete	Redundancy	Mean I	Mean I/s	Rmerge	Rsigma
Inf - 2.37	225	274	82.1	2.45	180.34	32.53	0.0526	0.0293
2.37 - 1.64	523	529	98.9	3.43	211.18	36.70	0.0459	0.0266
1.64 - 1.32	738	738	100.0	3.57	168.27	37.00	0.0368	0.0251
1.32 - 1.15	740	740	100.0	3.49	118.01	35.65	0.0309	0.0261
1.15 - 1.04	768	769	99.9	3.43	97.25	34.75	0.0287	0.0264
1.04 - 0.97	708	708	100.0	3.21	84.33	32.05	0.0280	0.0280
0.97 - 0.91	759	759	100.0	3.08	71.88	30.31	0.0273	0.0292
0.91 - 0.86	818	819	99.9	2.95	60.30	28.48	0.0289	0.0310
0.86 - 0.82	797	800	99.6	2.80	53.05	27.19	0.0298	0.0328
0.82 - 0.79	710	716	99.2	2.65	46.21	25.27	0.0298	0.0356
0.79 - 0.76	799	807	99.0	2.53	41.36	23.37	0.0307	0.0372
0.76 - 0.74	673	683	98.5	2.47	38.41	23.32	0.0324	0.0386
0.74 - 0.72	661	673	98.2	2.37	35.36	21.85	0.0345	0.0412
0.72 - 0.70	802	816	98.3	2.26	32.20	20.37	0.0346	0.0432
0.70 - 0.68	811	830	97.7	2.20	27.91	18.90	0.0387	0.0469
0.68 - 0.66	957	985	97.2	2.05	26.76	18.11	0.0417	0.0497
0.66 - 0.65	537	554	96.9	2.00	24.04	17.03	0.0444	0.0534
0.65 - 0.64	566	586	96.6	1.96	20.95	15.88	0.0497	0.0573
0.64 - 0.62	1183	1231	96.1	1.87	20.07	14.93	0.0538	0.0605
0.62 - 0.61	656	697	94.1	1.77	17.41	13.60	0.0600	0.0665
0.61 - 0.60	367	456	80.5	1.32	15.50	11.92	0.0626	0.0782
0.70 - 0.60	5077	5339	95.1	1.92	22.43	16.10	0.0467	0.0557
Inf - 0.60	14798	15170	97.5	2.57	60.67	24.49	0.0354	0.0328

The structure contains a partially disorder solute molecule (trichloromethane).

Complete .cif-data of the compound are available under the CCDC number **CCDC-2125783.**

Table S33. Crystal data and structure refinement of **26·(CHCl$_3$)$_2$**.

Identification code	12872sadabs	
Empirical formula	$C_{25} H_{23} Bi Cl_6 F_5 N O S$	
Color	colourless	
Formula weight	902.18 g·mol^{-1}	
Temperature	100(2) K	
Wavelength	0.71073 Å	
Crystal system	Monoclinic	
Space group	*P*c, (no. 7)	
Unit cell dimensions	a = 9.4031(16) Å	α= 90°.
	b = 9.9413(8) Å	β= 95.557(8)°.
	c = 16.3864(7) Å	γ = 90°.
Volume	1524.6(3) Å^3	
Z	2	
Density (calculated)	1.965 Mg·m^{-3}	
Absorption coefficient	6.429 mm^{-1}	
F(000)	868 e	
Crystal size	0.21 x 0.11 x 0.11 mm^3	
θ range for data collection	2.989 to 30.508°.	
Index ranges	-13 ≤ h ≤ 13, -14≤ k ≤ 14, -23 ≤ l ≤ 23	
Reflections collected	27968	
Independent reflections	9241 [R_{int} = 0.0344]	
Reflections with I>2σ(I)	9137	
Completeness to θ = 25.242°	99.2 %	
Absorption correction	Gaussian	
Max. and min. transmission	0.38753 and 0.17189	
Refinement method	Full-matrix least-squares on F^2	
Data / restraints / parameters	9241 / 2 / 383	
Goodness-of-fit on F^2	1.058	
Final R indices [I>2σ(I)]	R_1 = 0.0191	wR^2 = 0.0484
R indices (all data)	R_1 = 0.0195	wR^2 = 0.0487
Absolute structure parameter	-0.0182(16)	
Extinction coefficient	n/a	
Largest diff. peak and hole	2.162 and -2.446 e·Å^{-3}	

Table S34. Bond lengths [Å] and angles [°] of **26·$(CHCl_3)_2$**.

Bi(1)-F(1)	2.137(2)	Bi(1)-F(2)	2.090(2)
Bi(1)-C(1)	2.201(3)	Bi(1)-C(9)	2.190(4)
Bi(1)-C(18)	2.194(3)	S(1)-O(1)	1.443(3)
S(1)-N(1)	1.538(3)	S(1)-C(2)	1.775(3)
S(1)-C(10)	1.772(3)	F(3)-C(17)	1.354(4)
F(4)-C(17)	1.336(5)	F(5)-C(17)	1.344(5)
N(1)-C(17)	1.394(4)	C(1)-C(2)	1.407(4)
C(1)-C(6)	1.394(5)	C(2)-C(3)	1.389(4)
C(3)-H(3)	0.9500	C(3)-C(4)	1.402(4)
C(4)-C(5)	1.391(5)	C(4)-C(7)	1.496(5)
C(5)-H(5)	0.9500	C(5)-C(6)	1.407(5)
C(6)-C(8)	1.504(6)	C(7)-H(7A)	0.9800
C(7)-H(7B)	0.9800	C(7)-H(7C)	0.9800
C(8)-H(8A)	0.9800	C(8)-H(8B)	0.9800
C(8)-H(8C)	0.9800	C(9)-C(10)	1.392(5)
C(9)-C(14)	1.400(5)	C(10)-C(11)	1.387(4)
C(11)-H(11)	0.9500	C(11)-C(12)	1.406(5)
C(12)-C(13)	1.385(5)	C(12)-C(15)	1.507(5)
C(13)-H(13)	0.9500	C(13)-C(14)	1.406(5)
C(14)-C(16)	1.502(5)	C(15)-H(15A)	0.9800
C(15)-H(15B)	0.9800	C(15)-H(15C)	0.9800
C(16)-H(16A)	0.9800	C(16)-H(16B)	0.9800
C(16)-H(16C)	0.9800	C(18)-C(19)	1.386(5)
C(18)-C(23)	1.389(5)	C(19)-H(19)	0.9500
C(19)-C(20)	1.393(6)	C(20)-H(20)	0.9500
C(20)-C(21)	1.380(7)	C(21)-H(21)	0.9500
C(21)-C(22)	1.384(6)	C(22)-H(22)	0.9500
C(22)-C(23)	1.389(5)	C(23)-H(23)	0.9500
Cl(4)-C(99)	1.749(4)	Cl(5A)-C(99)	1.817(8)
Cl(5B)-C(99)	1.716(7)	Cl(6A)-C(99)	1.795(7)
Cl(6B)-C(99)	1.708(7)	C(99)-H(99A)	1.0000
C(99)-H(99)	1.0000	Cl(1)-C(98)	1.762(4)
Cl(2)-C(98)	1.758(5)	Cl(3)-C(98)	1.763(4)
C(98)-H(98)	1.0000		

F(1)-Bi(1)-C(1)	86.00(10)	F(1)-Bi(1)-C(9)	86.32(11)
F(1)-Bi(1)-C(18)	85.18(11)	F(2)-Bi(1)-F(1)	172.76(9)
F(2)-Bi(1)-C(1)	99.45(11)	F(2)-Bi(1)-C(9)	97.30(12)
F(2)-Bi(1)-C(18)	87.69(11)	C(9)-Bi(1)-C(1)	100.36(13)
C(9)-Bi(1)-C(18)	129.52(13)	C(18)-Bi(1)-C(1)	128.43(12)
O(1)-S(1)-N(1)	120.89(17)	O(1)-S(1)-C(2)	107.63(16)
O(1)-S(1)-C(10)	109.33(16)	N(1)-S(1)-C(2)	109.27(15)
N(1)-S(1)-C(10)	100.42(16)	C(10)-S(1)-C(2)	108.71(15)
C(17)-N(1)-S(1)	121.5(3)	C(2)-C(1)-Bi(1)	118.6(2)
C(6)-C(1)-Bi(1)	120.8(2)	C(6)-C(1)-C(2)	119.7(3)
C(1)-C(2)-S(1)	122.3(2)	C(3)-C(2)-S(1)	116.0(2)
C(3)-C(2)-C(1)	121.5(3)	C(2)-C(3)-H(3)	120.2
C(2)-C(3)-C(4)	119.7(3)	C(4)-C(3)-H(3)	120.2
C(3)-C(4)-C(7)	120.0(3)	C(5)-C(4)-C(3)	118.2(3)
C(5)-C(4)-C(7)	121.8(3)	C(4)-C(5)-H(5)	118.4
C(4)-C(5)-C(6)	123.1(3)	C(6)-C(5)-H(5)	118.4
C(1)-C(6)-C(5)	117.7(3)	C(1)-C(6)-C(8)	122.8(4)
C(5)-C(6)-C(8)	119.5(3)	C(4)-C(7)-H(7A)	109.5
C(4)-C(7)-H(7B)	109.5	C(4)-C(7)-H(7C)	109.5
H(7A)-C(7)-H(7B)	109.5	H(7A)-C(7)-H(7C)	109.5
H(7B)-C(7)-H(7C)	109.5	C(6)-C(8)-H(8A)	109.5
C(6)-C(8)-H(8B)	109.5	C(6)-C(8)-H(8C)	109.5
H(8A)-C(8)-H(8B)	109.5	H(8A)-C(8)-H(8C)	109.5
H(8B)-C(8)-H(8C)	109.5	C(10)-C(9)-Bi(1)	118.1(3)
C(10)-C(9)-C(14)	120.1(3)	C(14)-C(9)-Bi(1)	120.3(3)
C(9)-C(10)-S(1)	122.5(3)	C(11)-C(10)-S(1)	115.8(2)
C(11)-C(10)-C(9)	121.6(3)	C(10)-C(11)-H(11)	120.3
C(10)-C(11)-C(12)	119.4(3)	C(12)-C(11)-H(11)	120.3
C(11)-C(12)-C(15)	120.5(3)	C(13)-C(12)-C(11)	118.2(3)
C(13)-C(12)-C(15)	121.3(3)	C(12)-C(13)-H(13)	118.4
C(12)-C(13)-C(14)	123.2(3)	C(14)-C(13)-H(13)	118.4
C(9)-C(14)-C(13)	117.3(3)	C(9)-C(14)-C(16)	122.8(3)
C(13)-C(14)-C(16)	119.9(3)	C(12)-C(15)-H(15A)	109.5
C(12)-C(15)-H(15B)	109.5	C(12)-C(15)-H(15C)	109.5
H(15A)-C(15)-H(15B)	109.5	H(15A)-C(15)-H(15C)	109.5
H(15B)-C(15)-H(15C)	109.5	C(14)-C(16)-H(16A)	109.5

C(14)-C(16)-H(16B)	109.5	C(14)-C(16)-H(16C)	109.5
H(16A)-C(16)-H(16B)	109.5	H(16A)-C(16)-H(16C)	109.5
H(16B)-C(16)-H(16C)	109.5	F(3)-C(17)-N(1)	114.7(3)
F(4)-C(17)-F(3)	105.5(3)	F(4)-C(17)-F(5)	106.8(3)
F(4)-C(17)-N(1)	109.5(3)	F(5)-C(17)-F(3)	105.0(3)
F(5)-C(17)-N(1)	114.7(4)	C(19)-C(18)-Bi(1)	118.6(3)
C(19)-C(18)-C(23)	122.9(3)	C(23)-C(18)-Bi(1)	118.5(2)
C(18)-C(19)-H(19)	121.3	C(18)-C(19)-C(20)	117.4(4)
C(20)-C(19)-H(19)	121.3	C(19)-C(20)-H(20)	119.7
C(21)-C(20)-C(19)	120.6(4)	C(21)-C(20)-H(20)	119.7
C(20)-C(21)-H(21)	119.5	C(20)-C(21)-C(22)	121.1(4)
C(22)-C(21)-H(21)	119.5	C(21)-C(22)-H(22)	120.2
C(21)-C(22)-C(23)	119.6(4)	C(23)-C(22)-H(22)	120.2
C(18)-C(23)-C(22)	118.4(3)	C(18)-C(23)-H(23)	120.8
C(22)-C(23)-H(23)	120.8	Cl(4)-C(99)-Cl(5A)	105.7(3)
Cl(4)-C(99)-Cl(6A)	105.0(3)	Cl(4)-C(99)-H(99A)	109.0
Cl(4)-C(99)-H(99)	108.9	Cl(5A)-C(99)-H(99)	108.9
Cl(5B)-C(99)-Cl(4)	114.8(3)	Cl(5B)-C(99)-Cl(6A)	109.9(3)
Cl(5B)-C(99)-H(99A)	109.0	Cl(6A)-C(99)-H(99A)	109.0
Cl(6B)-C(99)-Cl(4)	114.7(3)	Cl(6B)-C(99)-Cl(5A)	109.5(4)
Cl(6B)-C(99)-H(99)	108.9	Cl(1)-C(98)-Cl(3)	110.6(2)
Cl(1)-C(98)-H(98)	108.7	Cl(2)-C(98)-Cl(1)	110.5(2)
Cl(2)-C(98)-Cl(3)	109.6(2)	Cl(2)-C(98)-H(98)	108.7
Cl(3)-C(98)-H(98)	108.7		

8.5 Single crystal structure analysis of **28·CHCl3**

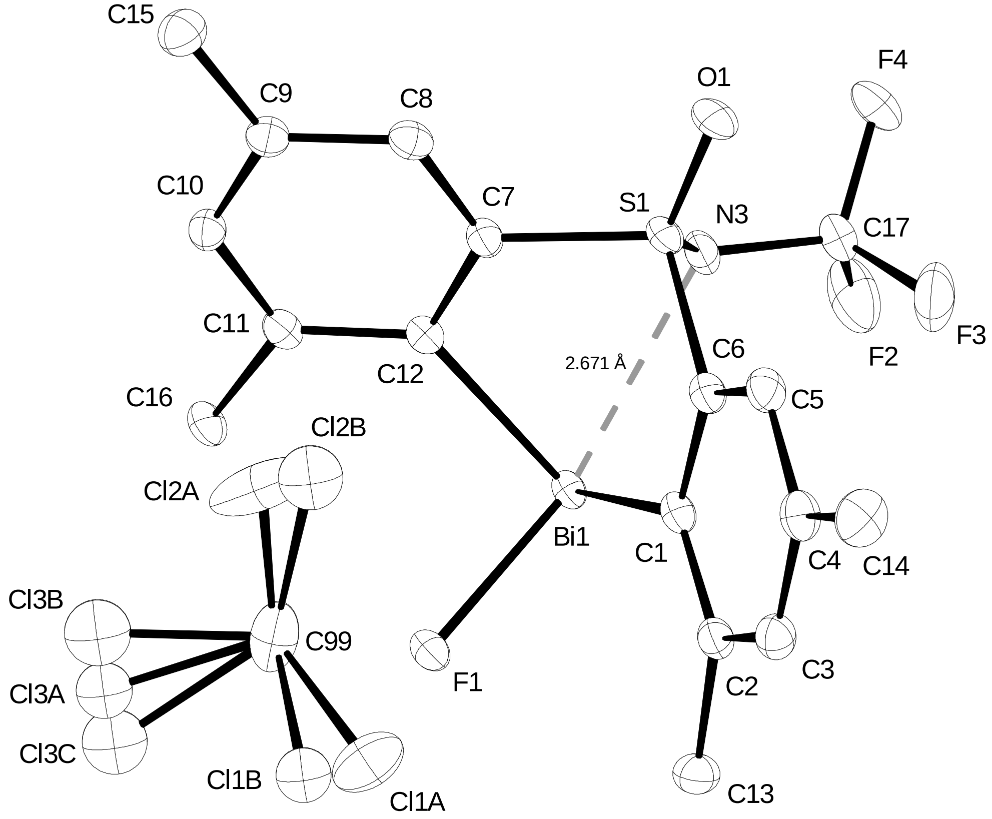

Figure S87. The molecular structure of complex **28·CHCl3**. H atoms have been removed for clarity.

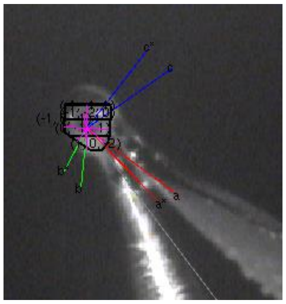

	h	k	l	distance (mm)
1.	0	-1	-1	0.04
2.	0	1	1	0.04
3.	1	0	1	0.05
4.	-1	0	-1	0.05
5.	-1	-1	1	0.05
6.	1	1	-1	0.05
7.	0	1	-2	0.035
8.	1	0	-2	0.035
9.	-1	-2	0	0.045
10.	3	1	1	0.055

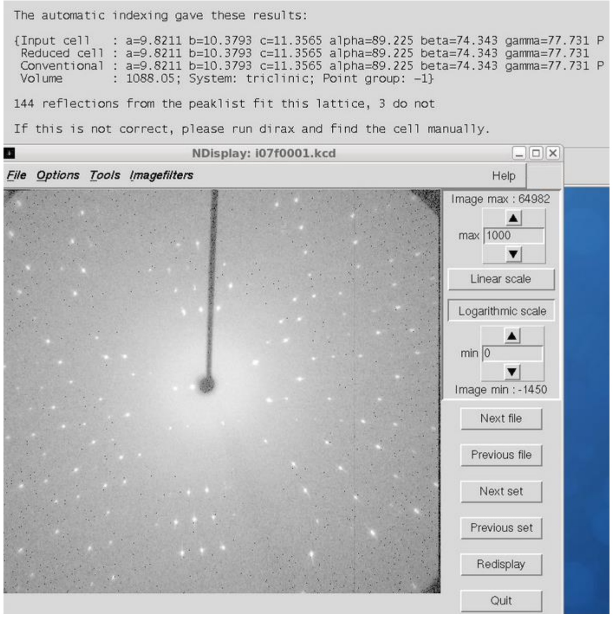

Figure S88. Crystal faces and unit cell determination of **28·CHCl3**.

INTENSITY STATISTICS FOR DATASET

Resolution	#Data	#Theory	%Complete	Redundancy	Mean I	Mean I/s	Rmerge	Rsigma
Inf - 2.32	178	183	97.3	5.24	212.79	68.69	0.0336	0.0137
2.32 - 1.56	416	416	100.0	5.36	161.14	65.05	0.0265	0.0135
1.56 - 1.23	624	624	100.0	5.25	111.27	59.60	0.0244	0.0144
1.23 - 1.08	579	579	100.0	5.00	78.83	52.96	0.0256	0.0159
1.08 - 0.98	612	612	100.0	4.72	66.42	47.55	0.0250	0.0173
0.98 - 0.91	601	601	100.0	4.38	52.24	41.13	0.0273	0.0195
0.91 - 0.86	554	554	100.0	4.18	45.89	38.37	0.0277	0.0217
0.86 - 0.81	683	683	100.0	3.96	38.79	33.90	0.0286	0.0241
0.81 - 0.78	550	550	100.0	3.80	32.86	30.58	0.0318	0.0269
0.78 - 0.75	574	574	100.0	3.58	29.49	27.80	0.0335	0.0298
0.75 - 0.72	722	722	100.0	3.40	27.02	25.48	0.0364	0.0328
0.72 - 0.70	522	522	100.0	3.23	24.93	23.53	0.0389	0.0359
0.70 - 0.68	612	613	99.8	3.18	19.56	19.98	0.0432	0.0413
0.68 - 0.66	660	660	100.0	3.01	18.55	18.76	0.0468	0.0451
0.66 - 0.64	779	779	100.0	2.88	16.45	16.62	0.0550	0.0511
0.64 - 0.63	412	412	100.0	2.79	15.37	15.24	0.0583	0.0558
0.63 - 0.62	442	442	100.0	2.71	14.66	14.62	0.0606	0.0587
0.62 - 0.60	985	985	100.0	2.60	12.69	12.90	0.0696	0.0683
0.60 - 0.59	564	565	99.8	2.46	10.66	10.97	0.0744	0.0839
0.59 - 0.58	774	803	96.4	2.30	9.12	8.96	0.0919	0.1044
0.68 - 0.58	4616	4646	99.4	2.66	13.75	13.84	0.0620	0.0636
Inf - 0.58	11843	11879	99.7	3.58	41.11	29.13	0.0309	0.0252

The structure contains a disordered solute molecule (dichloromethane) which has been partially described using isotropic atomic displacement parameters.

Complete .cif-data of the compound are available under the CCDC number **CCDC-2125771.**

Table S35. Crystal data and structure refinement of **28·CHCl$_3$**.

Identification code	12959sadabs	
Empirical formula	$C_{18}H_{17}BiCl_3F_4NOS$	
Color	colourless	
Formula weight	686.72 $g\cdot mol^{-1}$	
Temperature	100(2) K	
Wavelength	0.71073 Å	
Crystal system	Triclinic	
Space group	*P*-1, (no. 2)	
Unit cell dimensions	a = 9.8186(3) Å	α= 89.283(8)°.
	b = 10.3692(11) Å	β= 74.352(5)°.
	c = 11.3362(11) Å	γ = 77.753(4)°.
Volume	1084.86(16) Å^3	
Z	2	
Density (calculated)	2.102 $Mg\cdot m^{-3}$	
Absorption coefficient	8.636 mm^{-1}	
F(000)	652 e	
Crystal size	0.10 x 0.10 x 0.08 mm^3	
θ range for data collection	2.645 to 27.498°.	
Index ranges	$-12 \le h \le 12$, $-13 \le k \le 13$, $-14 \le l \le 14$	
Reflections collected	22632	
Independent reflections	4979 [R_{int} = 0.0271]	
Reflections with I>2σ(I)	4859	
Completeness to θ = 25.242°	99.9 %	
Absorption correction	Gaussian	
Max. and min. transmission	0.59088 and 0.46152	
Refinement method	Full-matrix least-squares on F^2	
Data / restraints / parameters	4979 / 0 / 277	
Goodness-of-fit on F^2	1.097	
Final R indices [I>2σ(I)]	R_1 = 0.0174	wR^2 = 0.0426
R indices (all data)	R_1 = 0.0180	wR^2 = 0.0430
Extinction coefficient	n/a	
Largest diff. peak and hole	0.986 and -1.268 $e\cdot Å^{-3}$	

Table S36. Bond lengths [Å] and angles [°] of **28·CHCl₃**.

Bi(1)-F(1)	2.1291(15)	Bi(1)-C(1)	2.292(2)
Bi(1)-C(12)	2.268(3)	Bi(1)-N(3)	2.670(2)
S(1)-O(1)	1.4407(19)	S(1)-N(3)	1.572(2)
S(1)-C(6)	1.771(3)	S(1)-C(7)	1.755(3)
F(2)-C(17)	1.337(3)	F(3)-C(17)	1.348(3)
F(4)-C(17)	1.336(3)	C(1)-C(2)	1.397(4)
C(1)-C(6)	1.393(3)	C(12)-C(7)	1.395(3)
C(12)-C(11)	1.395(3)	N(3)-C(17)	1.393(3)
C(2)-C(3)	1.407(4)	C(2)-C(13)	1.508(4)
C(3)-H(3)	0.9500	C(3)-C(4)	1.392(4)
C(4)-C(5)	1.399(4)	C(4)-C(14)	1.509(4)
C(5)-H(5)	0.9500	C(5)-C(6)	1.383(4)
C(7)-C(8)	1.387(4)	C(8)-H(8)	0.9500
C(8)-C(9)	1.397(4)	C(9)-C(10)	1.395(4)
C(9)-C(15)	1.505(4)	C(10)-H(10)	0.9500
C(10)-C(11)	1.400(4)	C(11)-C(16)	1.508(3)
C(13)-H(13A)	0.9800	C(13)-H(13B)	0.9800
C(13)-H(13C)	0.9800	C(14)-H(14A)	0.9800
C(14)-H(14B)	0.9800	C(14)-H(14C)	0.9800
C(15)-H(15A)	0.9800	C(15)-H(15B)	0.9800
C(15)-H(15C)	0.9800	C(16)-H(16A)	0.9800
C(16)-H(16B)	0.9800	C(16)-H(16C)	0.9800
C(99)-H(99A)	0.9900	C(99)-H(99B)	0.9900
C(99)-H(99)	1.0000	C(99)-Cl(1A)	1.753(3)
C(99)-Cl(2A)	1.679(4)	C(99)-Cl(3A)	1.747(4)
C(99)-Cl(1B)	1.620(10)	C(99)-Cl(3B)	1.726(4)
C(99)-Cl(2B)	1.900(6)	C(99)-Cl(3C)	1.897(5)
F(1)-Bi(1)-C(1)	90.02(7)	F(1)-Bi(1)-C(12)	89.18(7)
F(1)-Bi(1)-N(3)	153.66(6)	C(1)-Bi(1)-N(3)	73.85(8)
C(12)-Bi(1)-C(1)	88.53(9)	C(12)-Bi(1)-N(3)	70.13(8)
O(1)-S(1)-N(3)	120.87(12)	O(1)-S(1)-C(6)	111.24(12)
O(1)-S(1)-C(7)	112.57(12)	N(3)-S(1)-C(6)	106.70(12)
N(3)-S(1)-C(7)	99.00(12)	C(7)-S(1)-C(6)	104.78(12)
C(2)-C(1)-Bi(1)	124.91(18)	C(6)-C(1)-Bi(1)	117.77(18)

C(6)-C(1)-C(2)	117.3(2)	C(7)-C(12)-Bi(1)	116.79(18)
C(11)-C(12)-Bi(1)	125.56(18)	C(11)-C(12)-C(7)	117.6(2)
S(1)-N(3)-Bi(1)	97.69(10)	C(17)-N(3)-Bi(1)	120.00(17)
C(17)-N(3)-S(1)	119.61(19)	C(1)-C(2)-C(3)	118.5(2)
C(1)-C(2)-C(13)	121.8(2)	C(3)-C(2)-C(13)	119.7(2)
C(2)-C(3)-H(3)	118.4	C(4)-C(3)-C(2)	123.2(2)
C(4)-C(3)-H(3)	118.4	C(3)-C(4)-C(5)	118.2(2)
C(3)-C(4)-C(14)	121.7(3)	C(5)-C(4)-C(14)	120.1(2)
C(4)-C(5)-H(5)	121.0	C(6)-C(5)-C(4)	118.1(2)
C(6)-C(5)-H(5)	121.0	C(1)-C(6)-S(1)	116.22(19)
C(5)-C(6)-S(1)	119.1(2)	C(5)-C(6)-C(1)	124.7(2)
C(12)-C(7)-S(1)	117.83(19)	C(8)-C(7)-S(1)	117.90(19)
C(8)-C(7)-C(12)	124.2(2)	C(7)-C(8)-H(8)	120.9
C(7)-C(8)-C(9)	118.1(2)	C(9)-C(8)-H(8)	120.9
C(8)-C(9)-C(15)	120.4(2)	C(10)-C(9)-C(8)	118.1(2)
C(10)-C(9)-C(15)	121.5(2)	C(9)-C(10)-H(10)	118.3
C(9)-C(10)-C(11)	123.4(2)	C(11)-C(10)-H(10)	118.3
C(12)-C(11)-C(10)	118.4(2)	C(12)-C(11)-C(16)	122.6(2)
C(10)-C(11)-C(16)	119.0(2)	C(2)-C(13)-H(13A)	109.5
C(2)-C(13)-H(13B)	109.5	C(2)-C(13)-H(13C)	109.5
H(13A)-C(13)-H(13B)	109.5	H(13A)-C(13)-H(13C)	109.5
H(13B)-C(13)-H(13C)	109.5	C(4)-C(14)-H(14A)	109.5
C(4)-C(14)-H(14B)	109.5	C(4)-C(14)-H(14C)	109.5
H(14A)-C(14)-H(14B)	109.5	H(14A)-C(14)-H(14C)	109.5
H(14B)-C(14)-H(14C)	109.5	C(9)-C(15)-H(15A)	109.5
C(9)-C(15)-H(15B)	109.5	C(9)-C(15)-H(15C)	109.5
H(15A)-C(15)-H(15B)	109.5	H(15A)-C(15)-H(15C)	109.5
H(15B)-C(15)-H(15C)	109.5	C(11)-C(16)-H(16A)	109.5
C(11)-C(16)-H(16B)	109.5	C(11)-C(16)-H(16C)	109.5
H(16A)-C(16)-H(16B)	109.5	H(16A)-C(16)-H(16C)	109.5
H(16B)-C(16)-H(16C)	109.5	F(2)-C(17)-F(3)	106.0(2)
F(2)-C(17)-N(3)	109.3(2)	F(3)-C(17)-N(3)	115.8(2)
F(4)-C(17)-F(2)	106.3(2)	F(4)-C(17)-F(3)	106.0(2)
F(4)-C(17)-N(3)	112.8(2)	H(99A)-C(99)-H(99B)	108.8
Cl(1A)-C(99)-H(99)	109.3	Cl(2A)-C(99)-H(99)	109.3
Cl(2A)-C(99)-Cl(1A)	112.9(2)	Cl(2A)-C(99)-Cl(3A)	105.1(3)

Cl(3A)-C(99)-H(99)	109.3	Cl(3A)-C(99)-Cl(1A)	110.7(2)
Cl(1B)-C(99)-H(99A)	110.7	Cl(1B)-C(99)-H(99B)	110.7
Cl(1B)-C(99)-Cl(3B)	105.1(4)	Cl(3B)-C(99)-H(99A)	110.7
Cl(3B)-C(99)-H(99B)	110.7	Cl(3C)-C(99)-Cl(2B)	135.0(3)

8.6 Single crystal structure analysis of **37·CHCl3**

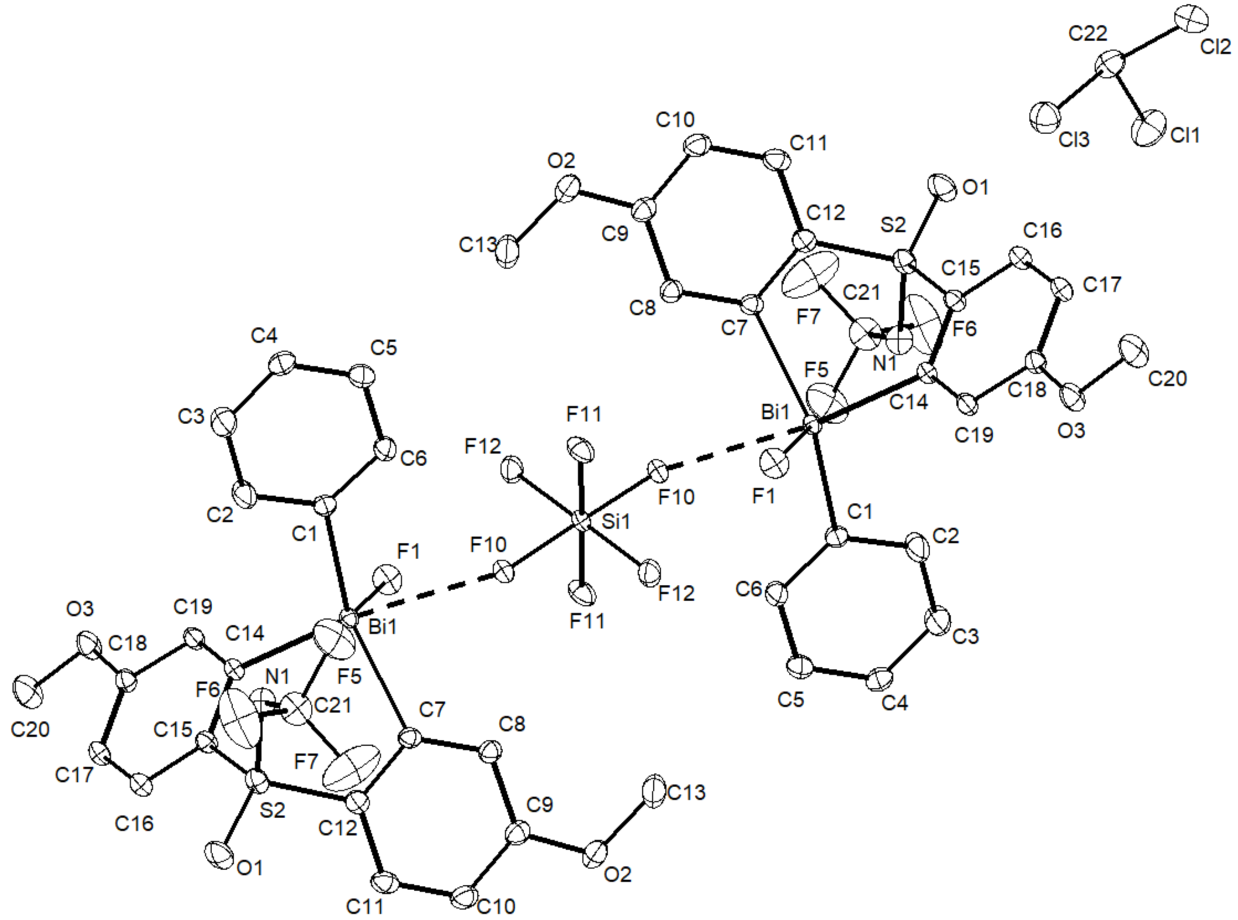

Figure S89. The molecular structure of complex **37·CHCl3**. H atoms have been removed for clarity.

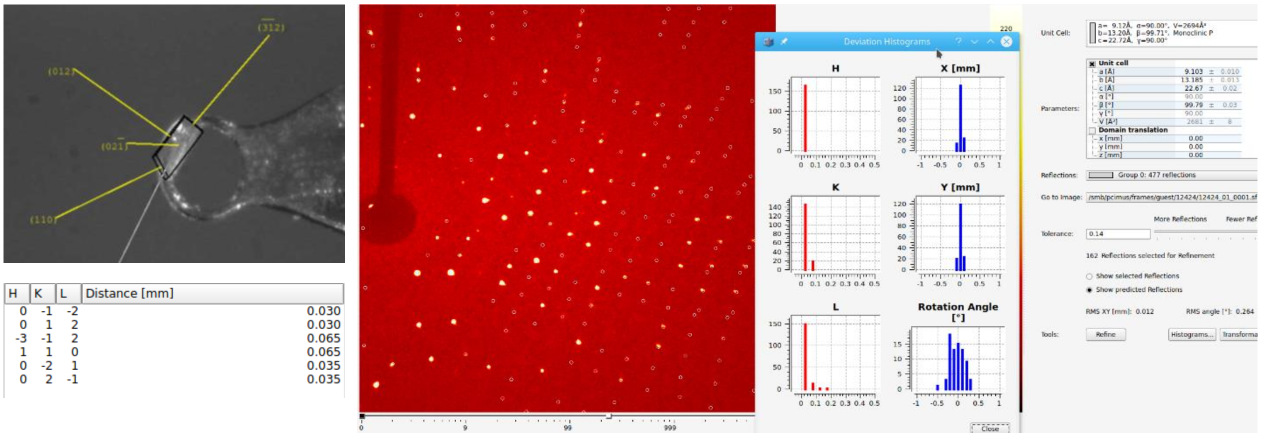

Figure S90. Crystal faces and unit cell determination of **37·CHCl3**.

INTENSITY STATISTICS FOR DATASET

Resolution	#Data	#Theory	%Complete	Redundancy	Mean I	Mean I/s	Rmerge	Rsigma
Inf - 2.64	171	176	97.2	18.56	114.25	110.00	0.0233	0.0077
2.64 - 1.76	388	388	100.0	23.13	84.15	112.44	0.0222	0.0068
1.76 - 1.39	565	565	100.0	23.87	57.81	102.30	0.0243	0.0072
1.39 - 1.21	562	562	100.0	23.78	47.60	96.12	0.0275	0.0076
1.21 - 1.09	609	609	100.0	22.07	36.94	82.19	0.0311	0.0087
1.09 - 1.01	557	557	100.0	17.73	30.98	66.00	0.0348	0.0108
1.01 - 0.95	587	587	100.0	15.30	28.16	56.50	0.0382	0.0124
0.95 - 0.90	585	585	100.0	13.56	25.66	49.16	0.0405	0.0143
0.90 - 0.86	594	594	100.0	12.75	21.32	42.61	0.0454	0.0166
0.86 - 0.83	487	487	100.0	12.16	18.40	37.60	0.0506	0.0187
0.83 - 0.80	593	593	100.0	11.75	18.92	37.75	0.0544	0.0197
0.80 - 0.78	457	457	100.0	11.49	16.86	32.86	0.0593	0.0219
0.78 - 0.75	746	746	100.0	10.91	15.18	29.93	0.0638	0.0247
0.75 - 0.73	575	575	100.0	10.66	14.21	28.20	0.0697	0.0273
0.73 - 0.71	647	647	100.0	10.28	12.59	25.20	0.0757	0.0309
0.71 - 0.70	324	324	100.0	9.86	12.76	23.99	0.0761	0.0326
0.70 - 0.68	782	782	100.0	9.80	11.45	22.28	0.0845	0.0354
0.68 - 0.67	418	418	100.0	9.41	10.76	20.21	0.0910	0.0396
0.67 - 0.66	429	429	100.0	9.24	9.99	18.48	0.0954	0.0425
0.66 - 0.64	1087	1111	97.8	8.37	9.35	16.92	0.1037	0.0496
0.74 - 0.64	3975	3999	99.4	9.44	11.15	21.26	0.0860	0.0379
Inf - 0.64	11163	11192	99.7	13.76	25.36	46.48	0.0365	0.0157

Anharmonic displacements of the heavy atom can probably cause the high residual electron density in the vicinity of the Bi atom.

Complete .cif-data of the compound are available under the CCDC number **CCDC-2125785.**

Table S37. Crystal data and structure refinement of **37·$CHCl_3$**.

Identification code	12424sadabs	
Empirical formula	$C_{44} H_{36} Bi_2 Cl_6 F_{14} N_2 O_6 S_2 Si$	
Color	colourless	
Formula weight	1677.62 g·mol^{-1}	
Temperature	100(2) K	
Wavelength	0.71073 Å	
Crystal system	Monoclinic	
Space group	$P2_1$/n, (no. 14)	
Unit cell dimensions	a = 9.0753(5) Å	α= 90°.
	b = 13.1811(7) Å	β= 99.637(2)°.
	c = 22.6088(12) Å	γ = 90°.
Volume	2666.4(2) Å^3	
Z	2	
Density (calculated)	2.090 Mg·m^{-3}	
Absorption coefficient	7.090 mm^{-1}	
F(000)	1604 e	
Crystal size	0.214 x 0.103 x 0.064 mm^3	
θ range for data collection	3.017 to 32.576°.	
Index ranges	-13 ≤ h ≤ 13, -19≤ k ≤ 19, -34 ≤ l ≤ 34	
Reflections collected	141515	
Independent reflections	9701 [R_{int} = 0.0356]	
Reflections with I>2σ(I)	8876	
Completeness to θ = 25.242°	99.9 %	
Absorption correction	Gaussian	
Max. and min. transmission	0.62675 and 0.26647	
Refinement method	Full-matrix least-squares on F^2	
Data / restraints / parameters	9701 / 0 / 351	
Goodness-of-fit on F^2	1.045	
Final R indices [I>2σ(I)]	R_1 = 0.0213	wR^2 = 0.0523
R indices (all data)	R_1 = 0.0249	wR^2 = 0.0538
Extinction coefficient	n/a	
Largest diff. peak and hole	2.741 and -0.997 e·Å^{-3}	

Table S38. Bond lengths [Å] and angles [°] of **37·CHCl$_3$**.

Bi(1)-F(10)	2.6607(13)	Bi(1)-F(1)	2.0414(16)
Bi(1)-C(14)	2.224(2)	Bi(1)-C(1)	2.207(2)
Bi(1)-C(7)	2.222(2)	S(2)-O(1)	1.4393(18)
S(2)-N(1)	1.556(2)	S(2)-C(12)	1.762(2)
S(2)-C(15)	1.743(2)	Si(1)-F(10)	1.7215(13)
Si(1)-F(10)#1	1.7215(13)	Si(1)-F(11)#1	1.6701(14)
Si(1)-F(11)	1.6701(13)	Si(1)-F(12)#1	1.6809(14)
Si(1)-F(12)	1.6809(14)	Cl(2)-C(22)	1.770(3)
Cl(3)-C(22)	1.753(3)	Cl(1)-C(22)	1.753(3)
F(5)-C(21)	1.333(3)	O(3)-C(18)	1.348(3)
O(3)-C(20)	1.439(3)	O(2)-C(9)	1.347(3)
O(2)-C(13)	1.429(3)	C(14)-C(19)	1.376(3)
C(14)-C(15)	1.398(3)	N(1)-C(21)	1.393(3)
F(7)-C(21)	1.339(4)	C(19)-H(19)	0.9500
C(19)-C(18)	1.412(3)	C(1)-C(6)	1.376(3)
C(1)-C(2)	1.380(3)	F(6)-C(21)	1.326(3)
C(12)-C(7)	1.394(3)	C(12)-C(11)	1.393(3)
C(16)-H(16)	0.9500	C(16)-C(17)	1.391(3)
C(16)-C(15)	1.381(3)	C(4)-H(4)	0.9500
C(4)-C(5)	1.377(3)	C(4)-C(3)	1.375(4)
C(18)-C(17)	1.392(3)	C(17)-H(17)	0.9500
C(7)-C(8)	1.381(3)	C(5)-H(5)	0.9500
C(5)-C(6)	1.399(3)	C(8)-H(8)	0.9500
C(8)-C(9)	1.408(3)	C(11)-H(11)	0.9500
C(11)-C(10)	1.376(4)	C(20)-H(20A)	0.9800
C(20)-H(20B)	0.9800	C(20)-H(20C)	0.9800
C(6)-H(6)	0.9500	C(10)-H(10)	0.9500
C(10)-C(9)	1.395(3)	C(3)-H(3)	0.9500
C(3)-C(2)	1.391(4)	C(2)-H(2)	0.9500
C(13)-H(13A)	0.9800	C(13)-H(13B)	0.9800
C(13)-H(13C)	0.9800	C(22)-H(22)	1.0000
F(1)-Bi(1)-F(10)	74.84(5)	F(1)-Bi(1)-C(14)	92.20(7)
F(1)-Bi(1)-C(1)	105.20(7)	F(1)-Bi(1)-C(7)	104.66(7)
C(14)-Bi(1)-F(10)	163.81(6)	C(1)-Bi(1)-F(10)	82.28(6)

C(1)-Bi(1)-C(14)	110.95(8)	C(1)-Bi(1)-C(7)	139.03(8)
C(7)-Bi(1)-F(10)	79.16(6)	C(7)-Bi(1)-C(14)	95.20(8)
O(1)-S(2)-N(1)	122.31(12)	O(1)-S(2)-C(12)	109.54(11)
O(1)-S(2)-C(15)	111.50(11)	N(1)-S(2)-C(12)	106.56(11)
N(1)-S(2)-C(15)	99.92(11)	C(15)-S(2)-C(12)	105.58(10)
F(10)-Si(1)-F(10)#1	180.00(13)	F(11)-Si(1)-F(10)#1	89.81(7)
F(11)-Si(1)-F(10)	90.19(7)	F(11)#1-Si(1)-F(10)	89.81(7)
F(11)#1-Si(1)-F(10)#1	90.19(7)	F(11)-Si(1)-F(11)#1	180.00(10)
F(11)#1-Si(1)-F(12)#1	90.57(8)	F(11)#1-Si(1)-F(12)	89.43(8)
F(11)-Si(1)-F(12)#1	89.43(8)	F(11)-Si(1)-F(12)	90.57(8)
F(12)#1-Si(1)-F(10)#1	89.58(7)	F(12)#1-Si(1)-F(10)	90.42(7)
F(12)-Si(1)-F(10)	89.58(7)	F(12)-Si(1)-F(10)#1	90.42(7)
F(12)#1-Si(1)-F(12)	180.0	Si(1)-F(10)-Bi(1)	141.26(7)
C(18)-O(3)-C(20)	117.62(19)	C(9)-O(2)-C(13)	117.6(2)
C(19)-C(14)-Bi(1)	124.04(15)	C(19)-C(14)-C(15)	119.53(19)
C(15)-C(14)-Bi(1)	116.18(15)	C(21)-N(1)-S(2)	118.22(19)
C(14)-C(19)-H(19)	120.5	C(14)-C(19)-C(18)	118.91(19)
C(18)-C(19)-H(19)	120.5	C(6)-C(1)-Bi(1)	121.20(16)
C(6)-C(1)-C(2)	123.8(2)	C(2)-C(1)-Bi(1)	114.73(17)
C(7)-C(12)-S(2)	119.82(17)	C(11)-C(12)-S(2)	120.13(17)
C(11)-C(12)-C(7)	120.0(2)	C(17)-C(16)-H(16)	120.5
C(15)-C(16)-H(16)	120.5	C(15)-C(16)-C(17)	119.1(2)
C(5)-C(4)-H(4)	119.9	C(3)-C(4)-H(4)	119.9
C(3)-C(4)-C(5)	120.3(2)	O(3)-C(18)-C(19)	115.31(19)
O(3)-C(18)-C(17)	123.6(2)	C(17)-C(18)-C(19)	121.1(2)
C(16)-C(17)-C(18)	119.5(2)	C(16)-C(17)-H(17)	120.2
C(18)-C(17)-H(17)	120.2	C(12)-C(7)-Bi(1)	114.87(15)
C(8)-C(7)-Bi(1)	122.22(16)	C(8)-C(7)-C(12)	121.8(2)
C(4)-C(5)-H(5)	119.5	C(4)-C(5)-C(6)	120.9(2)
C(6)-C(5)-H(5)	119.5	C(7)-C(8)-H(8)	121.2
C(7)-C(8)-C(9)	117.6(2)	C(9)-C(8)-H(8)	121.2
C(12)-C(11)-H(11)	120.4	C(10)-C(11)-C(12)	119.1(2)
C(10)-C(11)-H(11)	120.4	C(14)-C(15)-S(2)	119.40(16)
C(16)-C(15)-S(2)	118.71(16)	C(16)-C(15)-C(14)	121.9(2)
O(3)-C(20)-H(20A)	109.5	O(3)-C(20)-H(20B)	109.5
O(3)-C(20)-H(20C)	109.5	H(20A)-C(20)-H(20B)	109.5

H(20A)-C(20)-H(20C)	109.5	H(20B)-C(20)-H(20C)	109.5
C(1)-C(6)-C(5)	116.8(2)	C(1)-C(6)-H(6)	121.6
C(5)-C(6)-H(6)	121.6	C(11)-C(10)-H(10)	119.6
C(11)-C(10)-C(9)	120.8(2)	C(9)-C(10)-H(10)	119.6
F(5)-C(21)-N(1)	109.6(2)	F(5)-C(21)-F(7)	105.7(3)
F(7)-C(21)-N(1)	116.2(2)	F(6)-C(21)-F(5)	105.1(2)
F(6)-C(21)-N(1)	112.5(2)	F(6)-C(21)-F(7)	106.9(3)
C(4)-C(3)-H(3)	119.7	C(4)-C(3)-C(2)	120.6(2)
C(2)-C(3)-H(3)	119.7	C(1)-C(2)-C(3)	117.5(2)
C(1)-C(2)-H(2)	121.2	C(3)-C(2)-H(2)	121.2
O(2)-C(9)-C(8)	123.3(2)	O(2)-C(9)-C(10)	116.0(2)
C(10)-C(9)-C(8)	120.7(2)	O(2)-C(13)-H(13A)	109.5
O(2)-C(13)-H(13B)	109.5	O(2)-C(13)-H(13C)	109.5
H(13A)-C(13)-H(13B)	109.5	H(13A)-C(13)-H(13C)	109.5
H(13B)-C(13)-H(13C)	109.5	Cl(2)-C(22)-H(22)	108.5
Cl(3)-C(22)-Cl(2)	110.49(15)	Cl(3)-C(22)-H(22)	108.5
Cl(1)-C(22)-Cl(2)	109.83(15)	Cl(1)-C(22)-Cl(3)	110.98(15)
Cl(1)-C(22)-H(22)	108.5		

Symmetry transformations used to generate equivalent atoms:

#1 -x+1,-y+2,-z+1

<u>8.7 Single crystal structure analysis of **42**</u>

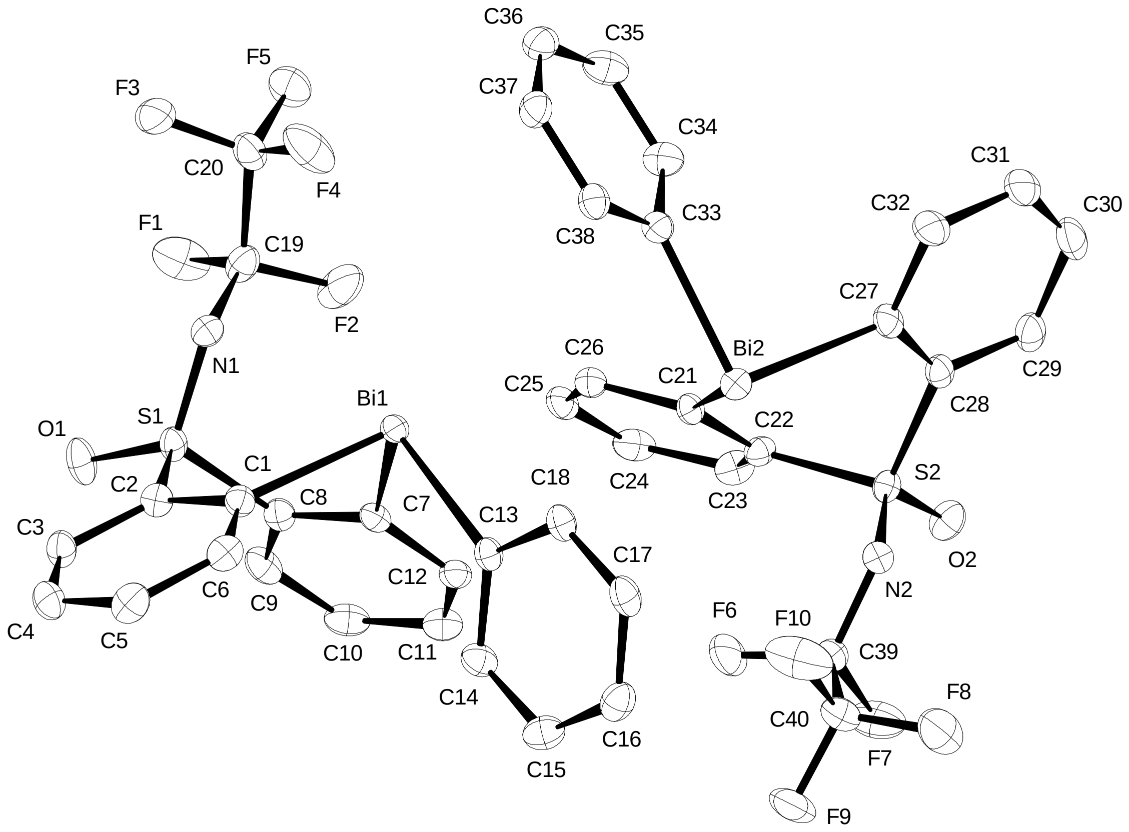

Figure S91. The molecular structure of complex **42**. H atoms have been removed for clarity.

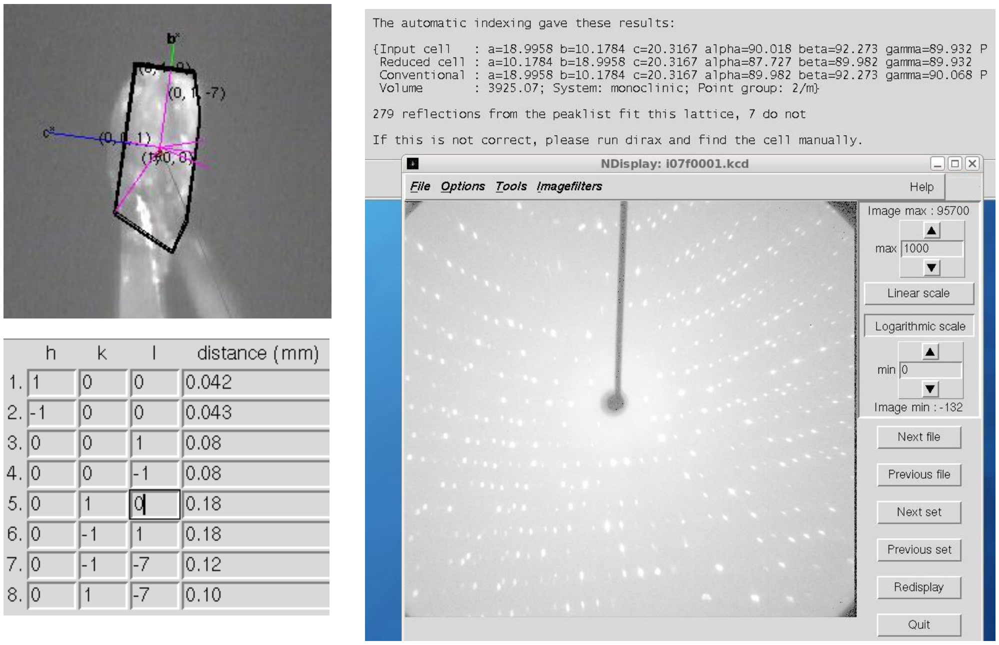

Figure S92. Crystal faces and unit cell determination of **42**.

INTENSITY STATISTICS FOR DATASET

Resolution	#Data	#Theory	%Complete	Redundancy	Mean I	Mean I/s	Rmerge	Rsigma
Inf - 2.51	293	308	95.1	6.90	94.27	27.14	0.0790	0.0420
2.51 - 1.66	701	701	100.0	9.16	80.90	33.32	0.0656	0.0275
1.66 - 1.32	955	955	100.0	9.70	62.26	32.66	0.0600	0.0271
1.32 - 1.15	966	966	100.0	9.78	40.73	31.06	0.0608	0.0264
1.15 - 1.04	1006	1006	100.0	9.40	37.58	29.51	0.0576	0.0272
1.04 - 0.96	1034	1034	100.0	9.08	29.65	27.71	0.0599	0.0288
0.96 - 0.90	1031	1031	100.0	8.74	23.01	25.19	0.0617	0.0300
0.90 - 0.86	858	858	100.0	8.43	19.96	23.54	0.0635	0.0314
0.86 - 0.82	1042	1042	100.0	8.01	18.65	22.35	0.0646	0.0340
0.82 - 0.79	896	896	100.0	7.68	13.84	19.37	0.0709	0.0377
0.79 - 0.76	1068	1068	100.0	7.23	13.99	18.61	0.0732	0.0401
0.76 - 0.73	1224	1224	100.0	6.84	11.72	16.07	0.0816	0.0443
0.73 - 0.71	969	971	99.8	6.44	10.92	15.71	0.0858	0.0475
0.71 - 0.69	1061	1063	99.8	6.04	9.20	13.43	0.0955	0.0550
0.69 - 0.68	549	550	99.8	5.69	7.91	11.59	0.1063	0.0623
0.68 - 0.66	1286	1288	99.8	5.36	7.40	11.24	0.1128	0.0691
0.66 - 0.65	679	681	99.7	5.15	6.41	10.03	0.1205	0.0768
0.65 - 0.63	1475	1487	99.2	4.90	5.95	9.11	0.1294	0.0837
0.63 - 0.62	869	875	99.3	4.43	5.38	8.35	0.1384	0.0961
0.62 - 0.61	870	893	97.4	4.26	4.91	7.32	0.1460	0.1039
0.61 - 0.60	574	617	93.0	3.79	4.60	6.84	0.1600	0.1144
0.70 - 0.60	6847	6938	98.7	4.91	6.39	9.62	0.1204	0.0795
Inf - 0.60	19406	19514	99.4	7.03	21.26	18.82	0.0682	0.0367

Complete .cif-data of the compound are available under the CCDC number **CCDC-2125772.**

Table S39. Crystal data and structure refinement of **42**.

Identification code	13474sadabs	
Empirical formula	$C_{20} H_{13} Bi F_5 N O S$	
Color	colourless	
Formula weight	619.35 g·mol^{-1}	
Temperature	100(2) K	
Wavelength	0.71073 Å	
Crystal system	Monoclinic	
Space group	$P2_1$/c, (no. 14)	
Unit cell dimensions	a = 18.967(4) Å	α= 90°.
	b = 10.1699(14) Å	β= 92.257(13)°.
	c = 20.304(4) Å	γ = 90°.
Volume	3913.6(13) Å^3	
Z	8	
Density (calculated)	2.102 Mg·m^{-3}	
Absorption coefficient	9.174 mm^{-1}	
F(000)	2336 e	
Crystal size	0.36 x 0.16 x 0.09 mm^3	
θ range for data collection	2.883 to 27.498°.	
Index ranges	$-24 \le h \le 24$, $-13 \le k \le 13$, $-26 \le l \le 26$	
Reflections collected	79722	
Independent reflections	8964 [R_{int} = 0.0629]	
Reflections with I>2σ(I)	8451	
Completeness to θ = 25.242°	99.5 %	
Absorption correction	Gaussian	
Max. and min. transmission	0.47322 and 0.08637	
Refinement method	Full-matrix least-squares on F^2	
Data / restraints / parameters	8964 / 0 / 523	
Goodness-of-fit on F^2	1.090	
Final R indices [I>2σ(I)]	R_1 = 0.0268	wR^2 = 0.0713
R indices (all data)	R_1 = 0.0291	wR^2 = 0.0741
Extinction coefficient	n/a	
Largest diff. peak and hole	1.907 and -2.387 e·Å^{-3}	

Table S40. Bond lengths [Å] and angles [°] of **42**.

Bi(1)-C(1)	2.260(3)	Bi(1)-C(7)	2.273(3)
Bi(1)-C(13)	2.240(4)	S(1)-O(1)	1.446(3)
S(1)-N(1)	1.555(3)	S(1)-C(2)	1.764(4)
S(1)-C(8)	1.781(4)	F(1)-C(19)	1.363(5)
F(2)-C(19)	1.373(4)	F(3)-C(20)	1.331(5)
F(4)-C(20)	1.327(5)	F(5)-C(20)	1.324(4)
N(1)-C(19)	1.395(5)	C(1)-C(2)	1.396(5)
C(1)-C(6)	1.390(5)	C(2)-C(3)	1.396(5)
C(3)-H(3)	0.9500	C(3)-C(4)	1.388(5)
C(4)-H(4)	0.9500	C(4)-C(5)	1.392(6)
C(5)-H(5)	0.9500	C(5)-C(6)	1.395(5)
C(6)-H(6)	0.9500	C(7)-C(8)	1.404(5)
C(7)-C(12)	1.381(5)	C(8)-C(9)	1.388(5)
C(9)-H(9)	0.9500	C(9)-C(10)	1.394(6)
C(10)-H(10)	0.9500	C(10)-C(11)	1.390(5)
C(11)-H(11)	0.9500	C(11)-C(12)	1.403(5)
C(12)-H(12)	0.9500	C(13)-C(14)	1.396(5)
C(13)-C(18)	1.397(5)	C(14)-H(14)	0.9500
C(14)-C(15)	1.389(5)	C(15)-H(15)	0.9500
C(15)-C(16)	1.388(5)	C(16)-H(16)	0.9500
C(16)-C(17)	1.393(5)	C(17)-H(17)	0.9500
C(17)-C(18)	1.390(5)	C(18)-H(18)	0.9500
C(19)-C(20)	1.541(5)	Bi(2)-C(21)	2.274(3)
Bi(2)-C(27)	2.262(4)	Bi(2)-C(33)	2.245(3)
S(2)-O(2)	1.443(3)	S(2)-N(2)	1.555(3)
S(2)-C(22)	1.776(3)	S(2)-C(28)	1.768(4)
F(6)-C(39)	1.370(4)	F(7)-C(39)	1.375(4)
F(8)-C(40)	1.330(5)	F(9)-C(40)	1.325(4)
F(10)-C(40)	1.328(5)	N(2)-C(39)	1.392(4)
C(21)-C(22)	1.397(5)	C(21)-C(26)	1.401(5)
C(22)-C(23)	1.387(5)	C(23)-H(23)	0.9500
C(23)-C(24)	1.386(5)	C(24)-H(24)	0.9500
C(24)-C(25)	1.390(6)	C(25)-H(25)	0.9500
C(25)-C(26)	1.398(5)	C(26)-H(26)	0.9500
C(27)-C(28)	1.393(5)	C(27)-C(32)	1.391(5)

C(28)-C(29)	1.396(5)	C(29)-H(29)	0.9500
C(29)-C(30)	1.405(6)	C(30)-H(30)	0.9500
C(30)-C(31)	1.372(6)	C(31)-H(31)	0.9500
C(31)-C(32)	1.402(6)	C(32)-H(32)	0.9500
C(33)-C(34)	1.399(5)	C(33)-C(38)	1.394(5)
C(34)-H(34)	0.9500	C(34)-C(35)	1.385(5)
C(35)-H(35)	0.9500	C(35)-C(36)	1.393(6)
C(36)-H(36)	0.9500	C(36)-C(37)	1.383(6)
C(37)-H(37)	0.9500	C(37)-C(38)	1.399(5)
C(38)-H(38)	0.9500	C(39)-C(40)	1.526(5)

C(1)-Bi(1)-C(7)	85.10(12)	C(13)-Bi(1)-C(1)	93.82(13)
C(13)-Bi(1)-C(7)	94.62(12)	O(1)-S(1)-N(1)	119.96(17)
O(1)-S(1)-C(2)	110.41(16)	O(1)-S(1)-C(8)	108.62(17)
N(1)-S(1)-C(2)	101.84(17)	N(1)-S(1)-C(8)	110.66(16)
C(2)-S(1)-C(8)	104.09(16)	C(19)-N(1)-S(1)	120.0(3)
C(2)-C(1)-Bi(1)	120.1(3)	C(6)-C(1)-Bi(1)	122.4(3)
C(6)-C(1)-C(2)	117.5(3)	C(1)-C(2)-S(1)	120.0(3)
C(1)-C(2)-C(3)	122.8(3)	C(3)-C(2)-S(1)	117.1(3)
C(2)-C(3)-H(3)	120.9	C(4)-C(3)-C(2)	118.3(3)
C(4)-C(3)-H(3)	120.9	C(3)-C(4)-H(4)	119.9
C(3)-C(4)-C(5)	120.2(3)	C(5)-C(4)-H(4)	119.9
C(4)-C(5)-H(5)	119.9	C(4)-C(5)-C(6)	120.3(3)
C(6)-C(5)-H(5)	119.9	C(1)-C(6)-C(5)	120.9(3)
C(1)-C(6)-H(6)	119.5	C(5)-C(6)-H(6)	119.5
C(8)-C(7)-Bi(1)	120.3(3)	C(12)-C(7)-Bi(1)	122.4(2)
C(12)-C(7)-C(8)	117.3(3)	C(7)-C(8)-S(1)	118.9(3)
C(9)-C(8)-S(1)	118.2(3)	C(9)-C(8)-C(7)	122.9(3)
C(8)-C(9)-H(9)	120.8	C(8)-C(9)-C(10)	118.4(3)
C(10)-C(9)-H(9)	120.8	C(9)-C(10)-H(10)	119.9
C(11)-C(10)-C(9)	120.2(3)	C(11)-C(10)-H(10)	119.9
C(10)-C(11)-H(11)	120.0	C(10)-C(11)-C(12)	120.0(4)
C(12)-C(11)-H(11)	120.0	C(7)-C(12)-C(11)	121.2(3)
C(7)-C(12)-H(12)	119.4	C(11)-C(12)-H(12)	119.4
C(14)-C(13)-Bi(1)	123.4(3)	C(14)-C(13)-C(18)	119.3(3)
C(18)-C(13)-Bi(1)	117.3(3)	C(13)-C(14)-H(14)	120.0

C(15)-C(14)-C(13)	120.0(3)	C(15)-C(14)-H(14)	120.0
C(14)-C(15)-H(15)	119.6	C(16)-C(15)-C(14)	120.7(4)
C(16)-C(15)-H(15)	119.6	C(15)-C(16)-H(16)	120.3
C(15)-C(16)-C(17)	119.3(3)	C(17)-C(16)-H(16)	120.3
C(16)-C(17)-H(17)	119.8	C(18)-C(17)-C(16)	120.3(3)
C(18)-C(17)-H(17)	119.8	C(13)-C(18)-H(18)	119.9
C(17)-C(18)-C(13)	120.3(3)	C(17)-C(18)-H(18)	119.9
F(1)-C(19)-F(2)	104.7(3)	F(1)-C(19)-N(1)	115.4(3)
F(1)-C(19)-C(20)	106.3(3)	F(2)-C(19)-N(1)	114.4(3)
F(2)-C(19)-C(20)	105.5(3)	N(1)-C(19)-C(20)	109.8(3)
F(3)-C(20)-C(19)	110.5(3)	F(4)-C(20)-F(3)	107.8(3)
F(4)-C(20)-C(19)	110.8(3)	F(5)-C(20)-F(3)	107.8(3)
F(5)-C(20)-F(4)	108.5(3)	F(5)-C(20)-C(19)	111.4(3)
C(27)-Bi(2)-C(21)	85.87(12)	C(33)-Bi(2)-C(21)	90.79(12)
C(33)-Bi(2)-C(27)	93.81(13)	O(2)-S(2)-N(2)	119.91(16)
O(2)-S(2)-C(22)	108.79(16)	O(2)-S(2)-C(28)	110.69(17)
N(2)-S(2)-C(22)	109.71(16)	N(2)-S(2)-C(28)	101.55(16)
C(28)-S(2)-C(22)	105.11(16)	C(39)-N(2)-S(2)	120.0(2)
C(22)-C(21)-Bi(2)	122.0(2)	C(22)-C(21)-C(26)	116.6(3)
C(26)-C(21)-Bi(2)	121.4(2)	C(21)-C(22)-S(2)	118.1(2)
C(23)-C(22)-S(2)	118.4(3)	C(23)-C(22)-C(21)	123.5(3)
C(22)-C(23)-H(23)	120.7	C(24)-C(23)-C(22)	118.7(3)
C(24)-C(23)-H(23)	120.7	C(23)-C(24)-H(24)	120.1
C(23)-C(24)-C(25)	119.7(3)	C(25)-C(24)-H(24)	120.1
C(24)-C(25)-H(25)	119.6	C(24)-C(25)-C(26)	120.7(3)
C(26)-C(25)-H(25)	119.6	C(21)-C(26)-H(26)	119.7
C(25)-C(26)-C(21)	120.7(3)	C(25)-C(26)-H(26)	119.7
C(28)-C(27)-Bi(2)	120.8(2)	C(32)-C(27)-Bi(2)	121.6(3)
C(32)-C(27)-C(28)	117.5(3)	C(27)-C(28)-S(2)	119.9(3)
C(27)-C(28)-C(29)	123.2(3)	C(29)-C(28)-S(2)	116.8(3)
C(28)-C(29)-H(29)	121.1	C(28)-C(29)-C(30)	117.7(4)
C(30)-C(29)-H(29)	121.1	C(29)-C(30)-H(30)	120.0
C(31)-C(30)-C(29)	120.1(4)	C(31)-C(30)-H(30)	120.0
C(30)-C(31)-H(31)	119.4	C(30)-C(31)-C(32)	121.1(4)
C(32)-C(31)-H(31)	119.4	C(27)-C(32)-C(31)	120.4(4)
C(27)-C(32)-H(32)	119.8	C(31)-C(32)-H(32)	119.8

C(34)-C(33)-Bi(2)	122.0(3)	C(38)-C(33)-Bi(2)	118.3(3)
C(38)-C(33)-C(34)	119.5(3)	C(33)-C(34)-H(34)	119.9
C(35)-C(34)-C(33)	120.2(3)	C(35)-C(34)-H(34)	119.9
C(34)-C(35)-H(35)	119.9	C(34)-C(35)-C(36)	120.2(4)
C(36)-C(35)-H(35)	119.9	C(35)-C(36)-H(36)	120.1
C(37)-C(36)-C(35)	119.9(4)	C(37)-C(36)-H(36)	120.1
C(36)-C(37)-H(37)	119.8	C(36)-C(37)-C(38)	120.3(3)
C(38)-C(37)-H(37)	119.8	C(33)-C(38)-C(37)	119.9(3)
C(33)-C(38)-H(38)	120.1	C(37)-C(38)-H(38)	120.1
F(6)-C(39)-F(7)	104.5(3)	F(6)-C(39)-N(2)	115.1(3)
F(6)-C(39)-C(40)	106.2(3)	F(7)-C(39)-N(2)	114.1(3)
F(7)-C(39)-C(40)	105.6(3)	N(2)-C(39)-C(40)	110.5(3)
F(8)-C(40)-C(39)	110.7(3)	F(9)-C(40)-F(8)	107.9(3)
F(9)-C(40)-F(10)	108.5(3)	F(9)-C(40)-C(39)	111.5(3)
F(10)-C(40)-F(8)	107.4(3)	F(10)-C(40)-C(39)	110.6(3)

8.8 Single crystal structure analysis of **47·CH_2Cl_2**

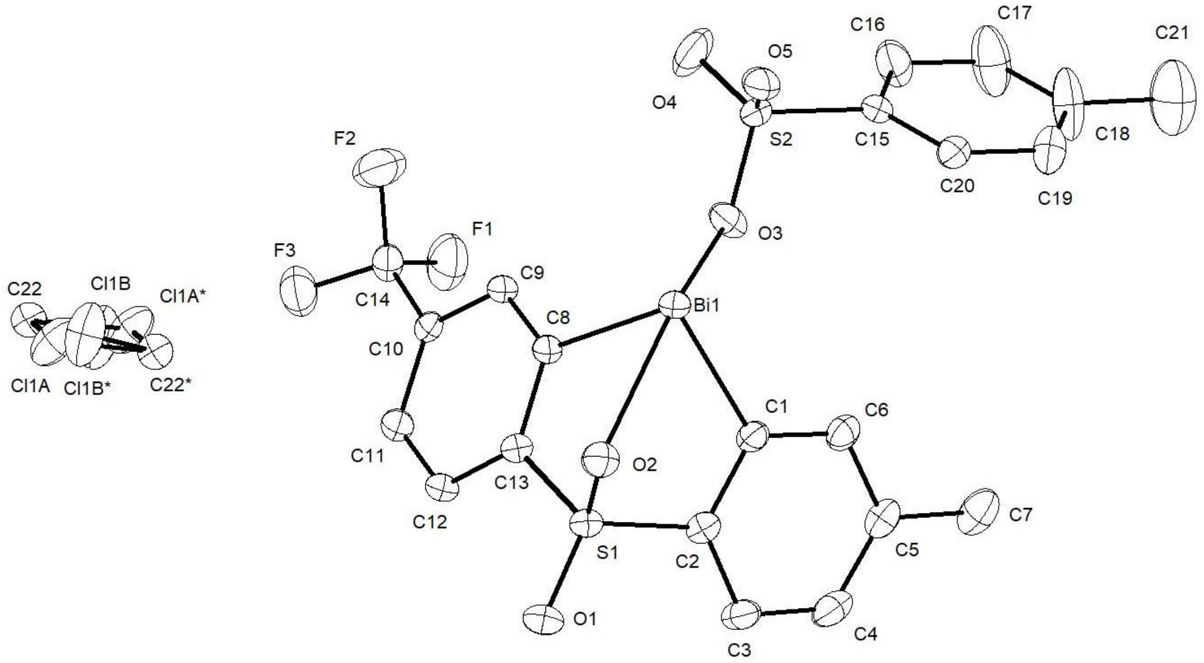

Figure S93. The molecular structure of complex **47·CH_2Cl_2**. H atoms have been removed for clarity.

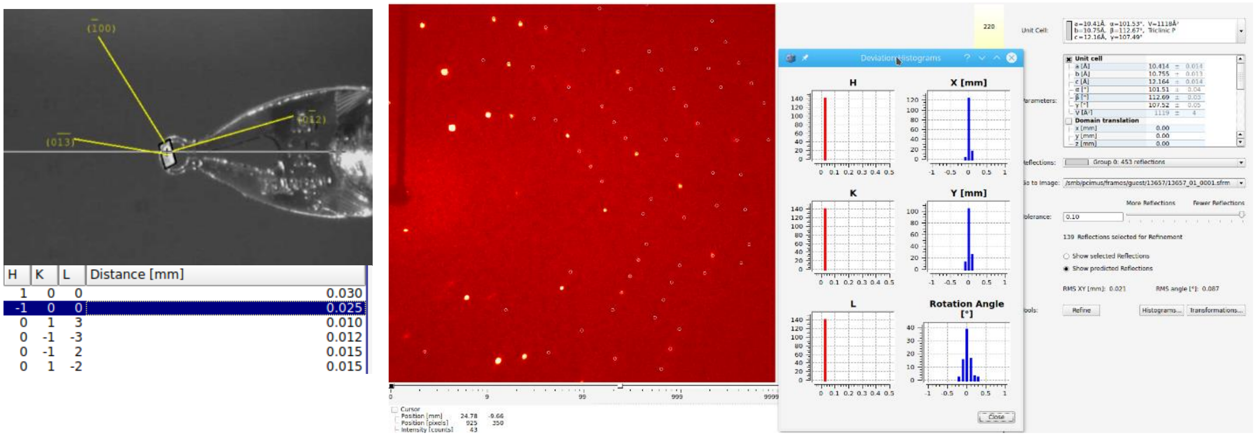

Figure S94. Crystal faces and unit cell determination of **47·CH_2Cl_2**.

INTENSITY STATISTICS FOR DATASET

Resolution	#Data	#Theory	%Complete	Redundancy	Mean I	Mean I/s	Rmerge	Rsigma
Inf - 2.80	207	212	97.6	4.46	215.13	71.73	0.0155	0.0125
2.80 - 1.88	476	490	97.1	4.62	151.59	68.81	0.0151	0.0127
1.88 - 1.49	675	692	97.5	4.54	102.04	61.34	0.0160	0.0138
1.49 - 1.30	696	712	97.8	4.51	80.51	56.58	0.0179	0.0148
1.30 - 1.18	695	704	98.7	4.39	57.71	48.85	0.0210	0.0168
1.18 - 1.10	643	652	98.6	4.13	46.19	43.00	0.0253	0.0194
1.10 - 1.03	733	754	97.2	3.24	47.06	37.16	0.0254	0.0231
1.03 - 0.98	659	698	94.4	2.68	36.65	29.38	0.0319	0.0290
0.98 - 0.93	774	824	93.9	2.32	32.19	25.99	0.0334	0.0341
0.93 - 0.90	545	586	93.0	2.13	28.98	22.80	0.0356	0.0390
0.90 - 0.86	879	958	91.8	1.96	23.95	18.78	0.0368	0.0465
0.86 - 0.84	484	526	92.0	1.92	22.18	17.52	0.0377	0.0508
0.84 - 0.81	808	886	91.2	1.84	18.52	14.97	0.0443	0.0607
0.81 - 0.79	643	708	90.8	1.80	18.85	14.35	0.0480	0.0634
0.79 - 0.77	653	718	90.9	1.71	16.39	12.64	0.0515	0.0729
0.77 - 0.75	758	844	89.8	1.70	15.40	11.71	0.0558	0.0792
0.75 - 0.73	808	902	89.6	1.61	14.02	10.38	0.0594	0.0888
0.73 - 0.72	470	526	89.4	1.60	13.07	9.71	0.0701	0.0969
0.72 - 0.70	946	1076	87.9	1.54	11.32	8.21	0.0773	0.1124
0.70 - 0.69	522	600	87.0	1.46	10.01	7.37	0.0910	0.1305
0.69 - 0.68	433	642	67.4	1.05	10.28	7.23	0.0808	0.1332
0.78 - 0.68	4293	4980	86.2	1.52	12.84	9.51	0.0661	0.0986
Inf - 0.68	13507	14710	91.8	2.50	39.23	26.67	0.0228	0.0311

Structure contains one disordered solute molecule (CH_2Cl_2) on a crystallographic special position (inversion centre). This has partially been described using isotropic displacement parameters. Complete .cif-data of the compound are available under the CCDC number **CCDC-2125767.**

Table S41. Crystal data and structure refinement of **47·CH_2Cl_2**.

Identification code	13657sadabs	
Empirical formula	$C_{21.125} H_{16.25} Bi Cl_{0.25} F_3 O_5 S_2$	
Color	colourless	
Formula weight	689.05 g·mol^{-1}	
Temperature	100(2) K	
Wavelength	0.71073 Å	
Crystal system	Triclinic	
Space group	*P*-1, (no. 2)	
Unit cell dimensions	a = 10.3492(4) Å	□= 101.582(2)°.
	b = 10.7121(5) Å	□= 112.493(2)°.
	c = 12.1107(5) Å	□ = 107.577(2)°.
Volume	1103.68(8) Å^3	
Z	2	
Density (calculated)	2.073 Mg·m^{-3}	
Absorption coefficient	8.264 mm^{-1}	
F(000)	658 e	
Crystal size	0.095 x 0.060 x 0.025 mm^3	
θ range for data collection	1.950 to 30.508°.	
Index ranges	-14 ≤ h ≤ 14, -15≤ k ≤ 15, -17 ≤ l ≤ 17	
Reflections collected	35210	
Independent reflections	6728 [R_{int} = 0.0251]	
Reflections with I>2σ(I)	6092	
Completeness to θ = 25.242°	100.0 %	
Absorption correction	Gaussian	
Max. and min. transmission	0.87120 and 0.59295	
Refinement method	Full-matrix least-squares on F^2	
Data / restraints / parameters	6728 / 6 / 313	
Goodness-of-fit on F^2	1.047	
Final R indices [I>2σ(I)]	R_1 = 0.0273	wR^2 = 0.0673
R indices (all data)	R_1 = 0.0331	wR^2 = 0.0705
Extinction coefficient	n/a	
Largest diff. peak and hole	1.966 and -1.037 e·Å^{-3}	

Table S42. Bond lengths [Å] and angles [°] of **47·CH_2Cl_2**.

Bi(1)-O(2)	2.547(3)	Bi(1)-O(3)	2.249(3)
Bi(1)-C(1)	2.281(3)	Bi(1)-C(8)	2.257(4)
S(1)-O(1)	1.431(3)	S(1)-O(2)	1.468(3)
S(1)-C(2)	1.758(4)	S(1)-C(13)	1.775(4)
S(2)-O(3)	1.501(3)	S(2)-O(4)	1.441(3)
S(2)-O(5)	1.452(3)	S(2)-C(15)	1.757(3)
F(1)-C(14)	1.351(5)	F(2)-C(14)	1.329(4)
F(3)-C(14)	1.336(4)	C(1)-C(2)	1.395(5)
C(1)-C(6)	1.388(5)	C(2)-C(3)	1.394(5)
C(3)-H(3)	0.9500	C(3)-C(4)	1.392(6)
C(4)-H(4)	0.9500	C(4)-C(5)	1.395(5)
C(5)-C(6)	1.409(5)	C(5)-C(7)	1.506(5)
C(6)-H(6)	0.9500	C(8)-C(13)	1.394(4)
C(8)-C(9)	1.386(5)	C(13)-C(12)	1.386(5)
C(12)-H(12)	0.9500	C(12)-C(11)	1.388(6)
C(11)-H(11)	0.9500	C(11)-C(10)	1.400(5)
C(10)-C(9)	1.395(5)	C(10)-C(14)	1.492(5)
C(9)-H(9)	0.9500	C(7)-H(7A)	0.9800
C(7)-H(7B)	0.9800	C(7)-H(7C)	0.9800
C(15)-C(16)	1.373(6)	C(15)-C(20)	1.388(5)
C(16)-H(16)	0.9500	C(16)-C(17)	1.396(6)
C(17)-H(17)	0.9500	C(17)-C(18)	1.374(7)
C(18)-C(19)	1.396(7)	C(18)-C(21)	1.508(7)
C(19)-H(19)	0.9500	C(19)-C(20)	1.388(5)
C(20)-H(20)	0.9500	C(21)-H(21A)	0.9800
C(21)-H(21B)	0.9800	C(21)-H(21C)	0.9800
C(22)-H(22B)	0.9900	C(22)-H(22A)	0.9900
C(22)-Cl(1A)	1.81(3)	C(22)-Cl(1B)	1.78(4)
O(3)-Bi(1)-O(2)	150.61(10)	O(3)-Bi(1)-C(1)	84.98(11)
O(3)-Bi(1)-C(8)	84.96(11)	C(1)-Bi(1)-O(2)	73.20(11)
C(8)-Bi(1)-O(2)	73.55(11)	C(8)-Bi(1)-C(1)	83.88(12)
O(1)-S(1)-O(2)	119.49(18)	O(1)-S(1)-C(2)	112.42(17)
O(1)-S(1)-C(13)	112.01(17)	O(2)-S(1)-C(2)	104.21(17)
O(2)-S(1)-C(13)	104.22(17)	C(2)-S(1)-C(13)	102.83(17)

O(3)-S(2)-C(15)	103.38(16)	O(4)-S(2)-O(3)	110.6(2)
O(4)-S(2)-O(5)	116.1(2)	O(4)-S(2)-C(15)	108.28(19)
O(5)-S(2)-O(3)	109.51(17)	O(5)-S(2)-C(15)	108.15(18)
S(1)-O(2)-Bi(1)	101.67(14)	S(2)-O(3)-Bi(1)	120.94(16)
C(2)-C(1)-Bi(1)	116.6(2)	C(6)-C(1)-Bi(1)	125.1(2)
C(6)-C(1)-C(2)	118.3(3)	C(1)-C(2)-S(1)	114.9(3)
C(3)-C(2)-S(1)	122.1(3)	C(3)-C(2)-C(1)	123.0(3)
C(2)-C(3)-H(3)	121.3	C(4)-C(3)-C(2)	117.4(3)
C(4)-C(3)-H(3)	121.3	C(3)-C(4)-H(4)	119.2
C(3)-C(4)-C(5)	121.5(3)	C(5)-C(4)-H(4)	119.2
C(4)-C(5)-C(6)	119.2(3)	C(4)-C(5)-C(7)	121.0(3)
C(6)-C(5)-C(7)	119.8(4)	C(1)-C(6)-C(5)	120.5(3)
C(1)-C(6)-H(6)	119.8	C(5)-C(6)-H(6)	119.8
C(13)-C(8)-Bi(1)	117.0(3)	C(9)-C(8)-Bi(1)	124.6(2)
C(9)-C(8)-C(13)	118.2(3)	C(8)-C(13)-S(1)	114.8(3)
C(12)-C(13)-S(1)	121.8(3)	C(12)-C(13)-C(8)	123.3(3)
C(13)-C(12)-H(12)	121.1	C(13)-C(12)-C(11)	117.8(3)
C(11)-C(12)-H(12)	121.1	C(12)-C(11)-H(11)	120.0
C(12)-C(11)-C(10)	120.0(3)	C(10)-C(11)-H(11)	120.0
C(11)-C(10)-C(14)	120.2(3)	C(9)-C(10)-C(11)	120.9(3)
C(9)-C(10)-C(14)	118.8(3)	C(8)-C(9)-C(10)	119.7(3)
C(8)-C(9)-H(9)	120.1	C(10)-C(9)-H(9)	120.1
C(5)-C(7)-H(7A)	109.5	C(5)-C(7)-H(7B)	109.5
C(5)-C(7)-H(7C)	109.5	H(7A)-C(7)-H(7B)	109.5
H(7A)-C(7)-H(7C)	109.5	H(7B)-C(7)-H(7C)	109.5
F(1)-C(14)-C(10)	111.5(3)	F(2)-C(14)-F(1)	105.4(3)
F(2)-C(14)-F(3)	107.2(3)	F(2)-C(14)-C(10)	112.8(3)
F(3)-C(14)-F(1)	105.2(3)	F(3)-C(14)-C(10)	114.1(3)
C(16)-C(15)-S(2)	119.8(3)	C(16)-C(15)-C(20)	121.5(3)
C(20)-C(15)-S(2)	118.7(3)	C(15)-C(16)-H(16)	120.7
C(15)-C(16)-C(17)	118.6(4)	C(17)-C(16)-H(16)	120.7
C(16)-C(17)-H(17)	119.4	C(18)-C(17)-C(16)	121.2(4)
C(18)-C(17)-H(17)	119.4	C(17)-C(18)-C(19)	119.4(4)
C(17)-C(18)-C(21)	120.3(5)	C(19)-C(18)-C(21)	120.2(5)
C(18)-C(19)-H(19)	119.9	C(20)-C(19)-C(18)	120.1(4)
C(20)-C(19)-H(19)	119.9	C(15)-C(20)-H(20)	120.4

C(19)-C(20)-C(15)	119.2(4)	C(19)-C(20)-H(20)	120.4
C(18)-C(21)-H(21A)	109.5	C(18)-C(21)-H(21B)	109.5
C(18)-C(21)-H(21C)	109.5	H(21A)-C(21)-H(21B)	109.5
H(21A)-C(21)-H(21C)	109.5	H(21B)-C(21)-H(21C)	109.5
H(22B)-C(22)-H(22A)	108.8	Cl(1A)-C(22)-H(22B)	110.7
Cl(1A)-C(22)-H(22A)	110.7	Cl(1B)-C(22)-H(22B)	110.7
Cl(1B)-C(22)-H(22A)	110.7	Cl(1B)-C(22)-Cl(1A)	105.2(16)

8.9 Single crystal structure analysis of **82**

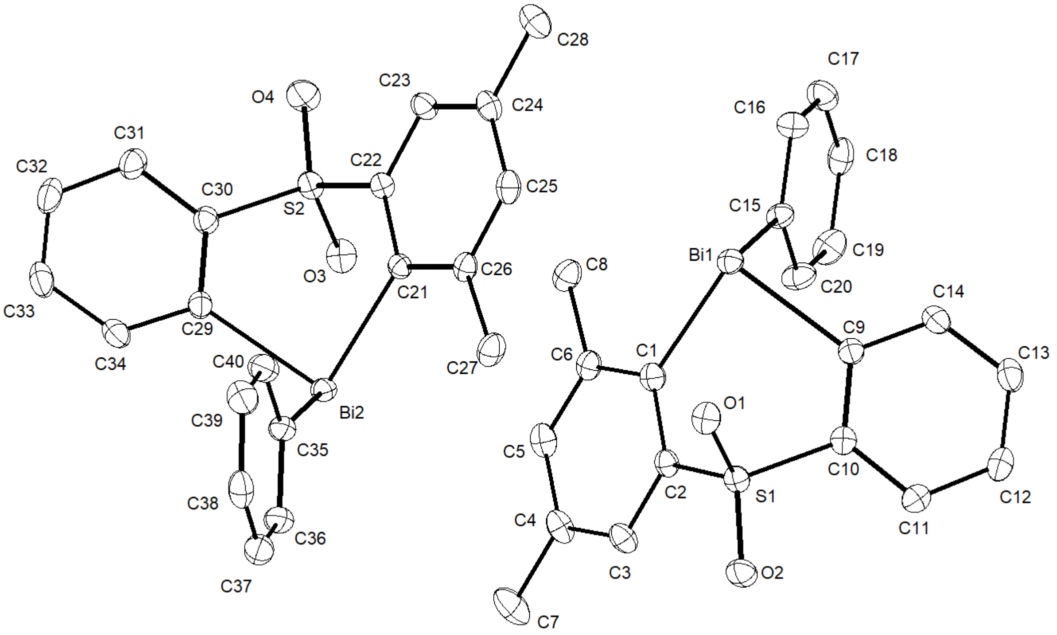

Figure S95. The molecular structure of complex **82**. H atoms have been removed for clarity.

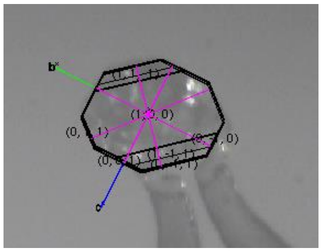

	h	k	l	distance (mm)
1.	1	0	0	0.10
2.	-1	0	0	0.06
3.	0	0	1	0.13
4.	0	-1	1	0.13
5.	0	1	-1	0.12
6.	0	-1	0	0.17
7.	0	1	0	0.14
8.	0	0	-1	0.13
9.	0	-1	-1	0.16
10.	0	1	1	0.15
11.	1	1	-1	0.13
12.	-1	1	-1	0.11
13.	1	-1	1	0.13
14.	-1	-1	1	0.12

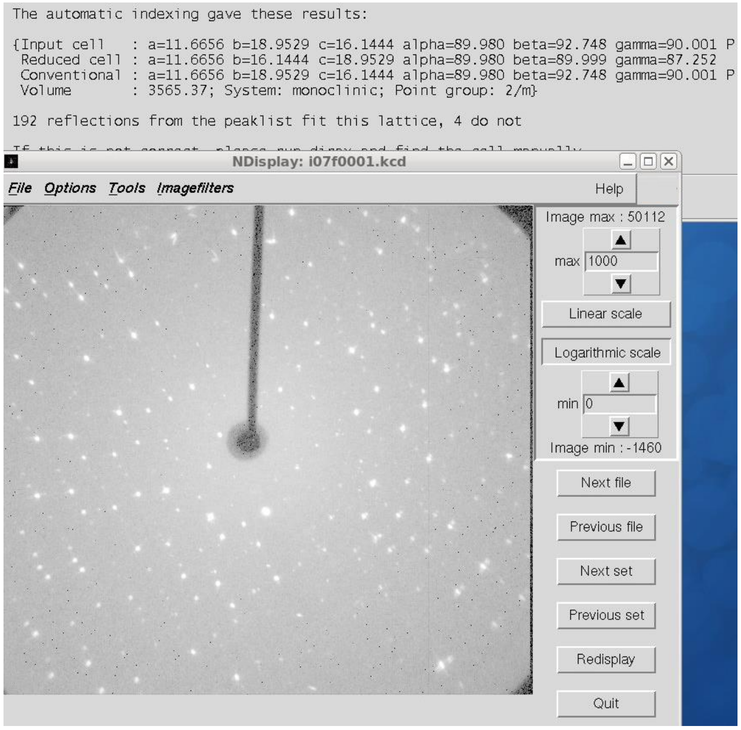

Figure S96. Crystal faces and unit cell determination of **82**.

INTENSITY STATISTICS FOR DATASET

Resolution	#Data	#Theory	%Complete	Redundancy	Mean I	Mean I/s	Rmerge	Rsigma
Inf - 2.45	225	282	79.8	4.66	85.91	52.44	0.0326	0.0200
2.45 - 1.69	530	555	95.5	4.72	109.66	47.18	0.0323	0.0213
1.69 - 1.36	734	738	99.5	4.79	102.74	42.82	0.0276	0.0196
1.36 - 1.19	763	764	99.9	4.64	73.78	39.81	0.0288	0.0190
1.19 - 1.08	759	761	99.7	4.50	67.23	37.95	0.0273	0.0197
1.08 - 1.01	694	695	99.9	4.26	54.90	35.39	0.0264	0.0212
1.01 - 0.95	752	755	99.6	4.08	47.23	32.33	0.0268	0.0222
0.95 - 0.90	771	771	100.0	3.90	39.34	30.23	0.0270	0.0237
0.90 - 0.86	786	786	100.0	3.70	36.32	28.56	0.0270	0.0249
0.86 - 0.83	671	673	99.7	3.53	28.86	26.71	0.0284	0.0270
0.83 - 0.80	782	783	99.9	3.45	27.89	26.48	0.0273	0.0278
0.80 - 0.77	894	896	99.8	3.25	26.02	25.00	0.0302	0.0292
0.77 - 0.75	690	690	100.0	3.15	22.42	23.95	0.0320	0.0307
0.75 - 0.73	759	761	99.7	2.98	19.35	21.45	0.0355	0.0341
0.73 - 0.71	870	873	99.7	2.85	19.62	19.68	0.0345	0.0357
0.71 - 0.70	456	456	100.0	2.86	18.23	19.72	0.0373	0.0377
0.70 - 0.68	1007	1007	100.0	2.71	16.12	17.51	0.0395	0.0421
0.68 - 0.67	558	558	100.0	2.66	12.99	15.36	0.0486	0.0498
0.67 - 0.65	1193	1196	99.7	2.55	13.13	14.81	0.0444	0.0536
0.65 - 0.64	931	936	99.5	2.43	10.78	11.87	0.0531	0.0709
0.74 - 0.64	5388	5400	99.8	2.67	15.10	16.47	0.0409	0.0462
Inf - 0.64	14825	14936	99.3	3.49	37.90	26.77	0.0298	0.0258

Several low angle reflections have likely been affected by the beam stop.

Complete .cif-data of the compound are available under the CCDC number **CCDC-2125773**.

Table S43. Crystal data and structure refinement of **82**.

Identification code	13366sadabs	
Empirical formula	$C_{20} H_{17} Bi O_2 S$	
Color	colourless	
Formula weight	530.38 g·mol^{-1}	
Temperature	100(2) K	
Wavelength	0.71073 Å	
Crystal system	Monoclinic	
Space group	$P2_1$/n, (no. 14)	
Unit cell dimensions	a = 11.678(2) Å	α= 90°.
	b = 18.979(2) Å	β= 92.751(12)°.
	c = 16.162(2) Å	γ = 90°.
Volume	3578.0(10) Å^3	
Z	8	
Density (calculated)	1.969 Mg·m^{-3}	
Absorption coefficient	9.981 mm^{-1}	
F(000)	2016 e	
Crystal size	0.26 x 0.25 x 0.16 mm^3	
θ range for data collection	3.076 to 32.032°.	
Index ranges	-17 ≤ h ≤ 17, -28≤ k ≤ 28, -24 ≤ l ≤ 24	
Reflections collected	46153	
Independent reflections	12366 [R_{int} = 0.0294]	
Reflections with I>2σ(I)	11021	
Completeness to θ = 25.242°	98.6 %	
Absorption correction	Gaussian	
Max. and min. transmission	0.24212 and 0.08881	
Refinement method	Full-matrix least-squares on F^2	
Data / restraints / parameters	12366 / 0 / 437	
Goodness-of-fit on F^2	1.108	
Final R indices [I>2σ(I)]	R_1 = 0.0215	wR^2 = 0.0514
R indices (all data)	R_1 = 0.0270	wR^2 = 0.0534
Extinction coefficient	n/a	
Largest diff. peak and hole	1.607 and -1.363 e·Å^{-3}	

Table S44. Bond lengths [Å] and angles [°] of **82**.

Bi(1)-C(15)	2.256(2)	Bi(1)-C(9)	2.272(2)
Bi(1)-C(1)	2.264(2)	Bi(2)-C(21)	2.268(2)
Bi(2)-C(29)	2.272(2)	Bi(2)-C(35)	2.262(2)
S(1)-O(2)	1.4468(17)	S(1)-O(1)	1.4481(18)
S(1)-C(10)	1.768(2)	S(1)-C(2)	1.771(2)
S(2)-O(4)	1.4413(18)	S(2)-O(3)	1.4483(18)
S(2)-C(22)	1.771(2)	S(2)-C(30)	1.764(2)
C(21)-C(22)	1.396(3)	C(21)-C(26)	1.402(3)
C(24)-C(23)	1.400(3)	C(24)-C(25)	1.393(3)
C(24)-C(28)	1.505(3)	C(15)-C(16)	1.393(3)
C(15)-C(20)	1.391(3)	C(33)-H(33)	0.9500
C(33)-C(34)	1.393(3)	C(33)-C(32)	1.379(4)
C(4)-C(3)	1.399(3)	C(4)-C(5)	1.392(3)
C(4)-C(7)	1.515(3)	C(9)-C(10)	1.394(3)
C(9)-C(14)	1.397(3)	C(16)-H(16)	0.9500
C(16)-C(17)	1.398(3)	C(27)-H(27A)	0.9800
C(27)-H(27B)	0.9800	C(27)-H(27C)	0.9800
C(27)-C(26)	1.505(3)	C(13)-H(13)	0.9500
C(13)-C(14)	1.392(3)	C(13)-C(12)	1.392(3)
C(3)-H(3)	0.9500	C(3)-C(2)	1.395(3)
C(10)-C(11)	1.399(3)	C(38)-H(38)	0.9500
C(38)-C(39)	1.399(4)	C(38)-C(37)	1.377(4)
C(29)-C(34)	1.397(3)	C(29)-C(30)	1.392(3)
C(22)-C(23)	1.389(3)	C(2)-C(1)	1.394(3)
C(23)-H(23)	0.9500	C(26)-C(25)	1.399(3)
C(11)-H(11)	0.9500	C(11)-C(12)	1.395(3)
C(31)-H(31)	0.9500	C(31)-C(32)	1.398(3)
C(31)-C(30)	1.395(3)	C(34)-H(34)	0.9500
C(1)-C(6)	1.404(3)	C(14)-H(14)	0.9500
C(20)-H(20)	0.9500	C(20)-C(19)	1.391(3)
C(25)-H(25)	0.9500	C(32)-H(32)	0.9500
C(12)-H(12)	0.9500	C(6)-C(5)	1.403(3)
C(6)-C(8)	1.510(3)	C(18)-H(18)	0.9500
C(18)-C(19)	1.388(4)	C(18)-C(17)	1.381(4)
C(5)-H(5)	0.9500	C(8)-H(8A)	0.9800

C(8)-H(8B)	0.9800	C(8)-H(8C)	0.9800
C(35)-C(40)	1.389(3)	C(35)-C(36)	1.399(3)
C(40)-H(40)	0.9500	C(40)-C(39)	1.392(3)
C(39)-H(39)	0.9500	C(36)-H(36)	0.9500
C(36)-C(37)	1.396(3)	C(28)-H(28A)	0.9800
C(28)-H(28B)	0.9800	C(28)-H(28C)	0.9800
C(37)-H(37)	0.9500	C(19)-H(19)	0.9500
C(17)-H(17)	0.9500	C(7)-H(7A)	0.9800
C(7)-H(7B)	0.9800	C(7)-H(7C)	0.9800

C(15)-Bi(1)-C(9)	89.14(7)	C(15)-Bi(1)-C(1)	93.91(8)
C(1)-Bi(1)-C(9)	88.82(8)	C(21)-Bi(2)-C(29)	88.80(8)
C(35)-Bi(2)-C(21)	95.84(8)	C(35)-Bi(2)-C(29)	91.84(8)
O(2)-S(1)-O(1)	118.17(10)	O(2)-S(1)-C(10)	109.56(10)
O(2)-S(1)-C(2)	109.79(10)	O(1)-S(1)-C(10)	105.82(10)
O(1)-S(1)-C(2)	106.81(10)	C(10)-S(1)-C(2)	105.97(9)
O(4)-S(2)-O(3)	119.13(11)	O(4)-S(2)-C(22)	109.72(10)
O(4)-S(2)-C(30)	109.63(11)	O(3)-S(2)-C(22)	106.27(10)
O(3)-S(2)-C(30)	105.97(11)	C(30)-S(2)-C(22)	105.21(10)
C(22)-C(21)-Bi(2)	119.51(14)	C(22)-C(21)-C(26)	117.91(19)
C(26)-C(21)-Bi(2)	122.58(15)	C(23)-C(24)-C(28)	120.5(2)
C(25)-C(24)-C(23)	118.0(2)	C(25)-C(24)-C(28)	121.5(2)
C(16)-C(15)-Bi(1)	119.62(16)	C(20)-C(15)-Bi(1)	121.58(16)
C(20)-C(15)-C(16)	118.8(2)	C(34)-C(33)-H(33)	119.7
C(32)-C(33)-H(33)	119.7	C(32)-C(33)-C(34)	120.7(2)
C(3)-C(4)-C(7)	120.3(2)	C(5)-C(4)-C(3)	118.3(2)
C(5)-C(4)-C(7)	121.4(2)	C(10)-C(9)-Bi(1)	121.81(15)
C(10)-C(9)-C(14)	117.5(2)	C(14)-C(9)-Bi(1)	120.65(16)
C(15)-C(16)-H(16)	119.8	C(15)-C(16)-C(17)	120.3(2)
C(17)-C(16)-H(16)	119.8	H(27A)-C(27)-H(27B)	109.5
H(27A)-C(27)-H(27C)	109.5	H(27B)-C(27)-H(27C)	109.5
C(26)-C(27)-H(27A)	109.5	C(26)-C(27)-H(27B)	109.5
C(26)-C(27)-H(27C)	109.5	C(14)-C(13)-H(13)	119.6
C(14)-C(13)-C(12)	120.8(2)	C(12)-C(13)-H(13)	119.6
C(4)-C(3)-H(3)	120.6	C(2)-C(3)-C(4)	118.8(2)
C(2)-C(3)-H(3)	120.6	C(9)-C(10)-S(1)	117.62(16)

C(9)-C(10)-C(11)	123.1(2)	C(11)-C(10)-S(1)	119.16(17)
C(39)-C(38)-H(38)	120.1	C(37)-C(38)-H(38)	120.1
C(37)-C(38)-C(39)	119.7(2)	C(34)-C(29)-Bi(2)	121.54(16)
C(30)-C(29)-Bi(2)	120.66(15)	C(30)-C(29)-C(34)	117.8(2)
C(21)-C(22)-S(2)	119.07(16)	C(23)-C(22)-S(2)	117.53(15)
C(23)-C(22)-C(21)	123.17(18)	C(3)-C(2)-S(1)	117.30(16)
C(1)-C(2)-S(1)	119.19(16)	C(1)-C(2)-C(3)	123.34(19)
C(24)-C(23)-H(23)	120.5	C(22)-C(23)-C(24)	119.1(2)
C(22)-C(23)-H(23)	120.5	C(21)-C(26)-C(27)	122.5(2)
C(25)-C(26)-C(21)	118.76(19)	C(25)-C(26)-C(27)	118.76(19)
C(10)-C(11)-H(11)	121.0	C(12)-C(11)-C(10)	117.9(2)
C(12)-C(11)-H(11)	121.0	C(32)-C(31)-H(31)	121.2
C(30)-C(31)-H(31)	121.2	C(30)-C(31)-C(32)	117.6(2)
C(33)-C(34)-C(29)	120.3(2)	C(33)-C(34)-H(34)	119.9
C(29)-C(34)-H(34)	119.9	C(2)-C(1)-Bi(1)	120.39(14)
C(2)-C(1)-C(6)	117.8(2)	C(6)-C(1)-Bi(1)	121.82(16)
C(9)-C(14)-H(14)	119.7	C(13)-C(14)-C(9)	120.5(2)
C(13)-C(14)-H(14)	119.7	C(15)-C(20)-H(20)	119.5
C(19)-C(20)-C(15)	121.0(2)	C(19)-C(20)-H(20)	119.5
C(24)-C(25)-C(26)	123.00(19)	C(24)-C(25)-H(25)	118.5
C(26)-C(25)-H(25)	118.5	C(33)-C(32)-C(31)	120.7(2)
C(33)-C(32)-H(32)	119.7	C(31)-C(32)-H(32)	119.7
C(13)-C(12)-C(11)	120.1(2)	C(13)-C(12)-H(12)	119.9
C(11)-C(12)-H(12)	119.9	C(1)-C(6)-C(8)	122.1(2)
C(5)-C(6)-C(1)	118.8(2)	C(5)-C(6)-C(8)	119.06(19)
C(19)-C(18)-H(18)	120.0	C(17)-C(18)-H(18)	120.0
C(17)-C(18)-C(19)	120.0(2)	C(4)-C(5)-C(6)	122.88(19)
C(4)-C(5)-H(5)	118.6	C(6)-C(5)-H(5)	118.6
C(6)-C(8)-H(8A)	109.5	C(6)-C(8)-H(8B)	109.5
C(6)-C(8)-H(8C)	109.5	H(8A)-C(8)-H(8B)	109.5
H(8A)-C(8)-H(8C)	109.5	H(8B)-C(8)-H(8C)	109.5
C(40)-C(35)-Bi(2)	124.28(16)	C(40)-C(35)-C(36)	118.8(2)
C(36)-C(35)-Bi(2)	116.86(17)	C(29)-C(30)-S(2)	118.08(16)
C(29)-C(30)-C(31)	123.0(2)	C(31)-C(30)-S(2)	118.90(17)
C(35)-C(40)-H(40)	119.7	C(35)-C(40)-C(39)	120.7(2)
C(39)-C(40)-H(40)	119.7	C(38)-C(39)-H(39)	120.0

C(40)-C(39)-C(38)	120.0(2)	C(40)-C(39)-H(39)	120.0
C(35)-C(36)-H(36)	119.8	C(37)-C(36)-C(35)	120.5(2)
C(37)-C(36)-H(36)	119.8	C(24)-C(28)-H(28A)	109.5
C(24)-C(28)-H(28B)	109.5	C(24)-C(28)-H(28C)	109.5
H(28A)-C(28)-H(28B)	109.5	H(28A)-C(28)-H(28C)	109.5
H(28B)-C(28)-H(28C)	109.5	C(38)-C(37)-C(36)	120.3(2)
C(38)-C(37)-H(37)	119.9	C(36)-C(37)-H(37)	119.9
C(20)-C(19)-H(19)	120.1	C(18)-C(19)-C(20)	119.8(2)
C(18)-C(19)-H(19)	120.1	C(16)-C(17)-H(17)	119.9
C(18)-C(17)-C(16)	120.2(2)	C(18)-C(17)-H(17)	119.9
C(4)-C(7)-H(7A)	109.5	C(4)-C(7)-H(7B)	109.5
C(4)-C(7)-H(7C)	109.5	H(7A)-C(7)-H(7B)	109.5
H(7A)-C(7)-H(7C)	109.5	H(7B)-C(7)-H(7C)	109.5

8.10 Single crystal structure analysis of **83**

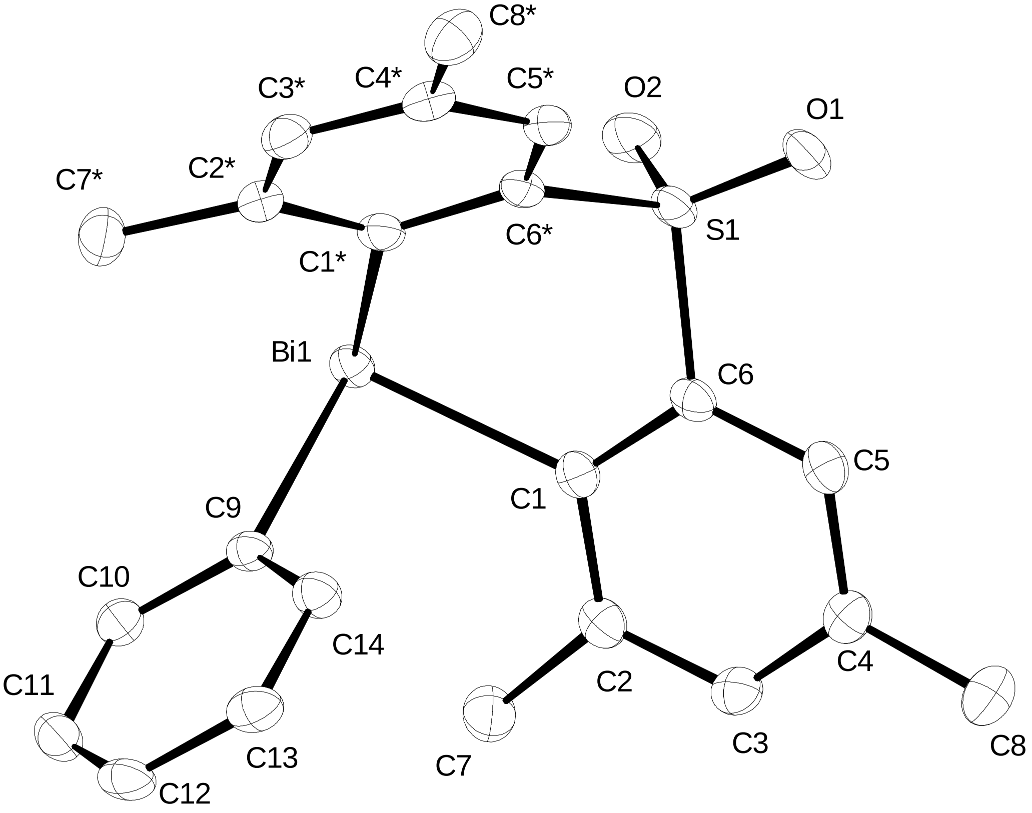

Figure S97. The molecular structure of complex **83**. H atoms have been removed for clarity.

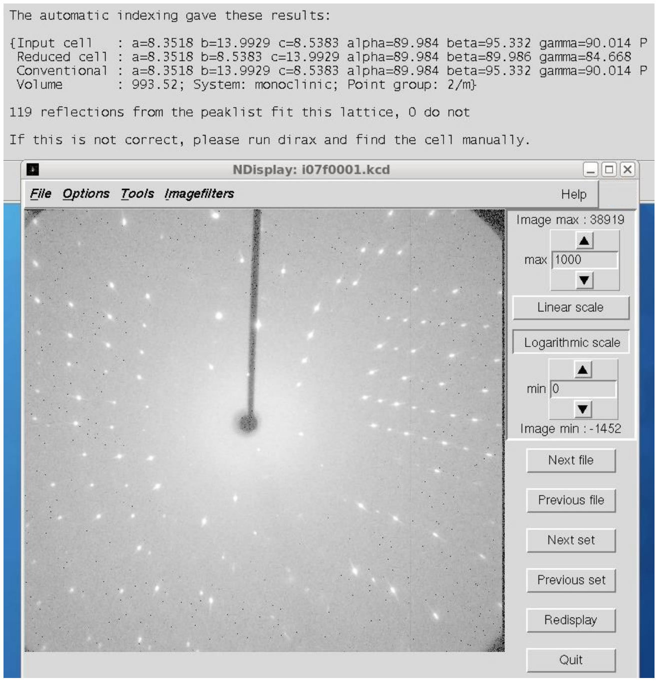

	h	k	l	distance (mm)
1.	-1	0	0	0.035
2.	1	0	0	0.035
3.	0	-1	1	0.07
4.	0	1	-1	0.04
5.	0	1	1	0.07
6.	0	-3	-1	0.06
7.	0	-1	0	0.08
8.	0	-1	-1	0.06
9.	0	1	0	0.08

Figure S98. Crystal faces and unit cell determination of **83**.

INTENSITY STATISTICS FOR DATASET

Resolution	#Data	#Theory	%Complete	Redundancy	Mean I	Mean I/s	Rmerge	Rsigma
Inf - 2.31	164	176	93.2	3.78	256.76	77.72	0.0273	0.0123
2.31 - 1.56	388	388	100.0	4.09	185.34	73.16	0.0180	0.0121
1.56 - 1.24	539	539	100.0	3.96	132.04	66.68	0.0181	0.0130
1.24 - 1.08	550	550	100.0	3.82	94.16	58.96	0.0176	0.0143
1.08 - 0.98	581	581	100.0	3.54	78.75	52.23	0.0182	0.0161
0.98 - 0.91	529	529	100.0	3.36	58.86	44.85	0.0200	0.0182
0.91 - 0.86	501	501	100.0	3.19	48.61	39.36	0.0206	0.0207
0.86 - 0.81	651	651	100.0	3.00	43.02	35.86	0.0235	0.0229
0.81 - 0.78	461	461	100.0	2.84	36.67	31.36	0.0248	0.0258
0.78 - 0.75	544	544	100.0	2.73	32.51	28.68	0.0266	0.0287
0.75 - 0.72	670	670	100.0	2.60	27.51	25.43	0.0302	0.0330
0.72 - 0.70	462	462	100.0	2.48	26.06	23.51	0.0318	0.0356
0.70 - 0.68	566	567	99.8	2.37	21.55	20.55	0.0349	0.0409
0.68 - 0.66	610	612	99.7	2.30	19.56	19.01	0.0398	0.0453
0.66 - 0.64	715	716	99.9	2.15	16.76	16.12	0.0486	0.0533
0.64 - 0.63	375	375	100.0	2.09	16.03	15.50	0.0512	0.0568
0.63 - 0.62	399	401	99.5	2.03	12.43	12.66	0.0575	0.0681
0.62 - 0.60	887	888	99.9	1.99	13.13	12.81	0.0569	0.0694
0.60 - 0.59	531	534	99.4	1.88	9.78	9.77	0.0769	0.0923
0.59 - 0.58	714	732	97.5	1.75	9.48	8.93	0.0777	0.1022
0.68 - 0.58	4231	4258	99.4	2.02	13.82	13.45	0.0536	0.0656
Inf - 0.58	10837	10877	99.6	2.71	46.32	30.74	0.0231	0.0236

Complete .cif-data of the compound are available under the CCDC number **CCDC-2125770.**

Table S45. Crystal data and structure refinement of **83**.

Identification code	12966sadabs	
Empirical formula	$C_{22} H_{21} Bi O_2 S$	
Color	colourless	
Formula weight	558.43 g·mol^{-1}	
Temperature	100(2) K	
Wavelength	0.71073 Å	
Crystal system	Monoclinic	
Space group	$P2_1$/m, (no. 11)	
Unit cell dimensions	a = 8.3414(5) Å	α= 90°.
	b = 13.9715(5) Å	β= 95.333(4)°.
	c = 8.5331(4) Å	γ = 90°.
Volume	990.16(8) Å^3	
Z	2	
Density (calculated)	1.873 Mg·m^{-3}	
Absorption coefficient	9.021 mm^{-1}	
F(000)	536 e	
Crystal size	0.16 x 0.14 x 0.07 mm^3	
θ range for data collection	2.806 to 27.499°.	
Index ranges	-10 ≤ h ≤ 10, -18≤ k ≤ 18, -11 ≤ l ≤ 11	
Reflections collected	15691	
Independent reflections	2366 [R_{int} = 0.0224]	
Reflections with I>2σ(I)	2340	
Completeness to θ = 25.242°	99.7 %	
Absorption correction	Gaussian	
Max. and min. transmission	0.55976 and 0.34887	
Refinement method	Full-matrix least-squares on F^2	
Data / restraints / parameters	2366 / 0 / 135	
Goodness-of-fit on F^2	1.200	
Final R indices [I>2σ(I)]	R_1 = 0.0132	wR^2 = 0.0329
R indices (all data)	R_1 = 0.0134	wR^2 = 0.0330
Extinction coefficient	n/a	
Largest diff. peak and hole	1.586 and -0.709 e·Å^{-3}	

Table S46. Bond lengths [Å] and angles [°] of **83**.

Bi(1)-C(1)	2.272(2)	Bi(1)-C(1)#1	2.272(2)
Bi(1)-C(9)	2.258(3)	S(1)-O(1)	1.449(2)
S(1)-O(2)	1.441(2)	S(1)-C(6)	1.765(2)
S(1)-C(6)#1	1.765(2)	C(1)-C(2)	1.399(3)
C(1)-C(6)	1.399(3)	C(2)-C(3)	1.399(3)
C(2)-C(7)	1.513(3)	C(3)-H(3)	0.9500
C(3)-C(4)	1.391(3)	C(4)-C(5)	1.388(3)
C(4)-C(8)	1.509(3)	C(5)-H(5)	0.9500
C(5)-C(6)	1.386(3)	C(7)-H(7A)	0.9800
C(7)-H(7B)	0.9800	C(7)-H(7C)	0.9800
C(8)-H(8A)	0.9800	C(8)-H(8B)	0.9800
C(8)-H(8C)	0.9800	C(9)-C(10)	1.393(4)
C(9)-C(14)	1.392(4)	C(10)-H(10)	0.9500
C(10)-C(11)	1.399(5)	C(11)-H(11)	0.9500
C(11)-C(12)	1.385(5)	C(12)-H(12)	0.9500
C(12)-C(13)	1.379(5)	C(13)-H(13)	0.9500
C(13)-C(14)	1.395(4)	C(14)-H(14)	0.9500
C(1)#1-Bi(1)-C(1)	91.08(10)	C(9)-Bi(1)-C(1)	88.95(7)
C(9)-Bi(1)-C(1)#1	88.95(7)	O(1)-S(1)-C(6)#1	108.61(9)
O(1)-S(1)-C(6)	108.61(9)	O(2)-S(1)-O(1)	118.48(14)
O(2)-S(1)-C(6)	107.28(9)	O(2)-S(1)-C(6)#1	107.28(9)
C(6)-S(1)-C(6)#1	105.90(13)	C(2)-C(1)-Bi(1)	121.24(15)
C(2)-C(1)-C(6)	117.50(19)	C(6)-C(1)-Bi(1)	121.03(15)
C(1)-C(2)-C(3)	119.03(19)	C(1)-C(2)-C(7)	122.38(19)
C(3)-C(2)-C(7)	118.59(19)	C(2)-C(3)-H(3)	118.6
C(4)-C(3)-C(2)	122.8(2)	C(4)-C(3)-H(3)	118.6
C(3)-C(4)-C(8)	121.4(2)	C(5)-C(4)-C(3)	118.0(2)
C(5)-C(4)-C(8)	120.6(2)	C(4)-C(5)-H(5)	120.3
C(6)-C(5)-C(4)	119.5(2)	C(6)-C(5)-H(5)	120.3
C(1)-C(6)-S(1)	118.73(16)	C(5)-C(6)-S(1)	118.17(16)
C(5)-C(6)-C(1)	123.02(19)	C(2)-C(7)-H(7A)	109.5
C(2)-C(7)-H(7B)	109.5	C(2)-C(7)-H(7C)	109.5
H(7A)-C(7)-H(7B)	109.5	H(7A)-C(7)-H(7C)	109.5
H(7B)-C(7)-H(7C)	109.5	C(4)-C(8)-H(8A)	109.5

C(4)-C(8)-H(8B)	109.5	C(4)-C(8)-H(8C)	109.5
H(8A)-C(8)-H(8B)	109.5	H(8A)-C(8)-H(8C)	109.5
H(8B)-C(8)-H(8C)	109.5	C(10)-C(9)-Bi(1)	121.1(2)
C(14)-C(9)-Bi(1)	119.5(2)	C(14)-C(9)-C(10)	119.4(3)
C(9)-C(10)-H(10)	119.9	C(9)-C(10)-C(11)	120.1(3)
C(11)-C(10)-H(10)	119.9	C(10)-C(11)-H(11)	120.1
C(12)-C(11)-C(10)	119.9(3)	C(12)-C(11)-H(11)	120.1
C(11)-C(12)-H(12)	119.9	C(13)-C(12)-C(11)	120.2(3)
C(13)-C(12)-H(12)	119.9	C(12)-C(13)-H(13)	119.8
C(12)-C(13)-C(14)	120.3(3)	C(14)-C(13)-H(13)	119.8
C(9)-C(14)-C(13)	120.1(3)	C(9)-C(14)-H(14)	120.0
C(13)-C(14)-H(14)	120.0		

Symmetry transformations used to generate equivalent atoms:

#1 x,-y+1/2,z

8.11 Single crystal structure analysis of **84**

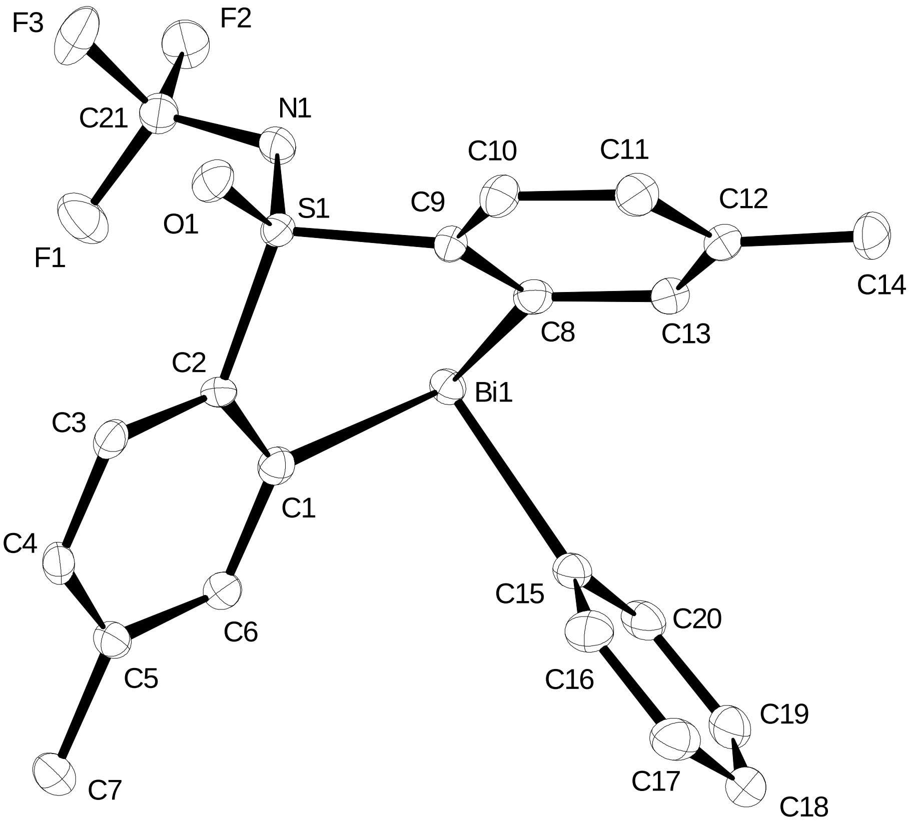

Figure S99. The molecular structure of complex **84**. H atoms have been removed for clarity.

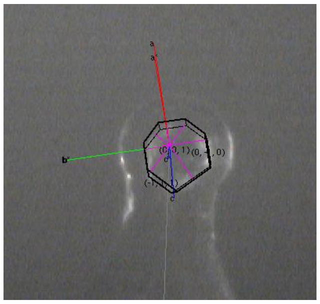

	h	k	l	distance (mm)
1.	0	0	-1	0.025
2.	0	0	1	0.025
3.	0	-1	0	0.09
4.	1	-1	-1	0.065
5.	-2	-1	1	0.1
6.	0	1	0	0.06
7.	-1	1	1	0.08
8.	1	1	-1	0.045
9.	1	0	-1	0.045

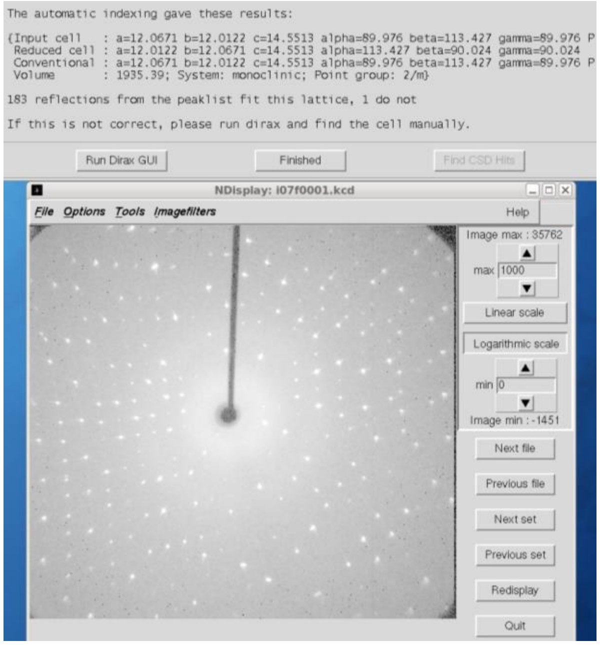

Figure S100. Crystal faces and unit cell determination of **84**.

INTENSITY STATISTICS FOR DATASET

Resolution	#Data	#Theory	%Complete	Redundancy	Mean I	Mean I/s	Rmerge	Rsigma
Inf - 2.70	115	123	93.5	6.65	178.07	44.73	0.0472	0.0254
2.70 - 1.80	272	272	100.0	7.41	134.96	44.51	0.0431	0.0189
1.80 - 1.42	386	386	100.0	7.45	109.93	44.14	0.0432	0.0192
1.42 - 1.24	378	378	100.0	7.30	77.53	40.70	0.0404	0.0191
1.24 - 1.12	385	385	100.0	6.89	63.56	37.92	0.0387	0.0208
1.12 - 1.04	386	386	100.0	6.59	58.14	35.42	0.0374	0.0216
1.04 - 0.98	374	374	100.0	6.22	47.90	31.96	0.0390	0.0230
0.98 - 0.92	464	464	100.0	5.91	45.50	31.23	0.0374	0.0240
0.92 - 0.88	397	397	100.0	5.71	33.63	28.05	0.0378	0.0262
0.88 - 0.85	330	330	100.0	5.33	35.79	27.26	0.0385	0.0274
0.85 - 0.82	388	388	100.0	5.21	29.55	25.36	0.0401	0.0290
0.82 - 0.79	459	459	100.0	4.91	26.78	23.21	0.0401	0.0315
0.79 - 0.77	339	339	100.0	4.87	24.60	22.15	0.0471	0.0333
0.77 - 0.75	375	375	100.0	4.50	22.96	20.85	0.0462	0.0359
0.75 - 0.73	422	422	100.0	4.44	21.70	19.80	0.0464	0.0374
0.73 - 0.71	459	459	100.0	4.22	19.59	17.96	0.0519	0.0408
0.71 - 0.70	261	261	100.0	4.07	16.52	16.93	0.0562	0.0436
0.70 - 0.68	558	558	100.0	3.99	16.59	16.61	0.0532	0.0458
0.68 - 0.67	287	287	100.0	3.75	15.04	14.86	0.0586	0.0503
0.67 - 0.66	339	339	100.0	3.68	14.67	14.39	0.0629	0.0523
0.66 - 0.65	289	294	98.3	3.56	12.95	13.89	0.0649	0.0563
0.75 - 0.65	2615	2620	99.8	3.99	17.11	16.61	0.0540	0.0449
Inf - 0.65	7663	7676	99.8	5.32	42.47	26.59	0.0423	0.0261

Complete .cif-data of the compound are available under the CCDC number **CCDC-2125777.**

Table S47. Crystal data and structure refinement of **84**.

Identification code	13398sadabs	
Empirical formula	$C_{21} H_{17} Bi F_3 N O S$	
Color	colourless	
Formula weight	597.40 g·mol^{-1}	
Temperature	100(2) K	
Wavelength	0.71073 Å	
Crystal system	Monoclinic	
Space group	$P2_1$/c, (no. 14)	
Unit cell dimensions	a = 14.5638(18) Å	α= 90°.
	b = 12.0288(11) Å	β= 113.442(5)°.
	c = 12.0882(3) Å	γ = 90°.
Volume	1942.9(3) Å^3	
Z	4	
Density (calculated)	2.042 Mg·m^{-3}	
Absorption coefficient	9.221 mm^{-1}	
F(000)	1136 e	
Crystal size	0.15 x 0.14 x 0.05 mm^3	
θ range for data collection	3.049 to 27.500°.	
Index ranges	-18 ≤ h ≤ 18, -15≤ k ≤ 15, -15 ≤ l ≤ 15	
Reflections collected	27988	
Independent reflections	4458 [R_{int} = 0.0410]	
Reflections with I>2σ(I)	4305	
Completeness to θ = 25.242°	99.9 %	
Absorption correction	Gaussian	
Max. and min. transmission	0.64788 and 0.31395	
Refinement method	Full-matrix least-squares on F^2	
Data / restraints / parameters	4458 / 0 / 255	
Goodness-of-fit on F^2	1.166	
Final R indices [I>2σ(I)]	R_1 = 0.0237	wR^2 = 0.0615
R indices (all data)	R_1 = 0.0247	wR^2 = 0.0621
Extinction coefficient	n/a	
Largest diff. peak and hole	2.160 and -1.636 e·Å^{-3}	

Table S48. Bond lengths [Å] and angles [°] of **84**.

Bi(1)-C(1)	2.271(3)	Bi(1)-C(8)	2.262(3)
Bi(1)-C(15)	2.252(3)	S(1)-O(1)	1.449(2)
S(1)-N(1)	1.553(3)	S(1)-C(2)	1.769(3)
S(1)-C(9)	1.762(3)	F(1)-C(21)	1.358(4)
F(2)-C(21)	1.340(4)	F(3)-C(21)	1.352(4)
N(1)-C(21)	1.383(4)	C(1)-C(2)	1.391(4)
C(1)-C(6)	1.393(5)	C(2)-C(3)	1.405(4)
C(3)-H(3)	0.9500	C(3)-C(4)	1.388(5)
C(4)-H(4)	0.9500	C(4)-C(5)	1.396(5)
C(5)-C(6)	1.405(5)	C(5)-C(7)	1.503(5)
C(6)-H(6)	0.9500	C(7)-H(7A)	0.9800
C(7)-H(7B)	0.9800	C(7)-H(7C)	0.9800
C(8)-C(9)	1.401(5)	C(8)-C(13)	1.391(5)
C(9)-C(10)	1.388(5)	C(10)-H(10)	0.9500
C(10)-C(11)	1.389(5)	C(11)-H(11)	0.9500
C(11)-C(12)	1.396(5)	C(12)-C(13)	1.395(5)
C(12)-C(14)	1.508(5)	C(13)-H(13)	0.9500
C(14)-H(14A)	0.9800	C(14)-H(14B)	0.9800
C(14)-H(14C)	0.9800	C(15)-C(16)	1.394(5)
C(15)-C(20)	1.402(5)	C(16)-H(16)	0.9500
C(16)-C(17)	1.393(5)	C(17)-H(17)	0.9500
C(17)-C(18)	1.390(6)	C(18)-H(18)	0.9500
C(18)-C(19)	1.391(6)	C(19)-H(19)	0.9500
C(19)-C(20)	1.387(5)	C(20)-H(20)	0.9500
C(8)-Bi(1)-C(1)	88.46(12)	C(15)-Bi(1)-C(1)	93.68(12)
C(15)-Bi(1)-C(8)	90.70(12)	O(1)-S(1)-N(1)	120.27(15)
O(1)-S(1)-C(2)	108.97(15)	O(1)-S(1)-C(9)	110.36(16)
N(1)-S(1)-C(2)	109.68(16)	N(1)-S(1)-C(9)	99.90(15)
C(9)-S(1)-C(2)	106.66(15)	C(21)-N(1)-S(1)	120.8(2)
C(2)-C(1)-Bi(1)	122.1(2)	C(2)-C(1)-C(6)	117.8(3)
C(6)-C(1)-Bi(1)	120.1(2)	C(1)-C(2)-S(1)	119.9(2)
C(1)-C(2)-C(3)	122.4(3)	C(3)-C(2)-S(1)	117.6(2)
C(2)-C(3)-H(3)	120.9	C(4)-C(3)-C(2)	118.2(3)
C(4)-C(3)-H(3)	120.9	C(3)-C(4)-H(4)	119.5

C(3)-C(4)-C(5)	121.1(3)	C(5)-C(4)-H(4)	119.5
C(4)-C(5)-C(6)	119.1(3)	C(4)-C(5)-C(7)	119.8(3)
C(6)-C(5)-C(7)	121.1(3)	C(1)-C(6)-C(5)	121.3(3)
C(1)-C(6)-H(6)	119.3	C(5)-C(6)-H(6)	119.3
C(5)-C(7)-H(7A)	109.5	C(5)-C(7)-H(7B)	109.5
C(5)-C(7)-H(7C)	109.5	H(7A)-C(7)-H(7B)	109.5
H(7A)-C(7)-H(7C)	109.5	H(7B)-C(7)-H(7C)	109.5
C(9)-C(8)-Bi(1)	121.1(2)	C(13)-C(8)-Bi(1)	121.6(2)
C(13)-C(8)-C(9)	117.2(3)	C(8)-C(9)-S(1)	120.7(3)
C(10)-C(9)-S(1)	116.9(3)	C(10)-C(9)-C(8)	122.4(3)
C(9)-C(10)-H(10)	120.6	C(9)-C(10)-C(11)	118.8(3)
C(11)-C(10)-H(10)	120.6	C(10)-C(11)-H(11)	119.7
C(10)-C(11)-C(12)	120.5(3)	C(12)-C(11)-H(11)	119.7
C(11)-C(12)-C(14)	120.2(3)	C(13)-C(12)-C(11)	119.2(3)
C(13)-C(12)-C(14)	120.6(3)	C(8)-C(13)-C(12)	121.7(3)
C(8)-C(13)-H(13)	119.1	C(12)-C(13)-H(13)	119.1
C(12)-C(14)-H(14A)	109.5	C(12)-C(14)-H(14B)	109.5
C(12)-C(14)-H(14C)	109.5	H(14A)-C(14)-H(14B)	109.5
H(14A)-C(14)-H(14C)	109.5	H(14B)-C(14)-H(14C)	109.5
C(16)-C(15)-Bi(1)	122.6(2)	C(16)-C(15)-C(20)	118.8(3)
C(20)-C(15)-Bi(1)	118.4(2)	C(15)-C(16)-H(16)	119.6
C(17)-C(16)-C(15)	120.9(3)	C(17)-C(16)-H(16)	119.6
C(16)-C(17)-H(17)	120.1	C(18)-C(17)-C(16)	119.8(4)
C(18)-C(17)-H(17)	120.1	C(17)-C(18)-H(18)	120.1
C(17)-C(18)-C(19)	119.8(3)	C(19)-C(18)-H(18)	120.1
C(18)-C(19)-H(19)	119.8	C(20)-C(19)-C(18)	120.4(3)
C(20)-C(19)-H(19)	119.8	C(15)-C(20)-H(20)	119.8
C(19)-C(20)-C(15)	120.3(4)	C(19)-C(20)-H(20)	119.8
F(1)-C(21)-N(1)	115.6(3)	F(2)-C(21)-F(1)	106.0(3)
F(2)-C(21)-F(3)	105.9(3)	F(2)-C(21)-N(1)	109.0(3)
F(3)-C(21)-F(1)	105.2(3)	F(3)-C(21)-N(1)	114.3(3)

8.12 Single crystal structure analysis of **87**

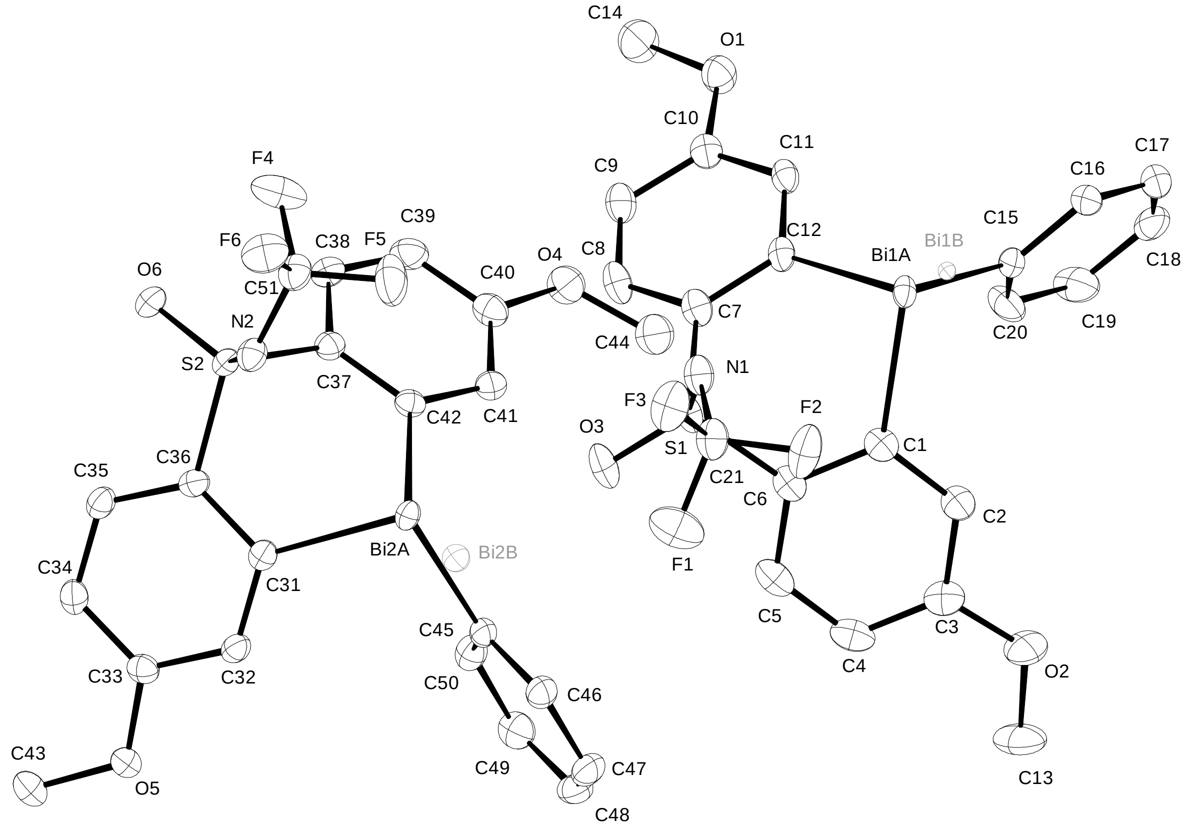

Figure S101. The molecular structure of complex **87**. H atoms have been removed for clarity.

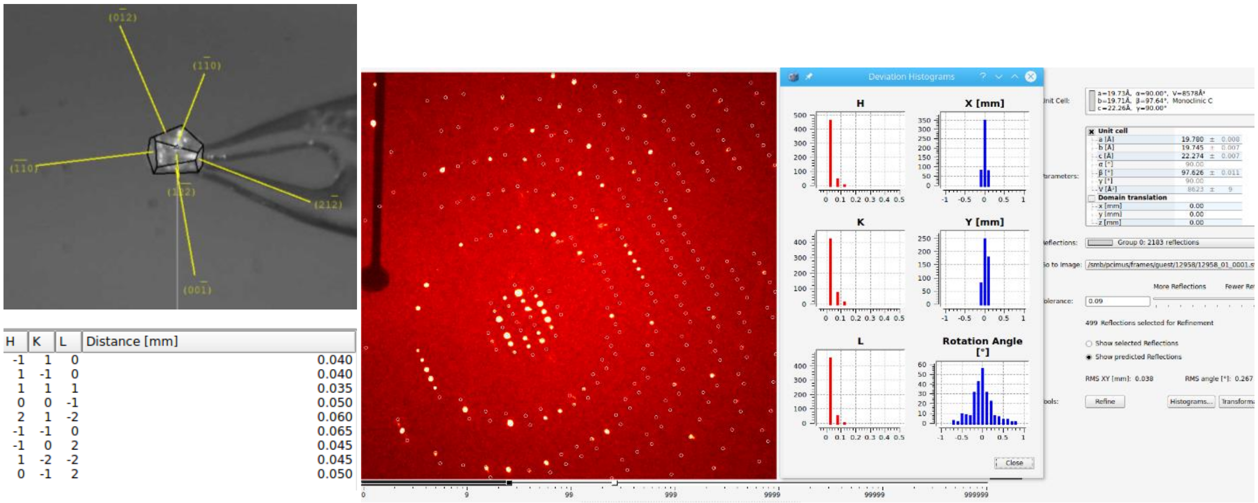

Figure S102. Crystal faces and unit cell determination of **87**.

INTENSITY STATISTICS FOR DATASET

Resolution	#Data	#Theory	%Complete	Redundancy	Mean I	Mean I/s	Rmerge	Rsigma
Inf - 2.44	325	326	99.7	17.23	99.06	141.82	0.0207	0.0060
2.44 - 1.62	760	760	100.0	18.11	65.40	123.87	0.0178	0.0061
1.62 - 1.28	1083	1083	100.0	18.14	44.97	112.66	0.0186	0.0065
1.28 - 1.11	1164	1164	100.0	17.33	30.48	91.58	0.0222	0.0076
1.11 - 1.01	1065	1065	100.0	12.79	27.11	70.93	0.0244	0.0097
1.01 - 0.94	1044	1044	100.0	9.95	21.53	55.03	0.0282	0.0125
0.94 - 0.88	1154	1154	100.0	8.38	17.18	46.18	0.0335	0.0157
0.88 - 0.83	1268	1268	100.0	7.65	14.77	38.34	0.0373	0.0186
0.83 - 0.80	892	892	100.0	7.25	11.65	31.76	0.0436	0.0226
0.80 - 0.77	1047	1047	100.0	6.95	12.10	30.46	0.0430	0.0236
0.77 - 0.74	1245	1245	100.0	6.67	10.42	27.03	0.0489	0.0276
0.74 - 0.72	917	917	100.0	6.38	9.10	23.16	0.0551	0.0321
0.72 - 0.70	1044	1044	100.0	6.17	8.31	21.10	0.0590	0.0362
0.70 - 0.68	1195	1195	100.0	5.94	7.79	19.42	0.0627	0.0397
0.68 - 0.66	1290	1290	100.0	5.69	6.64	16.88	0.0724	0.0475
0.66 - 0.64	1502	1506	99.7	5.42	5.89	14.69	0.0810	0.0552
0.64 - 0.63	798	798	100.0	5.18	5.56	13.58	0.0867	0.0611
0.63 - 0.62	887	896	99.0	5.07	5.04	12.29	0.0959	0.0678
0.62 - 0.61	905	912	99.2	4.92	4.74	11.37	0.1028	0.0742
0.61 - 0.59	1842	2043	90.2	3.28	4.21	8.43	0.1140	0.1120
0.59 - 0.58	245	882	27.8	0.40	3.24	4.02	0.1409	0.2400
0.68 - 0.58	7469	8327	89.7	4.29	5.24	12.37	0.0885	0.0727
Inf - 0.58	21672	22531	96.2	7.98	16.50	39.52	0.0272	0.0196

High residual density around the Bi atoms is possibly caused by anharmonic displacement of the heavy atoms. This has been taken into account by splitting the Bi atoms in two parts before the final refinement cycles. The structure contains a short inter D...A contact at O2 with no evidence of a missing hydrogen atom in the residual electron density map.

Complete .cif-data of the compound are available under the CCDC number **CCDC-2125782.**

Table S49. Crystal data and structure refinement of **87**.

Identification code	12958sadabs	
Empirical formula	$C_{21} H_{17} Bi F_3 N O_3 S$	
Color	colourless	
Formula weight	629.40 g·mol^{-1}	
Temperature	100(2) K	
Wavelength	0.71073 Å	
Crystal system	Monoclinic	
Space group	*C*2/c, (no. 15)	
Unit cell dimensions	a = 19.5226(11) Å	α= 90°.
	b = 19.4795(11) Å	β= 97.816(2)°.
	c = 22.0328(13) Å	γ = 90°.
Volume	8301.0(8) Å^3	
Z	16	
Density (calculated)	2.014 Mg·m^{-3}	
Absorption coefficient	8.646 mm^{-1}	
F(000)	4800 e	
Crystal size	0.131 x 0.115 x 0.081 mm^3	
θ range for data collection	1.484 to 27.500°.	
Index ranges	-25 ≤ h ≤ 25, -25≤ k ≤ 25, -28 ≤ l ≤ 28	
Reflections collected	114645	
Independent reflections	9541 [R_{int} = 0.0226]	
Reflections with I>2σ(I)	9092	
Completeness to θ = 25.242°	100.0 %	
Absorption correction	Gaussian	
Max. and min. transmission	0.56751 and 0.44320	
Refinement method	Full-matrix least-squares on F^2	
Data / restraints / parameters	9541 / 0 / 553	
Goodness-of-fit on F^2	1.119	
Final R indices [I>2σ(I)]	R_1 = 0.0148	wR^2 = 0.0330
R indices (all data)	R_1 = 0.0162	wR^2 = 0.0334
Extinction coefficient	n/a	
Largest diff. peak and hole	1.093 and -0.562 e·Å^{-3}	

Table S50. Bond lengths [Å] and angles [°] of **87**.

Bi(1A)-C(1)	2.266(2)	Bi(1A)-C(12)	2.259(2)
Bi(1A)-C(15)	2.253(2)	Bi(1B)-C(1)	2.636(5)
Bi(1B)-C(12)	2.589(5)	Bi(1B)-C(15)	1.678(5)
Bi(1B)-C(16)	2.457(5)	Bi(1B)-C(20)	2.826(5)
S(1)-O(3)	1.4467(17)	S(1)-N(1)	1.550(2)
S(1)-C(6)	1.765(2)	S(1)-C(7)	1.760(2)
F(1)-C(21)	1.350(3)	F(2)-C(21)	1.345(3)
F(3)-C(21)	1.342(3)	O(1)-C(10)	1.356(3)
O(1)-C(14)	1.430(3)	O(2)-C(3)	1.350(3)
O(2)-C(13)	1.426(3)	N(1)-C(21)	1.388(3)
C(1)-C(2)	1.382(3)	C(1)-C(6)	1.397(3)
C(2)-H(2)	0.9500	C(2)-C(3)	1.399(3)
C(3)-C(4)	1.387(4)	C(4)-H(4)	0.9500
C(4)-C(5)	1.382(4)	C(5)-H(5)	0.9500
C(5)-C(6)	1.393(3)	C(7)-C(8)	1.386(3)
C(7)-C(12)	1.399(3)	C(8)-H(8)	0.9500
C(8)-C(9)	1.382(4)	C(9)-H(9)	0.9500
C(9)-C(10)	1.398(3)	C(10)-C(11)	1.396(3)
C(11)-H(11)	0.9500	C(11)-C(12)	1.380(3)
C(13)-H(13A)	0.9800	C(13)-H(13B)	0.9800
C(13)-H(13C)	0.9800	C(14)-H(14A)	0.9800
C(14)-H(14B)	0.9800	C(14)-H(14C)	0.9800
C(15)-C(16)	1.395(3)	C(15)-C(20)	1.378(4)
C(16)-H(16)	0.9500	C(16)-C(17)	1.382(4)
C(17)-H(17)	0.9500	C(17)-C(18)	1.367(5)
C(18)-H(18)	0.9500	C(18)-C(19)	1.392(4)
C(19)-H(19)	0.9500	C(19)-C(20)	1.393(3)
C(20)-H(20)	0.9500	Bi(2A)-C(31)	2.257(2)
Bi(2A)-C(42)	2.279(2)	Bi(2A)-C(45)	2.247(2)
Bi(2B)-C(31)	2.787(8)	Bi(2B)-C(42)	2.718(8)
Bi(2B)-C(45)	1.480(9)	Bi(2B)-C(46)	2.232(9)
Bi(2B)-C(50)	2.688(8)	S(2)-O(6)	1.4488(16)
S(2)-N(2)	1.549(2)	S(2)-C(36)	1.758(2)
S(2)-C(37)	1.777(2)	F(4)-C(51)	1.339(3)
F(5)-C(51)	1.352(3)	F(6)-C(51)	1.339(3)

O(4)-C(40)	1.358(3)	O(4)-C(44)	1.433(3)
O(5)-C(33)	1.359(3)	O(5)-C(43)	1.430(3)
N(2)-C(51)	1.384(3)	C(31)-C(32)	1.383(3)
C(31)-C(36)	1.401(3)	C(32)-H(32)	0.9500
C(32)-C(33)	1.398(3)	C(33)-C(34)	1.395(3)
C(34)-H(34)	0.9500	C(34)-C(35)	1.387(3)
C(35)-H(35)	0.9500	C(35)-C(36)	1.382(3)
C(37)-C(38)	1.392(3)	C(37)-C(42)	1.393(3)
C(38)-H(38)	0.9500	C(38)-C(39)	1.382(3)
C(39)-H(39)	0.9500	C(39)-C(40)	1.391(3)
C(40)-C(41)	1.399(3)	C(41)-H(41)	0.9500
C(41)-C(42)	1.398(3)	C(43)-H(43A)	0.9800
C(43)-H(43B)	0.9800	C(43)-H(43C)	0.9800
C(44)-H(44A)	0.9800	C(44)-H(44B)	0.9800
C(44)-H(44C)	0.9800	C(45)-C(46)	1.396(3)
C(45)-C(50)	1.389(3)	C(46)-H(46)	0.9500
C(46)-C(47)	1.394(3)	C(47)-H(47)	0.9500
C(47)-C(48)	1.383(4)	C(48)-H(48)	0.9500
C(48)-C(49)	1.388(4)	C(49)-H(49)	0.9500
C(49)-C(50)	1.386(3)	C(50)-H(50)	0.9500

C(12)-Bi(1A)-C(1)	86.02(8)	C(15)-Bi(1A)-C(1)	92.90(8)
C(15)-Bi(1A)-C(12)	91.41(8)	C(1)-Bi(1B)-C(20)	78.07(11)
C(12)-Bi(1B)-C(1)	72.40(13)	C(12)-Bi(1B)-C(20)	82.12(12)
C(15)-Bi(1B)-C(1)	96.22(17)	C(15)-Bi(1B)-C(12)	96.00(17)
C(15)-Bi(1B)-C(16)	33.12(13)	C(15)-Bi(1B)-C(20)	20.21(11)
C(16)-Bi(1B)-C(1)	124.74(17)	C(16)-Bi(1B)-C(12)	118.25(16)
C(16)-Bi(1B)-C(20)	53.32(12)	O(3)-S(1)-N(1)	119.51(11)
O(3)-S(1)-C(6)	108.16(11)	O(3)-S(1)-C(7)	109.73(11)
N(1)-S(1)-C(6)	109.95(11)	N(1)-S(1)-C(7)	101.99(11)
C(7)-S(1)-C(6)	106.75(11)	C(10)-O(1)-C(14)	117.77(19)
C(3)-O(2)-C(13)	118.7(2)	C(21)-N(1)-S(1)	119.31(18)
C(2)-C(1)-Bi(1A)	120.80(17)	C(2)-C(1)-Bi(1B)	108.16(18)
C(2)-C(1)-C(6)	117.6(2)	C(6)-C(1)-Bi(1A)	121.59(17)
C(6)-C(1)-Bi(1B)	133.72(19)	C(1)-C(2)-H(2)	119.4
C(1)-C(2)-C(3)	121.1(2)	C(3)-C(2)-H(2)	119.4

O(2)-C(3)-C(2)	114.6(2)	O(2)-C(3)-C(4)	125.1(2)
C(4)-C(3)-C(2)	120.3(2)	C(3)-C(4)-H(4)	120.2
C(5)-C(4)-C(3)	119.5(2)	C(5)-C(4)-H(4)	120.2
C(4)-C(5)-H(5)	120.2	C(4)-C(5)-C(6)	119.6(2)
C(6)-C(5)-H(5)	120.2	C(1)-C(6)-S(1)	119.68(18)
C(5)-C(6)-S(1)	118.40(18)	C(5)-C(6)-C(1)	121.9(2)
C(8)-C(7)-S(1)	117.14(18)	C(8)-C(7)-C(12)	121.9(2)
C(12)-C(7)-S(1)	120.88(18)	C(7)-C(8)-H(8)	119.9
C(9)-C(8)-C(7)	120.1(2)	C(9)-C(8)-H(8)	119.9
C(8)-C(9)-H(9)	120.7	C(8)-C(9)-C(10)	118.6(2)
C(10)-C(9)-H(9)	120.7	O(1)-C(10)-C(9)	123.8(2)
O(1)-C(10)-C(11)	115.5(2)	C(11)-C(10)-C(9)	120.7(2)
C(10)-C(11)-H(11)	119.5	C(12)-C(11)-C(10)	121.0(2)
C(12)-C(11)-H(11)	119.5	C(7)-C(12)-Bi(1A)	120.43(17)
C(7)-C(12)-Bi(1B)	134.20(19)	C(11)-C(12)-Bi(1A)	121.91(16)
C(11)-C(12)-Bi(1B)	108.10(17)	C(11)-C(12)-C(7)	117.6(2)
O(2)-C(13)-H(13A)	109.5	O(2)-C(13)-H(13B)	109.5
O(2)-C(13)-H(13C)	109.5	H(13A)-C(13)-H(13B)	109.5
H(13A)-C(13)-H(13C)	109.5	H(13B)-C(13)-H(13C)	109.5
O(1)-C(14)-H(14A)	109.5	O(1)-C(14)-H(14B)	109.5
O(1)-C(14)-H(14C)	109.5	H(14A)-C(14)-H(14B)	109.5
H(14A)-C(14)-H(14C)	109.5	H(14B)-C(14)-H(14C)	109.5
C(16)-C(15)-Bi(1A)	117.40(18)	C(16)-C(15)-Bi(1B)	105.8(2)
C(20)-C(15)-Bi(1A)	123.34(17)	C(20)-C(15)-Bi(1B)	134.9(2)
C(20)-C(15)-C(16)	119.3(2)	Bi(1B)-C(16)-H(16)	79.0
C(15)-C(16)-Bi(1B)	41.10(15)	C(15)-C(16)-H(16)	120.0
C(17)-C(16)-Bi(1B)	160.9(2)	C(17)-C(16)-C(15)	120.0(3)
C(17)-C(16)-H(16)	120.0	C(16)-C(17)-H(17)	119.6
C(18)-C(17)-C(16)	120.8(2)	C(18)-C(17)-H(17)	119.6
C(17)-C(18)-H(18)	120.1	C(17)-C(18)-C(19)	119.9(2)
C(19)-C(18)-H(18)	120.1	C(18)-C(19)-H(19)	120.2
C(18)-C(19)-C(20)	119.5(3)	C(20)-C(19)-H(19)	120.2
Bi(1B)-C(20)-H(20)	94.9	C(15)-C(20)-Bi(1B)	24.87(14)
C(15)-C(20)-C(19)	120.6(2)	C(15)-C(20)-H(20)	119.7
C(19)-C(20)-Bi(1B)	145.4(2)	C(19)-C(20)-H(20)	119.7
F(1)-C(21)-N(1)	115.7(2)	F(2)-C(21)-F(1)	105.9(2)

F(2)-C(21)-N(1)	113.4(2)	F(3)-C(21)-F(1)	105.72(19)
F(3)-C(21)-F(2)	105.31(19)	F(3)-C(21)-N(1)	110.0(2)
C(31)-Bi(2A)-C(42)	85.28(7)	C(45)-Bi(2A)-C(31)	93.41(8)
C(45)-Bi(2A)-C(42)	97.61(8)	C(42)-Bi(2B)-C(31)	67.8(2)
C(45)-Bi(2B)-C(31)	95.5(4)	C(45)-Bi(2B)-C(42)	104.5(4)
C(45)-Bi(2B)-C(46)	37.8(2)	C(45)-Bi(2B)-C(50)	19.83(19)
C(46)-Bi(2B)-C(31)	120.5(3)	C(46)-Bi(2B)-C(42)	137.8(4)
C(46)-Bi(2B)-C(50)	57.5(2)	C(50)-Bi(2B)-C(31)	82.5(2)
C(50)-Bi(2B)-C(42)	85.7(2)	O(6)-S(2)-N(2)	119.37(10)
O(6)-S(2)-C(36)	109.92(10)	O(6)-S(2)-C(37)	108.02(10)
N(2)-S(2)-C(36)	102.40(11)	N(2)-S(2)-C(37)	110.11(10)
C(36)-S(2)-C(37)	106.24(10)	C(40)-O(4)-C(44)	118.14(19)
C(33)-O(5)-C(43)	117.49(19)	C(51)-N(2)-S(2)	119.49(17)
C(32)-C(31)-Bi(2A)	123.28(16)	C(32)-C(31)-Bi(2B)	107.4(2)
C(32)-C(31)-C(36)	117.5(2)	C(36)-C(31)-Bi(2A)	119.17(16)
C(36)-C(31)-Bi(2B)	135.0(2)	C(31)-C(32)-H(32)	119.8
C(31)-C(32)-C(33)	120.5(2)	C(33)-C(32)-H(32)	119.8
O(5)-C(33)-C(32)	115.3(2)	O(5)-C(33)-C(34)	123.7(2)
C(34)-C(33)-C(32)	121.0(2)	C(33)-C(34)-H(34)	120.6
C(35)-C(34)-C(33)	118.8(2)	C(35)-C(34)-H(34)	120.6
C(34)-C(35)-H(35)	120.3	C(36)-C(35)-C(34)	119.4(2)
C(36)-C(35)-H(35)	120.3	C(31)-C(36)-S(2)	120.22(17)
C(35)-C(36)-S(2)	117.06(17)	C(35)-C(36)-C(31)	122.6(2)
C(38)-C(37)-S(2)	117.47(17)	C(38)-C(37)-C(42)	122.1(2)
C(42)-C(37)-S(2)	120.41(17)	C(37)-C(38)-H(38)	120.4
C(39)-C(38)-C(37)	119.1(2)	C(39)-C(38)-H(38)	120.4
C(38)-C(39)-H(39)	119.9	C(38)-C(39)-C(40)	120.1(2)
C(40)-C(39)-H(39)	119.9	O(4)-C(40)-C(39)	114.9(2)
O(4)-C(40)-C(41)	124.7(2)	C(39)-C(40)-C(41)	120.4(2)
C(40)-C(41)-H(41)	119.9	C(42)-C(41)-C(40)	120.2(2)
C(42)-C(41)-H(41)	119.9	C(37)-C(42)-Bi(2A)	118.91(15)
C(37)-C(42)-Bi(2B)	136.5(2)	C(37)-C(42)-C(41)	118.1(2)
C(41)-C(42)-Bi(2A)	122.90(16)	C(41)-C(42)-Bi(2B)	105.4(2)
O(5)-C(43)-H(43A)	109.5	O(5)-C(43)-H(43B)	109.5
O(5)-C(43)-H(43C)	109.5	H(43A)-C(43)-H(43B)	109.5
H(43A)-C(43)-H(43C)	109.5	H(43B)-C(43)-H(43C)	109.5

O(4)-C(44)-H(44A)	109.5	O(4)-C(44)-H(44B)	109.5
O(4)-C(44)-H(44C)	109.5	H(44A)-C(44)-H(44B)	109.5
H(44A)-C(44)-H(44C)	109.5	H(44B)-C(44)-H(44C)	109.5
C(46)-C(45)-Bi(2A)	115.71(17)	C(46)-C(45)-Bi(2B)	101.8(4)
C(50)-C(45)-Bi(2A)	125.04(16)	C(50)-C(45)-Bi(2B)	139.0(4)
C(50)-C(45)-C(46)	119.1(2)	Bi(2B)-C(46)-H(46)	79.5
C(45)-C(46)-Bi(2B)	40.5(2)	C(45)-C(46)-H(46)	119.9
C(47)-C(46)-Bi(2B)	160.4(3)	C(47)-C(46)-C(45)	120.3(2)
C(47)-C(46)-H(46)	119.9	C(46)-C(47)-H(47)	120.1
C(48)-C(47)-C(46)	119.9(2)	C(48)-C(47)-H(47)	120.1
C(47)-C(48)-H(48)	119.9	C(47)-C(48)-C(49)	120.2(2)
C(49)-C(48)-H(48)	119.9	C(48)-C(49)-H(49)	120.1
C(50)-C(49)-C(48)	119.9(2)	C(50)-C(49)-H(49)	120.1
Bi(2B)-C(50)-H(50)	98.6	C(45)-C(50)-Bi(2B)	21.2(2)
C(45)-C(50)-H(50)	119.7	C(49)-C(50)-Bi(2B)	141.7(3)
C(49)-C(50)-C(45)	120.7(2)	C(49)-C(50)-H(50)	119.7
F(4)-C(51)-F(5)	106.0(2)	F(4)-C(51)-F(6)	105.54(19)
F(4)-C(51)-N(2)	116.3(2)	F(5)-C(51)-N(2)	112.8(2)
F(6)-C(51)-F(5)	105.0(2)	F(6)-C(51)-N(2)	110.4(2)

8.13 Single crystal structure analysis of **94**

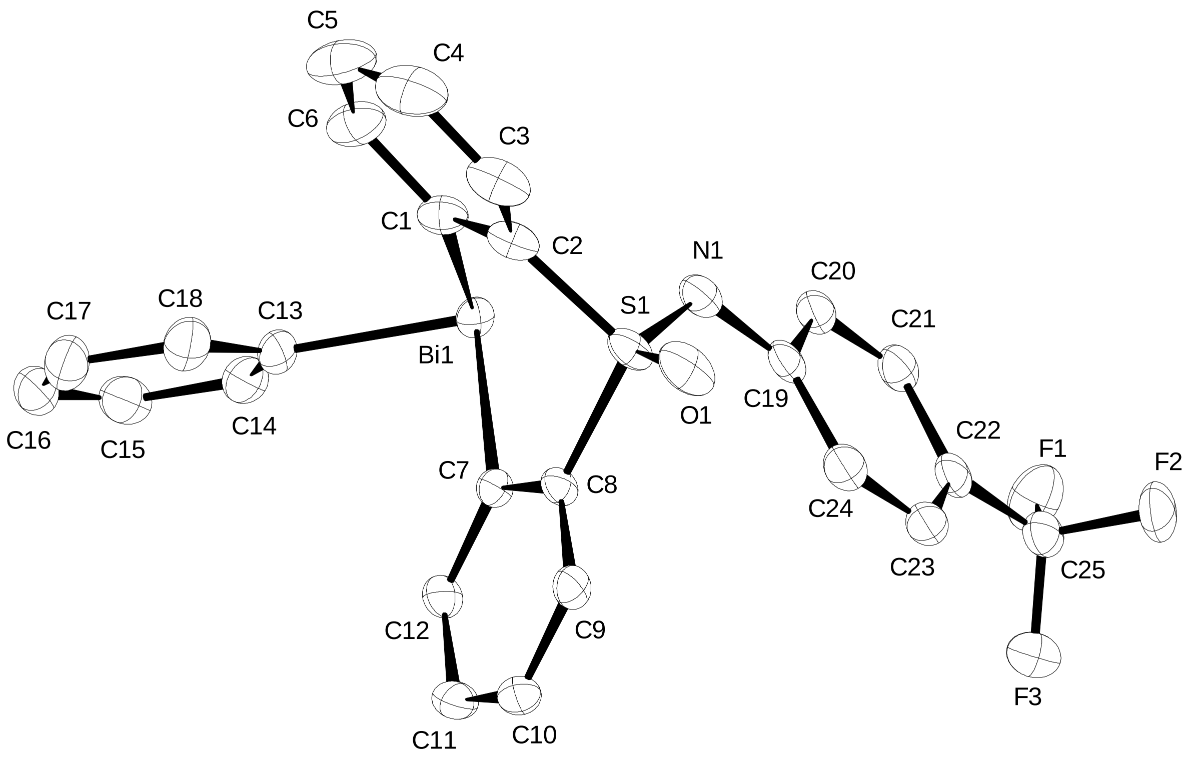

Figure S103. The molecular structure of complex **94**. H atoms have been removed for clarity.

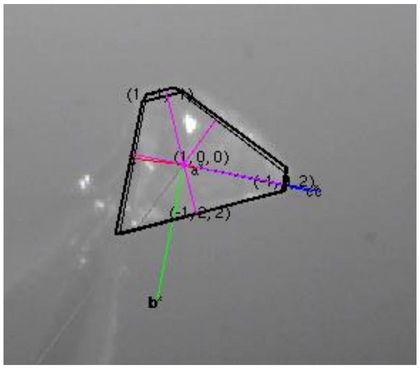

	h	k	l	distance (mm)
1.	-1	0	0	0.04
2.	1	0	0	0.04
3.	1	-1	-1	0.13
4.	-1	-1	1	0.10
5.	1	0	-2	0.09
6.	-1	2	2	0.09
7.	-1	0	2	0.18

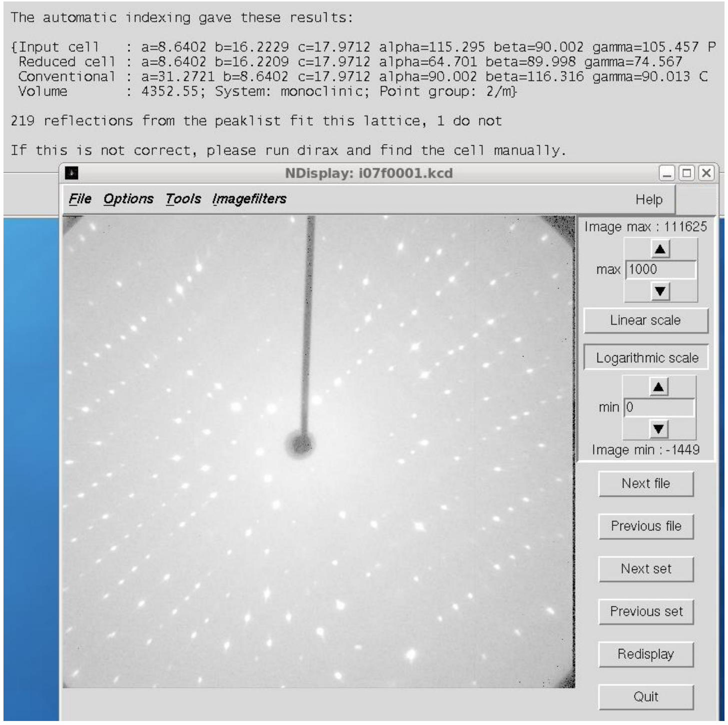

Figure S104. Crystal faces and unit cell determination of **94**.

INTENSITY STATISTICS FOR DATASET

Resolution	#Data	#Theory	%Complete	Redundancy	Mean I	Mean I/s	Rmerge	Rsigma
Inf - 2.52	163	178	91.6	7.02	158.82	100.06	0.0263	0.0103
2.52 - 1.66	389	390	99.7	8.78	125.35	97.26	0.0207	0.0079
1.66 - 1.32	545	545	100.0	8.86	89.36	90.45	0.0185	0.0082
1.32 - 1.15	537	537	100.0	8.62	60.17	78.15	0.0199	0.0091
1.15 - 1.04	568	568	100.0	8.08	53.37	71.40	0.0188	0.0100
1.04 - 0.96	563	563	100.0	7.55	41.71	60.22	0.0209	0.0114
0.96 - 0.91	499	499	100.0	7.18	33.67	54.18	0.0222	0.0129
0.91 - 0.86	575	575	100.0	6.79	28.64	48.38	0.0242	0.0148
0.86 - 0.82	587	587	100.0	6.45	23.63	42.12	0.0271	0.0168
0.82 - 0.79	508	508	100.0	6.23	20.82	37.53	0.0299	0.0190
0.79 - 0.76	599	599	100.0	5.83	19.26	34.57	0.0324	0.0209
0.76 - 0.74	446	446	100.0	5.63	18.26	32.53	0.0349	0.0230
0.74 - 0.71	772	772	100.0	5.40	13.86	26.93	0.0425	0.0280
0.71 - 0.70	304	304	100.0	5.19	13.28	24.94	0.0417	0.0298
0.70 - 0.68	619	619	100.0	4.91	12.07	22.50	0.0504	0.0344
0.68 - 0.66	711	711	100.0	4.77	10.78	20.90	0.0558	0.0384
0.66 - 0.65	380	380	100.0	4.57	9.39	18.51	0.0632	0.0435
0.65 - 0.63	858	858	100.0	4.37	8.33	16.08	0.0690	0.0499
0.63 - 0.62	450	450	100.0	3.84	7.60	14.20	0.0721	0.0593
0.62 - 0.61	511	511	100.0	3.52	7.23	12.88	0.0745	0.0659
0.61 - 0.60	254	260	97.7	3.40	6.13	11.24	0.0927	0.0770
0.70 - 0.60	3783	3789	99.8	4.31	9.13	17.30	0.0623	0.0472
Inf - 0.60	10838	10860	99.8	6.03	31.01	41.29	0.0252	0.0157

Complete .cif-data of the compound are available under the CCDC number **CCDC-2125775.**

Table S51. Crystal data and structure refinement of **94**.

Identification code	13472sadabs	
Empirical formula	$C_{25} H_{17} Bi F_3 N O S$	
Color	colourless	
Formula weight	645.43 g·mol^{-1}	
Temperature	100(2) K	
Wavelength	0.71073 Å	
Crystal system	Monoclinic	
Space group	*C*2/c, (no. 15)	
Unit cell dimensions	a = 31.2940(5) Å	α= 90°.
	b = 8.648(3) Å	β= 116.297(7)°.
	c = 17.9768(11) Å	γ = 90°.
Volume	4361.4(13) Å^3	
Z	8	
Density (calculated)	1.966 Mg·m^{-3}	
Absorption coefficient	8.224 mm^{-1}	
F(000)	2464 e	
Crystal size	0.27 x 0.26 x 0.08 mm^3	
θ range for data collection	2.884 to 27.499°.	
Index ranges	-40 ≤ h ≤ 40, -11≤ k ≤ 11, -23 ≤ l ≤ 23	
Reflections collected	38252	
Independent reflections	4999 [R_{int} = 0.0214]	
Reflections with I>2σ(I)	4777	
Completeness to θ = 25.242°	99.7 %	
Absorption correction	Gaussian	
Max. and min. transmission	0.9494 and 0.5858	
Refinement method	Full-matrix least-squares on F^2	
Data / restraints / parameters	4999 / 0 / 289	
Goodness-of-fit on F^2	1.086	
Final R indices [I>2σ(I)]	R_1 = 0.0166	wR^2 = 0.0419
R indices (all data)	R_1 = 0.0178	wR^2 = 0.0425
Extinction coefficient	n/a	
Largest diff. peak and hole	1.584 and -1.509 e·Å^{-3}	

Table S52. Bond lengths [Å] and angles [°] of **94**.

Bi(1)-C(1)	2.252(2)	Bi(1)-C(7)	2.264(2)
Bi(1)-C(13)	2.261(2)	S(1)-O(1)	1.4480(18)
S(1)-N(1)	1.545(2)	S(1)-C(2)	1.778(3)
S(1)-C(8)	1.784(2)	F(1)-C(25)	1.335(3)
F(2)-C(25)	1.349(3)	F(3)-C(25)	1.340(3)
N(1)-C(19)	1.403(3)	C(1)-C(2)	1.396(4)
C(1)-C(6)	1.396(4)	C(2)-C(3)	1.386(4)
C(3)-H(3)	0.9500	C(3)-C(4)	1.394(5)
C(4)-H(4)	0.9500	C(4)-C(5)	1.391(5)
C(5)-H(5)	0.9500	C(5)-C(6)	1.388(4)
C(6)-H(6)	0.9500	C(7)-C(8)	1.391(3)
C(7)-C(12)	1.390(3)	C(8)-C(9)	1.390(3)
C(9)-H(9)	0.9500	C(9)-C(10)	1.393(3)
C(10)-H(10)	0.9500	C(10)-C(11)	1.388(4)
C(11)-H(11)	0.9500	C(11)-C(12)	1.406(3)
C(12)-H(12)	0.9500	C(13)-C(14)	1.393(4)
C(13)-C(18)	1.390(4)	C(14)-H(14)	0.9500
C(14)-C(15)	1.394(4)	C(15)-H(15)	0.9500
C(15)-C(16)	1.380(5)	C(16)-H(16)	0.9500
C(16)-C(17)	1.389(6)	C(17)-H(17)	0.9500
C(17)-C(18)	1.393(4)	C(18)-H(18)	0.9500
C(19)-C(20)	1.398(3)	C(19)-C(24)	1.402(3)
C(20)-H(20)	0.9500	C(20)-C(21)	1.379(4)
C(21)-H(21)	0.9500	C(21)-C(22)	1.394(3)
C(22)-C(23)	1.384(4)	C(22)-C(25)	1.490(4)
C(23)-H(23)	0.9500	C(23)-C(24)	1.386(4)
C(24)-H(24)	0.9500		
C(1)-Bi(1)-C(7)	88.64(8)	C(1)-Bi(1)-C(13)	94.96(10)
C(13)-Bi(1)-C(7)	92.95(8)	O(1)-S(1)-N(1)	121.80(11)
O(1)-S(1)-C(2)	110.36(13)	O(1)-S(1)-C(8)	107.87(11)
N(1)-S(1)-C(2)	99.85(12)	N(1)-S(1)-C(8)	109.80(11)
C(2)-S(1)-C(8)	106.00(11)	C(19)-N(1)-S(1)	123.37(18)
C(2)-C(1)-Bi(1)	119.56(18)	C(6)-C(1)-Bi(1)	122.83(19)
C(6)-C(1)-C(2)	117.6(2)	C(1)-C(2)-S(1)	118.97(19)
C(3)-C(2)-S(1)	118.1(2)	C(3)-C(2)-C(1)	122.9(3)

C(2)-C(3)-H(3)	120.9	C(2)-C(3)-C(4)	118.3(3)
C(4)-C(3)-H(3)	120.9	C(3)-C(4)-H(4)	120.0
C(5)-C(4)-C(3)	120.0(3)	C(5)-C(4)-H(4)	120.0
C(4)-C(5)-H(5)	119.6	C(6)-C(5)-C(4)	120.7(3)
C(6)-C(5)-H(5)	119.6	C(1)-C(6)-H(6)	119.8
C(5)-C(6)-C(1)	120.4(3)	C(5)-C(6)-H(6)	119.8
C(8)-C(7)-Bi(1)	120.28(16)	C(12)-C(7)-Bi(1)	122.26(16)
C(12)-C(7)-C(8)	117.4(2)	C(7)-C(8)-S(1)	118.04(17)
C(9)-C(8)-S(1)	118.70(17)	C(9)-C(8)-C(7)	123.3(2)
C(8)-C(9)-H(9)	120.7	C(8)-C(9)-C(10)	118.5(2)
C(10)-C(9)-H(9)	120.7	C(9)-C(10)-H(10)	120.2
C(11)-C(10)-C(9)	119.6(2)	C(11)-C(10)-H(10)	120.2
C(10)-C(11)-H(11)	119.6	C(10)-C(11)-C(12)	120.8(2)
C(12)-C(11)-H(11)	119.6	C(7)-C(12)-C(11)	120.4(2)
C(7)-C(12)-H(12)	119.8	C(11)-C(12)-H(12)	119.8
C(14)-C(13)-Bi(1)	117.22(19)	C(18)-C(13)-Bi(1)	124.1(2)
C(18)-C(13)-C(14)	118.7(2)	C(13)-C(14)-H(14)	119.3
C(13)-C(14)-C(15)	121.3(3)	C(15)-C(14)-H(14)	119.3
C(14)-C(15)-H(15)	120.3	C(16)-C(15)-C(14)	119.4(3)
C(16)-C(15)-H(15)	120.3	C(15)-C(16)-H(16)	120.0
C(15)-C(16)-C(17)	120.0(3)	C(17)-C(16)-H(16)	120.0
C(16)-C(17)-H(17)	119.8	C(16)-C(17)-C(18)	120.4(3)
C(18)-C(17)-H(17)	119.8	C(13)-C(18)-C(17)	120.2(3)
C(13)-C(18)-H(18)	119.9	C(17)-C(18)-H(18)	119.9
C(20)-C(19)-N(1)	114.9(2)	C(20)-C(19)-C(24)	118.4(2)
C(24)-C(19)-N(1)	126.7(2)	C(19)-C(20)-H(20)	119.5
C(21)-C(20)-C(19)	121.0(2)	C(21)-C(20)-H(20)	119.5
C(20)-C(21)-H(21)	120.0	C(20)-C(21)-C(22)	120.1(2)
C(22)-C(21)-H(21)	120.0	C(21)-C(22)-C(25)	119.7(2)
C(23)-C(22)-C(21)	119.4(2)	C(23)-C(22)-C(25)	120.8(2)
C(22)-C(23)-H(23)	119.6	C(22)-C(23)-C(24)	120.7(2)
C(24)-C(23)-H(23)	119.6	C(19)-C(24)-H(24)	119.9
C(23)-C(24)-C(19)	120.3(2)	C(23)-C(24)-H(24)	119.9
F(1)-C(25)-F(2)	106.1(2)	F(1)-C(25)-F(3)	106.6(2)
F(1)-C(25)-C(22)	113.3(2)	F(2)-C(25)-C(22)	112.0(2)
F(3)-C(25)-F(2)	105.1(2)	F(3)-C(25)-C(22)	113.1(2)

8.14 Single crystal structure analysis of **95**

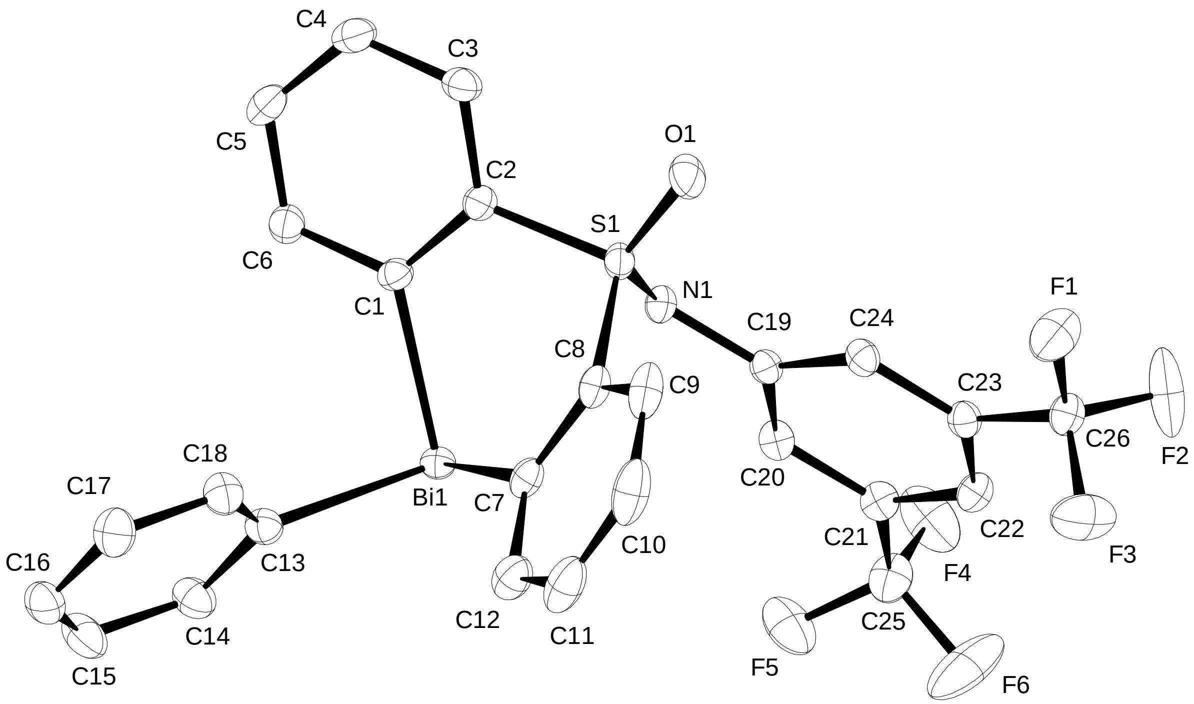

Figure S105. The molecular structure of complex **95**. H atoms have been removed for clarity.

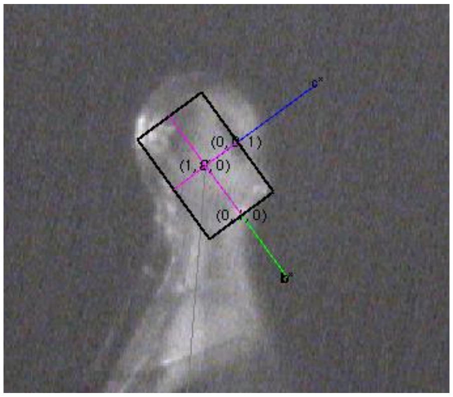

	h	k	l	distance (mm)
1.	1	0	0	0.04
2.	-1	0	0	0.04
3.	0	0	-1	0.07
4.	0	0	1	0.07
5.	0	1	0	0.11
6.	0	-1	0	0.11

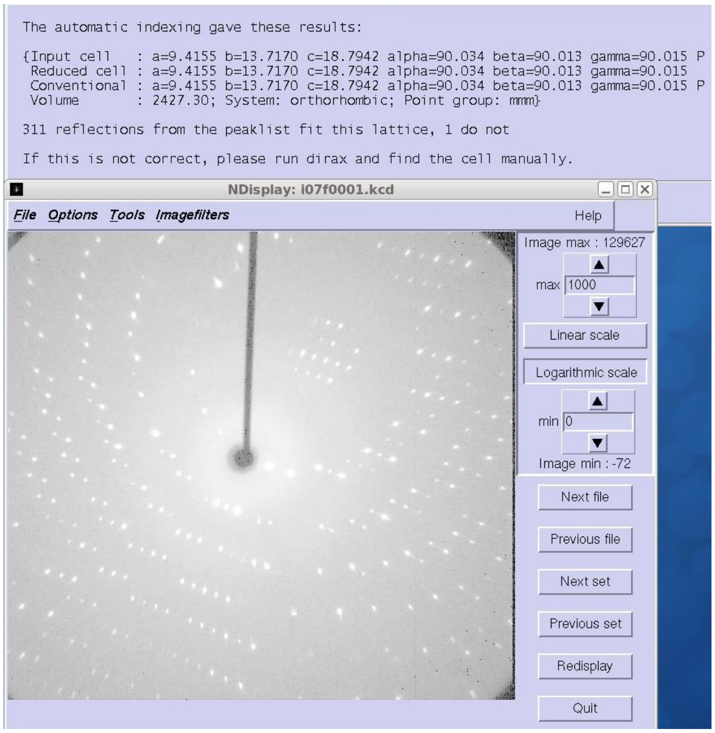

Figure S106. Crystal faces and unit cell determination of **95**.

INTENSITY STATISTICS FOR DATASET

Resolution	#Data	#Theory	%Complete	Redundancy	Mean I	Mean I/s	Rmerge	Rsigma
Inf - 2.53	104	120	86.7	7.78	199.65	71.59	0.0785	0.0186
2.53 - 1.66	240	242	99.2	11.58	180.72	84.39	0.0630	0.0128
1.66 - 1.31	341	341	100.0	12.37	131.25	83.03	0.0537	0.0114
1.31 - 1.14	331	331	100.0	12.17	92.42	77.76	0.0610	0.0118
1.14 - 1.03	347	347	100.0	11.85	80.84	73.03	0.0562	0.0123
1.03 - 0.95	355	355	100.0	11.36	61.83	68.03	0.0548	0.0131
0.95 - 0.89	357	357	100.0	10.53	48.57	58.99	0.0550	0.0148
0.89 - 0.85	291	291	100.0	10.27	49.10	57.37	0.0559	0.0152
0.85 - 0.81	352	352	100.0	9.82	34.05	48.84	0.0612	0.0169
0.81 - 0.77	434	434	100.0	9.27	34.26	46.69	0.0611	0.0183
0.77 - 0.75	247	247	100.0	8.88	27.10	40.97	0.0626	0.0205
0.75 - 0.72	417	417	100.0	8.59	26.29	39.44	0.0673	0.0215
0.72 - 0.70	332	332	100.0	8.26	21.98	34.61	0.0731	0.0241
0.70 - 0.68	355	355	100.0	7.79	19.45	31.11	0.0799	0.0273
0.68 - 0.66	416	416	100.0	7.40	17.04	27.83	0.0827	0.0311
0.66 - 0.65	219	219	100.0	7.33	15.58	26.47	0.0873	0.0333
0.65 - 0.63	487	487	100.0	6.92	14.35	23.88	0.0963	0.0363
0.63 - 0.62	276	276	100.0	6.43	12.46	20.78	0.1031	0.0419
0.62 - 0.61	295	295	100.0	6.40	11.04	19.26	0.1085	0.0460
0.61 - 0.60	296	296	100.0	6.08	10.98	18.71	0.1143	0.0472
0.60 - 0.59	275	308	89.3	4.71	8.43	14.08	0.1292	0.0696
0.69 - 0.59	2448	2481	98.7	6.61	13.49	22.60	0.0955	0.0391
Inf - 0.59	6767	6818	99.3	8.89	45.75	45.13	0.0624	0.0172

Complete .cif-data of the compound are available under the CCDC number **CCDC-2125789.**

Table S53. Crystal data and structure refinement of **95**.

Identification code	13471sadabs	
Empirical formula	$C_{26} H_{16} Bi F_6 N O S$	
Color	colourless	
Formula weight	713.44 g·mol^{-1}	
Temperature	100(2) K	
Wavelength	0.71073 Å	
Crystal system	Orthorhombic	
Space group	$P2_12_12_1$, (no. 19)	
Unit cell dimensions	a = 9.4093(5) Å	α= 90°.
	b = 13.7146(14) Å	β= 90°.
	c = 18.781(3) Å	γ = 90°.
Volume	2423.6(5) Å^3	
Z	4	
Density (calculated)	1.955 Mg·m^{-3}	
Absorption coefficient	7.427 mm^{-1}	
F(000)	1360 e	
Crystal size	0.22 x 0.14 x 0.08 mm^3	
θ range for data collection	2.629 to 27.496°.	
Index ranges	-12 ≤ h ≤ 12, -17≤ k ≤ 17, -24 ≤ l ≤ 24	
Reflections collected	34337	
Independent reflections	5556 [R_{int} = 0.0327]	
Reflections with I>2σ(I)	5518	
Completeness to θ = 25.242°	99.4 %	
Absorption correction	Gaussian	
Max. and min. transmission	0.98412 and 0.96052	
Refinement method	Full-matrix least-squares on F^2	
Data / restraints / parameters	5556 / 0 / 325	
Goodness-of-fit on F^2	1.095	
Final R indices [I>2σ(I)]	R_1 = 0.0121	wR^2 = 0.0300
R indices (all data)	R_1 = 0.0123	wR^2 = 0.0301
Absolute structure parameter	-0.0205(16)	
Extinction coefficient	n/a	
Largest diff. peak and hole	0.499 and -0.886 e·Å^{-3}	

Table S54. Bond lengths [Å] and angles [°] of **95**.

Bi(1)-C(1)	2.269(2)	Bi(1)-C(7)	2.262(3)
Bi(1)-C(13)	2.255(3)	S(1)-O(1)	1.454(2)
S(1)-N(1)	1.539(2)	S(1)-C(2)	1.766(3)
S(1)-C(8)	1.787(3)	F(1)-C(26)	1.328(3)
F(2)-C(26)	1.323(4)	F(3)-C(26)	1.341(4)
F(4)-C(25)	1.335(4)	F(5)-C(25)	1.332(4)
F(6)-C(25)	1.335(4)	N(1)-C(19)	1.403(4)
C(1)-C(2)	1.388(4)	C(1)-C(6)	1.391(4)
C(2)-C(3)	1.392(4)	C(3)-H(3)	0.9500
C(3)-C(4)	1.388(5)	C(4)-H(4)	0.9500
C(4)-C(5)	1.383(5)	C(5)-H(5)	0.9500
C(5)-C(6)	1.402(4)	C(6)-H(6)	0.9500
C(7)-C(8)	1.400(4)	C(7)-C(12)	1.396(4)
C(8)-C(9)	1.391(4)	C(9)-H(9)	0.9500
C(9)-C(10)	1.392(5)	C(10)-H(10)	0.9500
C(10)-C(11)	1.385(6)	C(11)-H(11)	0.9500
C(11)-C(12)	1.393(5)	C(12)-H(12)	0.9500
C(13)-C(14)	1.390(4)	C(13)-C(18)	1.393(4)
C(14)-H(14)	0.9500	C(14)-C(15)	1.394(4)
C(15)-H(15)	0.9500	C(15)-C(16)	1.386(5)
C(16)-H(16)	0.9500	C(16)-C(17)	1.391(5)
C(17)-H(17)	0.9500	C(17)-C(18)	1.391(4)
C(18)-H(18)	0.9500	C(19)-C(20)	1.406(4)
C(19)-C(24)	1.407(4)	C(20)-H(20)	0.9500
C(20)-C(21)	1.384(4)	C(21)-C(22)	1.386(5)
C(21)-C(25)	1.498(4)	C(22)-H(22)	0.9500
C(22)-C(23)	1.393(4)	C(23)-C(24)	1.383(4)
C(23)-C(26)	1.498(4)	C(24)-H(24)	0.9500
C(7)-Bi(1)-C(1)	87.45(10)	C(13)-Bi(1)-C(1)	91.86(10)
C(13)-Bi(1)-C(7)	93.07(10)	O(1)-S(1)-N(1)	121.35(14)
O(1)-S(1)-C(2)	110.09(13)	O(1)-S(1)-C(8)	108.03(15)
N(1)-S(1)-C(2)	101.68(14)	N(1)-S(1)-C(8)	108.37(13)
C(2)-S(1)-C(8)	106.34(13)	C(19)-N(1)-S(1)	120.3(2)
C(2)-C(1)-Bi(1)	120.4(2)	C(2)-C(1)-C(6)	117.3(2)

C(6)-C(1)-Bi(1)	122.2(2)	C(1)-C(2)-S(1)	119.7(2)
C(1)-C(2)-C(3)	123.4(3)	C(3)-C(2)-S(1)	116.9(2)
C(2)-C(3)-H(3)	120.9	C(4)-C(3)-C(2)	118.2(3)
C(4)-C(3)-H(3)	120.9	C(3)-C(4)-H(4)	120.1
C(5)-C(4)-C(3)	119.9(3)	C(5)-C(4)-H(4)	120.1
C(4)-C(5)-H(5)	119.6	C(4)-C(5)-C(6)	120.9(3)
C(6)-C(5)-H(5)	119.6	C(1)-C(6)-C(5)	120.3(3)
C(1)-C(6)-H(6)	119.9	C(5)-C(6)-H(6)	119.9
C(8)-C(7)-Bi(1)	121.4(2)	C(12)-C(7)-Bi(1)	121.4(2)
C(12)-C(7)-C(8)	117.2(3)	C(7)-C(8)-S(1)	118.0(2)
C(9)-C(8)-S(1)	119.1(2)	C(9)-C(8)-C(7)	122.8(3)
C(8)-C(9)-H(9)	120.8	C(8)-C(9)-C(10)	118.4(3)
C(10)-C(9)-H(9)	120.8	C(9)-C(10)-H(10)	119.9
C(11)-C(10)-C(9)	120.3(3)	C(11)-C(10)-H(10)	119.9
C(10)-C(11)-H(11)	119.8	C(10)-C(11)-C(12)	120.5(3)
C(12)-C(11)-H(11)	119.8	C(7)-C(12)-H(12)	119.6
C(11)-C(12)-C(7)	120.9(3)	C(11)-C(12)-H(12)	119.6
C(14)-C(13)-Bi(1)	119.2(2)	C(14)-C(13)-C(18)	119.0(3)
C(18)-C(13)-Bi(1)	121.8(2)	C(13)-C(14)-H(14)	119.6
C(13)-C(14)-C(15)	120.8(3)	C(15)-C(14)-H(14)	119.6
C(14)-C(15)-H(15)	120.1	C(16)-C(15)-C(14)	119.8(3)
C(16)-C(15)-H(15)	120.1	C(15)-C(16)-H(16)	120.0
C(15)-C(16)-C(17)	120.0(3)	C(17)-C(16)-H(16)	120.0
C(16)-C(17)-H(17)	120.0	C(16)-C(17)-C(18)	119.9(3)
C(18)-C(17)-H(17)	120.0	C(13)-C(18)-H(18)	119.7
C(17)-C(18)-C(13)	120.5(3)	C(17)-C(18)-H(18)	119.7
N(1)-C(19)-C(20)	116.6(3)	N(1)-C(19)-C(24)	125.1(2)
C(20)-C(19)-C(24)	118.3(3)	C(19)-C(20)-H(20)	119.9
C(21)-C(20)-C(19)	120.3(3)	C(21)-C(20)-H(20)	119.9
C(20)-C(21)-C(22)	121.5(3)	C(20)-C(21)-C(25)	119.8(3)
C(22)-C(21)-C(25)	118.6(3)	C(21)-C(22)-H(22)	120.9
C(21)-C(22)-C(23)	118.3(3)	C(23)-C(22)-H(22)	120.9
C(22)-C(23)-C(26)	117.1(3)	C(24)-C(23)-C(22)	121.4(3)
C(24)-C(23)-C(26)	121.4(3)	C(19)-C(24)-H(24)	119.9
C(23)-C(24)-C(19)	120.3(3)	C(23)-C(24)-H(24)	119.9
F(4)-C(25)-F(6)	106.1(3)	F(4)-C(25)-C(21)	112.1(3)

F(5)-C(25)-F(4)	106.1(3)	F(5)-C(25)-F(6)	106.7(3)
F(5)-C(25)-C(21)	112.9(3)	F(6)-C(25)-C(21)	112.5(3)
F(1)-C(26)-F(3)	105.7(2)	F(1)-C(26)-C(23)	113.5(3)
F(2)-C(26)-F(1)	107.6(3)	F(2)-C(26)-F(3)	105.7(3)
F(2)-C(26)-C(23)	112.4(2)	F(3)-C(26)-C(23)	111.5(2)

8.15 Single crystal structure analysis of **96a**

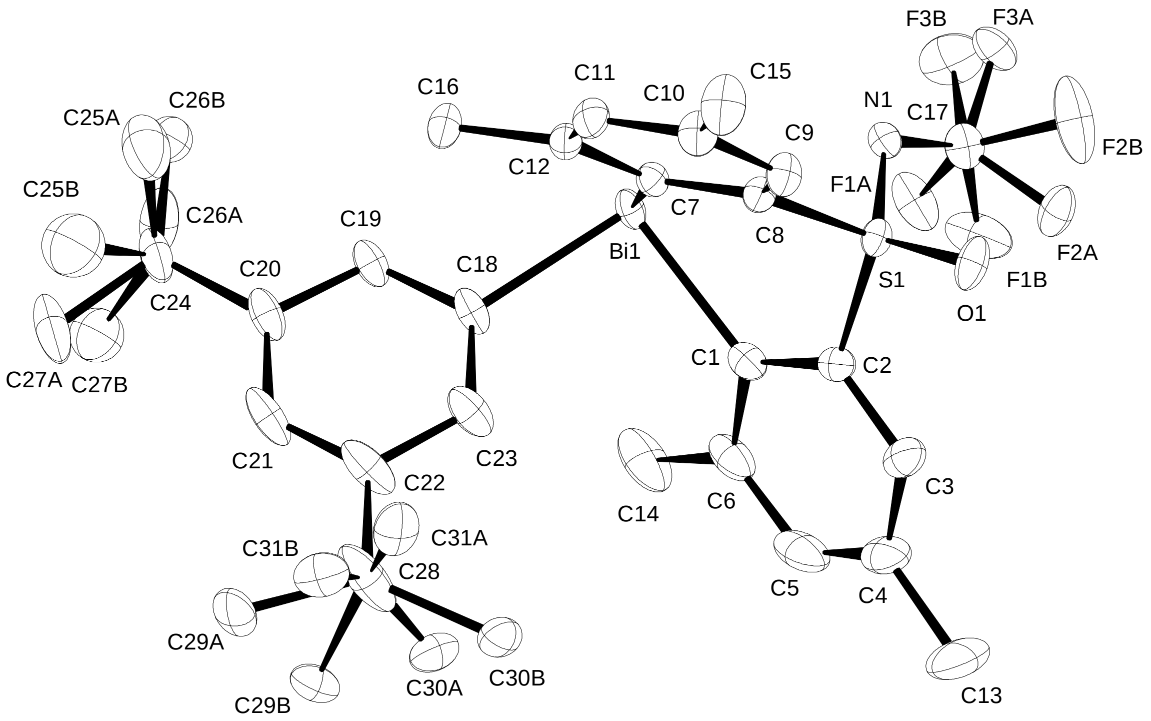

Figure S107. The molecular structure of complex **96a**. H atoms have been removed for clarity.

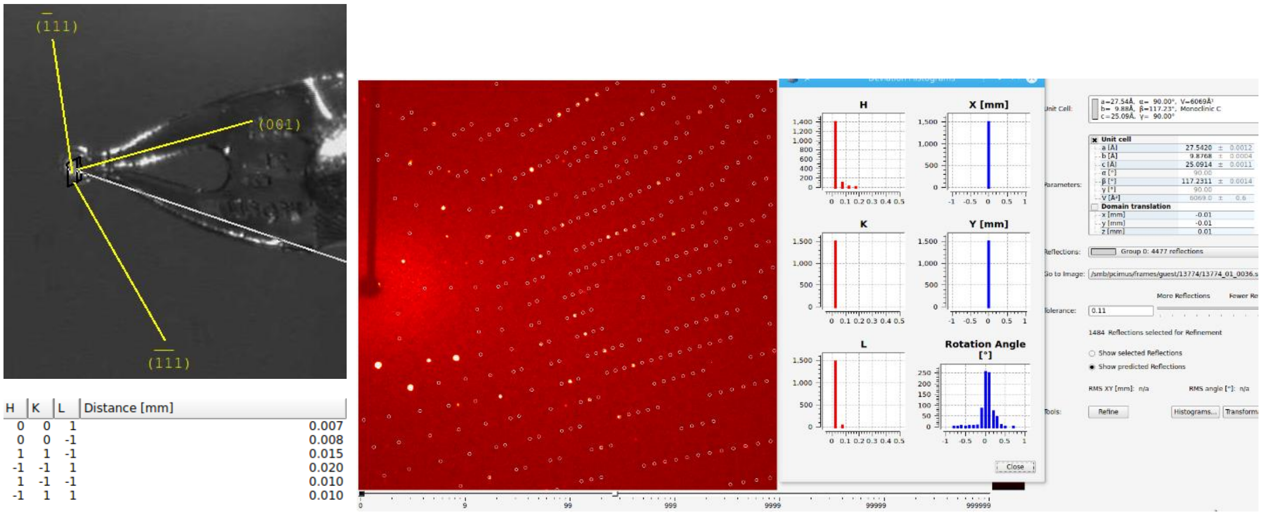

Figure S108. Crystal faces and unit cell determination of **96a**.

INTENSITY STATISTICS FOR DATASET

Resolution	#Data	#Theory	%Complete	Redundancy	Mean I	Mean I/s	Rmerge	Rsigma
Inf - 2.63	213	213	100.0	15.00	136.39	90.07	0.0344	0.0088
2.63 - 1.72	497	497	100.0	16.65	89.79	85.84	0.0263	0.0087
1.72 - 1.35	709	709	100.0	16.80	63.85	78.17	0.0238	0.0093
1.35 - 1.17	748	748	100.0	16.53	44.64	66.60	0.0272	0.0105
1.17 - 1.06	713	713	100.0	14.21	37.80	57.06	0.0317	0.0124
1.06 - 0.98	741	741	100.0	10.46	31.89	42.18	0.0378	0.0163
0.98 - 0.92	734	734	100.0	8.54	24.47	33.47	0.0468	0.0217
0.92 - 0.88	626	626	100.0	7.41	21.55	27.93	0.0532	0.0266
0.88 - 0.84	733	733	100.0	6.92	18.97	23.53	0.0609	0.0308
0.84 - 0.80	883	883	100.0	6.57	16.78	21.16	0.0679	0.0356
0.80 - 0.78	509	509	100.0	6.37	14.61	18.47	0.0732	0.0408
0.78 - 0.75	865	865	100.0	6.12	12.75	16.34	0.0826	0.0476
0.75 - 0.73	652	652	100.0	5.81	12.61	15.39	0.0877	0.0515
0.73 - 0.71	723	723	100.0	5.67	11.07	13.54	0.0995	0.0597
0.71 - 0.69	832	832	100.0	5.49	9.56	11.93	0.1094	0.0688
0.69 - 0.68	462	462	100.0	5.24	9.13	11.14	0.1143	0.0756
0.68 - 0.66	959	959	100.0	5.24	7.68	9.79	0.1302	0.0879
0.66 - 0.65	549	553	99.3	4.86	7.33	8.66	0.1373	0.0980
0.65 - 0.63	1167	1167	100.0	4.79	6.61	7.88	0.1541	0.1108
0.63 - 0.62	835	960	87.0	3.62	6.20	6.79	0.1635	0.1325
0.72 - 0.62	5166	5295	97.6	4.87	7.83	9.47	0.1296	0.0913
Inf - 0.62	14150	14279	99.1	8.10	23.62	28.58	0.0397	0.0270

Terminal CF_3 group shows a rotational disorder over two positions with 50:50 occupancy. Furthermore, two tert.-Butyl groups are disordered by rotation over two positions (50:50 and 75:25 occupancy). The minor component was treated using isotropic atomic displacement parameters. Complete .cif-data of the compound are available under the CCDC number **CCDC-2125786.**

Table S55. Crystal data and structure refinement of **96a**.

Identification code	13774sadabs	
Empirical formula	$C_{31} H_{37} Bi F_3 N O S$	
Color	colourless	
Formula weight	737.65 g·mol^{-1}	
Temperature	100(2) K	
Wavelength	0.71073 Å	
Crystal system	Monoclinic	
Space group	*C*2/c, (no. 15)	
Unit cell dimensions	a = 27.5420(12) Å	α= 90°.
	b = 9.8768(4) Å	β= 117.2310(10)°.
	c = 25.0914(11) Å	γ = 90°.
Volume	6069.1(5) Å^3	
Z	8	
Density (calculated)	1.615 Mg·m^{-3}	
Absorption coefficient	5.921 mm^{-1}	
F(000)	2912 e	
Crystal size	0.053 x 0.025 x 0.017 mm^3	
θ range for data collection	1.663 to 35.291°.	
Index ranges	$-44 \le h \le 44$, $-15 \le k \le 15$, $-40 \le l \le 40$	
Reflections collected	111776	
Independent reflections	13513 [R_{int} = 0.0399]	
Reflections with I>2σ(I)	11338	
Completeness to θ = 25.242°	99.9 %	
Absorption correction	Gaussian	
Max. and min. transmission	0.92368 and 0.72308	
Refinement method	Full-matrix least-squares on F^2	
Data / restraints / parameters	13513 / 0 / 425	
Goodness-of-fit on F^2	1.025	
Final R indices [I>2σ(I)]	R_1 = 0.0213	wR^2 = 0.0404
R indices (all data)	R_1 = 0.0317	wR^2 = 0.0425
Extinction coefficient	n/a	
Largest diff. peak and hole	0.885 and -0.727 e·Å^{-3}	

Table S56. Bond lengths [Å] and angles [°] of **96a**.

Bi(1)-C(1)	2.2687(16)	Bi(1)-C(7)	2.2810(14)
Bi(1)-C(18)	2.2477(14)	S(1)-O(1)	1.4416(11)
S(1)-N(1)	1.5437(13)	S(1)-C(2)	1.7786(15)
S(1)-C(8)	1.7586(15)	F(1A)-C(17)	1.399(2)
F(1B)-C(17)	1.291(3)	F(2A)-C(17)	1.352(2)
F(2B)-C(17)	1.409(3)	F(3A)-C(17)	1.263(3)
F(3B)-C(17)	1.374(3)	N(1)-C(17)	1.376(2)
C(1)-C(2)	1.396(2)	C(1)-C(6)	1.403(2)
C(2)-C(3)	1.379(2)	C(3)-H(3)	0.9500
C(3)-C(4)	1.392(3)	C(4)-C(5)	1.388(3)
C(4)-C(13)	1.504(3)	C(5)-H(5)	0.9500
C(5)-C(6)	1.394(3)	C(6)-C(14)	1.509(3)
C(7)-C(8)	1.3967(19)	C(7)-C(12)	1.407(2)
C(8)-C(9)	1.387(2)	C(9)-H(9)	0.9500
C(9)-C(10)	1.391(2)	C(10)-C(11)	1.388(2)
C(10)-C(15)	1.504(2)	C(11)-H(11)	0.9500
C(11)-C(12)	1.399(2)	C(12)-C(16)	1.507(2)
C(13)-H(13A)	0.9800	C(13)-H(13B)	0.9800
C(13)-H(13C)	0.9800	C(14)-H(14A)	0.9800
C(14)-H(14B)	0.9800	C(14)-H(14C)	0.9800
C(15)-H(15A)	0.9800	C(15)-H(15B)	0.9800
C(15)-H(15C)	0.9800	C(16)-H(16A)	0.9800
C(16)-H(16B)	0.9800	C(16)-H(16C)	0.9800
C(18)-C(19)	1.390(2)	C(18)-C(23)	1.383(2)
C(19)-H(19)	0.9500	C(19)-C(20)	1.398(2)
C(20)-C(21)	1.394(3)	C(20)-C(24)	1.532(3)
C(21)-H(21)	0.9500	C(21)-C(22)	1.392(3)
C(22)-C(23)	1.400(2)	C(22)-C(28)	1.534(3)
C(23)-H(23)	0.9500	C(24)-C(25A)	1.548(3)
C(24)-C(25B)	1.505(11)	C(24)-C(26A)	1.502(3)
C(24)-C(26B)	1.543(7)	C(24)-C(27A)	1.521(3)
C(24)-C(27B)	1.631(9)	C(25A)-H(25A)	0.9800
C(25A)-H(25B)	0.9800	C(25A)-H(25C)	0.9800
C(25B)-H(25D)	0.9800	C(25B)-H(25E)	0.9800
C(25B)-H(25F)	0.9800	C(26A)-H(26A)	0.9800

C(26A)-H(26B)	0.9800	C(26A)-H(26C)	0.9800
C(26B)-H(26D)	0.9800	C(26B)-H(26E)	0.9800
C(26B)-H(26F)	0.9800	C(27A)-H(27A)	0.9800
C(27A)-H(27B)	0.9800	C(27A)-H(27C)	0.9800
C(27B)-H(27D)	0.9800	C(27B)-H(27E)	0.9800
C(27B)-H(27F)	0.9800	C(28)-C(29A)	1.628(4)
C(28)-C(29B)	1.656(4)	C(28)-C(30A)	1.274(4)
C(28)-C(30B)	1.722(4)	C(28)-C(31A)	1.664(4)
C(28)-C(31B)	1.361(4)	C(29A)-H(29A)	0.9800
C(29A)-H(29B)	0.9800	C(29A)-H(29C)	0.9800
C(29B)-H(29D)	0.9800	C(29B)-H(29E)	0.9800
C(29B)-H(29F)	0.9800	C(30A)-H(30A)	0.9800
C(30A)-H(30B)	0.9800	C(30A)-H(30C)	0.9800
C(30B)-H(30D)	0.9800	C(30B)-H(30E)	0.9800
C(30B)-H(30F)	0.9800	C(31A)-H(31A)	0.9800
C(31A)-H(31B)	0.9800	C(31A)-H(31C)	0.9800
C(31B)-H(31D)	0.9800	C(31B)-H(31E)	0.9800
C(31B)-H(31F)	0.9800		

C(1)-Bi(1)-C(7)	90.59(5)	C(18)-Bi(1)-C(1)	94.33(6)
C(18)-Bi(1)-C(7)	92.76(5)	O(1)-S(1)-N(1)	119.75(7)
O(1)-S(1)-C(2)	107.86(7)	O(1)-S(1)-C(8)	110.59(7)
N(1)-S(1)-C(2)	109.20(7)	N(1)-S(1)-C(8)	101.64(7)
C(8)-S(1)-C(2)	107.12(7)	C(17)-N(1)-S(1)	119.50(11)
C(2)-C(1)-Bi(1)	120.27(10)	C(2)-C(1)-C(6)	117.17(15)
C(6)-C(1)-Bi(1)	122.53(12)	C(1)-C(2)-S(1)	119.28(11)
C(3)-C(2)-S(1)	116.56(12)	C(3)-C(2)-C(1)	123.87(14)
C(2)-C(3)-H(3)	120.5	C(2)-C(3)-C(4)	118.93(17)
C(4)-C(3)-H(3)	120.5	C(3)-C(4)-C(13)	120.1(2)
C(5)-C(4)-C(3)	117.80(17)	C(5)-C(4)-C(13)	122.1(2)
C(4)-C(5)-H(5)	118.2	C(4)-C(5)-C(6)	123.50(17)
C(6)-C(5)-H(5)	118.2	C(1)-C(6)-C(14)	121.73(17)
C(5)-C(6)-C(1)	118.55(17)	C(5)-C(6)-C(14)	119.70(17)
C(8)-C(7)-Bi(1)	119.29(10)	C(8)-C(7)-C(12)	116.75(13)
C(12)-C(7)-Bi(1)	123.90(10)	C(7)-C(8)-S(1)	121.14(11)
C(9)-C(8)-S(1)	114.51(11)	C(9)-C(8)-C(7)	124.32(14)

C(8)-C(9)-H(9)	120.7	C(8)-C(9)-C(10)	118.53(14)
C(10)-C(9)-H(9)	120.7	C(9)-C(10)-C(15)	119.79(14)
C(11)-C(10)-C(9)	118.28(14)	C(11)-C(10)-C(15)	121.91(15)
C(10)-C(11)-H(11)	118.4	C(10)-C(11)-C(12)	123.20(14)
C(12)-C(11)-H(11)	118.4	C(7)-C(12)-C(16)	123.39(13)
C(11)-C(12)-C(7)	118.86(13)	C(11)-C(12)-C(16)	117.73(14)
C(4)-C(13)-H(13A)	109.5	C(4)-C(13)-H(13B)	109.5
C(4)-C(13)-H(13C)	109.5	H(13A)-C(13)-H(13B)	109.5
H(13A)-C(13)-H(13C)	109.5	H(13B)-C(13)-H(13C)	109.5
C(6)-C(14)-H(14A)	109.5	C(6)-C(14)-H(14B)	109.5
C(6)-C(14)-H(14C)	109.5	H(14A)-C(14)-H(14B)	109.5
H(14A)-C(14)-H(14C)	109.5	H(14B)-C(14)-H(14C)	109.5
C(10)-C(15)-H(15A)	109.5	C(10)-C(15)-H(15B)	109.5
C(10)-C(15)-H(15C)	109.5	H(15A)-C(15)-H(15B)	109.5
H(15A)-C(15)-H(15C)	109.5	H(15B)-C(15)-H(15C)	109.5
C(12)-C(16)-H(16A)	109.5	C(12)-C(16)-H(16B)	109.5
C(12)-C(16)-H(16C)	109.5	H(16A)-C(16)-H(16B)	109.5
H(16A)-C(16)-H(16C)	109.5	H(16B)-C(16)-H(16C)	109.5
F(1B)-C(17)-F(2B)	104.2(2)	F(1B)-C(17)-F(3B)	104.7(2)
F(1B)-C(17)-N(1)	122.93(16)	F(2A)-C(17)-F(1A)	102.92(19)
F(2A)-C(17)-N(1)	115.61(14)	F(3A)-C(17)-F(1A)	107.76(18)
F(3A)-C(17)-F(2A)	110.22(18)	F(3A)-C(17)-N(1)	111.44(18)
F(3B)-C(17)-F(2B)	98.6(2)	F(3B)-C(17)-N(1)	111.41(17)
N(1)-C(17)-F(1A)	108.26(14)	N(1)-C(17)-F(2B)	112.04(15)
C(19)-C(18)-Bi(1)	116.97(11)	C(23)-C(18)-Bi(1)	122.43(11)
C(23)-C(18)-C(19)	120.27(14)	C(18)-C(19)-H(19)	119.6
C(18)-C(19)-C(20)	120.82(15)	C(20)-C(19)-H(19)	119.6
C(19)-C(20)-C(24)	119.95(15)	C(21)-C(20)-C(19)	117.55(16)
C(21)-C(20)-C(24)	122.42(14)	C(20)-C(21)-H(21)	118.6
C(22)-C(21)-C(20)	122.84(14)	C(22)-C(21)-H(21)	118.6
C(21)-C(22)-C(23)	117.86(16)	C(21)-C(22)-C(28)	121.67(15)
C(23)-C(22)-C(28)	120.44(18)	C(18)-C(23)-C(22)	120.63(17)
C(18)-C(23)-H(23)	119.7	C(22)-C(23)-H(23)	119.7
C(20)-C(24)-C(25A)	106.76(17)	C(20)-C(24)-C(26B)	115.7(3)
C(20)-C(24)-C(27B)	106.6(3)	C(25B)-C(24)-C(20)	112.8(4)
C(25B)-C(24)-C(26B)	109.5(5)	C(25B)-C(24)-C(27B)	107.1(5)

C(26A)-C(24)-C(20)	110.73(16)	C(26A)-C(24)-C(25A)	109.3(2)
C(26A)-C(24)-C(27A)	109.2(2)	C(26B)-C(24)-C(27B)	104.2(4)
C(27A)-C(24)-C(20)	112.54(18)	C(27A)-C(24)-C(25A)	108.2(2)
C(24)-C(25A)-H(25A)	109.5	C(24)-C(25A)-H(25B)	109.5
C(24)-C(25A)-H(25C)	109.5	H(25A)-C(25A)-H(25B)	109.5
H(25A)-C(25A)-H(25C)	109.5	H(25B)-C(25A)-H(25C)	109.5
C(24)-C(25B)-H(25D)	109.5	C(24)-C(25B)-H(25E)	109.5
C(24)-C(25B)-H(25F)	109.5	H(25D)-C(25B)-H(25E)	109.5
H(25D)-C(25B)-H(25F)	109.5	H(25E)-C(25B)-H(25F)	109.5
C(24)-C(26A)-H(26A)	109.5	C(24)-C(26A)-H(26B)	109.5
C(24)-C(26A)-H(26C)	109.5	H(26A)-C(26A)-H(26B)	109.5
H(26A)-C(26A)-H(26C)	109.5	H(26B)-C(26A)-H(26C)	109.5
C(24)-C(26B)-H(26D)	109.5	C(24)-C(26B)-H(26E)	109.5
C(24)-C(26B)-H(26F)	109.5	H(26D)-C(26B)-H(26E)	109.5
H(26D)-C(26B)-H(26F)	109.5	H(26E)-C(26B)-H(26F)	109.5
C(24)-C(27A)-H(27A)	109.5	C(24)-C(27A)-H(27B)	109.5
C(24)-C(27A)-H(27C)	109.5	H(27A)-C(27A)-H(27B)	109.5
H(27A)-C(27A)-H(27C)	109.5	H(27B)-C(27A)-H(27C)	109.5
C(24)-C(27B)-H(27D)	109.5	C(24)-C(27B)-H(27E)	109.5
C(24)-C(27B)-H(27F)	109.5	H(27D)-C(27B)-H(27E)	109.5
H(27D)-C(27B)-H(27F)	109.5	H(27E)-C(27B)-H(27F)	109.5
C(22)-C(28)-C(29A)	107.6(2)	C(22)-C(28)-C(29B)	110.84(18)
C(22)-C(28)-C(30B)	112.61(18)	C(22)-C(28)-C(31A)	100.3(2)
C(29A)-C(28)-C(31A)	98.1(3)	C(29B)-C(28)-C(30B)	94.7(2)
C(30A)-C(28)-C(22)	112.9(3)	C(30A)-C(28)-C(29A)	118.8(3)
C(30A)-C(28)-C(31A)	116.7(3)	C(31B)-C(28)-C(22)	117.4(3)
C(31B)-C(28)-C(29B)	112.3(2)	C(31B)-C(28)-C(30B)	106.5(2)
C(28)-C(29A)-H(29A)	109.5	C(28)-C(29A)-H(29B)	109.5
C(28)-C(29A)-H(29C)	109.5	H(29A)-C(29A)-H(29B)	109.5
H(29A)-C(29A)-H(29C)	109.5	H(29B)-C(29A)-H(29C)	109.5
C(28)-C(29B)-H(29D)	109.5	C(28)-C(29B)-H(29E)	109.5
C(28)-C(29B)-H(29F)	109.5	H(29D)-C(29B)-H(29E)	109.5
H(29D)-C(29B)-H(29F)	109.5	H(29E)-C(29B)-H(29F)	109.5
C(28)-C(30A)-H(30A)	109.5	C(28)-C(30A)-H(30B)	109.5
C(28)-C(30A)-H(30C)	109.5	H(30A)-C(30A)-H(30B)	109.5
H(30A)-C(30A)-H(30C)	109.5	H(30B)-C(30A)-H(30C)	109.5

C(28)-C(30B)-H(30D)	109.5	C(28)-C(30B)-H(30E)	109.5
C(28)-C(30B)-H(30F)	109.5	H(30D)-C(30B)-H(30E)	109.5
H(30D)-C(30B)-H(30F)	109.5	H(30E)-C(30B)-H(30F)	109.5
C(28)-C(31A)-H(31A)	109.5	C(28)-C(31A)-H(31B)	109.5
C(28)-C(31A)-H(31C)	109.5	H(31A)-C(31A)-H(31B)	109.5
H(31A)-C(31A)-H(31C)	109.5	H(31B)-C(31A)-H(31C)	109.5
C(28)-C(31B)-H(31D)	109.5	C(28)-C(31B)-H(31E)	109.5
C(28)-C(31B)-H(31F)	109.5	H(31D)-C(31B)-H(31E)	109.5
H(31D)-C(31B)-H(31F)	109.5	H(31E)-C(31B)-H(31F)	109.5

8.16 Single crystal structure analysis of **96b**

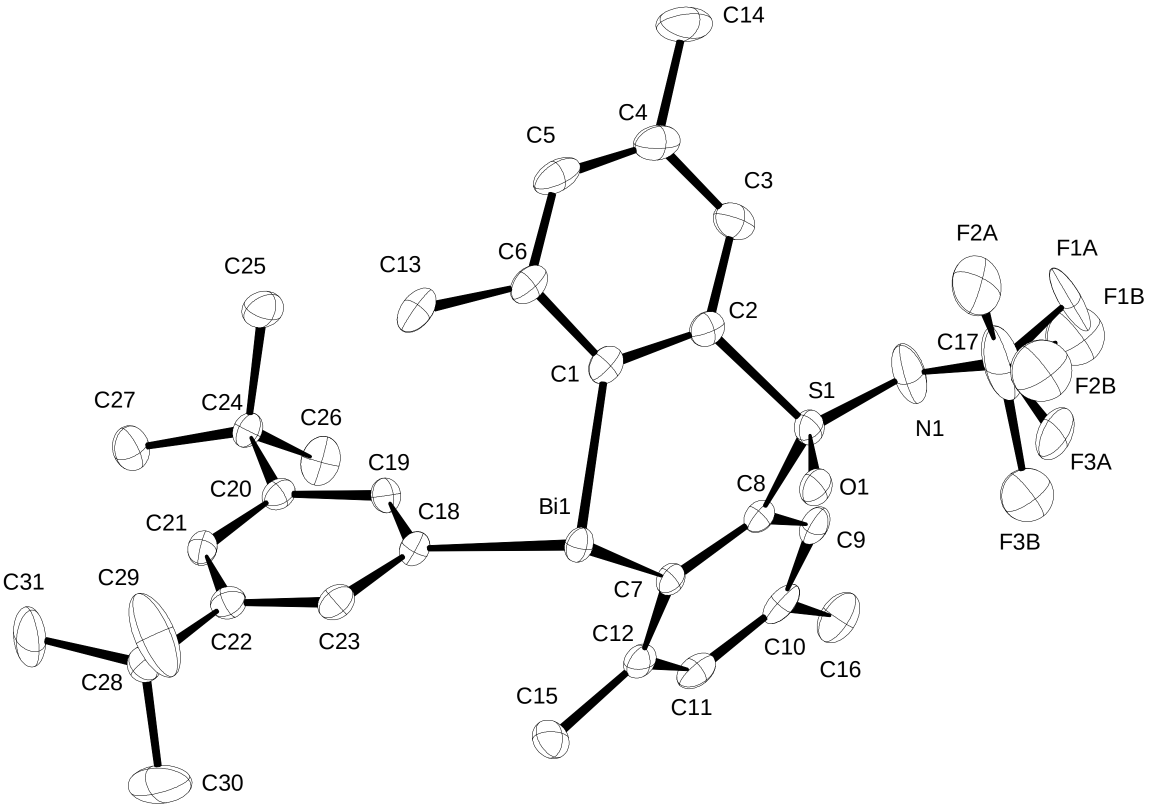

Figure S109. The molecular structure of complex **96b**. H atoms have been removed for clarity.

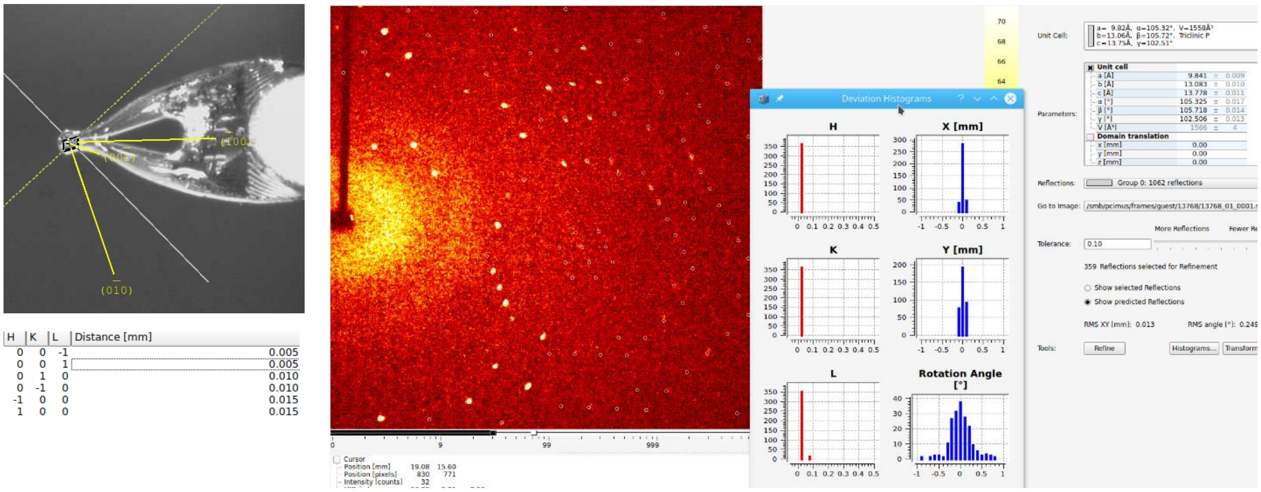

Figure S110. Crystal faces and unit cell determination of **96b**.

INTENSITY STATISTICS FOR DATASET

Resolution	#Data	#Theory	%Complete	Redundancy	Mean I	Mean I/s	Rmerge	Rsigma
Inf - 2.76	151	151	100.0	9.28	200.04	75.37	0.0271	0.0112
2.76 - 1.83	356	356	100.0	9.40	136.99	63.53	0.0291	0.0127
1.83 - 1.45	519	519	100.0	9.39	100.13	55.71	0.0325	0.0149
1.45 - 1.27	497	497	100.0	9.23	76.57	46.65	0.0381	0.0173
1.27 - 1.15	534	534	100.0	8.85	61.04	39.44	0.0446	0.0210
1.15 - 1.07	484	484	100.0	7.75	55.55	33.14	0.0512	0.0254
1.07 - 1.01	495	495	100.0	6.12	45.89	25.66	0.0586	0.0334
1.01 - 0.96	483	483	100.0	5.18	44.68	22.50	0.0616	0.0385
0.96 - 0.91	620	620	100.0	4.47	36.86	17.77	0.0727	0.0485
0.91 - 0.88	437	437	100.0	4.09	32.28	15.54	0.0765	0.0572
0.88 - 0.85	490	490	100.0	3.96	28.35	13.44	0.0865	0.0656
0.85 - 0.82	602	602	100.0	3.71	26.78	12.02	0.0890	0.0733
0.82 - 0.80	400	400	100.0	3.65	25.05	10.76	0.0951	0.0818
0.80 - 0.78	523	523	100.0	3.49	23.30	10.00	0.1110	0.0909
0.78 - 0.76	524	524	100.0	3.51	21.79	9.36	0.1188	0.0981
0.76 - 0.74	595	597	99.7	3.25	18.16	7.57	0.1342	0.1215
0.74 - 0.72	661	662	99.8	3.25	16.99	6.94	0.1468	0.1347
0.72 - 0.71	340	341	99.7	3.08	14.52	5.81	0.1758	0.1610
0.71 - 0.70	387	389	99.5	3.07	14.13	5.73	0.1791	0.1678
0.70 - 0.68	808	815	99.1	2.82	13.21	4.99	0.1916	0.1970
0.68 - 0.67	147	247	59.5	1.11	11.76	3.48	0.2099	0.2900
0.77 - 0.67	3201	3314	96.6	2.97	15.70	6.30	0.1571	0.1533
Inf - 0.67	10053	10166	98.9	5.01	41.60	20.95	0.0507	0.0467

Terminal CF_3 group shows a rotational disorder over two positions with 60:40 occupancy. The minor component was treated using isotropic displacement parameters.

Complete .cif-data of the compound are available under the CCDC number **CCDC-2125780.**

Table S57. Crystal data and structure refinement of **96b**.

Identification code	13768sadabs	
Empirical formula	$C_{31} H_{37} Bi F_3 N O S$	
Color	colourless	
Formula weight	737.65 g·mol^{-1}	
Temperature	100(2) K	
Wavelength	0.71073 Å	
Crystal system	Triclinic	
Space group	*P*-1, (no. 2)	
Unit cell dimensions	a = 9.6878(4) Å	α= 105.256(2)°.
	b = 12.8720(6) Å	β= 105.669(2)°.
	c = 13.5320(7) Å	γ = 102.566(2)°.
Volume	1489.90(12) Å^3	
Z	2	
Density (calculated)	1.644 Mg·m^{-3}	
Absorption coefficient	6.030 mm^{-1}	
F(000)	728 e	
Crystal size	0.040 x 0.025 x 0.012 mm^3	
θ range for data collection	1.664 to 31.789°.	
Index ranges	-14 ≤ h ≤ 14, -19≤ k ≤ 19, -19 ≤ l ≤ 20	
Reflections collected	50980	
Independent reflections	10053 [R_{int} = 0.0513]	
Reflections with I>2σ(I)	8491	
Completeness to θ = 25.242°	100.0 %	
Absorption correction	Gaussian	
Max. and min. transmission	0.94778 and 0.83829	
Refinement method	Full-matrix least-squares on F^2	
Data / restraints / parameters	10053 / 0 / 365	
Goodness-of-fit on F^2	1.047	
Final R indices [I>2σ(I)]	R_1 = 0.0319	wR^2 = 0.0595
R indices (all data)	R_1 = 0.0454	wR^2 = 0.0628
Extinction coefficient	n/a	
Largest diff. peak and hole	1.727 and -1.766 e·Å^{-3}	

Table S58. Bond lengths [Å] and angles [°] of **96b**.

Bi(1)-C(1)	2.285(3)	Bi(1)-C(7)	2.268(3)
Bi(1)-C(18)	2.247(3)	S(1)-O(1)	1.451(2)
S(1)-N(1)	1.527(3)	S(1)-C(2)	1.771(3)
S(1)-C(8)	1.774(3)	F(1A)-C(17)	1.321(6)
F(1B)-C(17)	1.401(11)	F(2A)-C(17)	1.512(7)
F(2B)-C(17)	1.163(8)	F(3A)-C(17)	1.269(5)
F(3B)-C(17)	1.706(8)	N(1)-C(17)	1.293(5)
C(1)-C(2)	1.400(5)	C(1)-C(6)	1.410(4)
C(2)-C(3)	1.386(4)	C(3)-H(3)	0.9500
C(3)-C(4)	1.393(5)	C(4)-C(5)	1.383(5)
C(4)-C(14)	1.514(5)	C(5)-H(5)	0.9500
C(5)-C(6)	1.396(4)	C(6)-C(13)	1.512(5)
C(7)-C(8)	1.392(4)	C(7)-C(12)	1.404(4)
C(8)-C(9)	1.388(4)	C(9)-H(9)	0.9500
C(9)-C(10)	1.395(4)	C(10)-C(11)	1.389(5)
C(10)-C(16)	1.505(4)	C(11)-H(11)	0.9500
C(11)-C(12)	1.399(4)	C(12)-C(15)	1.501(4)
C(13)-H(13A)	0.9800	C(13)-H(13B)	0.9800
C(13)-H(13C)	0.9800	C(14)-H(14A)	0.9800
C(14)-H(14B)	0.9800	C(14)-H(14C)	0.9800
C(15)-H(15A)	0.9800	C(15)-H(15B)	0.9800
C(15)-H(15C)	0.9800	C(16)-H(16A)	0.9800
C(16)-H(16B)	0.9800	C(16)-H(16C)	0.9800
C(18)-C(19)	1.396(4)	C(18)-C(23)	1.386(4)
C(19)-H(19)	0.9500	C(19)-C(20)	1.396(4)
C(20)-C(21)	1.390(4)	C(20)-C(24)	1.531(4)
C(21)-H(21)	0.9500	C(21)-C(22)	1.395(4)
C(22)-C(23)	1.394(4)	C(22)-C(28)	1.528(4)
C(23)-H(23)	0.9500	C(24)-C(25)	1.537(4)
C(24)-C(26)	1.529(4)	C(24)-C(27)	1.527(4)
C(25)-H(25A)	0.9800	C(25)-H(25B)	0.9800
C(25)-H(25C)	0.9800	C(26)-H(26A)	0.9800
C(26)-H(26B)	0.9800	C(26)-H(26C)	0.9800
C(27)-H(27A)	0.9800	C(27)-H(27B)	0.9800
C(27)-H(27C)	0.9800	C(28)-C(29)	1.526(5)

C(28)-C(30)	1.512(5)	C(28)-C(31)	1.520(5)
C(29)-H(29A)	0.9800	C(29)-H(29B)	0.9800
C(29)-H(29C)	0.9800	C(30)-H(30A)	0.9800
C(30)-H(30B)	0.9800	C(30)-H(30C)	0.9800
C(31)-H(31A)	0.9800	C(31)-H(31B)	0.9800
C(31)-H(31C)	0.9800		

C(7)-Bi(1)-C(1)	88.46(10)	C(18)-Bi(1)-C(1)	94.93(10)
C(18)-Bi(1)-C(7)	95.56(10)	O(1)-S(1)-N(1)	120.76(14)
O(1)-S(1)-C(2)	106.88(13)	O(1)-S(1)-C(8)	107.44(13)
N(1)-S(1)-C(2)	109.15(17)	N(1)-S(1)-C(8)	106.38(17)
C(2)-S(1)-C(8)	105.22(13)	C(17)-N(1)-S(1)	126.1(3)
C(2)-C(1)-Bi(1)	119.5(2)	C(2)-C(1)-C(6)	116.4(3)
C(6)-C(1)-Bi(1)	123.9(2)	C(1)-C(2)-S(1)	117.3(2)
C(3)-C(2)-S(1)	118.2(3)	C(3)-C(2)-C(1)	124.4(3)
C(2)-C(3)-H(3)	120.8	C(2)-C(3)-C(4)	118.5(3)
C(4)-C(3)-H(3)	120.8	C(3)-C(4)-C(14)	119.6(4)
C(5)-C(4)-C(3)	118.4(3)	C(5)-C(4)-C(14)	122.0(3)
C(4)-C(5)-H(5)	118.4	C(4)-C(5)-C(6)	123.2(3)
C(6)-C(5)-H(5)	118.4	C(1)-C(6)-C(13)	122.7(3)
C(5)-C(6)-C(1)	119.2(3)	C(5)-C(6)-C(13)	118.1(3)
C(8)-C(7)-Bi(1)	120.1(2)	C(8)-C(7)-C(12)	117.3(3)
C(12)-C(7)-Bi(1)	122.6(2)	C(7)-C(8)-S(1)	116.8(2)
C(9)-C(8)-S(1)	118.9(2)	C(9)-C(8)-C(7)	124.1(3)
C(8)-C(9)-H(9)	120.8	C(8)-C(9)-C(10)	118.5(3)
C(10)-C(9)-H(9)	120.8	C(9)-C(10)-C(16)	120.8(3)
C(11)-C(10)-C(9)	118.1(3)	C(11)-C(10)-C(16)	121.1(3)
C(10)-C(11)-H(11)	118.3	C(10)-C(11)-C(12)	123.4(3)
C(12)-C(11)-H(11)	118.3	C(7)-C(12)-C(15)	122.9(3)
C(11)-C(12)-C(7)	118.5(3)	C(11)-C(12)-C(15)	118.6(3)
C(6)-C(13)-H(13A)	109.5	C(6)-C(13)-H(13B)	109.5
C(6)-C(13)-H(13C)	109.5	H(13A)-C(13)-H(13B)	109.5
H(13A)-C(13)-H(13C)	109.5	H(13B)-C(13)-H(13C)	109.5
C(4)-C(14)-H(14A)	109.5	C(4)-C(14)-H(14B)	109.5
C(4)-C(14)-H(14C)	109.5	H(14A)-C(14)-H(14B)	109.5
H(14A)-C(14)-H(14C)	109.5	H(14B)-C(14)-H(14C)	109.5

C(12)-C(15)-H(15A)	109.5	C(12)-C(15)-H(15B)	109.5
C(12)-C(15)-H(15C)	109.5	H(15A)-C(15)-H(15B)	109.5
H(15A)-C(15)-H(15C)	109.5	H(15B)-C(15)-H(15C)	109.5
C(10)-C(16)-H(16A)	109.5	C(10)-C(16)-H(16B)	109.5
C(10)-C(16)-H(16C)	109.5	H(16A)-C(16)-H(16B)	109.5
H(16A)-C(16)-H(16C)	109.5	H(16B)-C(16)-H(16C)	109.5
F(1A)-C(17)-F(2A)	96.8(5)	F(1B)-C(17)-F(3B)	87.6(5)
F(2B)-C(17)-F(1B)	113.6(7)	F(2B)-C(17)-F(3B)	92.2(6)
F(2B)-C(17)-N(1)	140.5(6)	F(3A)-C(17)-F(1A)	109.1(4)
F(3A)-C(17)-F(2A)	99.5(4)	F(3A)-C(17)-N(1)	128.2(5)
N(1)-C(17)-F(1A)	114.0(4)	N(1)-C(17)-F(1B)	105.9(5)
N(1)-C(17)-F(2A)	102.5(4)	N(1)-C(17)-F(3B)	88.2(4)
C(19)-C(18)-Bi(1)	125.2(2)	C(23)-C(18)-Bi(1)	115.1(2)
C(23)-C(18)-C(19)	119.5(3)	C(18)-C(19)-H(19)	119.7
C(18)-C(19)-C(20)	120.6(3)	C(20)-C(19)-H(19)	119.7
C(19)-C(20)-C(24)	119.7(3)	C(21)-C(20)-C(19)	118.0(3)
C(21)-C(20)-C(24)	122.2(3)	C(20)-C(21)-H(21)	118.5
C(20)-C(21)-C(22)	123.0(3)	C(22)-C(21)-H(21)	118.5
C(21)-C(22)-C(28)	122.5(3)	C(23)-C(22)-C(21)	117.1(3)
C(23)-C(22)-C(28)	120.3(3)	C(18)-C(23)-C(22)	121.7(3)
C(18)-C(23)-H(23)	119.1	C(22)-C(23)-H(23)	119.1
C(20)-C(24)-C(25)	107.8(2)	C(26)-C(24)-C(20)	110.3(2)
C(26)-C(24)-C(25)	109.3(3)	C(27)-C(24)-C(20)	113.1(2)
C(27)-C(24)-C(25)	108.5(2)	C(27)-C(24)-C(26)	107.8(2)
C(24)-C(25)-H(25A)	109.5	C(24)-C(25)-H(25B)	109.5
C(24)-C(25)-H(25C)	109.5	H(25A)-C(25)-H(25B)	109.5
H(25A)-C(25)-H(25C)	109.5	H(25B)-C(25)-H(25C)	109.5
C(24)-C(26)-H(26A)	109.5	C(24)-C(26)-H(26B)	109.5
C(24)-C(26)-H(26C)	109.5	H(26A)-C(26)-H(26B)	109.5
H(26A)-C(26)-H(26C)	109.5	H(26B)-C(26)-H(26C)	109.5
C(24)-C(27)-H(27A)	109.5	C(24)-C(27)-H(27B)	109.5
C(24)-C(27)-H(27C)	109.5	H(27A)-C(27)-H(27B)	109.5
H(27A)-C(27)-H(27C)	109.5	H(27B)-C(27)-H(27C)	109.5
C(29)-C(28)-C(22)	110.3(3)	C(30)-C(28)-C(22)	109.2(3)
C(30)-C(28)-C(29)	110.4(4)	C(30)-C(28)-C(31)	107.7(3)
C(31)-C(28)-C(22)	112.3(3)	C(31)-C(28)-C(29)	107.0(3)

C(28)-C(29)-H(29A)	109.5	C(28)-C(29)-H(29B)	109.5
C(28)-C(29)-H(29C)	109.5	H(29A)-C(29)-H(29B)	109.5
H(29A)-C(29)-H(29C)	109.5	H(29B)-C(29)-H(29C)	109.5
C(28)-C(30)-H(30A)	109.5	C(28)-C(30)-H(30B)	109.5
C(28)-C(30)-H(30C)	109.5	H(30A)-C(30)-H(30B)	109.5
H(30A)-C(30)-H(30C)	109.5	H(30B)-C(30)-H(30C)	109.5
C(28)-C(31)-H(31A)	109.5	C(28)-C(31)-H(31B)	109.5
C(28)-C(31)-H(31C)	109.5	H(31A)-C(31)-H(31B)	109.5
H(31A)-C(31)-H(31C)	109.5	H(31B)-C(31)-H(31C)	109.5

8.17 Single crystal structure analysis of **97**

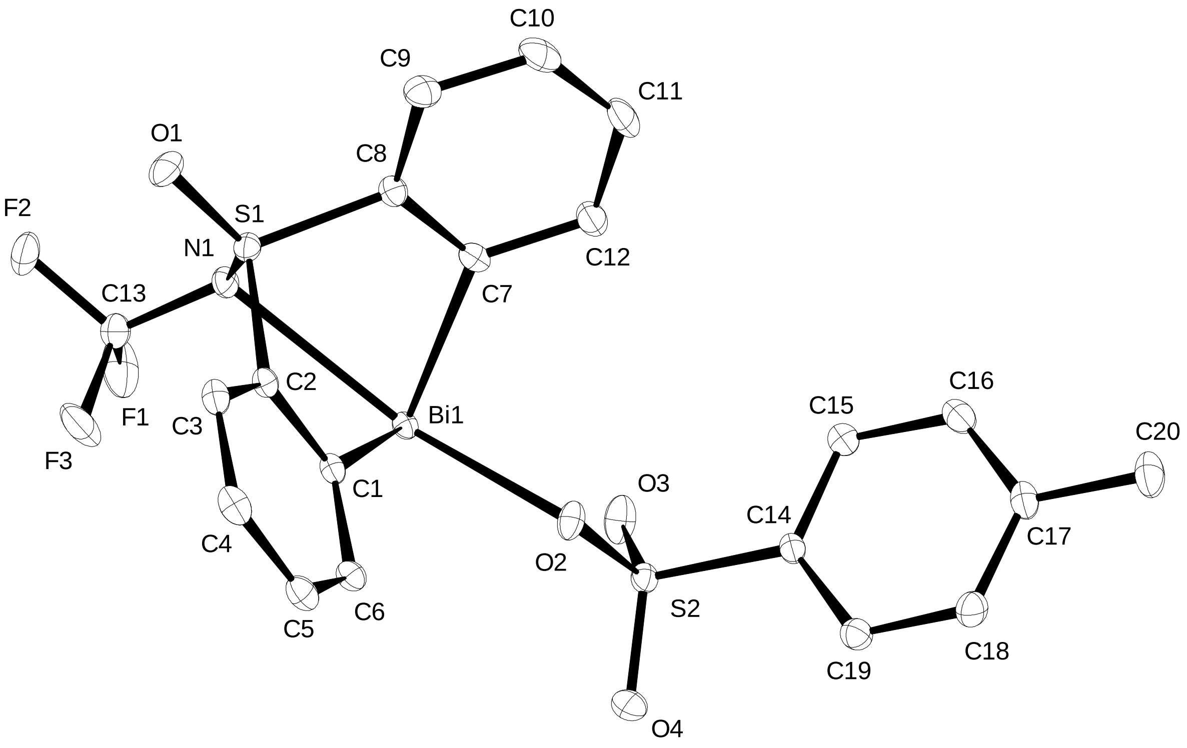

Figure S111. The molecular structure of complex **97**. H atoms have been removed for clarity.

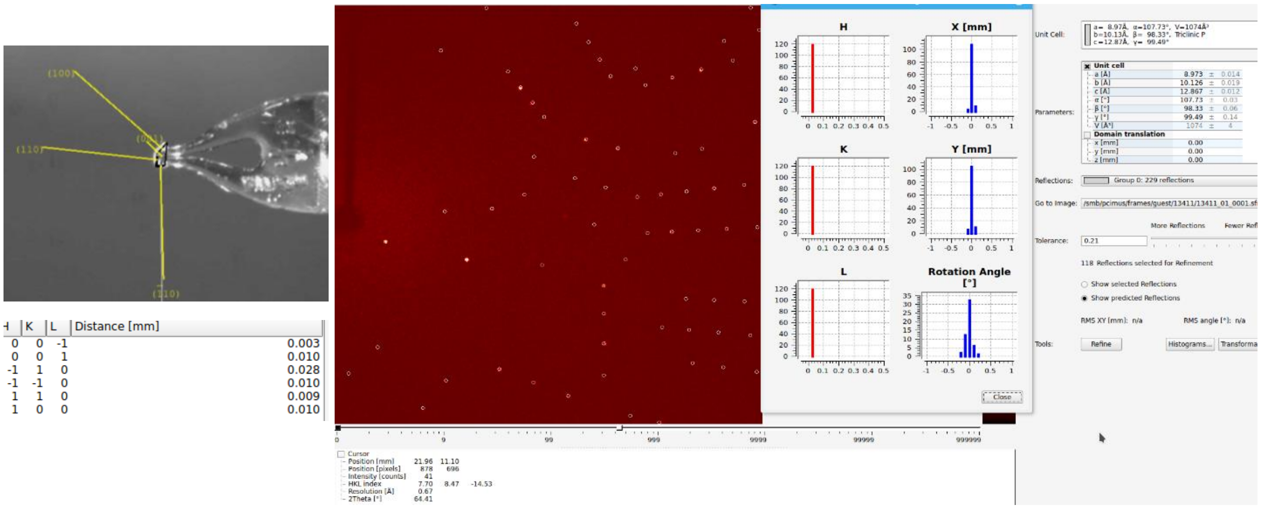

Figure S112. Crystal faces and unit cell determination of **97**.

INTENSITY STATISTICS FOR DATASET

Resolution	#Data	#Theory	%Complete	Redundancy	Mean I	Mean I/s	Rmerge	Rsigma
Inf - 2.64	120	120	100.0	9.33	205.66	100.01	0.0218	0.0083
2.64 - 1.75	283	283	100.0	9.50	168.22	95.79	0.0198	0.0090
1.75 - 1.40	389	389	100.0	9.48	119.82	84.91	0.0209	0.0098
1.40 - 1.22	401	401	100.0	9.31	96.47	74.91	0.0229	0.0109
1.22 - 1.10	424	424	100.0	8.74	78.84	65.41	0.0260	0.0124
1.10 - 1.03	359	359	100.0	6.77	69.96	52.42	0.0285	0.0154
1.03 - 0.97	390	390	100.0	5.62	64.96	46.06	0.0295	0.0179
0.97 - 0.92	415	415	100.0	4.82	59.56	40.91	0.0331	0.0208
0.92 - 0.88	392	392	100.0	4.15	47.81	32.95	0.0344	0.0246
0.88 - 0.84	478	478	100.0	3.98	43.71	30.00	0.0360	0.0272
0.84 - 0.82	283	283	100.0	3.88	40.08	27.80	0.0385	0.0300
0.82 - 0.79	464	464	100.0	3.70	40.99	27.52	0.0403	0.0307
0.79 - 0.77	358	358	100.0	3.50	36.01	23.59	0.0419	0.0357
0.77 - 0.75	370	370	100.0	3.43	34.60	22.55	0.0477	0.0374
0.75 - 0.73	445	445	100.0	3.37	32.76	21.14	0.0477	0.0403
0.73 - 0.71	481	482	99.8	3.15	29.48	18.95	0.0500	0.0457
0.71 - 0.70	267	270	98.9	3.15	28.18	18.39	0.0548	0.0473
0.70 - 0.68	554	555	99.8	3.06	27.93	17.59	0.0586	0.0513
0.68 - 0.67	326	331	98.5	2.90	22.06	14.24	0.0623	0.0597
0.67 - 0.66	330	333	99.1	2.86	23.82	14.84	0.0661	0.0607
0.66 - 0.65	345	348	99.1	2.78	21.58	13.56	0.0751	0.0655
0.75 - 0.65	2748	2764	99.4	3.06	27.02	17.25	0.0566	0.0509
Inf - 0.65	7874	7890	99.8	4.93	55.39	37.56	0.0282	0.0228

Complete .cif-data of the compound are available under the CCDC number **CCDC-2125768.**

Table S59. Crystal data and structure refinement of **97**.

Identification code	13411sad	
Empirical formula	$C_{20} H_{15} Bi F_3 N O_4 S_2$	
Color	colourless	
Formula weight	663.43 g·mol^{-1}	
Temperature	100(2) K	
Wavelength	0.71073 Å	
Crystal system	Triclinic	
Space group	*P*-1, (no. 2)	
Unit cell dimensions	a = 8.8643(5) Å	α= 107.647(2)°.
	b = 9.9728(5) Å	β= 98.624(2)°.
	c = 12.7272(7) Å	γ = 99.176(2)°.
Volume	1034.66(10) Å^3	
Z	2	
Density (calculated)	2.129 Mg·m^{-3}	
Absorption coefficient	8.777 mm^{-1}	
F(000)	632 e	
Crystal size	0.064 x 0.028 x 0.022 mm^3	
θ range for data collection	1.718 to 33.142°.	
Index ranges	-13 ≤ h ≤ 13, -15≤ k ≤ 15, -19 ≤ l ≤ 19	
Reflections collected	38872	
Independent reflections	7874 [R_{int} = 0.0282]	
Reflections with I>2σ(I)	7365	
Completeness to θ = 25.242°	100.0 %	
Absorption correction	Gaussian	
Max. and min. transmission	0.90767 and 0.68917	
Refinement method	Full-matrix least-squares on F^2	
Data / restraints / parameters	7874 / 0 / 281	
Goodness-of-fit on F^2	1.062	
Final R indices [I>2σ(I)]	R_1 = 0.0160	wR^2 = 0.0335
R indices (all data)	R_1 = 0.0185	wR^2 = 0.0340
Extinction coefficient	n/a	
Largest diff. peak and hole	0.798 and -1.016 e·Å^{-3}	

Table S60. Bond lengths [Å] and angles [°] of **97**.

Bi(1)-O(2)	2.2831(12)	Bi(1)-N(1)	2.6074(14)
Bi(1)-C(1)	2.2643(15)	Bi(1)-C(7)	2.2729(16)
S(1)-O(1)	1.4367(12)	S(1)-N(1)	1.5822(14)
S(1)-C(2)	1.7698(16)	S(1)-C(8)	1.7598(17)
S(2)-O(2)	1.5089(12)	S(2)-O(3)	1.4445(13)
S(2)-O(4)	1.4390(13)	S(2)-C(14)	1.7613(17)
F(1)-C(13)	1.336(2)	F(2)-C(13)	1.340(2)
F(3)-C(13)	1.347(2)	N(1)-C(13)	1.394(2)
C(1)-C(2)	1.393(2)	C(1)-C(6)	1.389(2)
C(2)-C(3)	1.391(2)	C(3)-H(3)	0.9500
C(3)-C(4)	1.389(2)	C(4)-H(4)	0.9500
C(4)-C(5)	1.392(2)	C(5)-H(5)	0.9500
C(5)-C(6)	1.395(2)	C(6)-H(6)	0.9500
C(7)-C(8)	1.392(2)	C(7)-C(12)	1.394(2)
C(8)-C(9)	1.392(2)	C(9)-H(9)	0.9500
C(9)-C(10)	1.389(3)	C(10)-H(10)	0.9500
C(10)-C(11)	1.386(3)	C(11)-H(11)	0.9500
C(11)-C(12)	1.396(2)	C(12)-H(12)	0.9500
C(14)-C(15)	1.389(2)	C(14)-C(19)	1.388(2)
C(15)-H(15)	0.9500	C(15)-C(16)	1.390(3)
C(16)-H(16)	0.9500	C(16)-C(17)	1.392(3)
C(17)-C(18)	1.390(2)	C(17)-C(20)	1.503(3)
C(18)-H(18)	0.9500	C(18)-C(19)	1.388(3)
C(19)-H(19)	0.9500	C(20)-H(20A)	0.9800
C(20)-H(20B)	0.9800	C(20)-H(20C)	0.9800
O(2)-Bi(1)-N(1)	152.46(4)	C(1)-Bi(1)-O(2)	84.00(5)
C(1)-Bi(1)-N(1)	75.22(5)	C(1)-Bi(1)-C(7)	84.83(6)
C(7)-Bi(1)-O(2)	88.04(5)	C(7)-Bi(1)-N(1)	72.47(5)
O(1)-S(1)-N(1)	120.17(8)	O(1)-S(1)-C(2)	111.40(8)
O(1)-S(1)-C(8)	113.66(8)	N(1)-S(1)-C(2)	106.19(7)
N(1)-S(1)-C(8)	100.34(8)	C(8)-S(1)-C(2)	103.29(8)
O(2)-S(2)-C(14)	104.16(7)	O(3)-S(2)-O(2)	109.05(7)
O(3)-S(2)-C(14)	108.95(8)	O(4)-S(2)-O(2)	110.32(8)
O(4)-S(2)-O(3)	116.51(8)	O(4)-S(2)-C(14)	107.12(8)
S(2)-O(2)-Bi(1)	114.32(6)	S(1)-N(1)-Bi(1)	98.46(6)

C(13)-N(1)-Bi(1)	114.57(10)	C(13)-N(1)-S(1)	117.91(12)
C(2)-C(1)-Bi(1)	117.92(11)	C(6)-C(1)-Bi(1)	124.33(11)
C(6)-C(1)-C(2)	117.75(14)	C(1)-C(2)-S(1)	115.56(12)
C(3)-C(2)-S(1)	120.64(12)	C(3)-C(2)-C(1)	123.79(15)
C(2)-C(3)-H(3)	121.3	C(4)-C(3)-C(2)	117.41(15)
C(4)-C(3)-H(3)	121.3	C(3)-C(4)-H(4)	120.0
C(3)-C(4)-C(5)	120.04(16)	C(5)-C(4)-H(4)	120.0
C(4)-C(5)-H(5)	119.3	C(4)-C(5)-C(6)	121.41(16)
C(6)-C(5)-H(5)	119.3	C(1)-C(6)-C(5)	119.61(15)
C(1)-C(6)-H(6)	120.2	C(5)-C(6)-H(6)	120.2
C(8)-C(7)-Bi(1)	116.66(12)	C(8)-C(7)-C(12)	117.06(15)
C(12)-C(7)-Bi(1)	126.12(12)	C(7)-C(8)-S(1)	116.96(12)
C(9)-C(8)-S(1)	118.48(13)	C(9)-C(8)-C(7)	124.23(16)
C(8)-C(9)-H(9)	121.4	C(10)-C(9)-C(8)	117.23(16)
C(10)-C(9)-H(9)	121.4	C(9)-C(10)-H(10)	119.9
C(11)-C(10)-C(9)	120.23(16)	C(11)-C(10)-H(10)	119.9
C(10)-C(11)-H(11)	119.4	C(10)-C(11)-C(12)	121.30(17)
C(12)-C(11)-H(11)	119.4	C(7)-C(12)-C(11)	119.92(16)
C(7)-C(12)-H(12)	120.0	C(11)-C(12)-H(12)	120.0
F(1)-C(13)-F(2)	106.45(14)	F(1)-C(13)-F(3)	106.58(14)
F(1)-C(13)-N(1)	108.45(14)	F(2)-C(13)-F(3)	105.02(14)
F(2)-C(13)-N(1)	113.76(14)	F(3)-C(13)-N(1)	115.96(14)
C(15)-C(14)-S(2)	119.86(13)	C(19)-C(14)-S(2)	119.47(13)
C(19)-C(14)-C(15)	120.67(16)	C(14)-C(15)-H(15)	120.5
C(14)-C(15)-C(16)	119.09(16)	C(16)-C(15)-H(15)	120.5
C(15)-C(16)-H(16)	119.4	C(15)-C(16)-C(17)	121.26(16)
C(17)-C(16)-H(16)	119.4	C(16)-C(17)-C(20)	120.92(16)
C(18)-C(17)-C(16)	118.45(16)	C(18)-C(17)-C(20)	120.63(17)
C(17)-C(18)-H(18)	119.4	C(19)-C(18)-C(17)	121.22(16)
C(19)-C(18)-H(18)	119.4	C(14)-C(19)-H(19)	120.4
C(18)-C(19)-C(14)	119.30(16)	C(18)-C(19)-H(19)	120.4
C(17)-C(20)-H(20A)	109.5	C(17)-C(20)-H(20B)	109.5
C(17)-C(20)-H(20C)	109.5	H(20A)-C(20)-H(20B)	109.5
H(20A)-C(20)-H(20C)	109.5	H(20B)-C(20)-H(20C)	109.5

<u>8.18 Single crystal structure analysis of **108**</u>

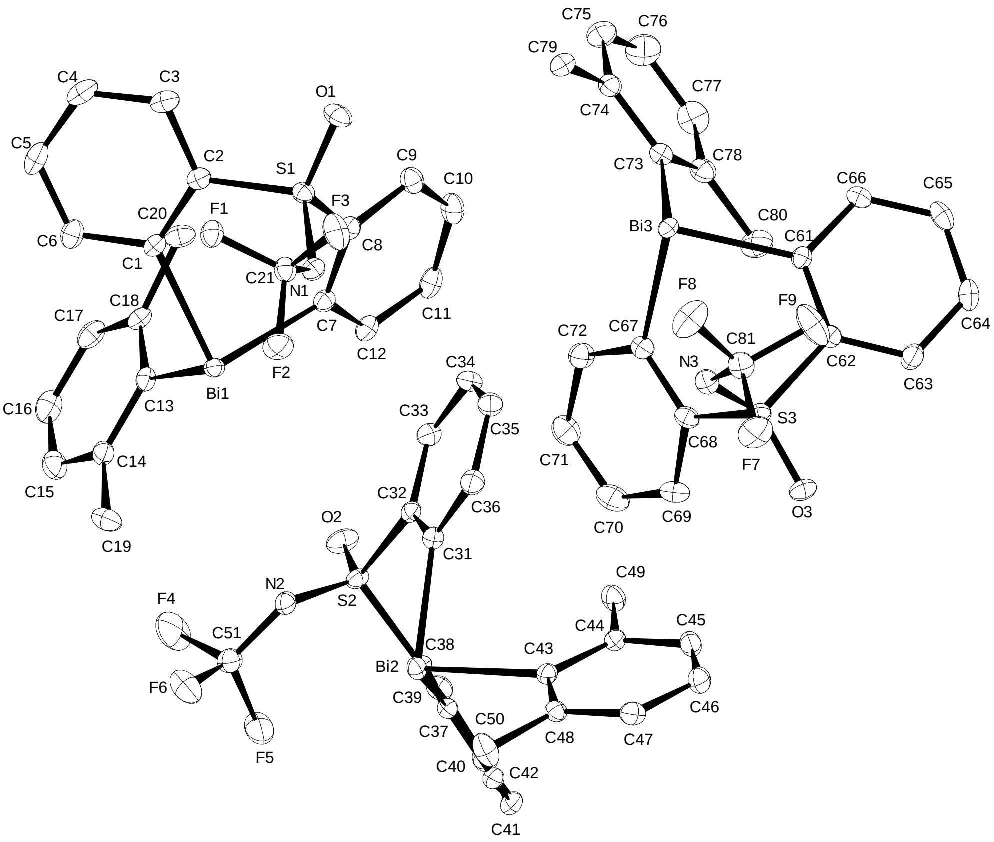

Figure S113. The molecular structure of complex **108**. H atoms have been removed for clarity.

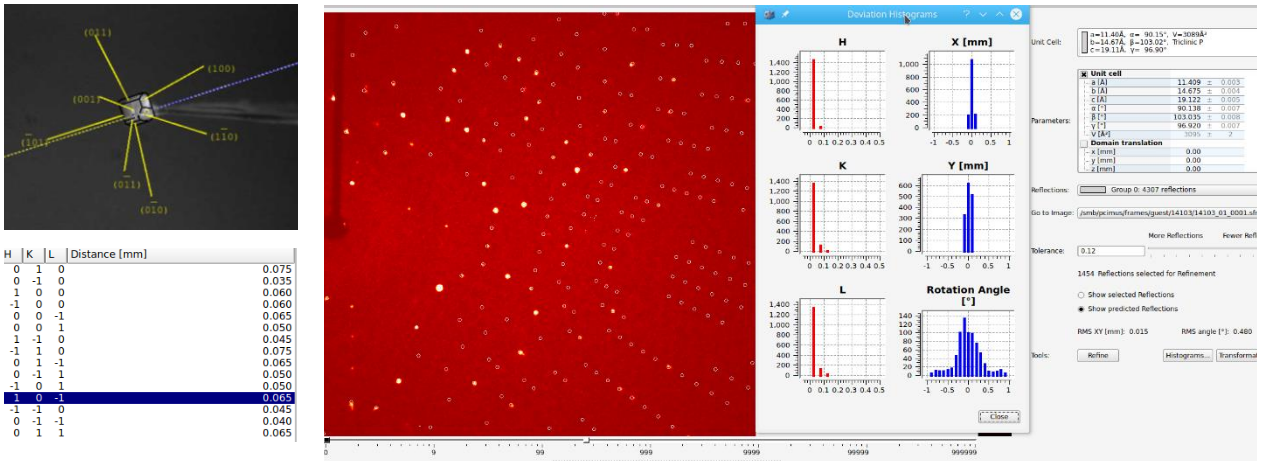

Figure S114. Crystal faces and unit cell determination of **108**.

INTENSITY STATISTICS FOR DATASET

Resolution	#Data	#Theory	%Complete	Redundancy	Mean I	Mean I/s	Rmerge	Rsigma
Inf - 2.56	373	373	100.0	9.31	112.80	45.01	0.0305	0.0202
2.56 - 1.71	860	860	100.0	9.53	83.20	41.61	0.0350	0.0203
1.71 - 1.36	1230	1230	100.0	9.44	60.13	38.45	0.0425	0.0212
1.36 - 1.18	1294	1294	100.0	9.11	42.84	33.92	0.0518	0.0229
1.18 - 1.07	1294	1294	100.0	8.09	37.91	29.96	0.0579	0.0254
1.07 - 1.00	1126	1126	100.0	6.03	32.66	24.40	0.0565	0.0313
1.00 - 0.94	1244	1244	100.0	5.05	29.92	21.91	0.0550	0.0349
0.94 - 0.89	1330	1330	100.0	4.33	23.27	18.16	0.0572	0.0410
0.89 - 0.85	1271	1271	100.0	4.00	22.01	17.20	0.0551	0.0435
0.85 - 0.82	1160	1160	100.0	3.81	19.11	15.27	0.0615	0.0482
0.82 - 0.79	1337	1337	100.0	3.66	17.92	14.40	0.0649	0.0522
0.79 - 0.77	1012	1012	100.0	3.53	16.56	12.99	0.0653	0.0564
0.77 - 0.74	1686	1687	99.9	3.41	14.89	12.29	0.0732	0.0619
0.74 - 0.72	1288	1291	99.8	3.25	13.37	11.37	0.0789	0.0691
0.72 - 0.71	754	756	99.7	3.21	13.84	10.97	0.0779	0.0698
0.71 - 0.69	1500	1507	99.5	3.07	11.49	10.08	0.0885	0.0796
0.69 - 0.67	1750	1763	99.3	2.96	10.27	8.98	0.0970	0.0895
0.67 - 0.66	889	898	99.0	2.90	9.94	8.79	0.1038	0.0943
0.66 - 0.65	1019	1033	98.6	2.73	9.33	8.01	0.1051	0.1023
0.65 - 0.64	1062	1084	98.0	2.74	8.84	7.67	0.1161	0.1076
0.64 - 0.63	1087	1114	97.6	2.68	7.73	6.95	0.1204	0.1185
0.73 - 0.63	8683	8779	98.9	2.92	10.38	8.96	0.0963	0.0900
Inf - 0.63	24566	24664	99.6	4.70	24.91	17.89	0.0507	0.0406

Complete .cif-data of the compound are available under the CCDC number **CCDC-2125788.**

Table S61. Crystal data and structure refinement of **108**.

Identification code	14103sadabs	
Empirical formula	$C_{21} H_{17} Bi F_3 N O S$	
Color	colourless	
Formula weight	597.40 g·mol^{-1}	
Temperature	100(2) K	
Wavelength	0.71073 Å	
Crystal system	Triclinic	
Space group	*P*-1, (no. 2)	
Unit cell dimensions	a = 11.2194(5) Å	α= 90.139(2)°.
	b = 14.4438(7) Å	β= 103.049(2)°.
	c = 18.8023(9) Å	γ = 96.900(2)°.
Volume	2945.4(2) Å^3	
Z	6	
Density (calculated)	2.021 Mg·m^{-3}	
Absorption coefficient	9.124 mm^{-1}	
F(000)	1704 e	
Crystal size	0.133 x 0.131 x 0.128 mm^3	
θ range for data collection	1.112 to 34.338°.	
Index ranges	$-17 \le h \le 17$, $-22\le k \le 22$, $-29 \le l \le 29$	
Reflections collected	115922	
Independent reflections	24566 [R_{int} = 0.0510]	
Reflections with I>2σ(I)	20930	
Completeness to θ = 25.242°	100.0 %	
Absorption correction	Gaussian	
Max. and min. transmission	0.48154 and 0.41484	
Refinement method	Full-matrix least-squares on F^2	
Data / restraints / parameters	24566 / 0 / 763	
Goodness-of-fit on F^2	1.020	
Final R indices [I>2σ(I)]	R_1 = 0.0257	wR^2 = 0.0509
R indices (all data)	R_1 = 0.0356	wR^2 = 0.0534
Extinction coefficient	n/a	
Largest diff. peak and hole	2.392 and -1.671 e·Å^{-3}	

Table S62. Bond lengths [Å] and angles [°] of **108**.

Bi(1)-C(1)	2.270(2)	Bi(1)-C(7)	2.262(2)
Bi(1)-C(13)	2.290(2)	S(1)-O(1)	1.4447(16)
S(1)-N(1)	1.545(2)	S(1)-C(2)	1.775(2)
S(1)-C(8)	1.768(2)	F(1)-C(21)	1.356(3)
F(2)-C(21)	1.338(3)	F(3)-C(21)	1.355(2)
N(1)-C(21)	1.383(3)	C(1)-C(2)	1.388(3)
C(1)-C(6)	1.391(3)	C(2)-C(3)	1.394(3)
C(3)-H(3)	0.9500	C(3)-C(4)	1.387(4)
C(4)-H(4)	0.9500	C(4)-C(5)	1.389(4)
C(5)-H(5)	0.9500	C(5)-C(6)	1.389(3)
C(6)-H(6)	0.9500	C(7)-C(8)	1.393(3)
C(7)-C(12)	1.391(3)	C(8)-C(9)	1.391(3)
C(9)-H(9)	0.9500	C(9)-C(10)	1.383(3)
C(10)-H(10)	0.9500	C(10)-C(11)	1.389(3)
C(11)-H(11)	0.9500	C(11)-C(12)	1.394(3)
C(12)-H(12)	0.9500	C(13)-C(14)	1.410(3)
C(13)-C(18)	1.403(4)	C(14)-C(15)	1.393(3)
C(14)-C(19)	1.508(4)	C(15)-H(15)	0.9500
C(15)-C(16)	1.374(4)	C(16)-H(16)	0.9500
C(16)-C(17)	1.384(4)	C(17)-H(17)	0.9500
C(17)-C(18)	1.405(3)	C(18)-C(20)	1.504(3)
C(19)-H(19A)	0.9800	C(19)-H(19B)	0.9800
C(19)-H(19C)	0.9800	C(20)-H(20A)	0.9800
C(20)-H(20B)	0.9800	C(20)-H(20C)	0.9800
Bi(2)-C(31)	2.247(2)	Bi(2)-C(37)	2.269(2)
Bi(2)-C(43)	2.286(2)	S(2)-O(2)	1.4435(19)
S(2)-N(2)	1.544(2)	S(2)-C(32)	1.764(2)
S(2)-C(38)	1.781(2)	F(4)-C(51)	1.340(3)
F(5)-C(51)	1.331(3)	F(6)-C(51)	1.352(3)
N(2)-C(51)	1.382(3)	C(31)-C(32)	1.388(3)
C(31)-C(36)	1.388(3)	C(32)-C(33)	1.389(3)
C(33)-H(33)	0.9500	C(33)-C(34)	1.390(3)
C(34)-H(34)	0.9500	C(34)-C(35)	1.377(4)
C(35)-H(35)	0.9500	C(35)-C(36)	1.390(3)
C(36)-H(36)	0.9500	C(37)-C(38)	1.387(3)

C(37)-C(42)	1.395(3)	C(38)-C(39)	1.392(3)
C(39)-H(39)	0.9500	C(39)-C(40)	1.394(3)
C(40)-H(40)	0.9500	C(40)-C(41)	1.379(4)
C(41)-H(41)	0.9500	C(41)-C(42)	1.390(4)
C(42)-H(42)	0.9500	C(43)-C(44)	1.408(3)
C(43)-C(48)	1.402(3)	C(44)-C(45)	1.398(3)
C(44)-C(49)	1.510(3)	C(45)-H(45)	0.9500
C(45)-C(46)	1.371(4)	C(46)-H(46)	0.9500
C(46)-C(47)	1.383(3)	C(47)-H(47)	0.9500
C(47)-C(48)	1.398(3)	C(48)-C(50)	1.510(3)
C(49)-H(49A)	0.9800	C(49)-H(49B)	0.9800
C(49)-H(49C)	0.9800	C(50)-H(50A)	0.9800
C(50)-H(50B)	0.9800	C(50)-H(50C)	0.9800
Bi(3)-C(61)	2.250(2)	Bi(3)-C(67)	2.251(2)
Bi(3)-C(73)	2.284(2)	S(3)-O(3)	1.4440(17)
S(3)-N(3)	1.546(2)	S(3)-C(62)	1.775(2)
S(3)-C(68)	1.765(2)	F(7)-C(81)	1.353(3)
F(8)-C(81)	1.338(3)	F(9)-C(81)	1.351(3)
N(3)-C(81)	1.378(3)	C(61)-C(62)	1.394(3)
C(61)-C(66)	1.391(3)	C(62)-C(63)	1.386(3)
C(63)-H(63)	0.9500	C(63)-C(64)	1.389(3)
C(64)-H(64)	0.9500	C(64)-C(65)	1.385(4)
C(65)-H(65)	0.9500	C(65)-C(66)	1.389(3)
C(66)-H(66)	0.9500	C(67)-C(68)	1.395(3)
C(67)-C(72)	1.391(3)	C(68)-C(69)	1.387(3)
C(69)-H(69)	0.9500	C(69)-C(70)	1.381(4)
C(70)-H(70)	0.9500	C(70)-C(71)	1.383(4)
C(71)-H(71)	0.9500	C(71)-C(72)	1.391(3)
C(72)-H(72)	0.9500	C(73)-C(74)	1.408(3)
C(73)-C(78)	1.404(3)	C(74)-C(75)	1.389(4)
C(74)-C(79)	1.516(3)	C(75)-H(75)	0.9500
C(75)-C(76)	1.382(4)	C(76)-H(76)	0.9500
C(76)-C(77)	1.384(4)	C(77)-H(77)	0.9500
C(77)-C(78)	1.397(4)	C(78)-C(80)	1.508(3)
C(79)-H(79A)	0.9800	C(79)-H(79B)	0.9800
C(79)-H(79C)	0.9800	C(80)-H(80A)	0.9800

C(80)-H(80B)	0.9800	C(80)-H(80C)	0.9800

C(1)-Bi(1)-C(13)	91.45(8)	C(7)-Bi(1)-C(1)	89.05(8)
C(7)-Bi(1)-C(13)	97.38(8)	O(1)-S(1)-N(1)	120.52(11)
O(1)-S(1)-C(2)	109.16(10)	O(1)-S(1)-C(8)	110.65(11)
N(1)-S(1)-C(2)	109.47(11)	N(1)-S(1)-C(8)	99.86(11)
C(8)-S(1)-C(2)	106.04(10)	C(21)-N(1)-S(1)	120.65(16)
C(2)-C(1)-Bi(1)	122.19(16)	C(2)-C(1)-C(6)	117.4(2)
C(6)-C(1)-Bi(1)	120.32(16)	C(1)-C(2)-S(1)	118.35(17)
C(1)-C(2)-C(3)	123.0(2)	C(3)-C(2)-S(1)	118.59(17)
C(2)-C(3)-H(3)	121.0	C(4)-C(3)-C(2)	118.0(2)
C(4)-C(3)-H(3)	121.0	C(3)-C(4)-H(4)	119.9
C(3)-C(4)-C(5)	120.3(2)	C(5)-C(4)-H(4)	119.9
C(4)-C(5)-H(5)	119.8	C(4)-C(5)-C(6)	120.4(2)
C(6)-C(5)-H(5)	119.8	C(1)-C(6)-H(6)	119.6
C(5)-C(6)-C(1)	120.8(2)	C(5)-C(6)-H(6)	119.6
C(8)-C(7)-Bi(1)	120.60(16)	C(12)-C(7)-Bi(1)	121.91(16)
C(12)-C(7)-C(8)	117.4(2)	C(7)-C(8)-S(1)	120.08(17)
C(9)-C(8)-S(1)	117.04(17)	C(9)-C(8)-C(7)	122.9(2)
C(8)-C(9)-H(9)	120.8	C(10)-C(9)-C(8)	118.4(2)
C(10)-C(9)-H(9)	120.8	C(9)-C(10)-H(10)	119.9
C(9)-C(10)-C(11)	120.2(2)	C(11)-C(10)-H(10)	119.9
C(10)-C(11)-H(11)	119.8	C(10)-C(11)-C(12)	120.4(2)
C(12)-C(11)-H(11)	119.8	C(7)-C(12)-C(11)	120.6(2)
C(7)-C(12)-H(12)	119.7	C(11)-C(12)-H(12)	119.7
C(14)-C(13)-Bi(1)	113.16(17)	C(18)-C(13)-Bi(1)	125.78(15)
C(18)-C(13)-C(14)	120.9(2)	C(13)-C(14)-C(19)	122.9(2)
C(15)-C(14)-C(13)	118.6(2)	C(15)-C(14)-C(19)	118.6(2)
C(14)-C(15)-H(15)	119.3	C(16)-C(15)-C(14)	121.3(2)
C(16)-C(15)-H(15)	119.3	C(15)-C(16)-H(16)	120.0
C(15)-C(16)-C(17)	119.9(2)	C(17)-C(16)-H(16)	120.0
C(16)-C(17)-H(17)	119.4	C(16)-C(17)-C(18)	121.1(2)
C(18)-C(17)-H(17)	119.4	C(13)-C(18)-C(17)	118.1(2)
C(13)-C(18)-C(20)	124.6(2)	C(17)-C(18)-C(20)	117.3(2)
C(14)-C(19)-H(19A)	109.5	C(14)-C(19)-H(19B)	109.5
C(14)-C(19)-H(19C)	109.5	H(19A)-C(19)-H(19B)	109.5

H(19A)-C(19)-H(19C)	109.5	H(19B)-C(19)-H(19C)	109.5
C(18)-C(20)-H(20A)	109.5	C(18)-C(20)-H(20B)	109.5
C(18)-C(20)-H(20C)	109.5	H(20A)-C(20)-H(20B)	109.5
H(20A)-C(20)-H(20C)	109.5	H(20B)-C(20)-H(20C)	109.5
F(1)-C(21)-N(1)	115.9(2)	F(2)-C(21)-F(1)	106.27(18)
F(2)-C(21)-F(3)	106.08(19)	F(2)-C(21)-N(1)	109.79(18)
F(3)-C(21)-F(1)	104.70(17)	F(3)-C(21)-N(1)	113.43(19)
C(31)-Bi(2)-C(37)	88.89(8)	C(31)-Bi(2)-C(43)	91.42(8)
C(37)-Bi(2)-C(43)	95.52(8)	O(2)-S(2)-N(2)	120.46(11)
O(2)-S(2)-C(32)	110.83(11)	O(2)-S(2)-C(38)	108.64(11)
N(2)-S(2)-C(32)	100.63(10)	N(2)-S(2)-C(38)	108.98(11)
C(32)-S(2)-C(38)	106.31(10)	C(51)-N(2)-S(2)	120.49(17)
C(32)-C(31)-Bi(2)	121.71(15)	C(36)-C(31)-Bi(2)	120.79(18)
C(36)-C(31)-C(32)	117.2(2)	C(31)-C(32)-S(2)	119.39(17)
C(31)-C(32)-C(33)	123.3(2)	C(33)-C(32)-S(2)	117.35(18)
C(32)-C(33)-H(33)	121.1	C(32)-C(33)-C(34)	117.8(2)
C(34)-C(33)-H(33)	121.1	C(33)-C(34)-H(34)	119.9
C(35)-C(34)-C(33)	120.3(2)	C(35)-C(34)-H(34)	119.9
C(34)-C(35)-H(35)	119.7	C(34)-C(35)-C(36)	120.6(2)
C(36)-C(35)-H(35)	119.7	C(31)-C(36)-C(35)	120.7(2)
C(31)-C(36)-H(36)	119.6	C(35)-C(36)-H(36)	119.6
C(38)-C(37)-Bi(2)	121.83(15)	C(38)-C(37)-C(42)	117.2(2)
C(42)-C(37)-Bi(2)	121.00(17)	C(37)-C(38)-S(2)	118.83(17)
C(37)-C(38)-C(39)	123.2(2)	C(39)-C(38)-S(2)	117.90(18)
C(38)-C(39)-H(39)	121.0	C(38)-C(39)-C(40)	118.0(2)
C(40)-C(39)-H(39)	121.0	C(39)-C(40)-H(40)	119.9
C(41)-C(40)-C(39)	120.2(2)	C(41)-C(40)-H(40)	119.9
C(40)-C(41)-H(41)	119.7	C(40)-C(41)-C(42)	120.6(2)
C(42)-C(41)-H(41)	119.7	C(37)-C(42)-H(42)	119.6
C(41)-C(42)-C(37)	120.8(2)	C(41)-C(42)-H(42)	119.6
C(44)-C(43)-Bi(2)	124.85(16)	C(48)-C(43)-Bi(2)	114.82(15)
C(48)-C(43)-C(44)	120.26(19)	C(43)-C(44)-C(49)	125.3(2)
C(45)-C(44)-C(43)	118.5(2)	C(45)-C(44)-C(49)	116.2(2)
C(44)-C(45)-H(45)	119.3	C(46)-C(45)-C(44)	121.4(2)
C(46)-C(45)-H(45)	119.3	C(45)-C(46)-H(46)	119.9
C(45)-C(46)-C(47)	120.1(2)	C(47)-C(46)-H(46)	119.9

C(46)-C(47)-H(47)	119.7	C(46)-C(47)-C(48)	120.5(2)
C(48)-C(47)-H(47)	119.7	C(43)-C(48)-C(50)	122.9(2)
C(47)-C(48)-C(43)	119.2(2)	C(47)-C(48)-C(50)	117.9(2)
C(44)-C(49)-H(49A)	109.5	C(44)-C(49)-H(49B)	109.5
C(44)-C(49)-H(49C)	109.5	H(49A)-C(49)-H(49B)	109.5
H(49A)-C(49)-H(49C)	109.5	H(49B)-C(49)-H(49C)	109.5
C(48)-C(50)-H(50A)	109.5	C(48)-C(50)-H(50B)	109.5
C(48)-C(50)-H(50C)	109.5	H(50A)-C(50)-H(50B)	109.5
H(50A)-C(50)-H(50C)	109.5	H(50B)-C(50)-H(50C)	109.5
F(4)-C(51)-F(6)	103.6(2)	F(4)-C(51)-N(2)	113.7(2)
F(5)-C(51)-F(4)	106.8(2)	F(5)-C(51)-F(6)	105.1(2)
F(5)-C(51)-N(2)	116.9(2)	F(6)-C(51)-N(2)	109.6(2)
C(61)-Bi(3)-C(67)	88.63(8)	C(61)-Bi(3)-C(73)	97.63(8)
C(67)-Bi(3)-C(73)	94.23(8)	O(3)-S(3)-N(3)	120.77(11)
O(3)-S(3)-C(62)	108.72(10)	O(3)-S(3)-C(68)	110.84(10)
N(3)-S(3)-C(62)	108.84(10)	N(3)-S(3)-C(68)	100.31(11)
C(68)-S(3)-C(62)	106.33(11)	C(81)-N(3)-S(3)	120.60(17)
C(62)-C(61)-Bi(3)	121.80(15)	C(66)-C(61)-Bi(3)	121.06(16)
C(66)-C(61)-C(62)	117.1(2)	C(61)-C(62)-S(3)	118.73(16)
C(63)-C(62)-S(3)	117.86(18)	C(63)-C(62)-C(61)	123.2(2)
C(62)-C(63)-H(63)	120.8	C(62)-C(63)-C(64)	118.5(2)
C(64)-C(63)-H(63)	120.8	C(63)-C(64)-H(64)	120.3
C(65)-C(64)-C(63)	119.4(2)	C(65)-C(64)-H(64)	120.3
C(64)-C(65)-H(65)	119.3	C(64)-C(65)-C(66)	121.3(2)
C(66)-C(65)-H(65)	119.3	C(61)-C(66)-H(66)	119.8
C(65)-C(66)-C(61)	120.4(2)	C(65)-C(66)-H(66)	119.8
C(68)-C(67)-Bi(3)	121.28(15)	C(72)-C(67)-Bi(3)	121.67(17)
C(72)-C(67)-C(68)	117.0(2)	C(67)-C(68)-S(3)	119.38(16)
C(69)-C(68)-S(3)	117.34(18)	C(69)-C(68)-C(67)	123.2(2)
C(68)-C(69)-H(69)	121.0	C(70)-C(69)-C(68)	118.1(2)
C(70)-C(69)-H(69)	121.0	C(69)-C(70)-H(70)	119.8
C(69)-C(70)-C(71)	120.4(2)	C(71)-C(70)-H(70)	119.8
C(70)-C(71)-H(71)	119.7	C(70)-C(71)-C(72)	120.6(2)
C(72)-C(71)-H(71)	119.7	C(67)-C(72)-H(72)	119.7
C(71)-C(72)-C(67)	120.6(2)	C(71)-C(72)-H(72)	119.7
C(74)-C(73)-Bi(3)	113.78(16)	C(78)-C(73)-Bi(3)	126.55(17)

C(78)-C(73)-C(74)	119.7(2)	C(73)-C(74)-C(79)	122.3(2)
C(75)-C(74)-C(73)	119.6(2)	C(75)-C(74)-C(79)	118.1(2)
C(74)-C(75)-H(75)	119.6	C(76)-C(75)-C(74)	120.7(2)
C(76)-C(75)-H(75)	119.6	C(75)-C(76)-H(76)	120.1
C(75)-C(76)-C(77)	119.9(3)	C(77)-C(76)-H(76)	120.1
C(76)-C(77)-H(77)	119.5	C(76)-C(77)-C(78)	120.9(3)
C(78)-C(77)-H(77)	119.5	C(73)-C(78)-C(80)	123.6(2)
C(77)-C(78)-C(73)	119.2(2)	C(77)-C(78)-C(80)	117.2(2)
C(74)-C(79)-H(79A)	109.5	C(74)-C(79)-H(79B)	109.5
C(74)-C(79)-H(79C)	109.5	H(79A)-C(79)-H(79B)	109.5
H(79A)-C(79)-H(79C)	109.5	H(79B)-C(79)-H(79C)	109.5
C(78)-C(80)-H(80A)	109.5	C(78)-C(80)-H(80B)	109.5
C(78)-C(80)-H(80C)	109.5	H(80A)-C(80)-H(80B)	109.5
H(80A)-C(80)-H(80C)	109.5	H(80B)-C(80)-H(80C)	109.5
F(7)-C(81)-N(3)	113.9(2)	F(8)-C(81)-F(7)	105.8(2)
F(8)-C(81)-F(9)	105.9(2)	F(8)-C(81)-N(3)	109.5(2)
F(9)-C(81)-F(7)	104.66(19)	F(9)-C(81)-N(3)	116.3(2)

8.19 Single crystal structure analysis of **110**

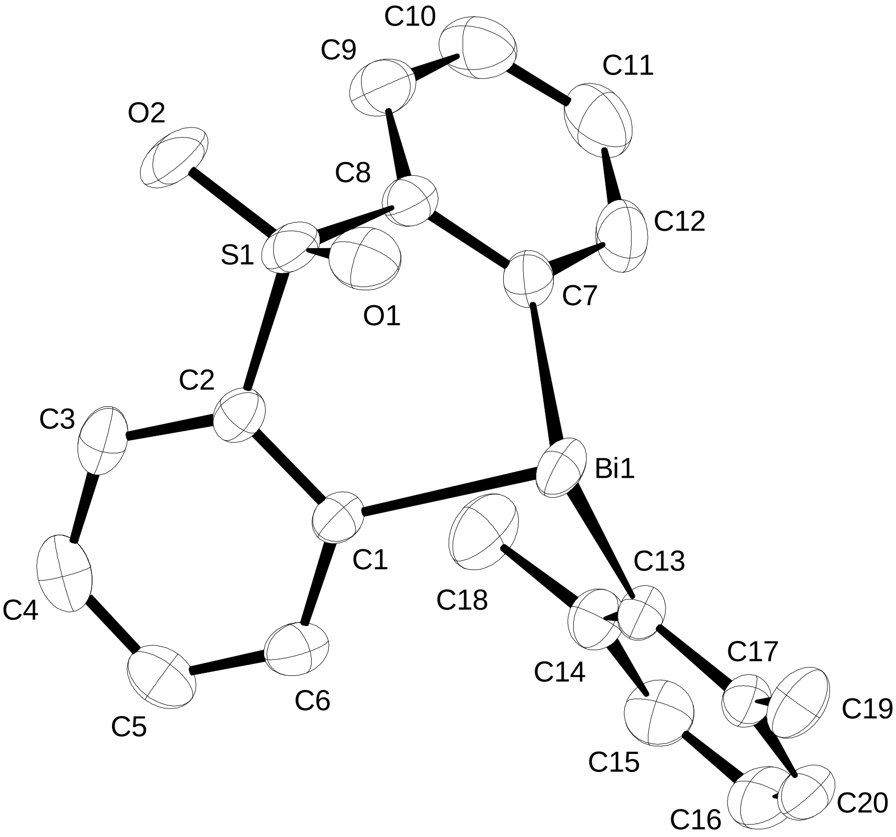

Figure S115. The molecular structure of complex **110**. H atoms have been removed for clarity.

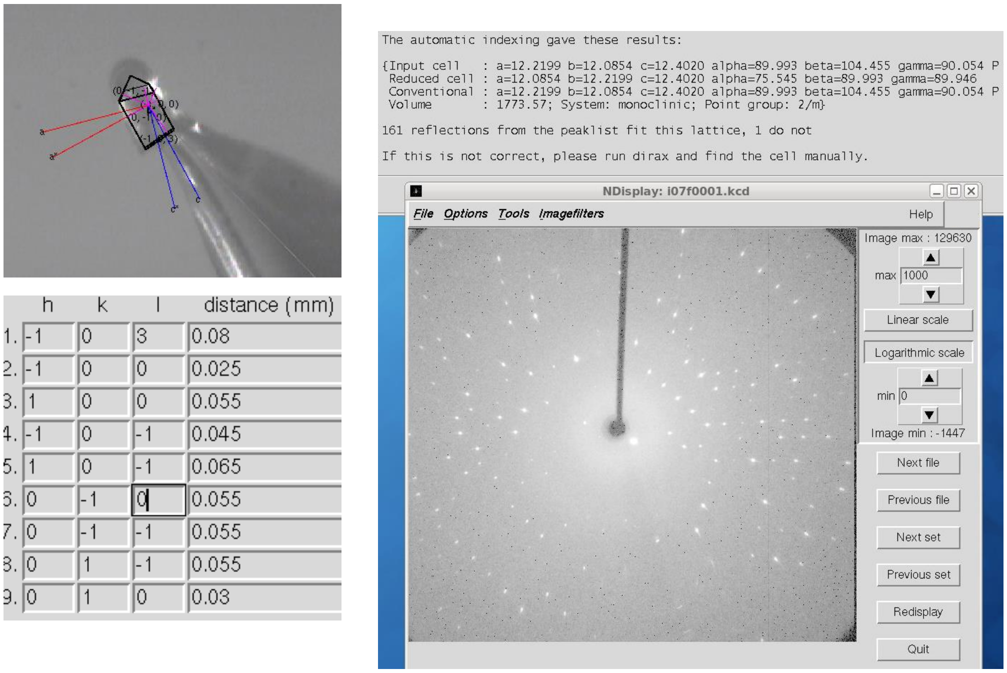

Figure S116. Crystal faces and unit cell determination of **110**.

INTENSITY STATISTICS FOR DATASET

Resolution	#Data	#Theory	%Complete	Redundancy	Mean I	Mean I/s	Rmerge	Rsigma
Inf - 2.69	107	115	93.0	15.76	160.64	132.82	0.0305	0.0068
2.69 - 1.80	244	244	100.0	13.01	131.41	111.80	0.0247	0.0068
1.80 - 1.42	353	353	100.0	11.69	89.41	90.02	0.0213	0.0078
1.42 - 1.23	362	362	100.0	11.06	59.12	74.59	0.0230	0.0092
1.23 - 1.12	351	351	100.0	10.63	43.09	64.92	0.0248	0.0109
1.12 - 1.04	343	343	100.0	10.29	38.44	56.62	0.0274	0.0121
1.04 - 0.97	399	399	100.0	9.84	27.75	46.68	0.0321	0.0150
0.97 - 0.92	364	364	100.0	9.28	22.21	40.45	0.0371	0.0183
0.92 - 0.88	351	351	100.0	8.81	17.74	32.96	0.0444	0.0224
0.88 - 0.85	319	319	100.0	8.63	16.75	31.22	0.0486	0.0241
0.85 - 0.82	346	346	100.0	8.10	13.32	26.02	0.0577	0.0304
0.82 - 0.79	423	423	100.0	7.72	11.41	21.52	0.0698	0.0362
0.79 - 0.77	304	304	100.0	7.37	10.36	19.74	0.0758	0.0407
0.77 - 0.75	342	342	100.0	6.93	8.30	16.31	0.0943	0.0524
0.75 - 0.73	398	398	100.0	6.83	8.21	15.09	0.1030	0.0552
0.73 - 0.71	427	427	100.0	6.41	6.22	11.84	0.1279	0.0741
0.71 - 0.70	242	242	100.0	6.47	5.67	10.87	0.1427	0.0806
0.70 - 0.68	499	499	100.0	6.04	4.94	8.95	0.1637	0.1014
0.68 - 0.67	280	280	100.0	5.85	4.55	7.85	0.1784	0.1168
0.67 - 0.66	286	286	100.0	5.81	4.18	6.82	0.2106	0.1363
0.66 - 0.65	284	284	100.0	5.67	3.64	5.61	0.2395	0.1725
0.75 - 0.65	2416	2416	100.0	6.19	5.49	9.89	0.1480	0.0926
Inf - 0.65	7024	7032	99.9	8.41	27.04	35.73	0.0345	0.0185

Complete .cif-data of the compound are available under the CCDC number **CCDC-2125776.**

Table S63. Crystal data and structure refinement of **110**.

Identification code	13419sadabs	
Empirical formula	$C_{20} H_{17} Bi O_2 S$	
Color	colourless	
Formula weight	530.38 g·mol^{-1}	
Temperature	200(2) K	
Wavelength	0.71073 Å	
Crystal system	Monoclinic	
Space group	$P2_1$/n, (no. 14)	
Unit cell dimensions	a = 12.2318(14) Å	α= 90°.
	b = 12.0932(15) Å	β= 104.495(10)°.
	c = 12.4071(15) Å	γ = 90°.
Volume	1776.9(4) Å^3	
Z	4	
Density (calculated)	1.983 Mg·m^{-3}	
Absorption coefficient	10.049 mm^{-1}	
F(000)	1008 e	
Crystal size	0.135 x 0.085 x 0.075 mm^3	
θ range for data collection	2.685 to 33.089°.	
Index ranges	-18 ≤ h ≤ 18, -18≤ k ≤ 18, -19 ≤ l ≤ 19	
Reflections collected	57888	
Independent reflections	6744 [R_{int} = 0.0352]	
Reflections with I>2σ(I)	5785	
Completeness to θ = 25.242°	99.8 %	
Absorption correction	Gaussian	
Max. and min. transmission	0.50263 and 0.32091	
Refinement method	Full-matrix least-squares on F^2	
Data / restraints / parameters	6744 / 0 / 219	
Goodness-of-fit on F^2	1.181	
Final R indices [I>2σ(I)]	R_1 = 0.0250	wR^2 = 0.0423
R indices (all data)	R_1 = 0.0351	wR^2 = 0.0447
Extinction coefficient	n/a	
Largest diff. peak and hole	0.441 and -0.969 e·Å^{-3}	

Table S64. Bond lengths [Å] and angles [°] of **110**.

Bi(1)-C(1)	2.267(2)	Bi(1)-C(7)	2.275(2)
Bi(1)-C(13)	2.288(2)	S(1)-O(1)	1.450(2)
S(1)-O(2)	1.4408(18)	S(1)-C(2)	1.767(2)
S(1)-C(8)	1.770(2)	C(1)-C(2)	1.395(3)
C(1)-C(6)	1.381(3)	C(2)-C(3)	1.389(3)
C(3)-H(3)	0.9500	C(3)-C(4)	1.388(4)
C(4)-H(4)	0.9500	C(4)-C(5)	1.386(4)
C(5)-H(5)	0.9500	C(5)-C(6)	1.382(4)
C(6)-H(6)	0.9500	C(7)-C(8)	1.396(3)
C(7)-C(12)	1.389(3)	C(8)-C(9)	1.386(3)
C(9)-H(9)	0.9500	C(9)-C(10)	1.382(4)
C(10)-H(10)	0.9500	C(10)-C(11)	1.382(4)
C(11)-H(11)	0.9500	C(11)-C(12)	1.388(4)
C(12)-H(12)	0.9500	C(13)-C(14)	1.404(3)
C(13)-C(17)	1.406(3)	C(14)-C(15)	1.401(4)
C(14)-C(18)	1.509(4)	C(15)-H(15)	0.9500
C(15)-C(16)	1.374(4)	C(16)-H(16)	0.9500
C(16)-C(20)	1.379(4)	C(17)-C(19)	1.514(3)
C(17)-C(20)	1.396(3)	C(18)-H(18A)	0.9800
C(18)-H(18B)	0.9800	C(18)-H(18C)	0.9800
C(19)-H(19A)	0.9800	C(19)-H(19B)	0.9800
C(19)-H(19C)	0.9800	C(20)-H(20)	0.9500
C(1)-Bi(1)-C(7)	87.09(8)	C(1)-Bi(1)-C(13)	94.87(8)
C(7)-Bi(1)-C(13)	98.63(8)	O(1)-S(1)-C(2)	106.87(11)
O(1)-S(1)-C(8)	107.38(12)	O(2)-S(1)-O(1)	118.54(12)
O(2)-S(1)-C(2)	109.07(12)	O(2)-S(1)-C(8)	109.50(12)
C(2)-S(1)-C(8)	104.57(11)	C(2)-C(1)-Bi(1)	120.79(16)
C(6)-C(1)-Bi(1)	121.41(16)	C(6)-C(1)-C(2)	117.7(2)
C(1)-C(2)-S(1)	118.02(17)	C(3)-C(2)-S(1)	119.77(18)
C(3)-C(2)-C(1)	122.2(2)	C(2)-C(3)-H(3)	120.7
C(4)-C(3)-C(2)	118.6(2)	C(4)-C(3)-H(3)	120.7
C(3)-C(4)-H(4)	120.1	C(5)-C(4)-C(3)	119.9(2)
C(5)-C(4)-H(4)	120.1	C(4)-C(5)-H(5)	119.8
C(6)-C(5)-C(4)	120.5(2)	C(6)-C(5)-H(5)	119.8

C(1)-C(6)-C(5)	121.1(2)	C(1)-C(6)-H(6)	119.5
C(5)-C(6)-H(6)	119.5	C(8)-C(7)-Bi(1)	120.20(17)
C(12)-C(7)-Bi(1)	123.08(18)	C(12)-C(7)-C(8)	116.7(2)
C(7)-C(8)-S(1)	118.51(18)	C(9)-C(8)-S(1)	118.87(18)
C(9)-C(8)-C(7)	122.6(2)	C(8)-C(9)-H(9)	120.5
C(10)-C(9)-C(8)	119.0(2)	C(10)-C(9)-H(9)	120.5
C(9)-C(10)-H(10)	120.0	C(9)-C(10)-C(11)	120.0(3)
C(11)-C(10)-H(10)	120.0	C(10)-C(11)-H(11)	120.0
C(10)-C(11)-C(12)	120.1(3)	C(12)-C(11)-H(11)	120.0
C(7)-C(12)-H(12)	119.2	C(11)-C(12)-C(7)	121.6(3)
C(11)-C(12)-H(12)	119.2	C(14)-C(13)-Bi(1)	126.09(16)
C(14)-C(13)-C(17)	119.6(2)	C(17)-C(13)-Bi(1)	114.34(16)
C(13)-C(14)-C(18)	124.4(2)	C(15)-C(14)-C(13)	118.5(2)
C(15)-C(14)-C(18)	117.1(2)	C(14)-C(15)-H(15)	119.1
C(16)-C(15)-C(14)	121.8(3)	C(16)-C(15)-H(15)	119.1
C(15)-C(16)-H(16)	120.1	C(15)-C(16)-C(20)	119.7(2)
C(20)-C(16)-H(16)	120.1	C(13)-C(17)-C(19)	122.8(2)
C(20)-C(17)-C(13)	120.0(2)	C(20)-C(17)-C(19)	117.3(2)
C(14)-C(18)-H(18A)	109.5	C(14)-C(18)-H(18B)	109.5
C(14)-C(18)-H(18C)	109.5	H(18A)-C(18)-H(18B)	109.5
H(18A)-C(18)-H(18C)	109.5	H(18B)-C(18)-H(18C)	109.5
C(17)-C(19)-H(19A)	109.5	C(17)-C(19)-H(19B)	109.5
C(17)-C(19)-H(19C)	109.5	H(19A)-C(19)-H(19B)	109.5
H(19A)-C(19)-H(19C)	109.5	H(19B)-C(19)-H(19C)	109.5
C(16)-C(20)-C(17)	120.4(2)	C(16)-C(20)-H(20)	119.8
C(17)-C(20)-H(20)	119.8		

8.20 Single crystal structure analysis of **146**

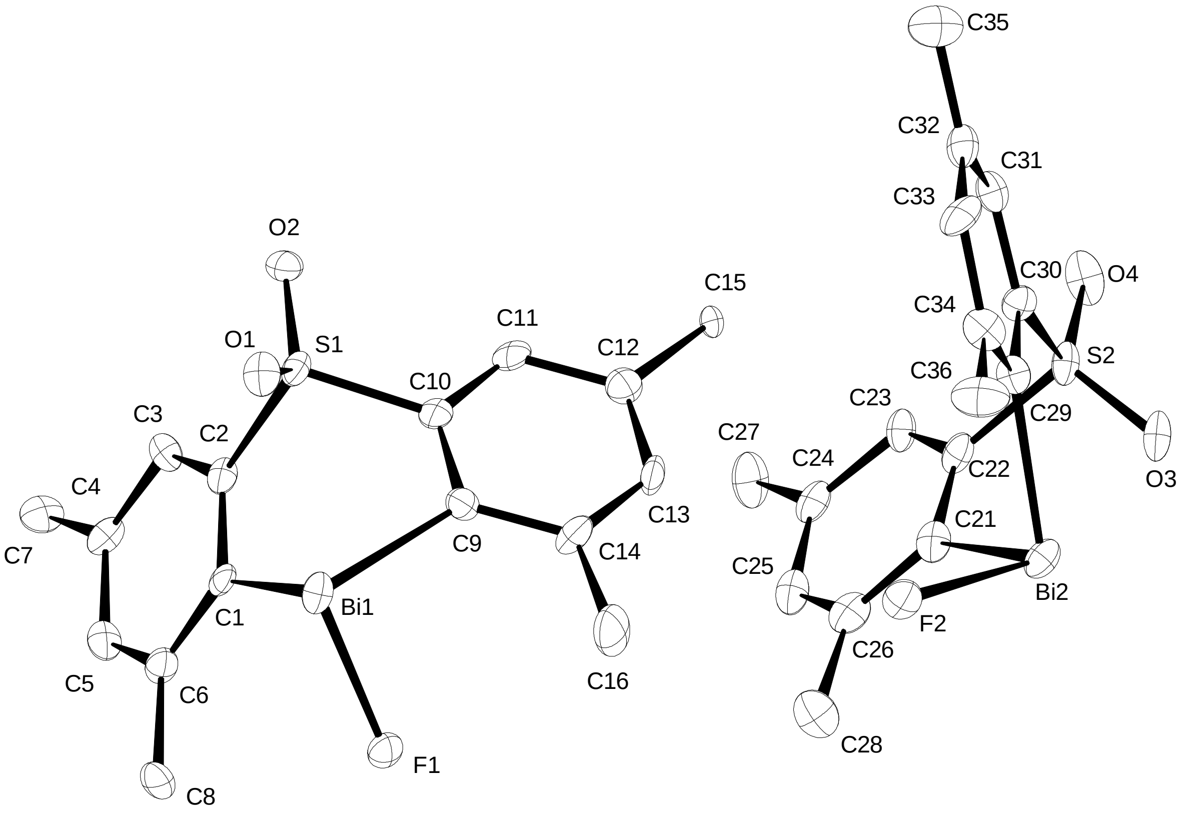

Figure S117. The molecular structure of complex **146**. H atoms have been removed for clarity.

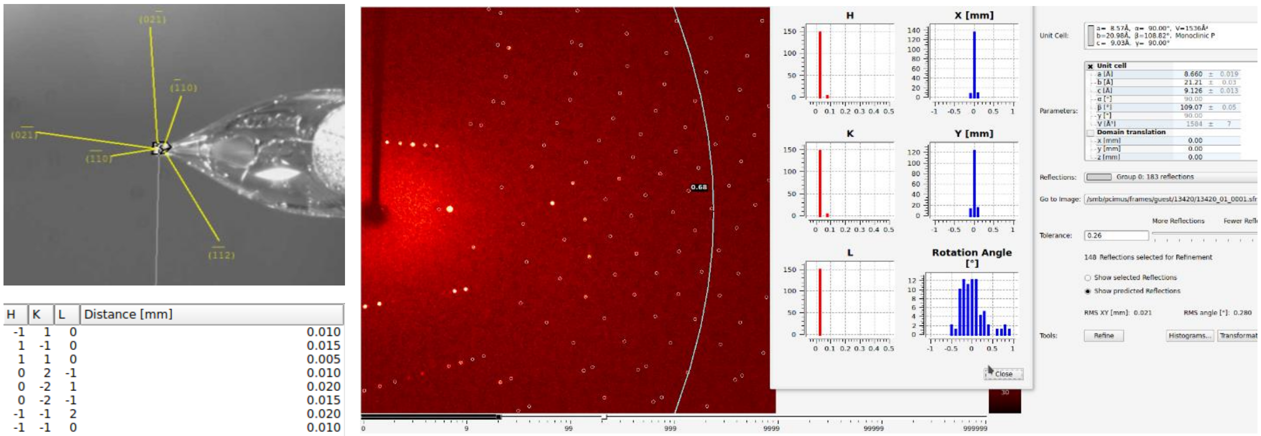

Figure S118. Crystal faces and unit cell determination of **146**.

INTENSITY STATISTICS FOR DATASET

Resolution	#Data	#Theory	%Complete	Redundancy	Mean I	Mean I/s	Rmerge	Rsigma
Inf - 2.84	77	77	100.0	14.84	117.92	71.87	0.0358	0.0119
2.84 - 1.90	181	181	100.0	16.40	90.36	65.15	0.0365	0.0127
1.90 - 1.49	254	254	100.0	16.60	64.21	54.42	0.0440	0.0151
1.49 - 1.30	260	260	100.0	16.60	47.52	46.73	0.0590	0.0183
1.30 - 1.18	261	261	100.0	15.94	40.75	38.93	0.0703	0.0216
1.18 - 1.09	269	269	100.0	14.58	33.47	31.01	0.0873	0.0273
1.09 - 1.03	233	233	100.0	11.58	31.92	25.66	0.0957	0.0340
1.03 - 0.97	305	305	100.0	9.07	26.92	19.13	0.1121	0.0460
0.97 - 0.93	237	237	100.0	8.06	18.89	13.57	0.1465	0.0641
0.93 - 0.89	297	297	100.0	7.07	22.15	13.81	0.1410	0.0645
0.89 - 0.86	249	249	100.0	6.90	16.79	11.30	0.1713	0.0808
0.86 - 0.83	294	294	100.0	6.31	14.90	9.03	0.1900	0.0998
0.83 - 0.81	216	216	100.0	6.16	14.51	8.68	0.2063	0.1071
0.81 - 0.79	231	231	100.0	6.25	12.11	7.48	0.2252	0.1233
0.79 - 0.77	272	272	100.0	5.76	12.11	6.92	0.2431	0.1378
0.77 - 0.75	293	293	100.0	5.76	9.21	5.31	0.2778	0.1784
0.75 - 0.74	173	173	100.0	5.64	10.44	5.73	0.2715	0.1633
0.74 - 0.72	343	343	100.0	5.43	9.00	4.95	0.3119	0.1951
0.72 - 0.71	183	183	100.0	5.32	7.45	3.96	0.3718	0.2451
0.71 - 0.69	401	401	100.0	5.08	7.23	3.67	0.3734	0.2637
0.69 - 0.68	92	129	71.3	2.40	5.33	2.31	0.4045	0.4854
0.78 - 0.68	1624	1661	97.8	5.20	8.55	4.64	0.3127	0.2122
Inf - 0.68	5121	5158	99.3	8.91	25.50	19.24	0.0832	0.0554

Refined as an inversion twin (TWIN -1 0 0 0 -1 0 0 0 -1, BASF 0.23763). A resolution cutoff (SHEL 99 0.7) was applied to exclude high-resolution reflections with poorly determined intensities.

Complete .cif-data of the compound are available under the CCDC number **CCDC-2125774.**

Table S65. Crystal data and structure refinement of **146**.

Identification code	13420sadabs	
Empirical formula	C_{16} H_{16} Bi F O_2 S	
Color	colourless	
Formula weight	500.33 g·mol^{-1}	
Temperature	100(2) K	
Wavelength	0.71073 Å	
Crystal system	Monoclinic	
Space group	$P2_1$, (no. 4)	
Unit cell dimensions	a = 8.5609(4) Å	α= 90°.
	b = 21.0051(9) Å	β= 108.693(2)°.
	c = 9.0247(4) Å	γ = 90°.
Volume	1537.24(12) Å^3	
Z	4	
Density (calculated)	2.162 Mg·m^{-3}	
Absorption coefficient	11.616 mm^{-1}	
F(000)	944 e	
Crystal size	0.054 x 0.044 x 0.021 mm^3	
θ range for data collection	1.939 to 30.501°.	
Index ranges	-12 ≤ h ≤ 12, -30≤ k ≤ 30, -12 ≤ l ≤ 12	
Reflections collected	44525	
Independent reflections	9365 [R_{int} = 0.0758]	
Reflections with I>2σ(I)	7501	
Completeness to θ = 25.242°	100.0 %	
Absorption correction	Gaussian	
Max. and min. transmission	0.86133 and 0.69535	
Refinement method	Full-matrix least-squares on F^2	
Data / restraints / parameters	9365 / 1 / 388	
Goodness-of-fit on F^2	1.019	
Final R indices [I>2σ(I)]	R_1 = 0.0426	wR^2 = 0.0648
R indices (all data)	R_1 = 0.0665	wR^2 = 0.0711
Absolute structure parameter	0.235(10)	
Extinction coefficient	n/a	
Largest diff. peak and hole	1.426 and -1.705 e·Å^{-3}	

Table S66. Bond lengths [Å] and angles [°] of **146**.

Bi(1)-F(1)	2.126(6)	Bi(1)-O(1)	2.747(8)
Bi(1)-C(9)	2.274(11)	Bi(1)-C(1)	2.286(11)
Bi(2)-F(2)	2.114(6)	Bi(2)-O(3)	2.729(8)
Bi(2)-C(25)	2.278(11)	Bi(2)-C(17)	2.276(12)
S(2)-O(3)	1.458(8)	S(2)-O(4)	1.448(9)
S(2)-C(18)	1.762(12)	S(2)-C(26)	1.761(11)
S(1)-O(1)	1.450(8)	S(1)-O(2)	1.428(8)
S(1)-C(2)	1.770(10)	S(1)-C(10)	1.766(11)
C(9)-C(14)	1.396(16)	C(9)-C(10)	1.382(16)
C(4)-C(3)	1.378(14)	C(4)-C(5)	1.390(15)
C(4)-C(7)	1.505(15)	C(18)-C(19)	1.381(16)
C(18)-C(17)	1.401(16)	C(6)-C(5)	1.406(17)
C(6)-C(8)	1.507(16)	C(6)-C(1)	1.402(16)
C(3)-H(3)	0.9500	C(3)-C(2)	1.388(15)
C(21)-H(21)	0.9500	C(21)-C(22)	1.397(17)
C(21)-C(20)	1.395(17)	C(5)-H(5)	0.9500
C(2)-C(1)	1.394(14)	C(7)-H(7A)	0.9800
C(7)-H(7B)	0.9800	C(7)-H(7C)	0.9800
C(27)-H(27)	0.9500	C(27)-C(28)	1.419(15)
C(27)-C(26)	1.373(15)	C(19)-H(19)	0.9500
C(19)-C(20)	1.398(16)	C(15)-H(15A)	0.9800
C(15)-H(15B)	0.9800	C(15)-H(15C)	0.9800
C(15)-C(12)	1.537(16)	C(8)-H(8A)	0.9800
C(8)-H(8B)	0.9800	C(8)-H(8C)	0.9800
C(25)-C(30)	1.378(16)	C(25)-C(26)	1.399(16)
C(30)-C(29)	1.390(17)	C(30)-C(32)	1.531(18)
C(24)-H(24A)	0.9800	C(24)-H(24B)	0.9800
C(24)-H(24C)	0.9800	C(24)-C(22)	1.483(17)
C(11)-H(11)	0.9500	C(11)-C(12)	1.399(16)
C(11)-C(10)	1.380(16)	C(28)-C(29)	1.401(17)
C(28)-C(31)	1.495(16)	C(22)-C(17)	1.409(16)
C(14)-C(16)	1.519(17)	C(14)-C(13)	1.394(16)
C(29)-H(29)	0.9500	C(12)-C(13)	1.394(17)
C(32)-H(32A)	0.9800	C(32)-H(32B)	0.9800
C(32)-H(32C)	0.9800	C(16)-H(16A)	0.9800

C(16)-H(16B)	0.9800	C(16)-H(16C)	0.9800
C(20)-C(23)	1.480(17)	C(31)-H(31A)	0.9800
C(31)-H(31B)	0.9800	C(31)-H(31C)	0.9800
C(13)-H(13)	0.9500	C(23)-H(23A)	0.9800
C(23)-H(23B)	0.9800	C(23)-H(23C)	0.9800

F(1)-Bi(1)-O(1)	155.8(2)	F(1)-Bi(1)-C(9)	91.0(3)
F(1)-Bi(1)-C(1)	92.1(3)	C(9)-Bi(1)-O(1)	70.9(3)
C(9)-Bi(1)-C(1)	88.4(4)	C(1)-Bi(1)-O(1)	72.0(3)
F(2)-Bi(2)-O(3)	154.8(2)	F(2)-Bi(2)-C(25)	91.3(4)
F(2)-Bi(2)-C(17)	90.3(3)	C(25)-Bi(2)-O(3)	72.3(3)
C(17)-Bi(2)-O(3)	70.7(3)	C(17)-Bi(2)-C(25)	88.3(4)
O(3)-S(2)-C(18)	105.2(5)	O(3)-S(2)-C(26)	105.2(5)
O(4)-S(2)-O(3)	118.9(5)	O(4)-S(2)-C(18)	110.4(5)
O(4)-S(2)-C(26)	110.9(5)	C(26)-S(2)-C(18)	105.3(6)
O(1)-S(1)-C(2)	105.5(5)	O(1)-S(1)-C(10)	105.1(5)
O(2)-S(1)-O(1)	119.0(5)	O(2)-S(1)-C(2)	110.7(5)
O(2)-S(1)-C(10)	110.9(5)	C(10)-S(1)-C(2)	104.6(6)
S(1)-O(1)-Bi(1)	96.2(4)	S(2)-O(3)-Bi(2)	96.6(4)
C(14)-C(9)-Bi(1)	126.4(8)	C(10)-C(9)-Bi(1)	116.3(8)
C(10)-C(9)-C(14)	117.2(10)	C(3)-C(4)-C(5)	119.0(10)
C(3)-C(4)-C(7)	120.3(10)	C(5)-C(4)-C(7)	120.8(10)
C(19)-C(18)-S(2)	120.1(9)	C(19)-C(18)-C(17)	123.8(11)
C(17)-C(18)-S(2)	116.1(9)	C(5)-C(6)-C(8)	120.9(11)
C(1)-C(6)-C(5)	117.9(10)	C(1)-C(6)-C(8)	121.1(11)
C(4)-C(3)-H(3)	120.9	C(4)-C(3)-C(2)	118.1(10)
C(2)-C(3)-H(3)	120.9	C(22)-C(21)-H(21)	118.0
C(20)-C(21)-H(21)	118.0	C(20)-C(21)-C(22)	123.9(11)
C(4)-C(5)-C(6)	123.2(10)	C(4)-C(5)-H(5)	118.4
C(6)-C(5)-H(5)	118.4	C(3)-C(2)-S(1)	119.0(8)
C(3)-C(2)-C(1)	124.2(10)	C(1)-C(2)-S(1)	116.7(8)
C(4)-C(7)-H(7A)	109.5	C(4)-C(7)-H(7B)	109.5
C(4)-C(7)-H(7C)	109.5	H(7A)-C(7)-H(7B)	109.5
H(7A)-C(7)-H(7C)	109.5	H(7B)-C(7)-H(7C)	109.5
C(28)-C(27)-H(27)	120.6	C(26)-C(27)-H(27)	120.6
C(26)-C(27)-C(28)	118.7(11)	C(18)-C(19)-H(19)	121.0

C(18)-C(19)-C(20)	118.0(11)	C(20)-C(19)-H(19)	121.0
H(15A)-C(15)-H(15B)	109.5	H(15A)-C(15)-H(15C)	109.5
H(15B)-C(15)-H(15C)	109.5	C(12)-C(15)-H(15A)	109.5
C(12)-C(15)-H(15B)	109.5	C(12)-C(15)-H(15C)	109.5
C(6)-C(8)-H(8A)	109.5	C(6)-C(8)-H(8B)	109.5
C(6)-C(8)-H(8C)	109.5	H(8A)-C(8)-H(8B)	109.5
H(8A)-C(8)-H(8C)	109.5	H(8B)-C(8)-H(8C)	109.5
C(30)-C(25)-Bi(2)	126.1(9)	C(30)-C(25)-C(26)	117.2(10)
C(26)-C(25)-Bi(2)	116.7(8)	C(25)-C(30)-C(29)	119.0(12)
C(25)-C(30)-C(32)	122.2(11)	C(29)-C(30)-C(32)	118.8(11)
H(24A)-C(24)-H(24B)	109.5	H(24A)-C(24)-H(24C)	109.5
H(24B)-C(24)-H(24C)	109.5	C(22)-C(24)-H(24A)	109.5
C(22)-C(24)-H(24B)	109.5	C(22)-C(24)-H(24C)	109.5
C(12)-C(11)-H(11)	120.8	C(10)-C(11)-H(11)	120.8
C(10)-C(11)-C(12)	118.4(12)	C(27)-C(28)-C(31)	120.2(10)
C(29)-C(28)-C(27)	115.9(10)	C(29)-C(28)-C(31)	123.9(10)
C(21)-C(22)-C(24)	120.1(11)	C(21)-C(22)-C(17)	117.2(11)
C(17)-C(22)-C(24)	122.7(11)	C(9)-C(14)-C(16)	120.8(11)
C(13)-C(14)-C(9)	119.5(11)	C(13)-C(14)-C(16)	119.6(11)
C(27)-C(26)-S(2)	118.9(9)	C(27)-C(26)-C(25)	124.6(10)
C(25)-C(26)-S(2)	116.5(8)	C(6)-C(1)-Bi(1)	125.9(8)
C(2)-C(1)-Bi(1)	116.5(7)	C(2)-C(1)-C(6)	117.6(10)
C(30)-C(29)-C(28)	124.5(12)	C(30)-C(29)-H(29)	117.8
C(28)-C(29)-H(29)	117.8	C(11)-C(12)-C(15)	120.6(11)
C(13)-C(12)-C(15)	121.1(11)	C(13)-C(12)-C(11)	118.2(11)
C(30)-C(32)-H(32A)	109.5	C(30)-C(32)-H(32B)	109.5
C(30)-C(32)-H(32C)	109.5	H(32A)-C(32)-H(32B)	109.5
H(32A)-C(32)-H(32C)	109.5	H(32B)-C(32)-H(32C)	109.5
C(14)-C(16)-H(16A)	109.5	C(14)-C(16)-H(16B)	109.5
C(14)-C(16)-H(16C)	109.5	H(16A)-C(16)-H(16B)	109.5
H(16A)-C(16)-H(16C)	109.5	H(16B)-C(16)-H(16C)	109.5
C(21)-C(20)-C(19)	118.5(11)	C(21)-C(20)-C(23)	121.4(11)
C(19)-C(20)-C(23)	120.1(11)	C(9)-C(10)-S(1)	117.3(9)
C(11)-C(10)-S(1)	118.4(9)	C(11)-C(10)-C(9)	124.3(11)
C(28)-C(31)-H(31A)	109.5	C(28)-C(31)-H(31B)	109.5
C(28)-C(31)-H(31C)	109.5	H(31A)-C(31)-H(31B)	109.5

H(31A)-C(31)-H(31C)	109.5	H(31B)-C(31)-H(31C)	109.5
C(18)-C(17)-Bi(2)	116.7(8)	C(18)-C(17)-C(22)	118.5(11)
C(22)-C(17)-Bi(2)	124.7(8)	C(14)-C(13)-H(13)	118.9
C(12)-C(13)-C(14)	122.3(11)	C(12)-C(13)-H(13)	118.9
C(20)-C(23)-H(23A)	109.5	C(20)-C(23)-H(23B)	109.5
C(20)-C(23)-H(23C)	109.5	H(23A)-C(23)-H(23B)	109.5
H(23A)-C(23)-H(23C)	109.5	H(23B)-C(23)-H(23C)	109.5

8.21 Single crystal structure analysis of **161·$CHCl_3$**

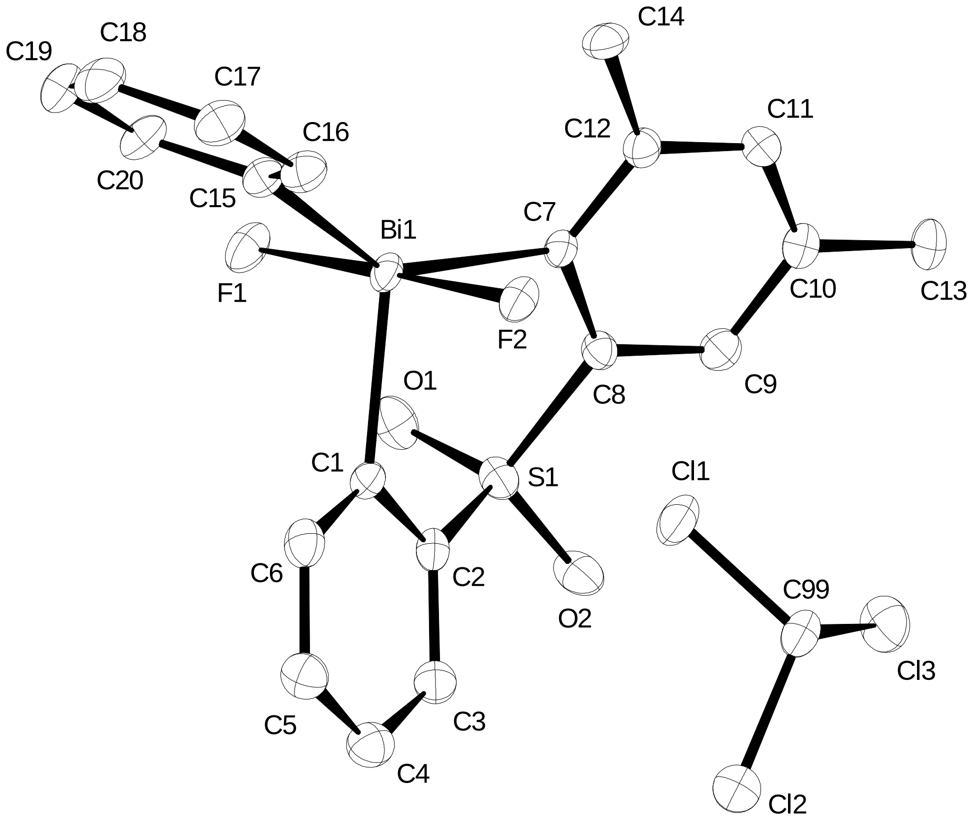

Figure S119. The molecular structure of complex **161·$CHCl_3$**. H atoms have been removed for clarity.

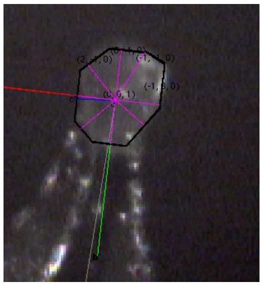

	h	k	l	distance (mm)
1.	0	0	-1	0.03
2.	0	0	1	0.03
3.	0	-1	0	0.08
4.	0	1	0	0.07
5.	2	1	0	0.07
6.	2	-1	0	0.07
7.	-1	-1	0	0.09
8.	-3	1	0	0.06
9.	1	0	0	0.06
10.	-1	0	0	0.07

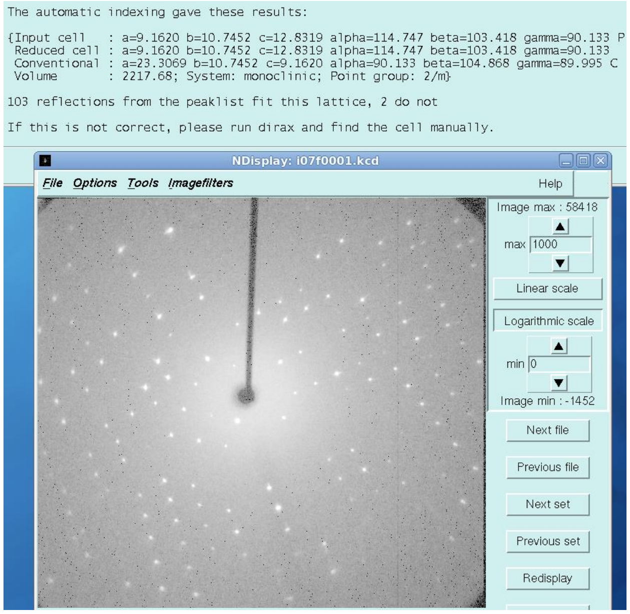

Figure S120. Crystal faces and unit cell determination of **161·$CHCl_3$**.

INTENSITY STATISTICS FOR DATASET

Resolution	#Data	#Theory	%Complete	Redundancy	Mean I	Mean I/s	Rmerge	Rsigma
Inf - 2.49	146	151	96.7	4.36	150.79	28.79	0.0700	0.0352
2.49 - 1.69	341	341	100.0	3.76	112.15	25.88	0.0694	0.0370
1.69 - 1.33	500	500	100.0	3.61	80.58	24.90	0.0715	0.0381
1.33 - 1.16	483	483	100.0	3.48	53.20	22.67	0.0707	0.0392
1.16 - 1.06	485	485	100.0	3.34	45.71	21.79	0.0749	0.0405
1.06 - 0.98	509	509	100.0	3.18	38.89	20.55	0.0708	0.0422
0.98 - 0.92	515	515	100.0	3.01	30.84	18.85	0.0714	0.0455
0.92 - 0.88	445	445	100.0	2.88	29.12	18.22	0.0720	0.0476
0.88 - 0.84	518	519	99.8	2.76	23.55	16.53	0.0728	0.0506
0.84 - 0.81	458	459	99.8	2.64	20.04	15.42	0.0778	0.0540
0.81 - 0.78	522	523	99.8	2.50	18.10	14.85	0.0791	0.0579
0.78 - 0.75	608	608	100.0	2.41	16.45	13.54	0.0772	0.0620
0.75 - 0.73	460	463	99.4	2.30	13.65	12.80	0.0827	0.0670
0.73 - 0.71	494	495	99.8	2.21	13.30	12.10	0.0844	0.0718
0.71 - 0.69	623	626	99.5	2.12	11.23	10.54	0.0895	0.0788
0.69 - 0.68	307	309	99.4	2.05	11.24	10.54	0.0854	0.0821
0.68 - 0.66	682	686	99.4	1.94	9.41	9.45	0.0987	0.0932
0.66 - 0.65	387	391	99.0	1.94	8.49	8.67	0.0893	0.1021
0.65 - 0.64	388	393	98.7	1.84	8.14	8.27	0.1011	0.1093
0.64 - 0.63	444	454	97.8	1.79	7.04	7.53	0.1110	0.1253
0.63 - 0.62	425	510	83.3	1.49	6.39	6.67	0.1116	0.1416
0.72 - 0.62	3488	3601	96.9	1.90	9.23	9.10	0.0949	0.0967
Inf - 0.62	9740	9865	98.7	2.58	28.74	15.17	0.0735	0.0495

Anharmonic displacements of the heavy atom can probably cause the high residual electron density in the vicinity of the Bi atom.

Complete .cif-data of the compound are available under the CCDC number **CCDC-2125781.**

Table S67. Crystal data and structure refinement of **161·CHCl$_3$**.

Identification code	13412sadabs	
Empirical formula	$C_{21} H_{18} Bi Cl_3 F_2 O_2 S$	
Color	colourless	
Formula weight	687.74 g·mol^{-1}	
Temperature	100(2) K	
Wavelength	0.71073 Å	
Crystal system	Triclinic	
Space group	*P*-1, (no. 2)	
Unit cell dimensions	a = 9.1789(8) Å	α= 114.760(6)°.
	b = 10.7675(5) Å	β= 103.408(14)°.
	c = 12.8484(14) Å	γ = 90.095(8)°.
Volume	1114.65(18) Å^3	
Z	2	
Density (calculated)	2.049 Mg·m^{-3}	
Absorption coefficient	8.395 mm^{-1}	
F(000)	656 e	
Crystal size	0.08 x 0.06 x 0.03 mm^3	
θ range for data collection	2.931 to 27.500°.	
Index ranges	-11 ≤ h ≤ 11, -13≤ k ≤ 13, -16 ≤ l ≤ 16	
Reflections collected	15895	
Independent reflections	5104 [R_{int} = 0.0716]	
Reflections with I>2σ(I)	4920	
Completeness to θ = 25.242°	99.9 %	
Absorption correction	Gaussian	
Max. and min. transmission	0.5099 and 0.3053	
Refinement method	Full-matrix least-squares on F^2	
Data / restraints / parameters	5104 / 0 / 273	
Goodness-of-fit on F^2	1.054	
Final R indices [I>2σ(I)]	R_1 = 0.0341	wR^2 = 0.0851
R indices (all data)	R_1 = 0.0354	wR^2 = 0.0864
Extinction coefficient	n/a	
Largest diff. peak and hole	2.670 and -2.815 e·Å^{-3}	

Table S68. Bond lengths [Å] and angles [°] of **161·$CHCl_3$**.

Bi(1)-F(1)	2.098(3)	Bi(1)-F(2)	2.153(3)
Bi(1)-C(1)	2.192(4)	Bi(1)-C(7)	2.194(4)
Bi(1)-C(15)	2.199(4)	S(1)-O(1)	1.441(4)
S(1)-O(2)	1.446(3)	S(1)-C(2)	1.772(4)
S(1)-C(8)	1.785(4)	C(1)-C(2)	1.406(6)
C(1)-C(6)	1.370(6)	C(2)-C(3)	1.392(6)
C(3)-H(3)	0.9500	C(3)-C(4)	1.389(6)
C(4)-H(4)	0.9500	C(4)-C(5)	1.387(7)
C(5)-H(5)	0.9500	C(5)-C(6)	1.390(7)
C(6)-H(6)	0.9500	C(7)-C(8)	1.400(6)
C(7)-C(12)	1.387(6)	C(8)-C(9)	1.393(6)
C(9)-H(9)	0.9500	C(9)-C(10)	1.391(6)
C(10)-C(11)	1.401(7)	C(10)-C(13)	1.505(6)
C(11)-H(11)	0.9500	C(11)-C(12)	1.403(6)
C(12)-C(14)	1.523(6)	C(13)-H(13A)	0.9800
C(13)-H(13B)	0.9800	C(13)-H(13C)	0.9800
C(14)-H(14A)	0.9800	C(14)-H(14B)	0.9800
C(14)-H(14C)	0.9800	C(15)-C(16)	1.378(7)
C(15)-C(20)	1.386(6)	C(16)-H(16)	0.9500
C(16)-C(17)	1.403(6)	C(17)-H(17)	0.9500
C(17)-C(18)	1.388(7)	C(18)-H(18)	0.9500
C(18)-C(19)	1.390(7)	C(19)-H(19)	0.9500
C(19)-C(20)	1.391(6)	C(20)-H(20)	0.9500
Cl(1)-C(99)	1.757(5)	Cl(2)-C(99)	1.774(5)
Cl(3)-C(99)	1.761(5)	C(99)-H(99)	1.0000
F(1)-Bi(1)-F(2)	176.29(9)	F(1)-Bi(1)-C(1)	92.47(14)
F(1)-Bi(1)-C(7)	100.83(14)	F(1)-Bi(1)-C(15)	90.16(15)
F(2)-Bi(1)-C(1)	87.04(14)	F(2)-Bi(1)-C(7)	82.87(13)
F(2)-Bi(1)-C(15)	86.70(15)	C(1)-Bi(1)-C(7)	100.05(16)
C(1)-Bi(1)-C(15)	114.63(16)	C(7)-Bi(1)-C(15)	143.15(17)
O(1)-S(1)-O(2)	119.4(2)	O(1)-S(1)-C(2)	107.40(19)
O(1)-S(1)-C(8)	106.7(2)	O(2)-S(1)-C(2)	106.9(2)
O(2)-S(1)-C(8)	107.3(2)	C(2)-S(1)-C(8)	108.8(2)
C(2)-C(1)-Bi(1)	119.5(3)	C(6)-C(1)-Bi(1)	120.5(3)
C(6)-C(1)-C(2)	119.6(4)	C(1)-C(2)-S(1)	122.9(3)

C(3)-C(2)-S(1)	116.6(3)	C(3)-C(2)-C(1)	120.1(4)
C(2)-C(3)-H(3)	120.3	C(4)-C(3)-C(2)	119.4(4)
C(4)-C(3)-H(3)	120.3	C(3)-C(4)-H(4)	119.8
C(5)-C(4)-C(3)	120.4(5)	C(5)-C(4)-H(4)	119.8
C(4)-C(5)-H(5)	120.1	C(4)-C(5)-C(6)	119.8(4)
C(6)-C(5)-H(5)	120.1	C(1)-C(6)-C(5)	120.7(4)
C(1)-C(6)-H(6)	119.6	C(5)-C(6)-H(6)	119.6
C(8)-C(7)-Bi(1)	117.9(3)	C(12)-C(7)-Bi(1)	119.9(3)
C(12)-C(7)-C(8)	121.0(4)	C(7)-C(8)-S(1)	124.4(3)
C(9)-C(8)-S(1)	115.3(3)	C(9)-C(8)-C(7)	120.0(4)
C(8)-C(9)-H(9)	119.8	C(10)-C(9)-C(8)	120.4(4)
C(10)-C(9)-H(9)	119.8	C(9)-C(10)-C(11)	118.5(4)
C(9)-C(10)-C(13)	119.5(4)	C(11)-C(10)-C(13)	121.9(4)
C(10)-C(11)-H(11)	119.0	C(10)-C(11)-C(12)	122.0(4)
C(12)-C(11)-H(11)	119.0	C(7)-C(12)-C(11)	118.0(4)
C(7)-C(12)-C(14)	121.8(4)	C(11)-C(12)-C(14)	120.2(4)
C(10)-C(13)-H(13A)	109.5	C(10)-C(13)-H(13B)	109.5
C(10)-C(13)-H(13C)	109.5	H(13A)-C(13)-H(13B)	109.5
H(13A)-C(13)-H(13C)	109.5	H(13B)-C(13)-H(13C)	109.5
C(12)-C(14)-H(14A)	109.5	C(12)-C(14)-H(14B)	109.5
C(12)-C(14)-H(14C)	109.5	H(14A)-C(14)-H(14B)	109.5
H(14A)-C(14)-H(14C)	109.5	H(14B)-C(14)-H(14C)	109.5
C(16)-C(15)-Bi(1)	118.0(3)	C(16)-C(15)-C(20)	123.2(4)
C(20)-C(15)-Bi(1)	118.5(3)	C(15)-C(16)-H(16)	120.9
C(15)-C(16)-C(17)	118.3(4)	C(17)-C(16)-H(16)	120.9
C(16)-C(17)-H(17)	120.2	C(18)-C(17)-C(16)	119.7(5)
C(18)-C(17)-H(17)	120.2	C(17)-C(18)-H(18)	119.7
C(17)-C(18)-C(19)	120.6(4)	C(19)-C(18)-H(18)	119.7
C(18)-C(19)-H(19)	119.7	C(18)-C(19)-C(20)	120.5(4)
C(20)-C(19)-H(19)	119.7	C(15)-C(20)-C(19)	117.7(4)
C(15)-C(20)-H(20)	121.1	C(19)-C(20)-H(20)	121.1
Cl(1)-C(99)-Cl(2)	109.9(3)	Cl(1)-C(99)-Cl(3)	110.6(3)
Cl(1)-C(99)-H(99)	108.6	Cl(2)-C(99)-H(99)	108.6
Cl(3)-C(99)-Cl(2)	110.3(3)	Cl(3)-C(99)-H(99)	108.6

8.22 Single crystal structure analysis of **162·($CHCl_3$)$_2$**

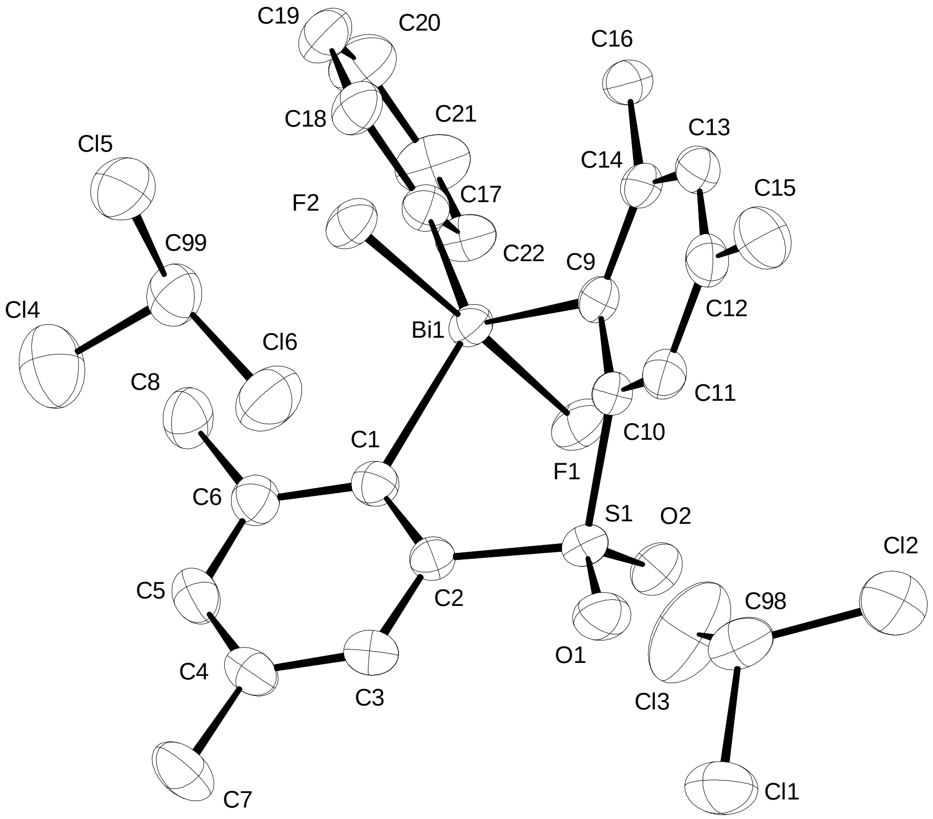

Figure S121. The molecular structure of complex **162·($CHCl_3$)$_2$**. H atoms have been removed for clarity.

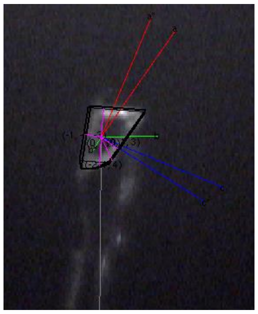

	h	k	l	distance (mm)
1.	0	-1	0	0.045
2.	0	1	0	0.04
3.	-1	-1	-3	0.03
4.	0	1	3	0.04
5.	2	0	-1	0.055
6.	-2	1	4	0.05
7.	-4	-1	2	0.055

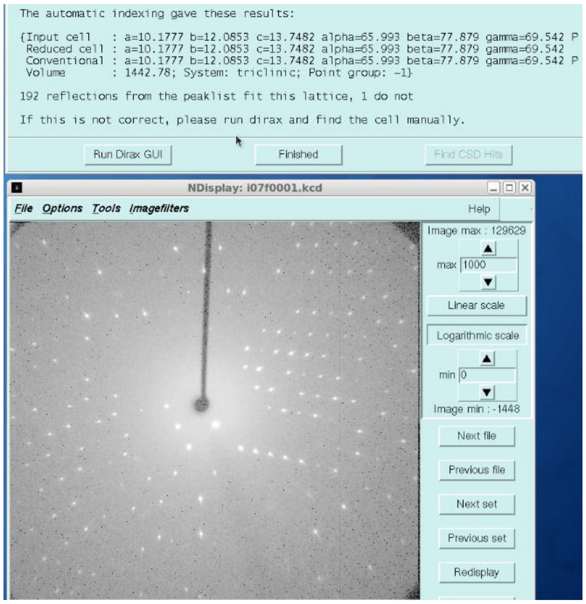

Figure S122. Crystal faces and unit cell determination of **162·($CHCl_3$)$_2$**.

INTENSITY STATISTICS FOR DATASET

Resolution	#Data	#Theory	%Complete	Redundancy	Mean I	Mean I/s	Rmerge	Rsigma
Inf - 2.41	205	212	96.7	8.07	176.36	68.57	0.0433	0.0143
2.41 - 1.63	486	486	100.0	7.41	107.08	58.90	0.0335	0.0149
1.63 - 1.29	701	701	100.0	7.10	68.59	52.04	0.0286	0.0162
1.29 - 1.13	684	684	100.0	6.89	43.57	45.05	0.0294	0.0183
1.13 - 1.03	670	670	100.0	6.63	33.66	39.76	0.0328	0.0205
1.03 - 0.95	766	766	100.0	6.23	26.65	34.54	0.0375	0.0235
0.95 - 0.90	621	621	100.0	5.89	20.75	29.65	0.0442	0.0274
0.90 - 0.85	753	753	100.0	5.54	16.87	25.54	0.0520	0.0320
0.85 - 0.82	588	588	100.0	5.27	13.74	21.78	0.0621	0.0372
0.82 - 0.78	879	879	100.0	5.05	11.39	18.89	0.0723	0.0441
0.78 - 0.76	497	496	100.2	4.77	9.87	16.69	0.0814	0.0514
0.76 - 0.73	881	882	99.9	4.62	8.09	14.06	0.0961	0.0616
0.73 - 0.71	678	678	100.0	4.31	7.27	12.24	0.1115	0.0718
0.71 - 0.69	769	769	100.0	4.24	6.20	10.52	0.1314	0.0862
0.69 - 0.68	388	388	100.0	4.06	5.34	8.68	0.1547	0.1046
0.68 - 0.66	928	928	100.0	3.95	4.74	7.45	0.1741	0.1267
0.66 - 0.65	486	486	100.0	3.80	4.36	6.61	0.1891	0.1495
0.65 - 0.63	1063	1063	100.0	3.68	3.66	4.91	0.2227	0.2066
0.63 - 0.62	605	605	100.0	3.52	2.92	3.43	0.2761	0.3056
0.62 - 0.61	609	609	100.0	3.46	2.62	2.74	0.3101	0.3814
0.61 - 0.60	416	450	92.4	3.11	2.36	2.26	0.3453	0.4699
0.70 - 0.60	4898	4932	99.3	3.71	3.91	5.57	0.2060	0.1946
Inf - 0.60	13673	13714	99.7	5.02	21.30	21.25	0.0464	0.0344

Complete .cif-data of the compound are available under the CCDC number **CCDC-2125787.**

Table S69. Crystal data and structure refinement of **162·$(CHCl_3)_2$**.

Identification code	12970sadabs	
Empirical formula	$C_{24} H_{23} Bi Cl_6 F_2 O_2 S$	
Color	colourless	
Formula weight	835.16 g·mol^{-1}	
Temperature	100(2) K	
Wavelength	0.71073 Å	
Crystal system	Triclinic	
Space group	*P*-1, (no. 2)	
Unit cell dimensions	a = 10.1796(6) Å	α= 66.062(5)°.
	b = 12.0694(11) Å	β= 77.875(6)°.
	c = 13.7273(10) Å	γ = 69.463(7)°.
Volume	1439.0(2) Å^3	
Z	2	
Density (calculated)	1.927 Mg·m^{-3}	
Absorption coefficient	6.791 mm^{-1}	
F(000)	804 e	
Crystal size	0.1 x 0.08 x 0.07 mm^3	
θ range for data collection	2.793 to 27.500°.	
Index ranges	$-13 \le h \le 13$, $-15 \le k \le 15$, $-17 \le l \le 17$	
Reflections collected	40835	
Independent reflections	6614 [R_{int} = 0.0374]	
Reflections with I>2σ(I)	6292	
Completeness to θ = 25.242°	99.9 %	
Absorption correction	Gaussian	
Max. and min. transmission	0.65862 and 0.49371	
Refinement method	Full-matrix least-squares on F^2	
Data / restraints / parameters	6614 / 0 / 329	
Goodness-of-fit on F^2	1.088	
Final R indices [I>2σ(I)]	R_1 = 0.0182	wR^2 = 0.0421
R indices (all data)	R_1 = 0.0201	wR^2 = 0.0430
Extinction coefficient	n/a	
Largest diff. peak and hole	1.098 and -0.769 e·Å^{-3}	

Table S70. Bond lengths [Å] and angles [°] of **162·(CHCl$_3$)$_2$**.

Bi(1)-F(1)	2.1055(15)	Bi(1)-F(2)	2.1370(14)
Bi(1)-C(1)	2.192(2)	Bi(1)-C(9)	2.192(2)
Bi(1)-C(17)	2.199(2)	S(1)-O(1)	1.4320(19)
S(1)-O(2)	1.434(2)	S(1)-C(2)	1.783(2)
S(1)-C(10)	1.783(2)	C(1)-C(2)	1.398(3)
C(1)-C(6)	1.389(4)	C(2)-C(3)	1.385(3)
C(3)-H(3)	0.9500	C(3)-C(4)	1.394(4)
C(4)-C(5)	1.383(4)	C(4)-C(7)	1.512(4)
C(5)-H(5)	0.9500	C(5)-C(6)	1.395(4)
C(6)-C(8)	1.509(4)	C(7)-H(7A)	0.9800
C(7)-H(7B)	0.9800	C(7)-H(7C)	0.9800
C(8)-H(8A)	0.9800	C(8)-H(8B)	0.9800
C(8)-H(8C)	0.9800	C(9)-C(10)	1.396(3)
C(9)-C(14)	1.393(3)	C(10)-C(11)	1.383(4)
C(11)-H(11)	0.9500	C(11)-C(12)	1.398(4)
C(12)-C(13)	1.389(4)	C(12)-C(15)	1.509(4)
C(13)-H(13)	0.9500	C(13)-C(14)	1.392(4)
C(14)-C(16)	1.503(3)	C(15)-H(15A)	0.9800
C(15)-H(15B)	0.9800	C(15)-H(15C)	0.9800
C(16)-H(16A)	0.9800	C(16)-H(16B)	0.9800
C(16)-H(16C)	0.9800	C(17)-C(18)	1.381(4)
C(17)-C(22)	1.379(4)	C(18)-H(18)	0.9500
C(18)-C(19)	1.387(4)	C(19)-H(19)	0.9500
C(19)-C(20)	1.382(4)	C(20)-H(20)	0.9500
C(20)-C(21)	1.385(4)	C(21)-H(21)	0.9500
C(21)-C(22)	1.392(4)	C(22)-H(22)	0.9500
Cl(1)-C(98)	1.735(3)	Cl(2)-C(98)	1.769(4)
Cl(3)-C(98)	1.743(3)	C(98)-H(98)	1.0000
Cl(4)-C(99)	1.750(3)	Cl(5)-C(99)	1.749(3)
Cl(6)-C(99)	1.758(3)	C(99)-H(99)	1.0000
F(1)-Bi(1)-F(2)	176.05(6)	F(1)-Bi(1)-C(1)	93.43(8)
F(1)-Bi(1)-C(9)	98.78(8)	F(1)-Bi(1)-C(17)	88.43(8)
F(2)-Bi(1)-C(1)	86.46(8)	F(2)-Bi(1)-C(9)	85.12(7)
F(2)-Bi(1)-C(17)	88.53(8)	C(1)-Bi(1)-C(9)	100.66(9)

C(1)-Bi(1)-C(17)	128.23(9)	C(9)-Bi(1)-C(17)	130.16(9)
O(1)-S(1)-O(2)	119.35(12)	O(1)-S(1)-C(2)	107.13(12)
O(1)-S(1)-C(10)	107.48(12)	O(2)-S(1)-C(2)	108.04(12)
O(2)-S(1)-C(10)	106.41(12)	C(10)-S(1)-C(2)	107.99(11)
C(2)-C(1)-Bi(1)	118.17(18)	C(6)-C(1)-Bi(1)	120.73(18)
C(6)-C(1)-C(2)	120.5(2)	C(1)-C(2)-S(1)	124.24(19)
C(3)-C(2)-S(1)	115.47(19)	C(3)-C(2)-C(1)	120.3(2)
C(2)-C(3)-H(3)	120.0	C(2)-C(3)-C(4)	120.1(2)
C(4)-C(3)-H(3)	120.0	C(3)-C(4)-C(7)	120.1(3)
C(5)-C(4)-C(3)	118.8(2)	C(5)-C(4)-C(7)	121.1(3)
C(4)-C(5)-H(5)	118.8	C(4)-C(5)-C(6)	122.4(2)
C(6)-C(5)-H(5)	118.8	C(1)-C(6)-C(5)	117.9(2)
C(1)-C(6)-C(8)	122.4(2)	C(5)-C(6)-C(8)	119.6(2)
C(4)-C(7)-H(7A)	109.5	C(4)-C(7)-H(7B)	109.5
C(4)-C(7)-H(7C)	109.5	H(7A)-C(7)-H(7B)	109.5
H(7A)-C(7)-H(7C)	109.5	H(7B)-C(7)-H(7C)	109.5
C(6)-C(8)-H(8A)	109.5	C(6)-C(8)-H(8B)	109.5
C(6)-C(8)-H(8C)	109.5	H(8A)-C(8)-H(8B)	109.5
H(8A)-C(8)-H(8C)	109.5	H(8B)-C(8)-H(8C)	109.5
C(10)-C(9)-Bi(1)	117.13(17)	C(14)-C(9)-Bi(1)	120.30(18)
C(14)-C(9)-C(10)	120.8(2)	C(9)-C(10)-S(1)	122.46(19)
C(11)-C(10)-S(1)	116.43(19)	C(11)-C(10)-C(9)	120.5(2)
C(10)-C(11)-H(11)	120.1	C(10)-C(11)-C(12)	119.8(2)
C(12)-C(11)-H(11)	120.1	C(11)-C(12)-C(15)	120.2(2)
C(13)-C(12)-C(11)	118.5(2)	C(13)-C(12)-C(15)	121.3(2)
C(12)-C(13)-H(13)	118.6	C(12)-C(13)-C(14)	122.8(2)
C(14)-C(13)-H(13)	118.6	C(9)-C(14)-C(16)	122.1(2)
C(13)-C(14)-C(9)	117.4(2)	C(13)-C(14)-C(16)	120.5(2)
C(12)-C(15)-H(15A)	109.5	C(12)-C(15)-H(15B)	109.5
C(12)-C(15)-H(15C)	109.5	H(15A)-C(15)-H(15B)	109.5
H(15A)-C(15)-H(15C)	109.5	H(15B)-C(15)-H(15C)	109.5
C(14)-C(16)-H(16A)	109.5	C(14)-C(16)-H(16B)	109.5
C(14)-C(16)-H(16C)	109.5	H(16A)-C(16)-H(16B)	109.5
H(16A)-C(16)-H(16C)	109.5	H(16B)-C(16)-H(16C)	109.5
C(18)-C(17)-Bi(1)	117.95(18)	C(22)-C(17)-Bi(1)	118.79(18)
C(22)-C(17)-C(18)	123.3(2)	C(17)-C(18)-H(18)	120.9

C(17)-C(18)-C(19)	118.2(2)	C(19)-C(18)-H(18)	120.9
C(18)-C(19)-H(19)	120.0	C(20)-C(19)-C(18)	120.0(3)
C(20)-C(19)-H(19)	120.0	C(19)-C(20)-H(20)	119.7
C(19)-C(20)-C(21)	120.5(3)	C(21)-C(20)-H(20)	119.7
C(20)-C(21)-H(21)	119.7	C(20)-C(21)-C(22)	120.5(3)
C(22)-C(21)-H(21)	119.7	C(17)-C(22)-C(21)	117.5(3)
C(17)-C(22)-H(22)	121.3	C(21)-C(22)-H(22)	121.3
Cl(1)-C(98)-Cl(2)	110.31(17)	Cl(1)-C(98)-Cl(3)	110.41(19)
Cl(1)-C(98)-H(98)	108.8	Cl(2)-C(98)-H(98)	108.8
Cl(3)-C(98)-Cl(2)	109.58(17)	Cl(3)-C(98)-H(98)	108.8
Cl(4)-C(99)-Cl(6)	111.12(18)	Cl(4)-C(99)-H(99)	108.7
Cl(5)-C(99)-Cl(4)	109.12(17)	Cl(5)-C(99)-Cl(6)	110.47(18)
Cl(5)-C(99)-H(99)	108.7	Cl(6)-C(99)-H(99)	108.7

8.23 Single crystal structure analysis of **175·CHCl3**

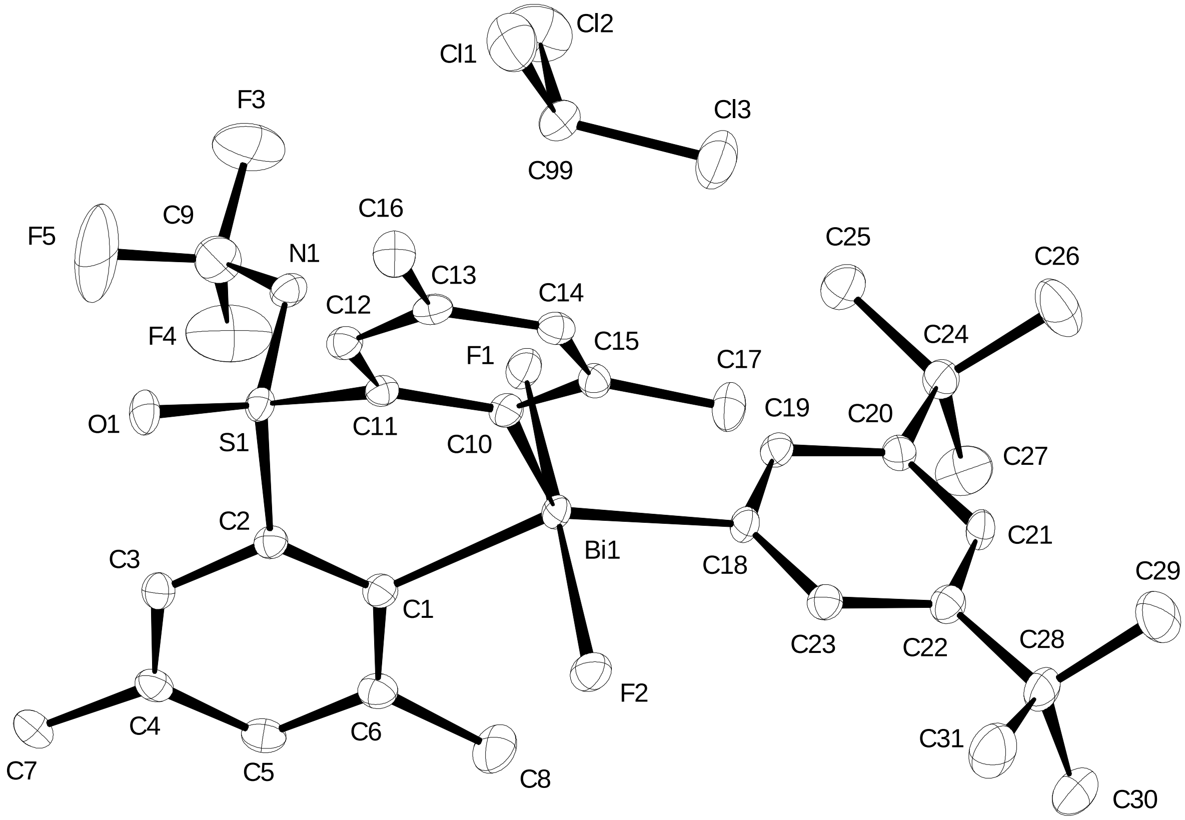

Figure S123. The molecular structure of complex **175·CHCl3**. H atoms have been removed for clarity.

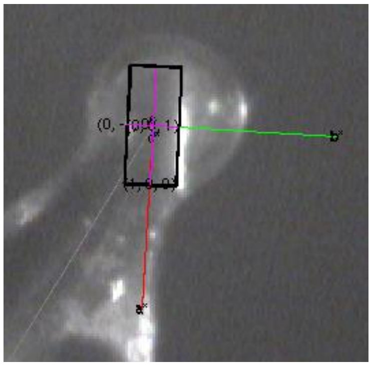

	h	k	l	distance (mm)
1.	0	-1	0	0.035
2.	0	1	0	0.035
3.	1	0	0	0.08
4.	-1	0	0	0.08
5.	0	0	-1	0.02
6.	0	0	1	0.02

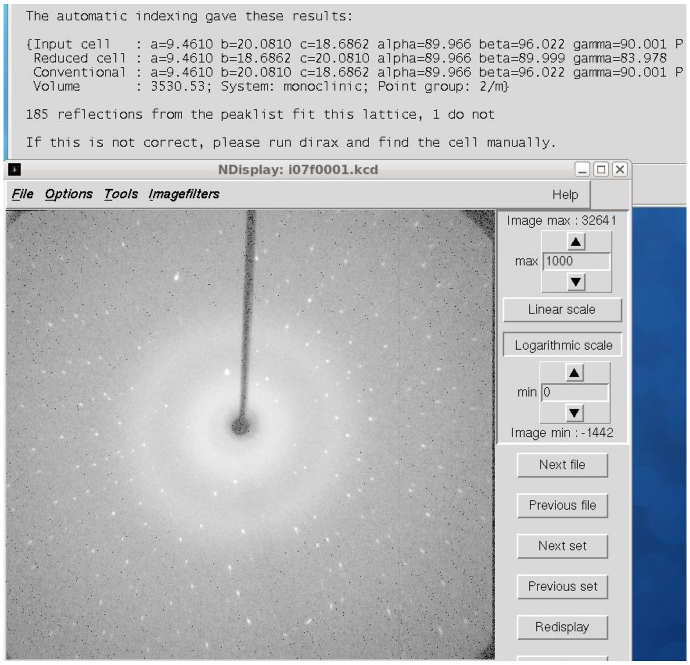

Figure S124. Crystal faces and unit cell determination of **175·CHCl3**.

INTENSITY STATISTICS FOR DATASET

Resolution	#Data	#Theory	%Complete	Redundancy	Mean I	Mean I/s	Rmerge	Rsigma
Inf - 2.46	259	271	95.6	8.07	69.24	64.06	0.0340	0.0113
2.46 - 1.65	607	607	100.0	7.58	50.16	51.72	0.0296	0.0139
1.65 - 1.31	854	854	100.0	7.40	33.36	42.80	0.0323	0.0169
1.31 - 1.14	874	874	100.0	7.13	24.44	33.82	0.0397	0.0211
1.14 - 1.03	921	921	100.0	6.83	20.06	28.18	0.0453	0.0256
1.03 - 0.96	792	792	100.0	6.47	17.06	23.64	0.0521	0.0306
0.96 - 0.90	931	931	100.0	6.05	14.52	19.74	0.0594	0.0368
0.90 - 0.85	946	946	100.0	5.77	12.45	16.98	0.0681	0.0439
0.85 - 0.82	719	719	100.0	5.48	10.54	14.39	0.0821	0.0527
0.82 - 0.78	1093	1093	100.0	5.27	9.49	12.78	0.0893	0.0611
0.78 - 0.76	642	642	100.0	4.96	8.61	11.39	0.0991	0.0712
0.76 - 0.73	1097	1097	100.0	4.81	7.30	9.60	0.1187	0.0854
0.73 - 0.71	857	857	100.0	4.48	6.98	8.77	0.1253	0.0970
0.71 - 0.69	914	914	100.0	4.34	6.55	8.11	0.1440	0.1067
0.69 - 0.68	526	526	100.0	4.25	5.55	6.72	0.1653	0.1296
0.68 - 0.66	1116	1118	99.8	4.02	5.66	6.69	0.1695	0.1349
0.66 - 0.65	628	628	100.0	3.87	4.67	5.44	0.2014	0.1658
0.65 - 0.63	1300	1300	100.0	3.85	4.16	4.85	0.2198	0.1910
0.63 - 0.62	761	764	99.6	3.55	4.04	4.31	0.2370	0.2194
0.62 - 0.61	800	800	100.0	3.54	3.49	3.60	0.2746	0.2700
0.61 - 0.60	483	488	99.0	3.32	3.49	3.32	0.2868	0.2958
0.70 - 0.60	6064	6074	99.8	3.83	4.65	5.31	0.2011	0.1775
Inf - 0.60	17120	17142	99.9	5.20	13.09	16.28	0.0621	0.0511

Complete .cif-data of the compound are available under the CCDC number **CCDC-2125784.**

Table S71. Crystal data and structure refinement of **175·$CHCl_3$**.

Identification code	13767sadabs	
Empirical formula	C_{32} H_{38} Bi Cl_3 F_5 N O S	
Color	colourless	
Formula weight	895.02 g·mol^{-1}	
Temperature	100(2) K	
Wavelength	0.71073 Å	
Crystal system	Monoclinic	
Space group	$P2_1$/c, (no. 14)	
Unit cell dimensions	a = 9.4545(4) Å	α= 90°.
	b = 20.066(3) Å	β= 96.017(5)°.
	c = 18.666(2) Å	γ = 90°.
Volume	3521.7(6) Å^3	
Z	4	
Density (calculated)	1.688 Mg·m^{-3}	
Absorption coefficient	5.346 mm^{-1}	
F(000)	1760 e	
Crystal size	0.16 x 0.07 x 0.04 mm^3	
θ range for data collection	2.725 to 30.508°.	
Index ranges	-13 ≤ h ≤ 13, -28≤ k ≤ 28, -26 ≤ l ≤ 26	
Reflections collected	64966	
Independent reflections	10764 [R_{int} = 0.0517]	
Reflections with I>2σ(I)	8902	
Completeness to θ = 25.242°	99.9 %	
Absorption correction	Gaussian	
Max. and min. transmission	0.81311 and 0.48146	
Refinement method	Full-matrix least-squares on F^2	
Data / restraints / parameters	10764 / 0 / 407	
Goodness-of-fit on F^2	1.070	
Final R indices [I>2σ(I)]	R_1 = 0.0277	wR^2 = 0.0497
R indices (all data)	R_1 = 0.0413	wR^2 = 0.0534
Extinction coefficient	n/a	
Largest diff. peak and hole	1.329 and -1.028 e·Å^{-3}	

Table S72. Bond lengths [Å] and angles [°] of **175·$CHCl_3$**.

Bi(01)-F(1)	2.1049(16)	Bi(01)-F(2)	2.1221(17)
Bi(01)-C(1)	2.179(3)	Bi(01)-C(10)	2.189(3)
Bi(01)-C(18)	2.197(2)	S(1)-O(1)	1.4437(19)
S(1)-N(1)	1.535(2)	S(1)-C(2)	1.795(3)
S(1)-C(11)	1.782(3)	F(3)-C(012)	1.336(4)
F(4)-C(012)	1.333(4)	F(5)-C(012)	1.343(4)
N(1)-C(012)	1.381(4)	C(1)-C(2)	1.401(4)
C(1)-C(6)	1.389(4)	C(2)-C(3)	1.392(4)
C(3)-H(3)	0.9500	C(3)-C(4)	1.389(4)
C(4)-C(5)	1.390(4)	C(4)-C(7)	1.507(4)
C(5)-H(5)	0.9500	C(5)-C(6)	1.395(4)
C(6)-C(8)	1.514(4)	C(7)-H(7A)	0.9800
C(7)-H(7B)	0.9800	C(7)-H(7C)	0.9800
C(8)-H(8A)	0.9800	C(8)-H(8B)	0.9800
C(8)-H(8C)	0.9800	C(10)-C(11)	1.401(3)
C(10)-C(15)	1.394(4)	C(11)-C(12)	1.384(4)
C(12)-H(12)	0.9500	C(12)-C(13)	1.398(4)
C(13)-C(14)	1.394(4)	C(13)-C(16)	1.503(4)
C(14)-H(14)	0.9500	C(14)-C(15)	1.395(4)
C(15)-C(17)	1.507(4)	C(16)-H(16A)	0.9800
C(16)-H(16B)	0.9800	C(16)-H(16C)	0.9800
C(17)-H(17A)	0.9800	C(17)-H(17B)	0.9800
C(17)-H(17C)	0.9800	C(18)-C(19)	1.377(4)
C(18)-C(23)	1.386(4)	C(19)-H(19)	0.9500
C(19)-C(20)	1.403(4)	C(20)-C(21)	1.392(4)
C(20)-C(24)	1.533(4)	C(21)-H(21)	0.9500
C(21)-C(22)	1.398(4)	C(22)-C(23)	1.399(4)
C(22)-C(28)	1.533(4)	C(23)-H(23)	0.9500
C(24)-C(25)	1.536(4)	C(24)-C(26)	1.527(4)
C(24)-C(27)	1.539(4)	C(25)-H(25A)	0.9800
C(25)-H(25B)	0.9800	C(25)-H(25C)	0.9800
C(26)-H(26A)	0.9800	C(26)-H(26B)	0.9800
C(26)-H(26C)	0.9800	C(27)-H(27A)	0.9800
C(27)-H(27B)	0.9800	C(27)-H(27C)	0.9800
C(28)-C(29)	1.531(4)	C(28)-C(30)	1.546(5)

C(28)-C(31)	1.526(4)	C(29)-H(29A)	0.9800
C(29)-H(29B)	0.9800	C(29)-H(29C)	0.9800
C(30)-H(30A)	0.9800	C(30)-H(30B)	0.9800
C(30)-H(30C)	0.9800	C(31)-H(31A)	0.9800
C(31)-H(31B)	0.9800	C(31)-H(31C)	0.9800
Cl(1)-C(99)	1.745(3)	Cl(2)-C(99)	1.748(3)
Cl(3)-C(99)	1.771(3)	C(99)-H(99)	1.0000

F(1)-Bi(01)-F(2)	172.58(7)	F(1)-Bi(01)-C(1)	88.31(8)
F(1)-Bi(01)-C(10)	96.83(8)	F(1)-Bi(01)-C(18)	88.77(8)
F(2)-Bi(01)-C(1)	88.77(8)	F(2)-Bi(01)-C(10)	90.43(8)
F(2)-Bi(01)-C(18)	88.47(8)	C(1)-Bi(01)-C(10)	101.25(10)
C(1)-Bi(01)-C(18)	135.03(10)	C(10)-Bi(01)-C(18)	123.64(10)
O(1)-S(1)-N(1)	119.68(13)	O(1)-S(1)-C(2)	106.04(12)
O(1)-S(1)-C(11)	109.17(12)	N(1)-S(1)-C(2)	110.95(12)
N(1)-S(1)-C(11)	98.55(12)	C(11)-S(1)-C(2)	112.47(12)
C(012)-N(1)-S(1)	121.4(2)	C(2)-C(1)-Bi(01)	120.30(19)
C(6)-C(1)-Bi(01)	119.26(19)	C(6)-C(1)-C(2)	120.4(2)
C(1)-C(2)-S(1)	124.9(2)	C(3)-C(2)-S(1)	114.4(2)
C(3)-C(2)-C(1)	120.2(2)	C(2)-C(3)-H(3)	120.0
C(4)-C(3)-C(2)	120.0(2)	C(4)-C(3)-H(3)	120.0
C(3)-C(4)-C(5)	118.8(2)	C(3)-C(4)-C(7)	120.8(3)
C(5)-C(4)-C(7)	120.3(3)	C(4)-C(5)-H(5)	118.9
C(4)-C(5)-C(6)	122.2(3)	C(6)-C(5)-H(5)	118.9
C(1)-C(6)-C(5)	118.1(3)	C(1)-C(6)-C(8)	121.6(2)
C(5)-C(6)-C(8)	120.3(3)	C(4)-C(7)-H(7A)	109.5
C(4)-C(7)-H(7B)	109.5	C(4)-C(7)-H(7C)	109.5
H(7A)-C(7)-H(7B)	109.5	H(7A)-C(7)-H(7C)	109.5
H(7B)-C(7)-H(7C)	109.5	C(6)-C(8)-H(8A)	109.5
C(6)-C(8)-H(8B)	109.5	C(6)-C(8)-H(8C)	109.5
H(8A)-C(8)-H(8B)	109.5	H(8A)-C(8)-H(8C)	109.5
H(8B)-C(8)-H(8C)	109.5	C(11)-C(10)-Bi(01)	119.46(19)
C(15)-C(10)-Bi(01)	120.44(19)	C(15)-C(10)-C(11)	119.4(2)
C(10)-C(11)-S(1)	124.6(2)	C(12)-C(11)-S(1)	113.26(19)
C(12)-C(11)-C(10)	121.6(2)	C(11)-C(12)-H(12)	120.2
C(11)-C(12)-C(13)	119.6(2)	C(13)-C(12)-H(12)	120.2

F(3)-C(012)-F(5)	104.9(3)	F(3)-C(012)-N(1)	109.3(3)
F(4)-C(012)-F(3)	105.9(3)	F(4)-C(012)-F(5)	105.4(3)
F(4)-C(012)-N(1)	115.8(3)	F(5)-C(012)-N(1)	114.7(3)
C(12)-C(13)-C(16)	120.4(2)	C(14)-C(13)-C(12)	118.3(2)
C(14)-C(13)-C(16)	121.3(2)	C(13)-C(14)-H(14)	118.6
C(13)-C(14)-C(15)	122.8(2)	C(15)-C(14)-H(14)	118.6
C(10)-C(15)-C(14)	118.2(2)	C(10)-C(15)-C(17)	122.1(2)
C(14)-C(15)-C(17)	119.7(2)	C(13)-C(16)-H(16A)	109.5
C(13)-C(16)-H(16B)	109.5	C(13)-C(16)-H(16C)	109.5
H(16A)-C(16)-H(16B)	109.5	H(16A)-C(16)-H(16C)	109.5
H(16B)-C(16)-H(16C)	109.5	C(15)-C(17)-H(17A)	109.5
C(15)-C(17)-H(17B)	109.5	C(15)-C(17)-H(17C)	109.5
H(17A)-C(17)-H(17B)	109.5	H(17A)-C(17)-H(17C)	109.5
H(17B)-C(17)-H(17C)	109.5	C(19)-C(18)-Bi(01)	117.94(18)
C(19)-C(18)-C(23)	124.6(2)	C(23)-C(18)-Bi(01)	117.36(18)
C(18)-C(19)-H(19)	121.0	C(18)-C(19)-C(20)	118.0(2)
C(20)-C(19)-H(19)	121.0	C(19)-C(20)-C(24)	121.5(2)
C(21)-C(20)-C(19)	117.7(2)	C(21)-C(20)-C(24)	120.7(2)
C(20)-C(21)-H(21)	118.0	C(20)-C(21)-C(22)	124.0(2)
C(22)-C(21)-H(21)	118.0	C(21)-C(22)-C(23)	117.6(2)
C(21)-C(22)-C(28)	121.0(2)	C(23)-C(22)-C(28)	121.4(2)
C(18)-C(23)-C(22)	118.1(2)	C(18)-C(23)-H(23)	121.0
C(22)-C(23)-H(23)	121.0	C(20)-C(24)-C(25)	112.2(2)
C(20)-C(24)-C(27)	107.7(2)	C(25)-C(24)-C(27)	108.0(3)
C(26)-C(24)-C(20)	111.0(2)	C(26)-C(24)-C(25)	108.1(3)
C(26)-C(24)-C(27)	109.8(3)	C(24)-C(25)-H(25A)	109.5
C(24)-C(25)-H(25B)	109.5	C(24)-C(25)-H(25C)	109.5
H(25A)-C(25)-H(25B)	109.5	H(25A)-C(25)-H(25C)	109.5
H(25B)-C(25)-H(25C)	109.5	C(24)-C(26)-H(26A)	109.5
C(24)-C(26)-H(26B)	109.5	C(24)-C(26)-H(26C)	109.5
H(26A)-C(26)-H(26B)	109.5	H(26A)-C(26)-H(26C)	109.5
H(26B)-C(26)-H(26C)	109.5	C(24)-C(27)-H(27A)	109.5
C(24)-C(27)-H(27B)	109.5	C(24)-C(27)-H(27C)	109.5
H(27A)-C(27)-H(27B)	109.5	H(27A)-C(27)-H(27C)	109.5
H(27B)-C(27)-H(27C)	109.5	C(22)-C(28)-C(30)	107.7(2)
C(29)-C(28)-C(22)	110.1(2)	C(29)-C(28)-C(30)	109.1(3)

C(31)-C(28)-C(22)	112.8(2)	C(31)-C(28)-C(29)	108.9(3)
C(31)-C(28)-C(30)	108.2(3)	C(28)-C(29)-H(29A)	109.5
C(28)-C(29)-H(29B)	109.5	C(28)-C(29)-H(29C)	109.5
H(29A)-C(29)-H(29B)	109.5	H(29A)-C(29)-H(29C)	109.5
H(29B)-C(29)-H(29C)	109.5	C(28)-C(30)-H(30A)	109.5
C(28)-C(30)-H(30B)	109.5	C(28)-C(30)-H(30C)	109.5
H(30A)-C(30)-H(30B)	109.5	H(30A)-C(30)-H(30C)	109.5
H(30B)-C(30)-H(30C)	109.5	C(28)-C(31)-H(31A)	109.5
C(28)-C(31)-H(31B)	109.5	C(28)-C(31)-H(31C)	109.5
H(31A)-C(31)-H(31B)	109.5	H(31A)-C(31)-H(31C)	109.5
H(31B)-C(31)-H(31C)	109.5	Cl(1)-C(99)-Cl(2)	112.07(16)
Cl(1)-C(99)-Cl(3)	109.47(16)	Cl(1)-C(99)-H(99)	108.5
Cl(2)-C(99)-Cl(3)	109.71(17)	Cl(2)-C(99)-H(99)	108.5
Cl(3)-C(99)-H(99)	108.5		

9. Computational details

All calculations were performed using the development version of ORCA 4.2.[48-49] Geometries were optimized using the hybrid Becke 3-parameter Lee-Yang-Parr functional (B3LYP)[50-51] in conjunction with dispersion corrections (D3BJ),[52] the def2-TZVP(-f) basis set,[53] the decontracted auxiliary def2/J basis set[54] and the default effective core potential (ECP) for Bi.[55] Fine integration grids (grid5) were applied. In addition, the chain of spheres approximation (RIJCOSX)[56] was employed with fine integration grids (gridx5), as well as the conductor-like polarizable continuum model (CPCM) using chloroform as solvent. Subsequent frequency calculations were performed using the solvation model to evaluate enthalpy and entropy corrections at 298.15 K and ensured that all local minima had only real frequencies while a single imaginary frequency confirmed the presence of transition states. Natural bond orbital (NBO) analysis[57] was performed at the RIJCOSX-B3LYP-D3BJ/def2-TZVP(-f) level of theory. The Chemcraft software was used to display molecular geometries.[58]

9.1 Benchmarking of DFT methods with XRD structure of dimeric species **4**

Table S73. Evaluation of several methods using def2-TZVP(-f) as basis and comparison to the XRD structure parameters of **4**. B3LYP-D3BJ (in green) shows higher agreement with solid state parameters.

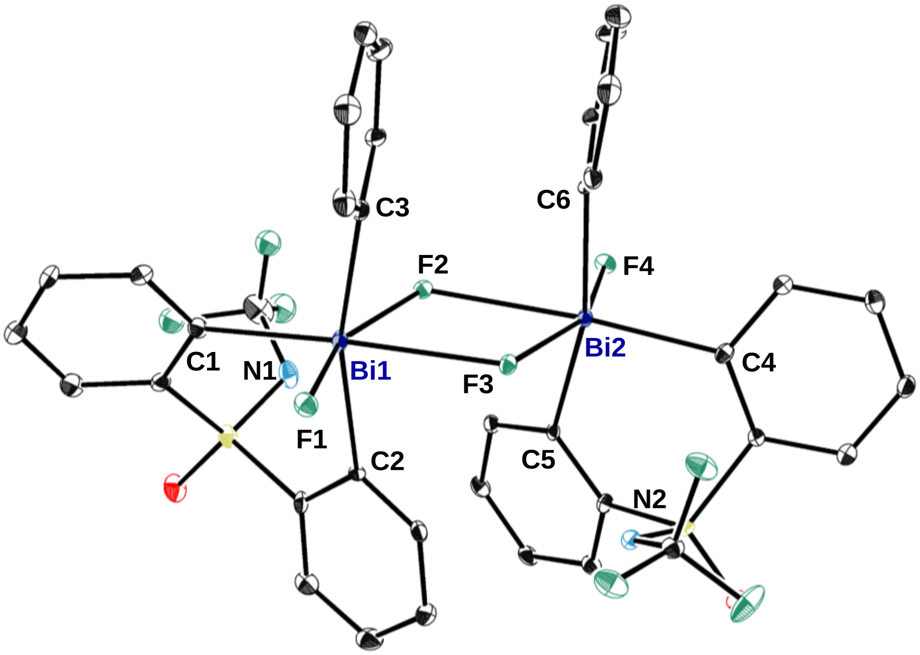

Parameters	XRD	B3LYP	B3LYP-D3BJ	wB97X-D3BJ	PBE0-D3BJ	M062X	M06
**Bi1-Bi2**	**3.92 Å**	**4.04 Å**	**3.88 Å**	**3.80 Å**	**3.85 Å**	**3.90 Å**	**3.86 Å**
Bi1-F1	2.09 Å	2.11 Å	2.11 Å	2.08 Å	2.09 Å	2.06 Å	2.09 Å
**C3-C6**	**3.62 Å**	**3.80 Å**	**3.57 Å**	**3.50 Å**	**3.56 Å**	**3.39 Å**	**3.49 Å**
**C2-C4**	**3.47 Å**	**3.76 Å**	**3.45 Å**	**3.40 Å**	**3.46 Å**	**3.88 Å**	**3.58 Å**
**RMSD**	**-**	**0.378**	**0.38**	**0.409**	**0.452**	**1.492**	**0.376**
Bi1-F2-Bi2	110.6 °	112.4 °	109.3 °	109.3 °	110.7 °	113.3 °	109.9 °

9.2 Reductive elimination from dimeric species **4**

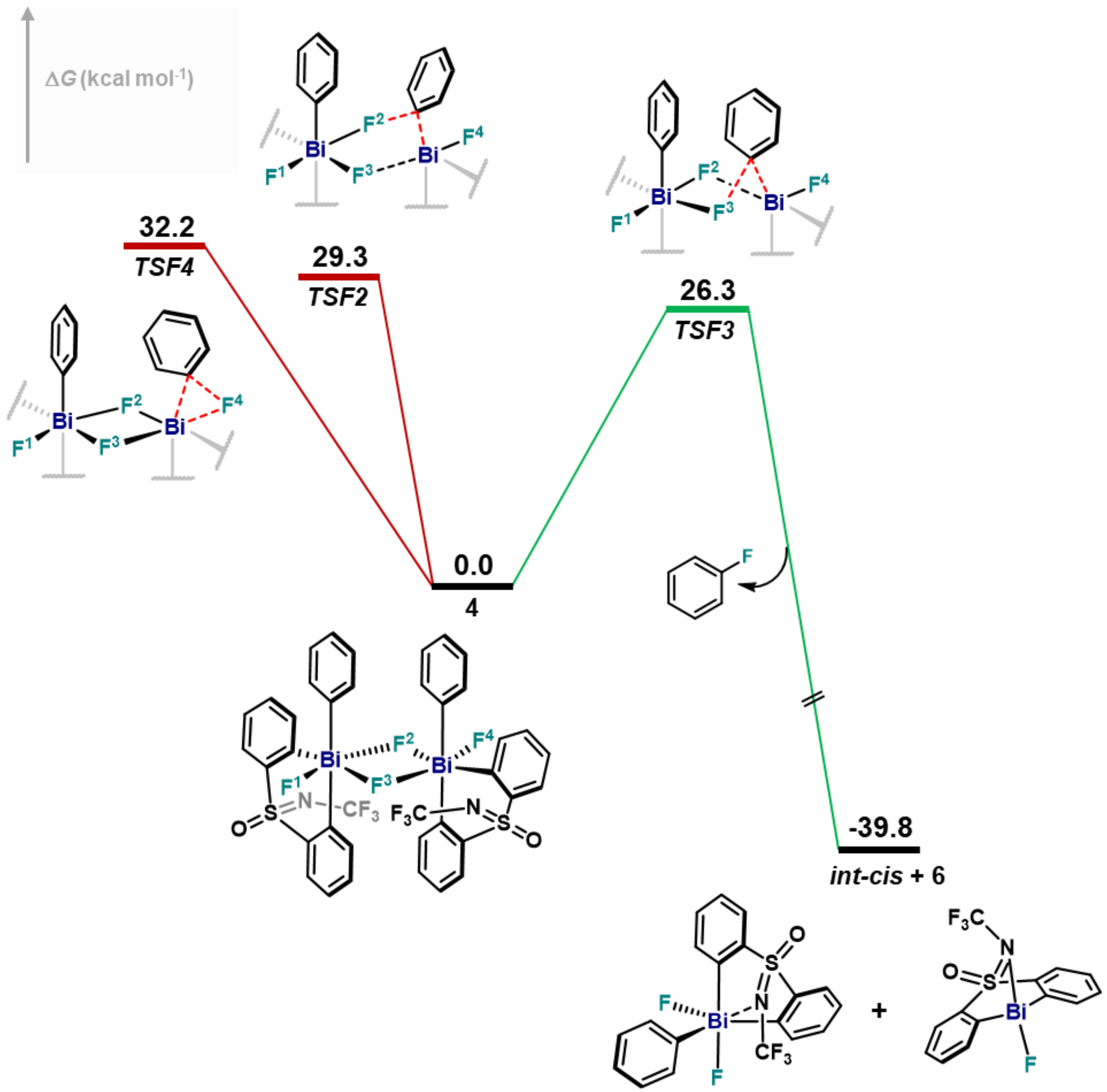

Figure S125. Gibbs energy profile of the reductive elimination of fluorobenzene from dimeric species **4**. Relative Gibbs energy values are given in kcal mol^{-1}.

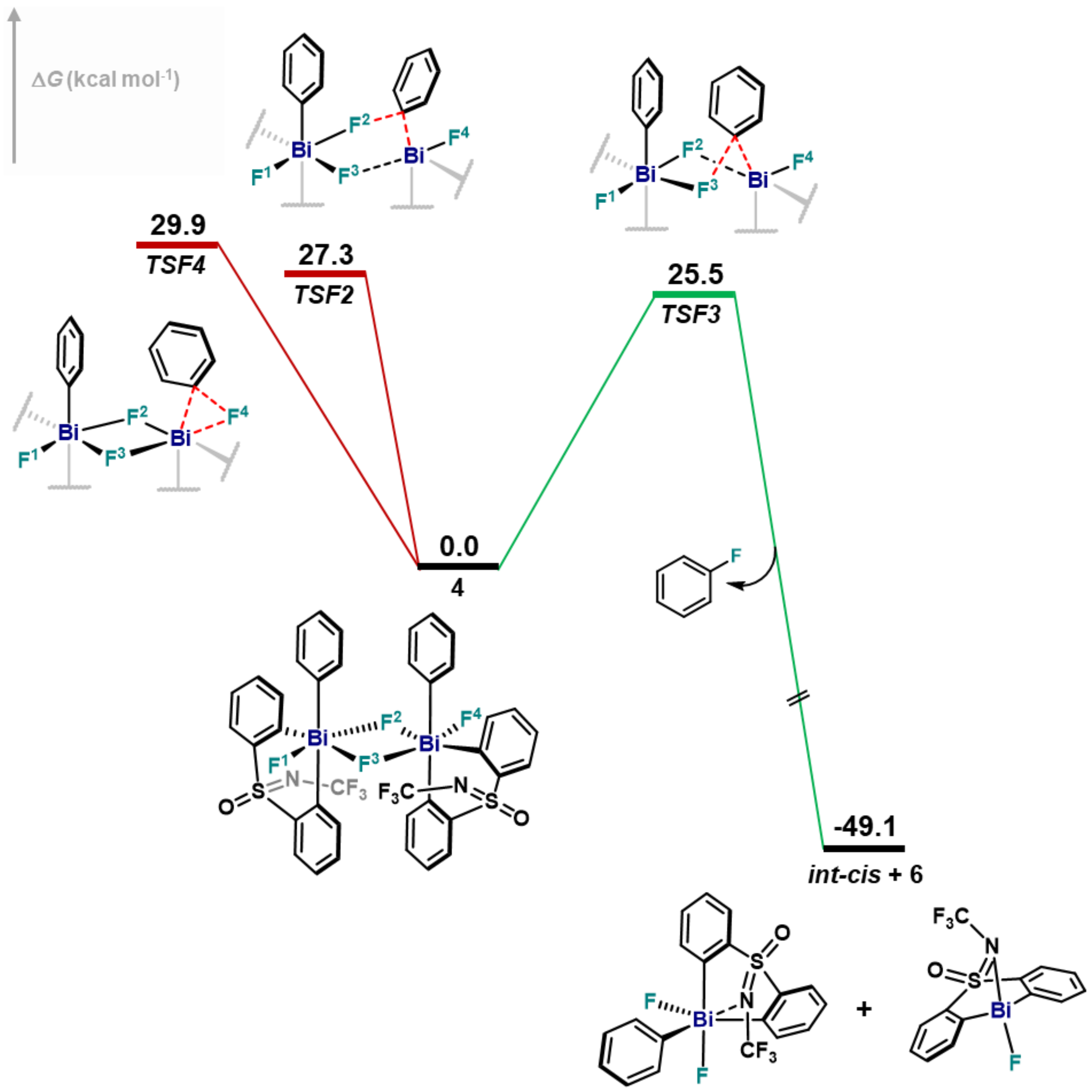

Figure S126. Gibbs energy profile of the reductive elimination of fluorobenzene from dimeric species **4**. Relative Gibbs energy values are given in kcal mol^{-1}. Thermal corrections calculated at 363 K.

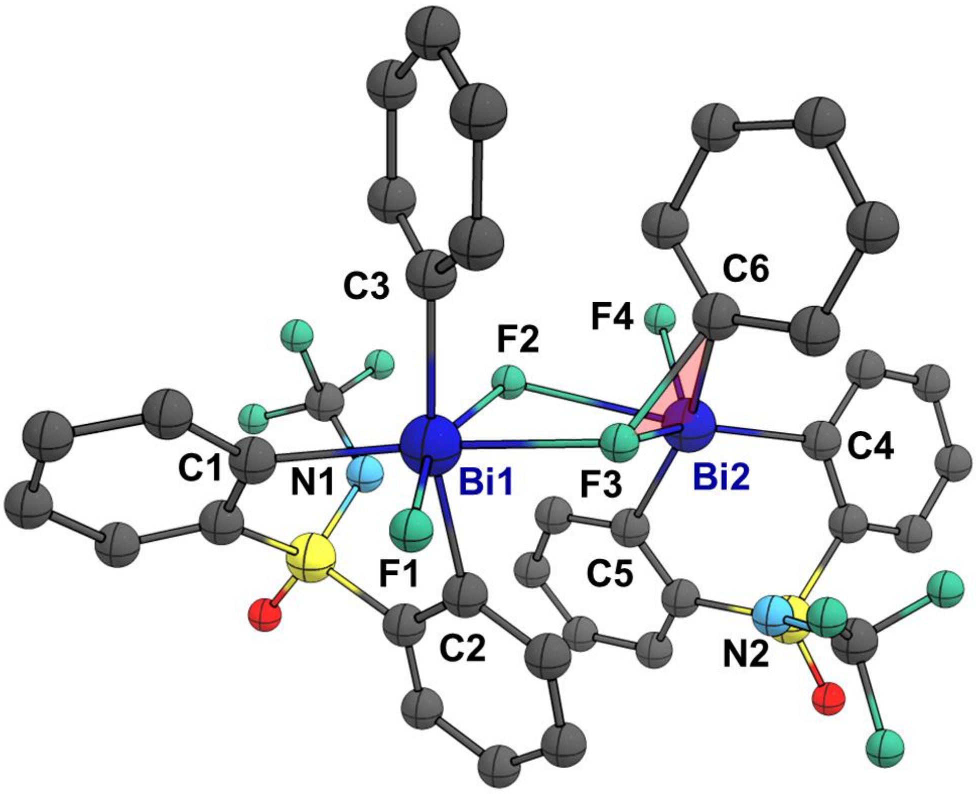

Figure S127. Optimized structure of **TSF3**.

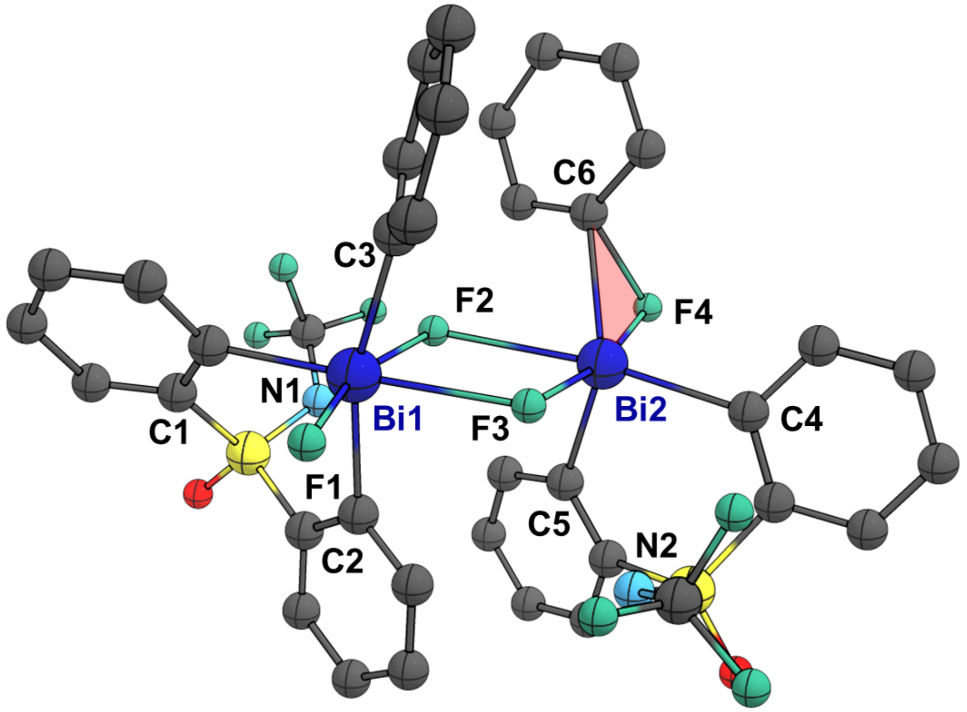

Figure S128. Optimized structure of **TSF4**.

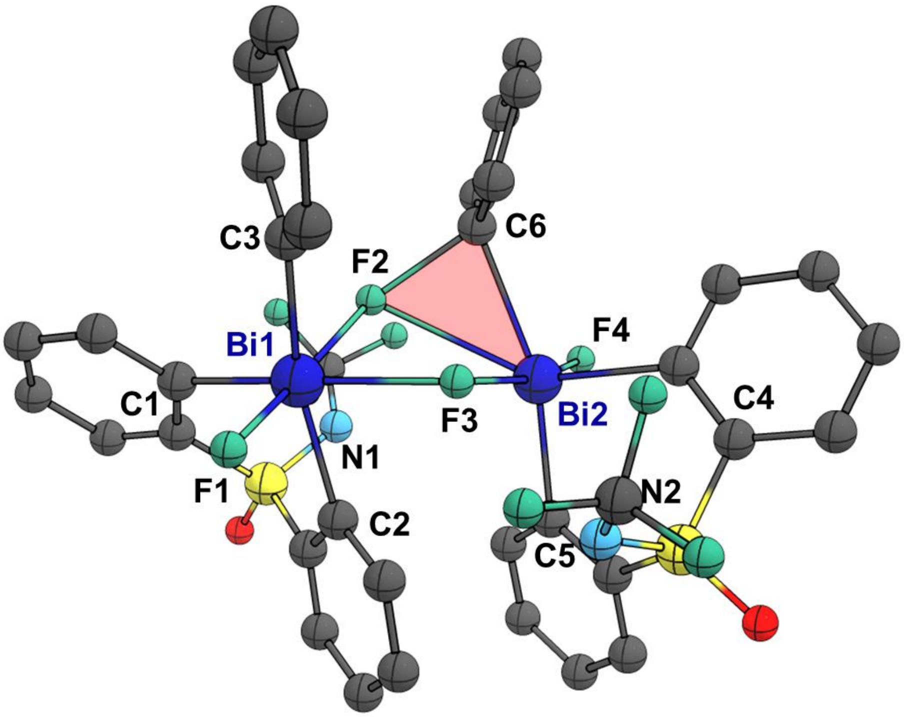

Figure S129. Optimized structure of **TSF2**.

9.3 Reductive elimination from monomeric species ***int-cis*** and ***int-trans***

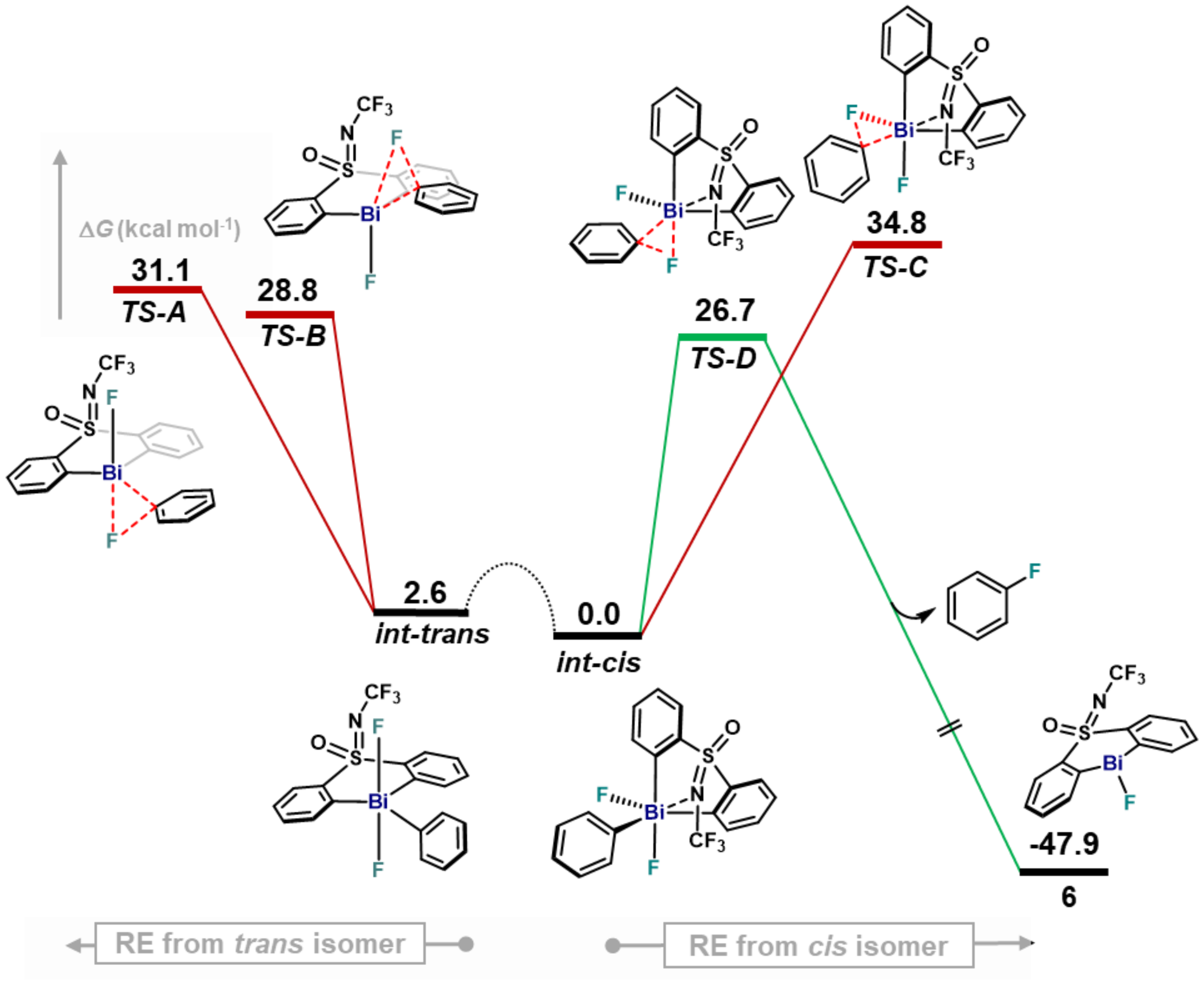

Figure S130. Gibbs energy profile of the reductive elimination of fluorobenzene from monomeric species ***int-cis*** and ***int-trans***. Relative Gibbs energy values are given in kcal mol^{-1}. Note: most stable species in this system is dimer **4** with Gibbs energy value of –7.6 kcal mol^{-1} relative to two ***int-cis*** molecules.

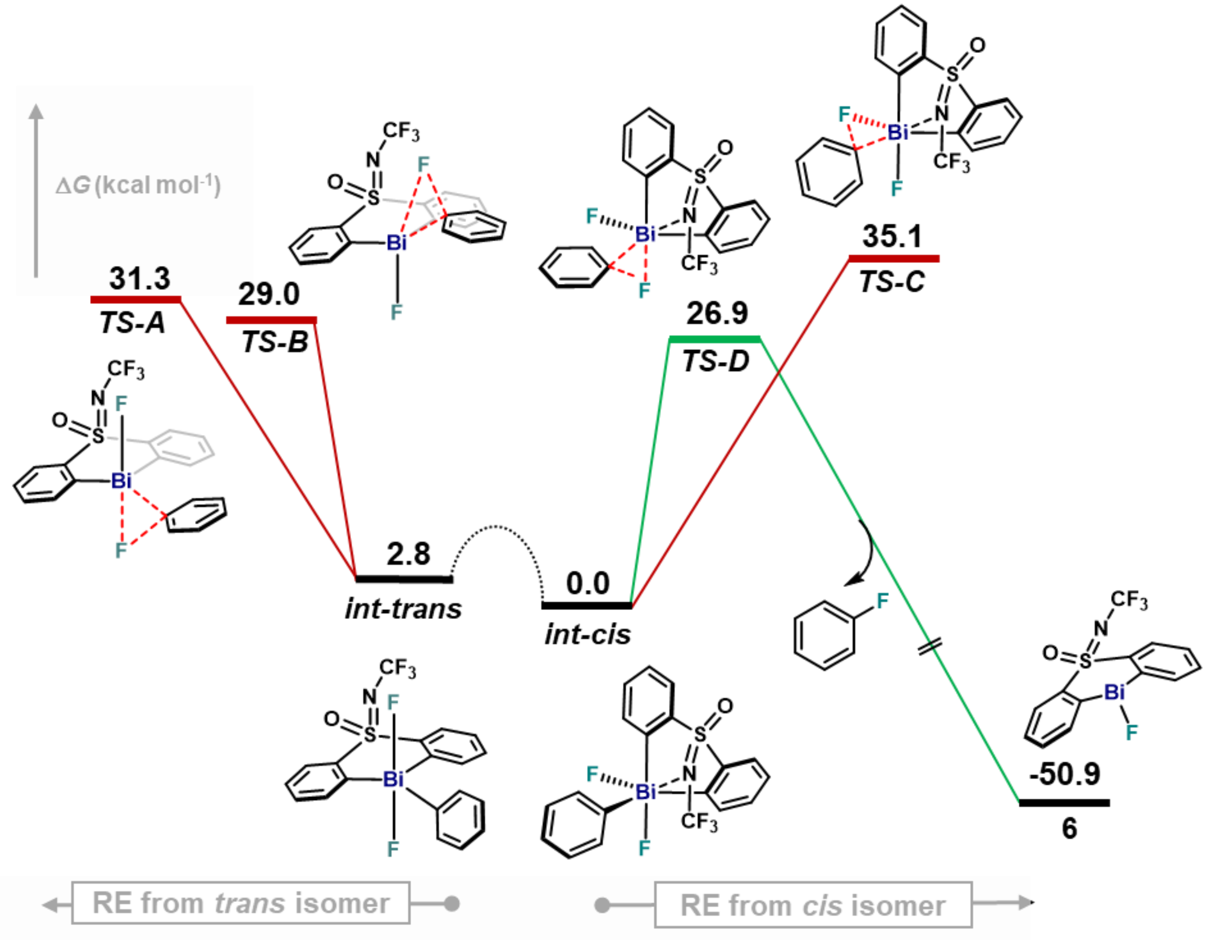

Figure S131. Gibbs energy profile of the reductive elimination of fluorobenzene from monomeric species ***int-cis*** and ***int-trans***. Relative Gibbs energy values are given in kcal mol^{-1}. Thermal corrections calculated at 363 K. Note: most stable species in this system is dimer **4** with Gibbs energy value of –3.7 kcal mol^{-1} relative to two ***int-cis*** molecules.

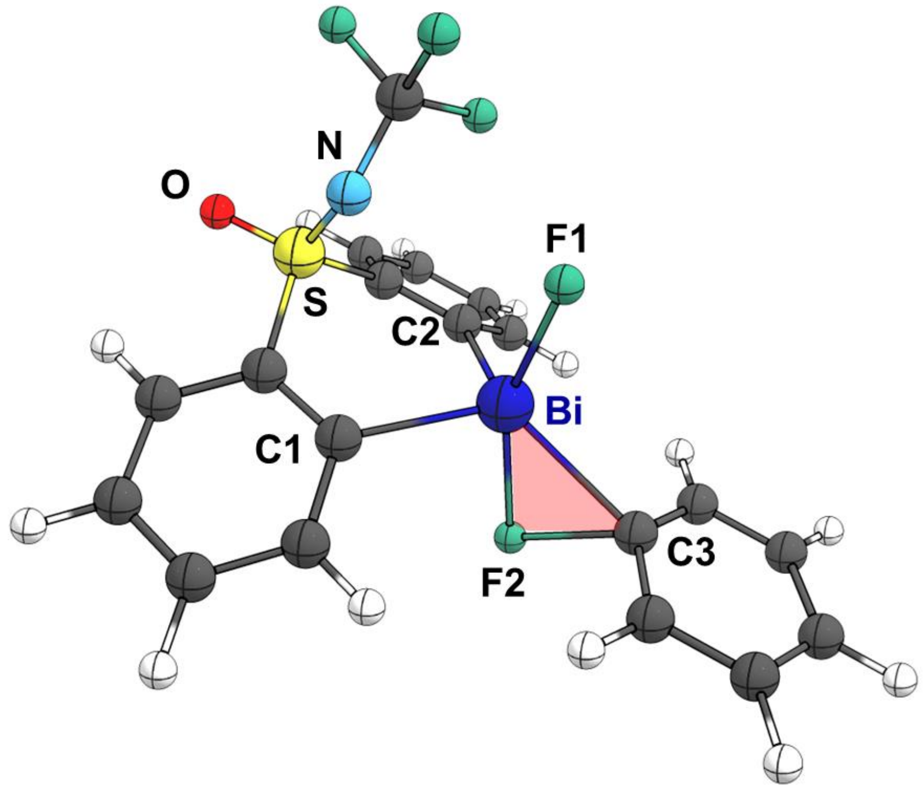

Figure S132. Optimized structure of **TS-A**.

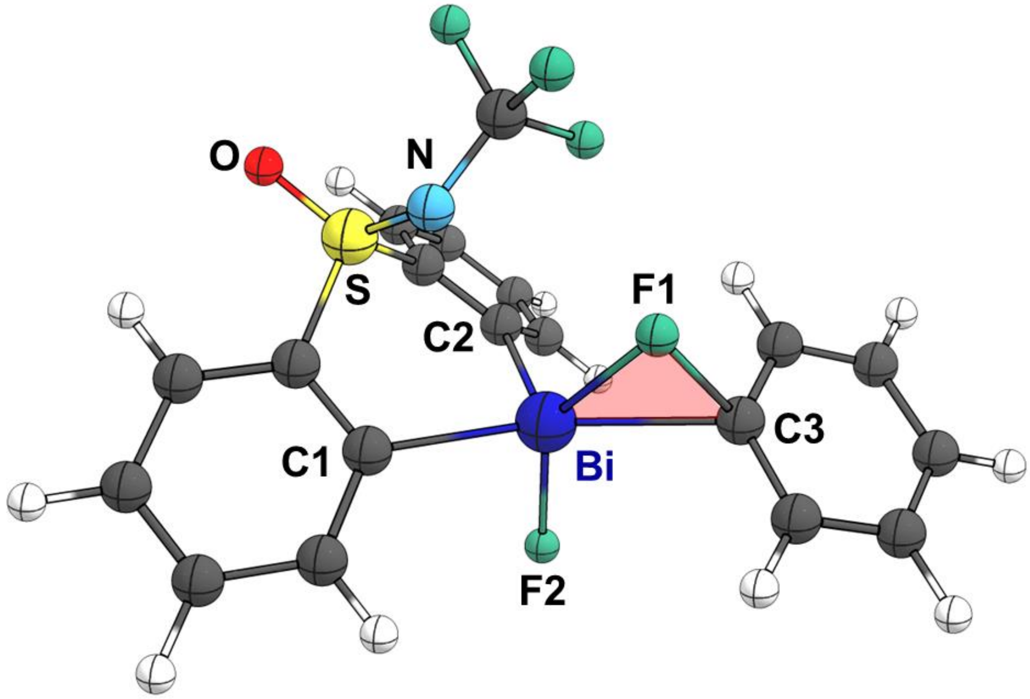

Figure S133. Optimized structure of **TS-B**.

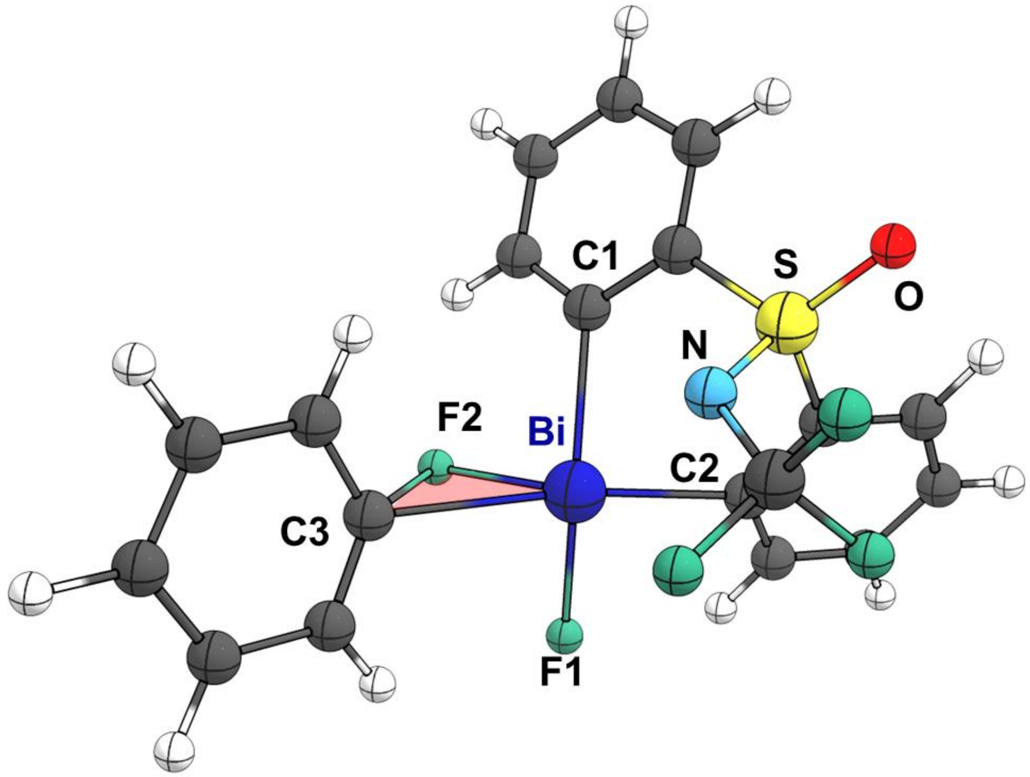

Figure S134. Optimized structure of **TS-C**.

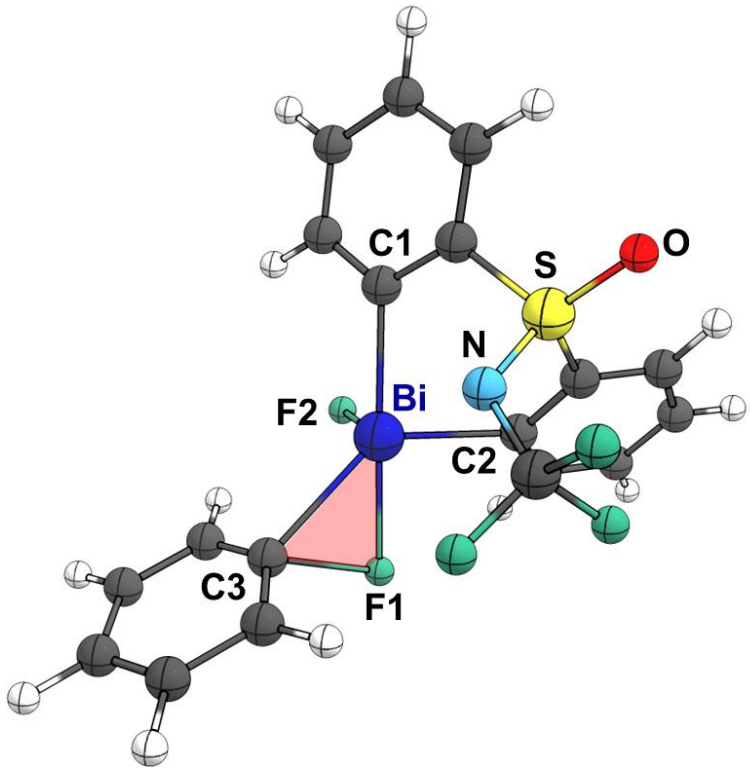

Figure S135. Optimized structure of **TS-D**.

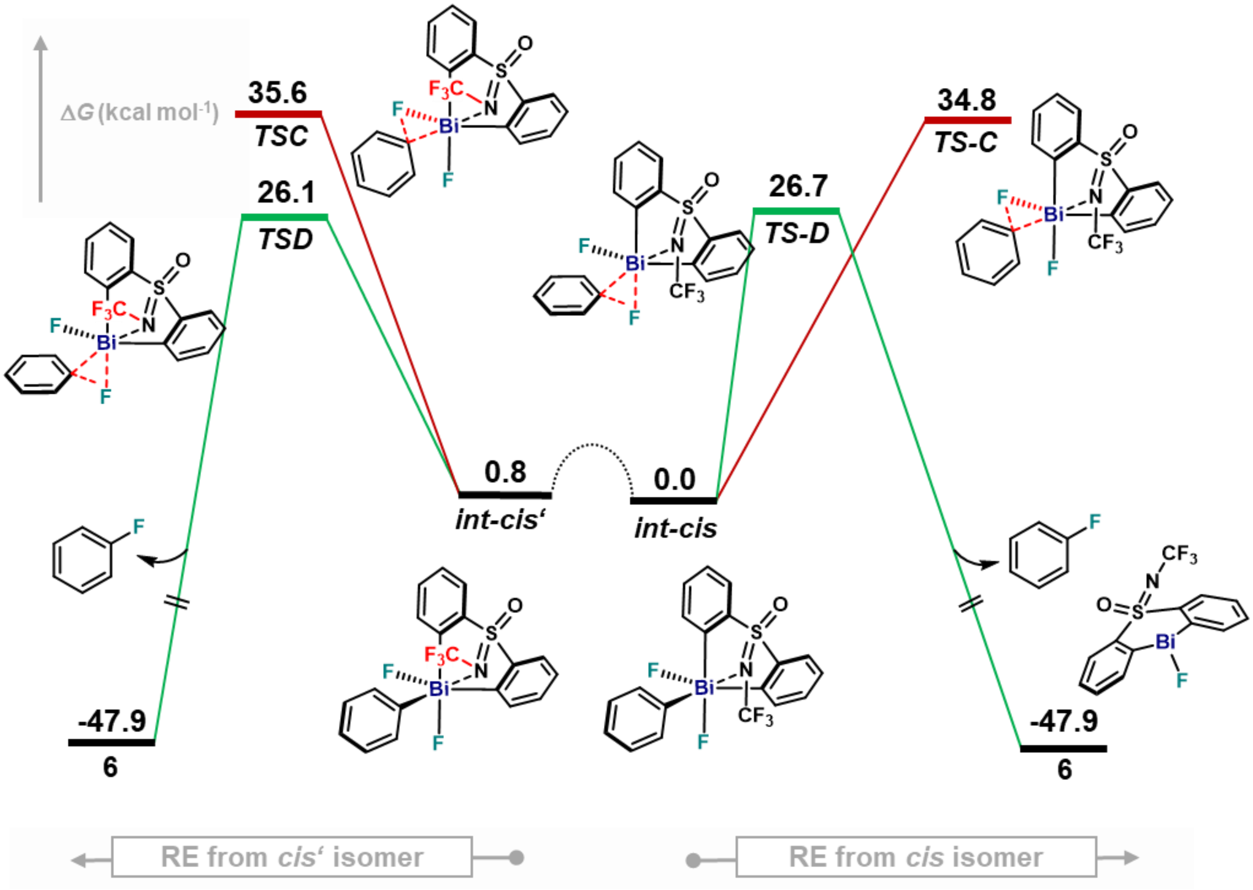

Figure S136. Gibbs energy profile of the reductive elimination of fluorobenzene from monomeric species ***int-cis***, including isomer ***int-cis'***. Relative Gibbs energy values are given in kcal mol^{-1}. Note: most stable species in this system is dimer **4** with Gibbs energy value of –7.6 kcal mol^{-1} relative to two ***int-cis*** molecules.

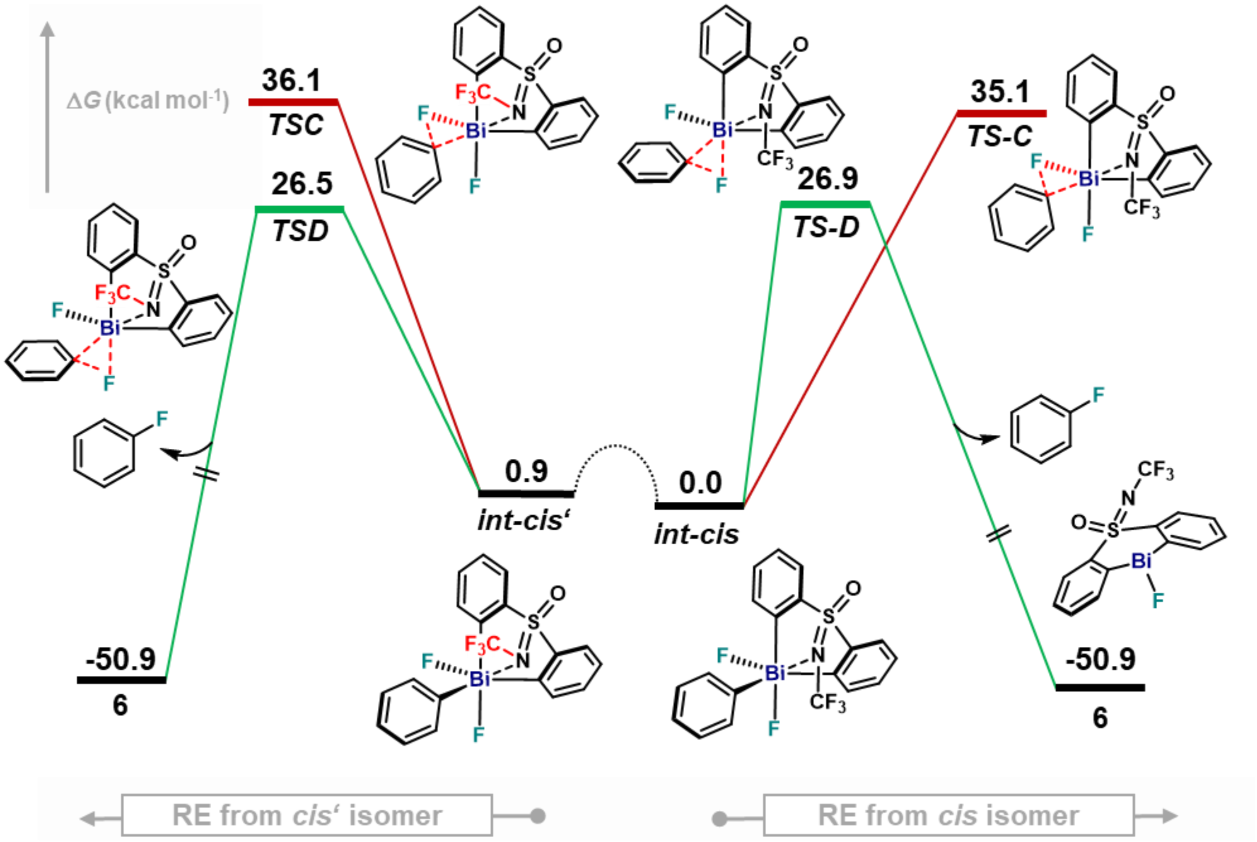

Figure S137. Gibbs energy profile of the reductive elimination of fluorobenzene from monomeric species ***int-cis***, including isomer ***int-cis'***. Relative Gibbs energy values are given in kcal mol^{-1}. Thermal corrections calculated at 363 K. Note: most stable species in this system is dimer **4** with Gibbs energy value of –3.7 kcal mol^{-1} relative to two ***int-cis*** molecules.

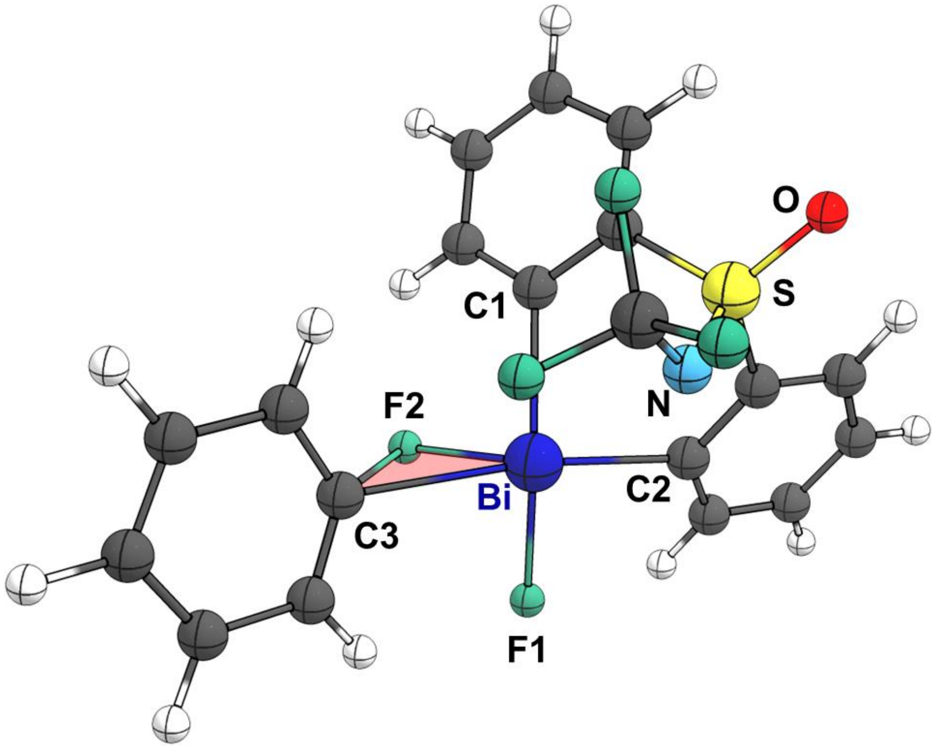

Figure S138. Optimized structure of **TSC**.

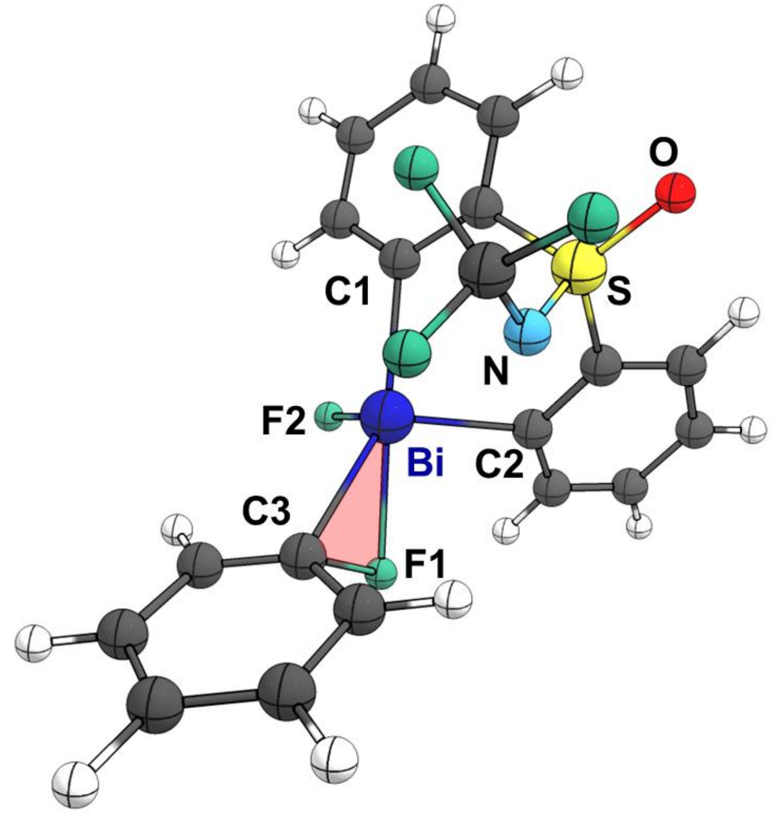

Figure S139. Optimized structure of **TSD**.

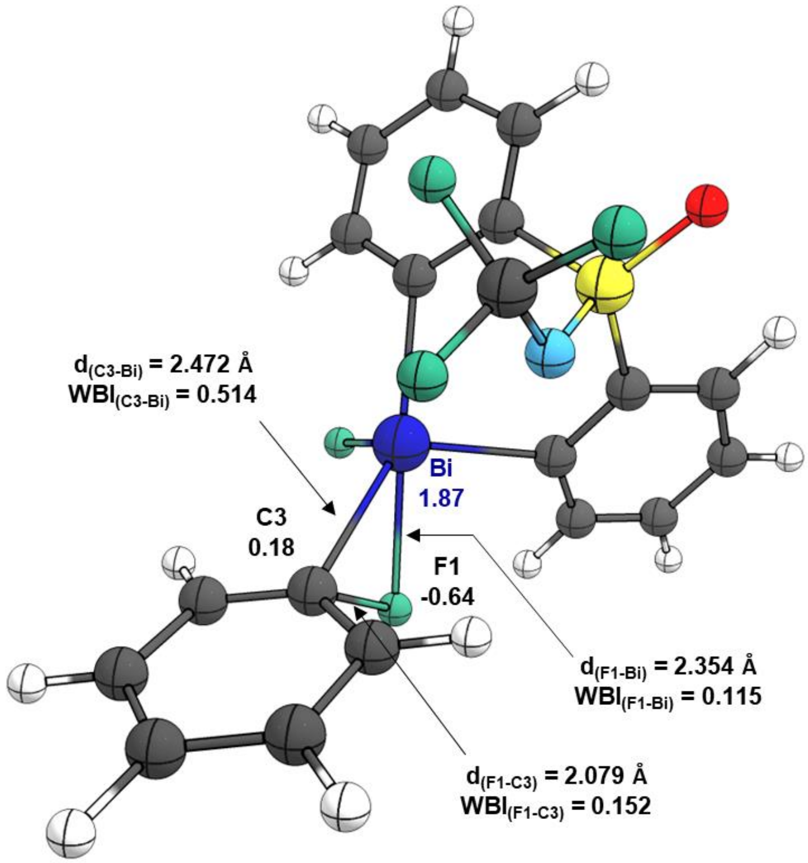

Figure S140. NBO analysis showing selected charges (q), bond orders (WBI) and distances (d) of **TSD**.

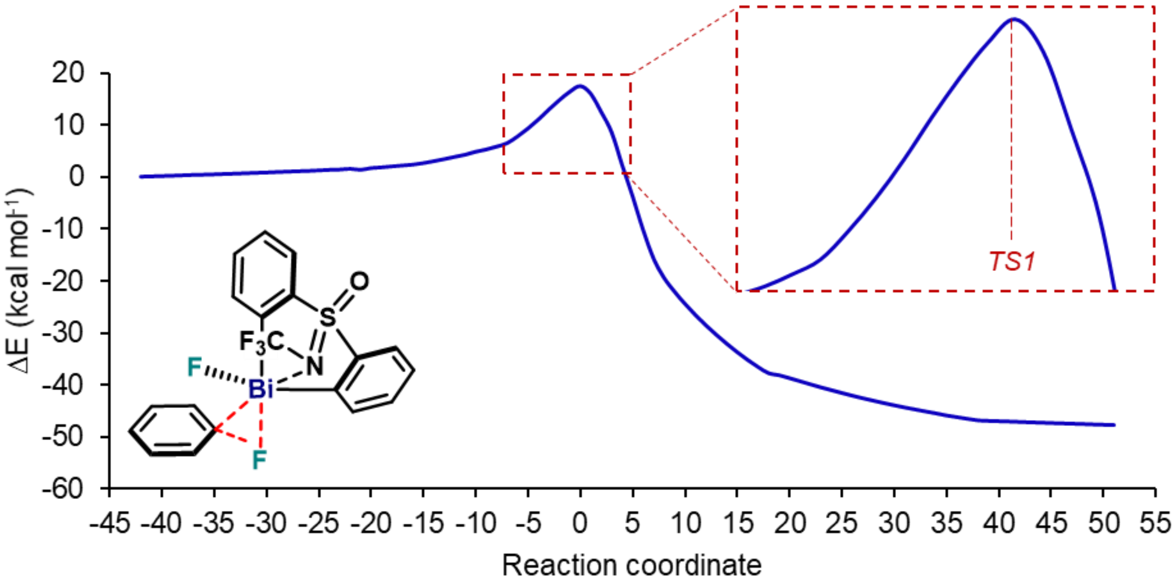

Figure S141. Intrinsic reaction coordinate (IRC) for **TSD** showing a *concerted synchronous* reductive elimination step.

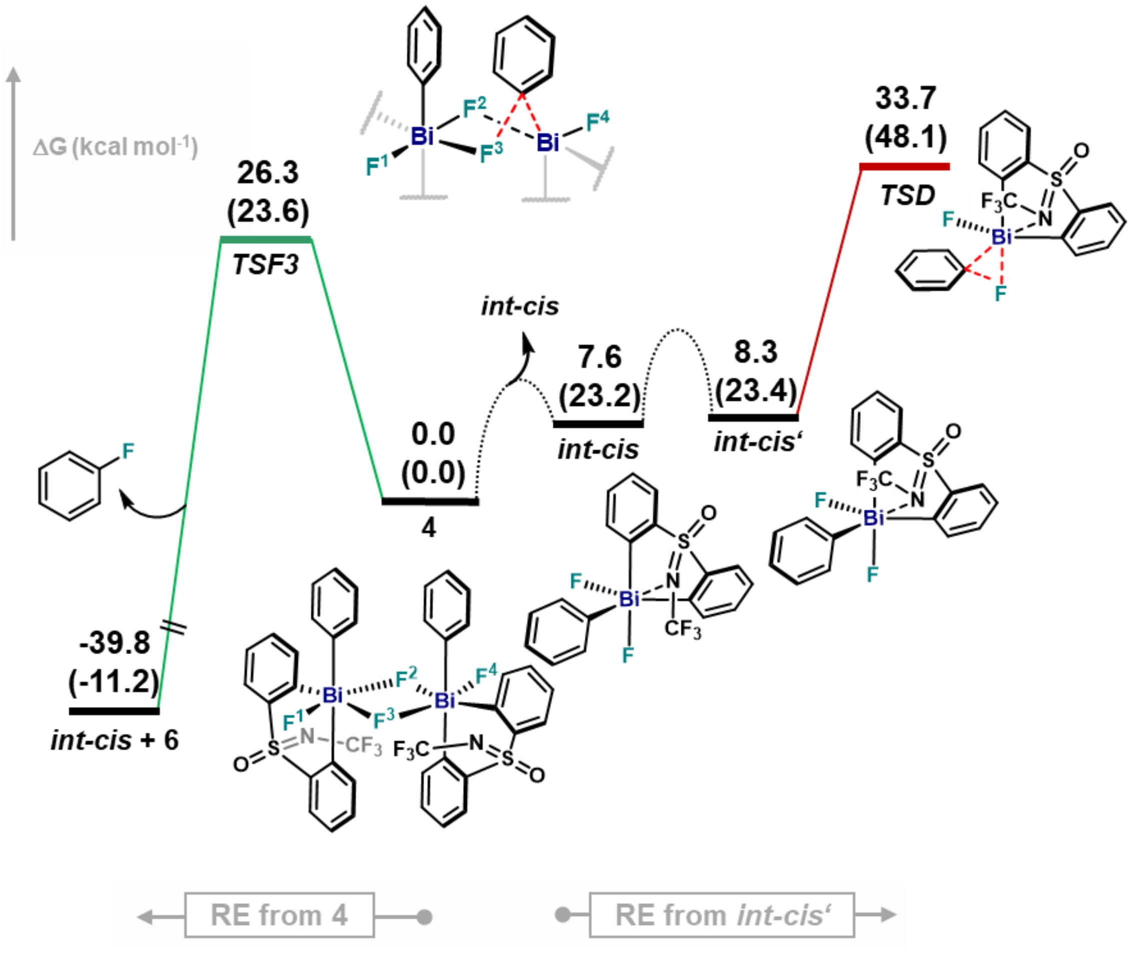

Figure S142. Gibbs energy profile with respective enthalpy values (in parentheses) of the reductive elimination of fluorobenzene from dimeric species **4**, including isomer ***int-cis'***. Relative Gibbs energy and enthalpy values are given in kcal mol^{-1}.

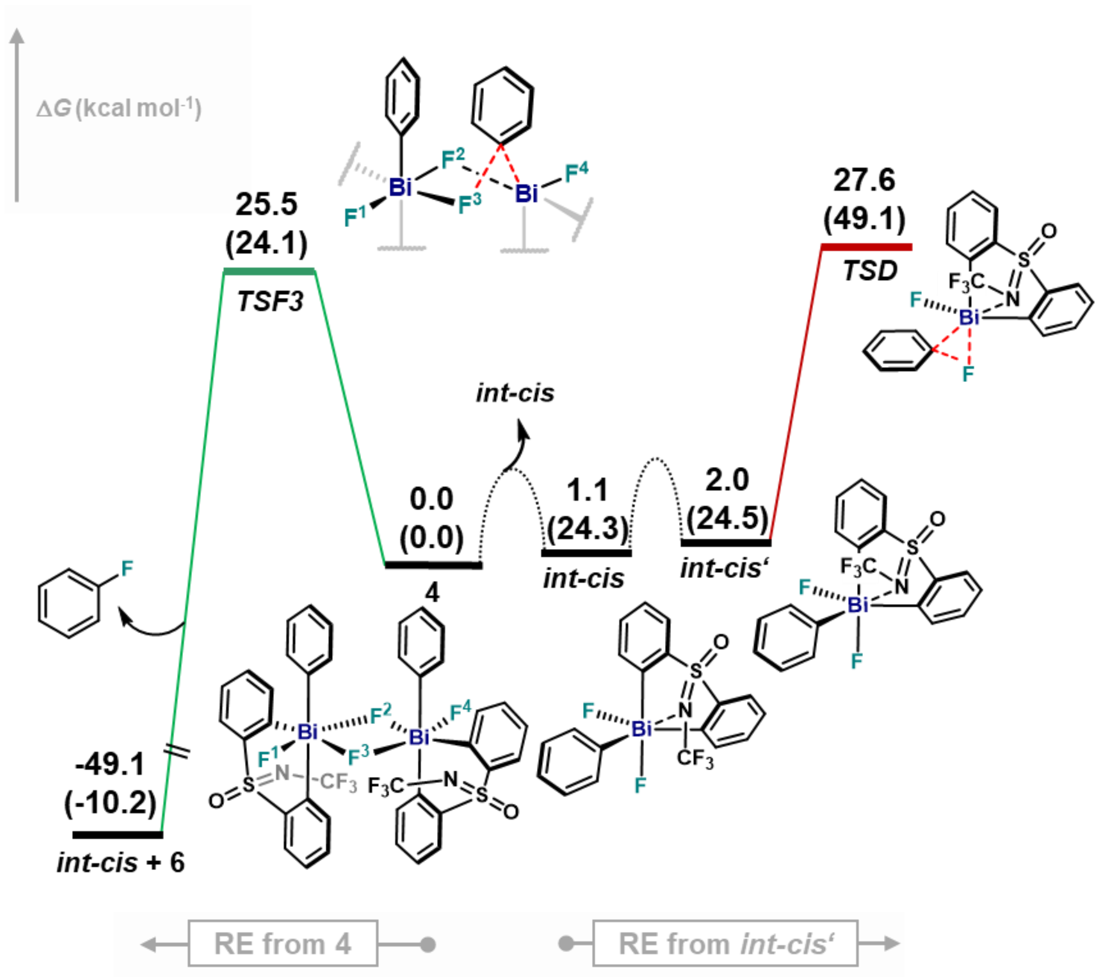

Figure S143. Gibbs energy profile with respective enthalpy values (in parentheses) of the reductive elimination of fluorobenzene from dimeric species **4**, including isomer ***int-cis'***. Relative Gibbs energy and enthalpy values are given in kcal mol^{-1}. Thermal corrections calculated at 363 K.

9.4 Reductive elimination from monomeric species **26**

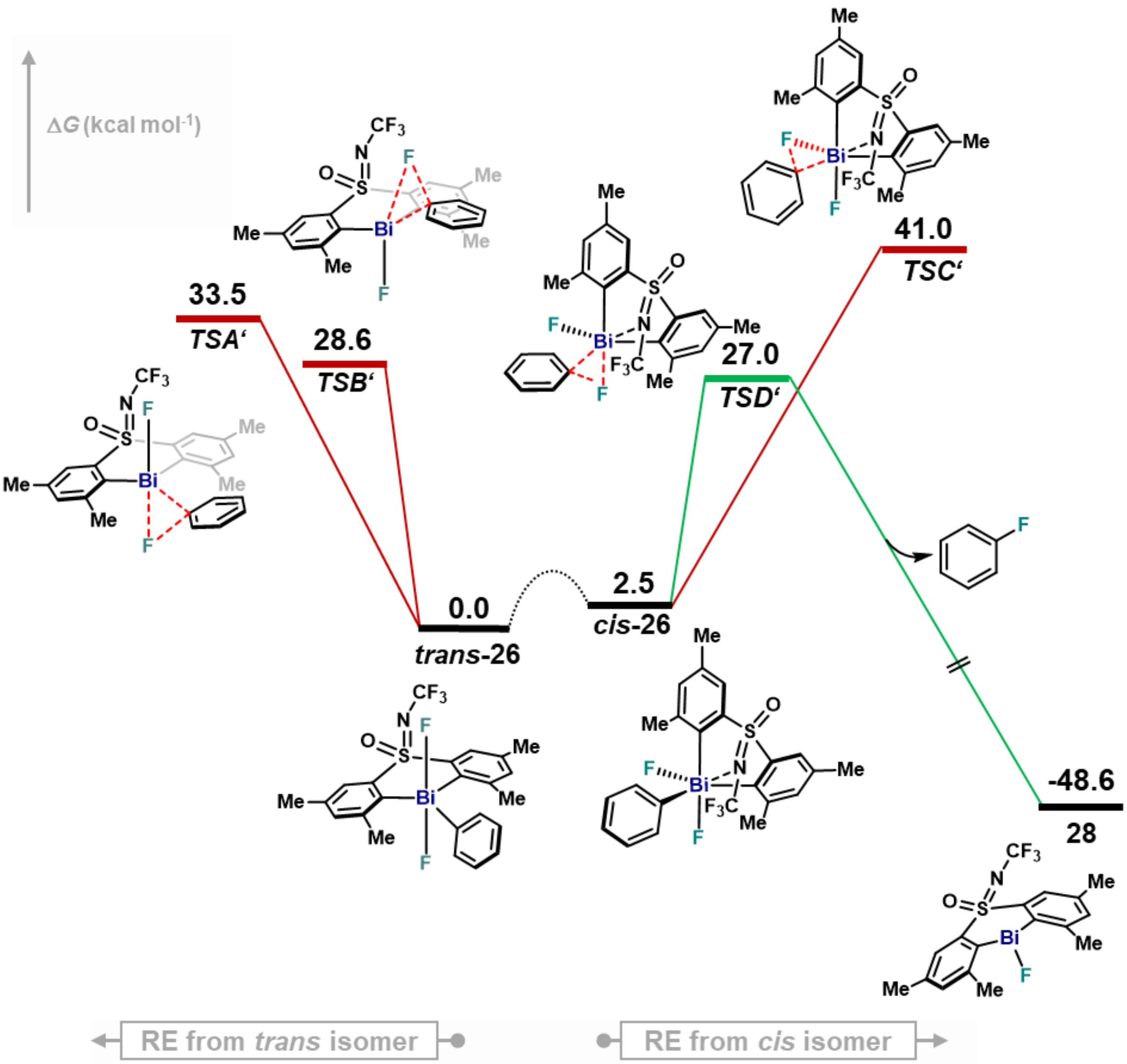

Figure S144. Gibbs energy profile of the reductive elimination of fluorobenzene from monomeric species **26**. Relative Gibbs energy values are given in kcal mol^{-1}.

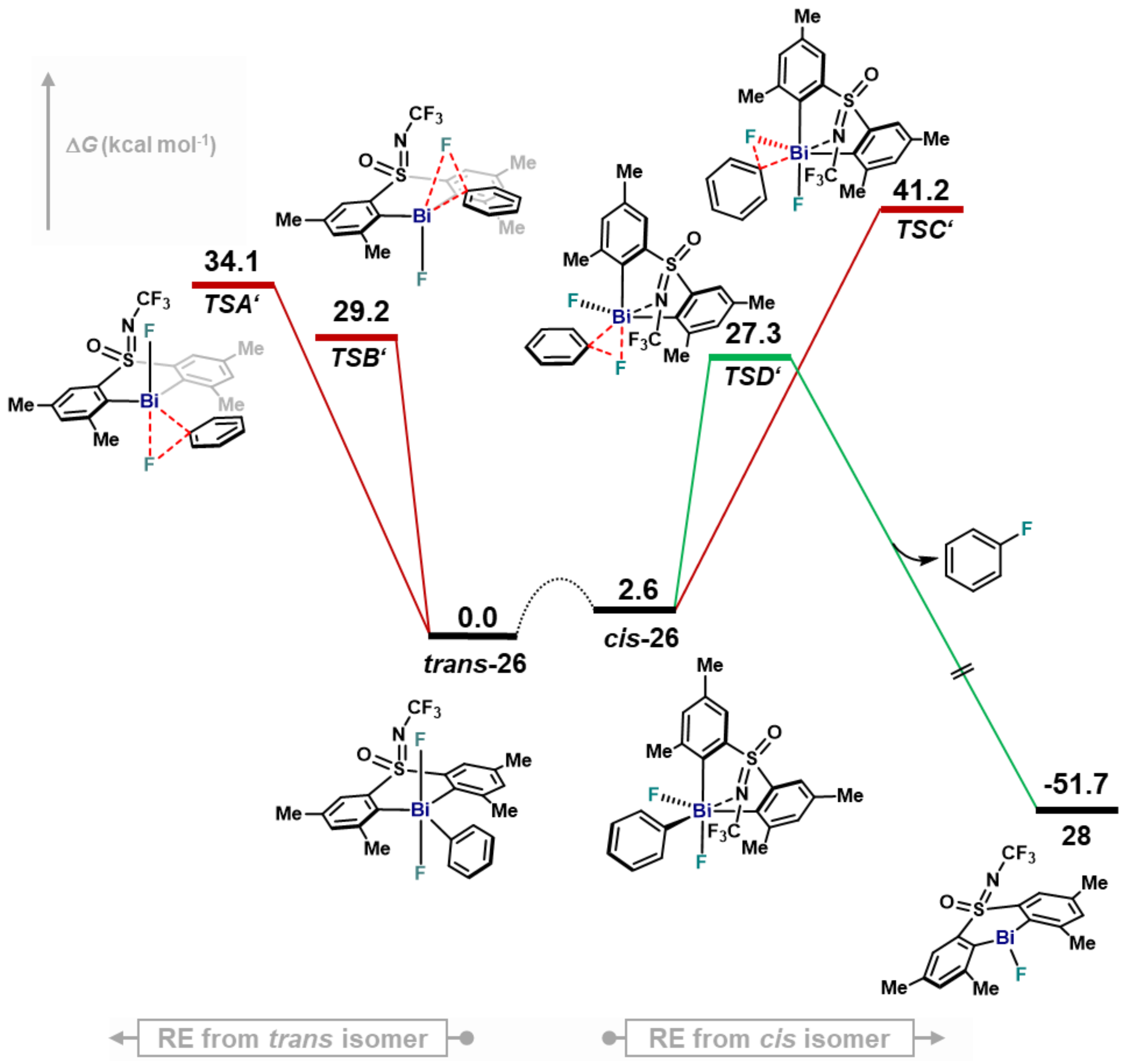

Figure S145. Gibbs energy profile of the reductive elimination of fluorobenzene from monomeric species **26**. Relative Gibbs energy values are given in kcal mol^{-1}. Thermal corrections calculated at 363 K.

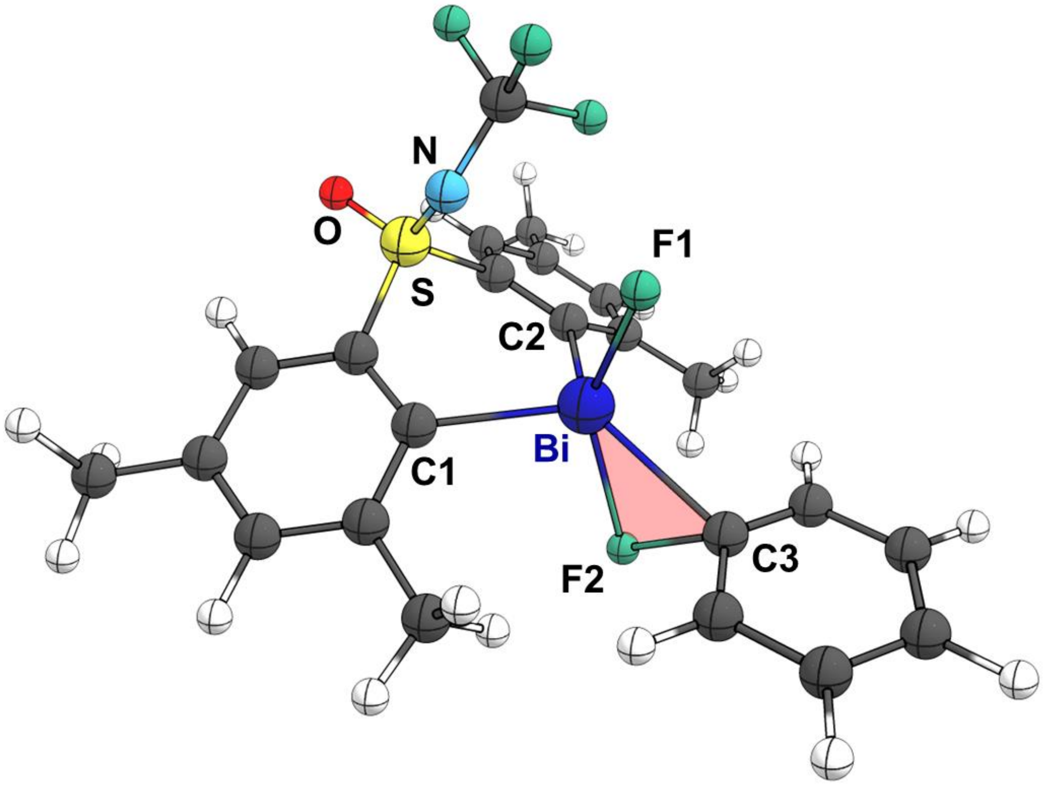

Figure S146. Optimized structure of **TSA'**.

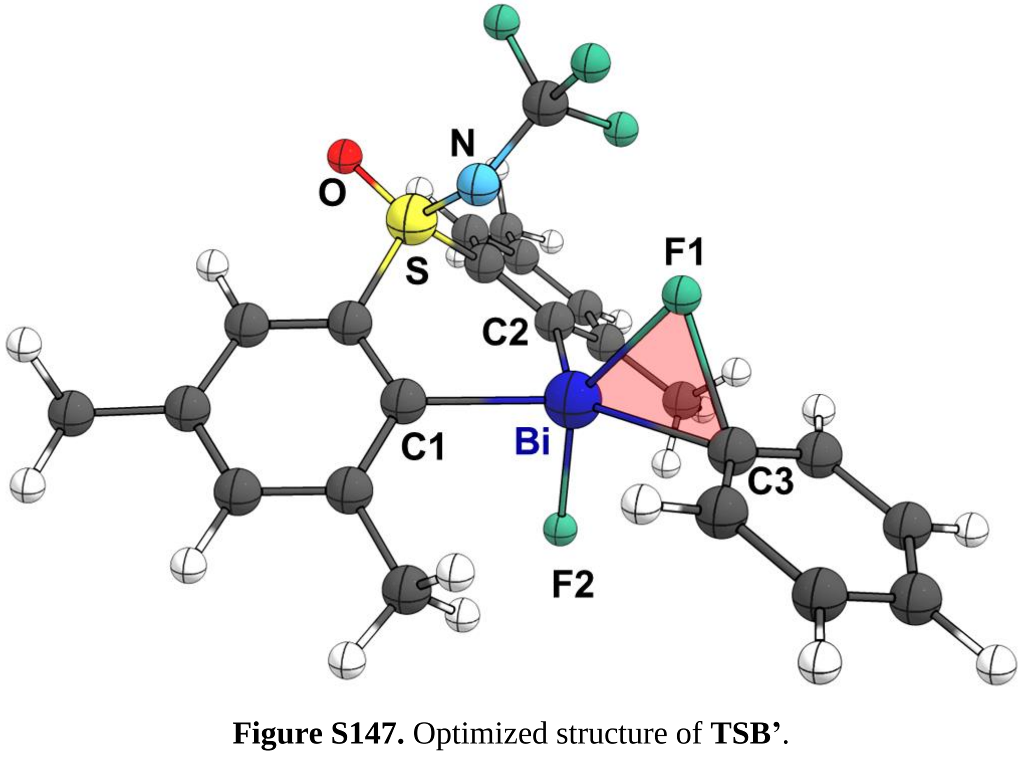

Figure S147. Optimized structure of **TSB'**.

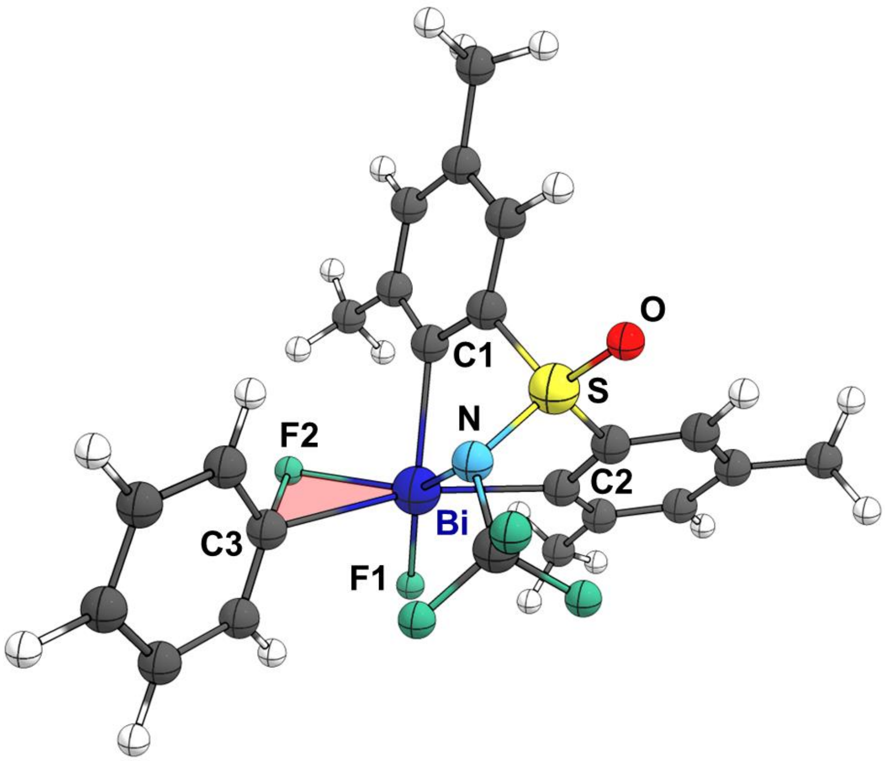

Figure S148. Optimized structure of **TSC'**.

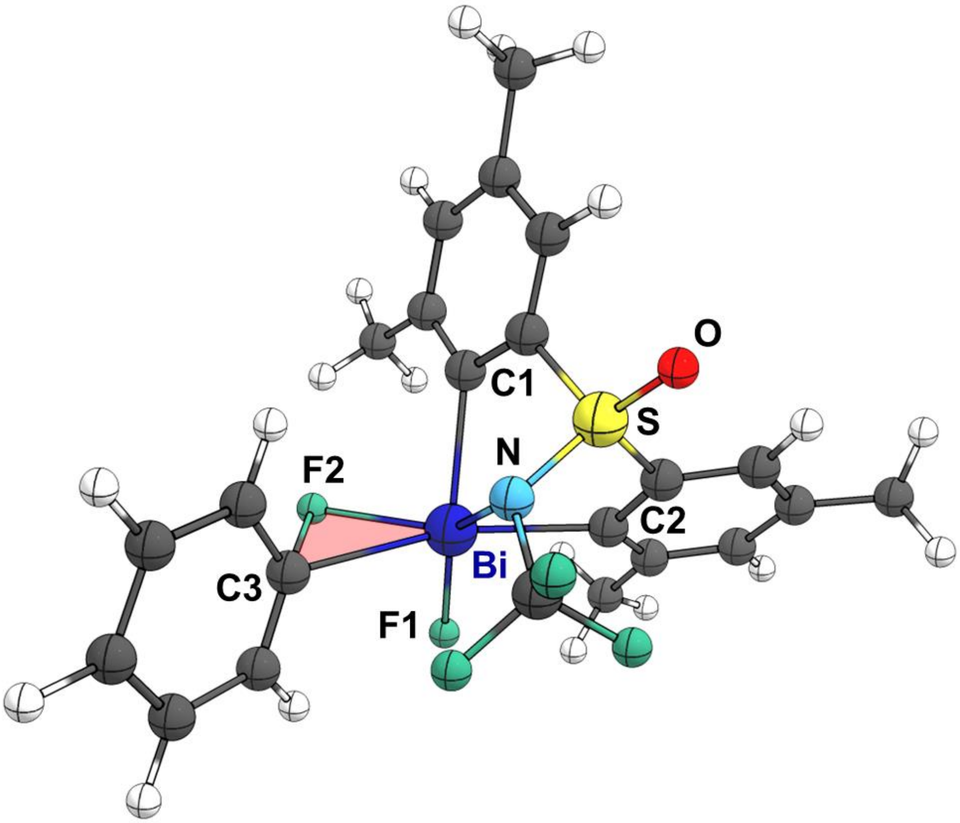

Figure S149. Optimized structure of **TSD'**.

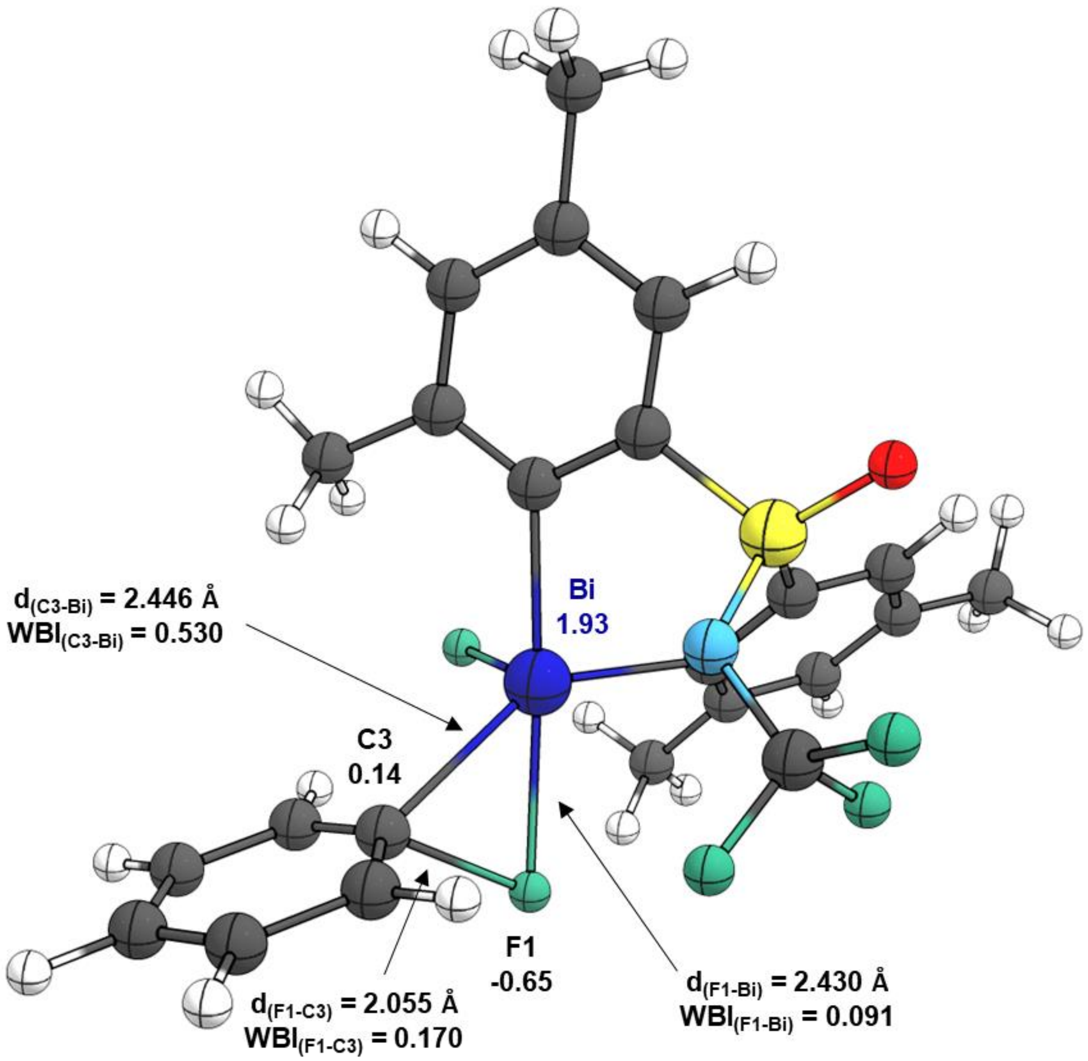

Figure S150. NBO analysis showing selected charges (q), bond orders (WBI) and distances (d) of **TSD'**.

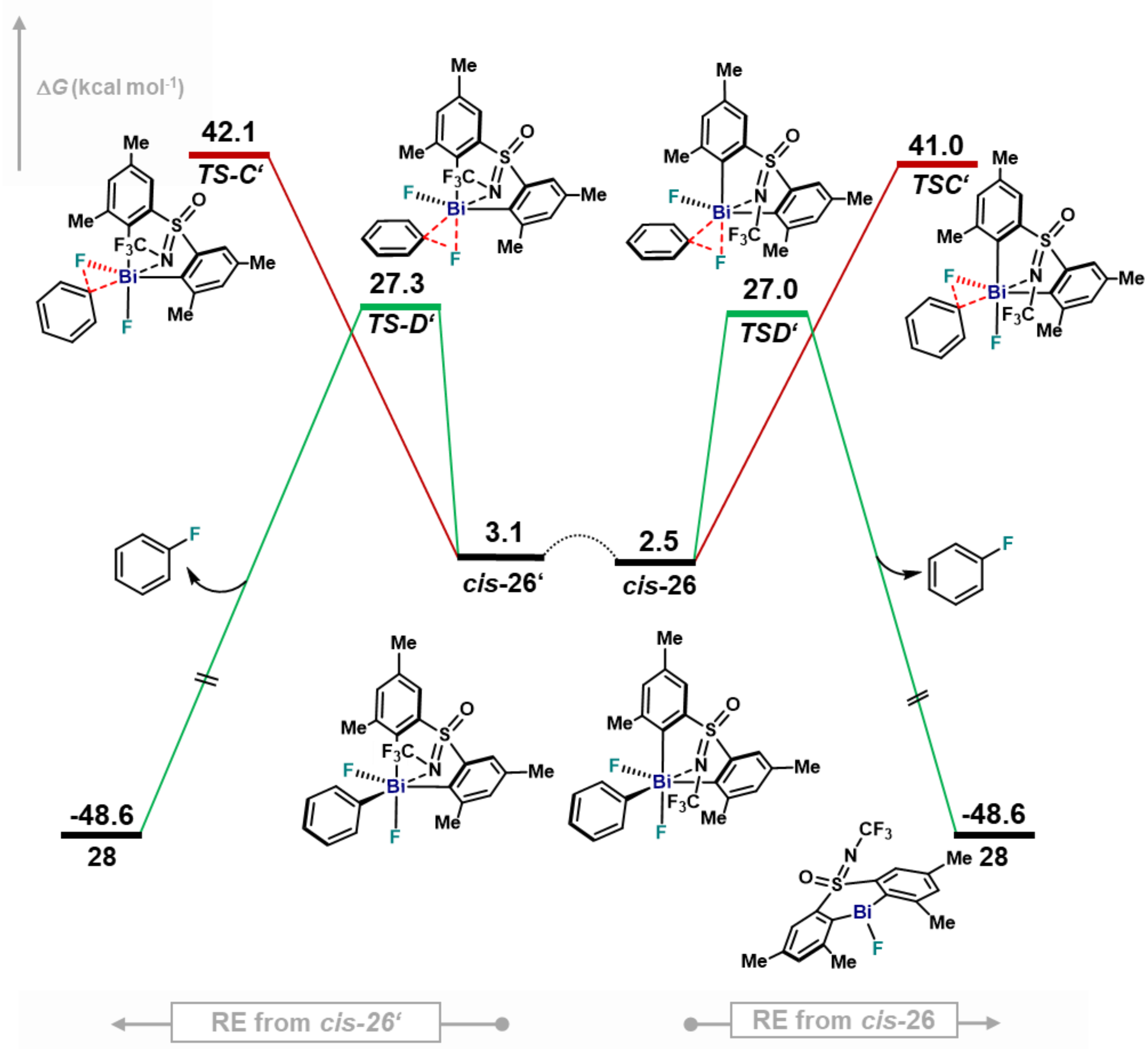

Figure S151. Gibbs energy profile of the reductive elimination of fluorobenzene from monomeric species ***cis*-26**, including isomer ***cis*-26'**. Relative Gibbs energy values are given in kcal mol^{-1}.

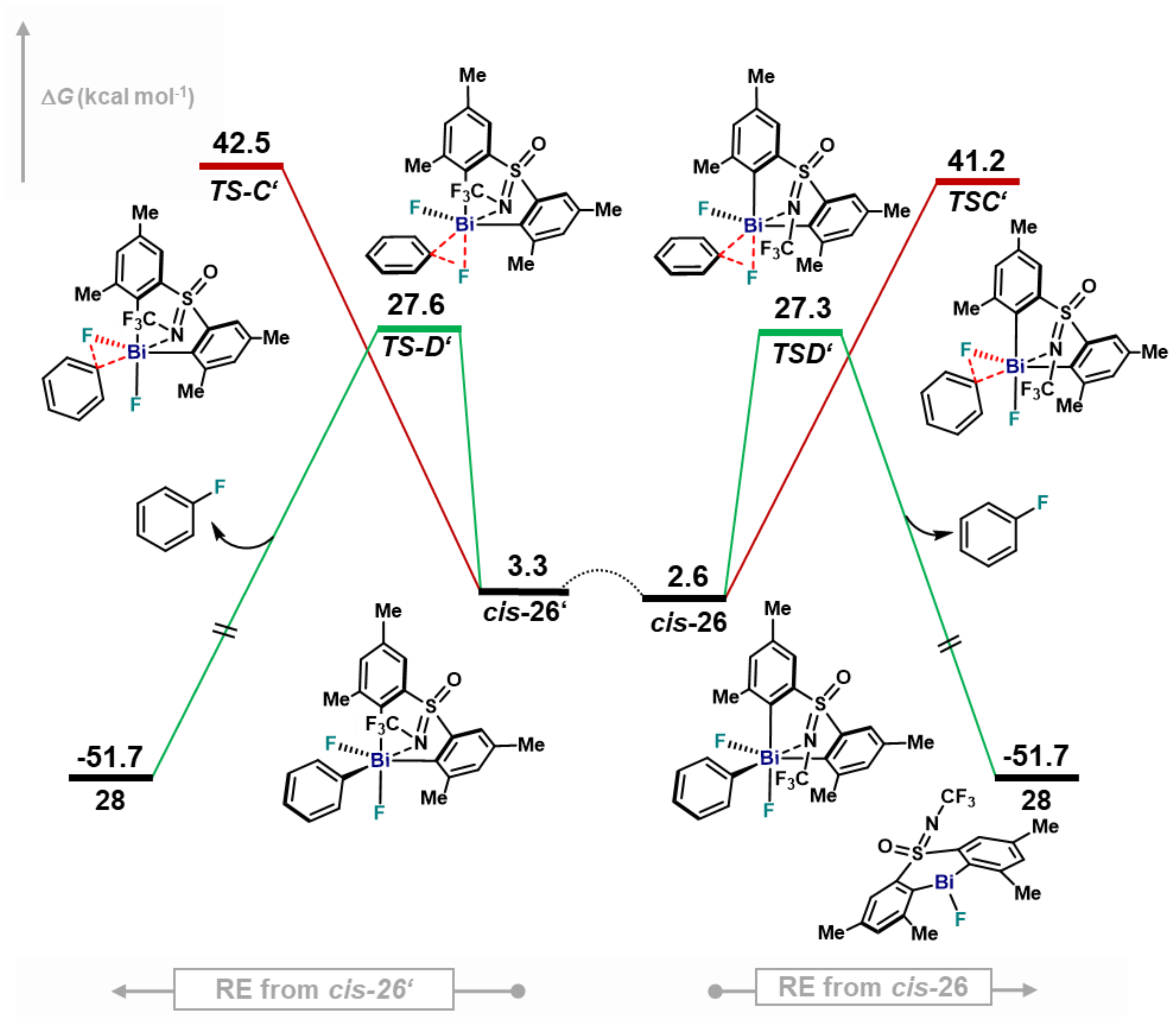

Figure S152. Gibbs energy profile of the reductive elimination of fluorobenzene from monomeric species ***cis*-26**, including isomer ***cis*-26'**. Relative Gibbs energy values are given in kcal mol^{-1}. Thermal corrections calculated at 363 K.

9.5 Reductive elimination from fluorobismuthonium species ***cis*-36**

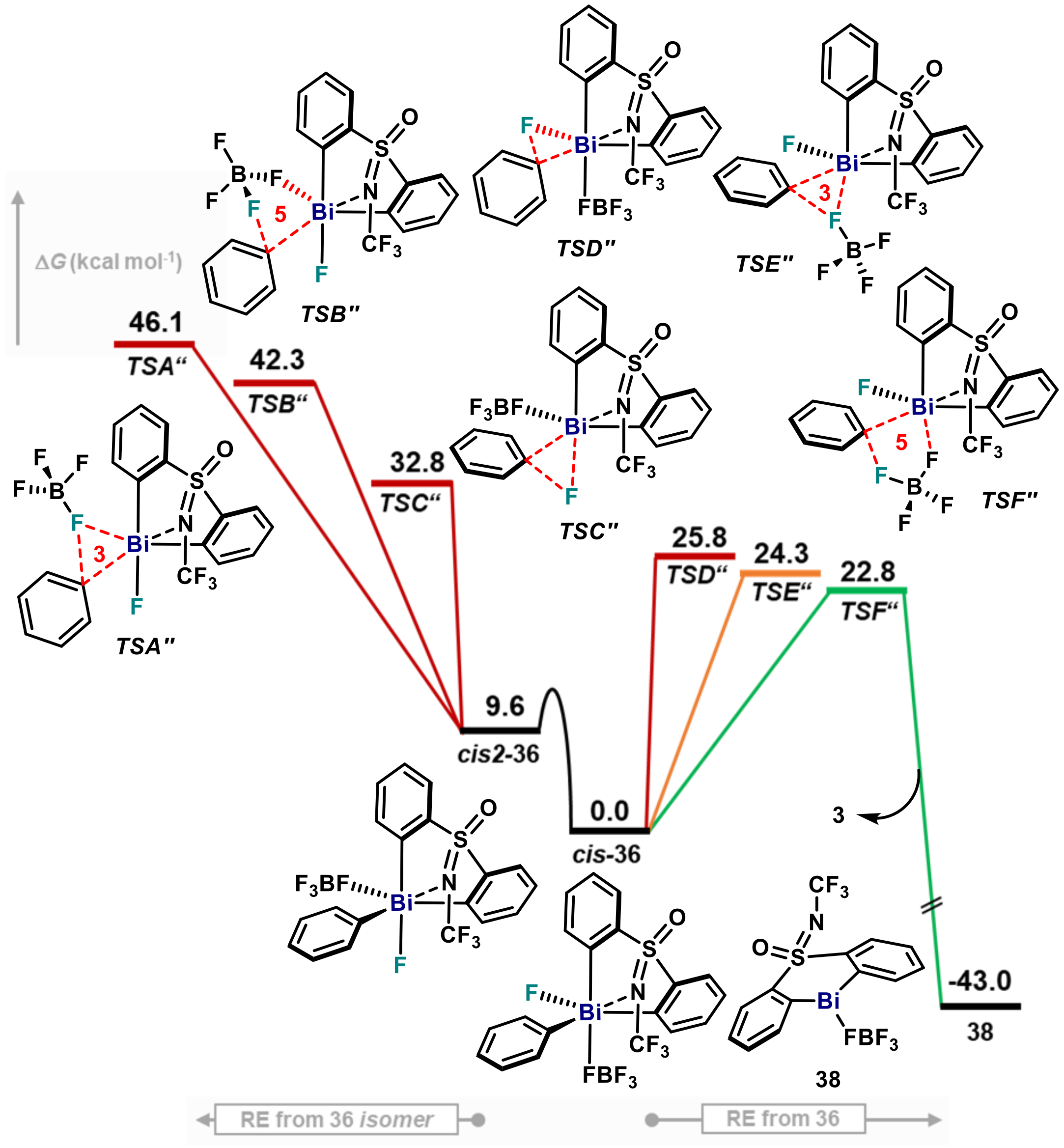

Figure S153. Gibbs energy profile of the reductive elimination of fluorobenzene from fluorobismuthonium species ***cis*-36**. Relative Gibbs energy values are given in kcal mol^{-1}.

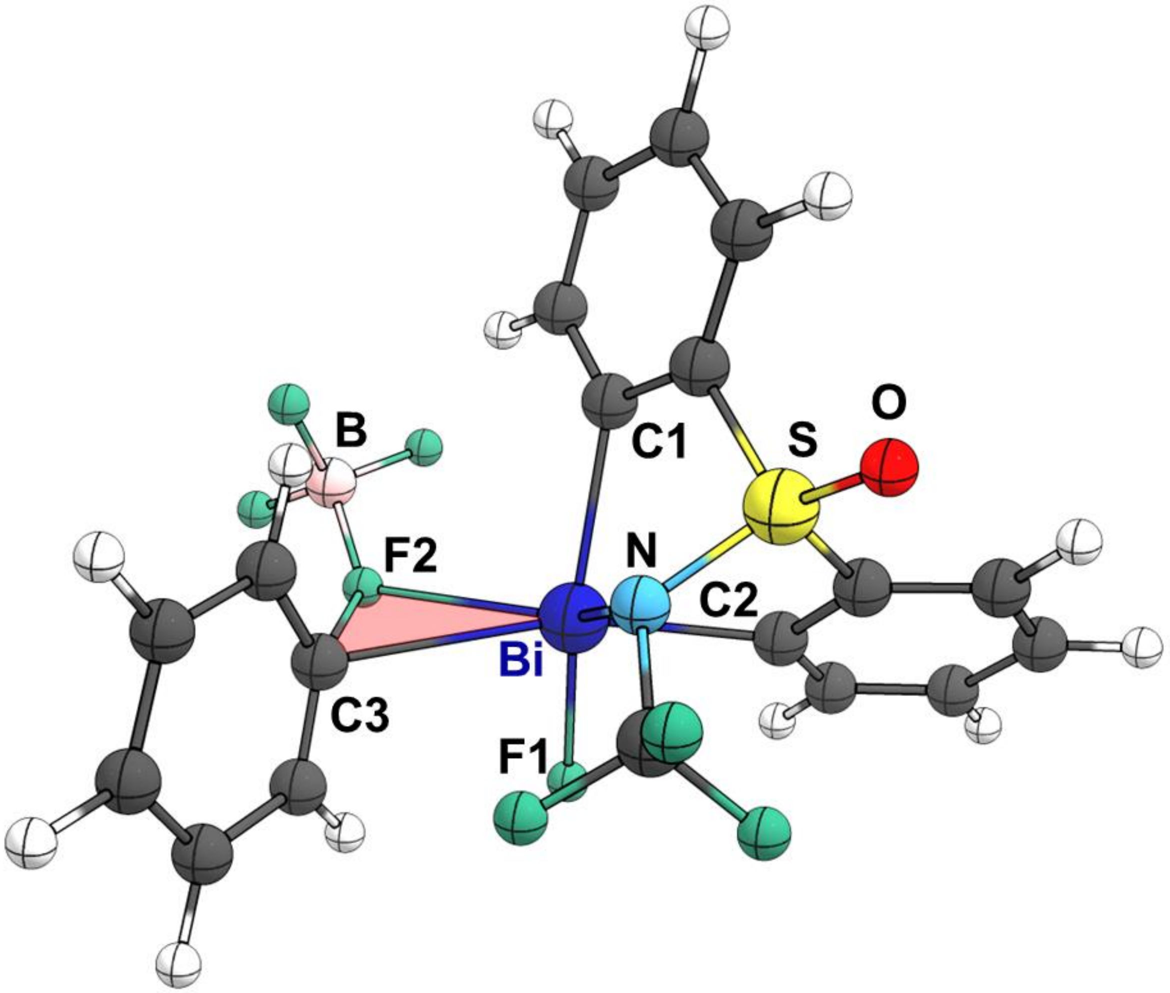

Figure S154. Optimized structure of **TSA''**.

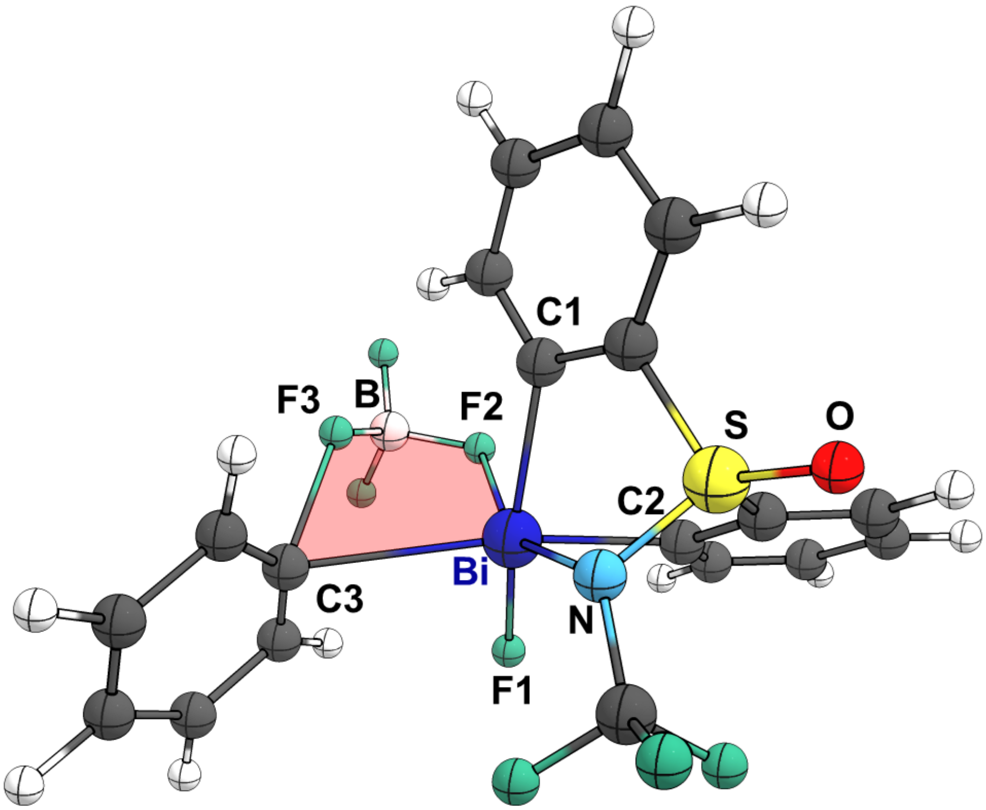

Figure S155. Optimized structure of **TSB''**.

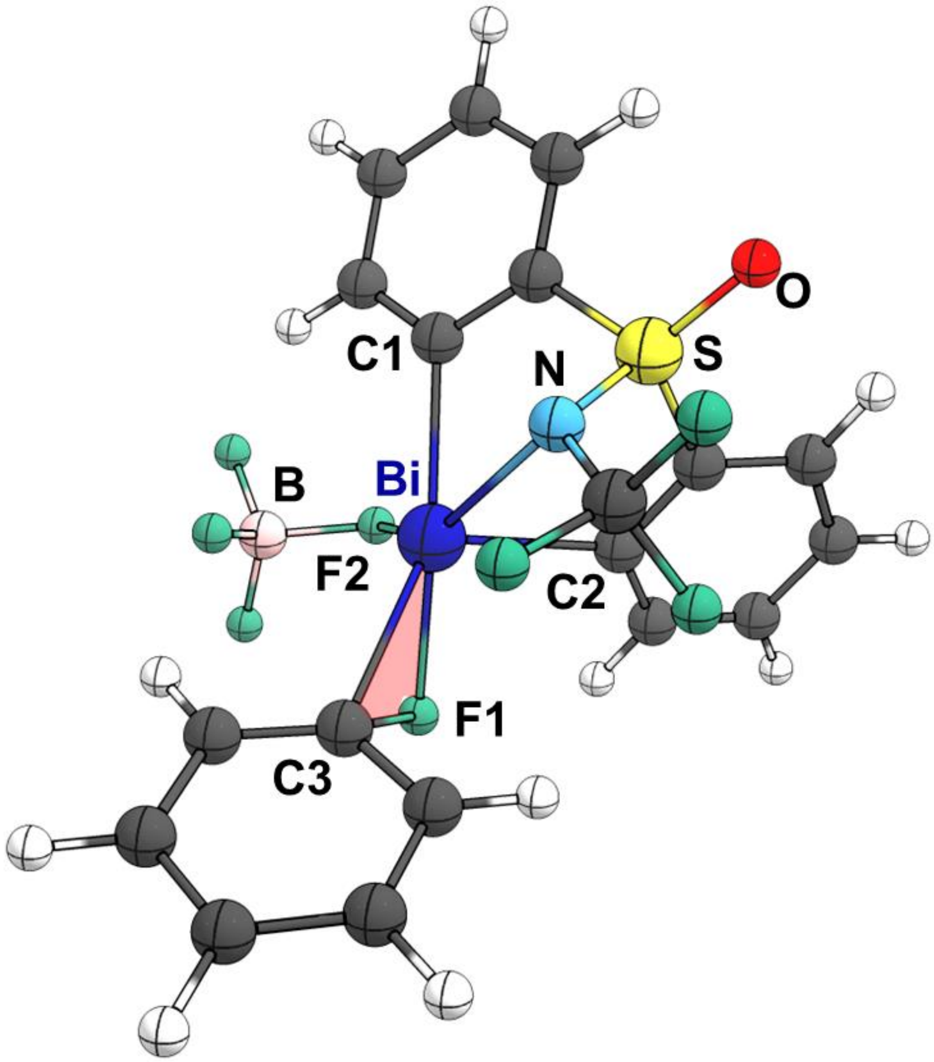

Figure S156. Optimized structure of **TSC''**.

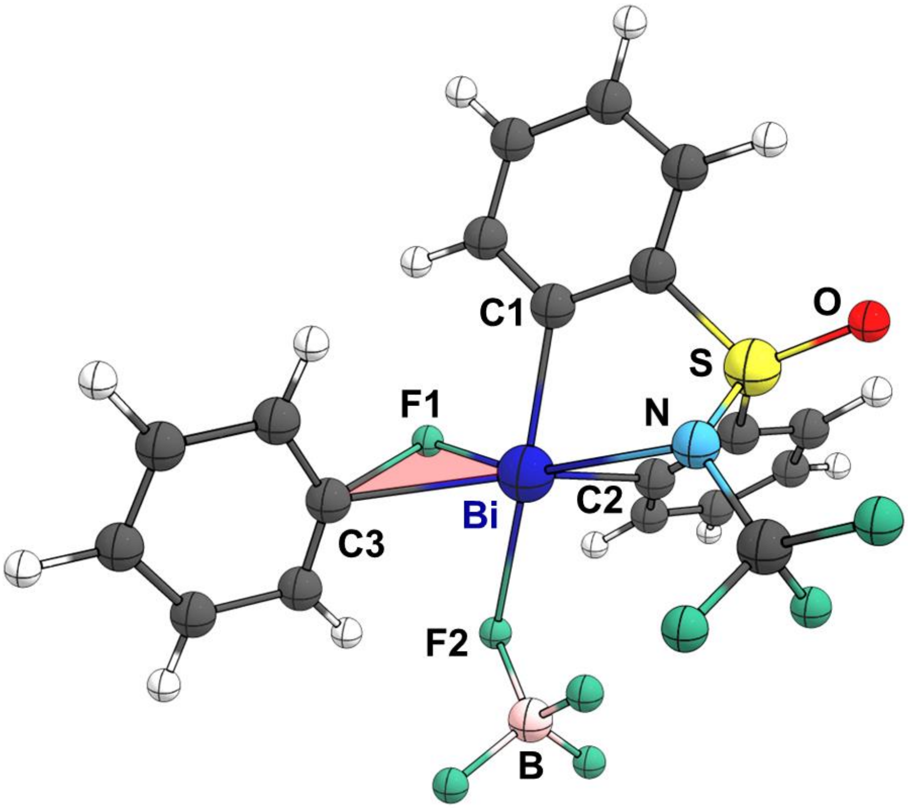

Figure S157. Optimized structure of **TSD''**.

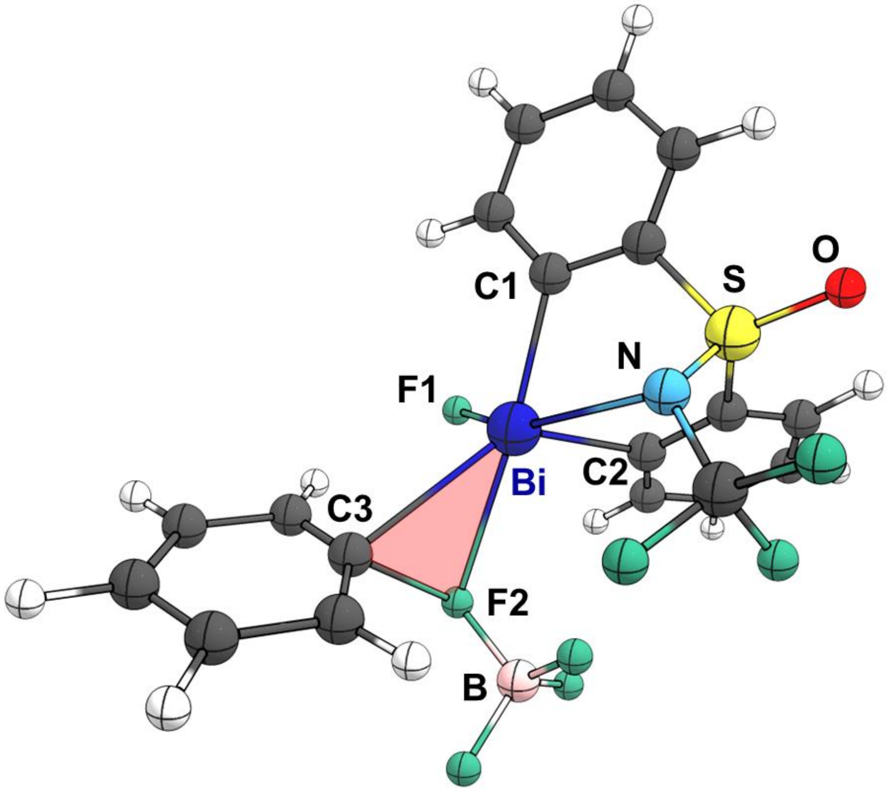

Figure S158. Optimized structure of **TSE''**.

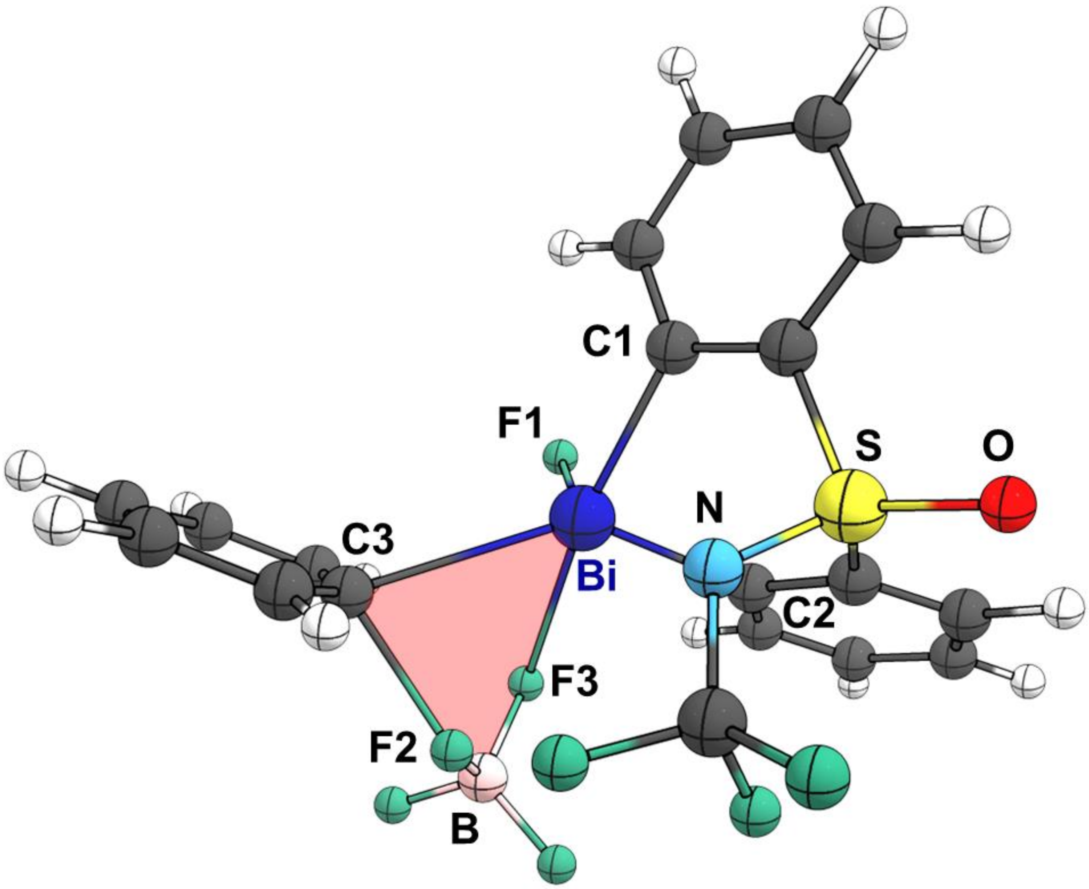

Figure S159. Optimized structure of **TSF''**.

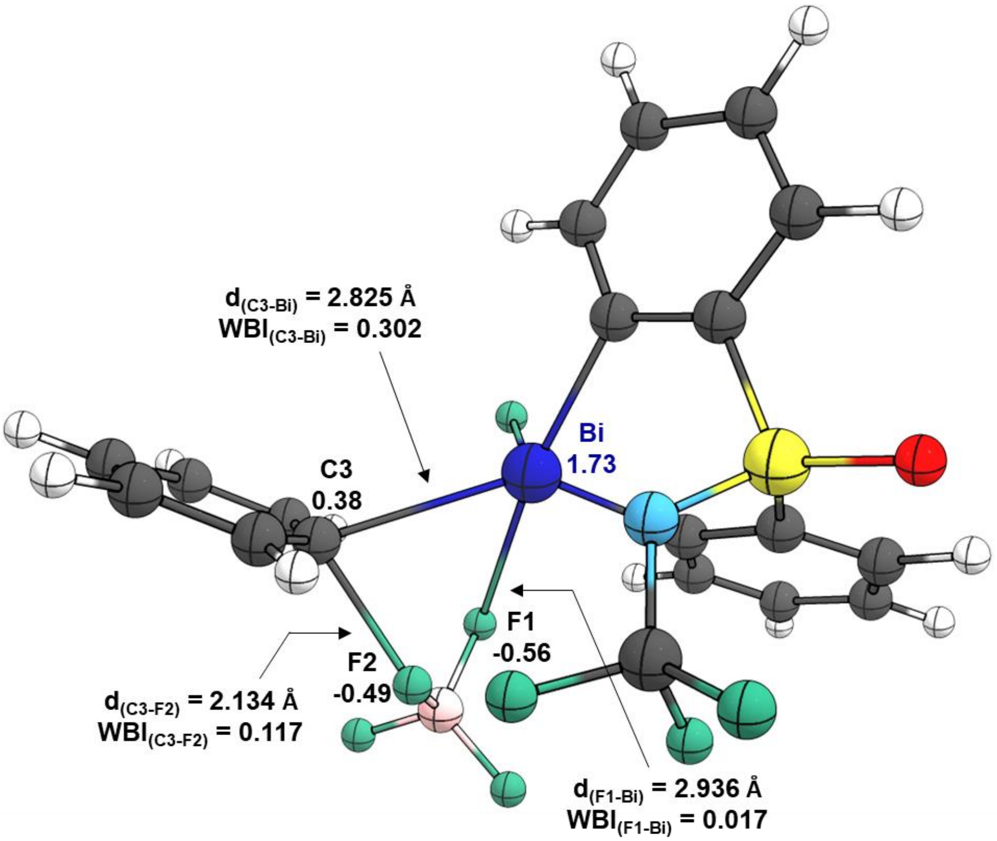

Figure S160. NBO analysis showing selected charges (q), bond orders (WBI) and distances (d) of **TSF''**.

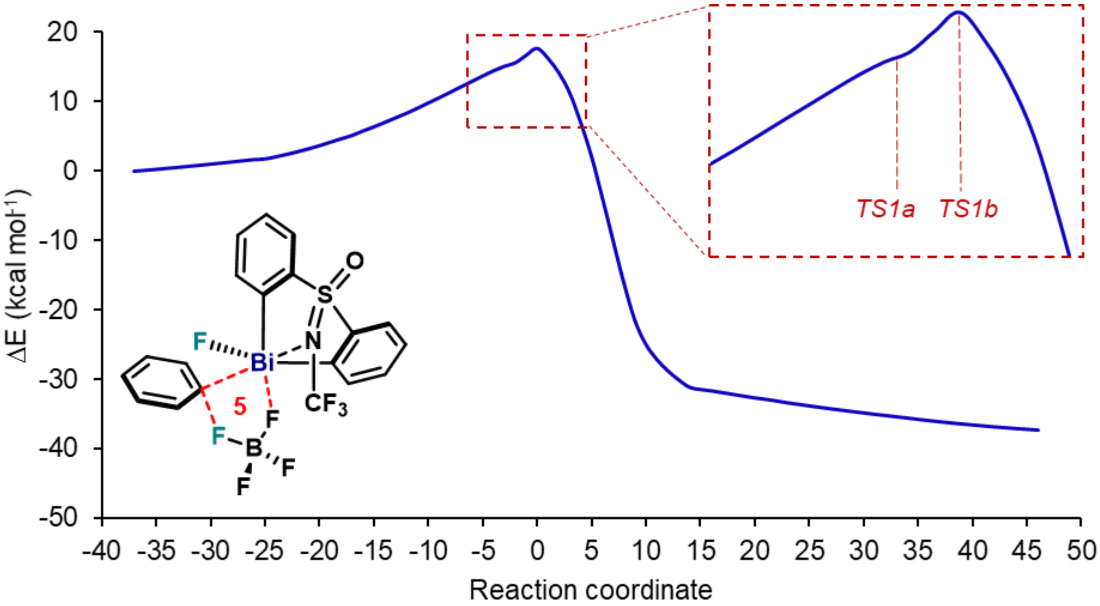

Figure S161. Intrinsic reaction coordinate (IRC) for **TSF''** showing a *concerted asynchronous* reductive elimination step.

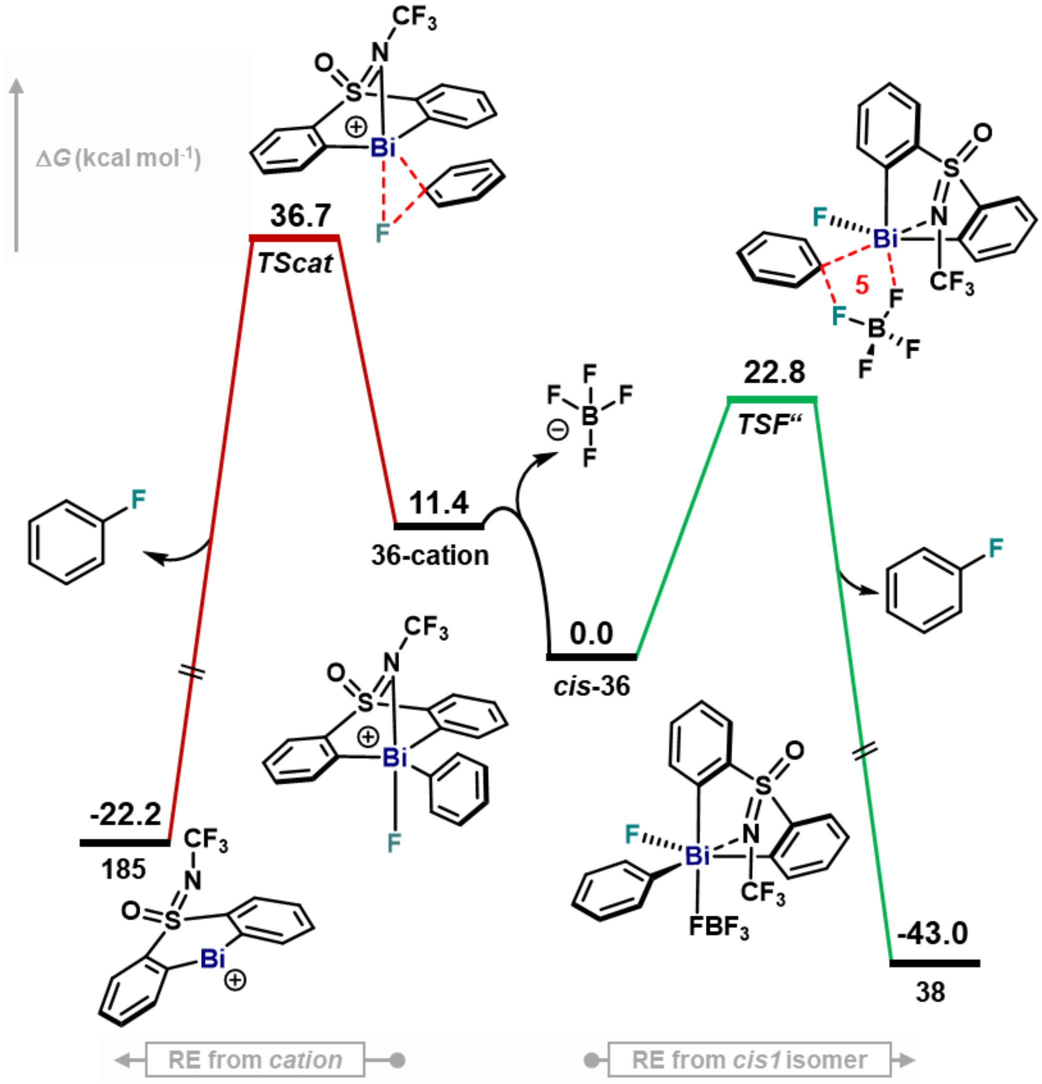

Figure S162. Gibbs energy profile of the reductive elimination of fluorobenzene from fluorobismuthonium species ***cis*-36**, including pure cationic species **36-cation**. Relative Gibbs energy values are given in kcal mol^{-1}.

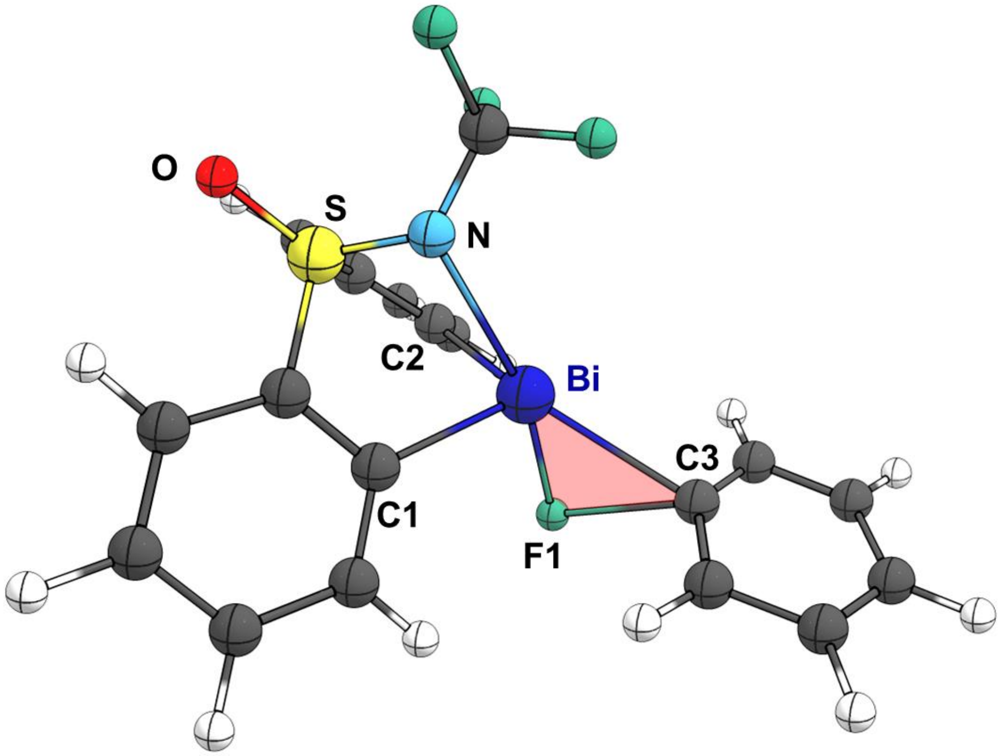

Figure S163. Optimized structure of **TScat**.

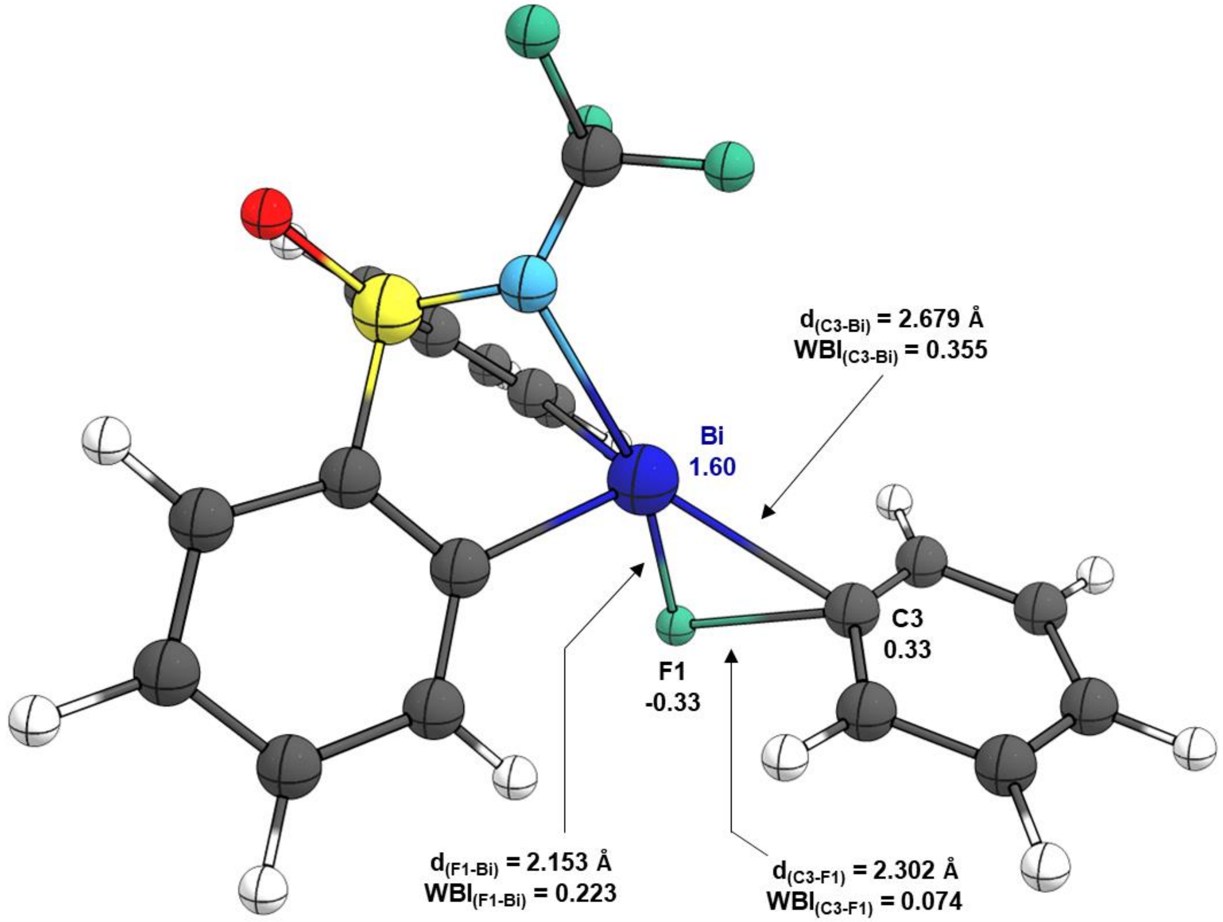

Figure S164. NBO analysis showing selected charges (q), bond orders (WBI) and distances (d) of **TScat**.

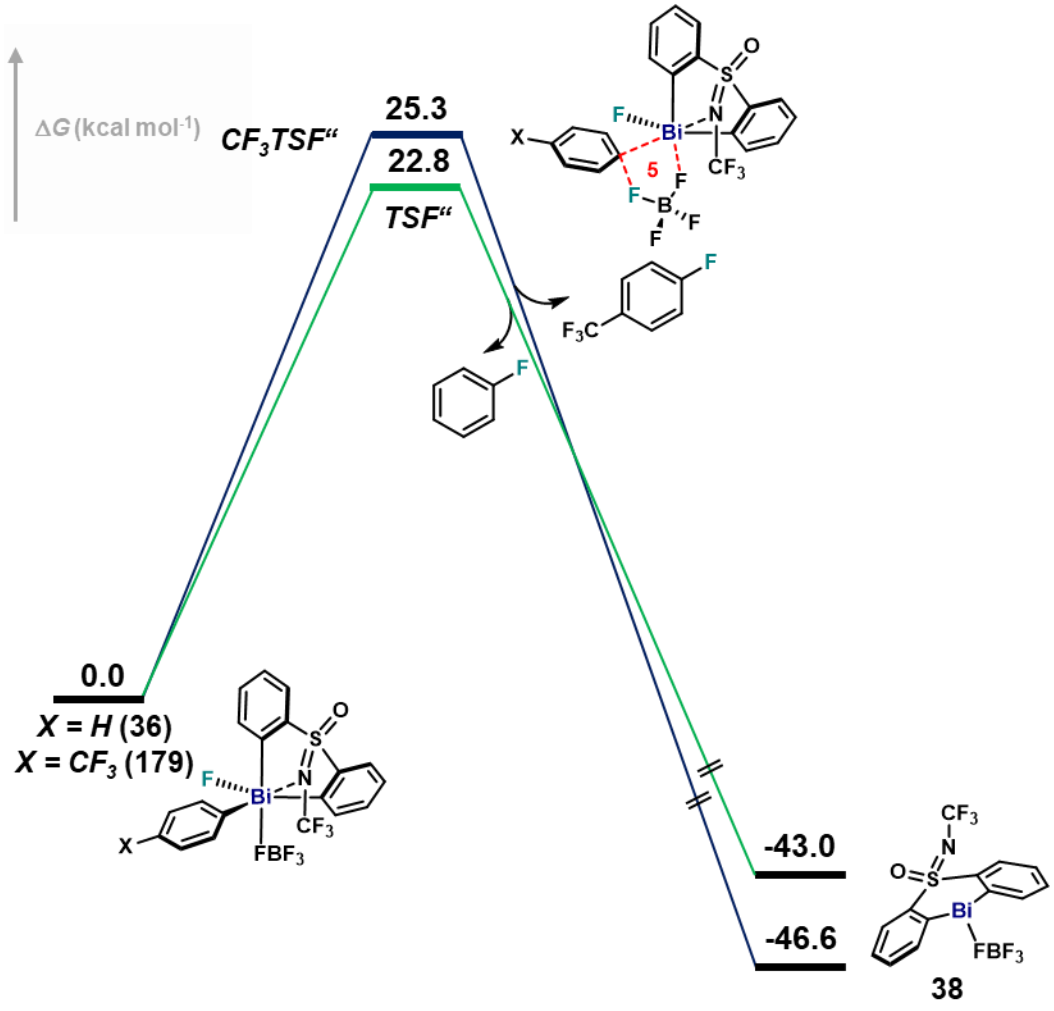

Figure S165. Gibbs energy profile of the reductive elimination of fluorobenzene from fluorobismuthonium species containing a *p*-CF_3 group in the pendant aryl ring (**179**).

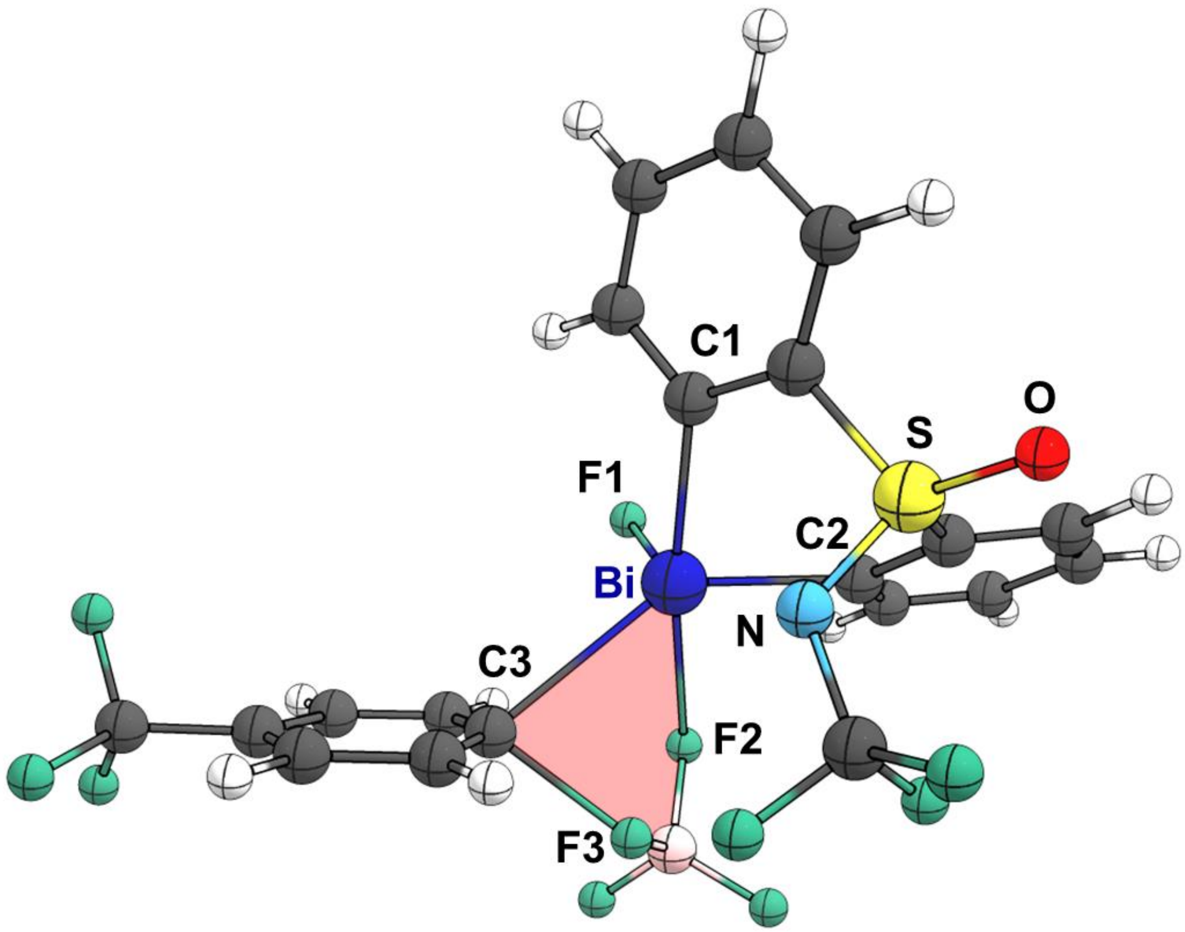

Figure S166. Optimized structure of **CF_3TSF''**.

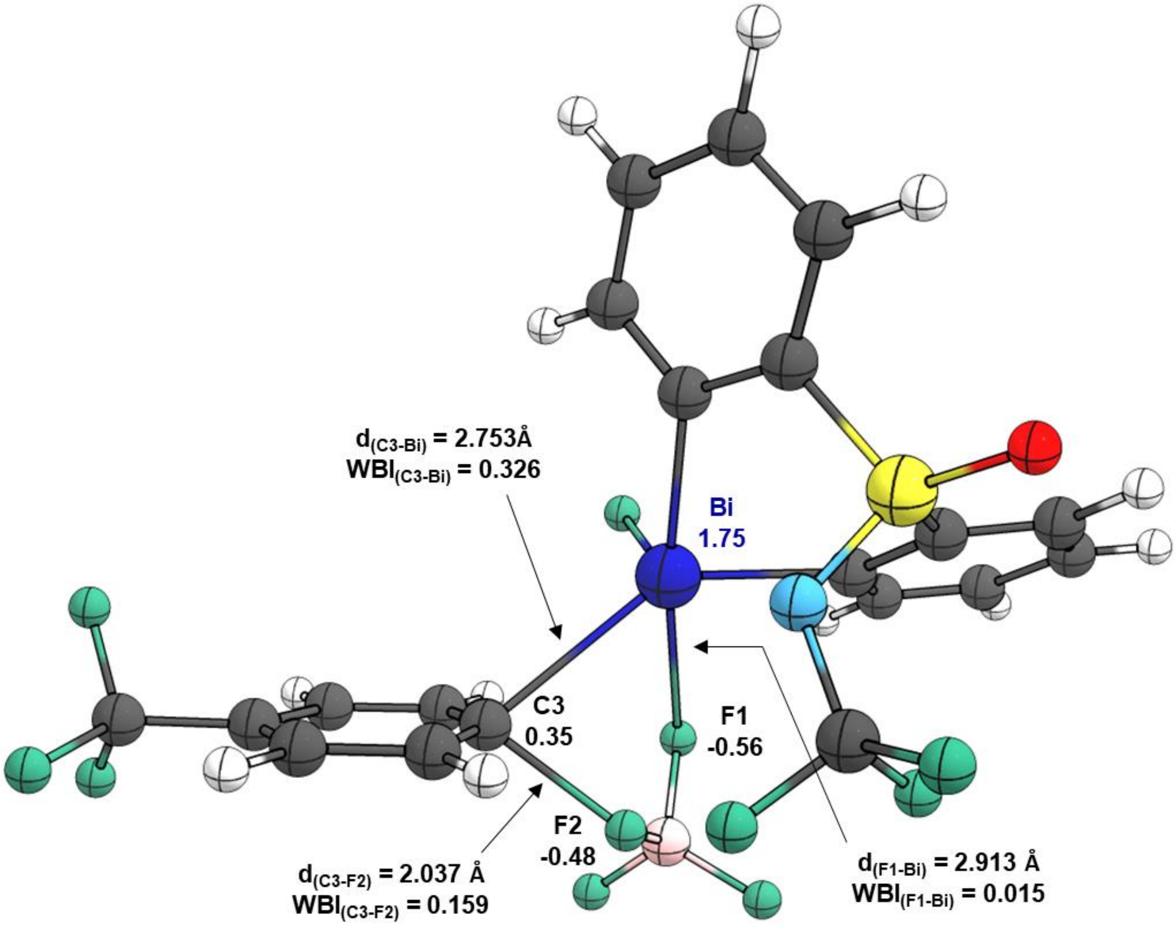

Figure S167. NBO analysis showing selected charges (q), bond orders (WBI) and distances (d) of **CF_3TSF''**.

9.6 XYZ coordinates of DFT optimized structures

Coordinates for **3**

C	9.359582	1.588451	2.851898
C	8.202601	1.233628	3.539422
C	10.512380	1.94183	3.546940
H	7.294782	0.955859	3.020966
H	11.408470	2.216161	3.005953
C	8.235517	1.243322	4.921874
C	10.511280	1.942006	4.938596
H	11.405400	2.216026	5.483599
C	9.363547	1.590038	5.642678
H	9.338355	1.582686	6.724057
H	9.355635	1.587228	1.769479
F	7.110191	0.896614	5.602782

Coordinates for **4**

Bi	0.586199	8.455820	4.360233
Bi	3.104851	10.612163	2.355633
S	-0.792399	10.571799	6.873393
S	1.161715	12.412226	-0.028710
F	2.319897	9.801709	4.626022
F	-0.906492	7.334340	3.387121
F	1.208064	11.243407	8.975400
F	2.069734	9.592835	7.862200
F	2.703219	11.624128	7.438984
F	1.472278	9.124713	2.117797
F	4.530242	11.899202	3.226965
O	-1.705059	11.238393	7.785441
O	0.630692	13.463997	-0.877370
N	0.670584	11.046055	6.733103
N	0.446014	11.047803	0.068698
C	-0.778852	10.186781	4.091734
C	-1.359469	10.804070	5.198721
C	-2.364999	11.747333	5.026250
H	-2.806226	12.220762	5.891626
C	-2.765860	12.088745	3.739701
H	-3.535673	12.836241	3.603392
C	-2.173286	11.484130	2.640861
H	-2.468947	11.760619	1.637395
C	-1.190459	10.511513	2.814850
H	-0.730994	10.045906	1.957314
C	-0.138248	7.931498	6.434340
C	-0.822556	8.828959	7.250120
C	-1.429159	8.432625	8.434551
H	-1.950968	9.155097	9.045483
C	-1.335779	7.098897	8.819242
H	-1.796046	6.776244	9.743557
C	-0.652627	6.190003	8.021944
H	-0.575672	5.153833	8.325351
C	-0.066881	6.602497	6.824974
H	0.448061	5.876627	6.207622
C	2.022737	6.797932	4.070471
C	1.715369	5.787832	3.178064
H	0.834526	5.841685	2.555289
C	2.574284	4.691770	3.119740
H	2.362718	3.887821	2.426373
C	3.689073	4.629751	3.947883
H	4.351097	3.774989	3.895406
C	3.967372	5.664194	4.834923
H	4.842933	5.620640	5.469041
C	3.128334	6.771924	4.903543
H	3.346230	7.588083	5.578291
C	1.613994	10.874185	7.722489
C	3.660033	11.287762	0.279650
C	2.870728	12.156317	-0.470975
C	3.334433	12.746158	-1.639415
H	2.696520	13.414338	-2.199813
C	4.621988	12.447777	-2.073636
H	4.994664	12.893827	-2.985940
C	5.422522	11.582365	-1.339580
H	6.423472	11.350228	-1.679819
C	4.947351	11.013701	-0.158024
H	5.592190	10.356598	0.412734
C	1.646258	12.238559	2.735268
C	1.084602	12.943978	1.672284
C	0.320149	14.078459	1.915446
H	-0.113713	14.616821	1.085138
C	0.101330	14.486190	3.226512
H	-0.509677	15.358182	3.416752
C	0.652920	13.773126	4.280597
H	0.470683	14.076265	5.303118
C	1.447772	12.655116	4.036268
H	1.868425	12.098846	4.858633
C	4.505987	8.921363	2.621136
C	4.342884	7.822778	1.795400
H	3.494296	7.734601	1.131566
C	5.303962	6.818116	1.857048
H	5.199581	5.944447	1.227636
C	6.380010	6.930543	2.730575
H	7.118868	6.140905	2.776667
C	6.507110	8.043835	3.552999
H	7.341922	8.126552	4.237248
C	5.562672	9.066859	3.500955
H	5.659565	9.947993	4.117890
C	0.449628	10.129189	-0.959764
F	1.573612	9.355519	-1.016729
F	0.320580	10.658678	-2.215213
F	-0.583151	9.270055	-0.801669

Coordinates for **TSF3**

Bi	0.801959	8.123895	4.616745
Bi	2.920825	10.560670	2.106617
S	-0.697591	10.663595	6.614742
S	1.158963	12.547925	0.029363
F	2.410691	9.618601	4.535283
F	-0.548029	6.705902	3.852280
F	1.115772	11.307920	8.836790
F	2.128373	9.651840	7.866224
F	2.740290	11.686308	7.436158
F	1.584622	8.133003	2.378391
F	4.608997	11.071375	3.200827

O	-1.625952	11.540038	7.307944
O	0.391982	13.411041	-0.847697
N	0.804717	11.010600	6.555889
N	0.804971	11.025169	0.174243
C	-0.564508	9.749252	3.952256
C	-1.121796	10.632751	4.878872
C	-2.029253	11.599734	4.467052
H	-2.451539	12.279500	5.192834
C	-2.366323	11.692215	3.120938
H	-3.055521	12.460066	2.796657
C	-1.820637	10.807927	2.204485
H	-2.075150	10.882397	1.156065
C	-0.925973	9.823298	2.621356
H	-0.497636	9.137784	1.906216
C	-0.158210	7.978522	6.680137
C	-0.887744	9.014499	7.256683
C	-1.665368	8.833149	8.393044
H	-2.215235	9.662634	8.814422
C	-1.703848	7.571256	8.976748
H	-2.298260	7.411510	9.866482
C	-0.979937	6.523731	8.420114
H	-1.007662	5.543574	8.878855
C	-0.220468	6.723745	7.267409
H	0.321719	5.890304	6.836615
C	2.375967	6.590414	4.887245
C	2.419639	5.492713	4.047373
H	1.694402	5.365884	3.256608
C	3.439902	4.564713	4.239735
H	3.504178	3.701000	3.590492
C	4.378067	4.752984	5.249721
H	5.173218	4.031368	5.386790
C	4.302709	5.863618	6.084423
H	5.032460	6.009571	6.870582
C	3.286885	6.800303	5.909272
H	3.232015	7.671978	6.548343
C	1.646760	10.914116	7.638743
C	3.751594	11.825119	0.368522
C	2.895454	12.652777	-0.352763
C	3.335245	13.538896	-1.325245
H	2.633615	14.169127	-1.853920
C	4.697632	13.579751	-1.603166
H	5.069072	14.252232	-2.364910
C	5.576652	12.760539	-0.902538
H	6.636484	12.799294	-1.120721
C	5.108984	11.892613	0.085362
H	5.813159	11.282777	0.637494
C	1.652564	12.326883	2.724157
C	1.026702	13.061401	1.722833
C	0.303484	14.210805	2.004018
H	-0.173507	14.764855	1.207462
C	0.195352	14.613713	3.332198
H	-0.378141	15.498401	3.574333
C	0.809940	13.880985	4.339174
H	0.712176	14.189640	5.371955
C	1.547745	12.734675	4.038223
H	2.000780	12.155527	4.827469
C	3.555260	8.123297	1.812312
C	3.489102	7.618673	0.544285
H	2.706552	7.872311	-0.152940
C	4.515032	6.727772	0.214037
H	4.524111	6.297718	-0.779170
C	5.488854	6.389001	1.147776
H	6.270227	5.690715	0.879485
C	5.460255	6.936161	2.425118
H	6.215421	6.681485	3.156945
C	4.452823	7.829861	2.799568
H	4.413607	8.269371	3.782582
C	0.668632	10.231705	-0.951985
F	1.712282	10.272424	-1.836344
F	-0.436667	10.517744	-1.690639
F	0.552772	8.938684	-0.570773

Coordinates for **TSF4**

Bi	0.416572	8.341914	4.177946
Bi	3.034916	10.351523	2.094028
S	-0.518659	10.474915	6.887262
S	0.969042	12.362536	-0.099471
F	2.200045	9.532494	4.484470
F	-1.185566	7.343097	3.231410
F	1.740464	10.830957	8.861954
F	2.252579	9.139486	7.605691
F	3.120317	11.085655	7.197952
F	1.248702	9.065822	1.924688
F	4.997543	11.413308	2.611068
O	-1.299366	11.175830	7.892499
O	0.462080	13.459943	-0.907587
N	0.968307	10.826830	6.679357
N	0.118906	11.090633	0.098694
C	-0.798273	10.196027	4.099489
C	-1.183431	10.845553	5.272717
C	-2.071723	11.912834	5.220461
H	-2.364832	12.408040	6.134974
C	-2.550450	12.343763	3.988264
H	-3.227322	13.186468	3.946709
C	-2.155371	11.703077	2.823633
H	-2.512442	12.044455	1.861018
C	-1.291811	10.610836	2.879351
H	-0.983840	10.118585	1.971601
C	-0.207509	7.791383	6.267925
C	-0.706324	8.720350	7.179213
C	-1.257614	8.330844	8.392994
H	-1.637720	9.074018	9.079075
C	-1.298223	6.976152	8.707517
H	-1.719398	6.660591	9.652677
C	-0.799591	6.037157	7.814064
H	-0.828212	4.983908	8.061824
C	-0.267637	6.443773	6.590723
H	0.100603	5.697774	5.896321
C	1.778615	6.686281	3.629271
C	1.394104	5.778607	2.658315
H	0.446209	5.876341	2.149467

C	2.268388	4.735330	2.361017
H	1.994844	4.012976	1.602500
C	3.481900	4.622621	3.032737
H	4.155226	3.809116	2.794498
C	3.835734	5.549699	4.007128
H	4.780110	5.464980	4.527747
C	2.977770	6.601879	4.316260
H	3.253873	7.335768	5.058882
C	1.963855	10.474678	7.558958
C	3.366191	10.973415	-0.079053
C	2.577159	11.915858	-0.734396
C	2.945661	12.464172	-1.956986
H	2.309634	13.196272	-2.433443
C	4.130482	12.046599	-2.552571
H	4.423870	12.459643	-3.508376
C	4.929006	11.104007	-1.919581
H	5.851095	10.775253	-2.381614
C	4.554166	10.581187	-0.682669
H	5.206969	9.864969	-0.202887
C	1.793667	12.160642	2.569441
C	1.149103	12.913953	1.589199
C	0.491754	14.096145	1.903669
H	0.001945	14.663815	1.125485
C	0.450610	14.515552	3.228884
H	-0.074232	15.426836	3.482603
C	1.060626	13.757566	4.217718
H	1.006442	14.066058	5.253554
C	1.742494	12.588148	3.887652
H	2.190821	11.994917	4.671000
C	5.087070	9.438128	3.289551
C	5.899693	8.655913	2.518480
H	5.903602	8.665562	1.441087
C	6.724491	7.780892	3.234738
H	7.366062	7.109690	2.678415
C	6.714599	7.771872	4.623423
H	7.362785	7.093346	5.161538
C	5.878817	8.633934	5.325496
H	5.868011	8.638221	6.407906
C	5.038094	9.523796	4.651231
H	4.382926	10.193560	5.181949
C	-0.118947	10.171250	-0.896004
F	0.963379	9.426160	-1.269363
F	-0.601645	10.699229	-2.064838
F	-1.048832	9.283474	-0.471461

Coordinates for **TSF2**

Bi	0.287163	8.585538	3.866721
Bi	2.816111	11.247376	2.516084
S	-0.426861	11.121471	6.314543
S	1.316309	12.479937	-0.527434
F	2.345370	9.240055	4.539776
F	-1.400953	7.728595	2.940422
F	1.871376	11.940698	8.075769
F	2.188936	9.898339	7.430634
F	3.247445	11.524373	6.446986
F	1.357327	9.407614	1.983741
F	4.242033	12.623902	3.281163
O	-1.102544	11.995034	7.258477
O	0.980834	13.288906	-1.688572
N	1.072759	11.335122	5.992490
N	0.527799	11.187306	-0.222067
C	-0.776395	10.533020	3.611282
C	-1.128892	11.330747	4.696924
C	-1.984664	12.412555	4.536190
H	-2.241117	13.023729	5.389660
C	-2.471916	12.708107	3.267897
H	-3.121447	13.562223	3.132920
C	-2.113398	11.921464	2.183728
H	-2.478374	12.156966	1.192840
C	-1.278651	10.819091	2.355872
H	-1.006911	10.210958	1.509451
C	-0.388107	8.350265	6.016558
C	-0.748636	9.431284	6.821474
C	-1.326774	9.248747	8.071227
H	-1.601254	10.104963	8.670257
C	-1.541892	7.954128	8.532387
H	-1.986448	7.802113	9.506859
C	-1.194531	6.867034	7.742248
H	-1.368013	5.859532	8.097698
C	-0.629257	7.065978	6.482870
H	-0.382494	6.205974	5.872046
C	1.324015	6.689034	3.390897
C	1.077398	6.075039	2.175300
H	0.379667	6.495304	1.465514
C	1.761619	4.895877	1.890802
H	1.593761	4.399259	0.943654
C	2.650997	4.359630	2.817622
H	3.176475	3.441257	2.589248
C	2.869864	4.996640	4.034088
H	3.563676	4.580918	4.753309
C	2.205344	6.183823	4.330606
H	2.397746	6.702263	5.259812
C	2.046113	11.169270	6.958558
C	3.709596	11.471588	0.454501
C	3.066748	12.110217	-0.604512
C	3.710949	12.373230	-1.807029
H	3.182744	12.872552	-2.606535
C	5.031770	11.967777	-1.964634
H	5.541286	12.157635	-2.899950
C	5.688351	11.320514	-0.926182
H	6.714904	10.999954	-1.049718
C	5.033954	11.086488	0.282067
H	5.572547	10.599315	1.085498
C	1.374855	12.974709	2.218664
C	0.999824	13.419539	0.955050
C	0.291469	14.602969	0.789532
H	0.016133	14.931750	-0.202579
C	-0.064371	15.339063	1.914515
H	-0.624626	16.256762	1.795334
C	0.293502	14.895079	3.179597
H	0.009047	15.463261	4.055840

C	1.022688	13.716943	3.333712
H	1.301956	13.383567	4.324833
C	4.078079	9.246979	3.514061
C	4.113973	8.257752	2.571554
H	3.286238	8.044483	1.914852
C	5.326135	7.565185	2.500906
H	5.430099	6.786941	1.756066
C	6.367726	7.865355	3.371748
H	7.293558	7.308935	3.314233
C	6.224656	8.870638	4.323257
H	7.031187	9.107350	5.005076
C	5.031307	9.592497	4.428030
H	4.889953	10.369788	5.161408
C	0.603000	10.063430	-1.015009
F	1.812879	9.432531	-1.023165
F	0.312974	10.279718	-2.338157
F	-0.296028	9.148131	-0.583474

Coordinates for **6**

Bi	5.233754	4.745415	3.072452
S	6.970929	2.580217	4.924984
O	7.766229	1.696485	5.754812
C	6.699065	3.171709	2.297165
C	7.335758	2.325650	3.198478
C	8.178786	1.294509	2.811795
H	8.646988	0.658373	3.550224
C	8.405608	1.114734	1.451144
H	9.067044	0.326348	1.117444
C	7.779762	1.943070	0.524876
H	7.953482	1.792574	-0.533217
C	6.926134	2.964572	0.943090
H	6.433290	3.586116	0.205811
C	4.358747	3.060473	4.334300
C	5.232250	2.245204	5.045965
C	4.821488	1.154453	5.797310
H	5.541110	0.544711	6.326335
C	3.458466	0.880733	5.853821
H	3.104538	0.043691	6.440677
C	2.558460	1.679187	5.156236
H	1.499657	1.456794	5.198944
C	3.004088	2.762614	4.397195
H	2.287571	3.361862	3.849214
F	3.931143	4.310721	1.486284
N	6.964870	4.136489	5.177232
C	8.160924	4.839946	5.221986
F	7.896852	6.163561	5.128124
F	8.850138	4.662132	6.378652
F	9.051173	4.557816	4.223767

Coordinates for ***int-trans***

Bi	0.044666	0.316486	-0.007020
S	-3.421730	0.667182	0.539046
F	0.220414	0.461214	-2.143129
F	0.038729	0.245727	2.103080
F	-2.490445	0.660294	4.118876
F	-4.242912	1.656760	3.306589
F	-2.259799	2.384874	2.820119
O	-4.833102	0.883634	0.274603
N	-2.918122	0.335655	1.956072
C	-1.539875	-1.169921	-0.420415
C	-1.201488	-2.369273	-1.024717
C	-2.205471	-3.196478	-1.526346
H	-1.938858	-4.139919	-1.984346
C	-3.535329	-2.806116	-1.456402
C	-3.880203	-1.595121	-0.864901
H	-4.912314	-1.282277	-0.796746
C	-2.879102	-0.790440	-0.338606
C	-2.545553	2.100167	-0.106468
C	-3.318816	3.209106	-0.428122
H	-4.395859	3.144093	-0.375292
C	-2.691430	4.393315	-0.799086
H	-3.291671	5.259361	-1.043475
C	-1.306515	4.462930	-0.856420
H	-0.819109	5.385439	-1.143410
C	-0.533170	3.342071	-0.558746
H	0.544509	3.397862	-0.637515
C	-1.153799	2.166955	-0.170082
H	-0.165201	-2.665977	-1.119561
H	-4.312394	-3.441124	-1.859968
C	2.236719	-0.014670	0.051917
C	2.840792	-0.197384	1.286013
H	2.261464	-0.169671	2.196538
C	4.215976	-0.415805	1.308045
H	4.713957	-0.562000	2.257904
C	4.942102	-0.446131	0.121662
H	6.010769	-0.616228	0.149333
C	4.303510	-0.258965	-1.099756
H	4.869360	-0.282480	-2.022265
C	2.928828	-0.039417	-1.148683
H	2.414397	0.108117	-2.086312
C	-2.975812	1.236851	3.001127

Coordinates for ***int-cis***

Bi	0.148938	0.181048	-0.988575
S	-2.712014	0.389732	0.802358
F	0.444529	-0.165466	-2.992814
F	1.744510	1.537389	-1.360816
F	0.237716	0.061356	3.026709
F	-1.771048	0.501529	3.729187
F	-0.723551	1.928758	2.476657
O	-3.967221	0.550867	1.508615
N	-1.412792	-0.083850	1.532335
C	-1.714513	-1.130458	-1.243246
C	-1.823137	-2.110357	-2.217003
C	-3.030327	-2.790152	-2.387095
H	-3.105096	-3.561267	-3.143261
C	-4.136506	-2.480068	-1.604569
C	-4.048546	-1.489659	-0.631891
H	-4.896374	-1.232134	-0.012465
C	-2.831122	-0.845482	-0.463210

C	-2.322730	1.907609	-0.051584
C	-3.207969	2.973140	0.011597
H	-4.140427	2.870264	0.548446
C	-2.858626	4.168972	-0.609125
H	-3.535167	5.011607	-0.561181
C	-1.647370	4.283233	-1.278890
H	-1.378256	5.217142	-1.754967
C	-0.764638	3.203293	-1.346478
H	0.179197	3.290762	-1.864801
C	-1.106997	2.017775	-0.723769
H	-0.978250	-2.340623	-2.851673
H	-5.070773	-3.006065	-1.747968
C	1.539835	-1.080418	0.186280
C	1.034982	-2.278380	0.663088
H	0.010831	-2.575198	0.479352
C	1.882171	-3.093662	1.407847
H	1.512844	-4.034707	1.794425
C	3.192374	-2.695859	1.655656
H	3.845232	-3.332760	2.238625
C	3.669216	-1.485993	1.161176
H	4.688937	-1.180056	1.356875
C	2.838349	-0.656090	0.411907
H	3.190849	0.285732	0.018475
C	-0.944689	0.590268	2.653380

Coordinates for ***int-cis’***

Bi	0.367284	0.726675	-0.725159
S	-2.571687	0.964296	0.941790
F	0.720319	0.418172	-2.724336
F	1.996255	2.073898	-0.983943
F	0.021861	-0.392131	3.216834
F	-1.472239	-1.570308	2.163553
F	-2.080736	-0.195544	3.723974
O	-3.863314	1.108772	1.582836
N	-1.268964	0.715048	1.772271
C	-1.513462	-0.564583	-1.055105
C	-1.575238	-1.543171	-2.035367
C	-2.767953	-2.234168	-2.255060
H	-2.803119	-3.008172	-3.011211
C	-3.908541	-1.933680	-1.519330
C	-3.870381	-0.938457	-0.548719
H	-4.746630	-0.683778	0.030834
C	-2.667860	-0.281533	-0.328788
C	-2.110745	2.458575	0.102316
C	-3.009422	3.514476	0.109756
H	-3.966227	3.409758	0.601767
C	-2.641019	4.705862	-0.509113
H	-3.328039	5.541209	-0.505515
C	-1.399569	4.825046	-1.119732
H	-1.117234	5.755352	-1.595135
C	-0.504967	3.752962	-1.131856
H	0.461357	3.841189	-1.606802
C	-0.868860	2.570645	-0.516053
H	-0.703853	-1.768583	-2.635269
H	-4.830683	-2.469669	-1.700042
C	1.721787	-0.615319	0.398757
C	1.295283	-1.920055	0.578716
H	0.343138	-2.265074	0.198637
C	2.125018	-2.788201	1.283155
H	1.814847	-3.813415	1.438189
C	3.340147	-2.335649	1.785658
H	3.979259	-3.014027	2.336122
C	3.741464	-1.017928	1.585519
H	4.688644	-0.670603	1.977818
C	2.928903	-0.134997	0.879538
H	3.229881	0.887112	0.704410
C	-1.214032	-0.330820	2.684040

Coordinates for **TS-A**

Bi	1.036930	9.581152	14.075867
S	3.122207	6.724577	14.333049
F	1.810123	11.628164	14.909261
O	4.197839	5.771844	14.544685
F	0.284869	5.284439	12.450765
C	1.741008	8.516443	15.938030
N	1.733146	6.269598	13.832494
F	1.741625	6.612977	11.541207
C	1.414246	9.053963	17.173970
H	0.706877	9.869286	17.251702
C	-0.031437	11.844752	14.091939
F	2.362930	4.673094	12.282808
C	3.748946	8.031017	13.277567
C	3.301672	7.002710	17.019577
H	4.036350	6.213884	16.940110
C	-1.880425	13.771035	13.658784
H	-2.624217	14.536305	13.482225
C	2.010390	8.555492	18.332481
C	2.951882	7.538642	18.255338
H	3.420956	7.159451	19.153242
C	2.961492	9.140261	12.974592
C	-0.890957	13.533746	12.711225
H	-0.854042	14.107959	11.794246
C	3.461185	10.101279	12.108222
H	2.883044	10.983835	11.867867
C	5.013383	7.866899	12.728944
H	5.603332	6.998349	12.984661
C	2.680934	7.490634	15.878869
C	0.082498	12.554628	12.923668
H	0.859591	12.376140	12.196883
C	5.496139	8.828967	11.847184
H	6.477370	8.706847	11.408405
C	1.548617	5.735487	12.570689
C	-1.912859	13.030183	14.834787
H	-2.677449	13.208749	15.580052
C	-0.957578	12.041613	15.084385
H	-0.974345	11.468897	15.998667
C	4.723316	9.939242	11.536690
H	1.741476	8.974632	19.293448
H	5.099306	10.688743	10.852291
F	-0.366563	8.271006	13.200780

Coordinates for **TS-B**

Bi	-3.469774	11.342702	12.823232
S	-5.788097	13.507873	11.533589
F	-3.563492	9.249454	12.558481
O	-6.834679	14.308080	10.927903
F	-3.291760	15.771991	13.020152
C	-5.675217	11.420345	13.334878
N	-4.709913	14.130648	12.468718
F	-2.741976	14.539108	11.322118
C	-6.242318	10.497548	14.195054
H	-5.624941	9.789857	14.732601
C	-1.101547	11.471069	13.357580
F	-4.343256	15.987616	11.131018
C	-4.949435	12.582233	10.258560
C	-7.871799	12.276966	12.772029
H	-8.485155	12.969393	12.212418
C	1.508041	10.747037	13.316127
H	2.548718	10.452337	13.297206
C	-7.628936	10.475168	14.356352
C	-8.437075	11.356072	13.649015
H	-9.511015	11.330846	13.775572
C	-3.901570	11.745112	10.631078
C	1.001162	11.565355	12.312152
H	1.638636	11.916027	11.510642
C	-3.221122	11.048832	9.643795
H	-2.415023	10.373242	9.900738
C	-5.333115	12.744713	8.935447
H	-6.154529	13.400344	8.682703
C	-6.491285	12.302913	12.637000
C	-0.336459	11.964816	12.328028
H	-0.734984	12.623553	11.570207
C	-4.629322	12.054795	7.952847
H	-4.904432	12.175997	6.913675
C	-3.807986	15.074347	11.993115
C	0.684215	10.314546	14.350246
H	1.073203	9.679542	15.136042
C	-0.660612	10.687412	14.396343
H	-1.307506	10.355810	15.194316
C	-3.580992	11.213556	8.305224
H	-8.073762	9.758536	15.034671
H	-3.038291	10.677030	7.537522
F	-2.289505	13.012092	13.993692

Coordinates for **TS-C**

Bi	3.730976	3.855390	5.129392
S	6.315553	1.843643	5.756026
C	5.419913	3.272636	3.628610
C	6.412863	2.409676	4.080595
C	7.444734	1.936921	3.283312
H	8.187796	1.259561	3.681699
C	7.493149	2.371236	1.961990
H	8.289715	2.028694	1.315038
C	6.520256	3.239483	1.478905
H	6.560668	3.569522	0.448239
C	5.487059	3.686562	2.305732
H	4.727468	4.352031	1.915754
C	3.602122	1.612583	5.623751
C	4.792366	0.918966	5.820731
C	4.823537	-0.447436	6.057706
H	5.765889	-0.955283	6.210342
C	3.613788	-1.133253	6.109552
H	3.611052	-2.197493	6.303994
C	2.415723	-0.453392	5.921151
H	1.478007	-0.992681	5.967253
C	2.403821	0.921920	5.677138
H	1.471018	1.451305	5.540423
C	3.602551	6.357078	4.789222
C	2.519928	6.944371	5.373517
H	1.623813	6.399408	5.629336
C	2.686591	8.307598	5.641986
H	1.879335	8.838614	6.129969
C	3.859534	8.965874	5.284505
H	3.965146	10.022478	5.490514
C	4.892185	8.280740	4.653789
H	5.803065	8.789578	4.365801
C	4.770027	6.916556	4.362473
H	5.549241	6.365775	3.856558
F	1.600973	3.849913	5.517148
F	2.963747	5.005483	3.359945
O	7.434409	1.001497	6.130362
N	6.054654	3.212857	6.497082
C	5.974851	3.249457	7.884971
F	5.389844	4.410534	8.259640
F	5.246112	2.249505	8.462203
F	7.187215	3.209991	8.493427

Coordinates for **TSC**

Bi	3.862795	3.870720	5.218121
S	6.412253	1.726066	5.629965
C	5.463319	3.246769	3.603648
C	6.445099	2.323352	3.952851
C	7.402419	1.843506	3.071026
H	8.138192	1.120116	3.394661
C	7.388302	2.331058	1.768353
H	8.129013	1.987161	1.058589
C	6.421884	3.254508	1.383657
H	6.411821	3.627341	0.366846
C	5.461790	3.705405	2.292019
H	4.704514	4.409309	1.972378
C	3.672055	1.637127	5.652944
C	4.847327	0.902928	5.772152
C	4.846466	-0.472040	5.957569
H	5.778320	-1.013616	6.044357
C	3.619569	-1.123908	6.040147
H	3.593776	-2.194428	6.193649
C	2.436346	-0.403036	5.931989
H	1.485175	-0.916065	5.997632
C	2.456826	0.980506	5.741149
H	1.536953	1.543397	5.666835

C	3.649582	6.366899	4.776003
C	2.572845	6.938616	5.380662
H	1.718242	6.371474	5.717518
C	2.693348	8.323354	5.550010
H	1.889764	8.852084	6.046486
C	3.814185	9.003116	5.082740
H	3.882189	10.074995	5.211012
C	4.841351	8.320964	4.440130
H	5.711724	8.845892	4.068017
C	4.766270	6.935541	4.245129
H	5.540682	6.384574	3.732640
F	1.756782	3.905785	5.734655
F	2.958874	4.888350	3.462196
O	7.516207	0.819513	5.878800
N	6.157995	2.961421	6.570024
C	7.126447	3.945560	6.724577
F	6.568769	5.181670	6.586579
F	7.680124	3.927465	7.961035
F	8.167450	3.901766	5.848128

Coordinates for **TS-D**

Bi	2.006756	10.135441	14.027740
S	2.930707	6.970102	14.646896
F	1.640339	11.270660	11.993656
O	3.332621	5.595199	14.867328
F	-0.296790	7.406881	12.884511
C	2.762727	9.305188	16.048996
N	1.445641	7.333167	14.277027
F	1.557084	6.629068	12.056649
C	2.953062	10.085779	17.177622
H	2.683892	11.134149	17.170166
C	-0.005078	11.375765	13.283993
F	0.424001	5.441957	13.470742
C	3.974785	7.720187	13.408677
C	3.719245	7.389083	17.217659
H	4.014178	6.348774	17.210005
C	-2.416679	12.572129	13.365234
H	-3.386252	13.050534	13.401275
C	3.511547	9.520590	18.325553
C	3.892440	8.183299	18.346510
H	4.327484	7.754756	19.239393
C	3.649717	9.001564	12.976266
C	-2.293350	11.298273	12.821485
H	-3.158652	10.775768	12.433922
C	4.401617	9.594256	11.979239
H	4.143712	10.582573	11.618450
C	5.048310	7.017131	12.882789
H	5.274581	6.019720	13.233167
C	3.143043	7.969555	16.096185
C	-1.048664	10.664121	12.758034
H	-0.938509	9.681772	12.327265
C	5.805035	7.623283	11.884008
H	6.642179	7.091064	11.452550
C	0.821845	6.711492	13.204029
C	-1.297549	13.238168	13.853271
H	-1.383403	14.234064	14.269029
C	-0.034034	12.641975	13.804934
H	0.851201	13.148877	14.159754
C	5.481968	8.898654	11.433248
H	3.653985	10.133975	19.206338
H	6.070556	9.358390	10.649746
F	3.099700	11.839892	14.456813

Coordinates for **TSD**

Bi	2.219737	10.451476	14.396229
S	2.748087	7.161508	14.762078
F	1.991738	11.775881	12.463621
O	2.964030	5.735867	14.909597
F	-0.813052	8.188416	14.750398
C	3.063508	9.393044	16.285390
N	1.336911	7.730480	14.359968
F	-0.196809	6.110817	14.913171
C	3.478074	10.087478	17.409197
H	3.348805	11.160102	17.468835
C	0.169562	11.397425	13.389451
F	0.348888	7.524471	16.462105
C	3.738310	7.847680	13.458244
C	3.886192	7.316895	17.260315
H	4.039725	6.249615	17.179351
C	-2.430828	11.974216	12.984380
H	-3.476032	12.203345	12.825734
C	4.080867	9.397415	18.462897
C	4.283748	8.023310	18.390347
H	4.754278	7.498530	19.211095
C	3.598392	9.193937	13.136932
C	-1.879759	10.829998	12.418673
H	-2.484762	10.161774	11.819300
C	4.347371	9.715596	12.097478
H	4.227120	10.754288	11.815463
C	4.623402	7.012218	12.793789
H	4.706948	5.969568	13.067585
C	3.272671	8.019734	16.232387
C	-0.529491	10.520934	12.603900
H	-0.087299	9.640966	12.161871
C	5.380903	7.552127	11.758173
H	6.073081	6.919391	11.218939
C	0.215878	7.390152	15.108437
C	-1.642472	12.830092	13.746778
H	-2.063361	13.725414	14.186236
C	-0.288584	12.556215	13.957725
H	0.340100	13.217237	14.535098
C	5.240793	8.891084	11.410875
H	4.397827	9.941968	19.343401
H	5.828245	9.300224	10.598942
F	3.412404	12.060826	14.931396

Coordinates for ***trans*-26**

Bi	0.735774	0.476009	-0.326862
S	-2.653357	0.110686	0.506901
F	0.736566	0.672182	-2.473610

F	0.921309	0.385847	1.776913
F	-1.455282	0.248384	4.004140
F	-3.411099	0.924020	3.338143
F	-1.629239	1.998849	2.731965
O	-4.098091	0.036254	0.373472
N	-1.971776	-0.125849	1.868669
C	-0.533334	-1.309135	-0.653111
C	-0.010368	-2.417776	-1.311132
C	-0.903381	-3.408139	-1.733987
H	-0.502795	-4.282377	-2.233273
C	-2.278172	-3.304143	-1.555951
C	-2.781539	-2.174557	-0.907342
H	-3.843580	-2.062230	-0.738782
C	-1.908972	-1.200333	-0.453429
C	-2.149339	1.701068	-0.169344
C	-3.165858	2.621491	-0.384315
H	-4.194200	2.323775	-0.238018
C	-2.851808	3.923411	-0.769612
C	-1.511629	4.260823	-0.940503
H	-1.257837	5.270674	-1.241145
C	-0.475557	3.343558	-0.753481
C	-0.814600	2.055061	-0.348247
C	2.949772	0.631547	-0.415137
C	3.663778	0.581350	0.771532
H	3.156218	0.474818	1.718298
C	5.051950	0.674836	0.700776
H	5.632588	0.638102	1.613623
C	5.685112	0.813981	-0.529660
H	6.764316	0.885543	-0.574953
C	4.938521	0.861619	-1.702763
H	5.431401	0.970328	-2.660437
C	3.549887	0.769499	-1.656644
H	2.954251	0.804650	-2.556694
C	-2.114603	0.739607	2.934355
C	1.459886	-2.553967	-1.594484
H	2.056541	-2.378403	-0.697185
H	1.693221	-3.551437	-1.962312
H	1.769465	-1.832475	-2.354140
C	-3.211706	-4.382753	-2.027001
H	-2.694061	-5.102192	-2.660318
H	-3.634749	-4.920986	-1.175112
H	-4.044391	-3.957422	-2.589853
C	0.951344	3.745664	-1.004792
H	1.586504	3.512071	-0.147449
H	1.348996	3.218832	-1.875276
H	1.026359	4.814876	-1.193883
C	-3.935298	4.944941	-0.970233
H	-4.893286	4.467654	-1.174659
H	-4.051303	5.556239	-0.071150
H	-3.693570	5.615617	-1.795481

Coordinates for ***cis*-26**

Bi	0.940013	0.428117	-1.214621
S	-1.690463	-0.072190	0.691096
F	1.273807	0.641062	-3.231647

F	2.289260	2.080052	-0.978642
F	1.534423	0.345744	2.468660
F	-0.334738	-0.047919	3.504425
F	-0.093524	1.780635	2.365993
O	-2.824214	-0.244490	1.575844
N	-0.219605	-0.244716	1.220170
C	-0.627503	-1.253129	-1.534416
C	-0.622059	-2.217558	-2.540796
C	-1.704093	-3.105544	-2.614279
H	-1.697375	-3.858888	-3.393842
C	-2.780851	-3.069263	-1.732592
C	-2.771279	-2.112562	-0.716436
H	-3.574148	-2.056865	0.006516
C	-1.694839	-1.245418	-0.640299
C	-1.780782	1.516275	-0.108623
C	-2.922212	2.278657	0.067097
H	-3.721054	1.916754	0.699736
C	-3.012450	3.514269	-0.573506
C	-1.957105	3.913289	-1.387075
H	-2.027754	4.864215	-1.901978
C	-0.798753	3.147581	-1.587652
C	-0.718264	1.934733	-0.908345
C	2.623985	-0.834402	-0.482334
C	2.308304	-2.138707	-0.144808
H	1.293004	-2.508082	-0.207257
C	3.334828	-2.970830	0.294299
H	3.111983	-3.993825	0.569030
C	4.635712	-2.485049	0.381177
H	5.430420	-3.135610	0.723314
C	4.921227	-1.168281	0.034258
H	5.934028	-0.792574	0.105138
C	3.906544	-0.319424	-0.402751
H	4.105748	0.709079	-0.662473
C	0.194918	0.445776	2.356436
C	0.510917	-2.341694	-3.523212
H	1.465996	-2.453767	-3.005164
H	0.370603	-3.209060	-4.166664
H	0.588330	-1.449317	-4.143814
C	-3.926084	-4.035363	-1.854496
H	-3.745185	-4.760615	-2.647014
H	-4.079626	-4.577011	-0.918878
H	-4.854882	-3.506255	-2.080426
C	0.269411	3.664636	-2.510094
H	1.196140	3.832352	-1.963158
H	0.492235	2.940150	-3.293918
H	-0.053669	4.595285	-2.973373
C	-4.215993	4.390819	-0.371951
H	-5.133741	3.801176	-0.368078
H	-4.153589	4.906575	0.589945
H	-4.287745	5.146242	-1.153777

Coordinates for **TSA’**

Bi	1.161291	9.809859	14.143349
S	2.994902	6.798082	14.298033
F	1.722909	12.024874	14.838473

O	3.955367	5.718520	14.450336
F	0.009488	5.710421	12.409753
C	1.812116	8.635001	16.005689
N	1.556373	6.520054	13.800292
F	1.597081	6.888473	11.510745
C	1.544246	9.122234	17.284163
C	-0.149896	11.969831	14.052761
F	2.011732	4.886692	12.228225
C	3.752664	8.068030	13.291922
C	3.281762	6.941900	16.963048
H	3.939134	6.097237	16.808723
C	-2.295620	13.537483	13.492720
H	-3.153756	14.156089	13.266931
C	2.156498	8.500078	18.379608
C	3.026998	7.425538	18.246896
C	3.081361	9.259020	13.015724
C	-1.289020	13.372578	12.549136
H	-1.350707	13.856946	11.583004
C	3.675654	10.173121	12.147543
C	4.988882	7.767676	12.745097
H	5.466741	6.828317	12.986112
C	2.667756	7.542938	15.877872
C	-0.168987	12.585567	12.827314
H	0.614203	12.474746	12.097147
C	5.595113	8.675555	11.876475
C	1.313826	6.025858	12.531995
C	-2.191787	12.916895	14.731620
H	-2.963002	13.042669	15.480852
C	-1.085866	12.122783	15.043558
H	-1.004460	11.656624	16.010263
C	4.921806	9.856893	11.591959
H	1.942400	8.880939	19.371308
H	5.375376	10.567895	10.911499
F	-0.317076	8.679960	13.158034
C	3.014949	11.469435	11.769902
H	3.653039	12.053494	11.109075
H	2.080419	11.274258	11.238344
H	2.795881	12.072416	12.652035
C	0.614774	10.278623	17.525188
H	-0.384027	10.043153	17.151065
H	0.528186	10.492460	18.589027
H	0.971756	11.180227	17.025189
C	6.934125	8.366536	11.269049
H	7.691870	8.245564	12.046546
H	6.895676	7.432125	10.704941
H	7.255121	9.161598	10.597144
C	3.670284	6.781947	19.442422
H	3.327418	5.750739	19.555674
H	4.755935	6.752582	19.329388
H	3.432128	7.323735	20.356992

Coordinates for **TSB’**

Bi	-3.235727	11.413898	12.267982
S	-5.848140	13.487910	11.491745
F	-3.365110	9.339024	11.823623

O	-7.027210	14.228563	11.083836
F	-3.236962	15.816808	12.687078
C	-5.306616	11.386103	13.187055
N	-4.688394	14.168600	12.276931
F	-3.123888	14.893252	10.725835
C	-5.659033	10.441622	14.146414
C	-1.016835	11.345216	13.299749
F	-4.771998	16.216556	11.203532
C	-5.152818	12.683341	10.054776
C	-7.622630	12.099026	12.989403
H	-8.354175	12.757292	12.540577
C	1.249889	10.230999	14.305742
H	2.149127	9.780797	14.704310
C	-7.008905	10.350159	14.507616
C	-7.998027	11.149884	13.942661
C	-3.985986	11.938233	10.189078
C	1.180764	10.569041	12.958628
H	2.020445	10.386360	12.300164
C	-3.372923	11.412563	9.056381
C	-5.751206	12.904015	8.825835
H	-6.654893	13.494845	8.763802
C	-6.287550	12.204674	12.641090
C	0.031009	11.156765	12.428726
H	-0.019614	11.434321	11.386961
C	-5.159277	12.373789	7.678101
C	-3.983716	15.233018	11.731719
C	0.168316	10.482042	15.143584
H	0.216881	10.233606	16.196156
C	-0.996232	11.069263	14.647470
H	-1.829446	11.288439	15.298123
C	-3.979231	11.648829	7.816439
H	-7.292194	9.620861	15.257586
H	-3.503855	11.250002	6.927836
F	-1.840748	13.159243	13.025014
C	-2.082286	10.642003	9.129559
H	-2.156451	9.816001	9.838425
H	-1.816007	10.234827	8.155610
H	-1.266681	11.297011	9.446775
C	-5.791435	12.585583	6.331243
H	-5.114397	12.295348	5.528547
H	-6.703020	11.989583	6.238456
H	-6.070063	13.631365	6.191199
C	-4.639128	9.544035	14.793502
H	-5.107775	8.880480	15.518212
H	-4.127794	8.934994	14.046411
H	-3.886821	10.135006	15.321145
C	-9.439617	11.010455	14.343781
H	-10.043812	10.678220	13.496229
H	-9.555477	10.287523	15.150372
H	-9.844664	11.968348	14.676172

Coordinates for **TSC’**

Bi	3.435478	3.923441	4.772595
S	6.078665	2.268235	5.664848
C	5.126190	3.232722	3.290787

C	6.192411	2.604078	3.926073
C	7.319233	2.129701	3.272916
H	8.108895	1.638620	3.825931
C	7.406229	2.304208	1.893058
C	6.333794	2.906533	1.238766
H	6.378836	3.014196	0.160673
C	5.187822	3.364734	1.901287
C	3.418495	1.670481	5.453262
C	4.674942	1.179309	5.803930
C	4.906054	-0.129741	6.194238
H	5.905539	-0.452854	6.452710
C	3.825909	-1.007786	6.253152
C	2.572904	-0.530744	5.878038
H	1.731857	-1.214674	5.895183
C	2.336731	0.789422	5.467989
C	3.716054	6.352810	4.984731
C	2.765549	7.009790	5.723476
H	1.765174	6.622922	5.847565
C	3.191044	8.189883	6.336065
H	2.490658	8.735254	6.955814
C	4.488507	8.659832	6.151126
H	4.800002	9.580605	6.625962
C	5.382541	7.958701	5.348482
H	6.389466	8.324260	5.192577
C	4.992981	6.775505	4.717089
H	5.669799	6.223751	4.080602
F	1.319407	4.083646	5.101126
F	2.894638	5.642180	3.286709
O	7.273028	1.643792	6.195672
N	5.623277	3.684986	6.203529
C	5.511018	3.894889	7.577752
F	4.788748	5.013840	7.794622
F	4.894785	2.895919	8.273328
F	6.709539	4.075661	8.183778
C	4.051537	3.935012	1.098326
H	4.291146	3.932003	0.035709
H	3.145662	3.341091	1.243469
H	3.815078	4.950303	1.413457
C	0.946787	1.176515	5.042501
H	0.941474	1.530819	4.009938
H	0.277561	0.320162	5.114030
H	0.560123	1.991188	5.651558
C	4.012515	-2.423619	6.721909
H	3.223331	-3.071503	6.340608
H	4.976705	-2.820151	6.401697
H	3.983771	-2.470336	7.814017
C	8.627850	1.858783	1.138940
H	9.067619	0.969299	1.591327
H	8.388914	1.640362	0.098011
H	9.389264	2.643617	1.147450

Coordinates for **TSD’**

Bi	3.283831	3.678434	5.158276
S	6.122993	2.200326	5.943094
C	5.131615	3.535392	3.783225

C	6.205995	2.811198	4.279302
C	7.349063	2.534898	3.545709
H	8.155799	1.958367	3.978239
C	7.43669	3.035782	2.246835
C	6.373193	3.790277	1.751966
H	6.443921	4.189141	0.746169
C	5.217283	4.057681	2.494205
C	3.489604	1.495322	5.748833
C	4.781501	1.024482	5.963619
C	5.059408	-0.30451	6.229433
H	6.079386	-0.62592	6.39193
C	3.99755	-1.2078	6.307215
C	2.704734	-0.7293	6.113177
H	1.875926	-1.42398	6.187047
C	2.416537	0.612825	5.829647
C	2.22804	5.81379	5.712509
C	2.901181	6.663184	6.555802
H	3.618504	6.317127	7.283283
C	2.61383	8.021908	6.409312
H	3.131134	8.731775	7.042272
C	1.680788	8.45563	5.472958
H	1.465153	9.511253	5.375349
C	1.019428	7.534979	4.667283
H	0.287398	7.863022	3.940172
C	1.276761	6.167321	4.786811
H	0.76844	5.438039	4.173618
F	1.52984	4.189598	6.760567
F	1.959881	3.271395	3.603856
O	7.356381	1.557851	6.350022
N	5.603182	3.491368	6.689511
C	5.474441	3.478015	8.075319
F	4.768268	4.565151	8.46146
F	4.826142	2.397732	8.598741
F	6.664139	3.536263	8.725817
C	4.111423	4.89287	1.906464
H	4.392049	5.28002	0.928023
H	3.195888	4.309086	1.804935
H	3.884089	5.743366	2.554287
C	0.992645	1.053405	5.632058
H	0.814766	1.31483	4.587907
H	0.303442	0.259614	5.916075
H	0.773778	1.941537	6.227192
C	4.254342	-2.65445	6.624022
H	3.358632	-3.25539	6.47111
H	5.05349	-3.05487	5.99774
H	4.566685	-2.76936	7.665044
C	8.669119	2.798258	1.419101
H	9.433288	3.546258	1.647655
H	9.097979	1.817038	1.625052
H	8.445954	2.865893	0.354365

Coordinates for ***cis*-26’**

Bi	0.895039	0.383065	-1.175536
S	-1.802440	-0.098941	0.660832
F	1.270679	0.623979	-3.179959

F	2.251614	2.016704	-0.855622
F	1.318411	-0.918987	2.491712
F	-0.114476	-2.346873	1.689724
F	-0.673482	-1.064534	3.342906
O	-2.975557	-0.285619	1.490245
N	-0.364927	-0.065069	1.297712
C	-0.706557	-1.277431	-1.543357
C	-0.692594	-2.209612	-2.581201
C	-1.768756	-3.102006	-2.686611
H	-1.748771	-3.836462	-3.483754
C	-2.855311	-3.092949	-1.816861
C	-2.867583	-2.151555	-0.787176
H	-3.686300	-2.105342	-0.081817
C	-1.796305	-1.282236	-0.673716
C	-1.823135	1.480749	-0.142794
C	-2.970218	2.240335	0.005846
H	-3.787235	1.874458	0.612585
C	-3.042037	3.481002	-0.628330
C	-1.962549	3.888551	-1.404337
H	-2.018025	4.844046	-1.912439
C	-0.798010	3.124828	-1.575580
C	-0.740211	1.903830	-0.909811
C	2.560141	-0.919484	-0.467418
C	2.288063	-2.273110	-0.378725
H	1.310562	-2.671338	-0.614480
C	3.305475	-3.122916	0.047373
H	3.113705	-4.185057	0.128321
C	4.555235	-2.605116	0.369500
H	5.342944	-3.269250	0.701561
C	4.798754	-1.238602	0.270031
H	5.771949	-0.837820	0.523500
C	3.793343	-0.372752	-0.152887
H	3.960948	0.690254	-0.231920
C	0.020021	-1.074544	2.173926
C	0.432102	-2.293846	-3.577963
H	1.402397	-2.349797	-3.081638
H	0.322012	-3.175643	-4.207608
H	0.452910	-1.408489	-4.213277
C	-3.989404	-4.067672	-1.969710
H	-3.806337	-4.757899	-2.792491
H	-4.127116	-4.649532	-1.055883
H	-4.927014	-3.542230	-2.165222
C	0.299468	3.646885	-2.459717
H	1.223375	3.759009	-1.894375
H	0.507966	2.948532	-3.271128
H	0.013339	4.606325	-2.887099
C	-4.254232	4.352016	-0.456986
H	-5.170856	3.764233	-0.524627
H	-4.243571	4.829850	0.526135
H	-4.284663	5.136478	-1.212319

C	6.228536	2.538133	3.880230
C	7.289213	2.059868	3.127309
H	8.111124	1.543235	3.604467
C	7.266846	2.260796	1.749385
C	6.152717	2.891239	1.201669
H	6.108790	3.020190	0.125792
C	5.073367	3.355977	1.965907
C	3.538763	1.664059	5.604892
C	4.810080	1.153121	5.855549
C	5.055127	-0.170232	6.184217
H	6.066572	-0.511494	6.359358
C	3.972617	-1.042527	6.283043
C	2.703825	-0.545145	6.003377
H	1.860656	-1.225474	6.042857
C	2.452043	0.791070	5.657318
C	3.709111	6.388993	4.900846
C	2.860672	7.060918	5.738723
H	1.954313	6.618209	6.123864
C	3.271917	8.350643	6.084455
H	2.658517	8.923850	6.767930
C	4.442738	8.889467	5.558228
H	4.741153	9.893223	5.829538
C	5.223513	8.151653	4.674993
H	6.128336	8.569839	4.253003
C	4.845099	6.859680	4.300961
H	5.426535	6.275733	3.602691
F	1.492217	4.022101	5.581055
F	2.668401	5.362461	3.487001
O	7.462585	1.510712	6.015059
N	5.778416	3.484580	6.400092
C	6.674975	4.536391	6.570728
F	6.026137	5.576345	7.131979
F	7.714189	4.231839	7.386598
F	7.242928	5.008009	5.420575
C	3.903212	3.973900	1.249171
H	4.001946	3.833459	0.173115
H	2.962970	3.525697	1.571404
H	3.825502	5.039777	1.459888
C	1.039359	1.194057	5.333593
H	0.985360	1.698045	4.368265
H	0.397242	0.314662	5.302505
H	0.653514	1.896406	6.070390
C	4.178395	-2.475717	6.686350
H	4.245202	-2.559643	7.774522
H	3.349284	-3.100424	6.354494
H	5.104866	-2.872670	6.269496
C	8.414233	1.811851	0.888047
H	8.849433	0.885026	1.263958
H	8.095220	1.655402	-0.142477
H	9.205371	2.566762	0.880944

Coordinates for **TS-C’**

Bi	3.562691	3.916859	5.015312
S	6.238131	2.181222	5.627653
C	5.119205	3.201533	3.356087

Coordinates for **TS-D’**

Bi	3.241177	3.670425	5.022993
S	6.117656	2.274897	5.840562
C	5.093490	3.483705	3.632048

C	6.186489	2.789660	4.135910
C	7.328355	2.521773	3.396198
H	8.149972	1.973104	3.836495
C	7.394903	2.991741	2.085459
C	6.311731	3.713086	1.583678
H	6.366842	4.093259	0.569672
C	5.157243	3.975859	2.329993
C	3.474518	1.529890	5.698398
C	4.776929	1.115481	5.958092
C	5.095785	-0.187624	6.298172
H	6.123813	-0.466834	6.485246
C	4.063360	-1.120909	6.411679
C	2.759800	-0.700257	6.166015
H	1.954378	-1.420007	6.257072
C	2.431668	0.613528	5.804381
C	2.226845	5.804611	5.672169
C	2.798060	6.495831	6.711301
H	3.373854	6.016313	7.487843
C	2.598730	7.877825	6.705535
H	3.042470	8.468112	7.497224
C	1.843260	8.483975	5.706927
H	1.693677	9.555302	5.719254
C	1.272524	7.715476	4.697944
H	0.677307	8.178143	3.920924
C	1.446634	6.329781	4.671521
H	0.999733	5.715894	3.902955
F	1.307655	4.122026	6.400731
F	1.934428	3.247319	3.467916
O	7.372574	1.667243	6.232795
N	5.512693	3.470866	6.672173
C	6.201034	4.673412	6.790932
F	5.901895	5.572458	5.800758
F	5.847242	5.283572	7.945141
F	7.552220	4.572552	6.795703
C	4.038374	4.785532	1.731052
H	4.321364	5.179059	0.755827
H	3.136819	4.182712	1.620089
H	3.785738	5.631836	2.375205
C	0.999880	0.985296	5.536631
H	0.846357	1.162592	4.470658
H	0.331809	0.186864	5.856202
H	0.728762	1.906435	6.053952
C	4.363544	-2.537673	6.813903
H	3.495889	-3.179874	6.666908
H	5.196825	-2.939655	6.235034
H	4.645624	-2.584898	7.868788
C	8.624225	2.760405	1.251289
H	9.367141	3.540062	1.440780
H	9.085954	1.801630	1.489185
H	8.386676	2.779696	0.187626

Coordinates for **28**

Bi	4.830895	4.401992	2.850408
S	6.719947	2.460791	4.773149
O	7.557207	1.671918	5.657002
N	6.726679	4.037324	4.860105
C	6.494542	3.015280	2.120421
C	7.156233	2.248374	3.068047
C	8.145102	1.322616	2.770411
H	8.624234	0.756867	3.557997
C	8.518550	1.162481	1.438436
C	7.885949	1.950561	0.476071
H	8.187920	1.844535	-0.560157
C	6.884468	2.875560	0.784226
C	4.084311	2.690109	4.189931
C	5.020122	1.941941	4.889426
C	4.705791	0.850034	5.685472
H	5.483785	0.310120	6.207808
C	3.368268	0.487649	5.812371
C	2.411643	1.246916	5.135347
H	1.366093	0.979789	5.244605
C	2.736133	2.339824	4.327848
C	6.497878	4.669785	6.073182
F	7.556897	4.611460	6.923456
F	6.256773	5.982332	5.846381
F	5.432269	4.208909	6.795647
F	3.574856	3.783760	1.273822
C	6.264544	3.701854	-0.310307
H	6.754771	3.514491	-1.264723
H	5.201433	3.482117	-0.407598
H	6.353715	4.768676	-0.088143
C	9.572098	0.164199	1.046055
H	10.172928	-0.133367	1.905186
H	9.110824	-0.736248	0.631439
H	10.234997	0.573260	0.282208
C	1.642484	3.118969	3.647783
H	1.731888	3.050648	2.563593
H	0.660867	2.752993	3.946024
H	1.701210	4.178856	3.910363
C	2.957191	-0.676739	6.670472
H	2.394068	-1.408874	6.087556
H	3.825527	-1.175250	7.100079
H	2.312605	-0.347203	7.488698

Coordinates for ***cis*-36**

Bi	1.982693	10.443244	14.078604
S	2.542291	7.280171	14.615333
F	2.910882	12.181074	14.625755
O	2.800404	5.870955	14.802339
F	-0.402016	7.959897	12.474252
C	2.308523	9.546406	16.105469
N	1.169403	7.806224	14.056169
F	1.483387	7.049557	11.877278
C	2.361948	10.269896	17.284069
H	2.145540	11.330015	17.289409
C	-0.111618	11.145726	13.931881
F	0.121214	5.972327	13.169547
C	3.815519	8.001652	13.592794
C	2.983422	7.529954	17.295376
H	3.221667	6.475502	17.280316

C	-2.697506	11.994694	13.950570
H	-3.725821	12.331642	13.954026
C	2.709465	9.620707	18.470719
C	3.019816	8.265987	18.476751
H	3.292678	7.775173	19.401069
C	3.691981	9.328240	13.183860
C	-1.740510	12.710948	13.239553
H	-2.016461	13.601346	12.690133
C	4.653148	9.925960	12.399025
H	4.557767	10.953212	12.081804
C	4.909618	7.243508	13.205752
H	4.991411	6.212868	13.521625
C	2.616126	8.188954	16.133583
C	-0.413044	12.289124	13.218837
H	0.332186	12.833042	12.660467
C	5.876198	7.833232	12.395953
H	6.728131	7.249817	12.074312
C	0.619132	7.208451	12.913610
C	-2.345994	10.848981	14.656420
H	-3.091513	10.291203	15.207633
C	-1.026127	10.404559	14.658655
H	-0.746891	9.511525	15.200587
C	5.750061	9.157820	11.999585
F	2.314167	11.866493	11.883915
H	2.743336	10.184178	19.394041
H	6.504301	9.609668	11.369160
B	1.805381	11.166789	10.715396
F	0.809375	11.939102	10.136038
F	1.280330	9.951604	11.201495
F	2.858962	10.940324	9.842266

Coordinates for ***cis2*-36'**

Bi	2.191226	10.474922	13.883799
S	2.544973	7.296543	14.655760
F	2.752650	11.898235	12.493803
O	2.713526	5.897621	14.977888
F	-0.650637	8.013488	12.907335
C	2.673826	9.717141	15.973692
N	1.151828	7.884396	14.222384
F	1.135794	7.179140	12.002084
C	2.839007	10.492235	17.103696
H	2.684487	11.558328	17.074329
C	0.065830	11.025567	13.675784
F	0.028209	6.027659	13.467264
C	3.711676	7.778706	13.406433
C	3.281975	7.725619	17.226648
H	3.432744	6.655479	17.256792
C	-2.563278	11.739363	13.499234
H	-3.605447	12.022372	13.428592
C	3.230373	9.881500	18.297689
C	3.463660	8.515090	18.357631
H	3.777843	8.054766	19.284428
C	3.605203	9.062028	12.875970
C	-1.690933	12.059168	12.463736
H	-2.048936	12.587878	11.589993
C	4.474392	9.481237	11.888496
H	4.410142	10.483603	11.491301
C	4.674697	6.888485	12.958375
H	4.735892	5.895146	13.380452
C	2.874504	8.339109	16.051823
C	-0.346132	11.706340	12.543712
H	0.346014	11.962312	11.754935
C	5.537336	7.303091	11.947784
H	6.288880	6.619884	11.575939
C	0.445886	7.282683	13.175929
C	-2.108489	11.063019	14.627203
H	-2.788823	10.819913	15.432777
C	-0.770520	10.694396	14.727799
H	-0.412747	10.159832	15.597920
C	5.435630	8.583396	11.417636
F	2.545375	12.662767	15.065772
H	3.360091	10.493018	19.180957
H	6.110974	8.897489	10.632640
B	3.984642	12.946421	15.095054
F	4.345947	13.200819	16.408426
F	4.259260	14.009035	14.266233
F	4.587158	11.747608	14.633047

Coordinates for **TSA''**

Bi	2.487474	10.159899	14.102936
S	2.874921	7.001830	14.475767
F	2.032130	11.403516	12.325137
O	2.746997	5.565881	14.570988
F	-0.453200	8.503174	13.650022
C	3.723726	9.136028	15.885974
N	1.543220	7.916283	14.458506
F	0.788978	7.292771	12.341049
C	4.401930	9.731231	16.940685
H	4.440269	10.807747	17.032339
C	0.311912	11.377373	15.319704
F	-0.131596	6.396248	14.082138
C	3.780690	7.497592	13.030360
C	4.351318	6.927848	16.761309
H	4.314872	5.850524	16.670593
C	-2.212652	10.857553	15.728809
H	-3.257727	10.633263	15.893146
C	5.040909	8.937985	17.897228
C	5.018575	7.549442	17.811452
H	5.516496	6.948064	18.560200
C	3.701683	8.844879	12.696258
C	-1.272467	10.461961	16.672527
H	-1.563926	9.931913	17.569959
C	4.385296	9.294415	11.579132
H	4.323057	10.333293	11.283535
C	4.526531	6.580776	12.305878
H	4.559233	5.539118	12.594583
C	3.718776	7.748037	15.838224
C	0.091869	10.753247	16.495944
H	0.851116	10.492422	17.216600
C	5.210912	7.046988	11.187342

H	5.794008	6.356876	10.592537
C	0.469710	7.519923	13.643079
C	-1.832724	11.548025	14.581830
H	-2.561808	11.851593	13.841750
C	-0.485516	11.866387	14.345738
H	-0.134867	12.375483	13.461226
C	5.138260	8.389588	10.828007
F	1.984150	12.629955	15.412502
H	5.562536	9.414196	18.718073
H	5.668676	8.738807	9.951343
B	2.756085	13.141270	16.583458
F	2.405587	12.359769	17.677127
F	2.419568	14.468008	16.753524
F	4.095660	12.972414	16.248099

Coordinates for **TSB’’**

Bi	2.213908	9.838686	14.259074
S	3.080990	6.718506	14.405525
F	1.640331	11.111465	12.515717
O	3.225022	5.278910	14.388820
F	-0.369455	7.768295	13.480970
C	3.539705	8.855375	15.983380
N	1.618270	7.373130	14.414404
F	1.086524	6.748110	12.229000
C	4.095674	9.468671	17.098931
H	3.956959	10.528479	17.269923
C	0.166278	11.342849	15.710365
F	0.155312	5.693122	13.873320
C	3.919703	7.482346	13.034589
C	4.507951	6.719677	16.725443
H	4.650462	5.660360	16.558475
C	-2.410704	11.480775	15.940057
H	-3.486748	11.537617	16.032024
C	4.844632	8.726374	18.014580
C	5.051467	7.362071	17.832389
H	5.632719	6.797036	18.548974
C	3.642304	8.826974	12.811752
C	-1.689156	10.694941	16.831791
H	-2.180288	10.133543	17.616321
C	4.267309	9.467279	11.754185
H	4.050916	10.506289	11.544715
C	4.802319	6.753781	12.252492
H	4.984775	5.706922	12.453630
C	3.763133	7.491415	15.844500
C	-0.285928	10.621816	16.751858
H	0.322849	10.046503	17.432879
C	5.423038	7.409963	11.194055
H	6.107579	6.866477	10.556488
C	0.660643	6.895155	13.514006
C	-1.771831	12.203863	14.938935
H	-2.329731	12.810353	14.236827
C	-0.370059	12.172844	14.800116
H	0.169230	12.701116	14.029327
C	5.157070	8.753621	10.949298
F	2.077374	11.977703	16.373351
H	5.273475	9.220976	18.877620
H	5.640725	9.253170	10.119356
B	3.047693	12.767441	15.620134
F	4.005001	13.223701	16.486044
F	2.391952	13.760376	14.936498
F	3.625413	11.824808	14.688746

Coordinates for **TSC’’**

Bi	2.063182	10.361561	14.494380
S	2.631910	7.162164	14.756531
F	2.100993	11.959873	12.888975
O	2.757690	5.724505	14.829405
F	-0.851563	8.085454	13.807158
C	3.008953	9.340227	16.357406
N	1.205983	7.854134	14.632646
F	0.692775	7.514667	12.388651
C	3.435579	9.971356	17.509645
H	3.307526	11.034808	17.648778
C	0.084279	11.353680	13.334954
F	-0.023683	6.077420	13.843847
C	3.620723	7.827928	13.439030
C	3.832338	7.207910	17.195663
H	3.970822	6.144547	17.054907
C	-2.406463	11.842252	12.504237
H	-3.417310	12.036962	12.172121
C	4.058121	9.216575	18.507925
C	4.259415	7.850440	18.352729
H	4.745776	7.279190	19.131847
C	3.456524	9.182899	13.165597
C	-1.612685	10.941889	11.803377
H	-1.988180	10.433553	10.924802
C	4.193628	9.754299	12.143812
H	4.070467	10.804152	11.909418
C	4.501959	7.023186	12.734807
H	4.601563	5.973028	12.972128
C	3.202210	7.971245	16.223274
C	-0.295494	10.692615	12.203122
H	0.348085	10.026571	11.649791
C	5.235800	7.609045	11.706763
H	5.924061	7.004190	11.131798
C	0.290518	7.382092	13.689745
C	-1.906801	12.509429	13.617558
H	-2.519118	13.215043	14.164205
C	-0.597929	12.286610	14.060393
H	-0.184125	12.801125	14.913355
C	5.082275	8.960287	11.414996
F	1.805775	12.704063	16.079045
H	4.392072	9.711567	19.410752
H	5.655765	9.403931	10.611394
B	3.145555	13.123349	15.858631
F	3.810821	13.258472	17.063383
F	3.200176	14.258734	15.089320
F	3.755206	12.006909	15.121607

Coordinates for **TSD’’**

Bi	1.565816	9.841895	13.831432
S	2.841227	6.919388	14.485749
F	1.930915	11.840840	14.564182
O	3.334770	5.574437	14.683390
F	-0.016780	7.033374	12.114447
C	2.057383	9.076748	15.920609
N	1.437941	7.182939	13.800518
F	2.062592	6.604966	11.632759
C	1.942581	9.807404	17.094564
H	1.561154	10.820117	17.076204
C	-0.289558	11.713924	14.146099
F	0.908374	5.190921	12.795669
C	4.030850	7.908107	13.597108
C	3.035550	7.225575	17.187705
H	3.467706	6.234352	17.199138
C	-2.572540	12.980426	14.267717
H	-3.518343	13.502800	14.315111
C	2.350546	9.248157	18.307695
C	2.886747	7.966576	18.357020
H	3.198836	7.542212	19.301726
C	3.645240	9.186784	13.206517
C	-1.761801	13.132029	13.149497
H	-2.057400	13.758271	12.317571
C	4.543736	9.995935	12.536596
H	4.258327	10.985091	12.207489
C	5.301388	7.413644	13.346881
H	5.573682	6.416488	13.664191
C	2.603489	7.799776	16.003228
C	-0.516115	12.483388	13.063355
H	0.144054	12.585701	12.216813
C	6.201609	8.231599	12.669887
H	7.195073	7.864169	12.450727
C	1.121318	6.517735	12.609876
C	-2.181216	12.175948	15.330475
H	-2.806909	12.049088	16.204571
C	-0.948717	11.498542	15.305890
H	-0.621677	10.870886	16.119617
C	5.826620	9.509345	12.272475
F	1.961211	11.486290	11.538215
H	2.252144	9.823726	19.219279
H	6.531412	10.137987	11.743823
B	1.412774	10.727705	10.449867
F	0.230623	11.344409	10.032542
F	1.127516	9.436711	10.957031
F	2.340810	10.655354	9.420182

Coordinates for **TSE’’**

Bi	1.784386	10.213234	14.258795
S	2.764884	7.103454	14.753236
F	2.738627	12.016757	14.724213
O	3.144151	5.716471	14.925818
F	-0.329988	7.623767	12.781643
C	2.429118	9.348128	16.259241
N	1.311140	7.499037	14.289676
F	1.566232	6.815072	12.077416
C	2.525524	10.079796	17.431825
H	2.221869	11.118482	17.456713
C	-0.324866	11.811116	13.236017
F	0.311699	5.640763	13.393468
C	3.907344	7.938435	13.667473
C	3.373510	7.406104	17.394072
H	3.699909	6.375787	17.357216
C	-2.657525	12.827739	13.747471
H	-3.630028	13.248209	13.963653
C	3.034181	9.476592	18.583833
C	3.454167	8.151324	18.566757
H	3.848068	7.693275	19.464039
C	3.621126	9.258996	13.333960
C	-1.514727	13.485908	14.187337
H	-1.574646	14.410735	14.746520
C	4.491150	9.939913	12.499224
H	4.292622	10.963743	12.212084
C	5.035511	7.278683	13.204006
H	5.226335	6.252370	13.485529
C	2.849395	8.022784	16.268361
C	-0.233611	12.974905	13.904948
H	0.685449	13.450228	14.213064
C	5.897302	7.971817	12.359589
H	6.777549	7.476904	11.971749
C	0.750232	6.905499	13.161530
C	-2.570162	11.645208	13.022040
H	-3.455463	11.128732	12.674072
C	-1.320364	11.077657	12.706969
H	-1.205256	10.172686	12.131829
C	5.626532	9.290709	12.011918
F	1.399889	11.848377	11.908025
H	3.106044	10.051361	19.498396
H	6.299834	9.822558	11.352029
B	1.331607	11.004741	10.700433
F	1.097438	9.701169	11.156818
F	2.551004	11.113781	10.057688
F	0.279325	11.457683	9.921300

Coordinates for **TSF’’**

Bi	1.897942	10.215820	14.081526
S	2.795331	7.092961	14.704542
F	2.830164	12.019192	14.541026
O	3.154526	5.709283	14.936711
F	-0.280354	7.470789	12.647374
C	2.412290	9.381831	16.132720
N	1.372354	7.480217	14.144510
F	1.708965	7.050008	11.881923
C	2.448226	10.136332	17.294922
H	2.166962	11.181543	17.278204
C	-0.572530	11.276377	13.215116
F	0.676146	5.566271	13.075837
C	4.006542	7.884812	13.662280
C	3.234566	7.445034	17.369891
H	3.538739	6.407353	17.376247
C	-2.652719	12.584162	14.091538

H	-3.512619	13.126888	14.459378
C	2.865885	9.548529	18.490421
C	3.255478	8.214347	18.529315
H	3.578975	7.768298	19.460172
C	3.761068	9.197685	13.269775
C	-1.533915	13.285960	13.659698
H	-1.499590	14.367313	13.689981
C	4.684454	9.835644	12.458427
H	4.516057	10.851740	12.129101
C	5.145230	7.192071	13.279288
H	5.302484	6.172762	13.603696
C	2.799674	8.048138	16.199394
C	-0.404338	12.612492	13.158948
H	0.477818	13.122207	12.804840
C	6.062307	7.842087	12.459314
H	6.952878	7.320429	12.135285
C	0.903157	6.899124	12.969452
C	-2.688269	11.195046	14.044127
H	-3.558878	10.641544	14.371376
C	-1.593521	10.462617	13.553438
H	-1.589661	9.386774	13.479921
C	5.832716	9.152038	12.054196
F	2.176799	11.652726	11.536353
H	2.890624	10.141776	19.395655
H	6.549326	9.650702	11.414227
B	1.213728	11.099142	10.665676
F	0.481503	12.099551	10.052795
F	0.308208	10.319576	11.522980
F	1.788541	10.227793	9.765875

Coordinates for **38**

Bi	1.377465	10.27803	14.1746
S	2.553833	7.376272	14.78275
O	2.857041	5.973442	14.94308
F	-0.44151	7.890394	12.61718
C	2.086846	9.599344	16.22594
N	1.117904	7.826002	14.21414
F	1.491188	7.081074	12.03648
C	2.117316	10.35953	17.38561
H	1.772664	11.38534	17.37995
F	0.186826	5.931997	13.32487
C	3.732562	8.221013	13.75626
C	3.054711	7.710069	17.44831
H	3.410449	6.688762	17.44958
C	2.607906	9.800257	18.56712
C	3.071625	8.488889	18.60146
H	3.448001	8.068782	19.52434
C	3.38515	9.516979	13.38808
C	4.282565	10.23345	12.60983
H	4.058029	11.24402	12.29844
C	4.924874	7.612857	13.39824
H	5.149874	6.602495	13.71115
C	2.551037	8.29174	16.29586
C	5.808556	8.345769	12.61199
H	6.744353	7.900742	12.30185
C	0.614726	7.185454	13.07121
C	5.487789	9.642679	12.22456
F	2.504413	12.31224	14.85931
H	2.629696	10.39868	19.46891
H	6.181518	10.20618	11.61384
B	2.647637	13.33047	13.79644
F	1.91143	14.43572	14.16522
F	2.119487	12.71637	12.64325
F	3.99281	13.60114	13.65056

Coordinates for **179**

Bi	1.992813	10.44165	14.09438
S	2.519971	7.281867	14.64117
F	2.912834	12.18558	14.62723
O	2.764865	5.871414	14.83279
F	-0.44465	7.968486	12.5287
C	2.324761	9.557567	16.12389
N	1.147863	7.820998	14.0907
F	1.431639	7.052817	11.91187
C	2.400183	10.2871	17.29706
H	2.196426	11.34971	17.29994
C	-0.09274	11.16967	13.94391
F	0.085422	5.984332	13.22741
C	3.791113	7.983114	13.60178
C	2.992875	7.540003	17.3144
H	3.220131	6.483064	17.30216
C	-2.66386	12.04966	13.92169
C	2.754874	9.63934	18.48266
C	3.050839	8.281607	18.49154
H	3.329539	7.792374	19.41494
C	3.678441	9.307698	13.18328
C	-1.68422	12.78578	13.26854
H	-1.93391	13.69922	12.74876
C	4.63352	9.890746	12.3812
H	4.543782	10.91618	12.05648
C	4.872527	7.211337	13.20626
H	4.947931	6.181958	13.52795
C	2.618357	8.196714	16.1537
C	-0.36386	12.34415	13.2731
H	0.400677	12.90282	12.75704
C	5.834904	7.785366	12.38014
H	6.676684	7.190665	12.05258
C	0.580922	7.217598	12.95745
C	-2.35072	10.87078	14.59417
H	-3.11928	10.30256	15.10052
C	-1.03973	10.41415	14.61434
H	-0.79023	9.496942	15.12903
C	5.717458	9.107757	11.97447
F	2.310608	11.85445	11.89997
H	2.805863	10.20683	19.40268
H	6.467964	9.546817	11.33074
B	1.755908	11.14672	10.75498
F	0.746991	11.92134	10.20513
F	1.237818	9.941815	11.27671
F	2.776293	10.90013	9.851064

C	-4.09785	12.50636	13.91491
F	-4.27177	13.6667	13.2562
F	-4.90767	11.59171	13.33225
F	-4.56807	12.68833	15.17162

Coordinates for **CF_3TSF''**

Bi	1.901407	10.21508	14.04259
S	2.788401	7.105218	14.67925
F	2.787708	12.04053	14.49307
O	3.145912	5.723165	14.91828
F	-0.2613	7.455573	12.57696
C	2.368879	9.393099	16.10382
N	1.37654	7.491713	14.09001
F	1.74485	7.093014	11.82749
C	2.374763	10.14684	17.26622
H	2.088531	11.1905	17.24519
C	-0.53168	11.21675	13.23193
F	0.7381	5.571404	12.99623
C	4.018024	7.898584	13.65946
C	3.170274	7.457384	17.3532
H	3.478036	6.42084	17.365
C	-2.61507	12.4996	14.19111
C	2.767686	9.557463	18.46956
C	3.161537	8.224963	18.51414
H	3.464985	7.778007	19.45123
C	3.779154	9.209877	13.25731
C	-1.51751	13.22341	13.73198
H	-1.50201	14.30434	13.7721
C	4.712897	9.850995	12.4621
H	4.548629	10.86635	12.12783
C	5.165724	7.209271	13.2983
H	5.320269	6.191113	13.62761
C	2.759705	8.060133	16.1741
C	-0.40609	12.56675	13.19586
H	0.457739	13.09614	12.82519
C	6.095425	7.860563	12.49338
H	6.992992	7.340497	12.18671
C	0.931811	6.909065	12.90459
C	-2.64016	11.11068	14.13116
H	-3.49688	10.54933	14.4761
C	-1.55437	10.40727	13.59943
H	-1.54785	9.332342	13.51056
C	5.870321	9.168807	12.0809
F	2.147288	11.61619	11.50093
H	2.768759	10.14864	19.37635
H	6.597236	9.66783	11.45308
B	1.135461	11.1154	10.66082
F	0.399825	12.13971	10.10649
F	0.233849	10.33976	11.56001
F	1.630244	10.22933	9.736154
C	-3.77829	13.25117	14.77851
F	-4.11866	14.31827	14.0245
F	-4.87599	12.48157	14.90354
F	-3.48744	13.72771	16.01302

Coordinates for **17**

C	-0.93523	0.003845	-5.72396
C	-2.12872	-0.22331	-5.06111
C	0.261089	0.212655	-5.06239
C	-2.11851	-0.24086	-3.67425
C	0.259457	0.193584	-3.67417
C	-0.92653	-0.03544	-2.98291
H	-3.03922	-0.38365	-5.6216
H	1.169237	0.383829	-5.6235
H	-3.03829	-0.4196	-3.13464
H	1.183374	0.352355	-3.13644
F	-0.93916	0.019562	-7.0767
C	-0.9423	-0.01338	-1.48487
F	-1.32722	1.195955	-0.99612
F	-1.80481	-0.91847	-0.96659
F	0.270031	-0.27151	-0.94836

Coordinates for **36-cation**

Bi	4.417451	4.280343	6.636699
S	6.600600	2.074138	5.851793
C	5.375152	4.210142	4.644632
C	6.241633	3.131566	4.472615
C	6.820244	2.855920	3.245495
H	7.488886	2.014135	3.129779
C	6.527070	3.701698	2.177500
H	6.976797	3.511611	1.212675
C	5.667486	4.779564	2.344200
H	5.445960	5.427666	1.506758
C	5.074816	5.039326	3.583127
H	4.394349	5.871942	3.698886
C	4.016119	2.095782	6.742008
C	5.048992	1.293417	6.258563
C	4.881375	-0.073386	6.112228
H	5.690956	-0.685139	5.738973
C	3.657417	-0.631363	6.474438
H	3.512633	-1.698903	6.380727
C	2.628647	0.170032	6.952501
H	1.681942	-0.274002	7.229965
C	2.795029	1.552248	7.079601
H	1.982148	2.170755	7.434050
O	7.602172	1.085226	5.541763
N	6.827218	3.172877	6.966344
C	7.249669	2.764593	8.244760
F	8.535648	2.362698	8.259591
F	7.139123	3.804761	9.088875
F	6.527722	1.744777	8.784376
F	2.509350	4.809684	6.115262
C	4.762260	5.916705	8.075343
C	6.011084	6.511854	8.072423
C	3.701878	6.305151	8.874471
C	6.203336	7.575767	8.949524
C	3.926820	7.371123	9.741897
C	5.168140	7.998280	9.777331
H	6.813631	6.175644	7.431025
H	2.737238	5.819570	8.833769

H	7.165968	8.068802	8.977578
H	3.124421	7.704787	10.386432
H	5.329975	8.825685	10.455487

Coordinates for **TScat**

Bi	5.408297	3.944563	7.158119
S	6.723041	1.460763	5.581384
C	5.935059	3.991470	4.974543
C	6.484213	2.817548	4.465745
C	6.784362	2.648224	3.124611
H	7.199532	1.717562	2.762759
C	6.541317	3.717457	2.265416
H	6.777954	3.620158	1.214640
C	5.994966	4.898899	2.751794
H	5.801990	5.720314	2.074124
C	5.686121	5.041212	4.107041
H	5.243197	5.962296	4.462952
C	4.358605	1.968435	6.812939
C	5.071236	1.019749	6.085700
C	4.519579	-0.196808	5.719149
H	5.094530	-0.911850	5.146876
C	3.213239	-0.468358	6.118432
H	2.763371	-1.416865	5.858265
C	2.490193	0.469964	6.845203
H	1.474409	0.250635	7.147252
C	3.058629	1.697610	7.196859
H	2.479772	2.419549	7.758294
O	7.407485	0.342318	4.978334
N	7.355660	2.247278	6.806884
C	7.796699	1.543100	7.934810
F	8.944684	0.870825	7.718542
F	8.026771	2.430362	8.925563
F	6.912767	0.632161	8.427835
F	3.682694	5.210505	6.924222
C	5.073593	6.290692	8.407131
C	5.712326	7.268843	7.734581
C	4.510017	6.181460	9.625990
C	5.894987	8.402848	8.550038
C	4.726997	7.347305	10.385535
C	5.406150	8.434142	9.849408
H	6.067692	7.227234	6.717124
H	3.970978	5.329941	10.009142
H	6.422131	9.245545	8.121233
H	4.339267	7.363725	11.396149
H	5.547725	9.322264	10.449924

Coordinates for **185**

Bi	4.829554	4.395373	6.787238
S	6.632945	2.047056	5.813368
C	5.452440	4.143743	4.622198
C	6.284924	3.052141	4.402554
C	6.791732	2.704232	3.162061
H	7.438614	1.846136	3.039712
C	6.443944	3.515030	2.084588
H	6.826300	3.284565	1.099653
C	5.615705	4.616550	2.272263
H	5.353603	5.237875	1.425773
C	5.118070	4.938604	3.537759
H	4.479604	5.804061	3.659387
C	4.114542	2.228433	6.700082
C	5.074582	1.325017	6.255929
C	4.850664	-0.034595	6.120805
H	5.630178	-0.698658	5.772931
C	3.589009	-0.509598	6.468260
H	3.377687	-1.567413	6.391495
C	2.607876	0.368283	6.917608
H	1.632048	-0.014373	7.187068
C	2.862284	1.737477	7.033790
H	2.083126	2.398787	7.390353
O	7.687678	1.085568	5.630413
N	6.837458	3.239705	6.914540
C	7.347688	2.890905	8.191410
F	8.642650	2.555066	8.136029
F	7.221951	3.961951	8.994386
F	6.701953	1.862850	8.791636

BF4 anion

B	-0.017034	20.233634	8.235443
F	-0.823302	21.389635	8.202293
F	0.781805	20.250473	9.396595
F	0.811526	20.206526	7.094905
F	-0.837835	19.087261	8.246194

10. NMR spectra of Bi(III) compounds

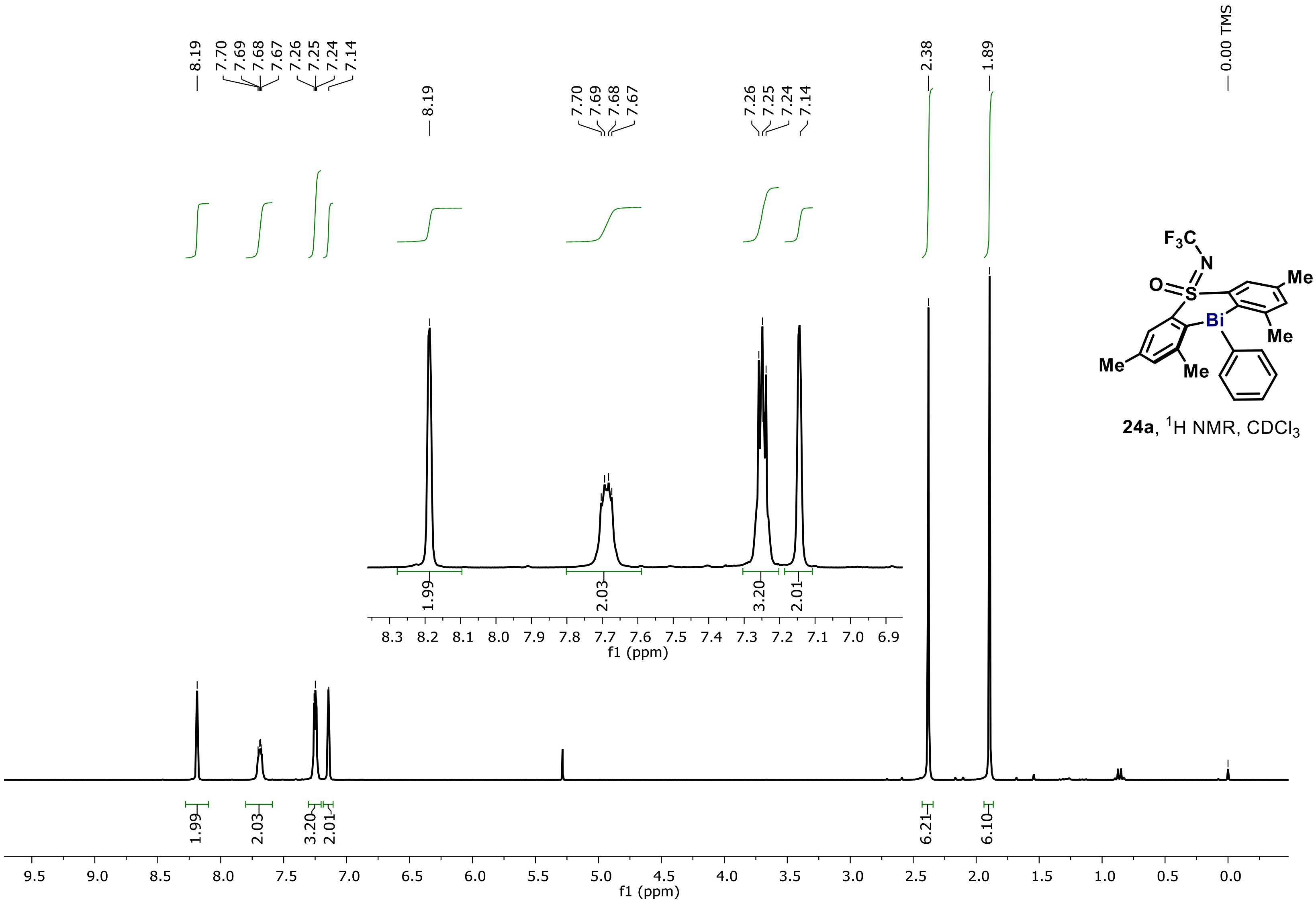

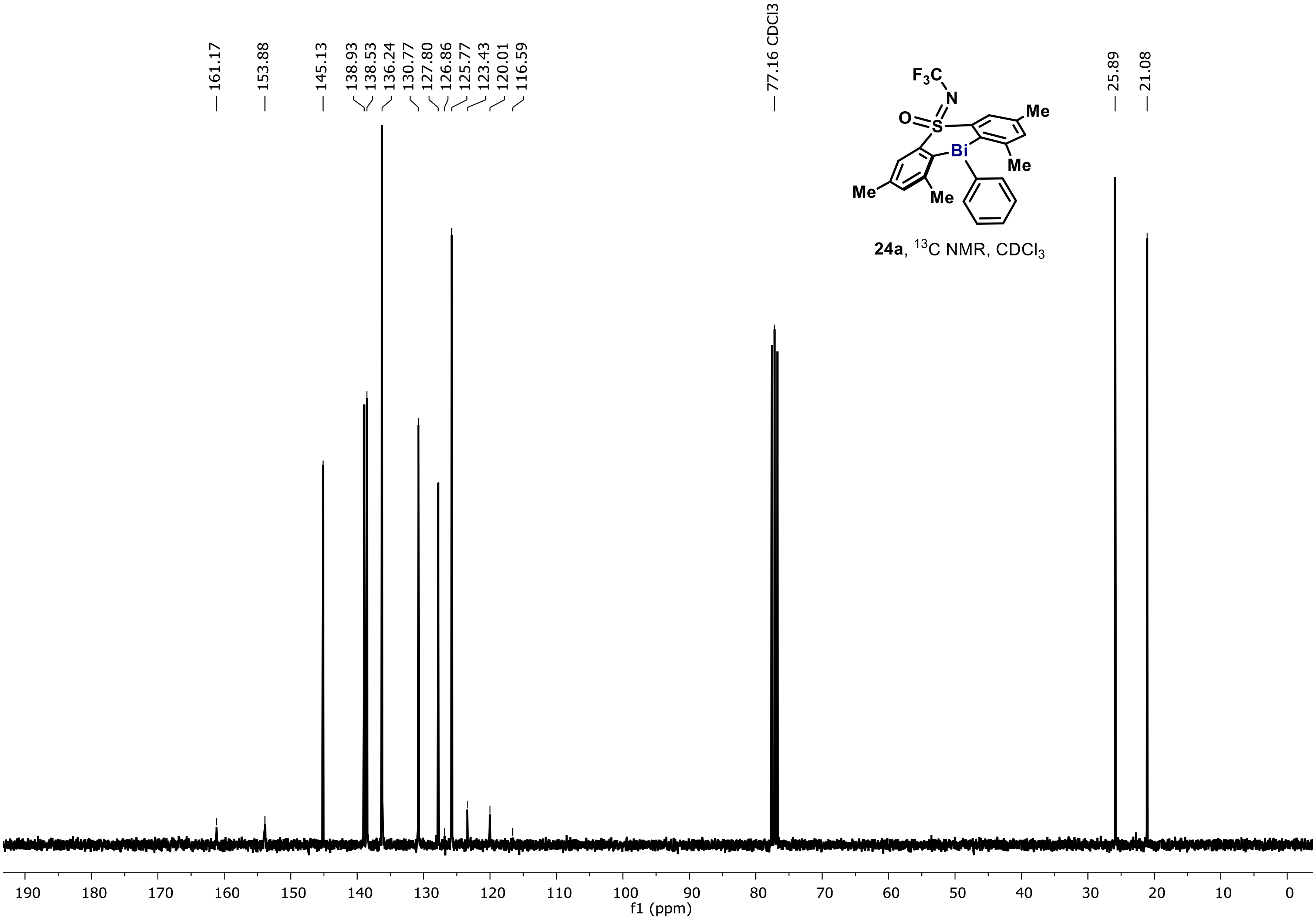
161.17
153.88
145.13
138.93
138.53
136.24
130.77
127.80
126.86
125.77
123.43
120.01
116.59
77.16 CDCl3
25.89
21.08
F3C
N
O
S
Me
Bi
Me
Me
Me
24a, 13C NMR, CDCl3
190
180
170
160
150
140
130
120
110
100
90
80
70
60
50
40
30
20
10
0
f1 (ppm)

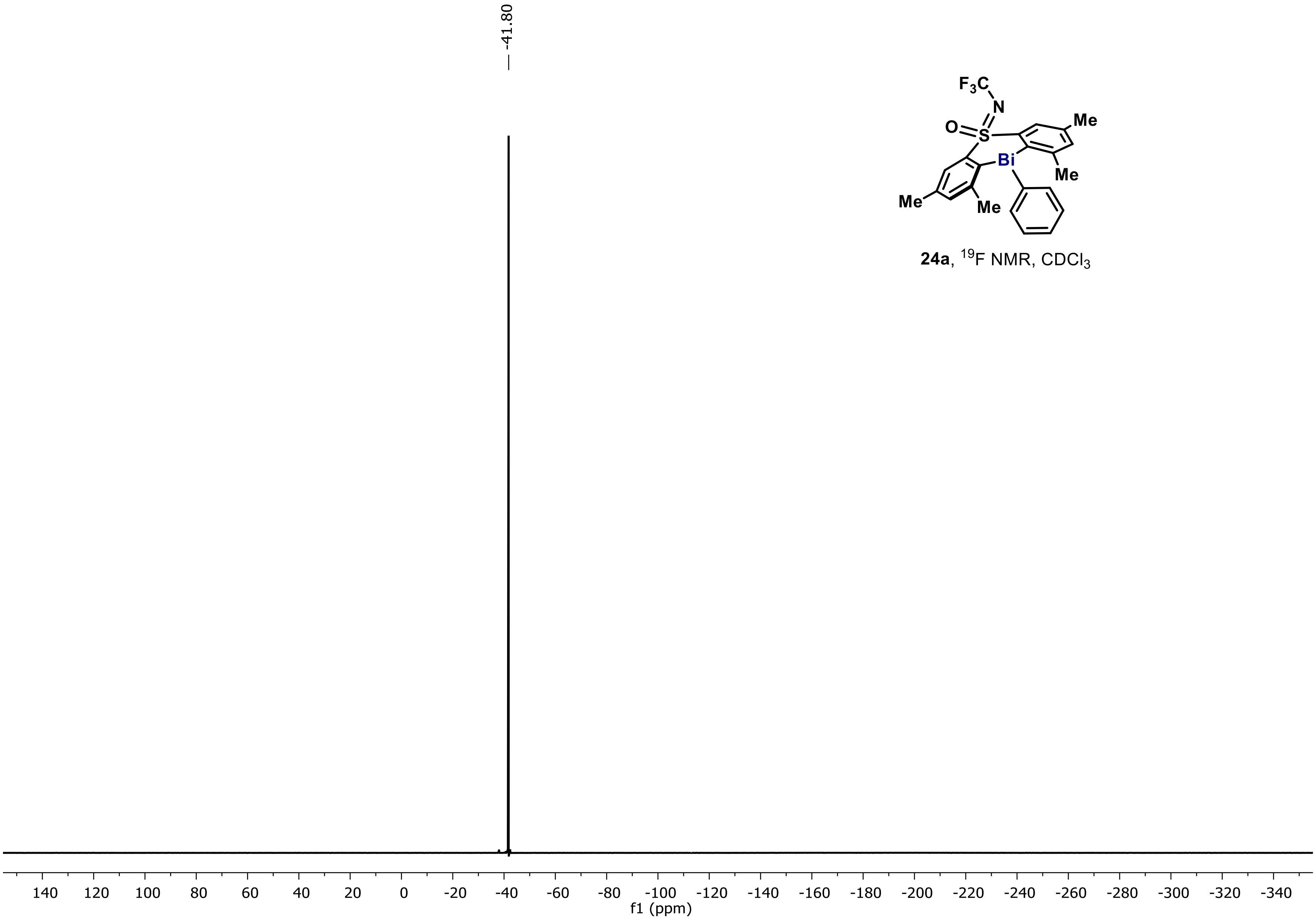

24a, ^{19}F NMR, $CDCl_3$

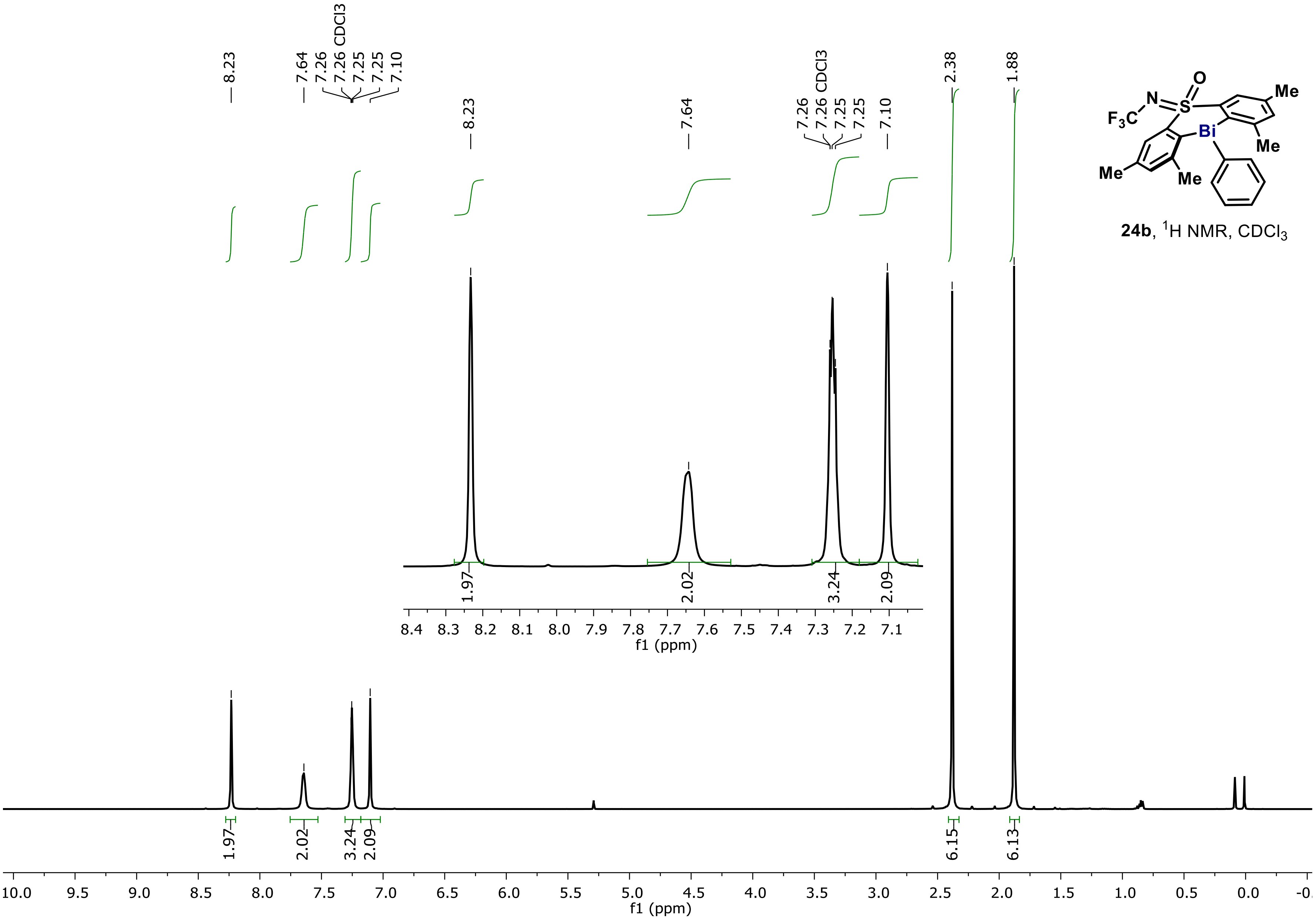

8.23
7.64
7.26
7.26 CDCl3
7.25
7.25
7.10
2.38
1.88
1.97
2.02
3.24
2.09
6.15
6.13
f1 (ppm)
F3C
N
S
O
Bi
Me
Me
Me
Me
24b, 1H NMR, CDCl3

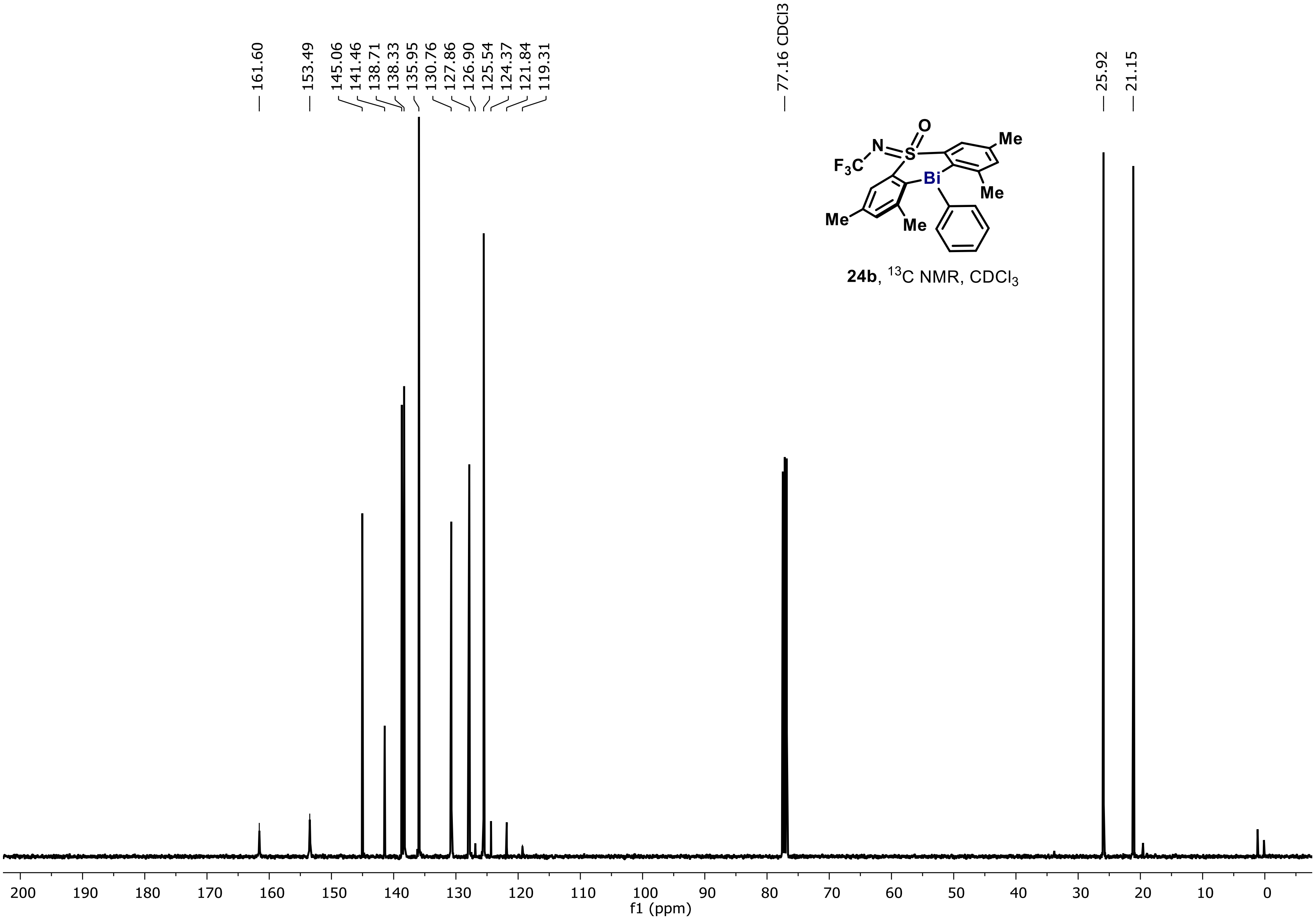
161.60
153.49
145.06
141.46
138.71
138.33
135.95
130.76
127.86
126.90
125.54
124.37
121.84
119.31
77.16 CDCl3
25.92
21.15
O
N
S
F_3C
Bi
Me
Me
Me
Me
24b, ^{13}C NMR, $CDCl_3$
200
190
180
170
160
150
140
130
120
110
100
90
80
70
60
50
40
30
20
10
0
f1 (ppm)

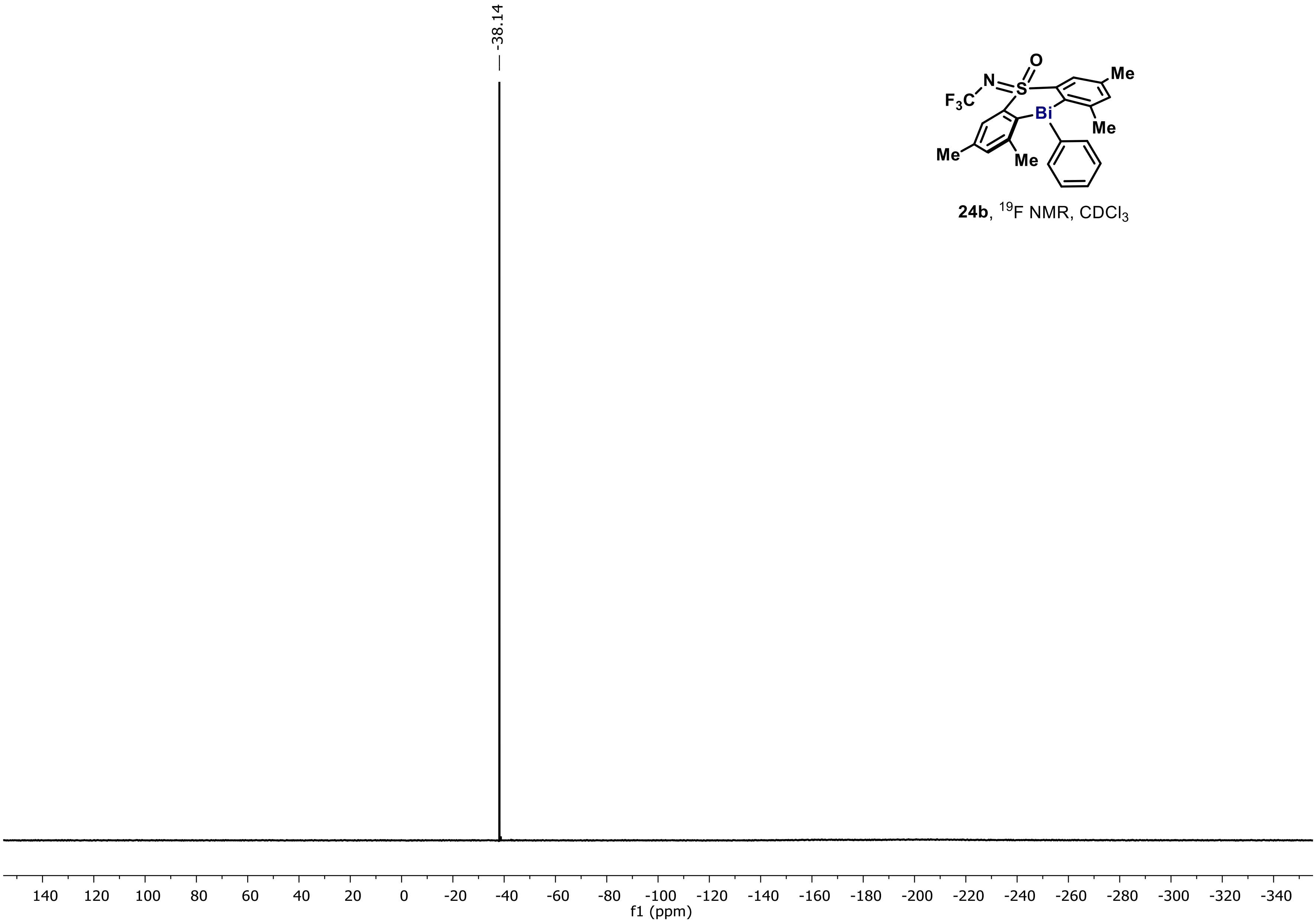
-38.14
O
N
S
F3C
Me
Bi
Me
Me
Me
24b, 19F NMR, CDCl3
140
120
100
80
60
40
20
0
-20
-40
-60
-80
-100
-120
-140
-160
-180
-200
-220
-240
-260
-280
-300
-320
-340
f1 (ppm)

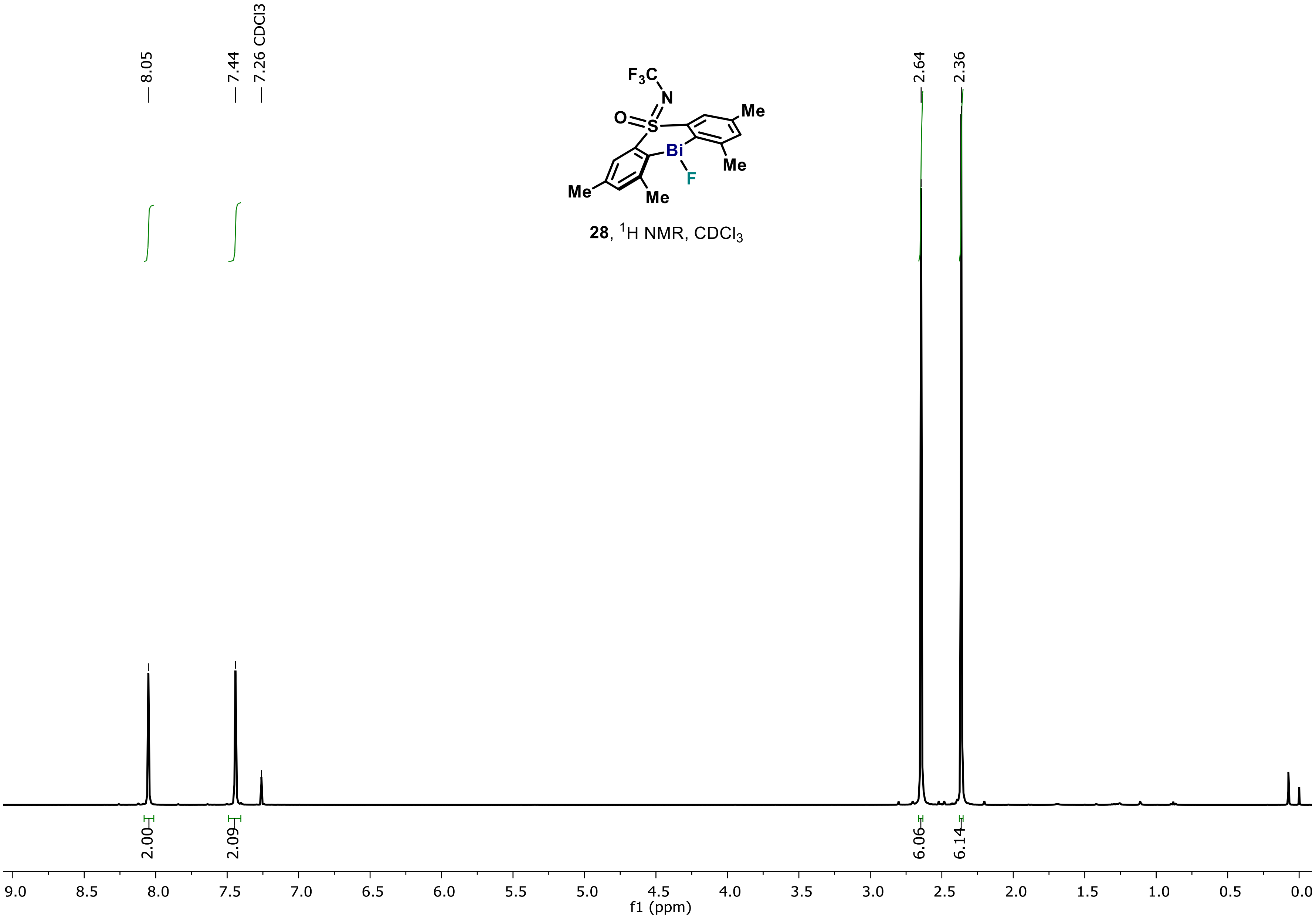

8.05
7.44
7.26 CDCl3
2.64
2.36
F3C
N
O
S
Me
Bi
F
Me
Me
Me
28, 1H NMR, CDCl3
2.00
2.09
6.06
6.14
9.0 8.5 8.0 7.5 7.0 6.5 6.0 5.5 5.0 4.5 4.0 3.5 3.0 2.5 2.0 1.5 1.0 0.5 0.0
f1 (ppm)

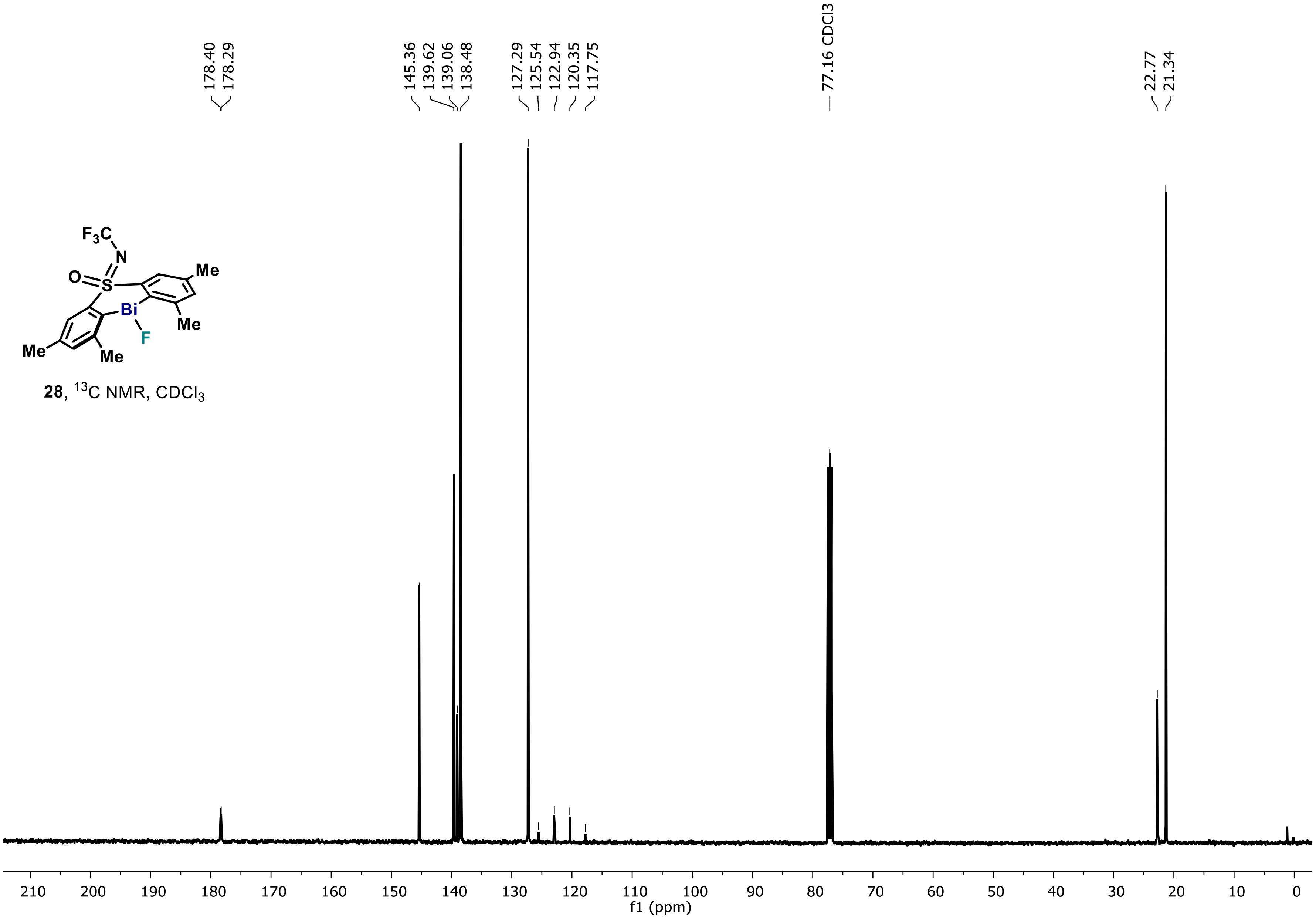
178.40
178.29
145.36
139.62
139.06
138.48
127.29
125.54
122.94
120.35
117.75
77.16 CDCl3
22.77
21.34
F3C
N
O
S
Me
Bi
F
Me
Me
Me
28, 13C NMR, CDCl3
210 200 190 180 170 160 150 140 130 120 110 100 90 80 70 60 50 40 30 20 10 0
f1 (ppm)

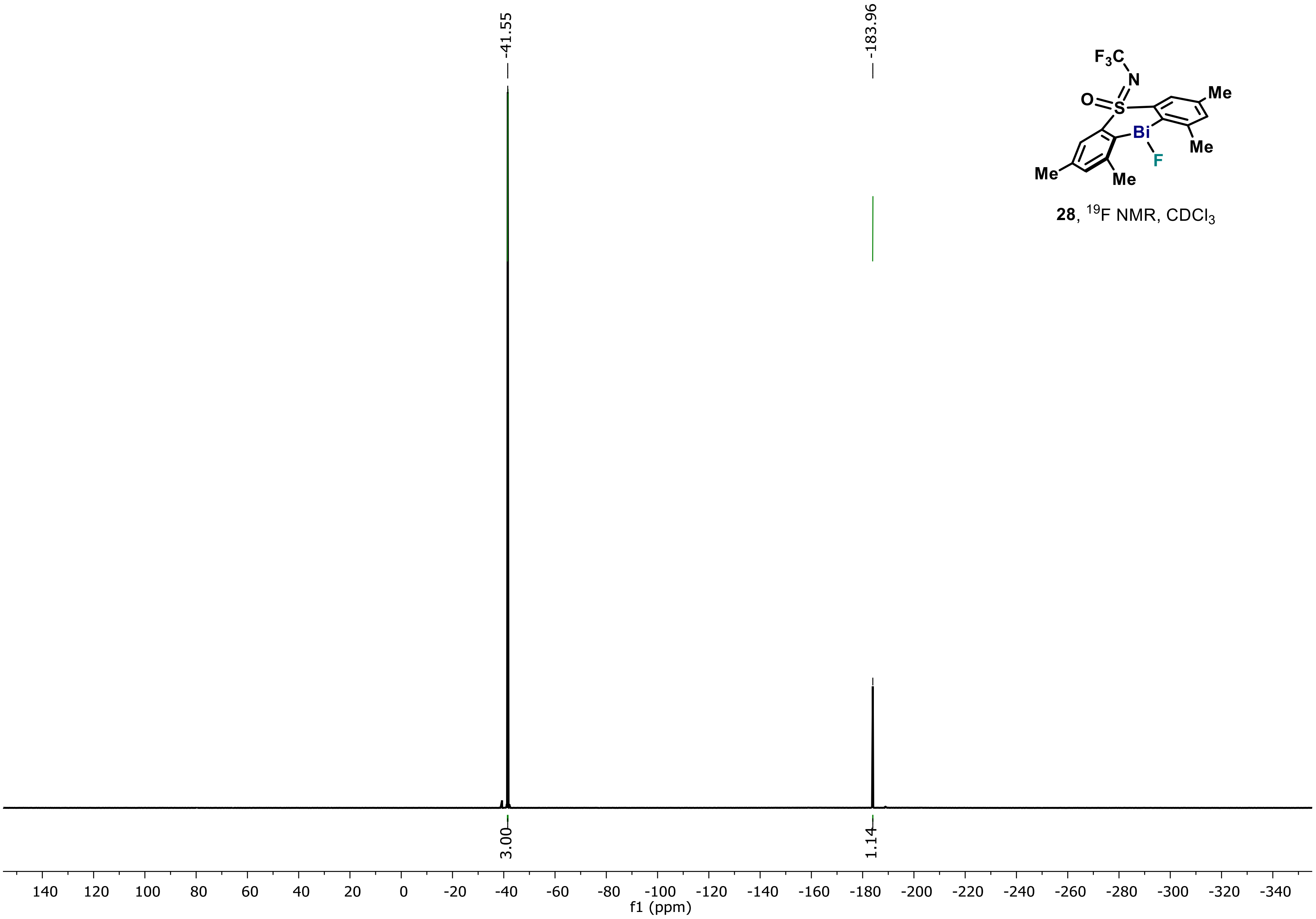

28, ^{19}F NMR, $CDCl_3$

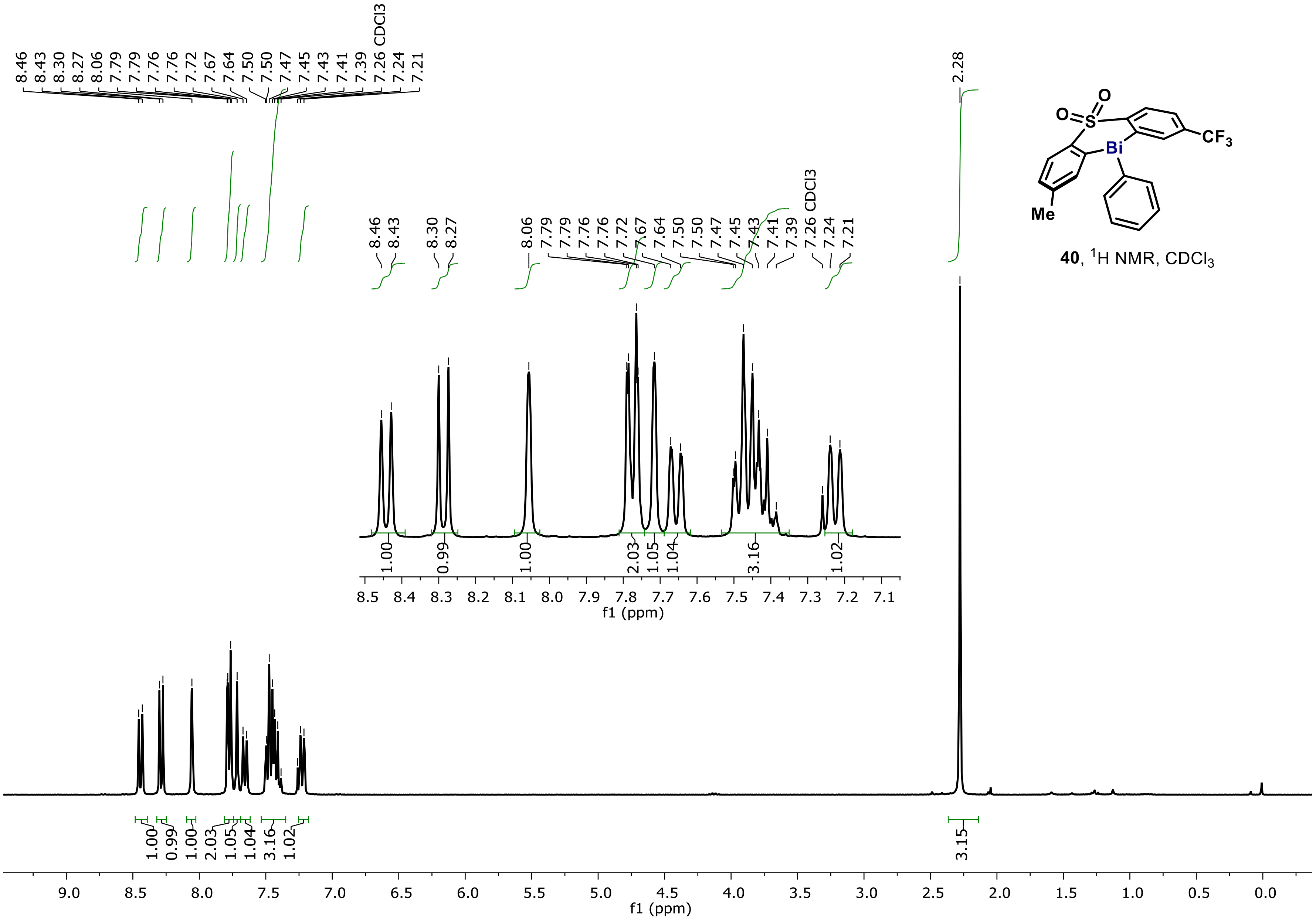
8.46 8.43 8.30 8.27 8.06 7.79 7.79 7.76 7.76 7.72 7.67 7.64 7.50 7.50 7.47 7.45 7.43 7.41 7.39 7.26 CDCl3 7.24 7.21
2.28
1.00 0.99 1.00 2.03 1.05 1.04 3.16 1.02
3.15
f1 (ppm)
Me
CF3
Bi
40, 1H NMR, CDCl3

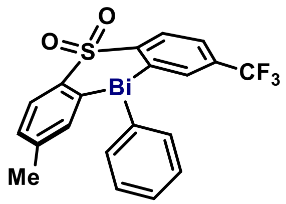

40, ^{19}F NMR, $CDCl_3$

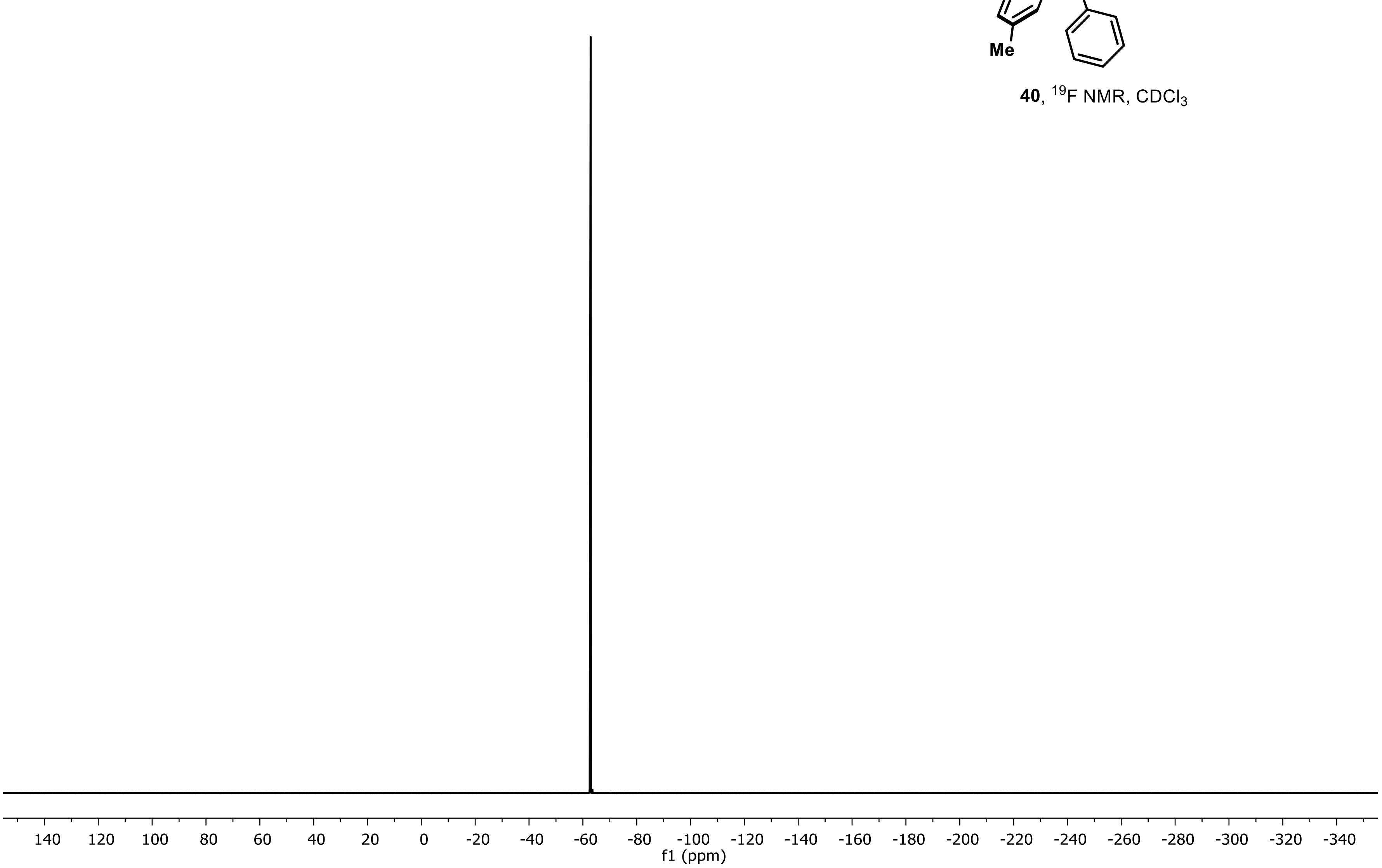

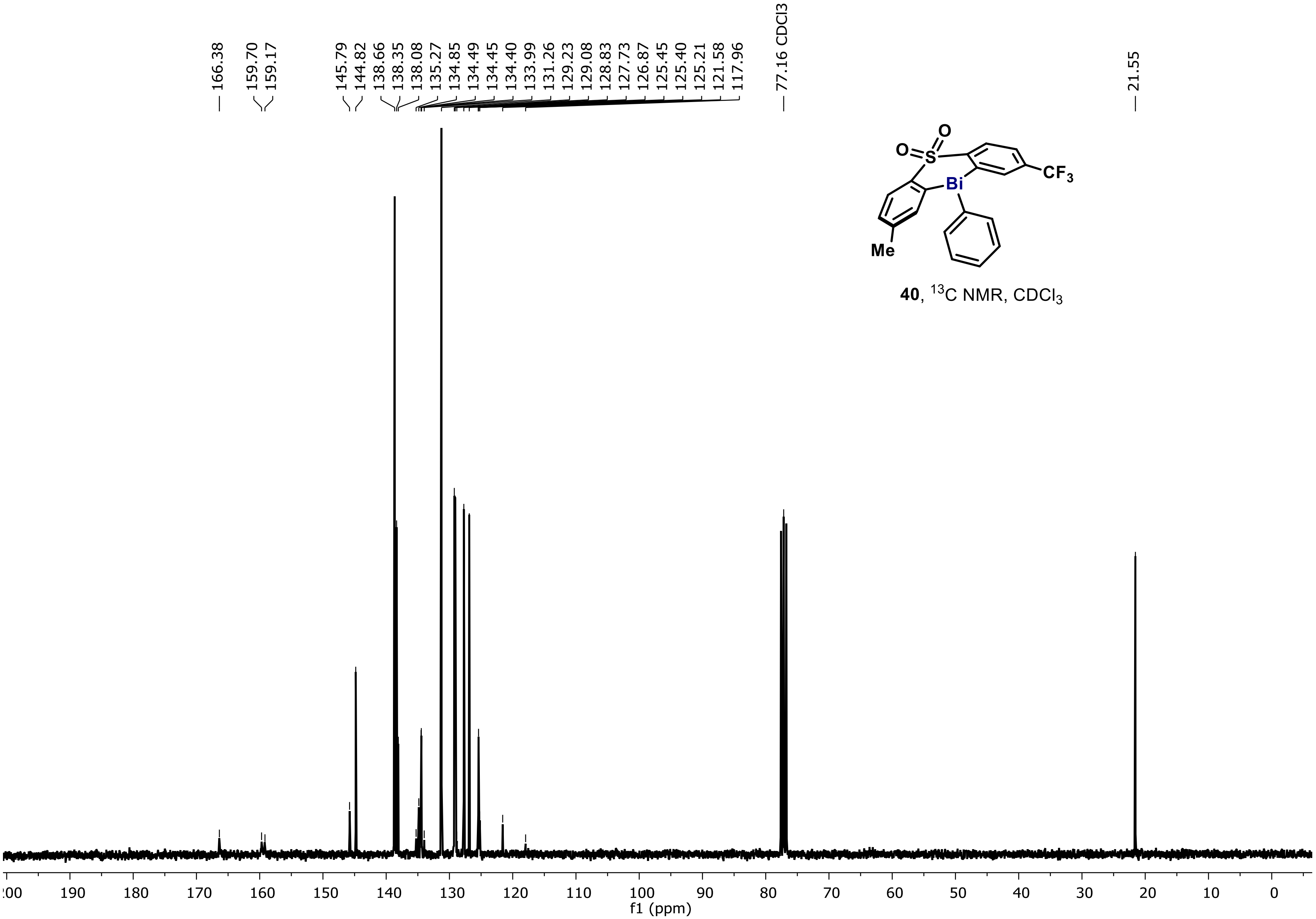
166.38
159.70
159.17
145.79
144.82
138.66
138.35
138.08
135.27
134.85
134.49
134.45
134.40
133.99
131.26
129.23
129.08
128.83
127.73
126.87
125.45
125.40
125.21
121.58
117.96
77.16 CDCl3
21.55
O
O
S
Bi
CF3
Me
40, 13C NMR, CDCl3
f1 (ppm)

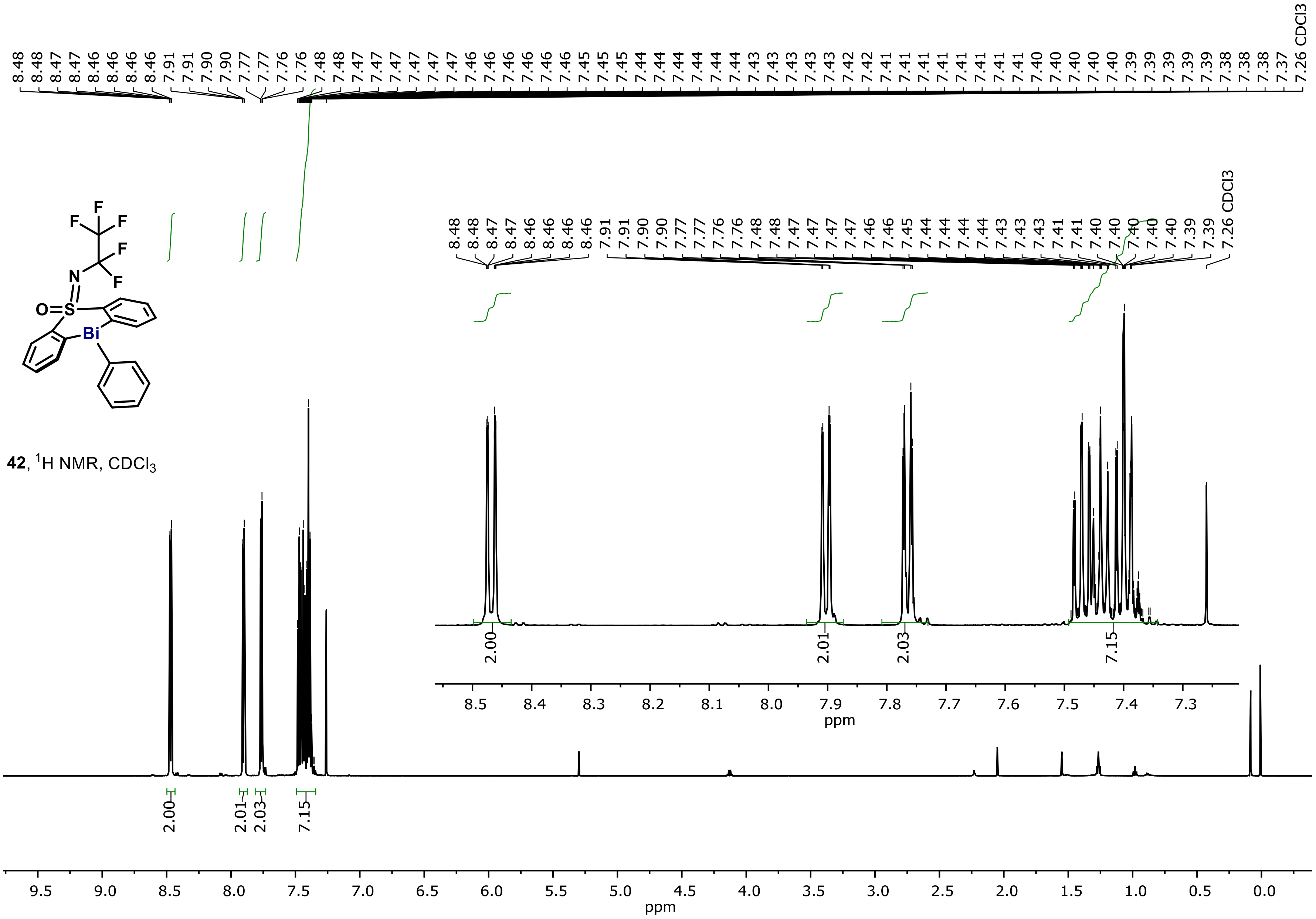

42, ^{1}H NMR, $CDCl_3$

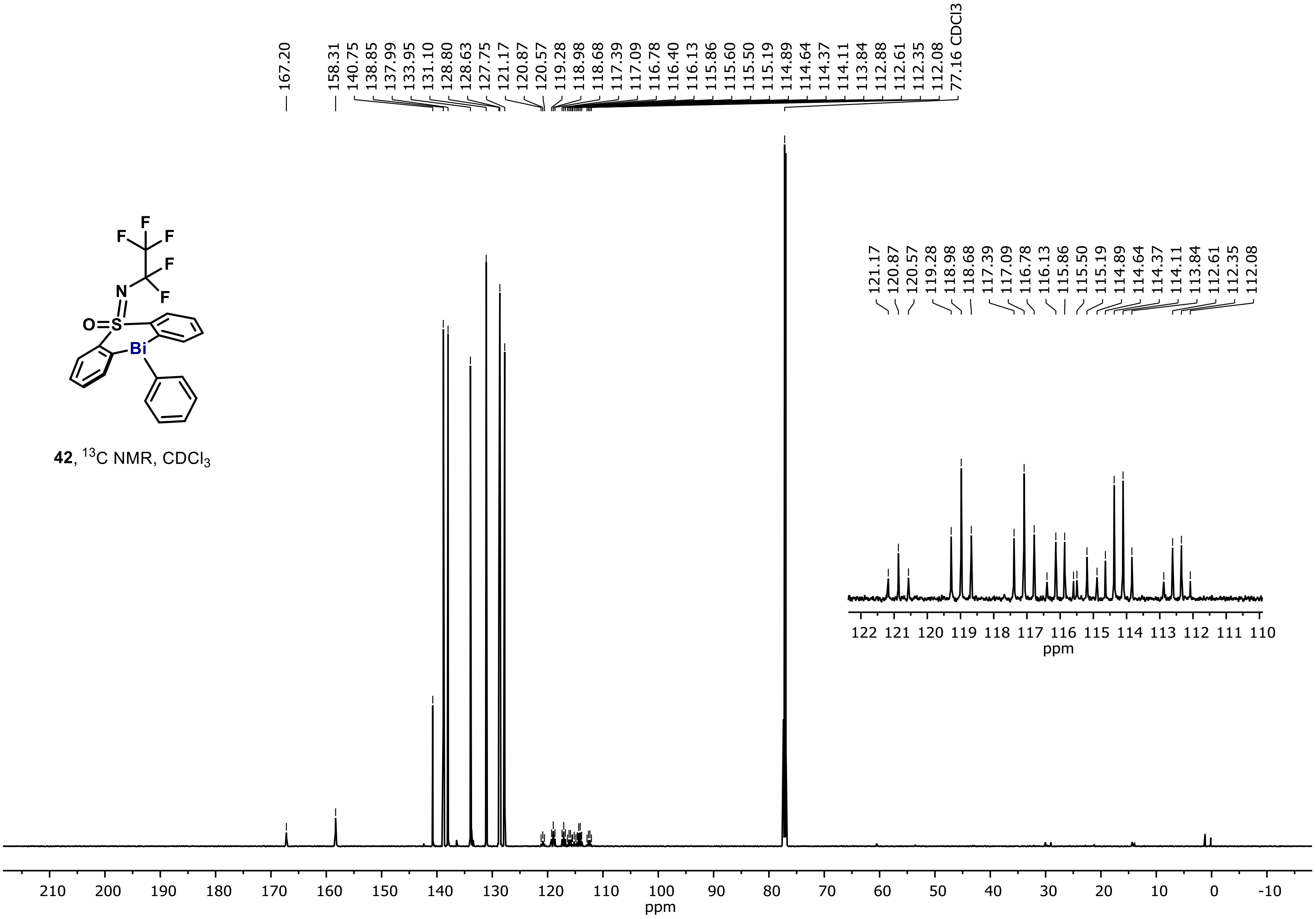

42, ^{13}C NMR, $CDCl_3$

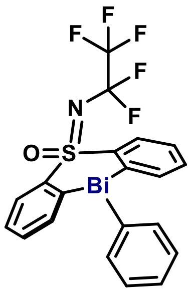

42, ^{19}F NMR, $CDCl_3$

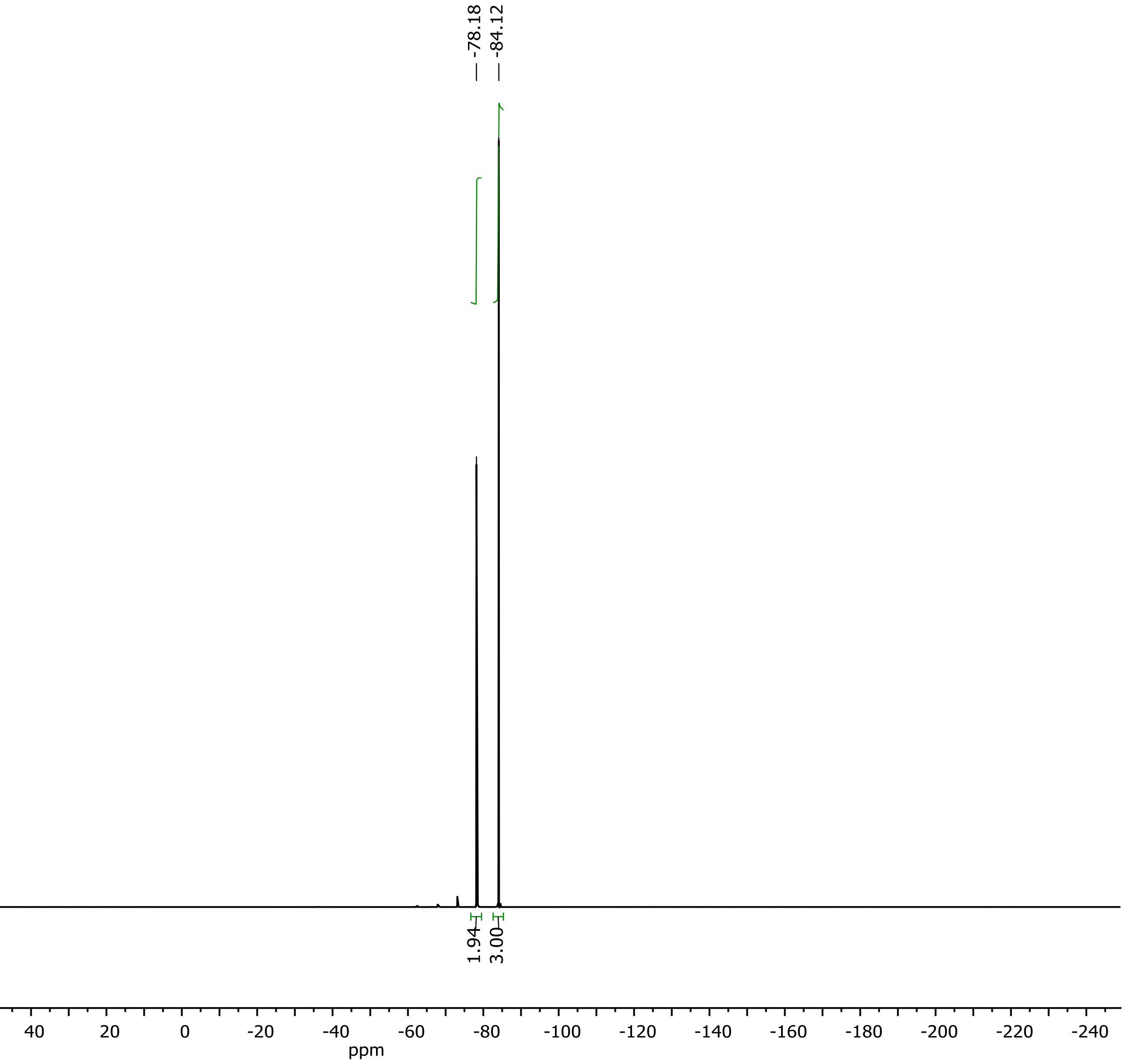

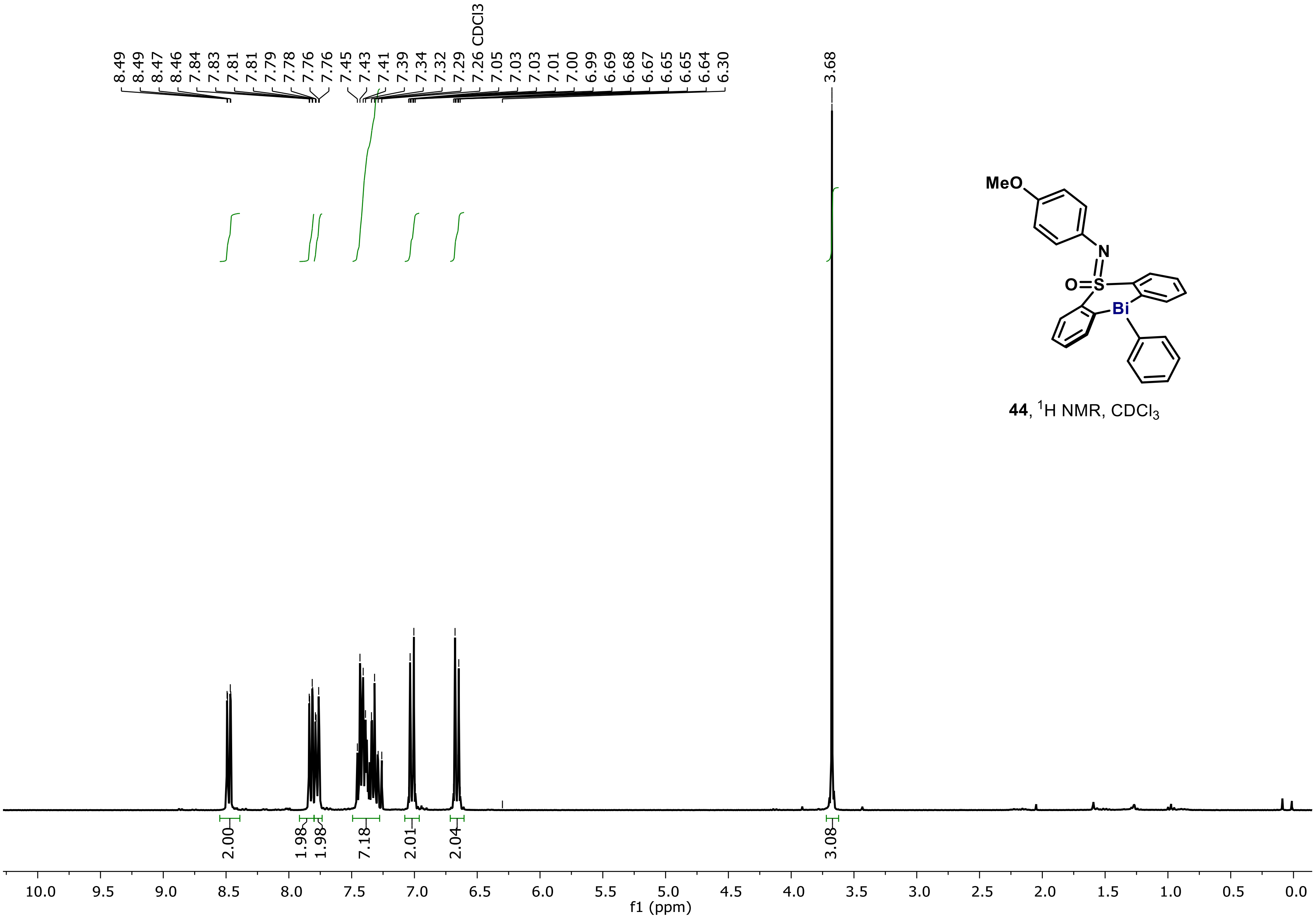

44, ^{1}H NMR, $CDCl_3$

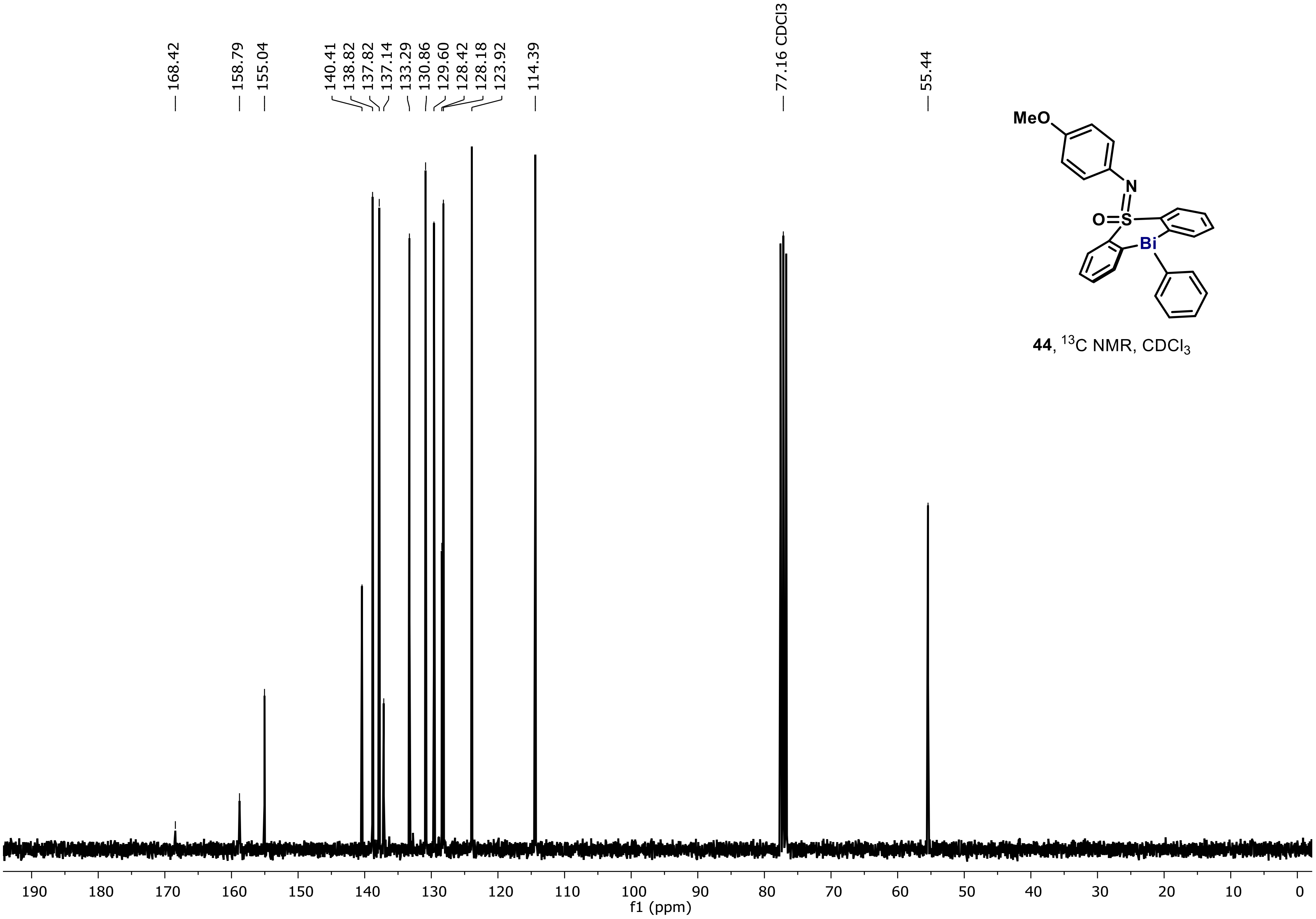

44, ^{13}C NMR, $CDCl_3$

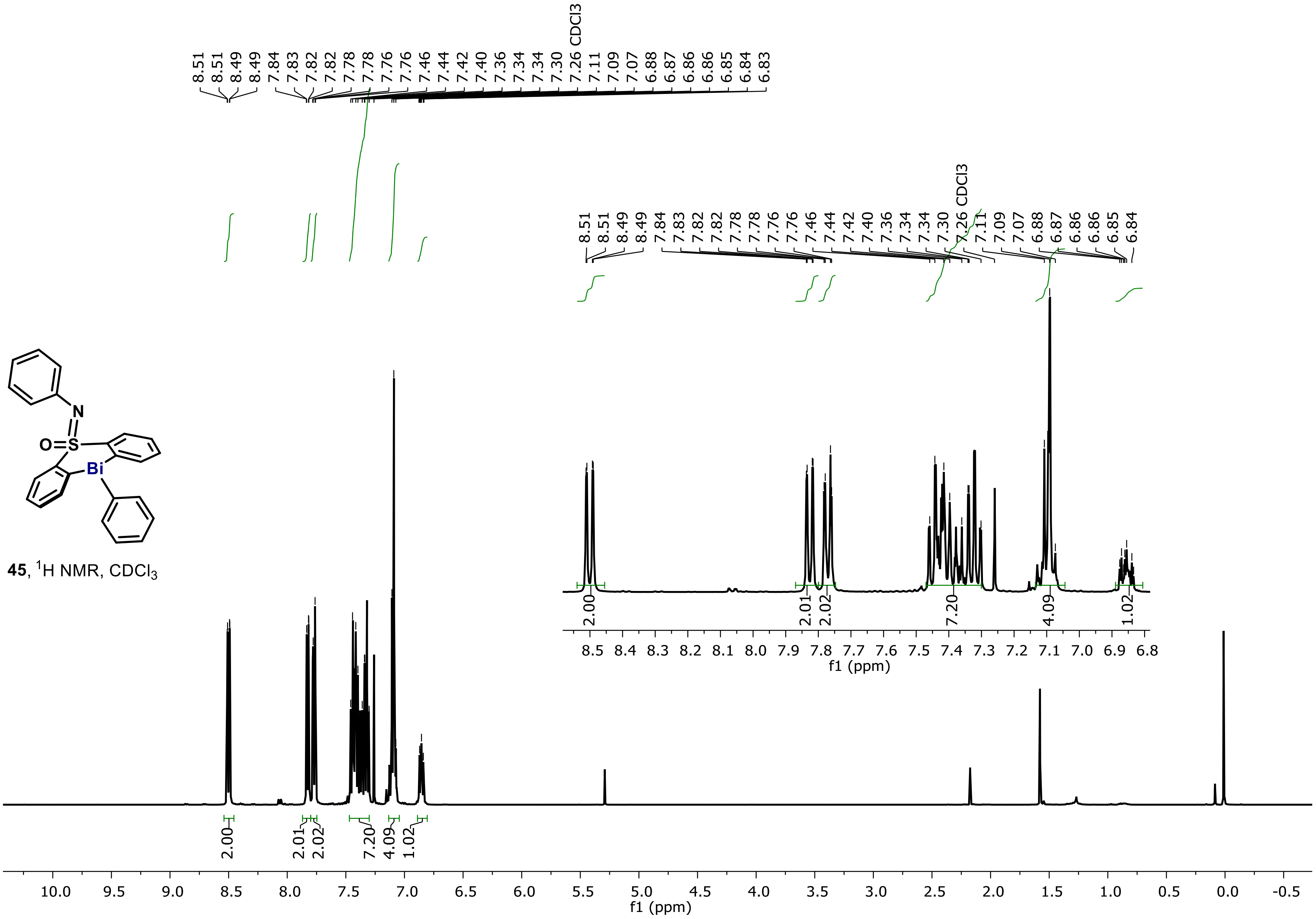

45, ^{1}H NMR, $CDCl_3$

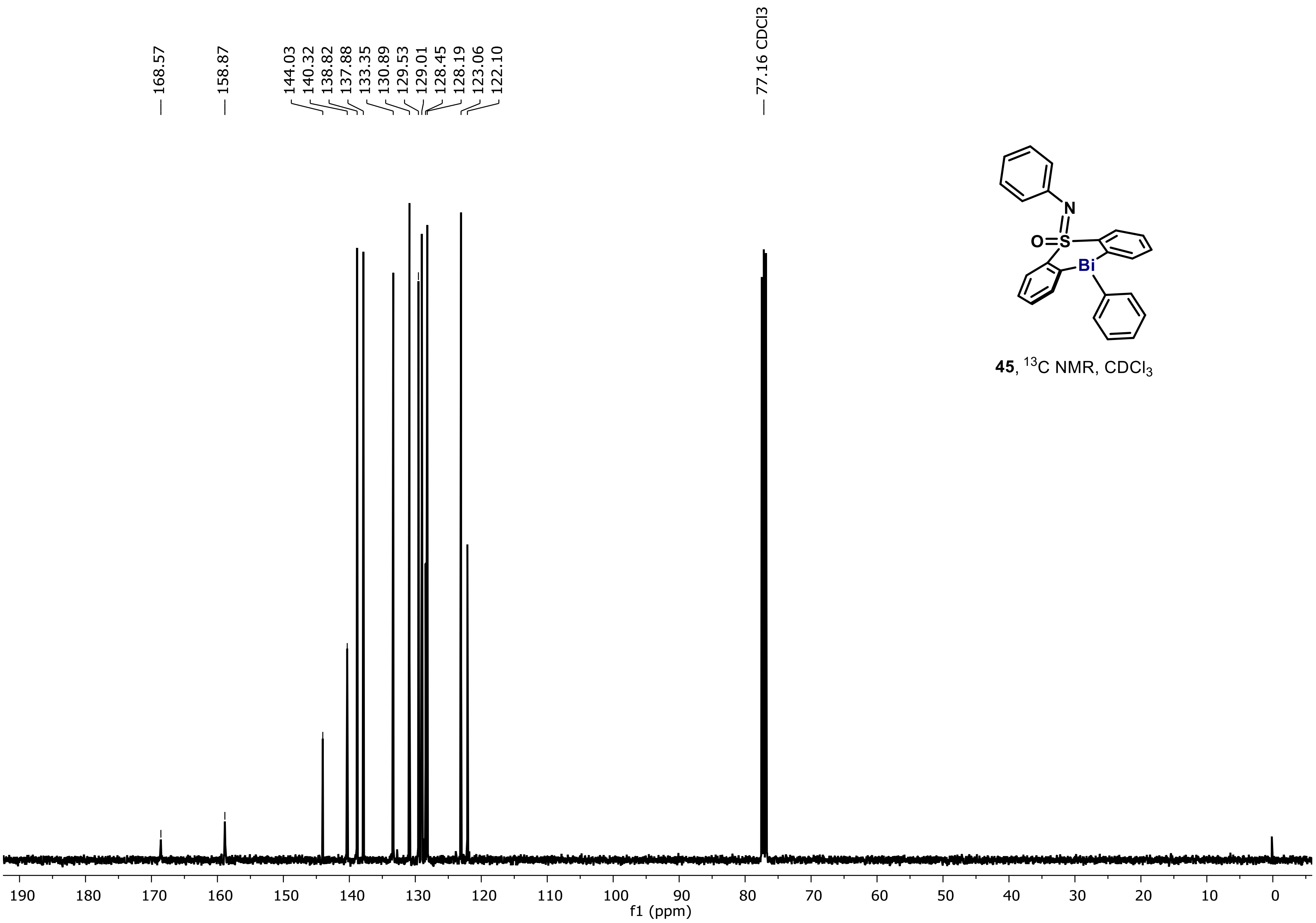

168.57
158.87
144.03
140.32
138.82
137.88
133.35
130.89
129.53
129.01
128.45
128.19
123.06
122.10
77.16 CDCl3
N
O
S
Bi
45, 13C NMR, CDCl3
190
180
170
160
150
140
130
120
110
100
90
80
70
60
50
40
30
20
10
0
f1 (ppm)

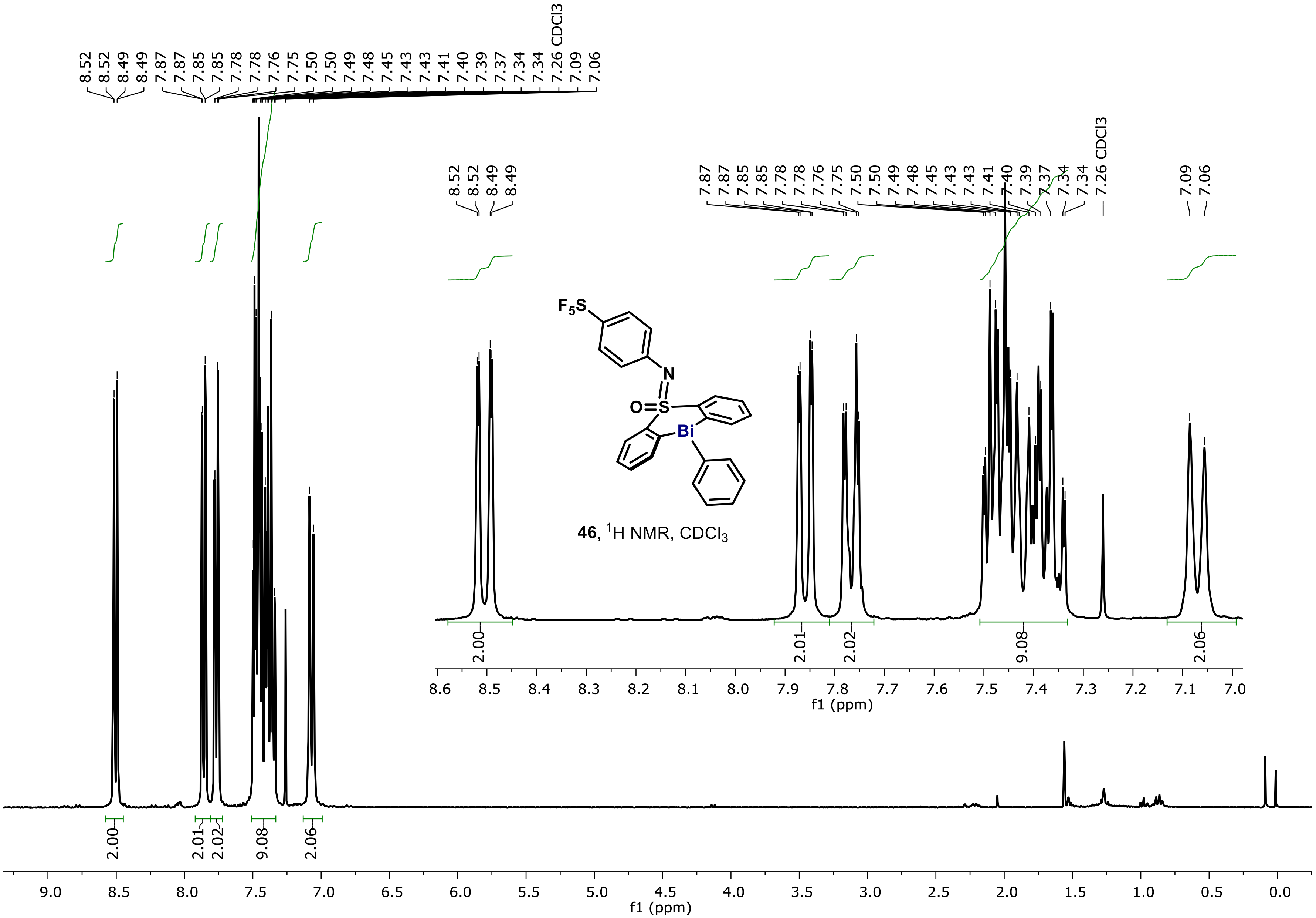
8.52 8.52 8.49 8.49 7.87 7.87 7.85 7.85 7.78 7.78 7.76 7.75 7.50 7.50 7.49 7.48 7.45 7.43 7.43 7.41 7.40 7.39 7.37 7.34 7.34 7.26 CDCl3 7.09 7.06
2.00
2.01
2.02
9.08
2.06
f1 (ppm)
F_5S
N
O
S
Bi
46, ¹H NMR, $CDCl_3$

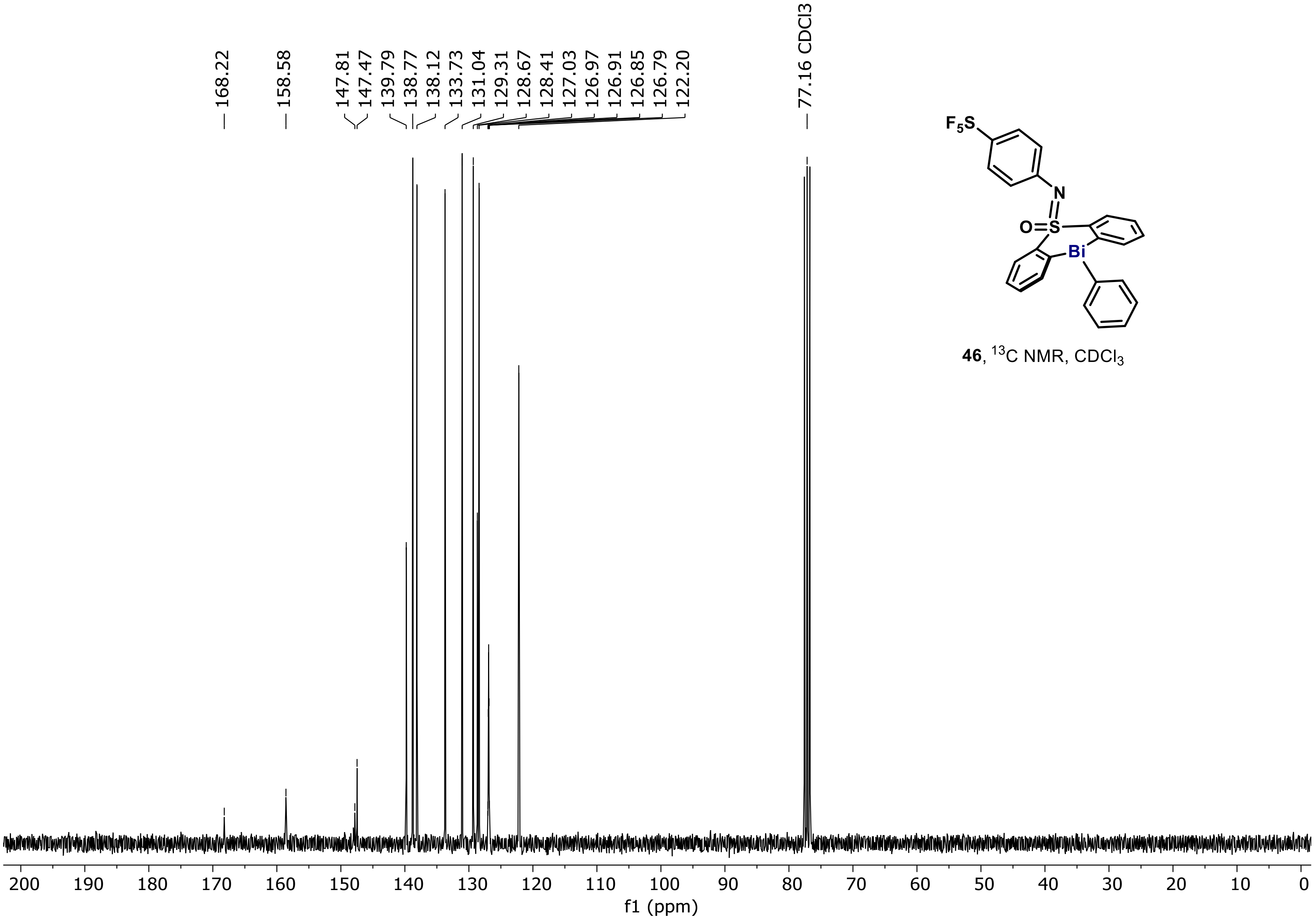
168.22
158.58
147.81
147.47
139.79
138.77
138.12
133.73
131.04
129.31
128.67
128.41
127.03
126.97
126.91
126.85
126.79
122.20
77.16 CDCl3
F5S
N
O=S
Bi
46, 13C NMR, CDCl3
200 190 180 170 160 150 140 130 120 110 100 90 80 70 60 50 40 30 20 10 0
f1 (ppm)

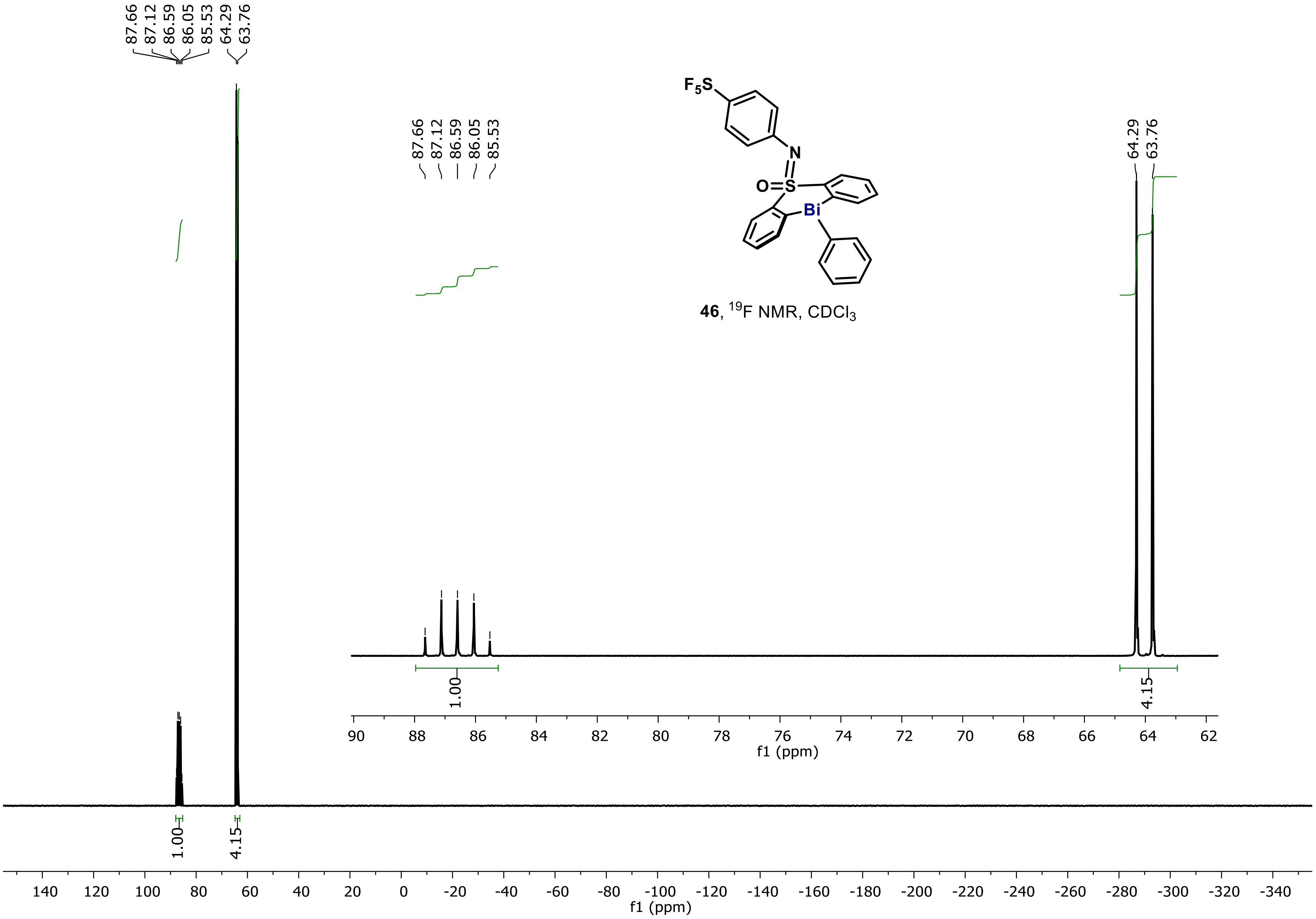

87.66
87.12
86.59
86.05
85.53
64.29
63.76
F5S
N
O=S
Bi
46, 19F NMR, CDCl3
1.00
4.15
f1 (ppm)

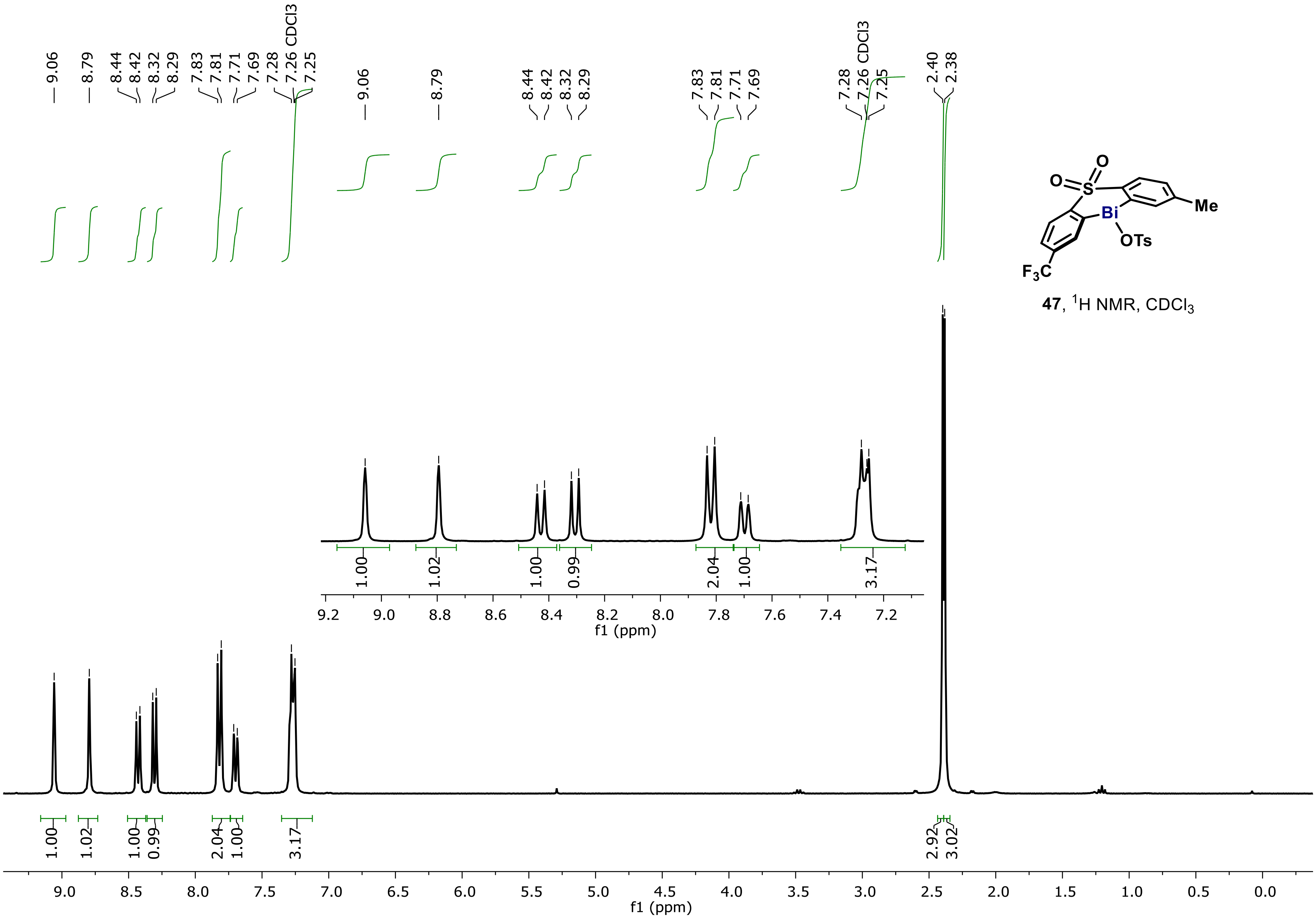

47, ^{1}H NMR, $CDCl_3$

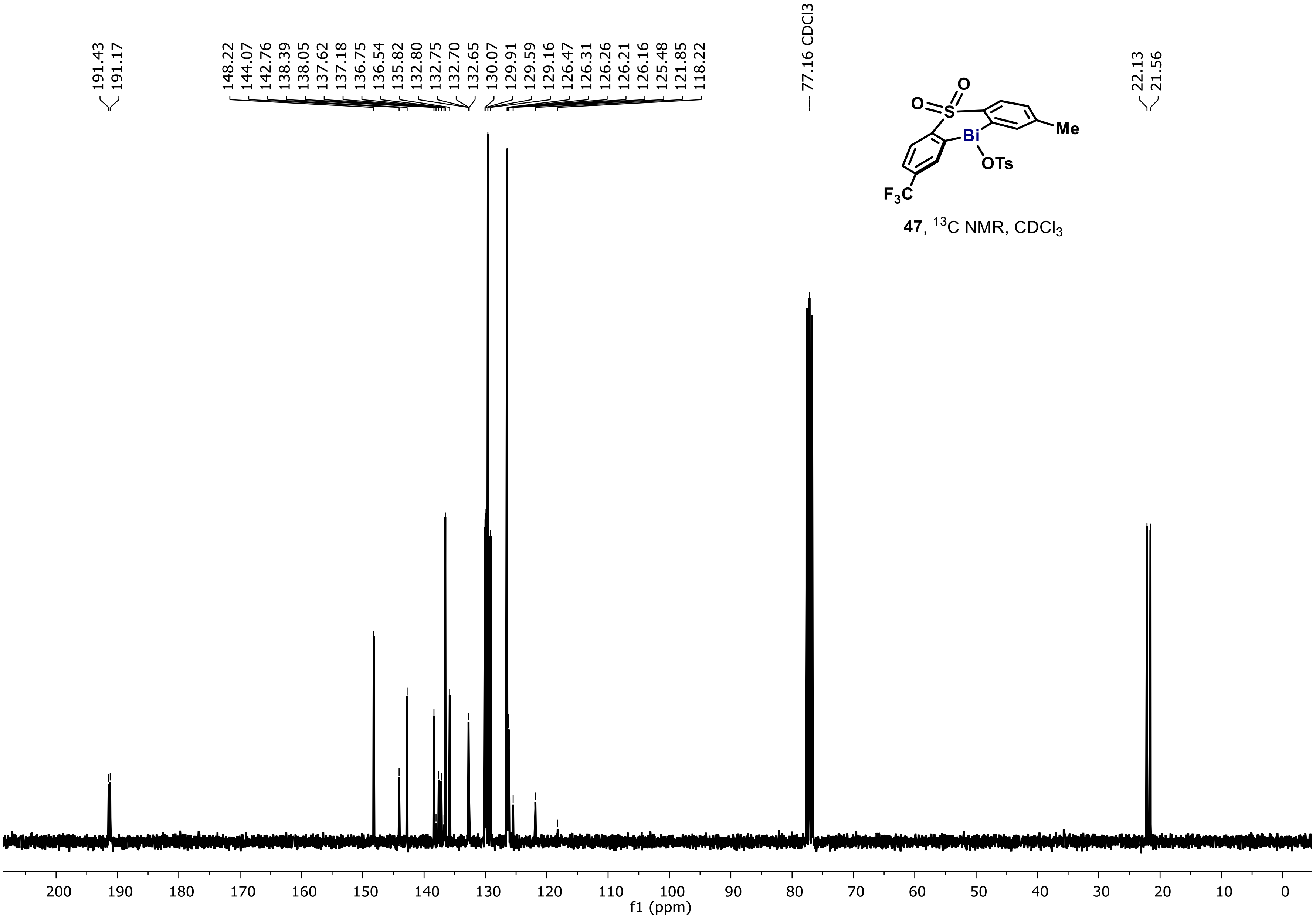

47, ^{13}C NMR, $CDCl_3$

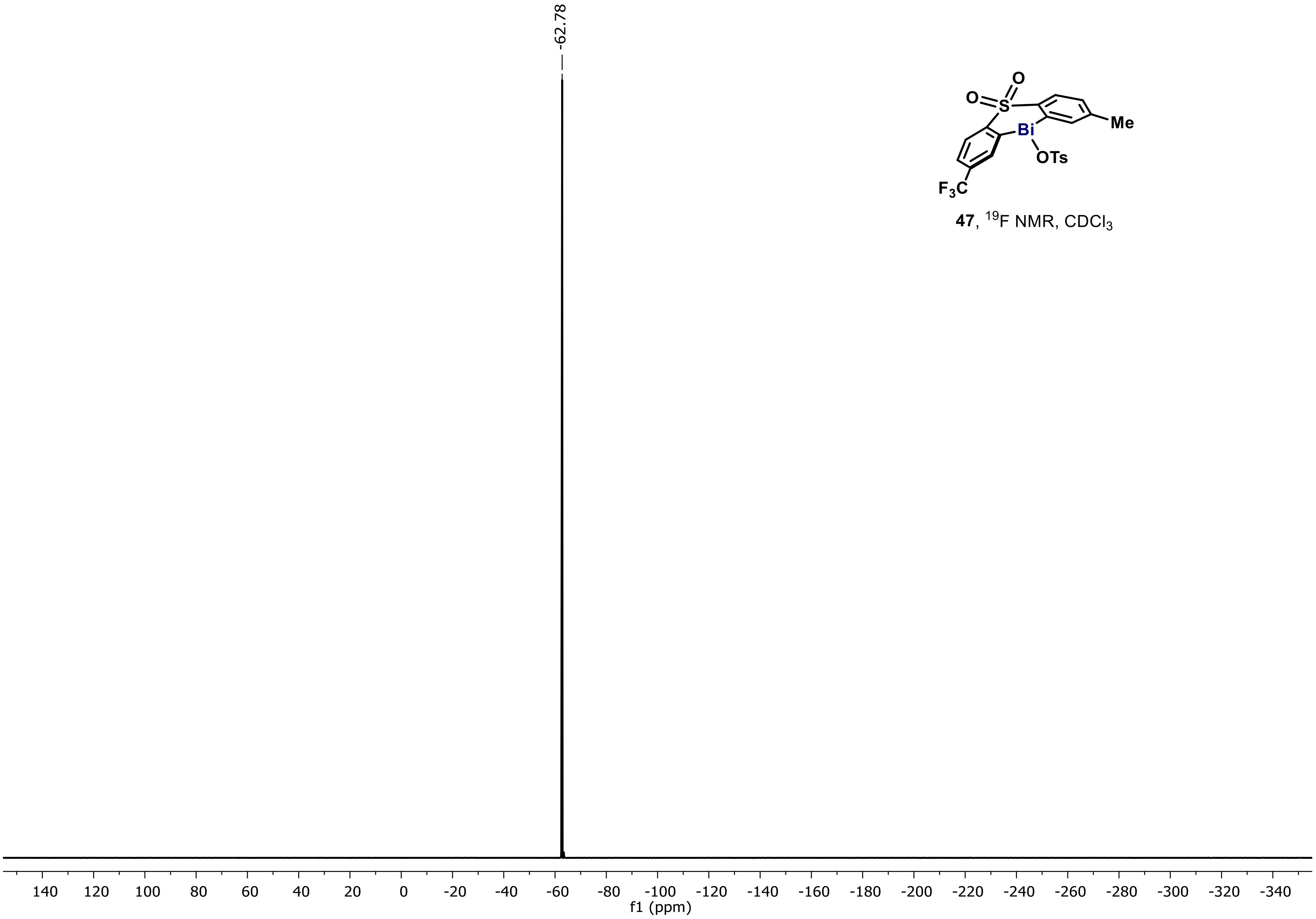
-62.78
O
O
S
Bi
Me
OTs
F3C
47, 19F NMR, CDCl3
140 120 100 80 60 40 20 0 -20 -40 -60 -80 -100 -120 -140 -160 -180 -200 -220 -240 -260 -280 -300 -320 -340
f1 (ppm)

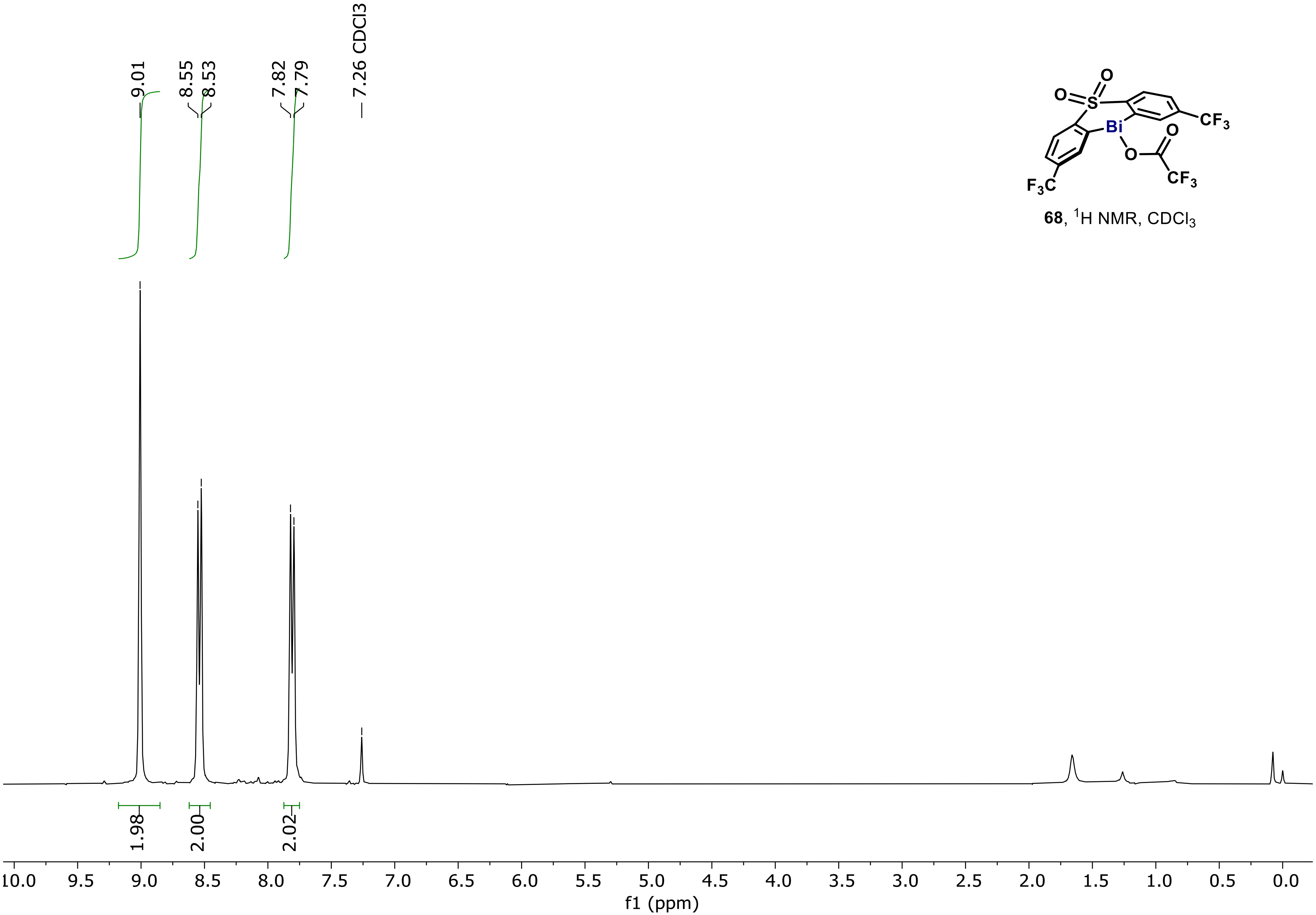

68, ^{1}H NMR, $CDCl_3$

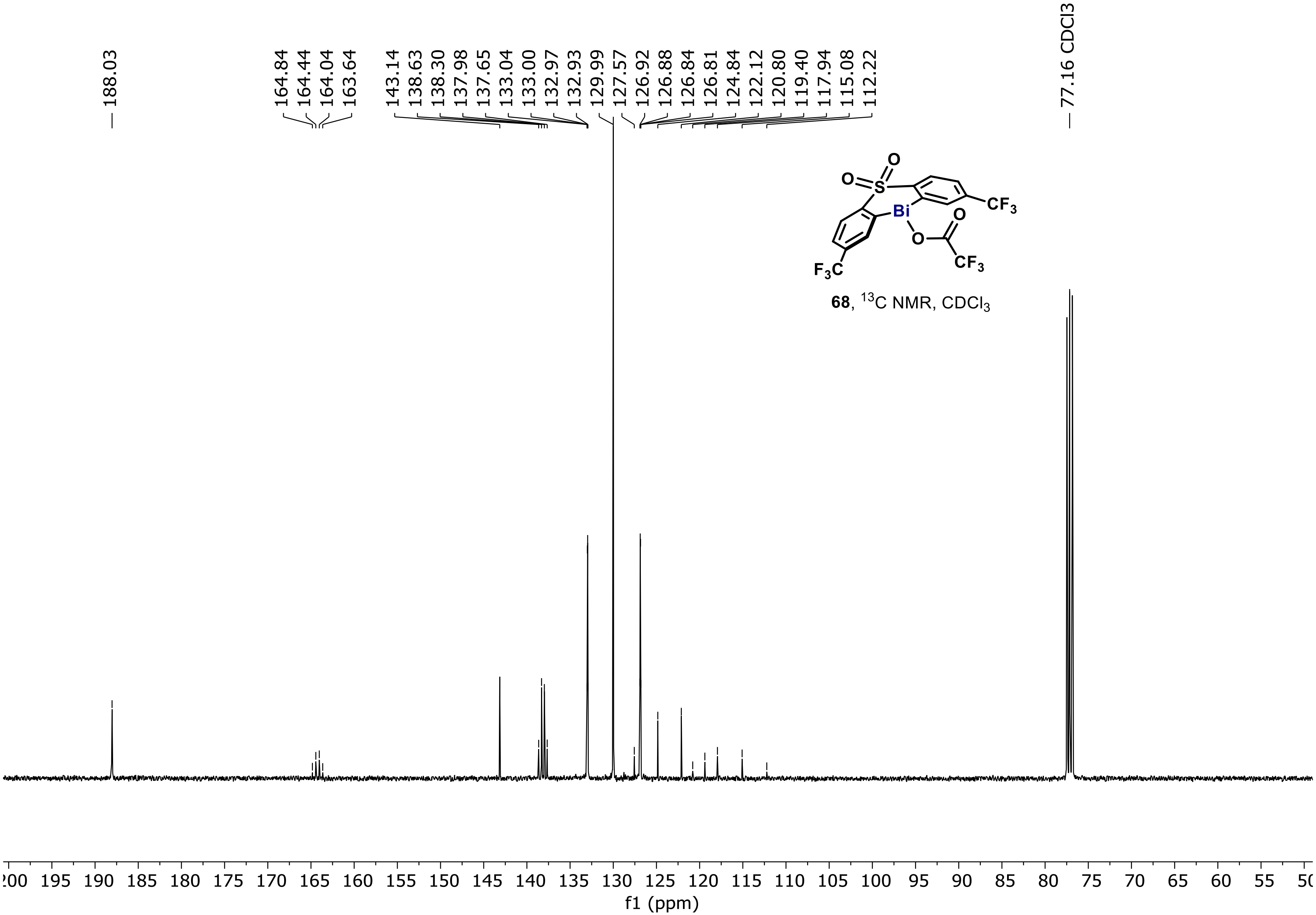

68, ^{13}C NMR, $CDCl_3$

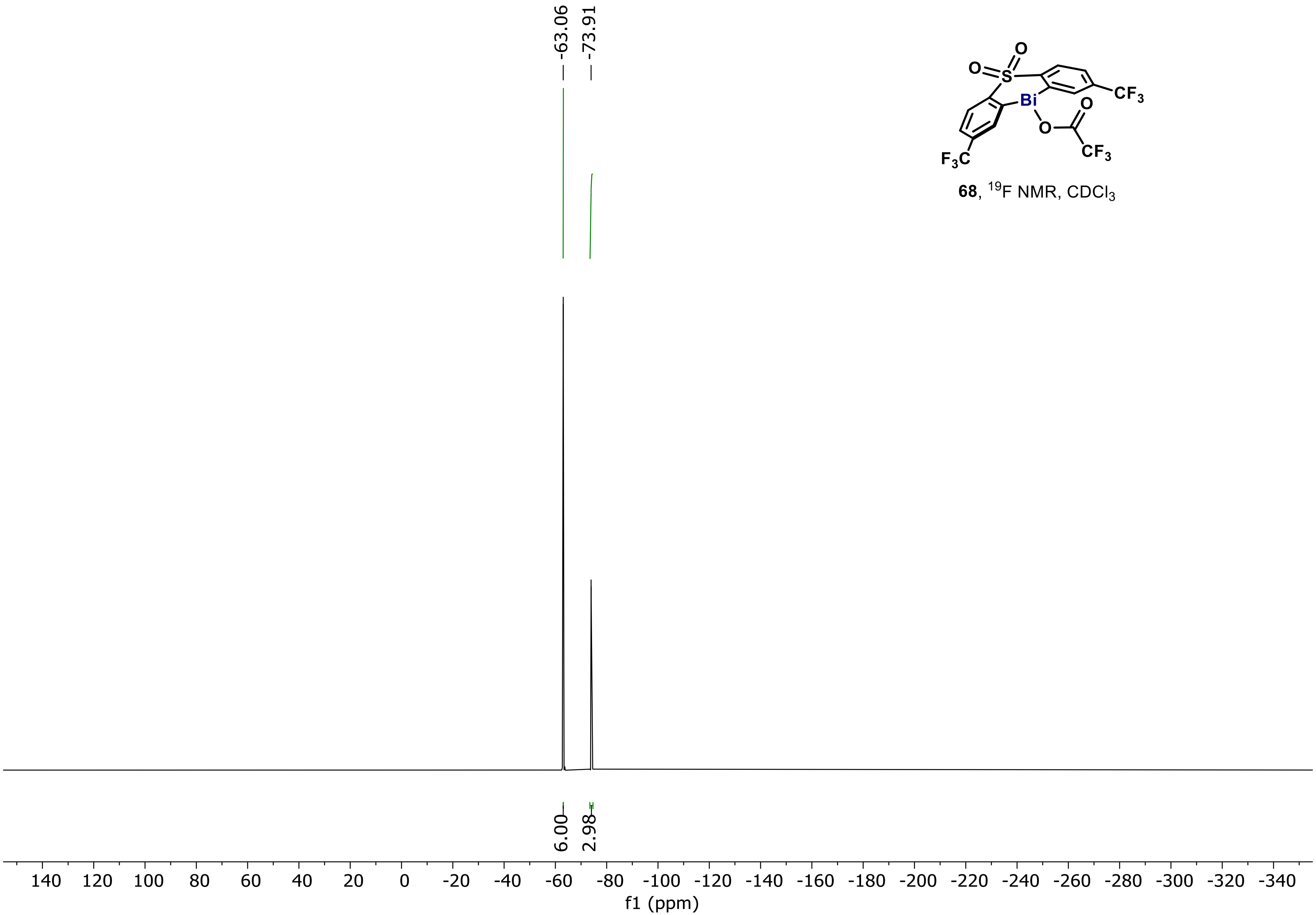

68, ^{19}F NMR, $CDCl_3$

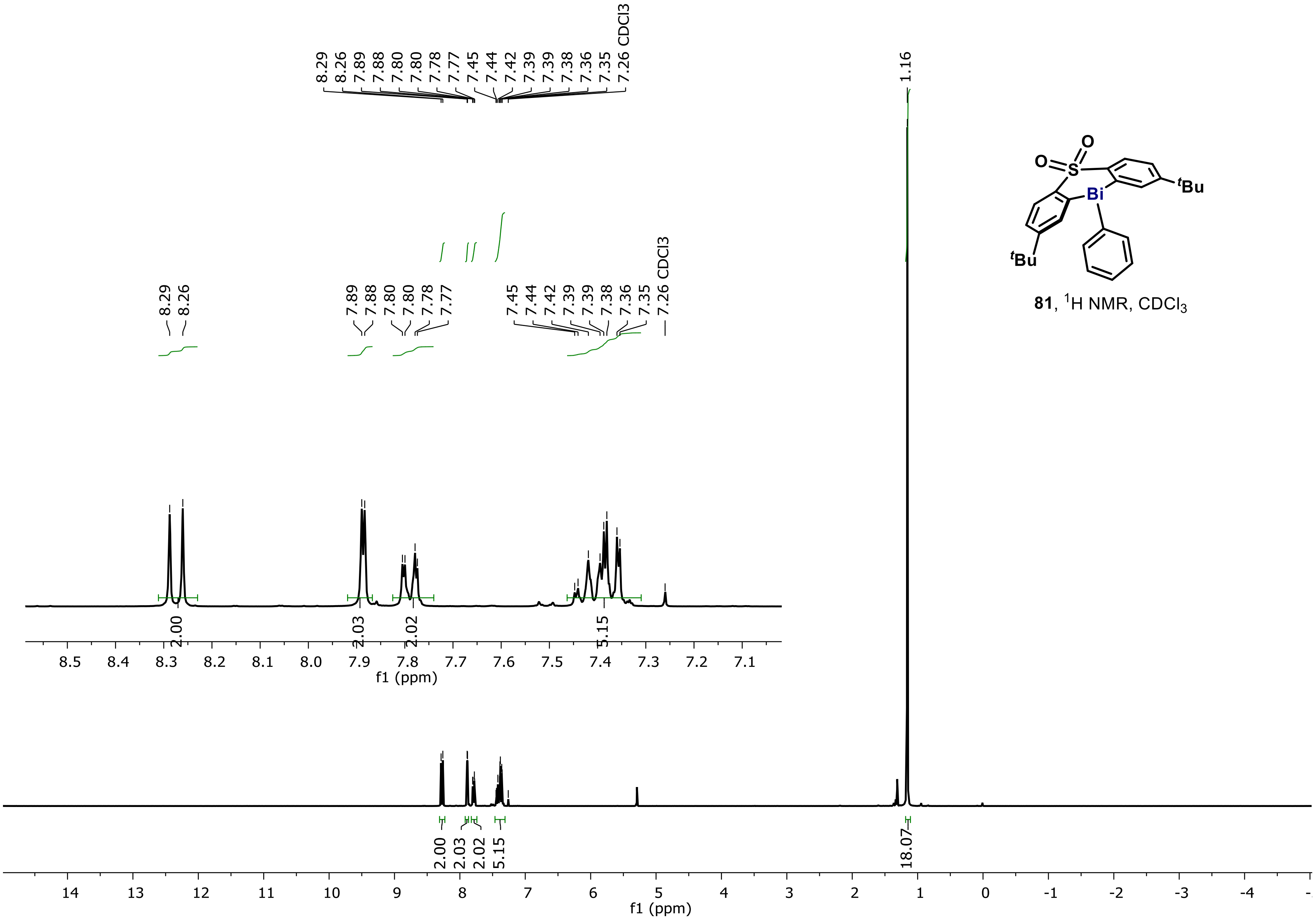

81, ^{1}H NMR, $CDCl_3$

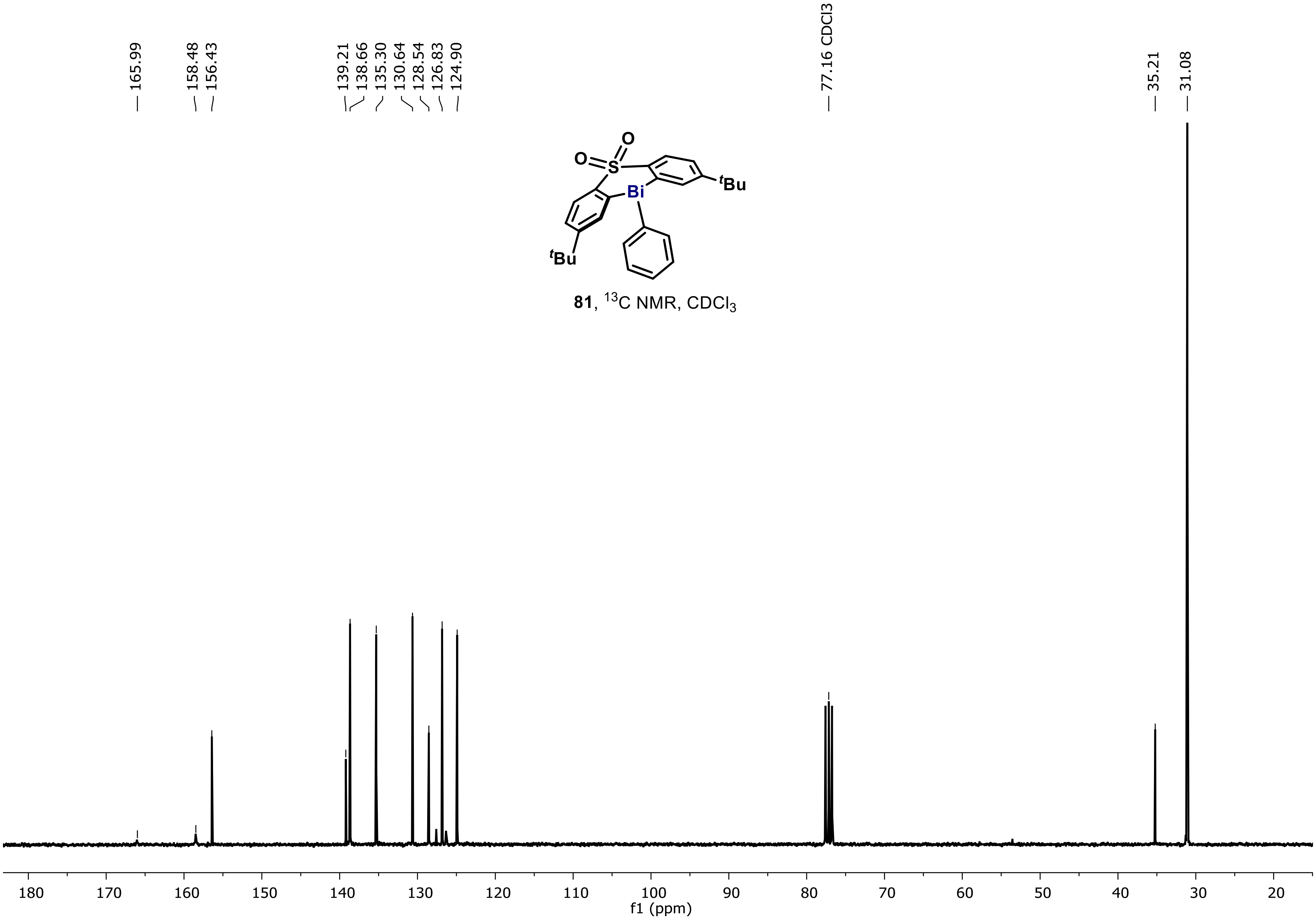
165.99
158.48
156.43
139.21
138.66
135.30
130.64
128.54
126.83
124.90
77.16 CDCl3
35.21
31.08
O
O
S
Bi
tBu
tBu
81, 13C NMR, CDCl3
180
170
160
150
140
130
120
110
100
90
80
70
60
50
40
30
20
f1 (ppm)

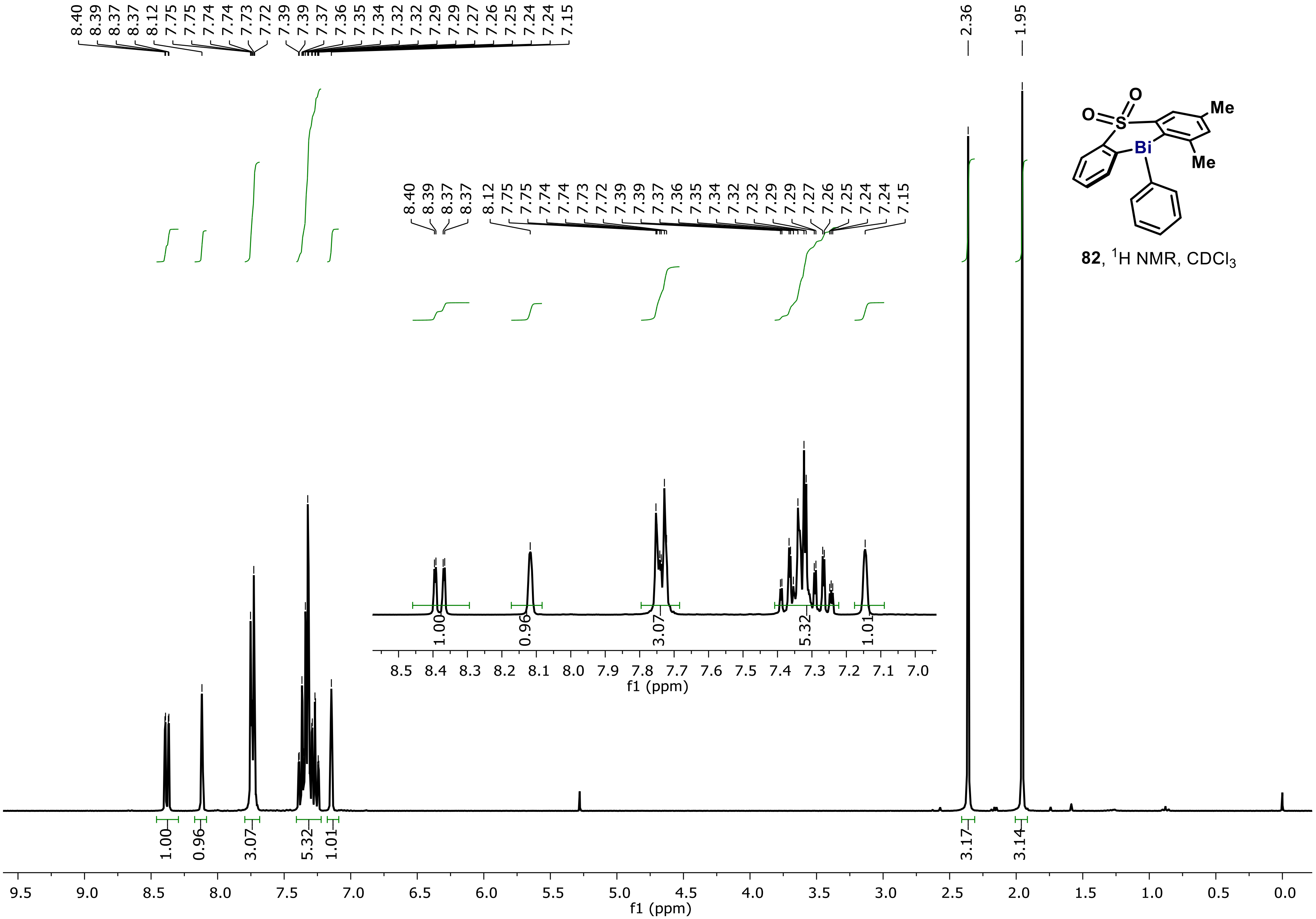

82, ^{1}H NMR, $CDCl_3$

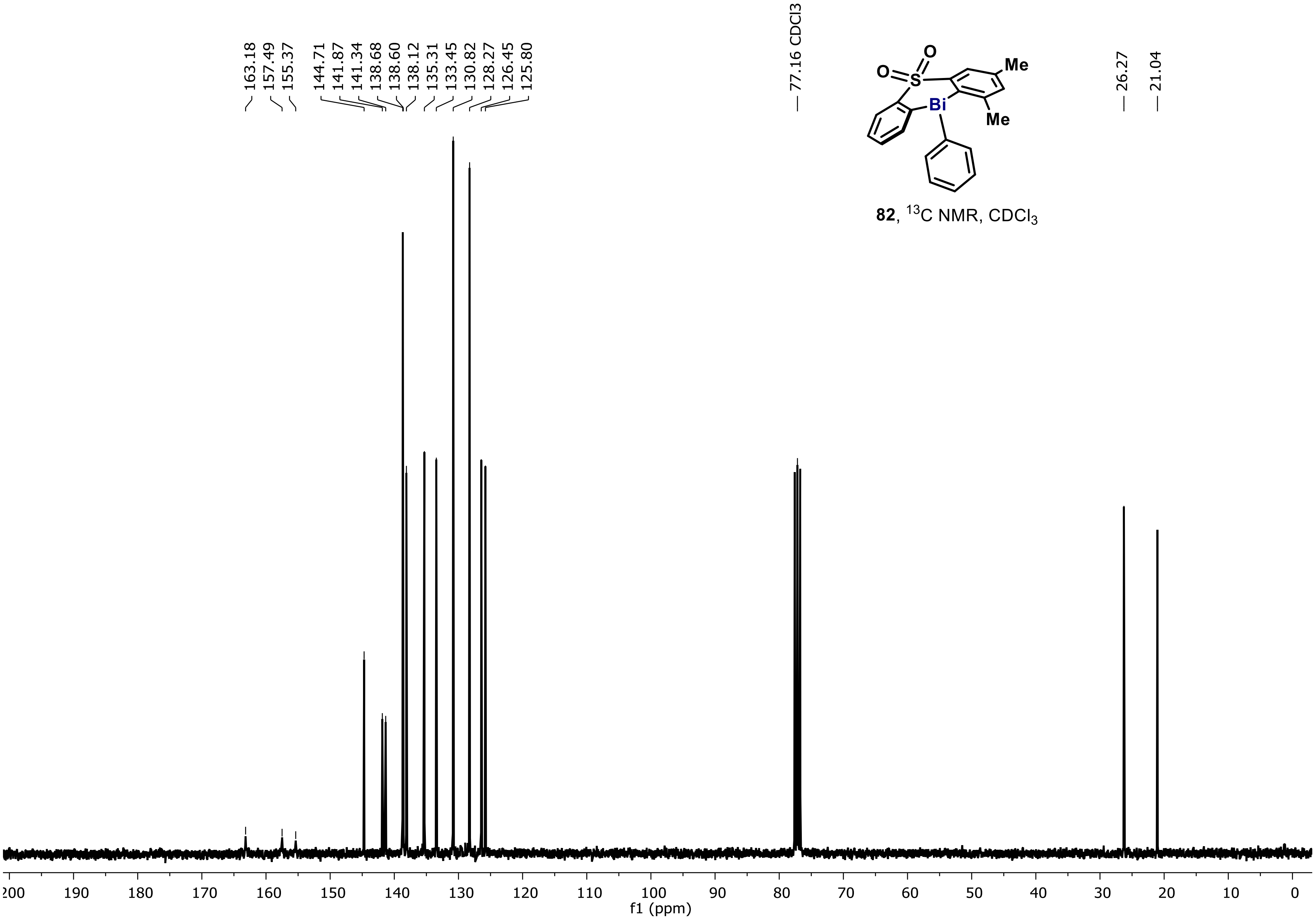
163.18
157.49
155.37
144.71
141.87
141.34
138.68
138.60
138.12
135.31
133.45
130.82
128.27
126.45
125.80
77.16 CDCl3
26.27
21.04
O
O
S
Me
Bi
Me
82, ^{13}C NMR, $CDCl_3$
200 190 180 170 160 150 140 130 120 110 100 90 80 70 60 50 40 30 20 10 0
f1 (ppm)

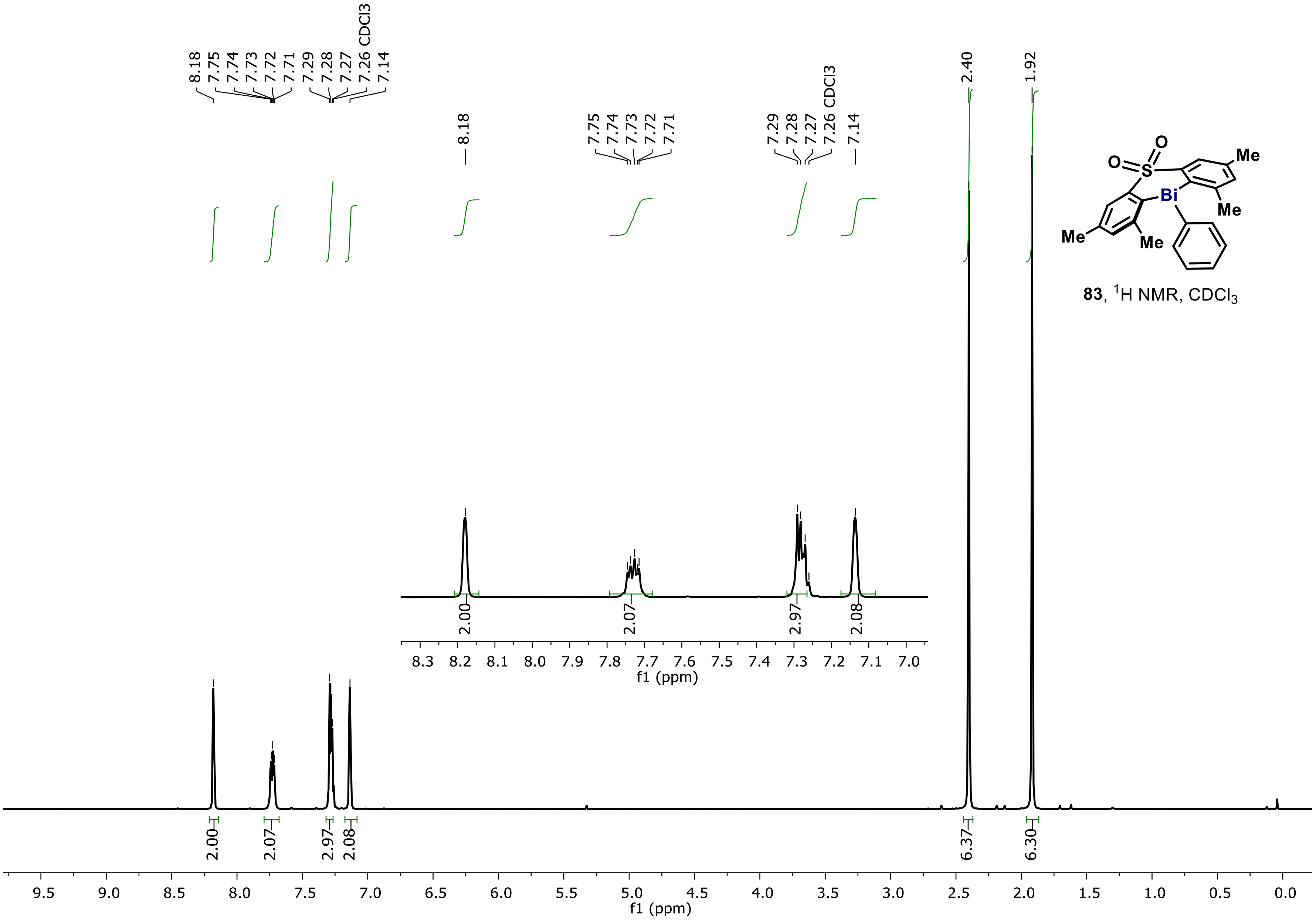

83, ^{1}H NMR, $CDCl_3$

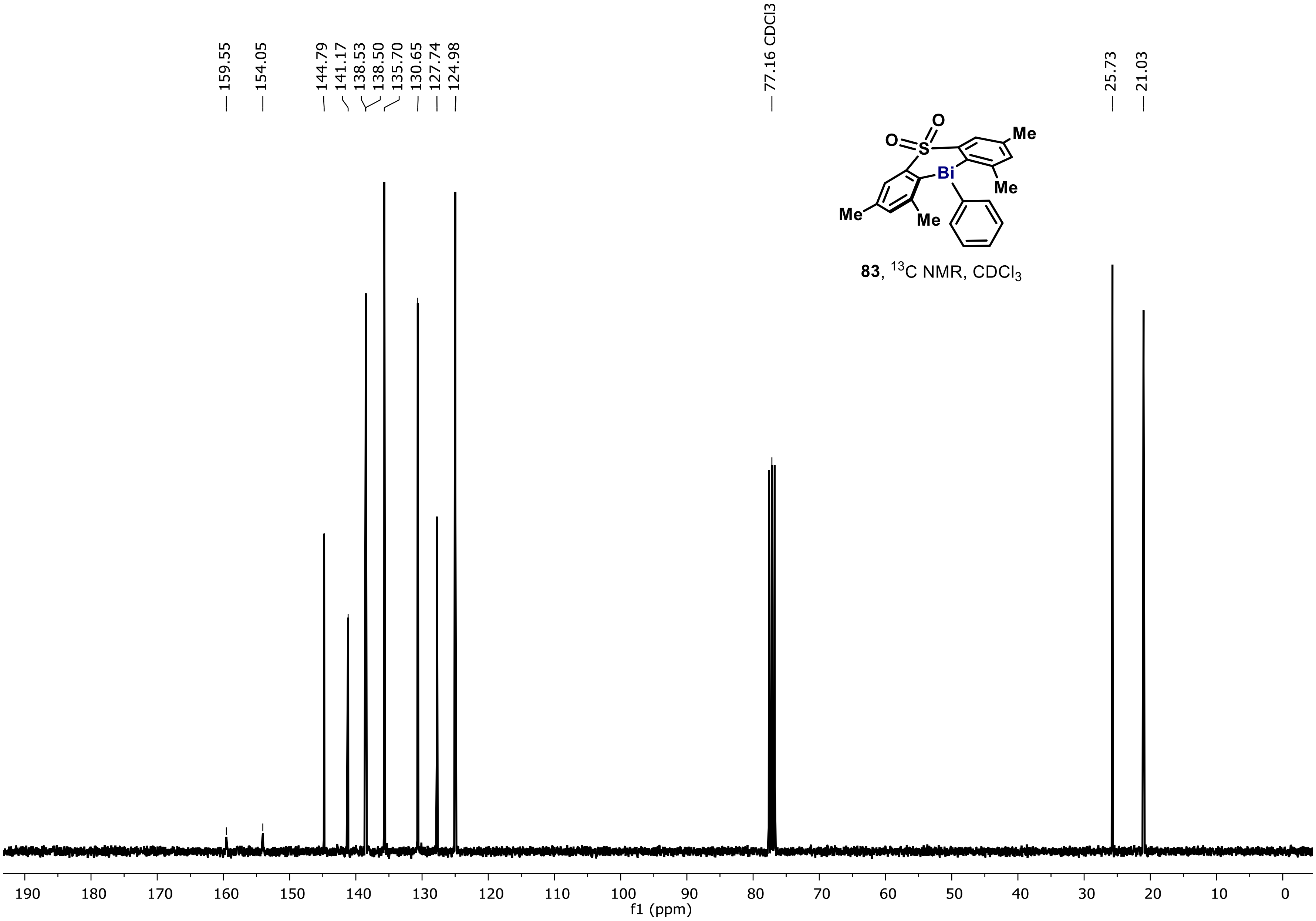

83, ^{13}C NMR, $CDCl_3$

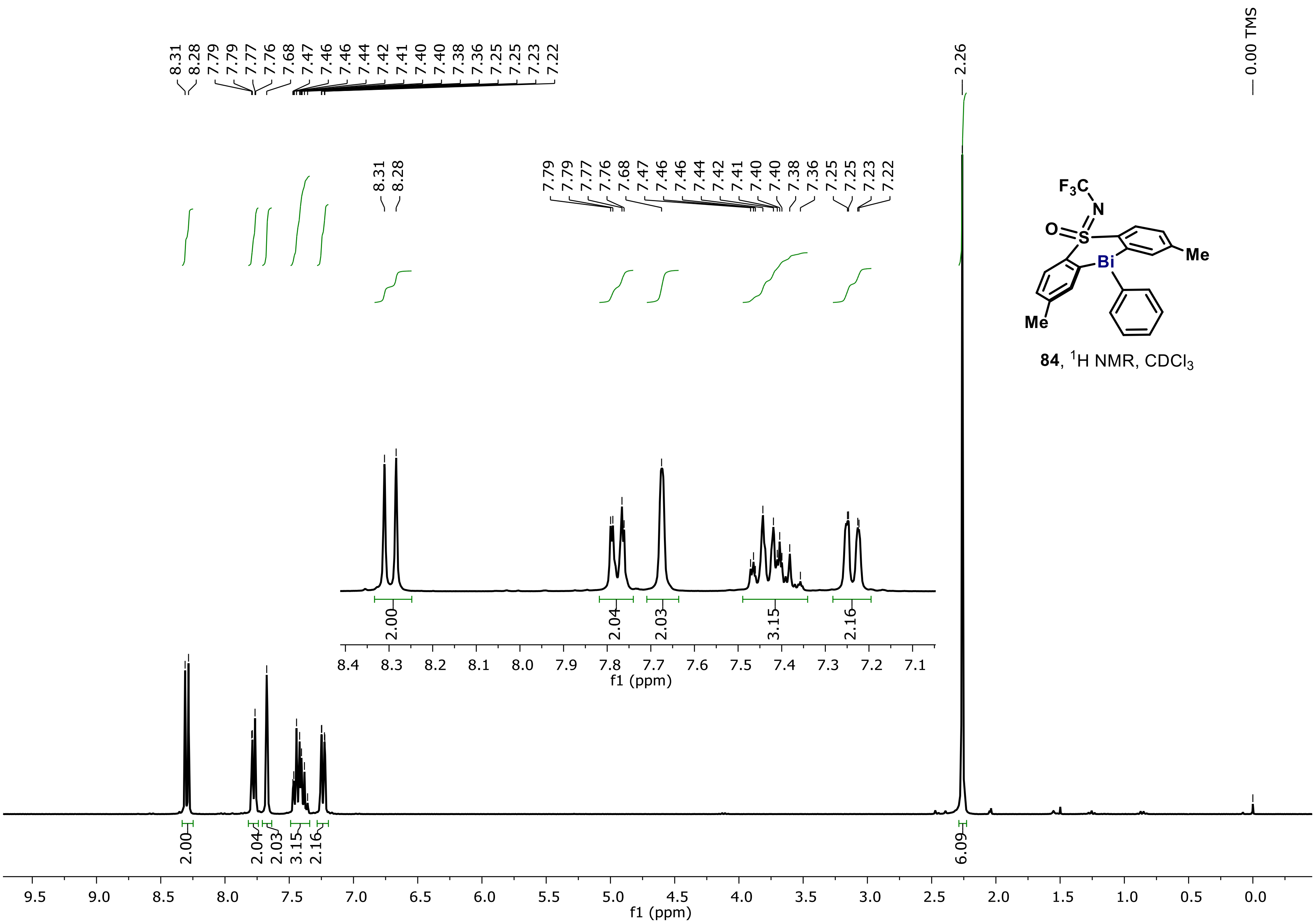

8.31
8.28
7.79
7.79
7.77
7.76
7.68
7.47
7.46
7.46
7.44
7.42
7.41
7.40
7.40
7.38
7.36
7.25
7.25
7.23
7.22
2.26
0.00 TMS
2.00
2.04
2.03
3.15
2.16
6.09
f1 (ppm)
F3C
N
O
S
Bi
Me
Me
84, 1H NMR, CDCl3

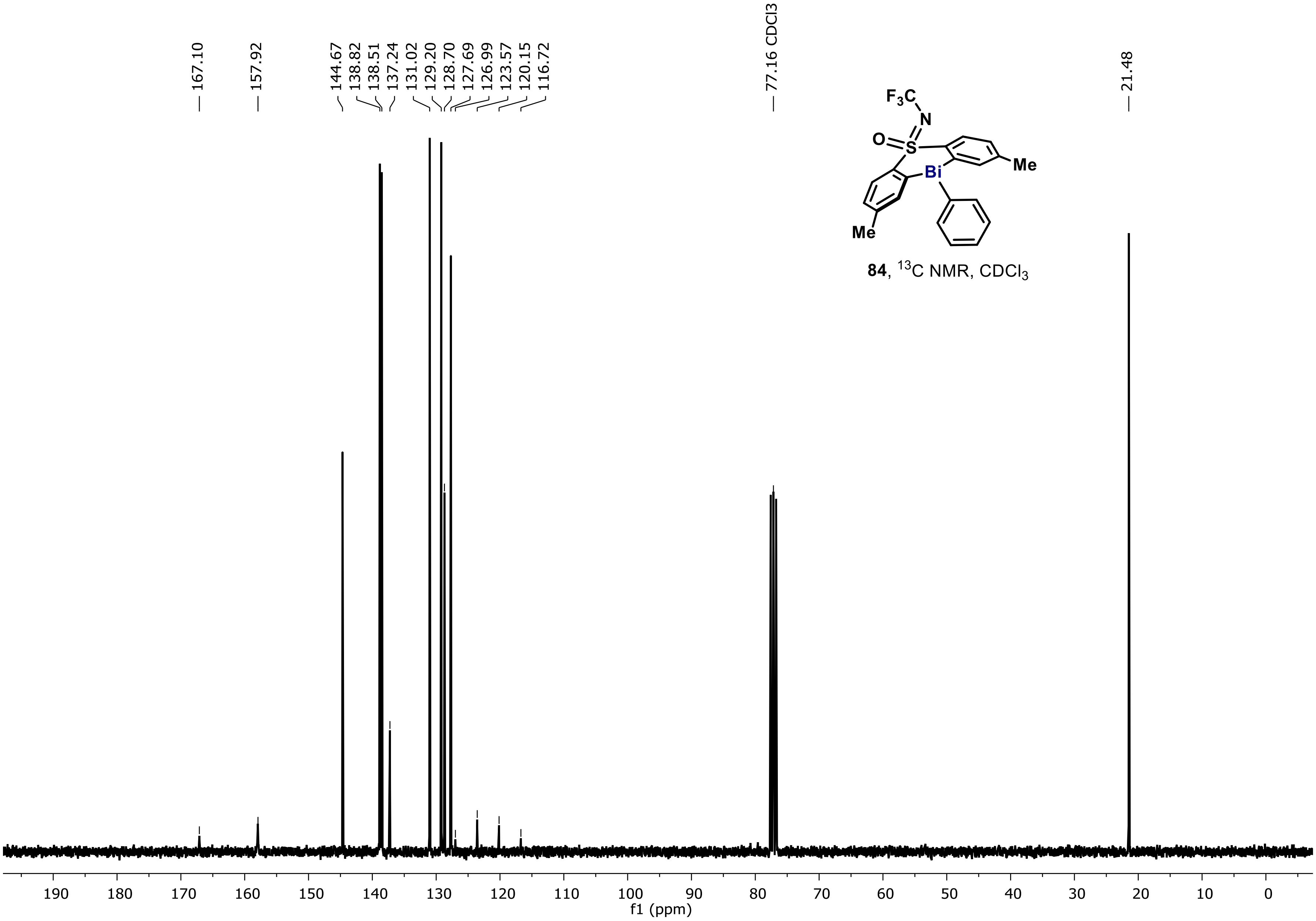

167.10
157.92
144.67
138.82
138.51
137.24
131.02
129.20
128.70
127.69
126.99
123.57
120.15
116.72
77.16 CDCl3
21.48
F3C
N
O
S
Bi
Me
Me
84, 13C NMR, CDCl3
190
180
170
160
150
140
130
120
110
100
90
80
70
60
50
40
30
20
10
0
f1 (ppm)

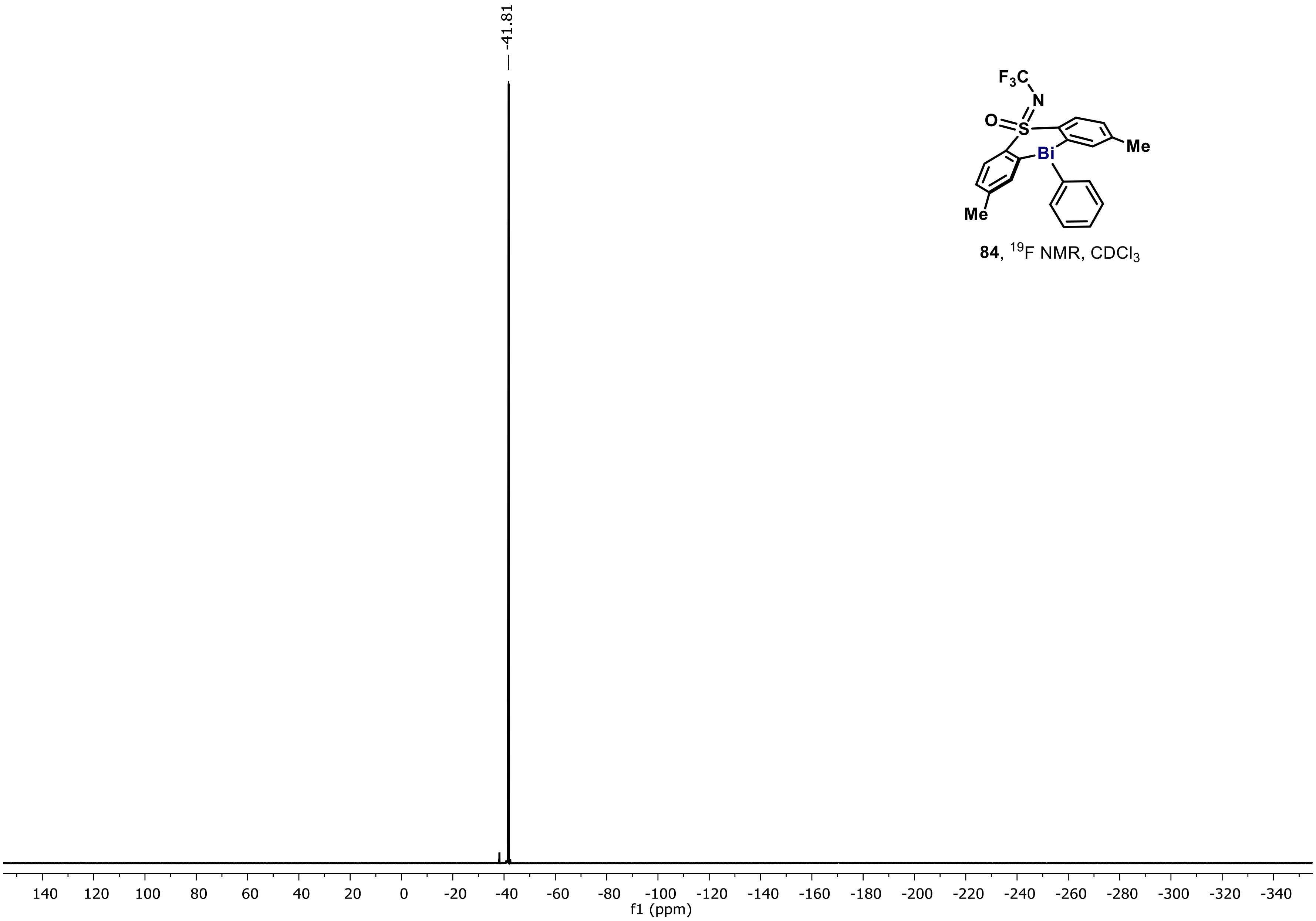

84, ^{19}F NMR, $CDCl_3$

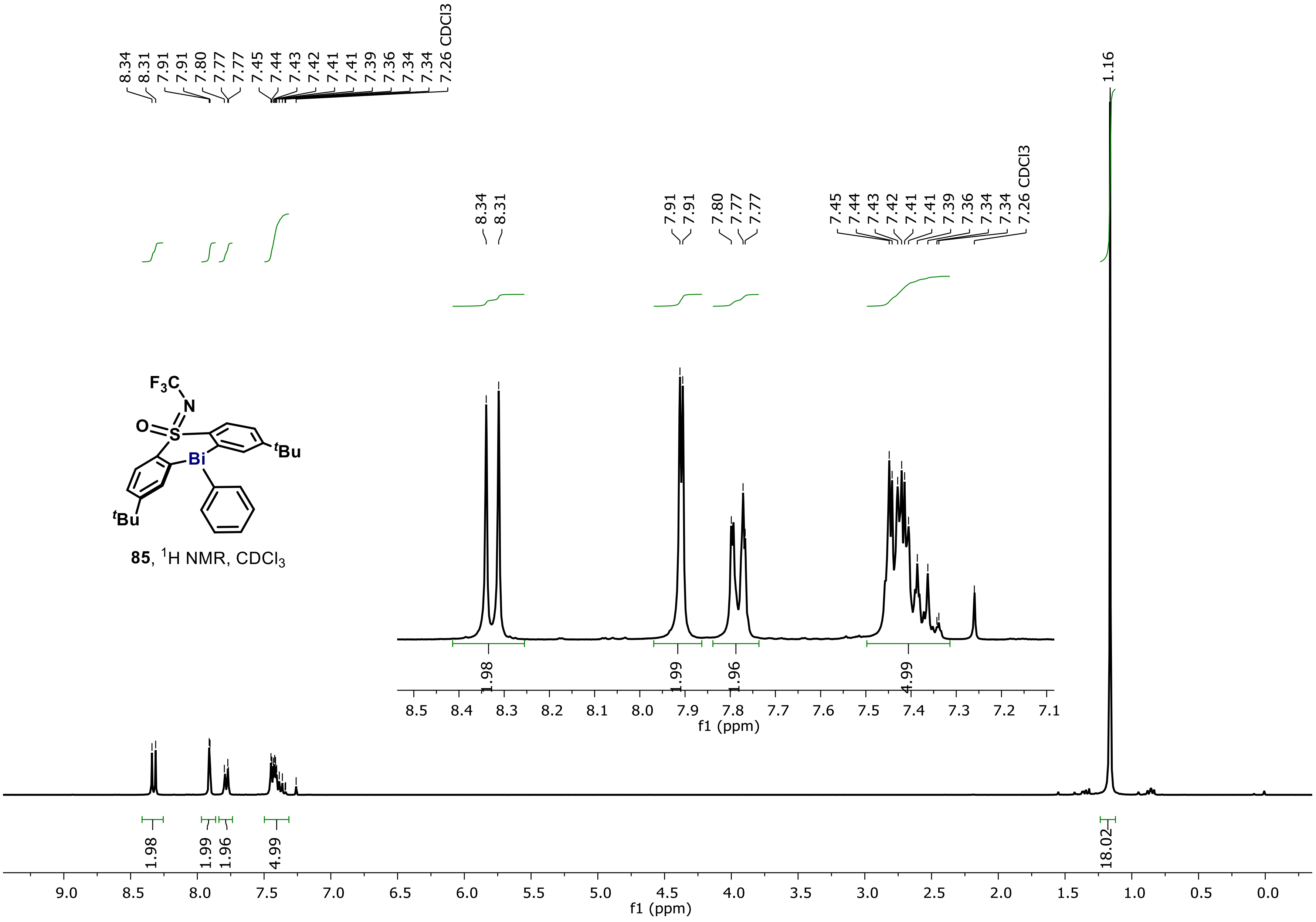

85, ^{1}H NMR, $CDCl_3$

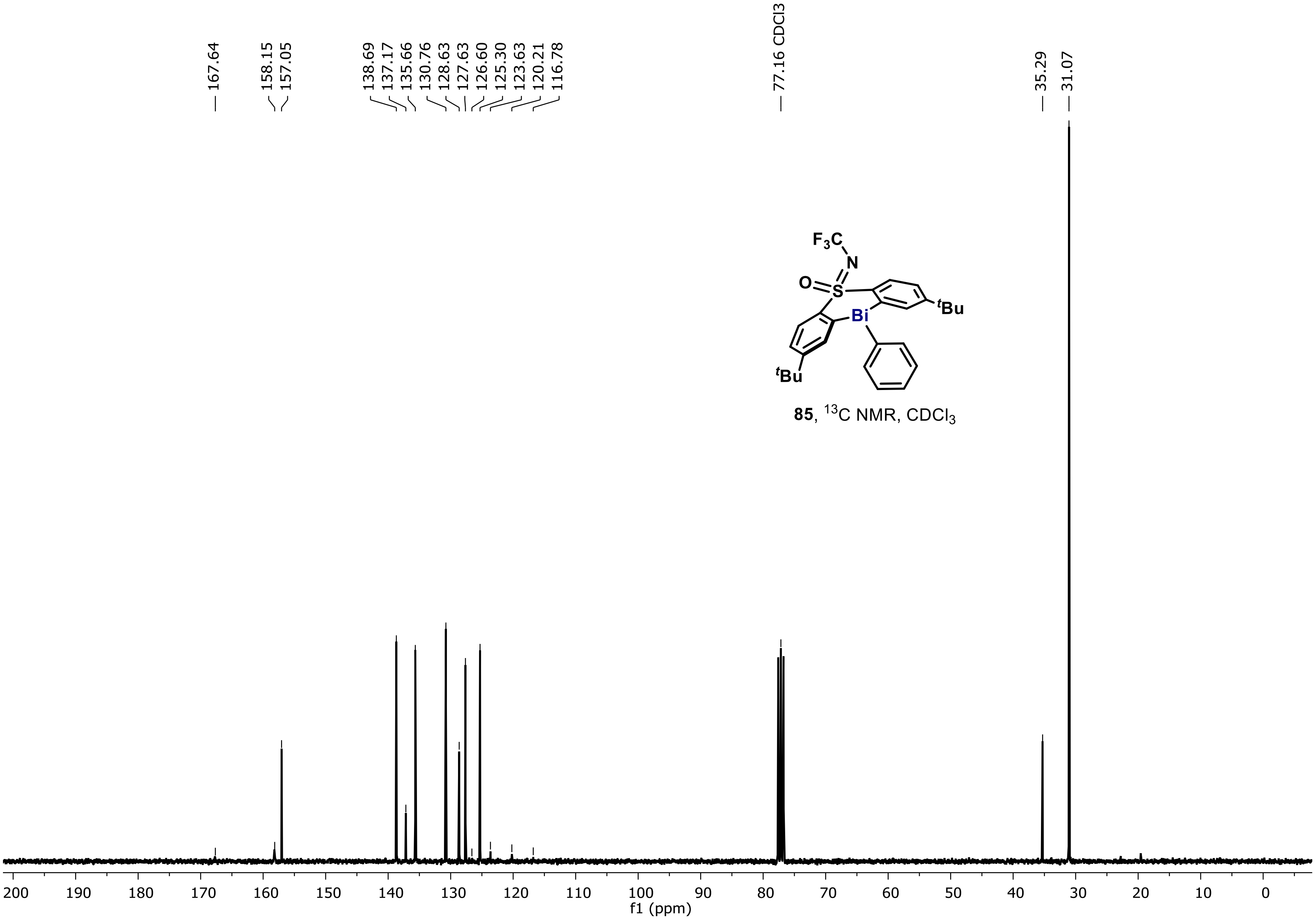
167.64
158.15
157.05
138.69
137.17
135.66
130.76
128.63
127.63
126.60
125.30
123.63
120.21
116.78
77.16 CDCl3
35.29
31.07
F3C
N
O
S
Bi
tBu
tBu
85, 13C NMR, CDCl3
200
190
180
170
160
150
140
130
120
110
100
90
80
70
60
50
40
30
20
10
0
f1 (ppm)

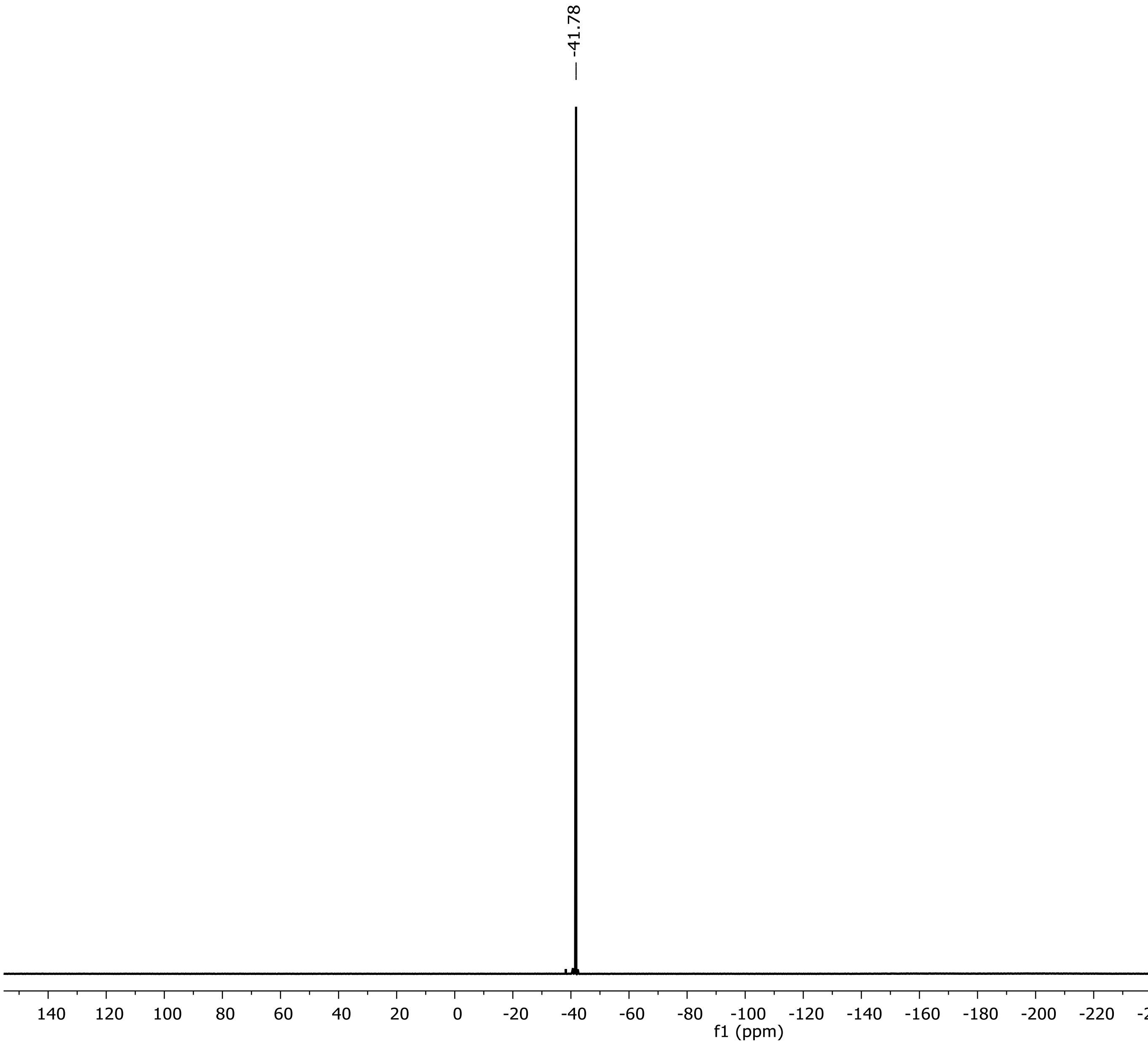

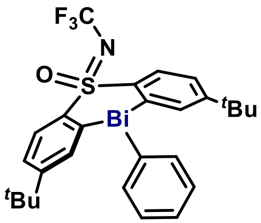

85, ^{19}F NMR, $CDCl_3$

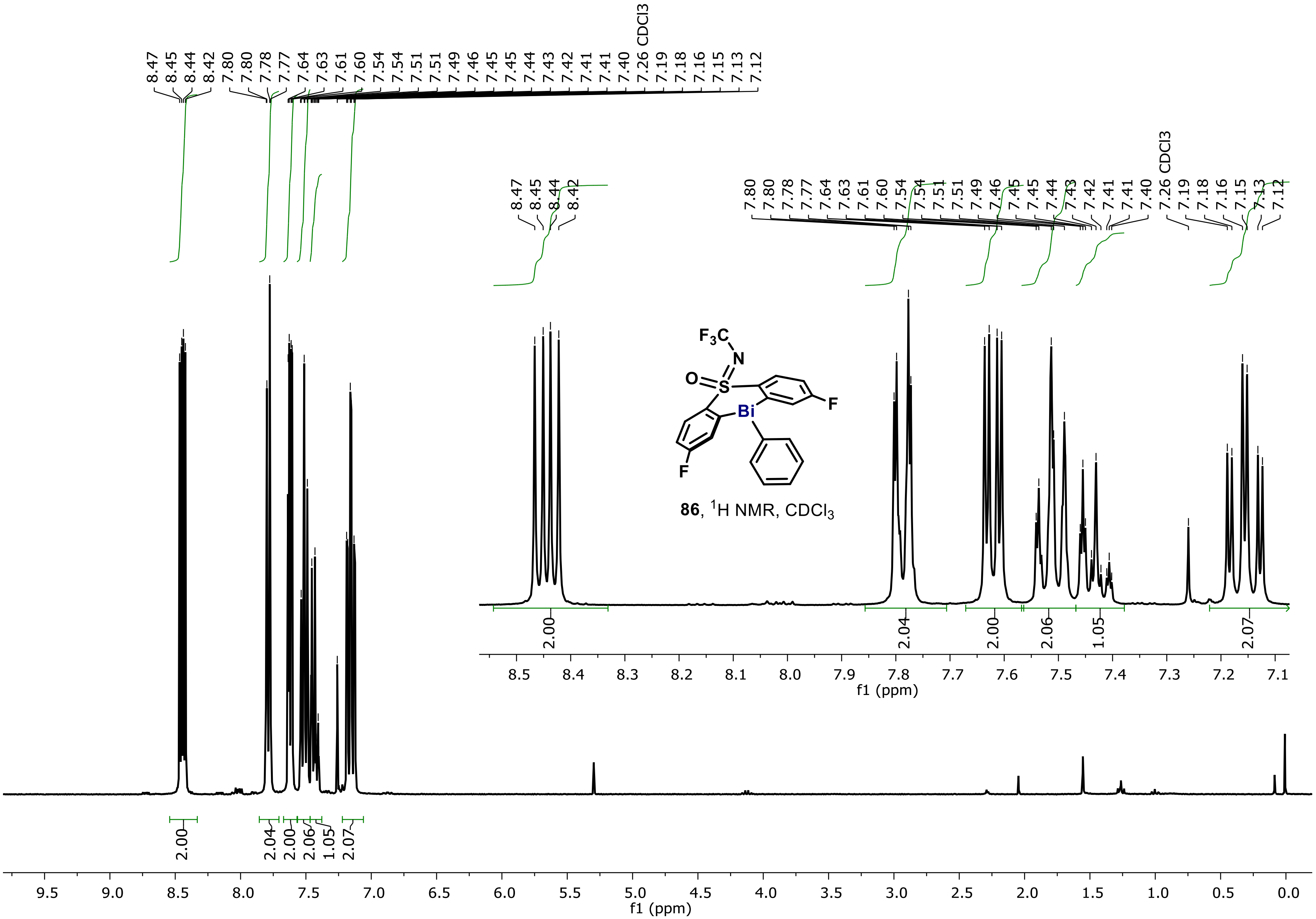
8.47
8.45
8.44
8.42
7.80
7.80
7.78
7.77
7.64
7.63
7.61
7.60
7.54
7.54
7.51
7.51
7.49
7.46
7.45
7.45
7.44
7.43
7.42
7.41
7.41
7.40
7.26 CDCl3
7.19
7.18
7.16
7.15
7.13
7.12
2.00
2.04
2.00
2.06
1.05
2.07
f1 (ppm)
F3C
N
O
S
Bi
F
F
86, 1H NMR, CDCl3

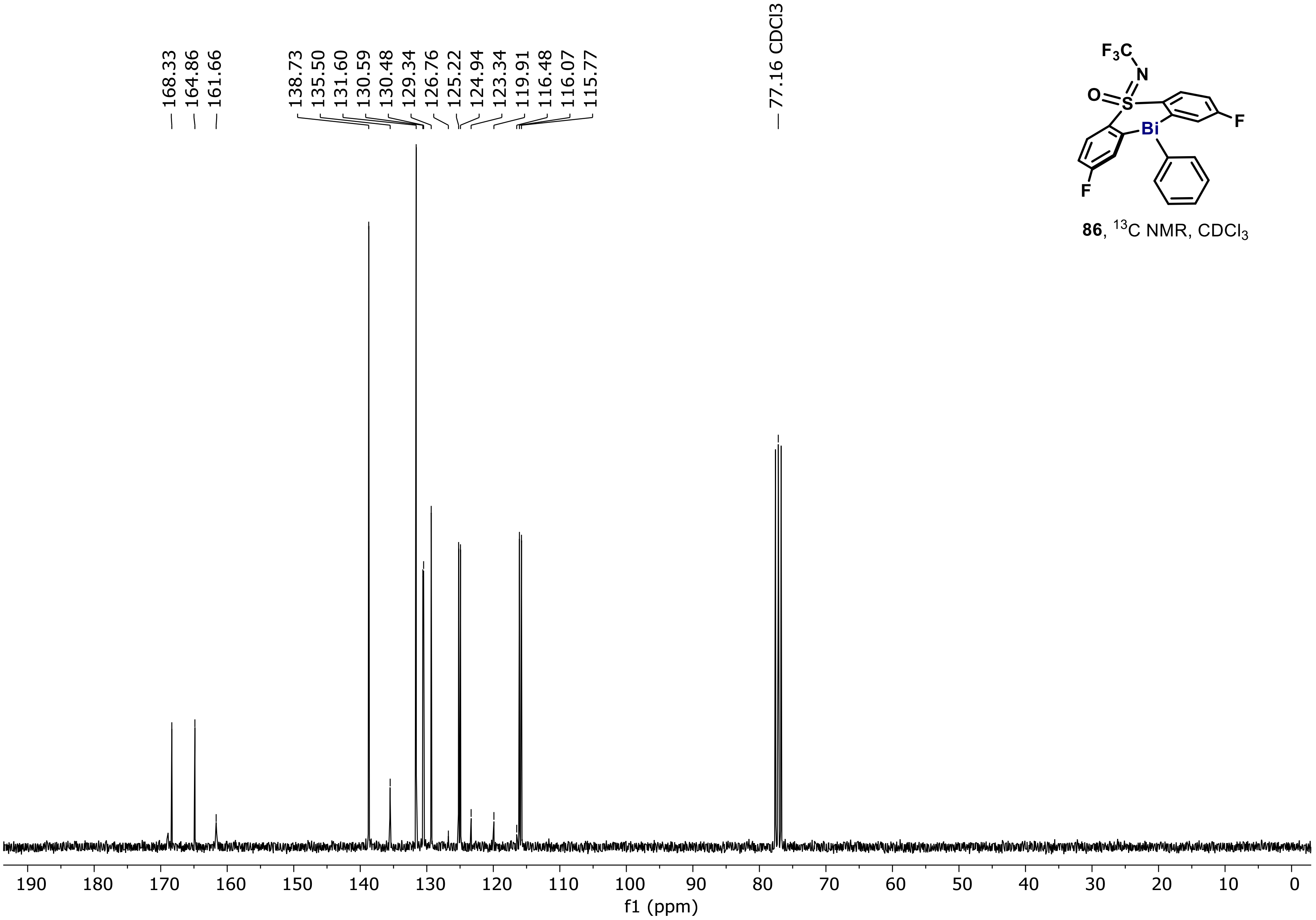

168.33
164.86
161.66
138.73
135.50
131.60
130.59
130.48
129.34
126.76
125.22
124.94
123.34
119.91
116.48
116.07
115.77
77.16 CDCl3
F3C
N
O
S
Bi
F
F
86, 13C NMR, CDCl3
190 180 170 160 150 140 130 120 110 100 90 80 70 60 50 40 30 20 10 0
f1 (ppm)

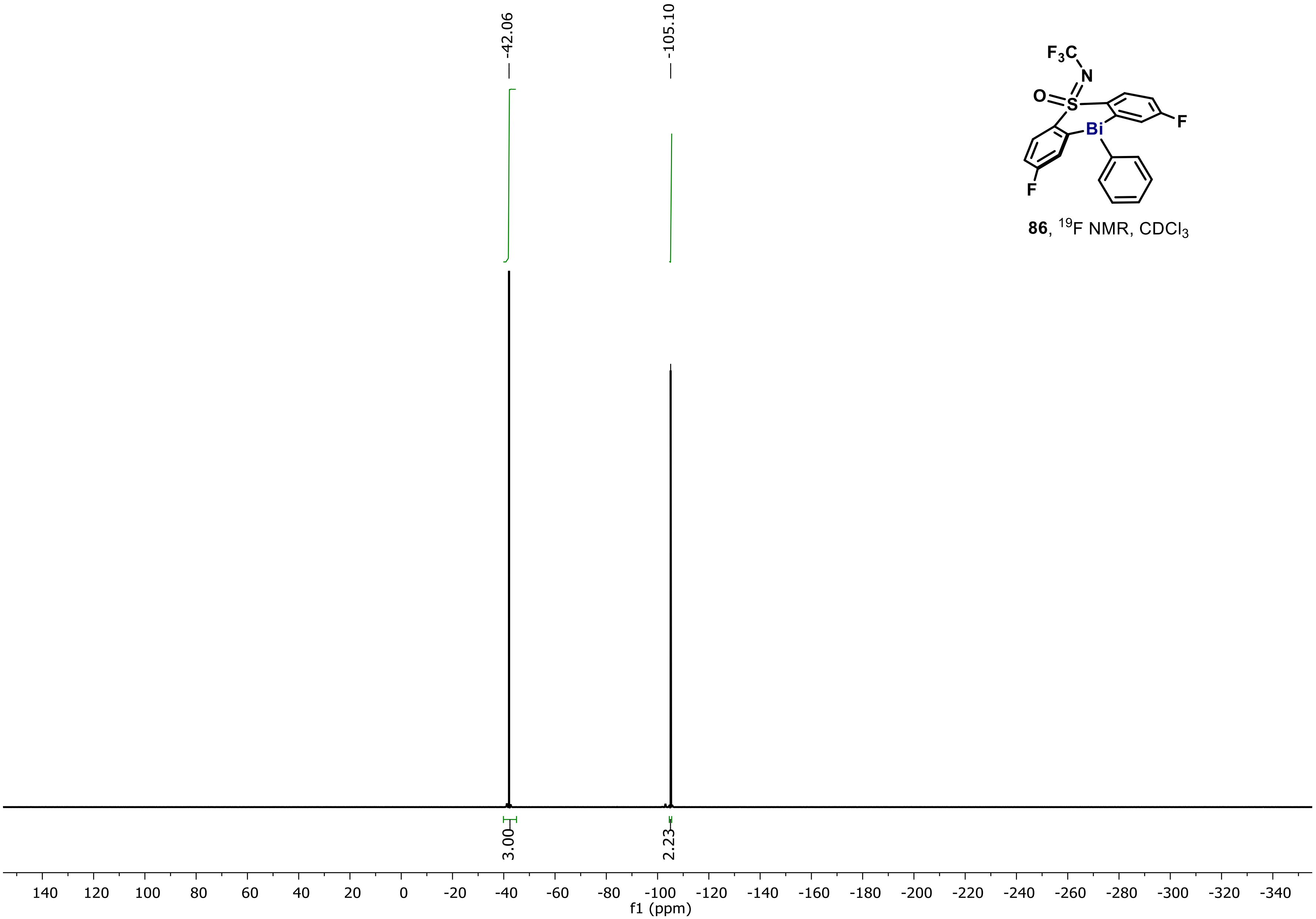

86, ^{19}F NMR, $CDCl_3$

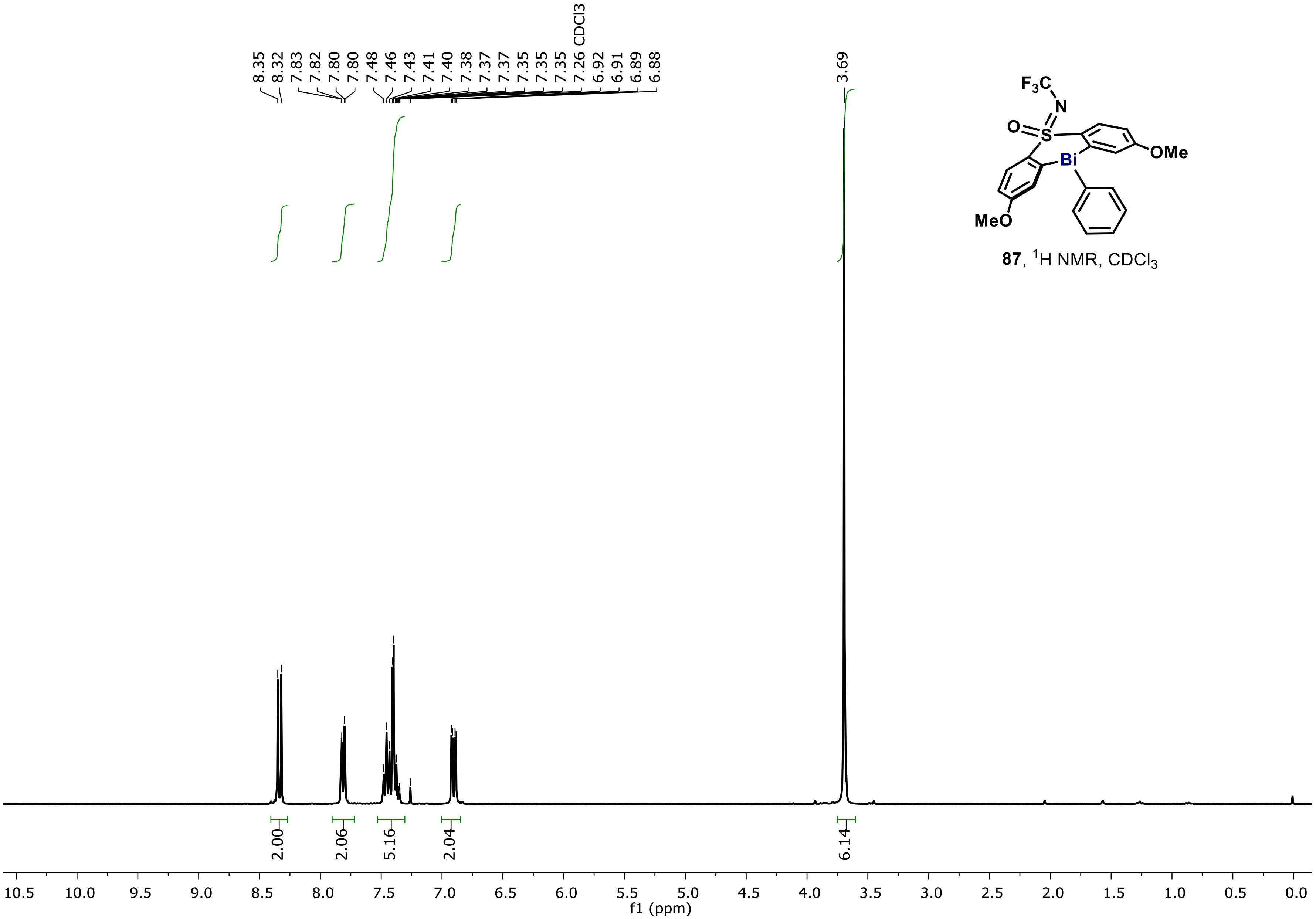

87, ^{1}H NMR, $CDCl_3$

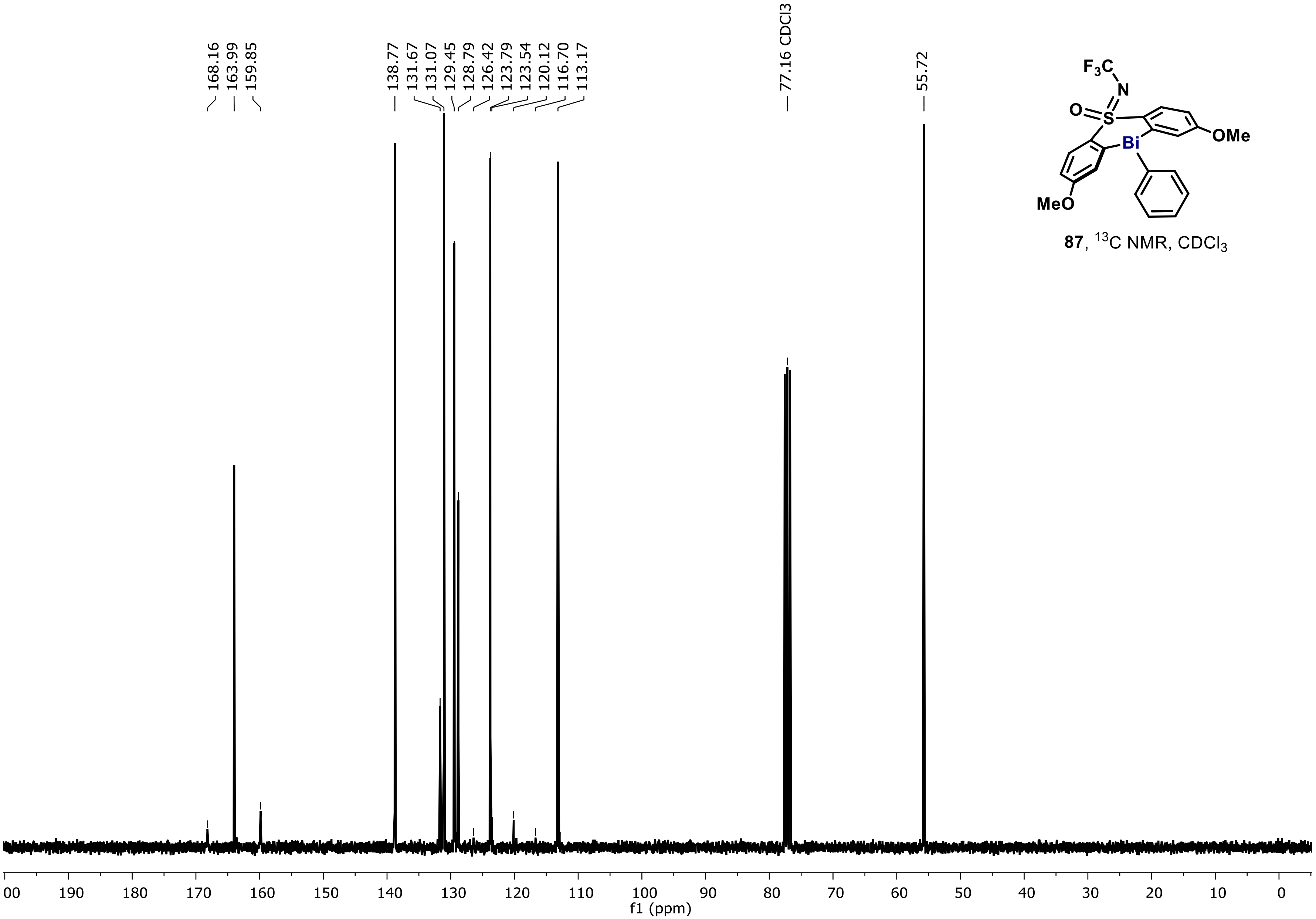

87, ^{13}C NMR, $CDCl_3$

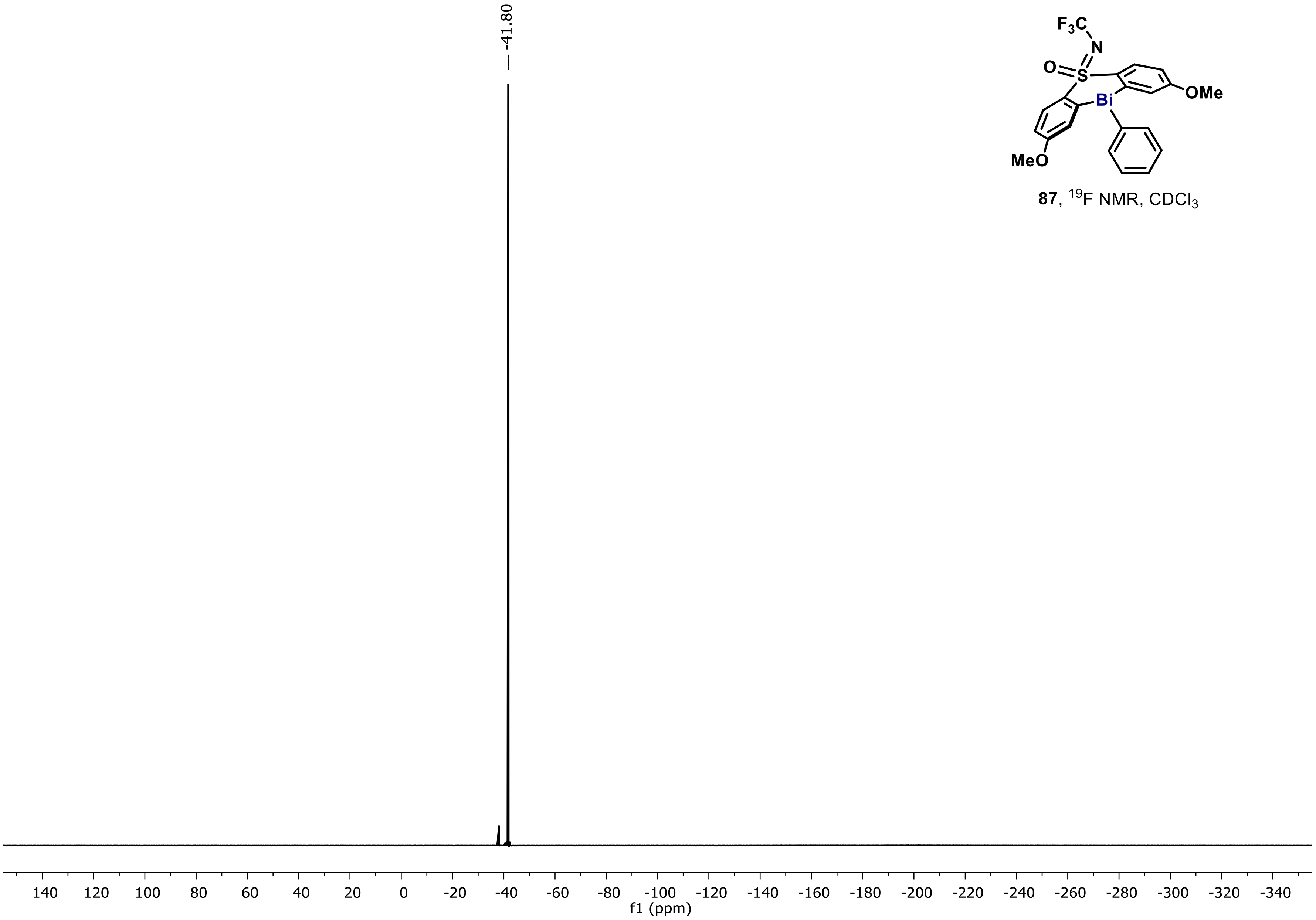

87, ^{19}F NMR, $CDCl_3$

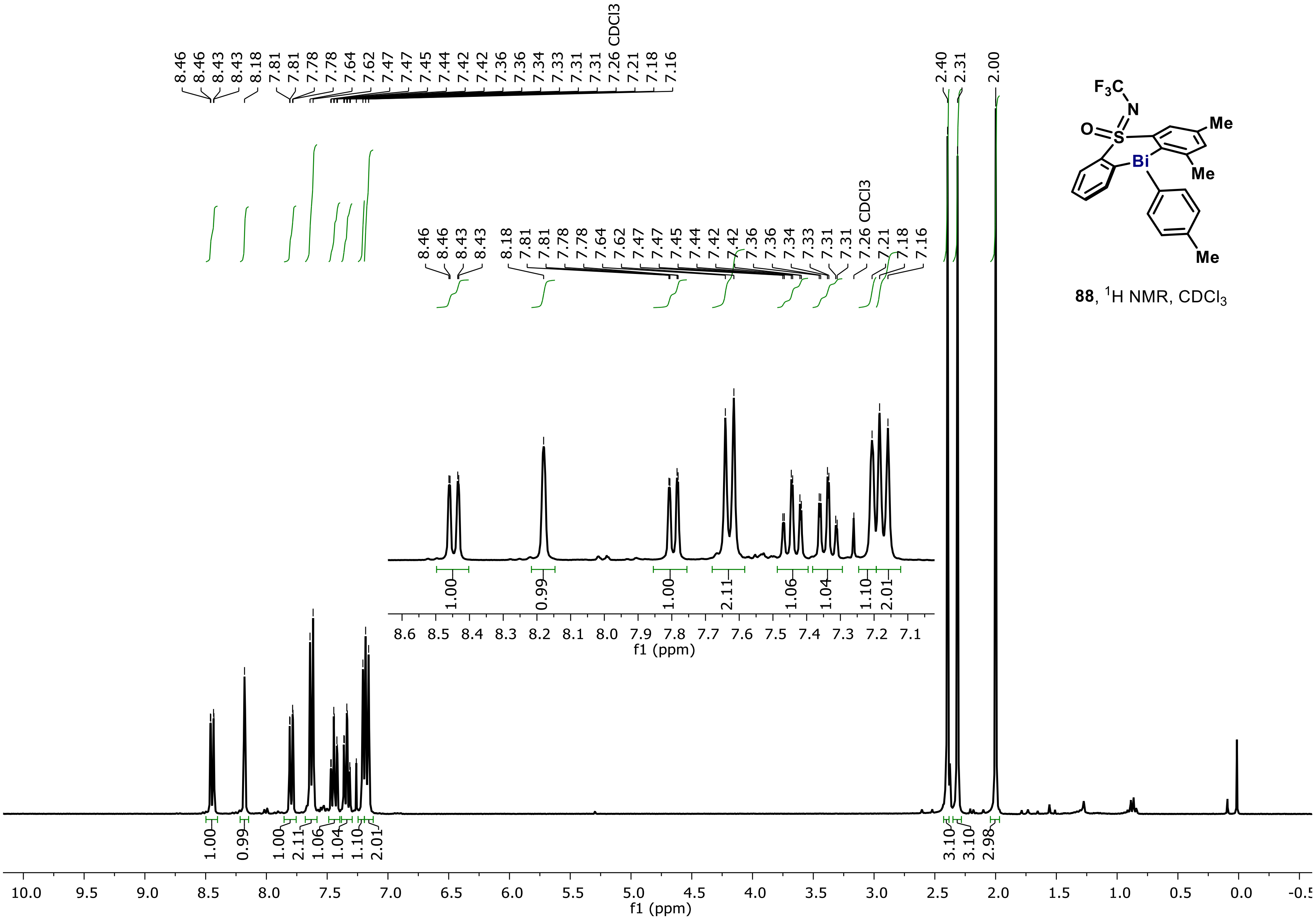

88, ^{1}H NMR, $CDCl_3$

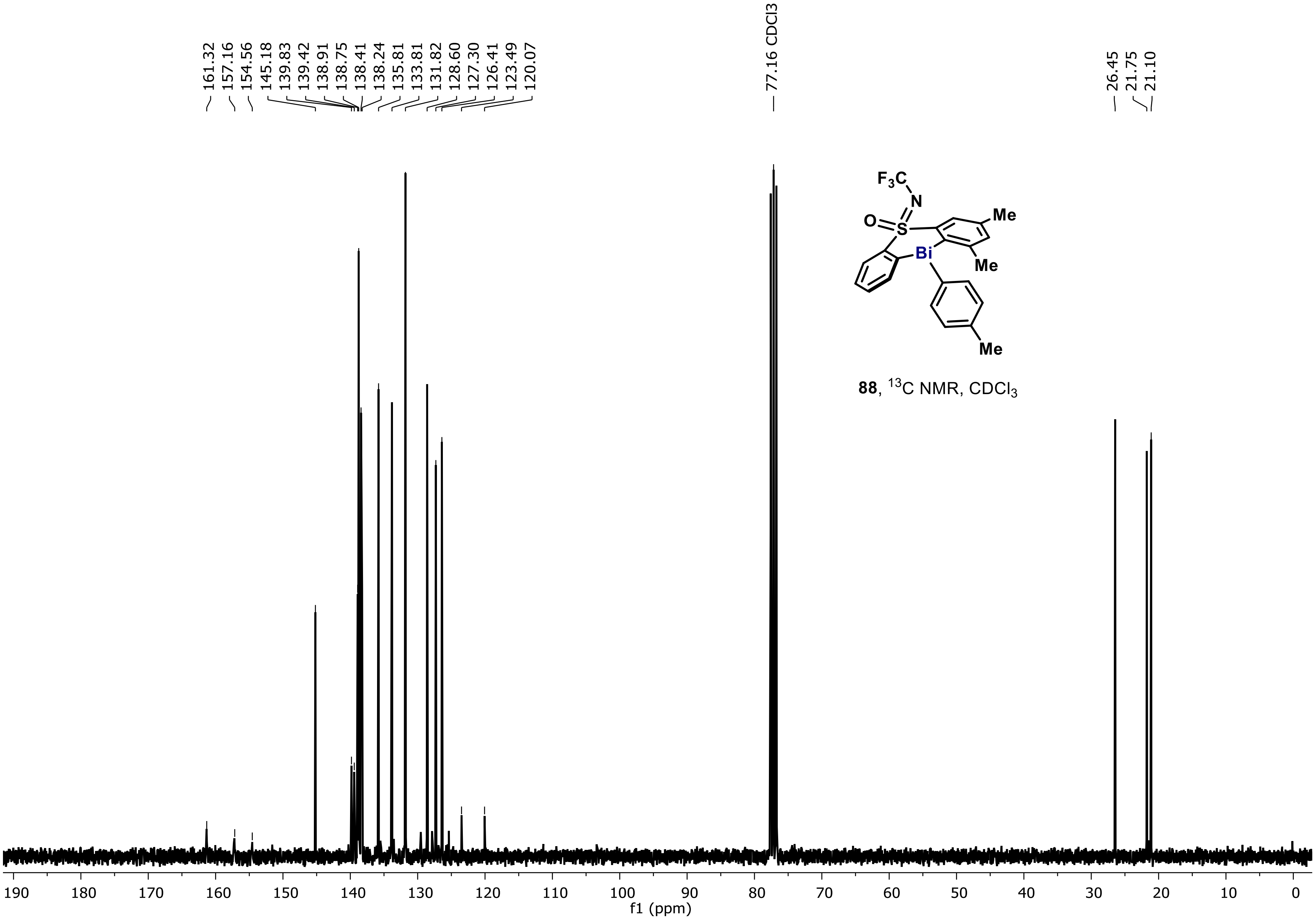

88, ^{13}C NMR, $CDCl_3$

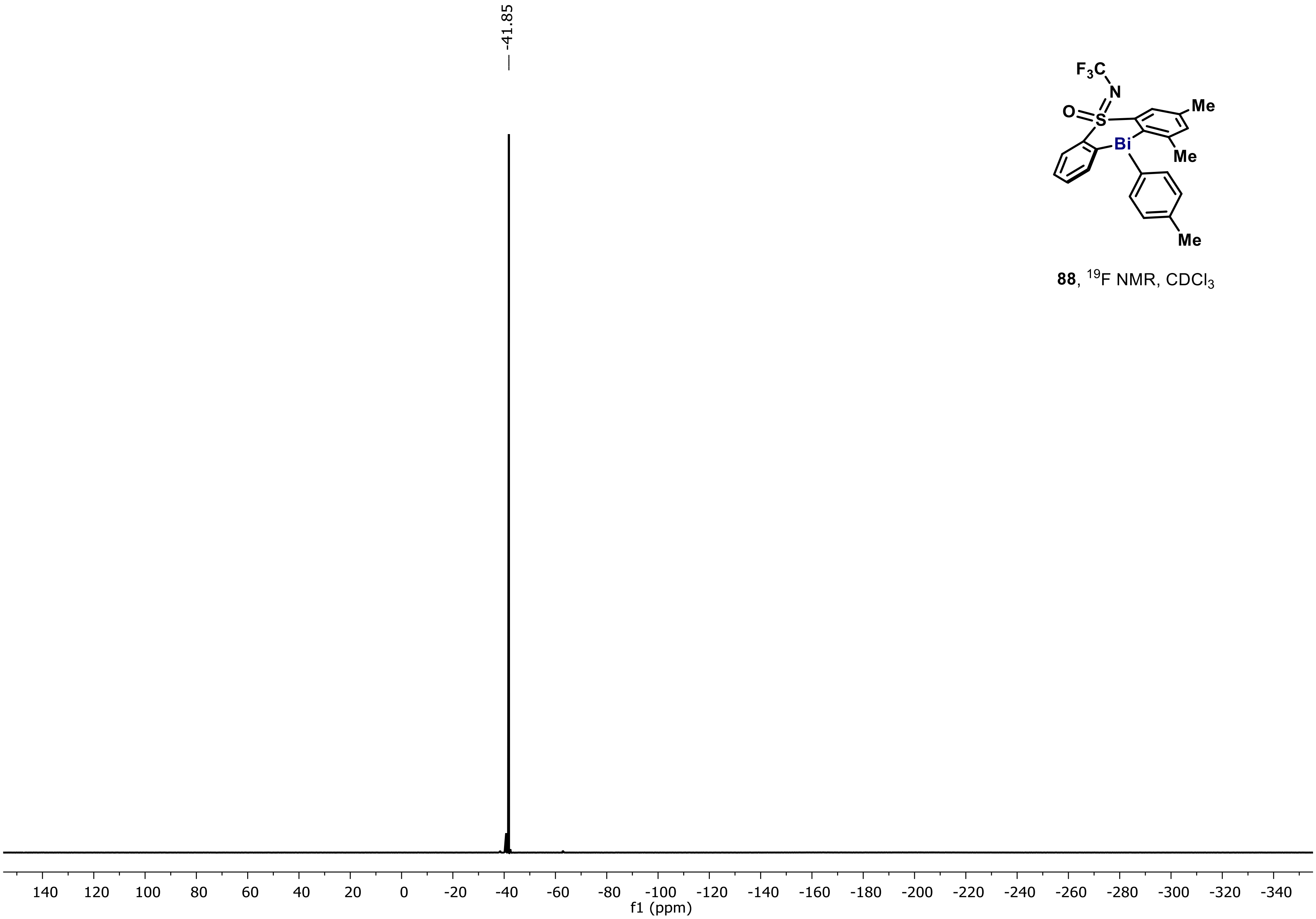

88, ^{19}F NMR, $CDCl_3$

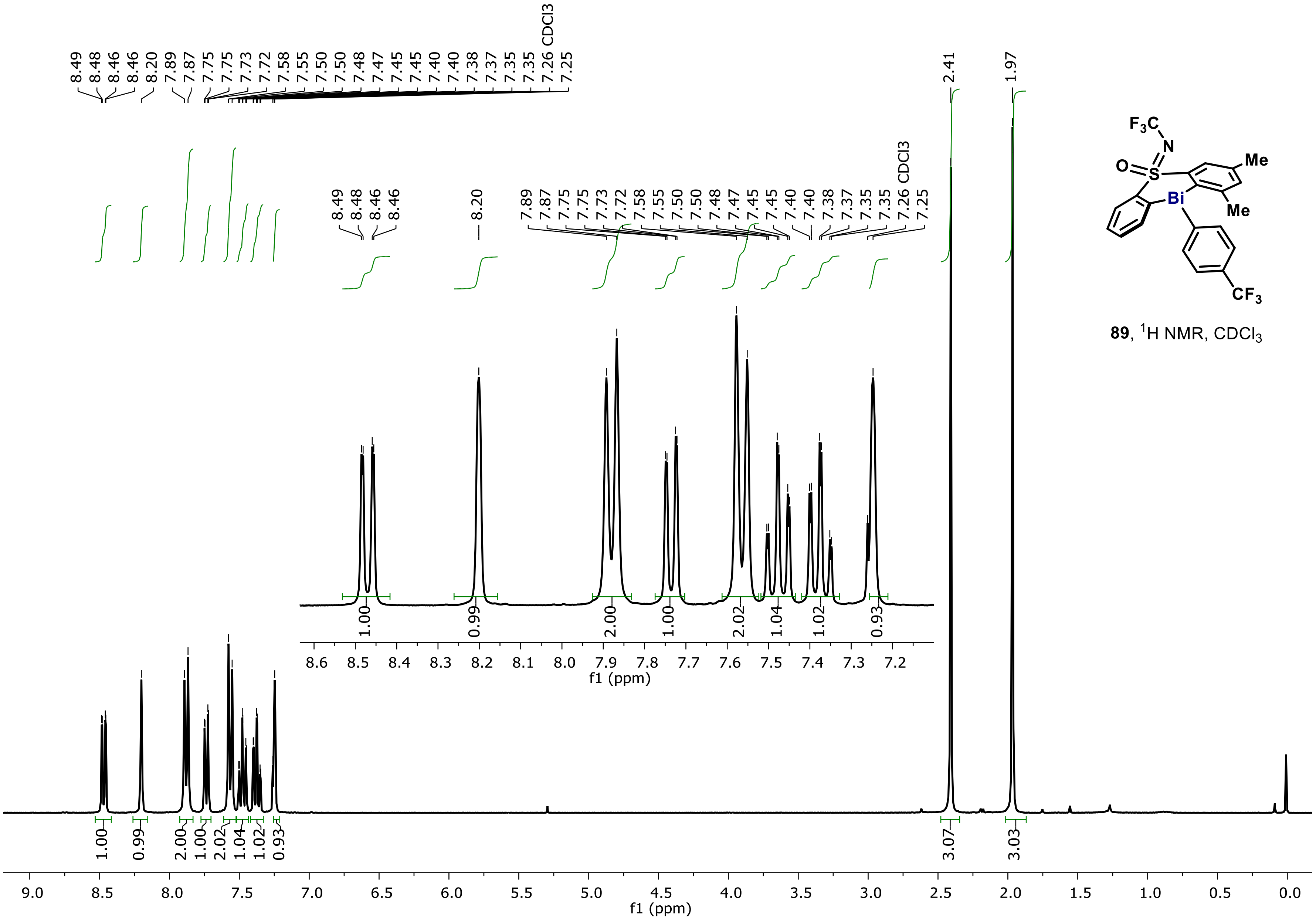

89, ^{1}H NMR, $CDCl_3$

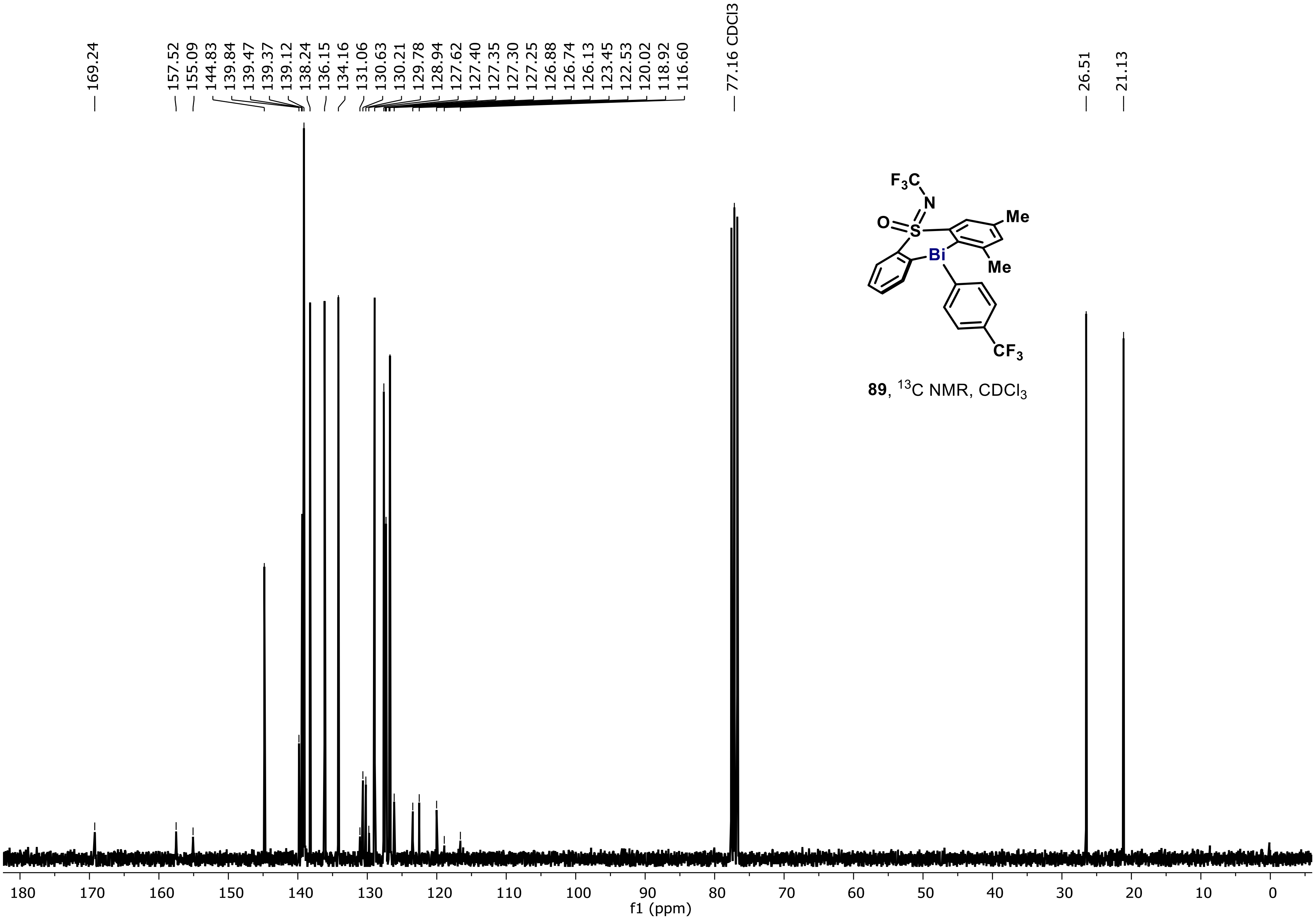

169.24
157.52
155.09
144.83
139.84
139.47
139.37
139.12
138.24
136.15
134.16
131.06
130.63
130.21
129.78
128.94
127.62
127.40
127.35
127.30
127.25
126.88
126.74
126.13
123.45
122.53
120.02
118.92
116.60
77.16 CDCl3
26.51
21.13
F3C
N
O
S
Me
Bi
Me
CF3
89, 13C NMR, CDCl3
180
170
160
150
140
130
120
110
100
90
80
70
60
50
40
30
20
10
0
f1 (ppm)

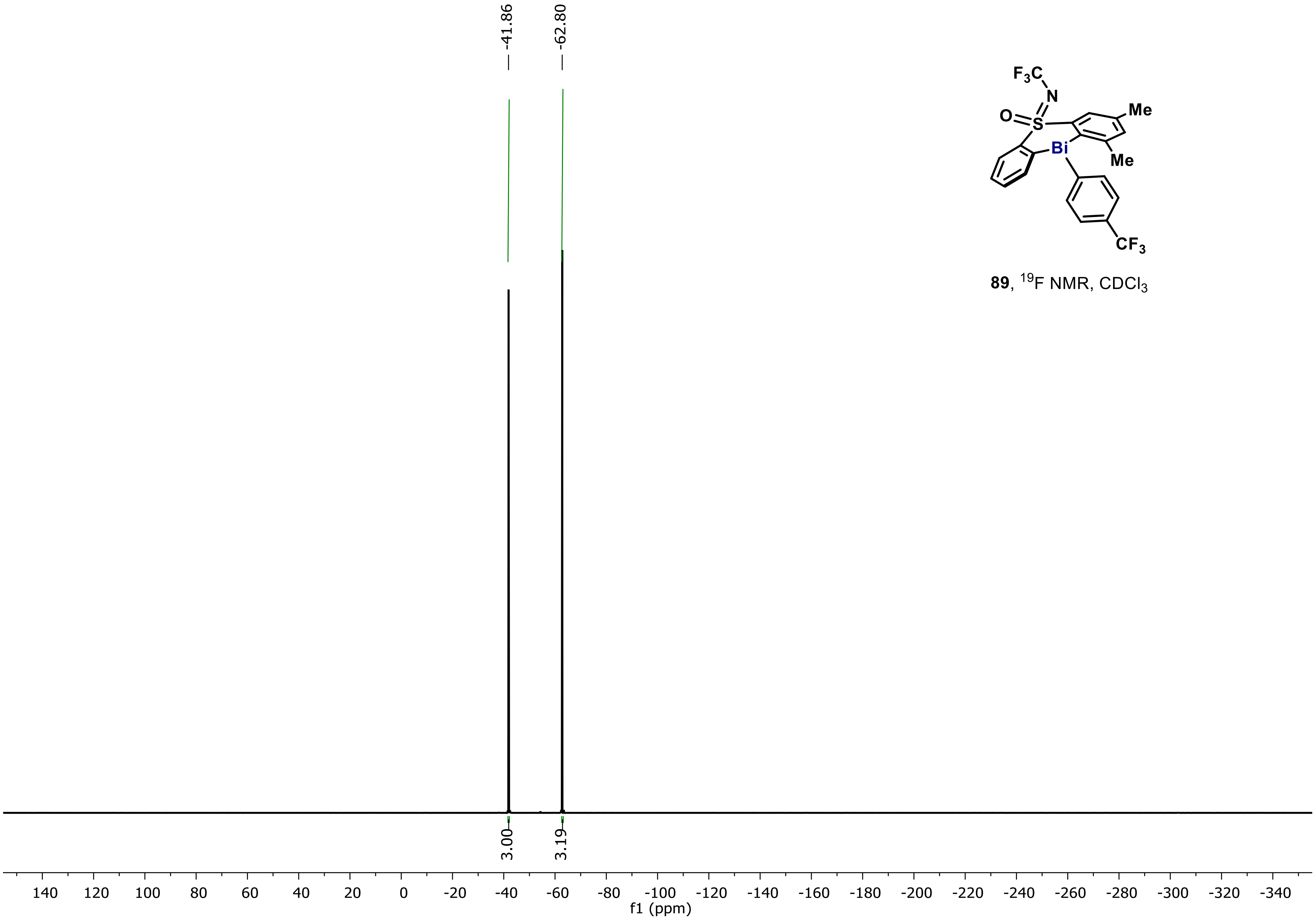

89, ^{19}F NMR, $CDCl_3$

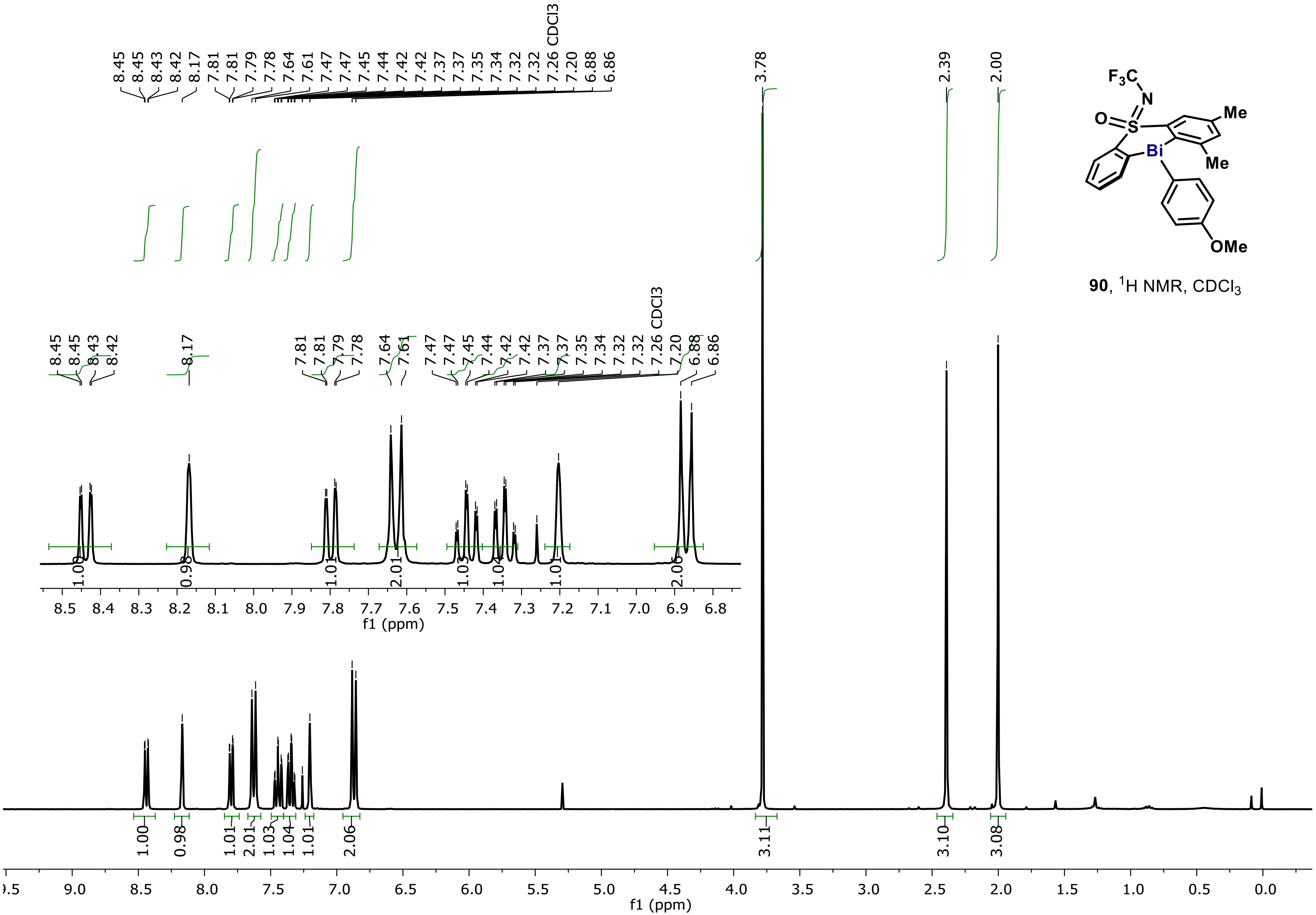

90, ^{1}H NMR, $CDCl_3$

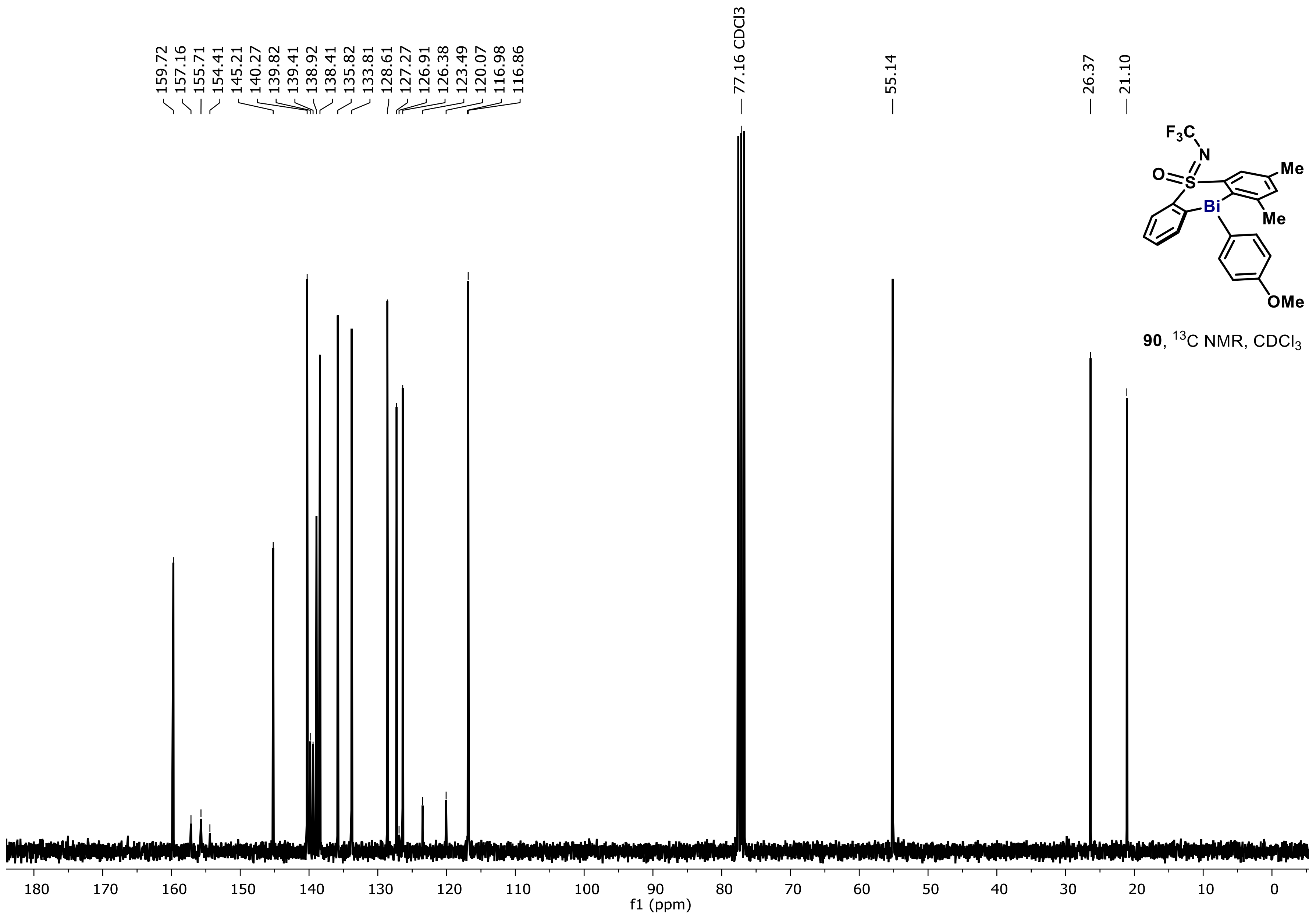
159.72
157.16
155.71
154.41
145.21
140.27
139.82
139.41
138.92
138.41
135.82
133.81
128.61
127.27
126.91
126.38
123.49
120.07
116.98
116.86
77.16 CDCl3
55.14
26.37
21.10
F3C
N
O
S
Me
Bi
Me
OMe
90, 13C NMR, CDCl3
f1 (ppm)

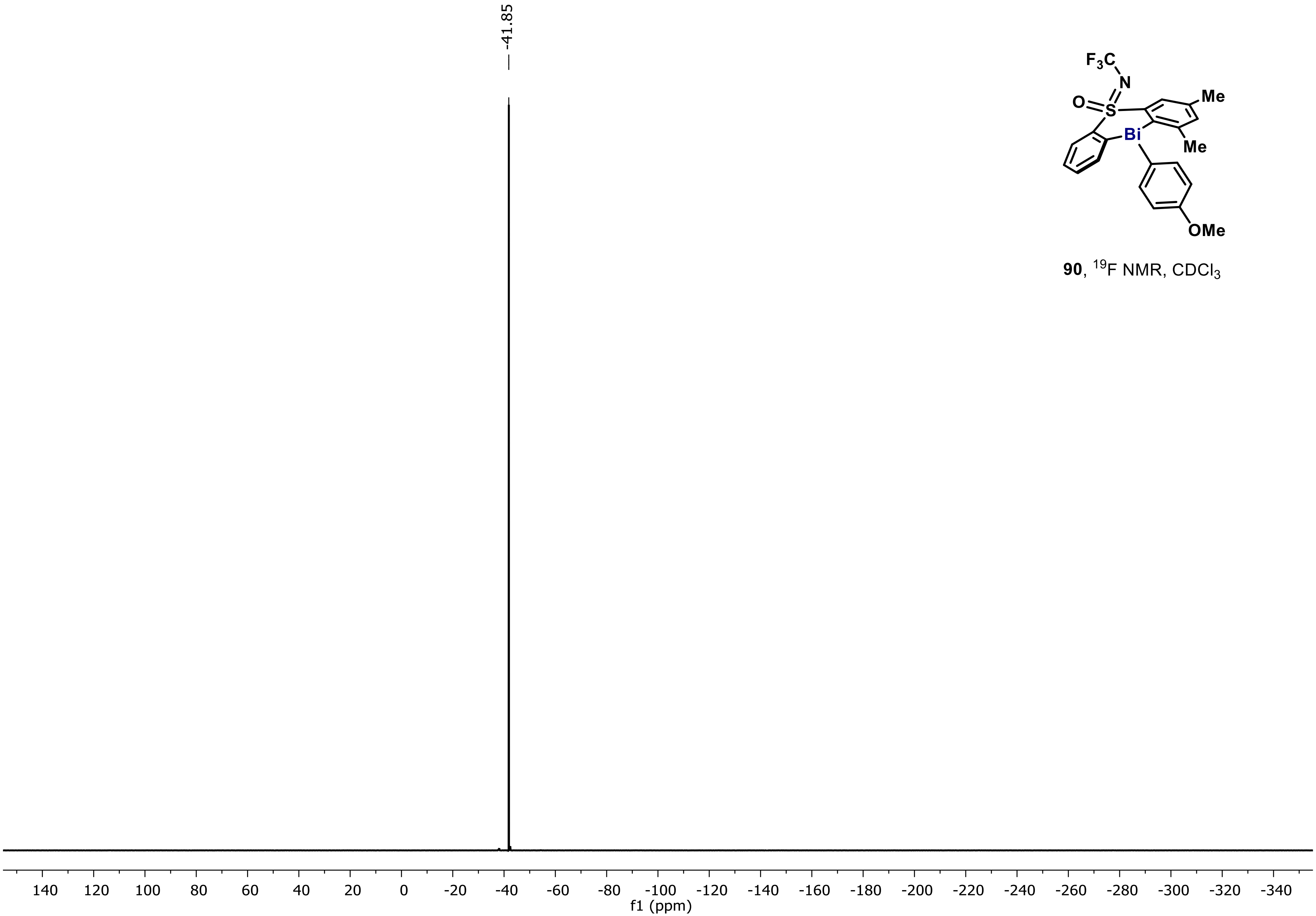

90, ^{19}F NMR, $CDCl_3$

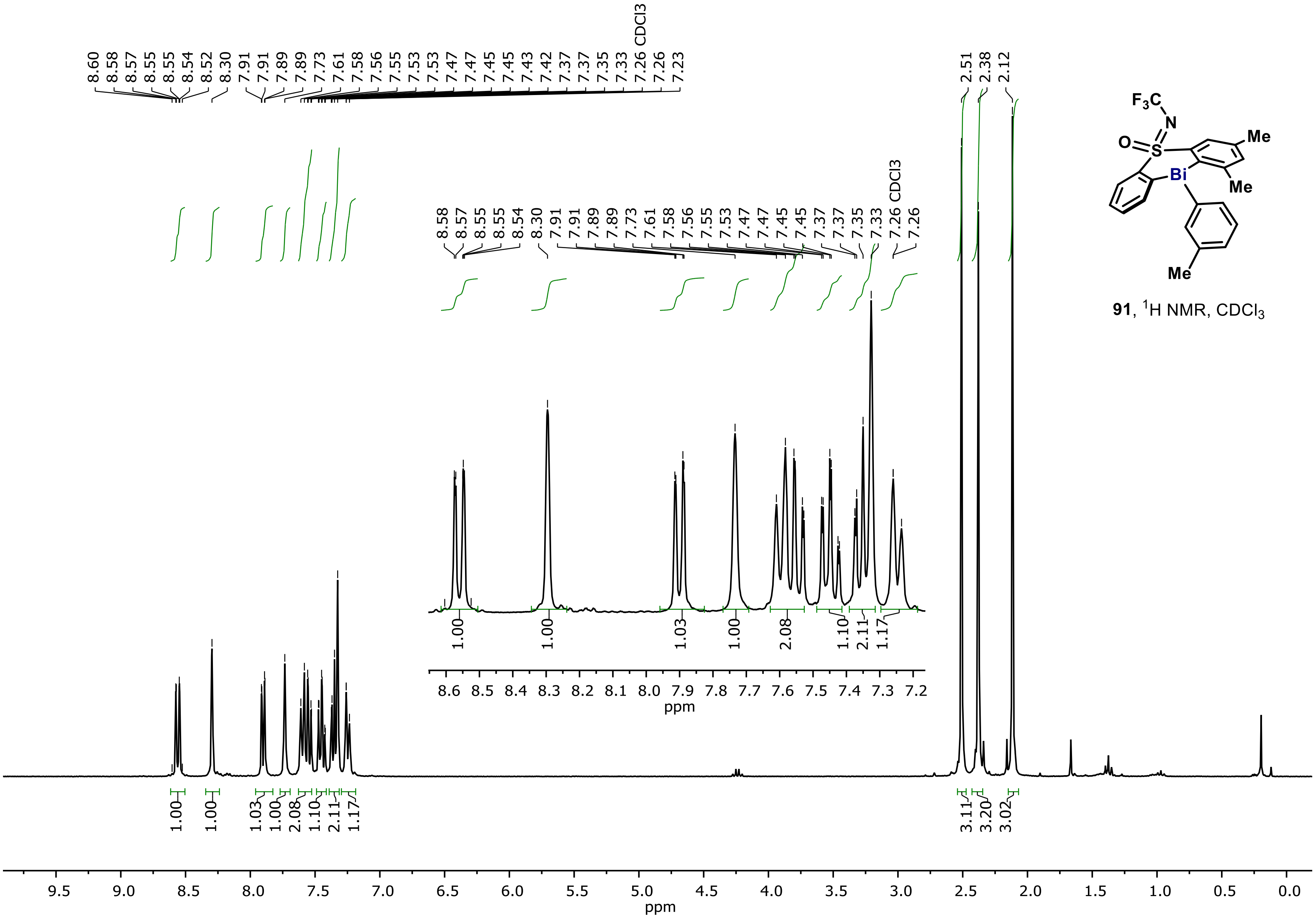

91, ^{1}H NMR, $CDCl_3$

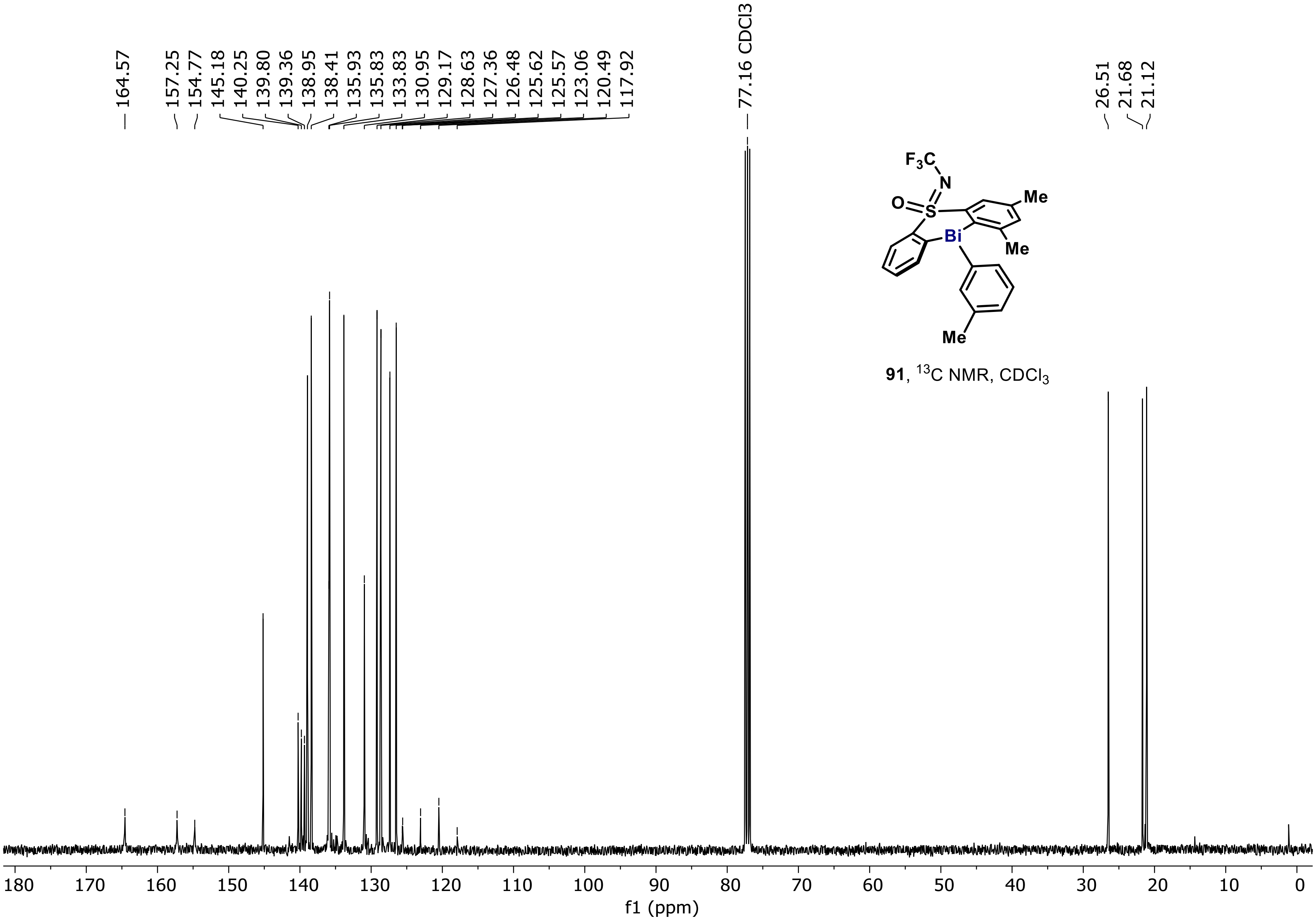
164.57
157.25
154.77
145.18
140.25
139.80
139.36
138.95
138.41
135.93
135.83
133.83
130.95
129.17
128.63
127.36
126.48
125.62
125.57
123.06
120.49
117.92
77.16 CDCl3
26.51
21.68
21.12
F3C
N
O
S
Bi
Me
Me
Me
91, 13C NMR, CDCl3
180
170
160
150
140
130
120
110
100
90
80
70
60
50
40
30
20
10
0
f1 (ppm)

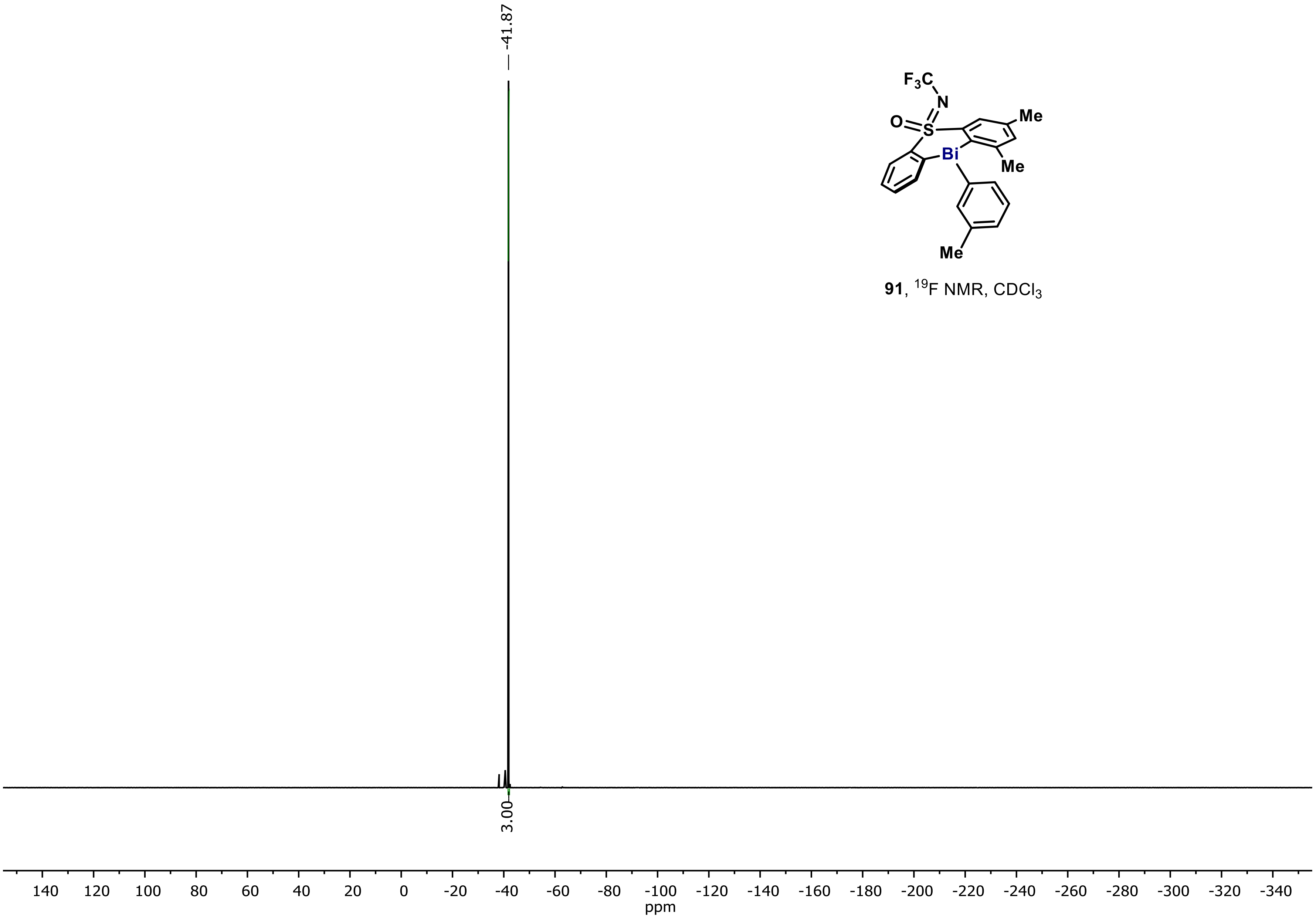

91, ^{19}F NMR, $CDCl_3$

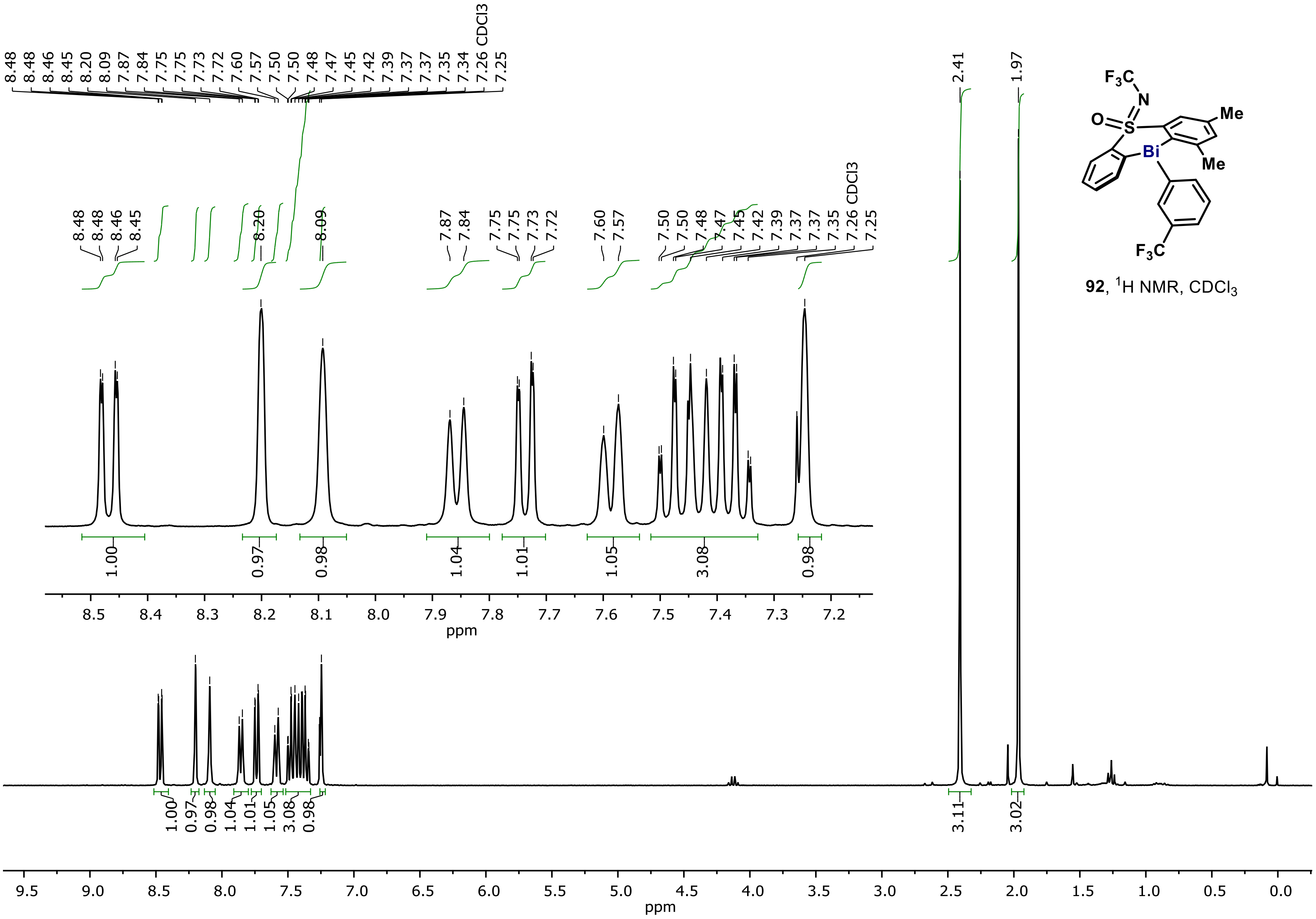

92, ^{1}H NMR, $CDCl_3$

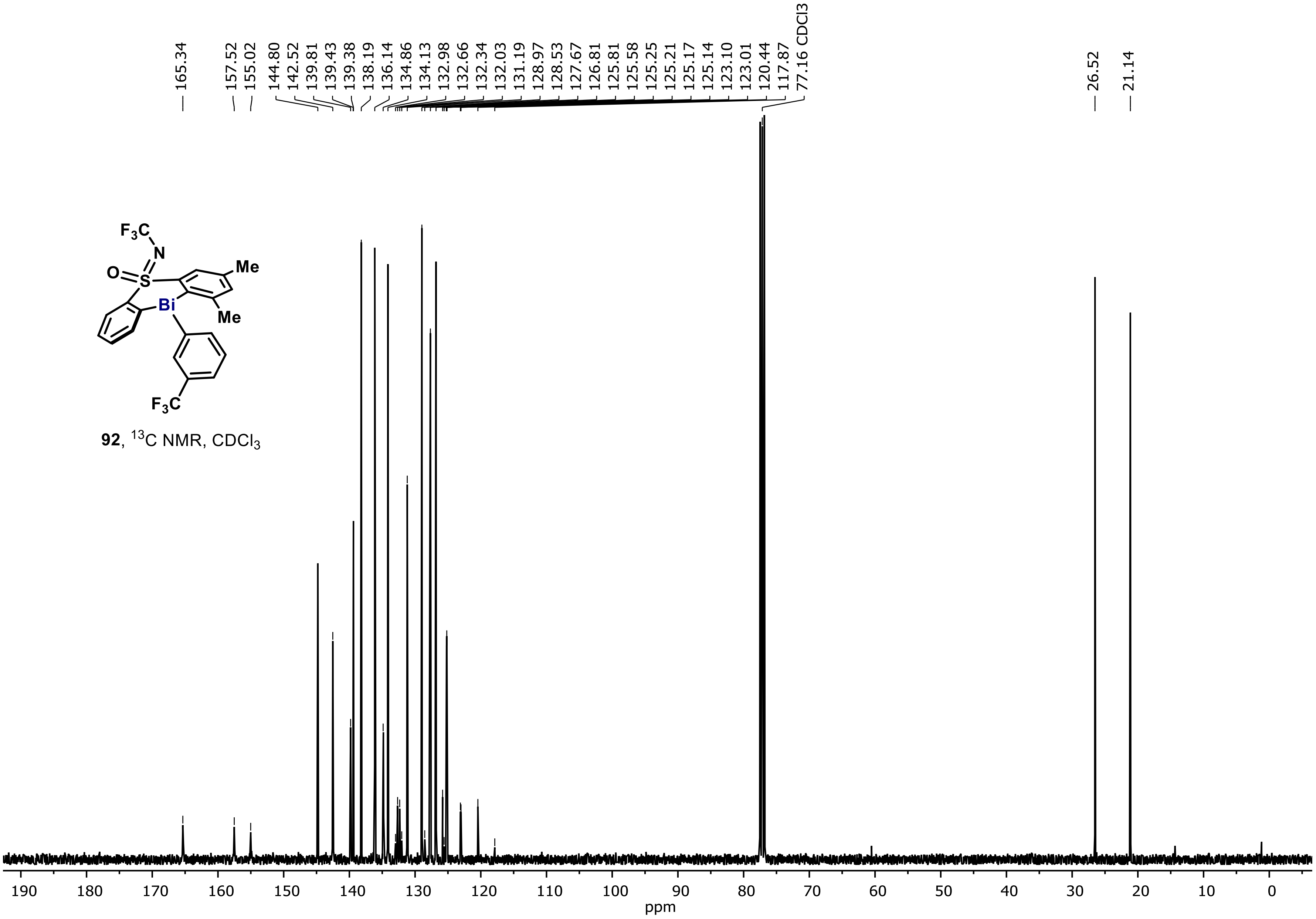

92, ^{13}C NMR, $CDCl_3$

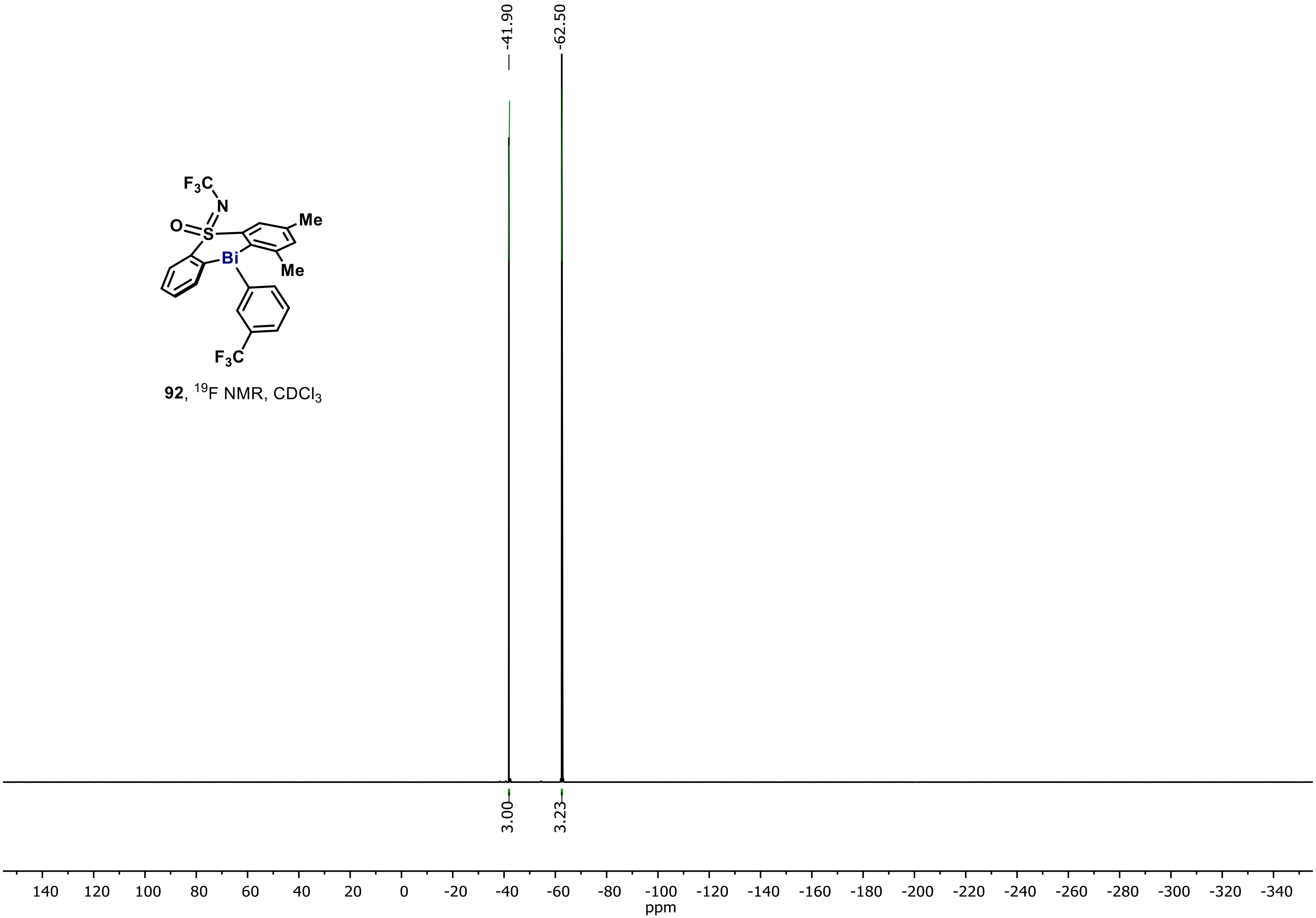

92, ^{19}F NMR, $CDCl_3$

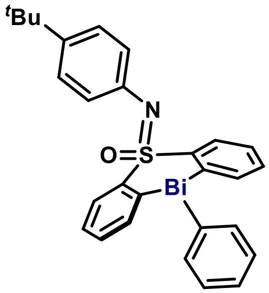

93, ^{1}H NMR, $CDCl_3$

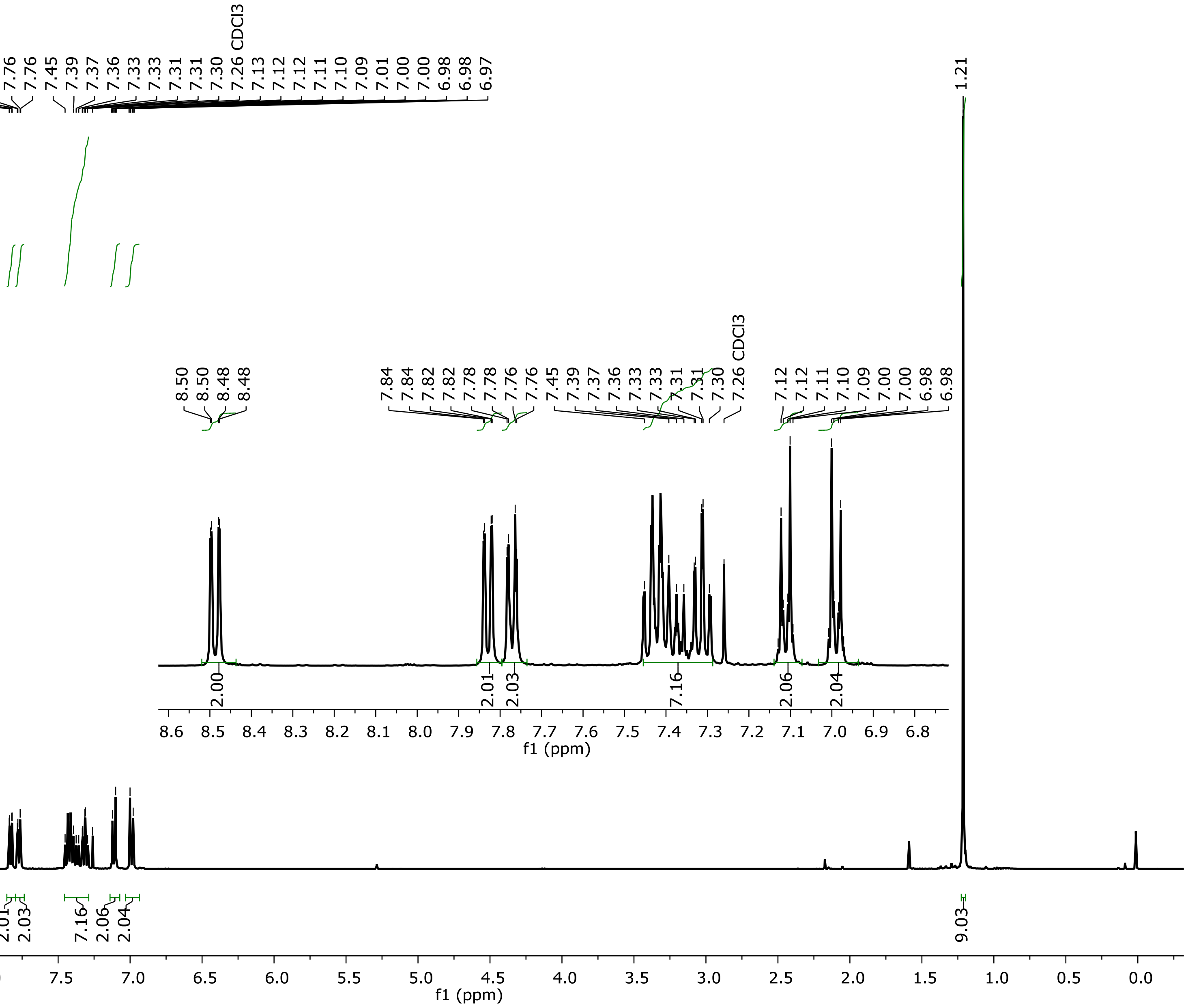

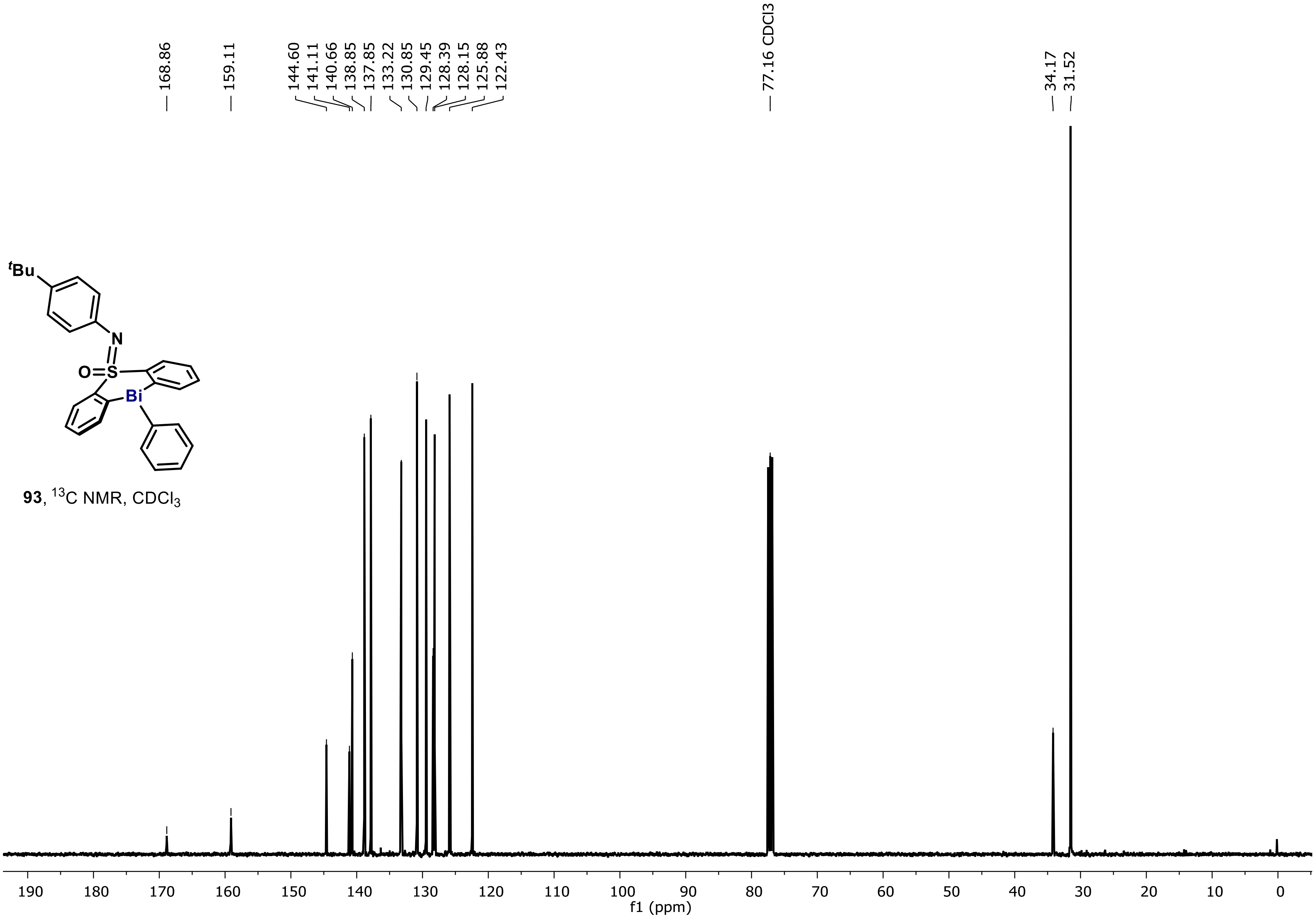

93, ^{13}C NMR, $CDCl_3$

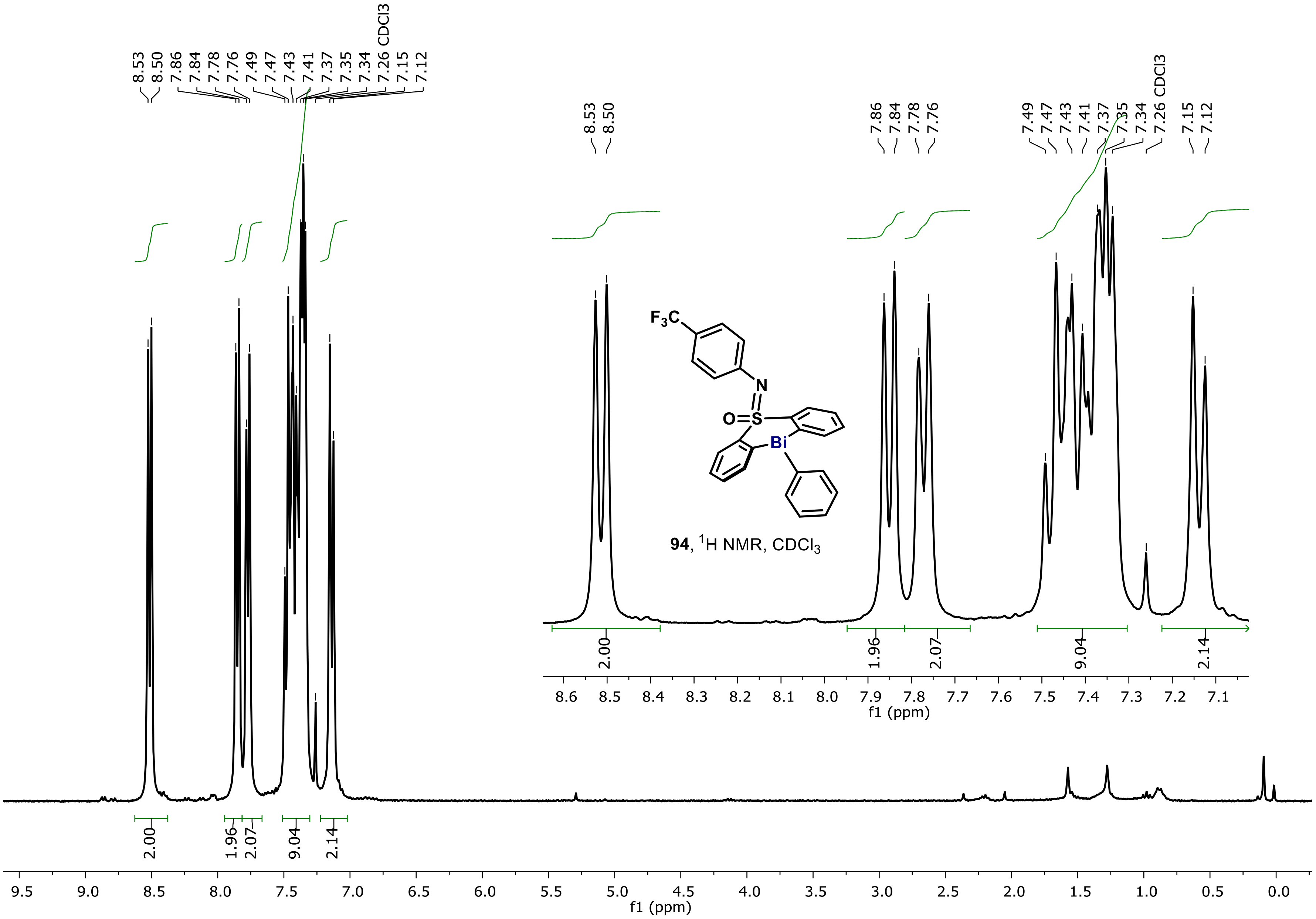
8.53
8.50
7.86
7.84
7.78
7.76
7.49
7.47
7.43
7.41
7.37
7.35
7.34
7.26 CDCl3
7.15
7.12
F3C
N
O=S
Bi
94, 1H NMR, CDCl3
2.00
1.96
2.07
9.04
2.14
f1 (ppm)

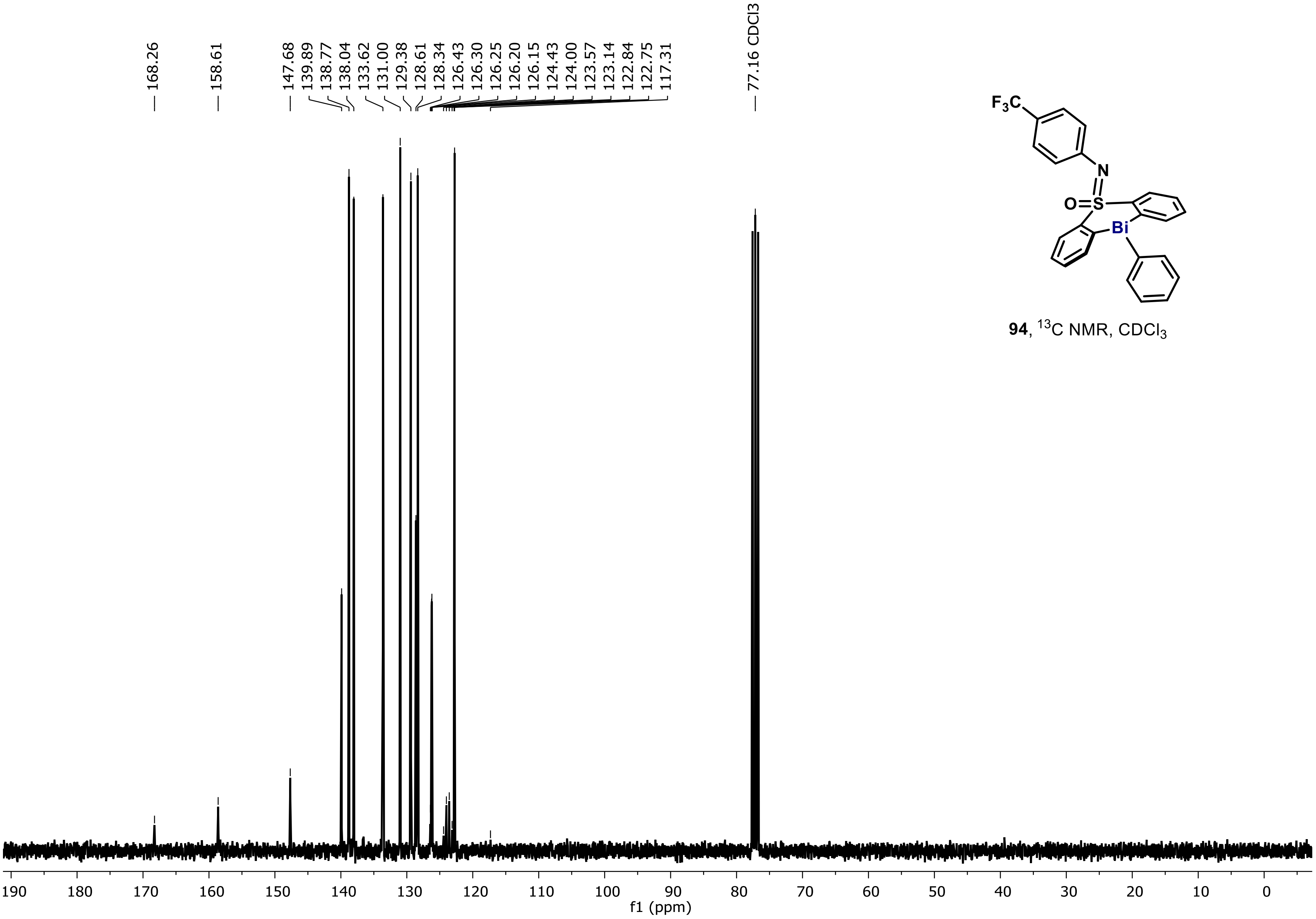

94, ^{13}C NMR, $CDCl_3$

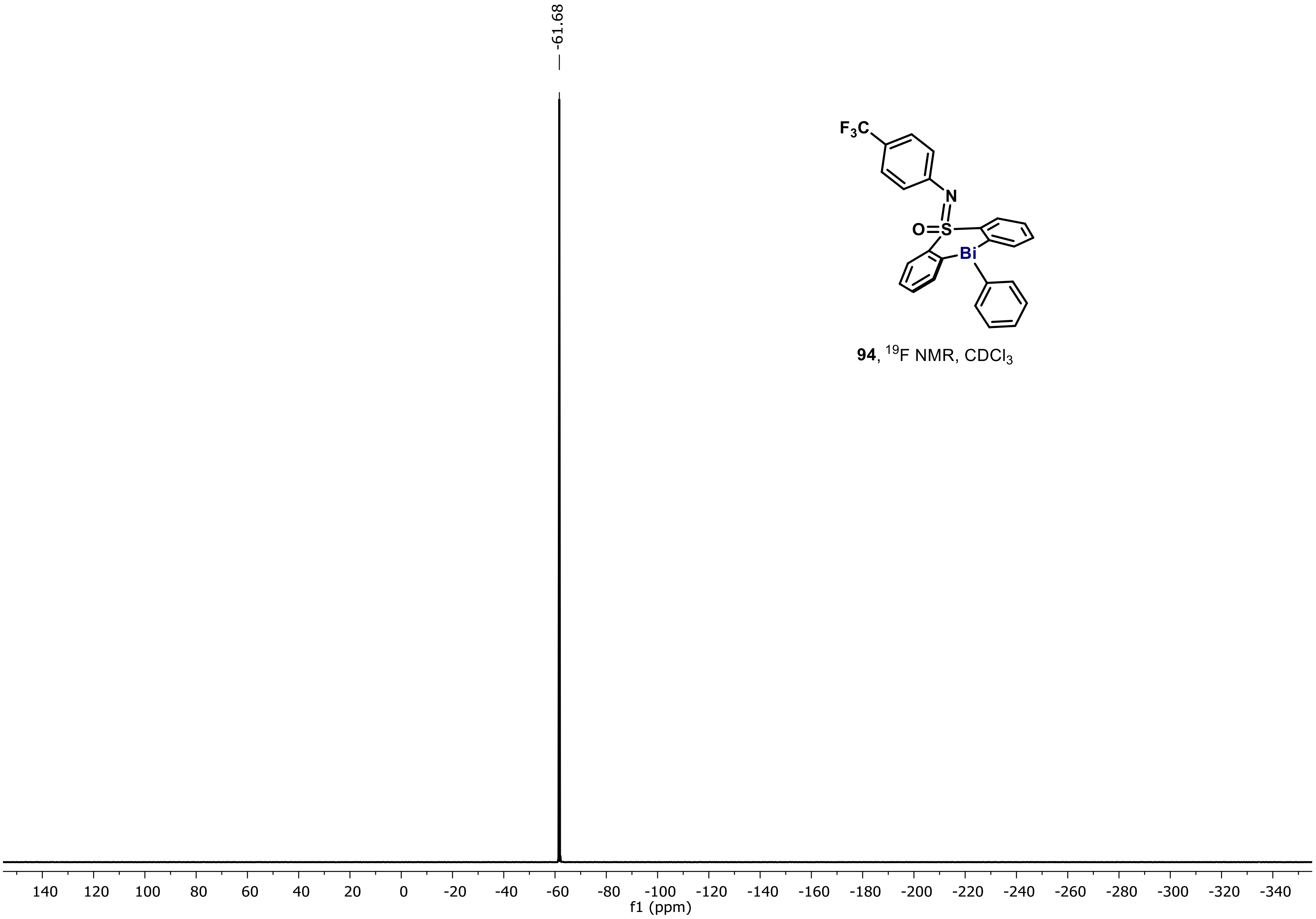
-61.68
F3C
N
O
S
Bi
94, 19F NMR, CDCl3
140 120 100 80 60 40 20 0 -20 -40 -60 -80 -100 -120 -140 -160 -180 -200 -220 -240 -260 -280 -300 -320 -340
f1 (ppm)

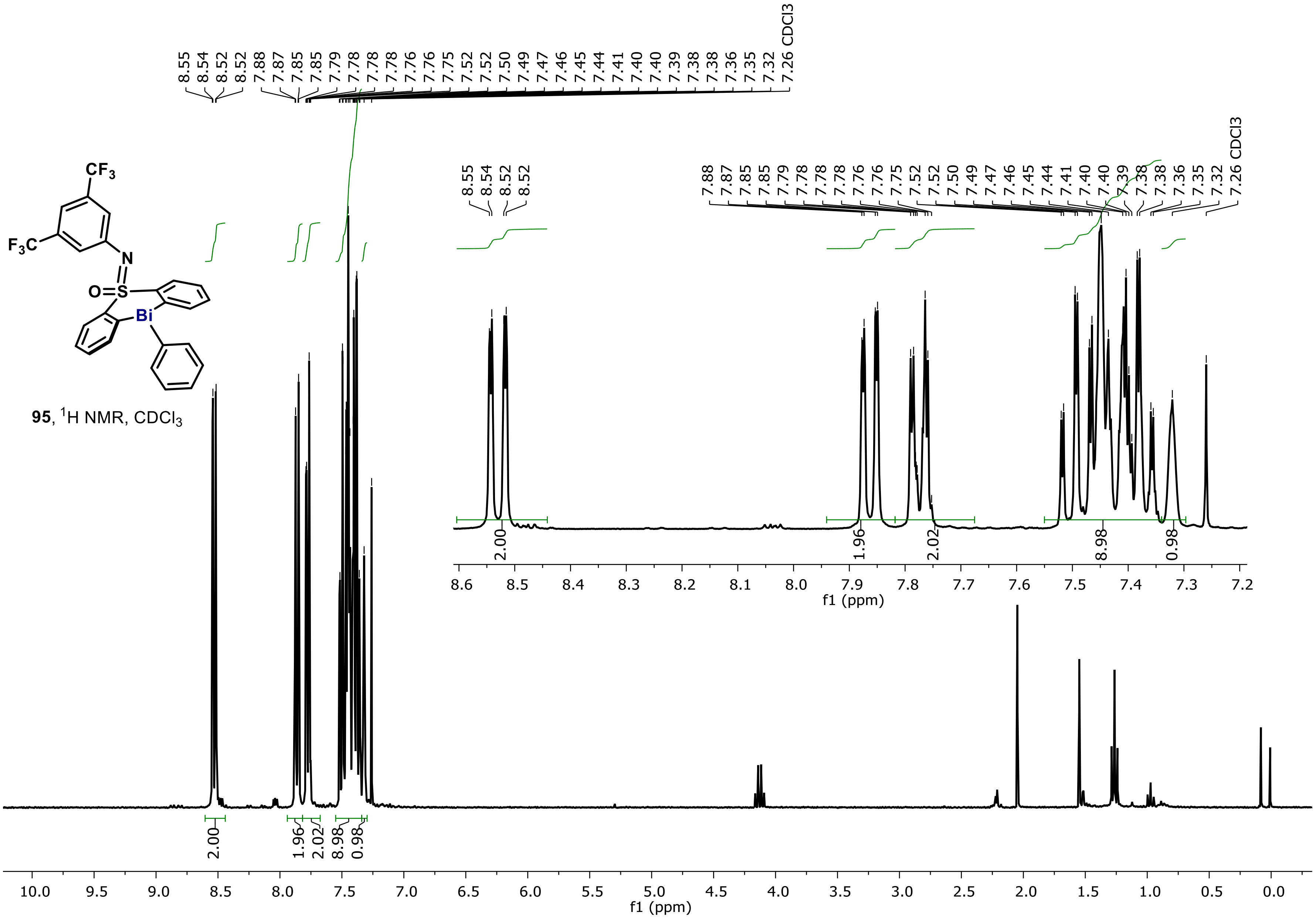

95, ^{1}H NMR, CDCl$_3$

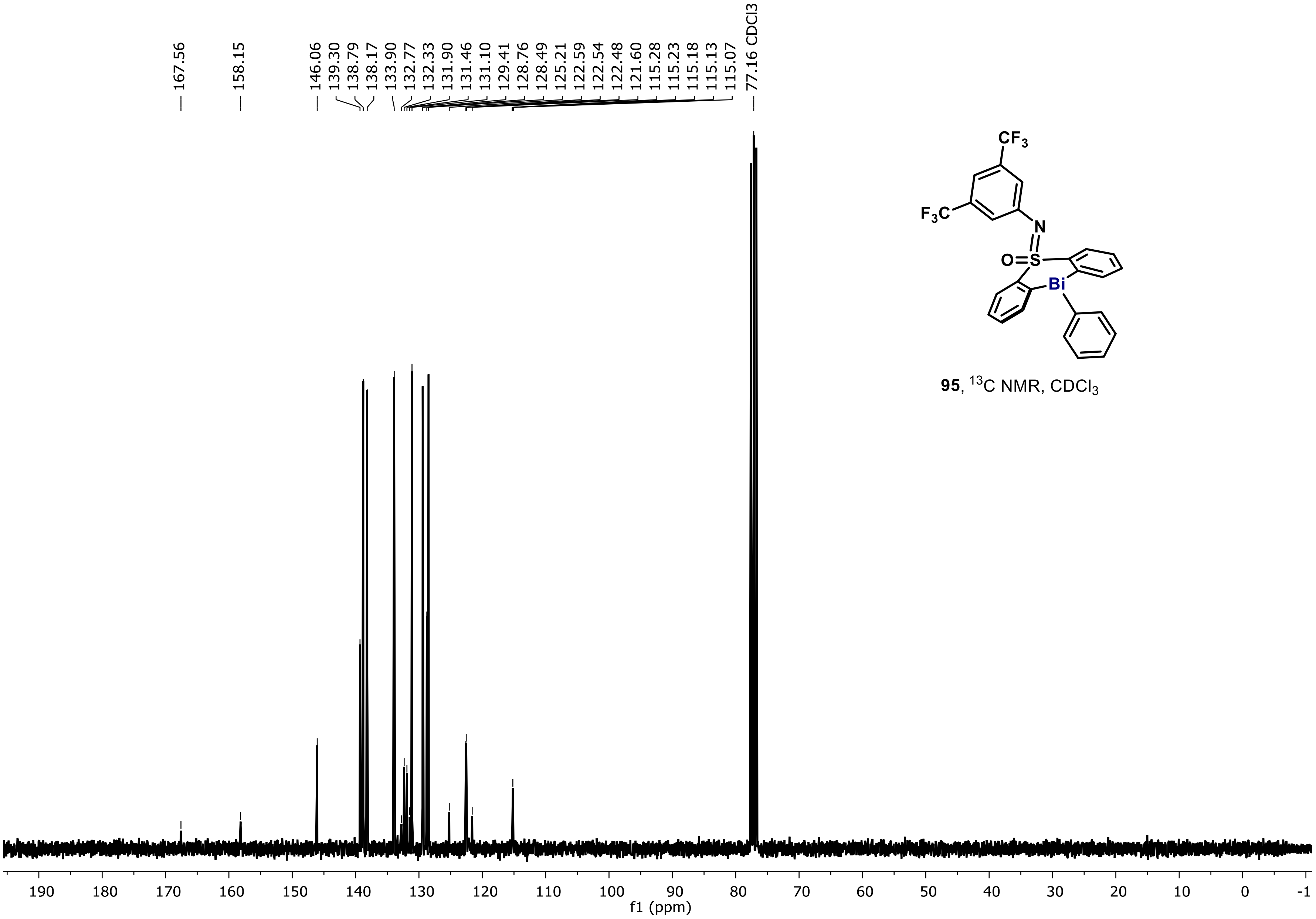

95, ^{13}C NMR, $CDCl_3$

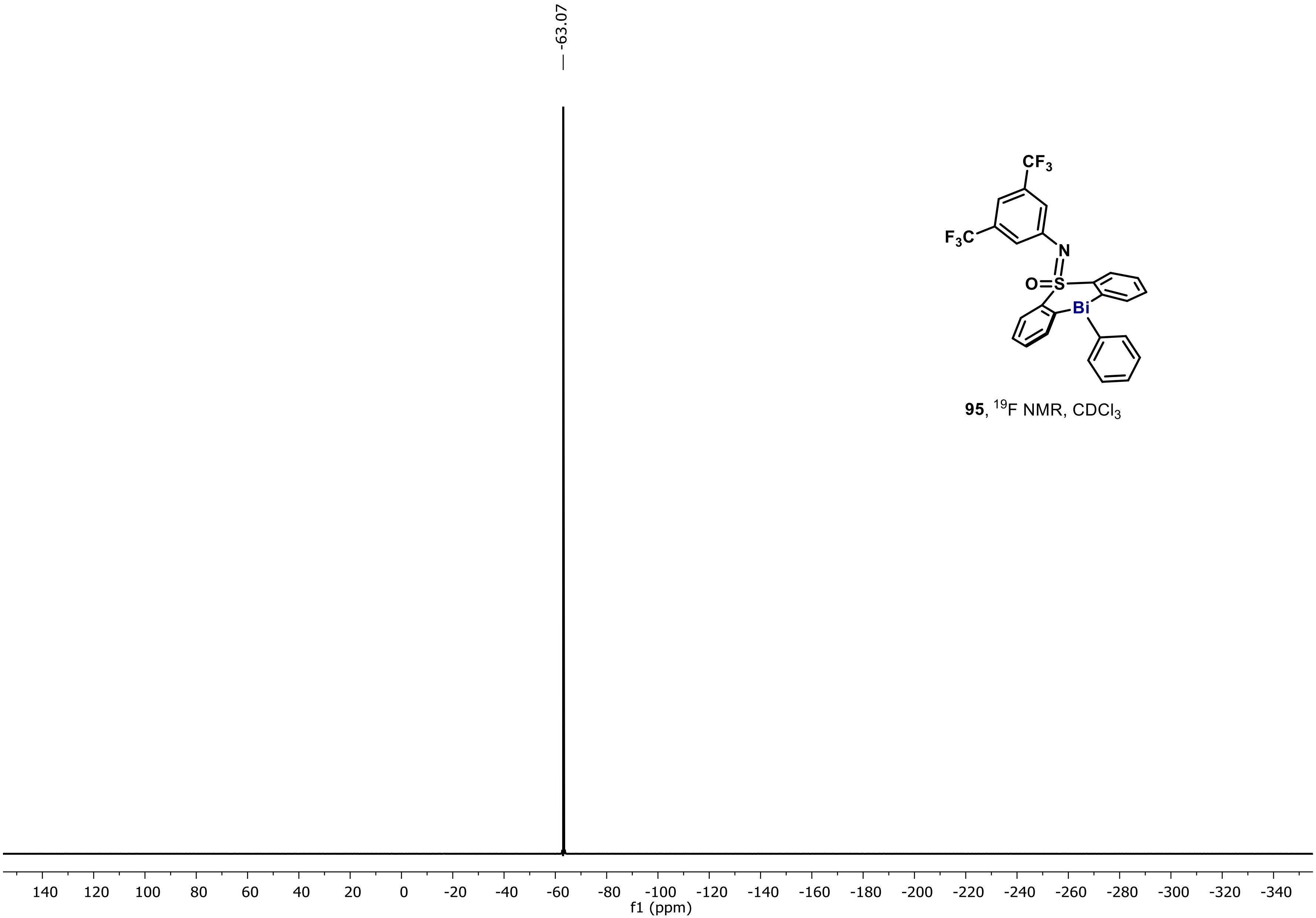

95, ^{19}F NMR, $CDCl_3$

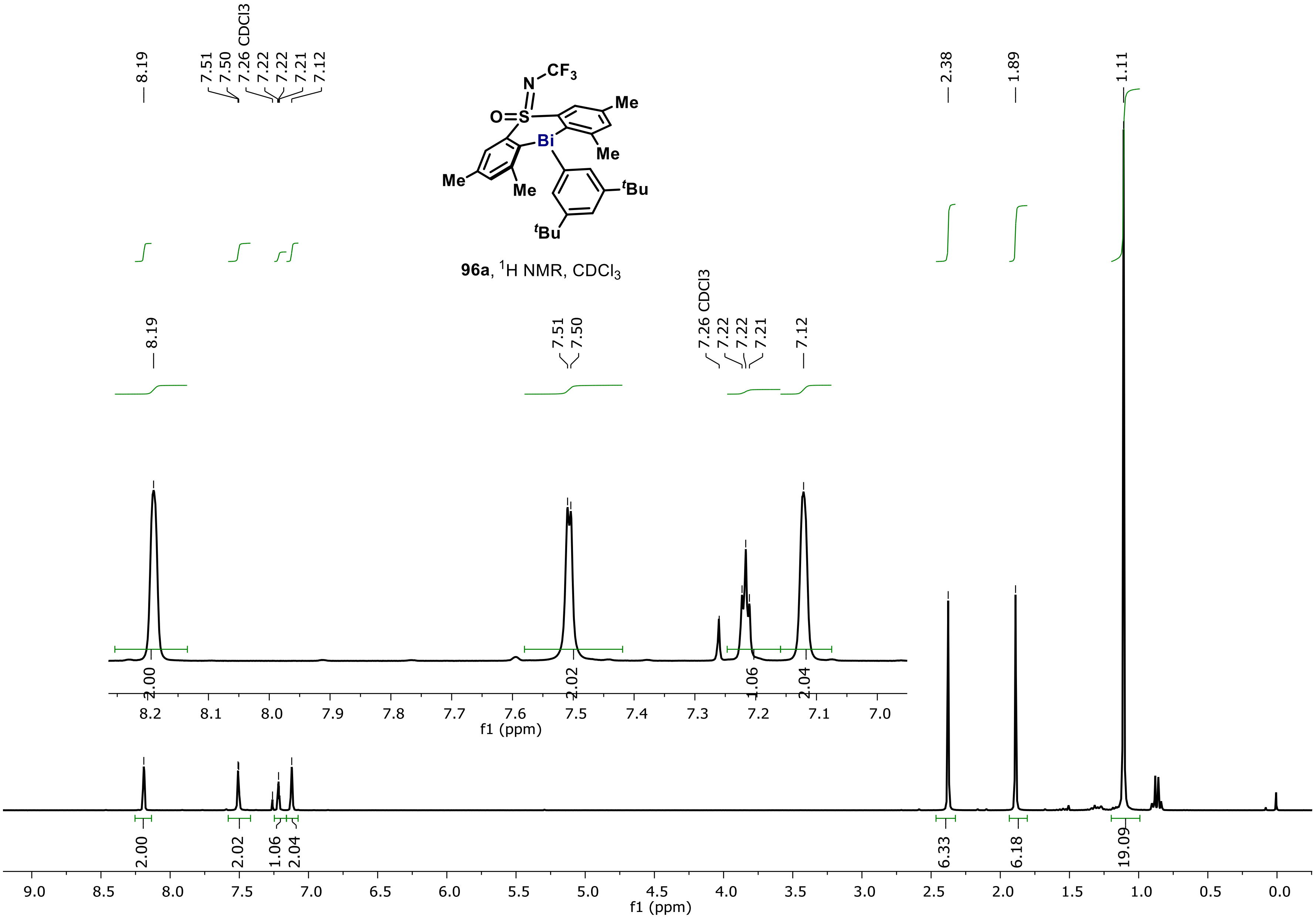
96a, ^{1}H NMR, $CDCl_3$
8.19
7.51
7.50
7.26 CDCl3
7.22
7.22
7.21
7.12
2.38
1.89
1.11
2.00
2.02
1.06
2.04
6.33
6.18
19.09
f1 (ppm)

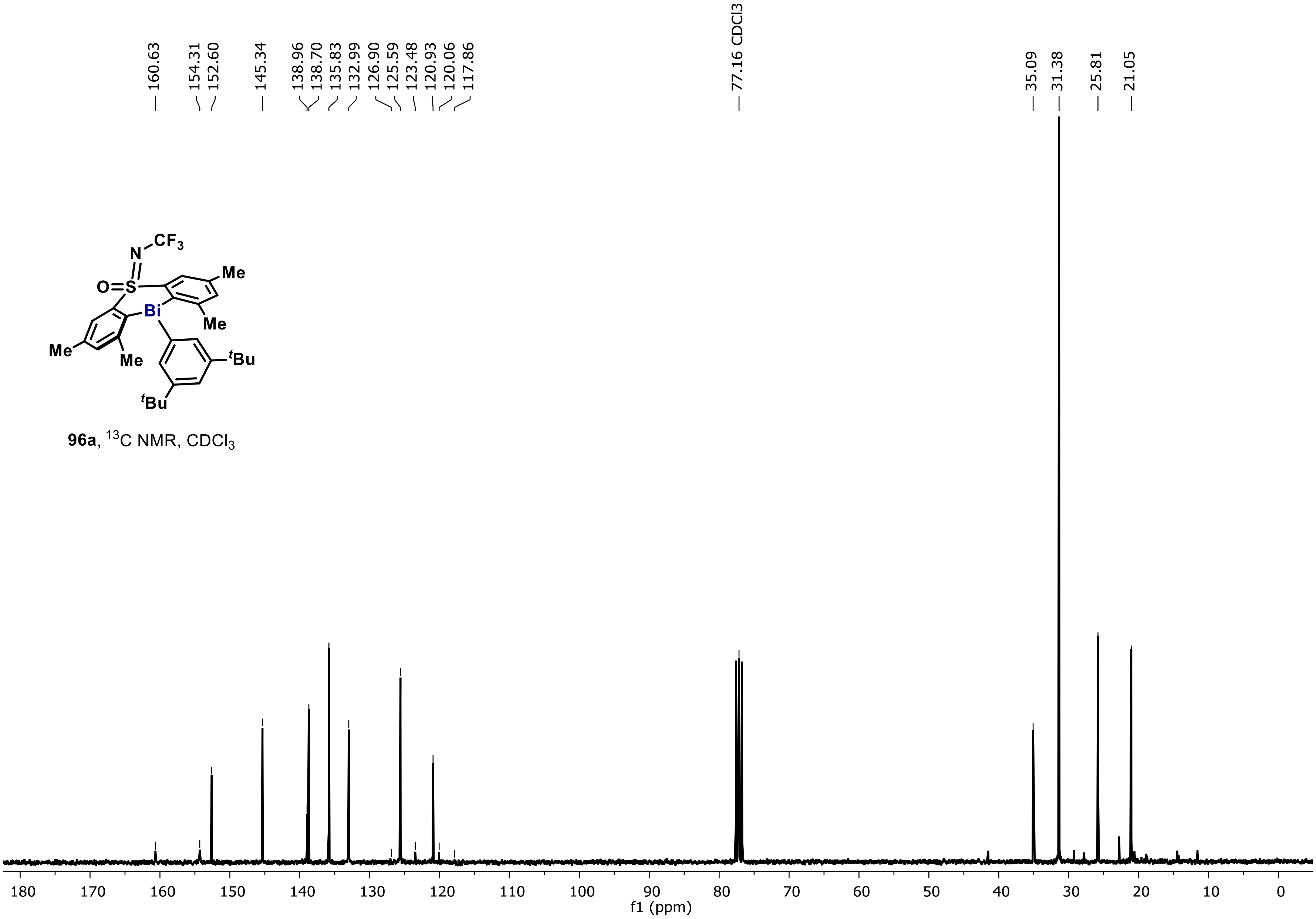

96a, ^{13}C NMR, $CDCl_3$

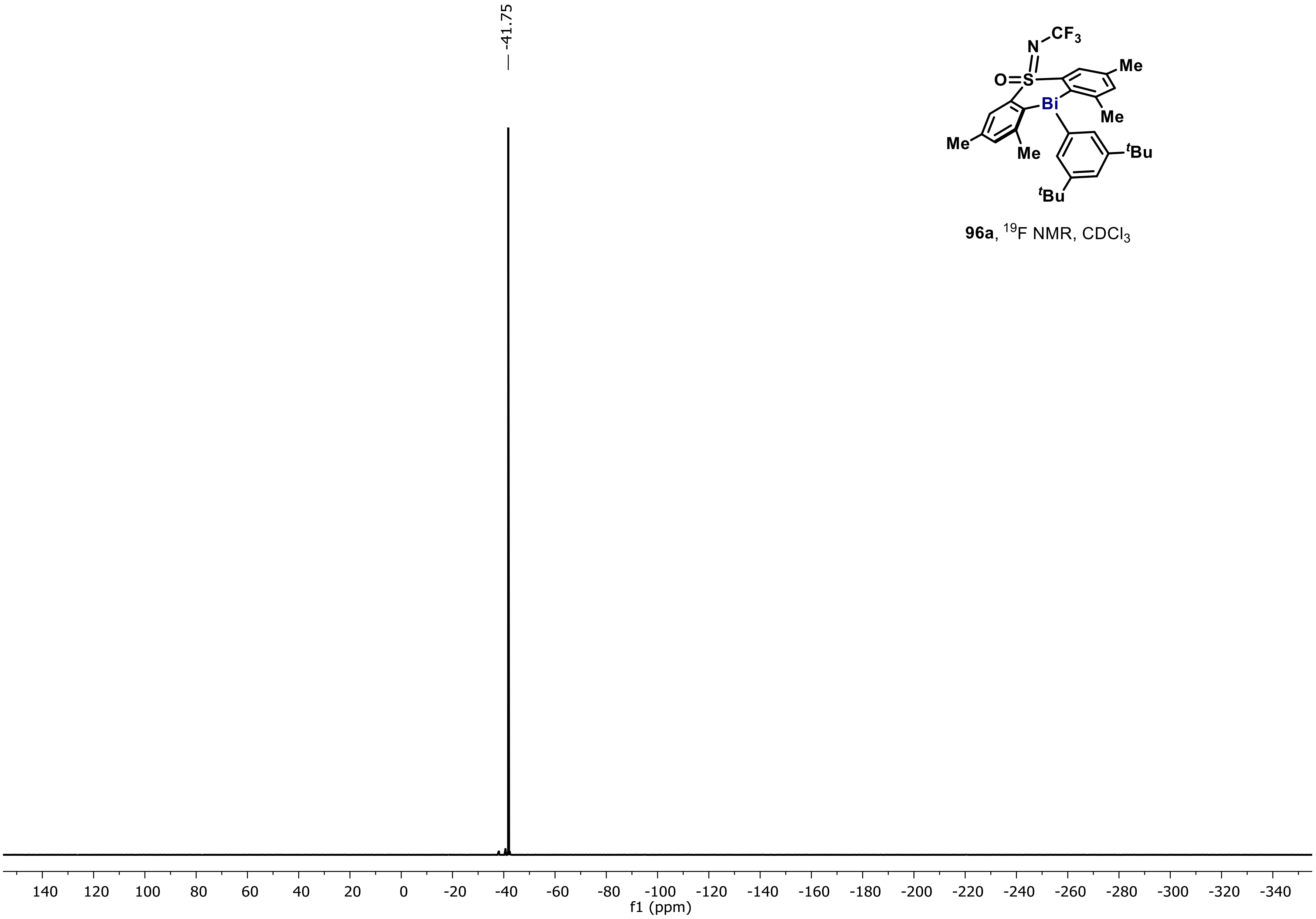

-41.75
N
CF3
O
S
Me
Bi
Me
Me
Me
tBu
tBu
96a, 19F NMR, CDCl3
140 120 100 80 60 40 20 0 -20 -40 -60 -80 -100 -120 -140 -160 -180 -200 -220 -240 -260 -280 -300 -320 -340
f1 (ppm)

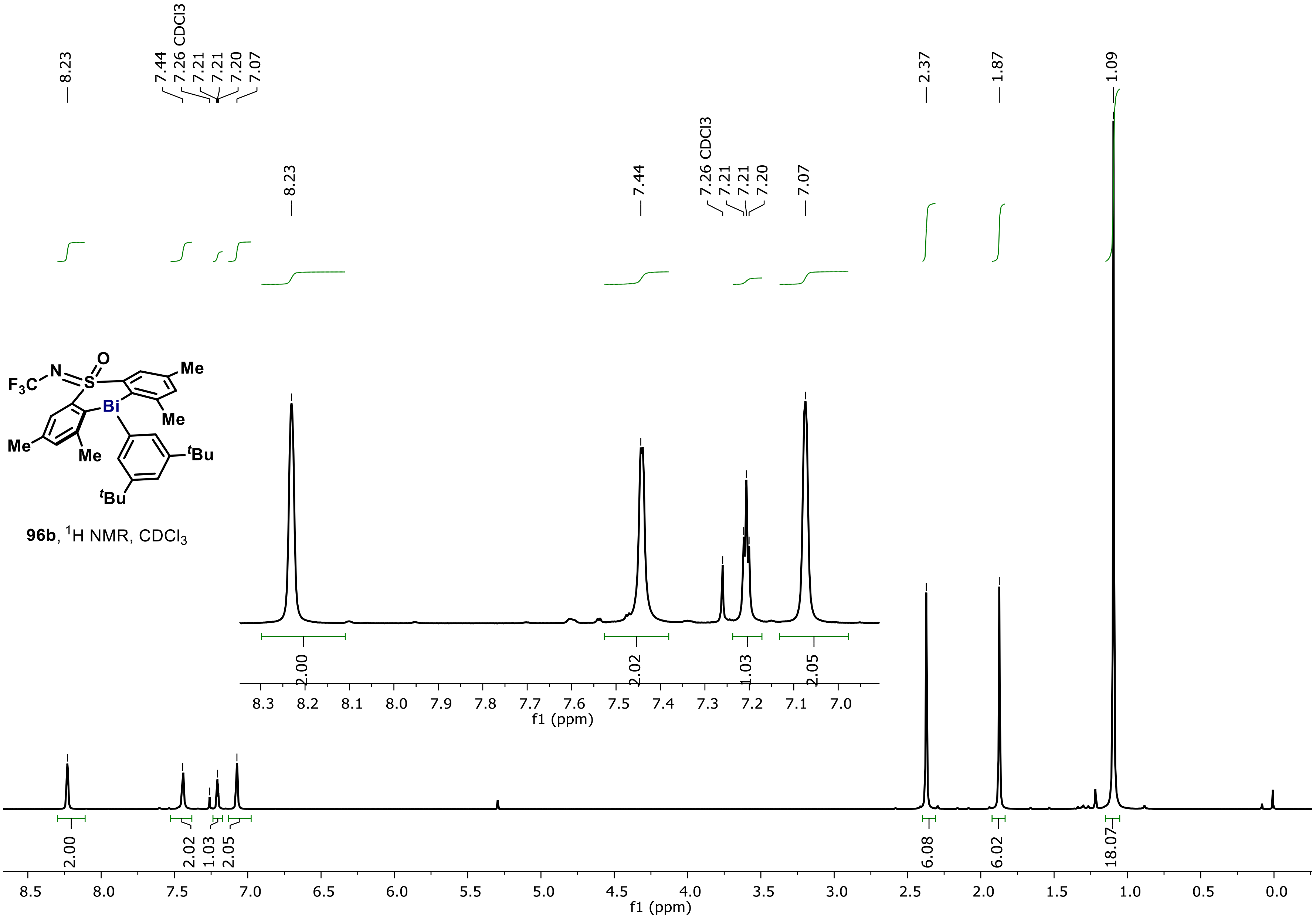

96b, ^{1}H NMR, $CDCl_3$

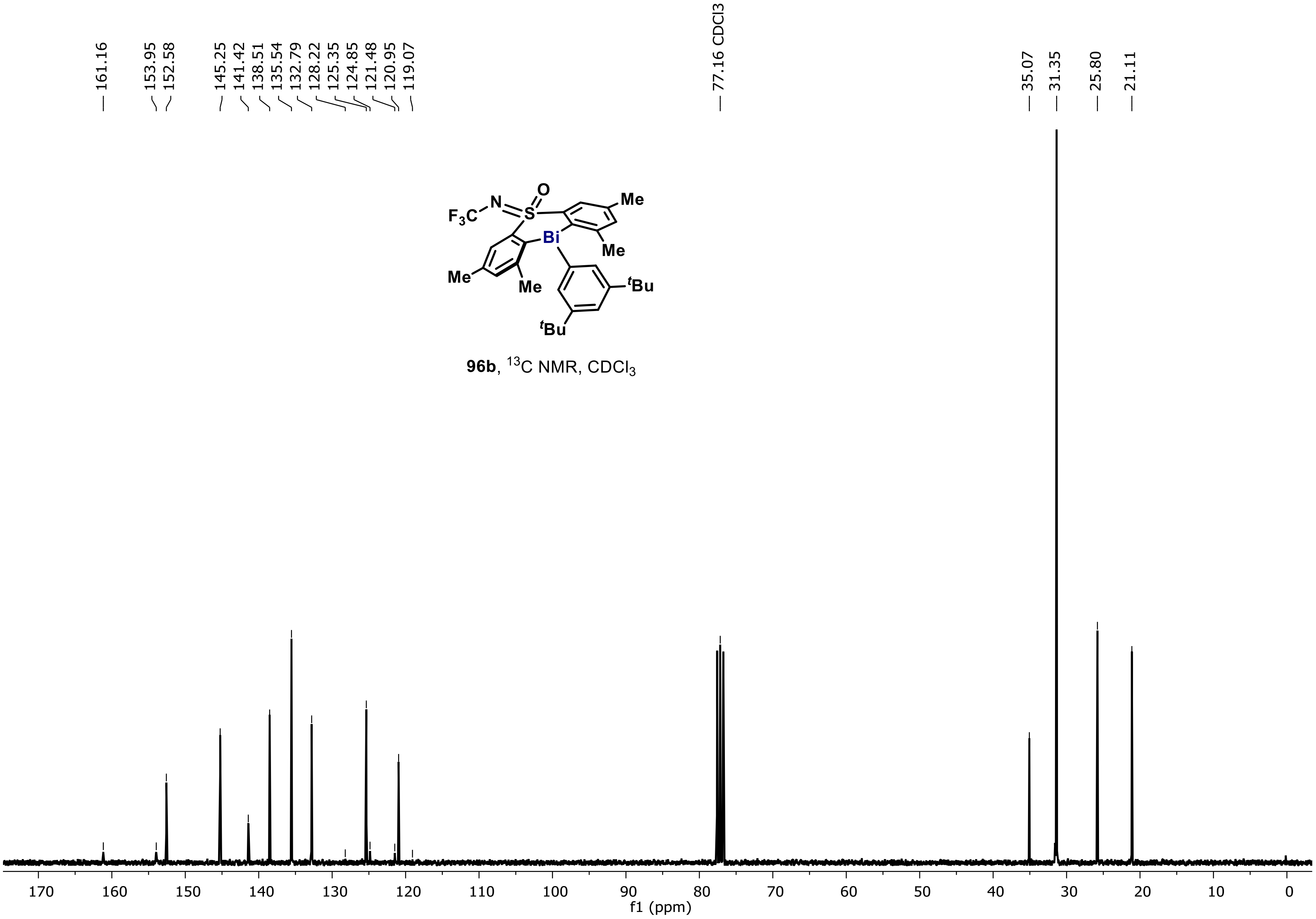
161.16
153.95
152.58
145.25
141.42
138.51
135.54
132.79
128.22
125.35
124.85
121.48
120.95
119.07
77.16 CDCl3
35.07
31.35
25.80
21.11
F3C
N
S
O
Bi
Me
Me
Me
Me
tBu
tBu
96b, 13C NMR, CDCl3
170 160 150 140 130 120 110 100 90 80 70 60 50 40 30 20 10 0
f1 (ppm)

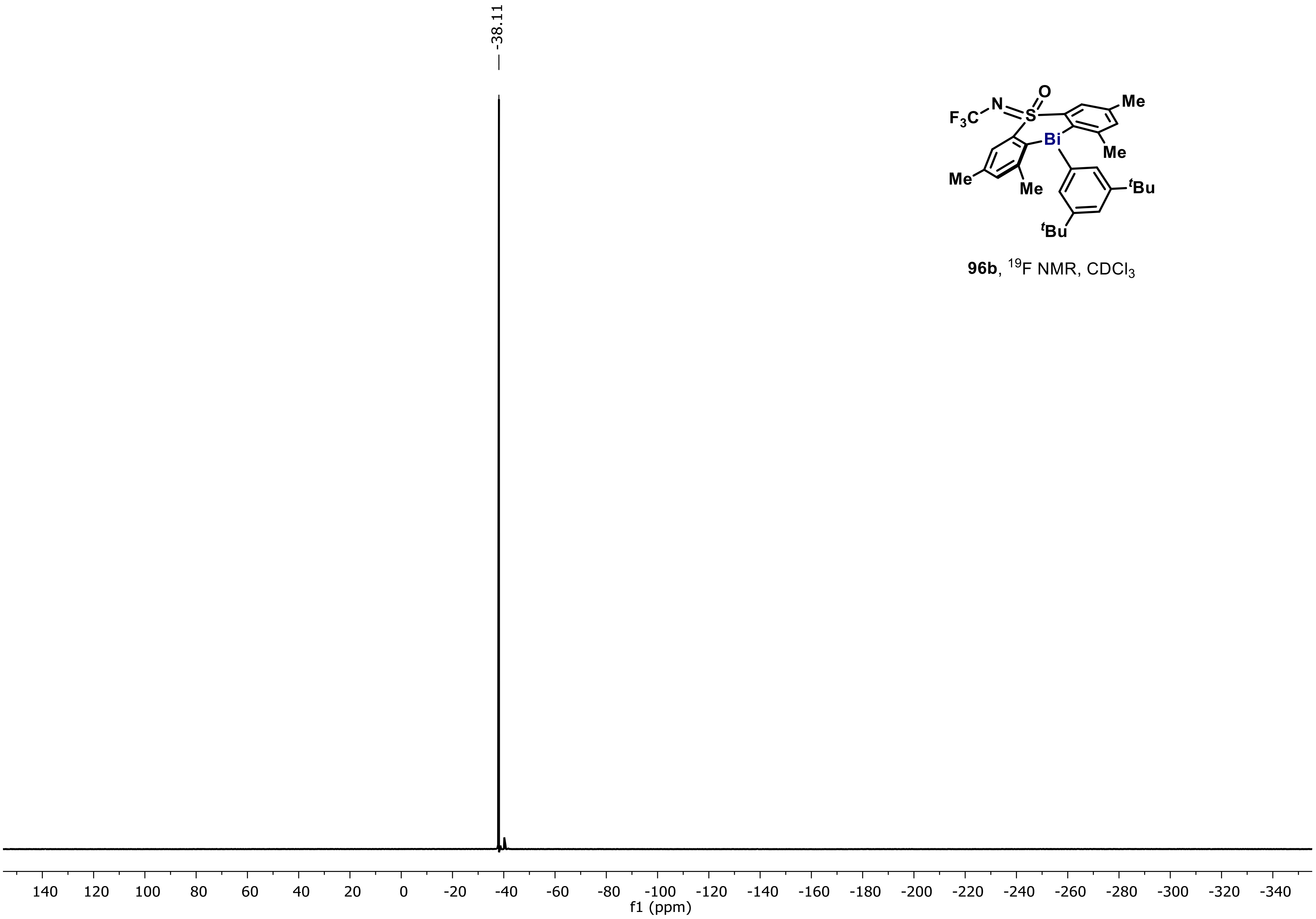

96b, ^{19}F NMR, $CDCl_3$

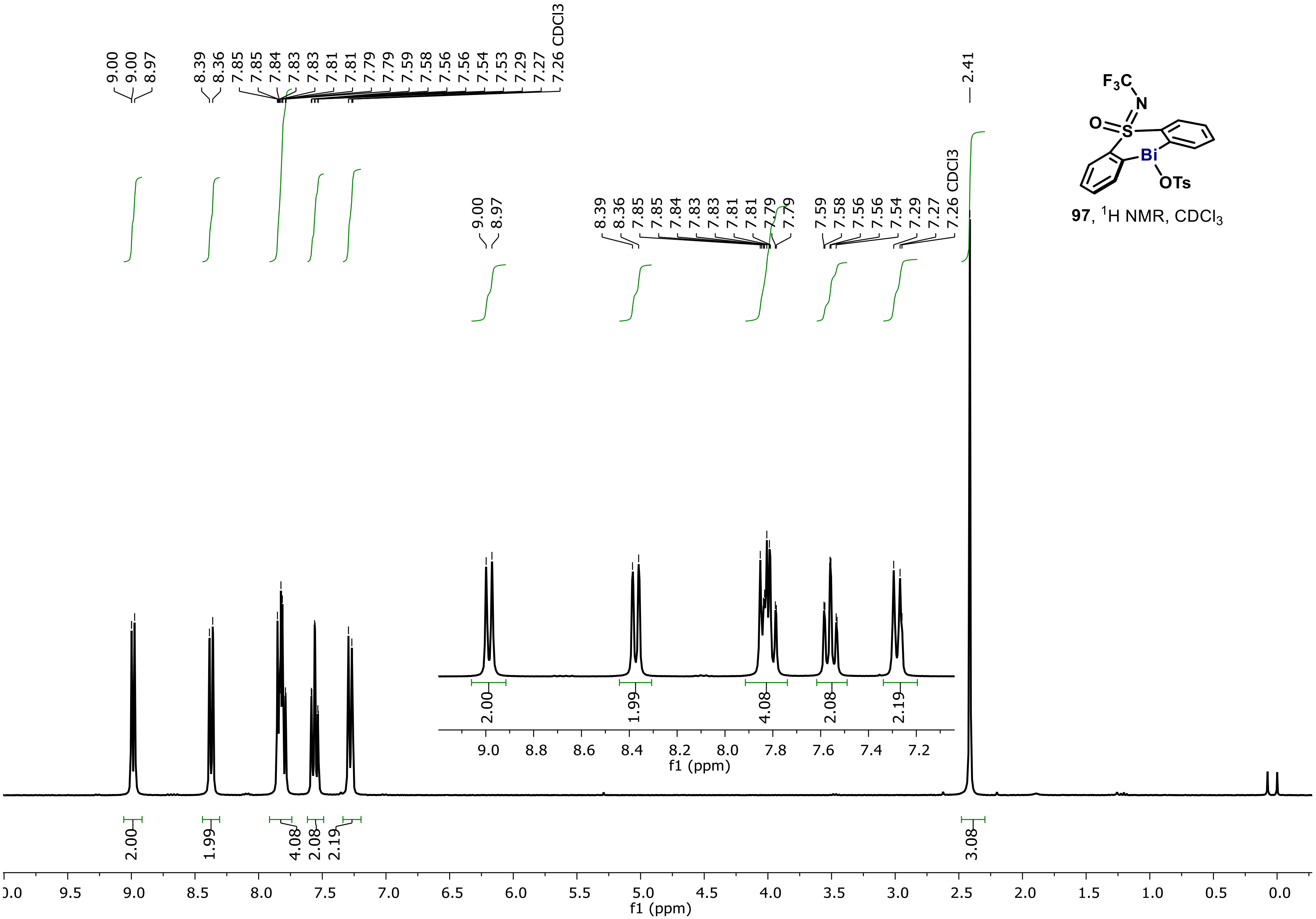

97, ^{1}H NMR, $CDCl_3$

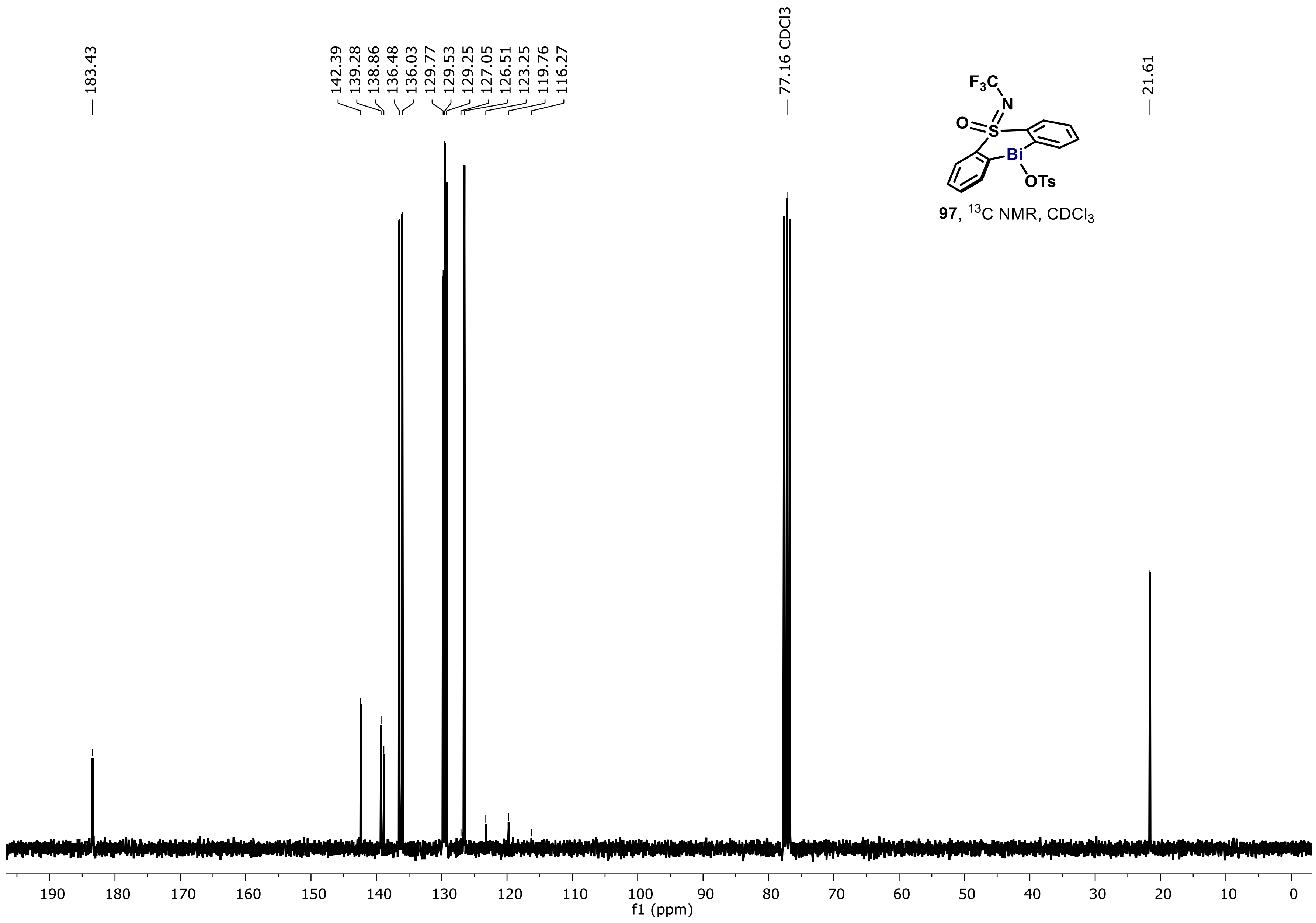

183.43
142.39
139.28
138.86
136.48
136.03
129.77
129.53
129.25
127.05
126.51
123.25
119.76
116.27
77.16 CDCl3
21.61
F3C
N
O
S
Bi
OTs
97, 13C NMR, CDCl3
190 180 170 160 150 140 130 120 110 100 90 80 70 60 50 40 30 20 10 0
f1 (ppm)

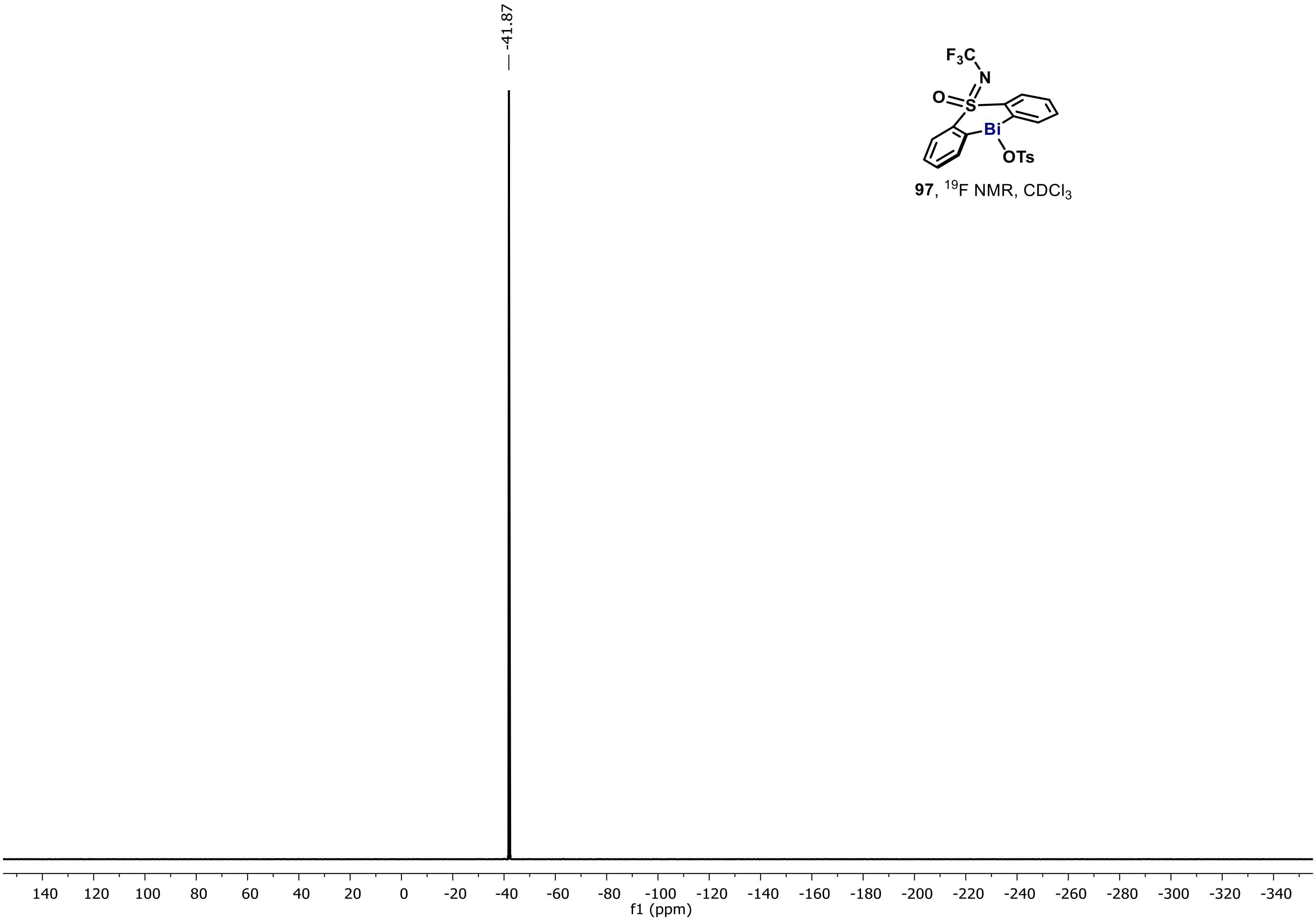

97, ^{19}F NMR, $CDCl_3$

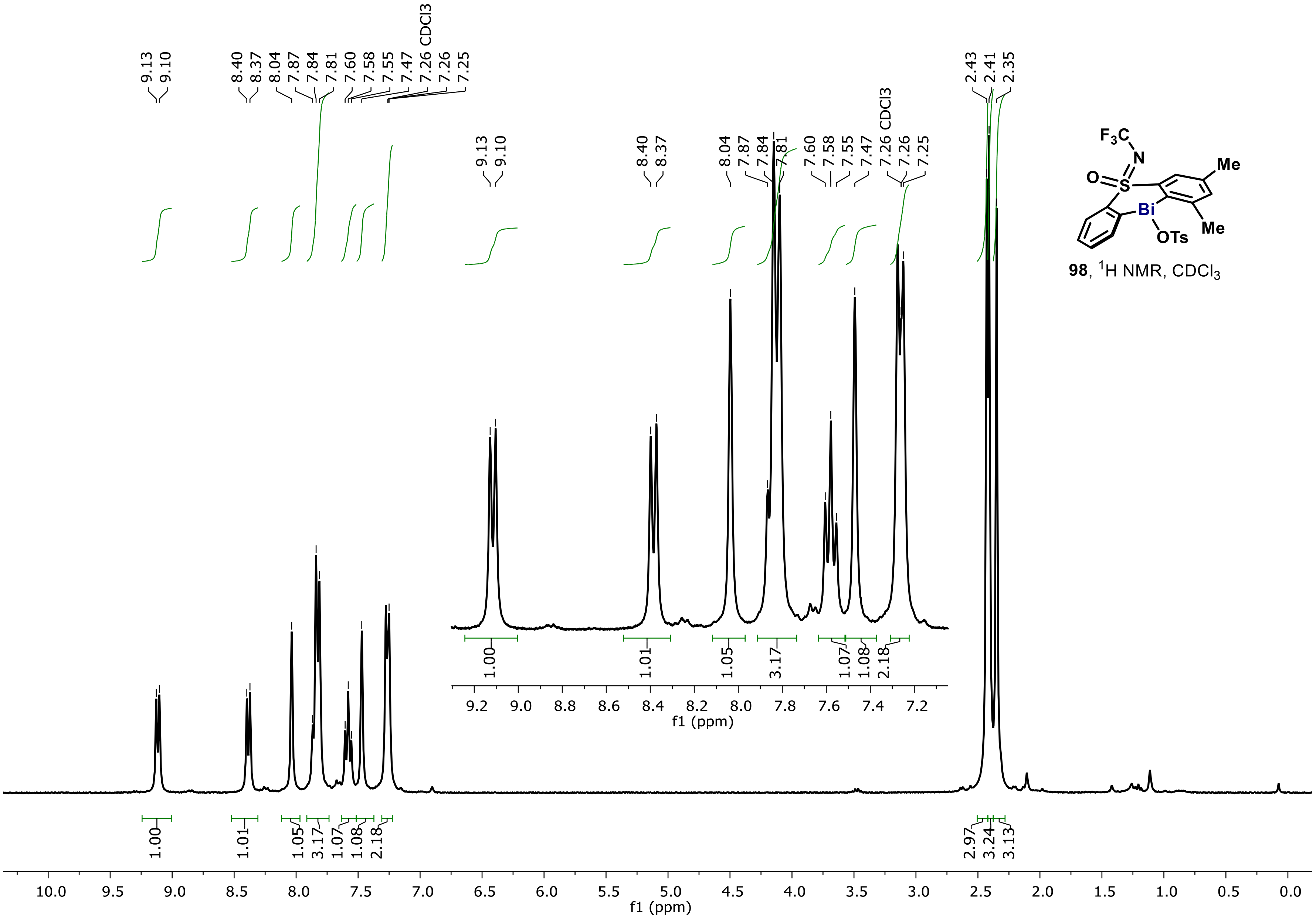
98, ^{1}H NMR, CDCl$_3$
f1 (ppm)

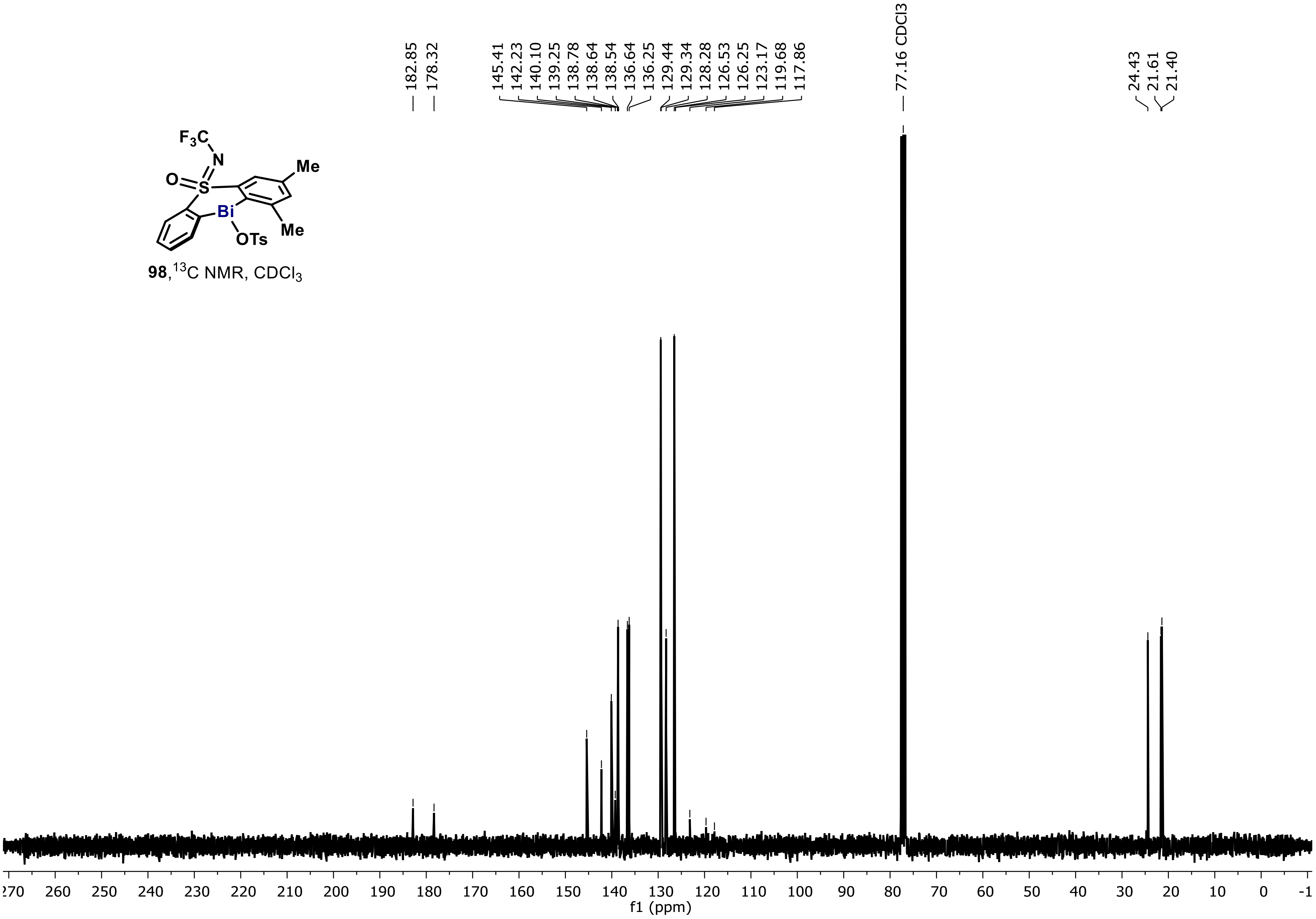

F3C
N
O
S
Me
Bi
Me
OTs
98, 13C NMR, CDCl3
182.85
178.32
145.41
142.23
140.10
139.25
138.78
138.64
138.54
136.64
136.25
129.44
129.34
128.28
126.53
126.25
123.17
119.68
117.86
77.16 CDCl3
24.43
21.61
21.40
270 260 250 240 230 220 210 200 190 180 170 160 150 140 130 120 110 100 90 80 70 60 50 40 30 20 10 0 -1
f1 (ppm)

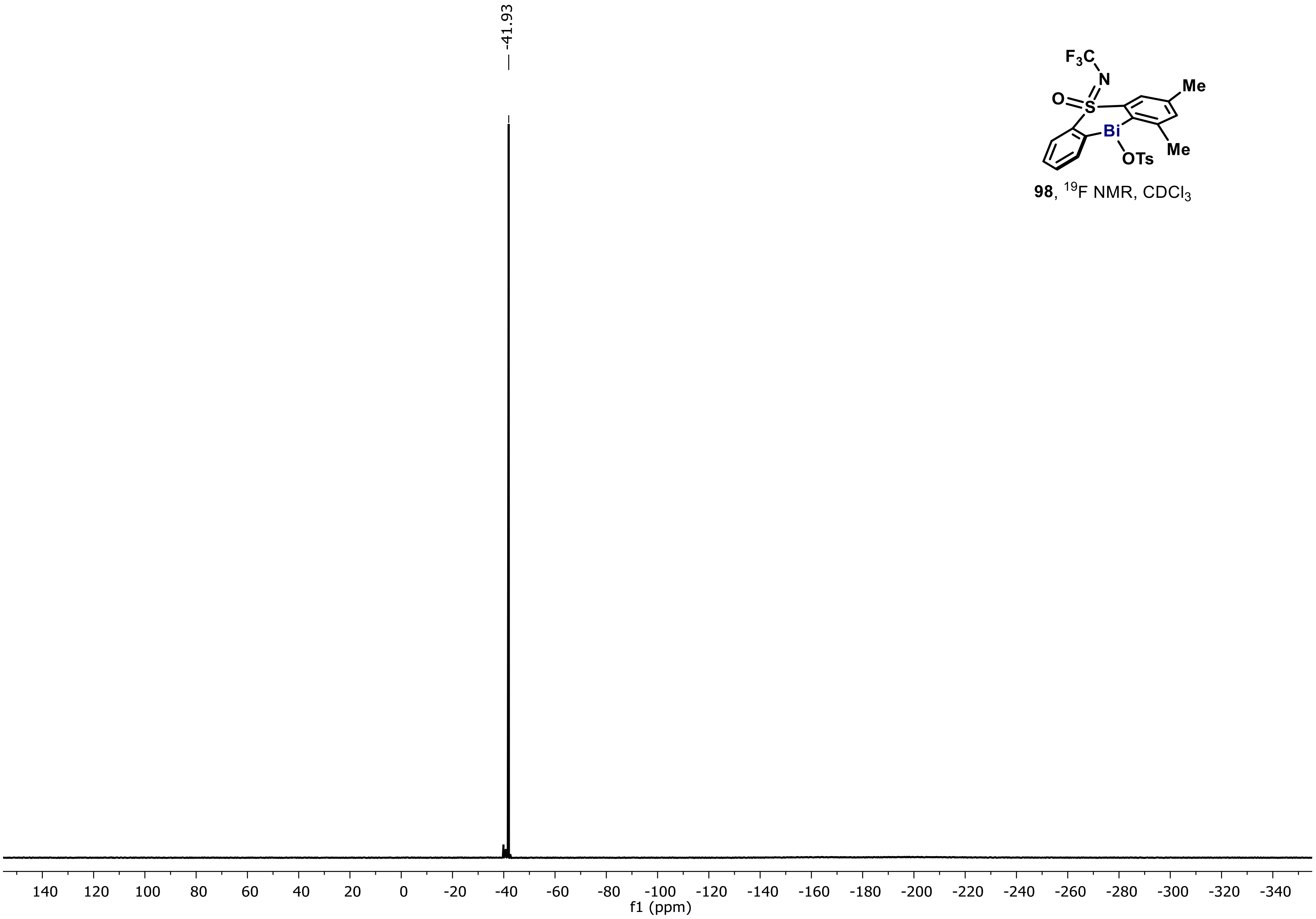

98, ^{19}F NMR, $CDCl_3$

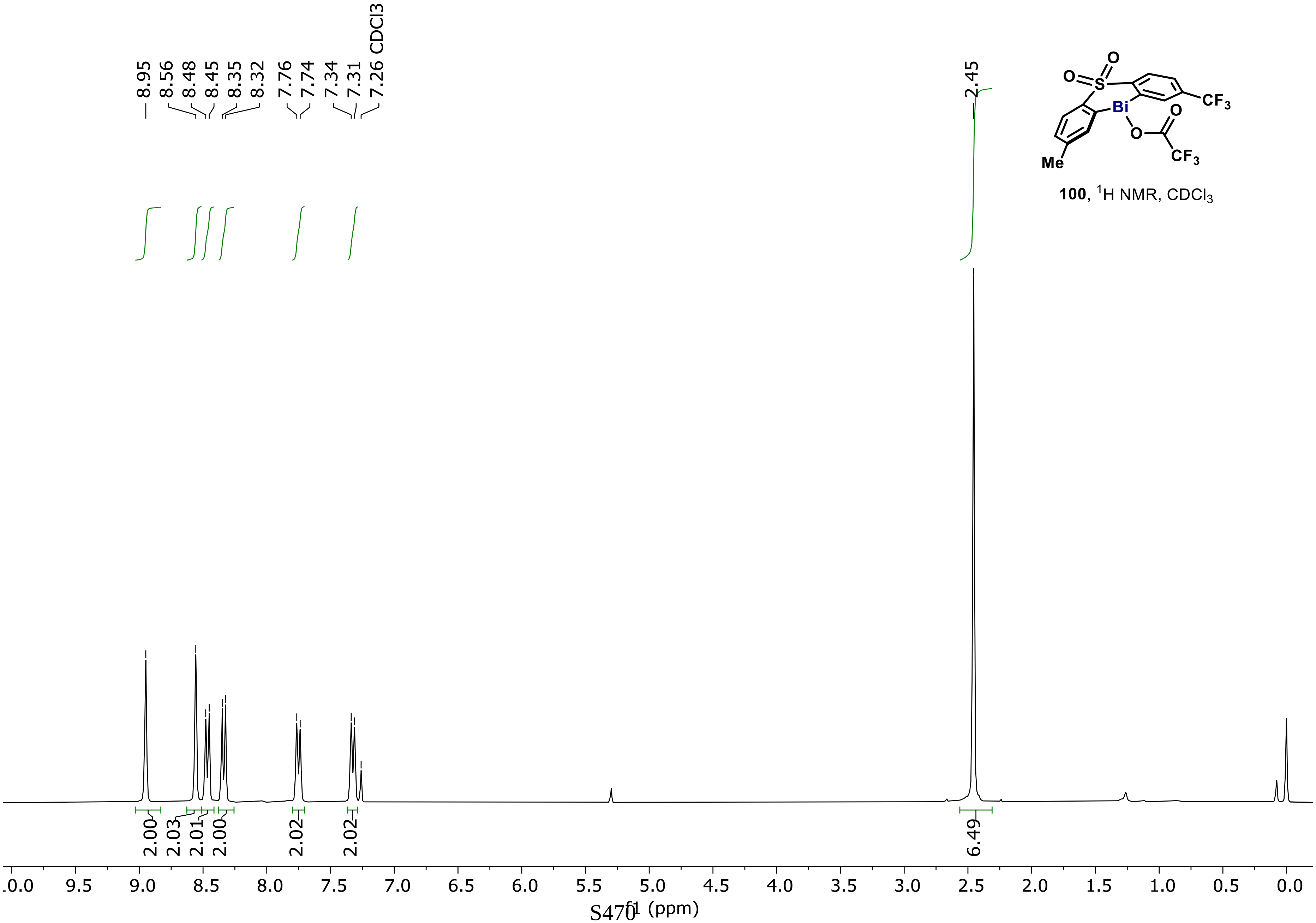

100, ^{1}H NMR, $CDCl_3$

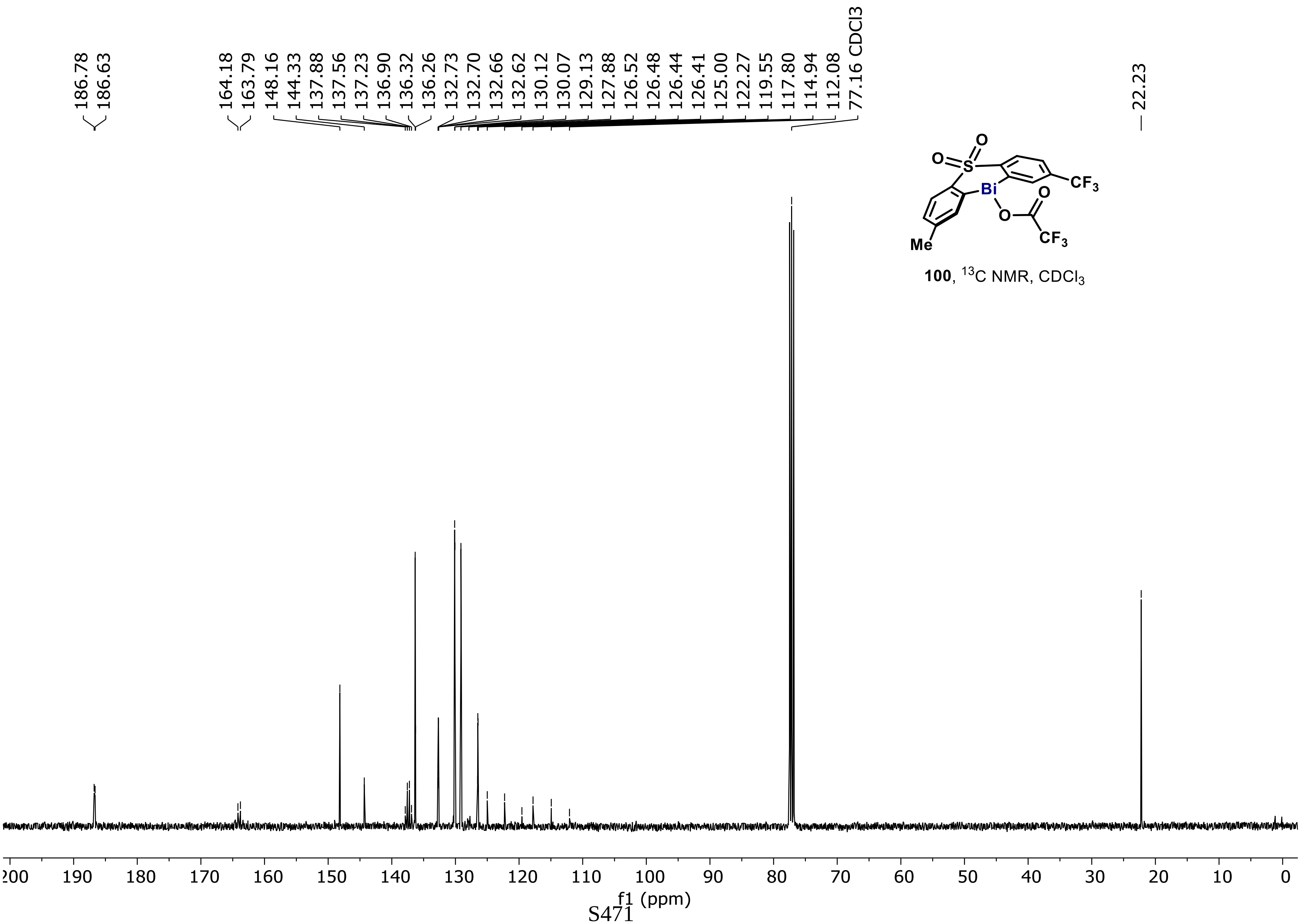

100, ^{13}C NMR, $CDCl_3$

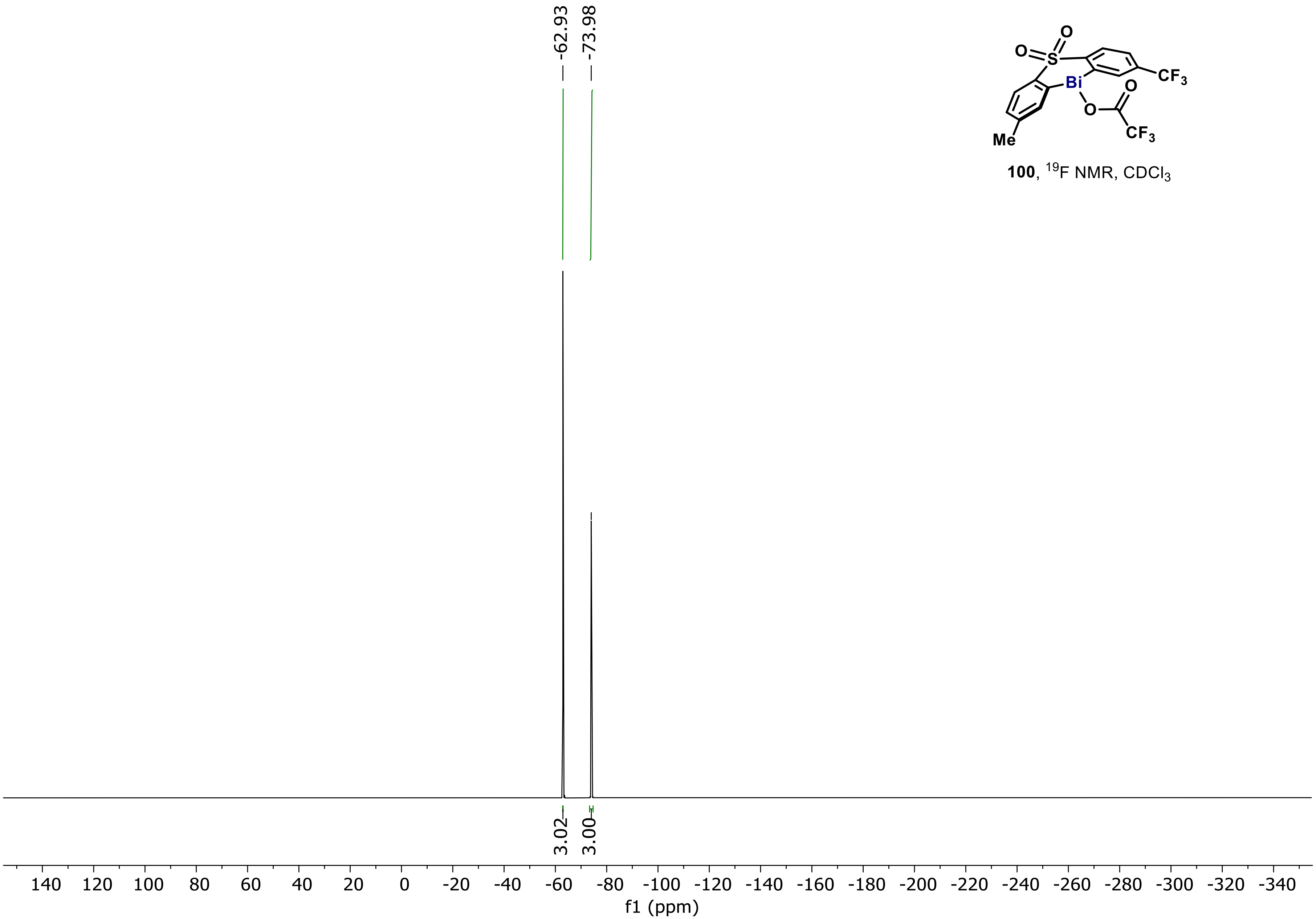

100, ^{19}F NMR, $CDCl_3$

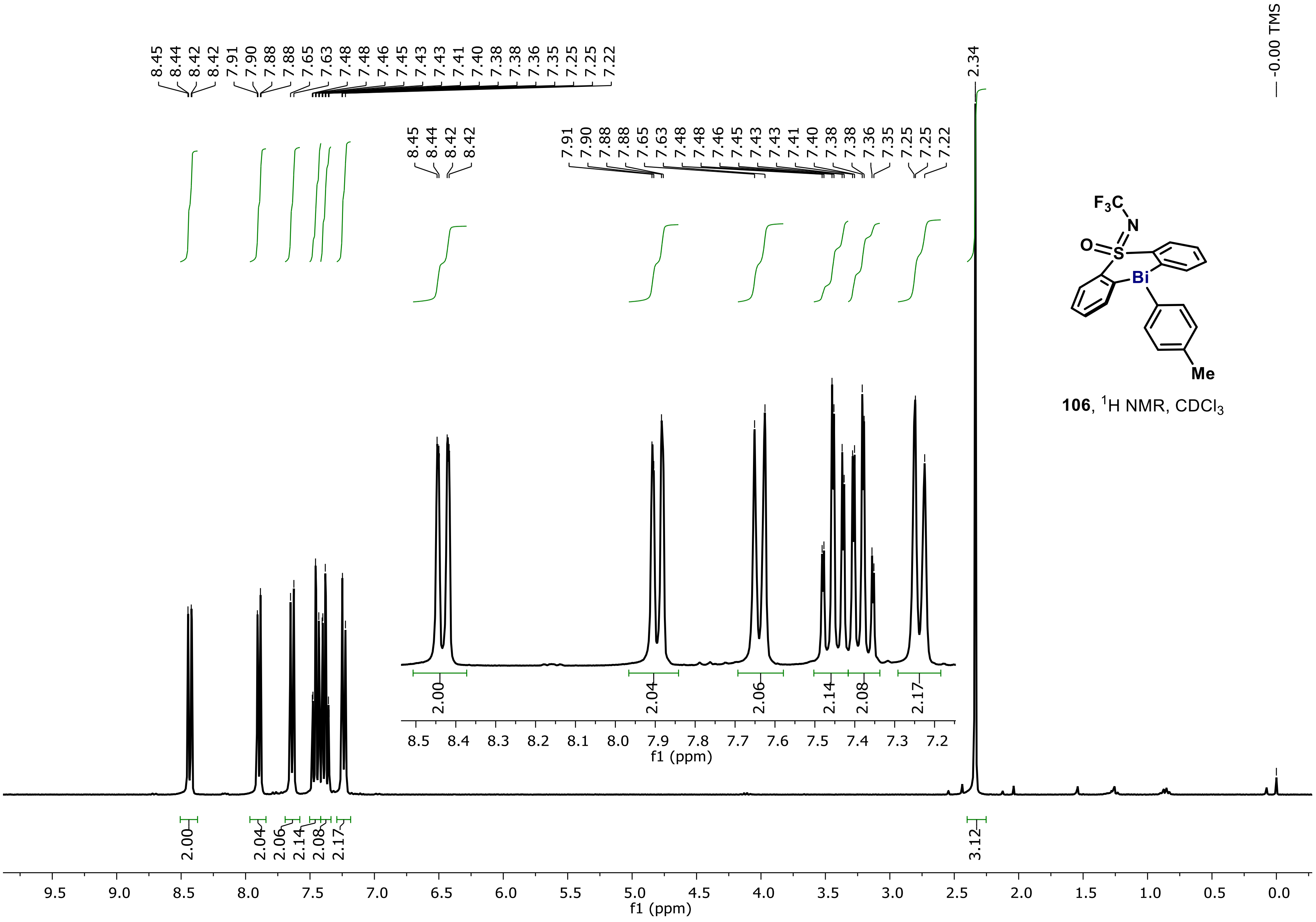

106, ^{1}H NMR, $CDCl_3$

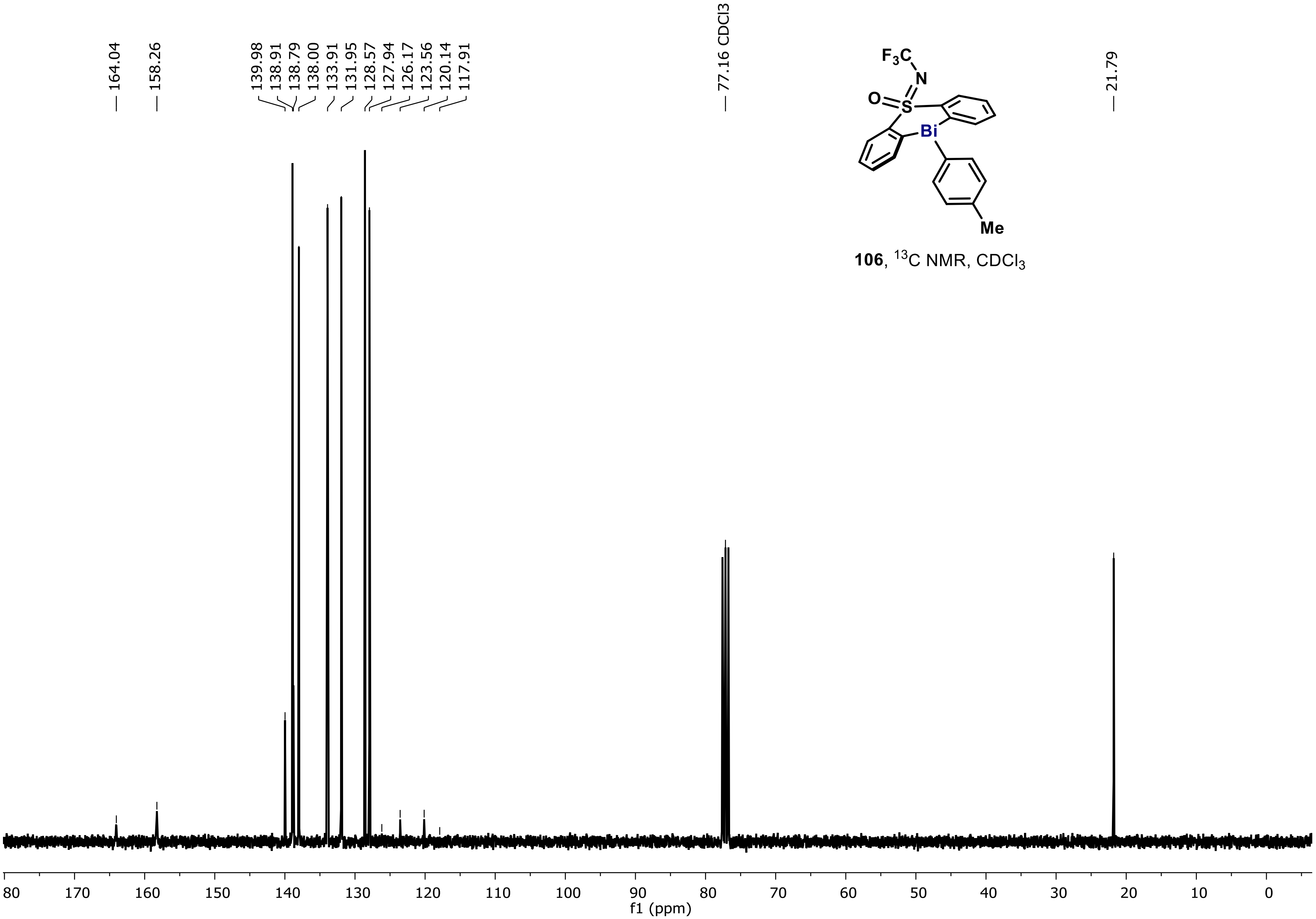
164.04
158.26
139.98
138.91
138.79
138.00
133.91
131.95
128.57
127.94
126.17
123.56
120.14
117.91
77.16 CDCl3
21.79
F3C
N
O
S
Bi
Me
106, 13C NMR, CDCl3
80
170
160
150
140
130
120
110
100
90
80
70
60
50
40
30
20
10
0
f1 (ppm)

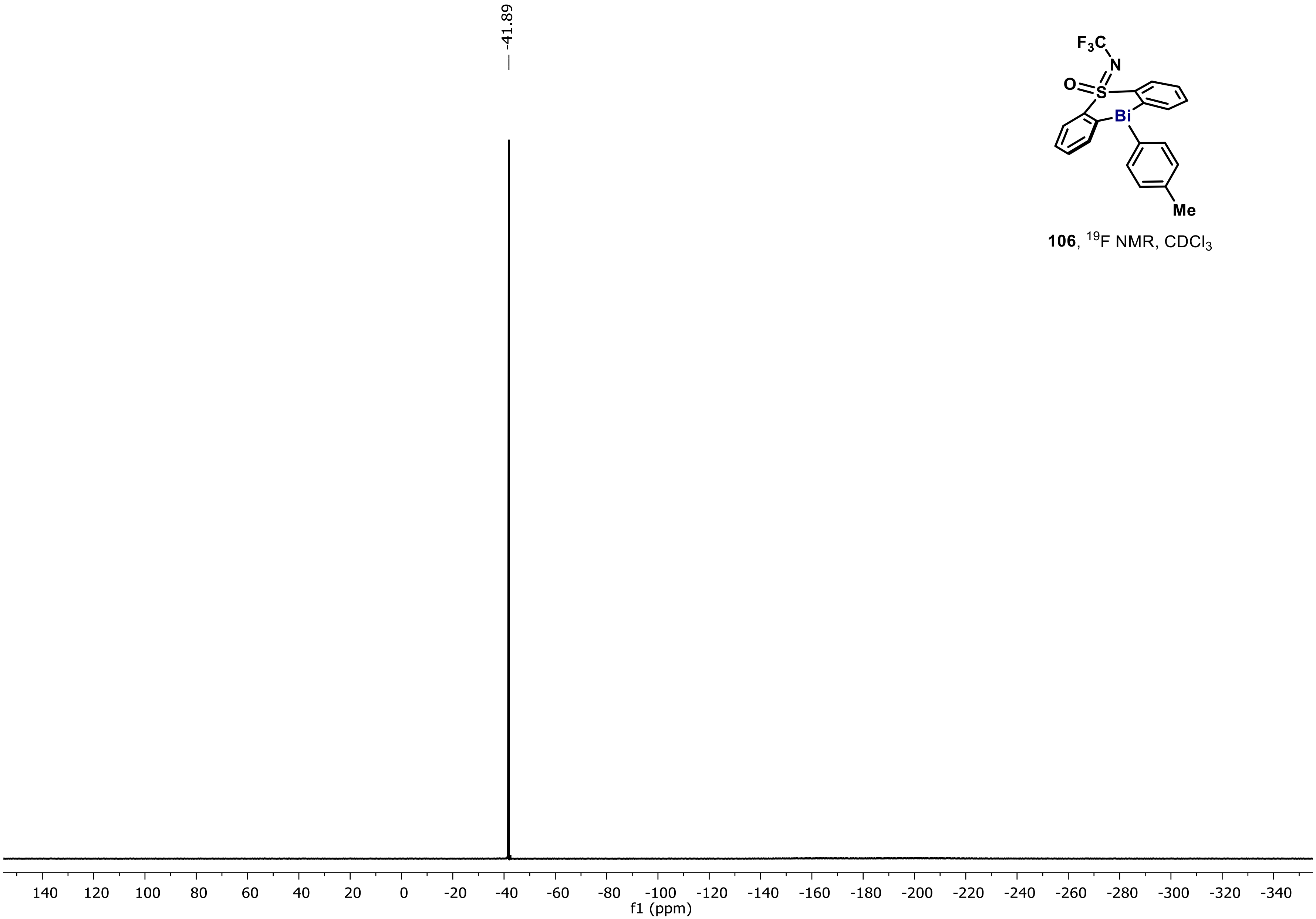

106, ^{19}F NMR, $CDCl_3$

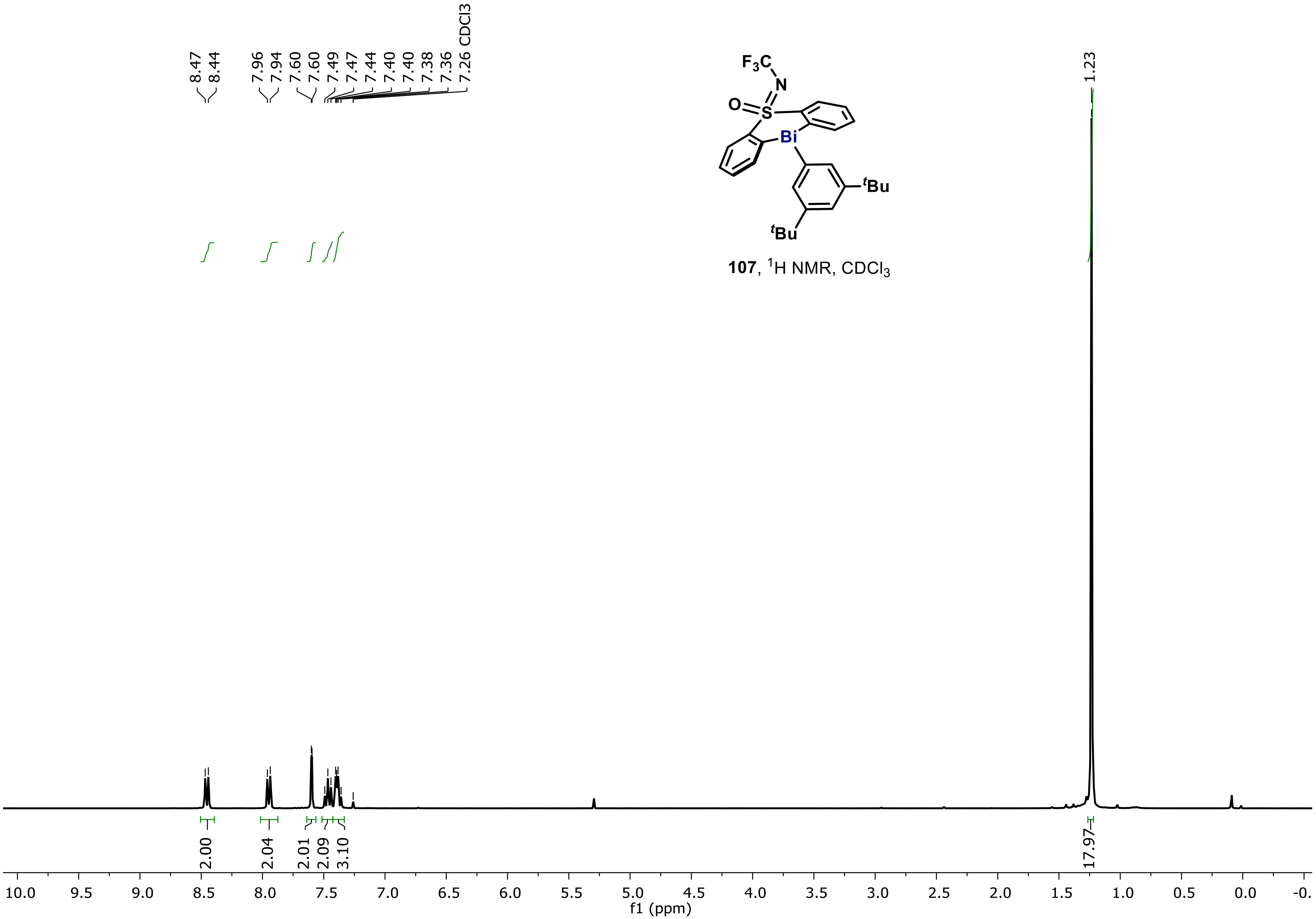

107, ^{1}H NMR, $CDCl_3$

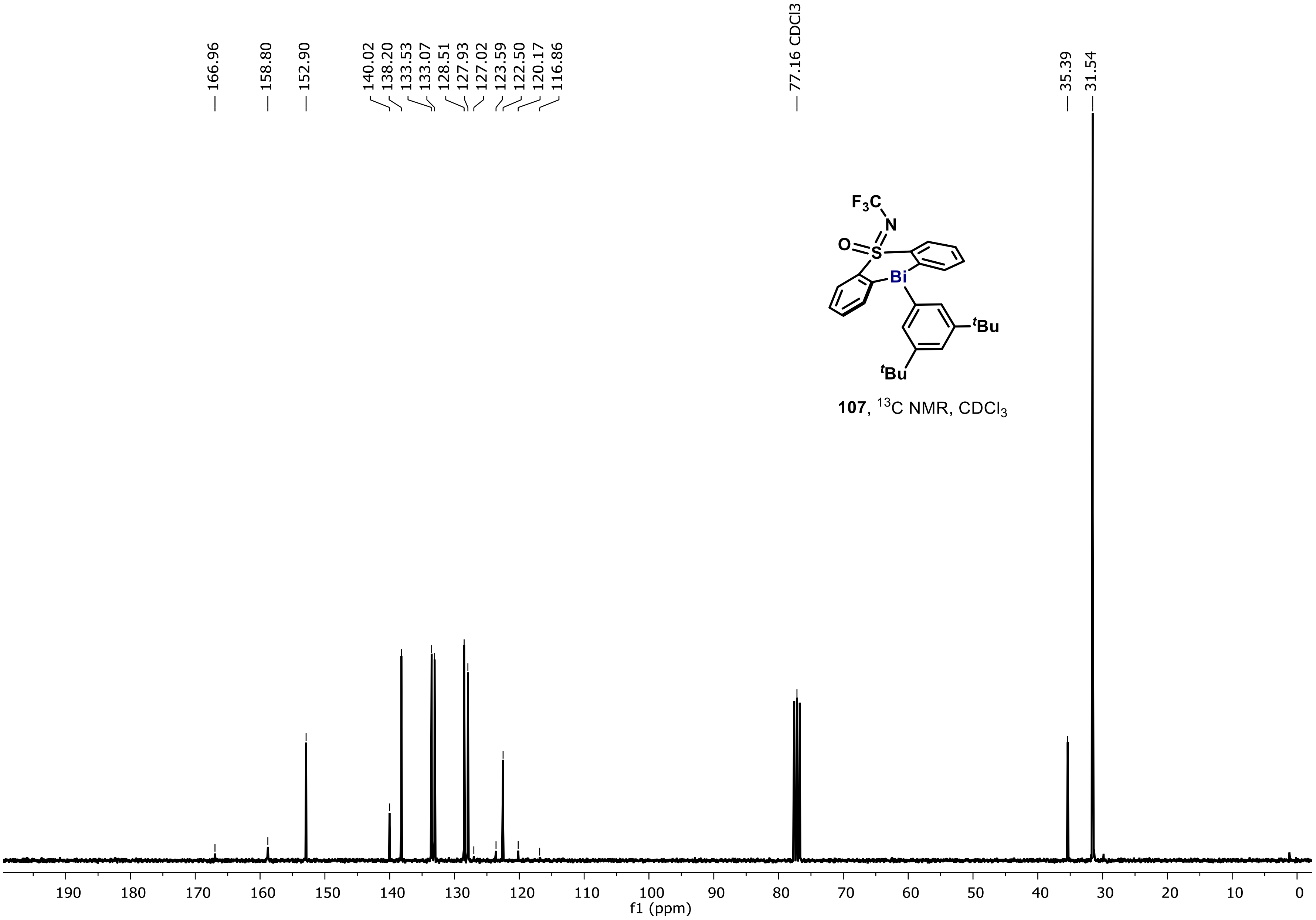
166.96
158.80
152.90
140.02
138.20
133.53
133.07
128.51
127.93
127.02
123.59
122.50
120.17
116.86
77.16 CDCl3
35.39
31.54
F3C
N
O
S
Bi
tBu
tBu
107, 13C NMR, CDCl3
190 180 170 160 150 140 130 120 110 100 90 80 70 60 50 40 30 20 10 0
f1 (ppm)

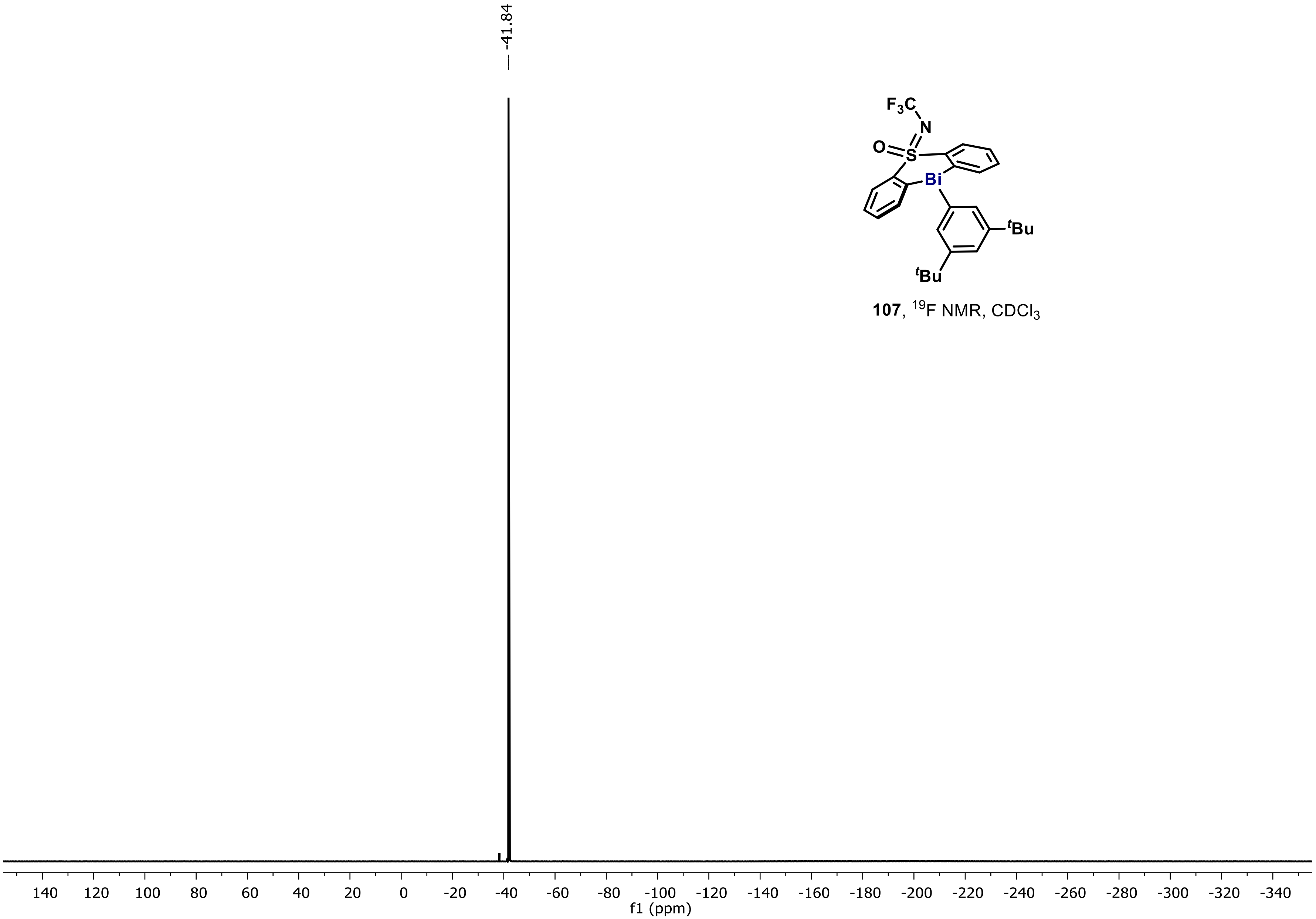

107, ^{19}F NMR, $CDCl_3$

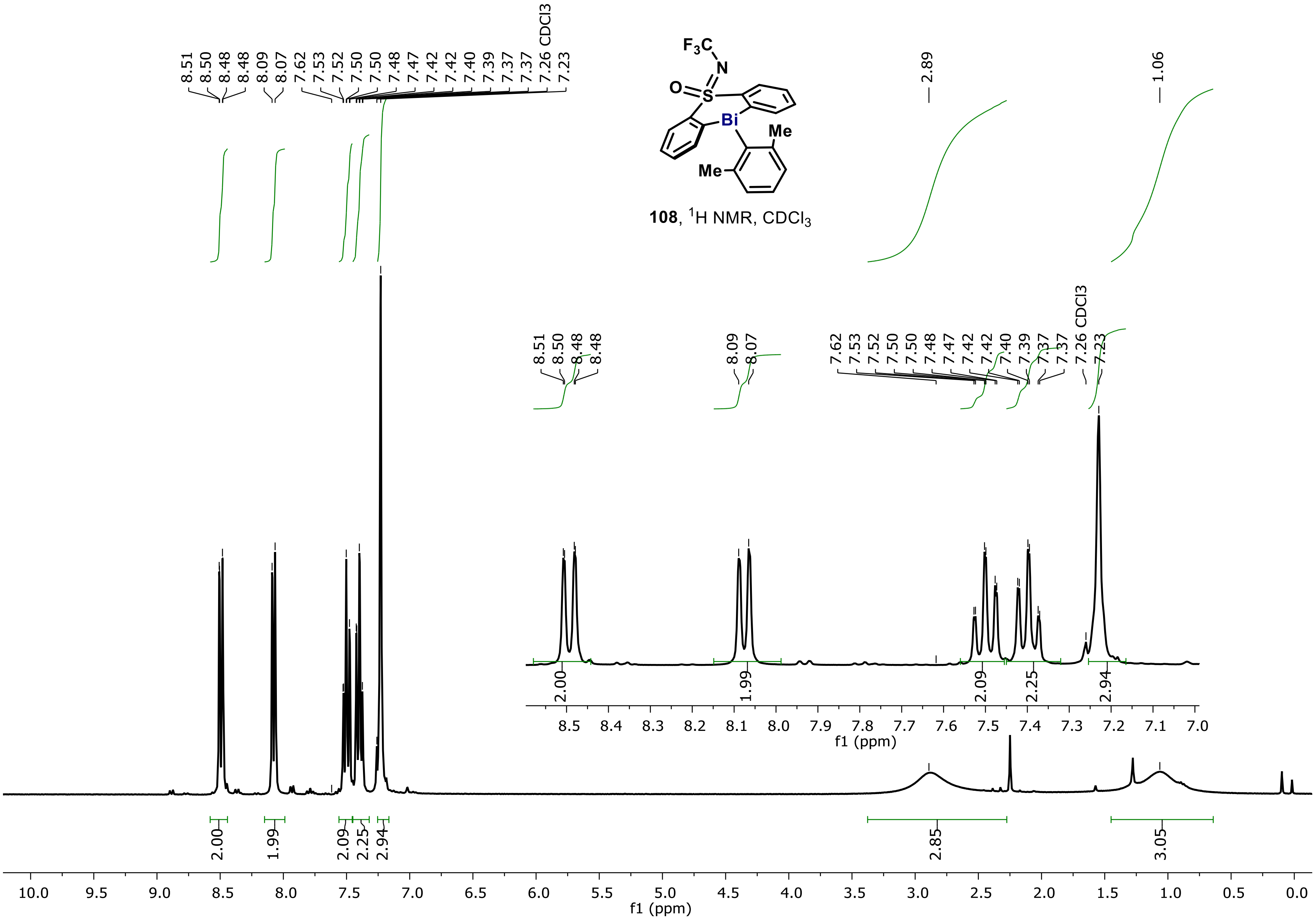

F3C
N
O
S
Bi
Me
Me
108, 1H NMR, CDCl3
8.51
8.50
8.48
8.48
8.09
8.07
7.62
7.53
7.52
7.50
7.50
7.48
7.47
7.42
7.42
7.40
7.39
7.37
7.37
7.26 CDCl3
7.23
2.89
1.06
2.00
1.99
2.09
2.25
2.94
2.85
3.05
f1 (ppm)

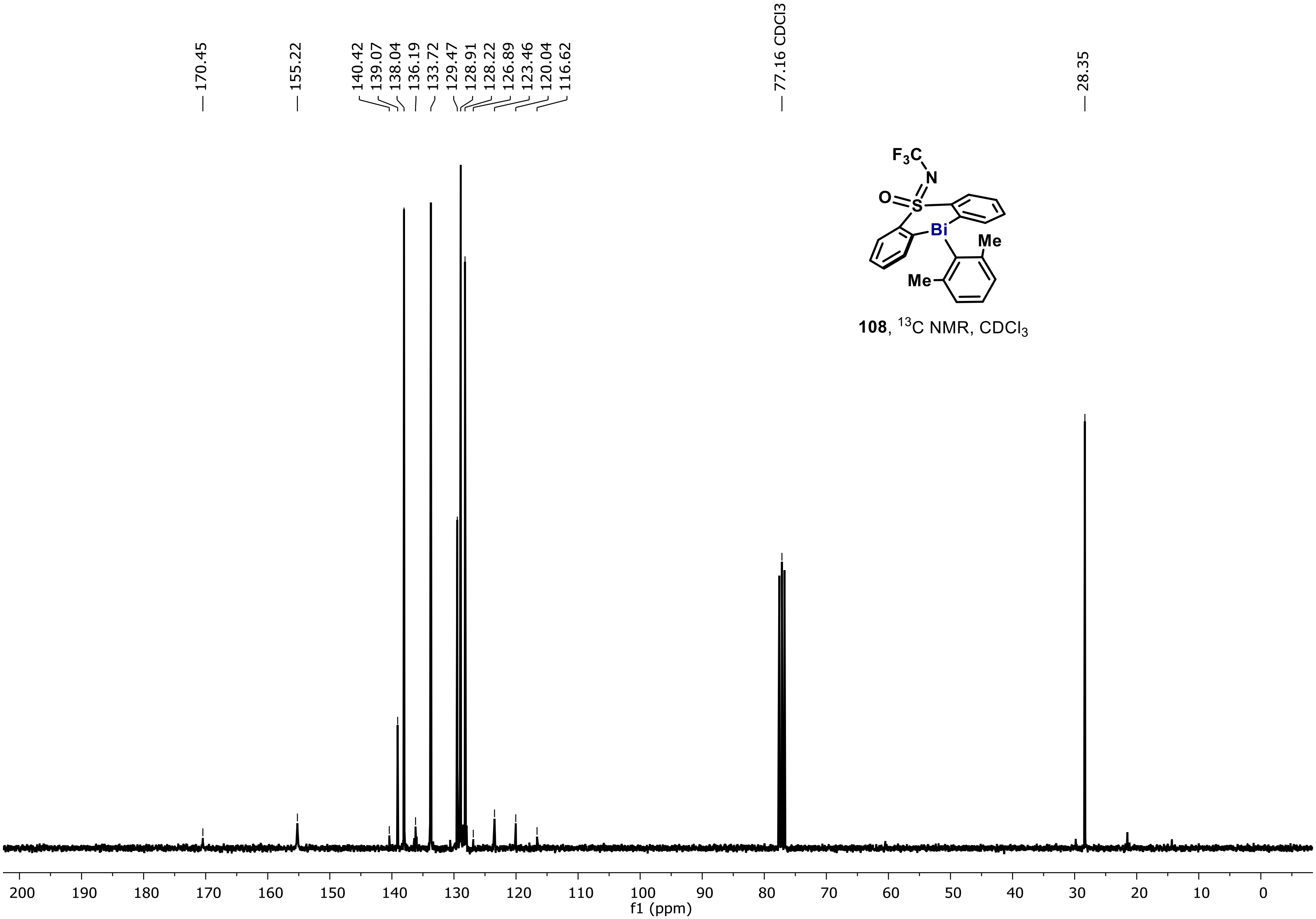
170.45
155.22
140.42
139.07
138.04
136.19
133.72
129.47
128.91
128.22
126.89
123.46
120.04
116.62
77.16 CDCl3
28.35
F_3C
N
O
S
Bi
Me
Me
108, ^{13}C NMR, $CDCl_3$
200 190 180 170 160 150 140 130 120 110 100 90 80 70 60 50 40 30 20 10 0
f1 (ppm)

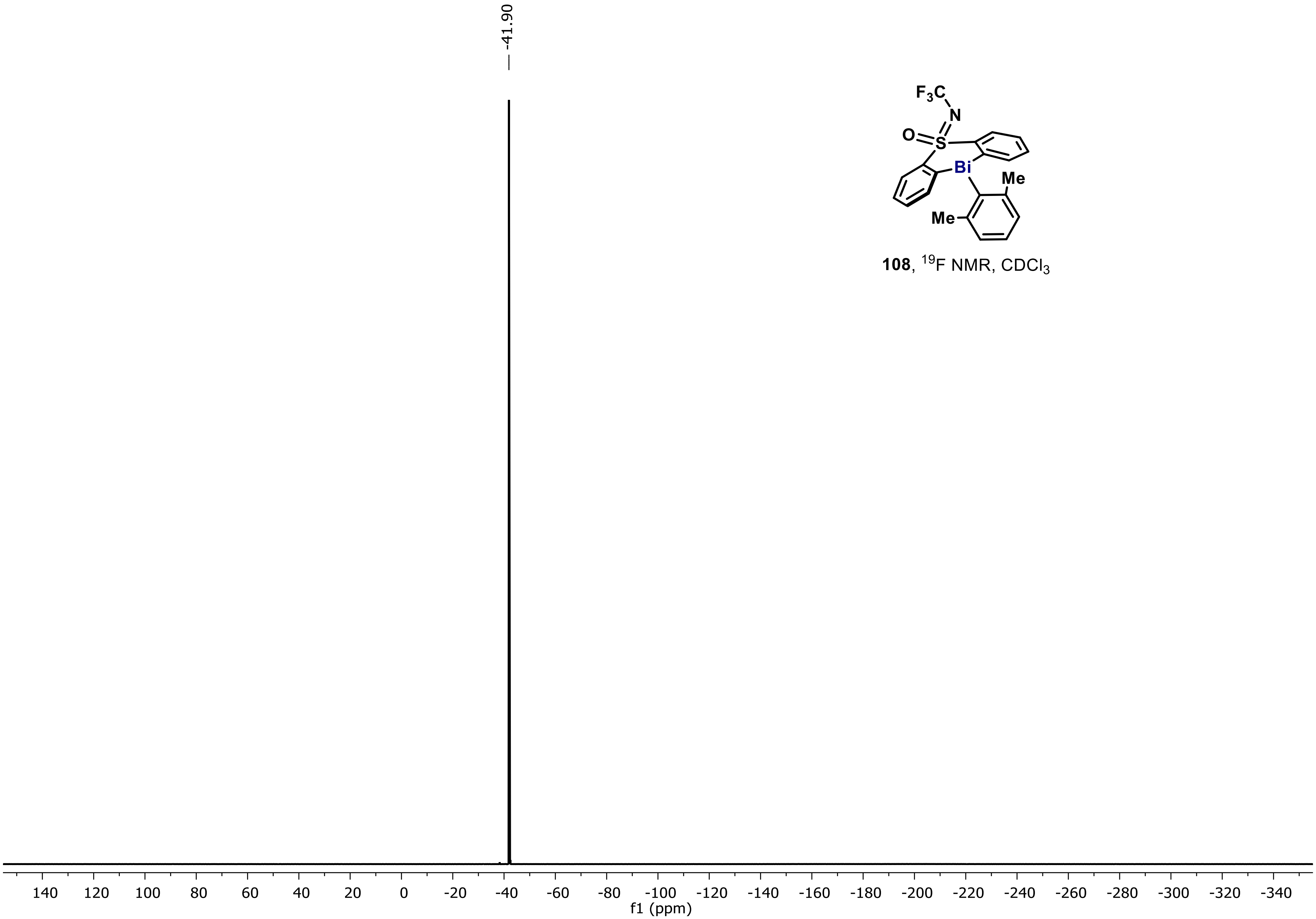

108, ^{19}F NMR, $CDCl_3$

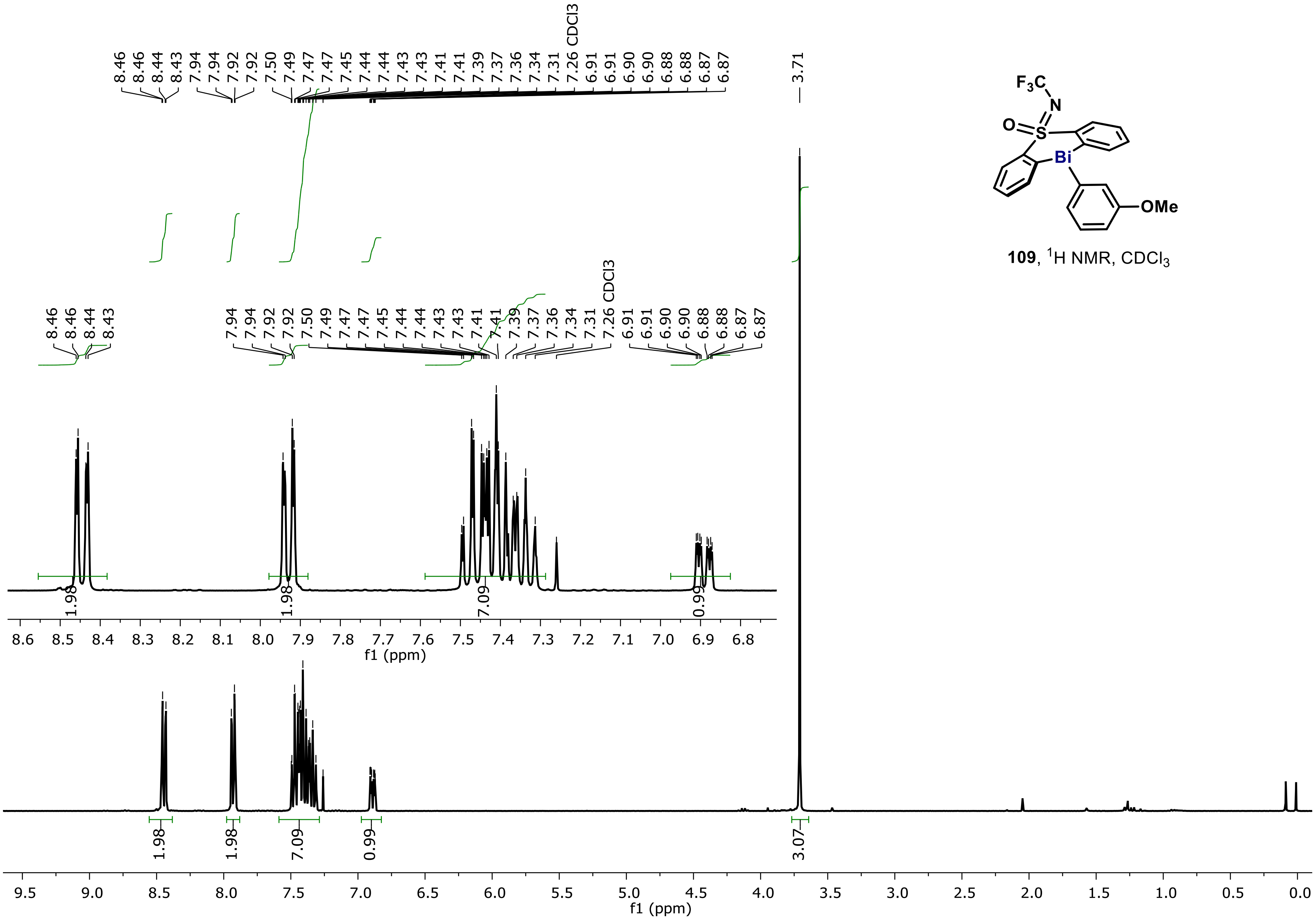

109, ^{1}H NMR, $CDCl_3$

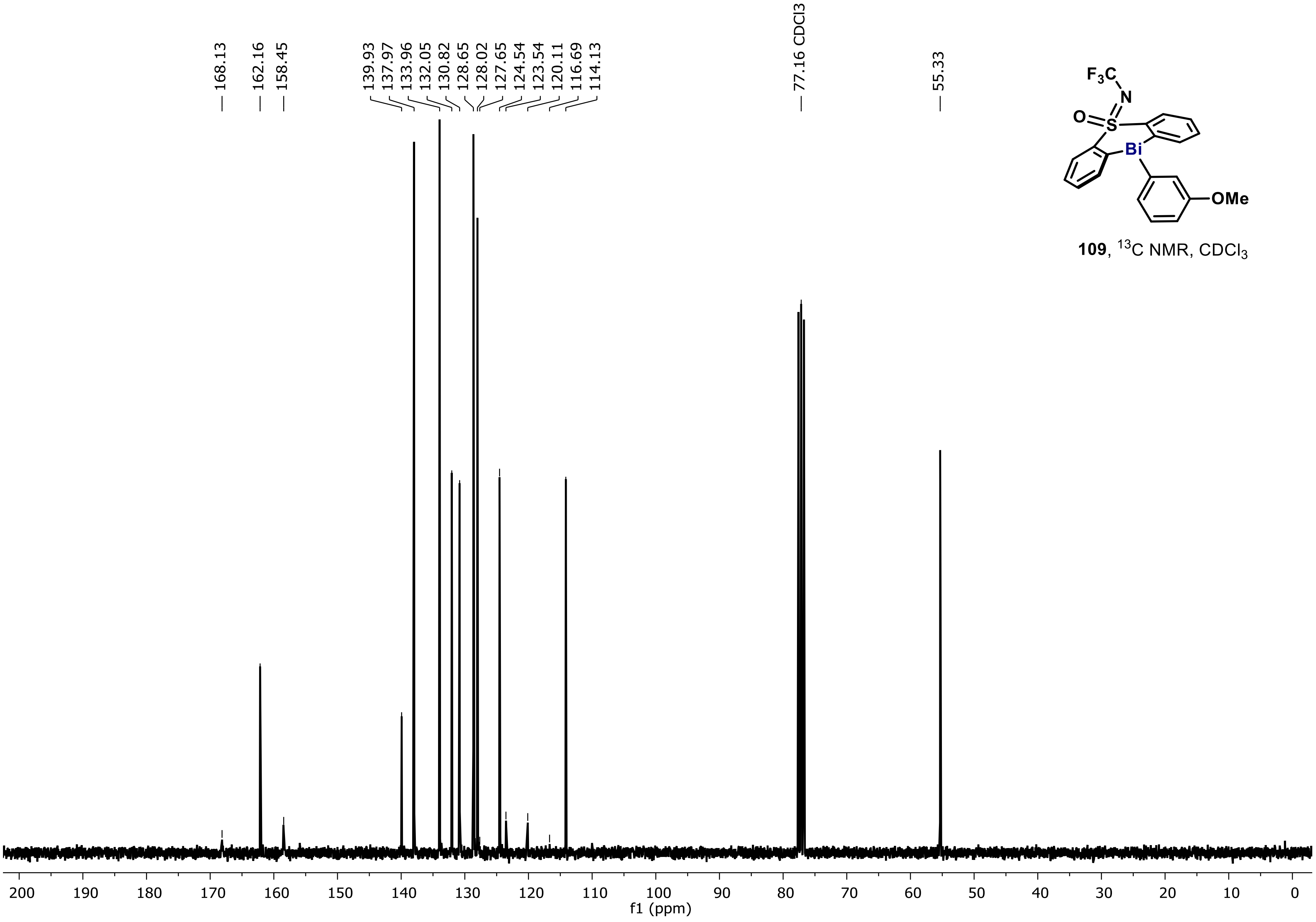

109, ^{13}C NMR, $CDCl_3$

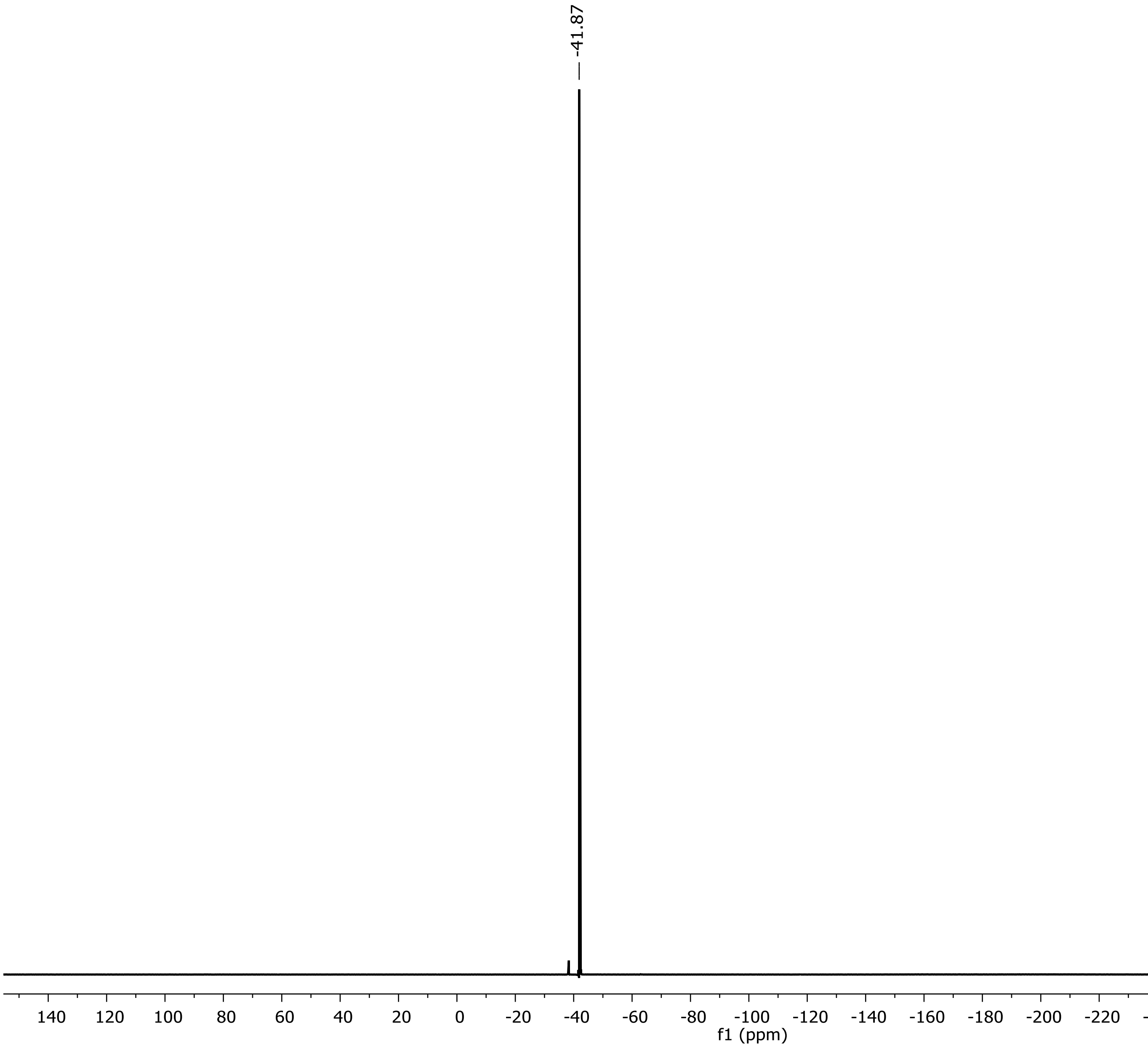

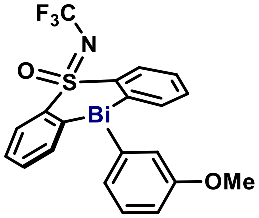

109, ^{19}F NMR, $CDCl_3$

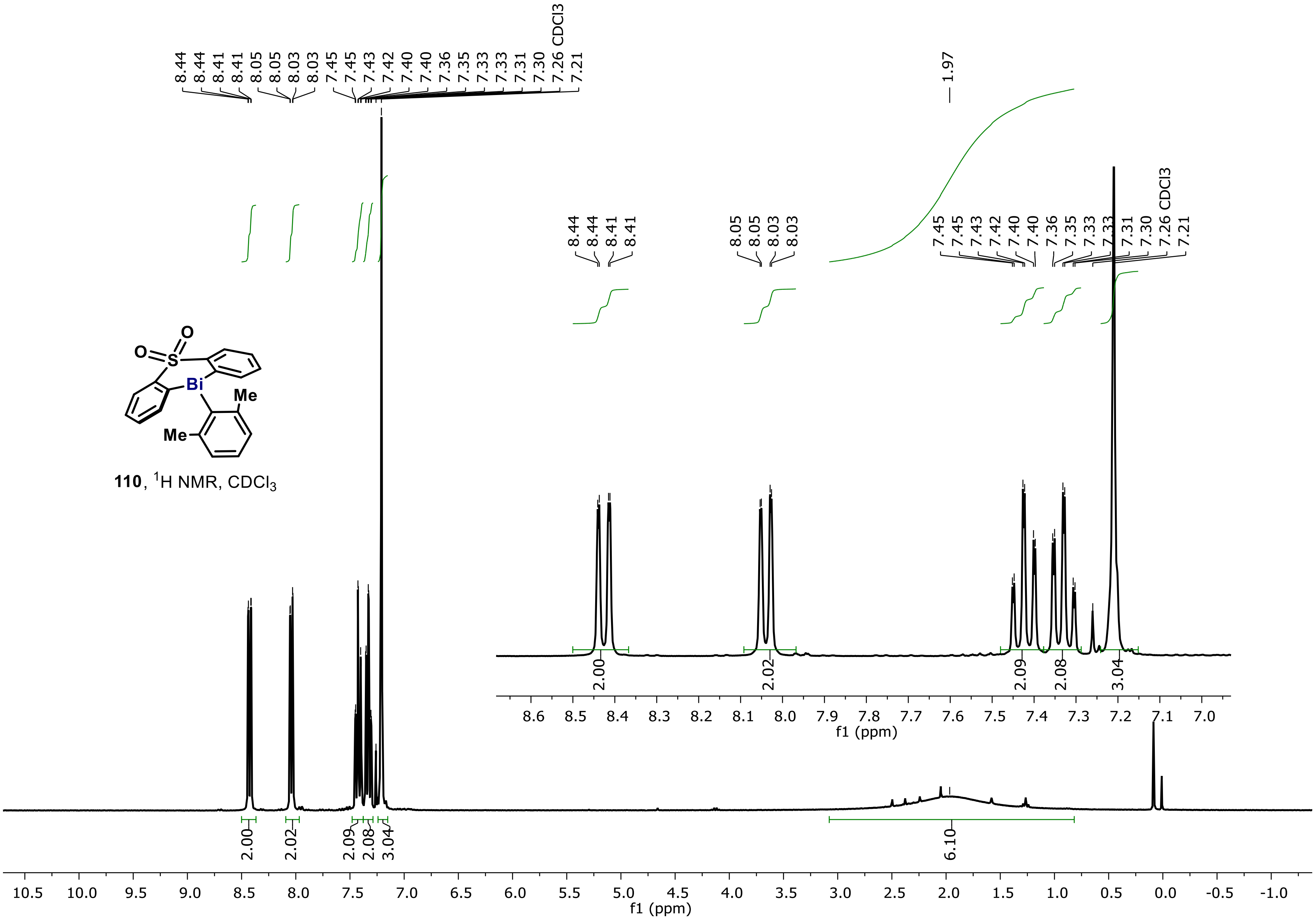

110, ^{1}H NMR, $CDCl_3$

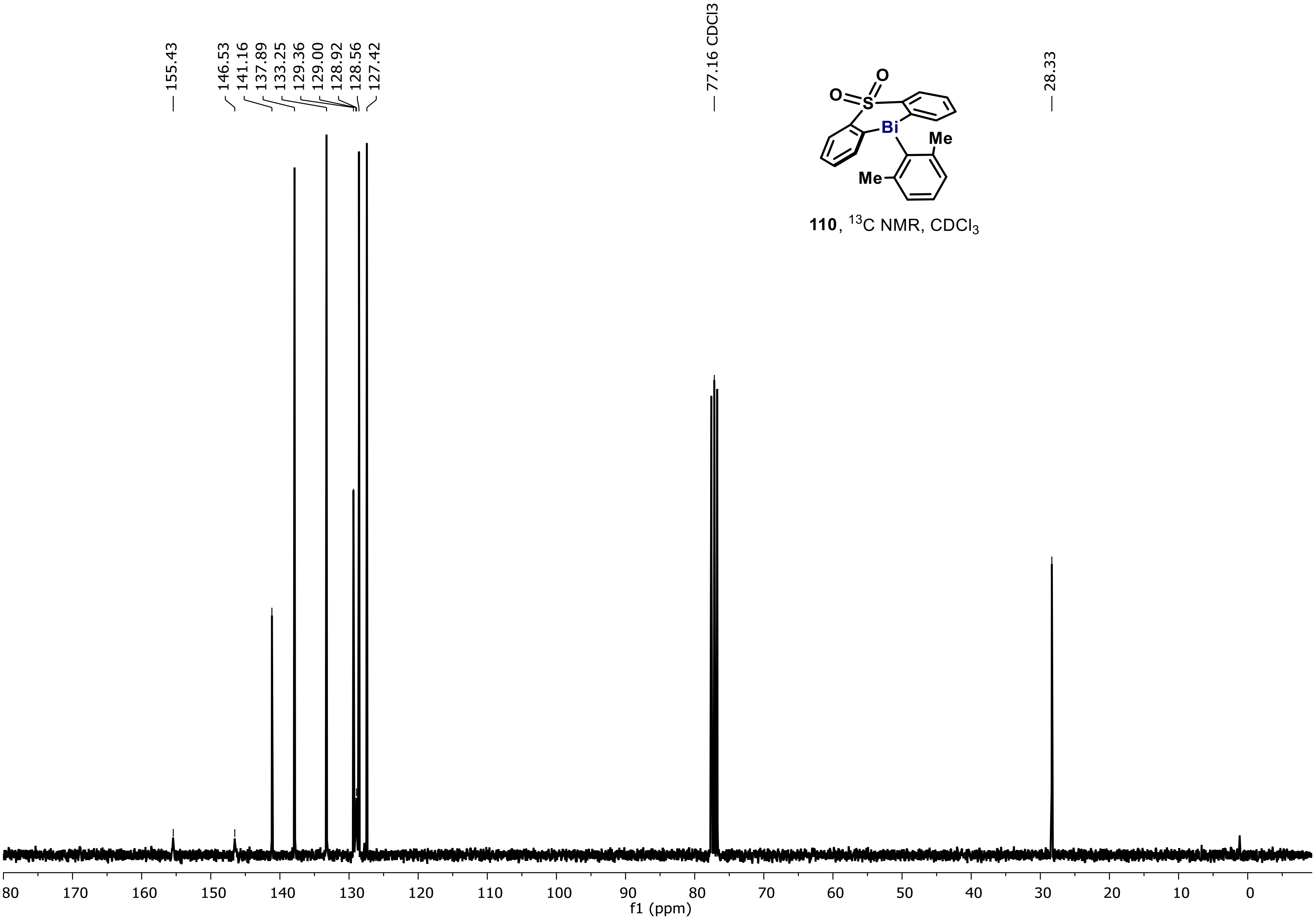

110, ^{13}C NMR, $CDCl_3$

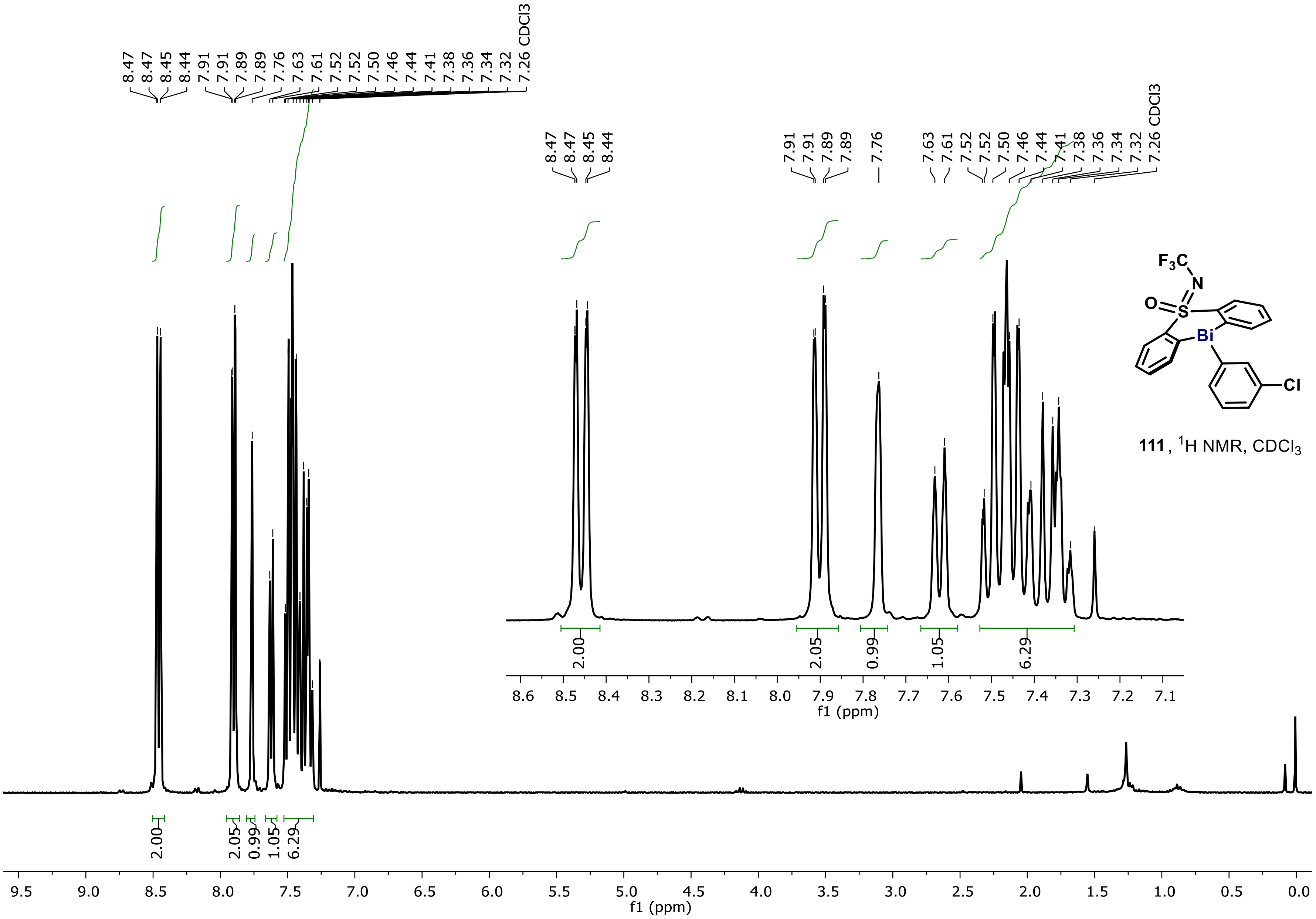

111, ^{1}H NMR, $CDCl_3$

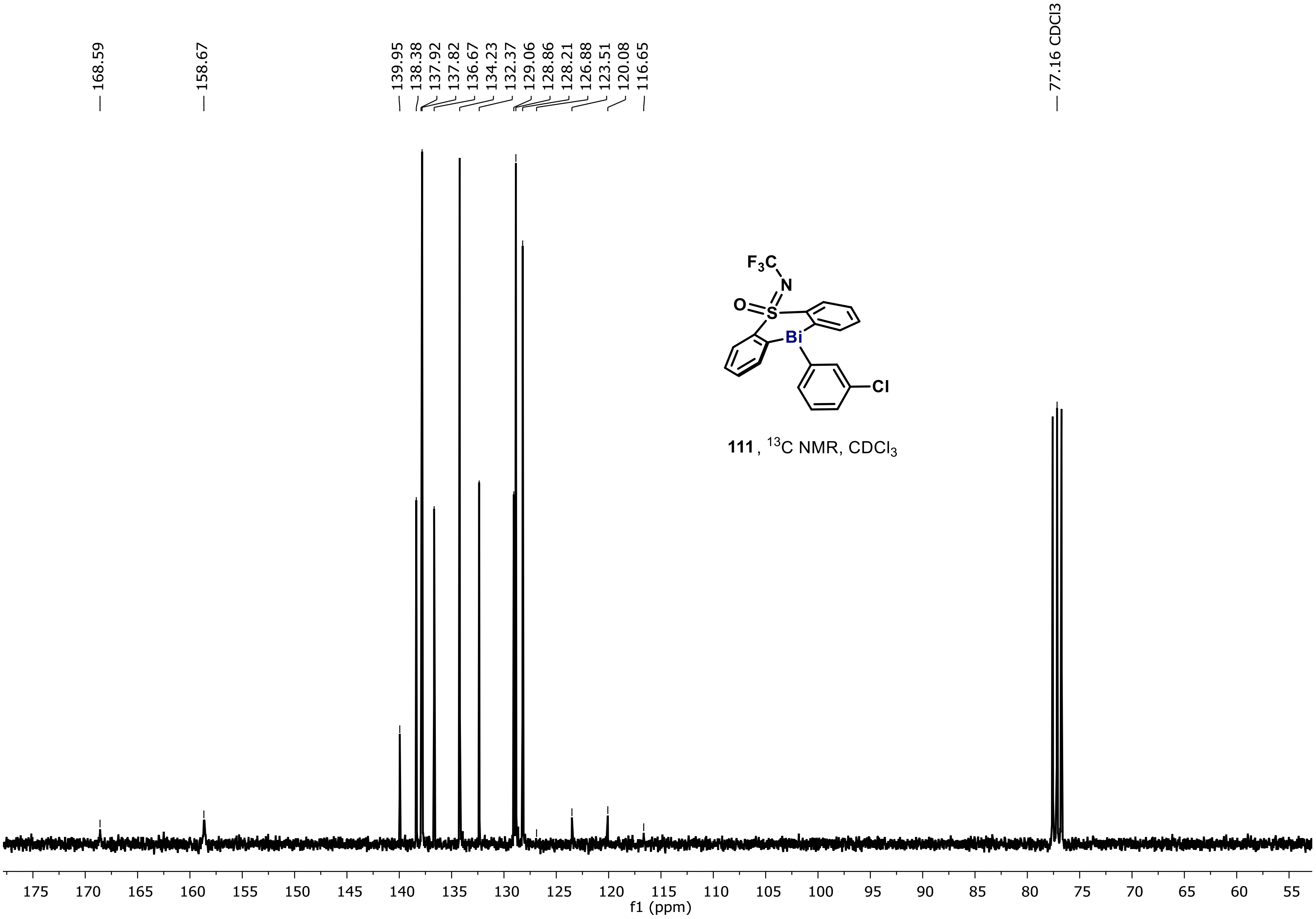

111, ^{13}C NMR, $CDCl_3$

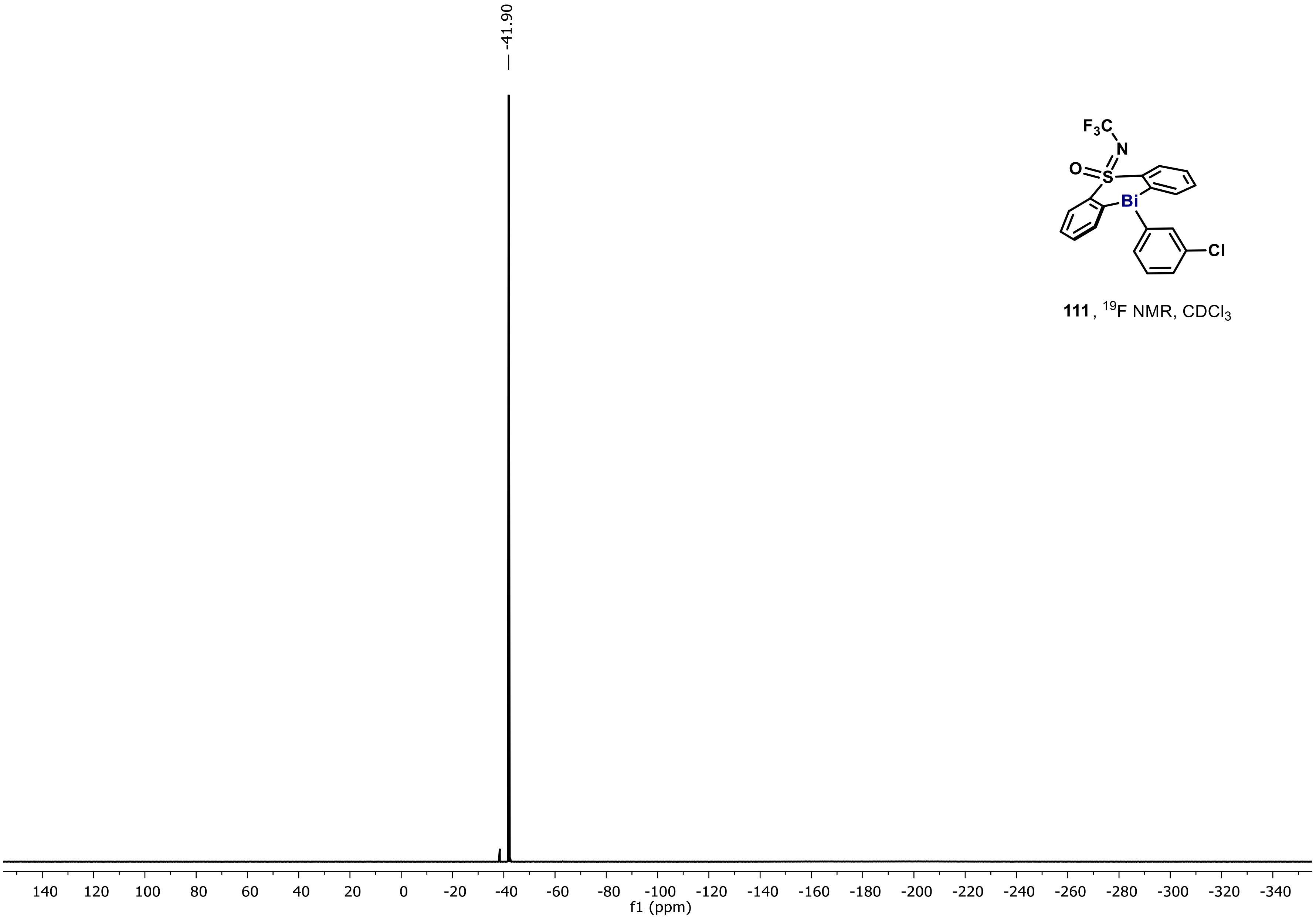

111, ^{19}F NMR, $CDCl_3$

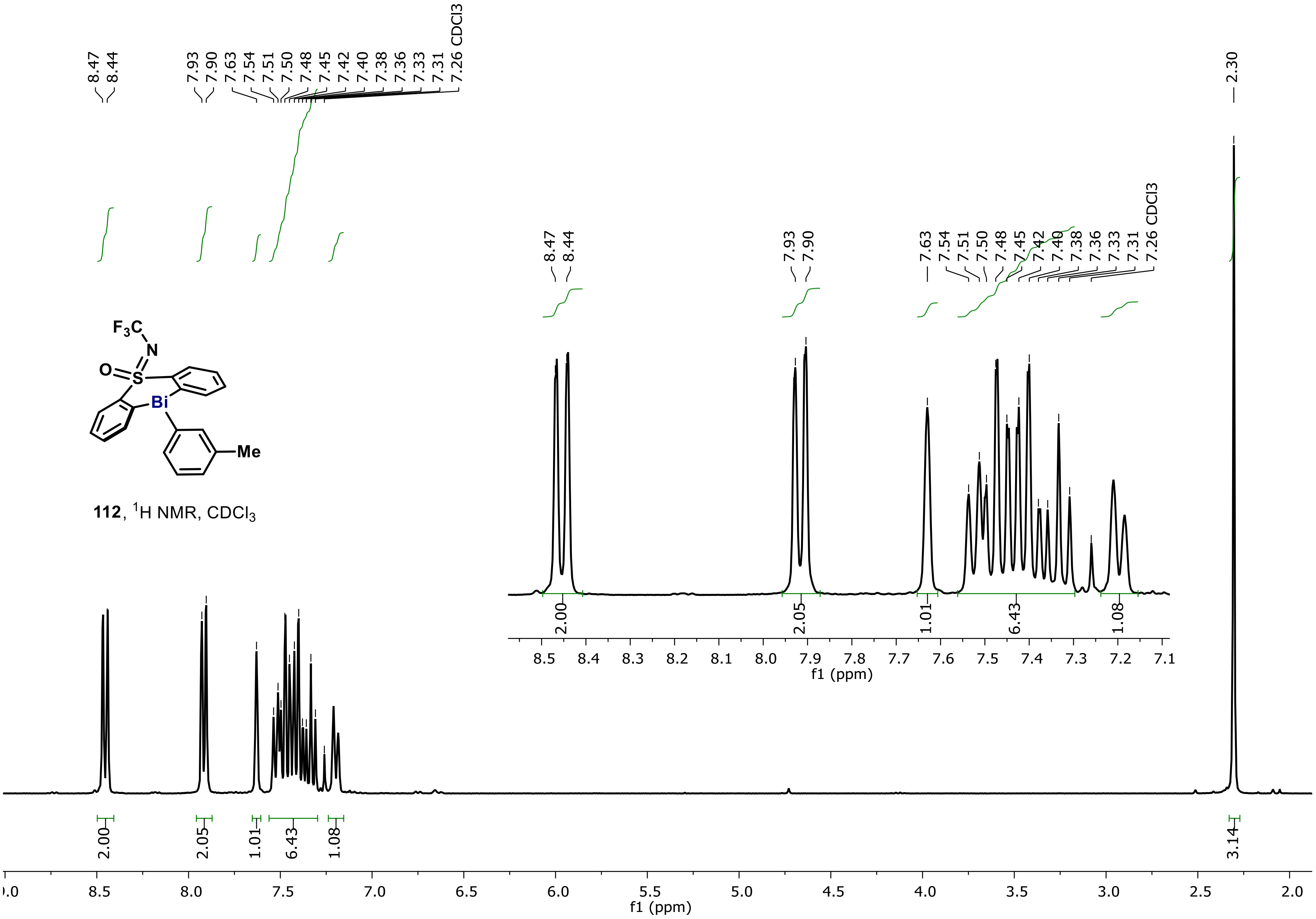

112, ^{1}H NMR, $CDCl_3$

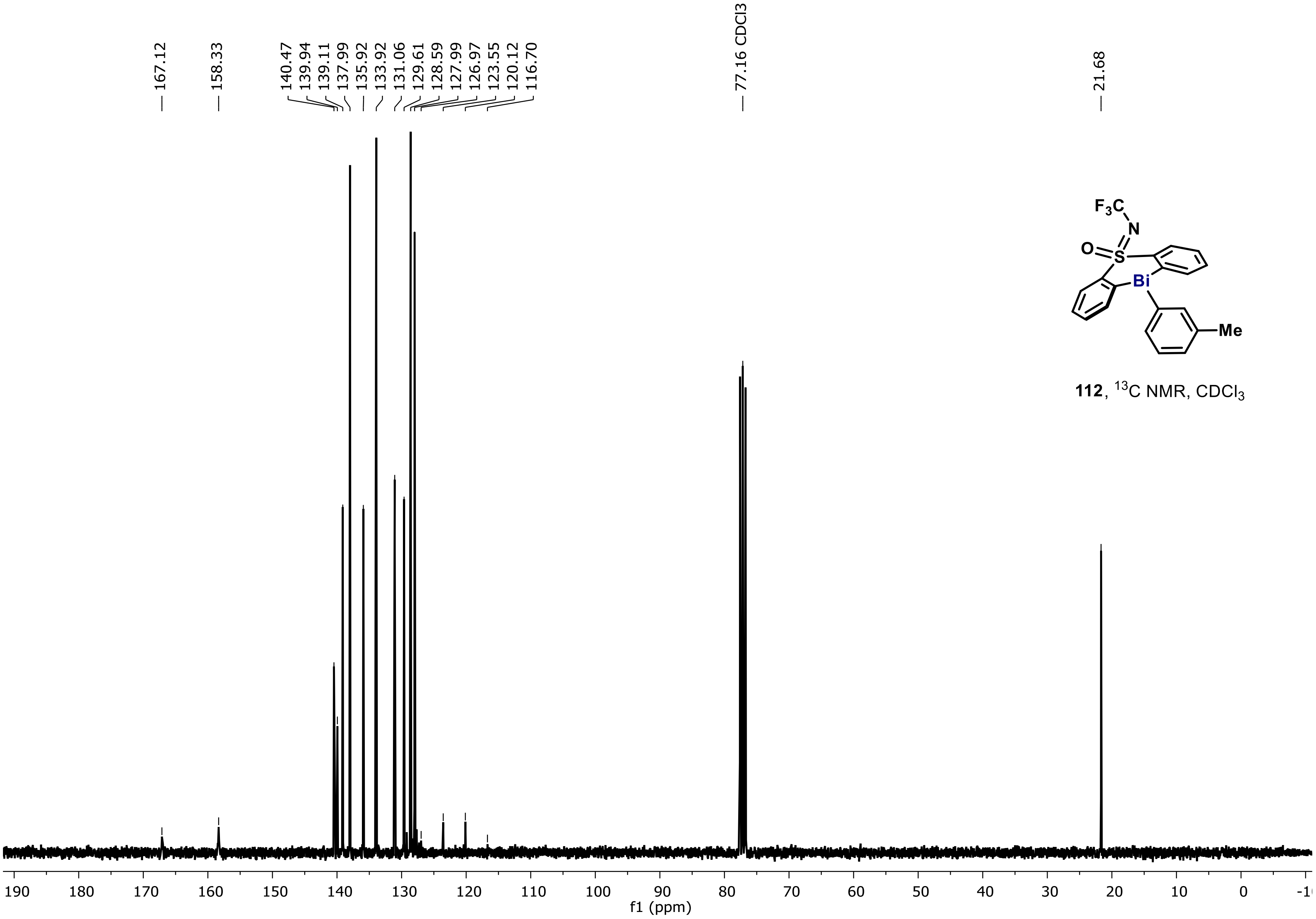

112, ^{13}C NMR, $CDCl_3$

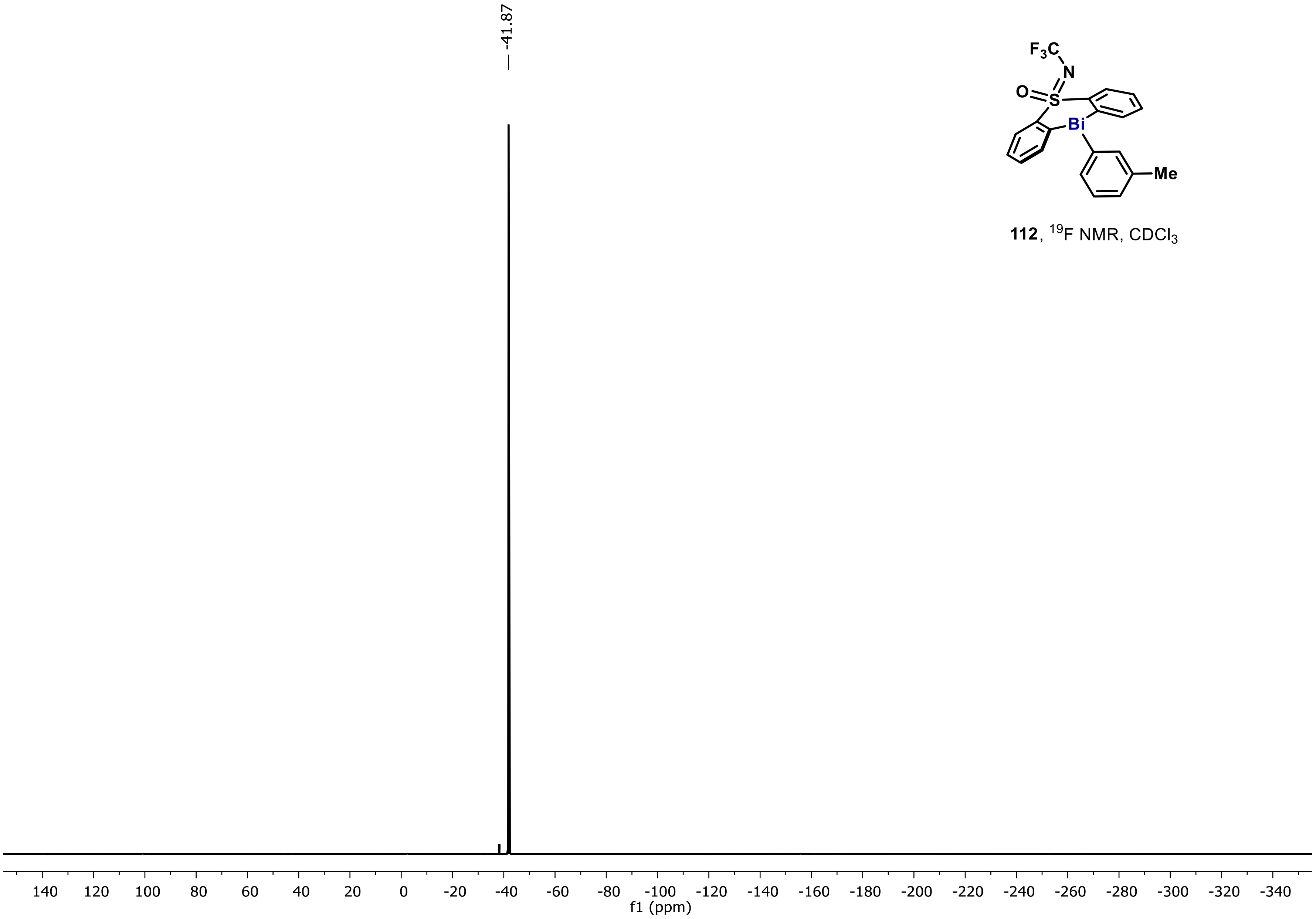

112, ^{19}F NMR, $CDCl_3$

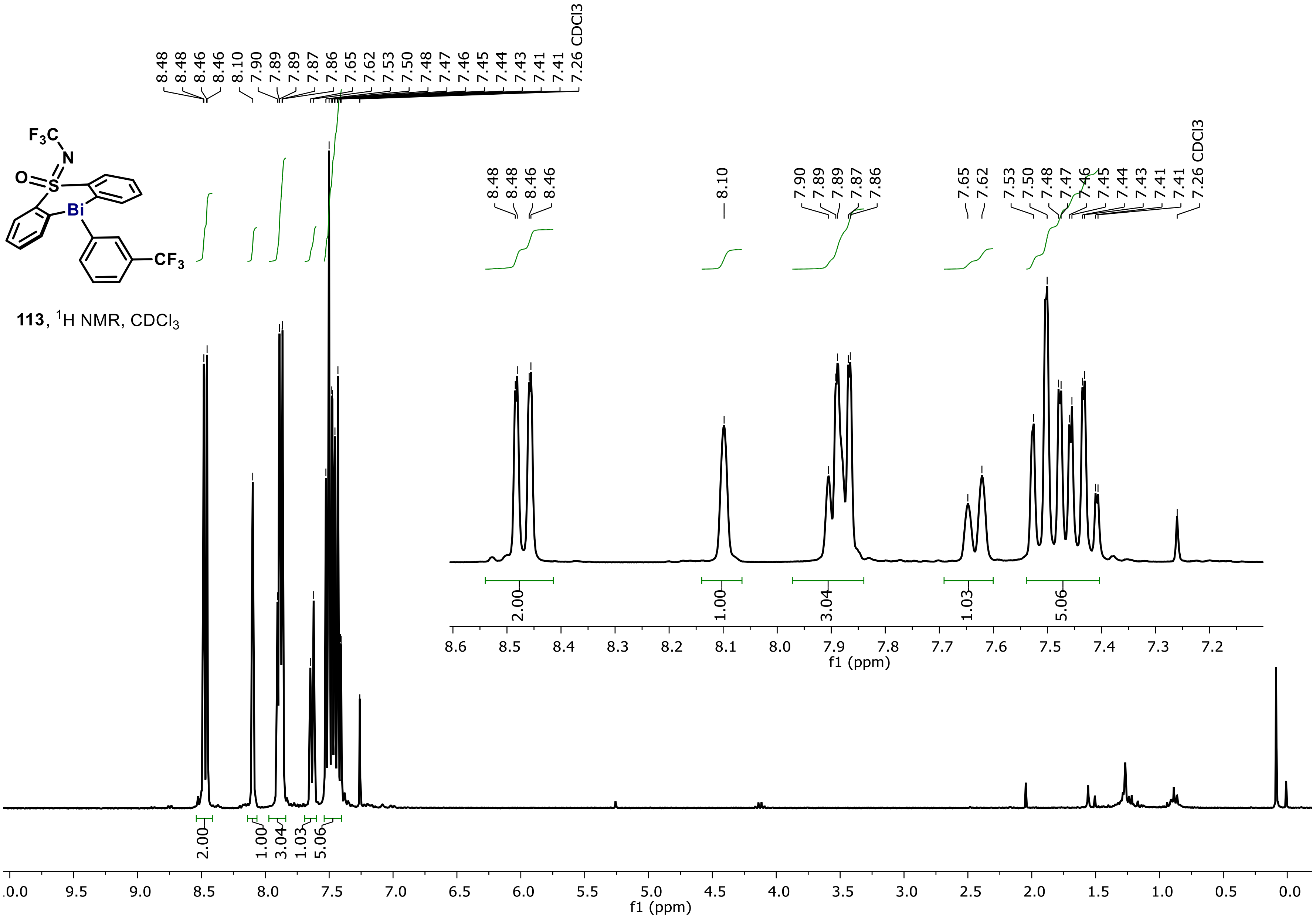
F3C
N
O
S
Bi
CF3
113, 1H NMR, CDCl3
8.48
8.48
8.46
8.46
8.10
7.90
7.89
7.89
7.87
7.86
7.65
7.62
7.53
7.50
7.48
7.47
7.46
7.45
7.44
7.43
7.41
7.41
7.26 CDCl3
2.00
1.00
3.04
1.03
5.06
f1 (ppm)

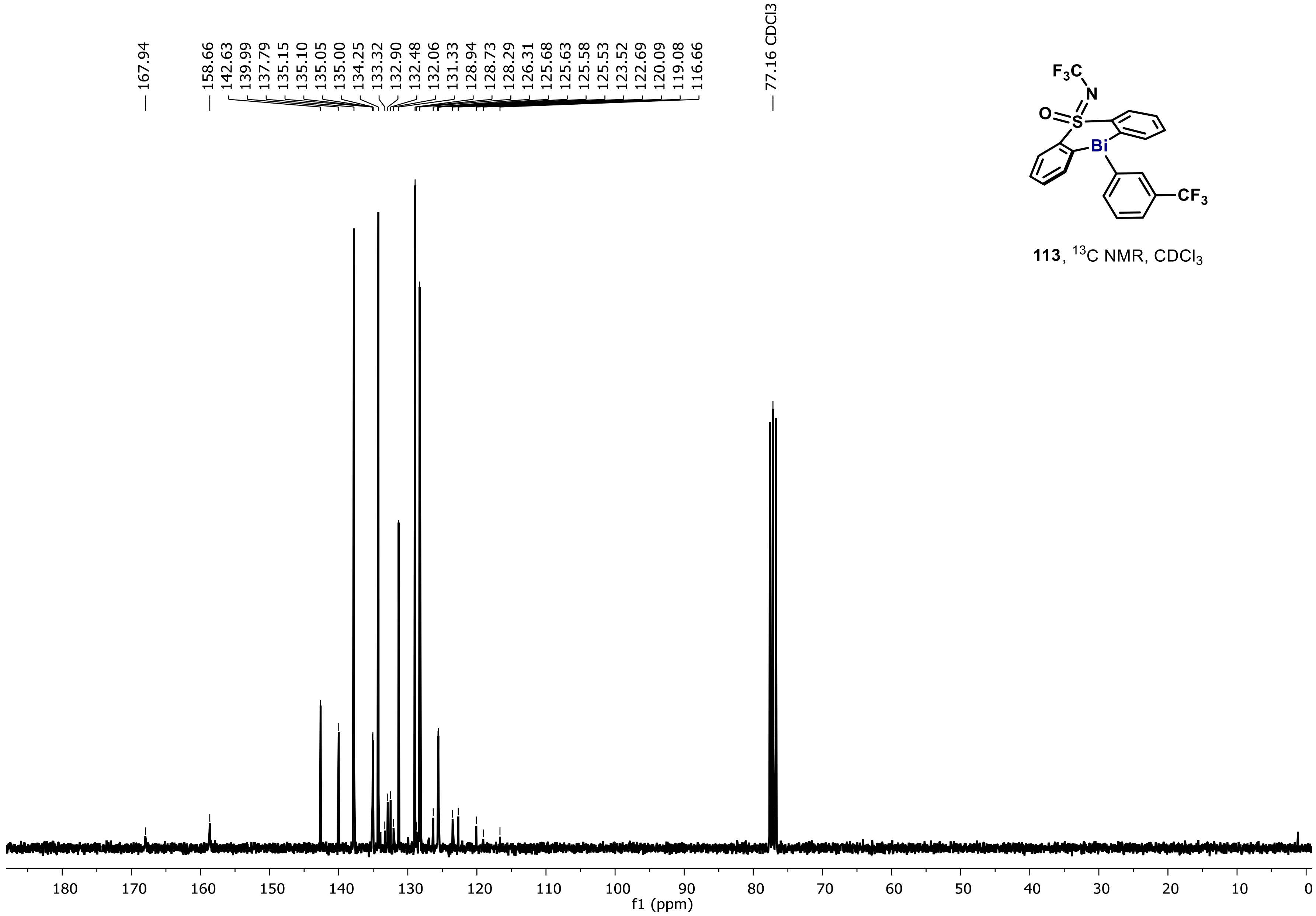

113, ^{13}C NMR, $CDCl_3$

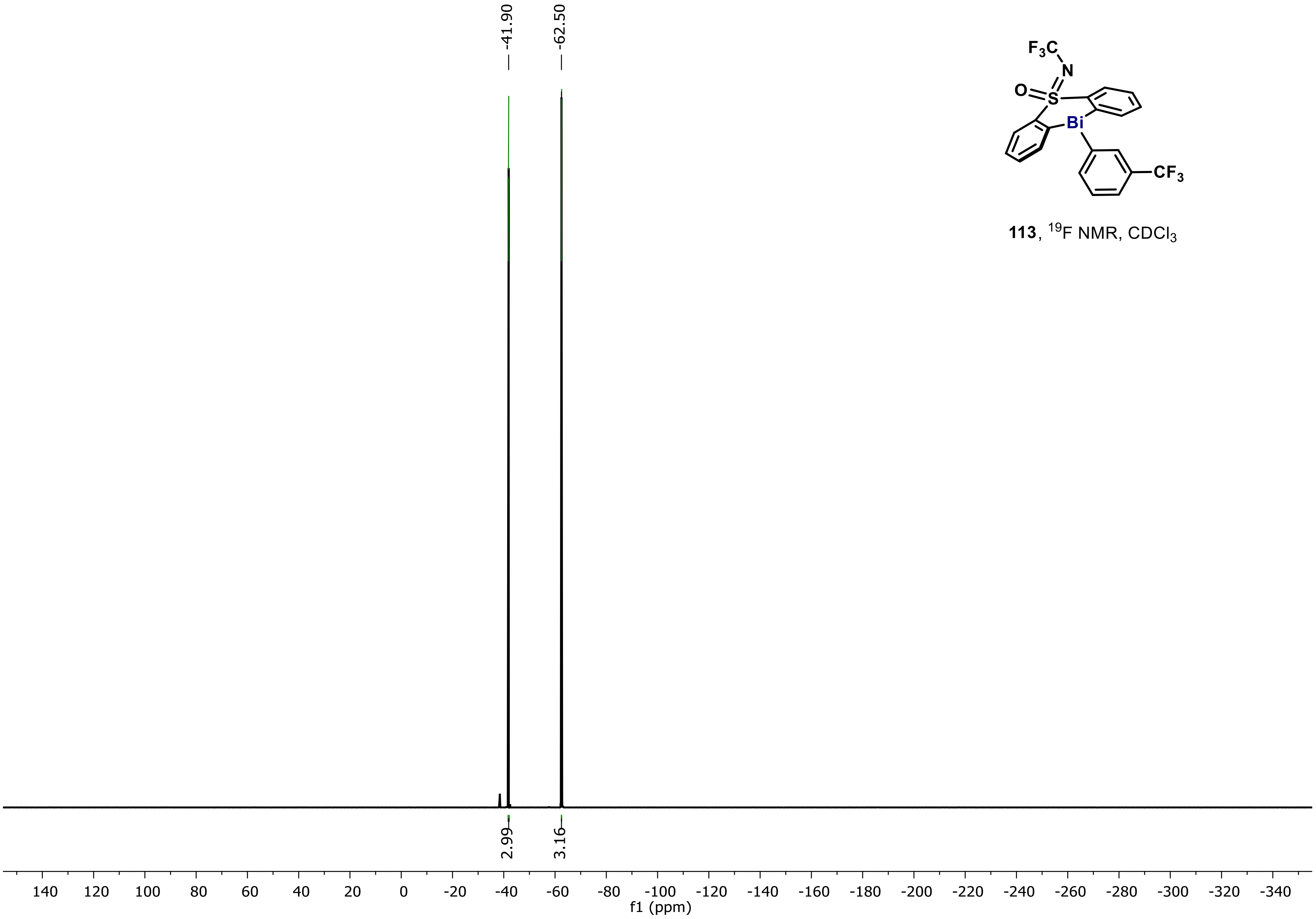

113, ^{19}F NMR, $CDCl_3$

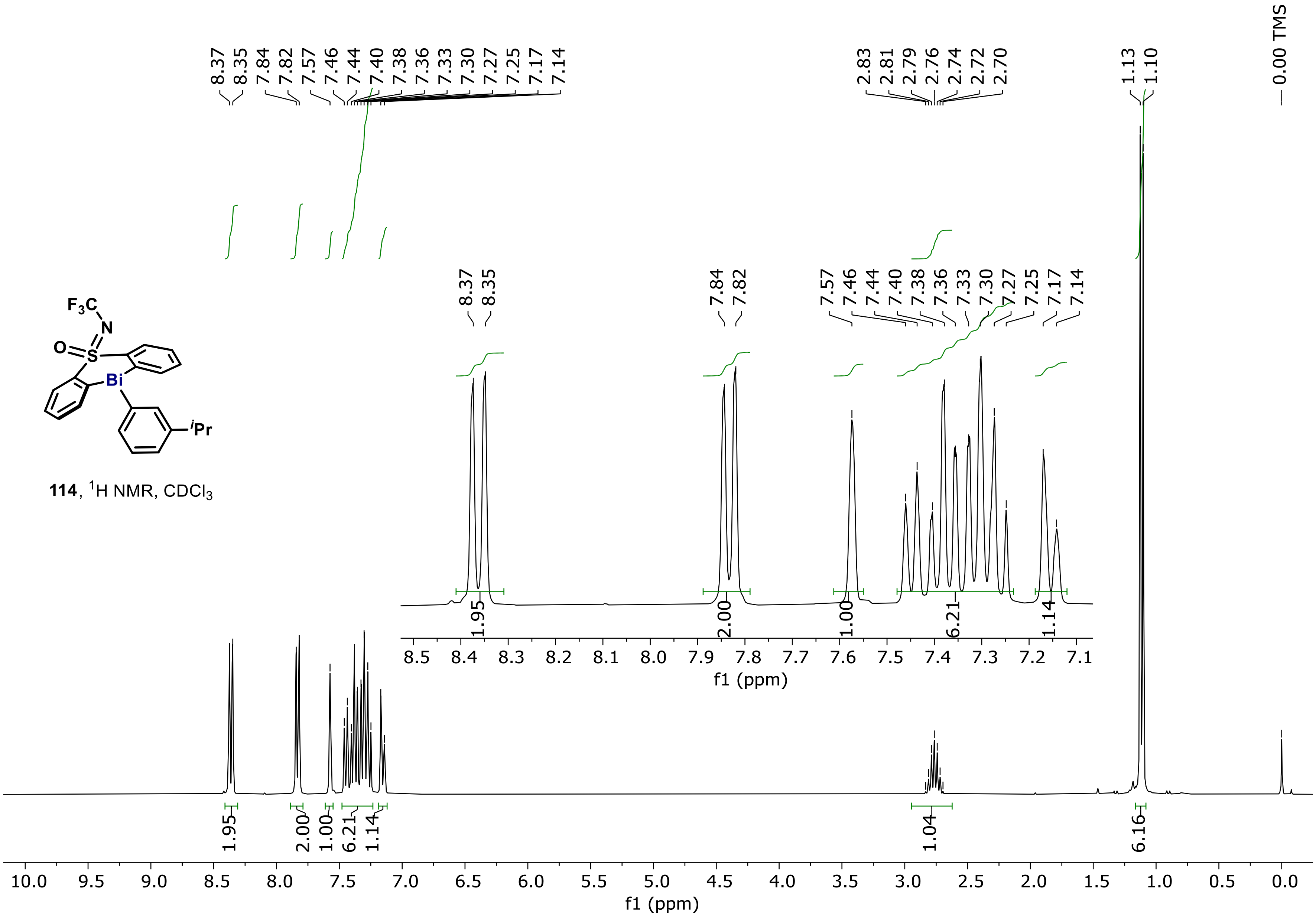

F3C
N
O
S
Bi
iPr
114, 1H NMR, CDCl3
0.00 TMS
f1 (ppm)

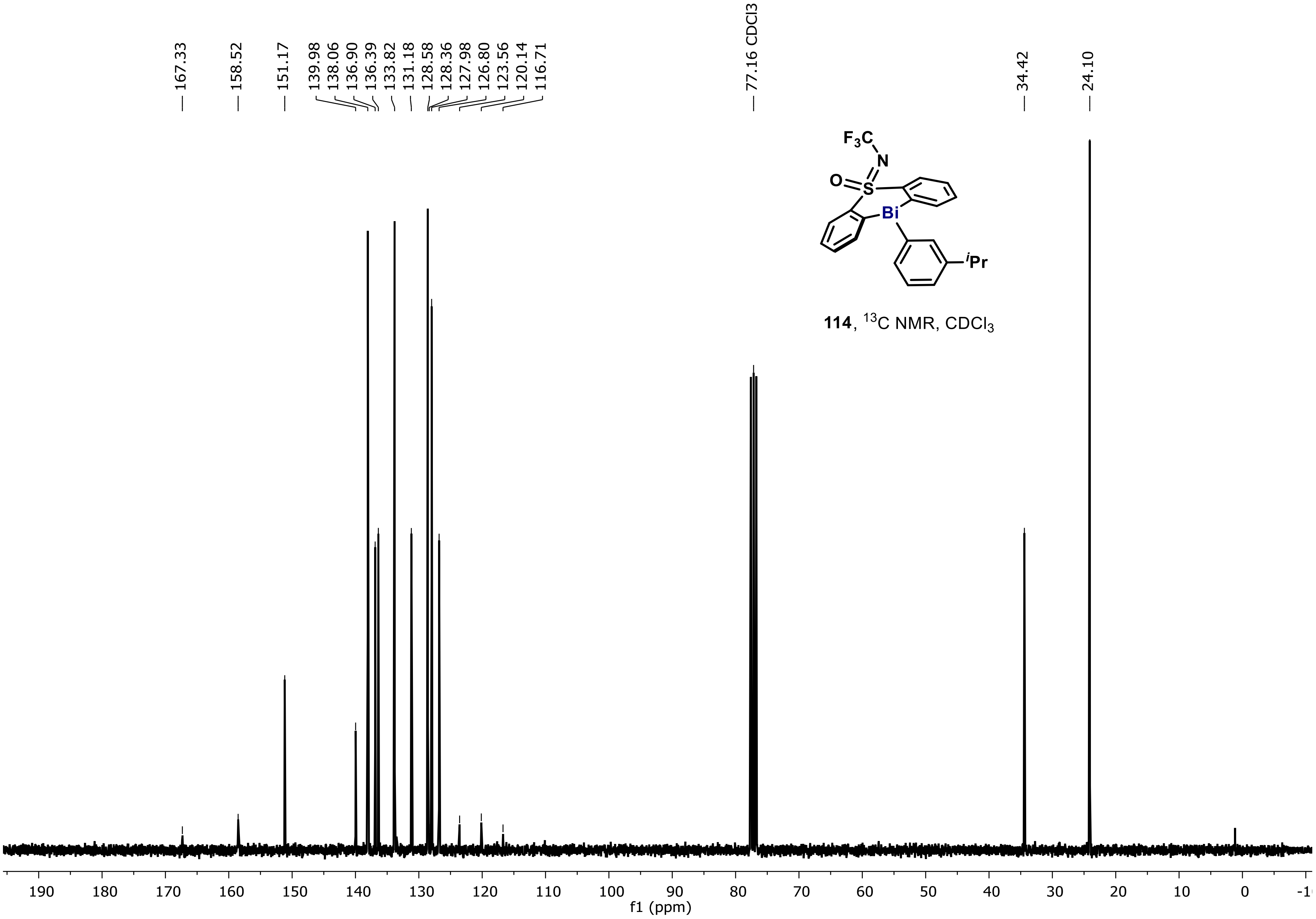

114, ^{13}C NMR, $CDCl_3$

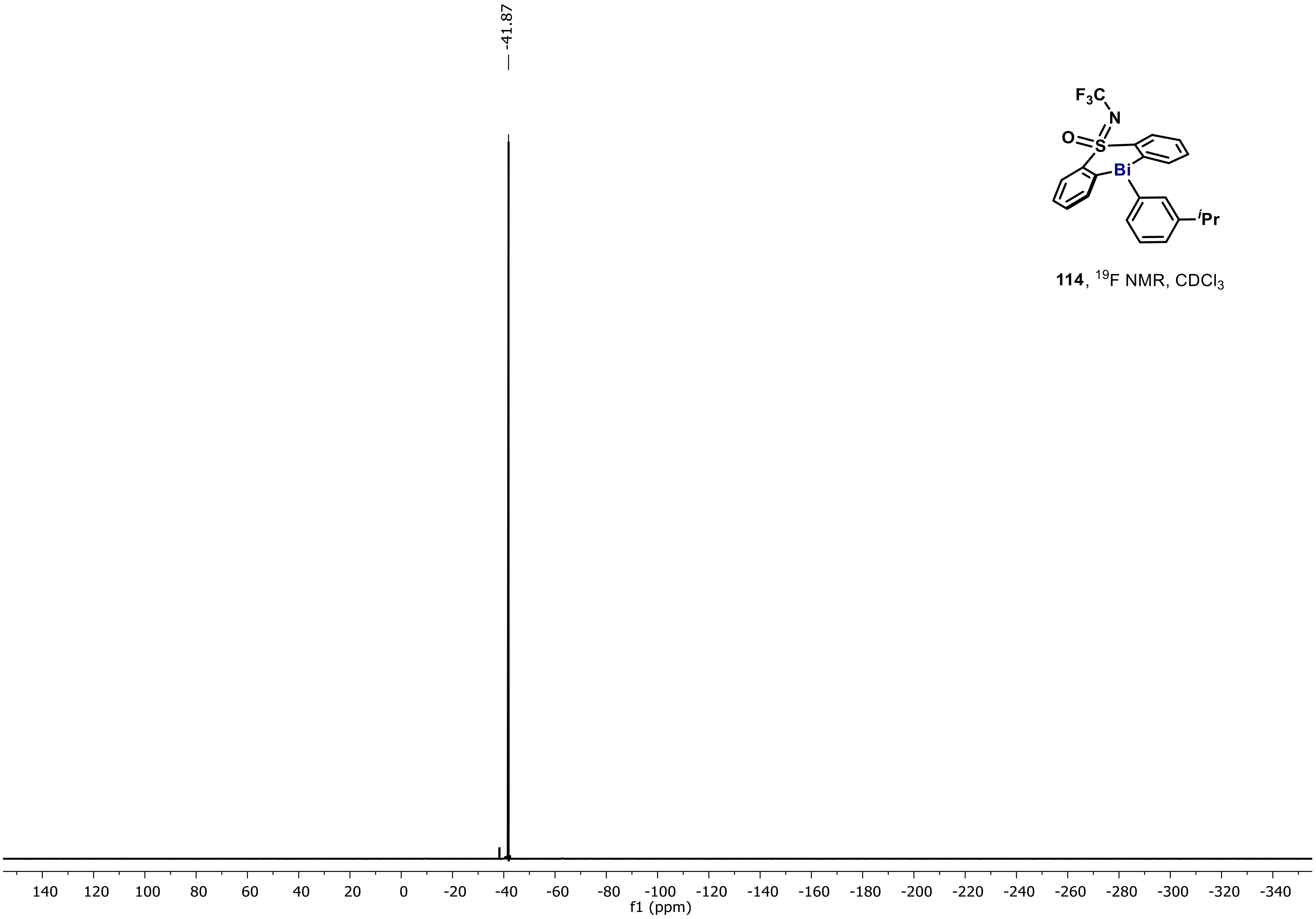

114, ^{19}F NMR, $CDCl_3$

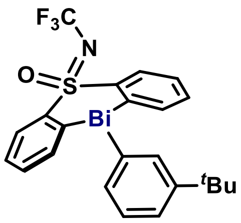

115, ^{1}H NMR, $CDCl_3$

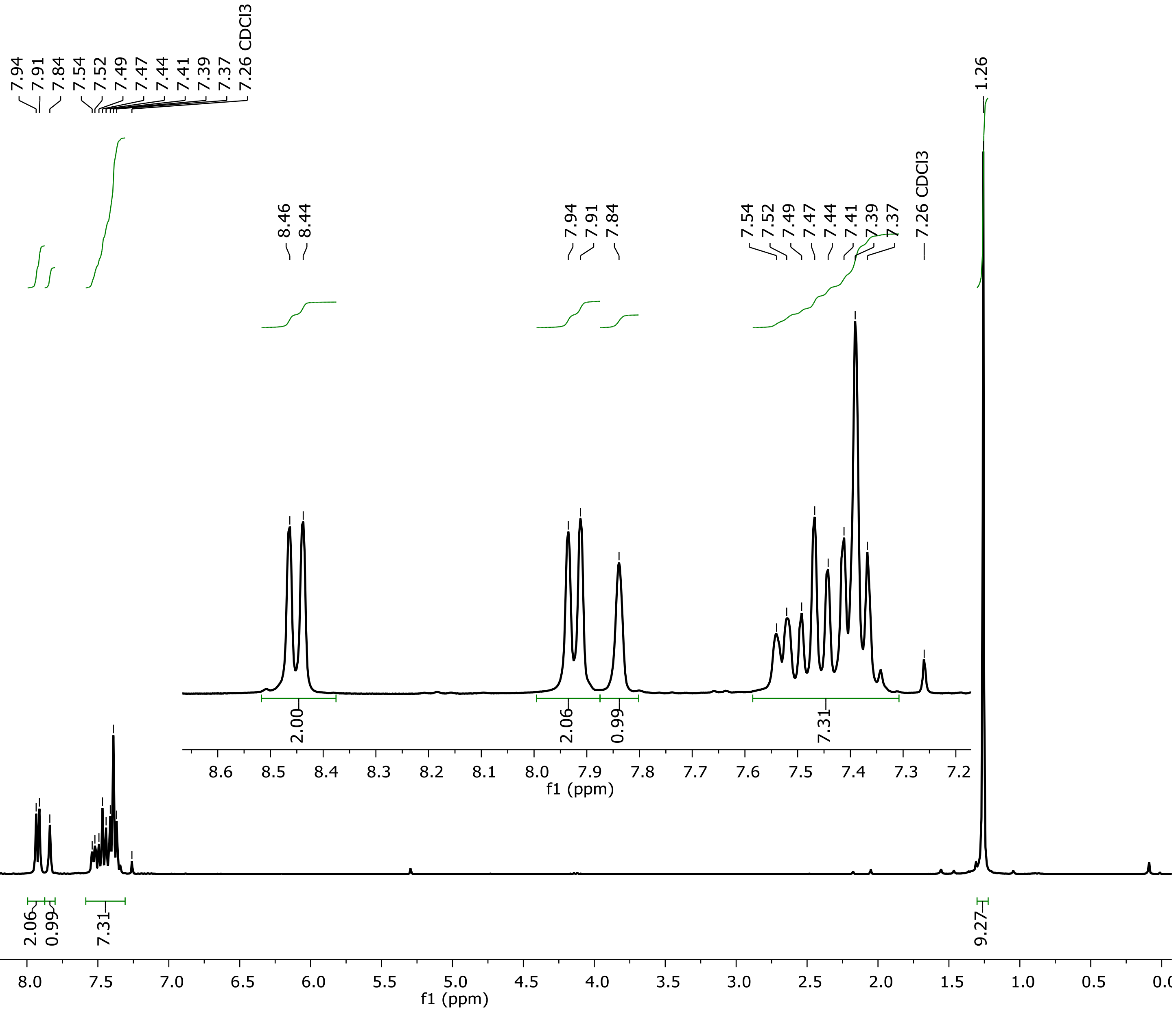

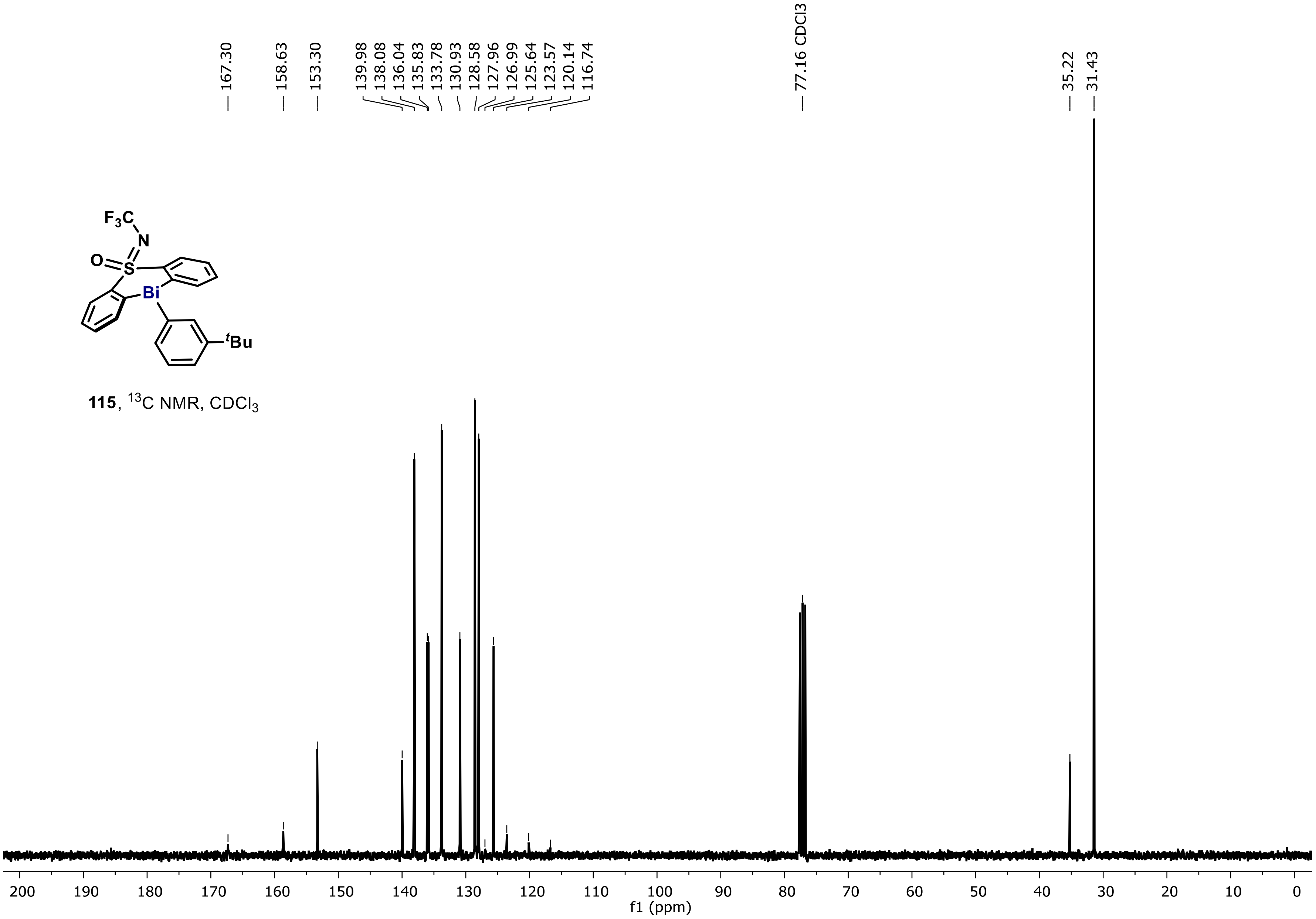

115, ^{13}C NMR, $CDCl_3$

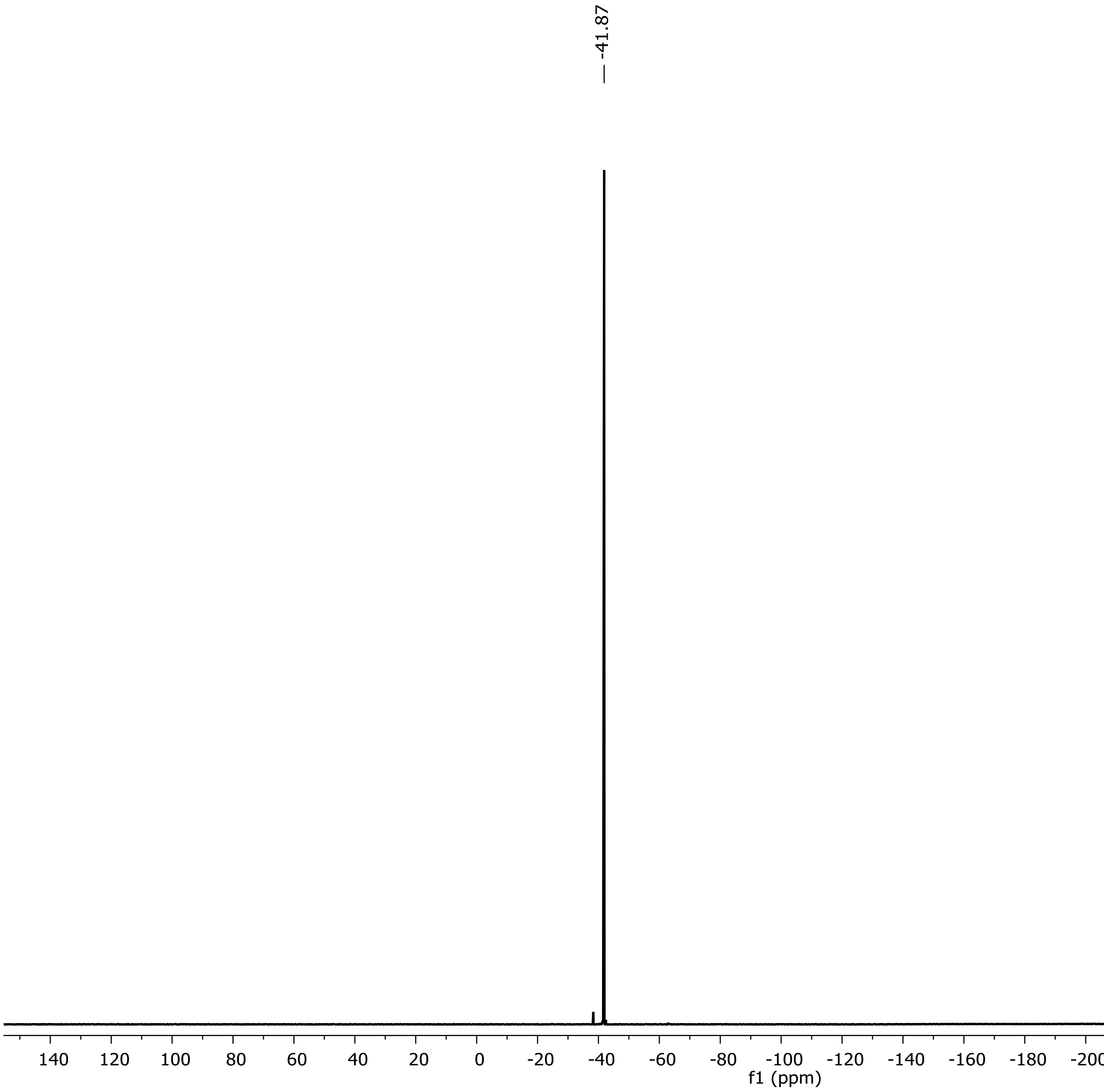

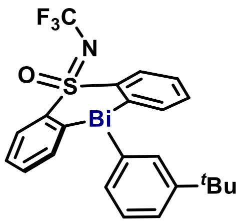

115, ^{19}F NMR, $CDCl_3$

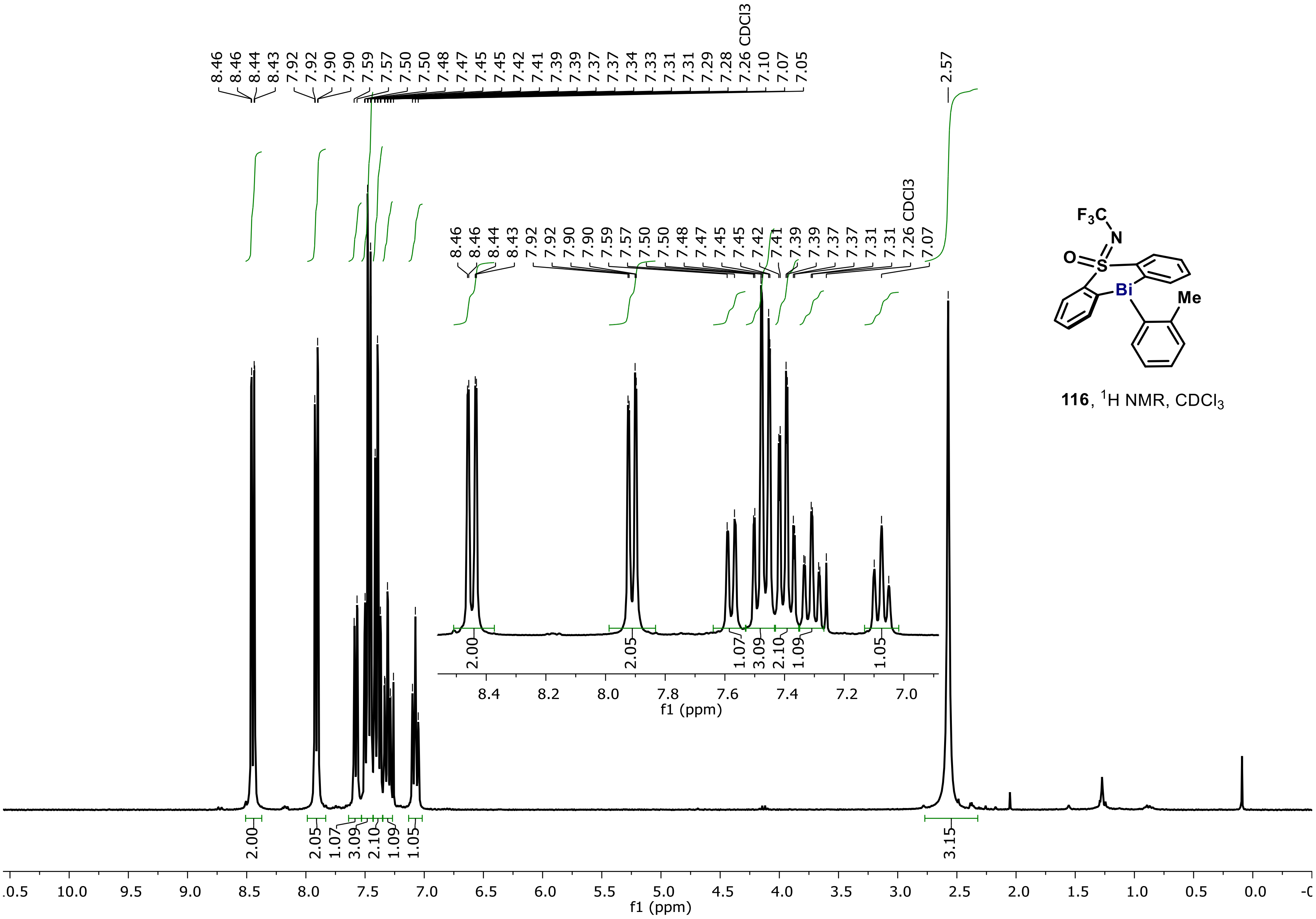
116, ¹H NMR, CDCl3
F3C
N
O
S
Bi
Me
f1 (ppm)

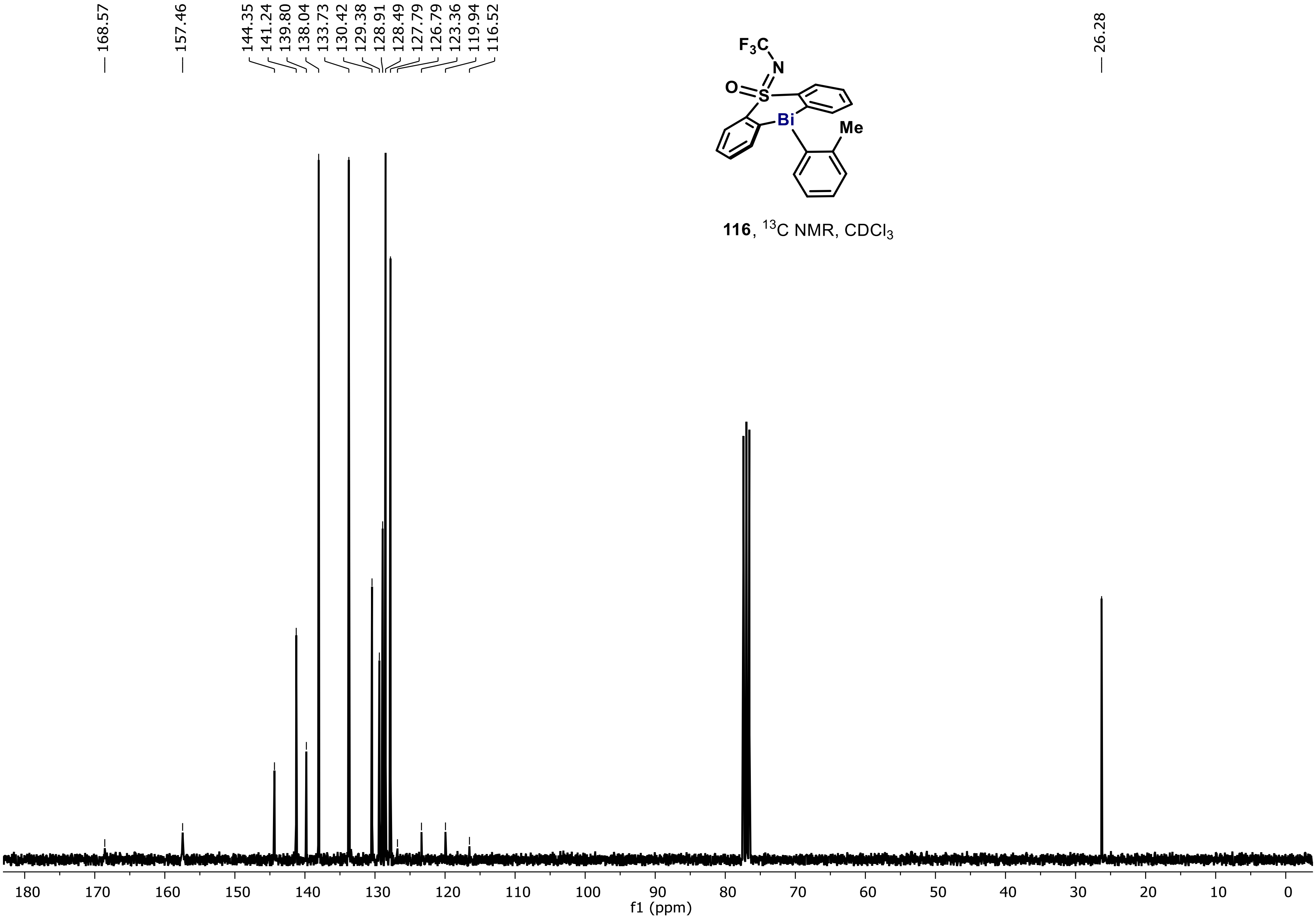

168.57
157.46
144.35
141.24
139.80
138.04
133.73
130.42
129.38
128.91
128.49
127.79
126.79
123.36
119.94
116.52
26.28
F3C
N
O
S
Bi
Me
116, 13C NMR, CDCl3
180
170
160
150
140
130
120
110
100
90
80
70
60
50
40
30
20
10
0
f1 (ppm)

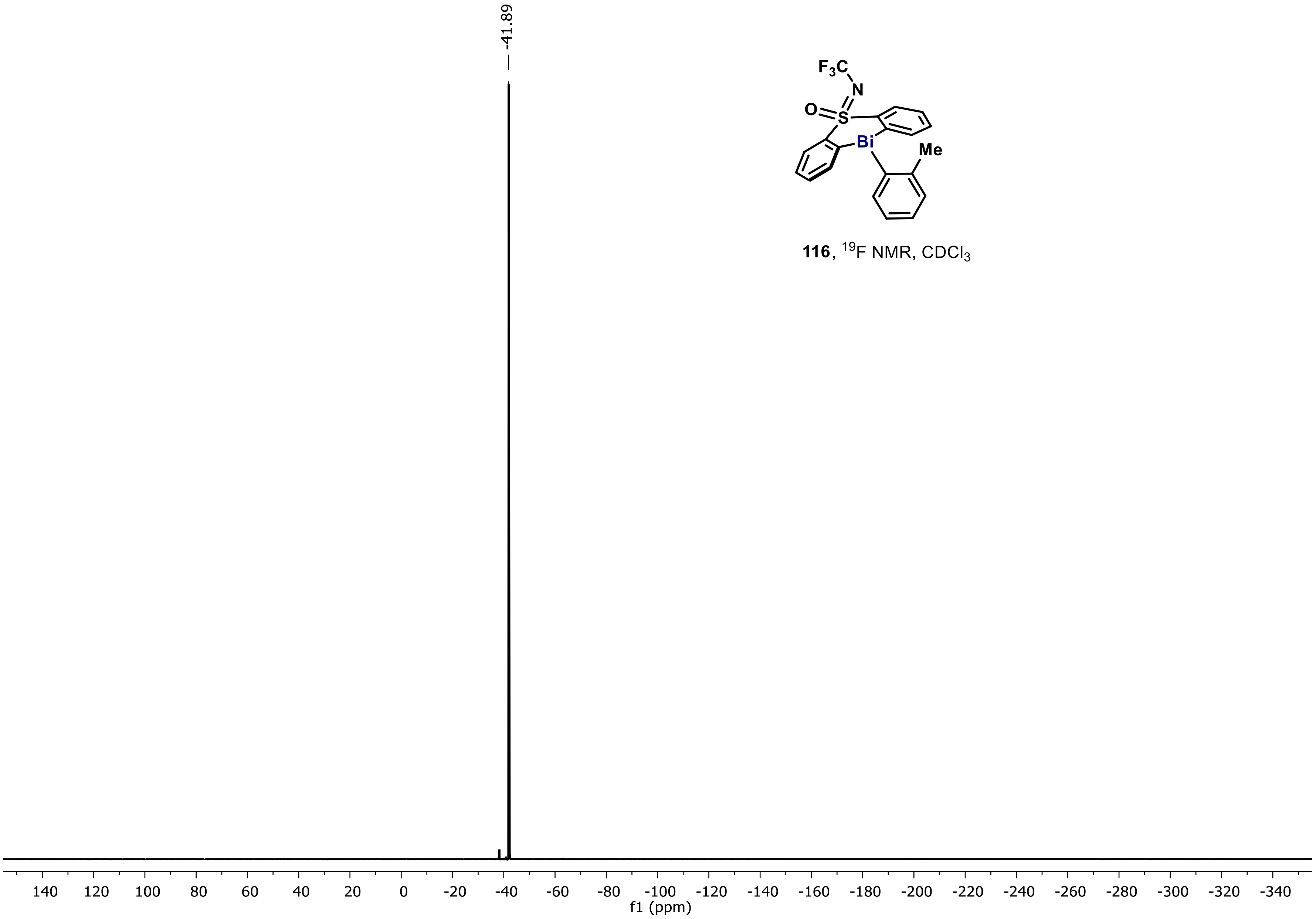

116, ^{19}F NMR, $CDCl_3$

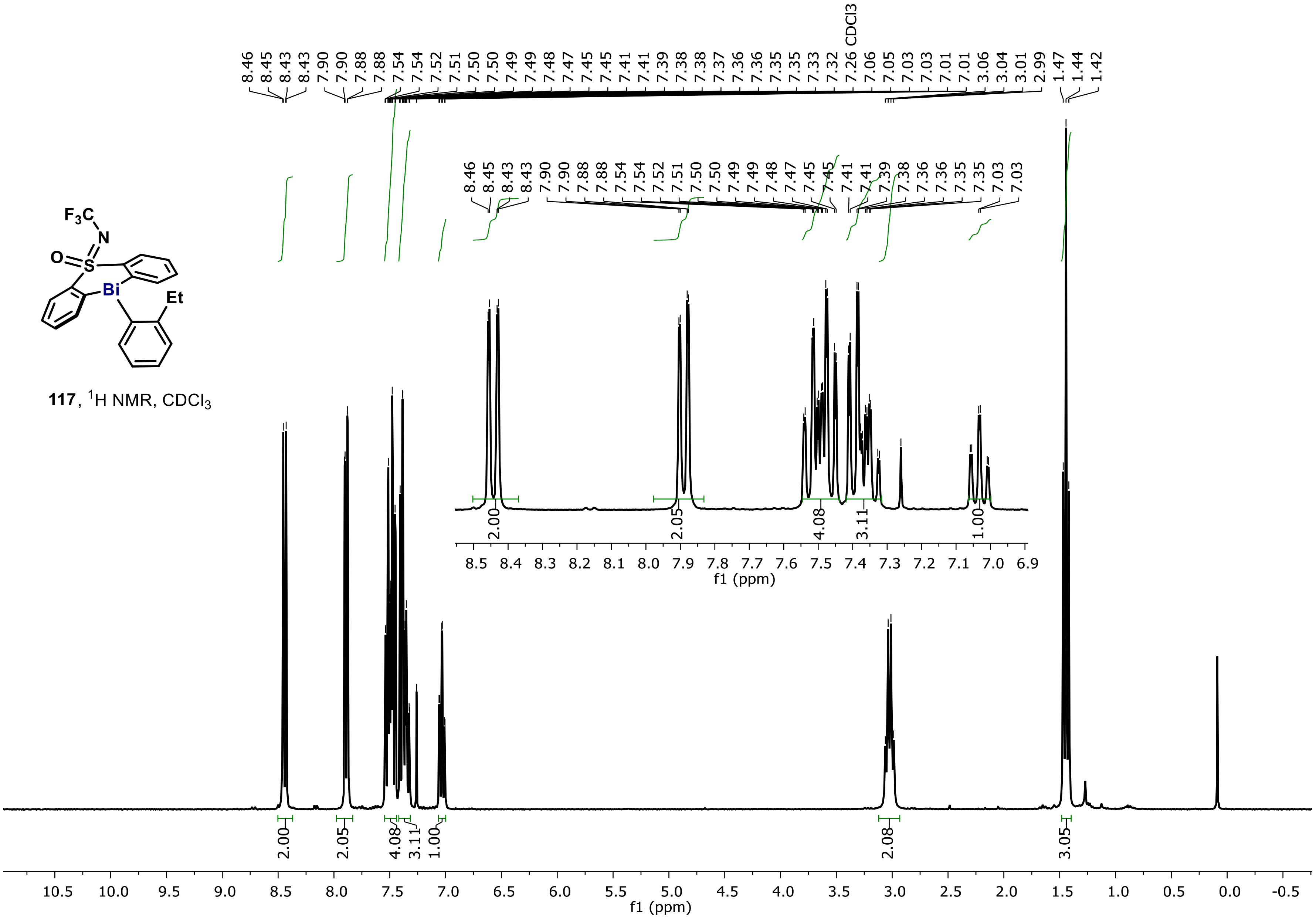

117, ^{1}H NMR, $CDCl_3$

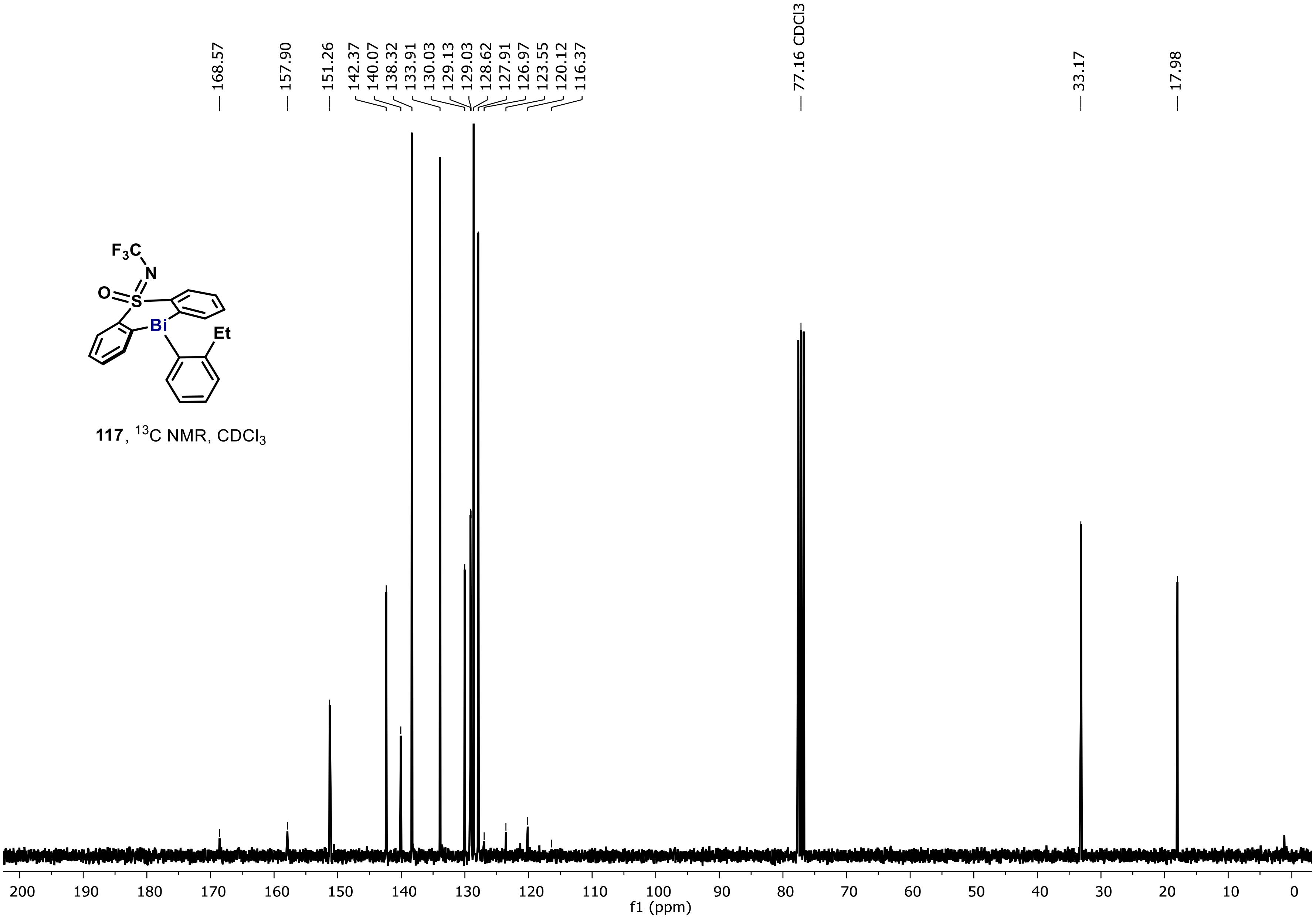
168.57
157.90
151.26
142.37
140.07
138.32
133.91
130.03
129.13
129.03
128.62
127.91
126.97
123.55
120.12
116.37
77.16 CDCl3
33.17
17.98
F_3C
N
O
S
Bi
Et
117, ^{13}C NMR, $CDCl_3$
200 190 180 170 160 150 140 130 120 110 100 90 80 70 60 50 40 30 20 10 0
f1 (ppm)

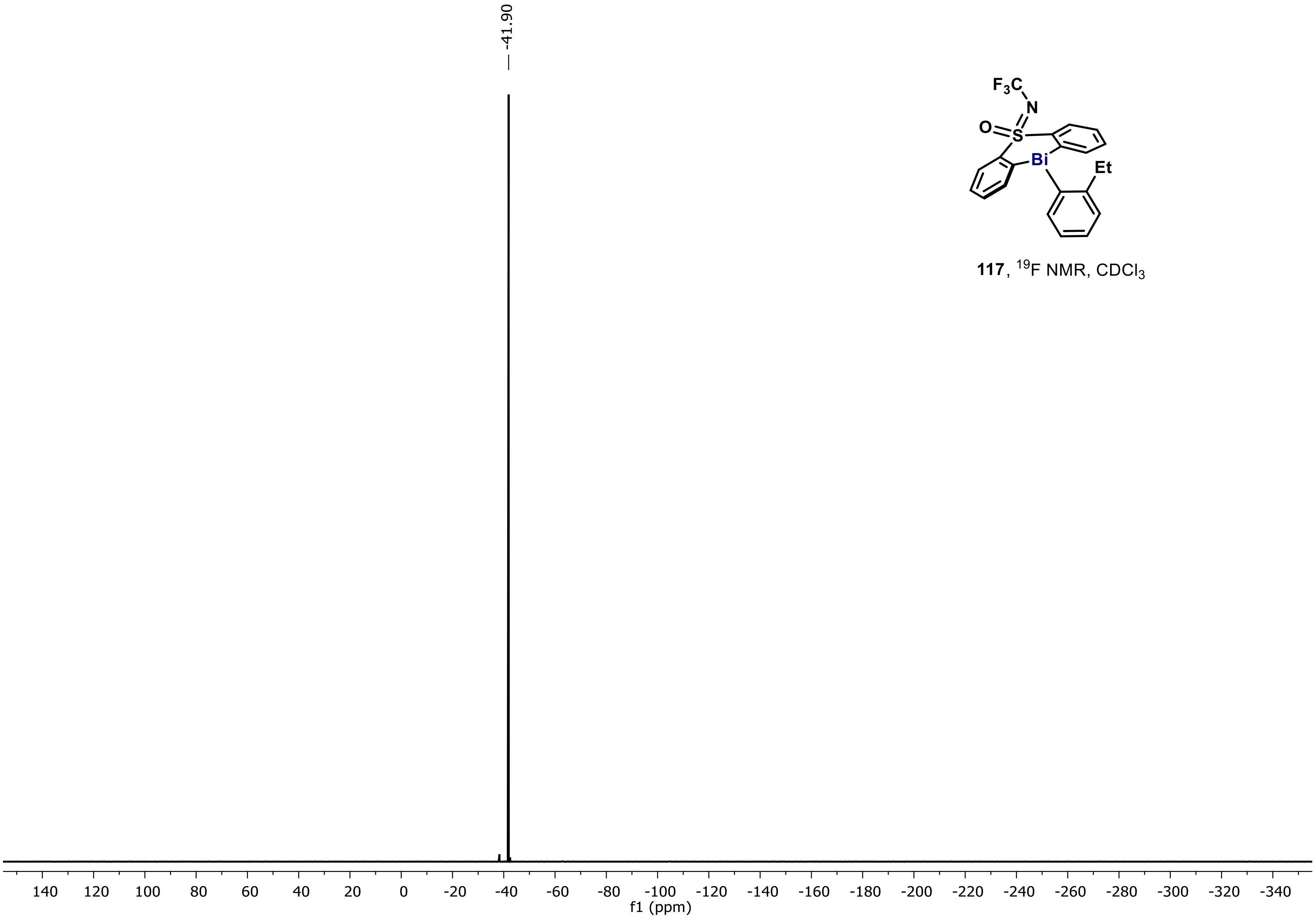

117, ^{19}F NMR, $CDCl_3$

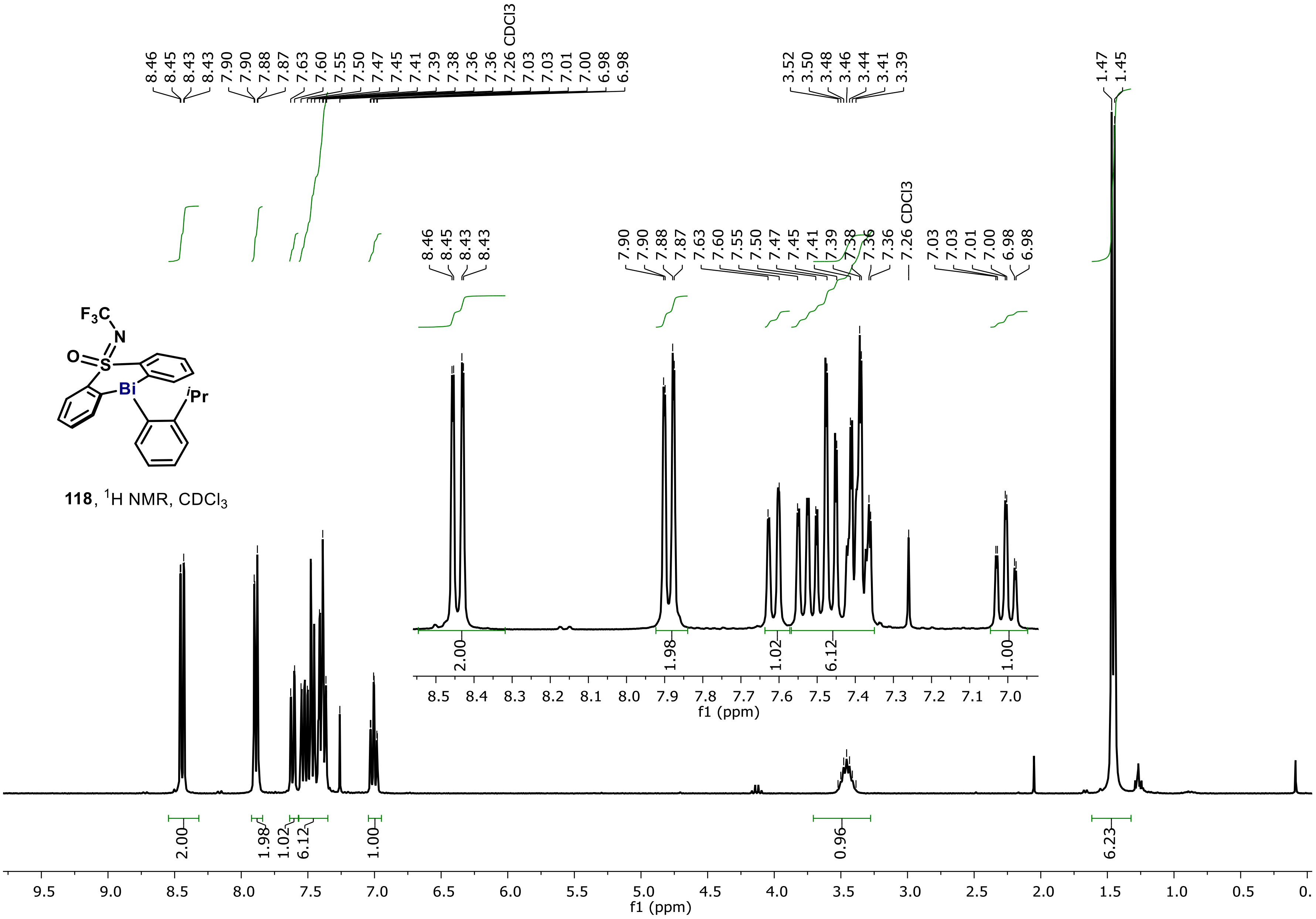

F3C
N
O
S
Bi
iPr
118, 1H NMR, CDCl3
8.46 8.45 8.43 8.43 7.90 7.90 7.88 7.87 7.63 7.60 7.55 7.50 7.47 7.45 7.41 7.39 7.38 7.36 7.36 7.26 CDCl3 7.03 7.03 7.01 7.00 6.98 6.98
3.52 3.50 3.48 3.46 3.44 3.41 3.39
1.47 1.45
2.00
1.98
1.02
6.12
1.00
0.96
6.23
f1 (ppm)

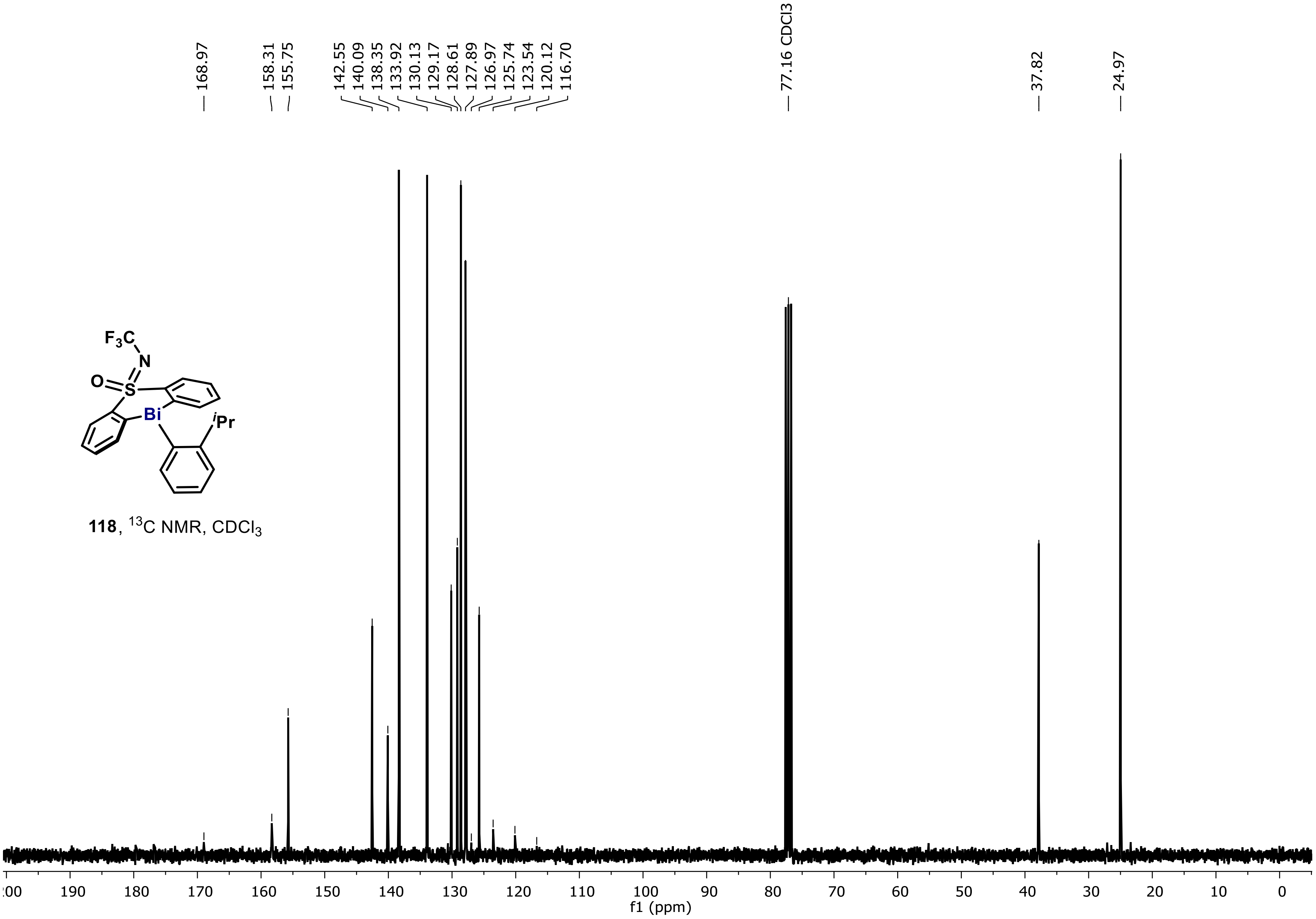

168.97
158.31
155.75
142.55
140.09
138.35
133.92
130.13
129.17
128.61
127.89
126.97
125.74
123.54
120.12
116.70
77.16 CDCl3
37.82
24.97
F3C
N
O
S
Bi
iPr
118, 13C NMR, CDCl3
200
190
180
170
160
150
140
130
120
110
100
90
80
70
60
50
40
30
20
10
0
f1 (ppm)

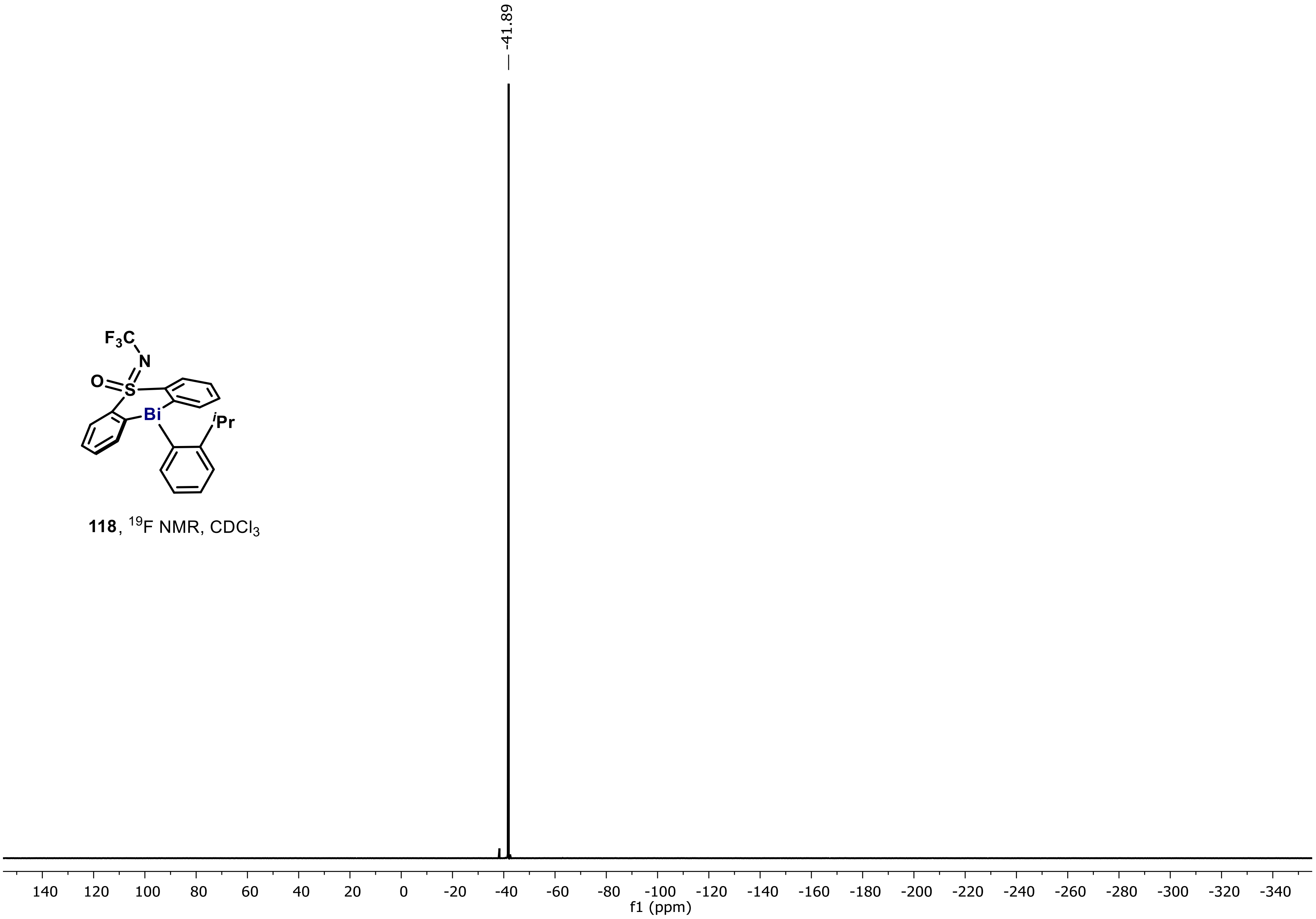

118, ^{19}F NMR, $CDCl_3$

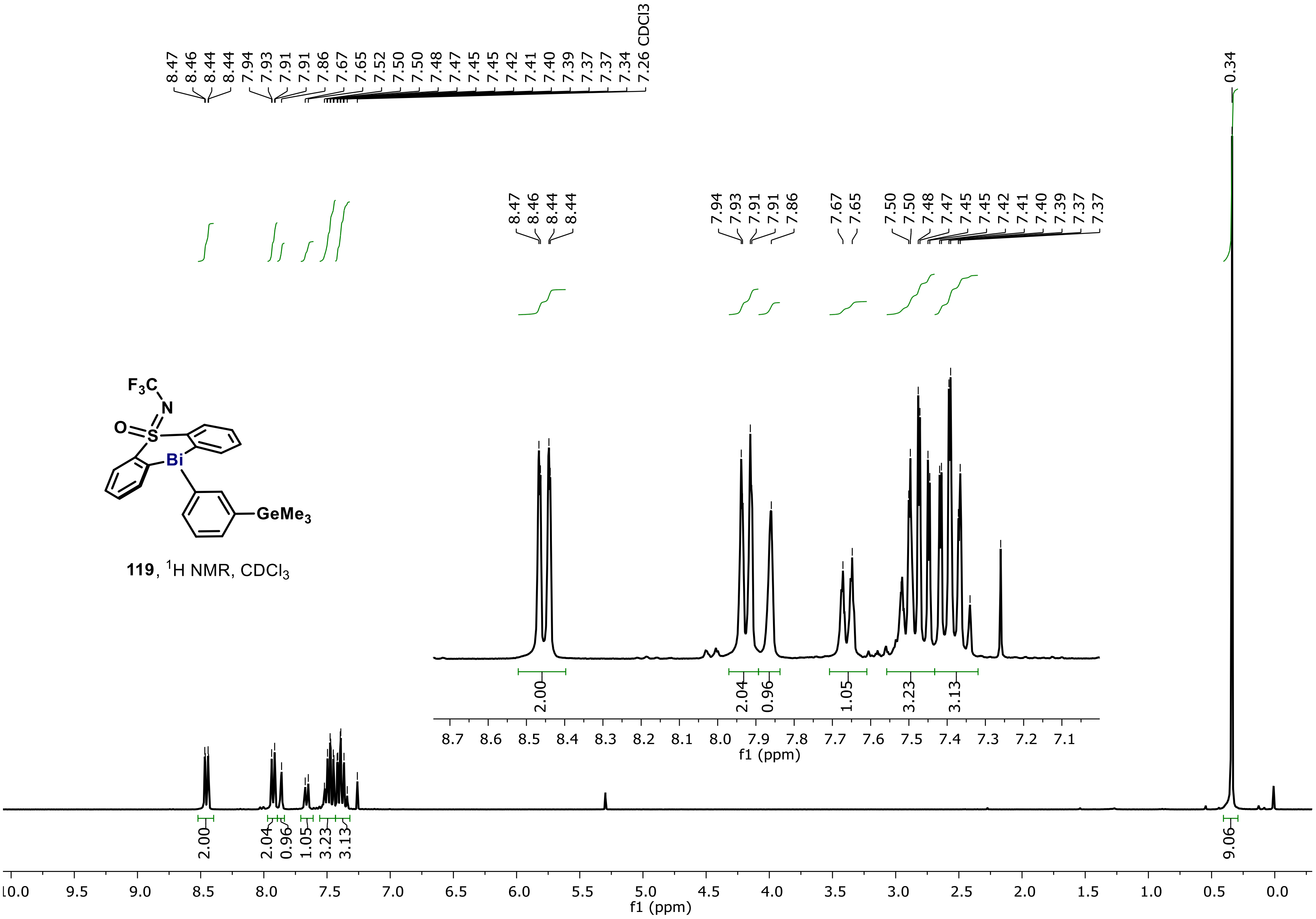

119, ^{1}H NMR, $CDCl_3$

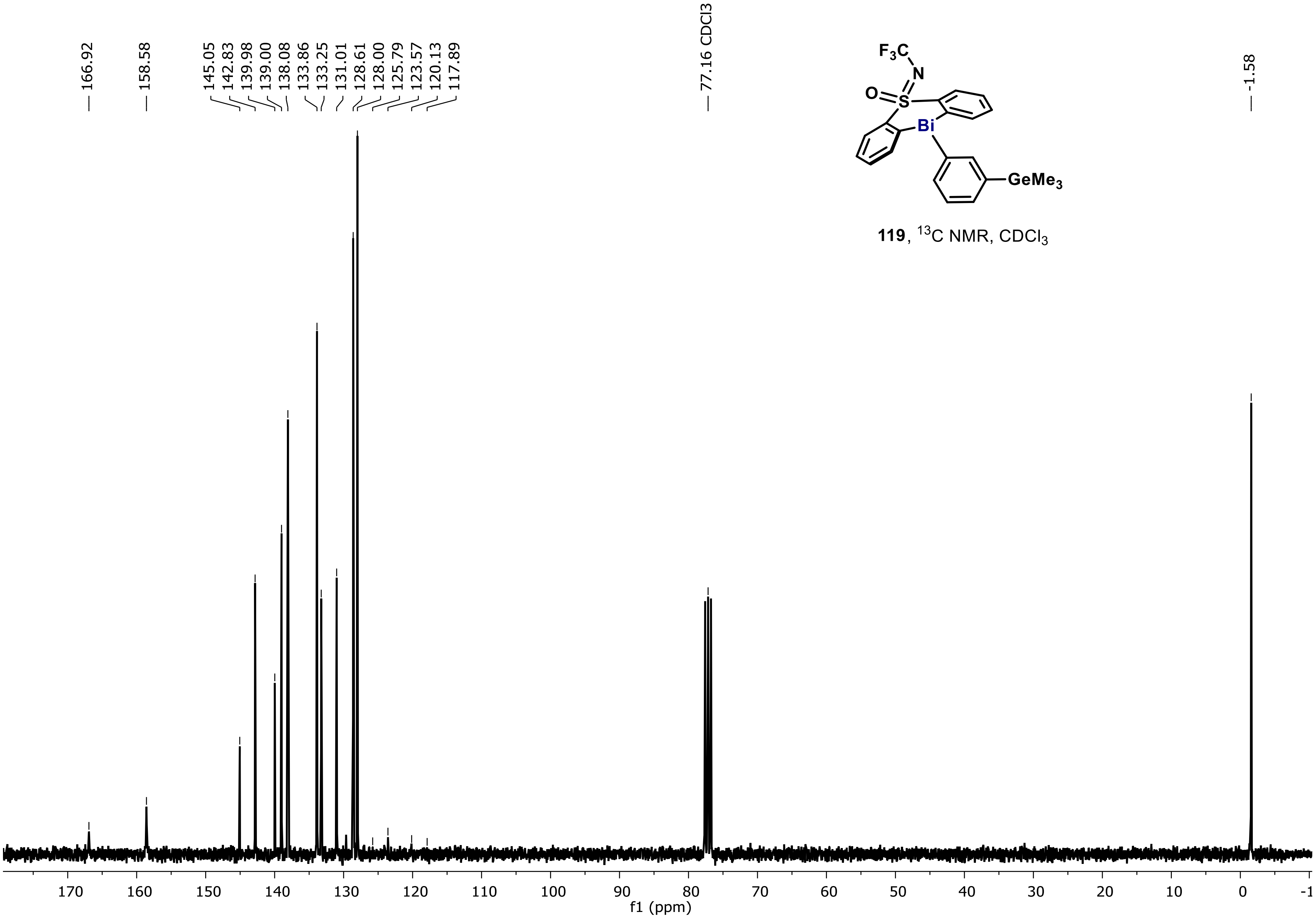
166.92
158.58
145.05
142.83
139.98
139.00
138.08
133.86
133.25
131.01
128.61
128.00
125.79
123.57
120.13
117.89
77.16 CDCl3
-1.58
F3C
N
O
S
Bi
GeMe3
119, 13C NMR, CDCl3
f1 (ppm)

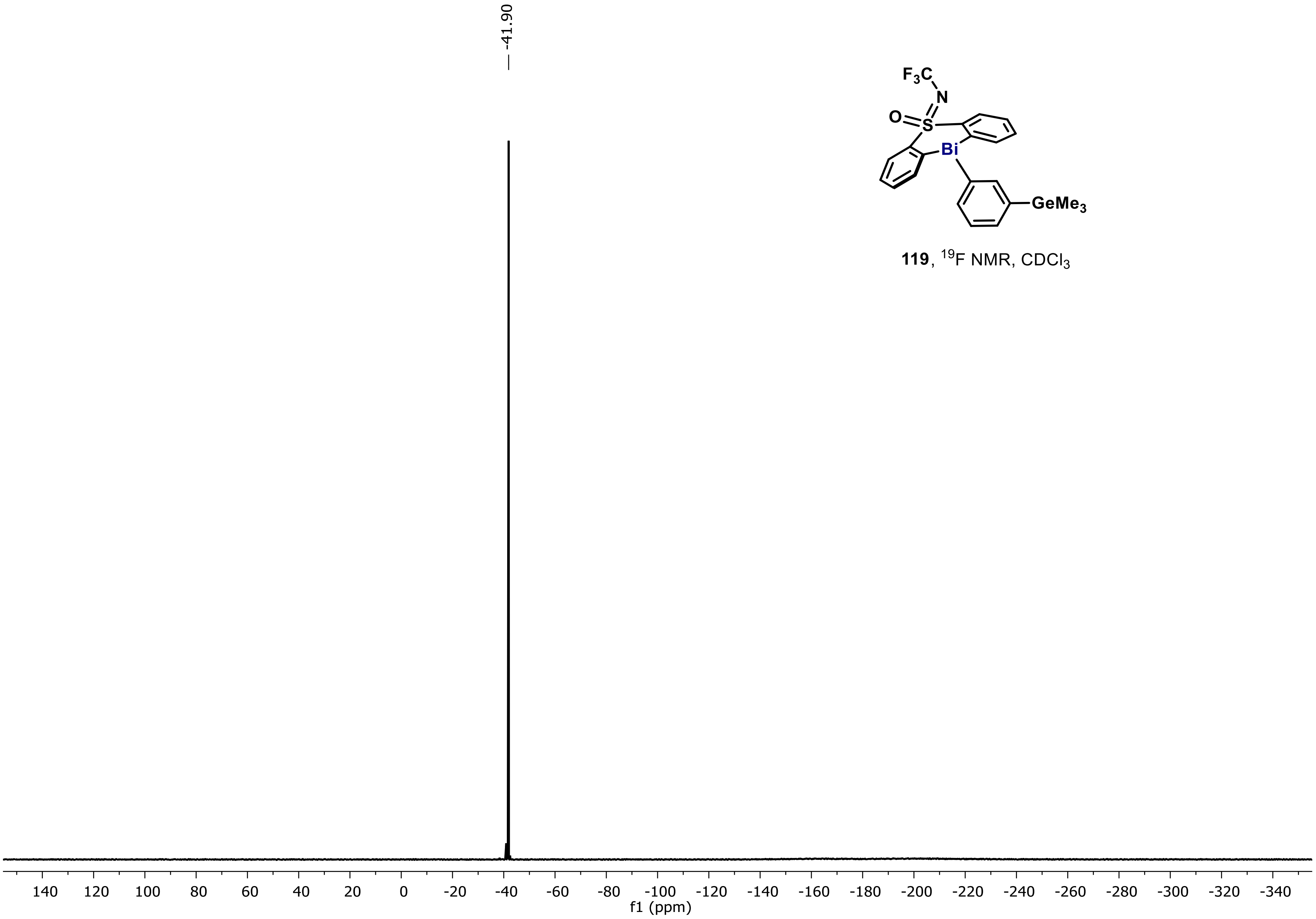

119, ^{19}F NMR, $CDCl_3$

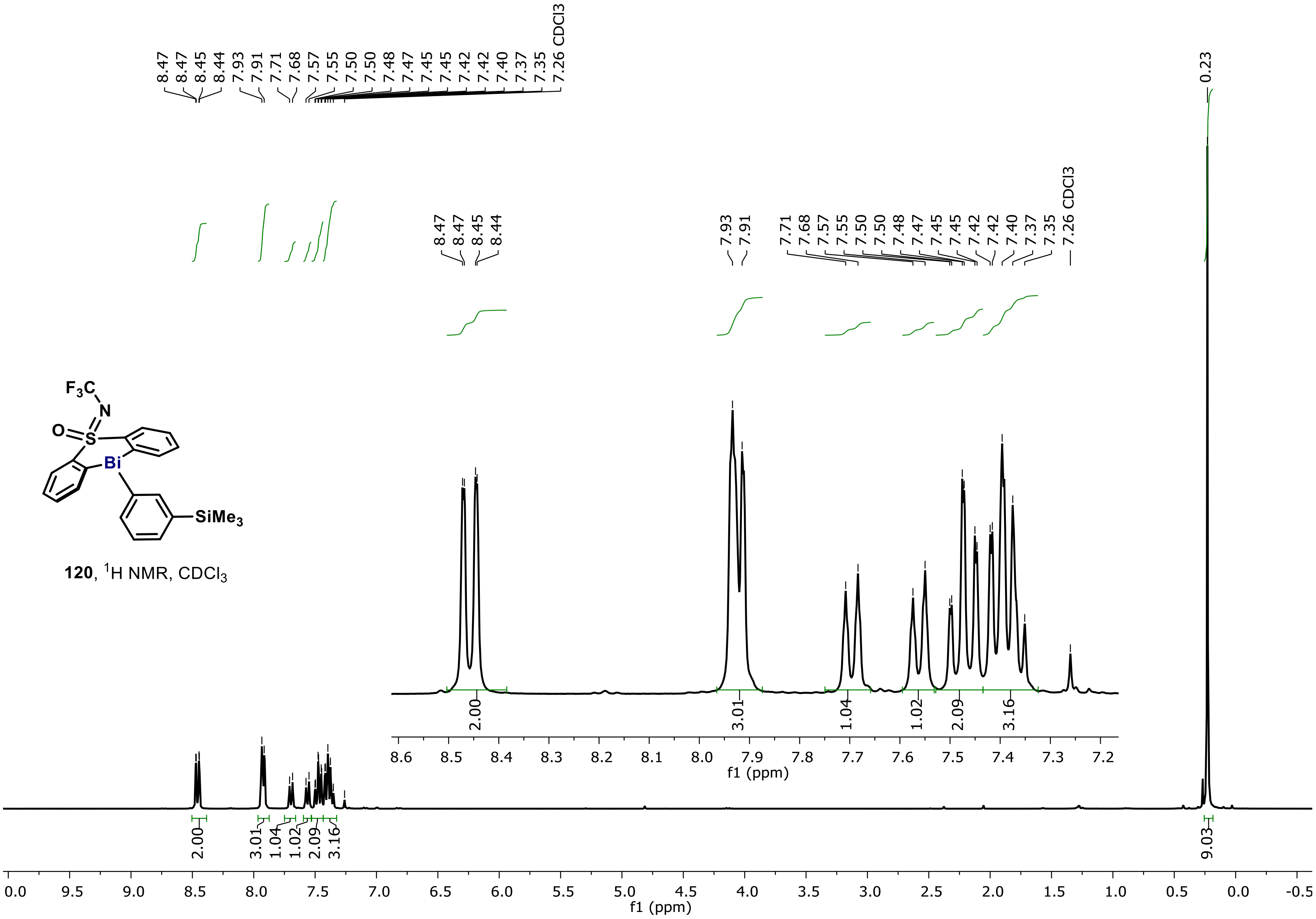

120, ^{1}H NMR, $CDCl_3$

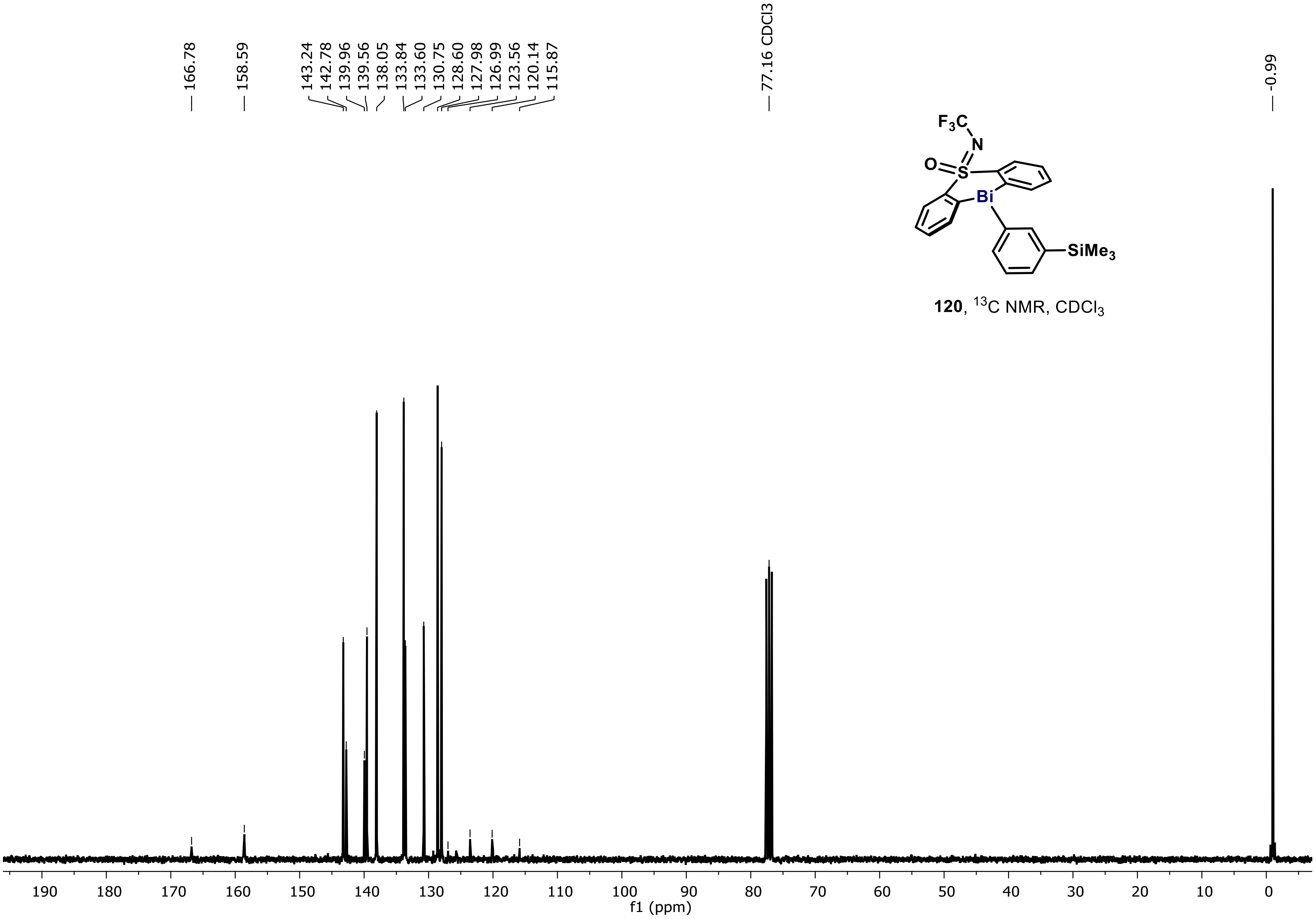

120, ^{13}C NMR, $CDCl_3$

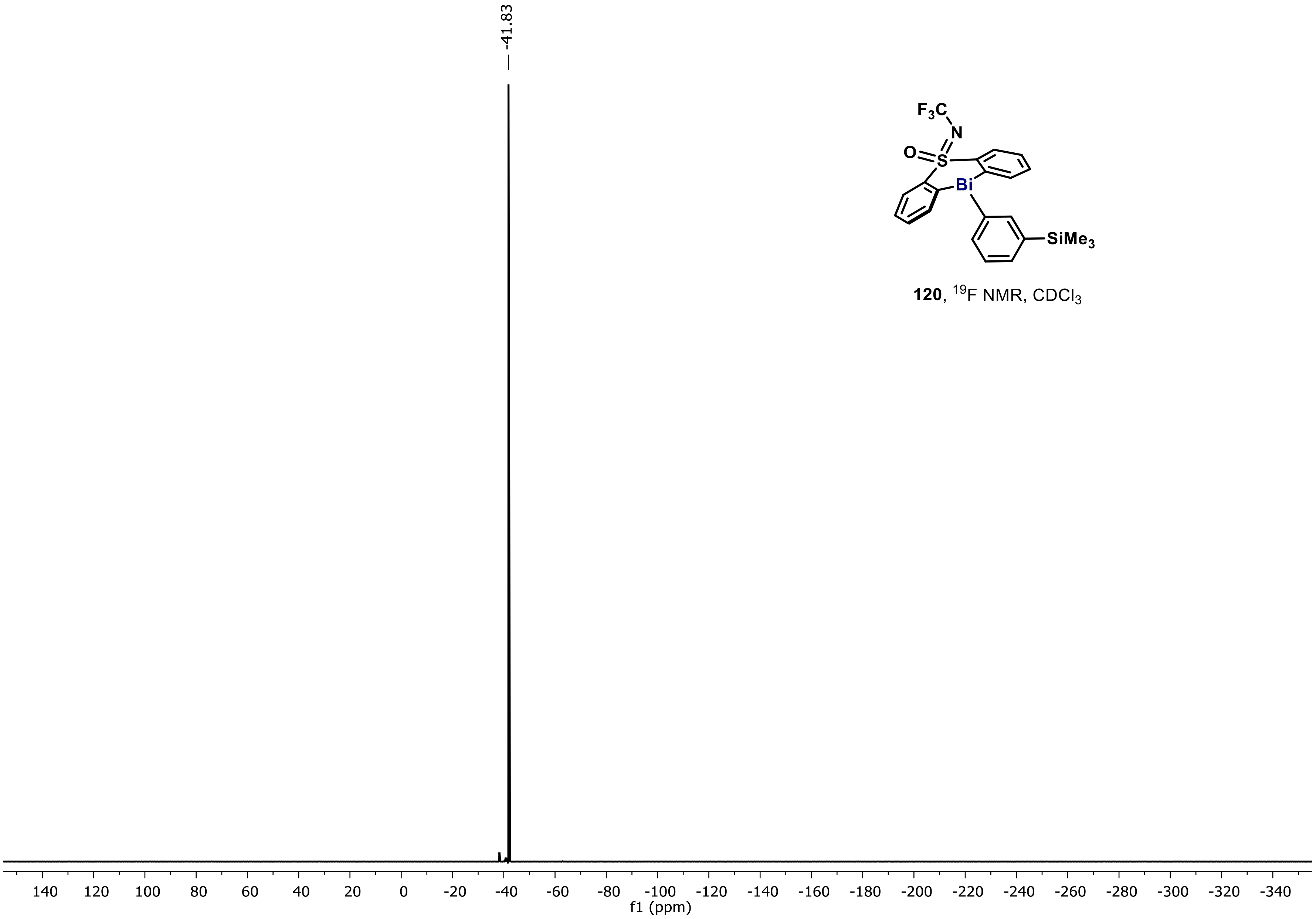

120, ^{19}F NMR, $CDCl_3$

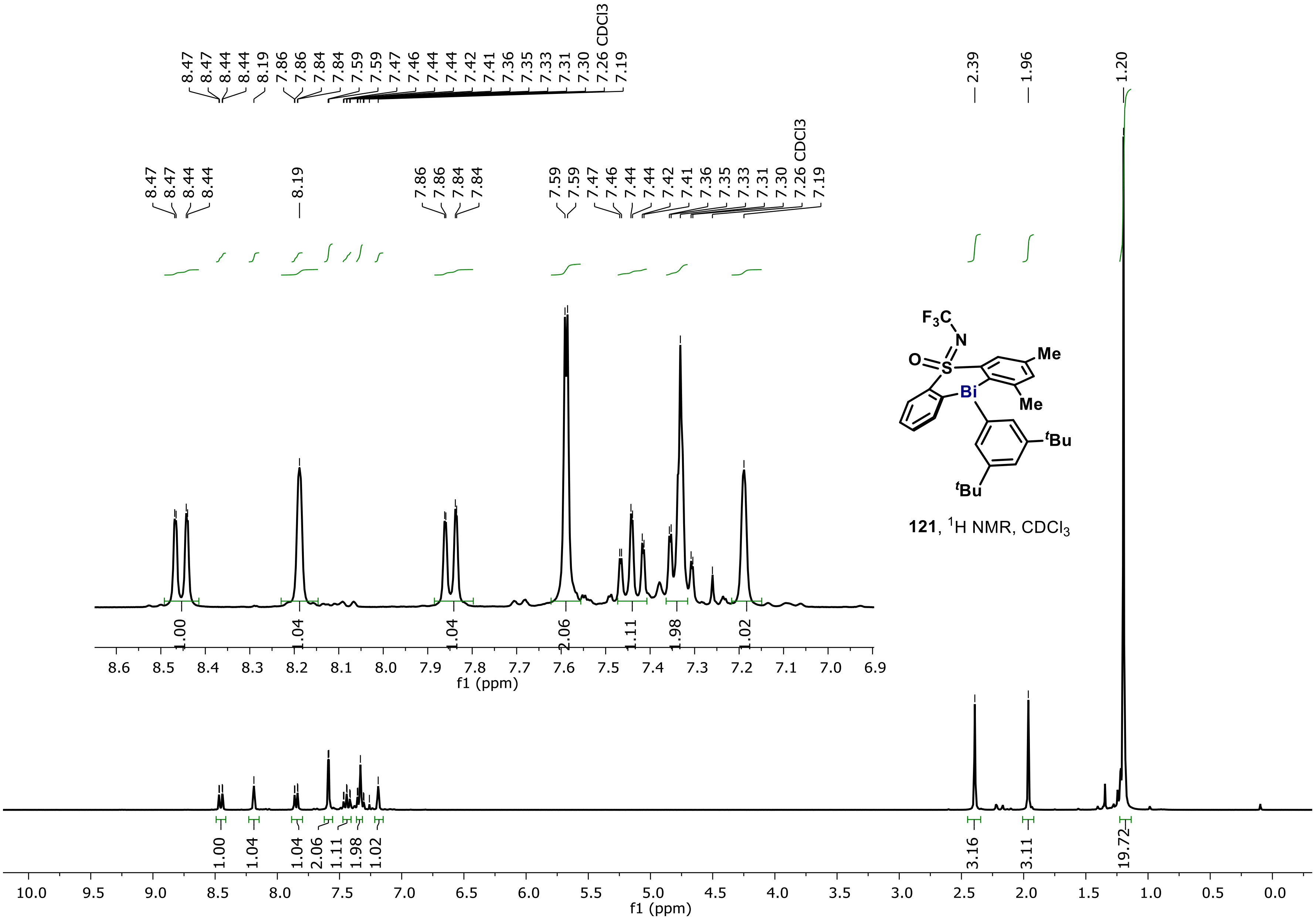

121, ^{1}H NMR, $CDCl_3$

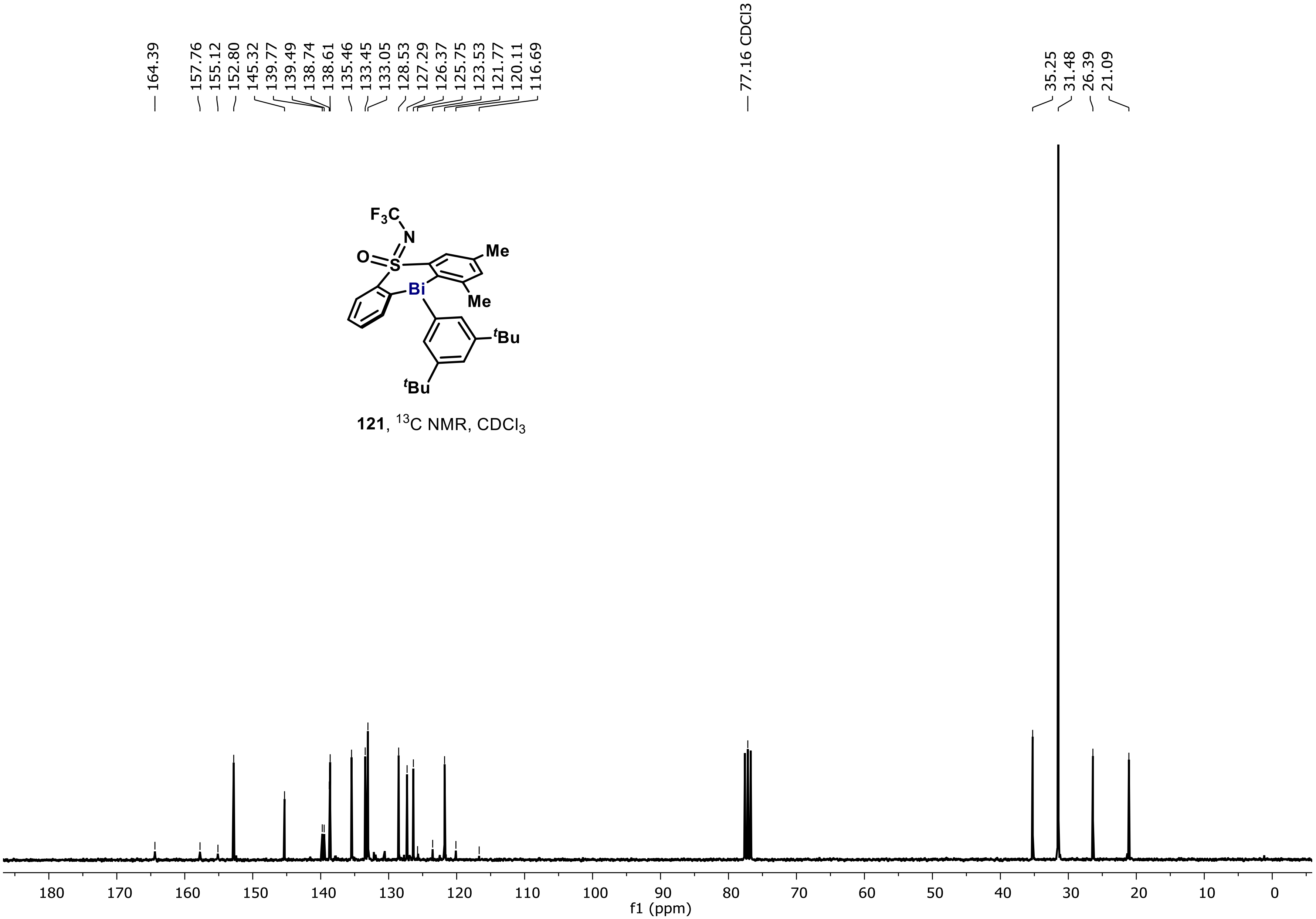

121, ^{13}C NMR, $CDCl_3$

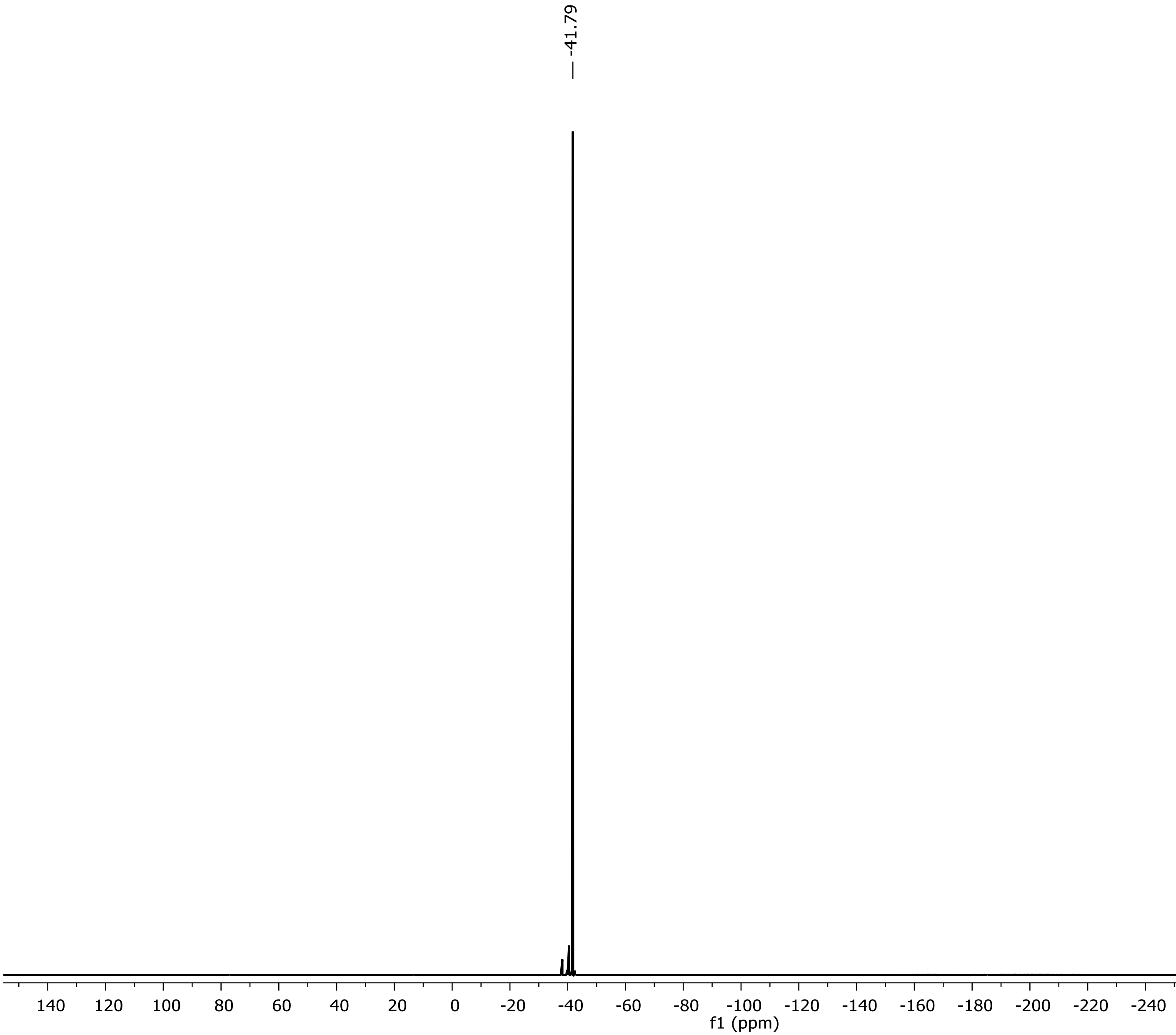

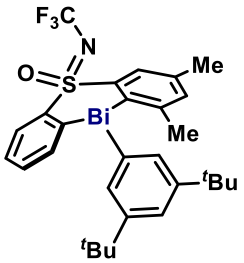

121, ^{19}F NMR, $CDCl_3$

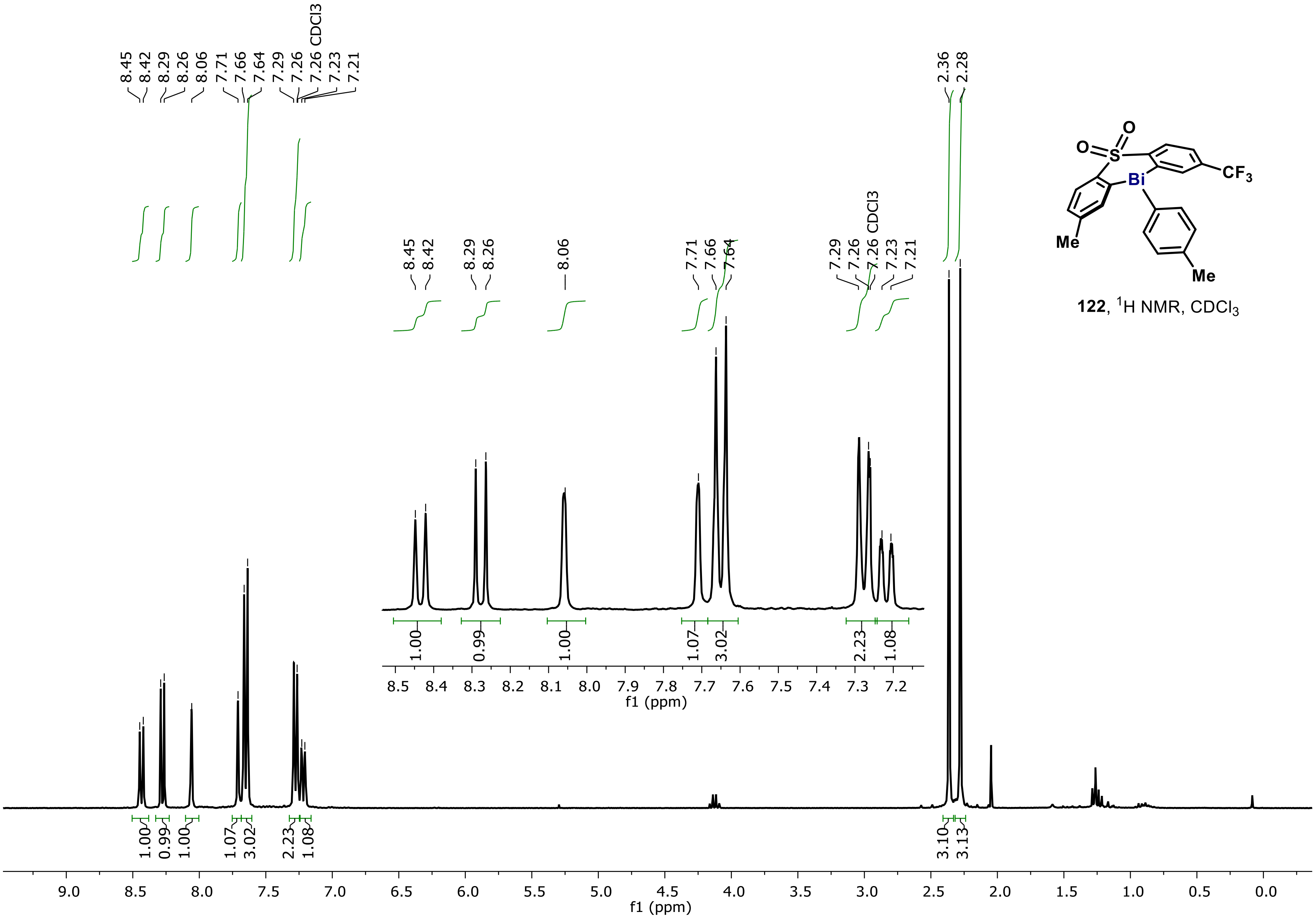

122, ^{1}H NMR, $CDCl_3$

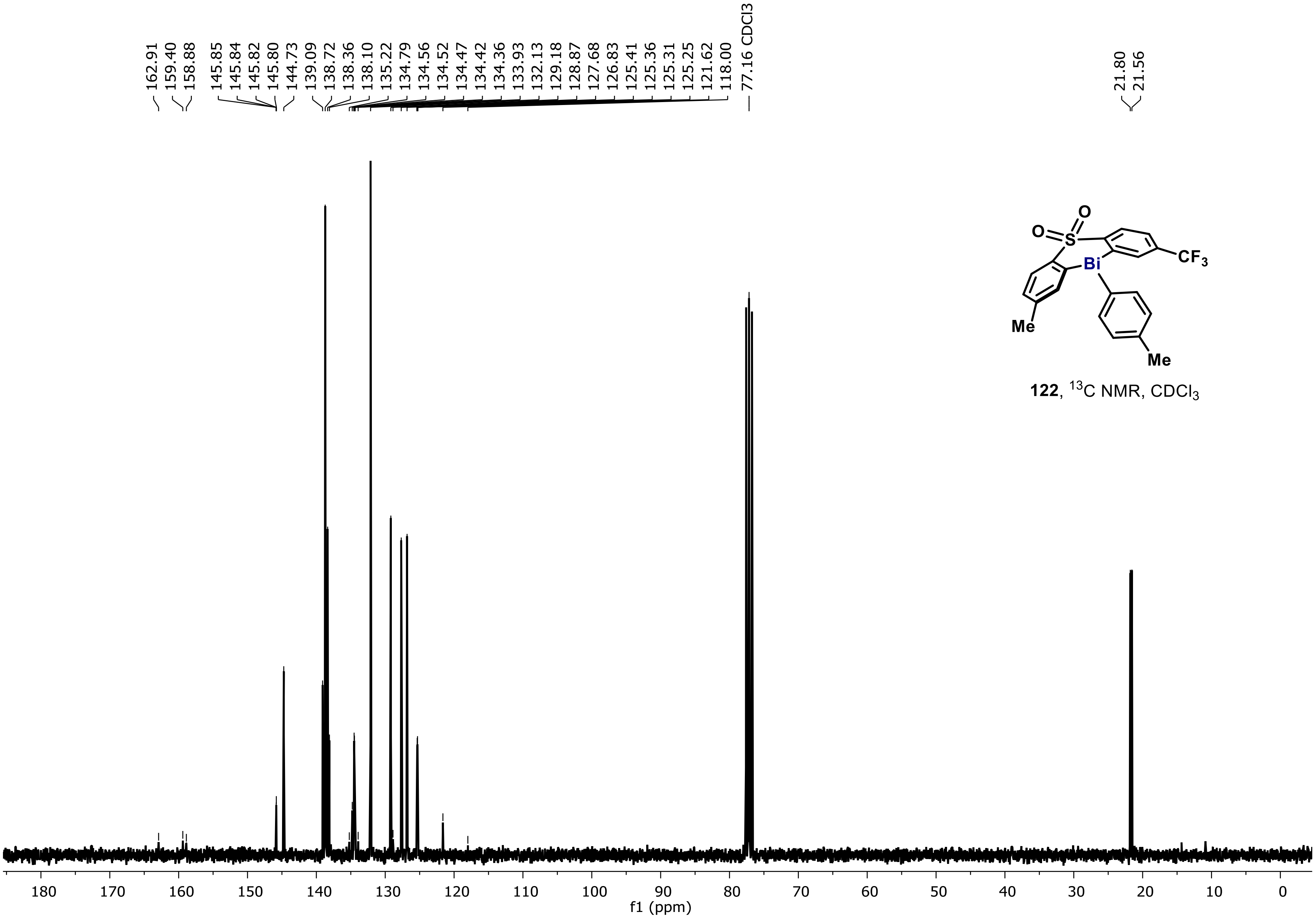
162.91
159.40
158.88
145.85
145.84
145.82
145.80
144.73
139.09
138.72
138.36
138.10
135.22
134.79
134.56
134.52
134.47
134.42
134.36
133.93
132.13
129.18
128.87
127.68
126.83
125.41
125.36
125.31
125.25
121.62
118.00
77.16 CDCl3
21.80
21.56
O
O
S
Bi
CF3
Me
Me
122, 13C NMR, CDCl3
180
170
160
150
140
130
120
110
100
90
80
70
60
50
40
30
20
10
0
f1 (ppm)

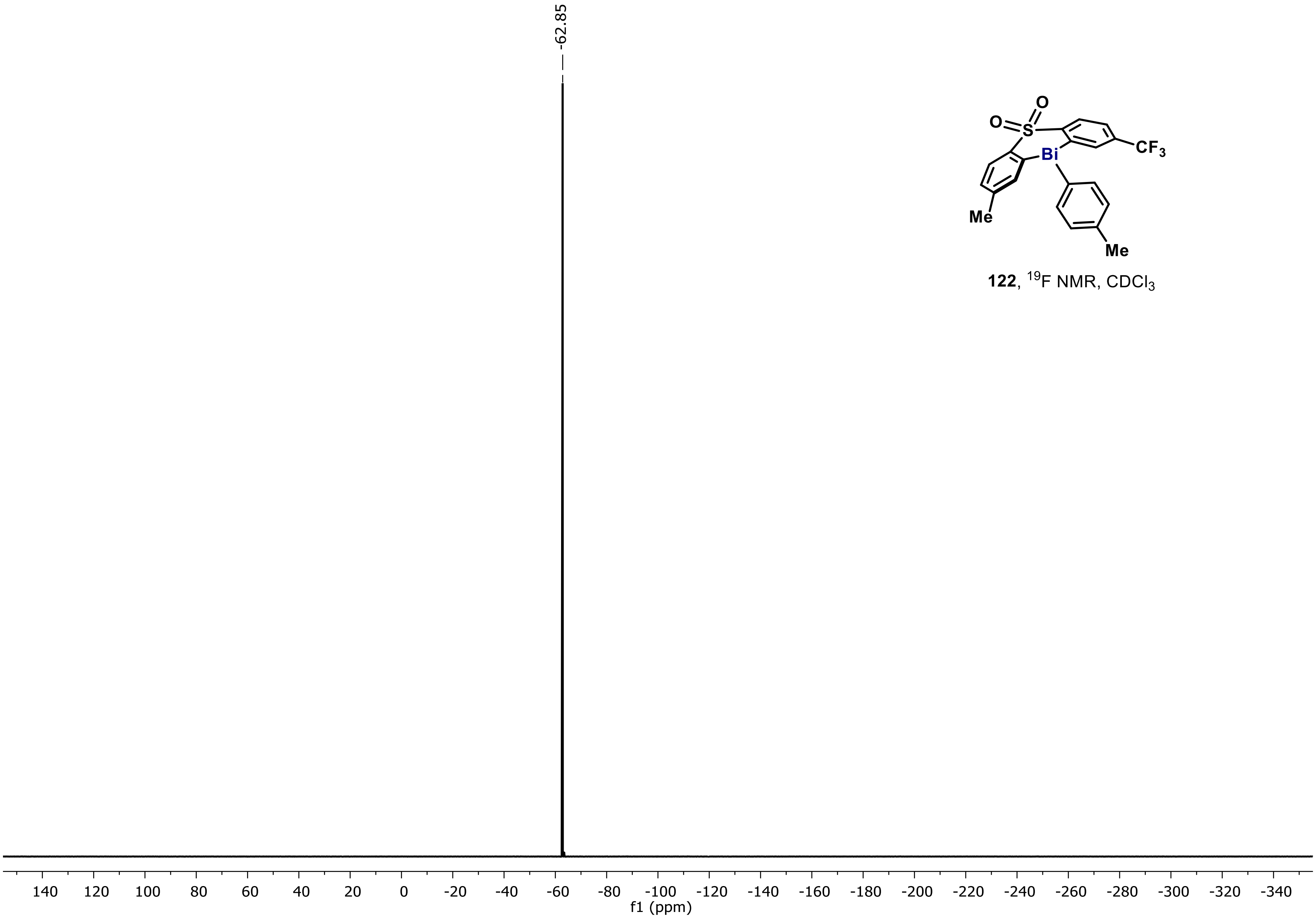

122, ^{19}F NMR, $CDCl_3$

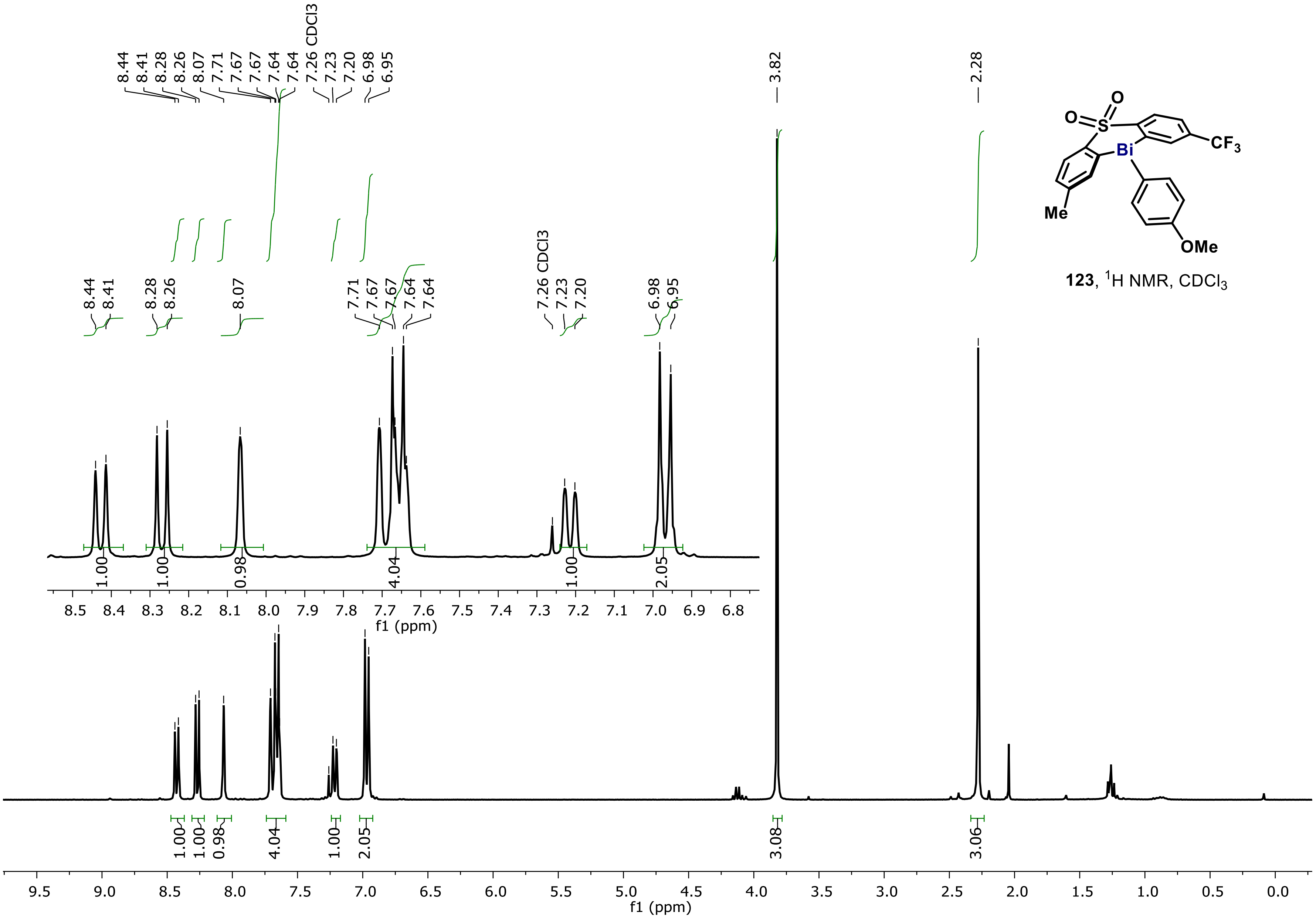

123, ^{1}H NMR, $CDCl_3$

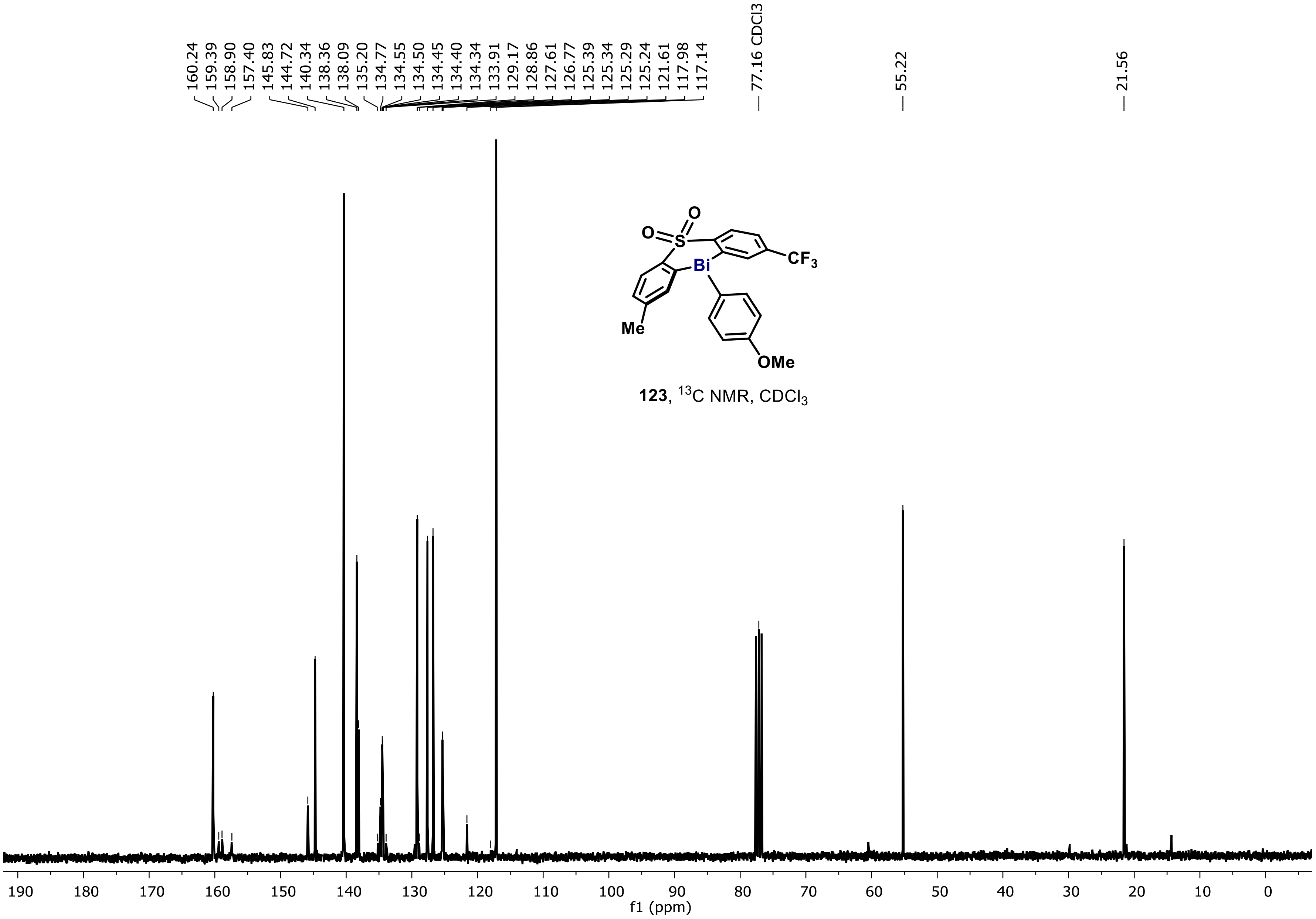

123, ^{13}C NMR, $CDCl_3$

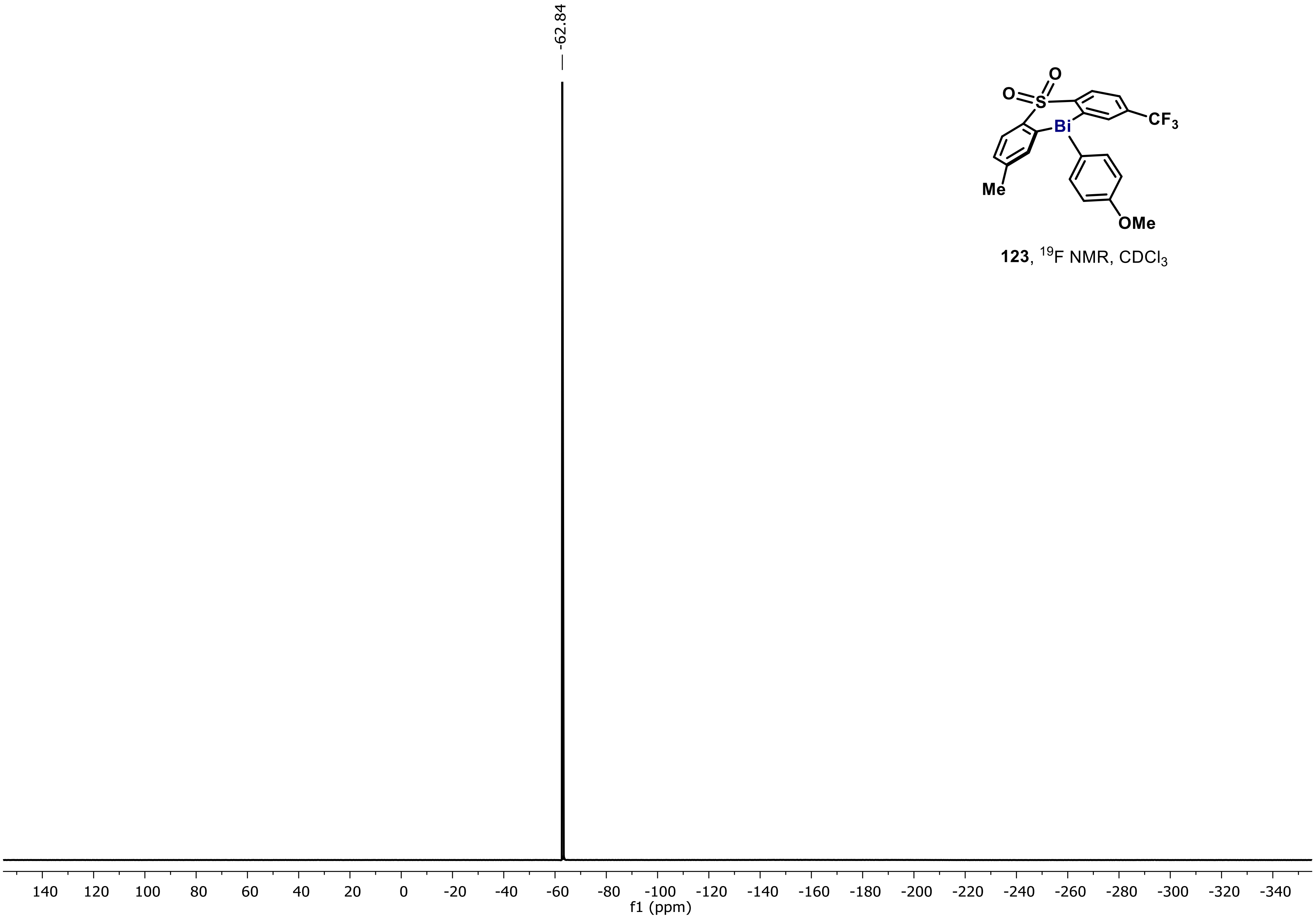

123, ^{19}F NMR, $CDCl_3$

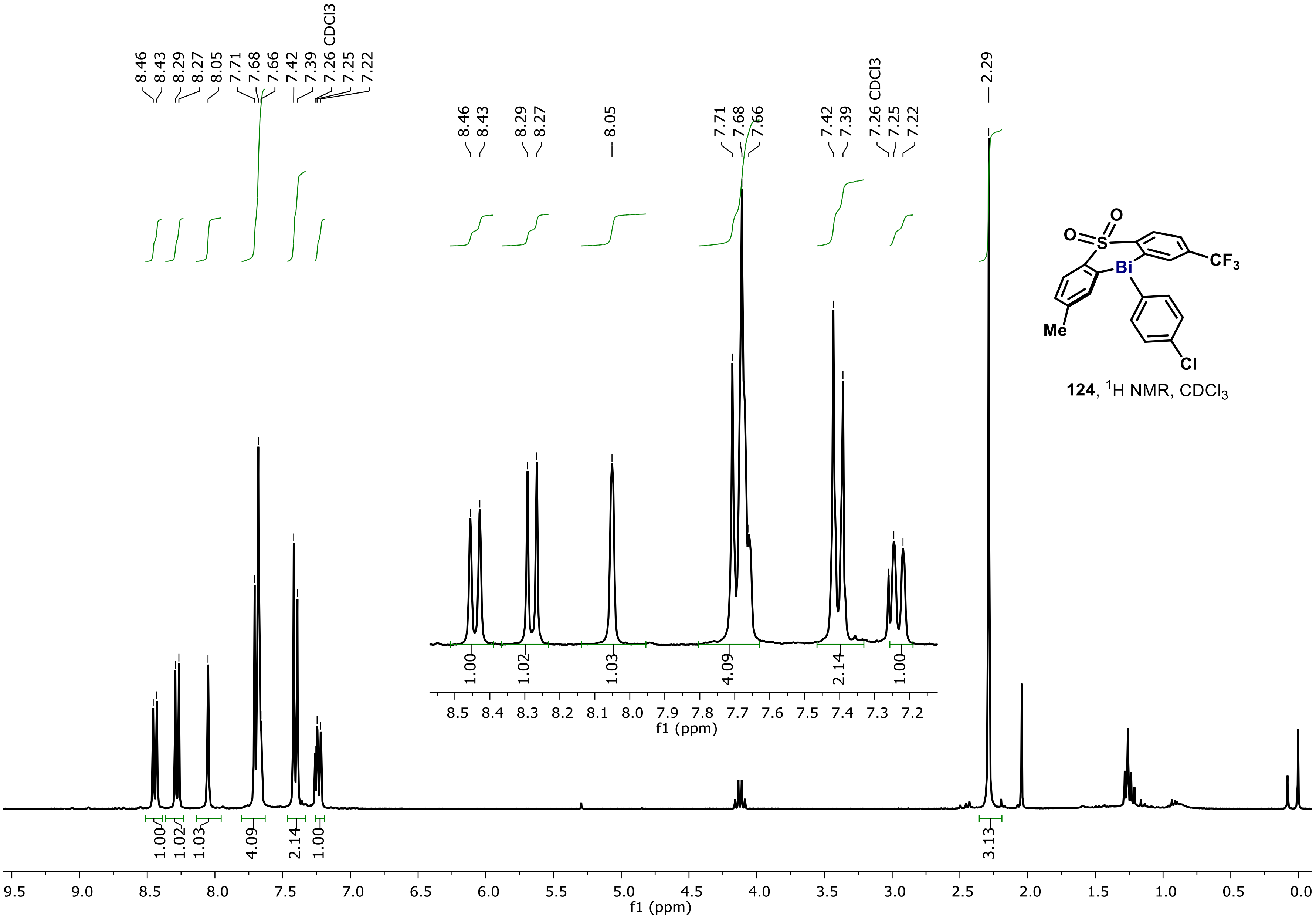

124, ^{1}H NMR, $CDCl_3$

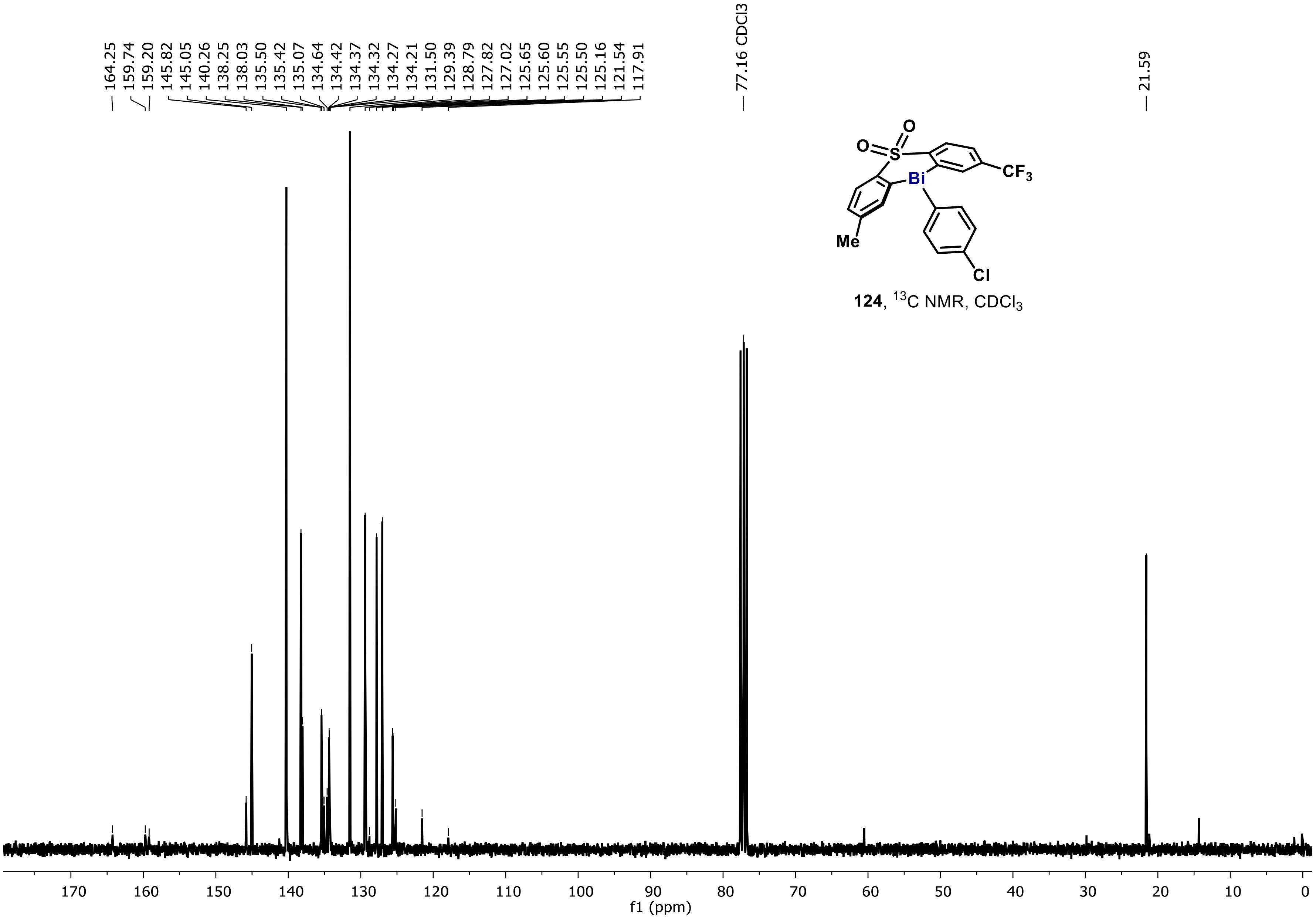

124, ^{13}C NMR, $CDCl_3$

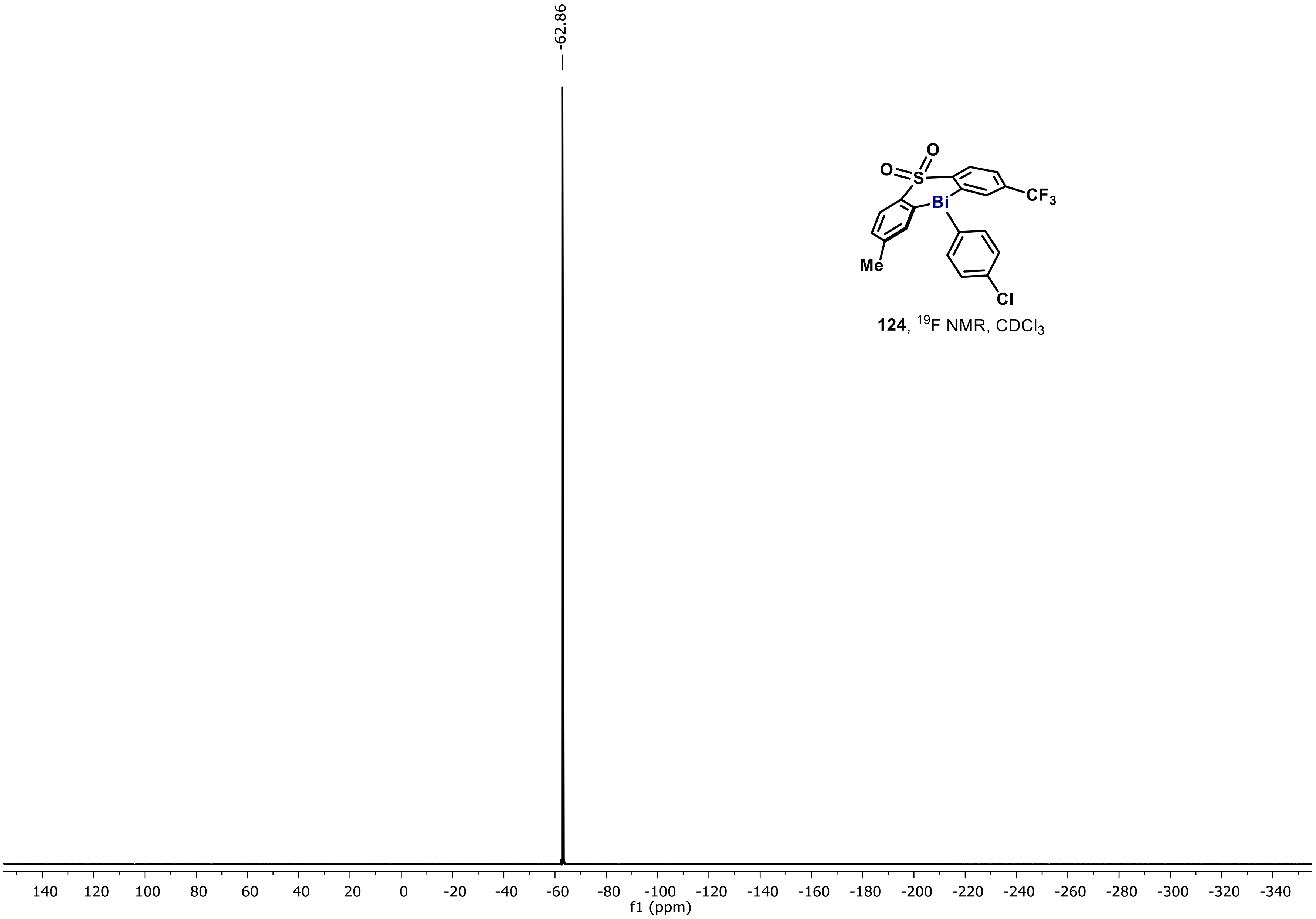

124, ^{19}F NMR, $CDCl_3$

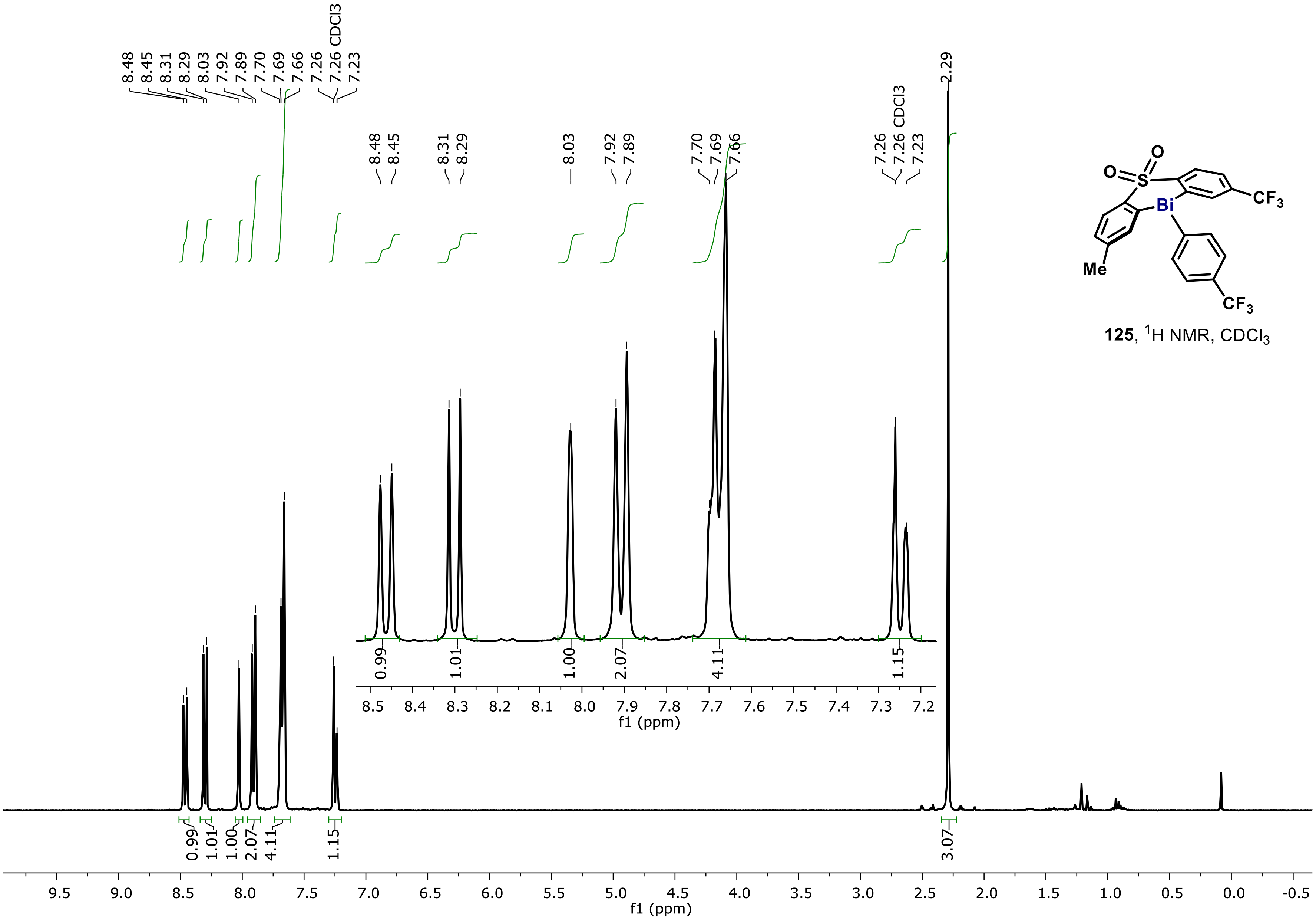

125, ^{1}H NMR, $CDCl_3$

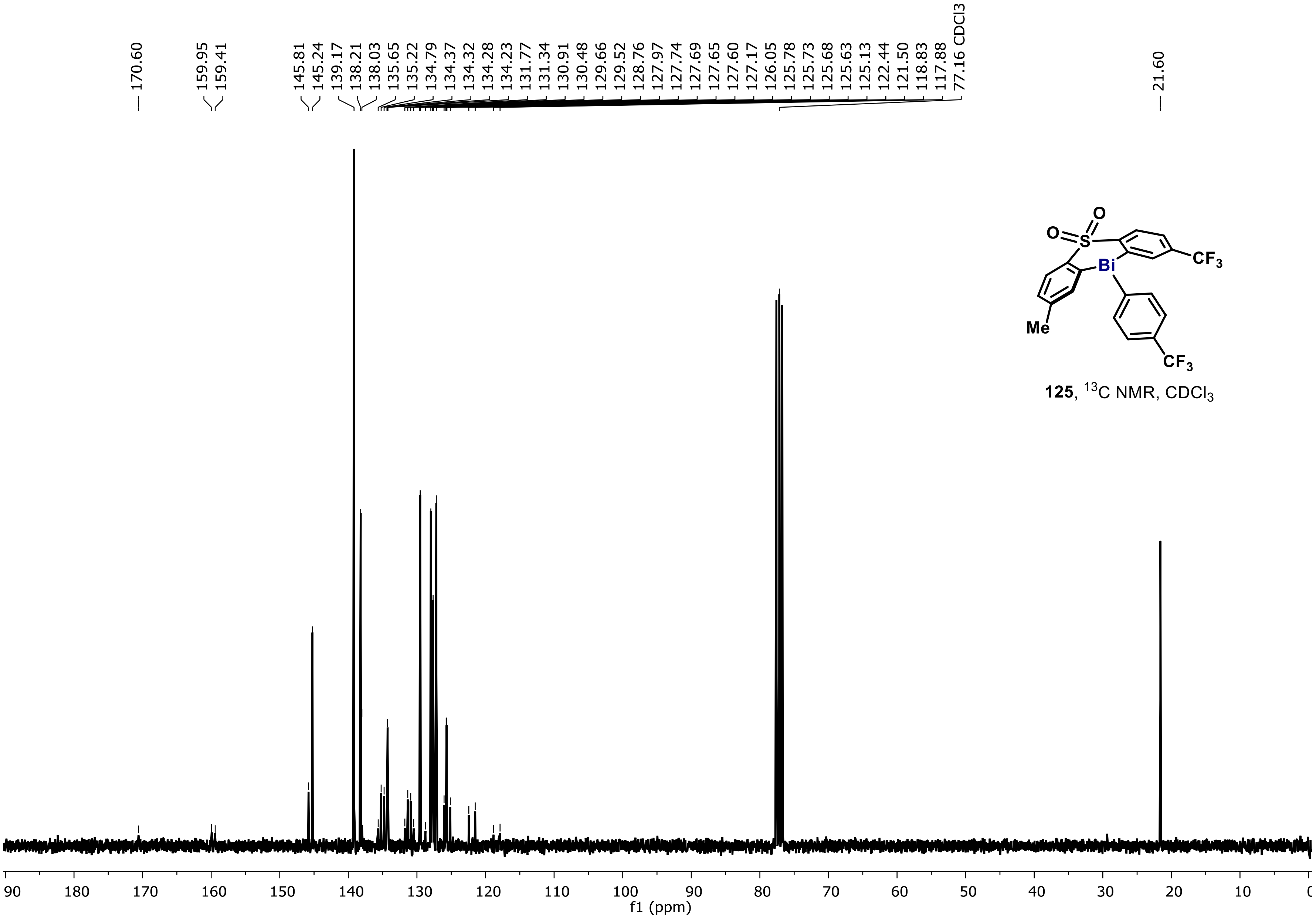

125, ^{13}C NMR, $CDCl_3$

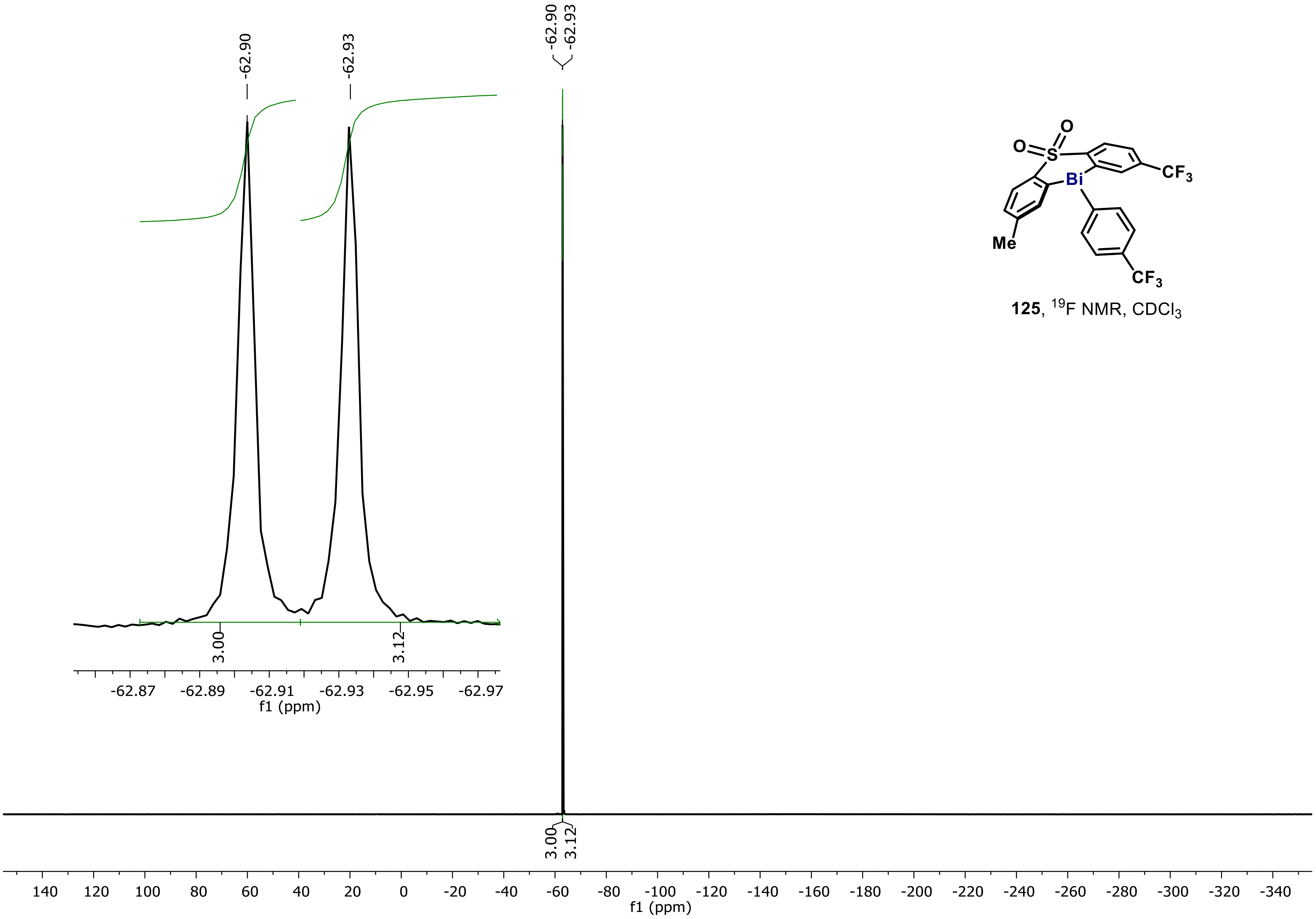

125, ^{19}F NMR, $CDCl_3$

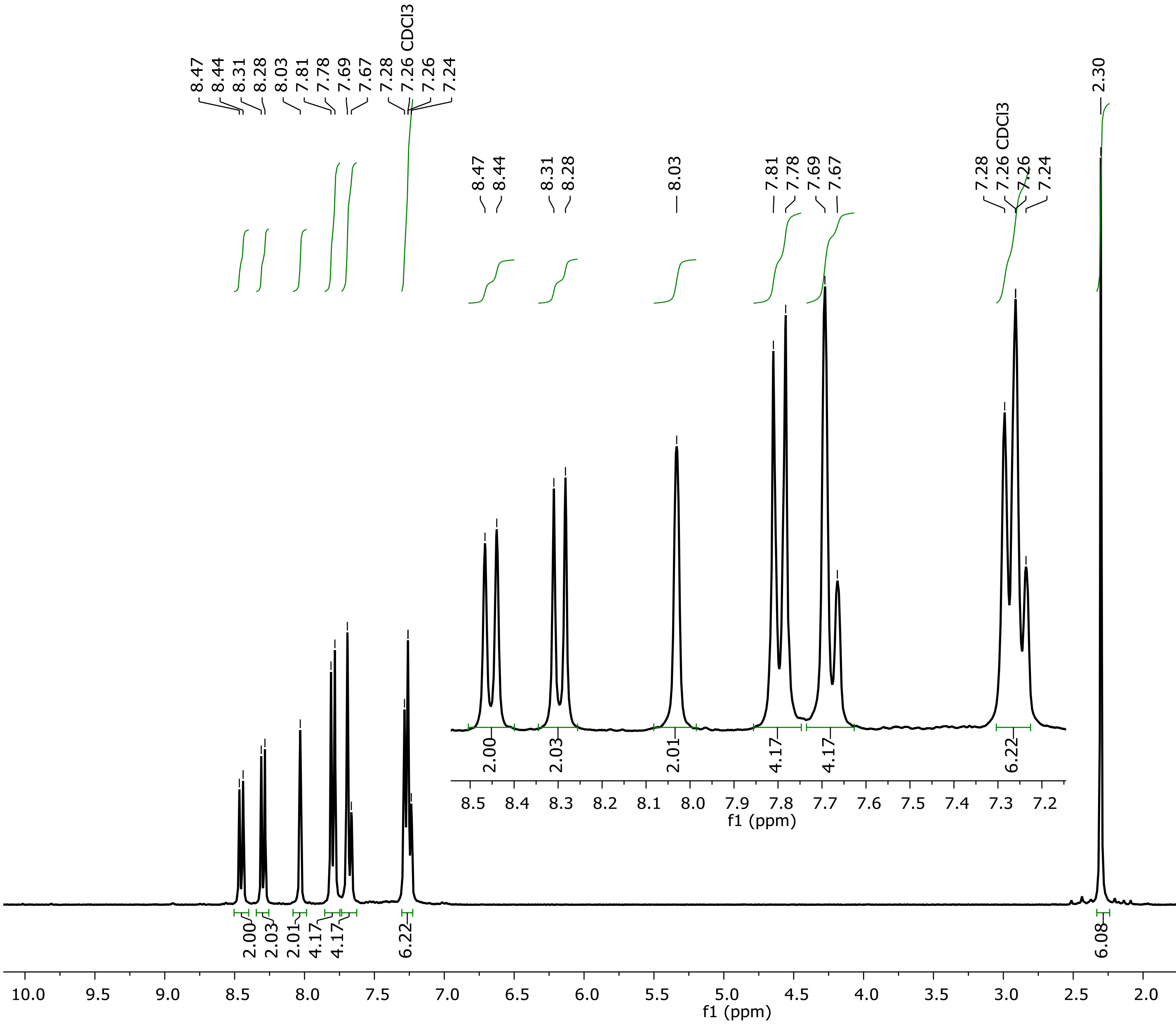

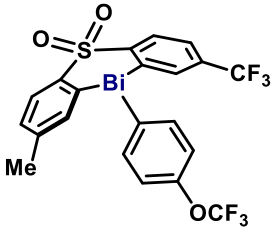

126, ^{1}H NMR, $CDCl_3$

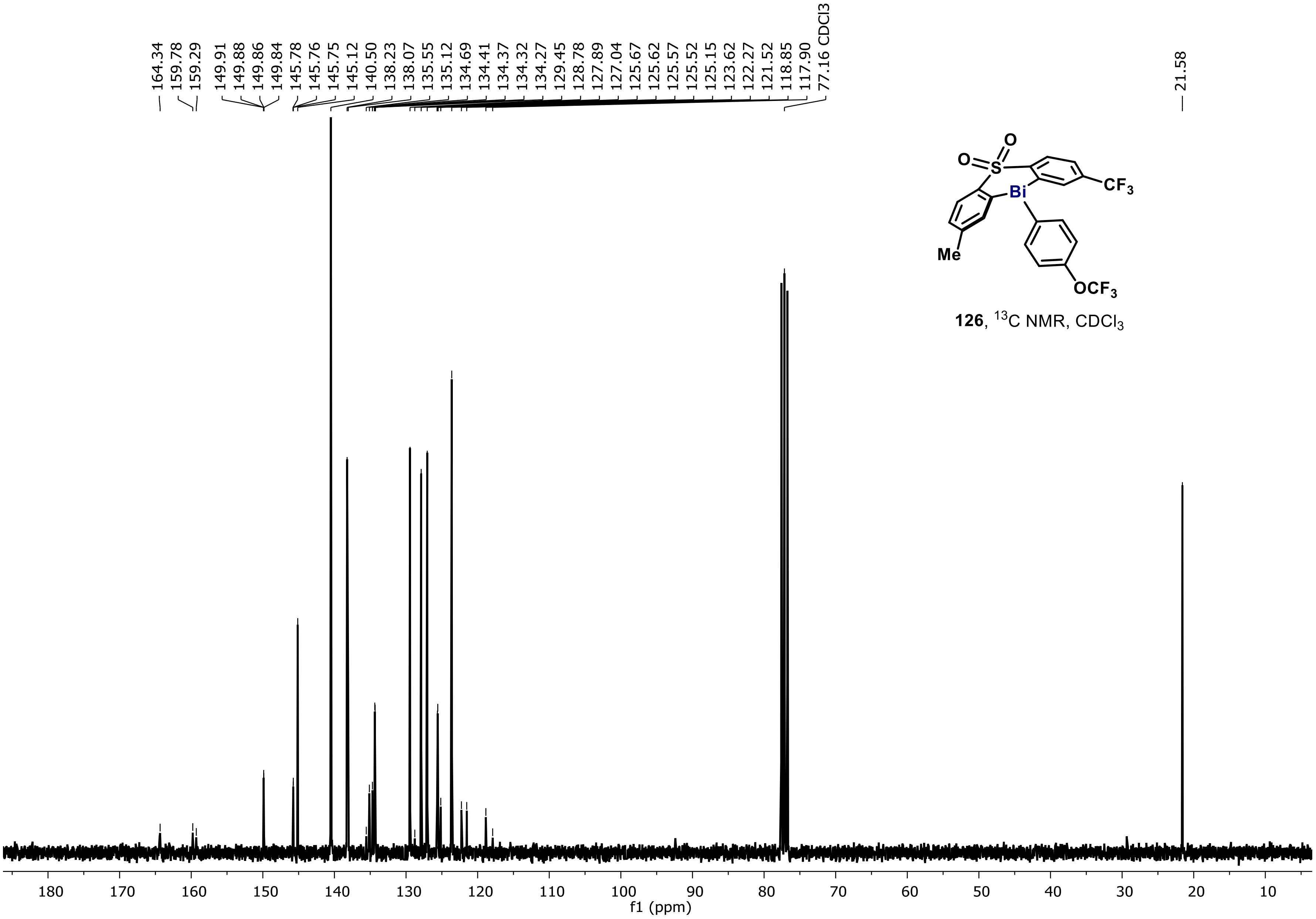
164.34
159.78
159.29
149.91
149.88
149.86
149.84
145.78
145.76
145.75
145.12
140.50
138.23
138.07
135.55
135.12
134.69
134.41
134.37
134.32
134.27
129.45
128.78
127.89
127.04
125.67
125.62
125.57
125.52
125.15
123.62
122.27
121.52
118.85
117.90
77.16 CDCl3
21.58
O
O
S
Bi
CF3
Me
OCF3
126, 13C NMR, CDCl3
180 170 160 150 140 130 120 110 100 90 80 70 60 50 40 30 20 10
f1 (ppm)

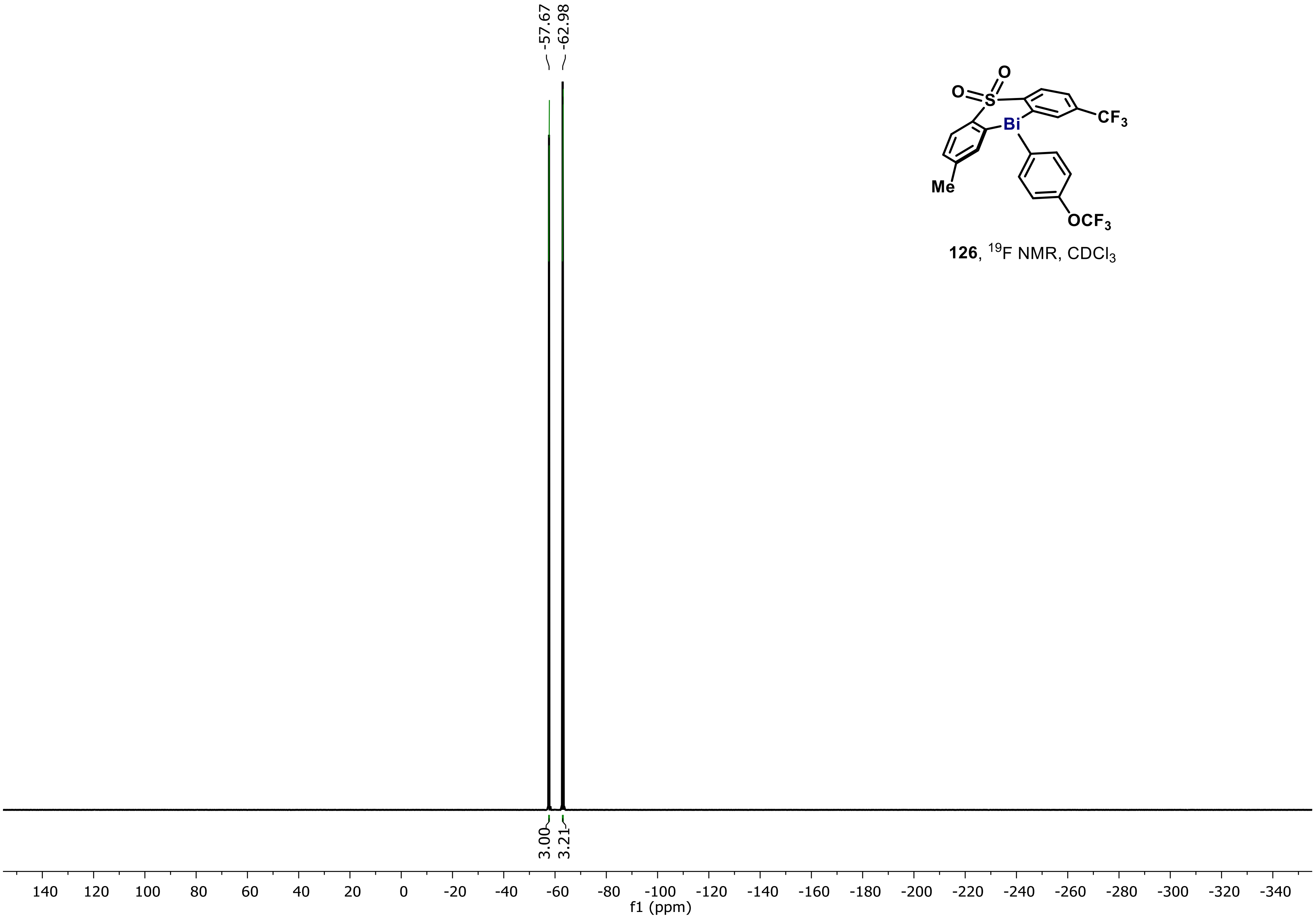

126, ^{19}F NMR, $CDCl_3$

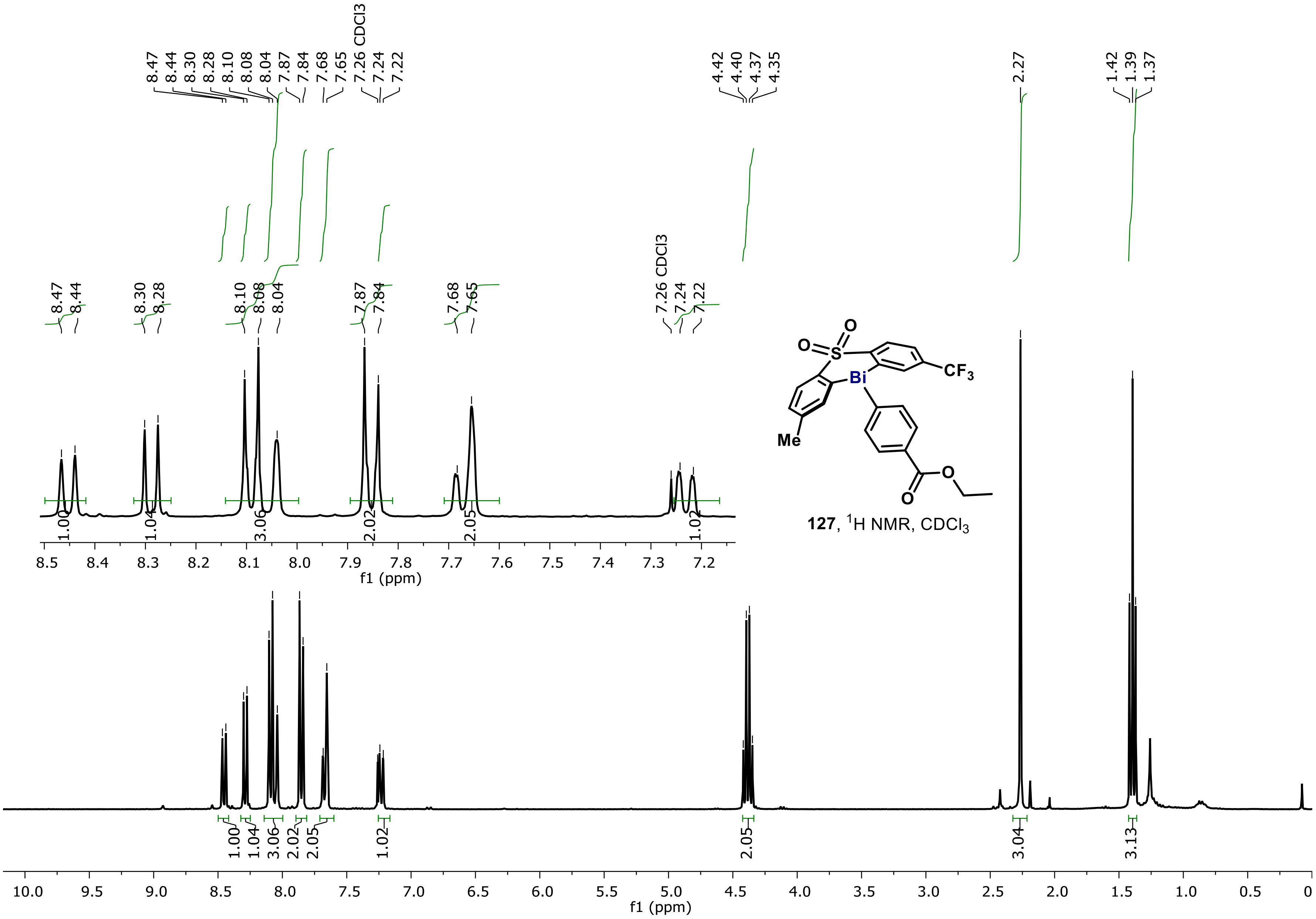

127, ^{1}H NMR, $CDCl_3$

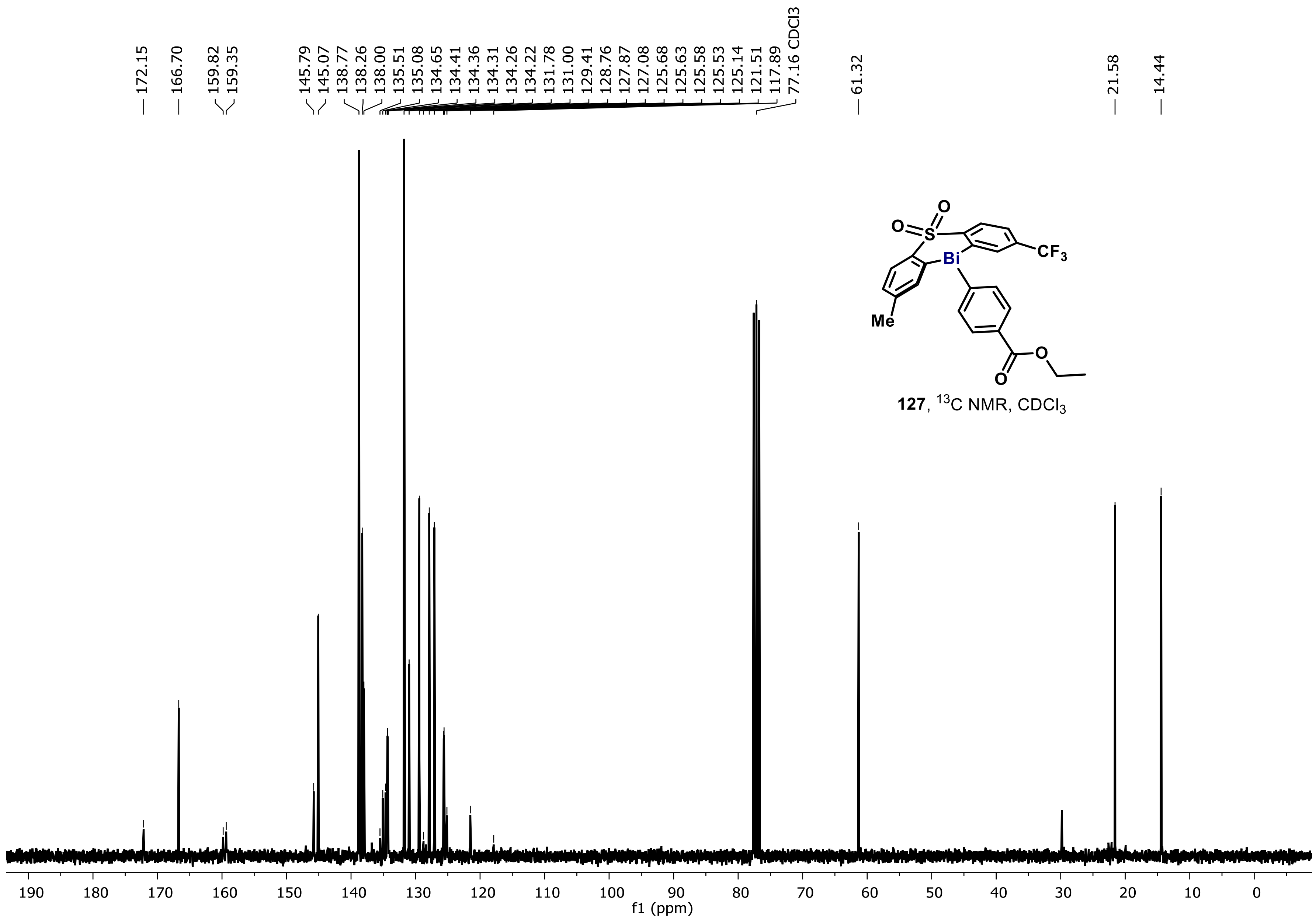

127, ^{13}C NMR, $CDCl_3$

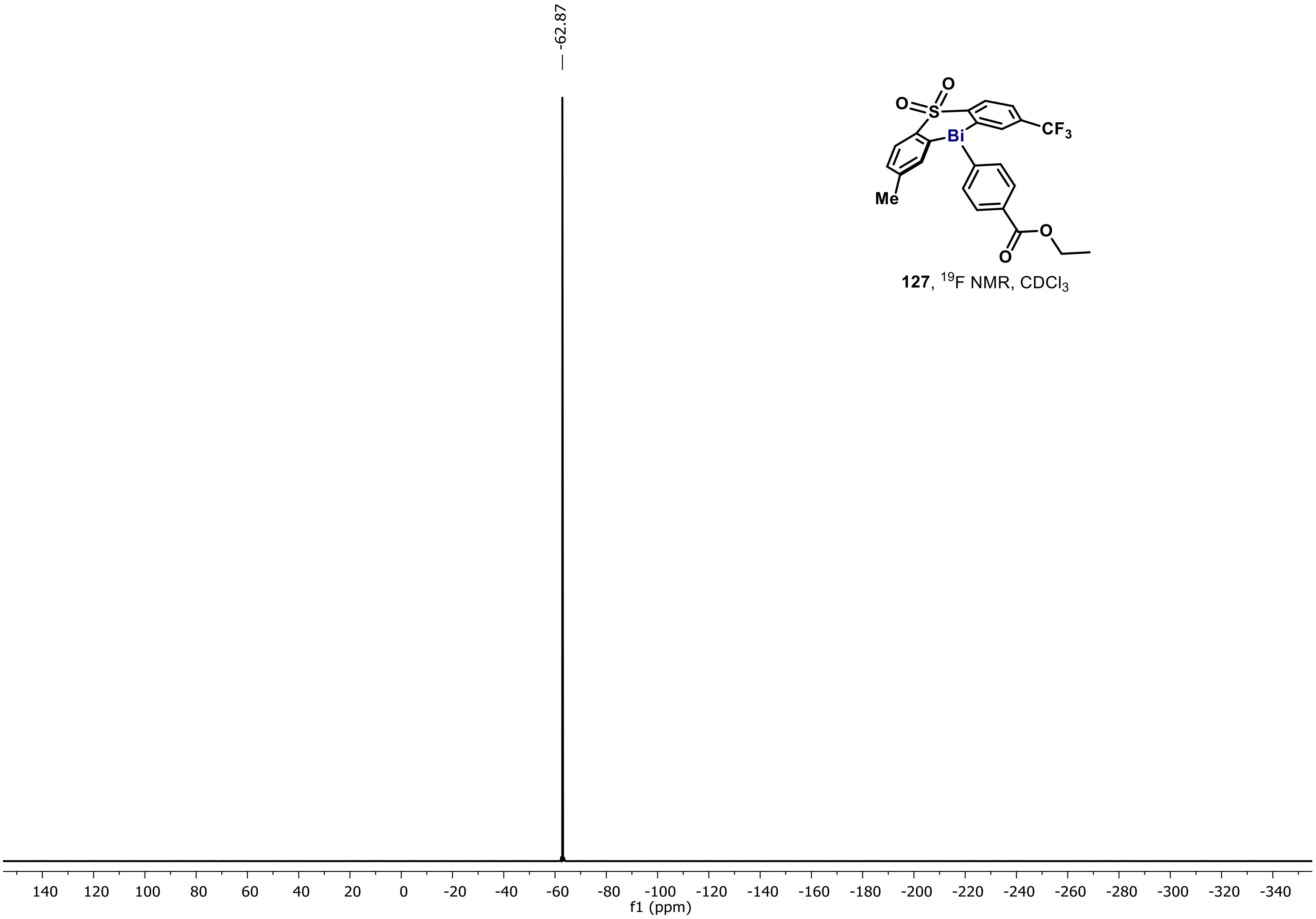

127, ^{19}F NMR, $CDCl_3$

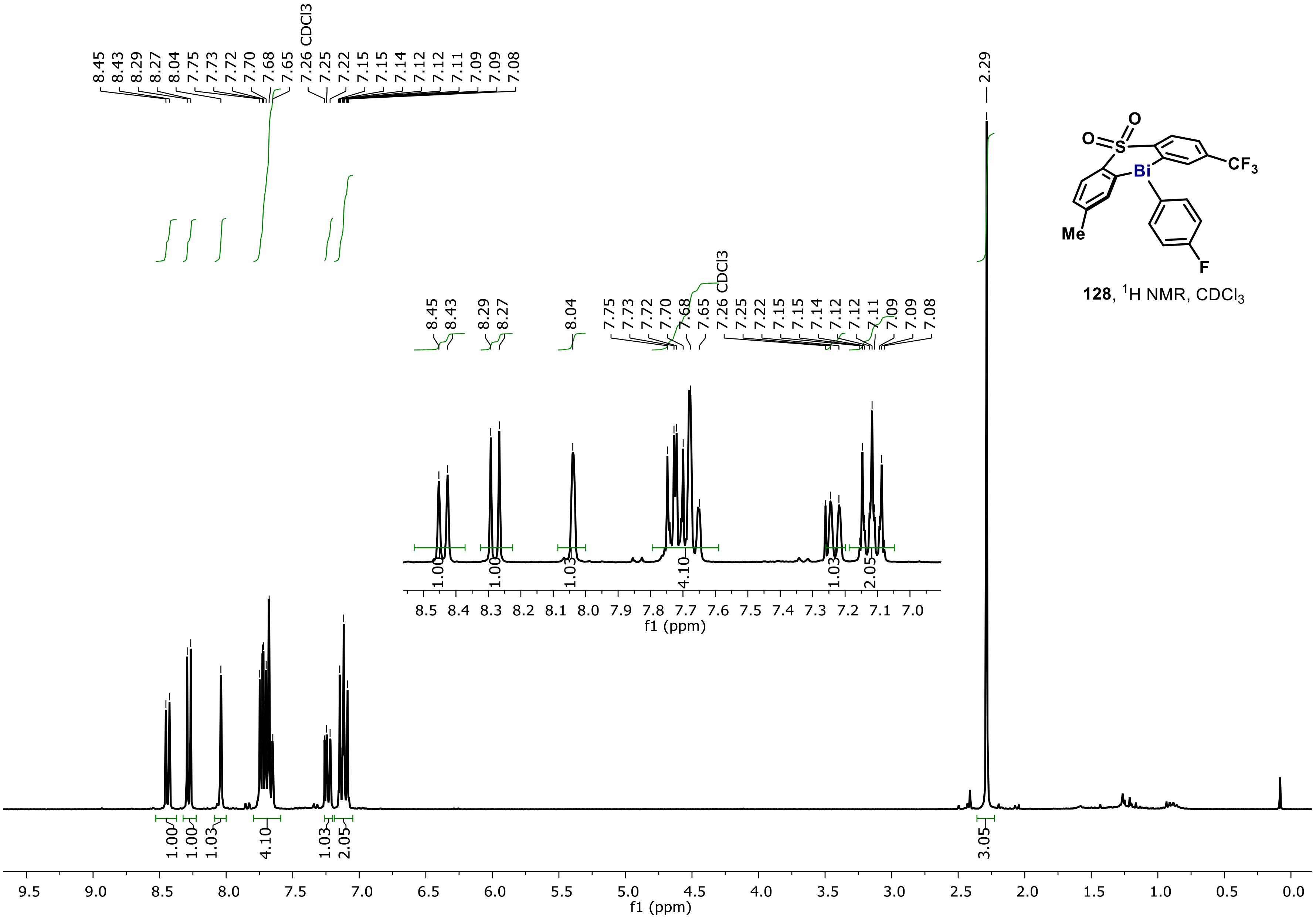
128, ^{1}H NMR, $CDCl_3$
f1 (ppm)

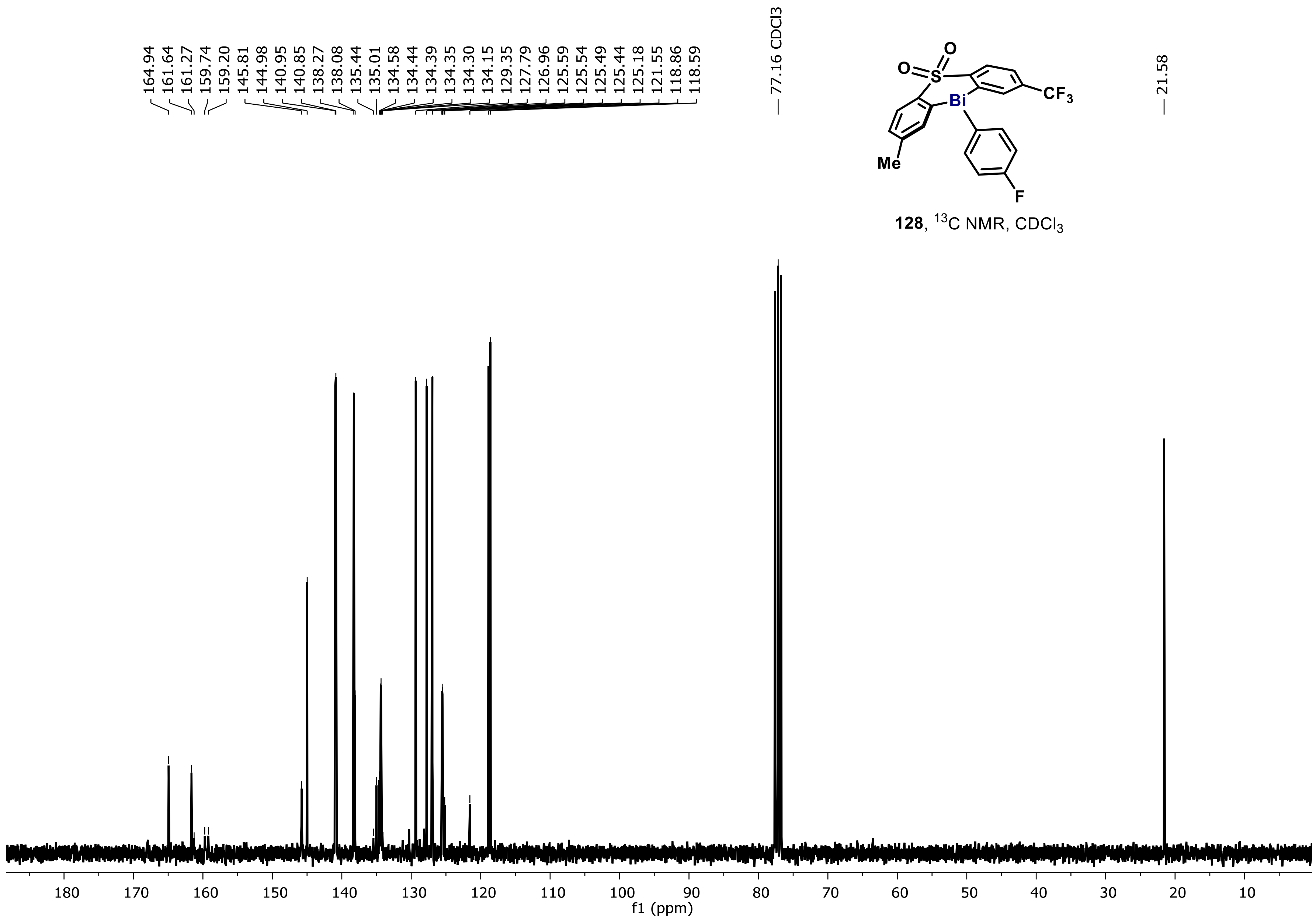

164.94
161.64
161.27
159.74
159.20
145.81
144.98
140.95
140.85
138.27
138.08
135.44
135.01
134.58
134.44
134.39
134.35
134.30
134.15
129.35
127.79
126.96
125.59
125.54
125.49
125.44
125.18
121.55
118.86
118.59
77.16 CDCl3
21.58
O
O
S
Bi
CF3
Me
F
128, 13C NMR, CDCl3
180
170
160
150
140
130
120
110
100
90
80
70
60
50
40
30
20
10
f1 (ppm)

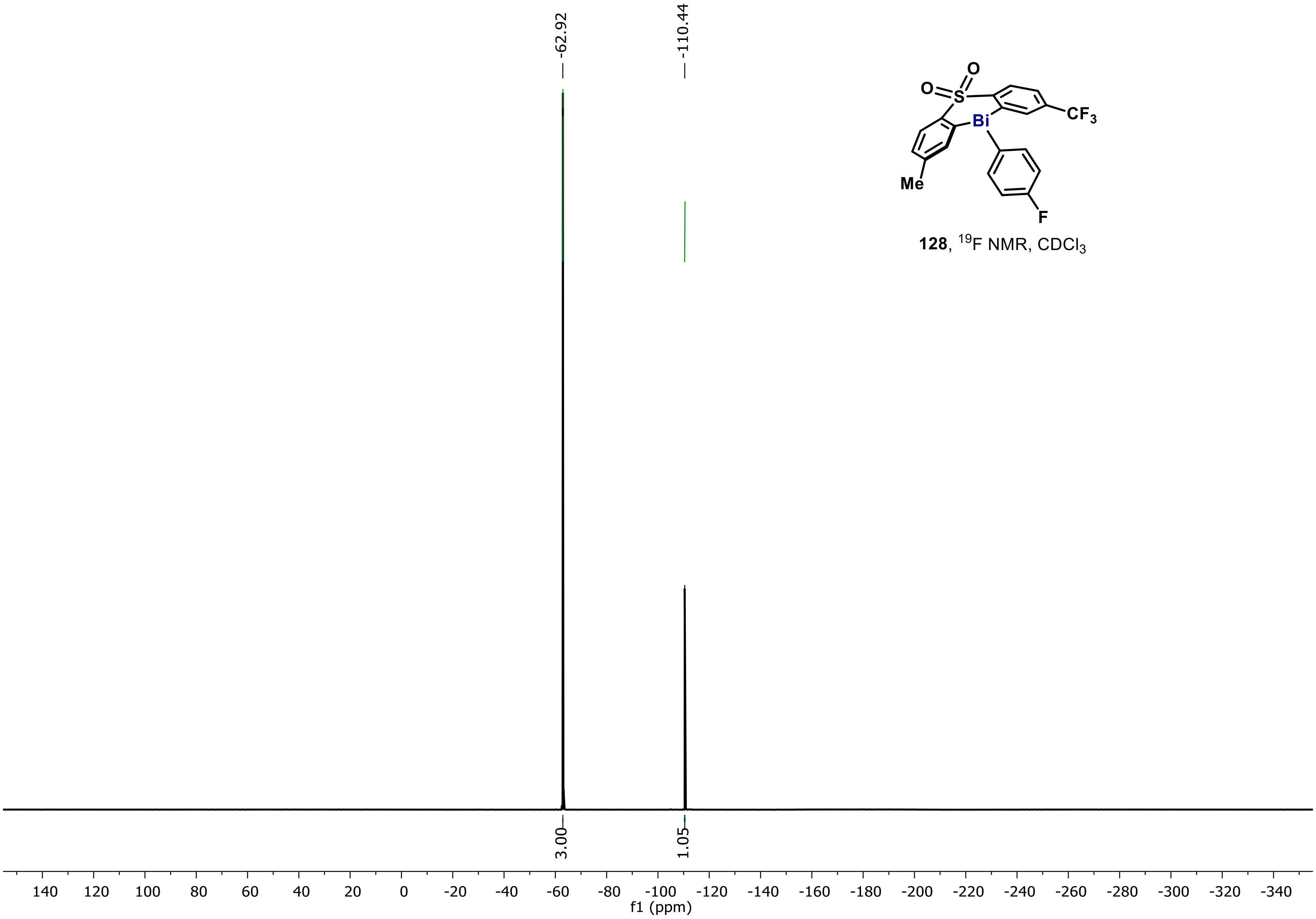

128, ^{19}F NMR, $CDCl_3$

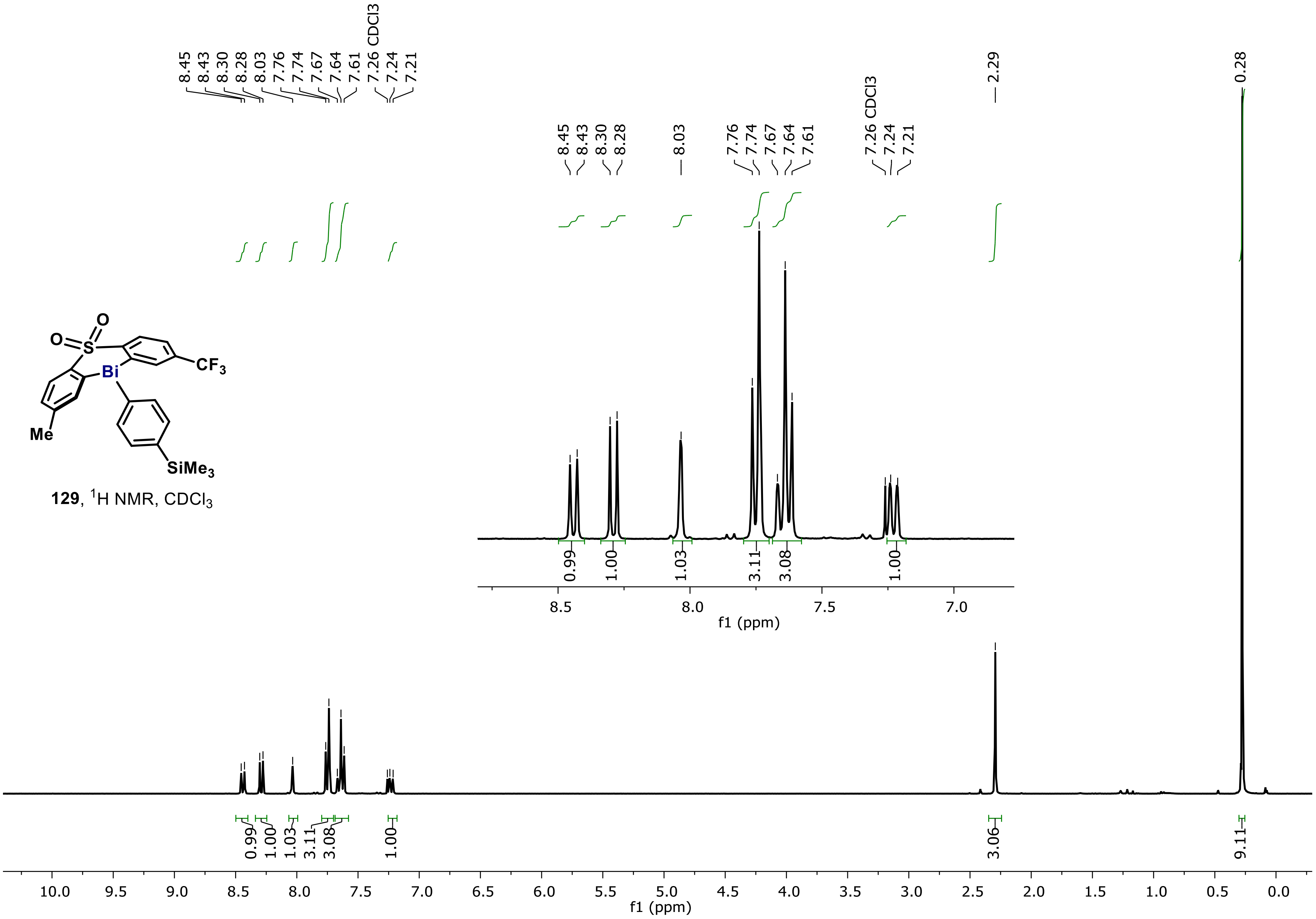

129, ^{1}H NMR, $CDCl_3$

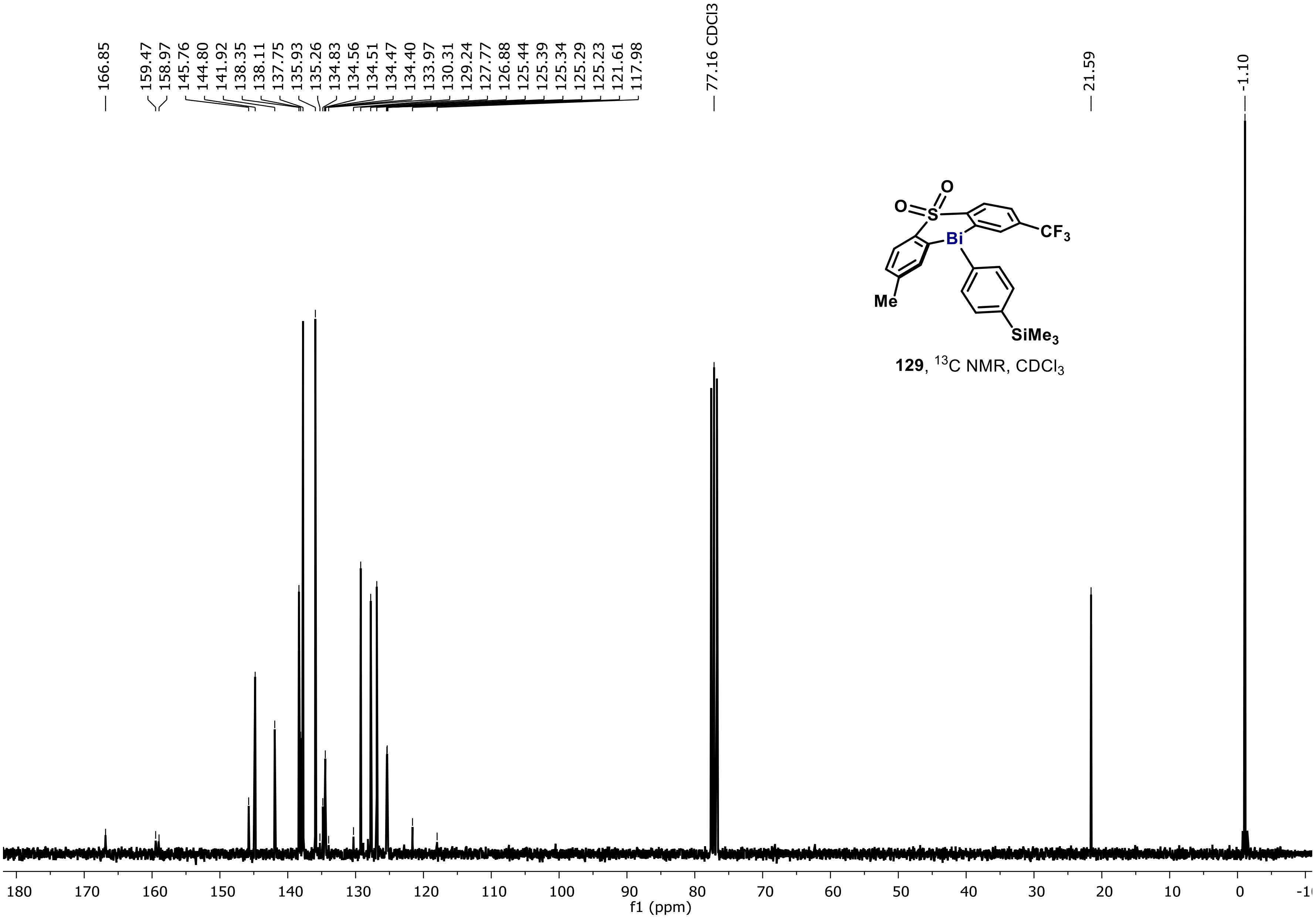
166.85
159.47
158.97
145.76
144.80
141.92
138.35
138.11
137.75
135.93
135.26
134.83
134.56
134.51
134.47
134.40
133.97
130.31
129.24
127.77
126.88
125.44
125.39
125.34
125.29
125.23
121.61
117.98
77.16 CDCl3
21.59
-1.10
O
O
S
Bi
CF_3
Me
$SiMe_3$
129, ^{13}C NMR, $CDCl_3$
180
170
160
150
140
130
120
110
100
90
80
70
60
50
40
30
20
10
0
f1 (ppm)

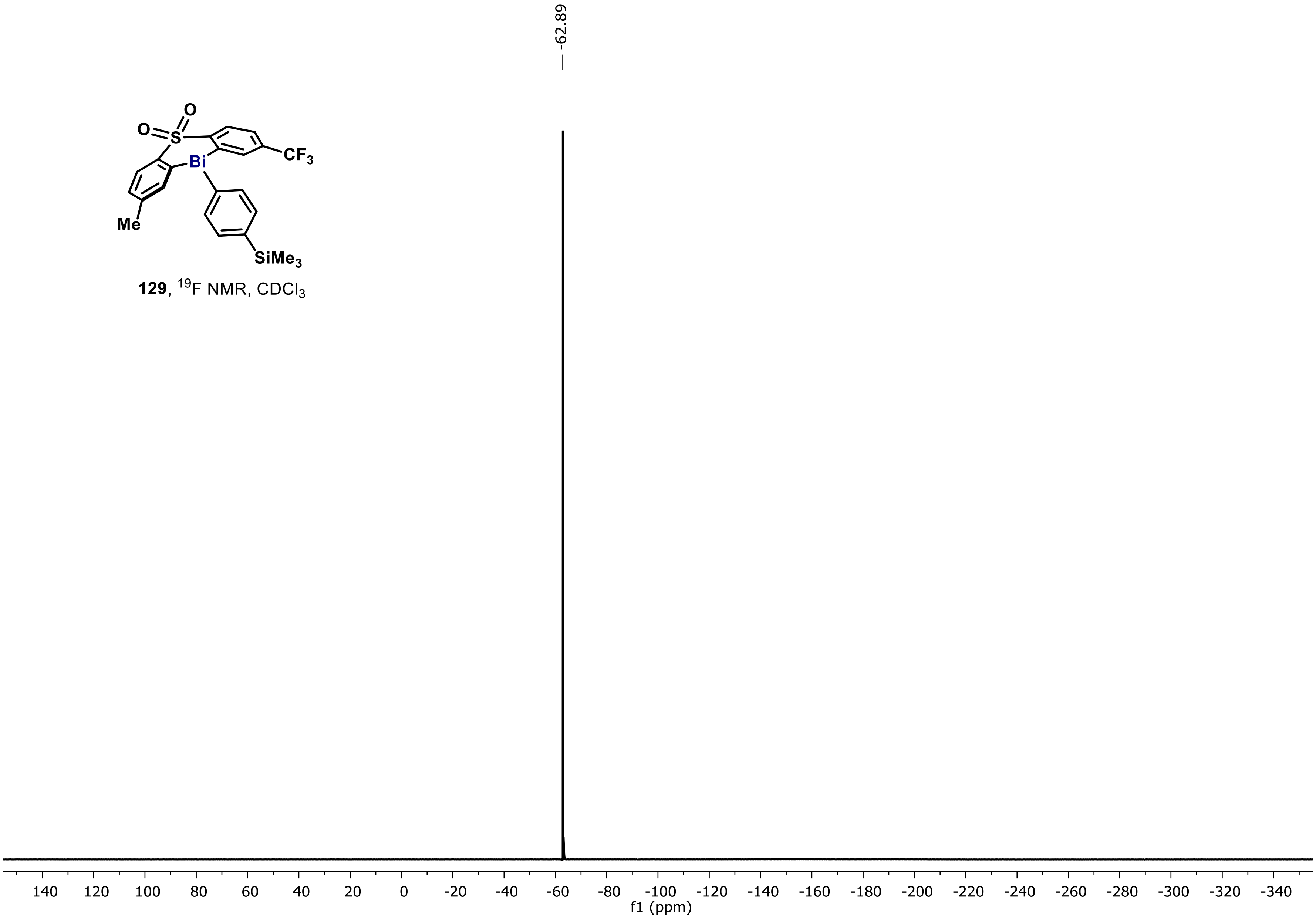

129, ^{19}F NMR, $CDCl_3$

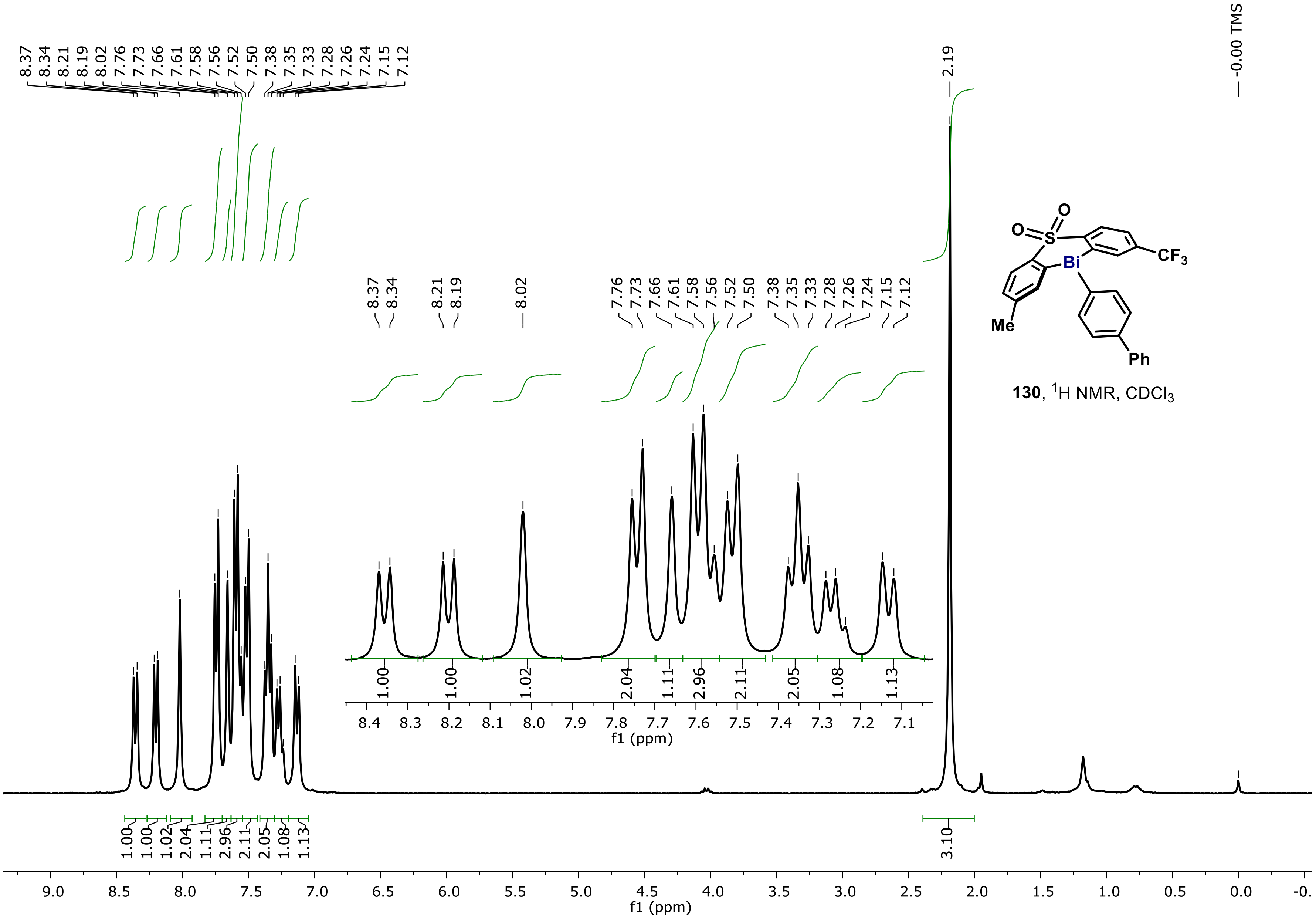
8.37 8.34 8.21 8.19 8.02 7.76 7.73 7.66 7.61 7.58 7.56 7.52 7.50 7.38 7.35 7.33 7.28 7.26 7.24 7.15 7.12
2.19
-0.00 TMS
130, ^{1}H NMR, CDCl$_3$
1.00 1.00 1.02 2.04 1.11 2.96 2.11 2.05 1.08 1.13
3.10
f1 (ppm)

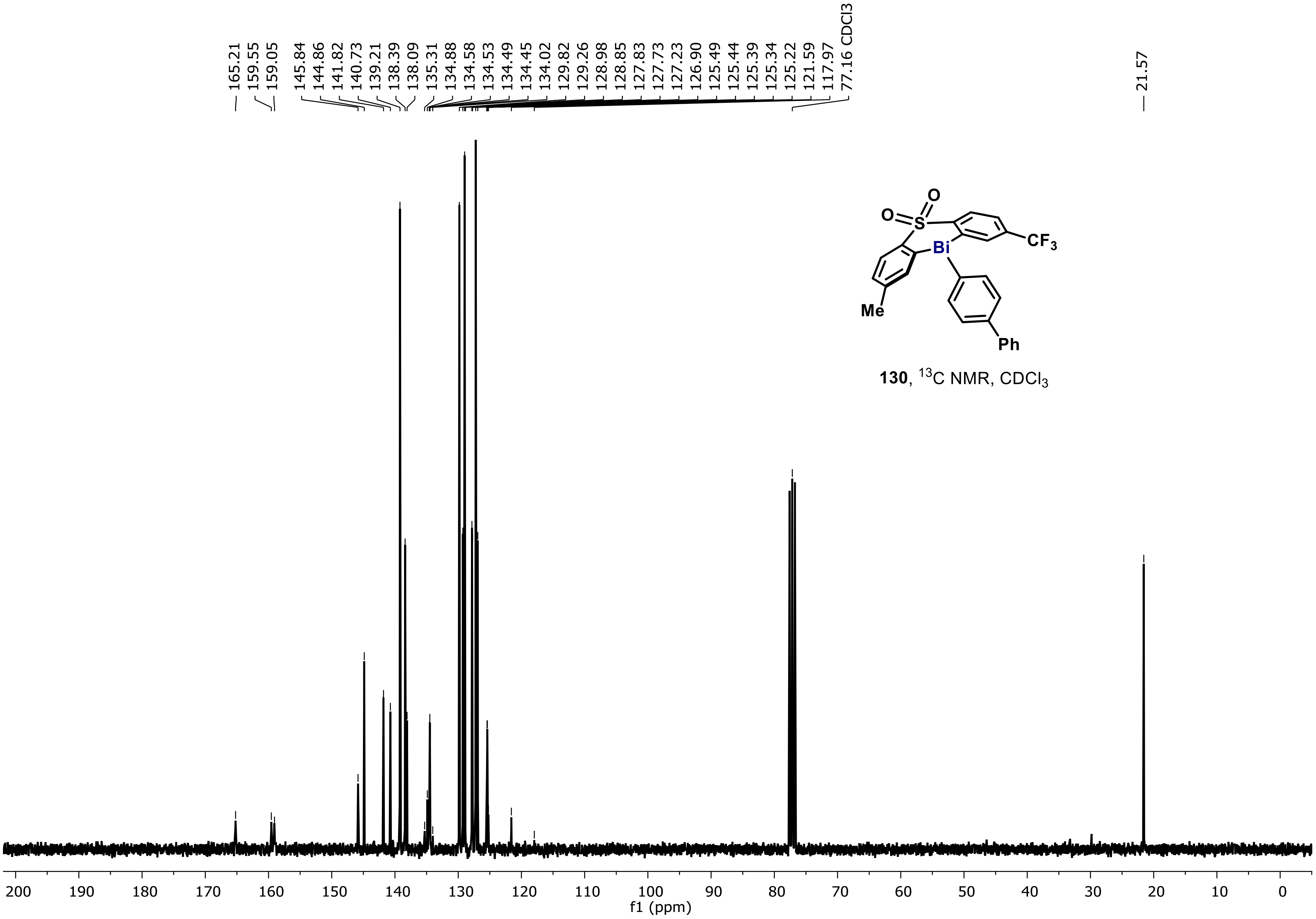

130, ^{13}C NMR, $CDCl_3$

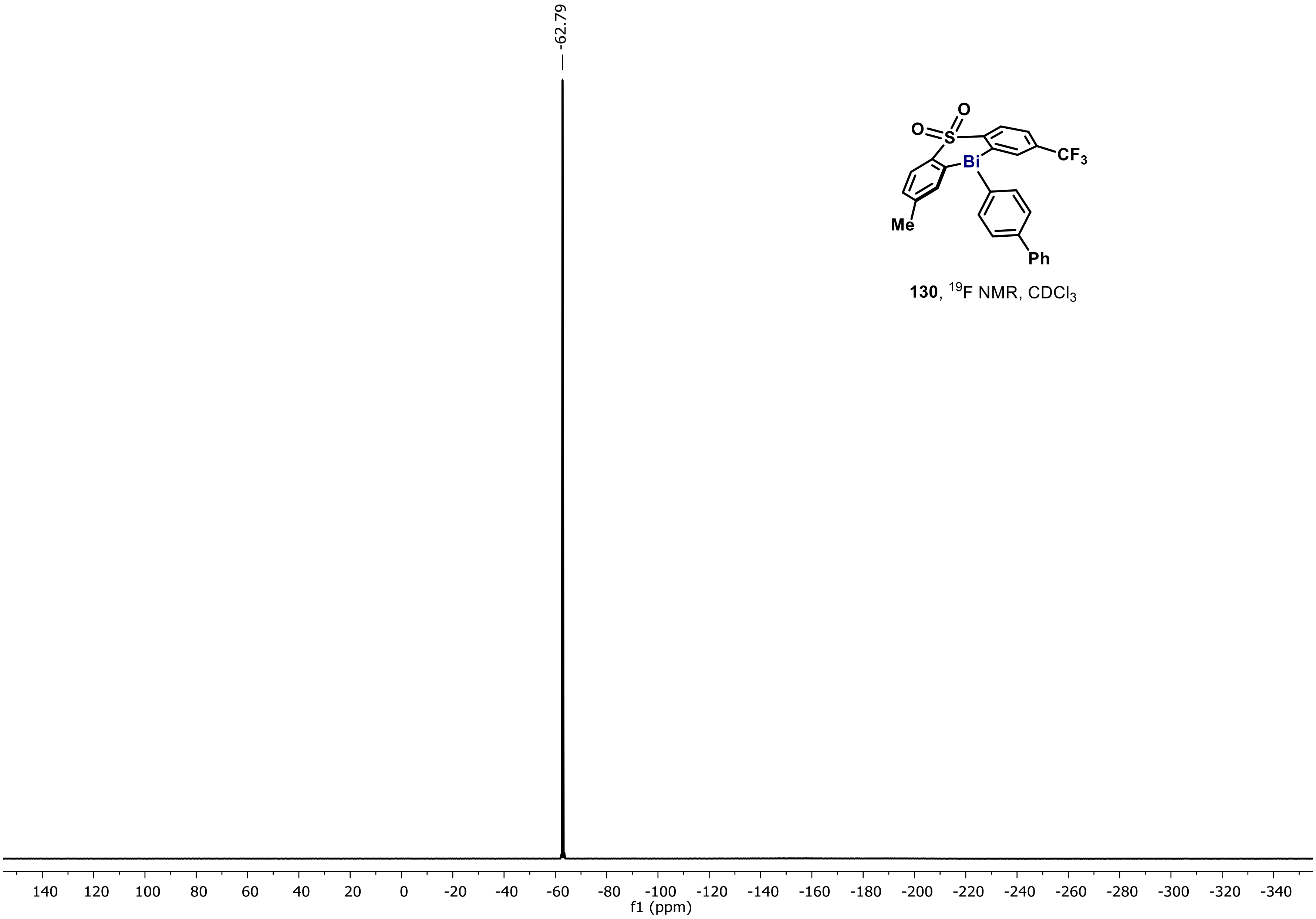
-62.79
O
O
S
Bi
CF3
Me
Ph
130, 19F NMR, CDCl3
140
120
100
80
60
40
20
0
-20
-40
-60
-80
-100
-120
-140
-160
-180
-200
-220
-240
-260
-280
-300
-320
-340
f1 (ppm)

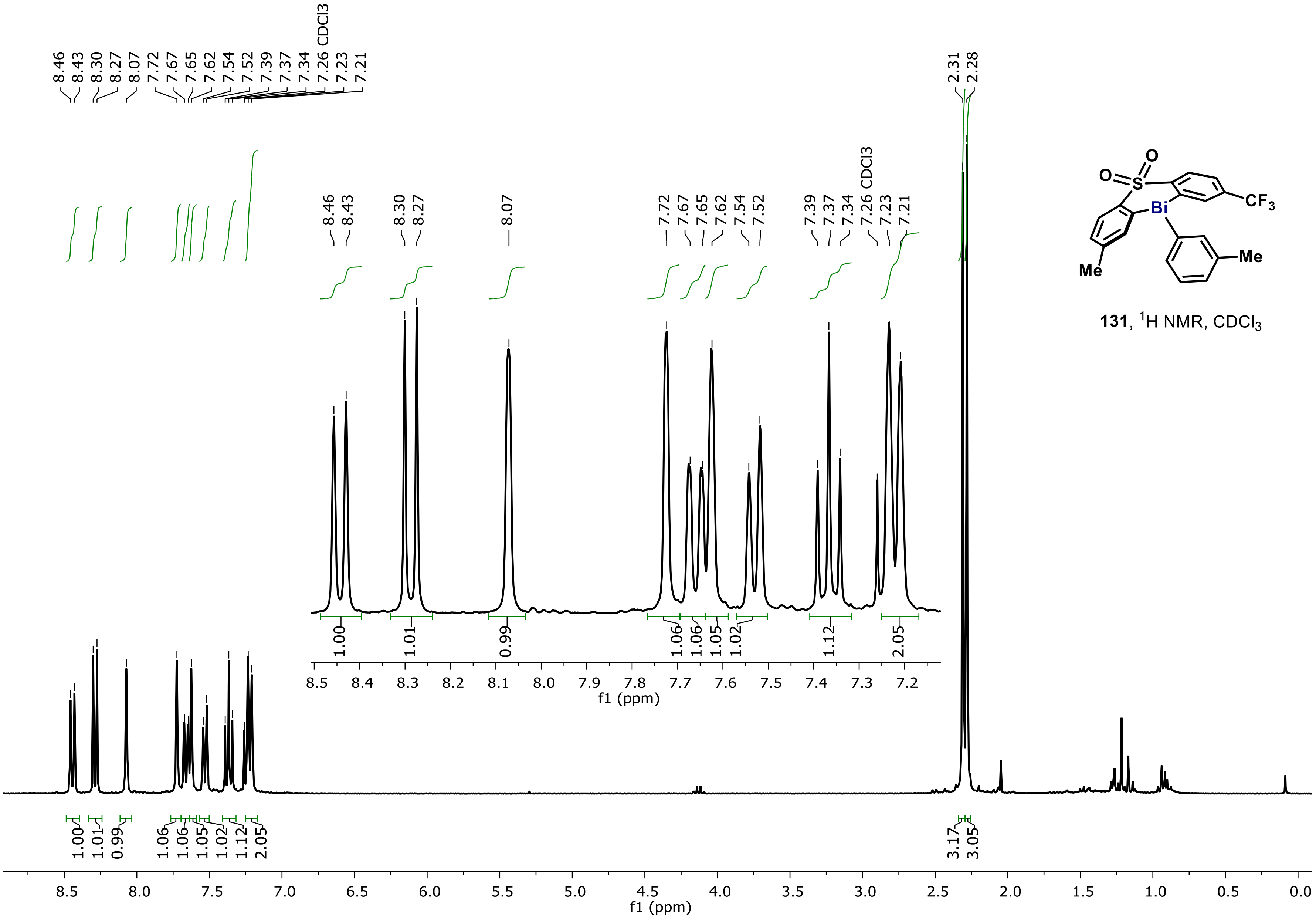

131, ^{1}H NMR, $CDCl_3$

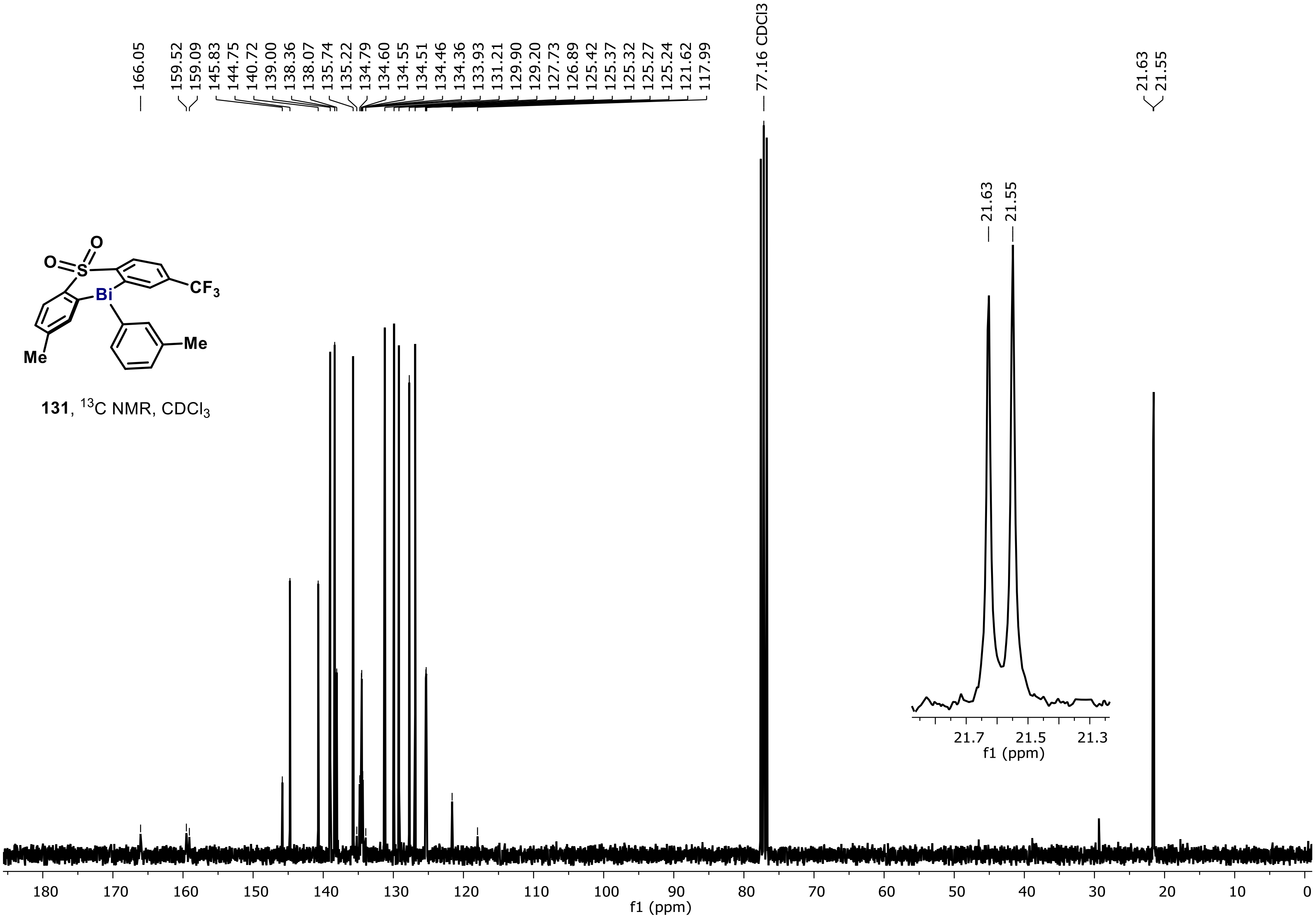

131, ^{13}C NMR, $CDCl_3$

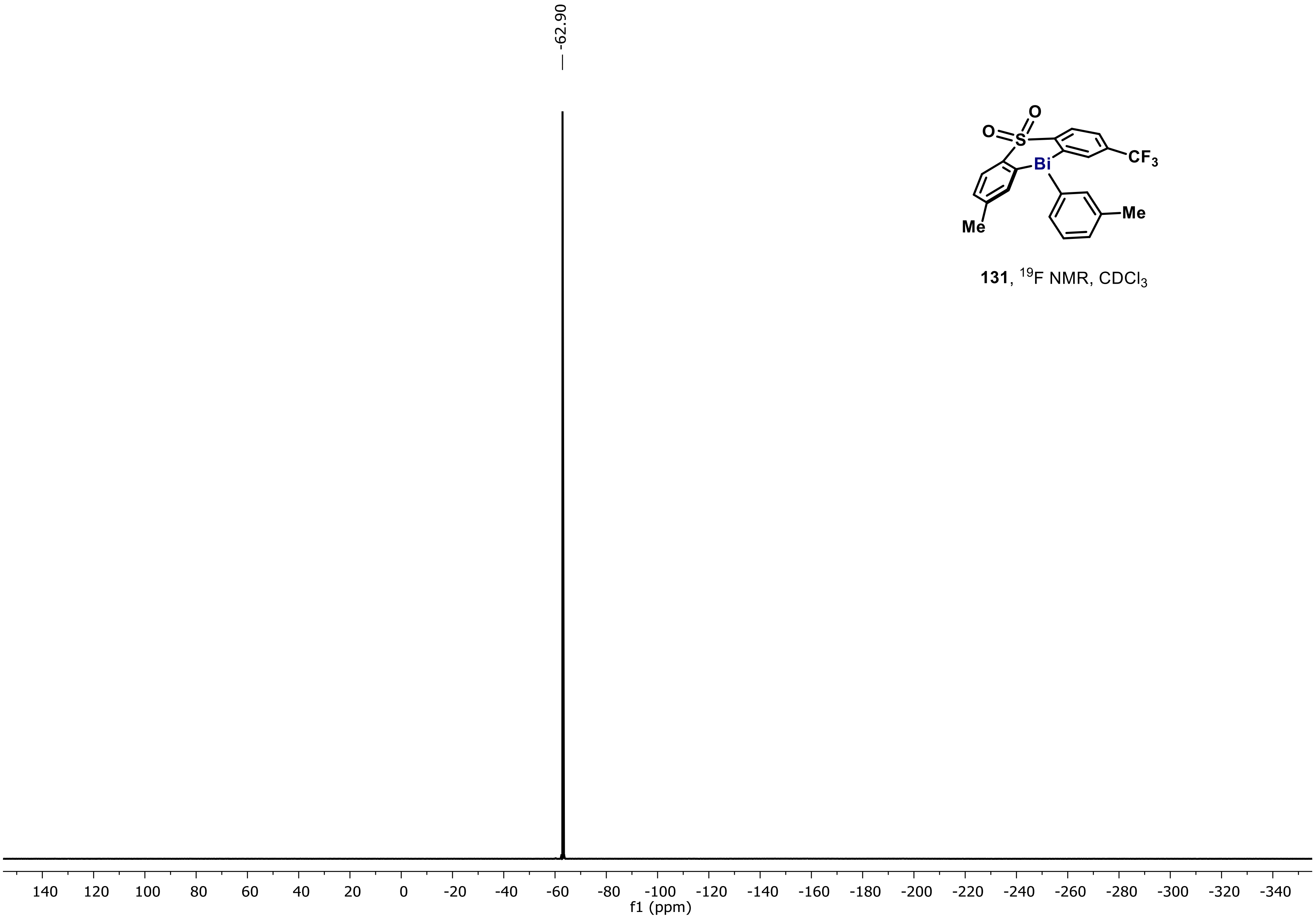

131, ^{19}F NMR, $CDCl_3$

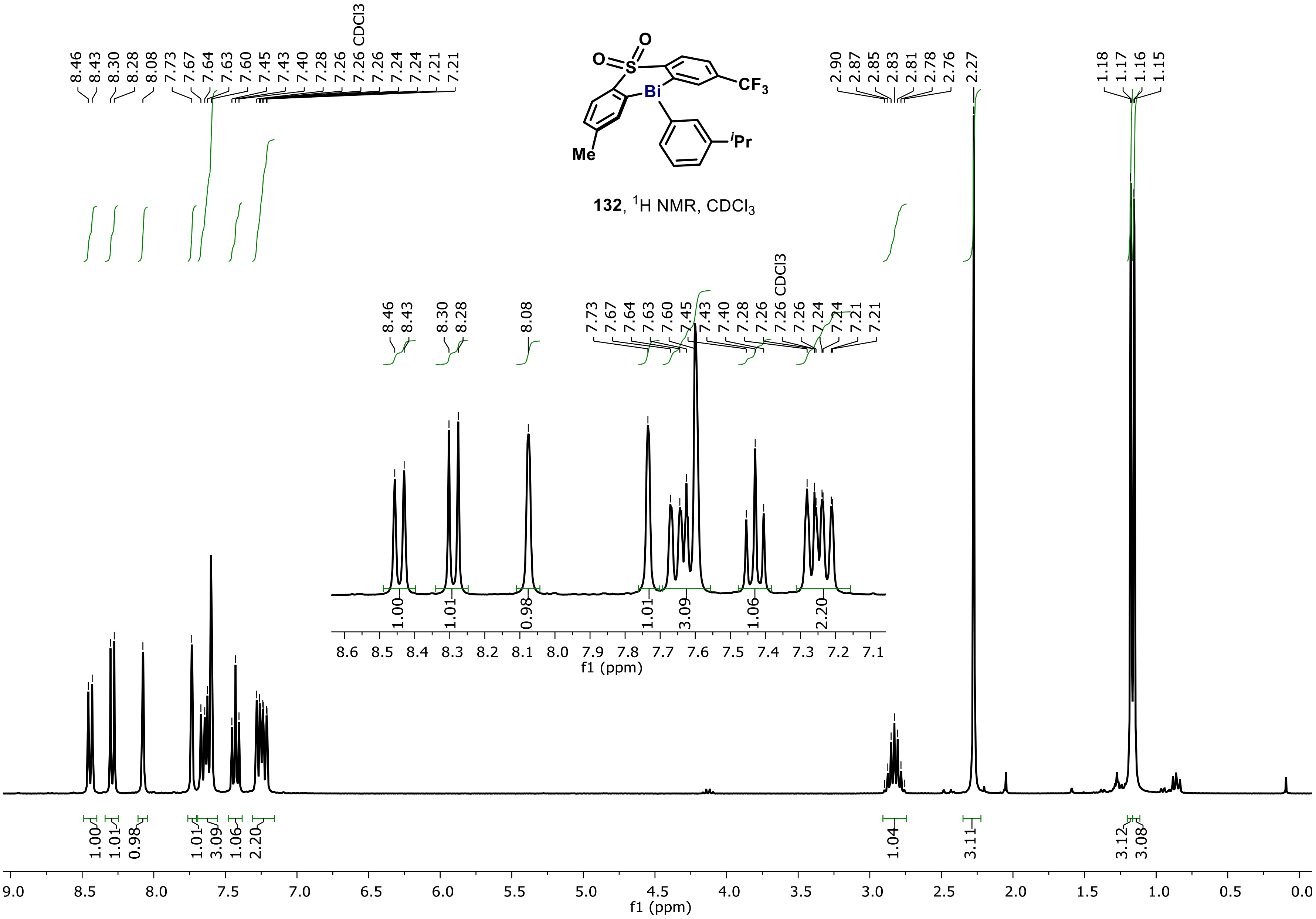
8.46
8.43
8.30
8.28
8.08
7.73
7.67
7.64
7.63
7.60
7.45
7.43
7.40
7.28
7.26 CDCl3
7.26
7.24
7.24
7.21
7.21
Bi
CF3
Me
iPr
132, 1H NMR, CDCl3
2.90
2.87
2.85
2.83
2.81
2.78
2.76
2.27
1.18
1.17
1.16
1.15
1.00
1.01
0.98
1.01
3.09
1.06
2.20
1.04
3.11
3.12
3.08
f1 (ppm)

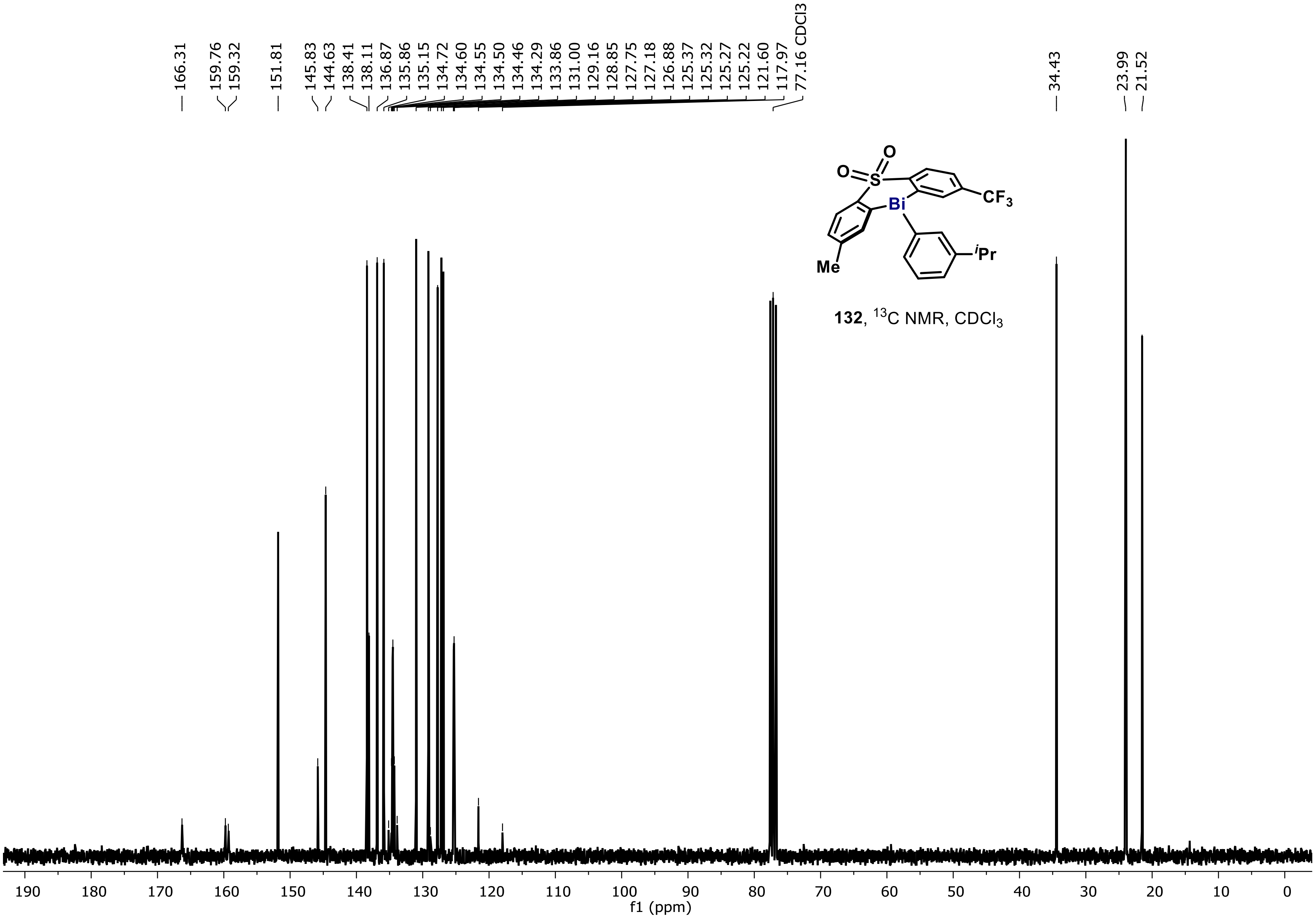

132, ^{13}C NMR, $CDCl_3$

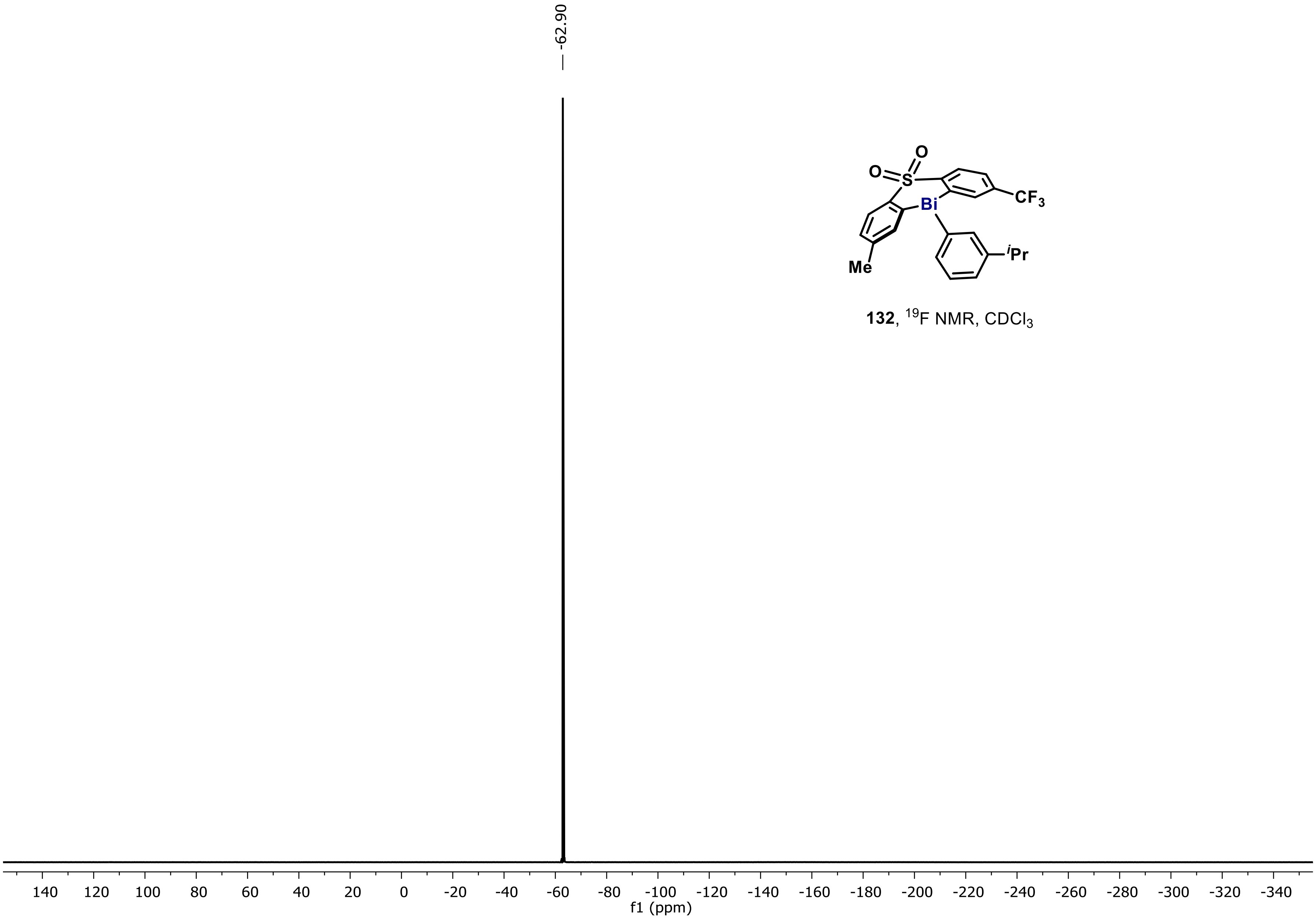

132, ^{19}F NMR, $CDCl_3$

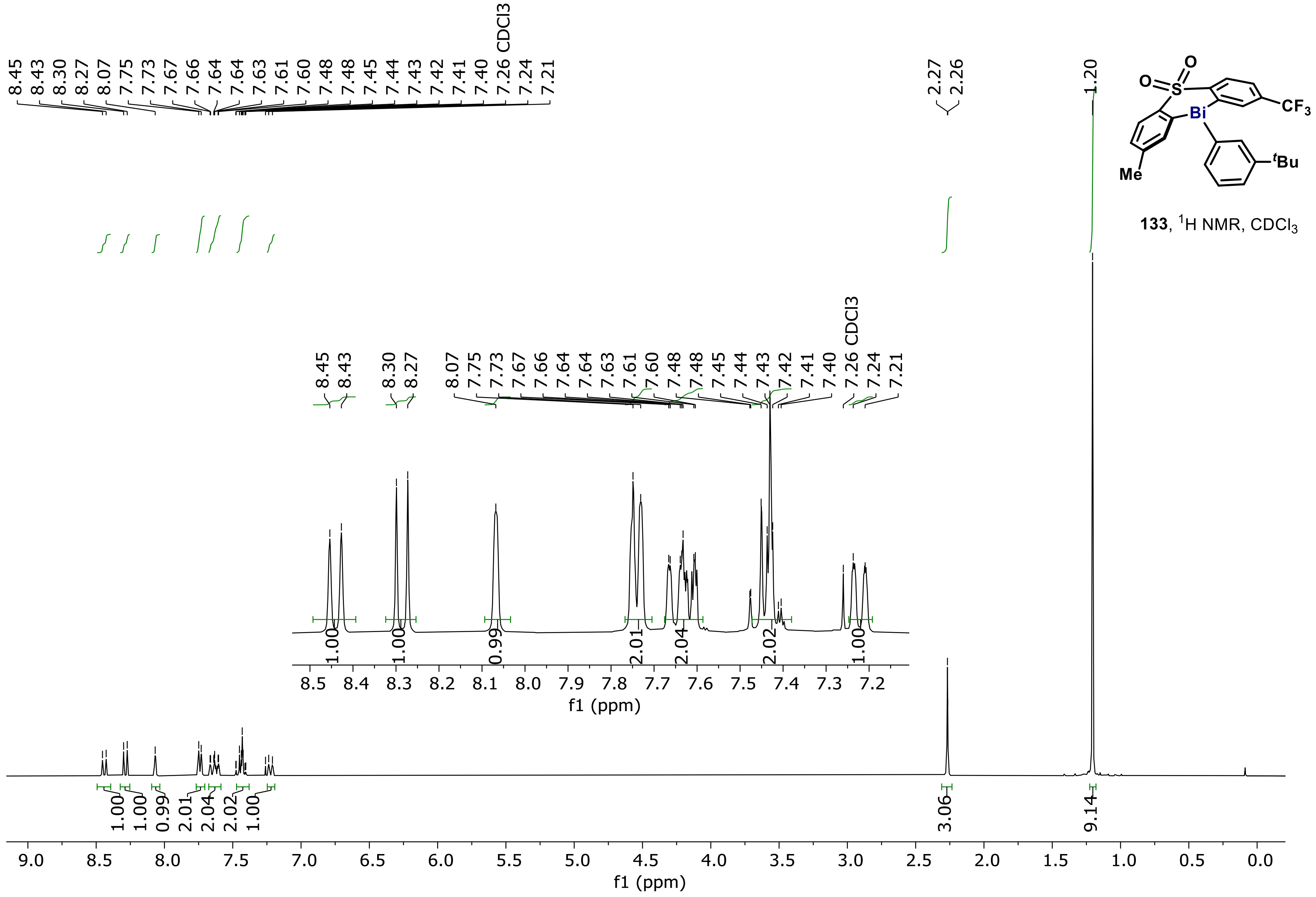
8.45 8.43 8.30 8.27 8.07 7.75 7.73 7.67 7.66 7.64 7.64 7.63 7.61 7.60 7.48 7.48 7.45 7.44 7.43 7.42 7.41 7.40 7.26 CDCl3 7.24 7.21
2.27 2.26
1.20
Bi
S
O
O
CF3
Me
tBu
133, 1H NMR, CDCl3
1.00 1.00 0.99 2.01 2.04 2.02 1.00
3.06
9.14
8.5 8.4 8.3 8.2 8.1 8.0 7.9 7.8 7.7 7.6 7.5 7.4 7.3 7.2
f1 (ppm)
9.0 8.5 8.0 7.5 7.0 6.5 6.0 5.5 5.0 4.5 4.0 3.5 3.0 2.5 2.0 1.5 1.0 0.5 0.0
f1 (ppm)

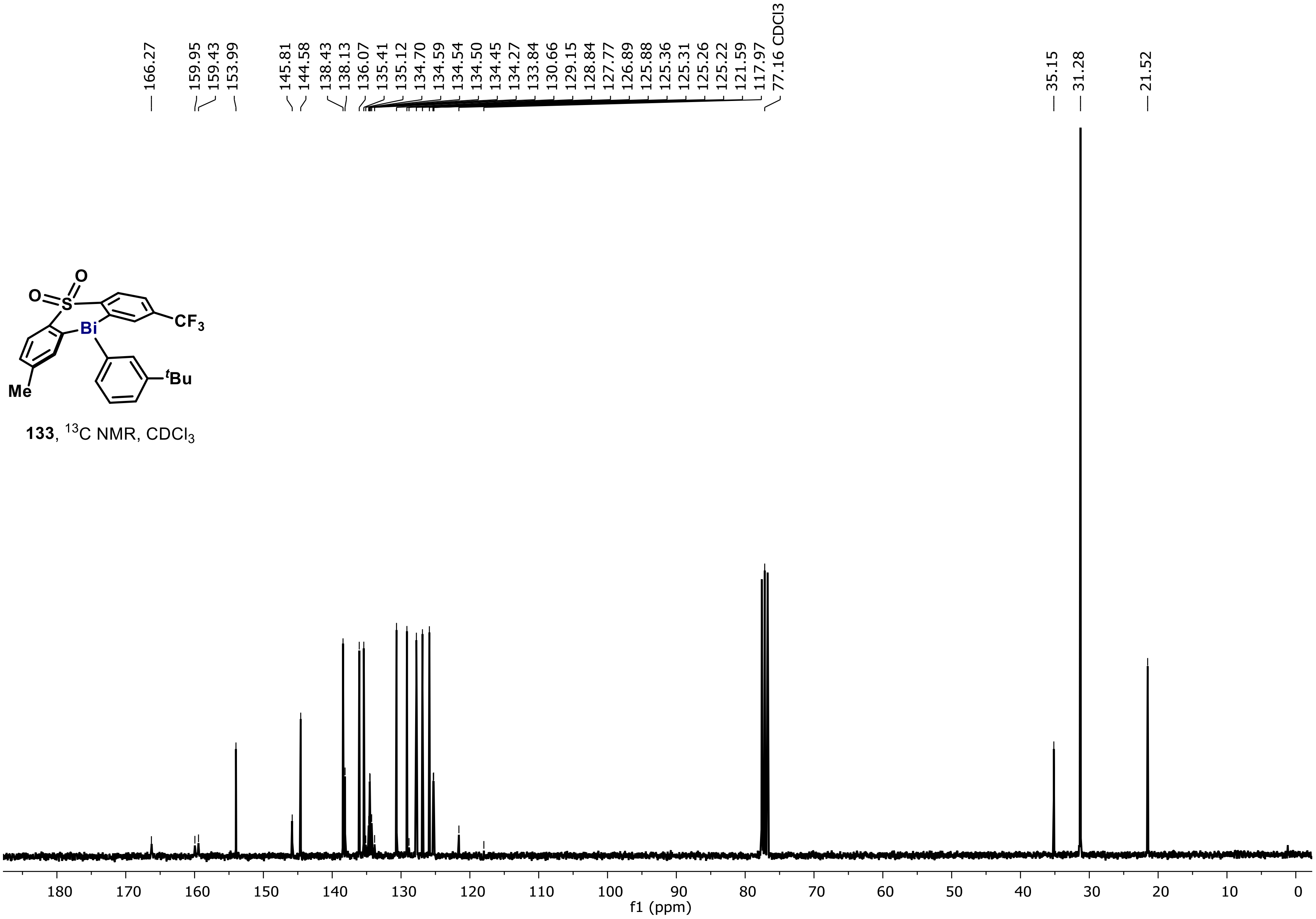

133, ^{13}C NMR, $CDCl_3$

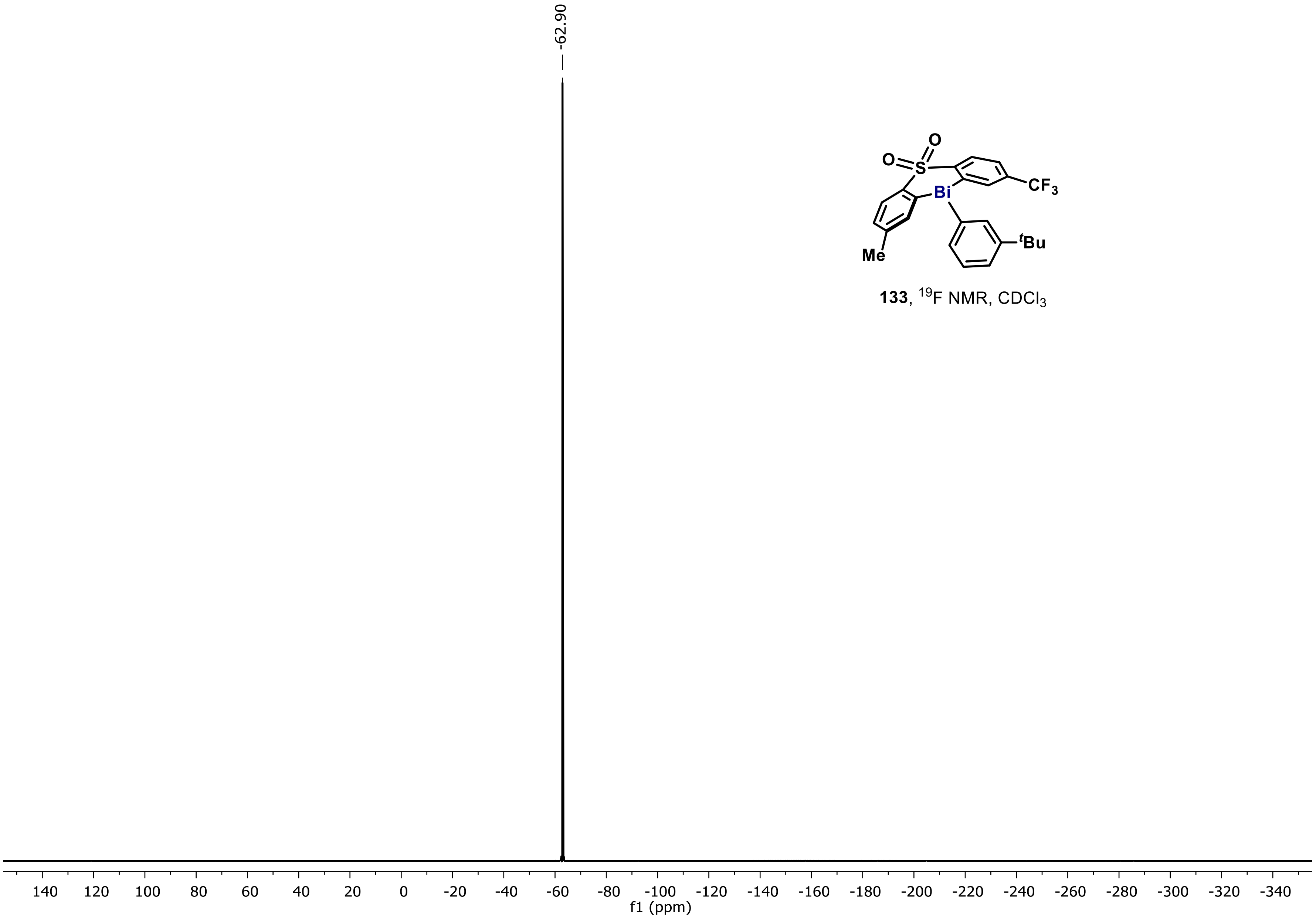

133, ^{19}F NMR, $CDCl_3$

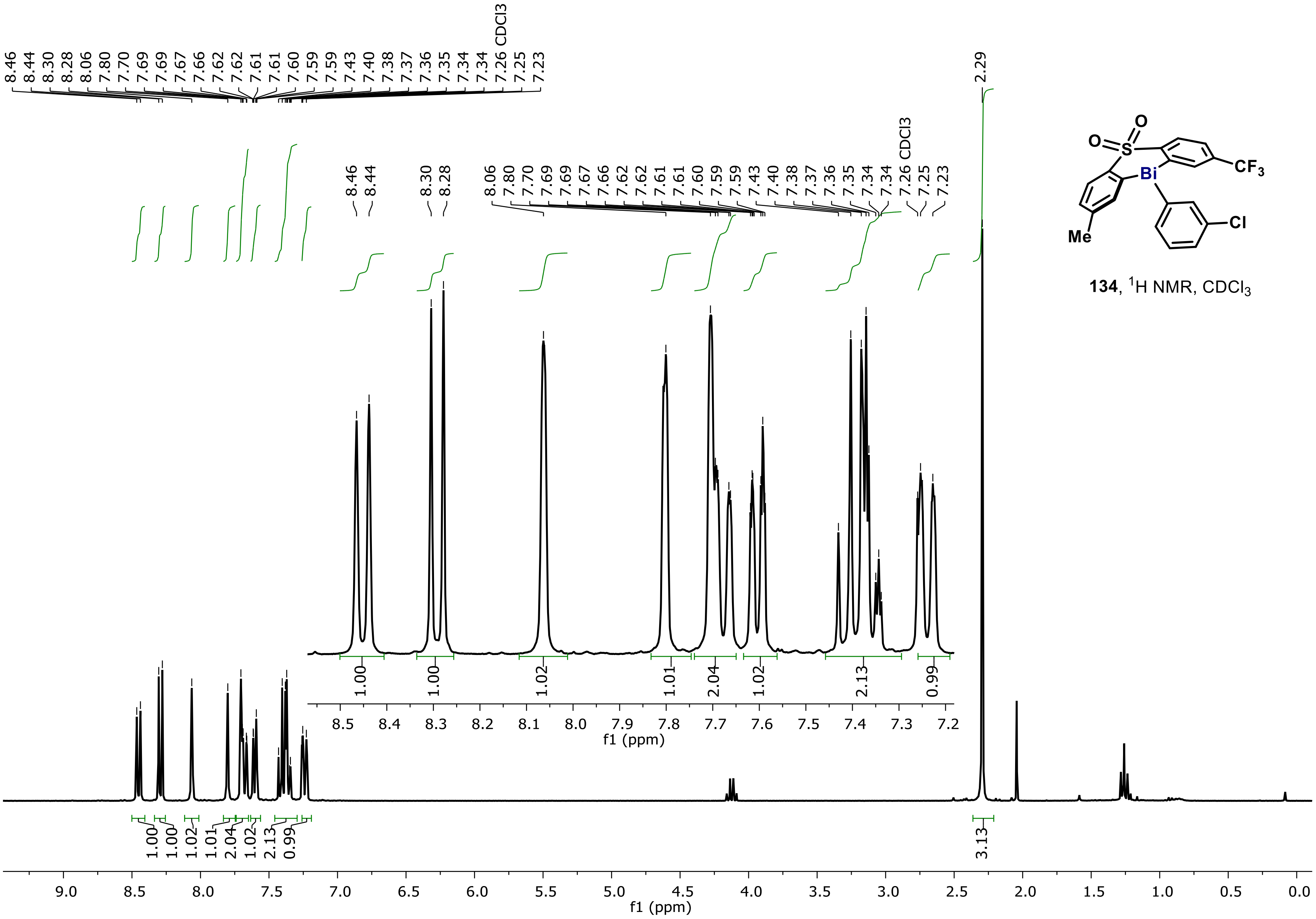

134, ^{1}H NMR, $CDCl_3$

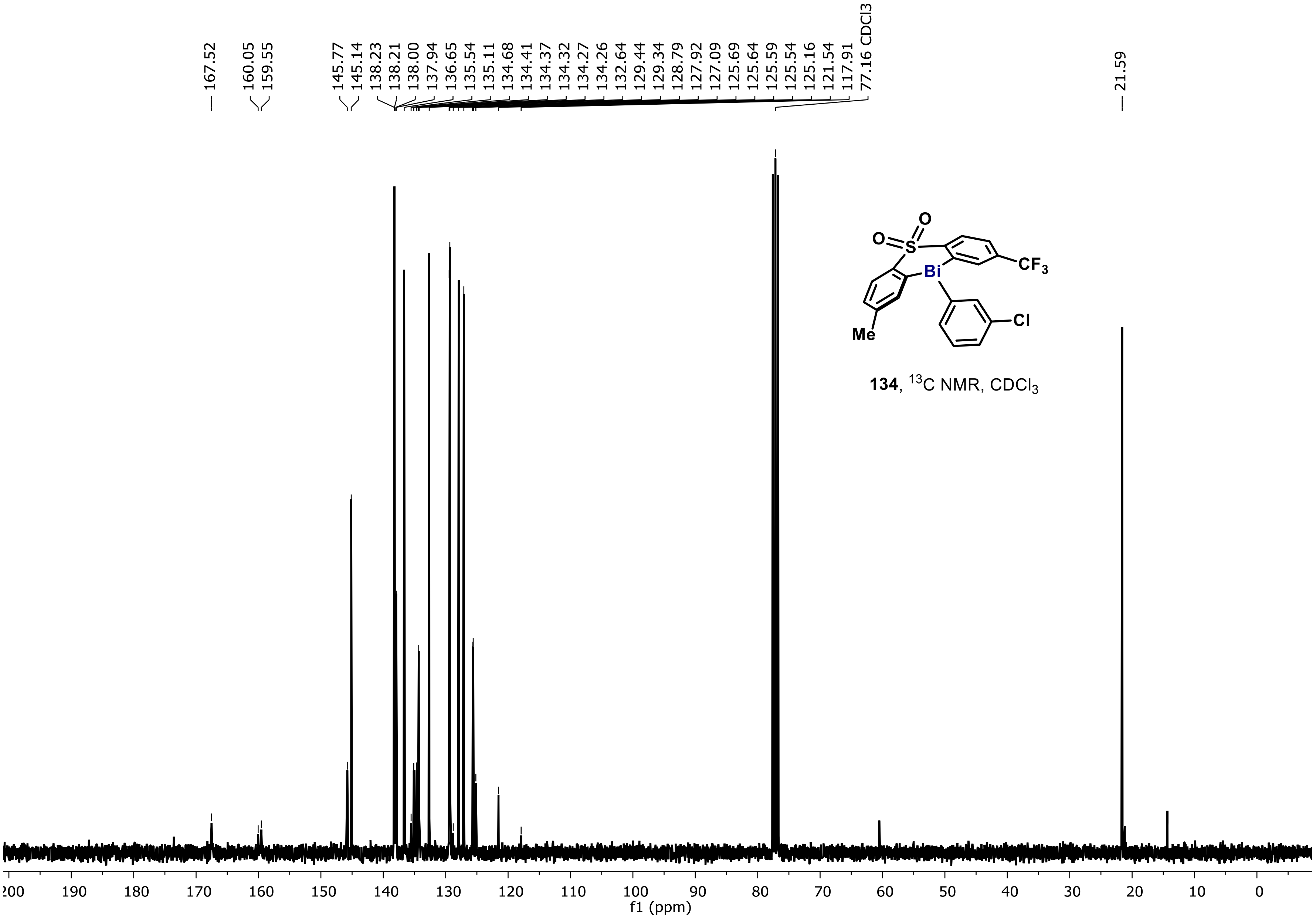

134, ^{13}C NMR, $CDCl_3$

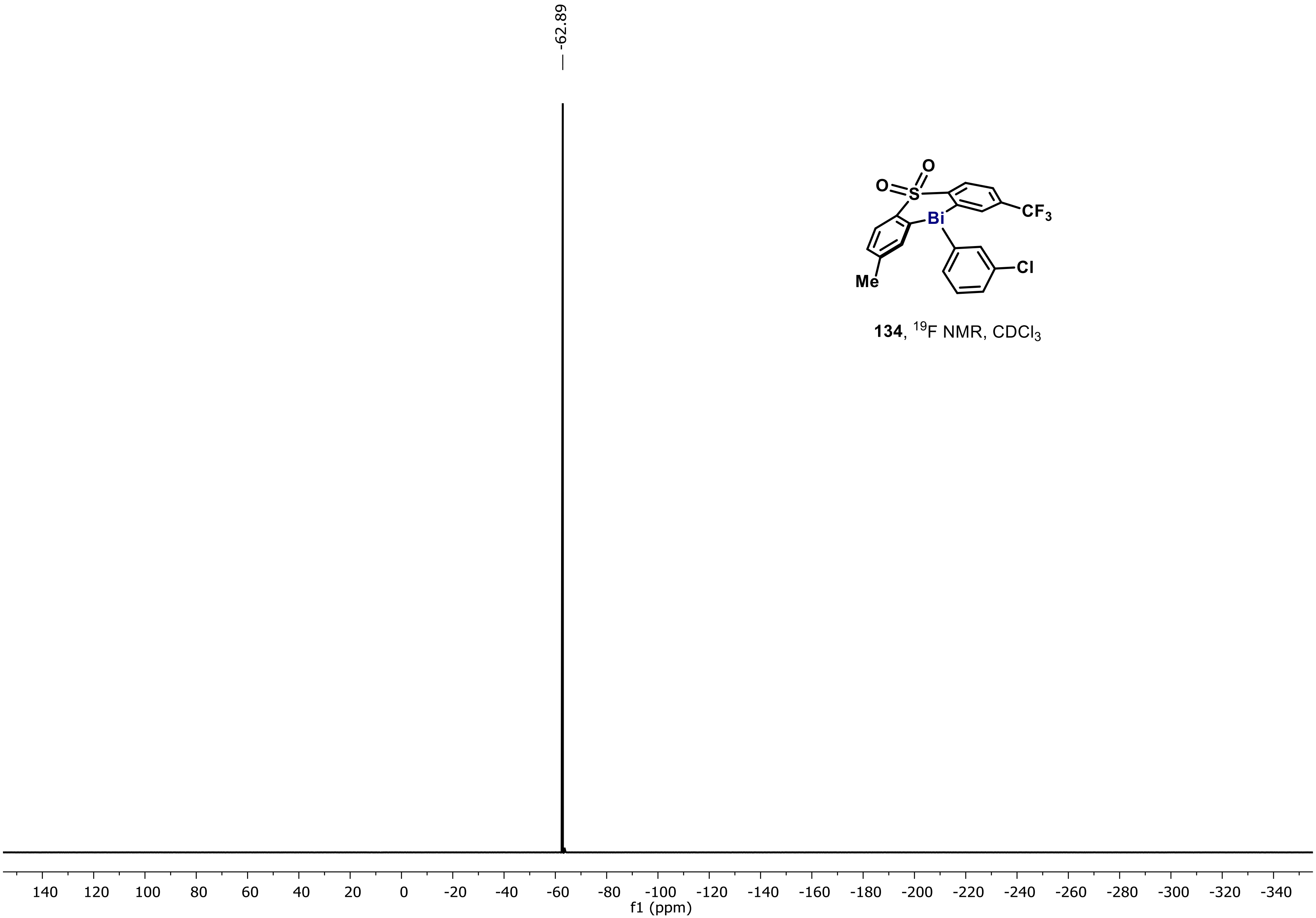

134, ^{19}F NMR, $CDCl_3$

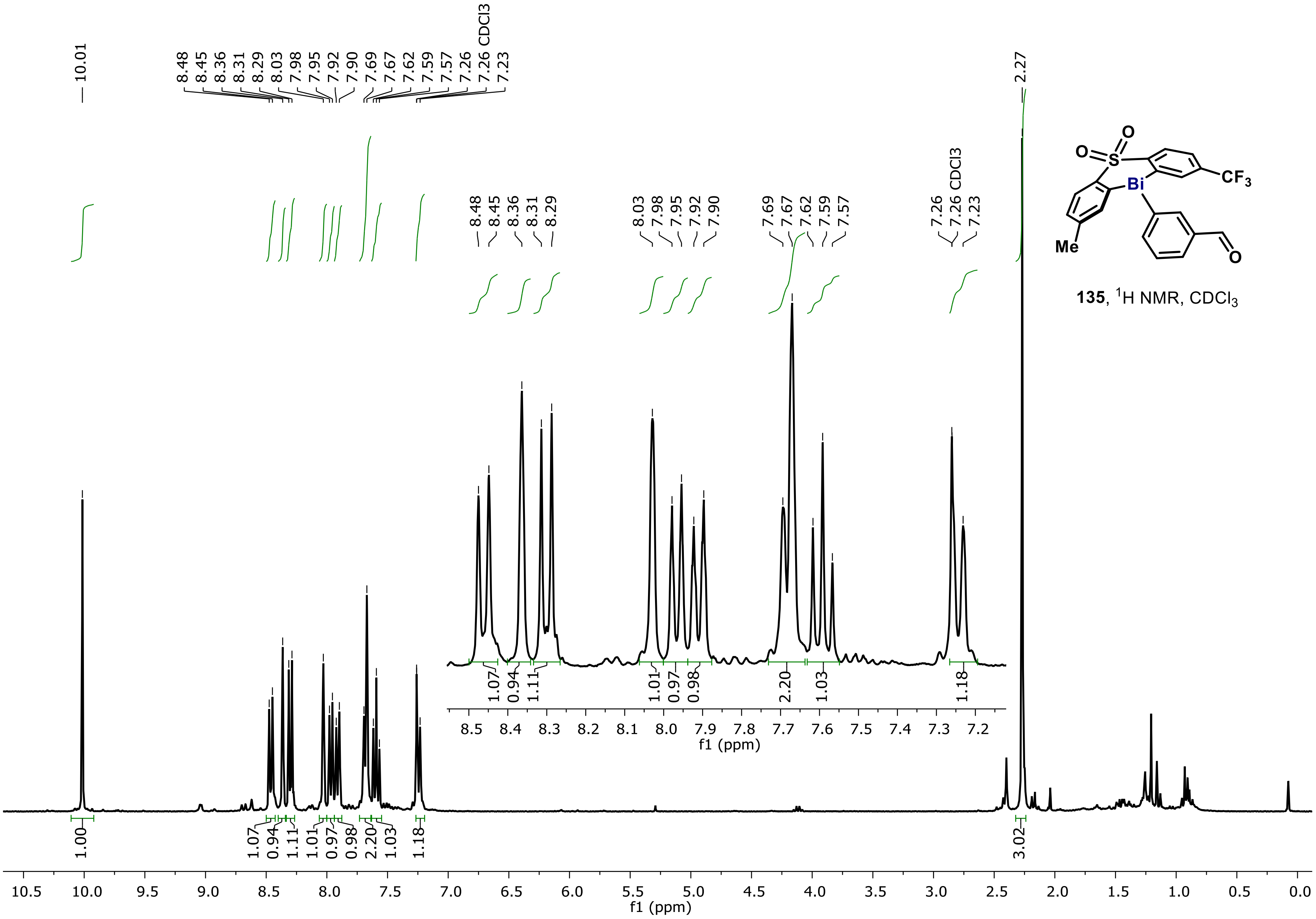

135, ^{1}H NMR, $CDCl_3$

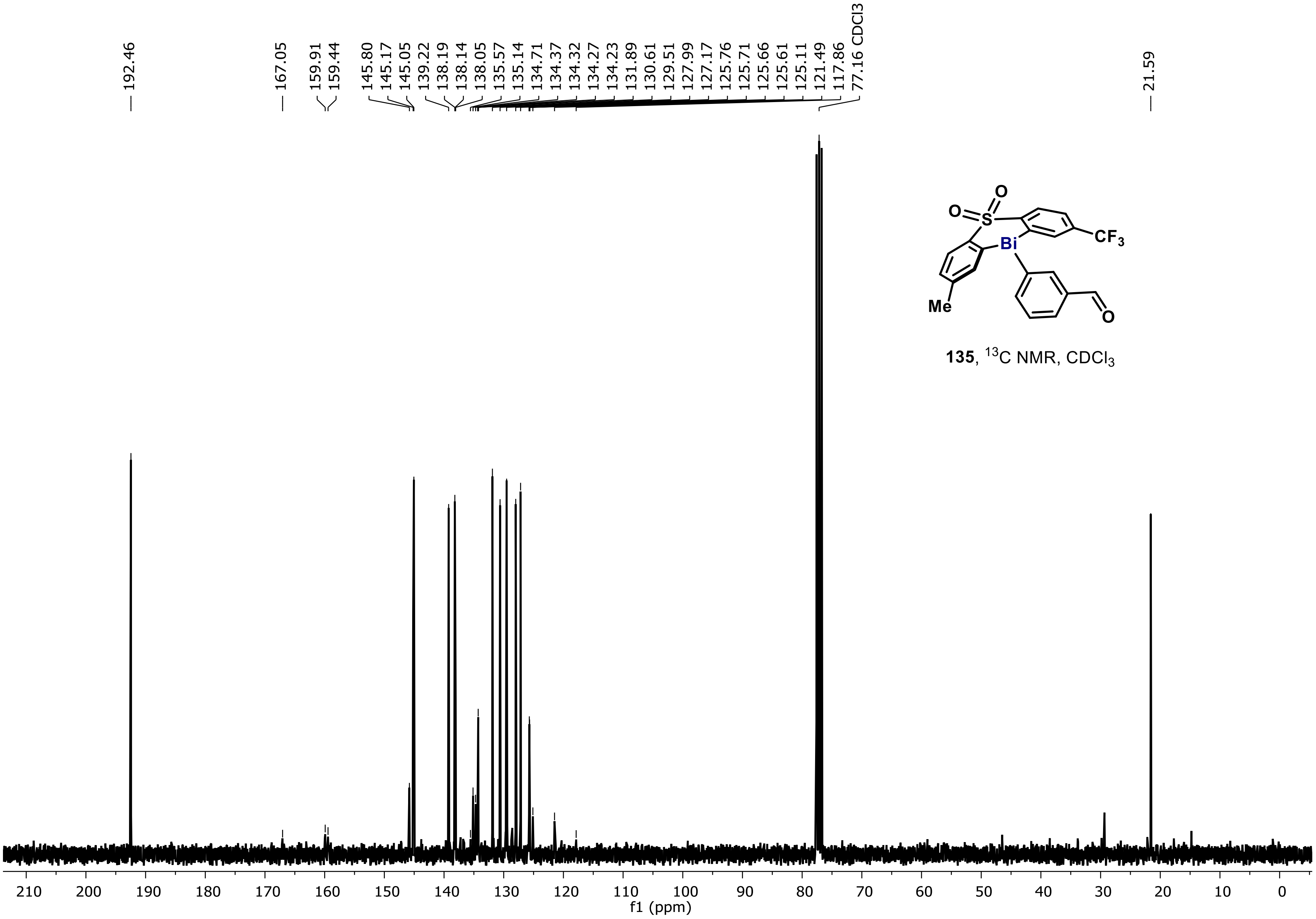

192.46
167.05
159.91
159.44
145.80
145.17
145.05
139.22
138.19
138.14
138.05
135.57
135.14
134.71
134.37
134.32
134.27
134.23
131.89
130.61
129.51
127.99
127.17
125.76
125.71
125.66
125.61
125.11
121.49
117.86
77.16 CDCl3
21.59
O
O
S
Bi
CF3
Me
O
135, 13C NMR, CDCl3
210 200 190 180 170 160 150 140 130 120 110 100 90 80 70 60 50 40 30 20 10 0
f1 (ppm)

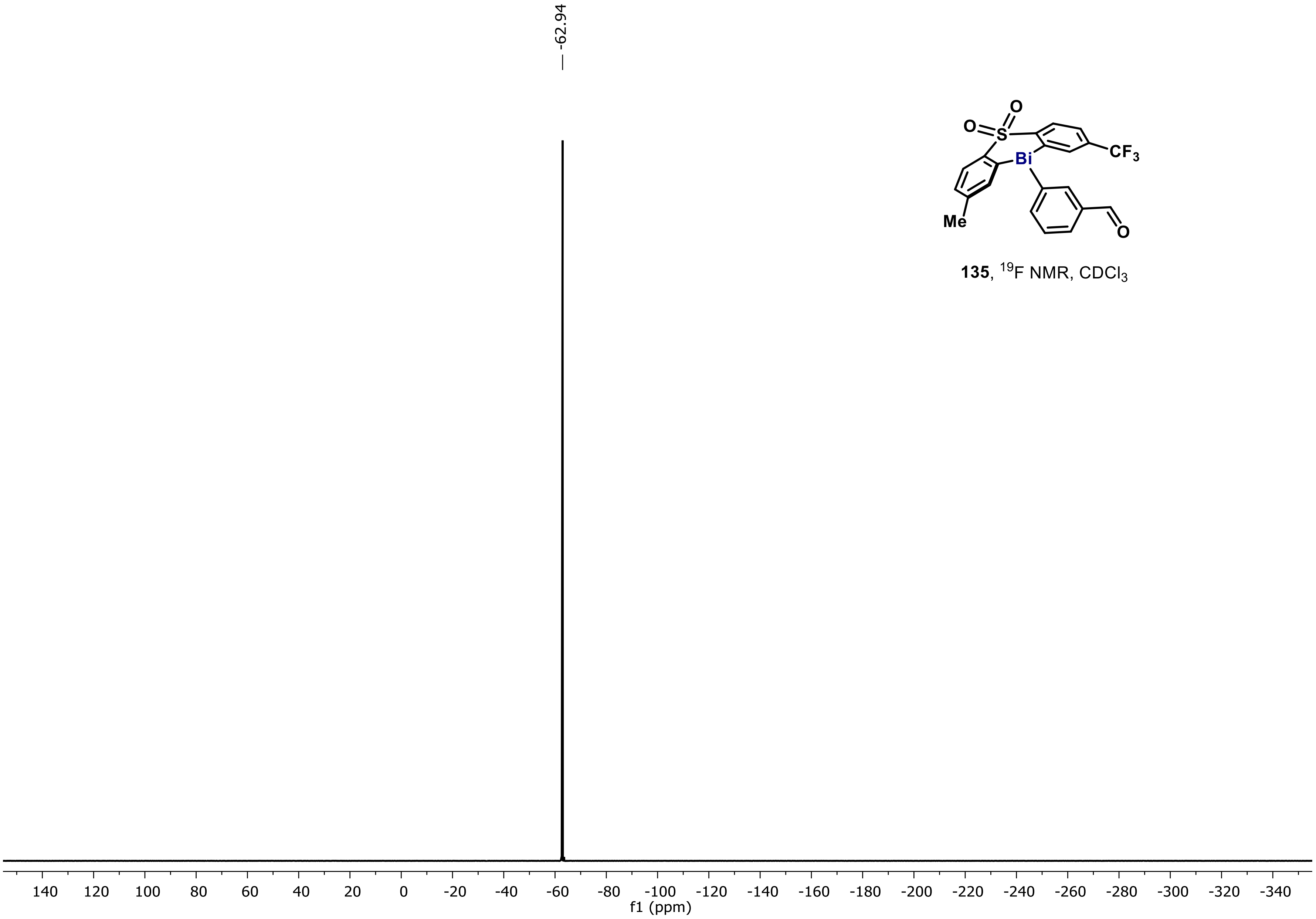
-62.94
O
O
S
CF3
Bi
Me
O
135, 19F NMR, CDCl3
140 120 100 80 60 40 20 0 -20 -40 -60 -80 -100 -120 -140 -160 -180 -200 -220 -240 -260 -280 -300 -320 -340
f1 (ppm)

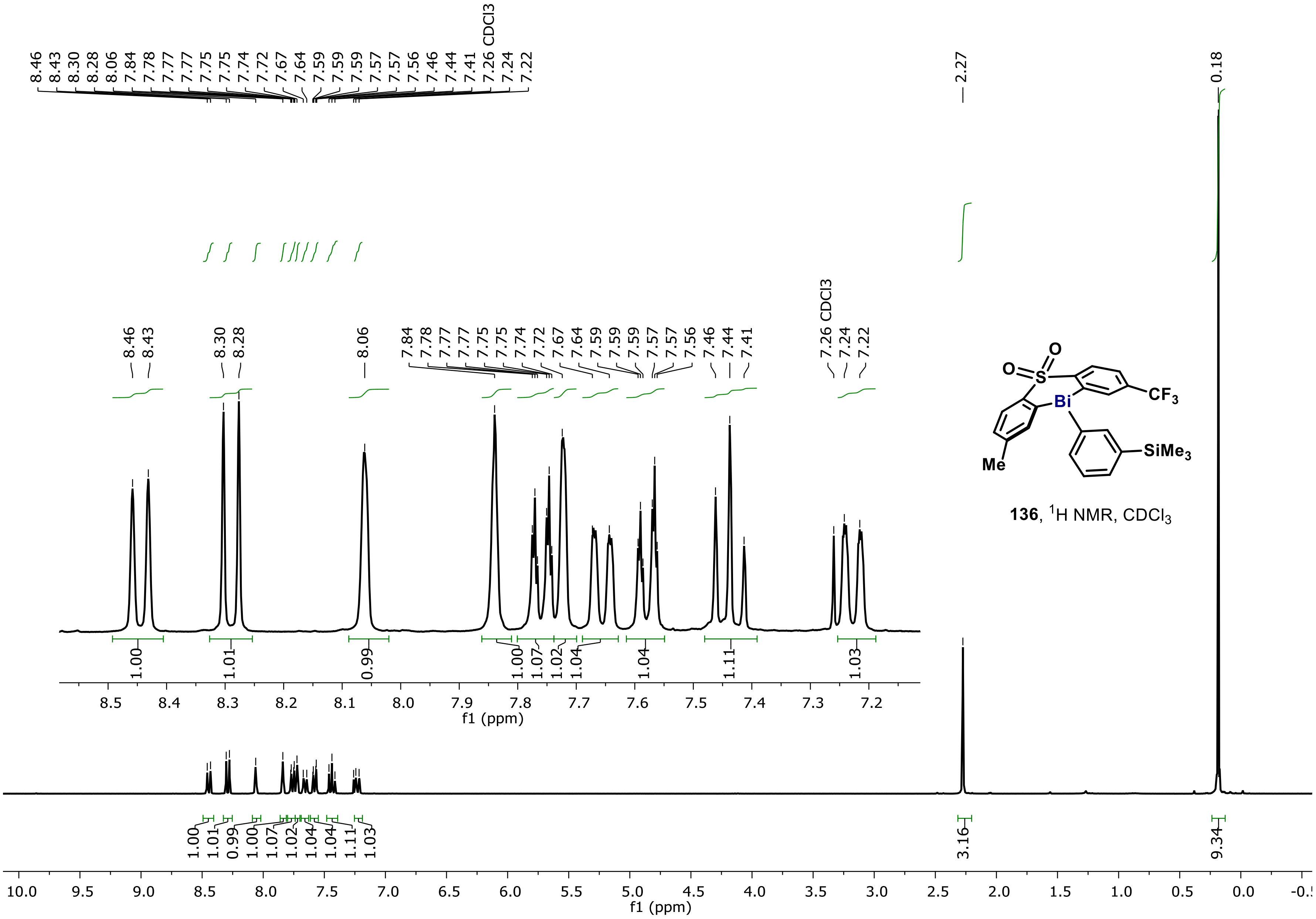
8.46
8.43
8.30
8.28
8.06
7.84
7.78
7.77
7.77
7.75
7.75
7.74
7.72
7.67
7.64
7.59
7.59
7.59
7.57
7.57
7.56
7.46
7.44
7.41
7.26 CDCl3
7.24
7.22
2.27
0.18
1.00
1.01
0.99
1.00
1.07
1.02
1.04
1.04
1.11
1.03
3.16
9.34
f1 (ppm)
Bi
SiMe3
CF3
Me
136, 1H NMR, CDCl3

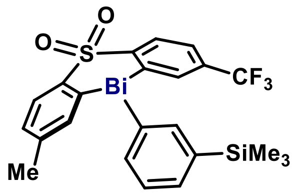

136, ^{13}C NMR, $CDCl_3$

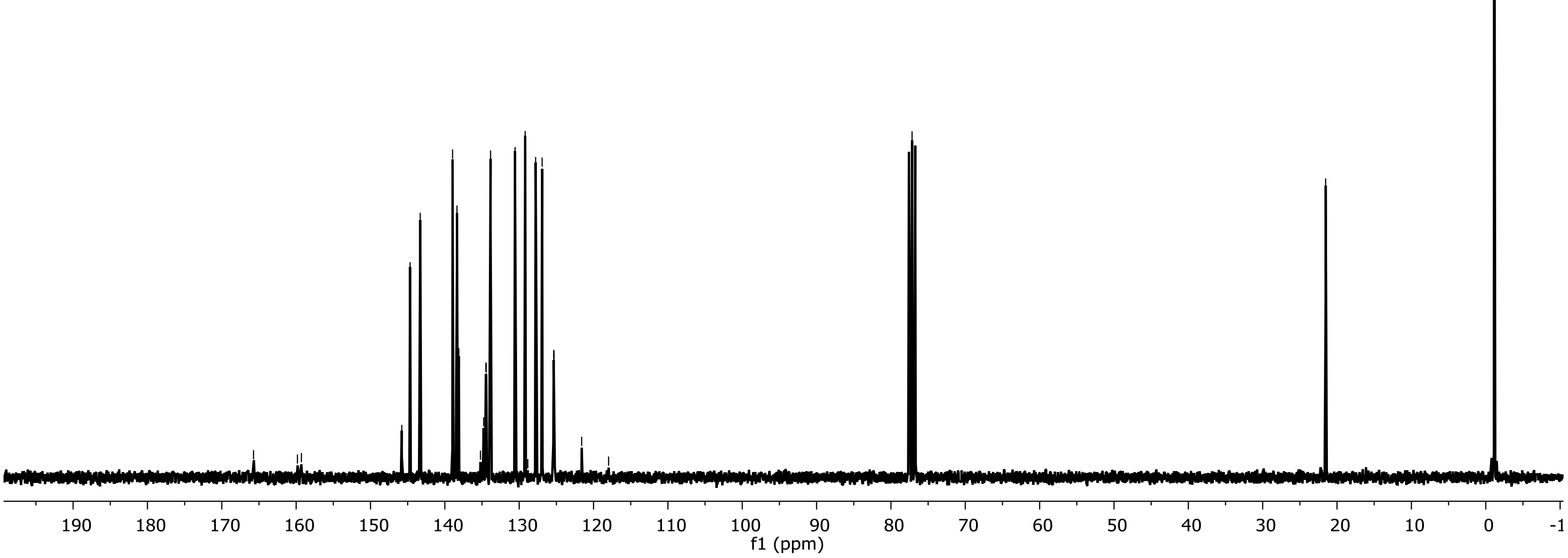

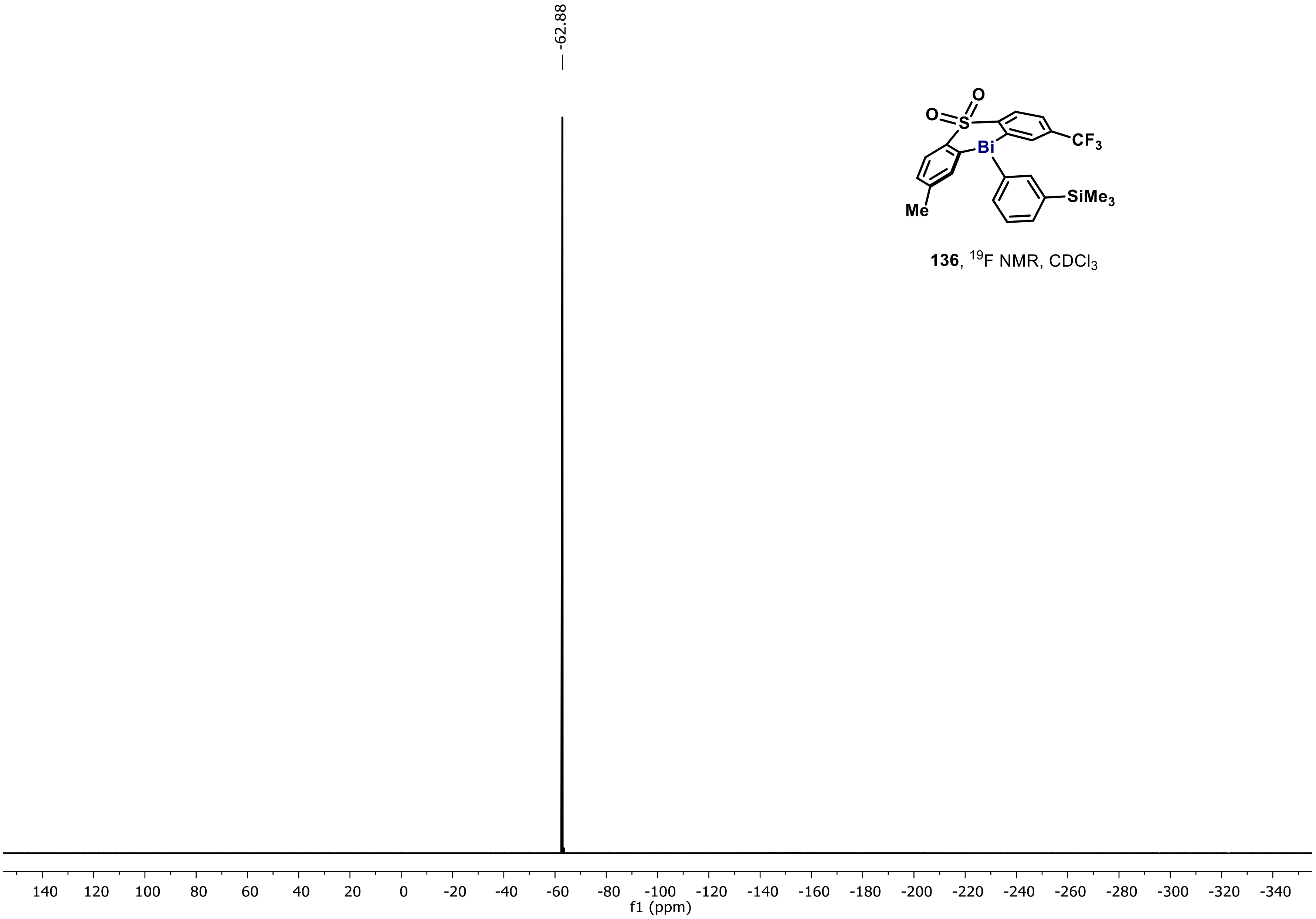

136, ^{19}F NMR, $CDCl_3$

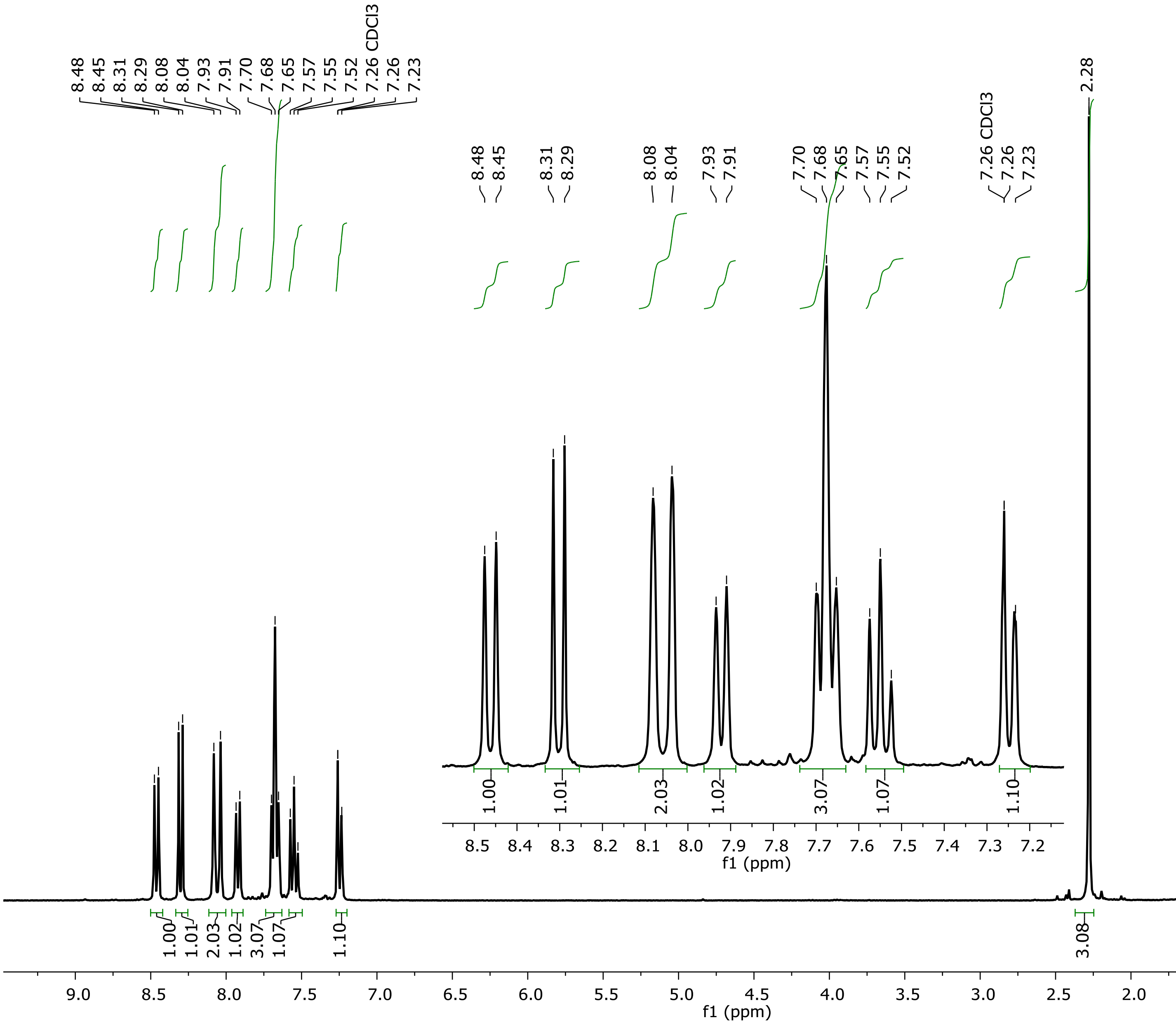

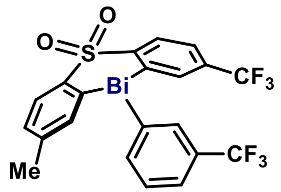

137, ^{1}H NMR, $CDCl_3$

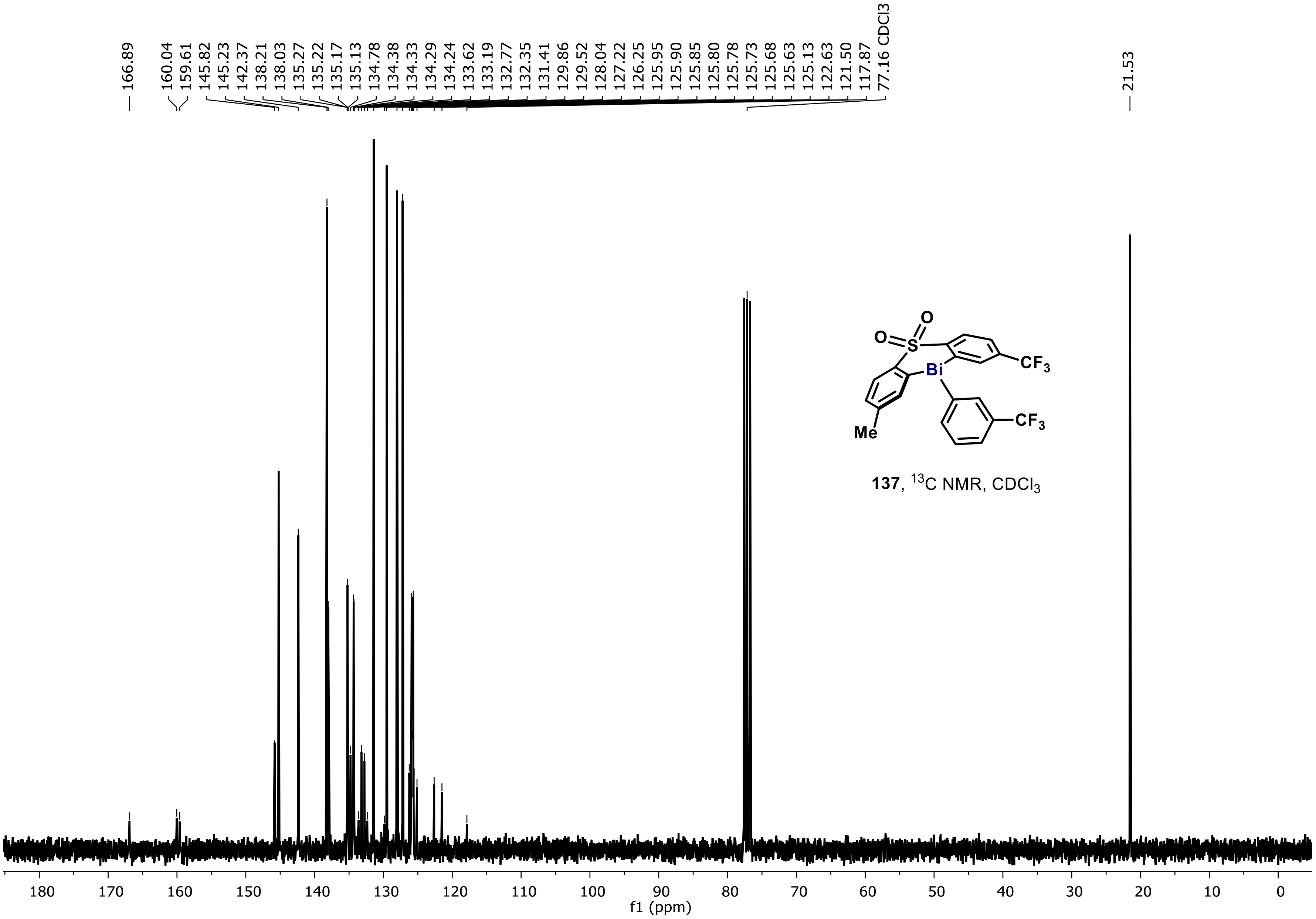
166.89
160.04
159.61
145.82
145.23
142.37
138.21
138.03
135.27
135.22
135.17
135.13
134.78
134.38
134.33
134.29
134.24
133.62
133.19
132.77
132.35
131.41
129.86
129.52
128.04
127.22
126.25
125.95
125.90
125.85
125.80
125.78
125.73
125.68
125.63
125.13
122.63
121.50
117.87
77.16 CDCl3
21.53
f1 (ppm)
O
O
S
Bi
CF3
CF3
Me
137, 13C NMR, CDCl3

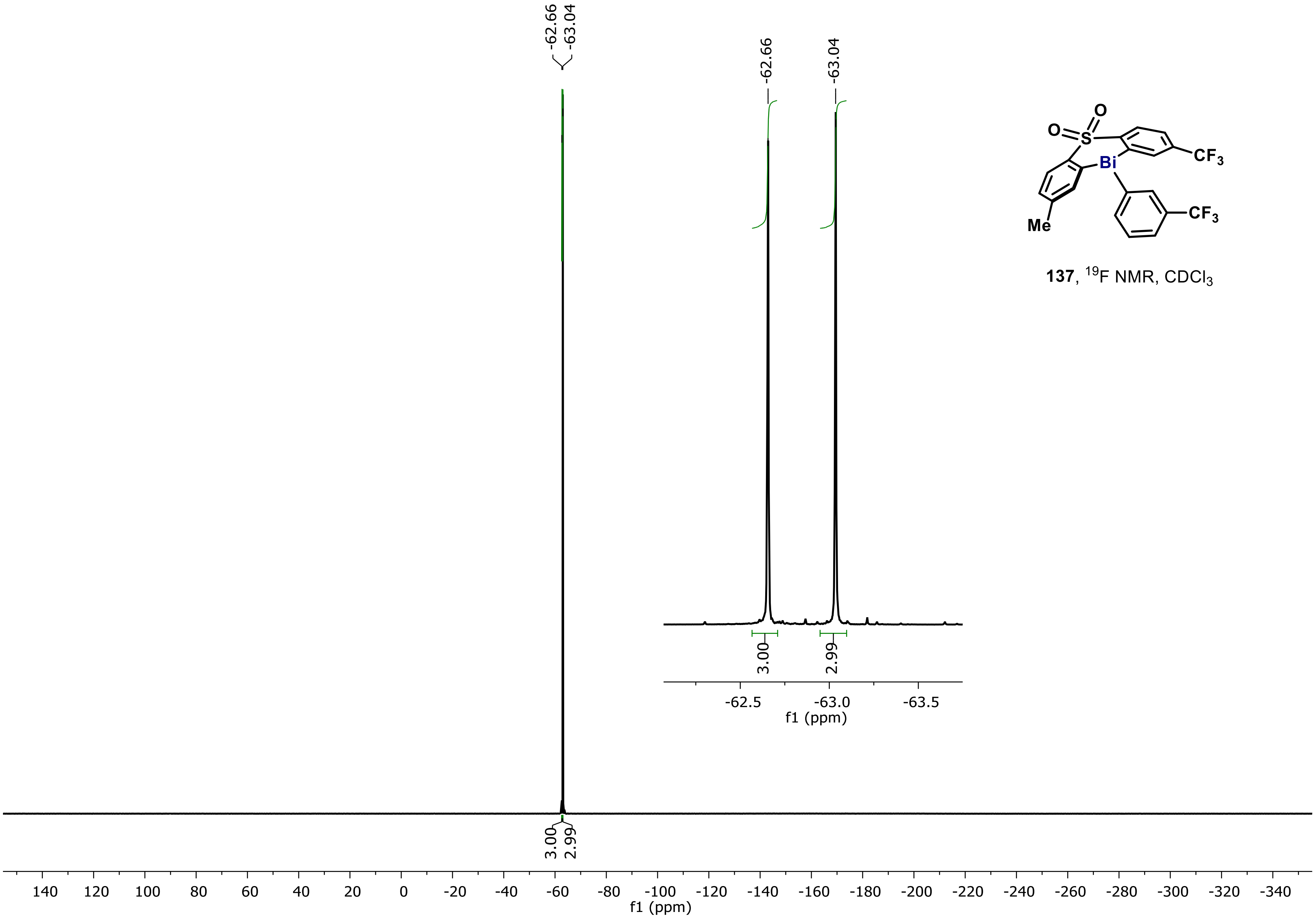

137, ^{19}F NMR, $CDCl_3$

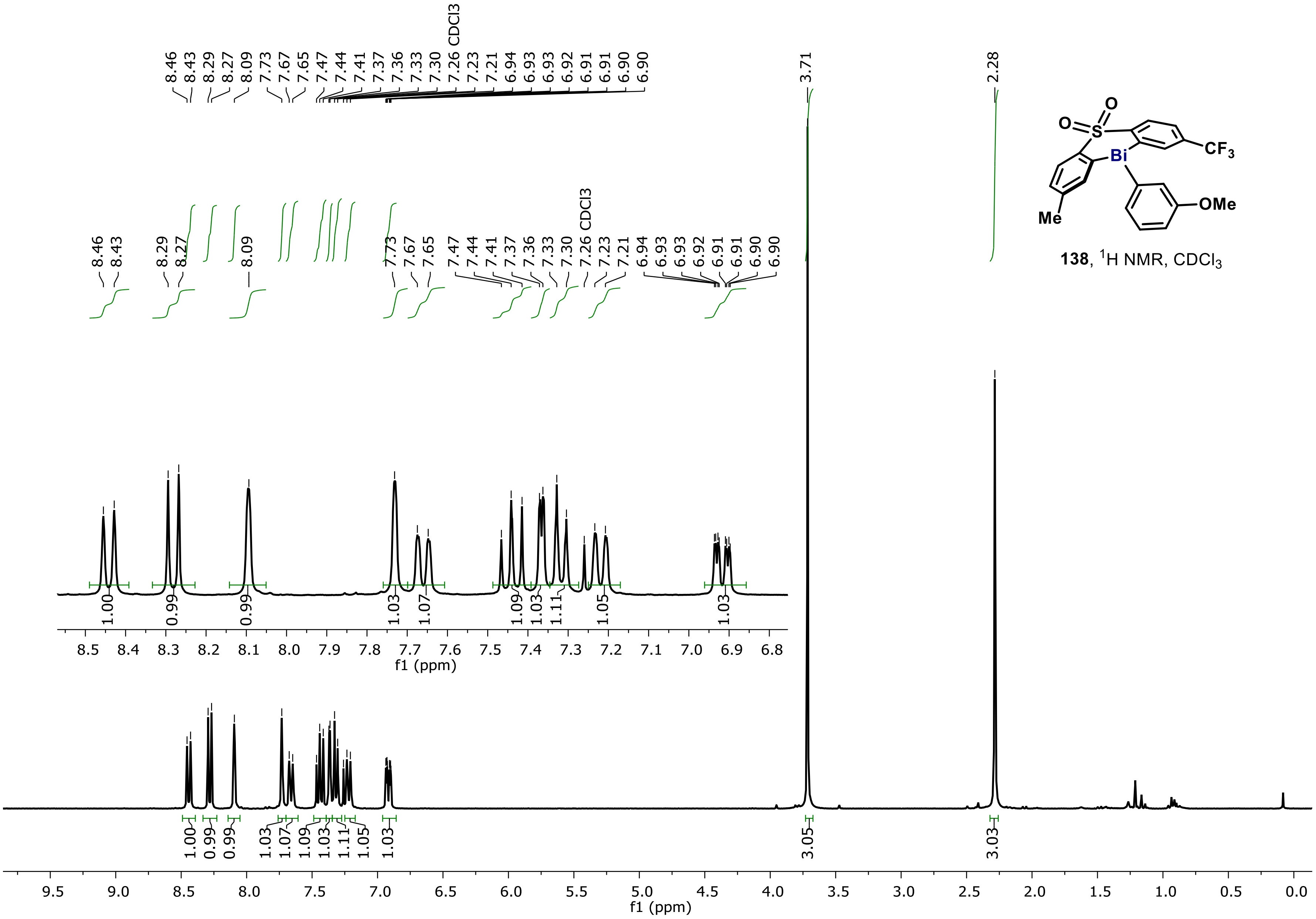
8.46 8.43 8.29 8.27 8.09 7.73 7.67 7.65 7.47 7.44 7.41 7.37 7.36 7.33 7.30 7.26 CDCl3 7.23 7.21 6.94 6.93 6.93 6.92 6.91 6.91 6.90 6.90
3.71
2.28
1.00 0.99 0.99 1.03 1.07 1.09 1.03 1.11 1.05 1.03
3.05
3.03
f1 (ppm)
Bi
CF3
OMe
Me
138, 1H NMR, CDCl3

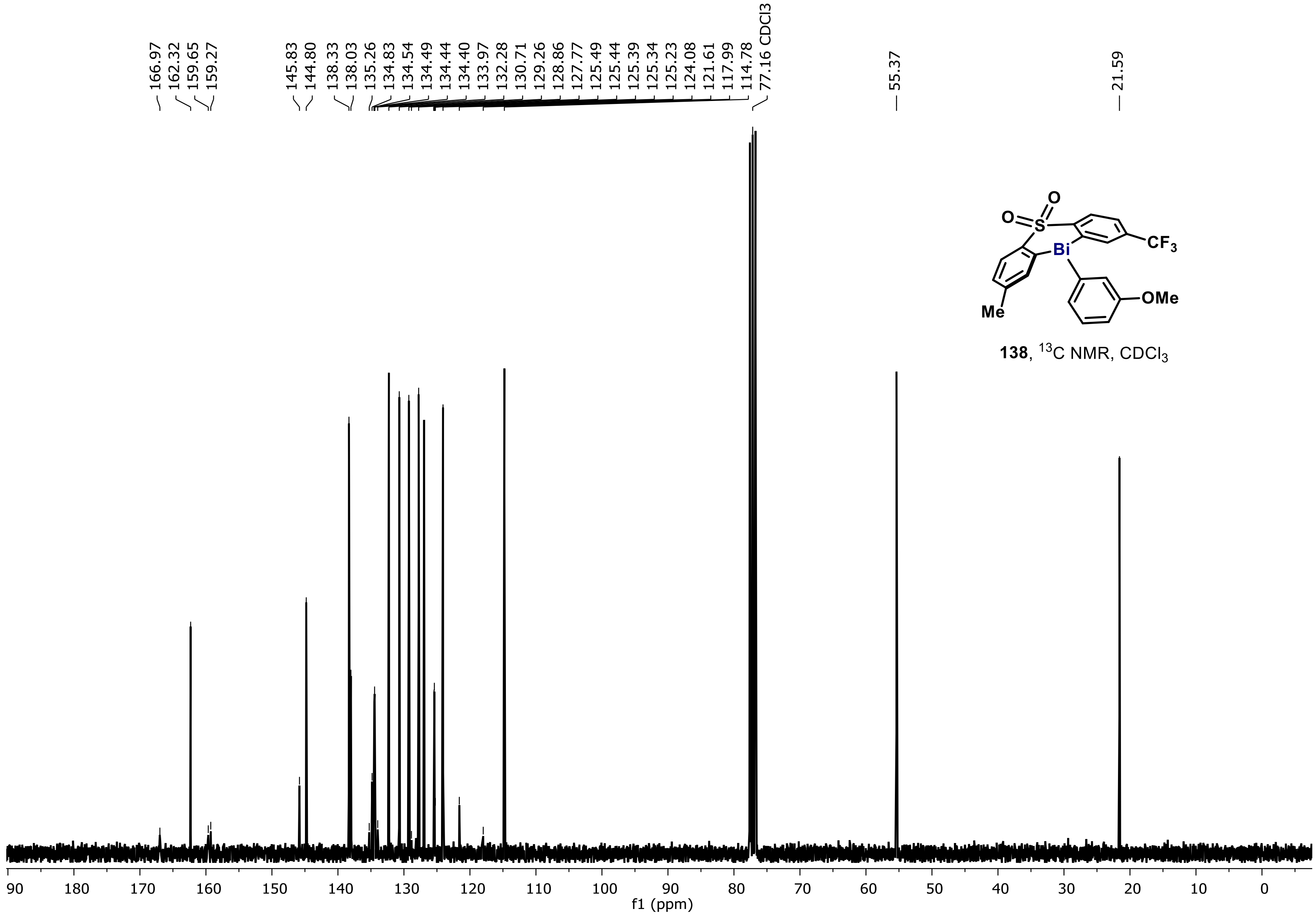

138, ^{13}C NMR, $CDCl_3$

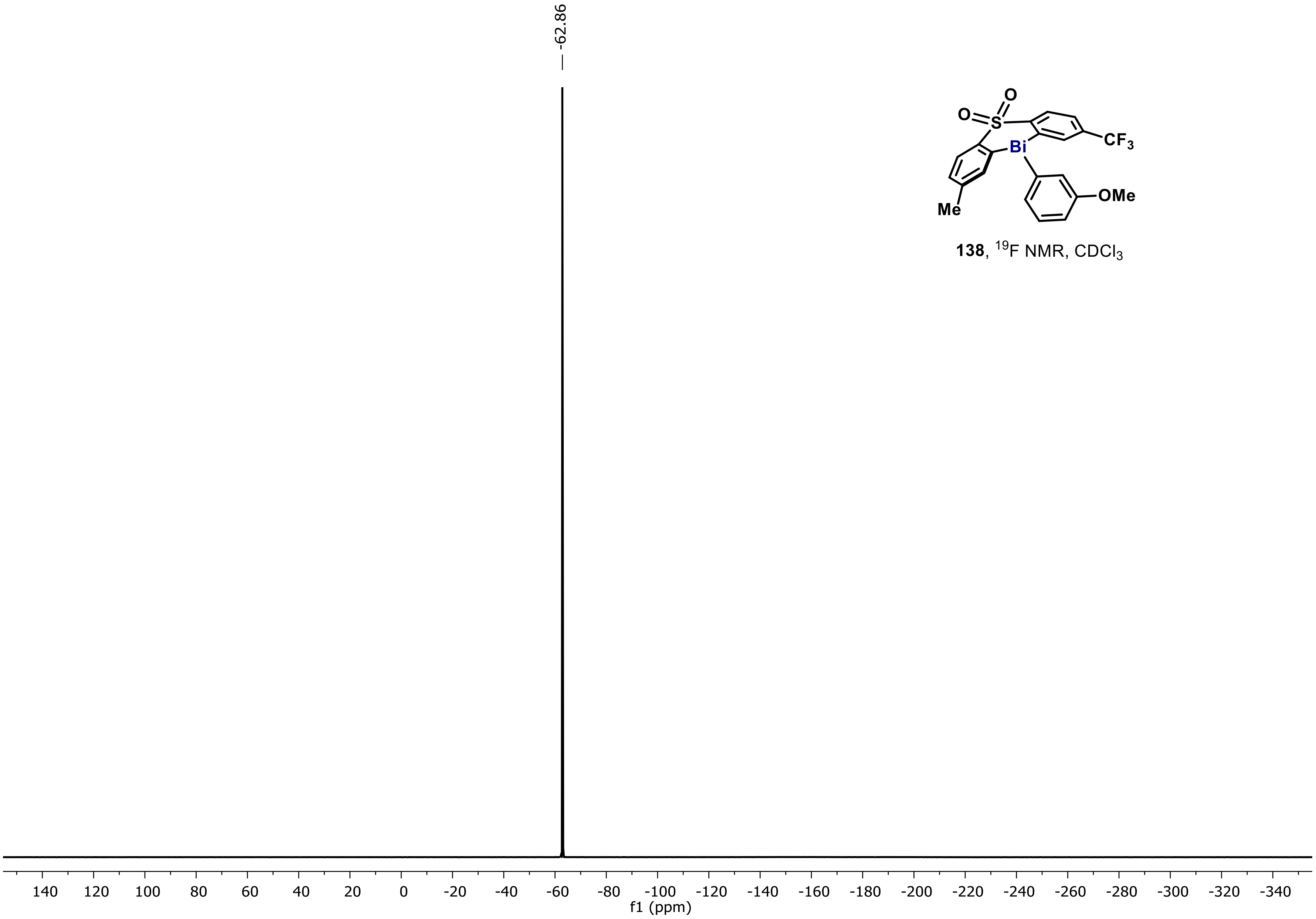

-62.86
O
O
S
CF3
Bi
OMe
Me
138, 19F NMR, CDCl3
140 120 100 80 60 40 20 0 -20 -40 -60 -80 -100 -120 -140 -160 -180 -200 -220 -240 -260 -280 -300 -320 -340
f1 (ppm)

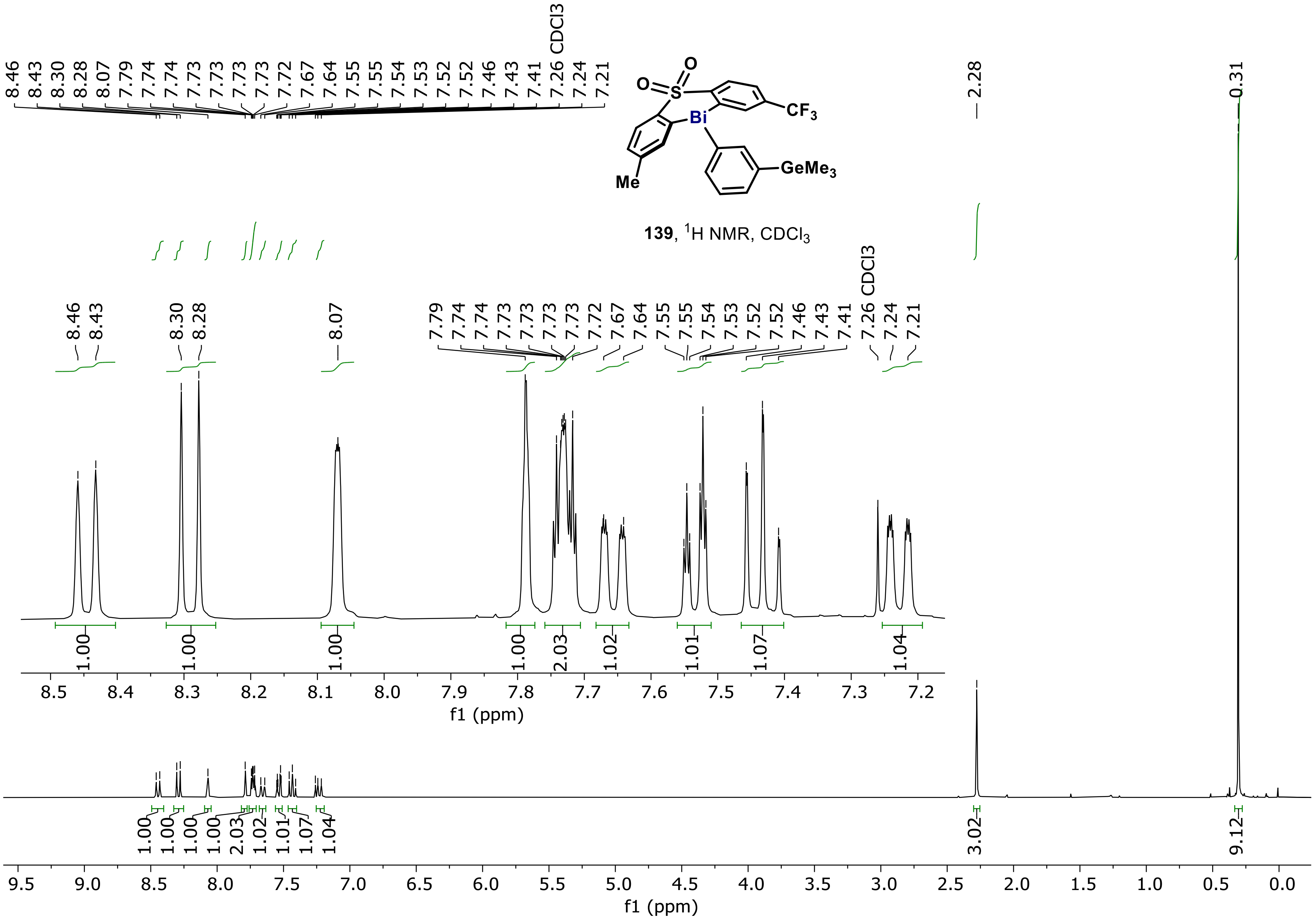

139, ^{1}H NMR, $CDCl_3$

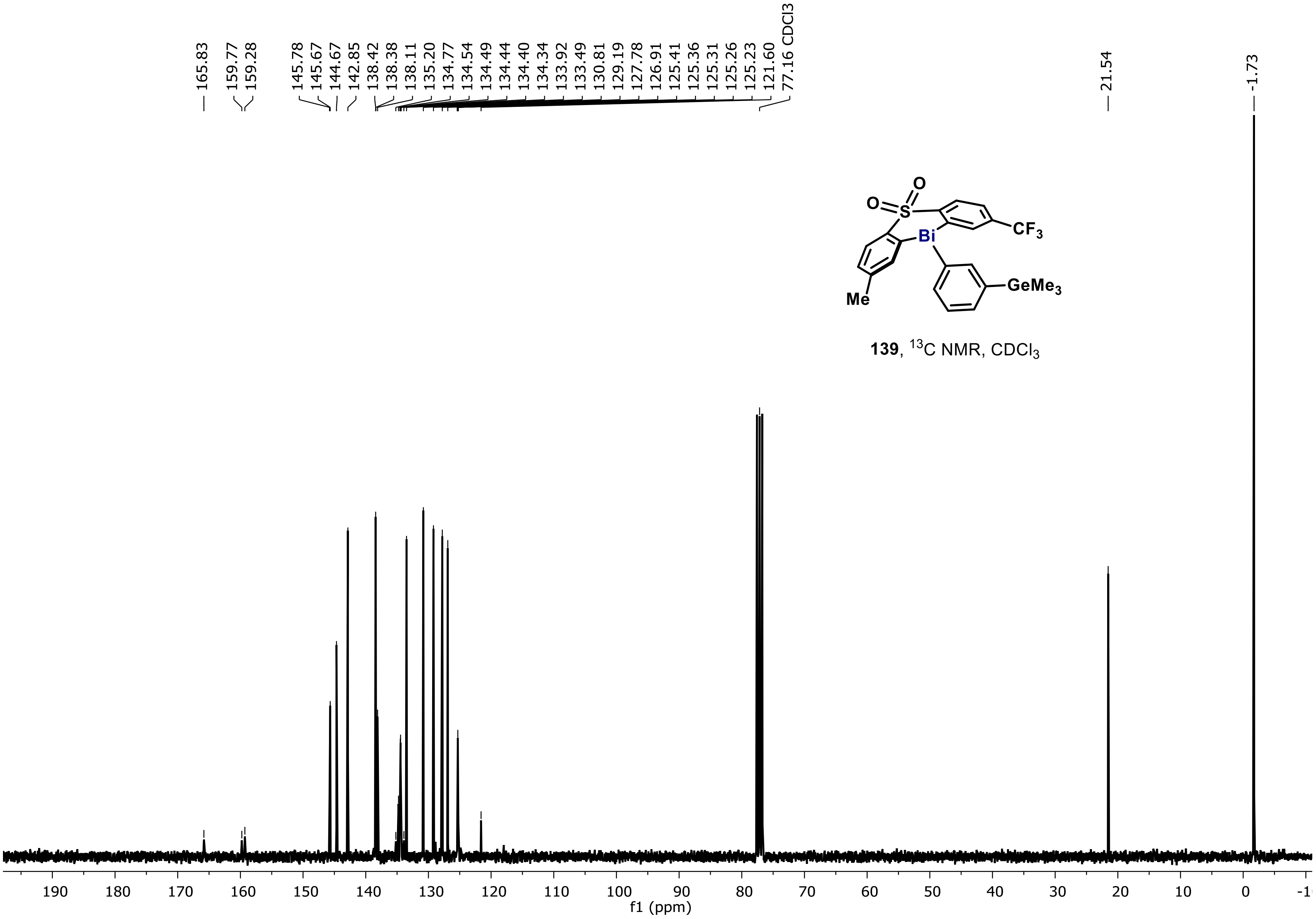

139, ^{13}C NMR, $CDCl_3$

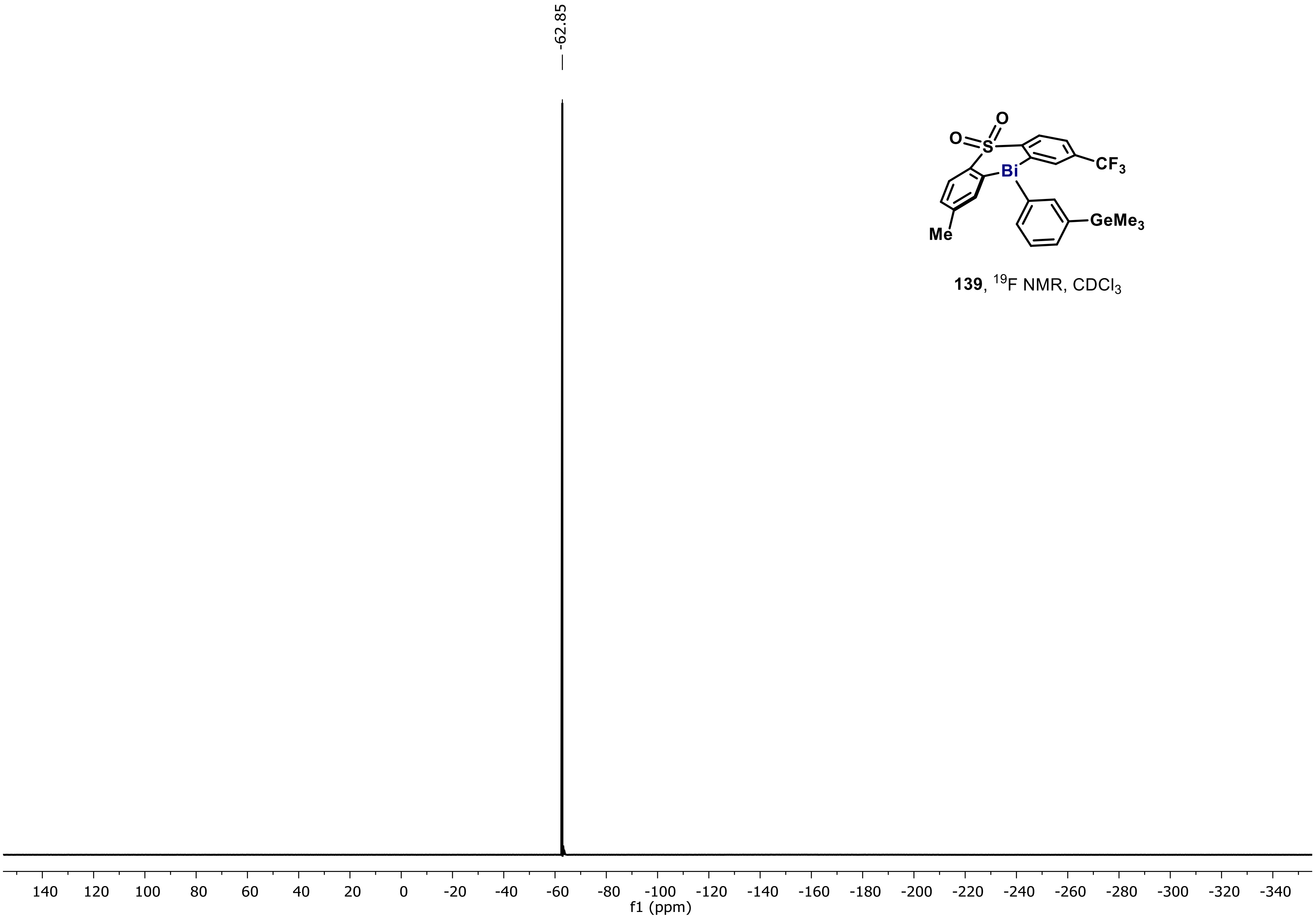

139, ^{19}F NMR, $CDCl_3$

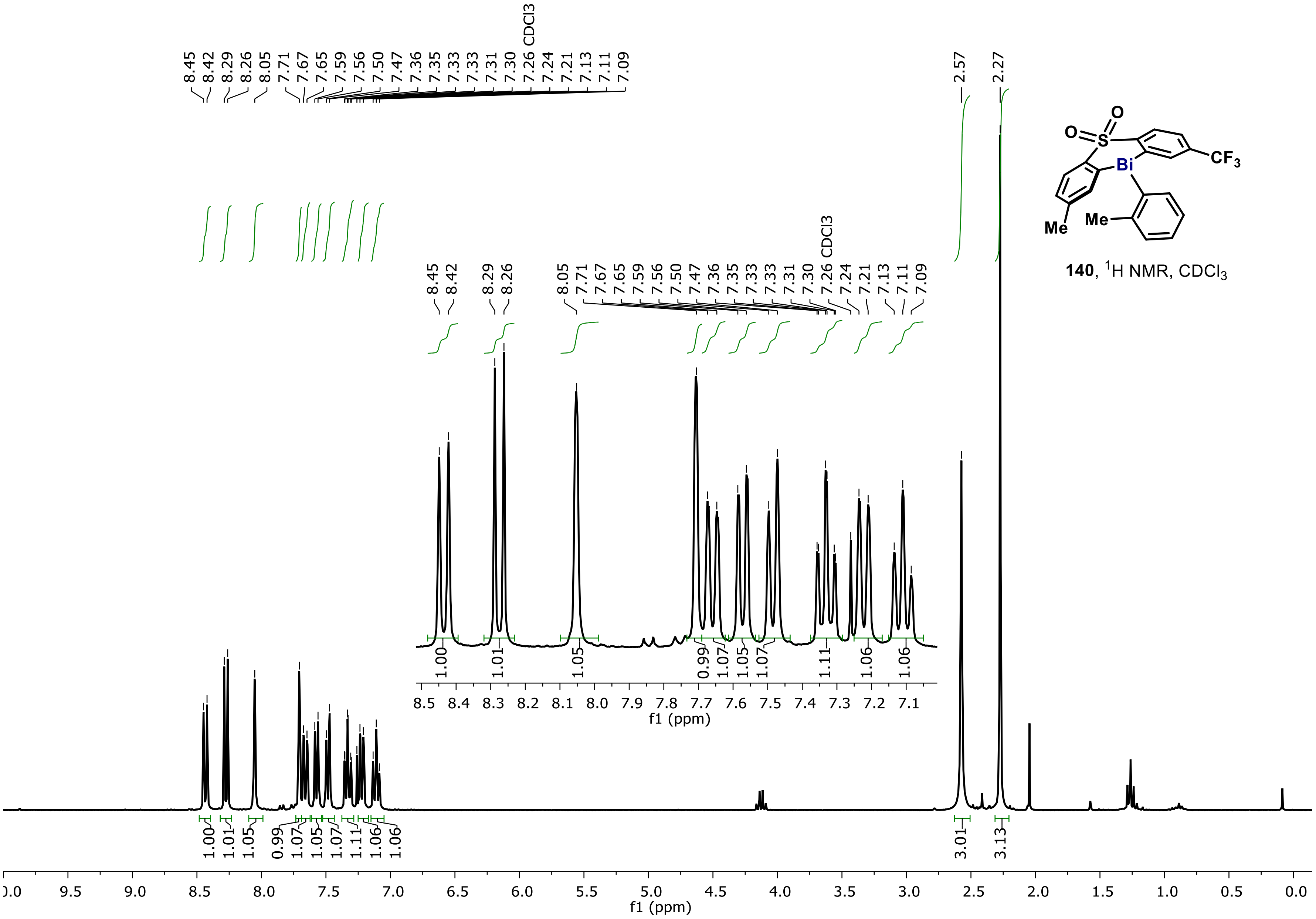

140, ^{1}H NMR, $CDCl_3$

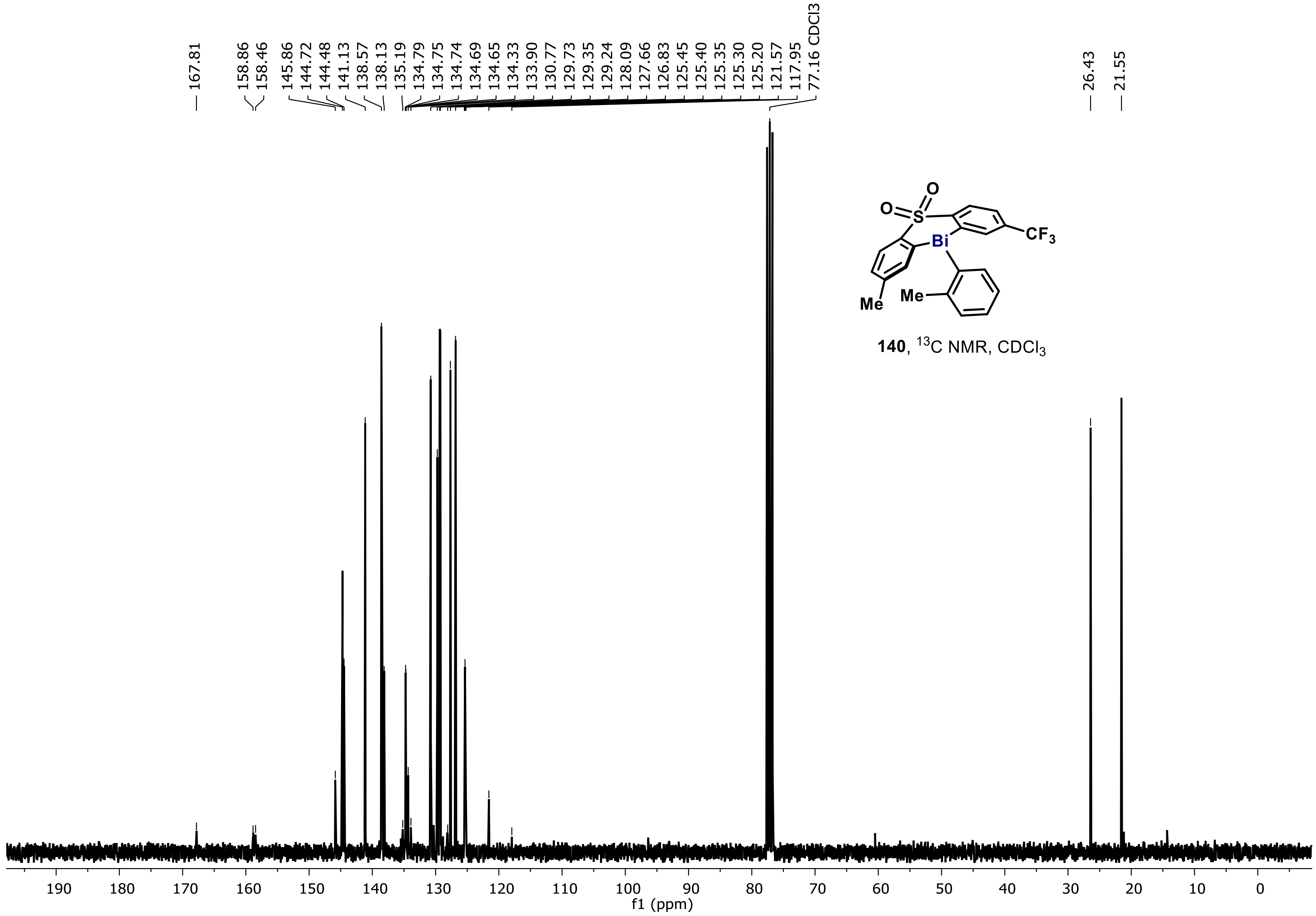

167.81
158.86
158.46
145.86
144.72
144.48
141.13
138.57
138.13
135.19
134.79
134.75
134.74
134.69
134.65
134.33
133.90
130.77
129.73
129.35
129.24
128.09
127.66
126.83
125.45
125.40
125.35
125.30
125.20
121.57
117.95
77.16 CDCl3
26.43
21.55
O
O
S
Bi
CF3
Me
Me
140, 13C NMR, CDCl3
190 180 170 160 150 140 130 120 110 100 90 80 70 60 50 40 30 20 10 0
f1 (ppm)

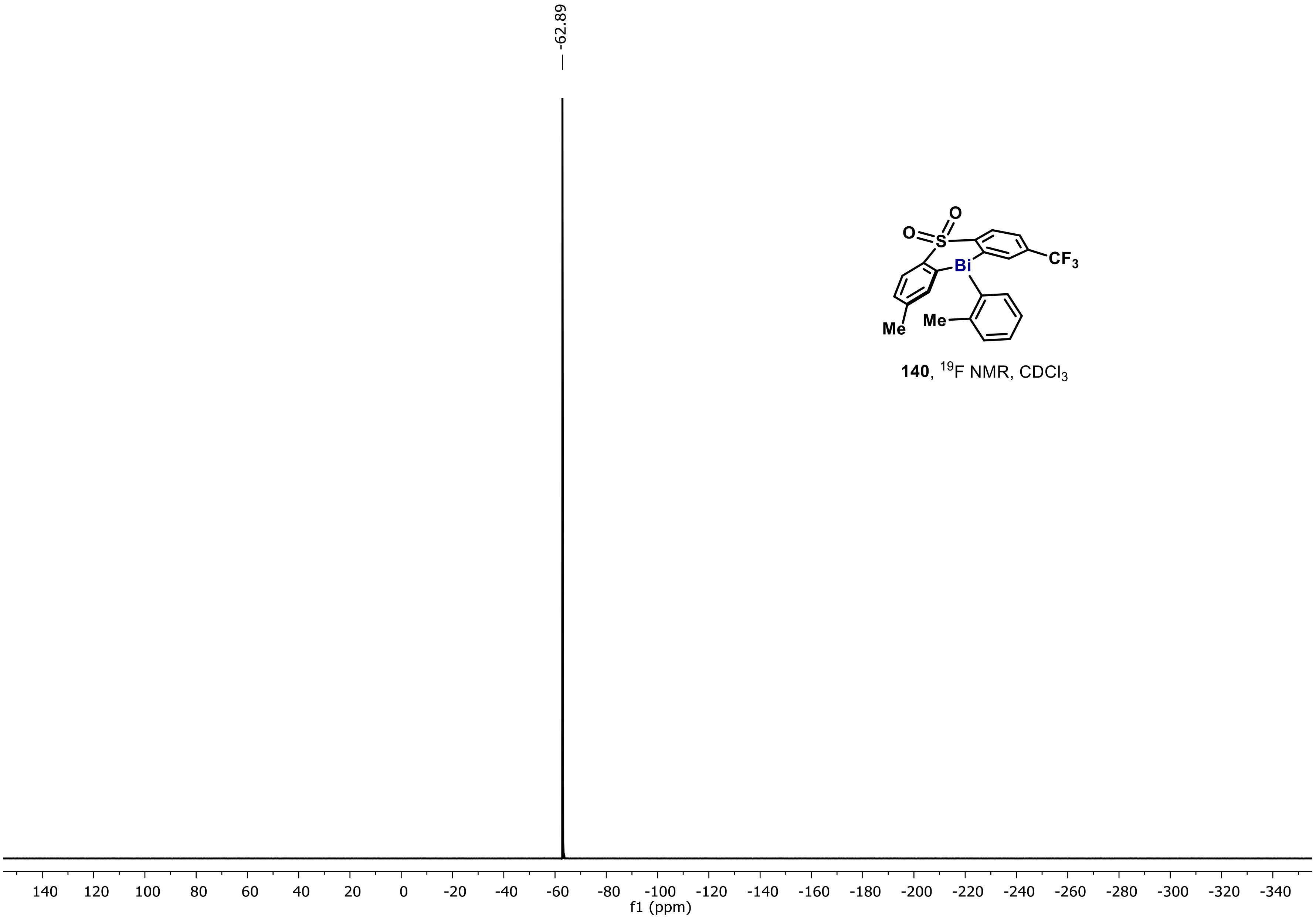
-62.89
O
O
S
Bi
CF3
Me
Me
140, 19F NMR, CDCl3
140 120 100 80 60 40 20 0 -20 -40 -60 -80 -100 -120 -140 -160 -180 -200 -220 -240 -260 -280 -300 -320 -340
f1 (ppm)

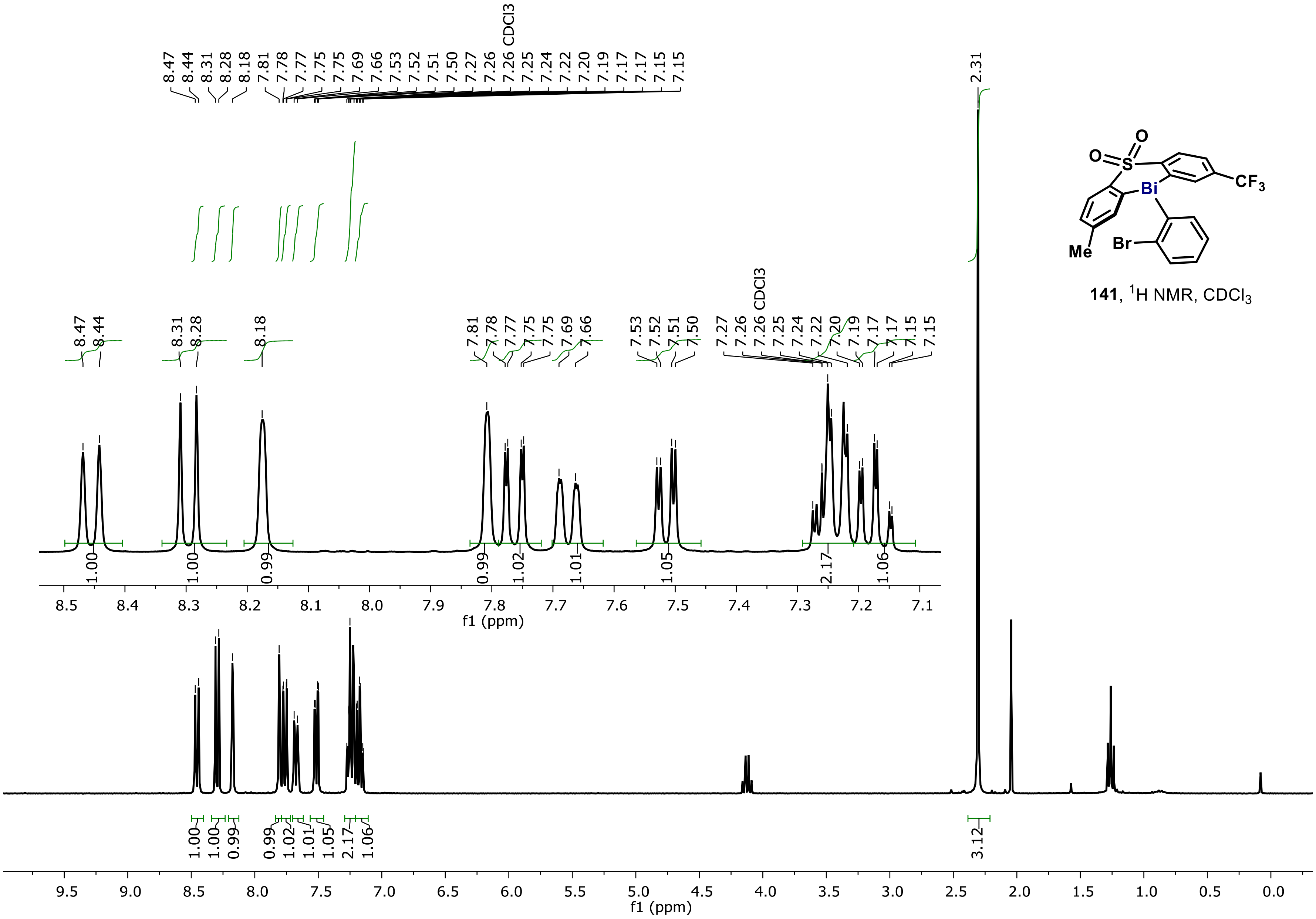

141, ^{1}H NMR, $CDCl_3$

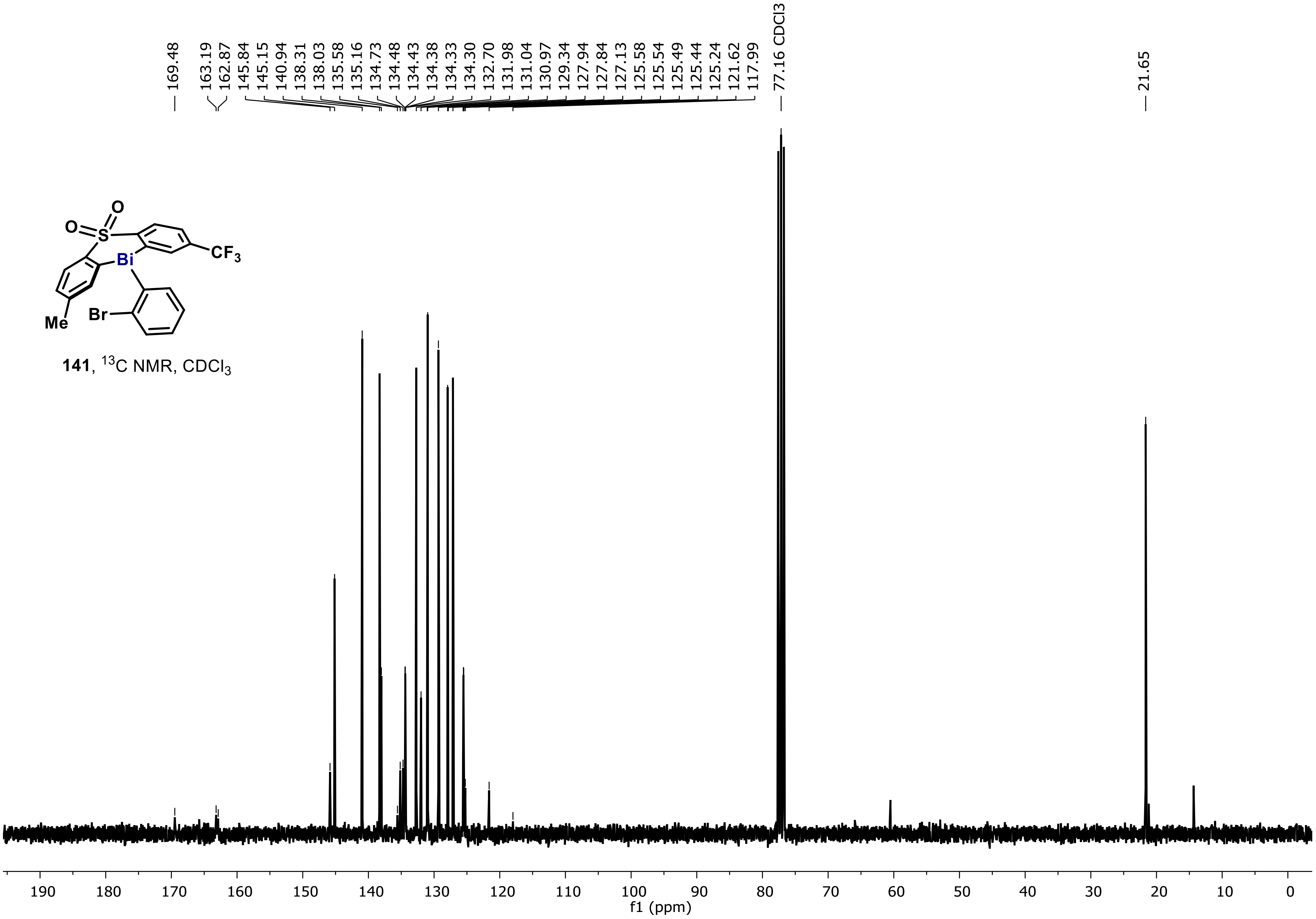

141, ^{13}C NMR, $CDCl_3$

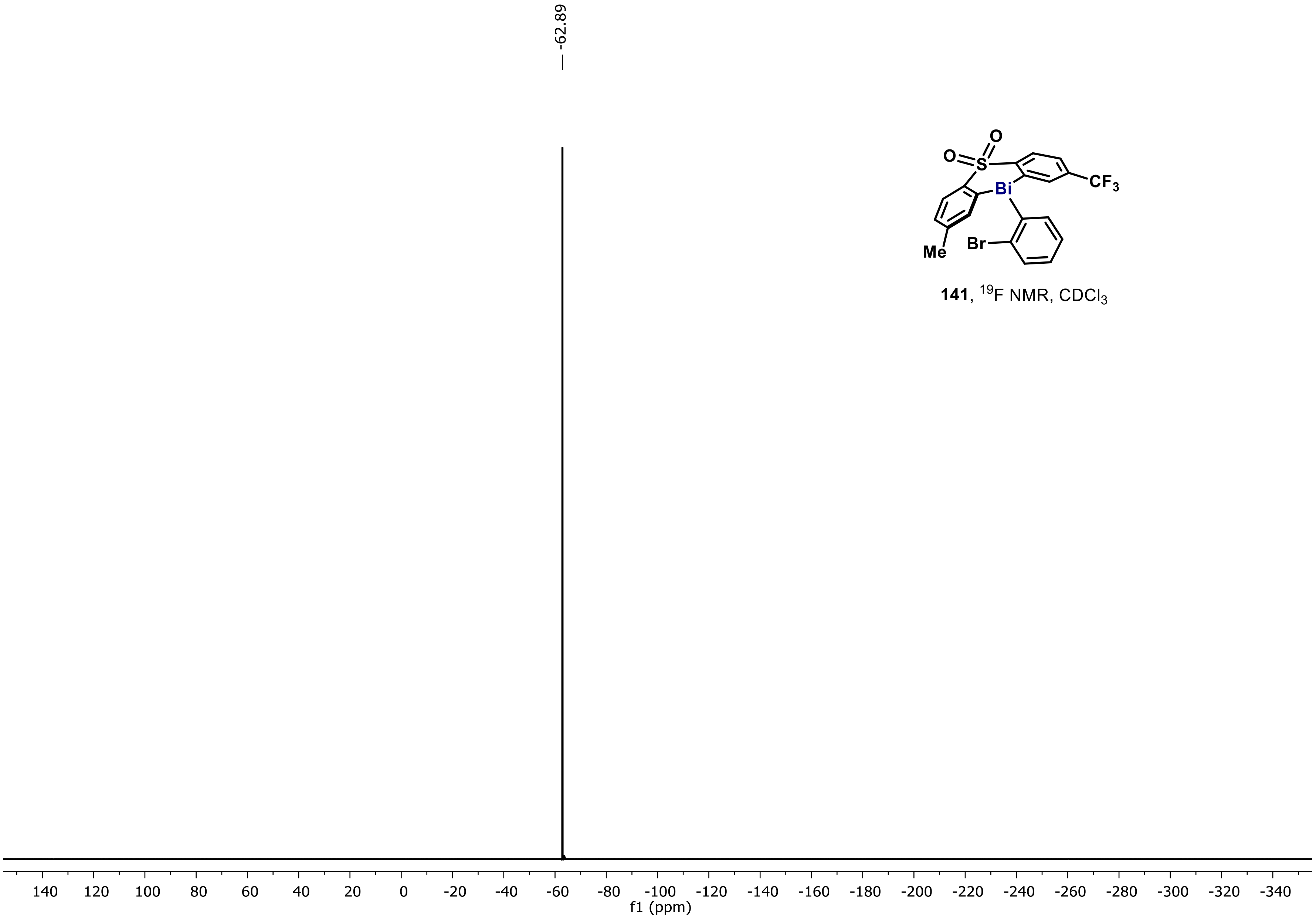

141, ^{19}F NMR, $CDCl_3$

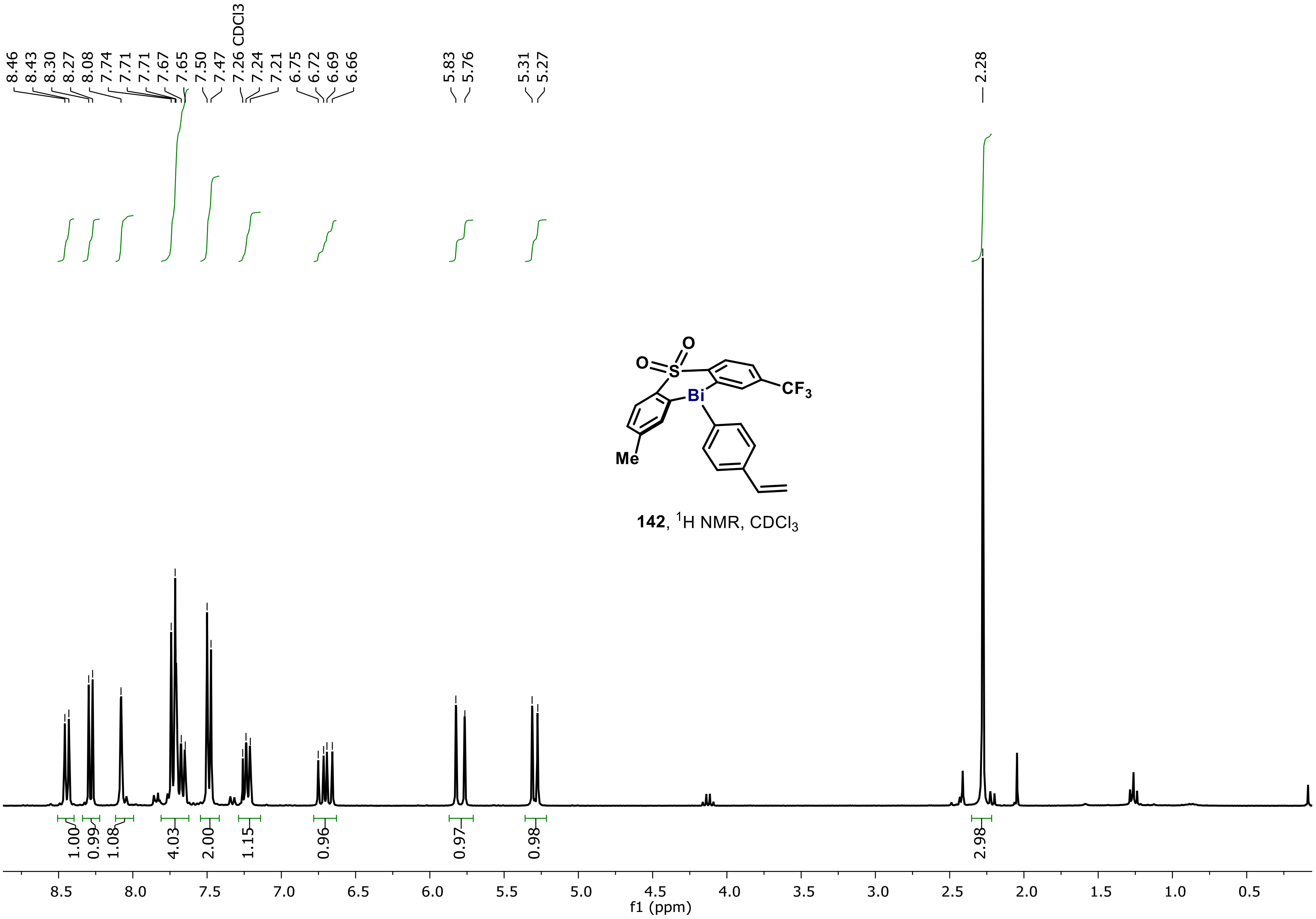
8.46
8.43
8.30
8.27
8.08
7.74
7.71
7.71
7.67
7.65
7.50
7.47
7.26 CDCl3
7.24
7.21
6.75
6.72
6.69
6.66
5.83
5.76
5.31
5.27
2.28
O
O
S
Bi
CF3
Me
142, 1H NMR, CDCl3
1.00
0.99
1.08
4.03
2.00
1.15
0.96
0.97
0.98
2.98
8.5
8.0
7.5
7.0
6.5
6.0
5.5
5.0
4.5
4.0
3.5
3.0
2.5
2.0
1.5
1.0
0.5
f1 (ppm)

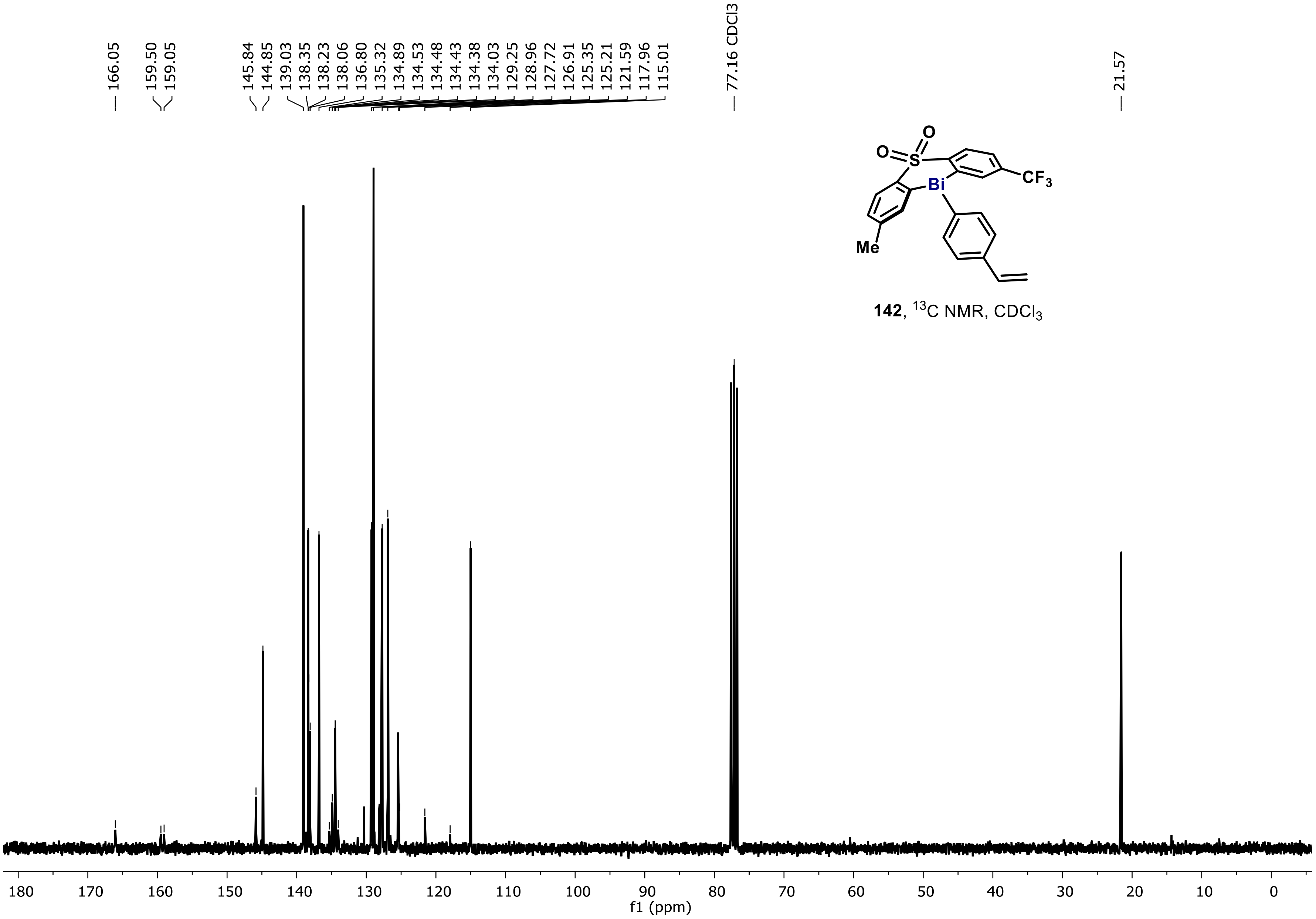

142, ^{13}C NMR, $CDCl_3$

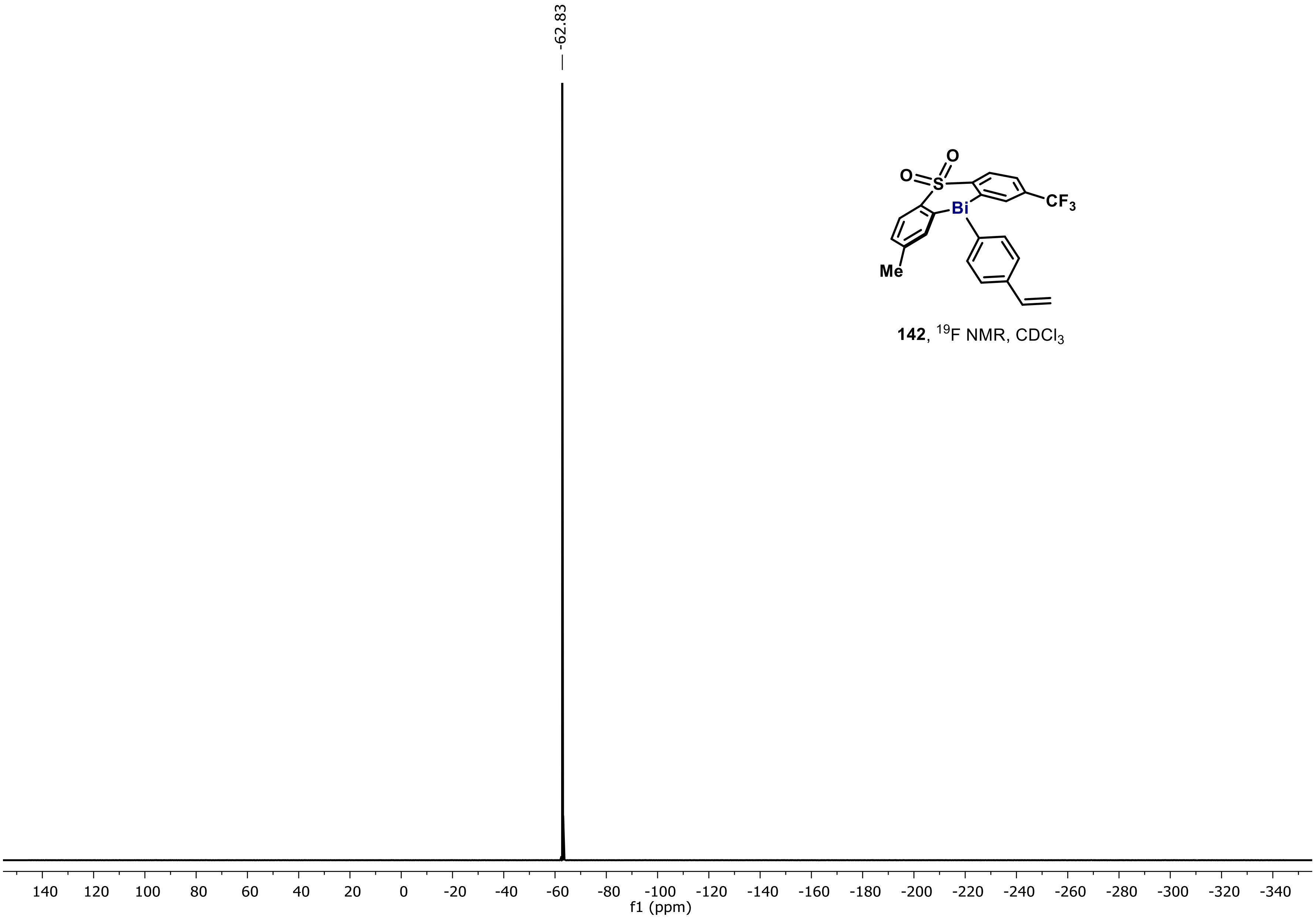
-62.83
O
O
S
Bi
CF3
Me
142, 19F NMR, CDCl3
140 120 100 80 60 40 20 0 -20 -40 -60 -80 -100 -120 -140 -160 -180 -200 -220 -240 -260 -280 -300 -320 -340
f1 (ppm)

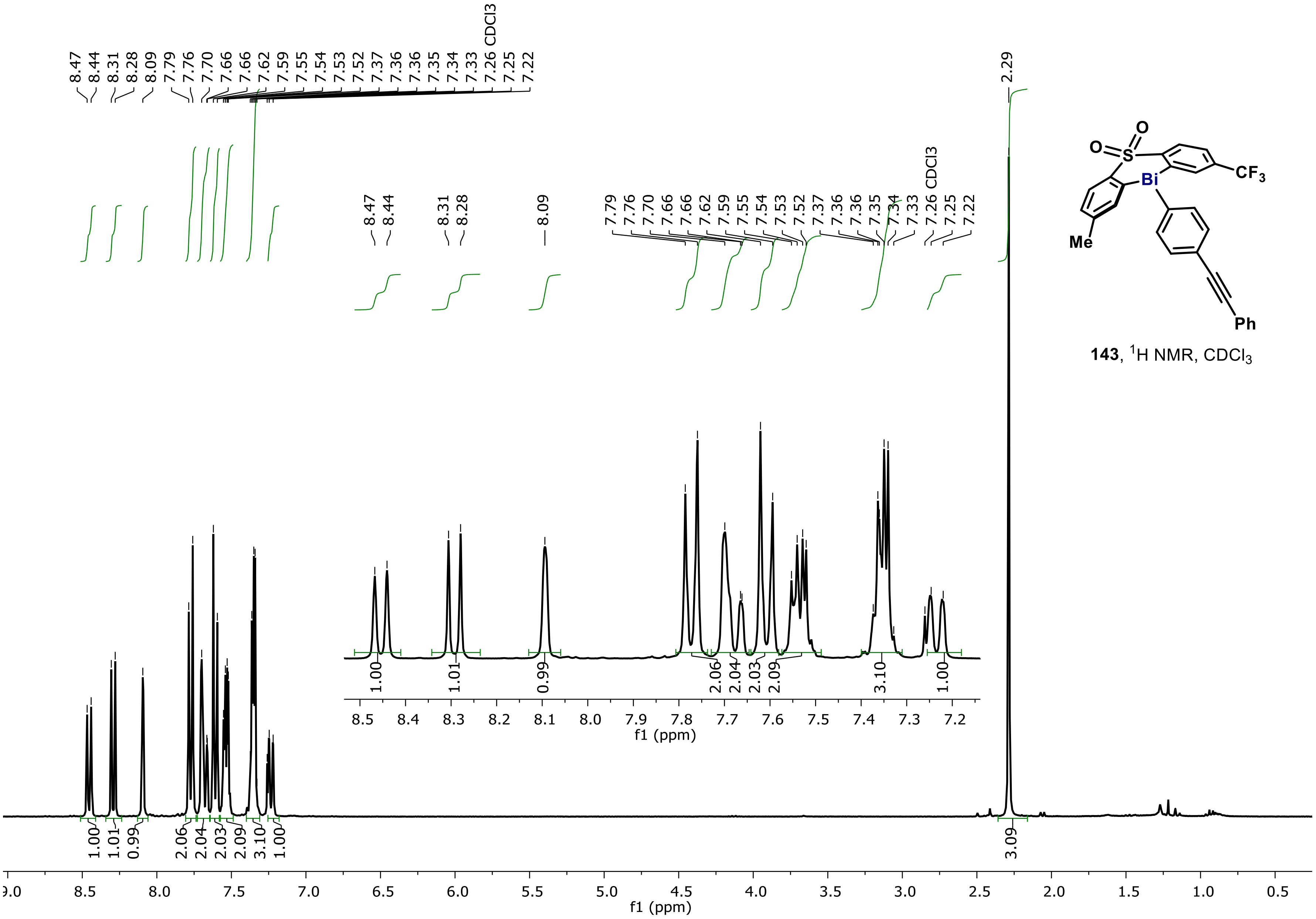

143, ^{1}H NMR, $CDCl_3$

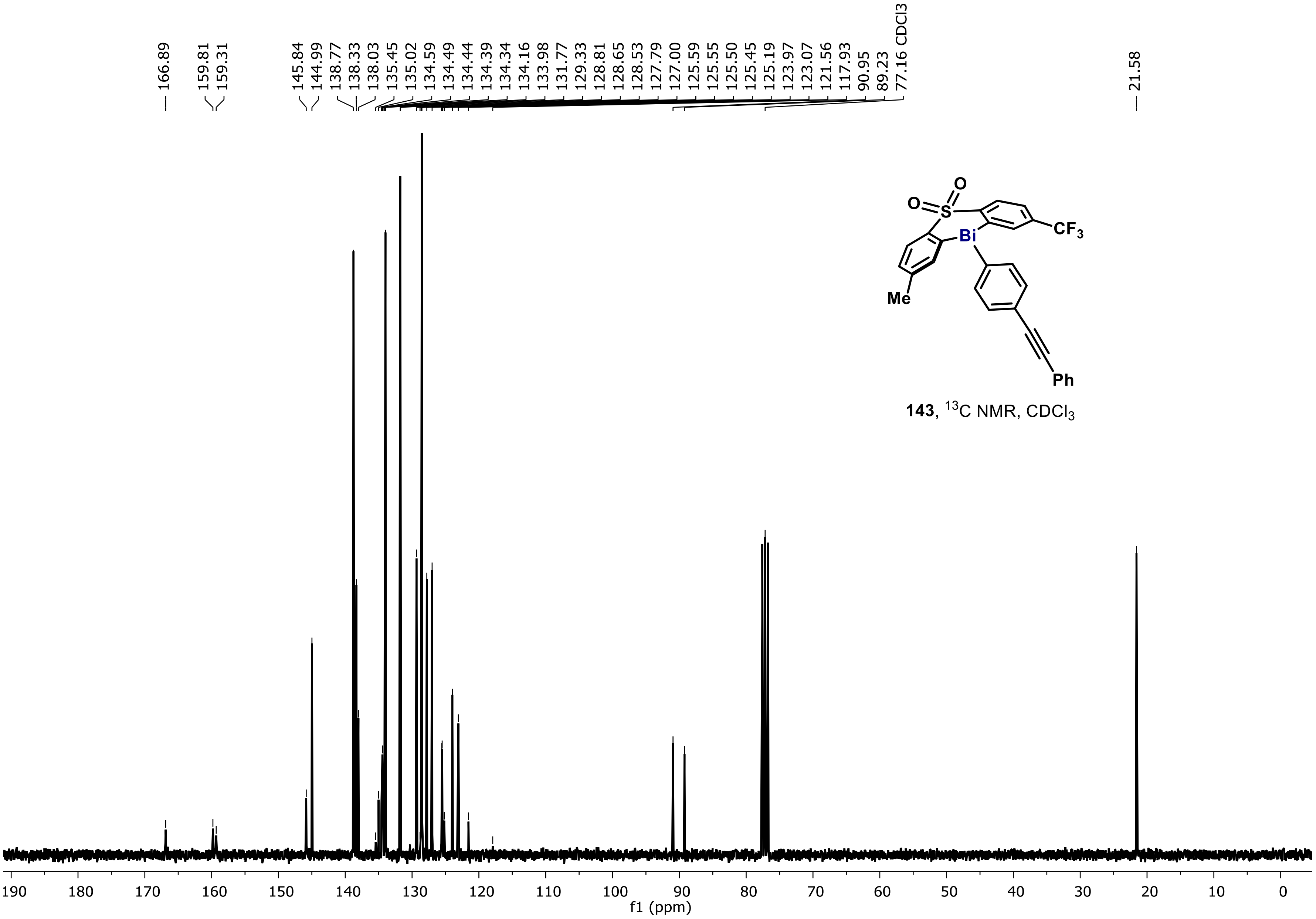

143, ^{13}C NMR, $CDCl_3$

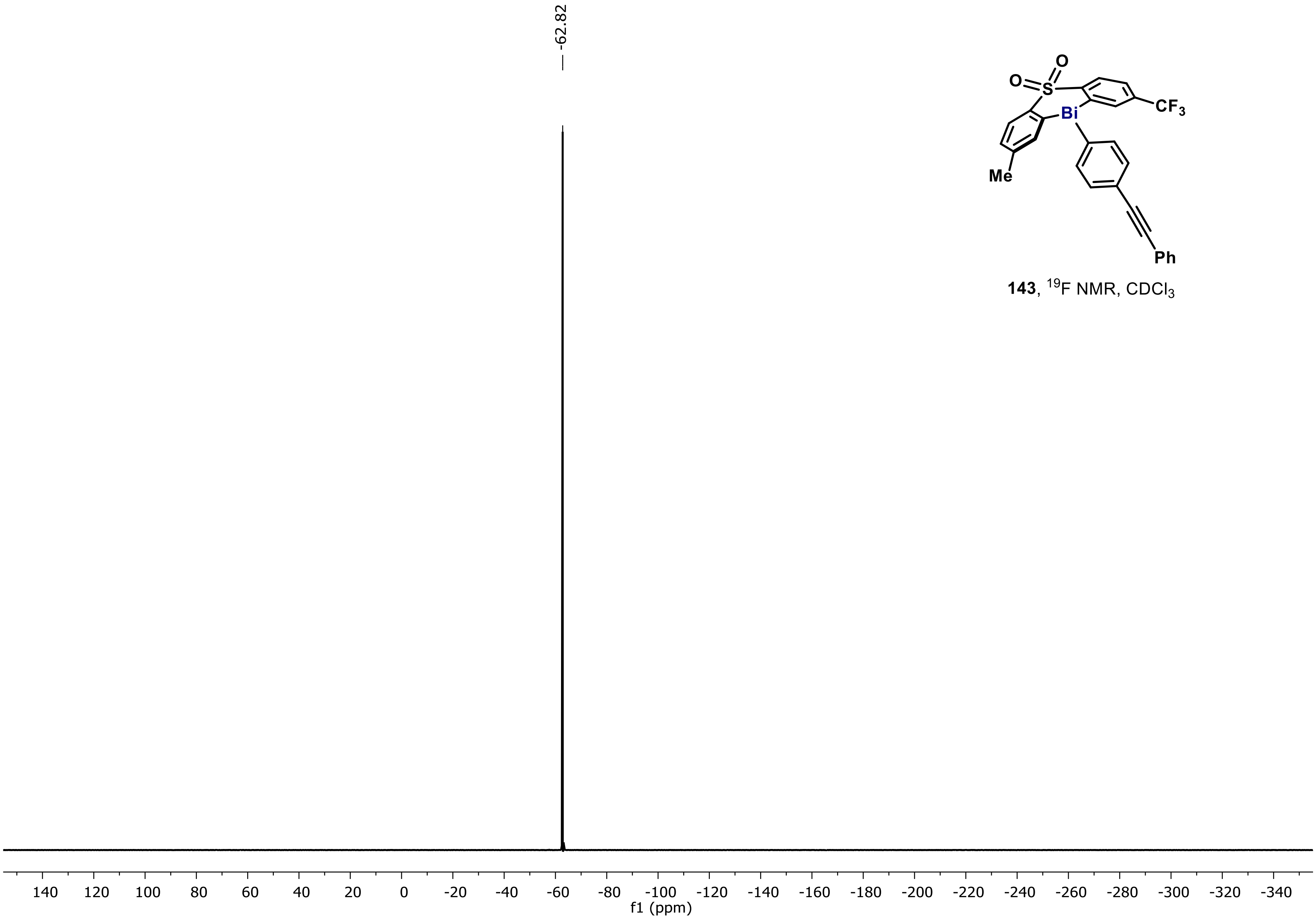

143, ^{19}F NMR, $CDCl_3$

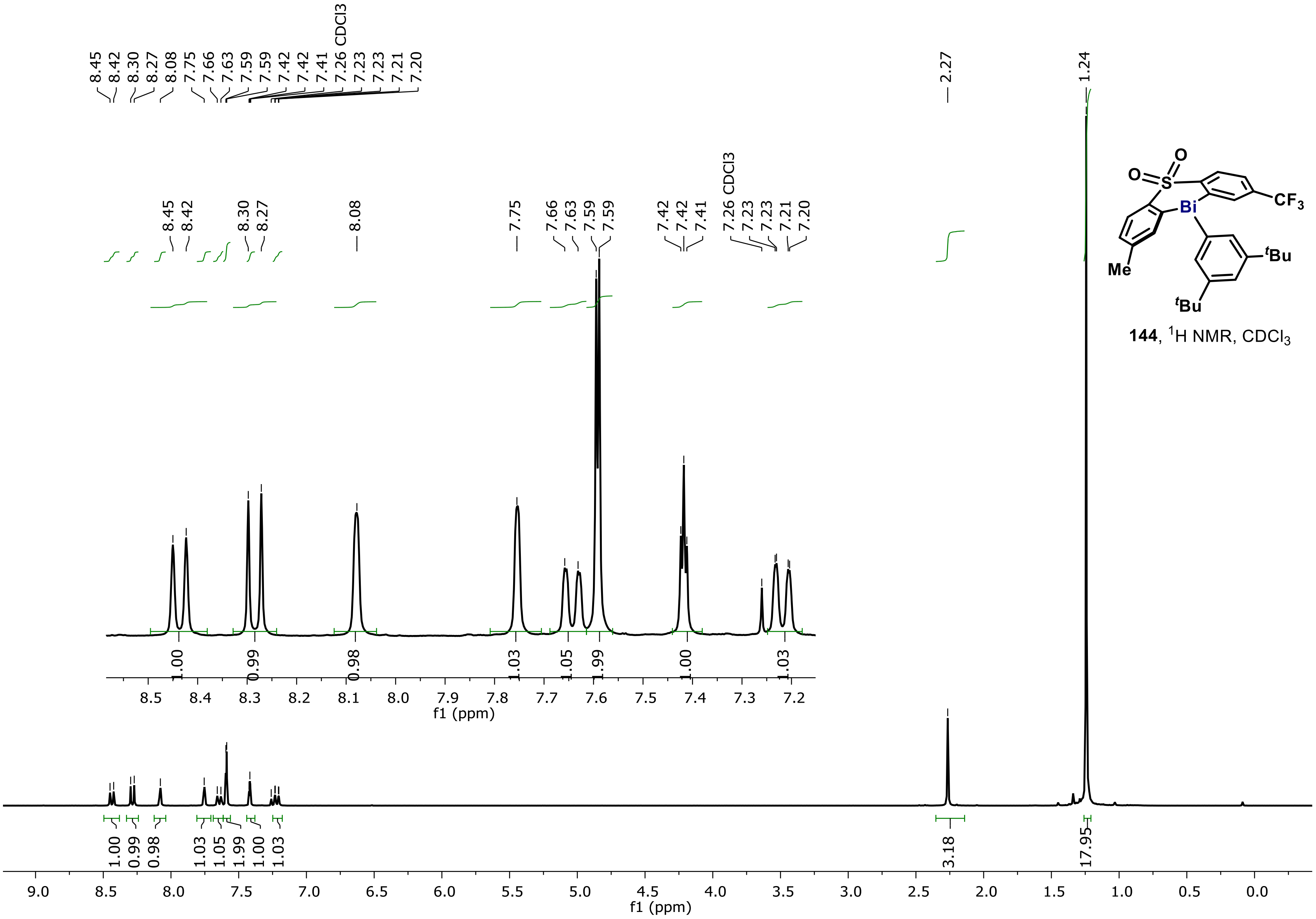

144, ¹H NMR, CDCl3
f1 (ppm)

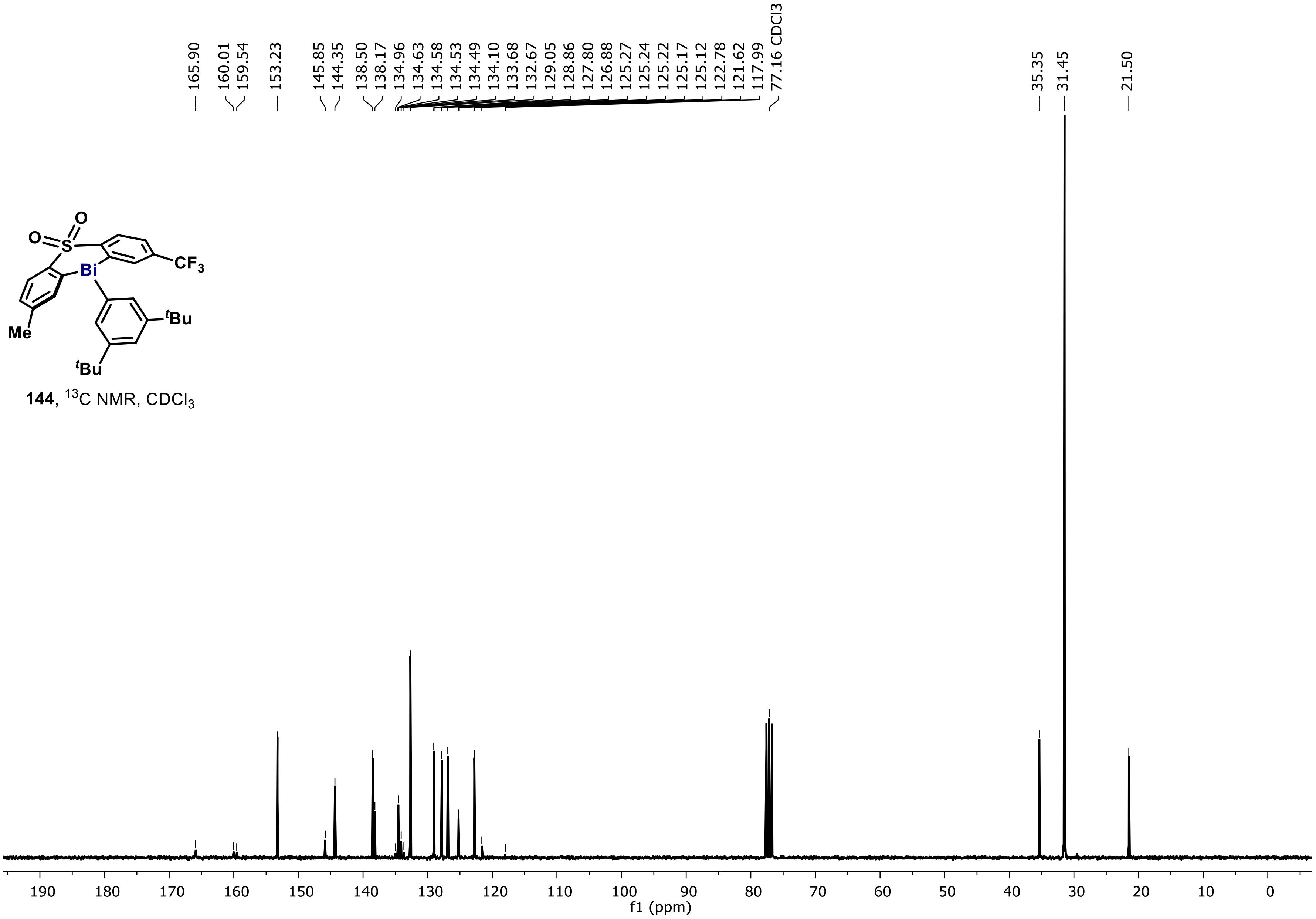

144, ^{13}C NMR, $CDCl_3$

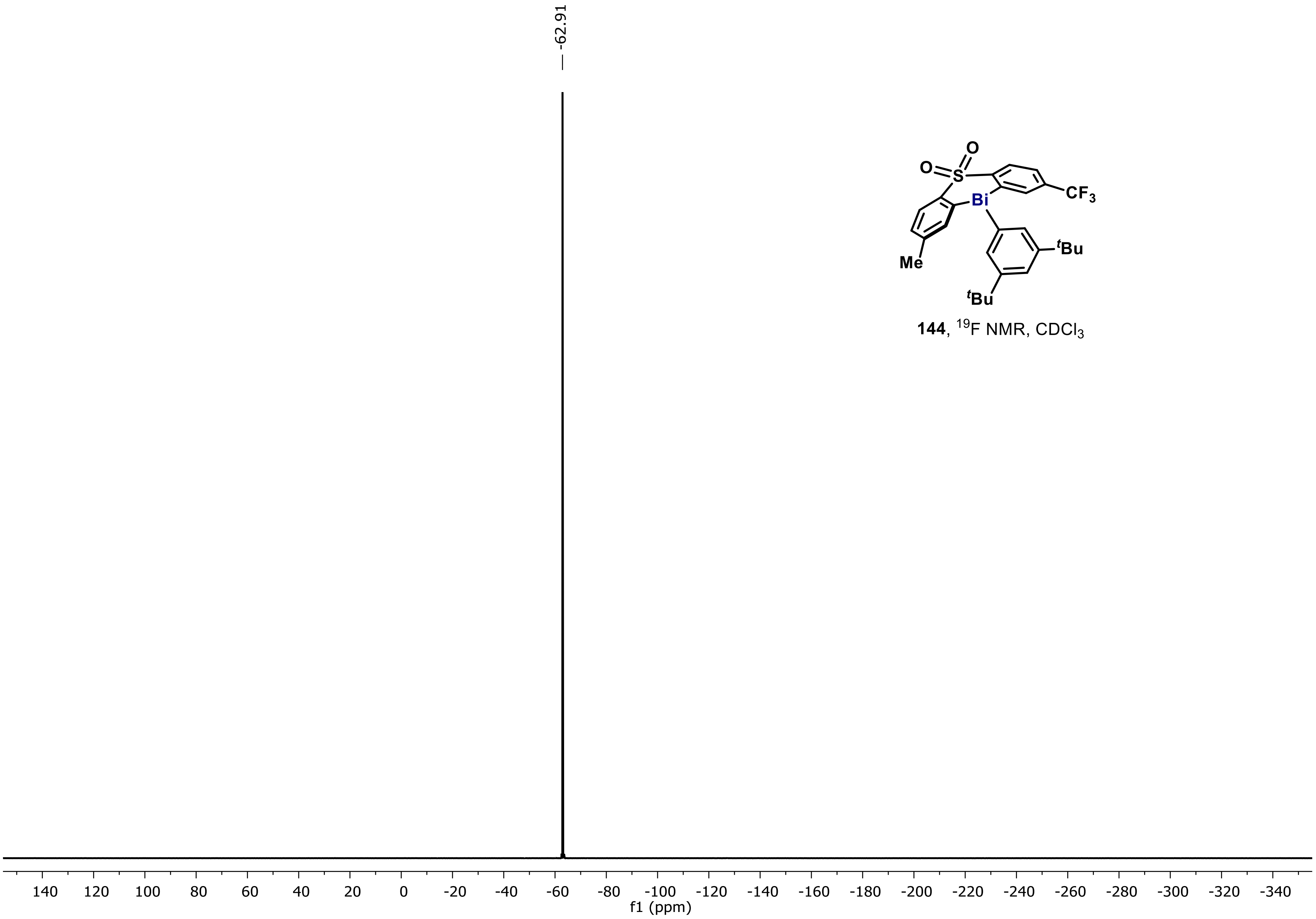
-62.91
O
O
S
Bi
CF3
Me
tBu
tBu
144, 19F NMR, CDCl3
140
120
100
80
60
40
20
0
-20
-40
-60
-80
-100
-120
-140
-160
-180
-200
-220
-240
-260
-280
-300
-320
-340
f1 (ppm)

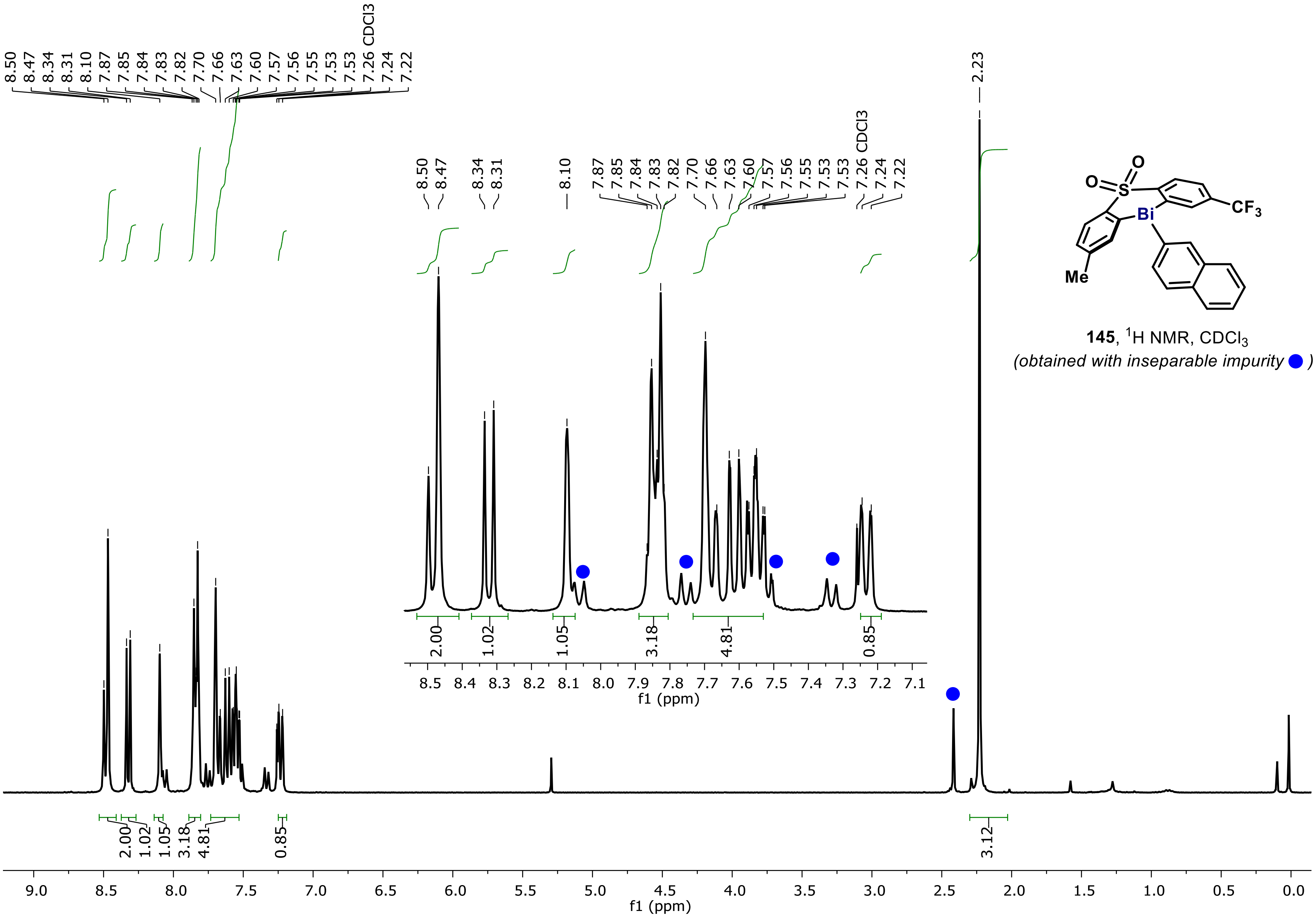

145, ^{1}H NMR, $CDCl_3$

(obtained with inseparable impurity ●)

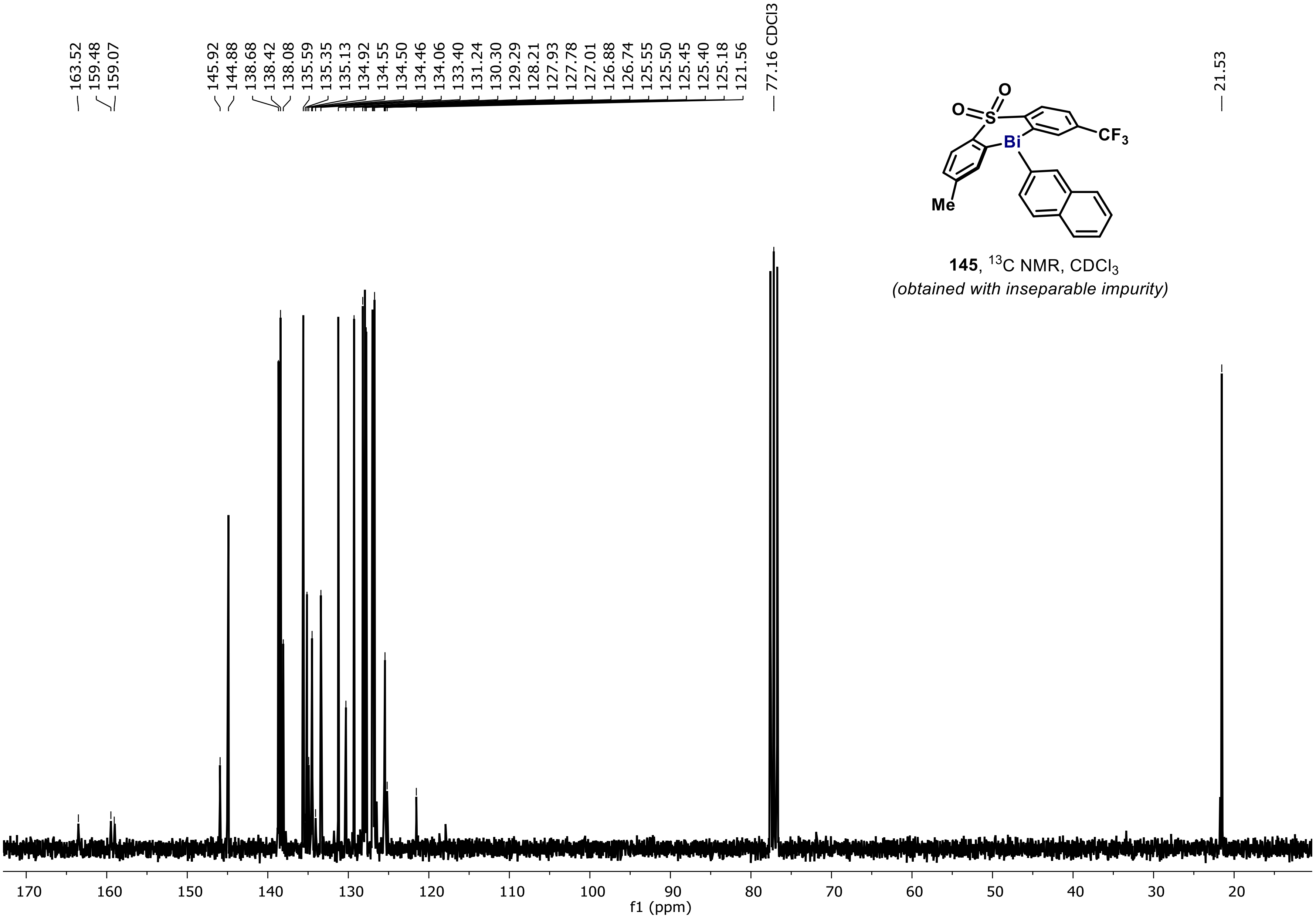

145, ^{13}C NMR, $CDCl_3$

(obtained with inseparable impurity)

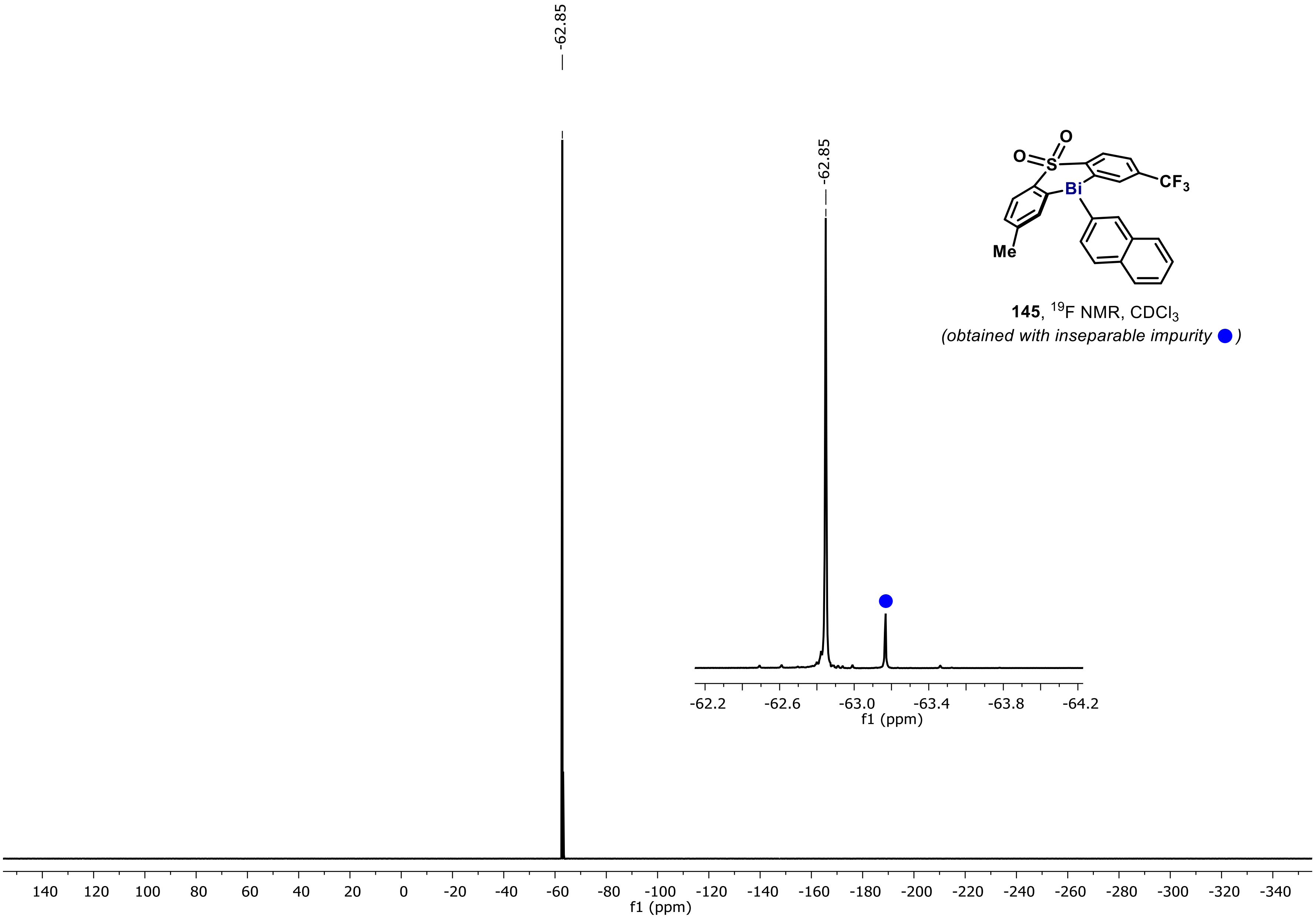

145, ^{19}F NMR, $CDCl_3$
(obtained with inseparable impurity ●)

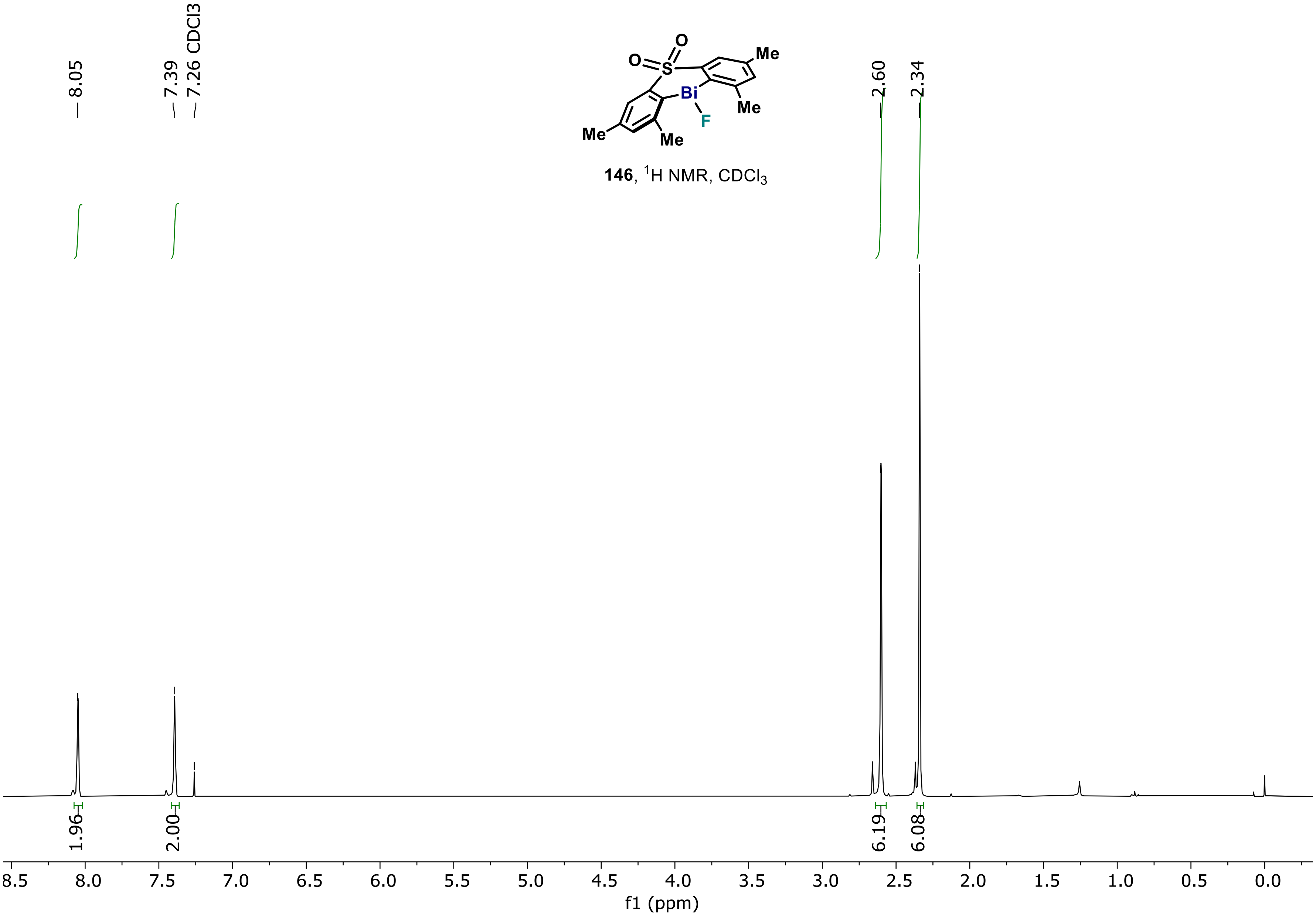
8.05
7.39
7.26 CDCl3
2.60
2.34
Me
O
O
S
Bi
F
Me
Me
Me
146, 1H NMR, CDCl3
1.96
2.00
6.19
6.08
8.5 8.0 7.5 7.0 6.5 6.0 5.5 5.0 4.5 4.0 3.5 3.0 2.5 2.0 1.5 1.0 0.5 0.0
f1 (ppm)

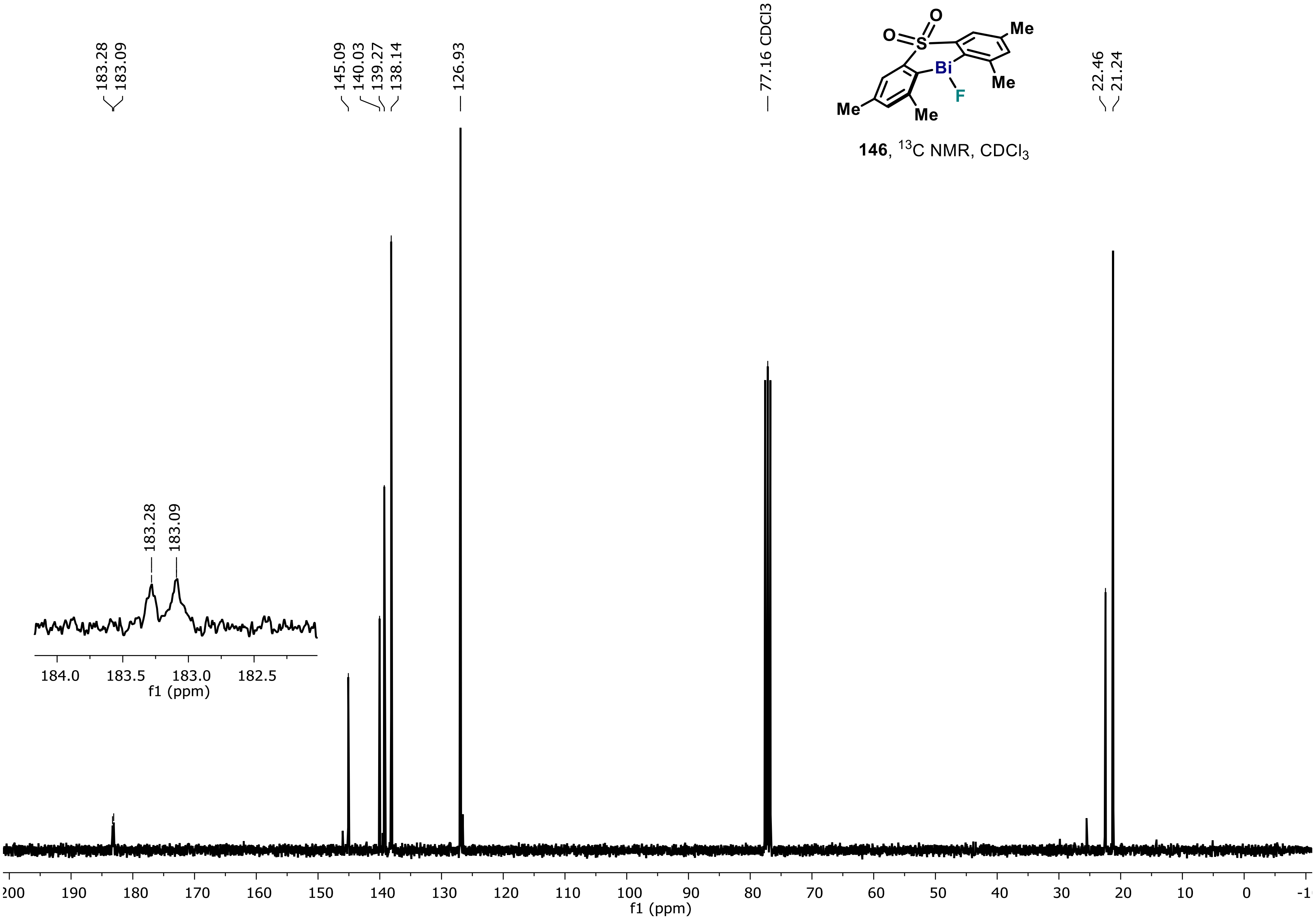

183.28
183.09
145.09
140.03
139.27
138.14
126.93
77.16 CDCl3
22.46
21.24
O
O
S
Me
Bi
F
Me
Me
Me
146, 13C NMR, CDCl3
184.0
183.5
183.0
182.5
f1 (ppm)
200
190
180
170
160
150
140
130
120
110
100
90
80
70
60
50
40
30
20
10
0
-1
f1 (ppm)

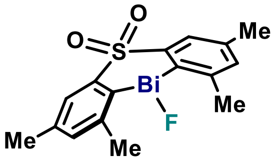

146, ^{19}F NMR, $CDCl_3$

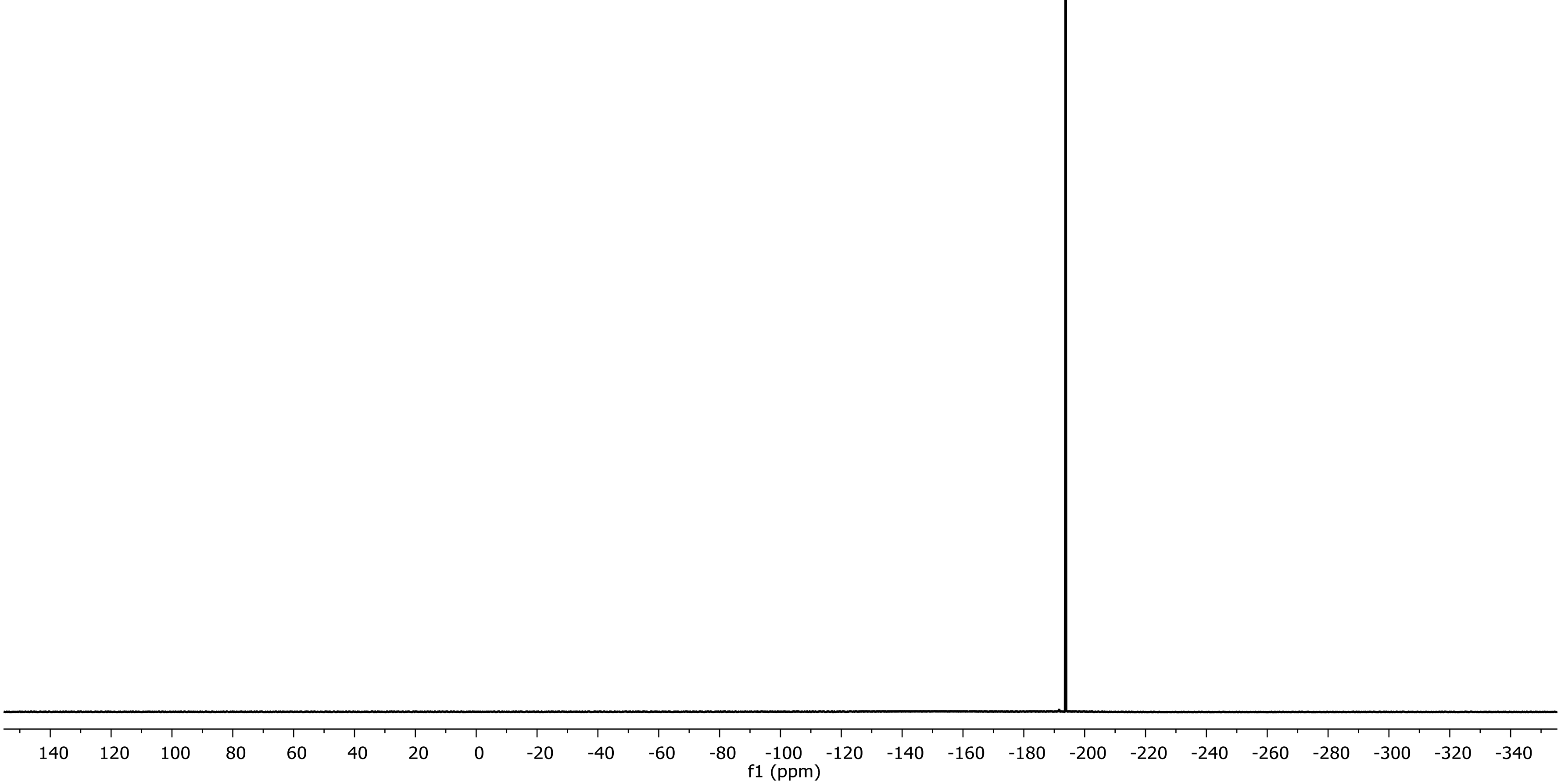

11. NMR spectra of Bi(V) compounds

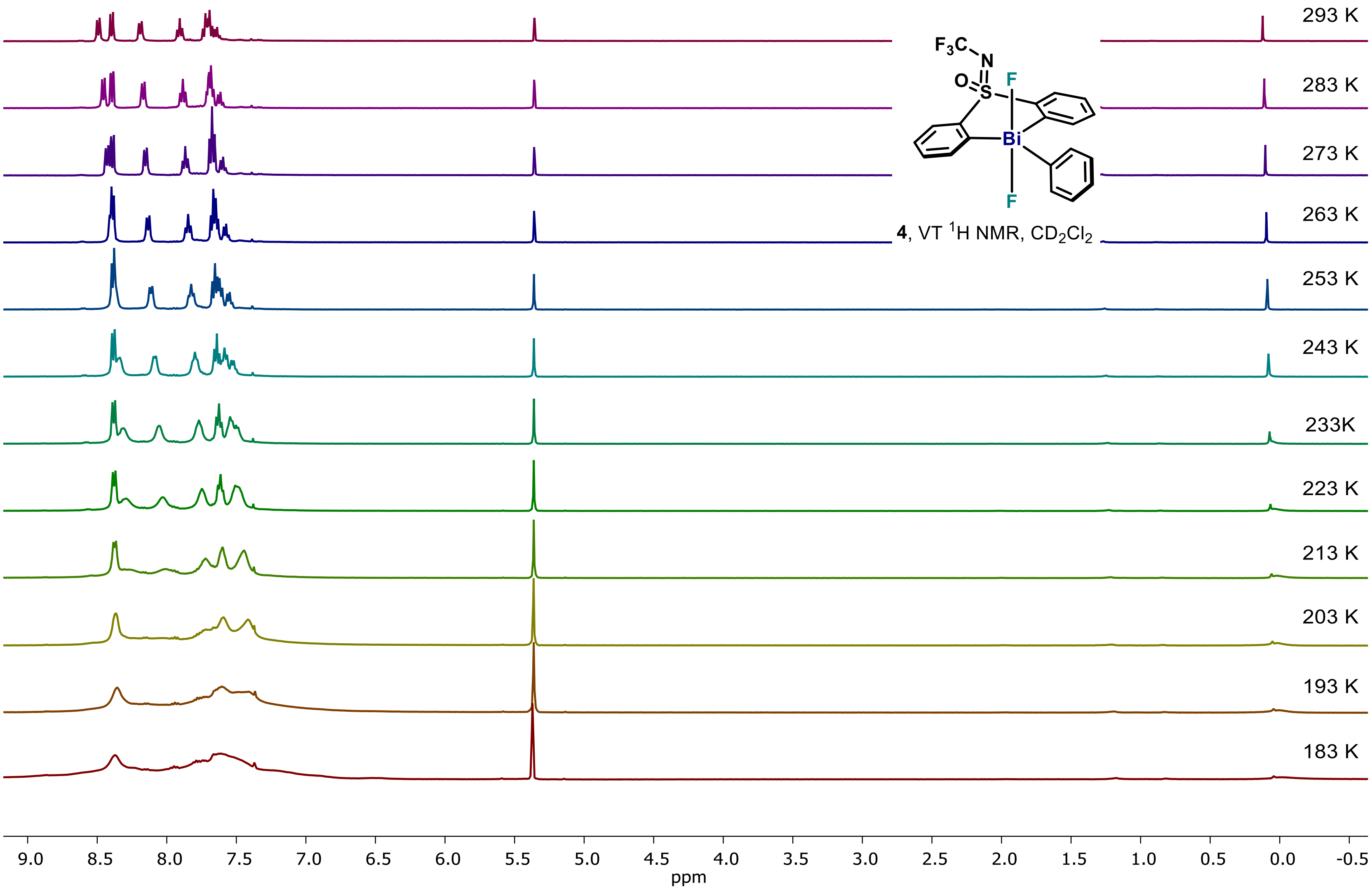

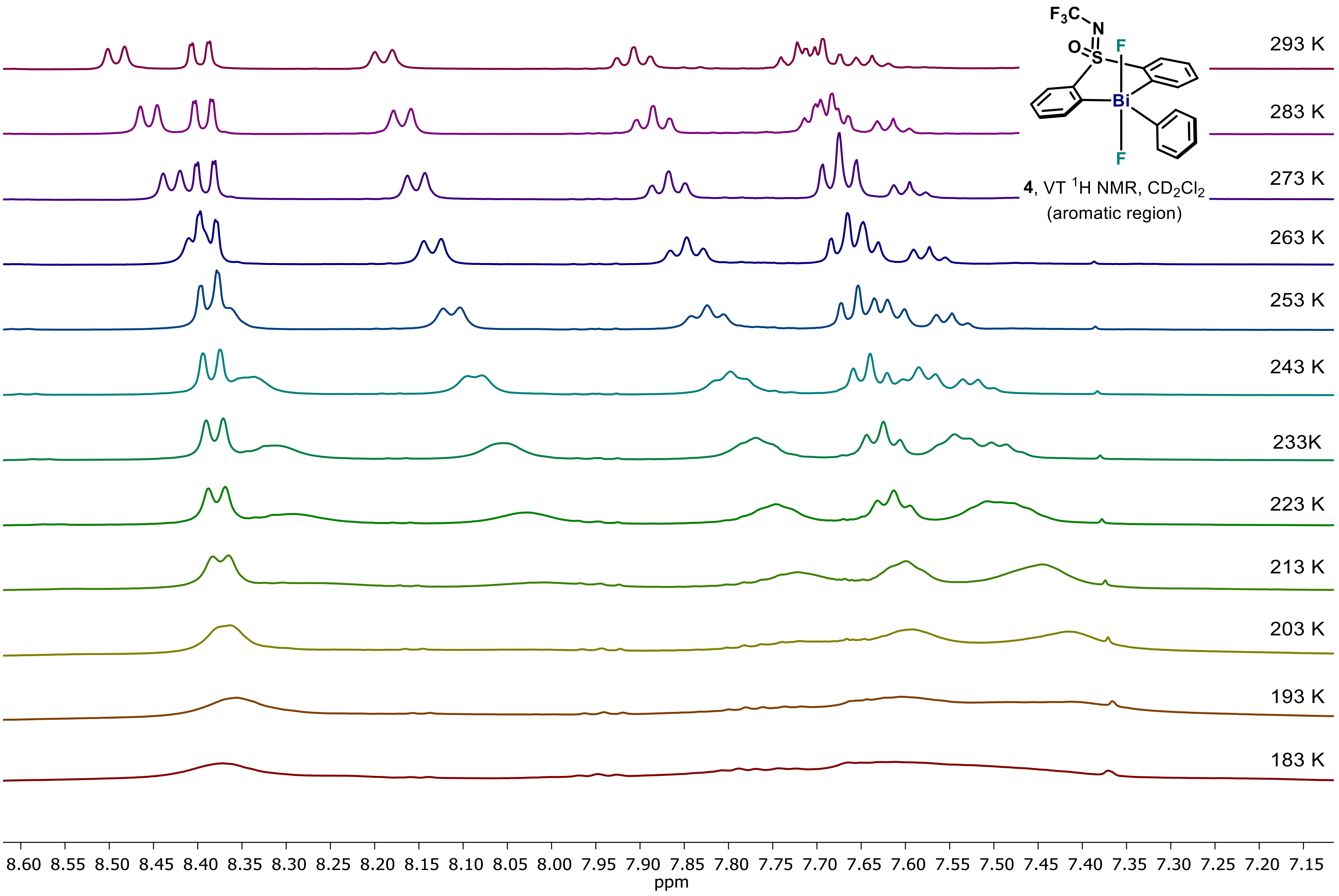
F3C
N
O
S
F
Bi
F
4, VT 1H NMR, CD2Cl2
(aromatic region)
293 K
283 K
273 K
263 K
253 K
243 K
233K
223 K
213 K
203 K
193 K
183 K
8.60 8.55 8.50 8.45 8.40 8.35 8.30 8.25 8.20 8.15 8.10 8.05 8.00 7.95 7.90 7.85 7.80 7.75 7.70 7.65 7.60 7.55 7.50 7.45 7.40 7.35 7.30 7.25 7.20 7.15
ppm

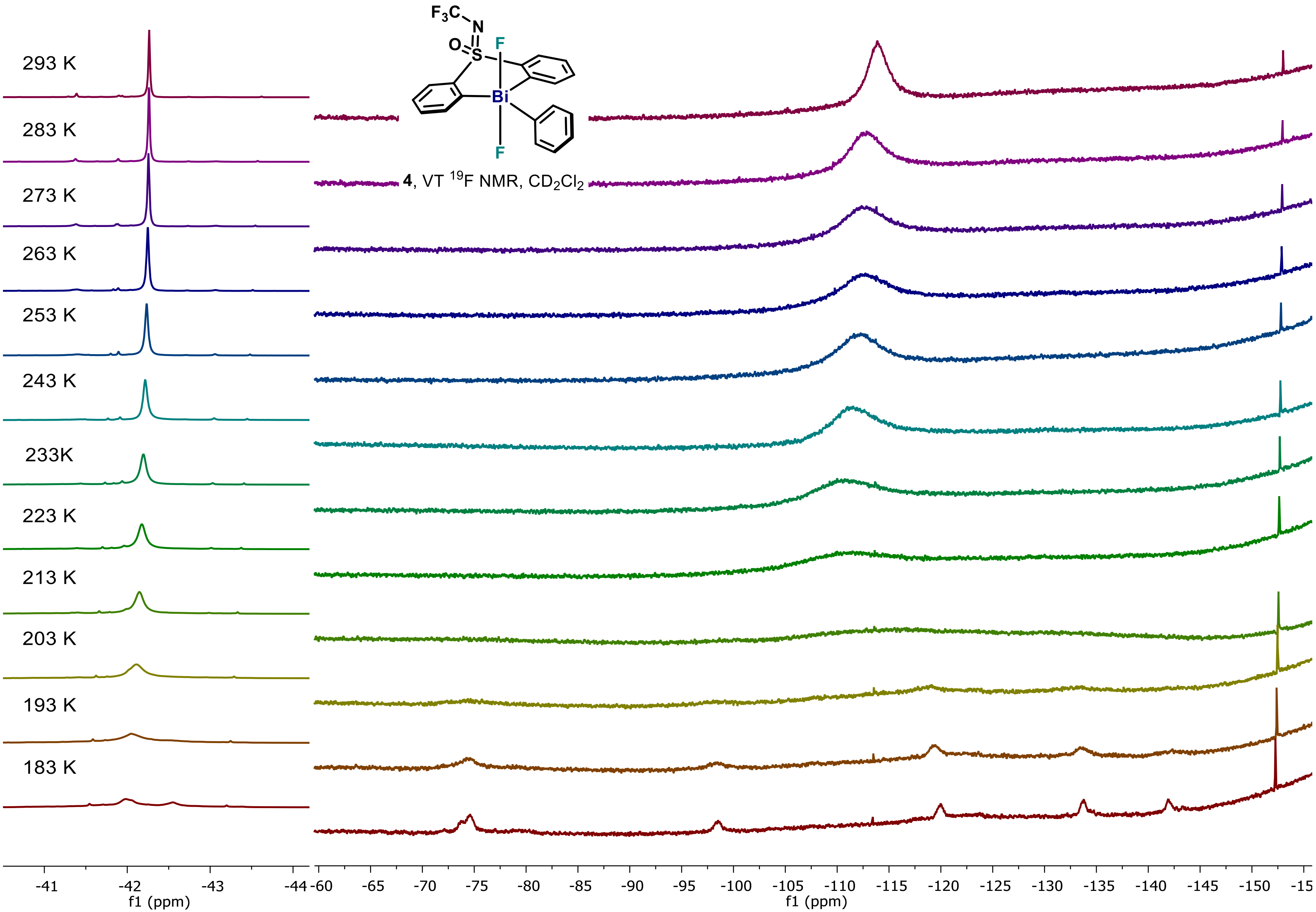

4, VT 19F NMR, CD2Cl2
293 K
283 K
273 K
263 K
253 K
243 K
233K
223 K
213 K
203 K
193 K
183 K
f1 (ppm)
f1 (ppm)

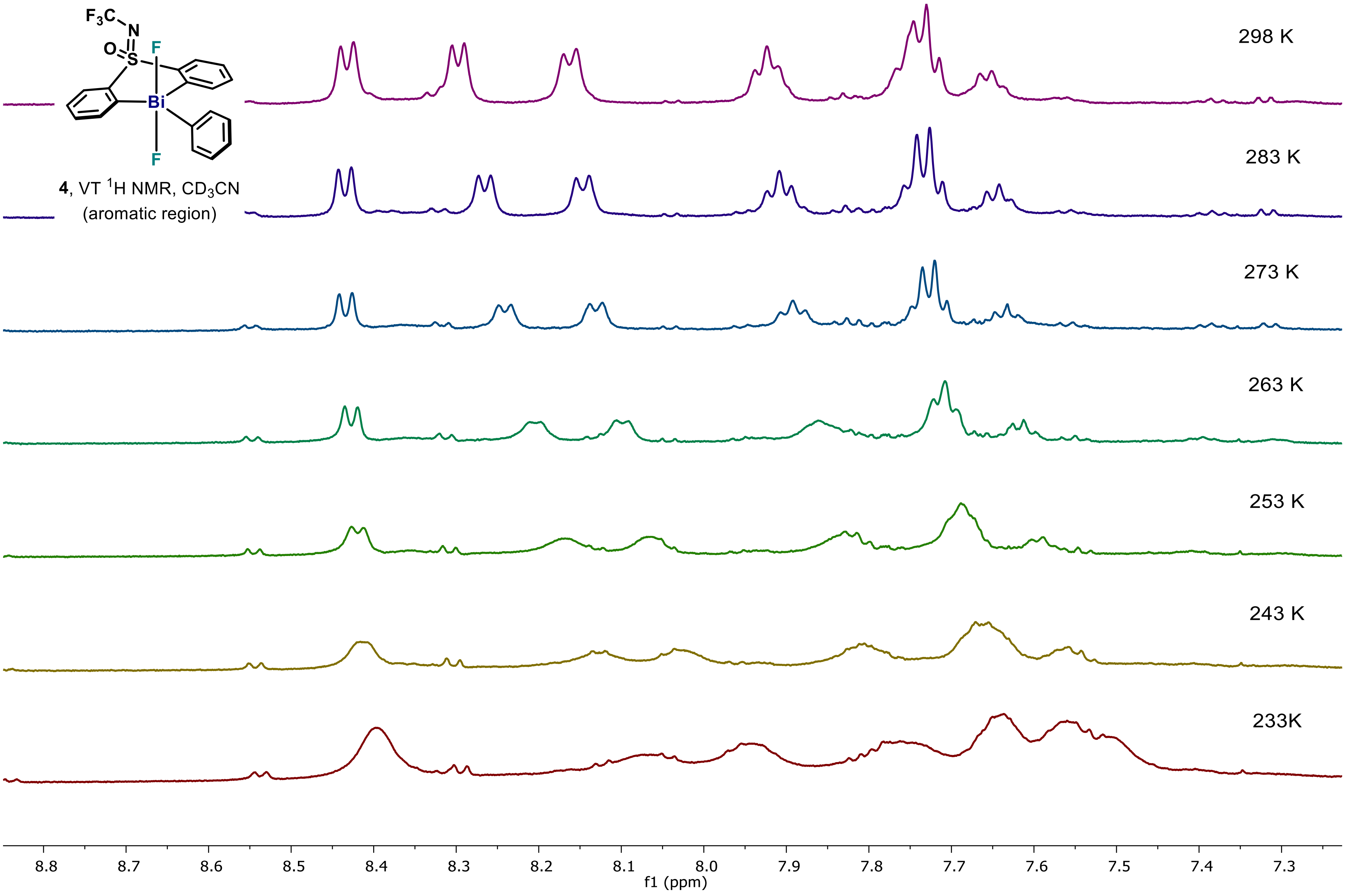

F_3C
N
O
S
F
Bi
F
4, VT ¹H NMR, CD_3CN
(aromatic region)
298 K
283 K
273 K
263 K
253 K
243 K
233K
8.8 8.7 8.6 8.5 8.4 8.3 8.2 8.1 8.0 7.9 7.8 7.7 7.6 7.5 7.4 7.3
f1 (ppm)

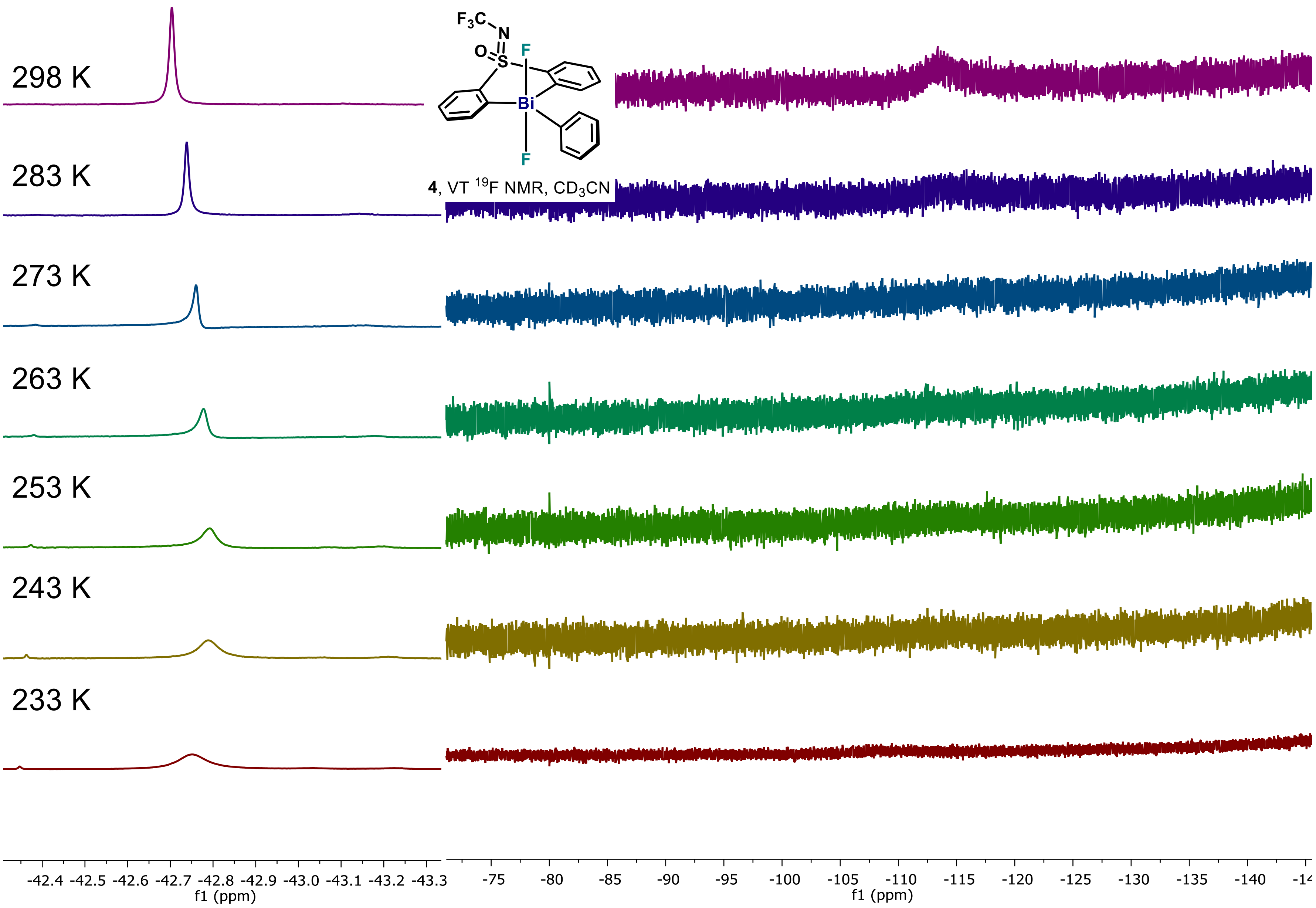
F3C
N
O
S
F
Bi
F
4, VT 19F NMR, CD3CN
298 K
283 K
273 K
263 K
253 K
243 K
233 K
-42.4 -42.5 -42.6 -42.7 -42.8 -42.9 -43.0 -43.1 -43.2 -43.3
f1 (ppm)
-75 -80 -85 -90 -95 -100 -105 -110 -115 -120 -125 -130 -135 -140
f1 (ppm)

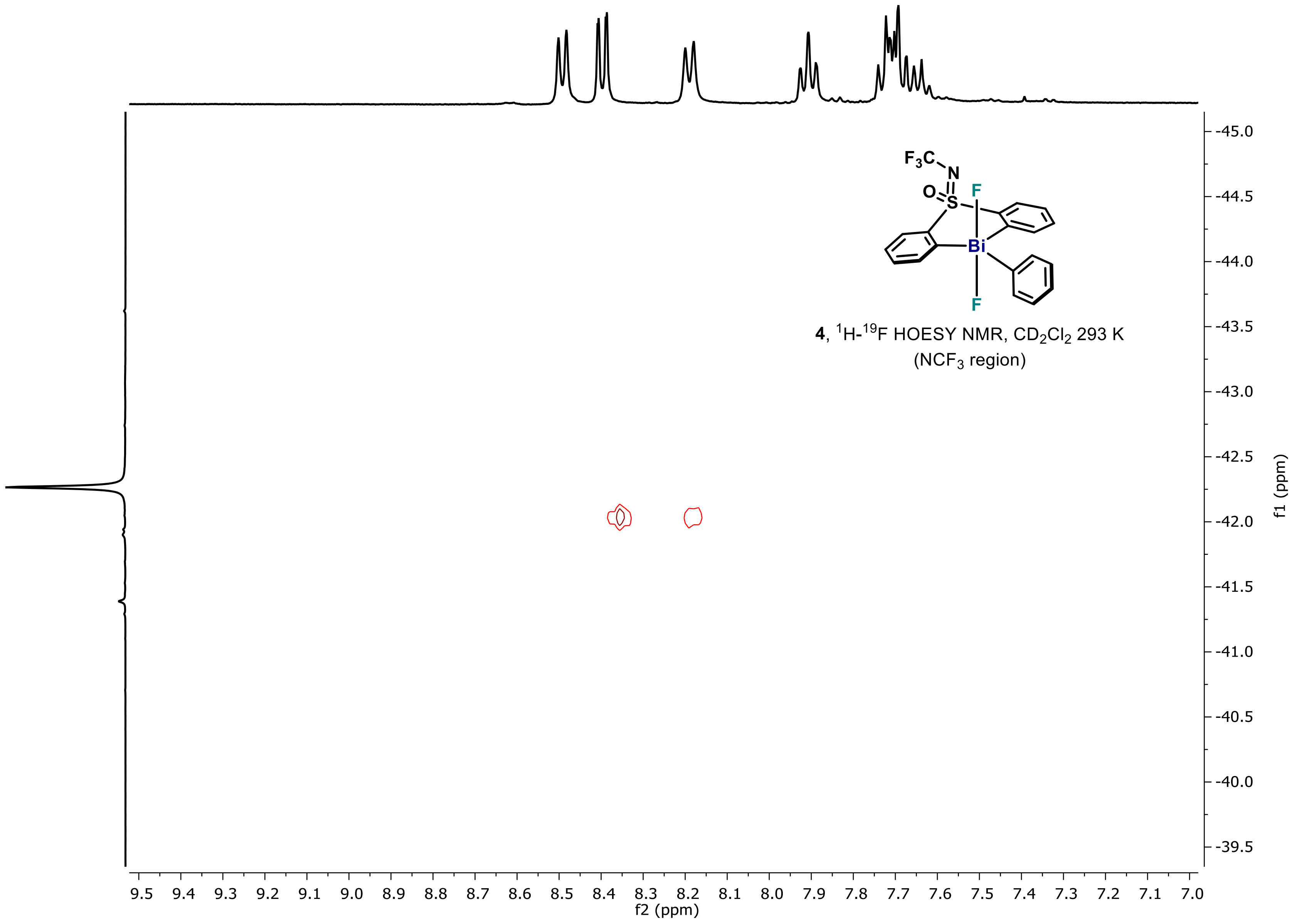

F3C
N
O
S
F
Bi
F
4, 1H-19F HOESY NMR, CD2Cl2 293 K
(NCF3 region)
-45.0
-44.5
-44.0
-43.5
-43.0
-42.5
-42.0
-41.5
-41.0
-40.5
-40.0
-39.5
f1 (ppm)
9.5 9.4 9.3 9.2 9.1 9.0 8.9 8.8 8.7 8.6 8.5 8.4 8.3 8.2 8.1 8.0 7.9 7.8 7.7 7.6 7.5 7.4 7.3 7.2 7.1 7.0
f2 (ppm)

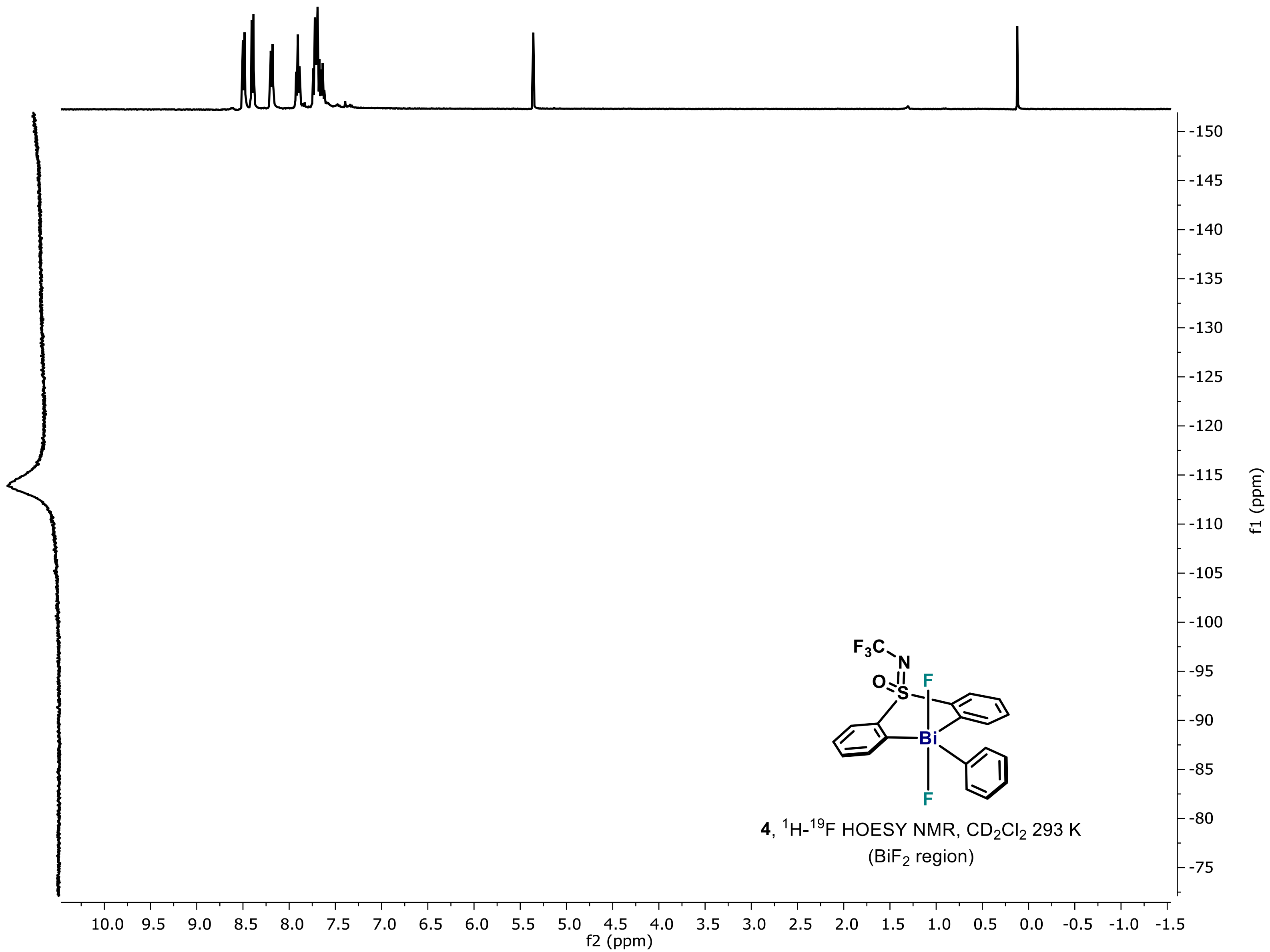
F3C
N
O
S
F
Bi
F
4, 1H-19F HOESY NMR, CD2Cl2 293 K
(BiF2 region)
f1 (ppm)
f2 (ppm)
-150
-145
-140
-135
-130
-125
-120
-115
-110
-105
-100
-95
-90
-85
-80
-75
10.0
9.5
9.0
8.5
8.0
7.5
7.0
6.5
6.0
5.5
5.0
4.5
4.0
3.5
3.0
2.5
2.0
1.5
1.0
0.5
0.0
-0.5
-1.0
-1.5

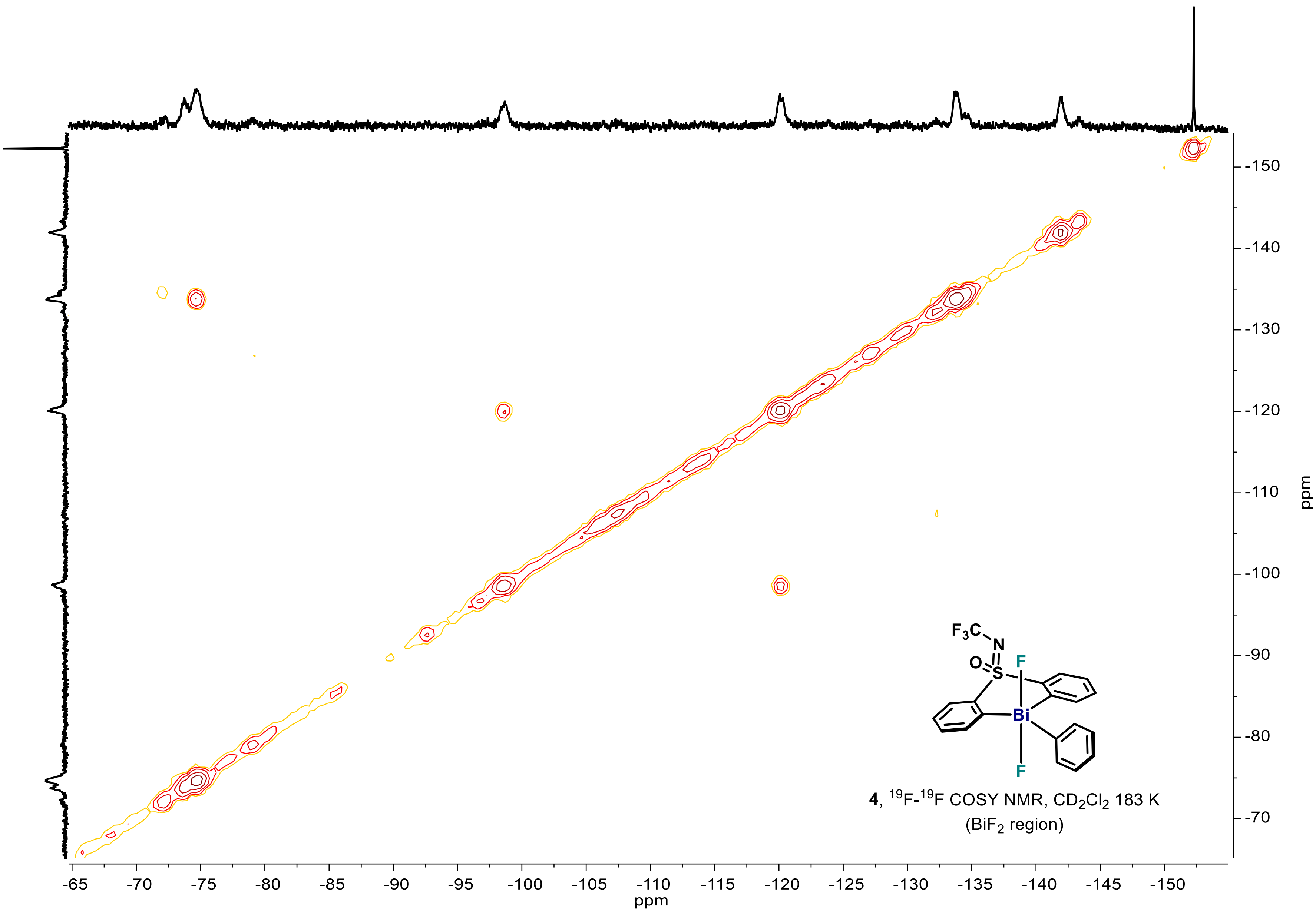

ppm
-150
-140
-130
-120
-110
-100
-90
-80
-70
-65
-75
-85
-95
-105
-115
-125
-135
-145
F3C
N
O
S
F
Bi
F
4, 19F-19F COSY NMR, CD2Cl2 183 K
(BiF2 region)

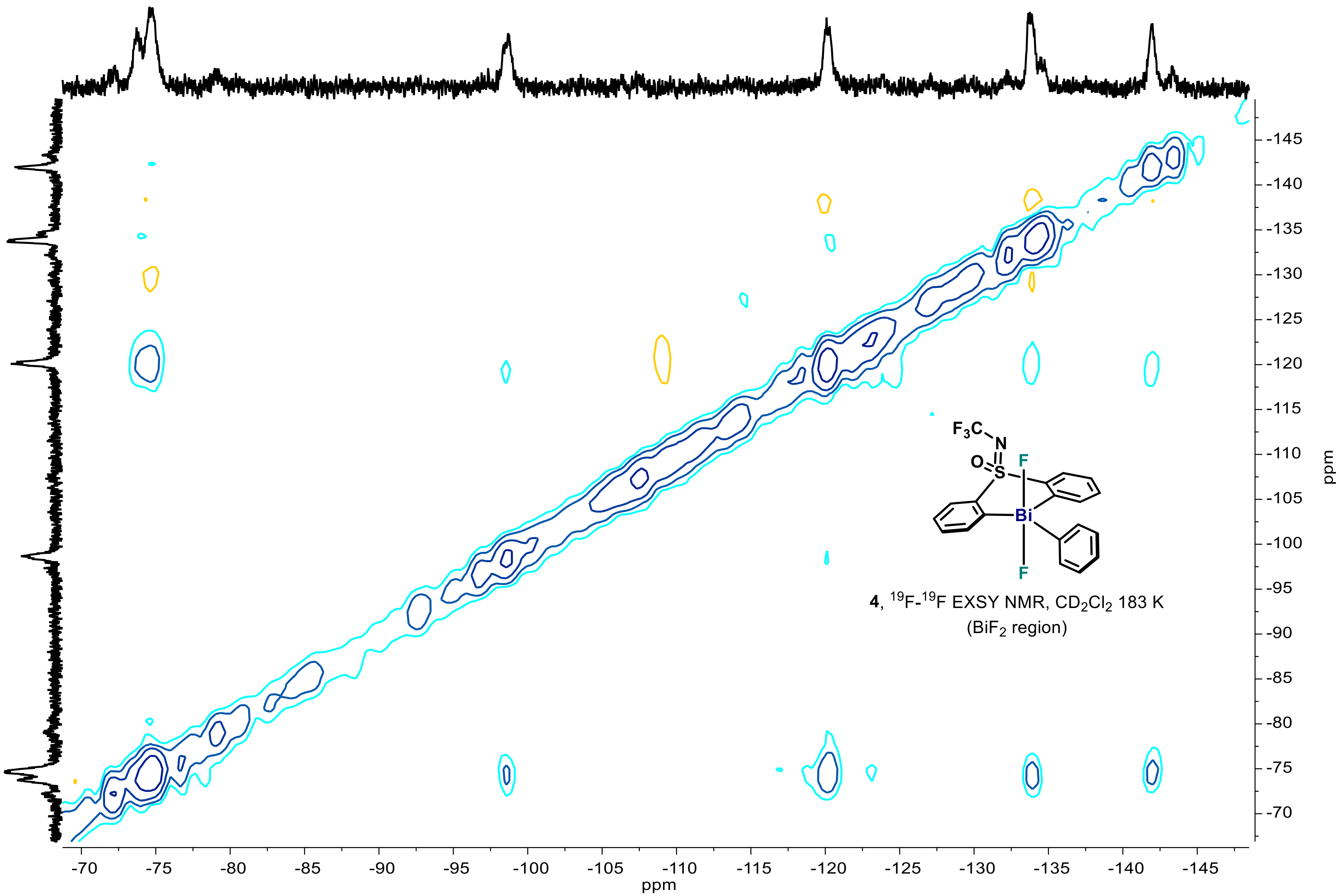

F3C
N
O
S
F
Bi
F
4, 19F-19F EXSY NMR, CD2Cl2 183 K
(BiF2 region)
ppm
ppm

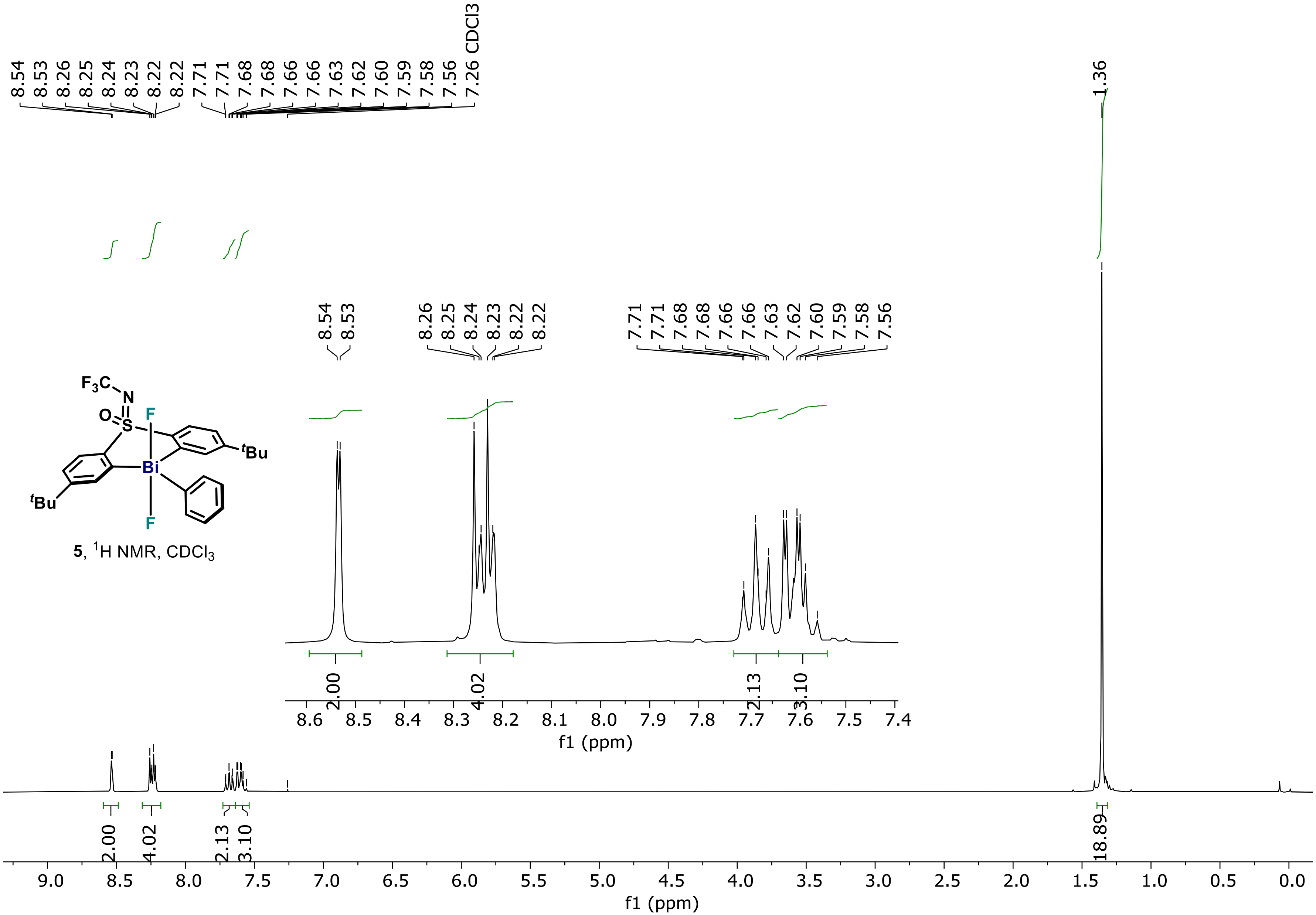
8.54 8.53 8.26 8.25 8.24 8.23 8.22 8.22 7.71 7.71 7.68 7.68 7.66 7.66 7.63 7.62 7.60 7.59 7.58 7.56 7.26 CDCl3
1.36
F3C N O S F Bi tBu tBu F
5, 1H NMR, CDCl3
2.00 4.02 2.13 3.10
8.6 8.5 8.4 8.3 8.2 8.1 8.0 7.9 7.8 7.7 7.6 7.5 7.4
f1 (ppm)
2.00 4.02 2.13 3.10 18.89
9.0 8.5 8.0 7.5 7.0 6.5 6.0 5.5 5.0 4.5 4.0 3.5 3.0 2.5 2.0 1.5 1.0 0.5 0.0
f1 (ppm)

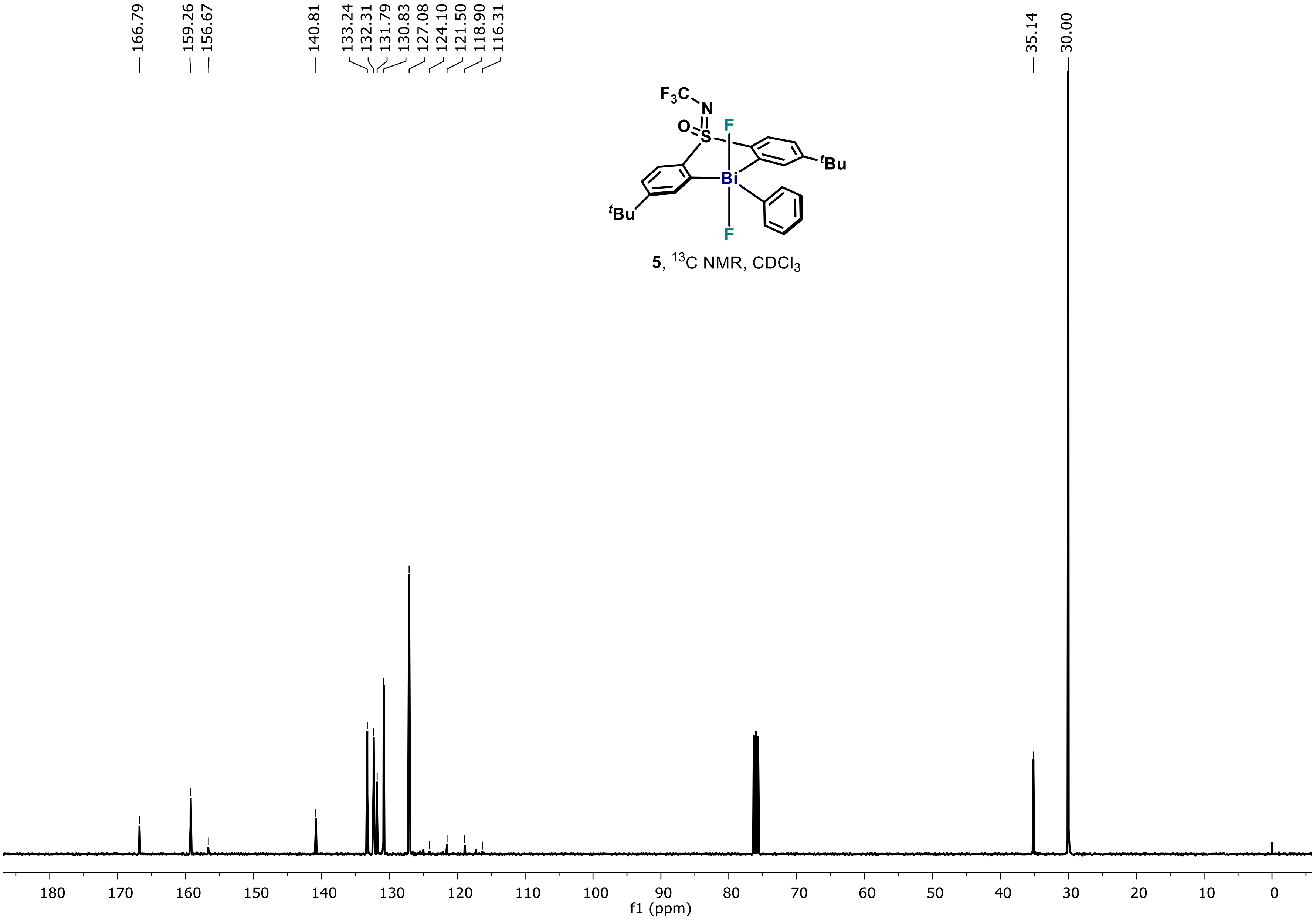
166.79
159.26
156.67
140.81
133.24
132.31
131.79
130.83
127.08
124.10
121.50
118.90
116.31
35.14
30.00
F3C
N
O
S
F
Bi
F
tBu
tBu
5, 13C NMR, CDCl3
180
170
160
150
140
130
120
110
100
90
80
70
60
50
40
30
20
10
0
f1 (ppm)

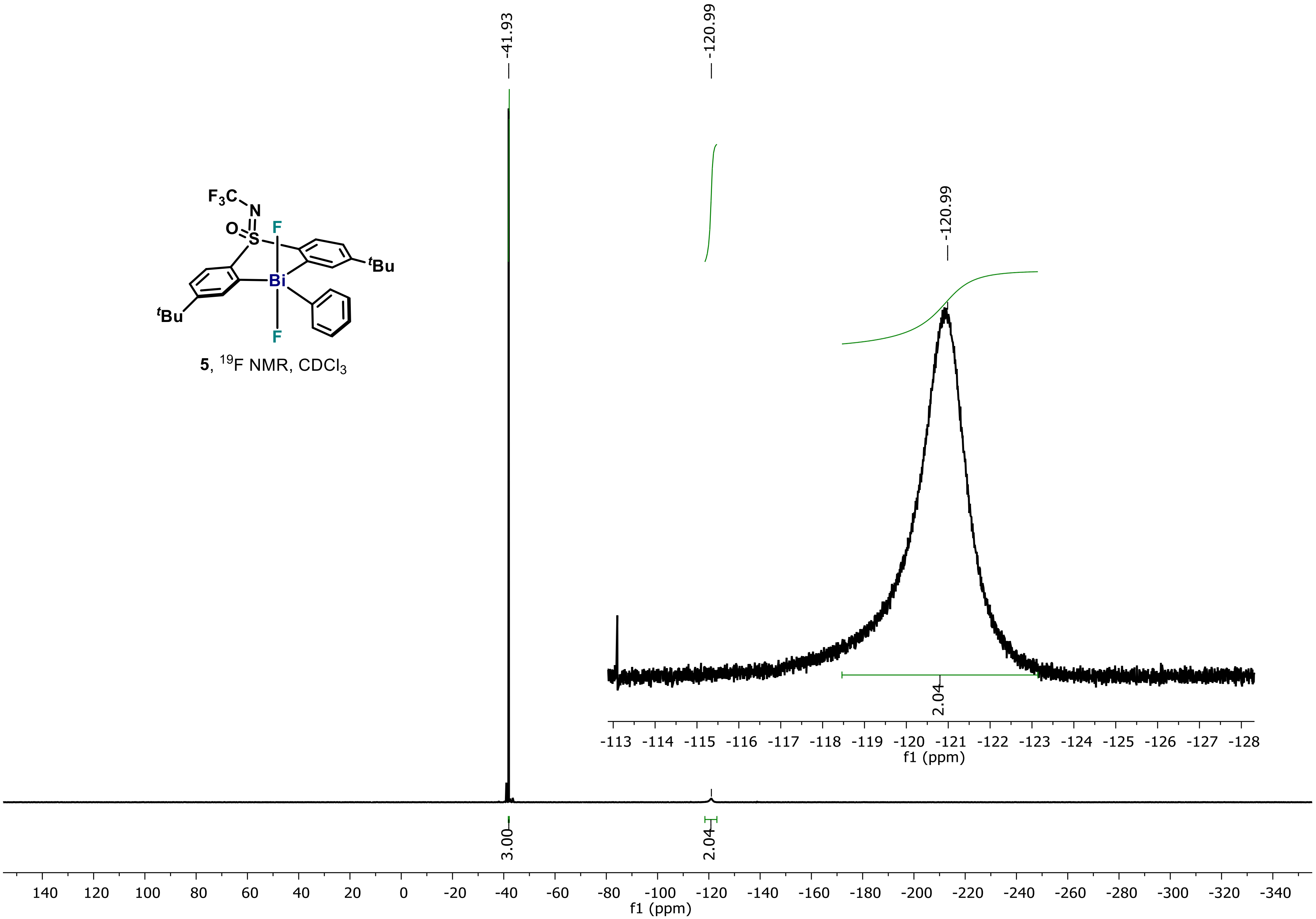

F_3C
N
O
S
F
Bi
F
^tBu
^tBu
5, ^{19}F NMR, $CDCl_3$
-41.93
-120.99
-120.99
2.04
-113 -114 -115 -116 -117 -118 -119 -120 -121 -122 -123 -124 -125 -126 -127 -128
f1 (ppm)
3.00
2.04
140 120 100 80 60 40 20 0 -20 -40 -60 -80 -100 -120 -140 -160 -180 -200 -220 -240 -260 -280 -300 -320 -340
f1 (ppm)

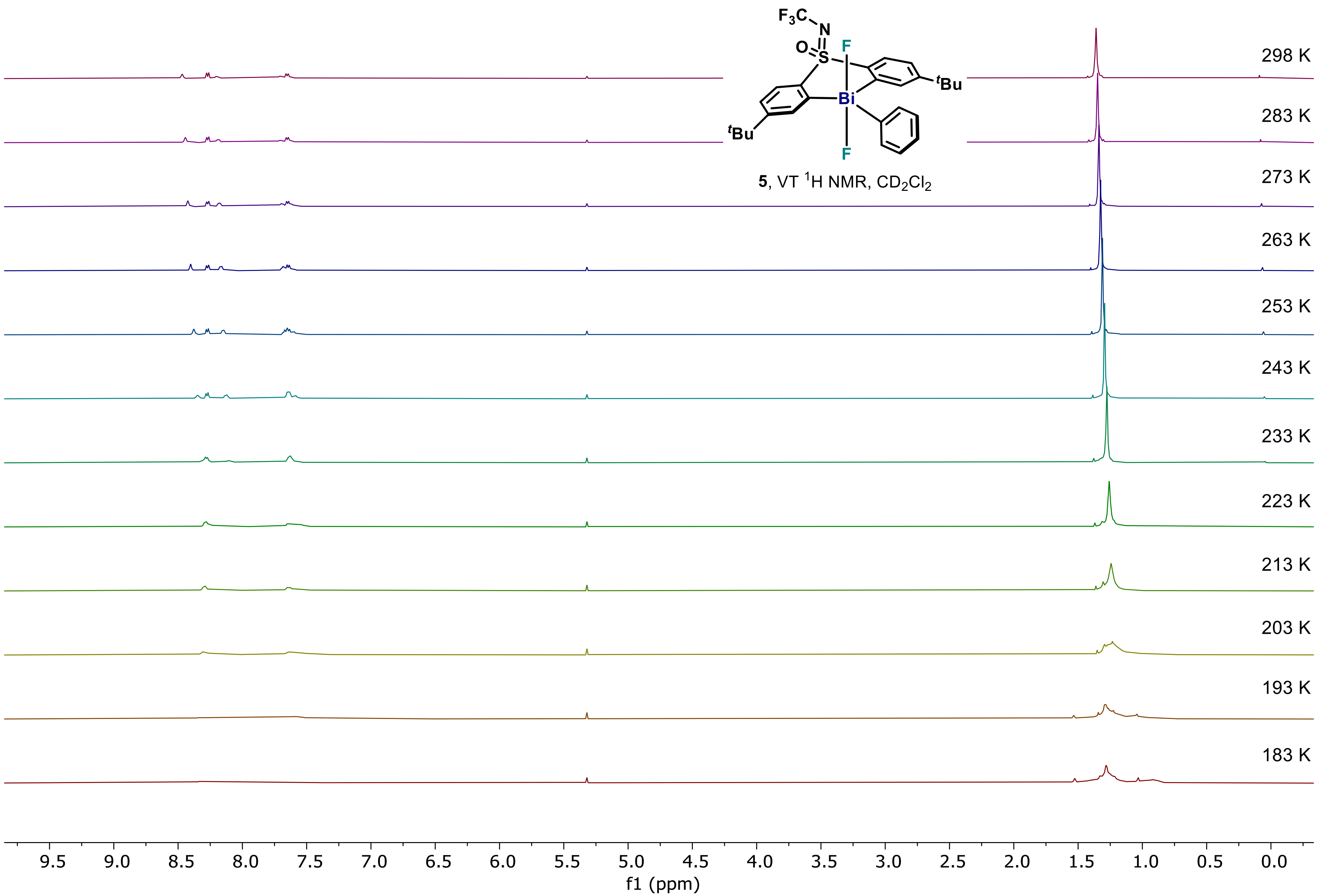
F3C
N
O
S
F
Bi
F
tBu
tBu
5, VT 1H NMR, CD2Cl2
298 K
283 K
273 K
263 K
253 K
243 K
233 K
223 K
213 K
203 K
193 K
183 K
9.5
9.0
8.5
8.0
7.5
7.0
6.5
6.0
5.5
5.0
4.5
4.0
3.5
3.0
2.5
2.0
1.5
1.0
0.5
0.0
f1 (ppm)

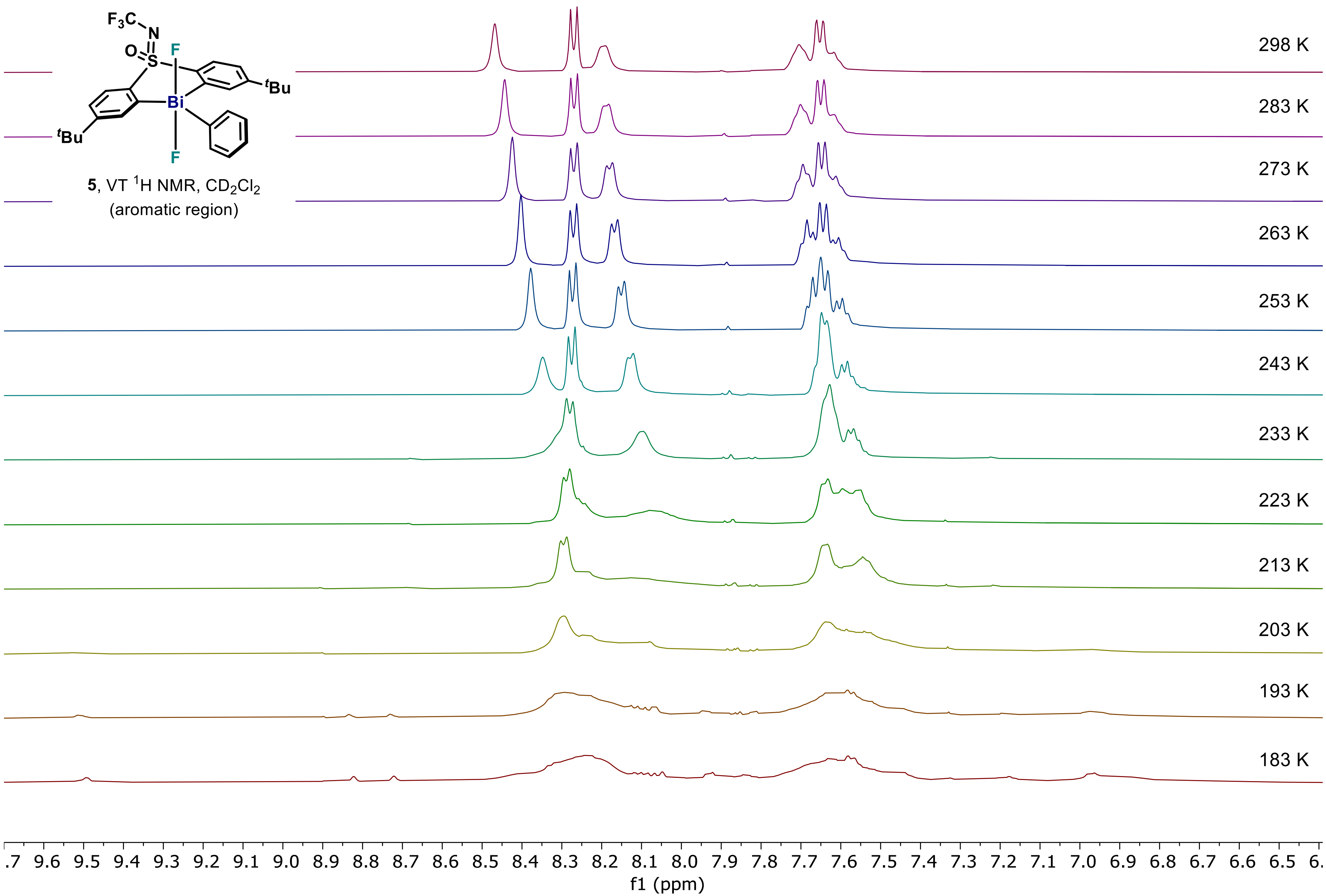

5, VT ^{1}H NMR, CD_2Cl_2
(aromatic region)
298 K
283 K
273 K
263 K
253 K
243 K
233 K
223 K
213 K
203 K
193 K
183 K
f1 (ppm)

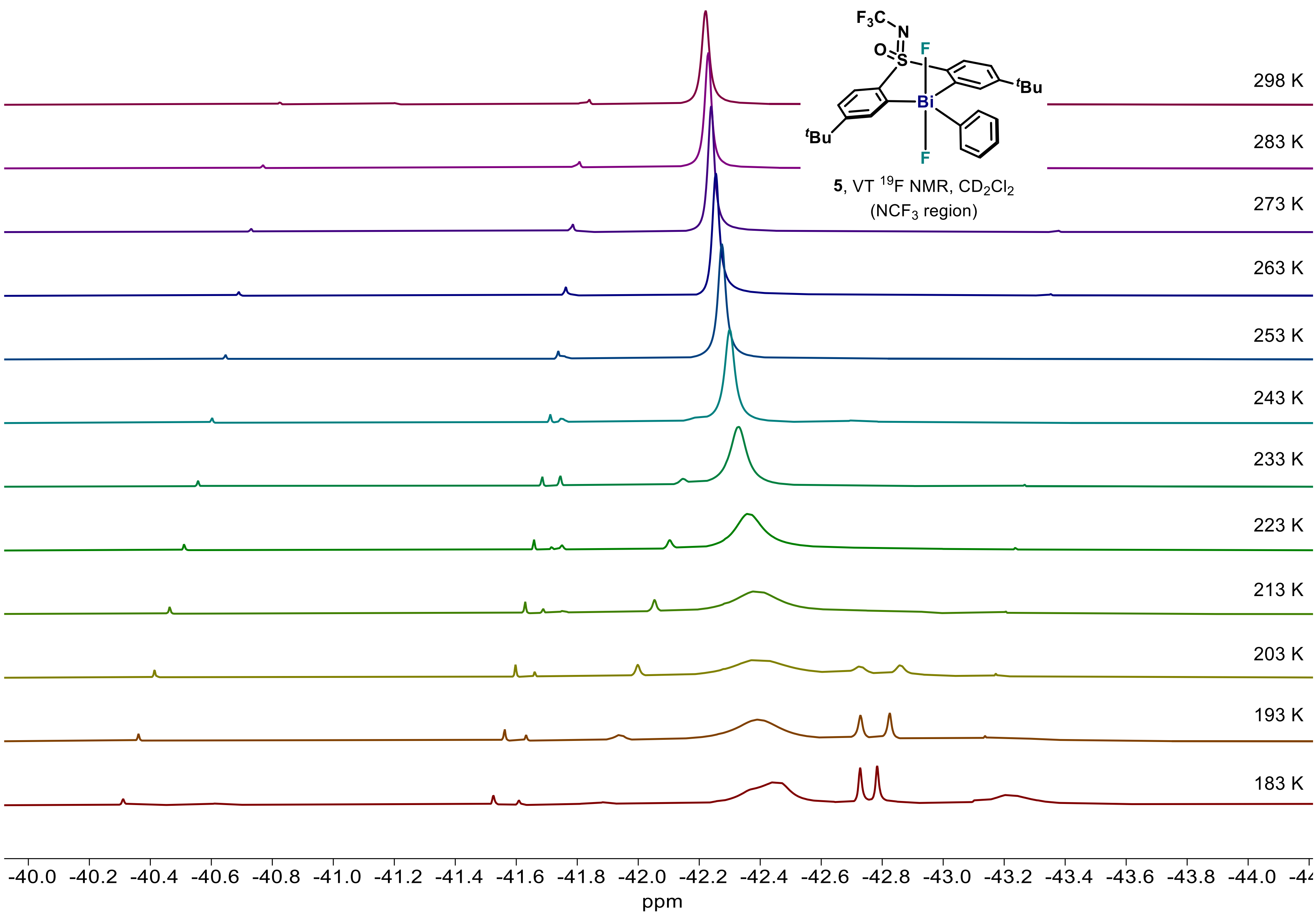

F_3C
N
O
S
F
Bi
F
^tBu
^tBu
5, VT ^{19}F NMR, CD_2Cl_2
(NCF_3 region)
298 K
283 K
273 K
263 K
253 K
243 K
233 K
223 K
213 K
203 K
193 K
183 K
-40.0 -40.2 -40.4 -40.6 -40.8 -41.0 -41.2 -41.4 -41.6 -41.8 -42.0 -42.2 -42.4 -42.6 -42.8 -43.0 -43.2 -43.4 -43.6 -43.8 -44.0
ppm

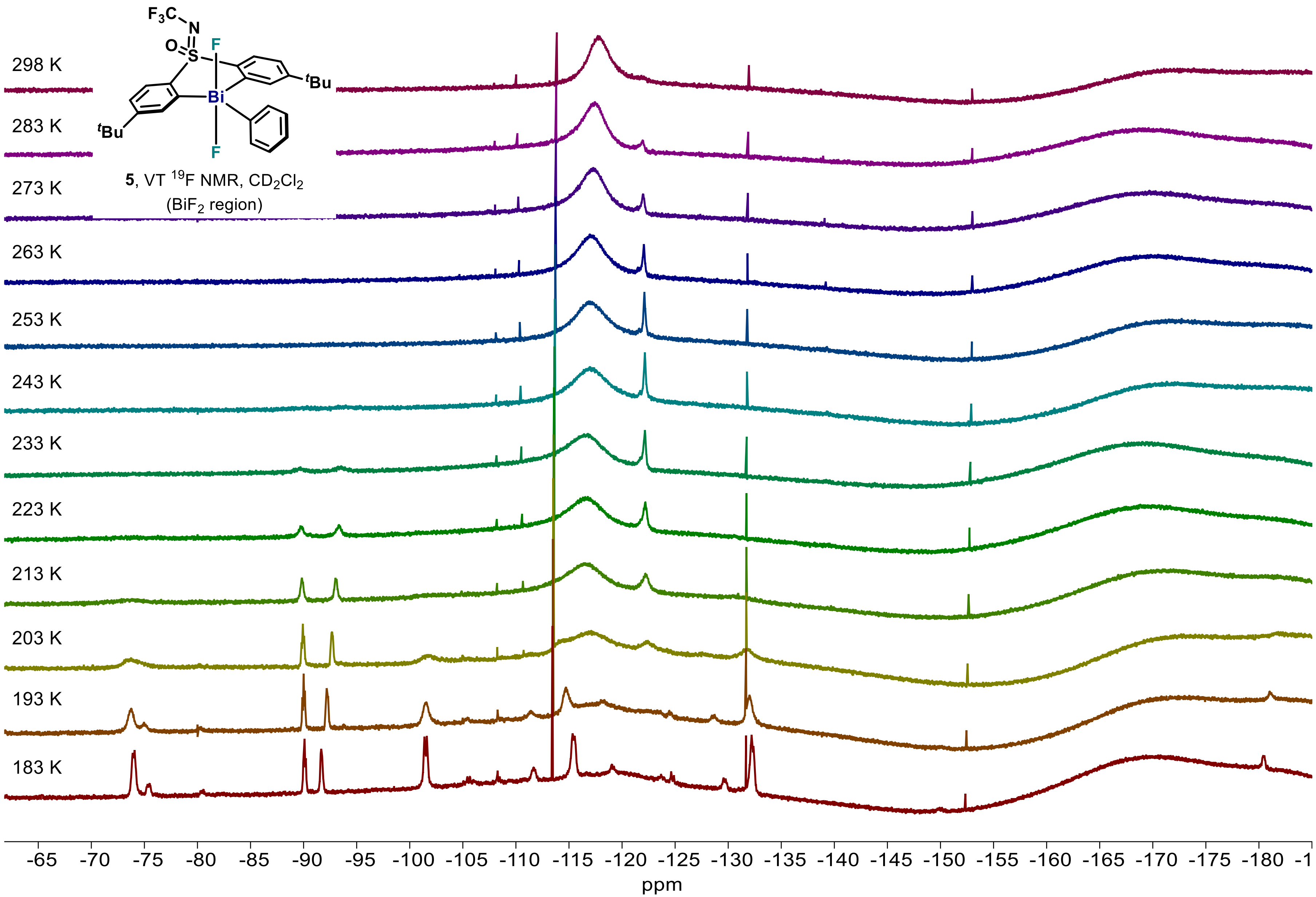

298 K
283 K
273 K
263 K
253 K
243 K
233 K
223 K
213 K
203 K
193 K
183 K
F3C
N
O
S
F
Bi
F
tBu
tBu
5, VT 19F NMR, CD2Cl2
(BiF2 region)
-65 -70 -75 -80 -85 -90 -95 -100 -105 -110 -115 -120 -125 -130 -135 -140 -145 -150 -155 -160 -165 -170 -175 -180 -1
ppm

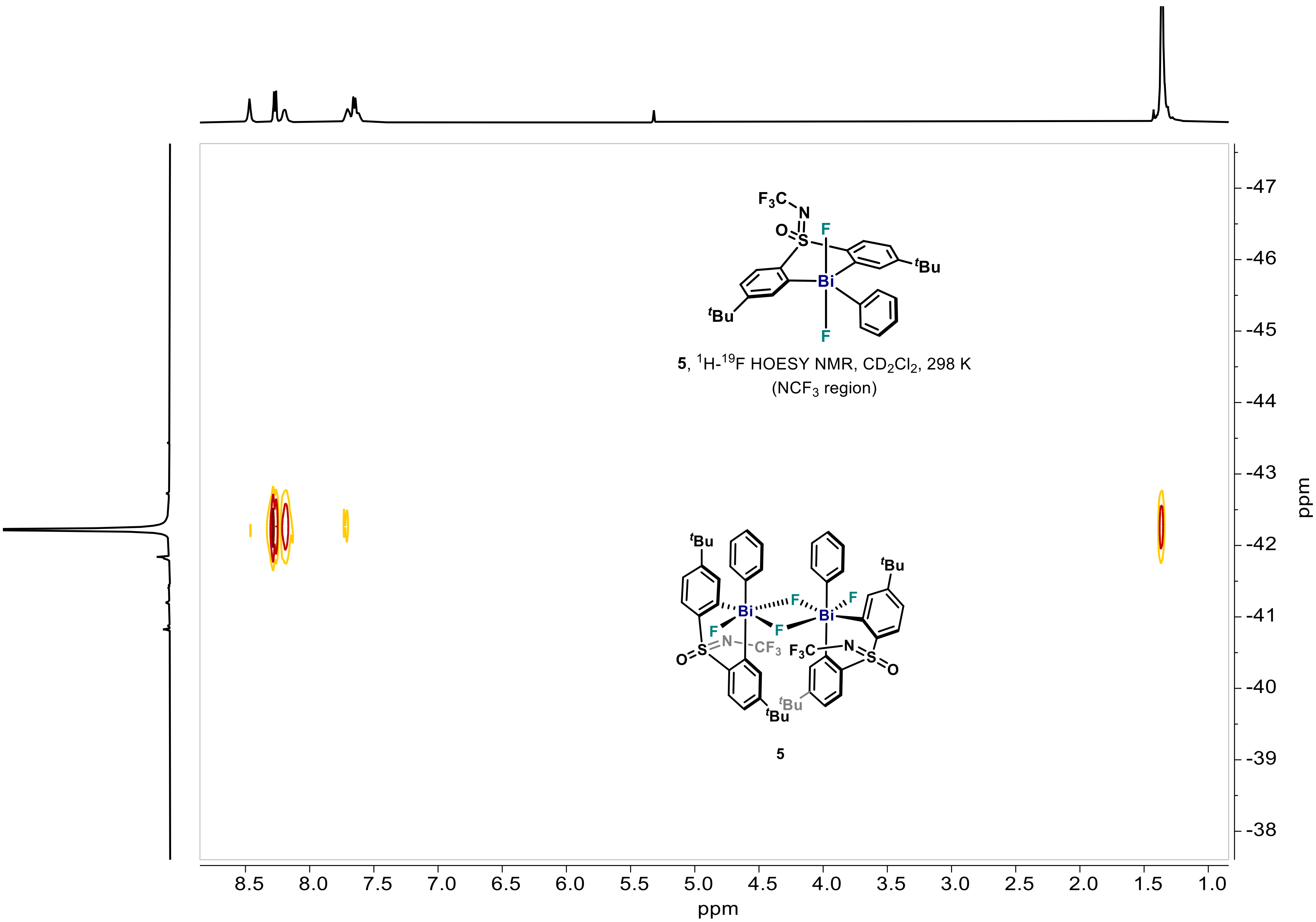
5, ^{1}H-^{19}F HOESY NMR, CD_2Cl_2, 298 K
(NCF_3 region)
5
ppm
ppm

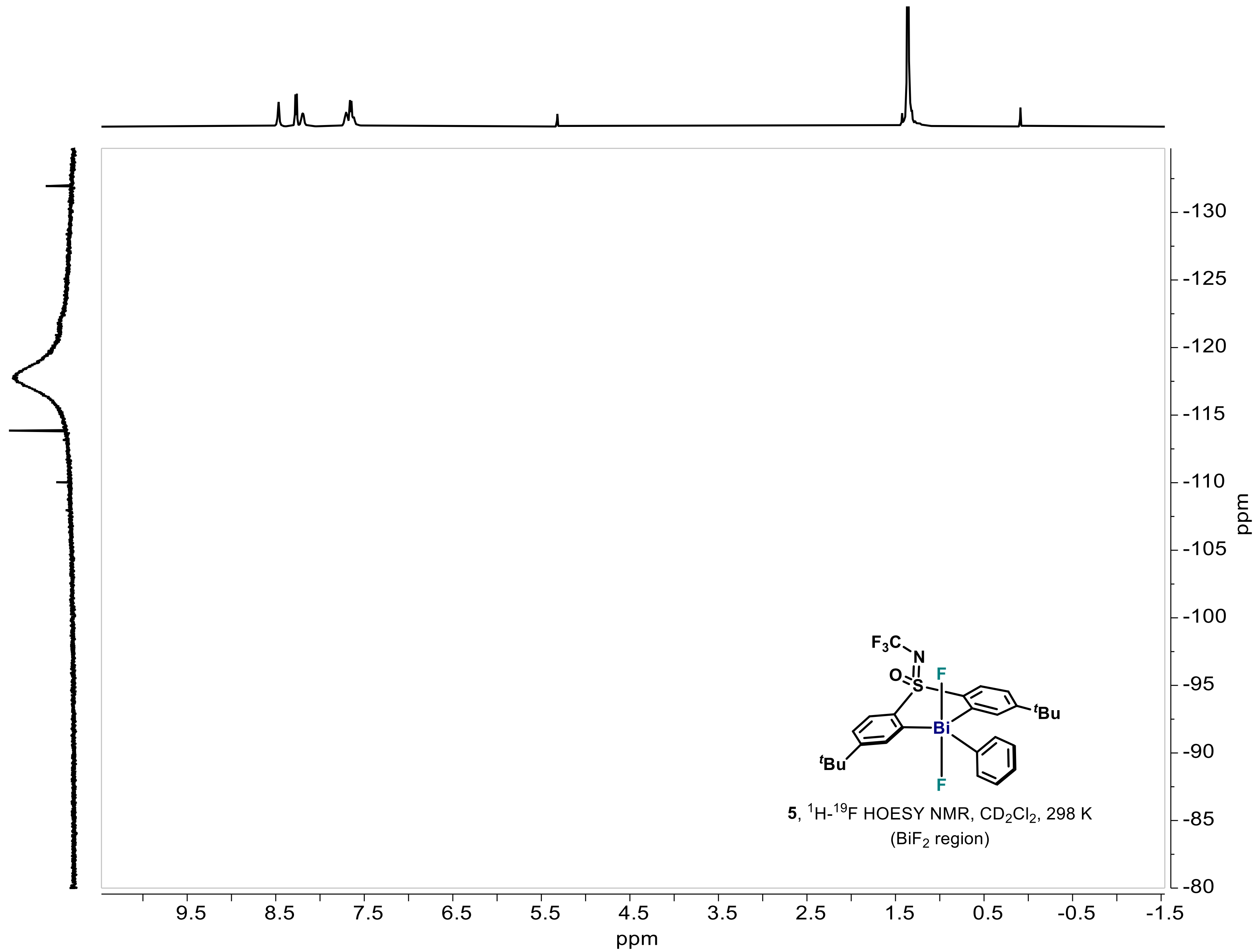

F3C
N
O
S
F
Bi
F
tBu
tBu
5, 1H-19F HOESY NMR, CD2Cl2, 298 K
(BiF2 region)
ppm
ppm

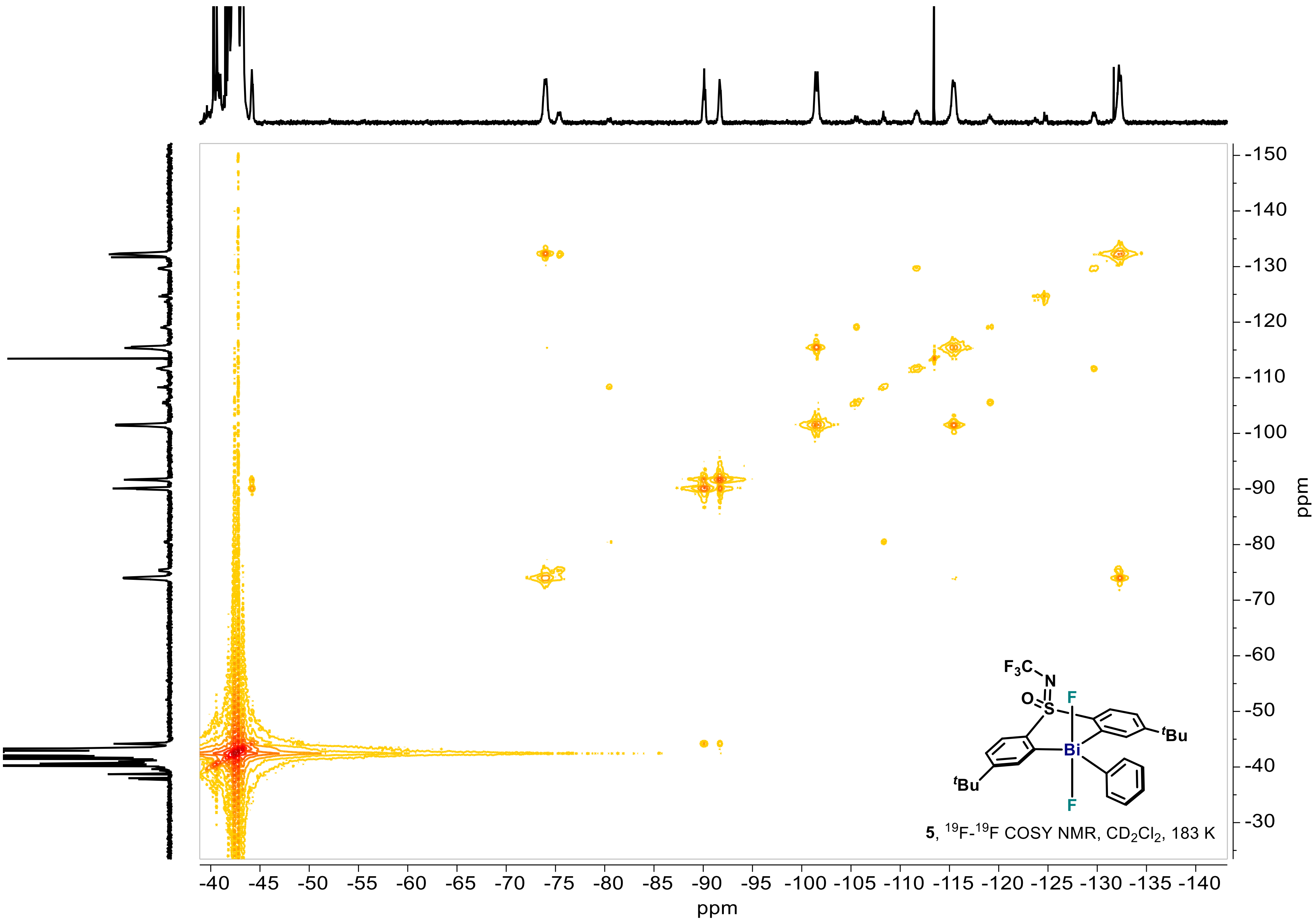

F3C
N
O
S
F
Bi
F
tBu
tBu
5, 19F-19F COSY NMR, CD2Cl2, 183 K
ppm
ppm

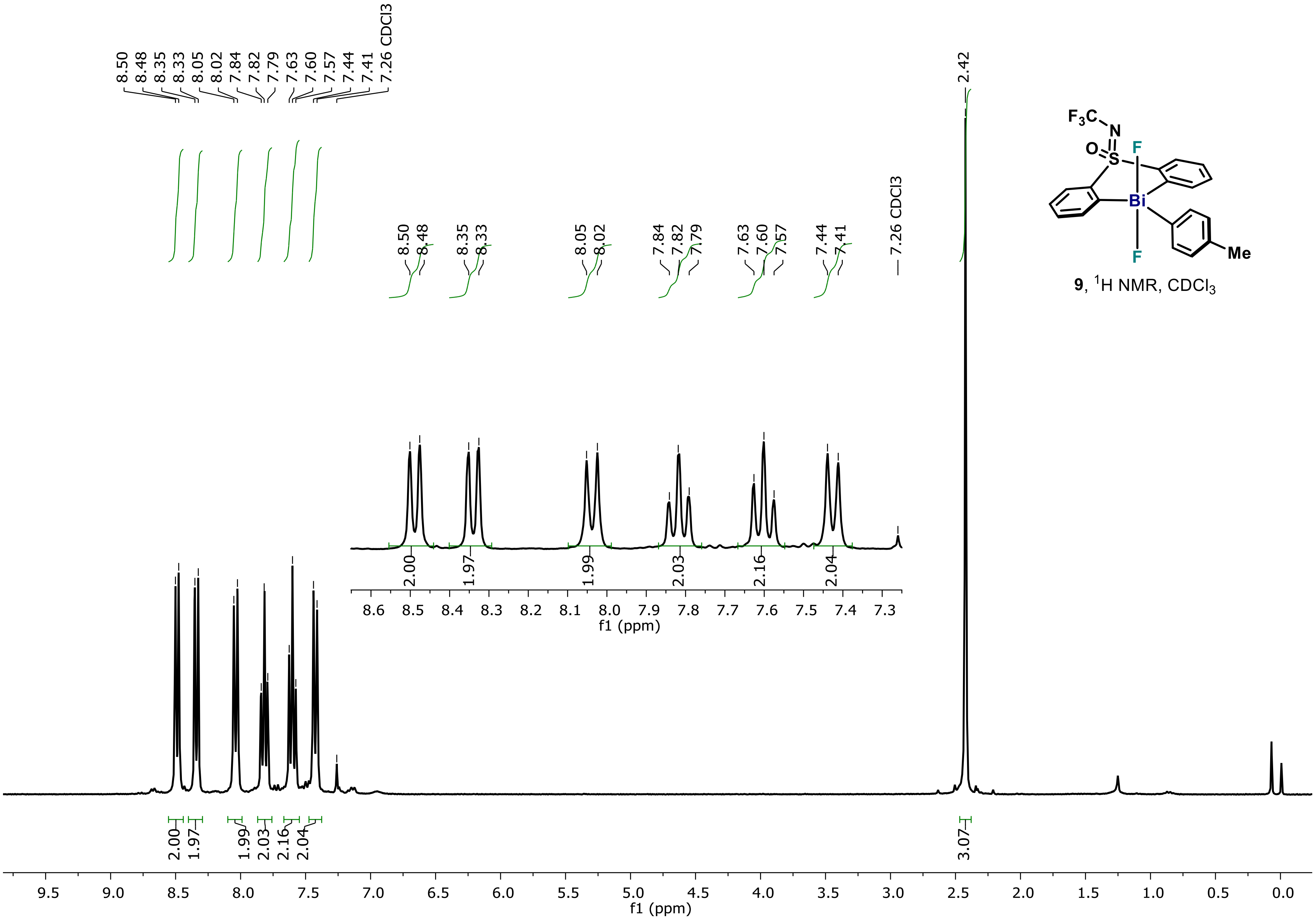
8.50 8.48 8.35 8.33 8.05 8.02 7.84 7.82 7.79 7.63 7.60 7.57 7.44 7.41 7.26 CDCl3
2.42
F3C N O S F Bi F Me
9, 1H NMR, CDCl3
2.00 1.97 1.99 2.03 2.16 2.04
3.07
f1 (ppm)

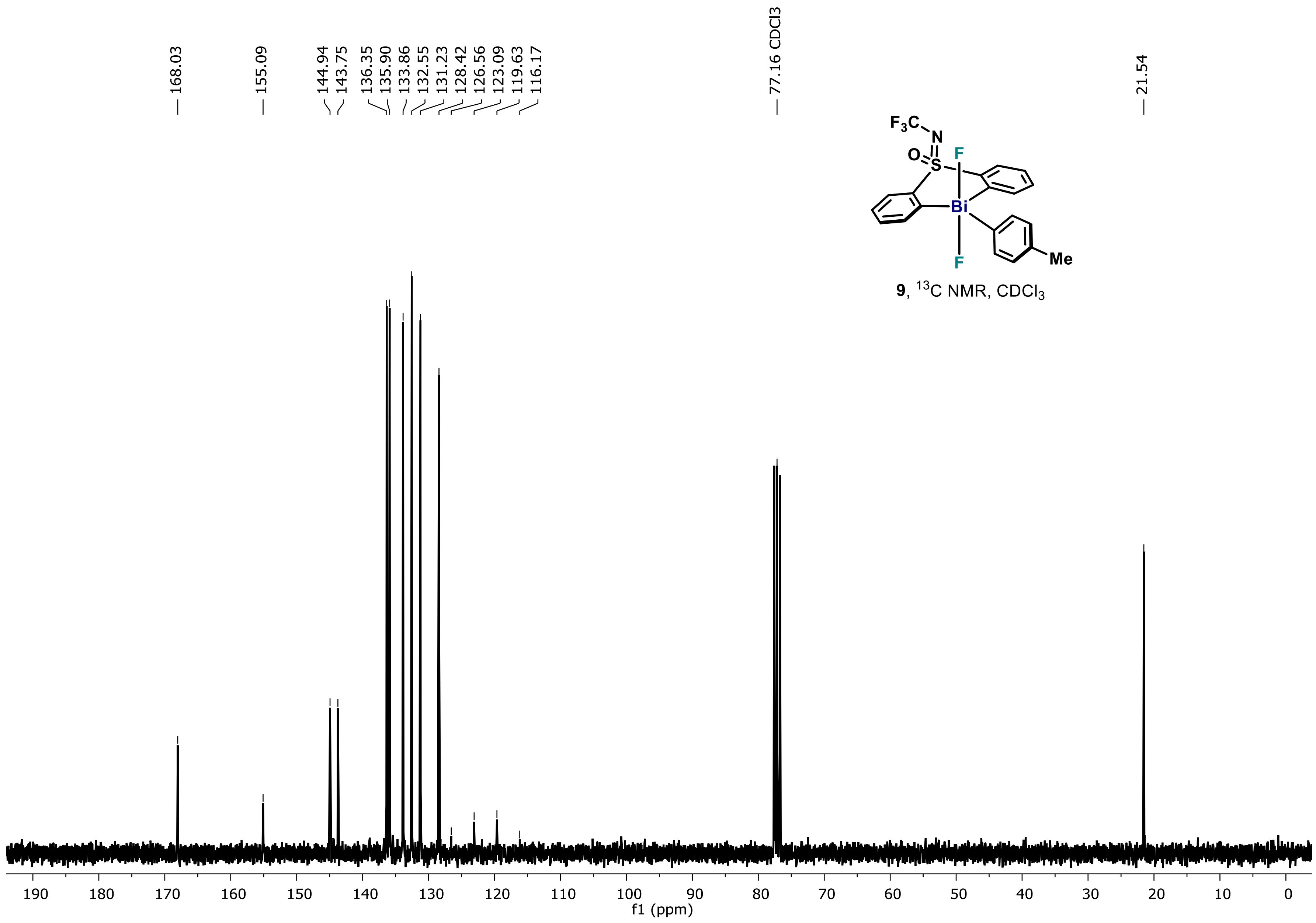

168.03
155.09
144.94
143.75
136.35
135.90
133.86
132.55
131.23
128.42
126.56
123.09
119.63
116.17
77.16 CDCl3
21.54
F3C
N
O
S
F
Bi
F
Me
9, 13C NMR, CDCl3
190 180 170 160 150 140 130 120 110 100 90 80 70 60 50 40 30 20 10 0
f1 (ppm)

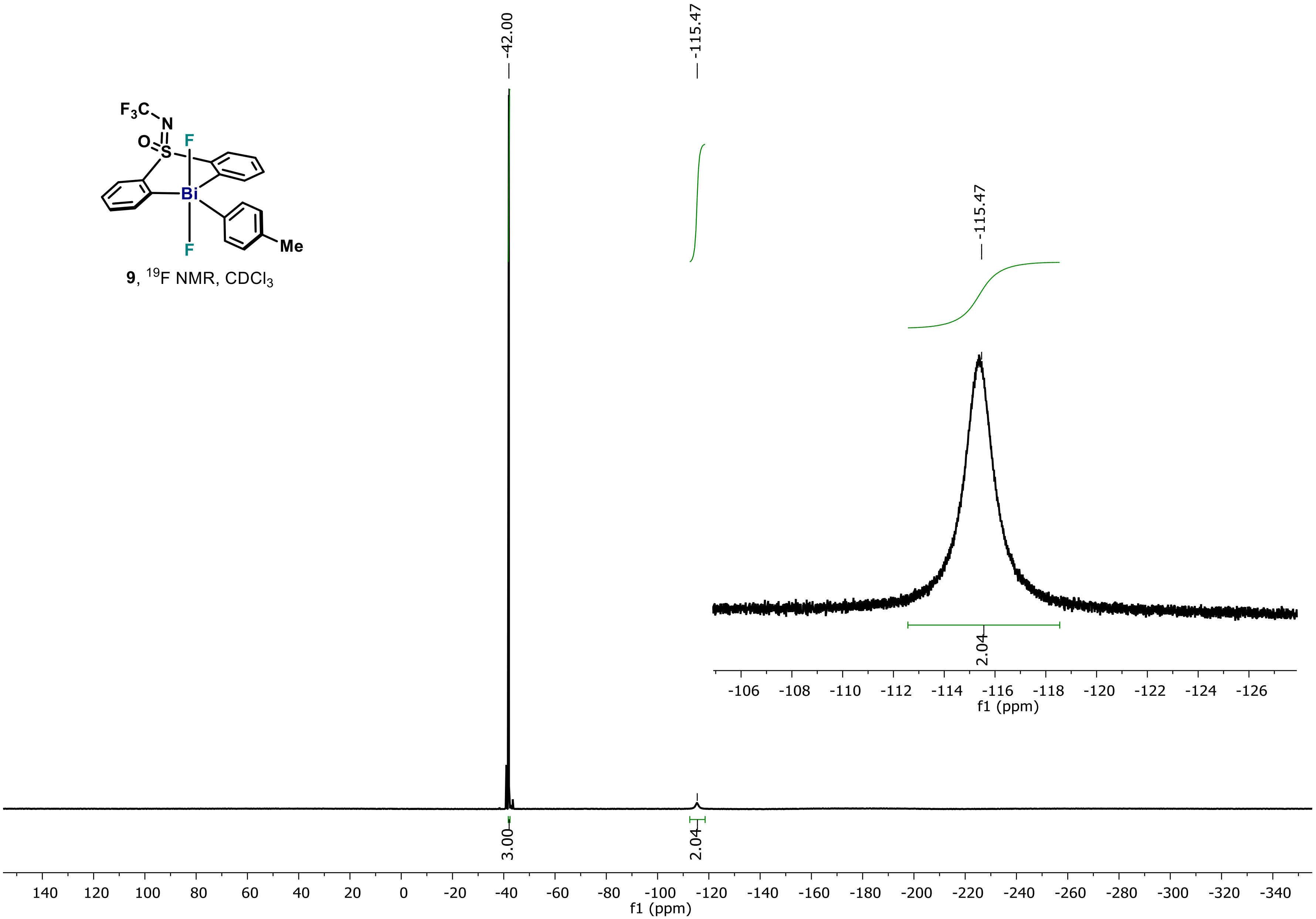
F3C
N
O
S
F
Bi
F
Me
9, 19F NMR, CDCl3
-42.00
-115.47
3.00
2.04
f1 (ppm)

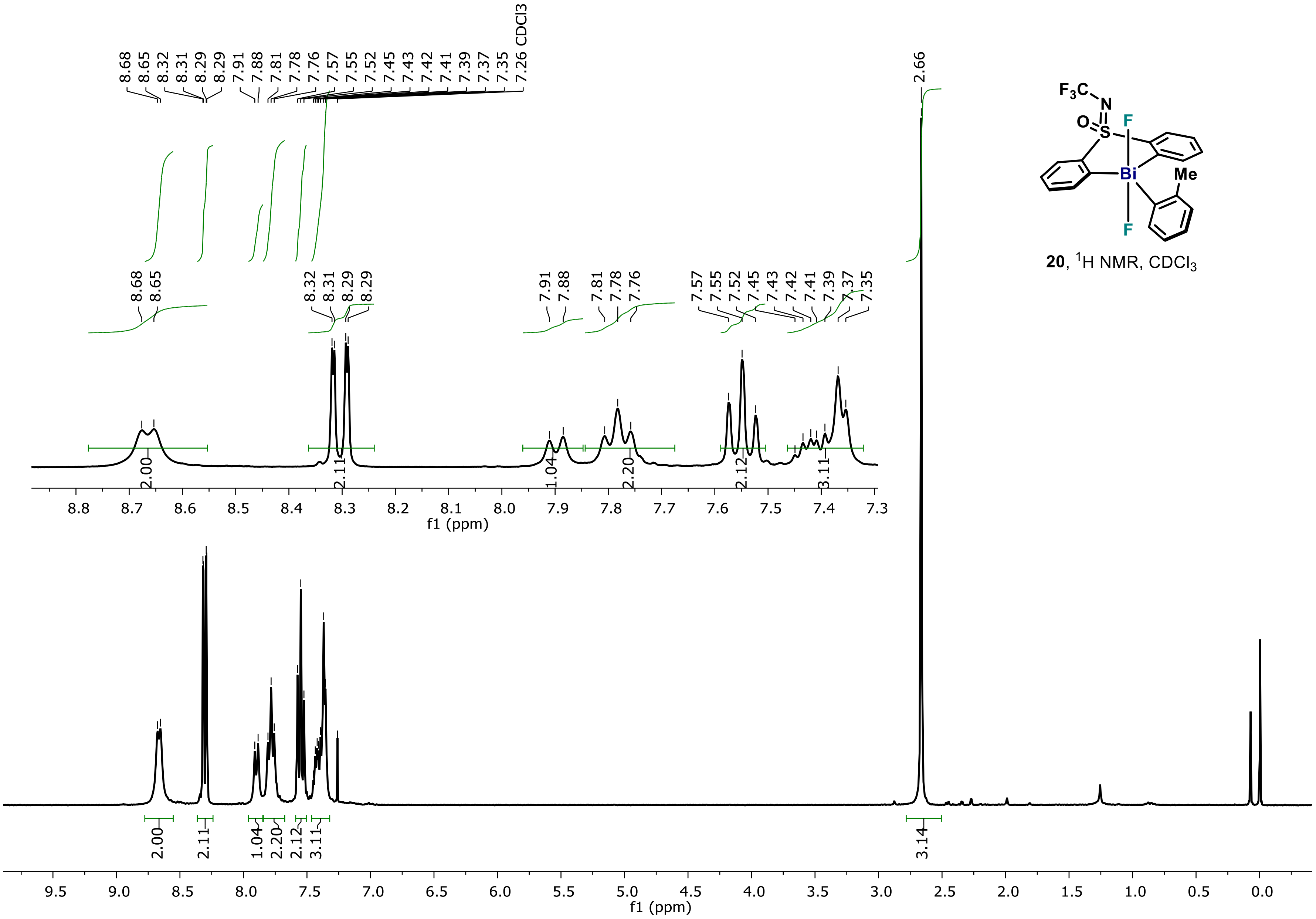

20, ^{1}H NMR, $CDCl_3$

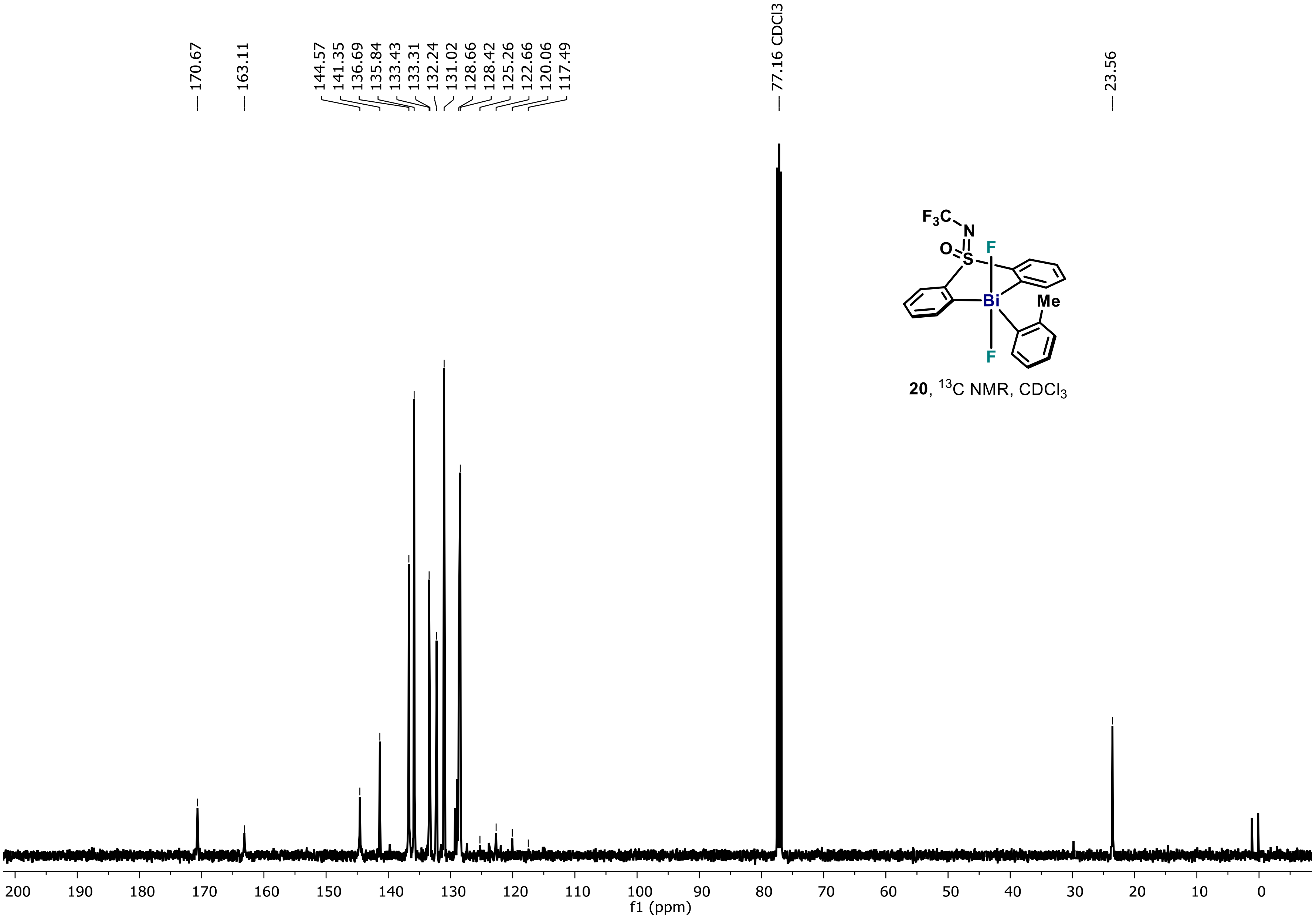

170.67
163.11
144.57
141.35
136.69
135.84
133.43
133.31
132.24
131.02
128.66
128.42
125.26
122.66
120.06
117.49
77.16 CDCl3
23.56
F3C
N
O
S
F
Bi
Me
F
20, 13C NMR, CDCl3
200 190 180 170 160 150 140 130 120 110 100 90 80 70 60 50 40 30 20 10 0
f1 (ppm)

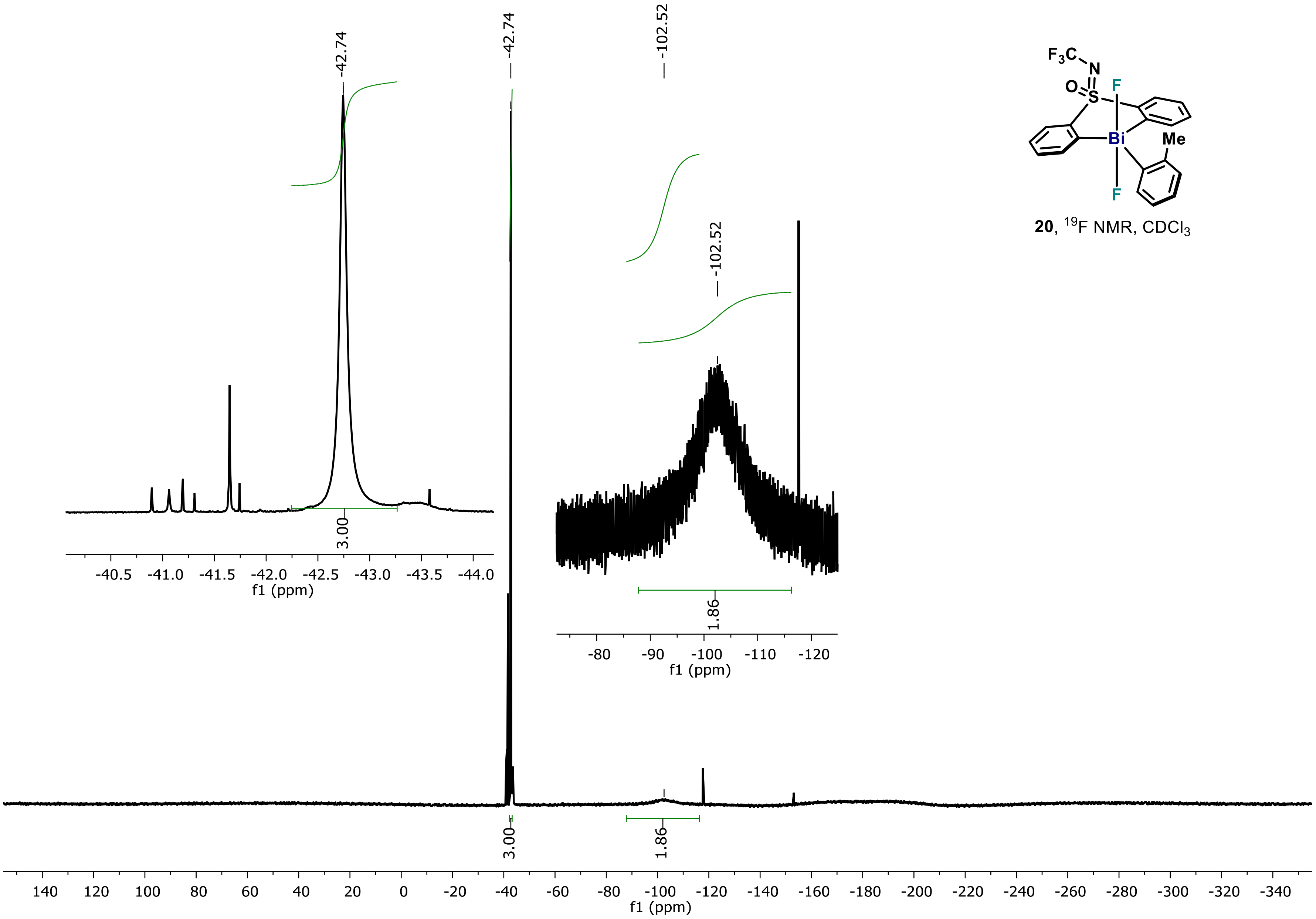

20, ^{19}F NMR, $CDCl_3$

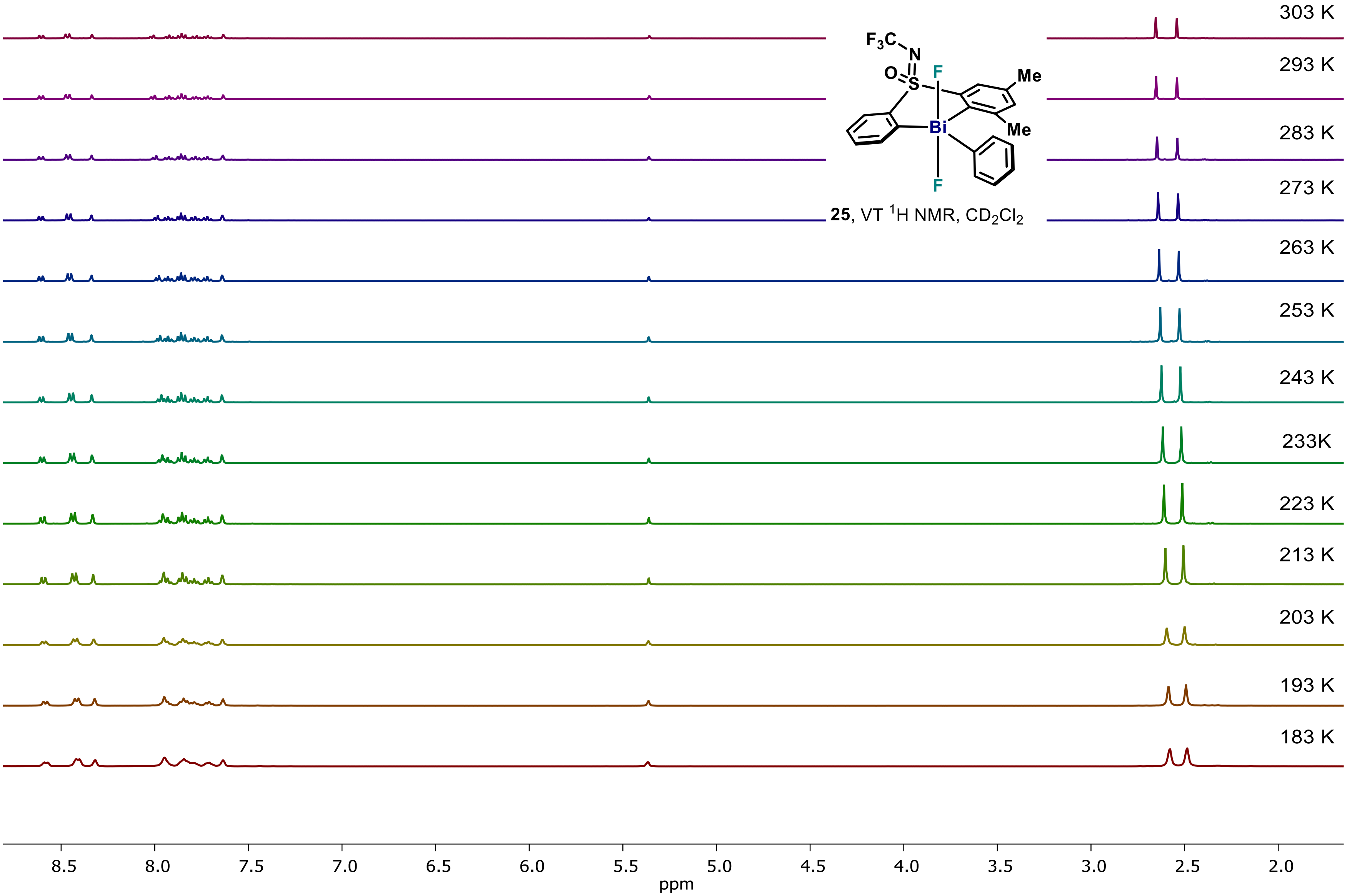

303 K
293 K
283 K
273 K
263 K
253 K
243 K
233K
223 K
213 K
203 K
193 K
183 K
F3C
N
O
S
F
Me
Bi
Me
F
25, VT 1H NMR, CD2Cl2
8.5
8.0
7.5
7.0
6.5
6.0
5.5
5.0
4.5
4.0
3.5
3.0
2.5
2.0
ppm

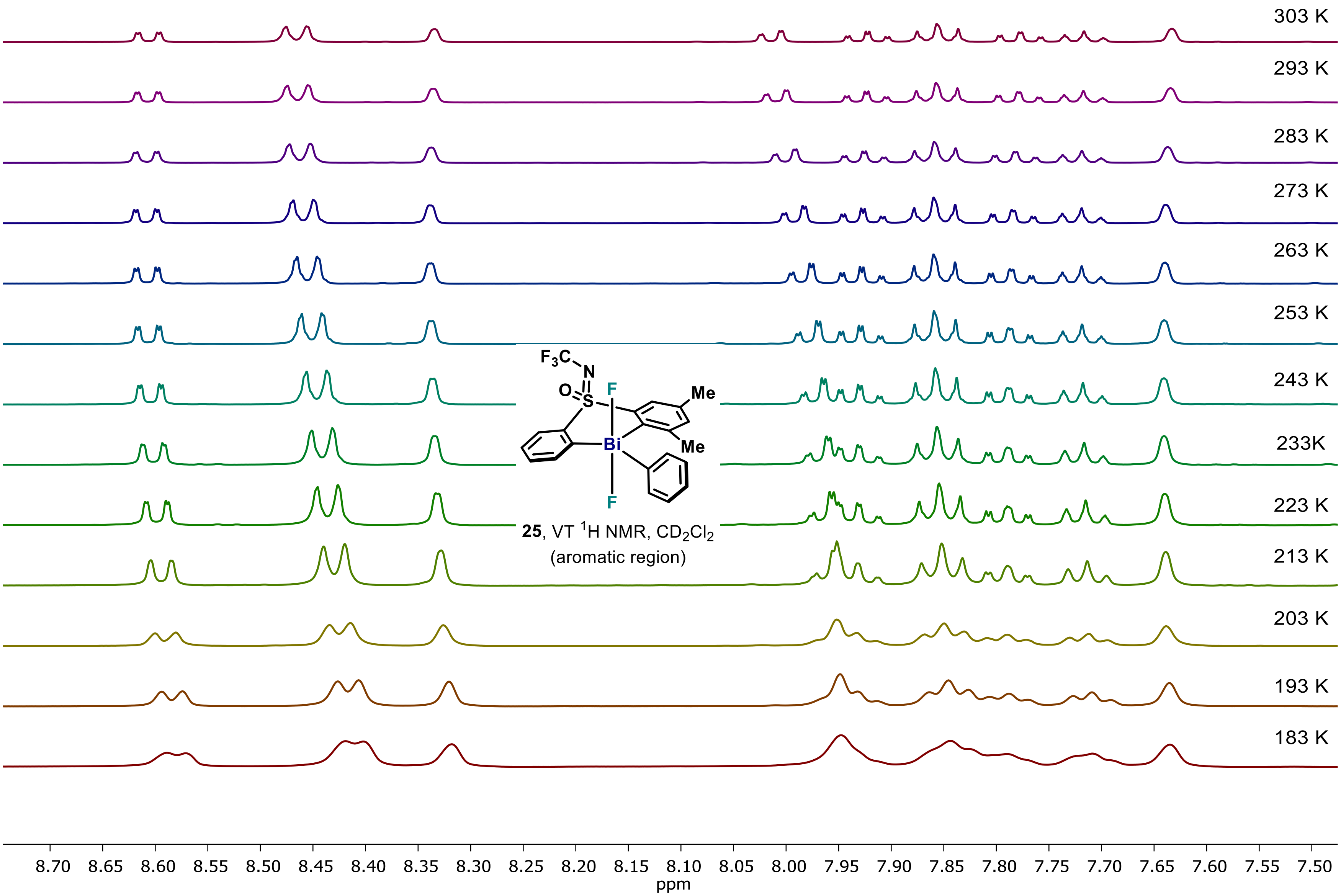

303 K
293 K
283 K
273 K
263 K
253 K
243 K
233K
223 K
213 K
203 K
193 K
183 K
F3C
N
O
S
F
Me
Bi
Me
F
25, VT 1H NMR, CD2Cl2
(aromatic region)
8.70 8.65 8.60 8.55 8.50 8.45 8.40 8.35 8.30 8.25 8.20 8.15 8.10 8.05 8.00 7.95 7.90 7.85 7.80 7.75 7.70 7.65 7.60 7.55 7.50
ppm

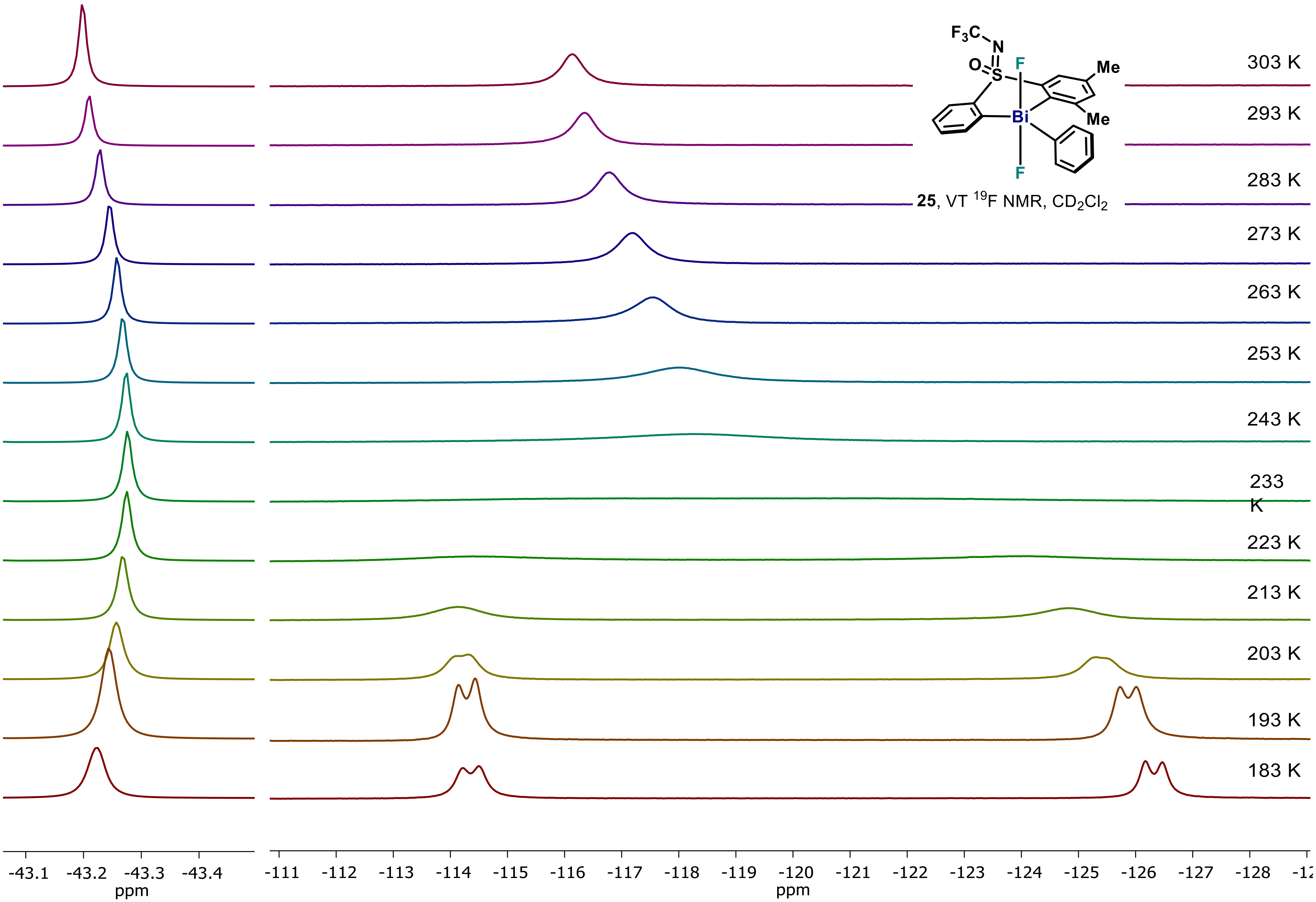

F_3C
N
O
S
F
Me
Bi
Me
F
25, VT ^{19}F NMR, CD_2Cl_2
303 K
293 K
283 K
273 K
263 K
253 K
243 K
233 K
223 K
213 K
203 K
193 K
183 K
-43.1 -43.2 -43.3 -43.4
ppm
-111 -112 -113 -114 -115 -116 -117 -118 -119 -120 -121 -122 -123 -124 -125 -126 -127 -128
ppm

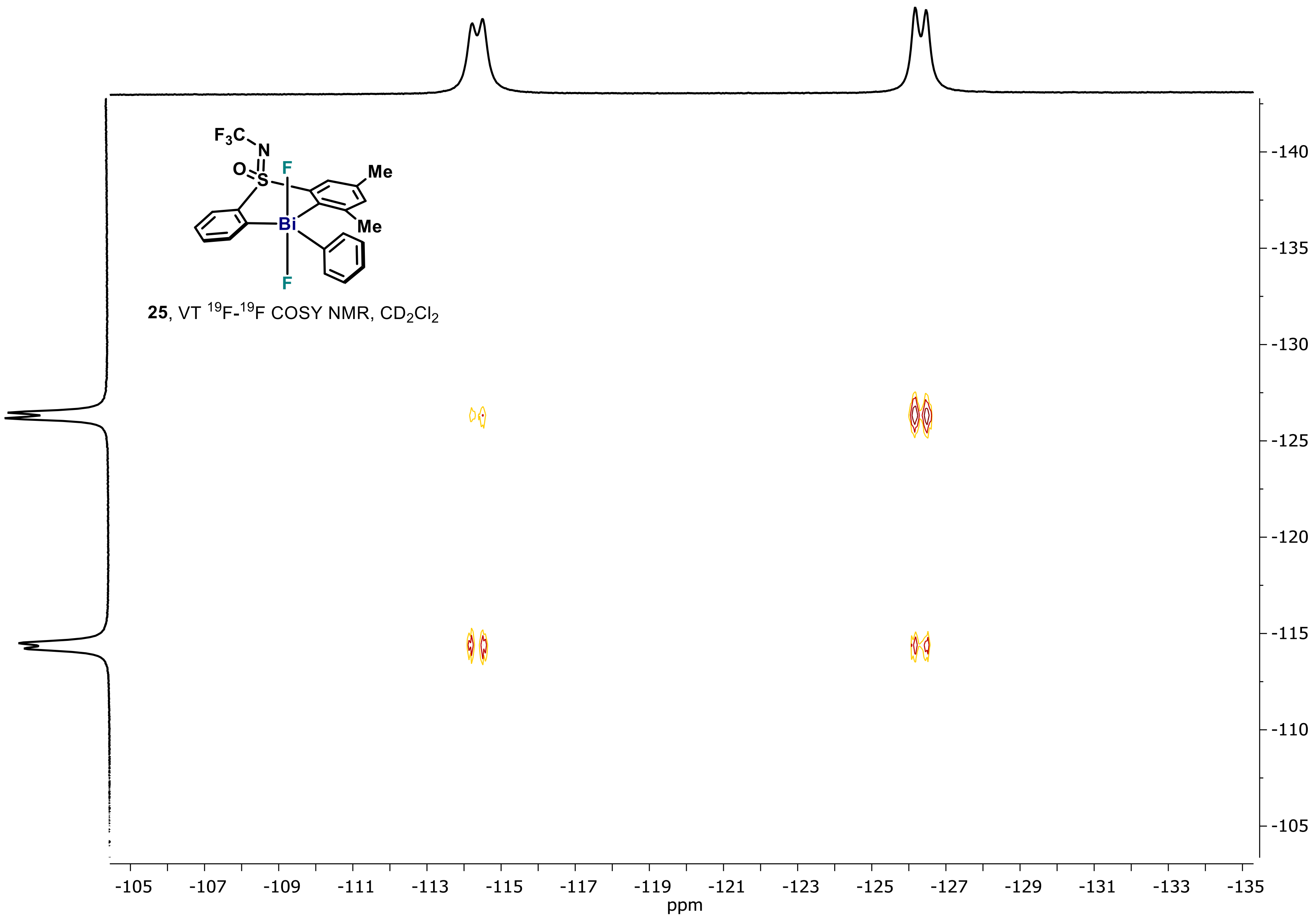

F3C
N
O
S
F
Me
Bi
Me
F
25, VT 19F-19F COSY NMR, CD2Cl2
-140
-135
-130
-125
-120
-115
-110
-105
ppm
-105
-107
-109
-111
-113
-115
-117
-119
-121
-123
-125
-127
-129
-131
-133
-135
ppm

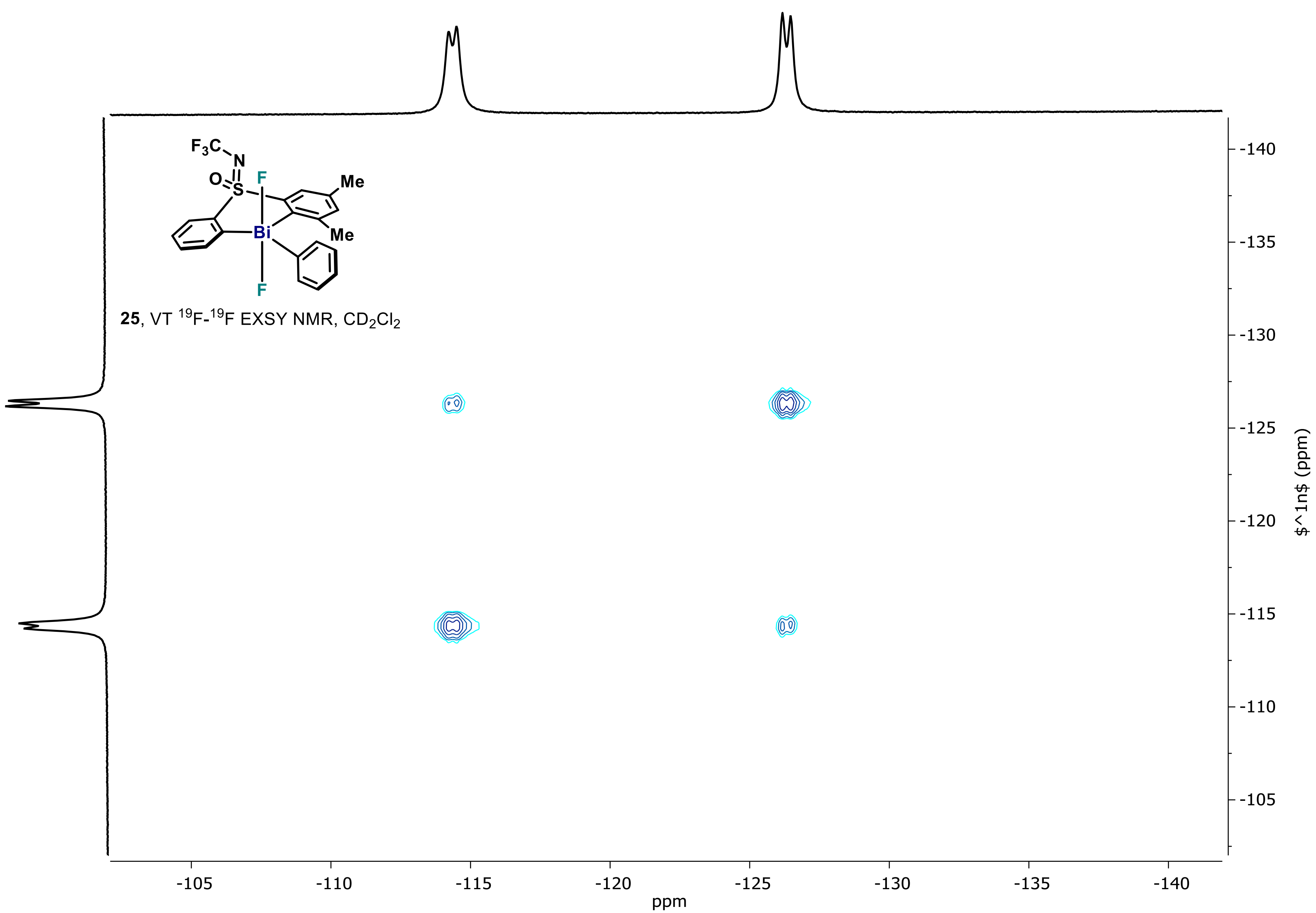
F3C
N
O
S
F
Me
Bi
Me
F
25, VT 19F-19F EXSY NMR, CD2Cl2
-140
-135
-130
-125
-120
-115
-110
-105
^1n (ppm)
-105
-110
-115
-120
-125
-130
-135
-140
ppm

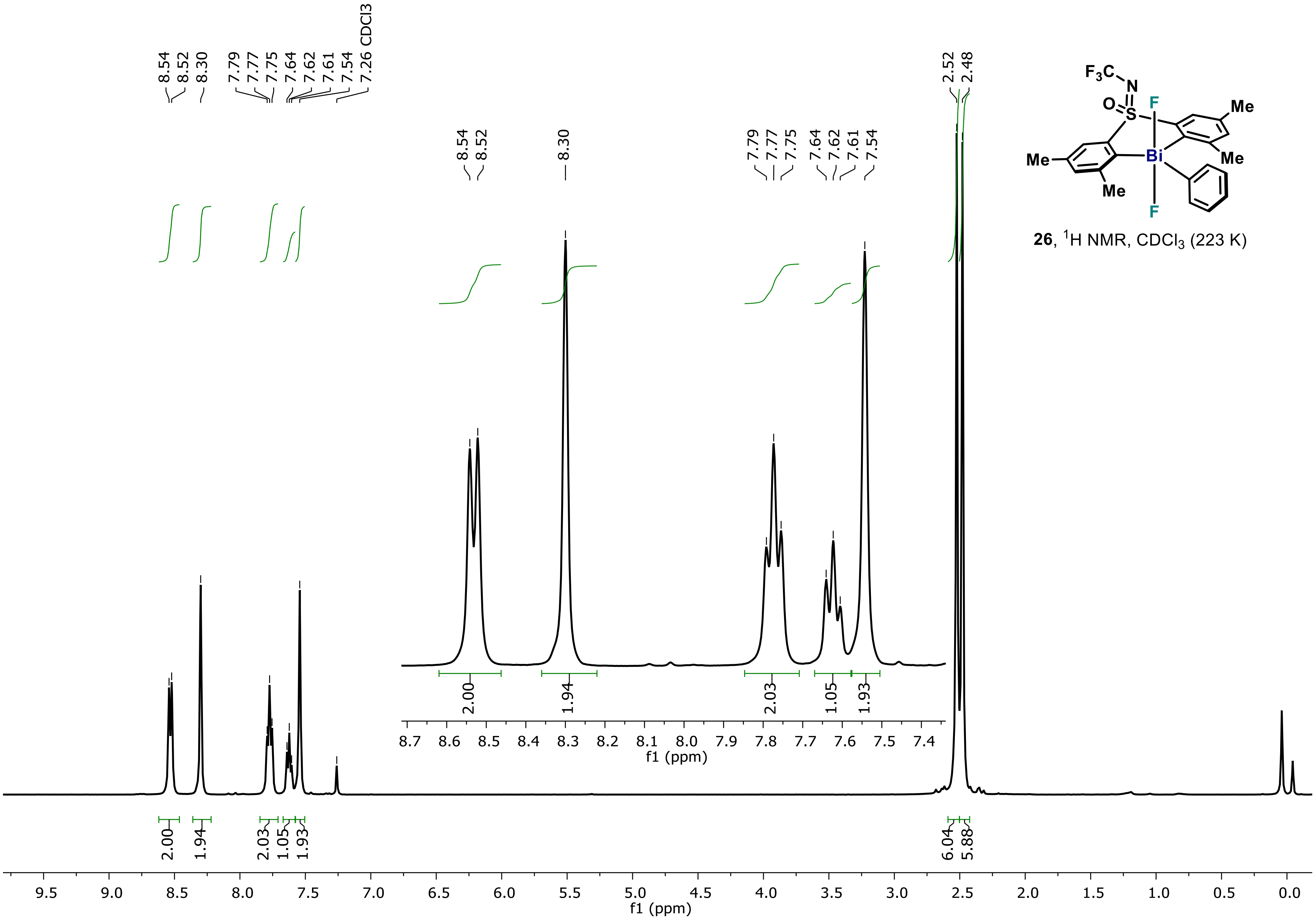

26, ^{1}H NMR, $CDCl_3$ (223 K)

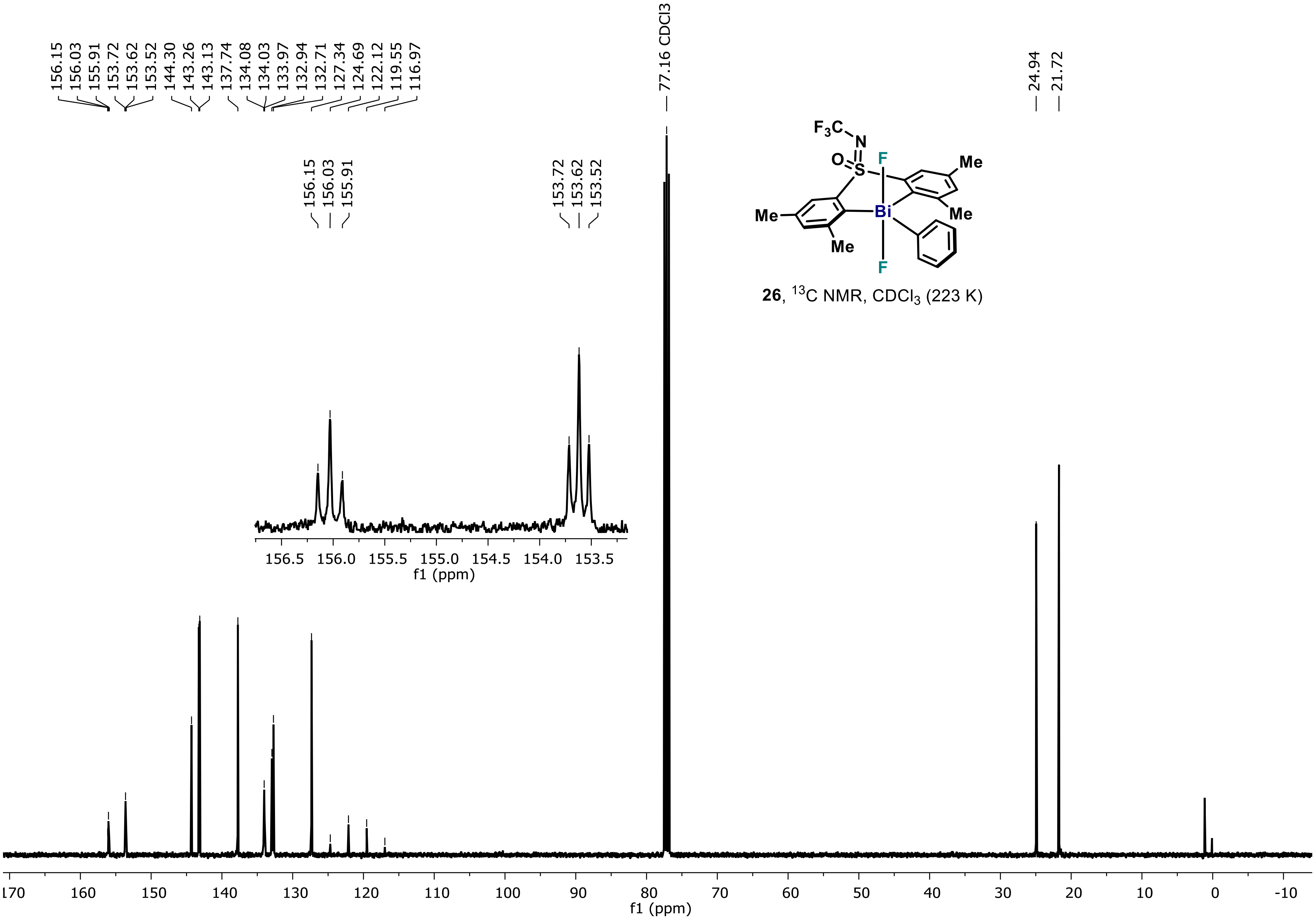

26, ^{13}C NMR, $CDCl_3$ (223 K)

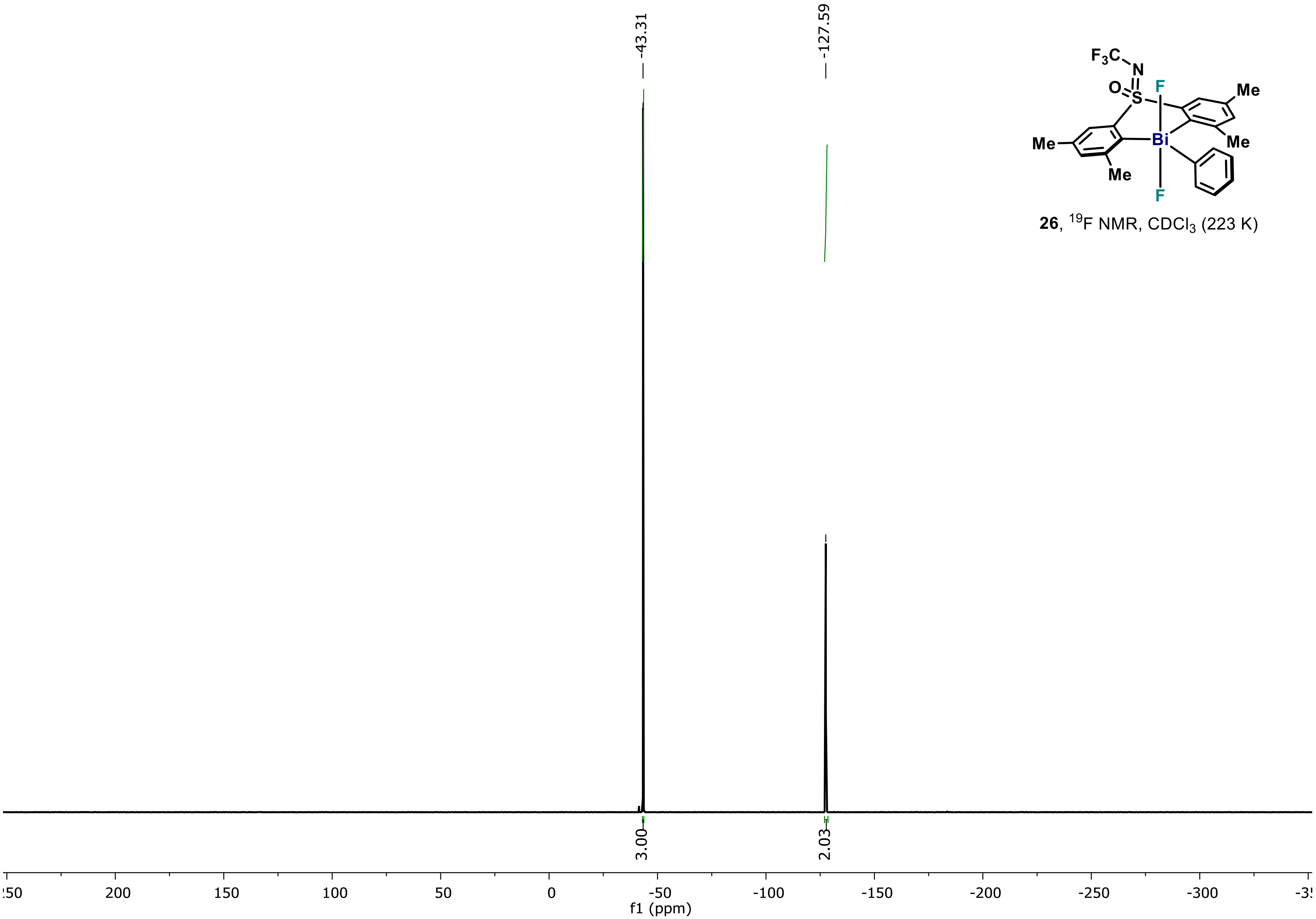

-43.31
-127.59
3.00
2.03
250
200
150
100
50
0
-50
-100
-150
-200
-250
-300
f1 (ppm)
F3C
N
O
S
F
Me
Me
Bi
Me
Me
F
26, 19F NMR, CDCl3 (223 K)

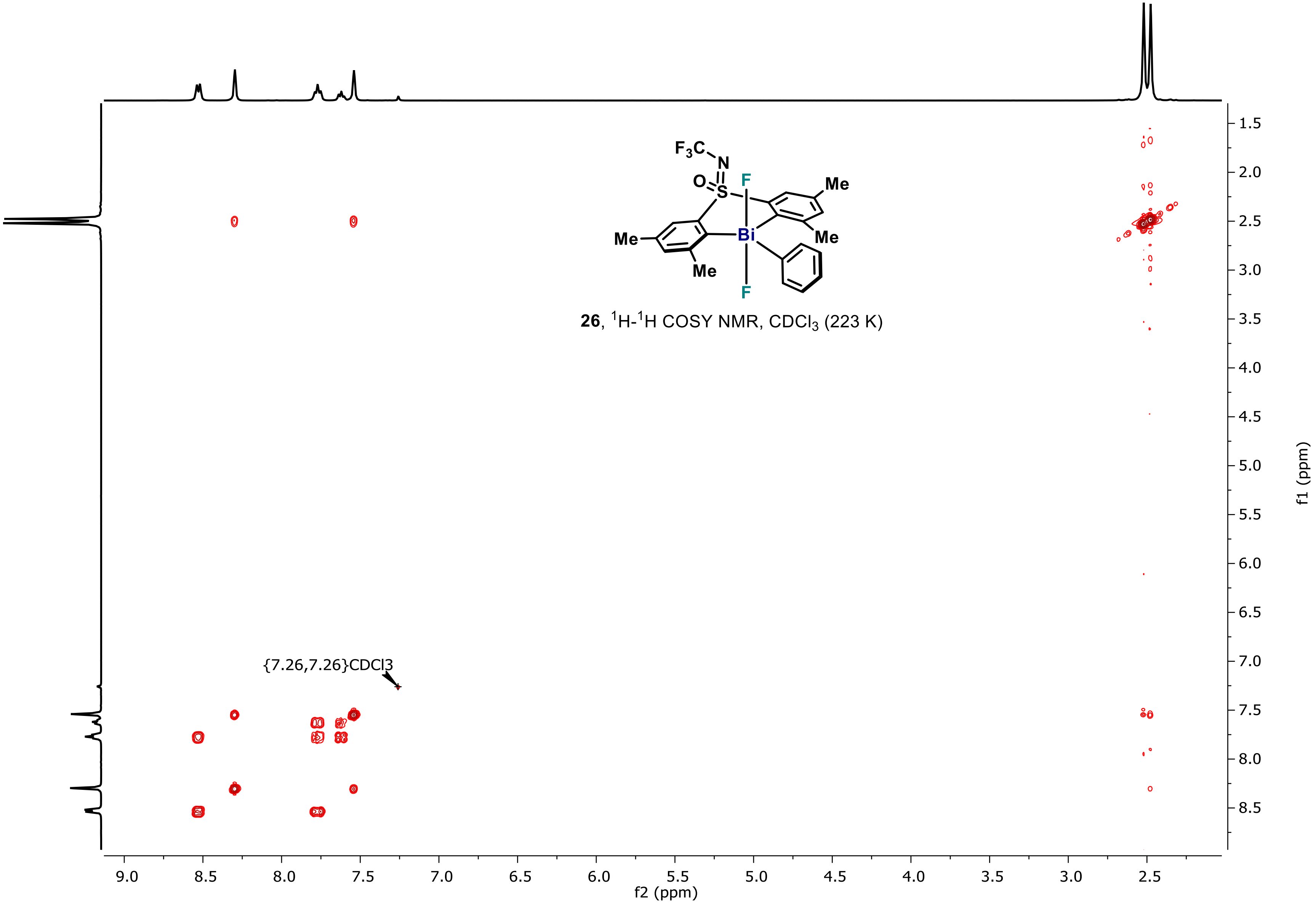

F3C
N
O
S
F
Me
Me
Bi
Me
Me
F
26, 1H-1H COSY NMR, CDCl3 (223 K)
{7.26,7.26}CDCl3
f1 (ppm)
f2 (ppm)

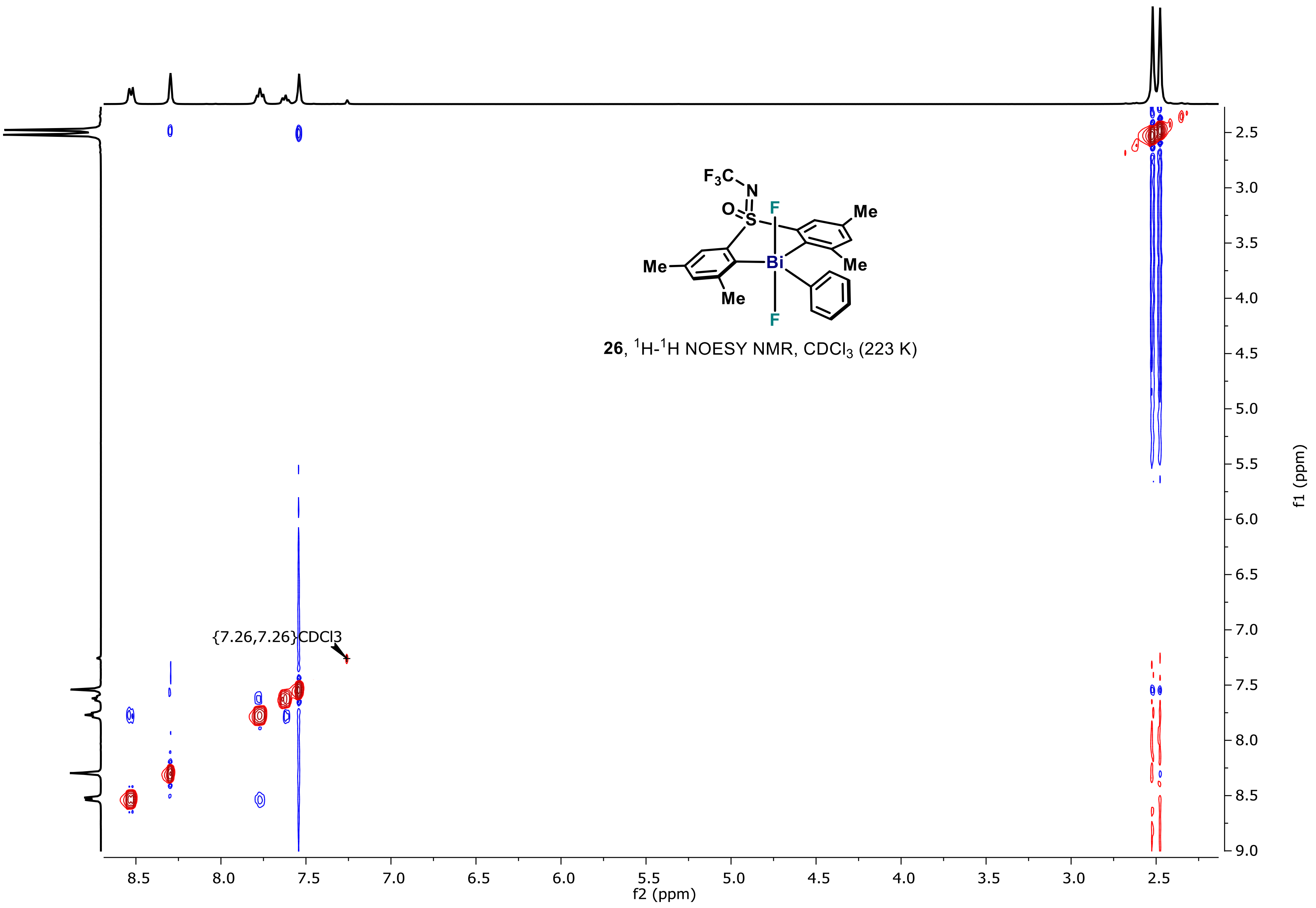
F3C
N
O
S
F
Me
Me
Bi
Me
Me
F
26, 1H-1H NOESY NMR, CDCl3 (223 K)
{7.26,7.26}CDCl3
f2 (ppm)
f1 (ppm)

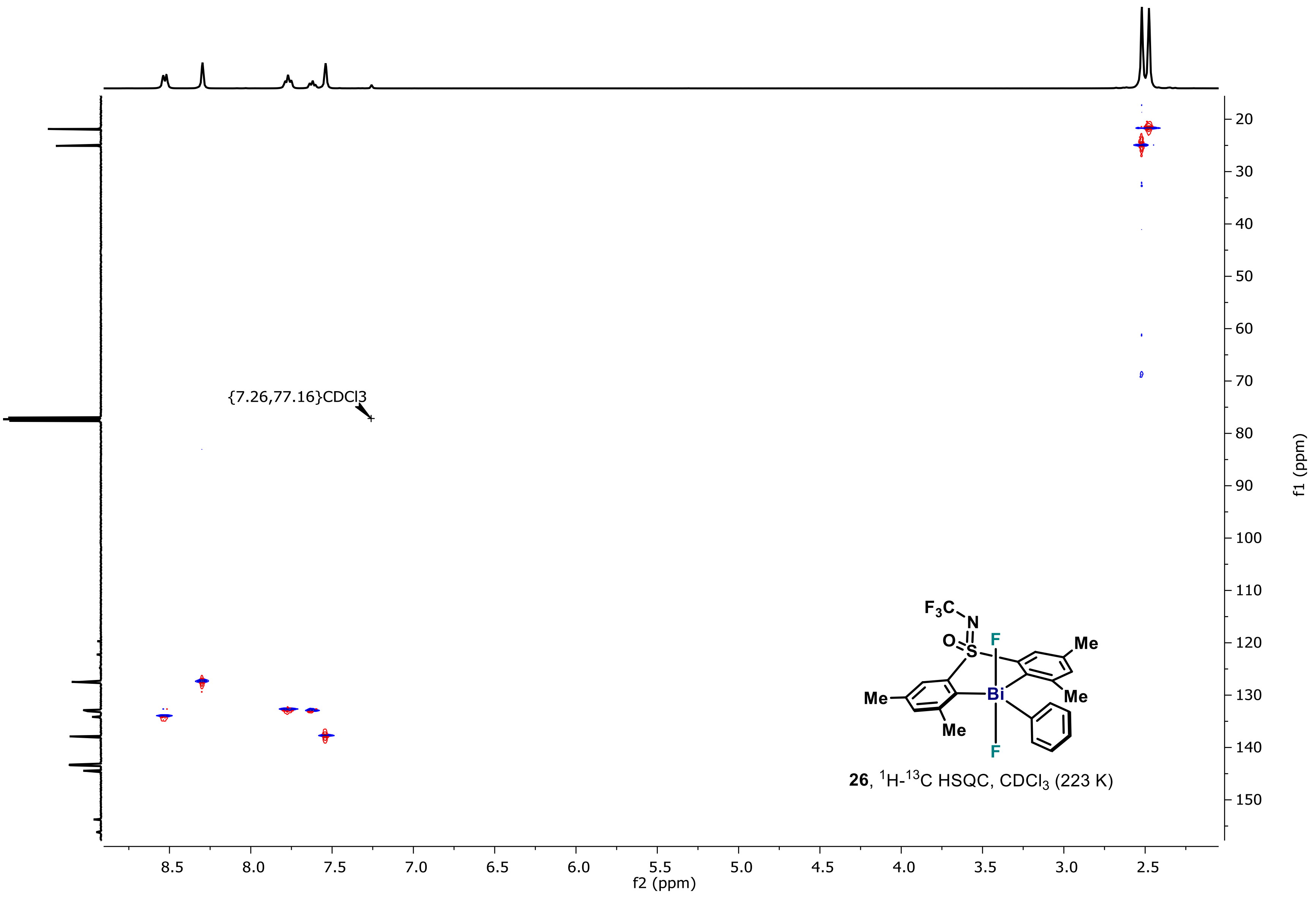

26, ^{1}H-^{13}C HSQC, $CDCl_3$ (223 K)

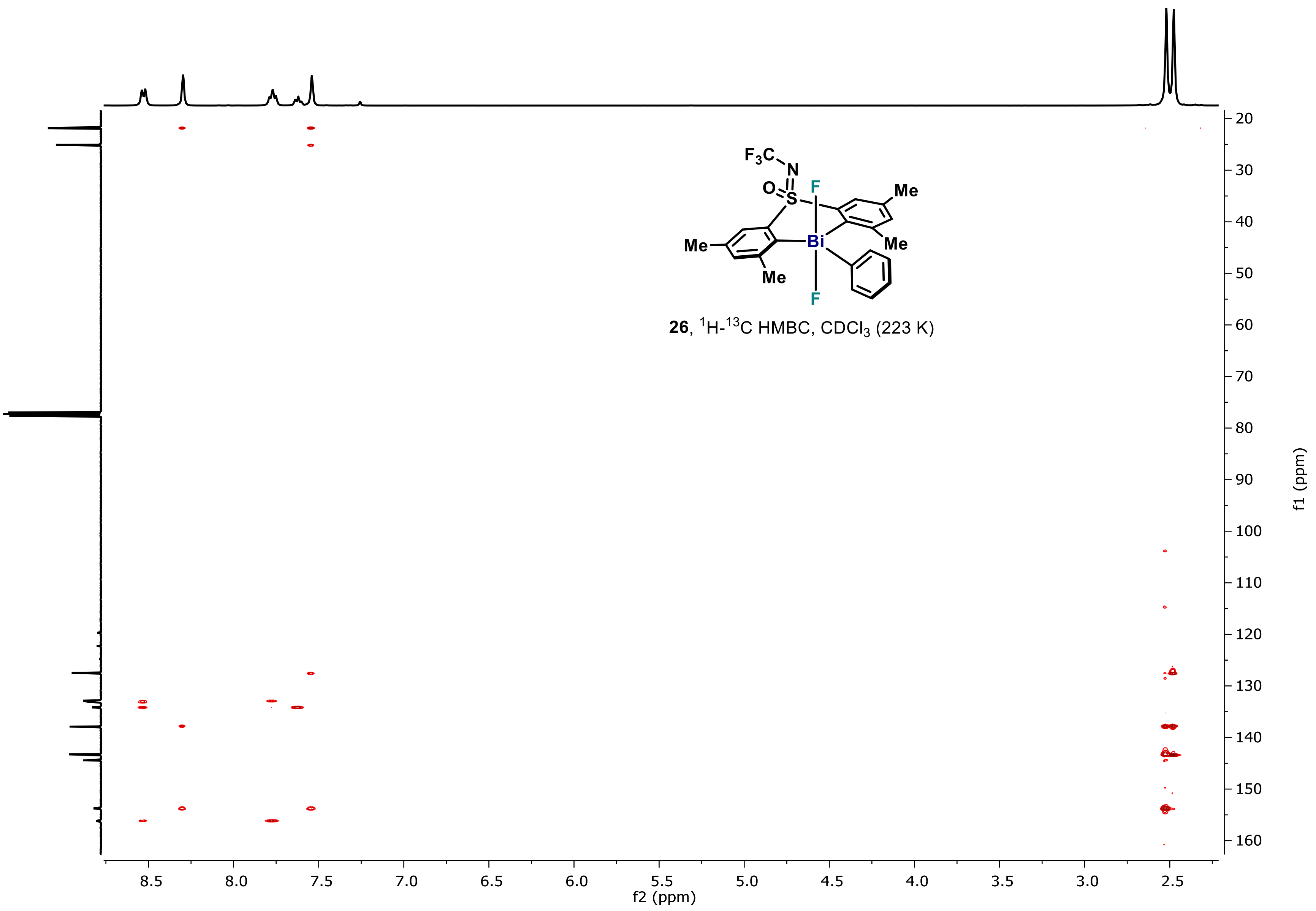
F3C
N
O
S
F
Me
Me
Bi
Me
Me
F
26, 1H-13C HMBC, CDCl3 (223 K)
f1 (ppm)
f2 (ppm)

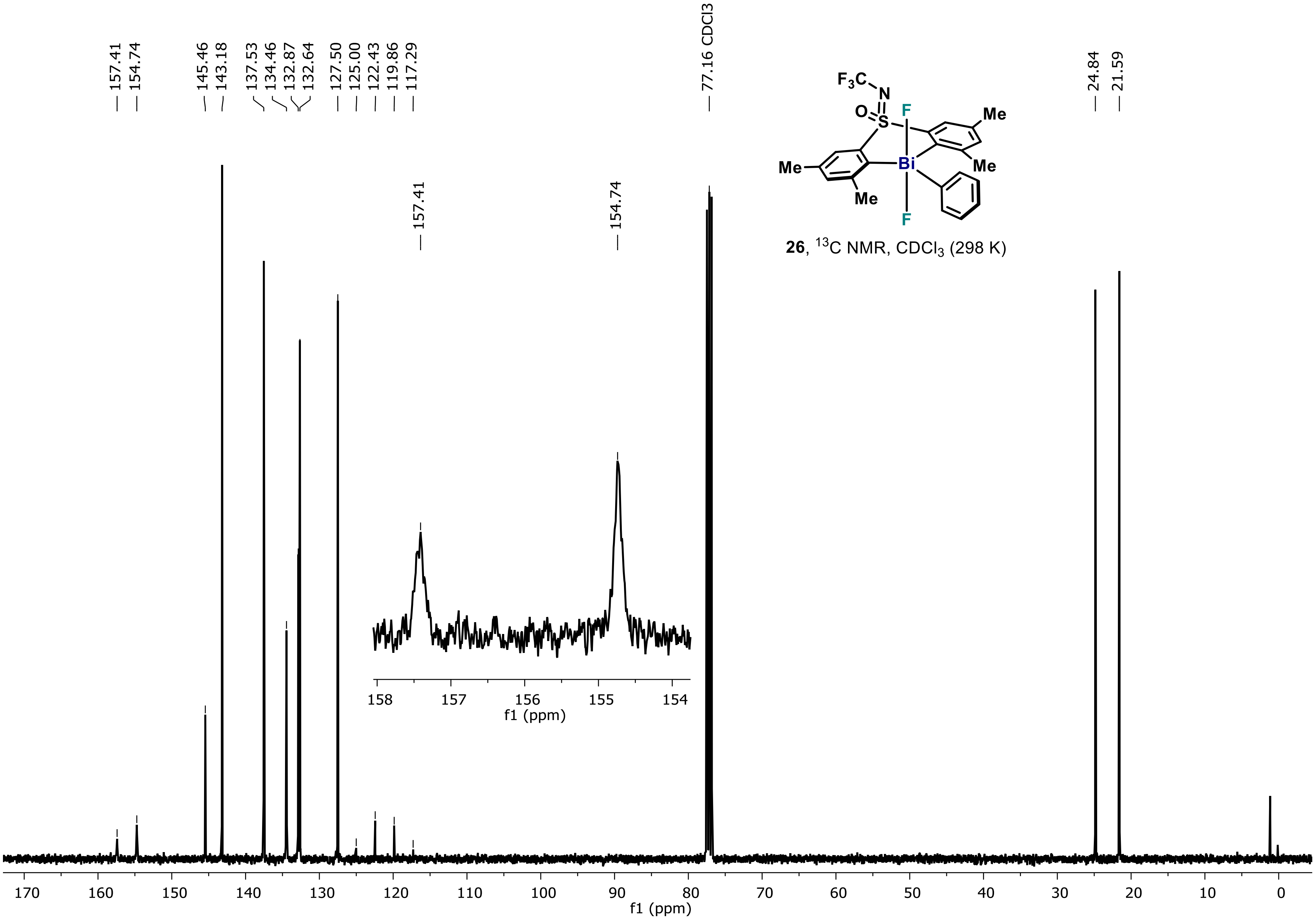
157.41
154.74
145.46
143.18
137.53
134.46
132.87
132.64
127.50
125.00
122.43
119.86
117.29
77.16 CDCl3
24.84
21.59
F3C
N
O
S
F
Me
Bi
Me
Me
Me
F
26, 13C NMR, CDCl3 (298 K)
157.41
154.74
158
157
156
155
154
f1 (ppm)
170
160
150
140
130
120
110
100
90
80
70
60
50
40
30
20
10
0
f1 (ppm)

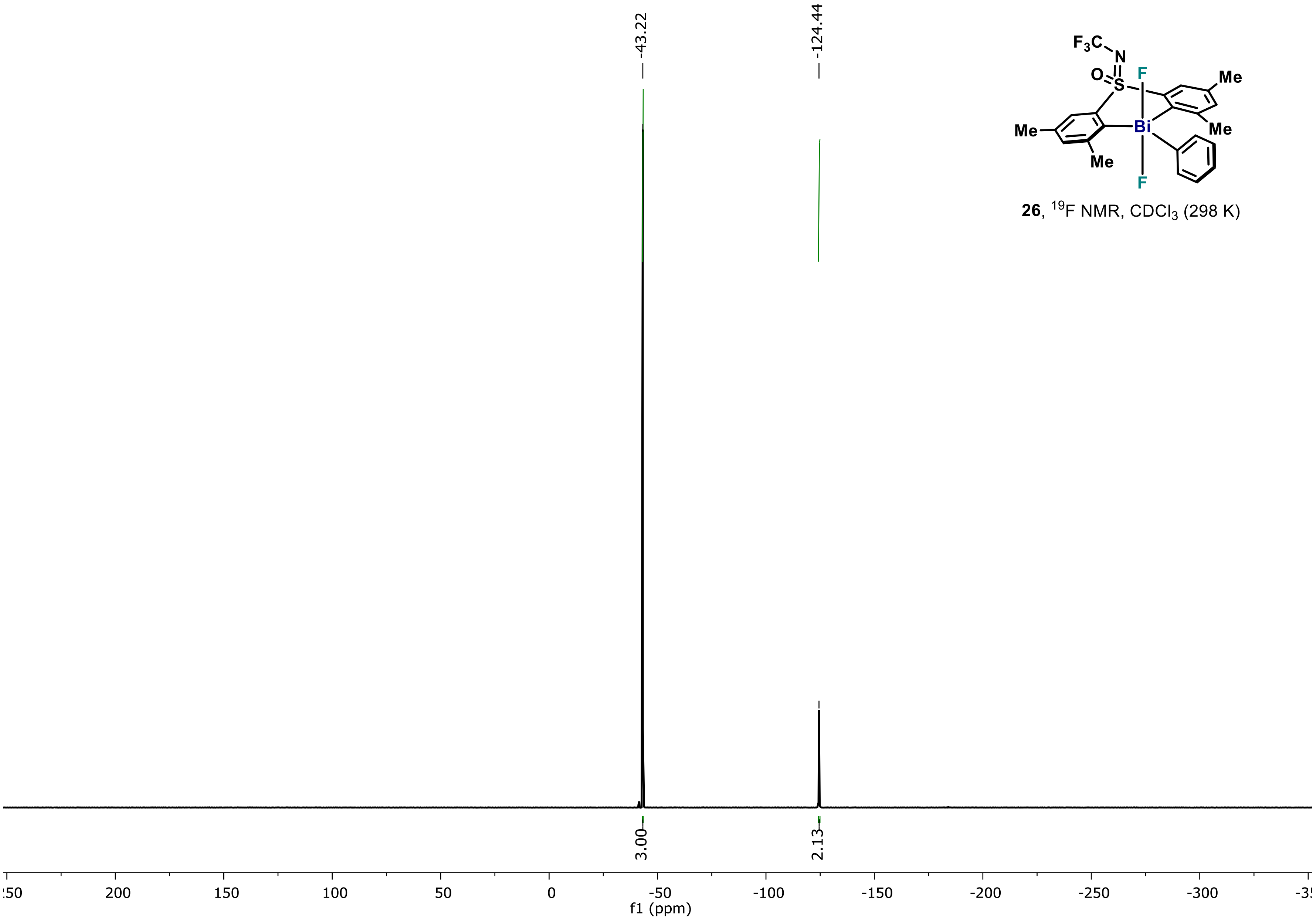

26, ^{19}F NMR, $CDCl_3$ (298 K)

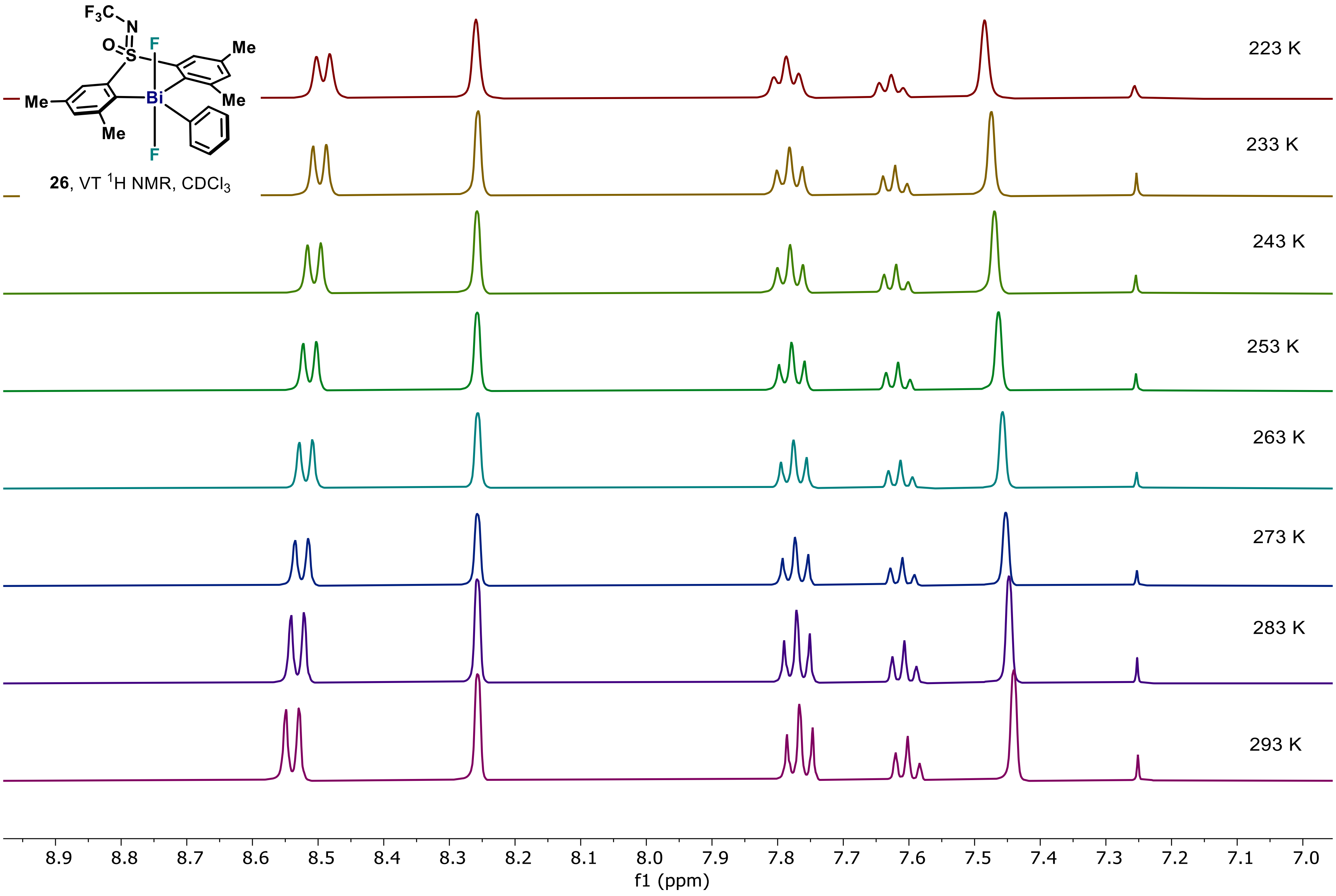

F3C
N
O
S
F
Me
Me
Bi
Me
Me
F
26, VT 1H NMR, CDCl3
223 K
233 K
243 K
253 K
263 K
273 K
283 K
293 K
8.9
8.8
8.7
8.6
8.5
8.4
8.3
8.2
8.1
8.0
7.9
7.8
7.7
7.6
7.5
7.4
7.3
7.2
7.1
7.0
f1 (ppm)

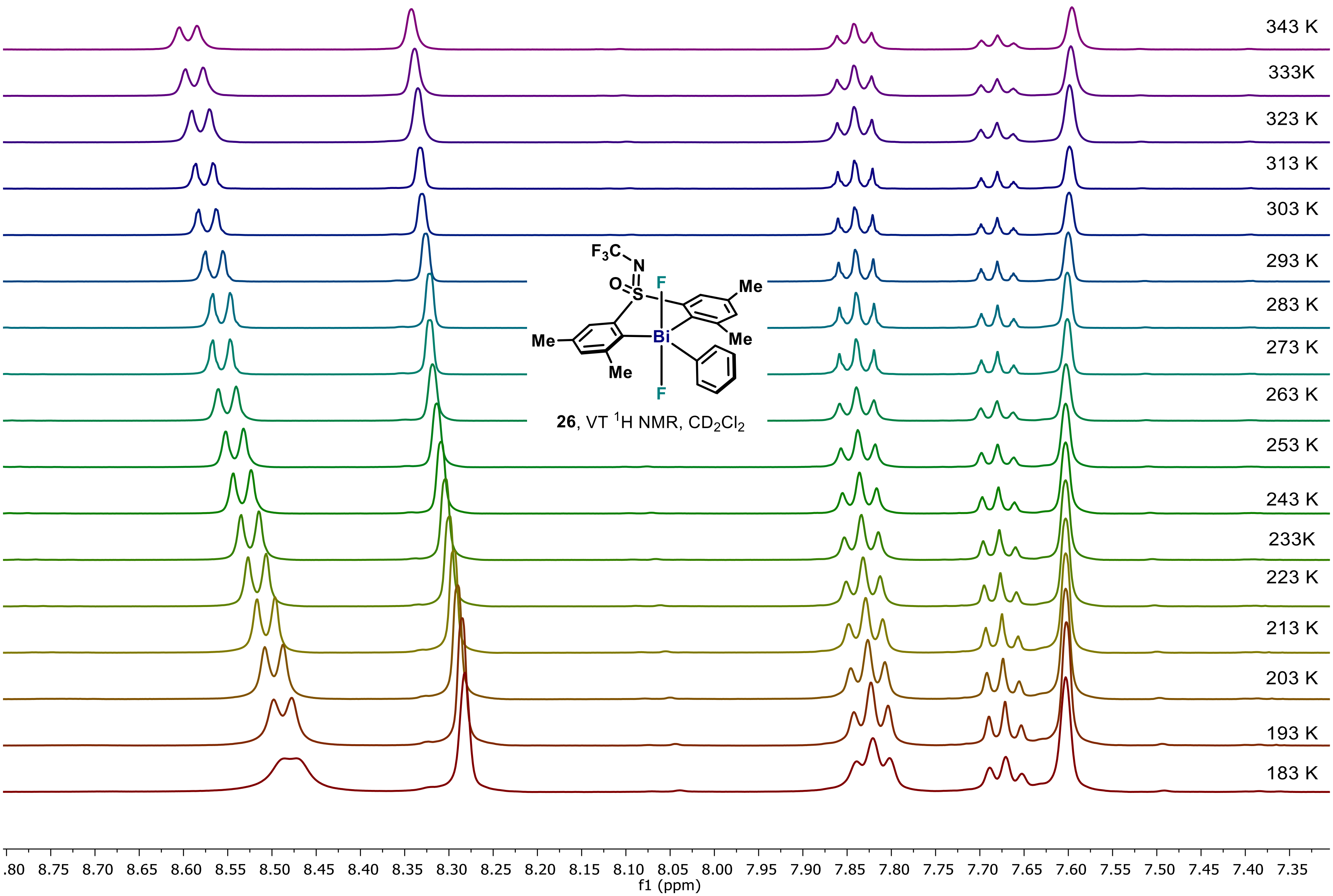

26, VT 1H NMR, CD2Cl2
343 K
333K
323 K
313 K
303 K
293 K
283 K
273 K
263 K
253 K
243 K
233K
223 K
213 K
203 K
193 K
183 K
.80 8.75 8.70 8.65 8.60 8.55 8.50 8.45 8.40 8.35 8.30 8.25 8.20 8.15 8.10 8.05 8.00 7.95 7.90 7.85 7.80 7.75 7.70 7.65 7.60 7.55 7.50 7.45 7.40 7.35
f1 (ppm)

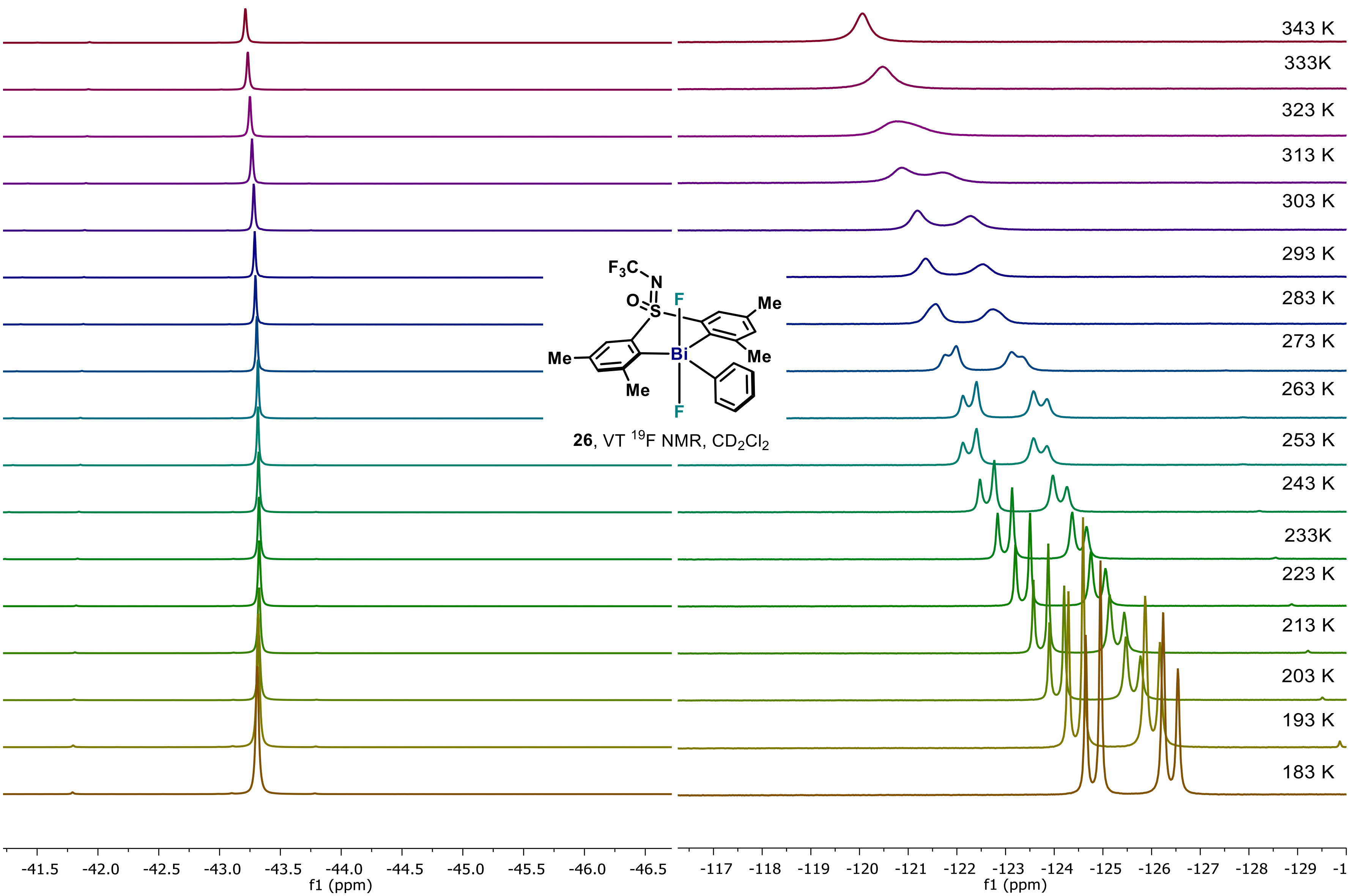

26, VT ^{19}F NMR, CD_2Cl_2

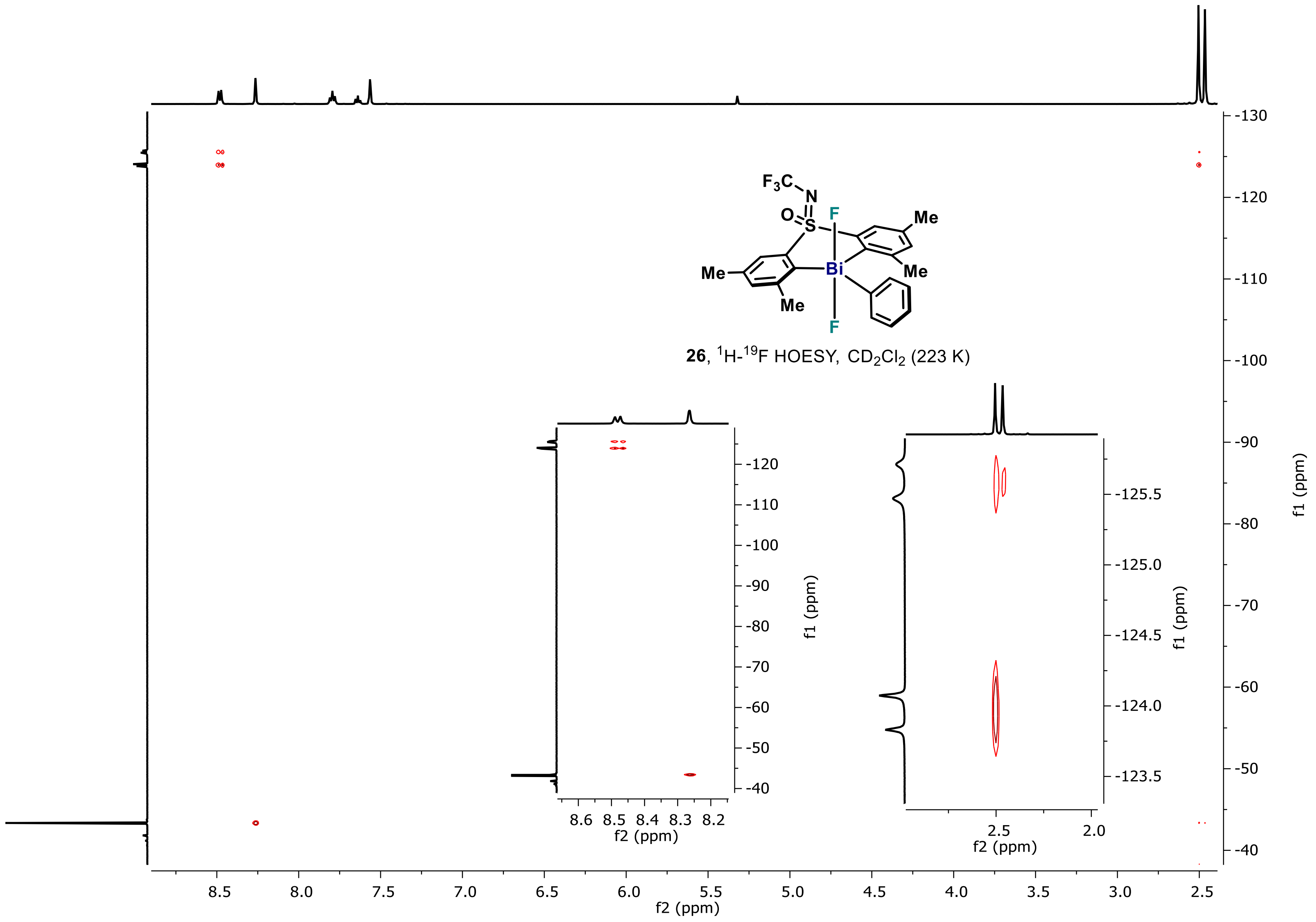
F3C
N
O
S
F
Me
Me
Bi
Me
Me
F
26, 1H-19F HOESY, CD2Cl2 (223 K)
f1 (ppm)
f2 (ppm)

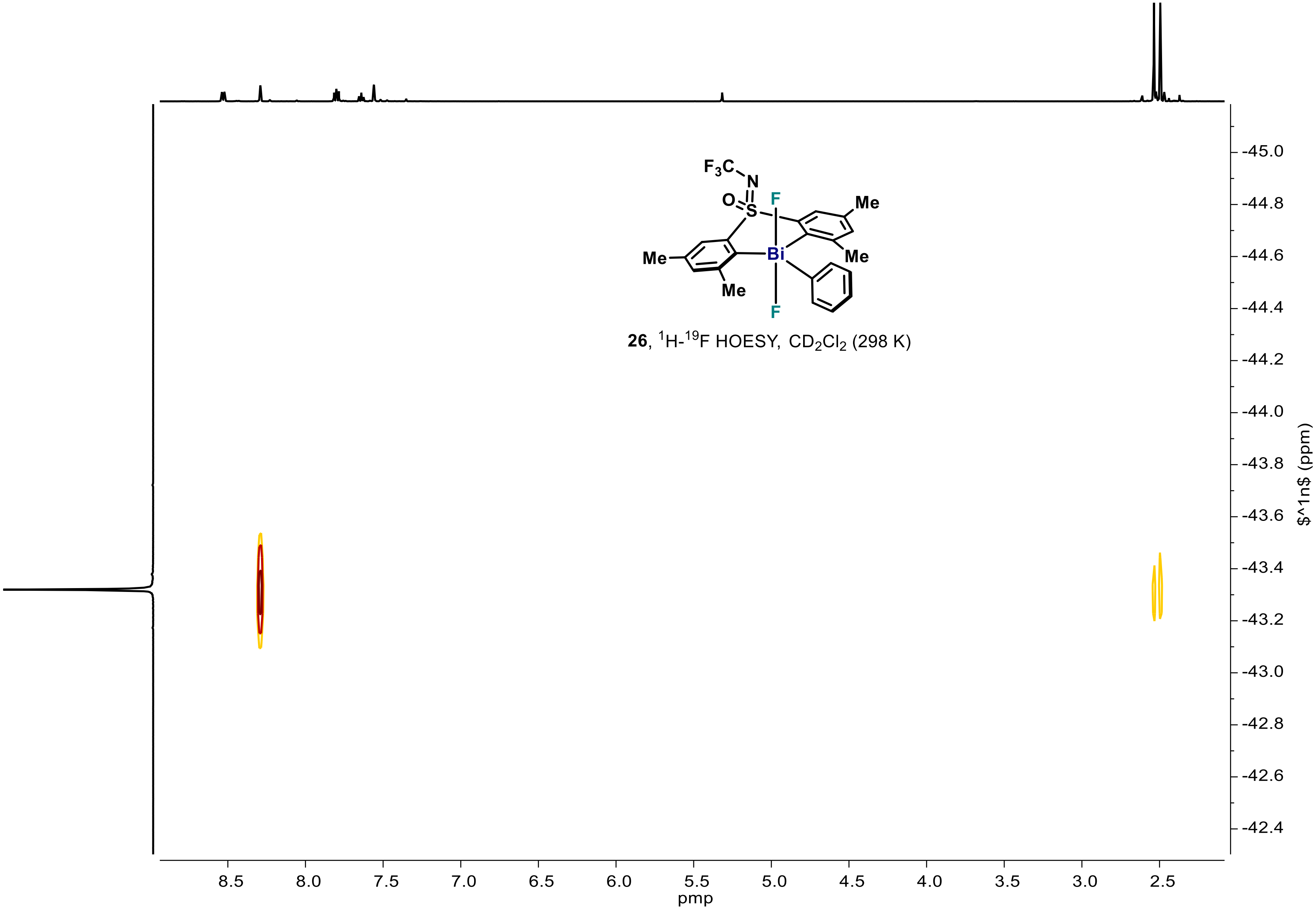
F3C
N
O
S
F
Me
Me
Bi
Me
Me
F
26, 1H-19F HOESY, CD2Cl2 (298 K)
-45.0
-44.8
-44.6
-44.4
-44.2
-44.0
-43.8
-43.6
-43.4
-43.2
-43.0
-42.8
-42.6
-42.4
^1n (ppm)
8.5
8.0
7.5
7.0
6.5
6.0
5.5
5.0
4.5
4.0
3.5
3.0
2.5
pmp

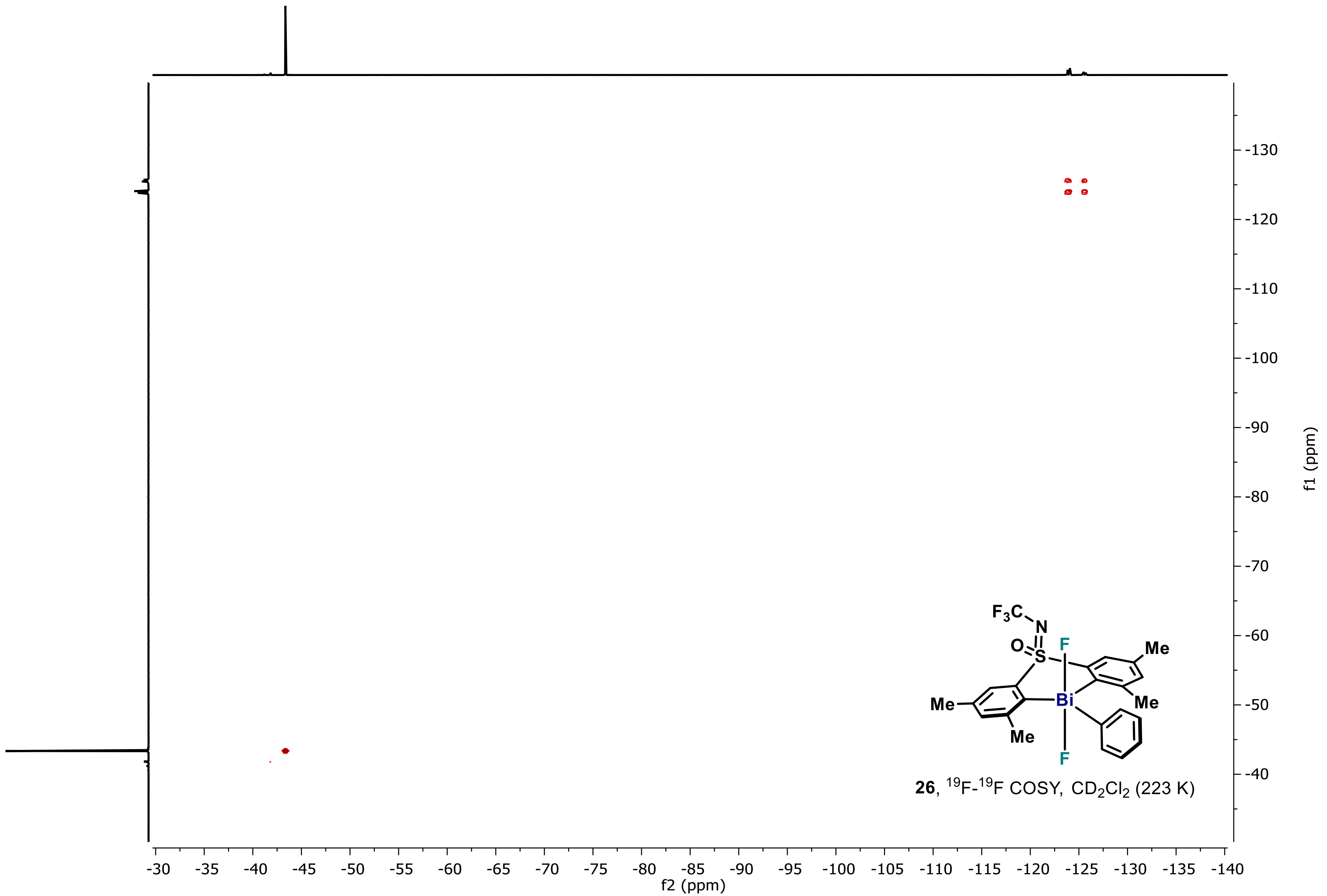
F3C
N
O
S
F
Bi
F
Me
Me
Me
Me
26, 19F-19F COSY, CD2Cl2 (223 K)
f1 (ppm)
f2 (ppm)

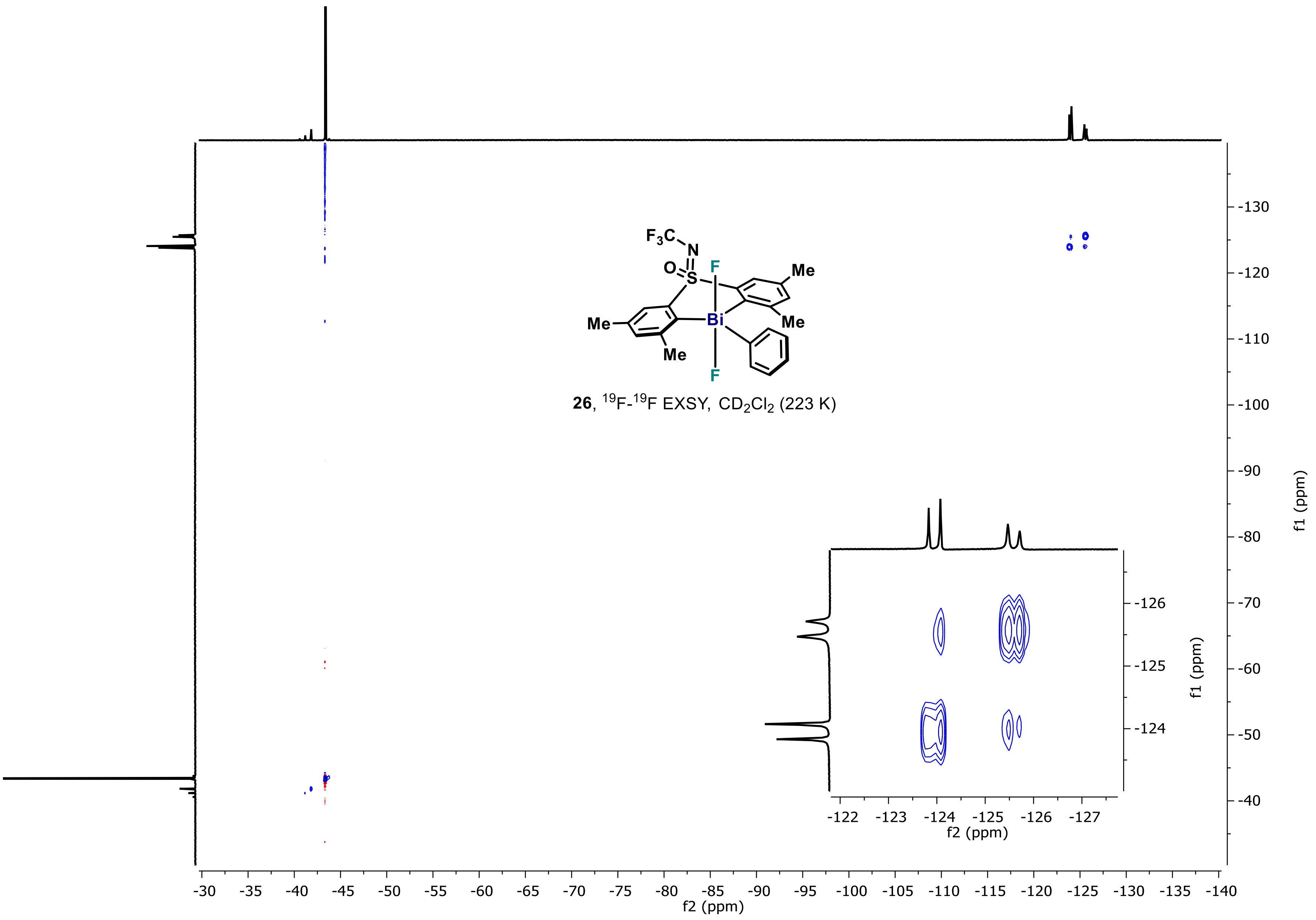
26, 19F-19F EXSY, CD2Cl2 (223 K)
f2 (ppm)
f1 (ppm)
F3C
N
O
S
F
Bi
Me
Me
Me
Me
F

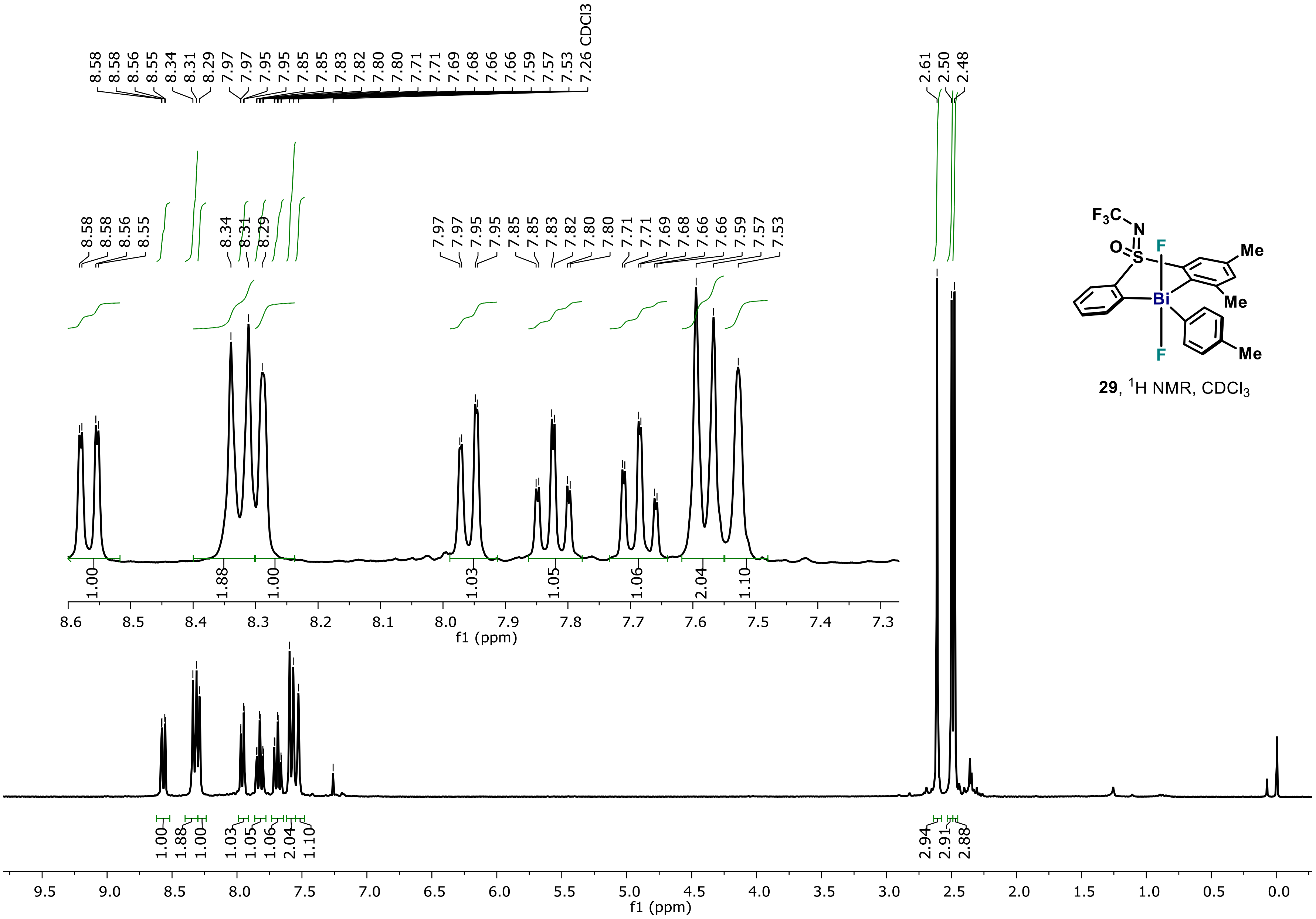

29, ^{1}H NMR, $CDCl_3$

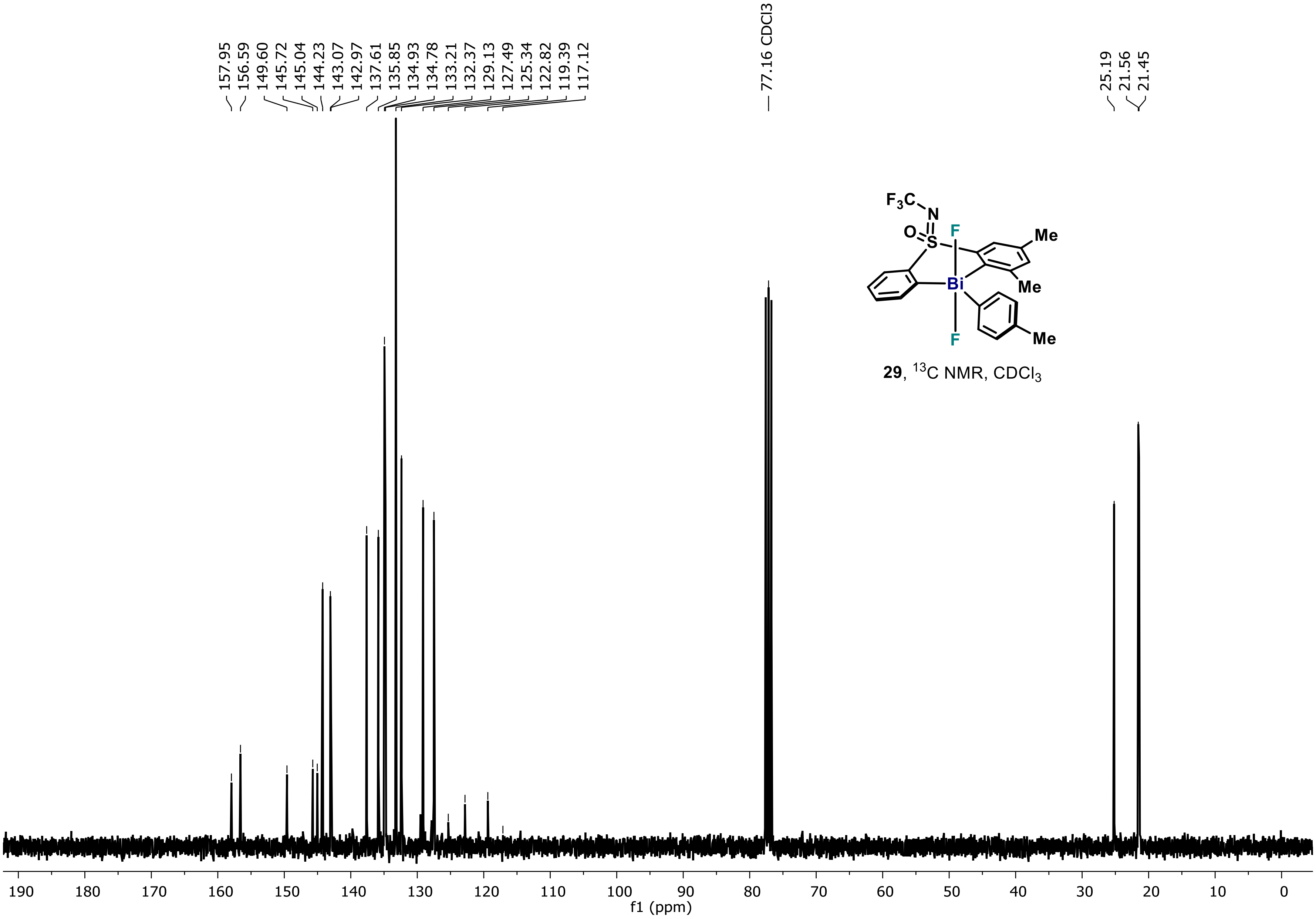

29, ^{13}C NMR, $CDCl_3$

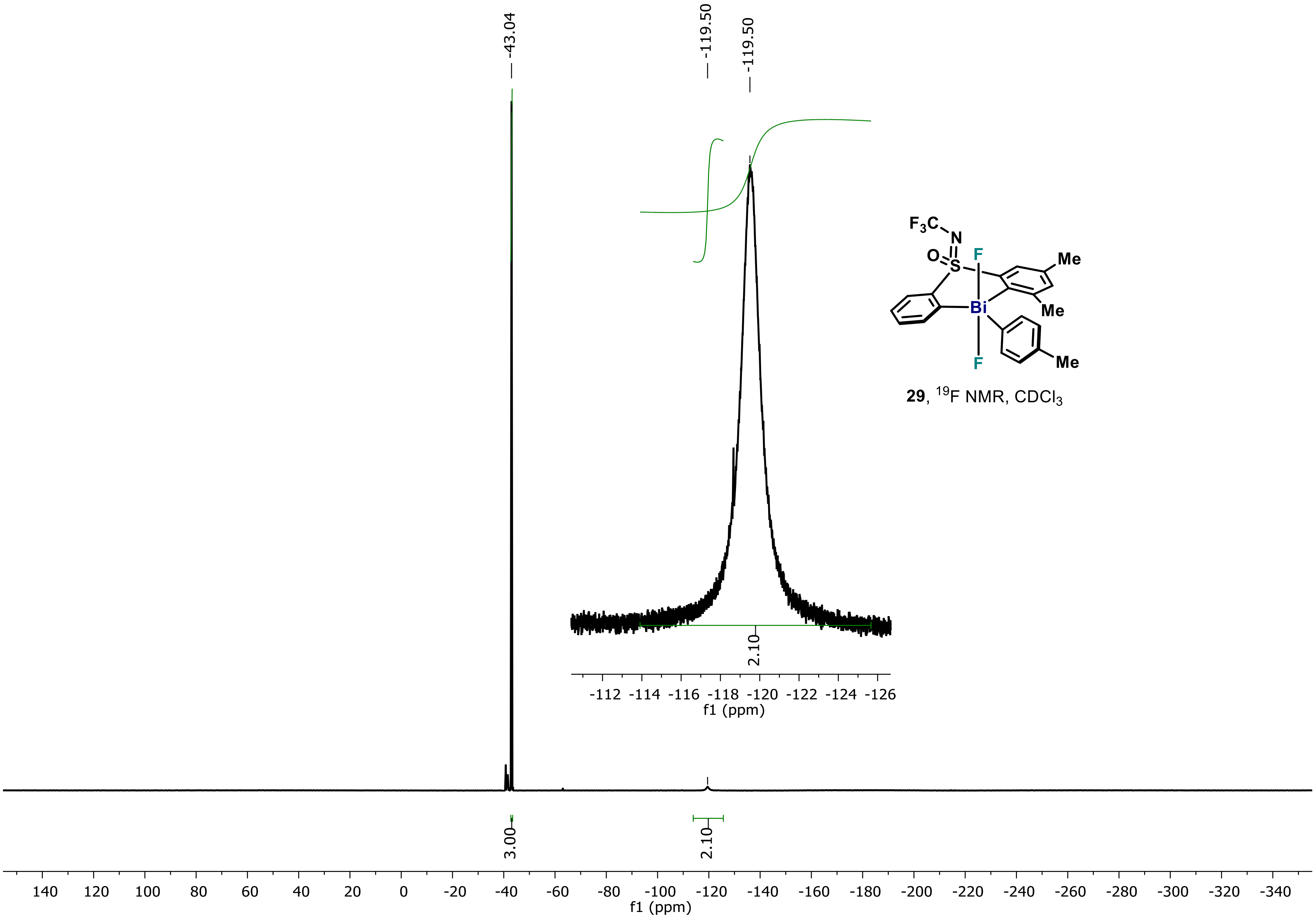

29, ^{19}F NMR, $CDCl_3$

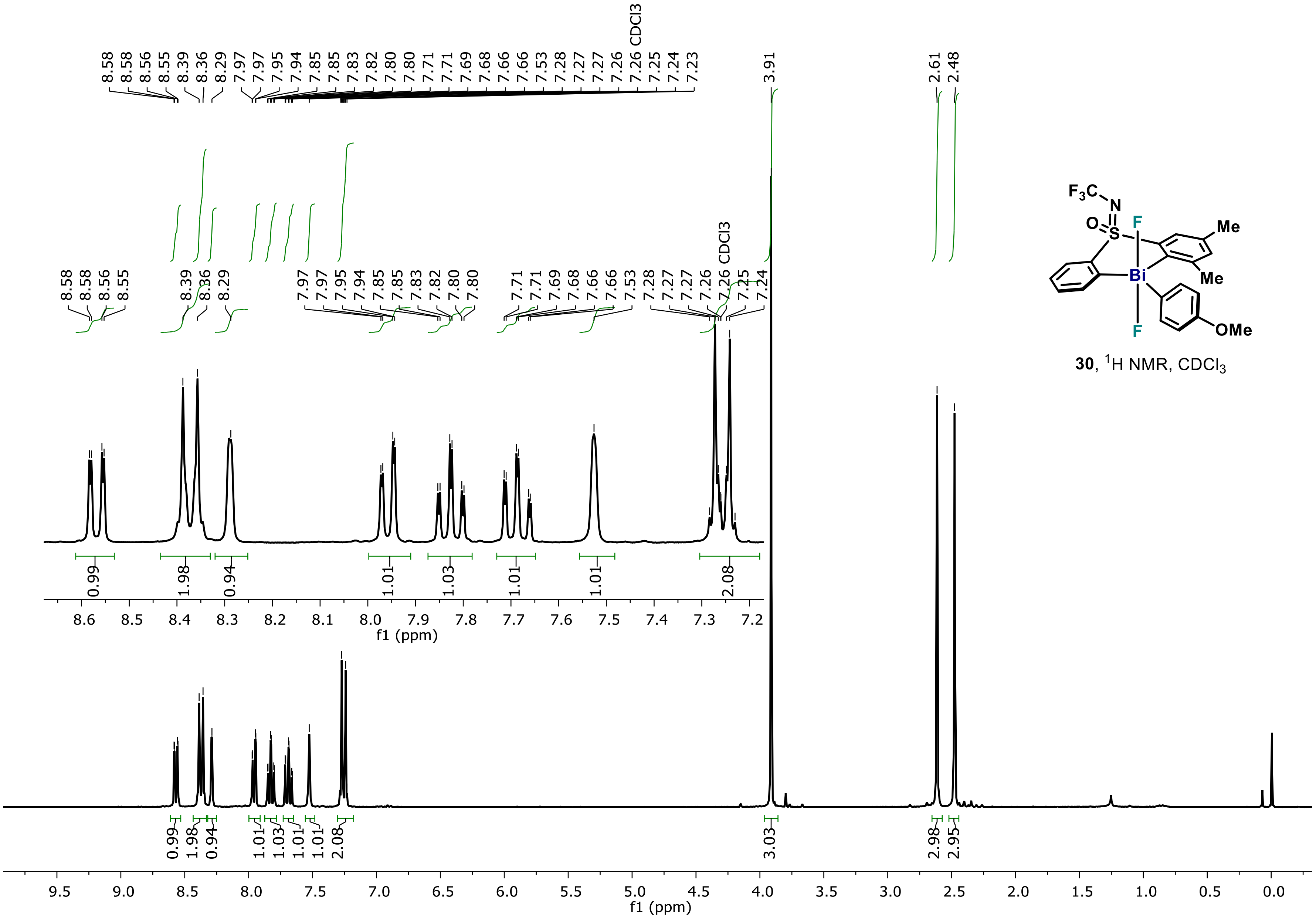
F3C
N
O
S
F
Me
Bi
Me
F
OMe
30, 1H NMR, CDCl3
CDCl3
3.91
2.61
2.48
0.99
1.98
0.94
1.01
1.03
1.01
1.01
2.08
3.03
2.98
2.95
f1 (ppm)

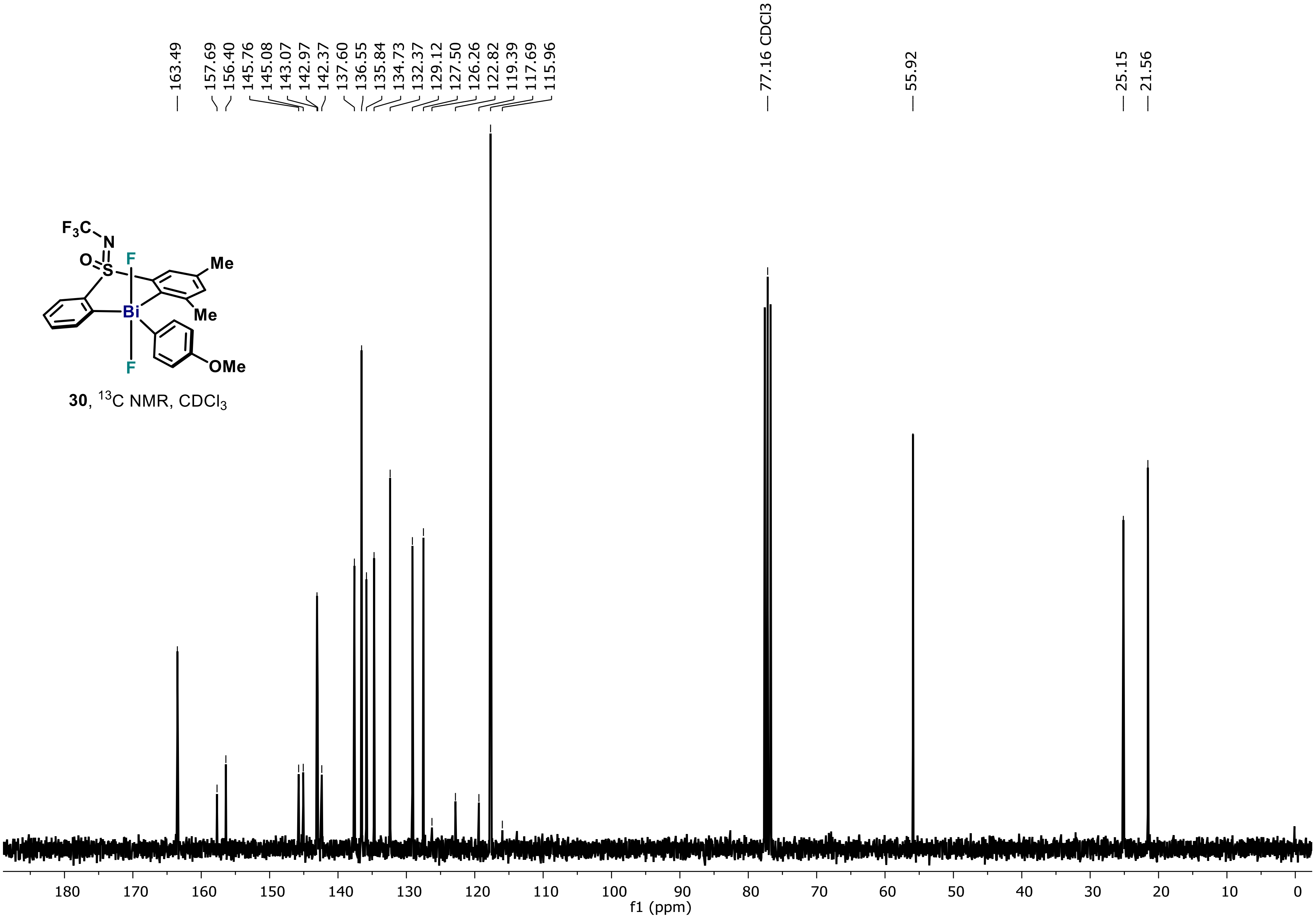

163.49
157.69
156.40
145.76
145.08
143.07
142.97
142.37
137.60
136.55
135.84
134.73
132.37
129.12
127.50
126.26
122.82
119.39
117.69
115.96
77.16 CDCl3
55.92
25.15
21.56
F3C
N
O
S
F
Me
Me
Bi
F
OMe
30, 13C NMR, CDCl3
180
170
160
150
140
130
120
110
100
90
80
70
60
50
40
30
20
10
0
f1 (ppm)

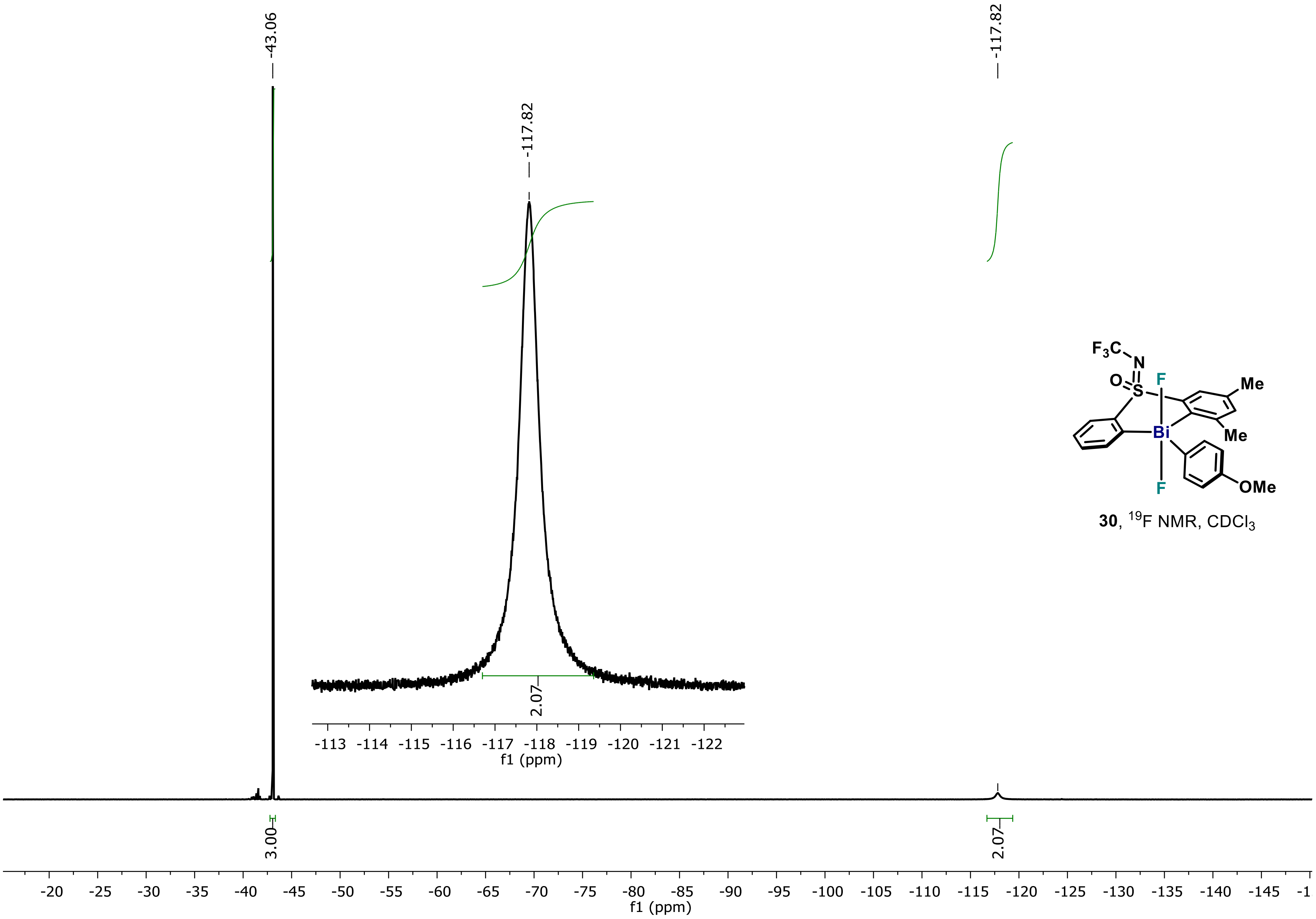

30, ^{19}F NMR, $CDCl_3$

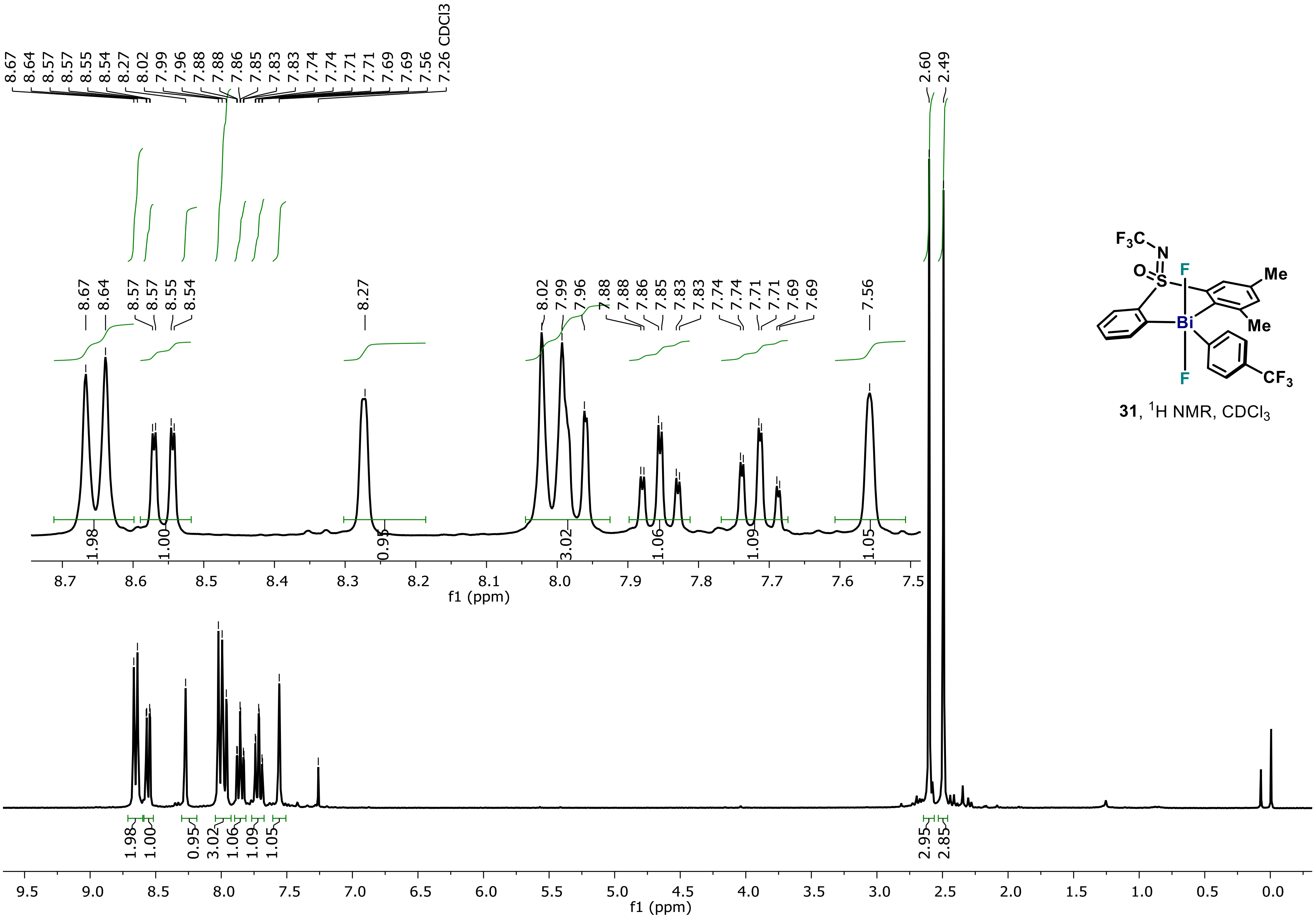

31, ^{1}H NMR, $CDCl_3$

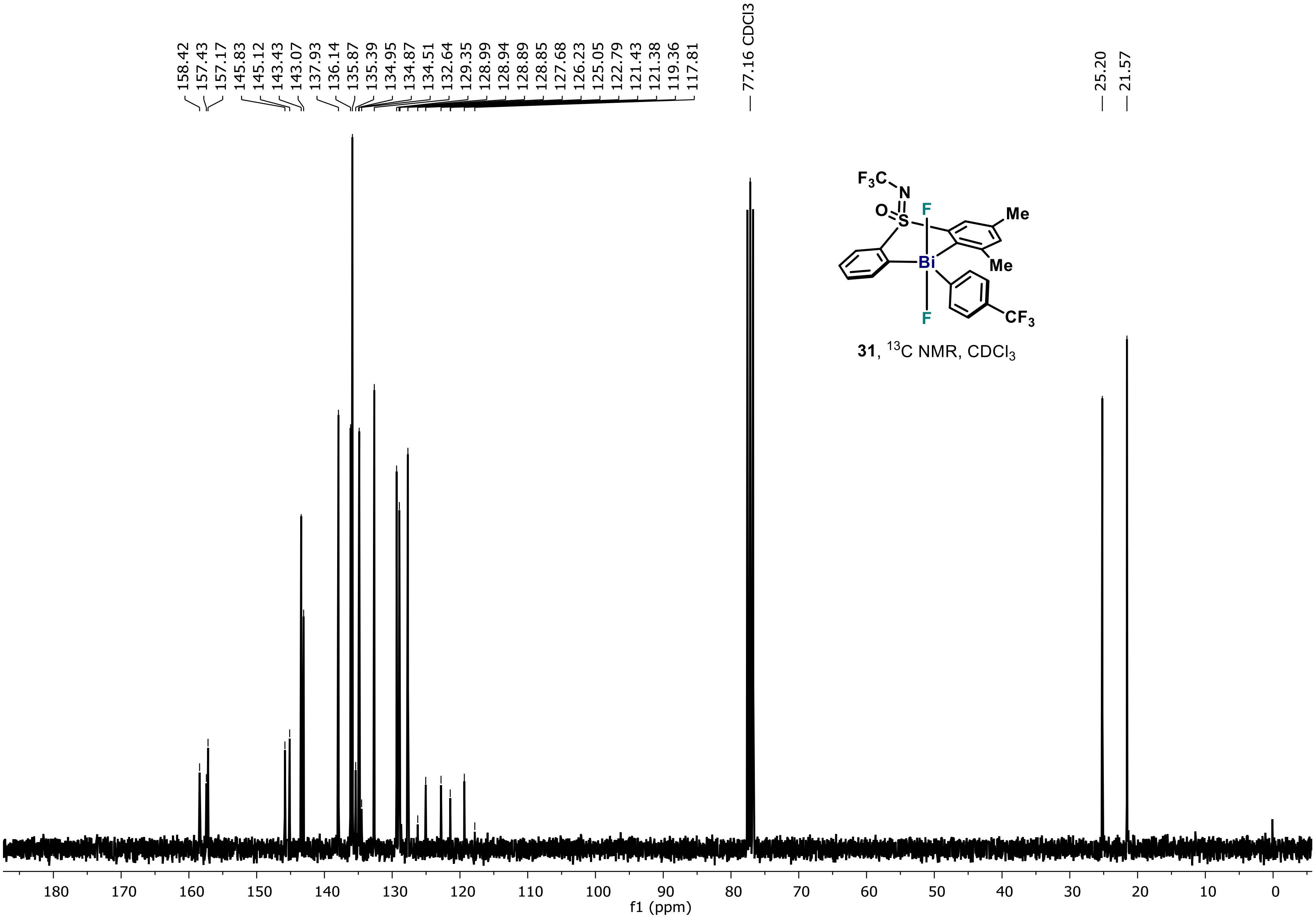
158.42
157.43
157.17
145.83
145.12
143.43
143.07
137.93
136.14
135.87
135.39
134.95
134.87
134.51
132.64
129.35
128.99
128.94
128.89
128.85
127.68
126.23
125.05
122.79
121.43
121.38
119.36
117.81
77.16 CDCl3
25.20
21.57
F3C
N
O
S
F
Me
Bi
Me
F
CF3
31, 13C NMR, CDCl3
180 170 160 150 140 130 120 110 100 90 80 70 60 50 40 30 20 10 0
f1 (ppm)

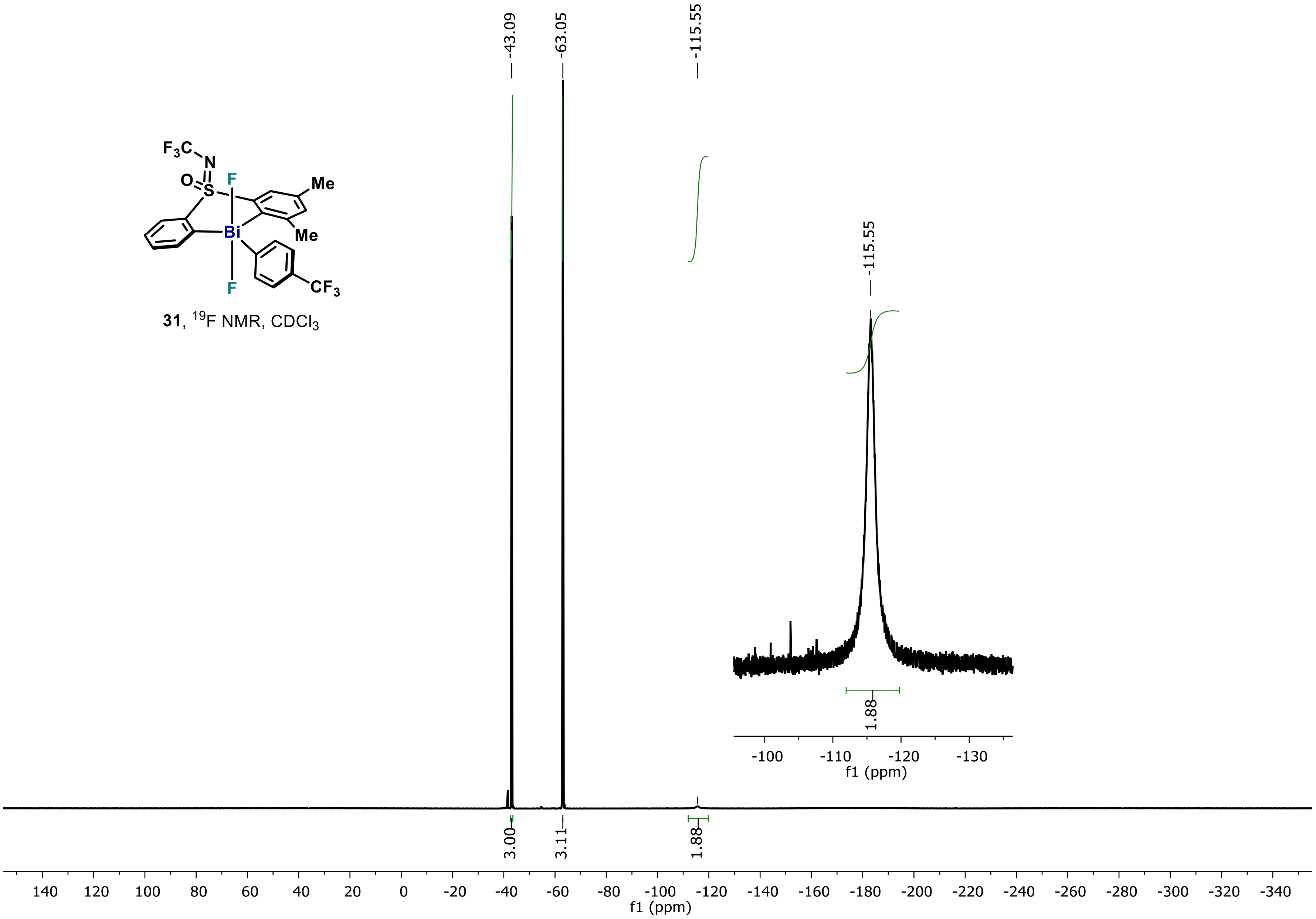
F3C
N
O
S
F
Me
Bi
Me
F
CF3
31, 19F NMR, CDCl3
-43.09
-63.05
-115.55
-115.55
1.88
-100
-110
-120
-130
f1 (ppm)
3.00
3.11
1.88
140
120
100
80
60
40
20
0
-20
-40
-60
-80
-100
-120
-140
-160
-180
-200
-220
-240
-260
-280
-300
-320
-340
f1 (ppm)

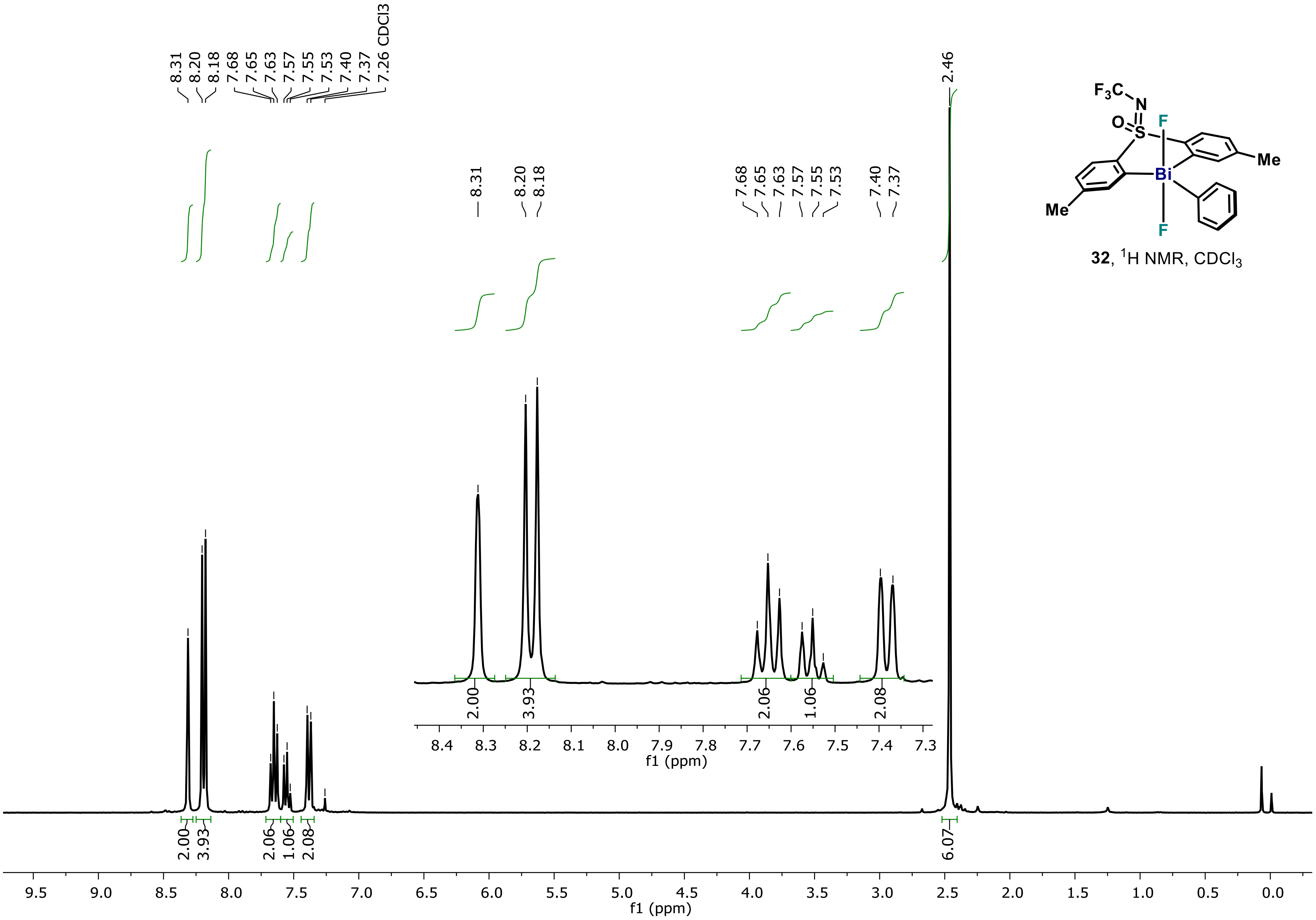
8.31
8.20
8.18
7.68
7.65
7.63
7.57
7.55
7.53
7.40
7.37
7.26 CDCl3
2.46
2.00
3.93
2.06
1.06
2.08
6.07
f1 (ppm)
F3C
N
O
S
F
Bi
Me
Me
F
32, 1H NMR, CDCl3

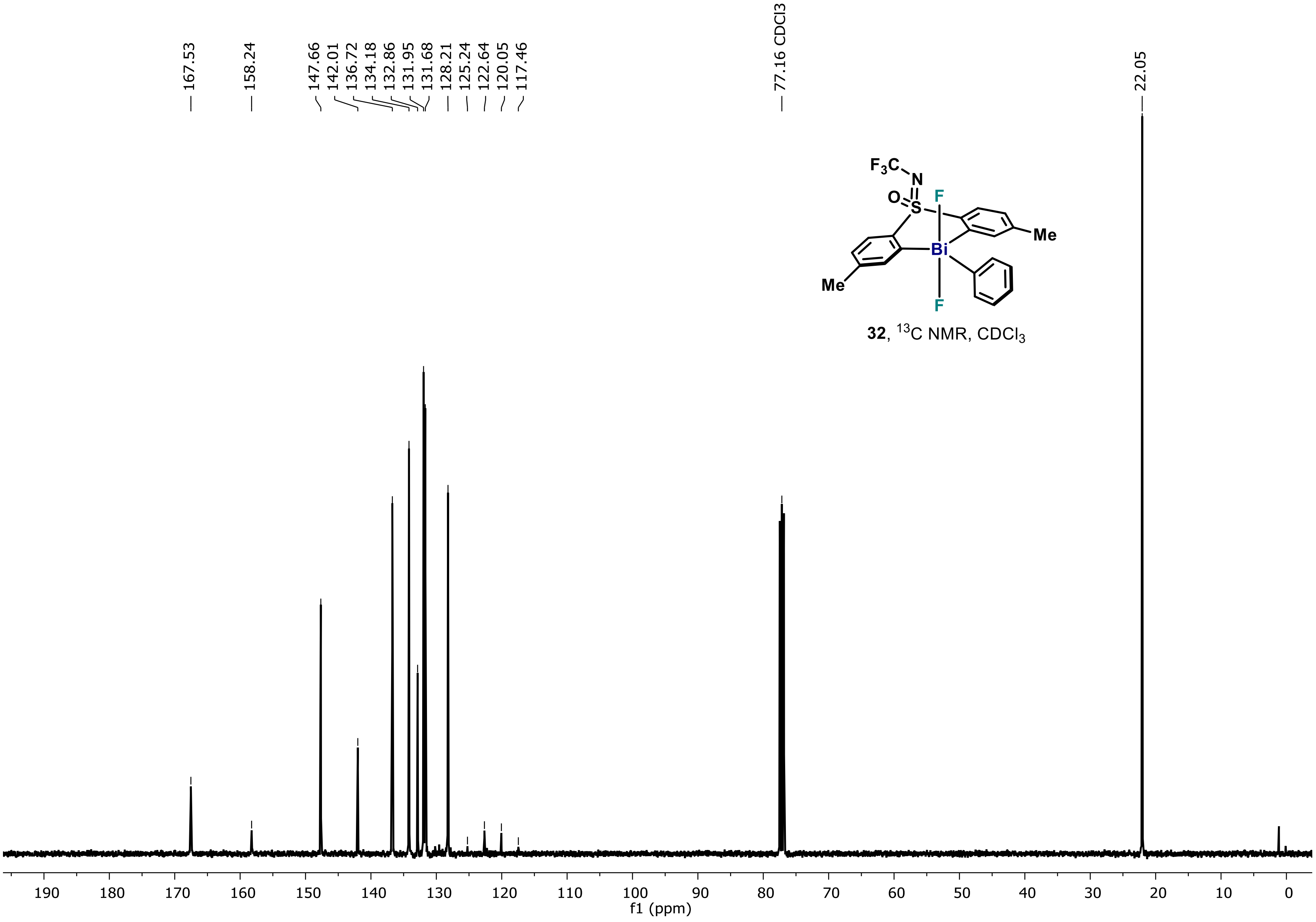

167.53
158.24
147.66
142.01
136.72
134.18
132.86
131.95
131.68
128.21
125.24
122.64
120.05
117.46
77.16 CDCl3
22.05
F3C
N
O
S
F
Bi
F
Me
Me
32, 13C NMR, CDCl3
190 180 170 160 150 140 130 120 110 100 90 80 70 60 50 40 30 20 10 0
f1 (ppm)

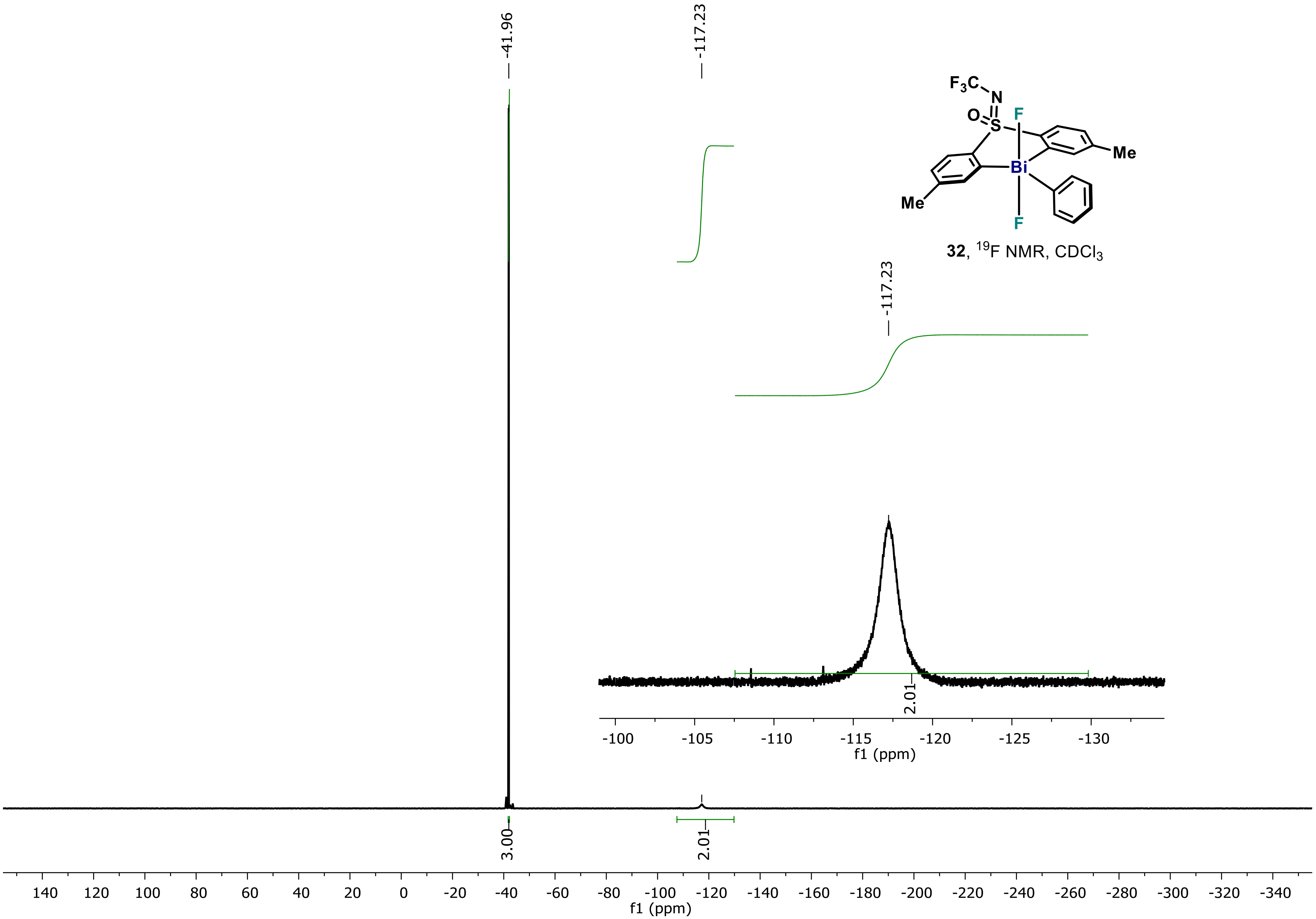

-41.96
-117.23
F3C
N
O
S
F
Bi
F
Me
Me
32, 19F NMR, CDCl3
-117.23
2.01
-100
-105
-110
-115
-120
-125
-130
f1 (ppm)
3.00
2.01
140
120
100
80
60
40
20
0
-20
-40
-60
-80
-100
-120
-140
-160
-180
-200
-220
-240
-260
-280
-300
-320
-340
f1 (ppm)

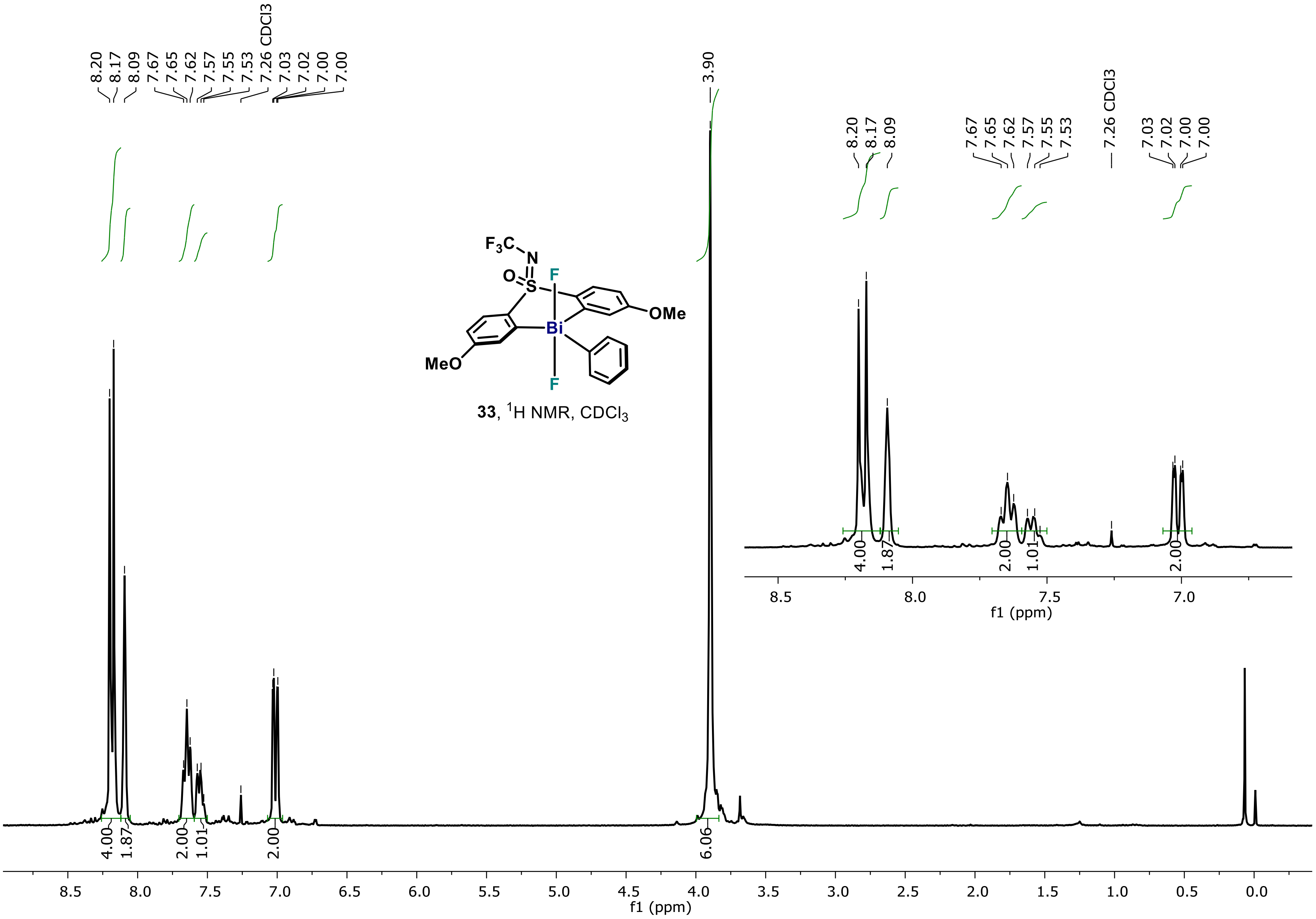
8.20
8.17
8.09
7.67
7.65
7.62
7.57
7.55
7.53
7.26 CDCl3
7.03
7.02
7.00
7.00
3.90
4.00
1.87
2.00
1.01
2.00
6.06
f1 (ppm)
F3C
N
O
S
F
Bi
F
OMe
MeO
33, 1H NMR, CDCl3

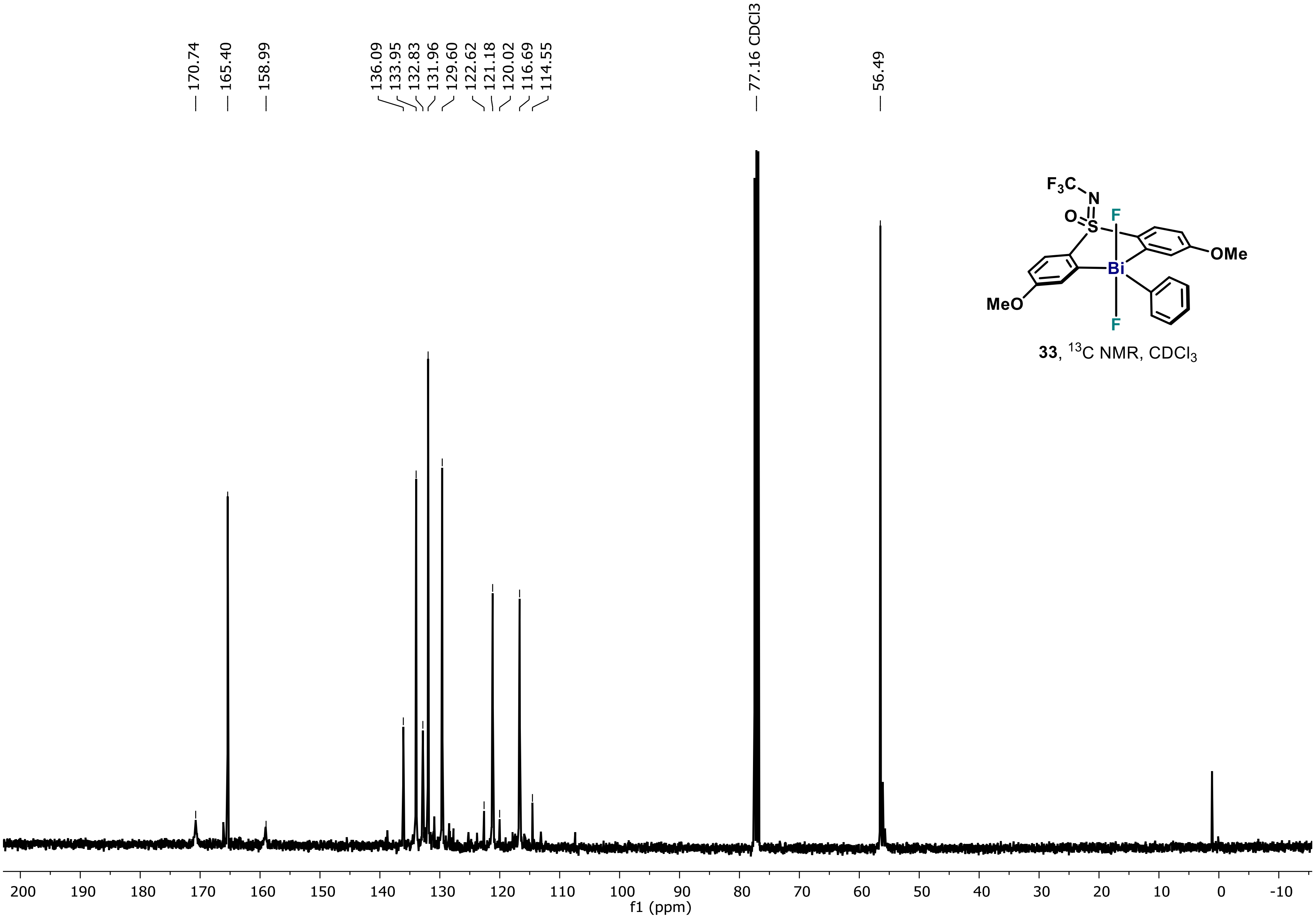

33, ^{13}C NMR, $CDCl_3$

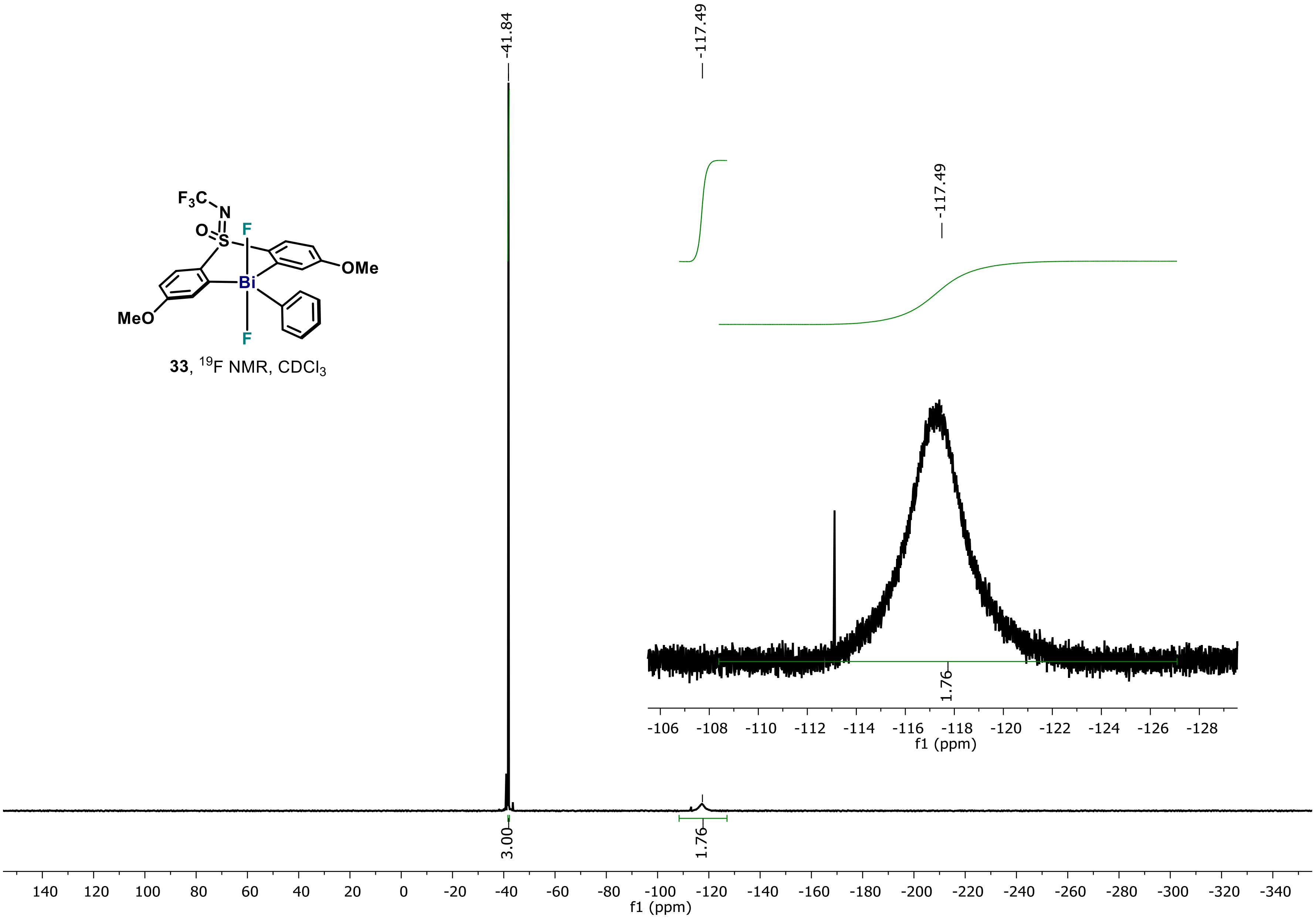
33, ^{19}F NMR, CDCl$_3$
-41.84
-117.49
-117.49
3.00
1.76
1.76
f1 (ppm)
f1 (ppm)

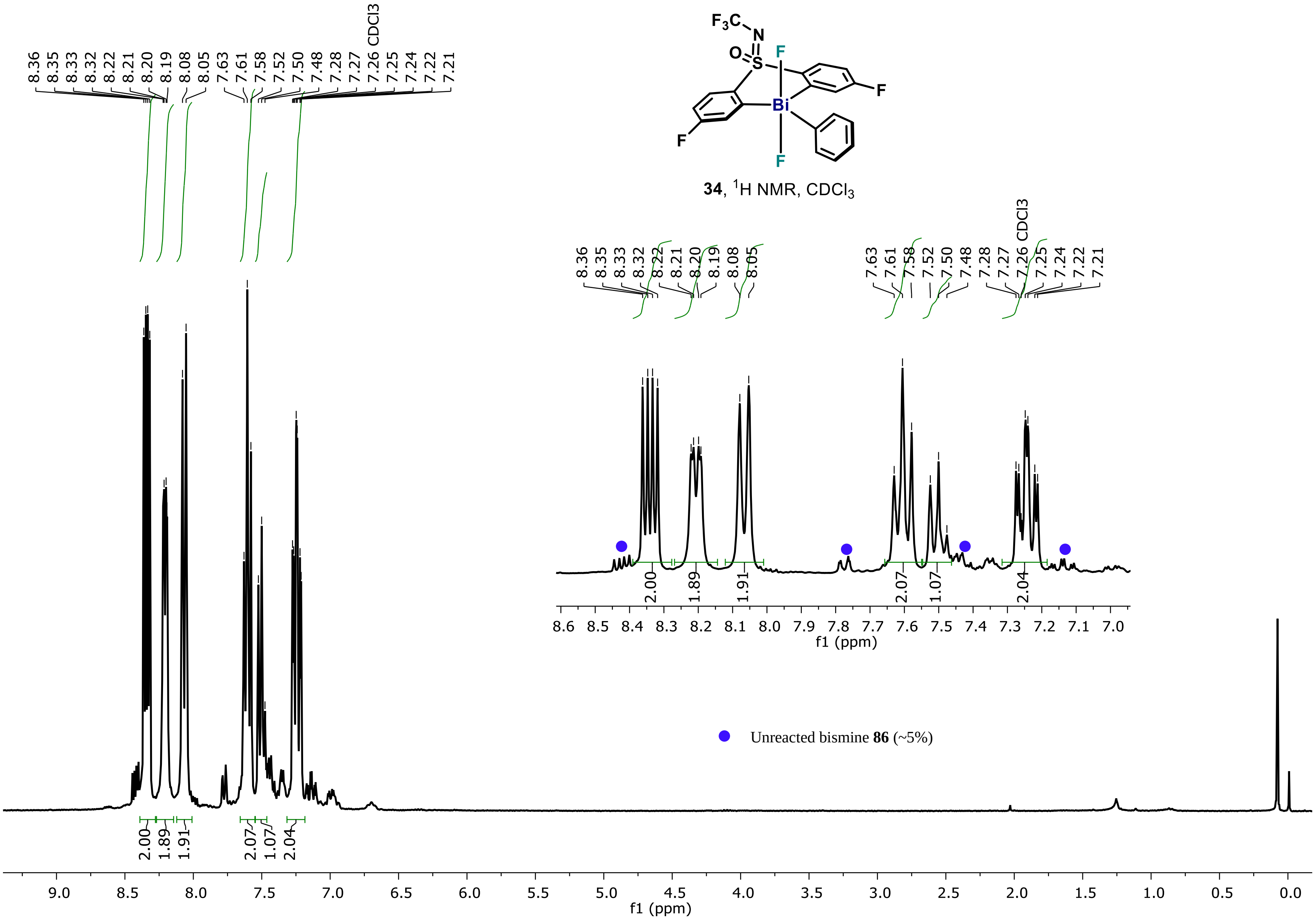

34, ^{1}H NMR, $CDCl_3$
Unreacted bismine 86 (~5%)
8.36 8.35 8.33 8.32 8.22 8.21 8.20 8.19 8.08 8.05 7.63 7.61 7.58 7.52 7.50 7.48 7.28 7.27 7.26 CDCl3 7.25 7.24 7.22 7.21
2.00 1.89 1.91 2.07 1.07 2.04
f1 (ppm)

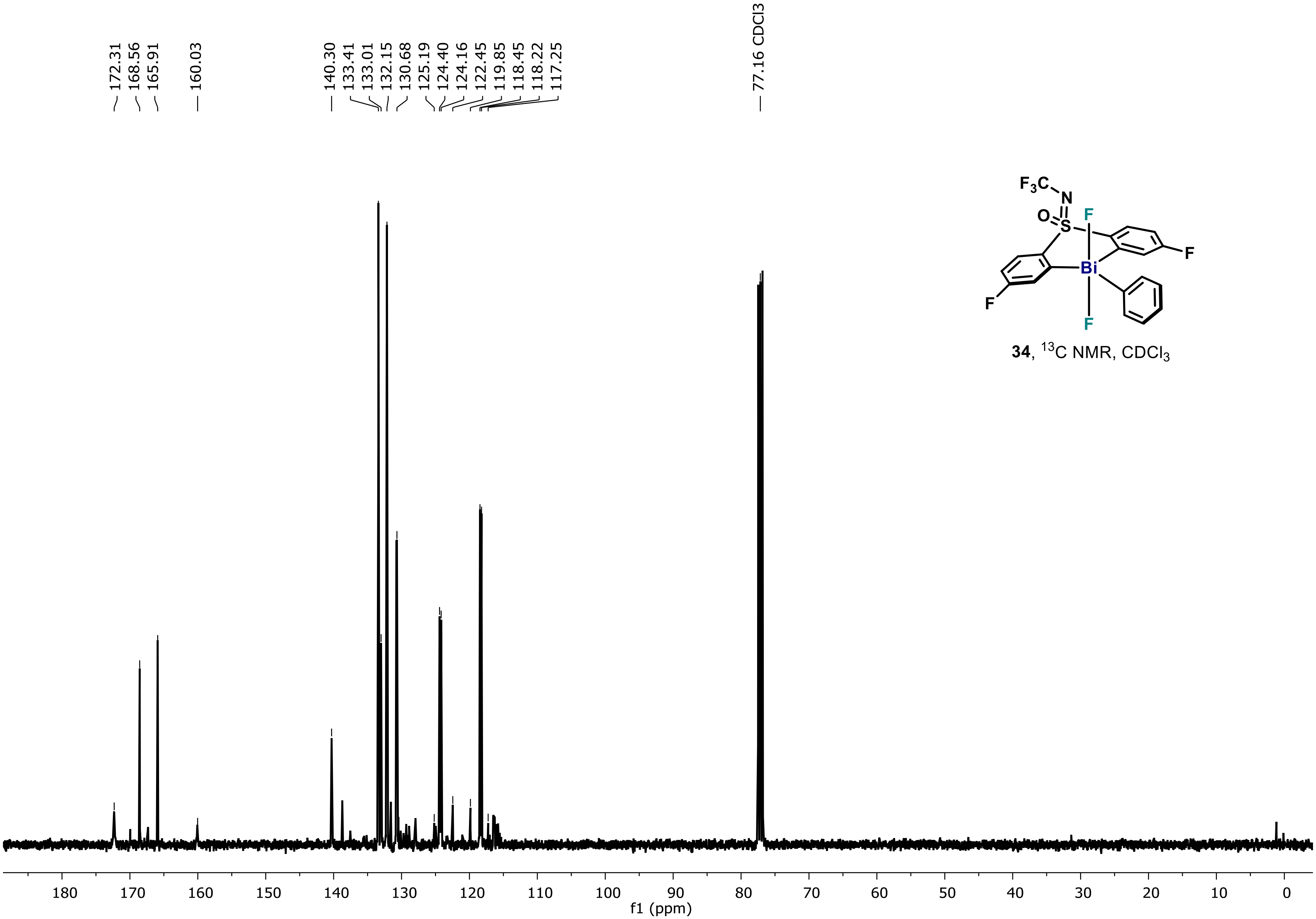

34, ^{13}C NMR, $CDCl_3$

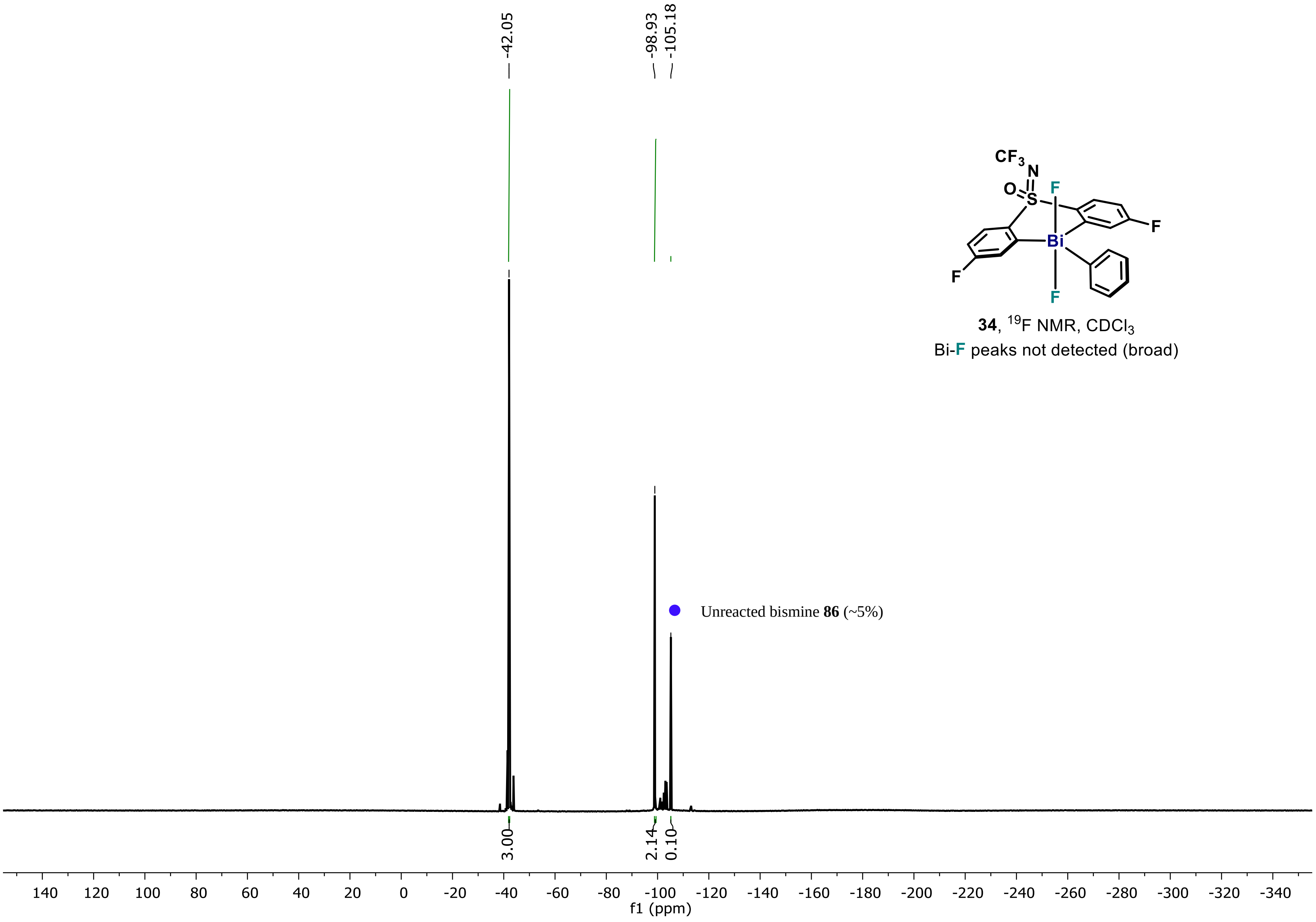

-42.05
-98.93
-105.18
CF3
N
O
S
F
Bi
F
F
F
34, 19F NMR, CDCl3
Bi-F peaks not detected (broad)
Unreacted bismine 86 (~5%)
3.00
2.14
0.10
140 120 100 80 60 40 20 0 -20 -40 -60 -80 -100 -120 -140 -160 -180 -200 -220 -240 -260 -280 -300 -320 -340
f1 (ppm)

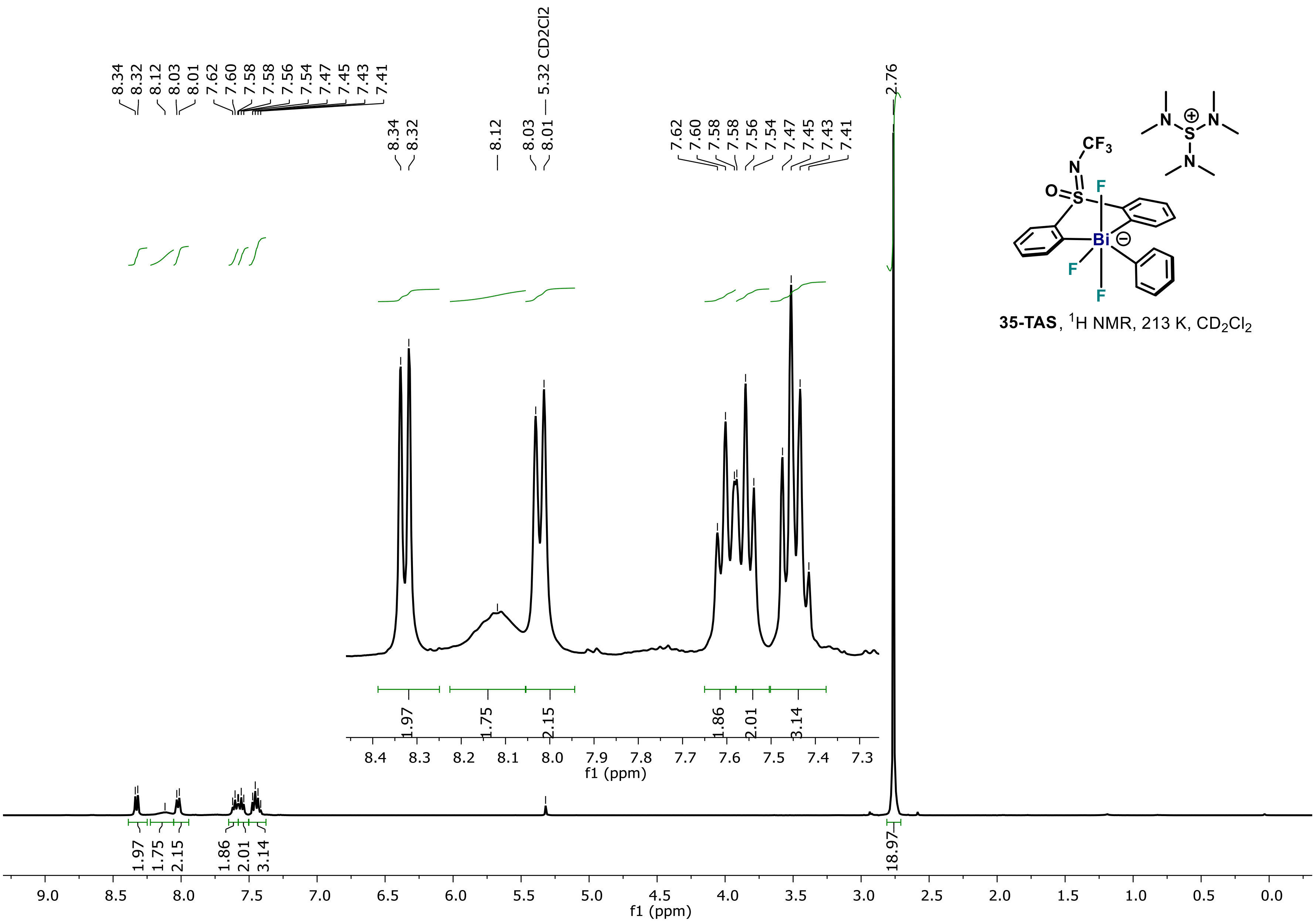

35-TAS, ^{1}H NMR, 213 K, CD_2Cl_2

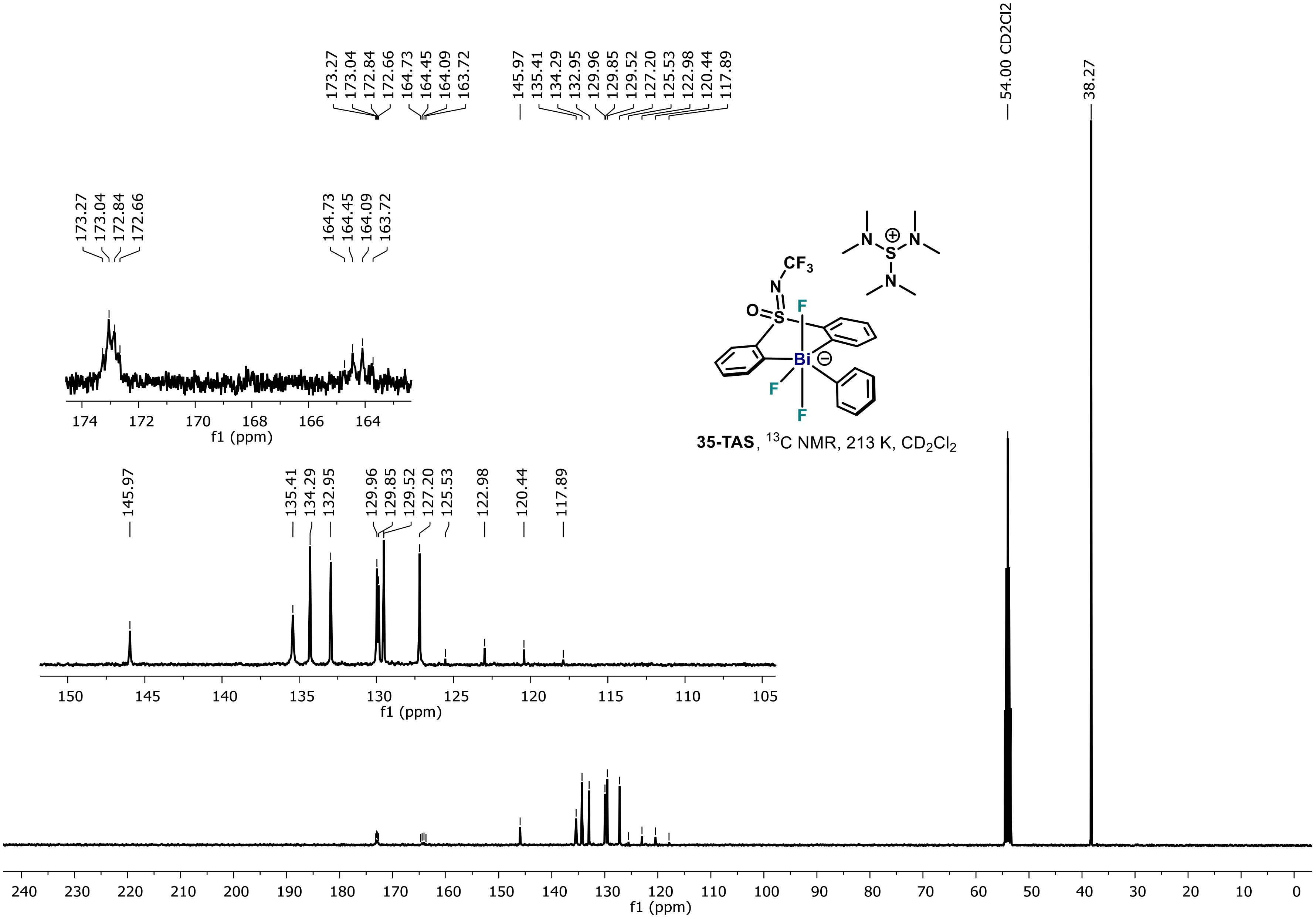

173.27
173.04
172.84
172.66
164.73
164.45
164.09
163.72
145.97
135.41
134.29
132.95
129.96
129.85
129.52
127.20
125.53
122.98
120.44
117.89
54.00 CD2Cl2
38.27
174
172
170
168
166
164
f1 (ppm)
CF3
N
O
S
F
Bi
F
F
N
S
N
N
35-TAS, 13C NMR, 213 K, CD2Cl2
150
145
140
135
130
125
120
115
110
105
f1 (ppm)
240
230
220
210
200
190
180
170
160
150
140
130
120
110
100
90
80
70
60
50
40
30
20
10
0
f1 (ppm)

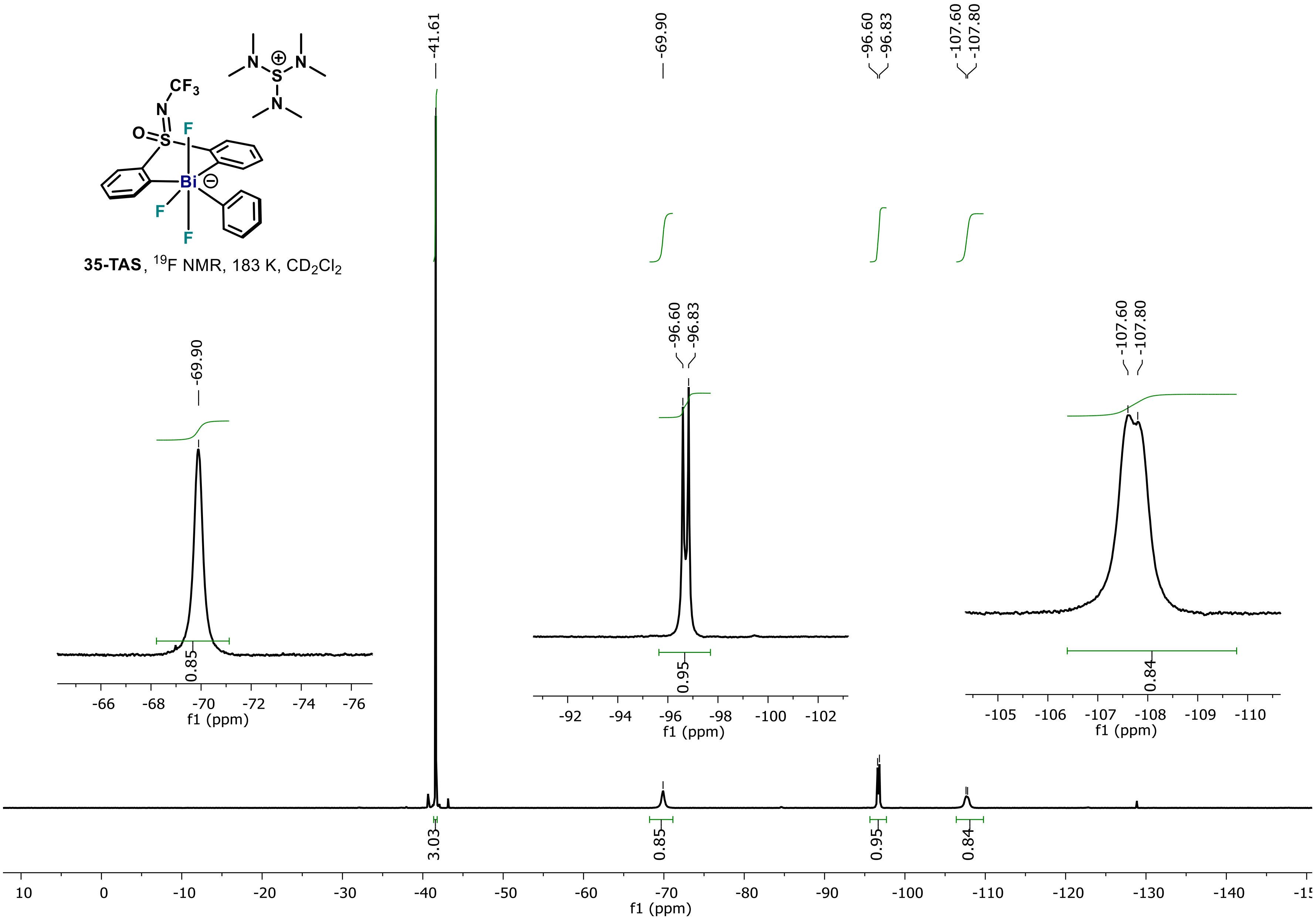
35-TAS, ¹⁹F NMR, 183 K, CD₂Cl₂
-41.61
-69.90
-96.60
-96.83
-107.60
-107.80
3.03
0.85
0.95
0.84
f1 (ppm)

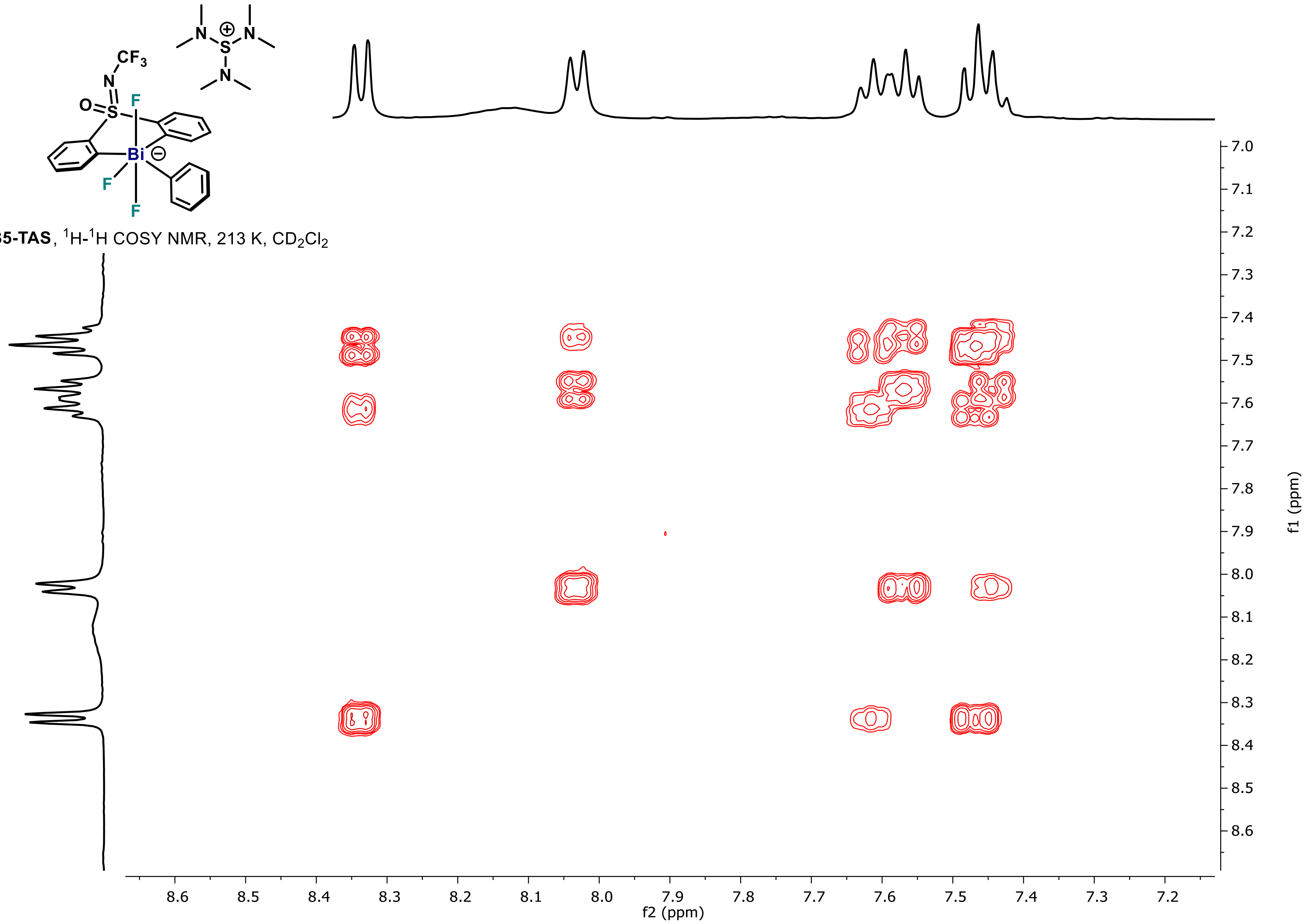

35-TAS, ^{1}H-^{1}H COSY NMR, 213 K, CD_2Cl_2

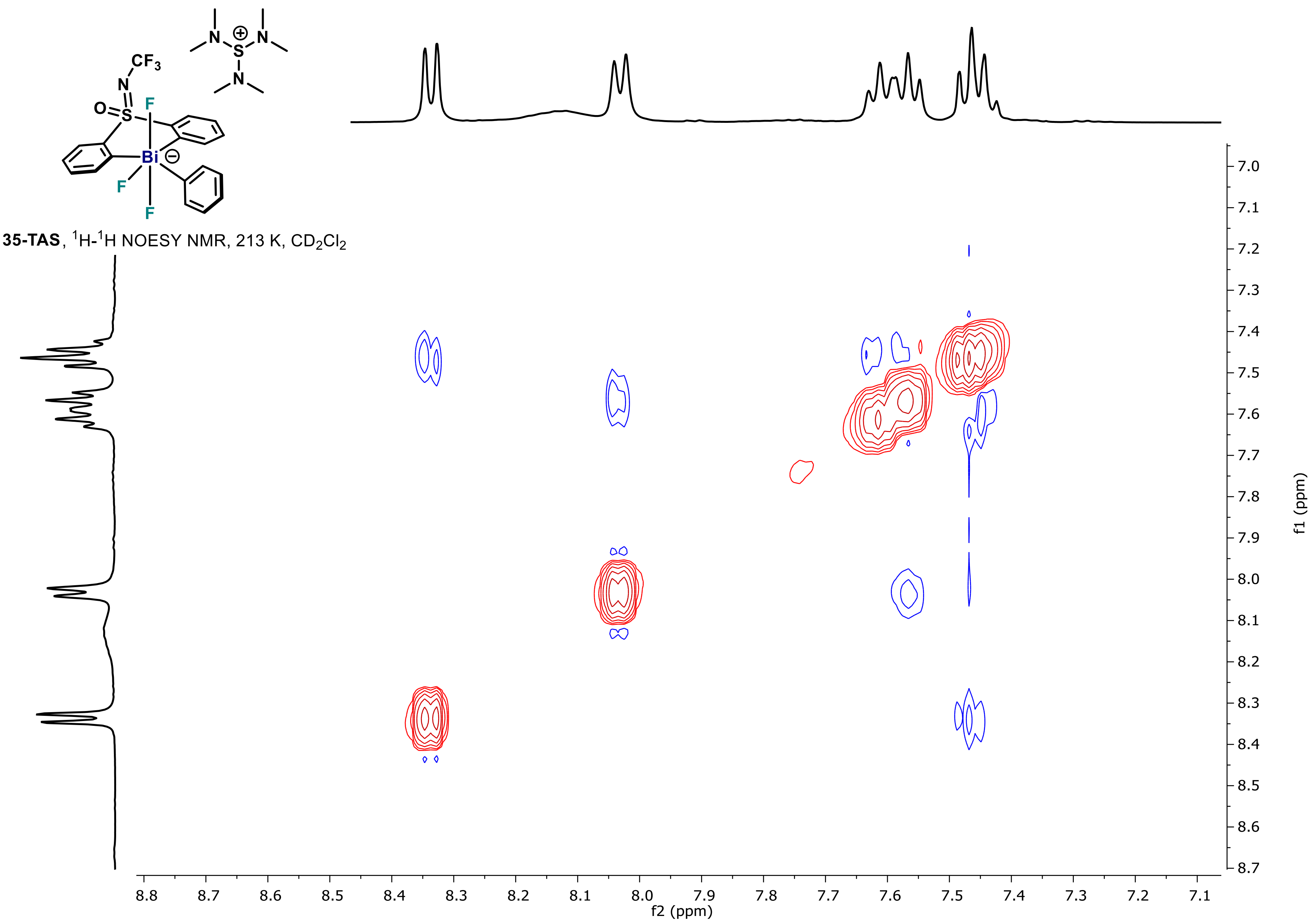

35-TAS, ^{1}H-^{1}H NOESY NMR, 213 K, CD_2Cl_2

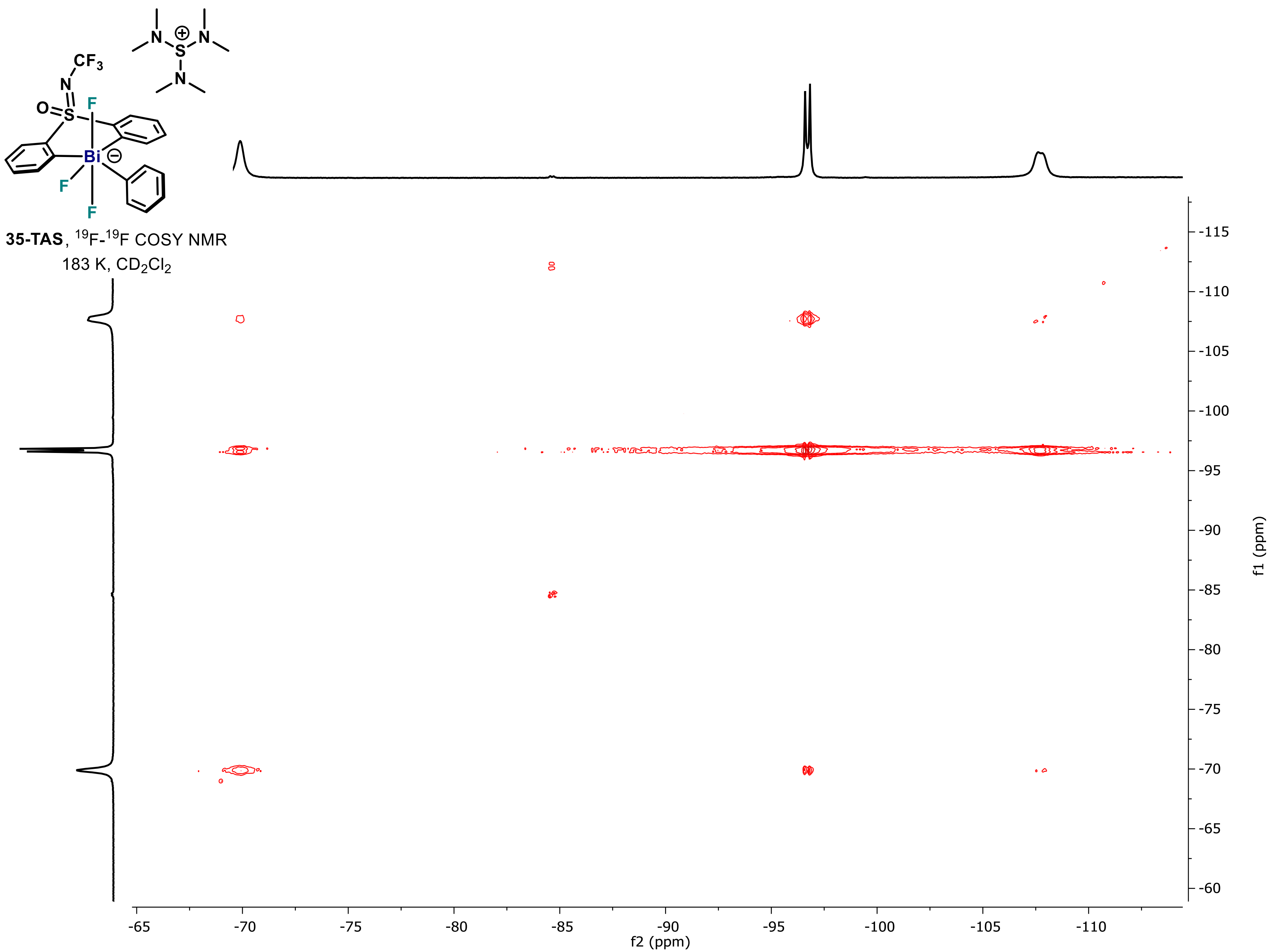

35-TAS, ^{19}F-^{19}F COSY NMR
183 K, CD_2Cl_2

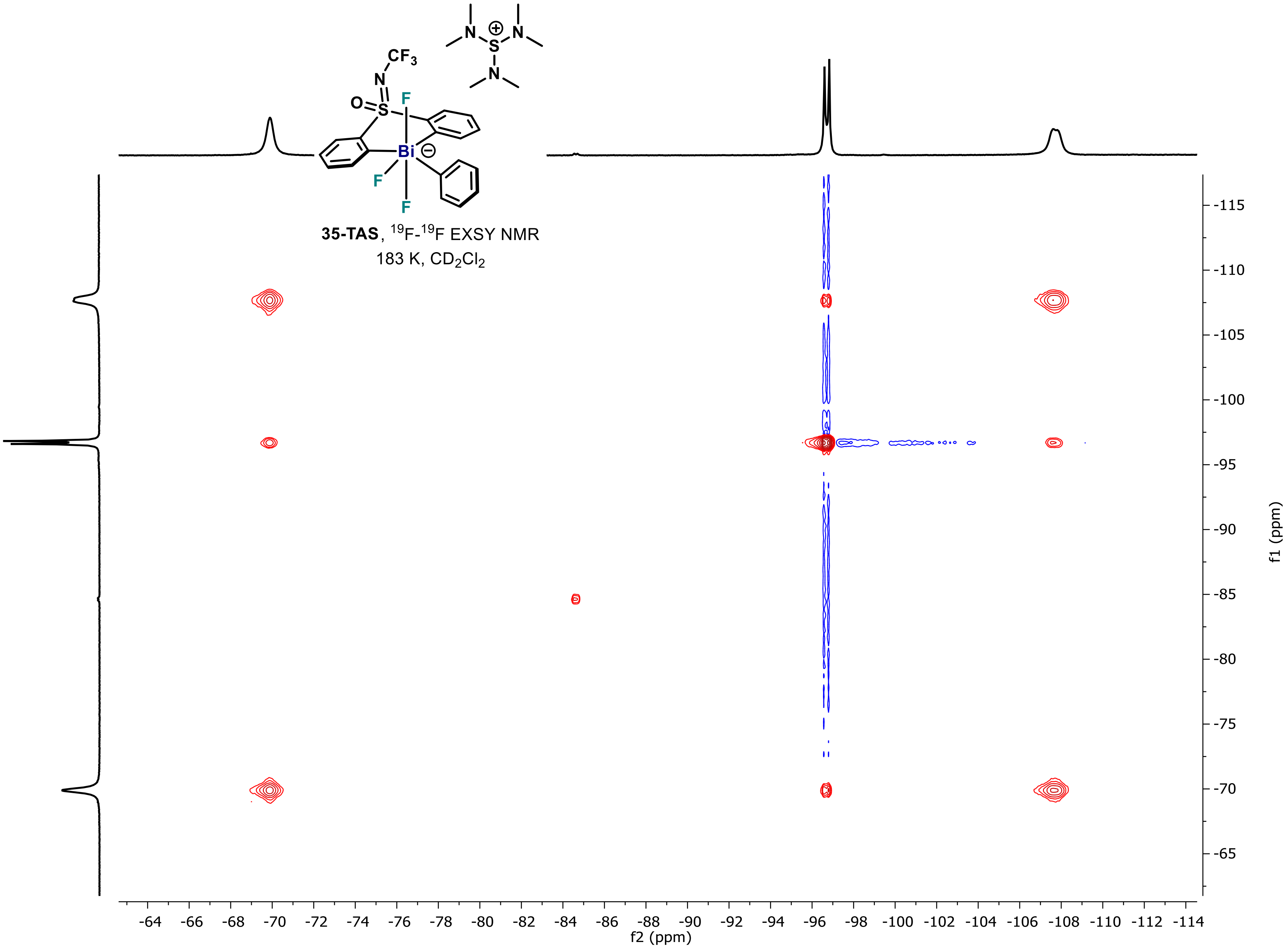

CF3
N
O
S
F
Bi
F
F
N
S
N
N
35-TAS, 19F-19F EXSY NMR
183 K, CD2Cl2
f2 (ppm)
f1 (ppm)

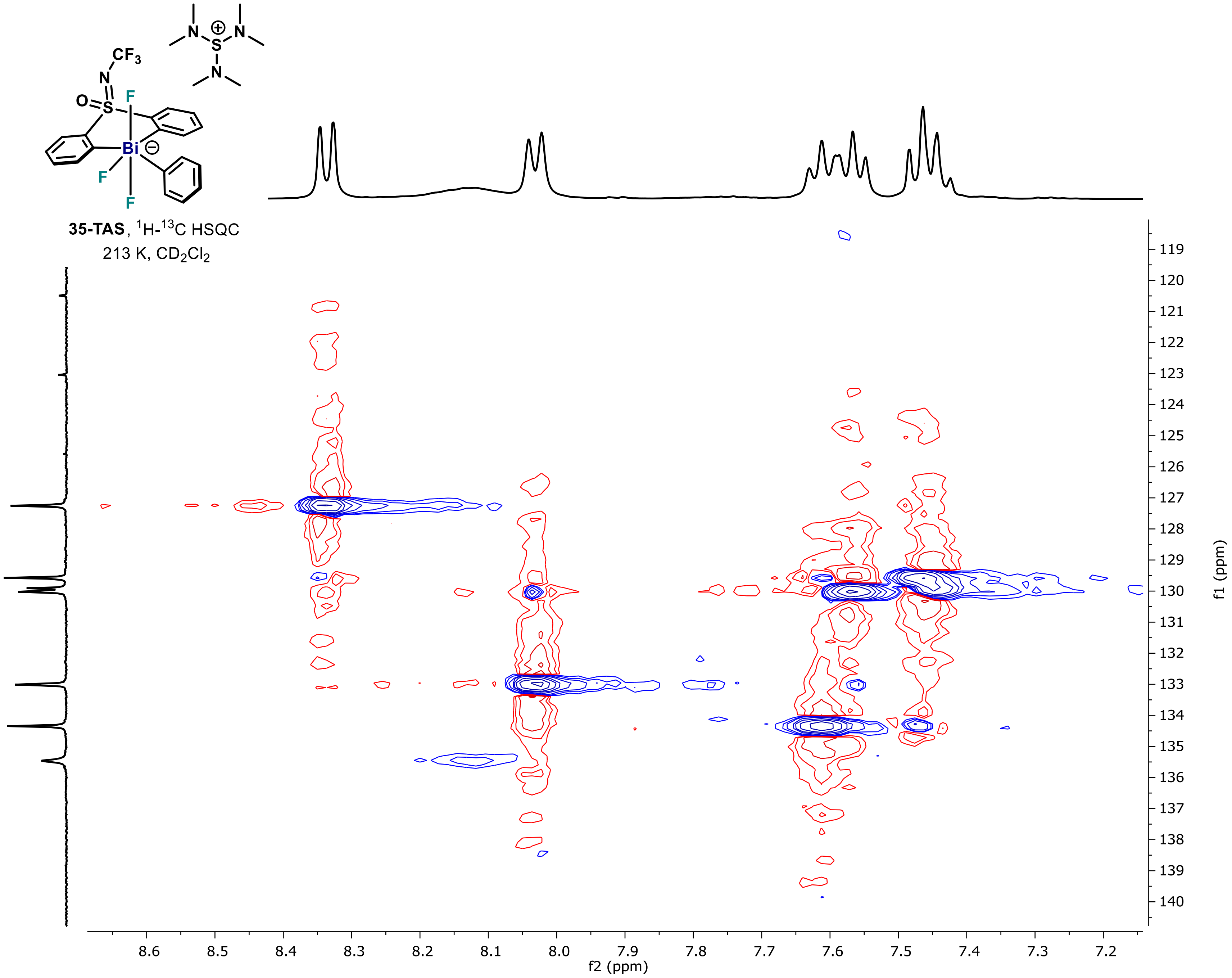
35-TAS, 1H-13C HSQC
213 K, CD2Cl2
f1 (ppm)
f2 (ppm)

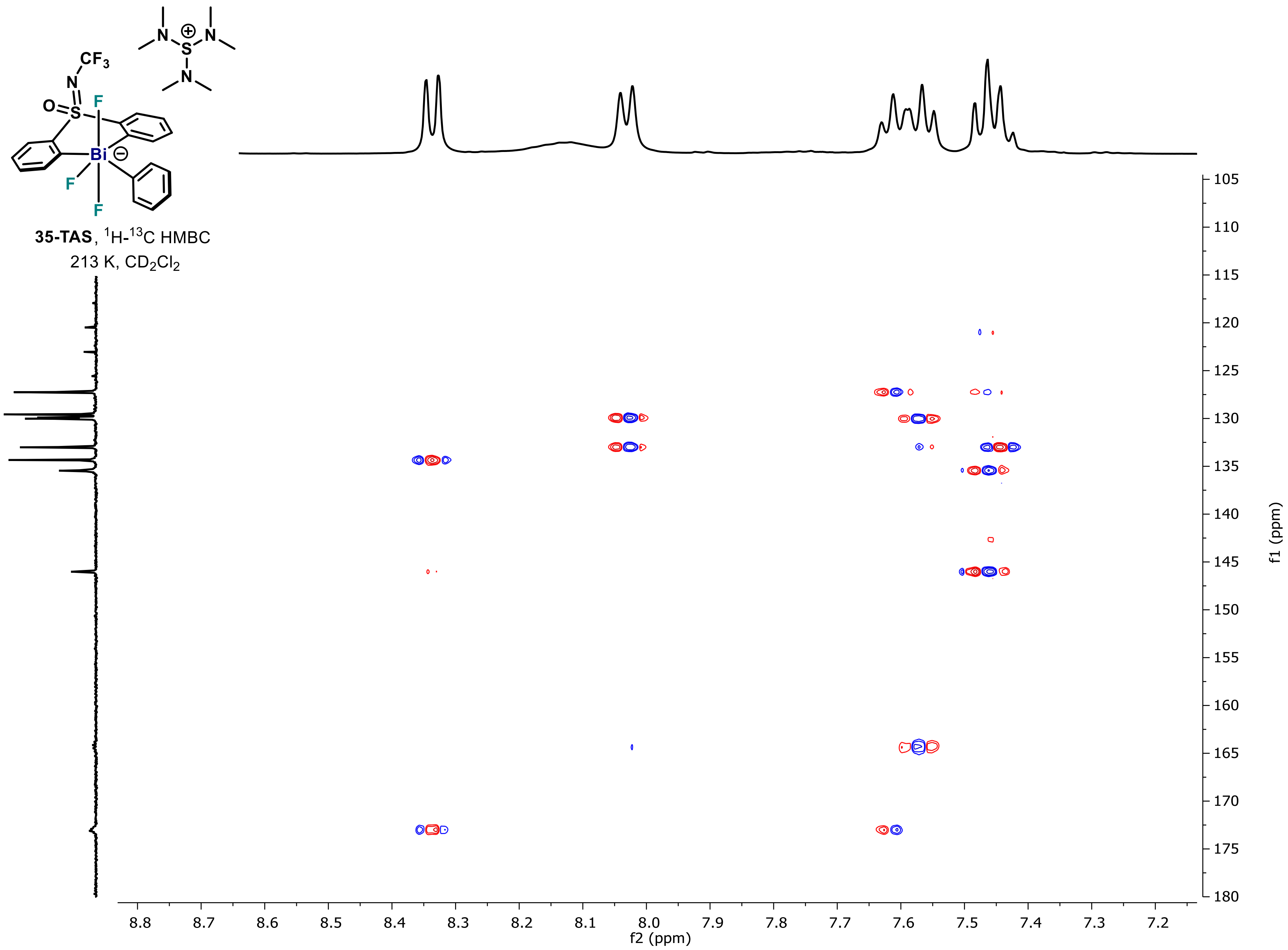
CF3
N
O
S
F
Bi
F
F
N
S
N
N
35-TAS, 1H-13C HMBC
213 K, CD2Cl2
f1 (ppm)
f2 (ppm)

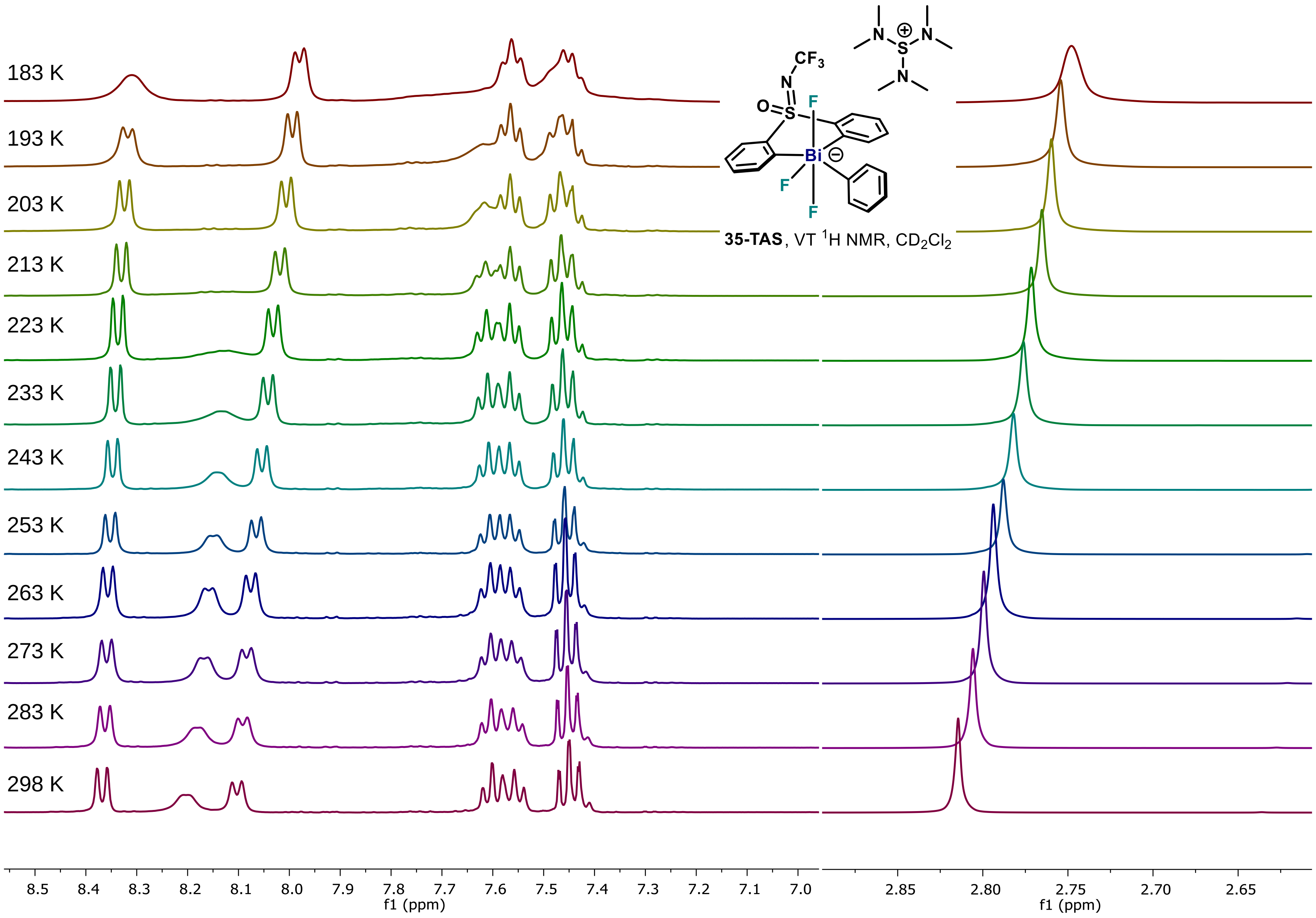
183 K
193 K
203 K
213 K
223 K
233 K
243 K
253 K
263 K
273 K
283 K
298 K
35-TAS, VT 1H NMR, CD2Cl2
8.5 8.4 8.3 8.2 8.1 8.0 7.9 7.8 7.7 7.6 7.5 7.4 7.3 7.2 7.1 7.0
f1 (ppm)
2.85 2.80 2.75 2.70 2.65
f1 (ppm)

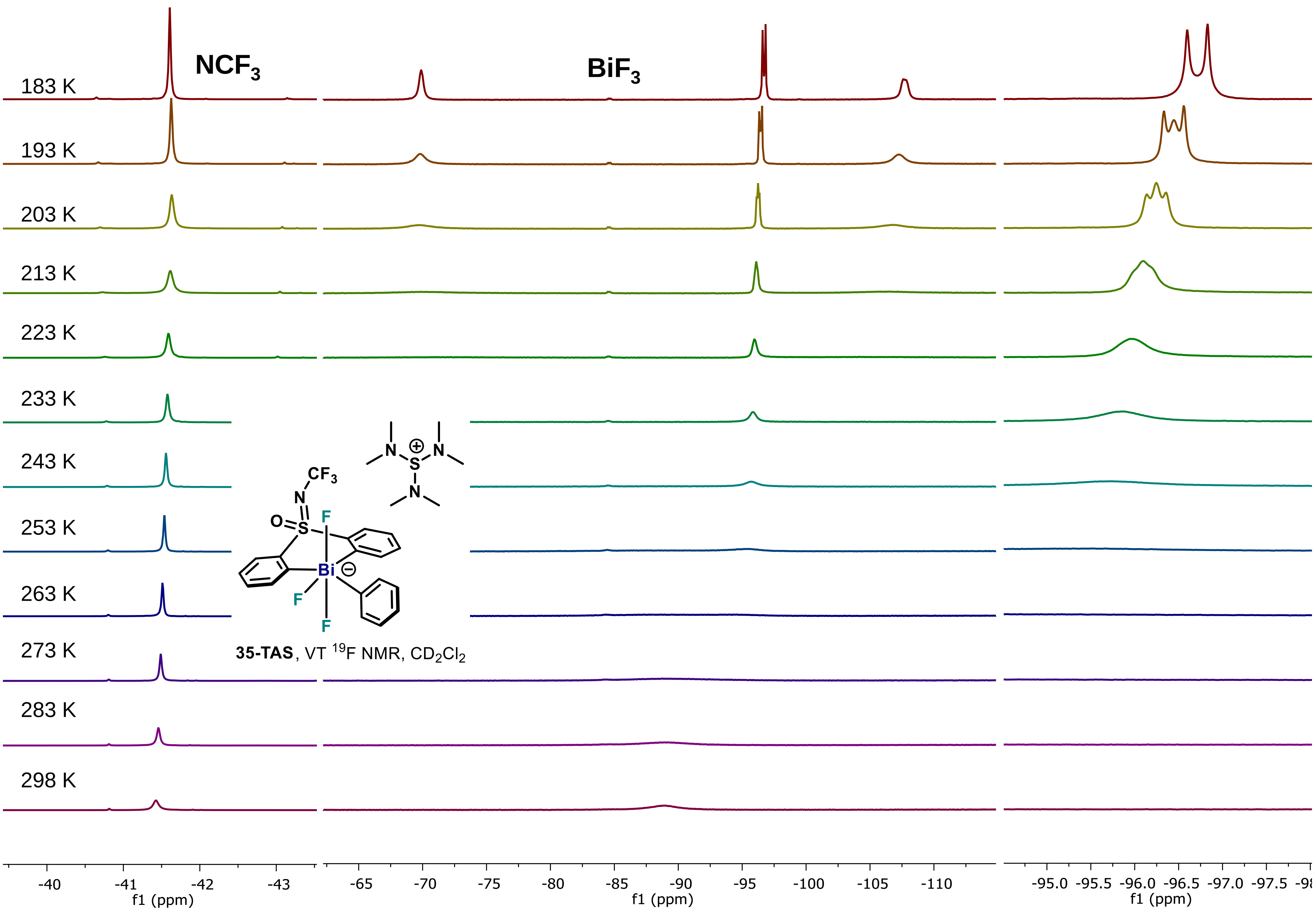
183 K
193 K
203 K
213 K
223 K
233 K
243 K
253 K
263 K
273 K
283 K
298 K
NCF3
BiF3
35-TAS, VT 19F NMR, CD2Cl2
-40 -41 -42 -43
f1 (ppm)
-65 -70 -75 -80 -85 -90 -95 -100 -105 -110
f1 (ppm)
-95.0 -95.5 -96.0 -96.5 -97.0 -97.5
f1 (ppm)

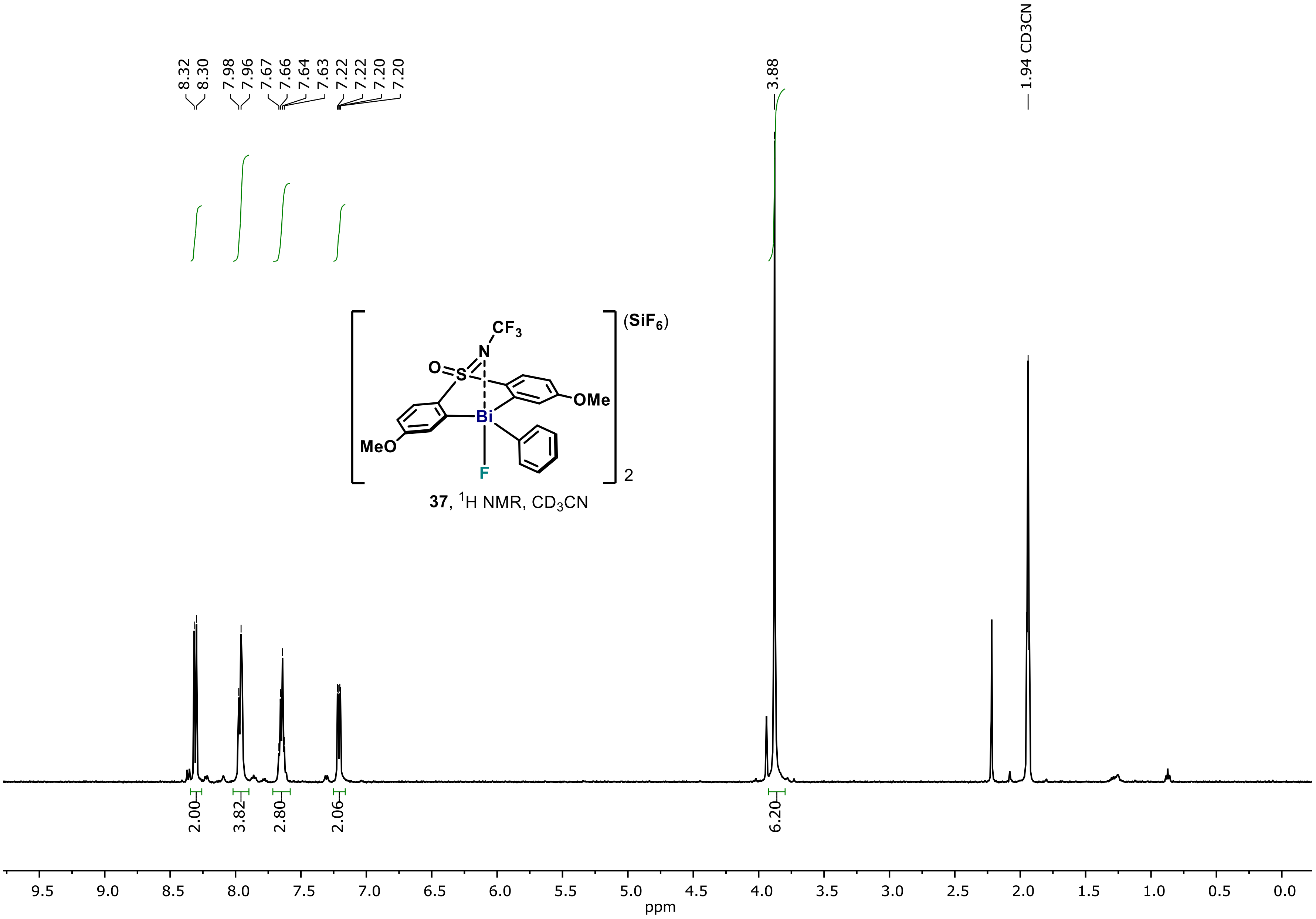

8.32
8.30
7.98
7.96
7.67
7.66
7.64
7.63
7.22
7.22
7.20
7.20
3.88
1.94 CD3CN
(SiF6)
CF3
N
O
S
OMe
MeO
Bi
F
2
37, 1H NMR, CD3CN
2.00
3.82
2.80
2.06
6.20
9.5
9.0
8.5
8.0
7.5
7.0
6.5
6.0
5.5
5.0
4.5
4.0
3.5
3.0
2.5
2.0
1.5
1.0
0.5
0.0
ppm

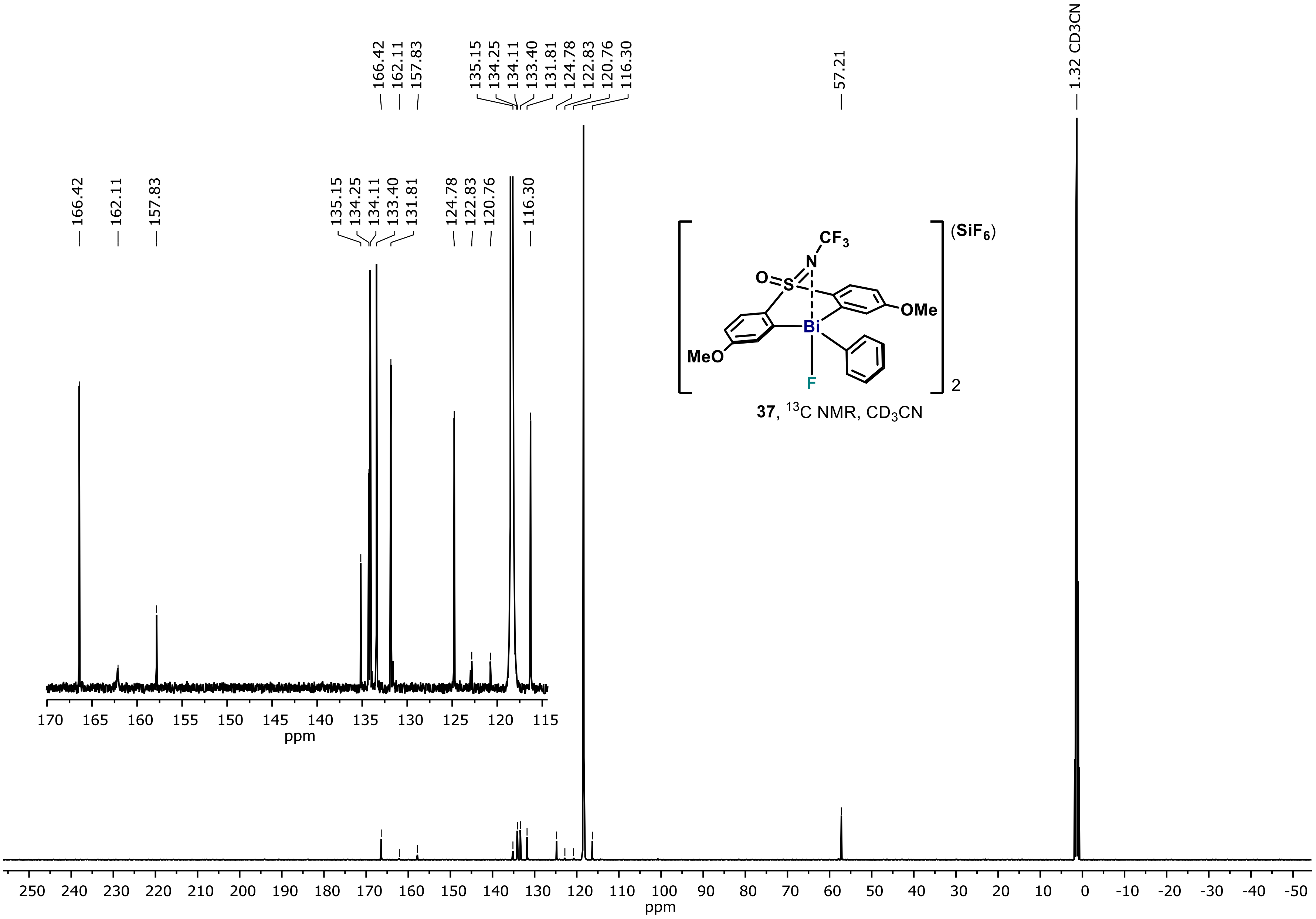
166.42
162.11
157.83
135.15
134.25
134.11
133.40
131.81
124.78
122.83
120.76
116.30
57.21
1.32 CD3CN
(SiF6)
CF3
N
O
S
OMe
MeO
Bi
F
2
37, 13C NMR, CD3CN
ppm

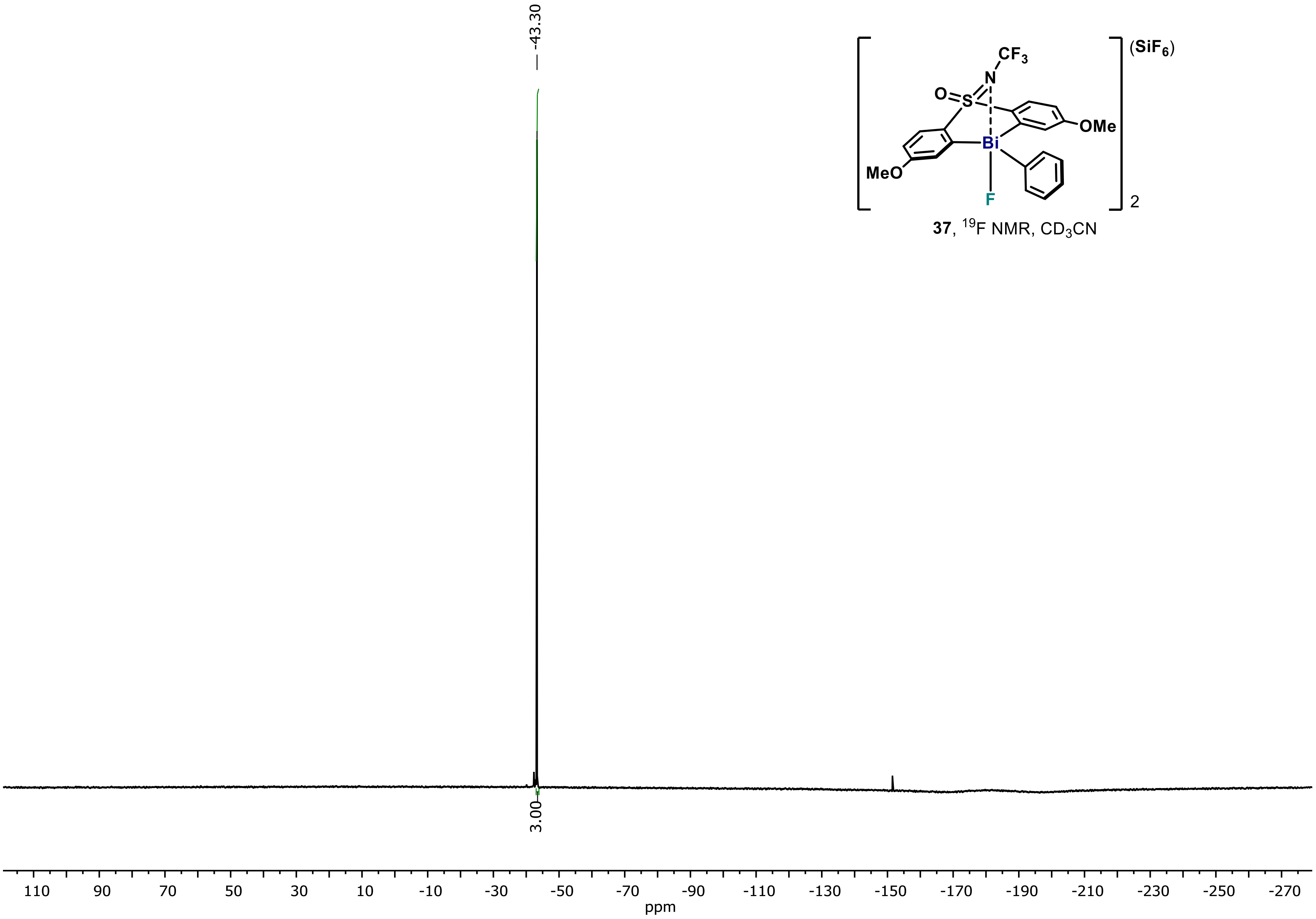

37, ^{19}F NMR, CD_3CN

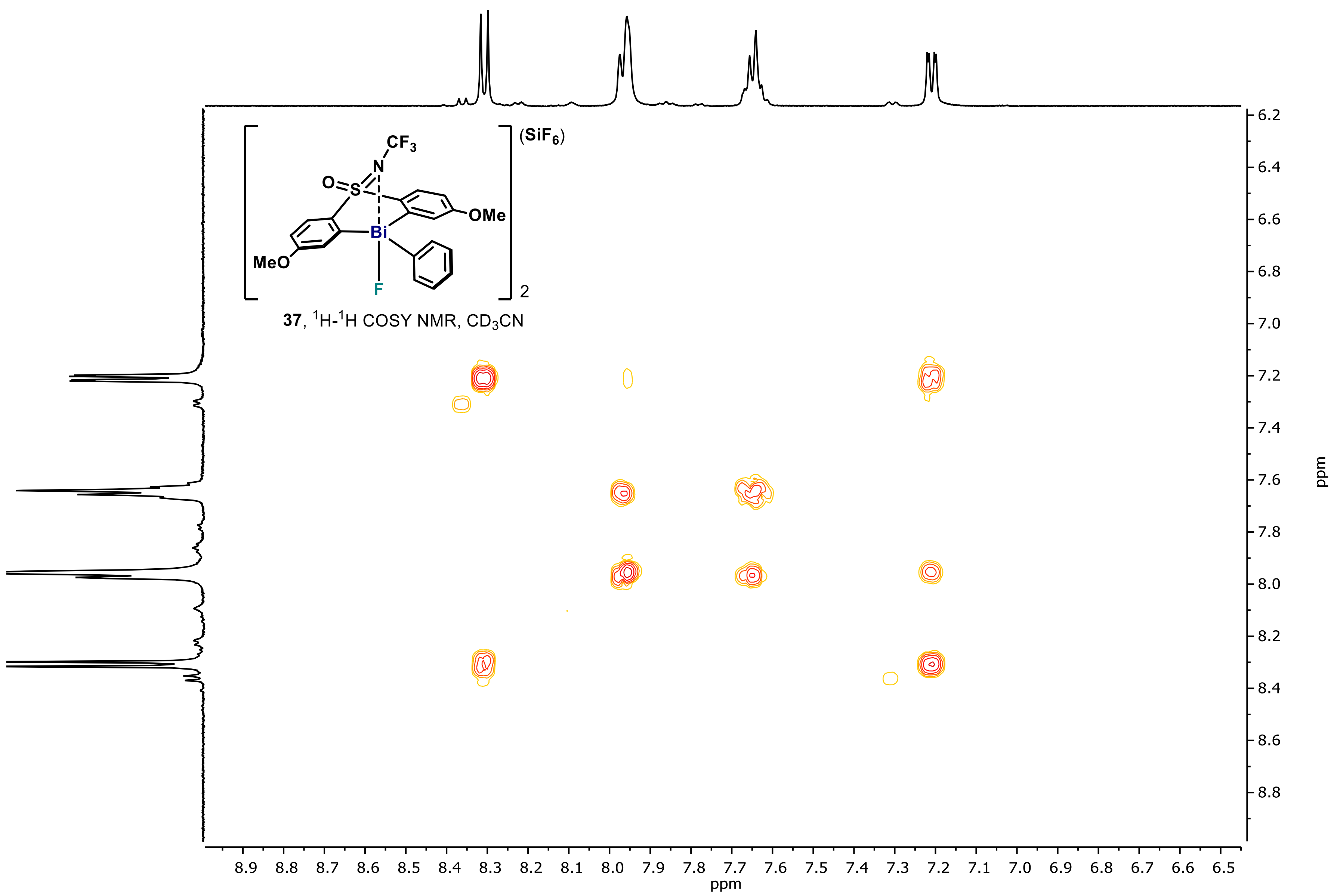
(SiF6)
CF3
N
O=S
OMe
MeO
Bi
F
2
37, 1H-1H COSY NMR, CD3CN
ppm
ppm

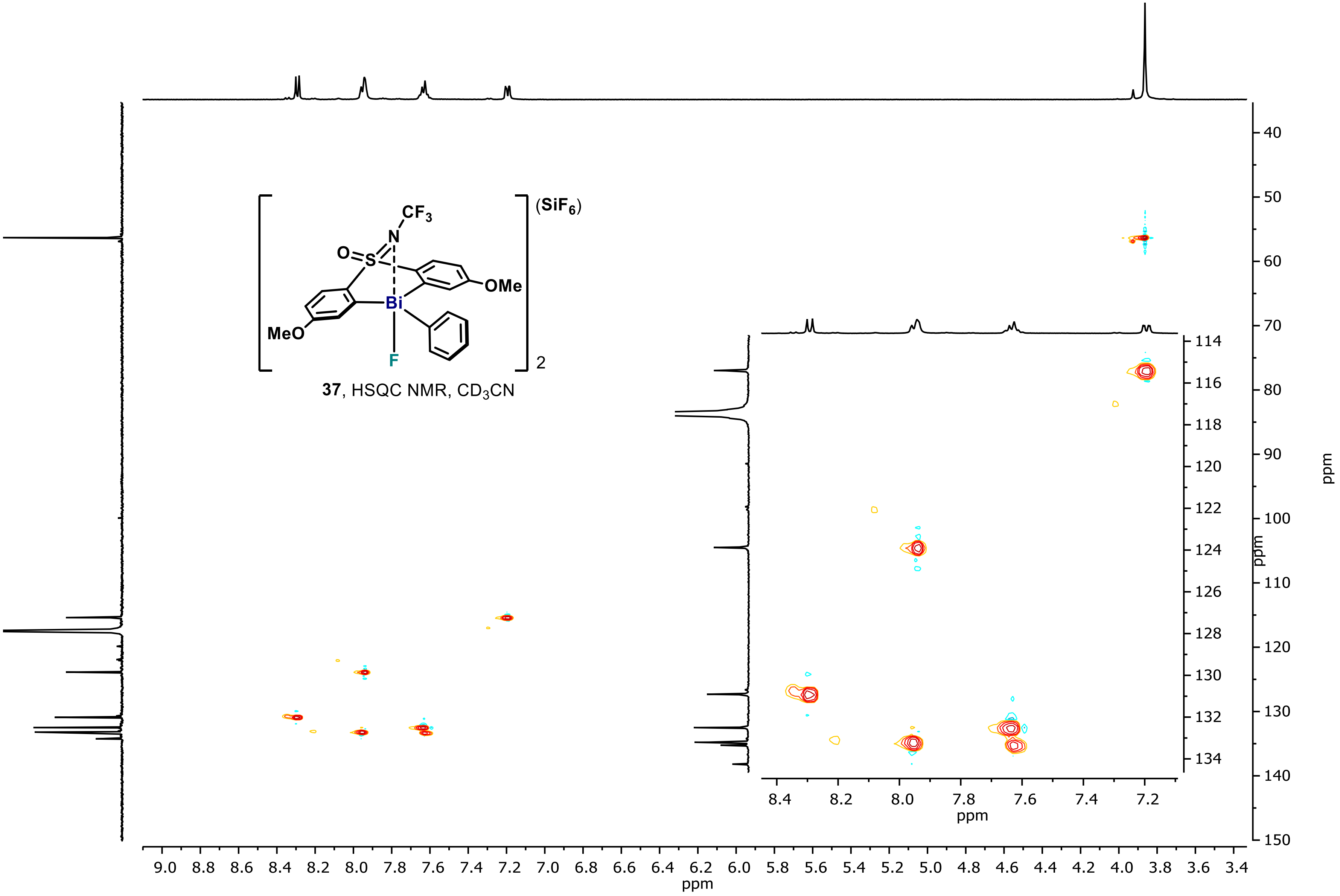
(SiF6)
CF3
N
O
S
OMe
Bi
MeO
F
2
37, HSQC NMR, CD3CN
ppm
ppm
ppm
ppm

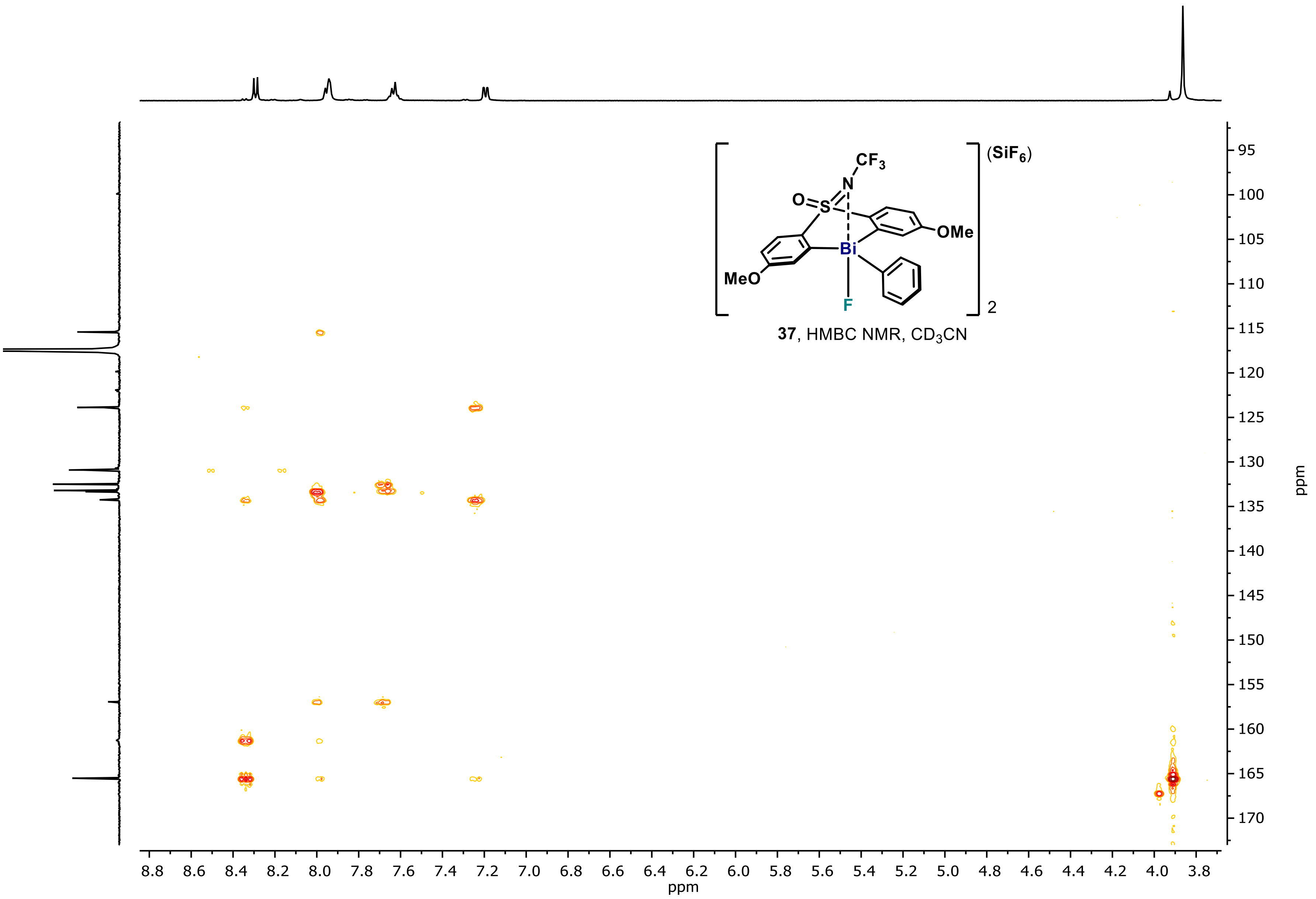
(SiF6)
CF3
N
O
S
OMe
Bi
MeO
F
2
37, HMBC NMR, CD3CN
ppm
ppm
8.8 8.6 8.4 8.2 8.0 7.8 7.6 7.4 7.2 7.0 6.8 6.6 6.4 6.2 6.0 5.8 5.6 5.4 5.2 5.0 4.8 4.6 4.4 4.2 4.0 3.8
95 100 105 110 115 120 125 130 135 140 145 150 155 160 165 170

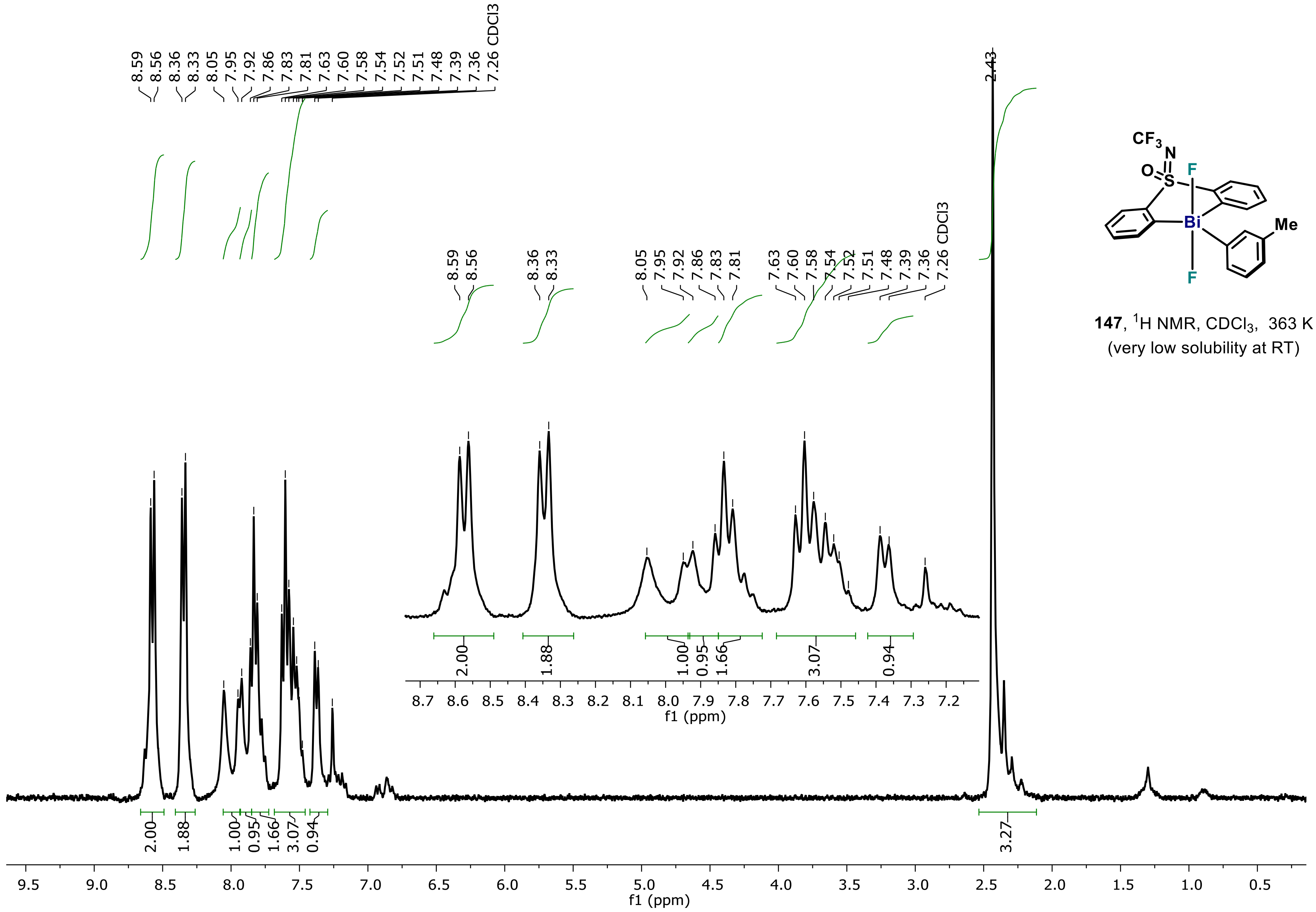

147, ^{1}H NMR, $CDCl_3$, 363 K
(very low solubility at RT)

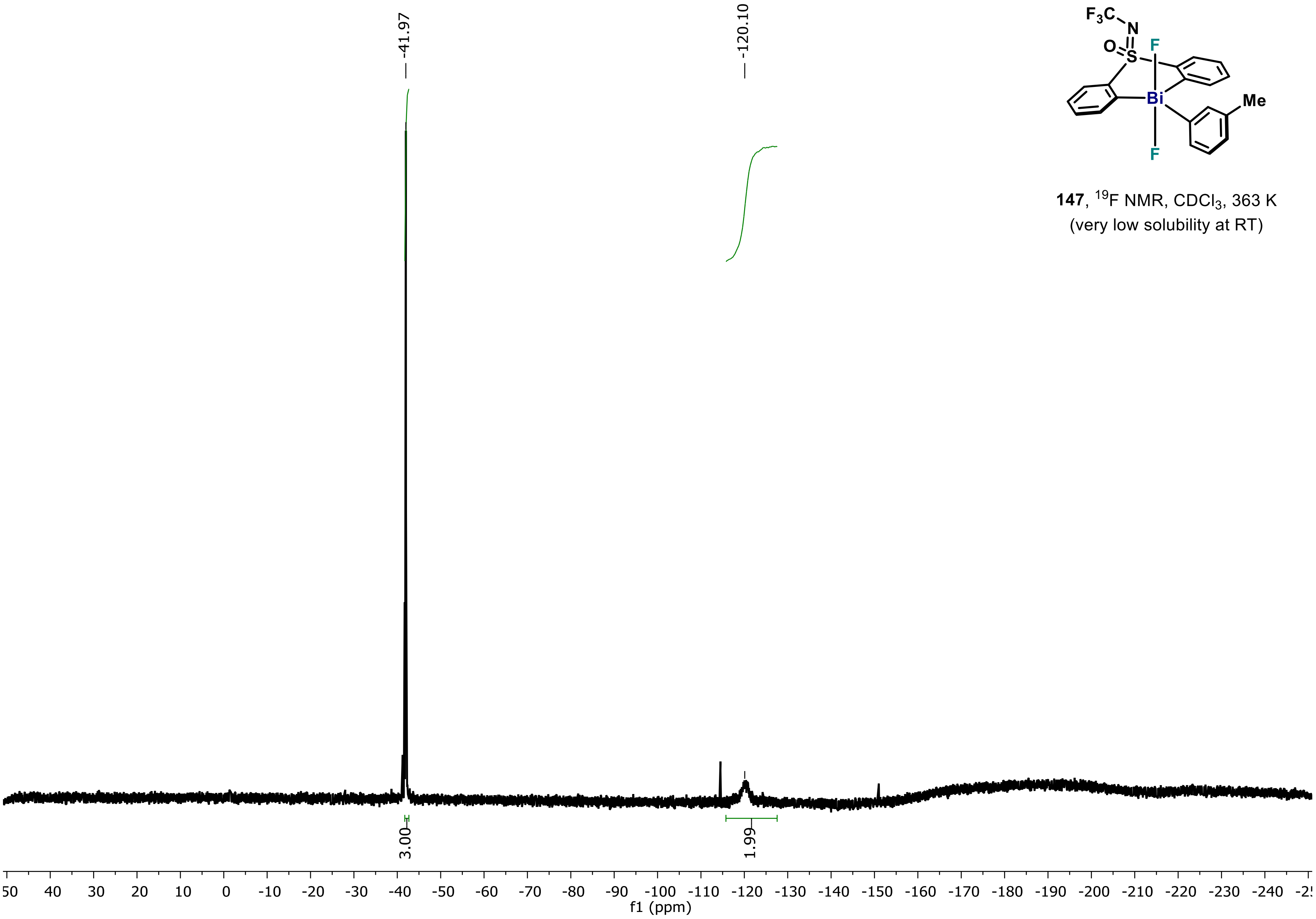

147, ^{19}F NMR, $CDCl_3$, 363 K
(very low solubility at RT)

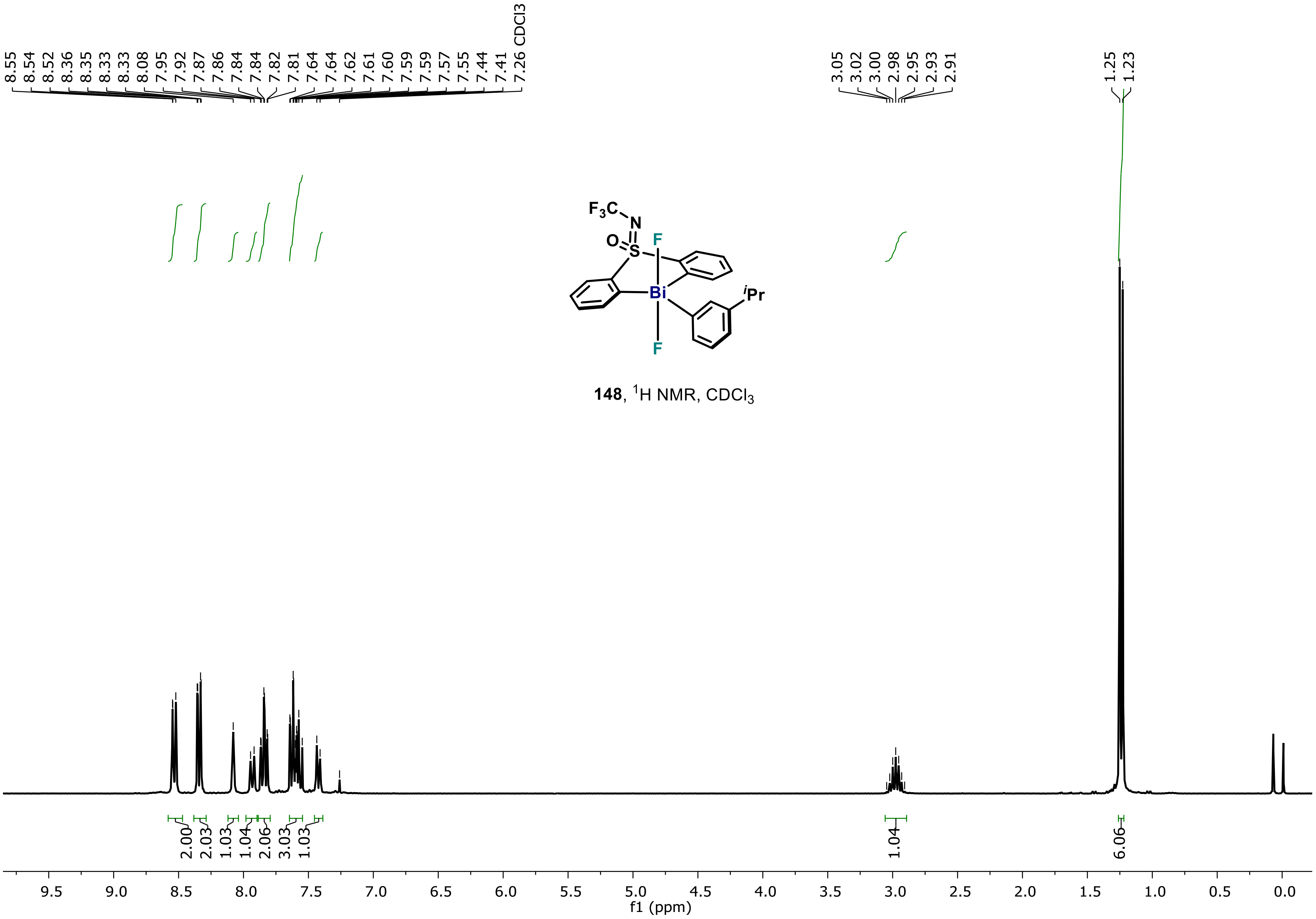

148, ^{1}H NMR, $CDCl_3$

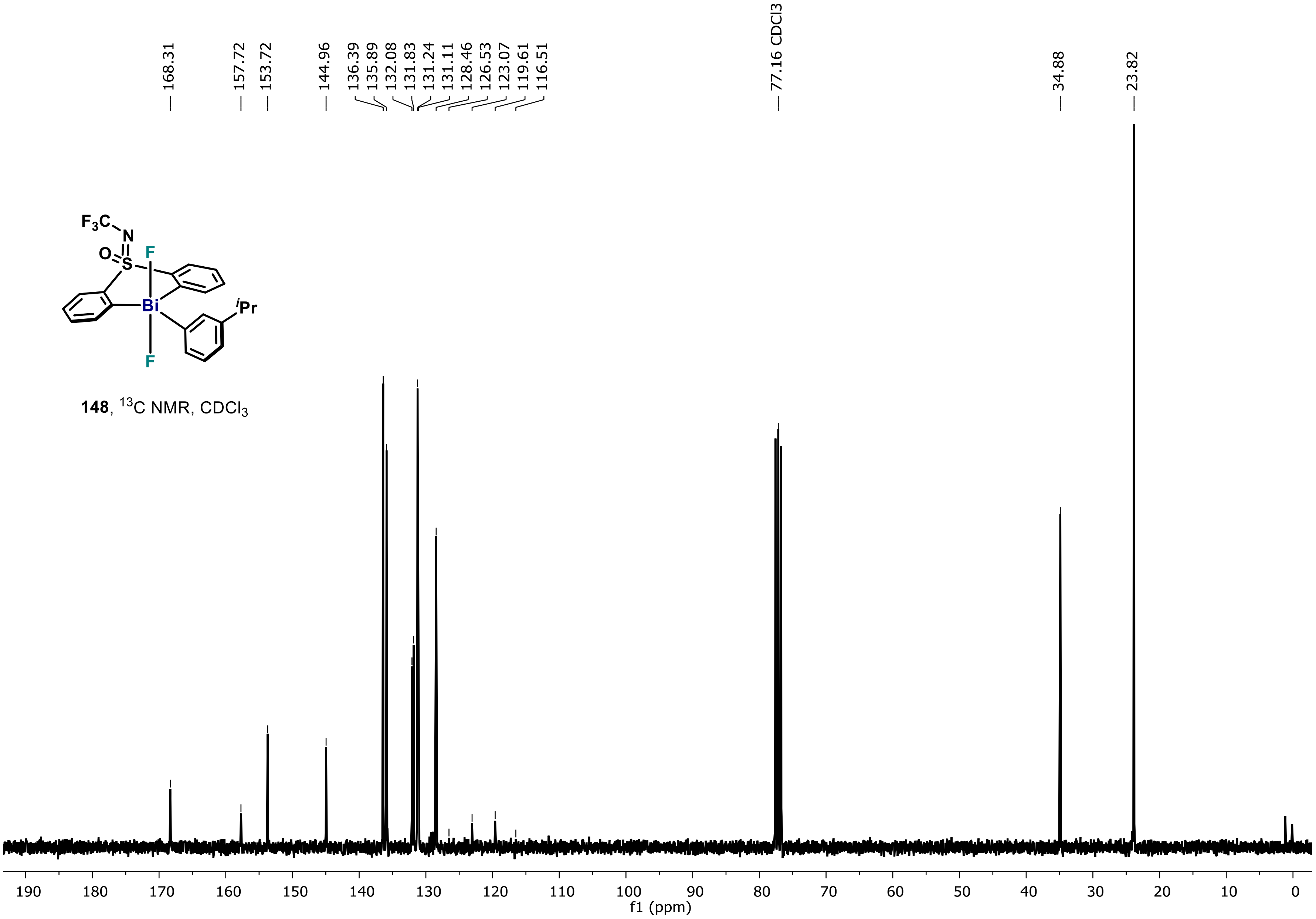

148, ^{13}C NMR, $CDCl_3$

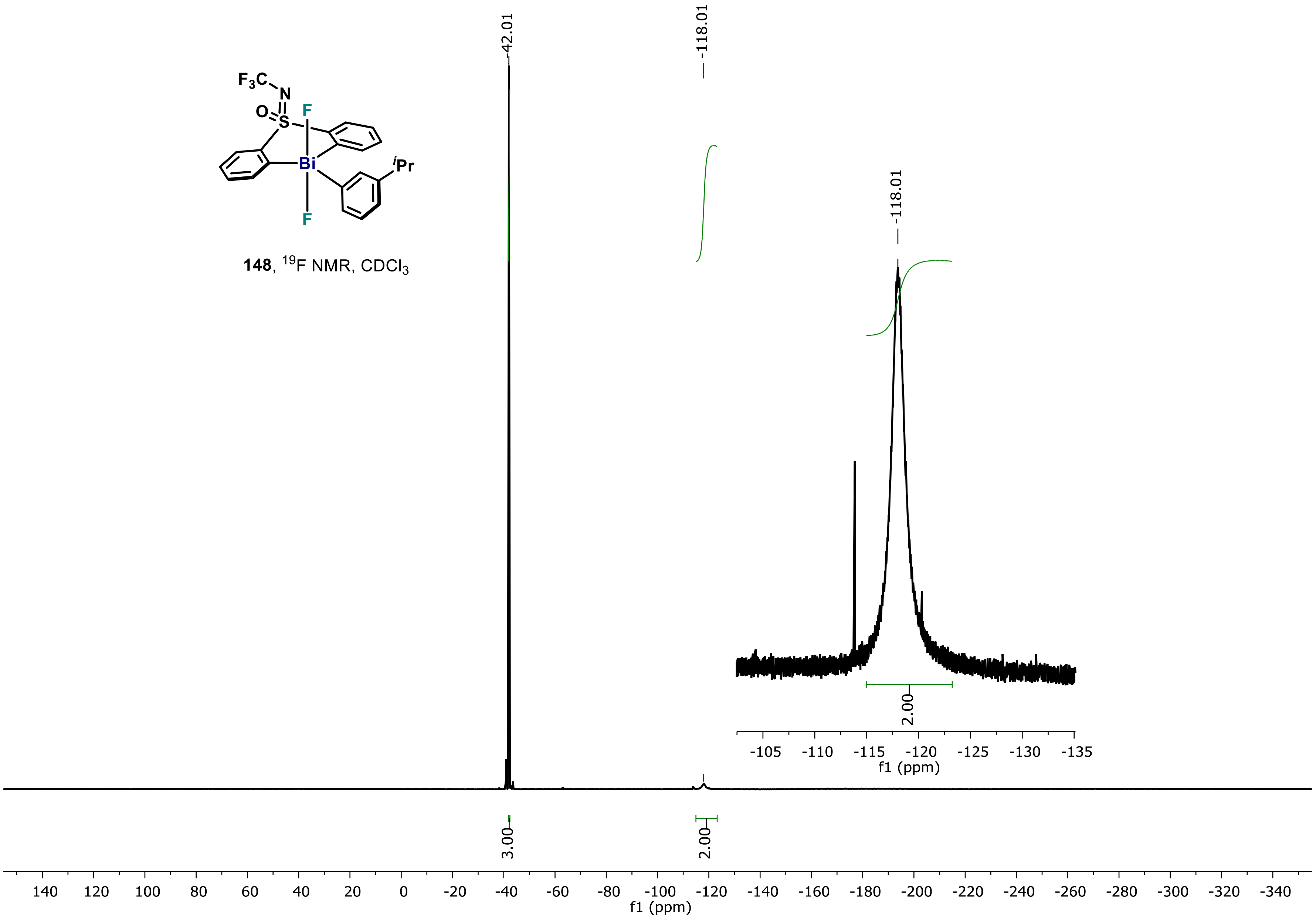

F3C
N
O
S
F
Bi
iPr
F
148, 19F NMR, CDCl3
-42.01
-118.01
-118.01
2.00
-105 -110 -115 -120 -125 -130 -135
f1 (ppm)
3.00
2.00
140 120 100 80 60 40 20 0 -20 -40 -60 -80 -100 -120 -140 -160 -180 -200 -220 -240 -260 -280 -300 -320 -340
f1 (ppm)

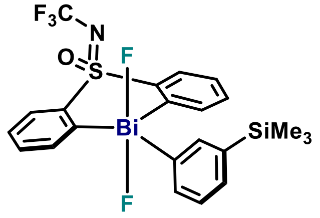

149, ^{1}H NMR, $CDCl_3$
(partial decomposition to **57** observed)

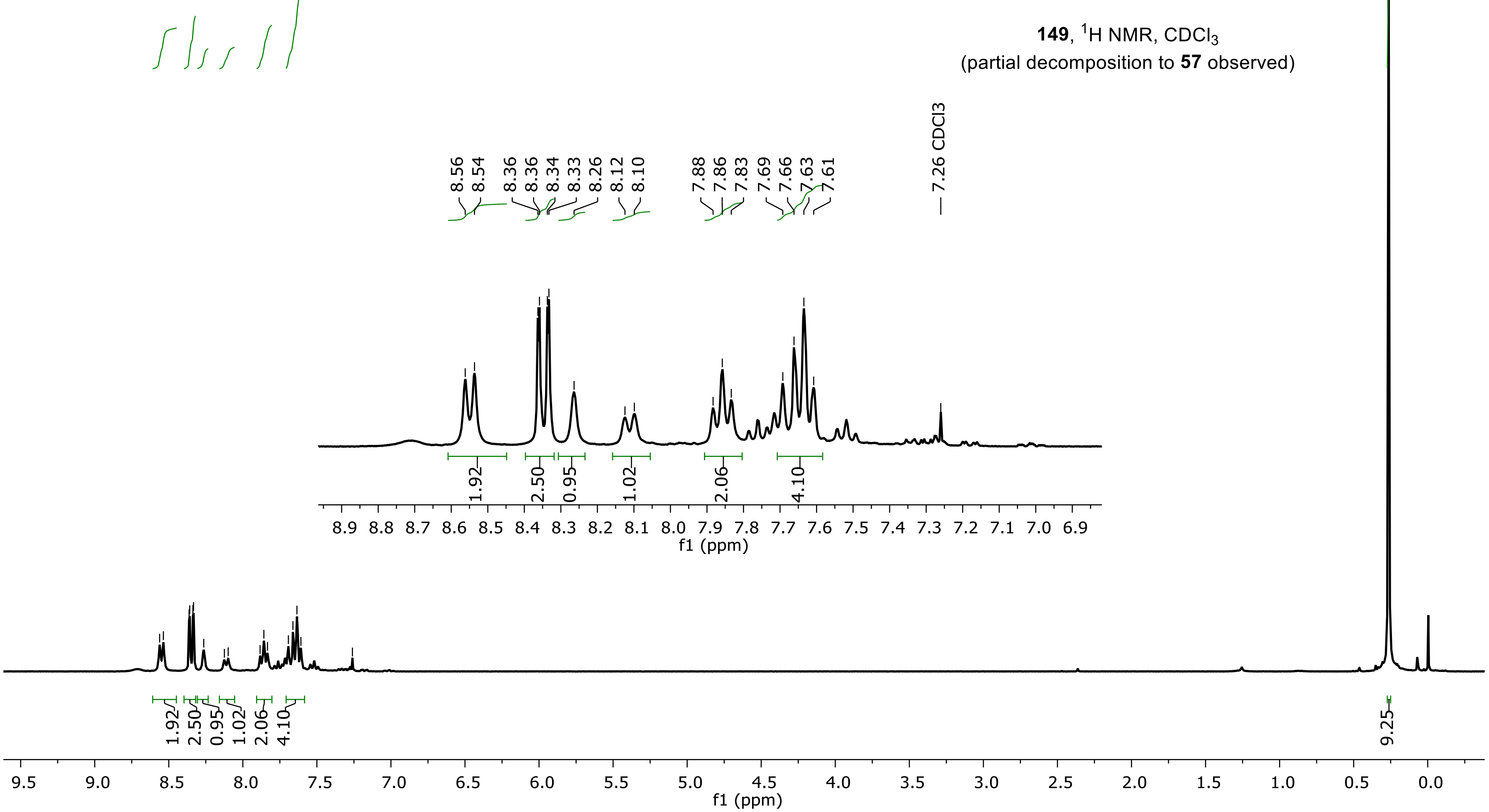

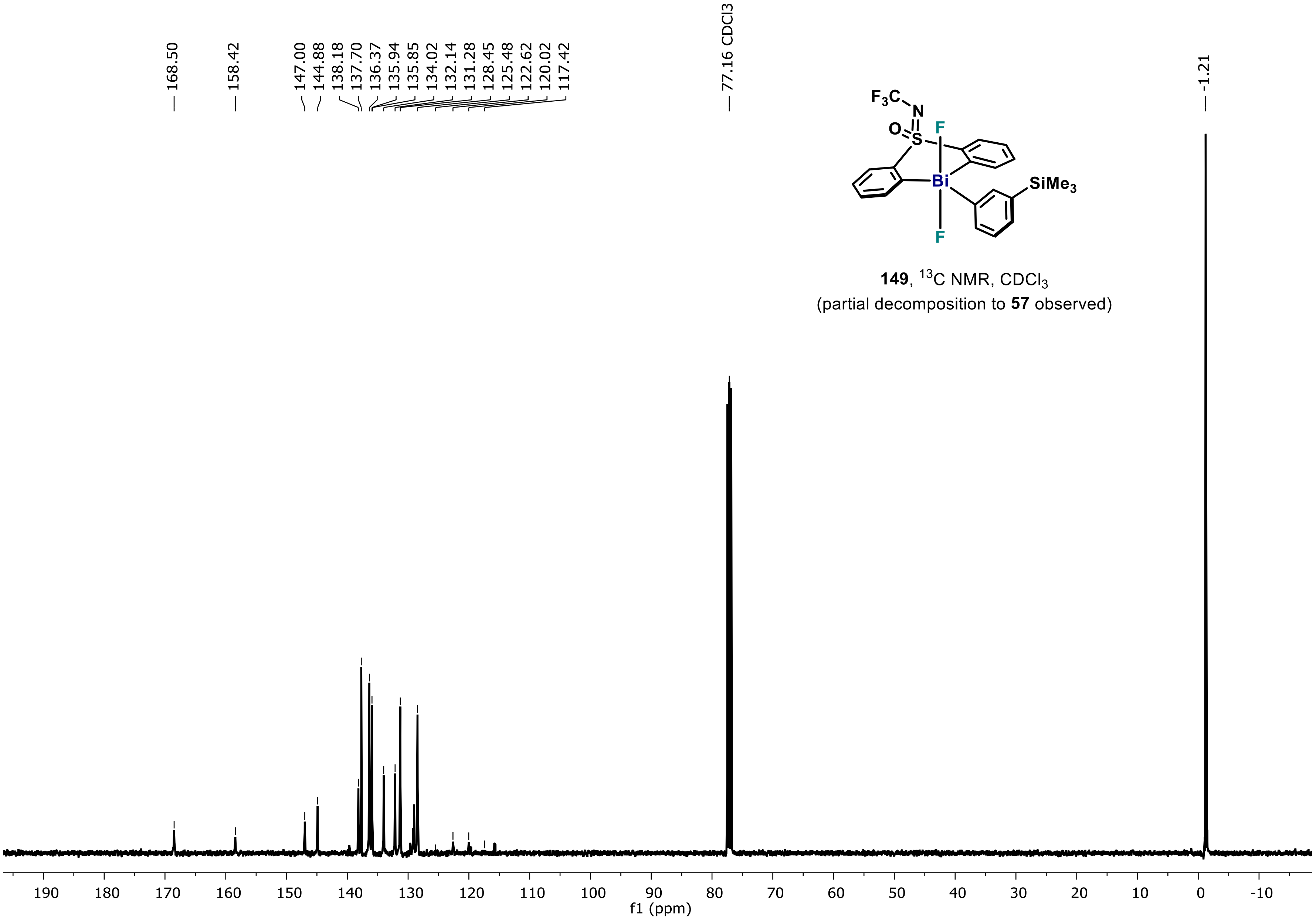

149, ^{13}C NMR, $CDCl_3$
(partial decomposition to **57** observed)

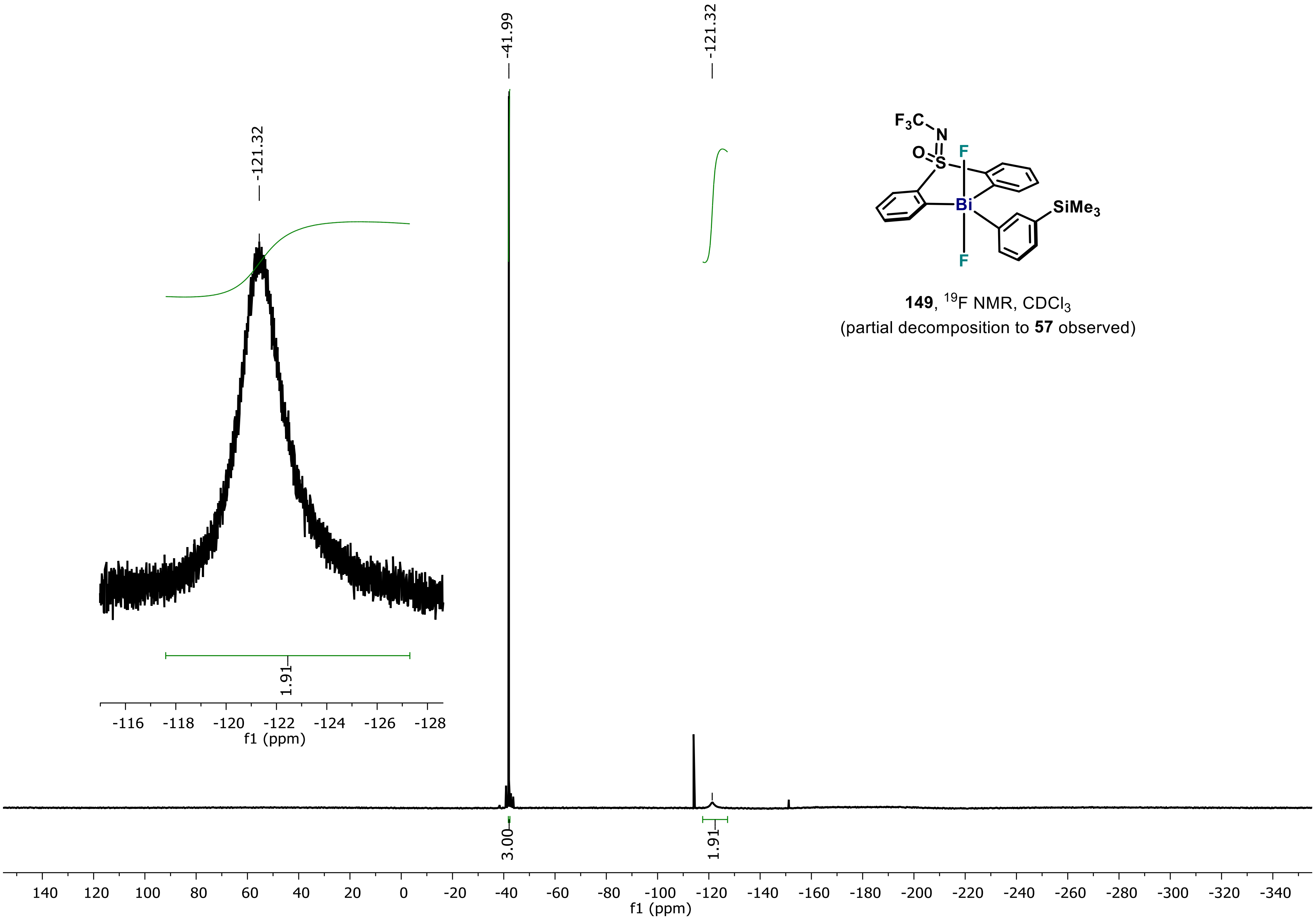
-41.99
-121.32
F3C
N
O
S
F
Bi
SiMe3
F
149, 19F NMR, CDCl3
(partial decomposition to 57 observed)
-121.32
1.91
-116 -118 -120 -122 -124 -126 -128
f1 (ppm)
3.00
1.91
140 120 100 80 60 40 20 0 -20 -40 -60 -80 -100 -120 -140 -160 -180 -200 -220 -240 -260 -280 -300 -320 -340
f1 (ppm)

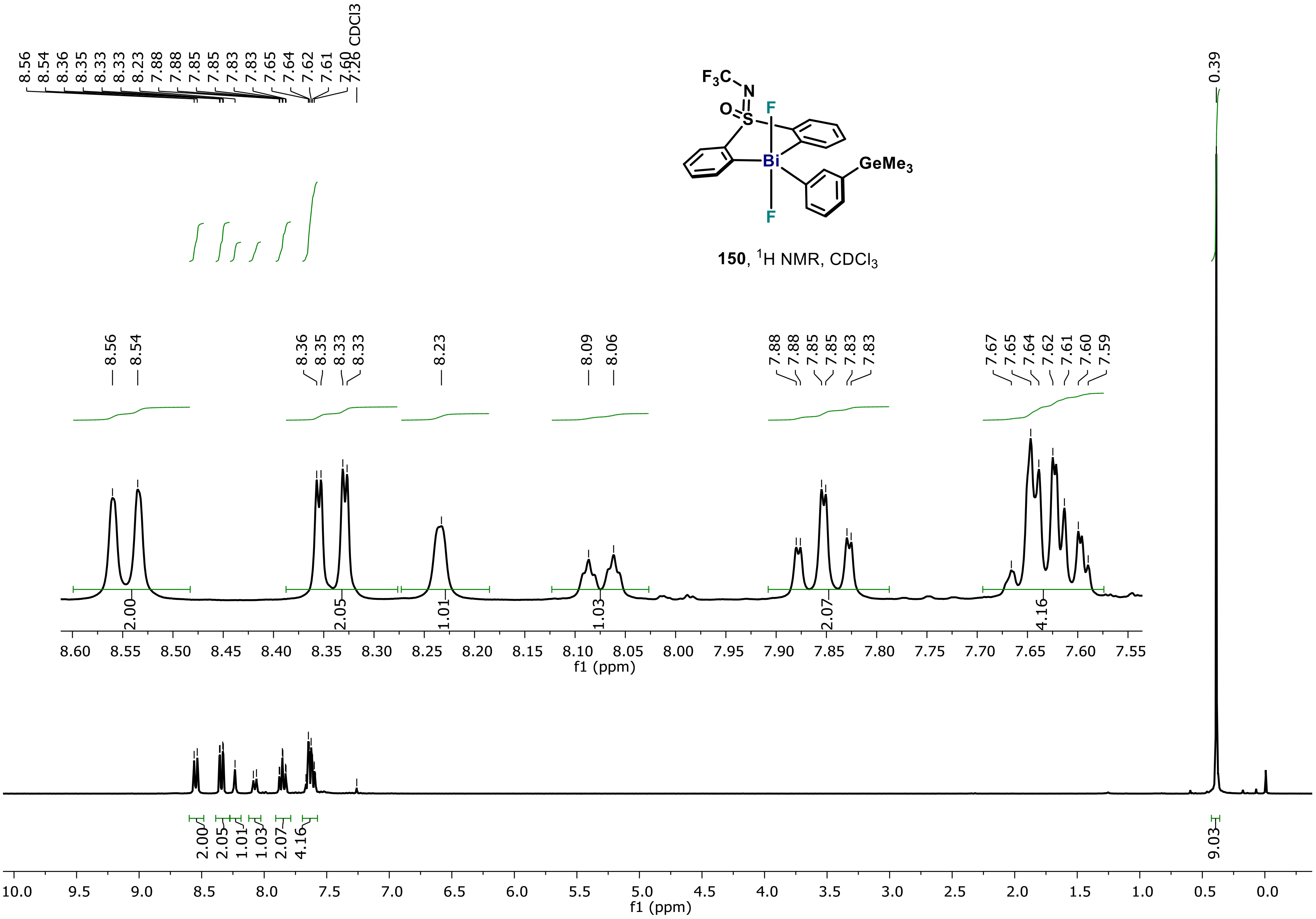

150, ^{1}H NMR, $CDCl_3$

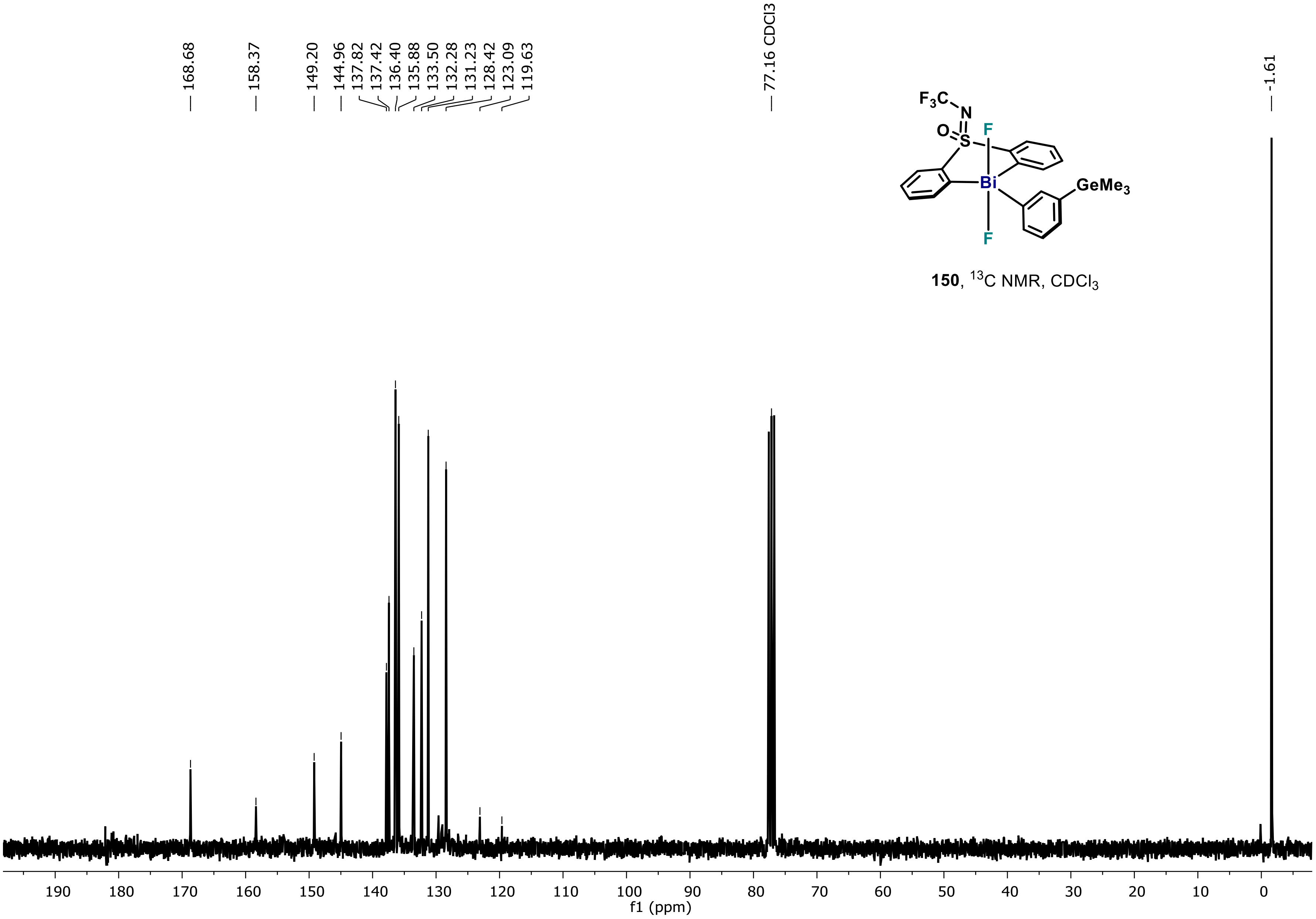
168.68
158.37
149.20
144.96
137.82
137.42
136.40
135.88
133.50
132.28
131.23
128.42
123.09
119.63
77.16 CDCl3
-1.61
F3C
N
O
S
F
Bi
GeMe3
F
150, 13C NMR, CDCl3
f1 (ppm)

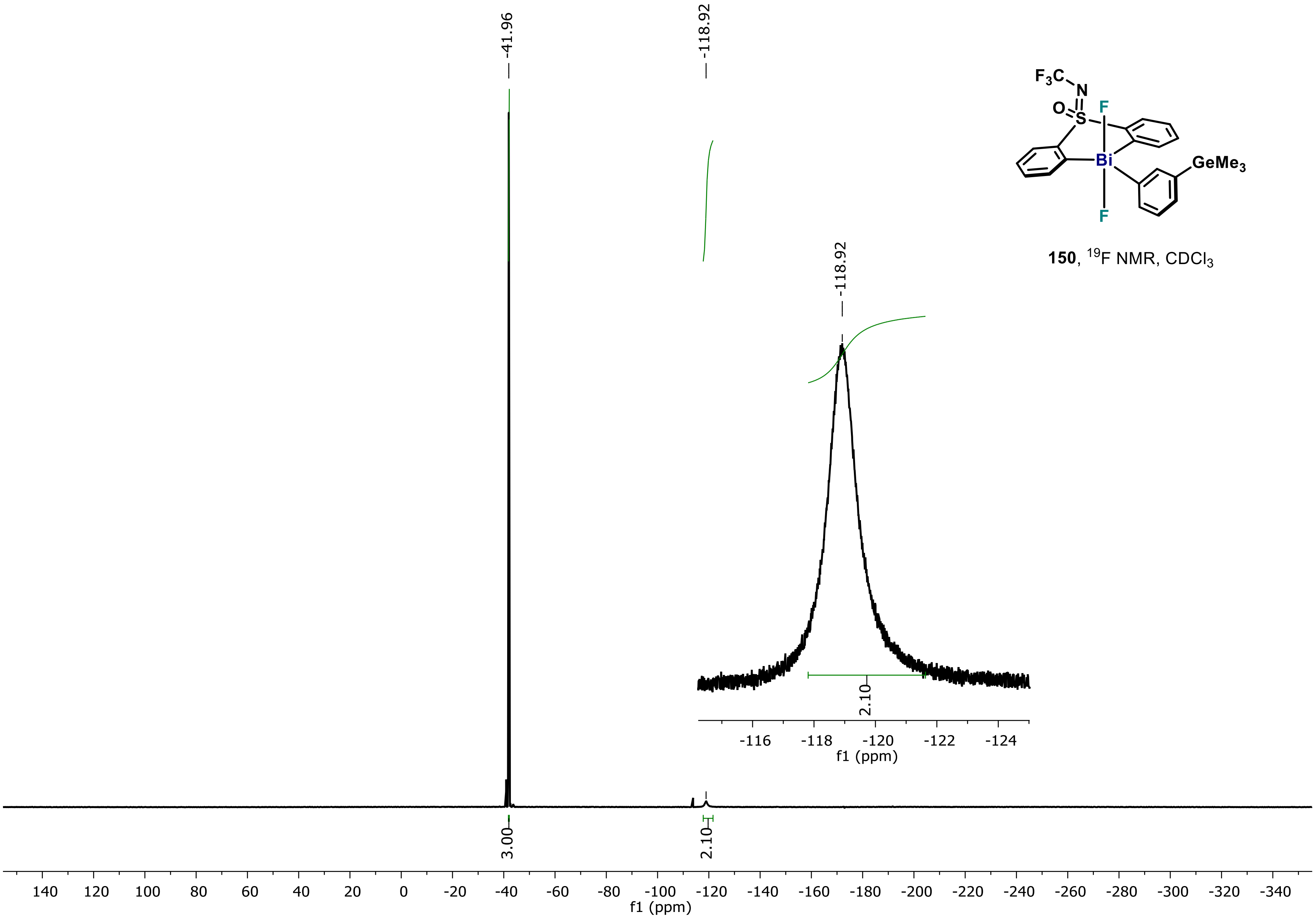

150, ^{19}F NMR, $CDCl_3$

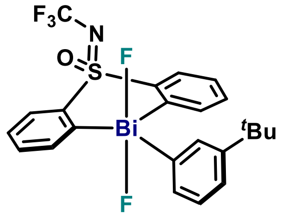

151, ^{1}H NMR, $CDCl_3$

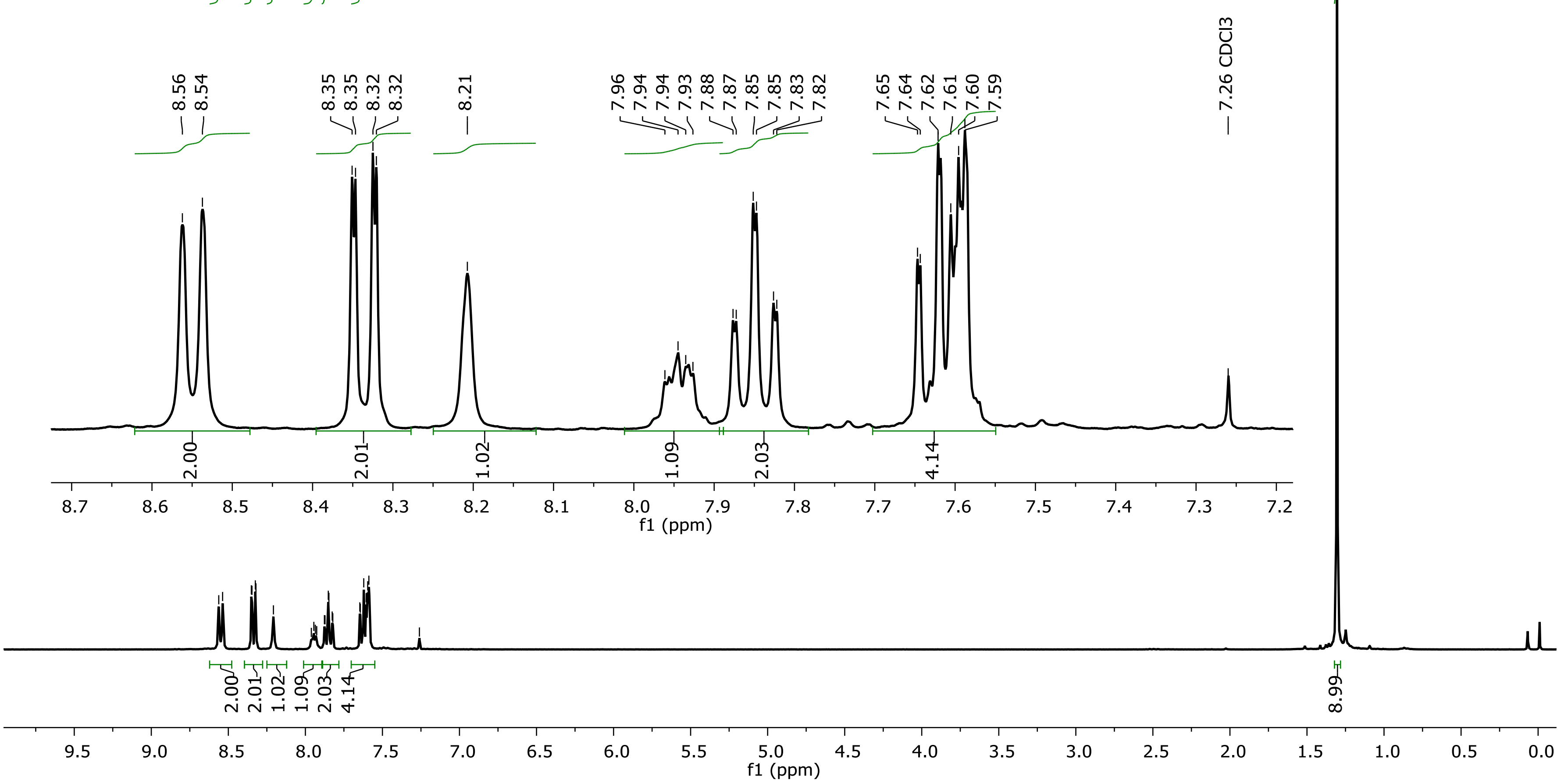

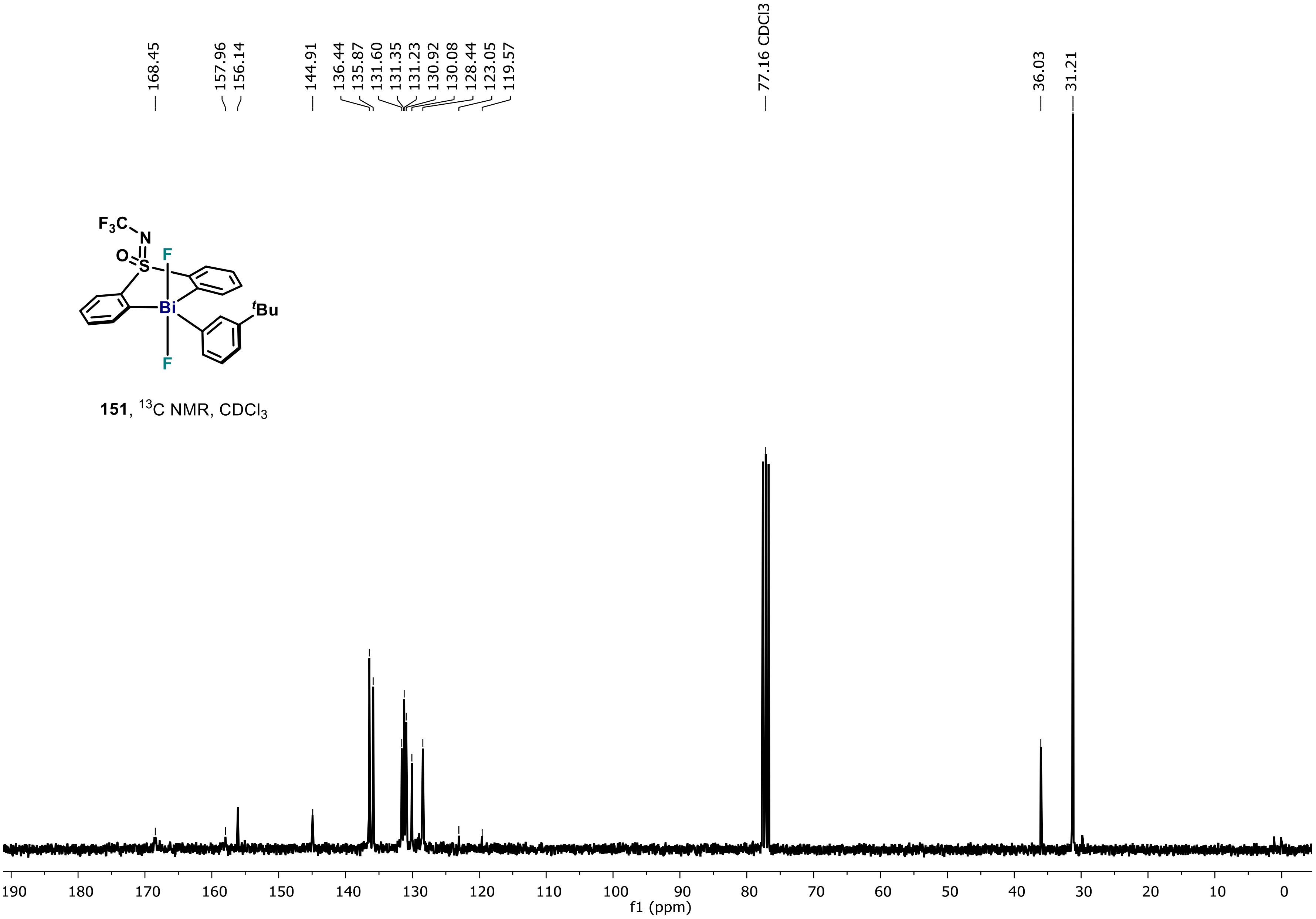

151, ^{13}C NMR, $CDCl_3$

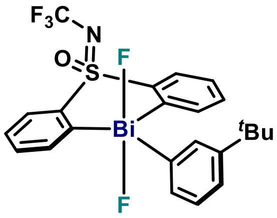

151, ^{19}F NMR, $CDCl_3$

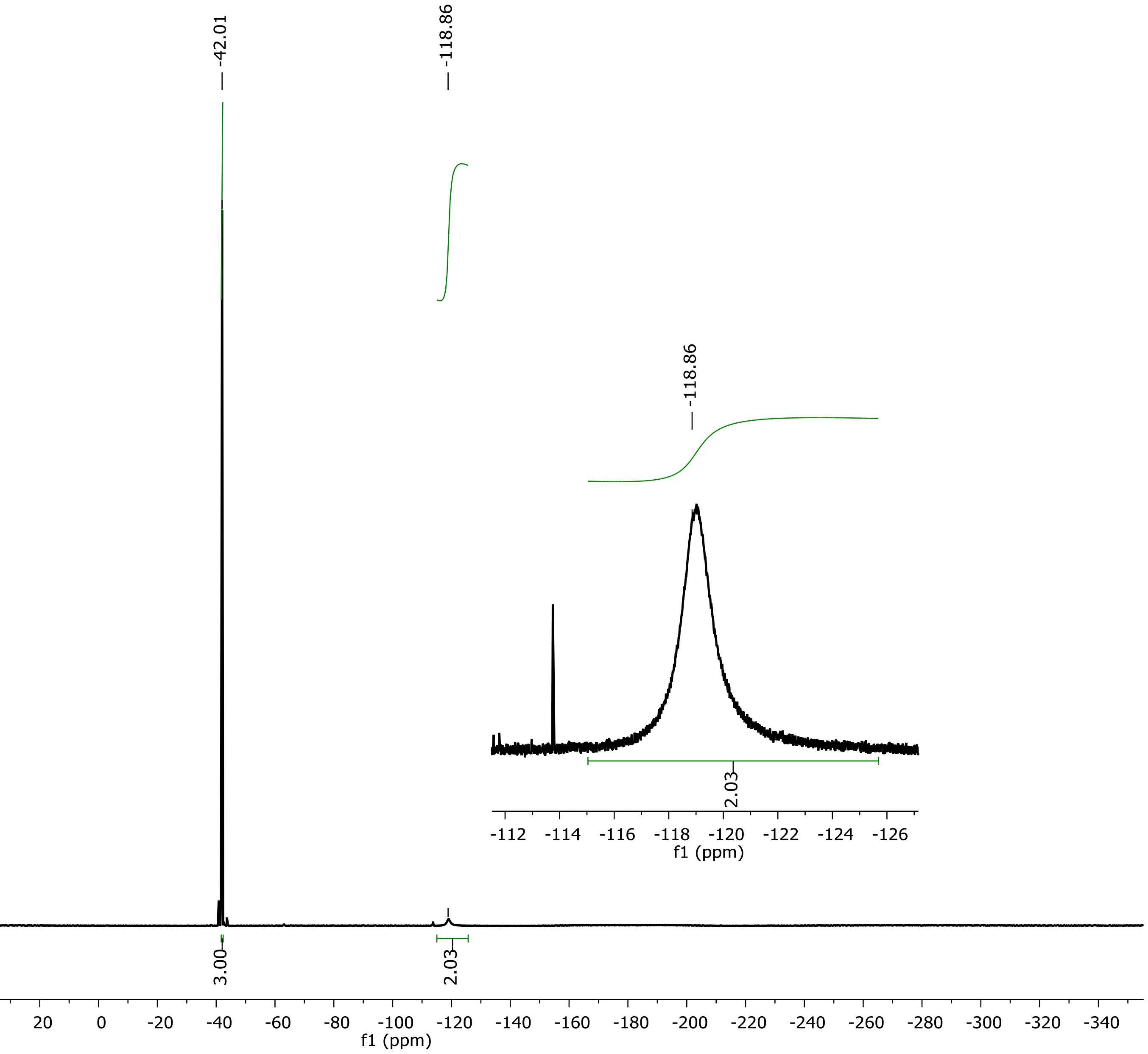

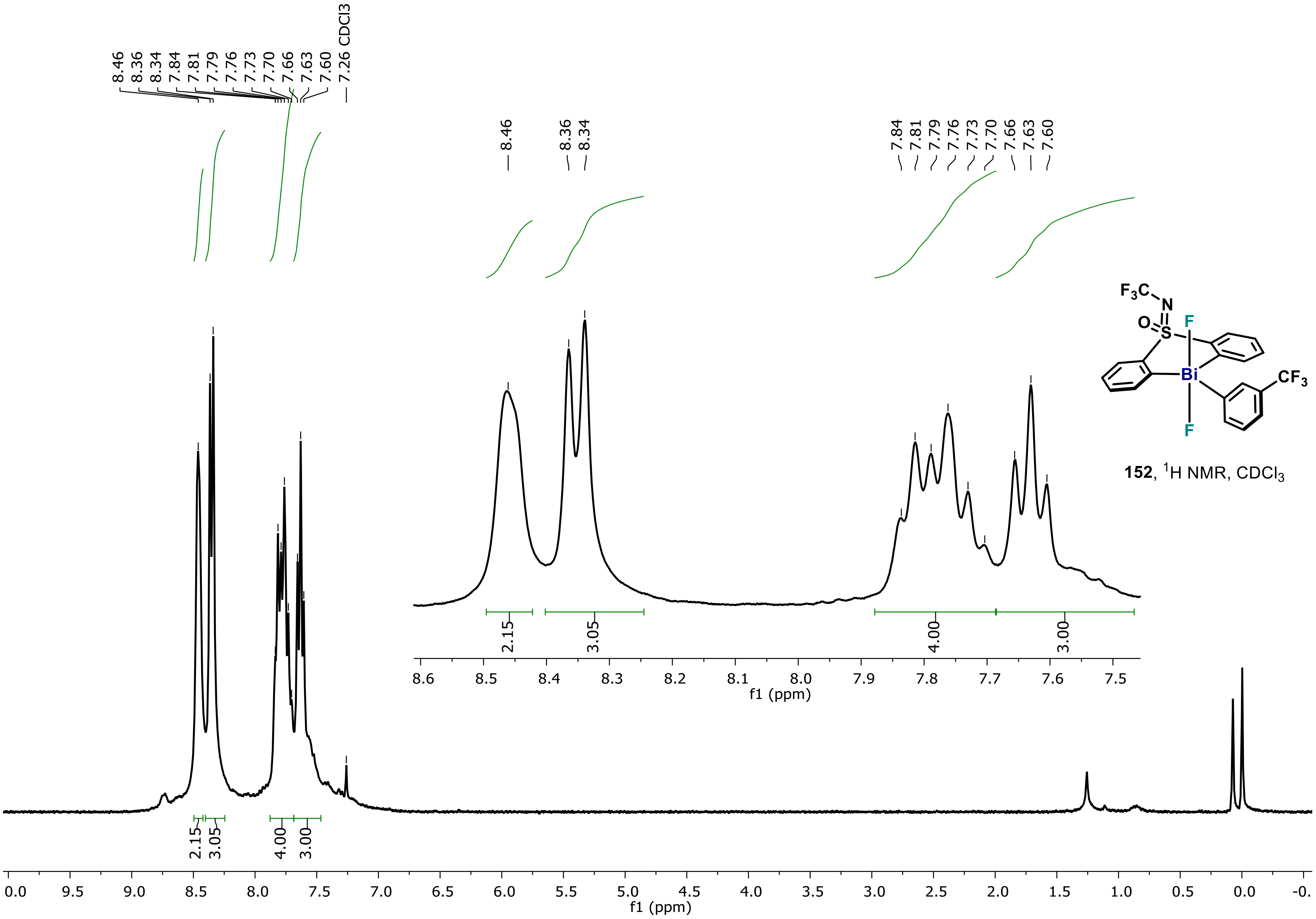

152, ^{1}H NMR, $CDCl_3$

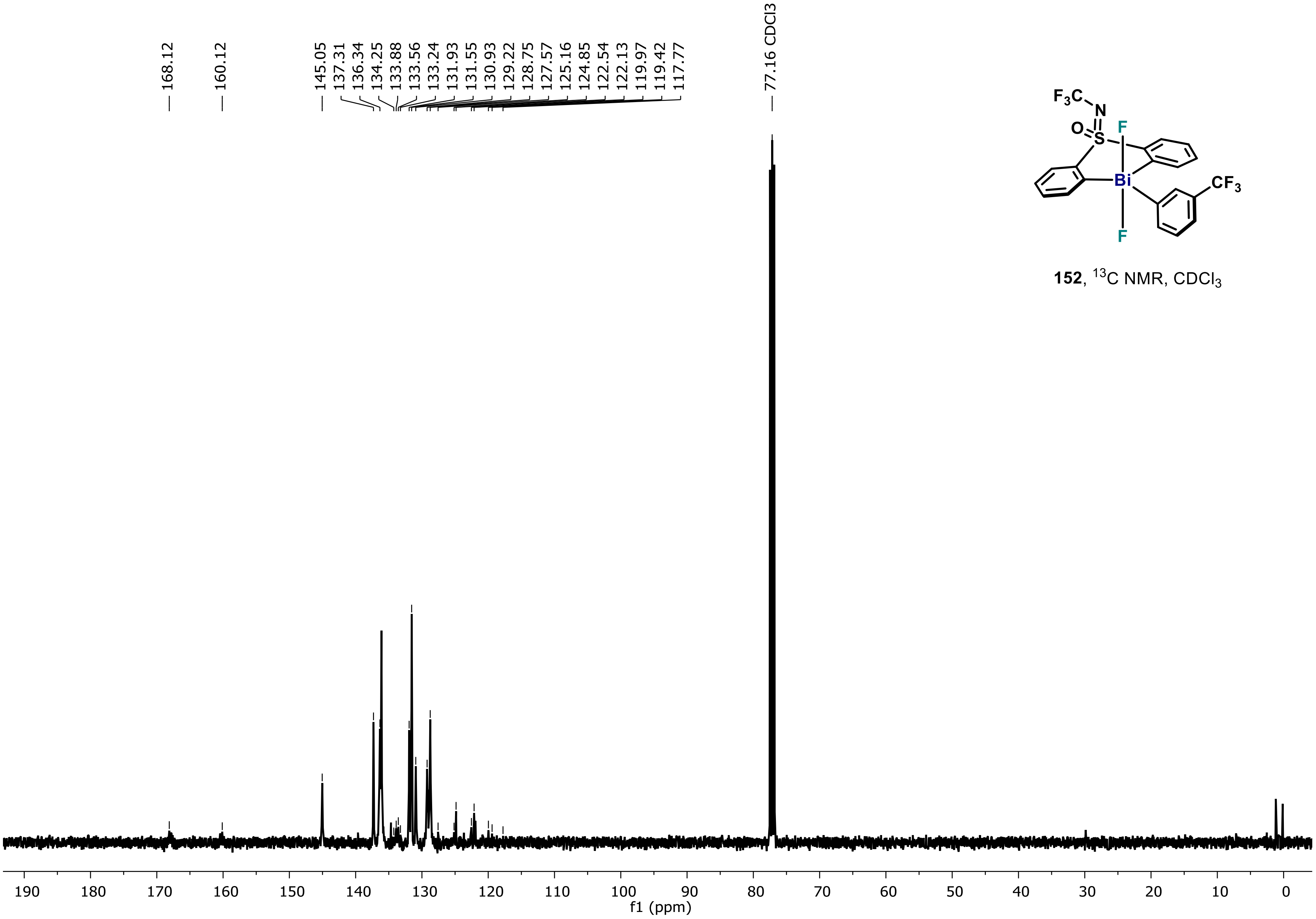

152, ^{13}C NMR, $CDCl_3$

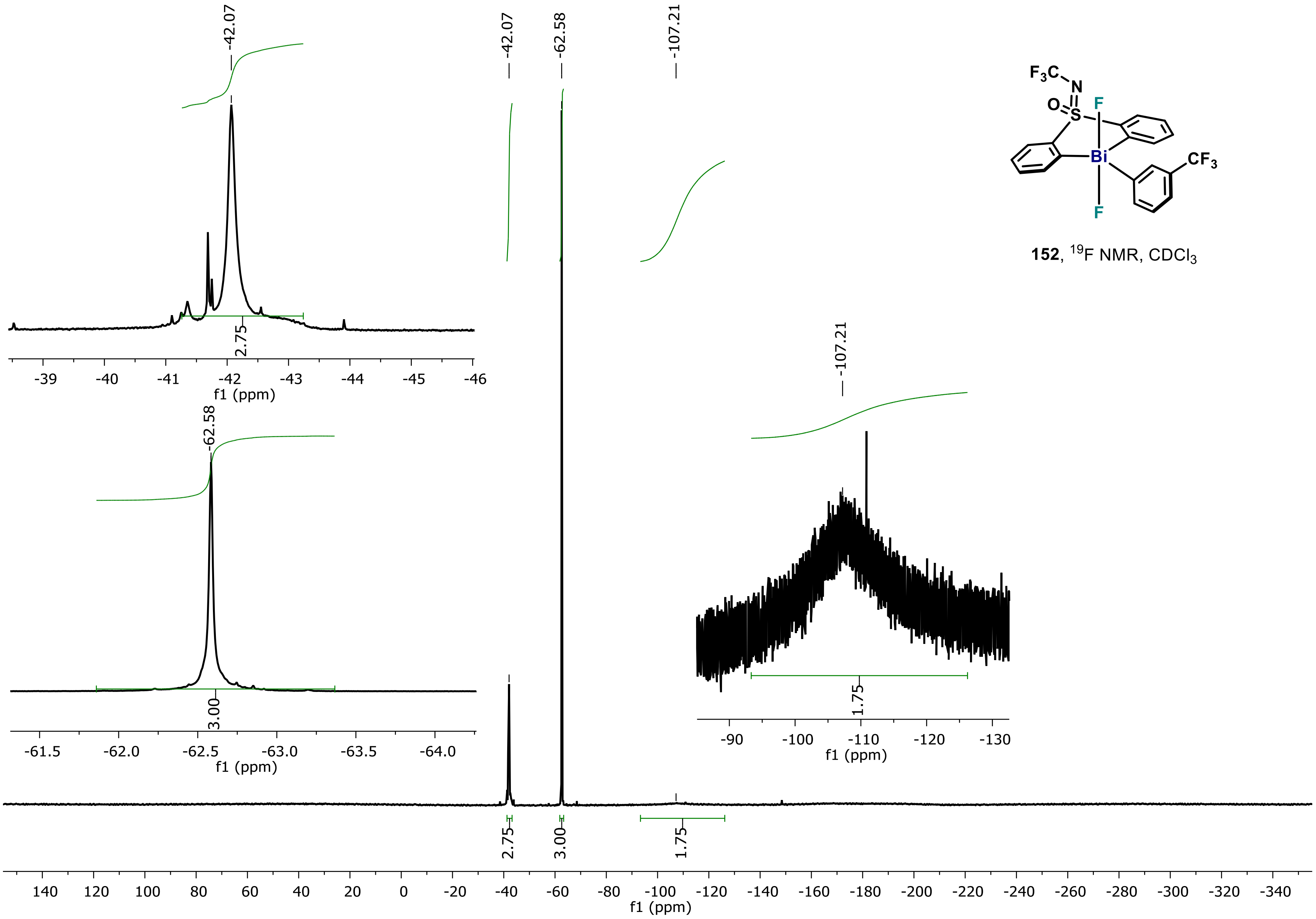

152, ^{19}F NMR, $CDCl_3$
-42.07
-62.58
-107.21
2.75
3.00
1.75
f1 (ppm)

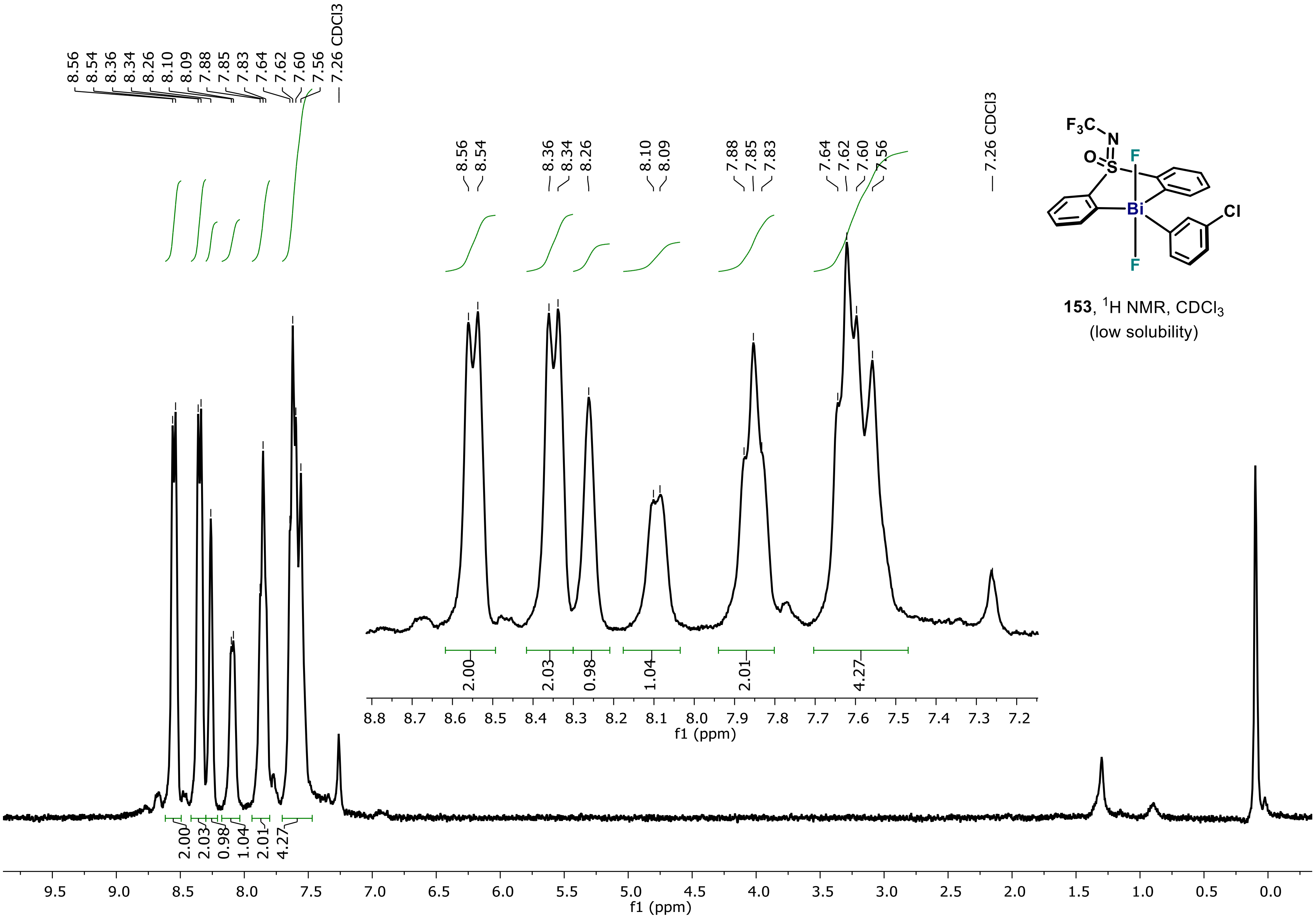

153, ^{1}H NMR, $CDCl_3$
(low solubility)

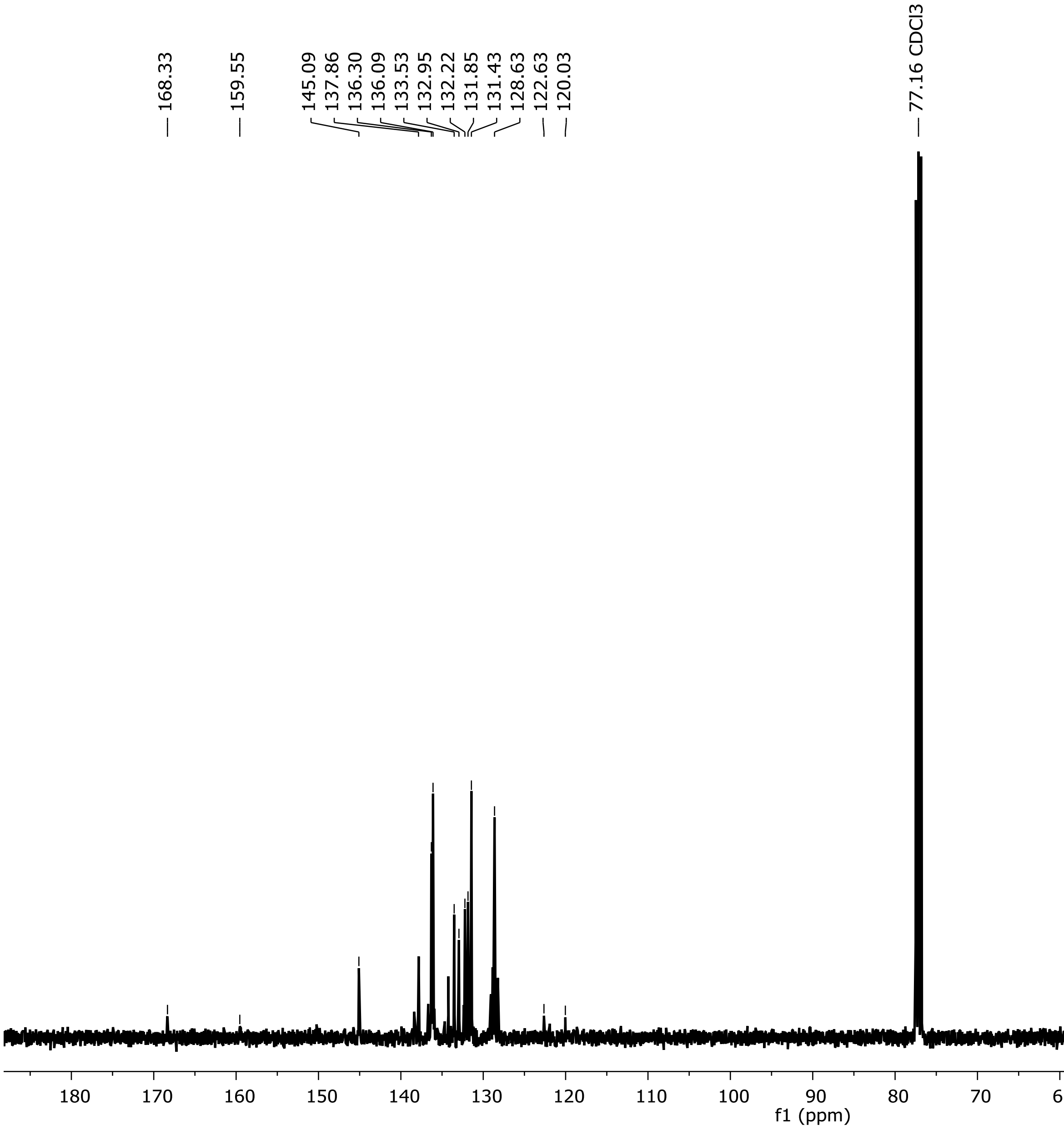

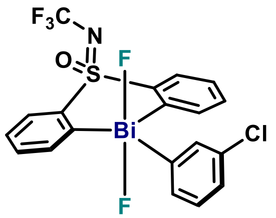

153, ^{13}C NMR, $CDCl_3$
(low solubility)

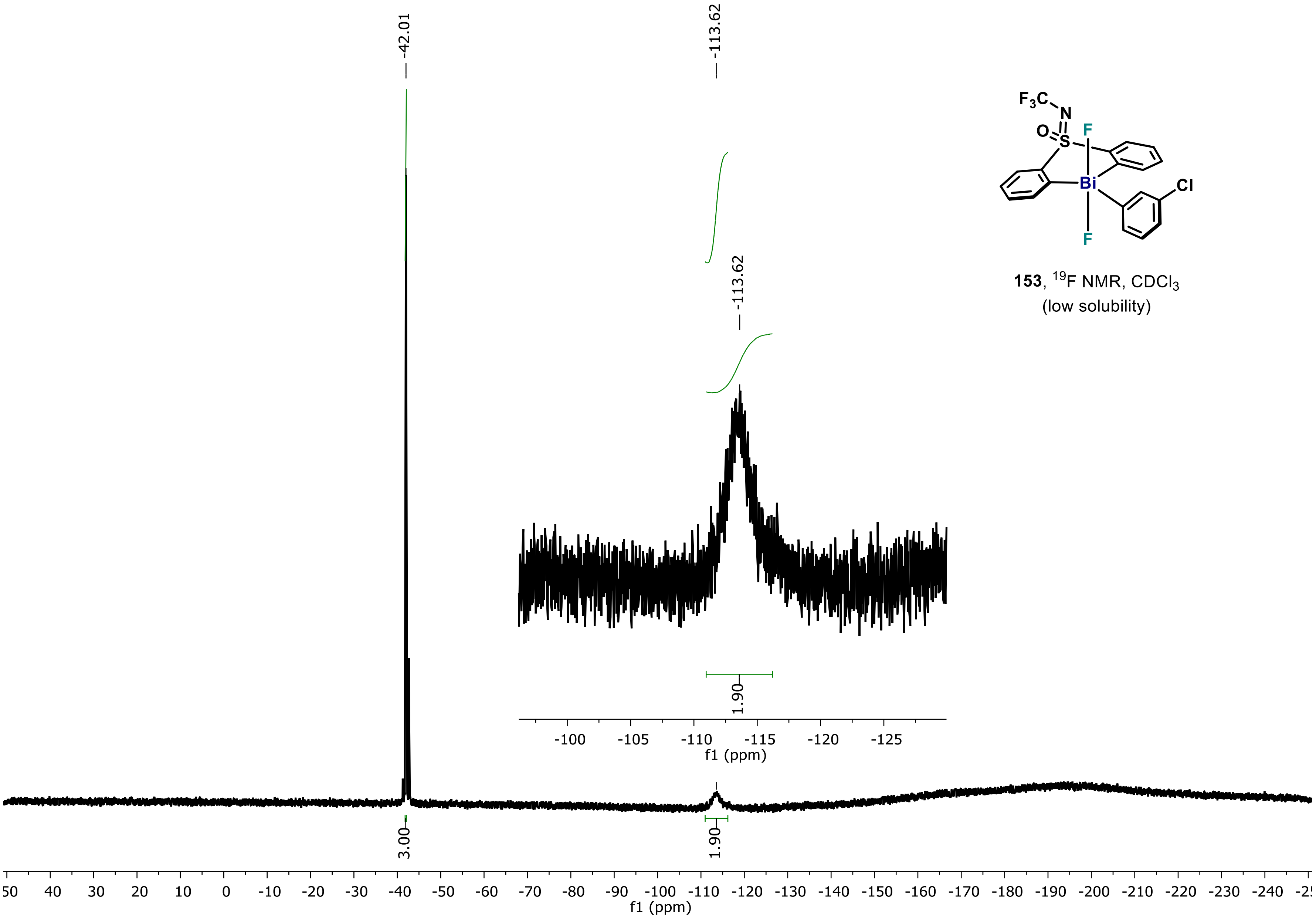

153, ^{19}F NMR, $CDCl_3$
(low solubility)

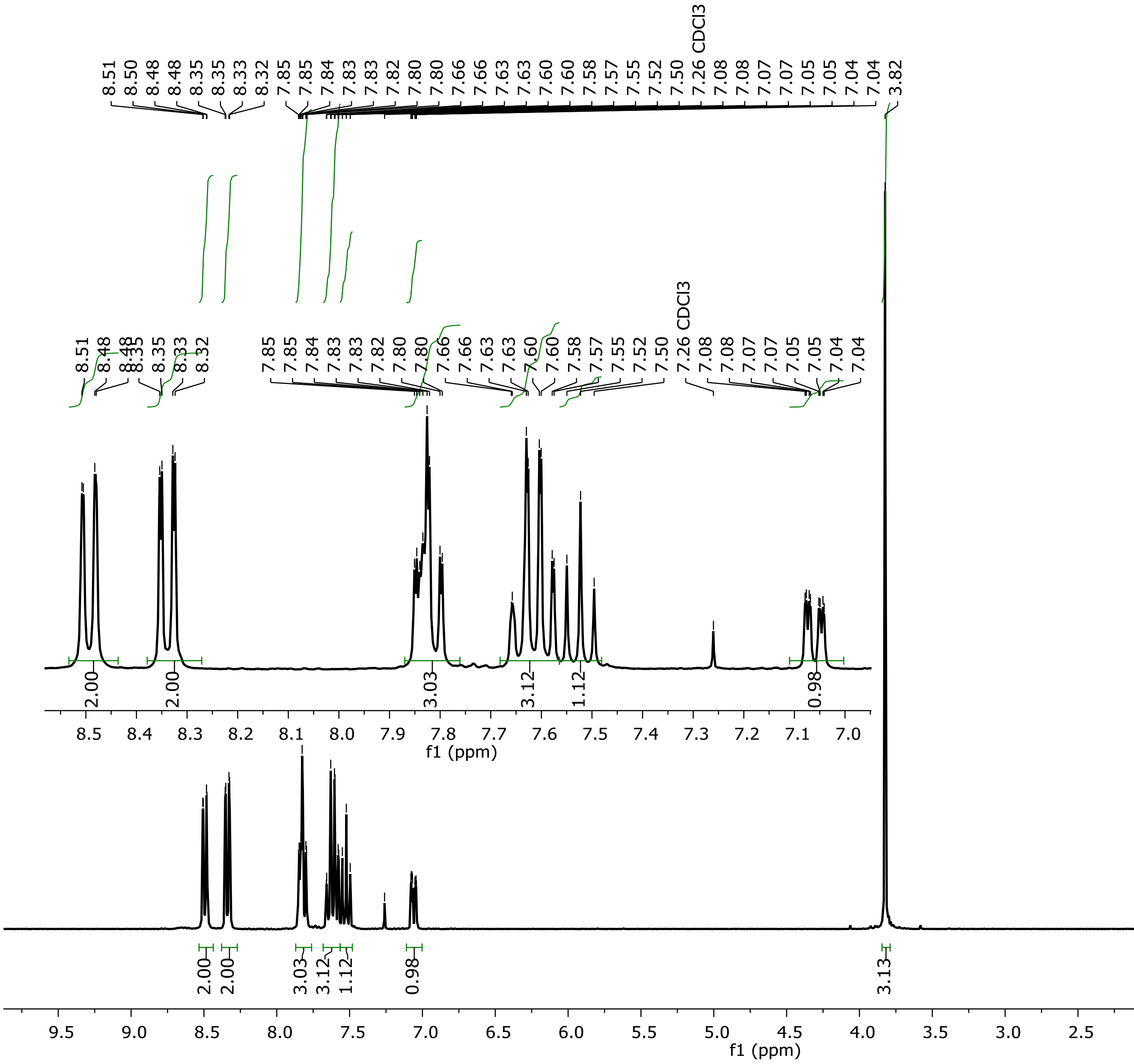

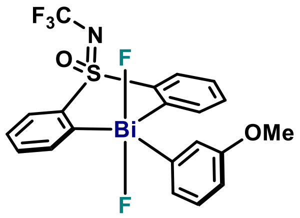

154, ^{1}H NMR, $CDCl_3$

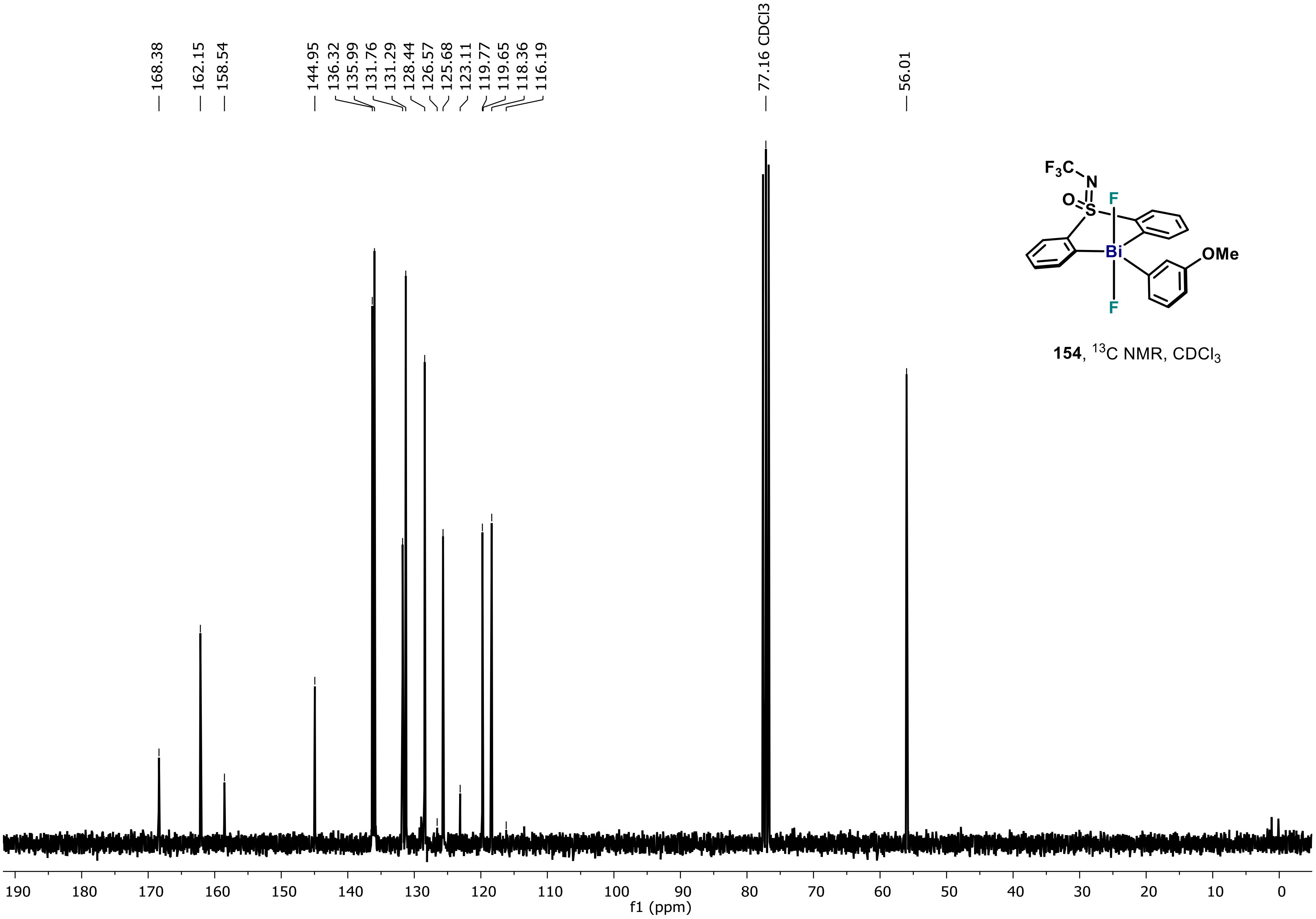

168.38
162.15
158.54
144.95
136.32
135.99
131.76
131.29
128.44
126.57
125.68
123.11
119.77
119.65
118.36
116.19
77.16 CDCl3
56.01
F3C
N
O
S
F
Bi
OMe
F
154, 13C NMR, CDCl3
190
180
170
160
150
140
130
120
110
100
90
80
70
60
50
40
30
20
10
0
f1 (ppm)

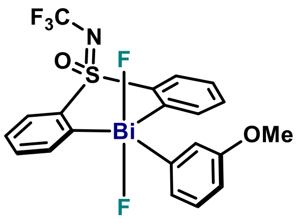

154, ^{19}F NMR, $CDCl_3$

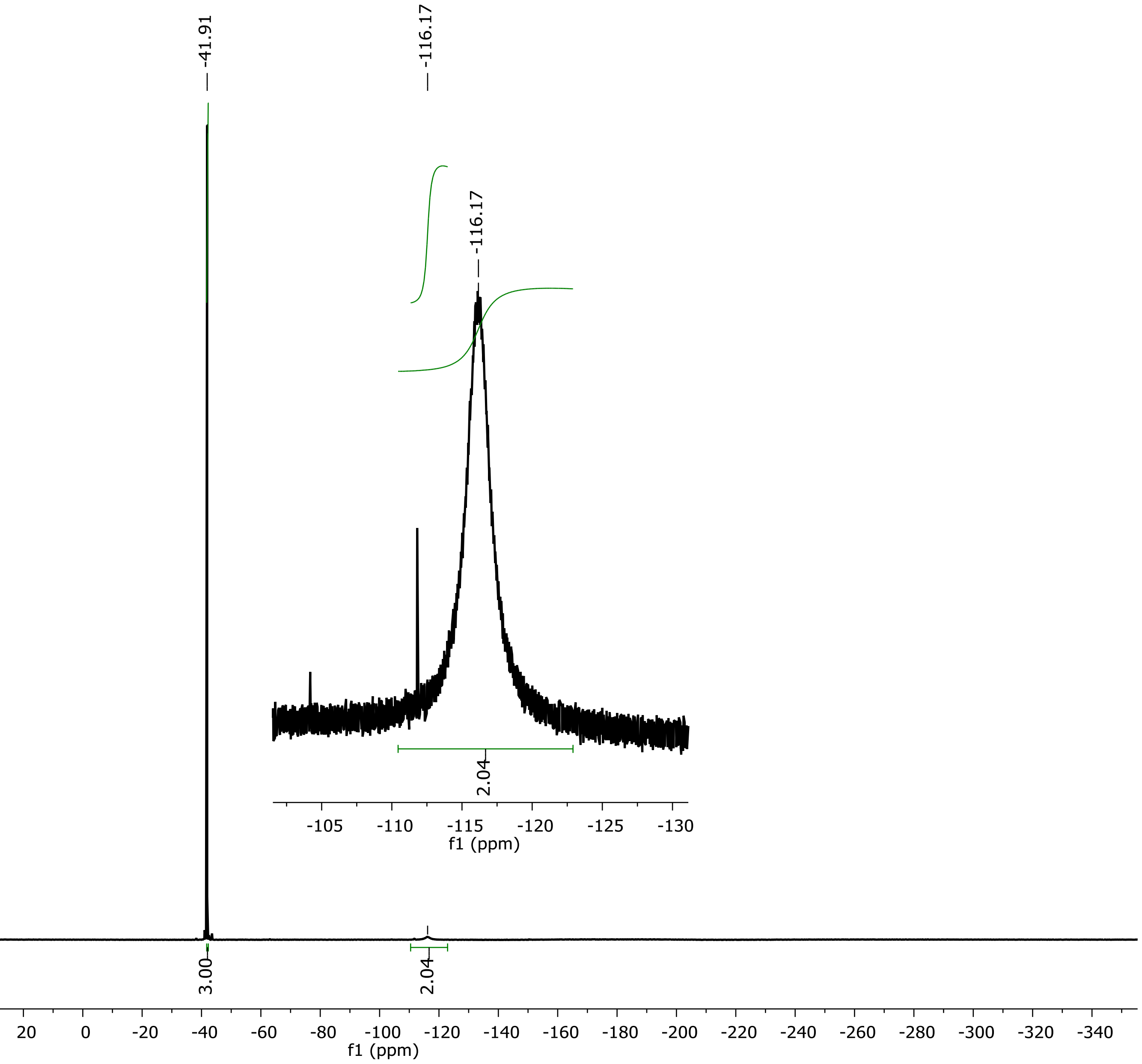

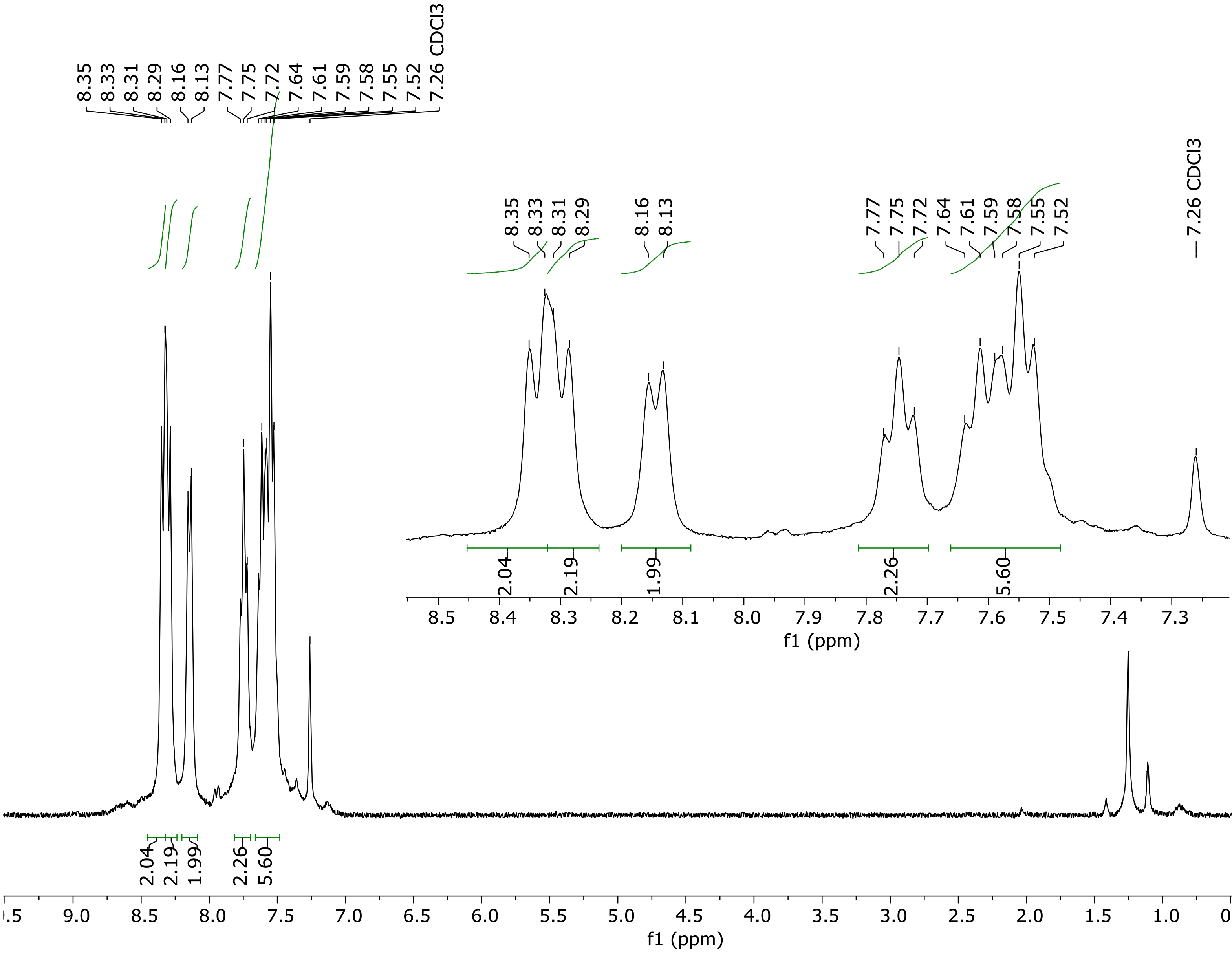

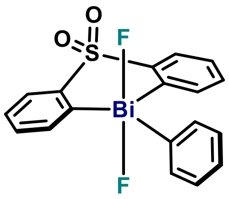

155, ^{1}H NMR, $CDCl_3$

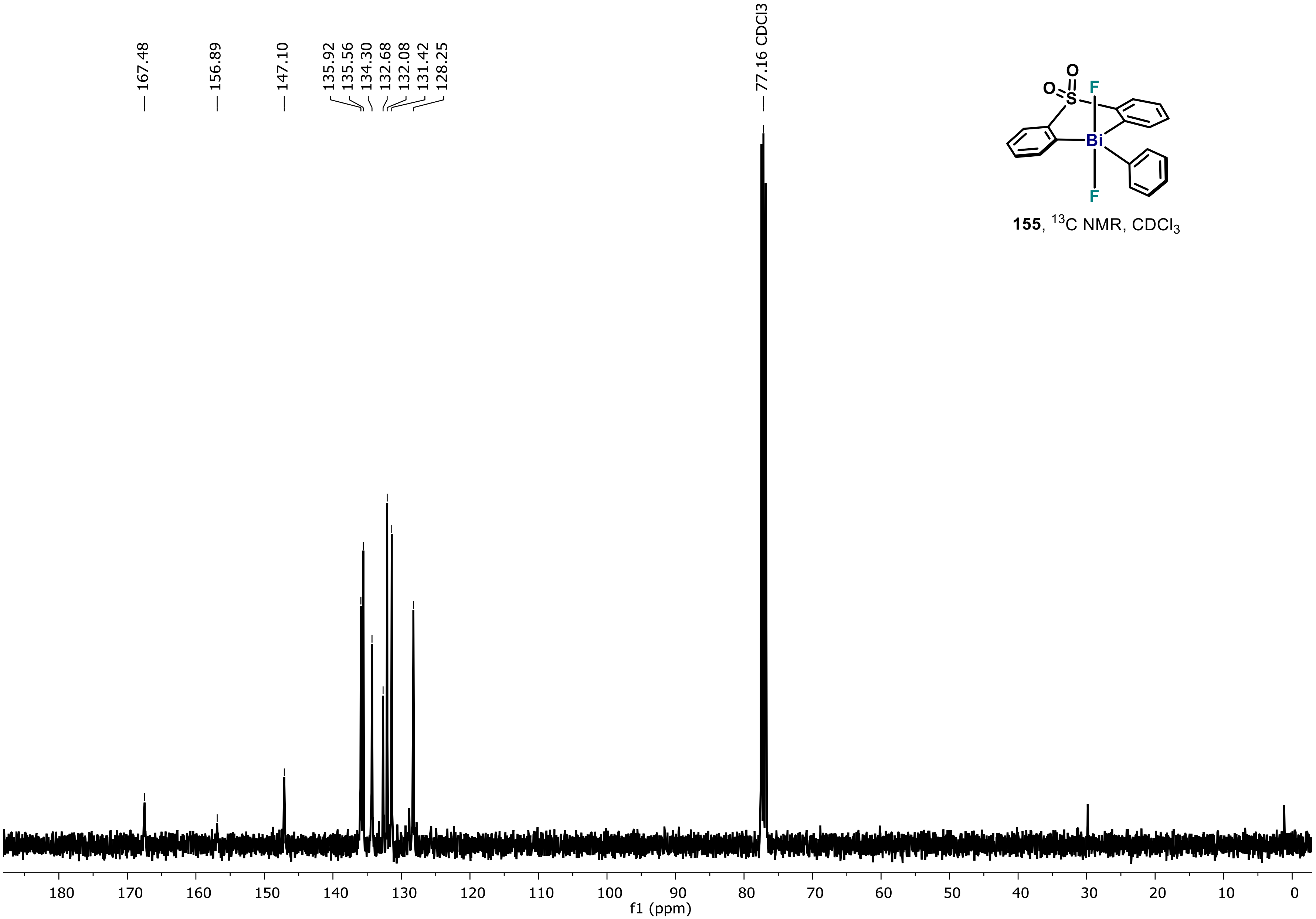

155, ^{13}C NMR, $CDCl_3$

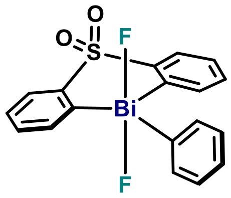

155, ^{19}F NMR, $CDCl_3$

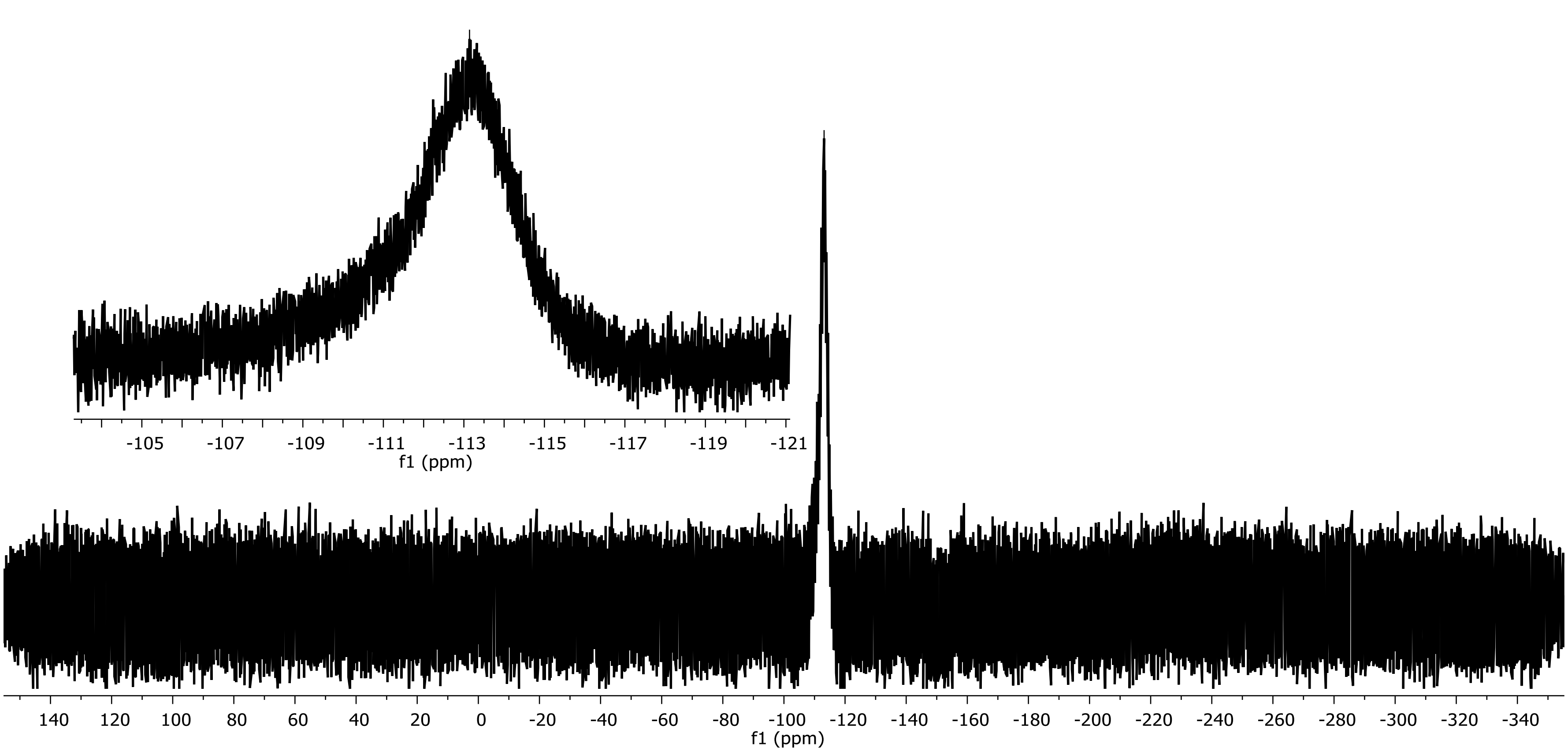

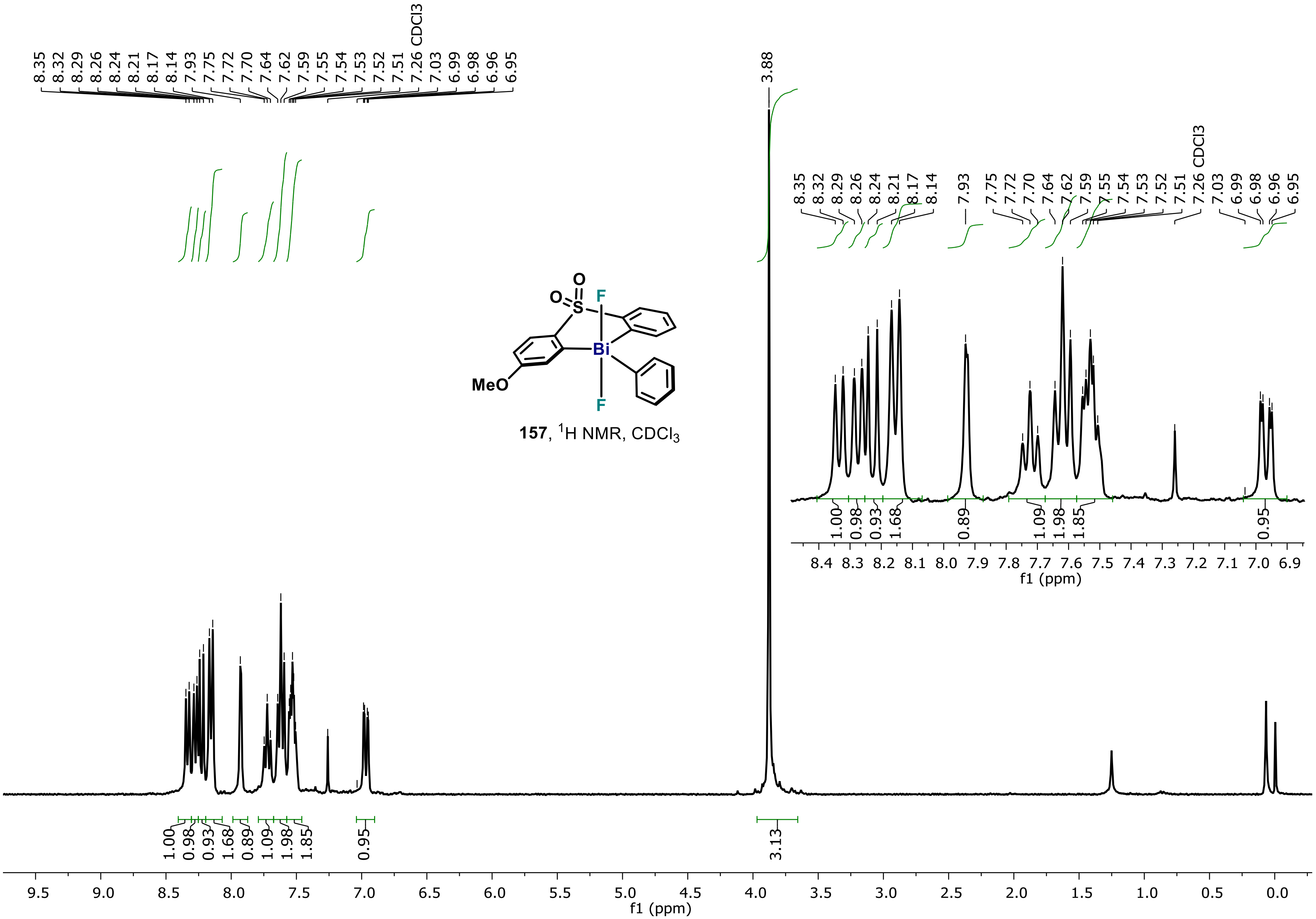
8.35
8.32
8.29
8.26
8.24
8.21
8.17
8.14
7.93
7.75
7.72
7.70
7.64
7.62
7.59
7.55
7.54
7.53
7.52
7.51
7.26 CDCl3
7.03
6.99
6.98
6.96
6.95
3.88
O
O
S
F
Bi
F
MeO
157, ¹H NMR, CDCl₃
1.00
0.98
0.93
1.68
0.89
1.09
1.98
1.85
0.95
3.13
f1 (ppm)

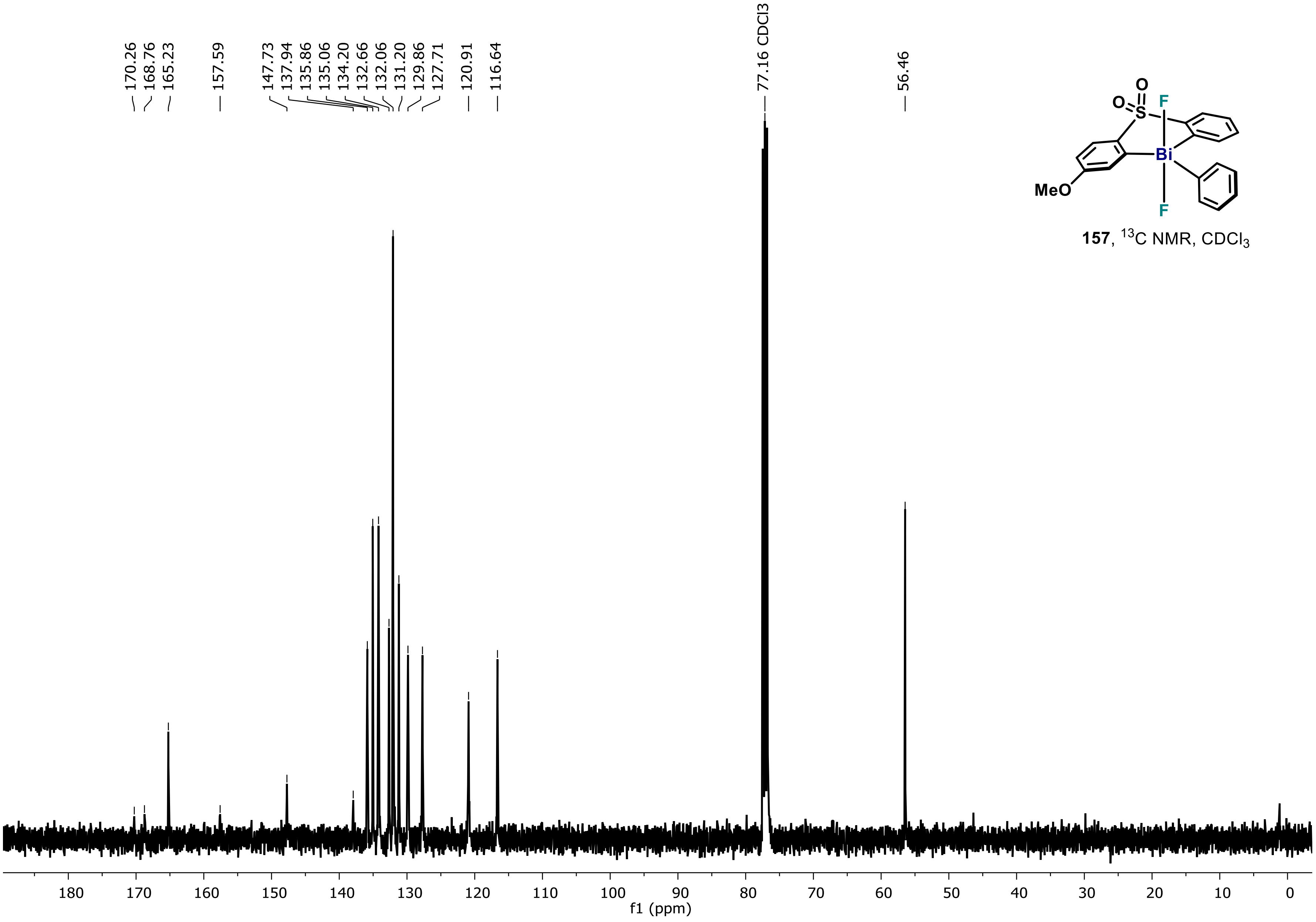
170.26
168.76
165.23
157.59
147.73
137.94
135.86
135.06
134.20
132.66
132.06
131.20
129.86
127.71
120.91
116.64
77.16 CDCl3
56.46
MeO
Bi
F
F
S
O
O
157, ^{13}C NMR, $CDCl_3$
180 170 160 150 140 130 120 110 100 90 80 70 60 50 40 30 20 10 0
f1 (ppm)

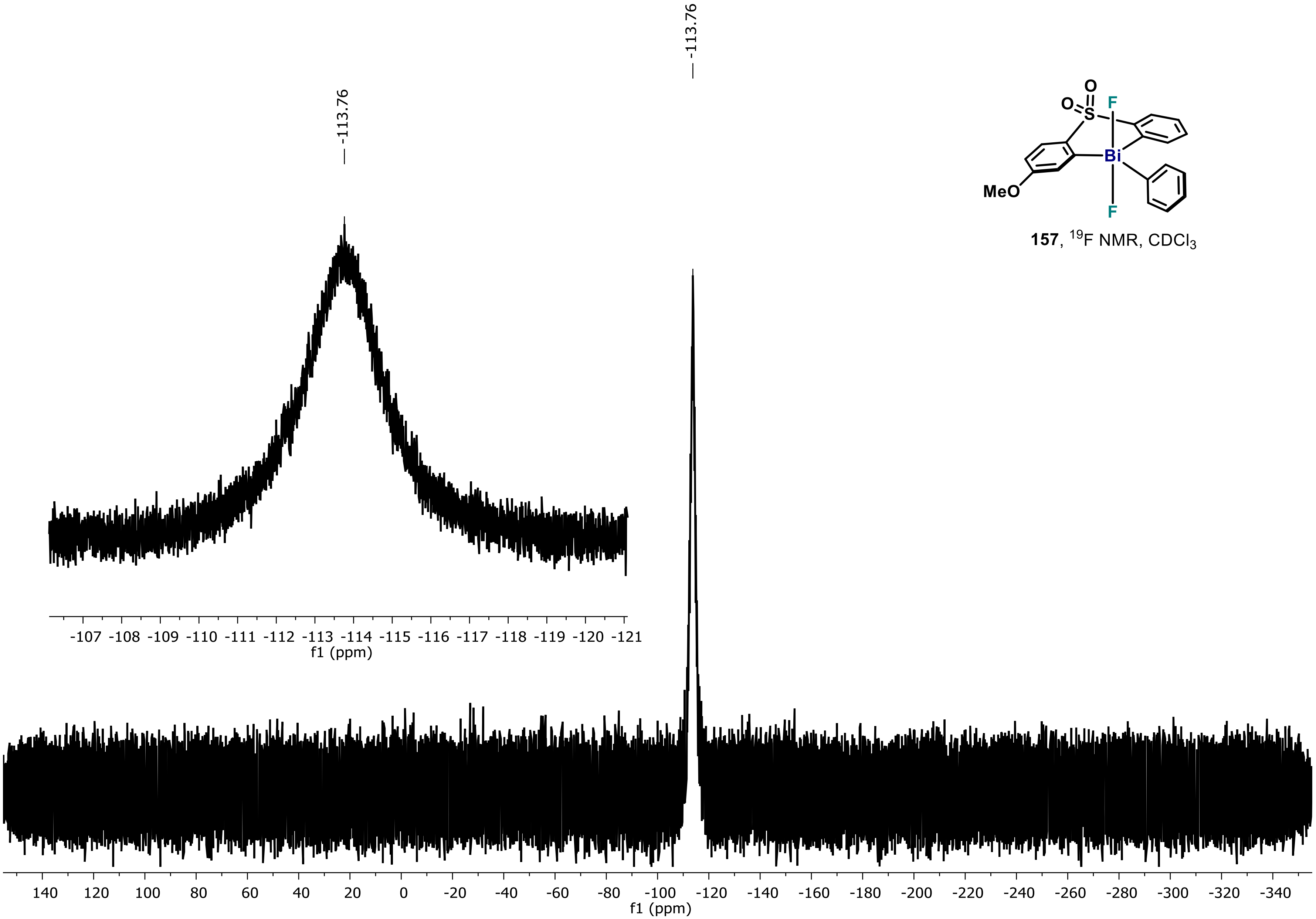

157, ^{19}F NMR, $CDCl_3$

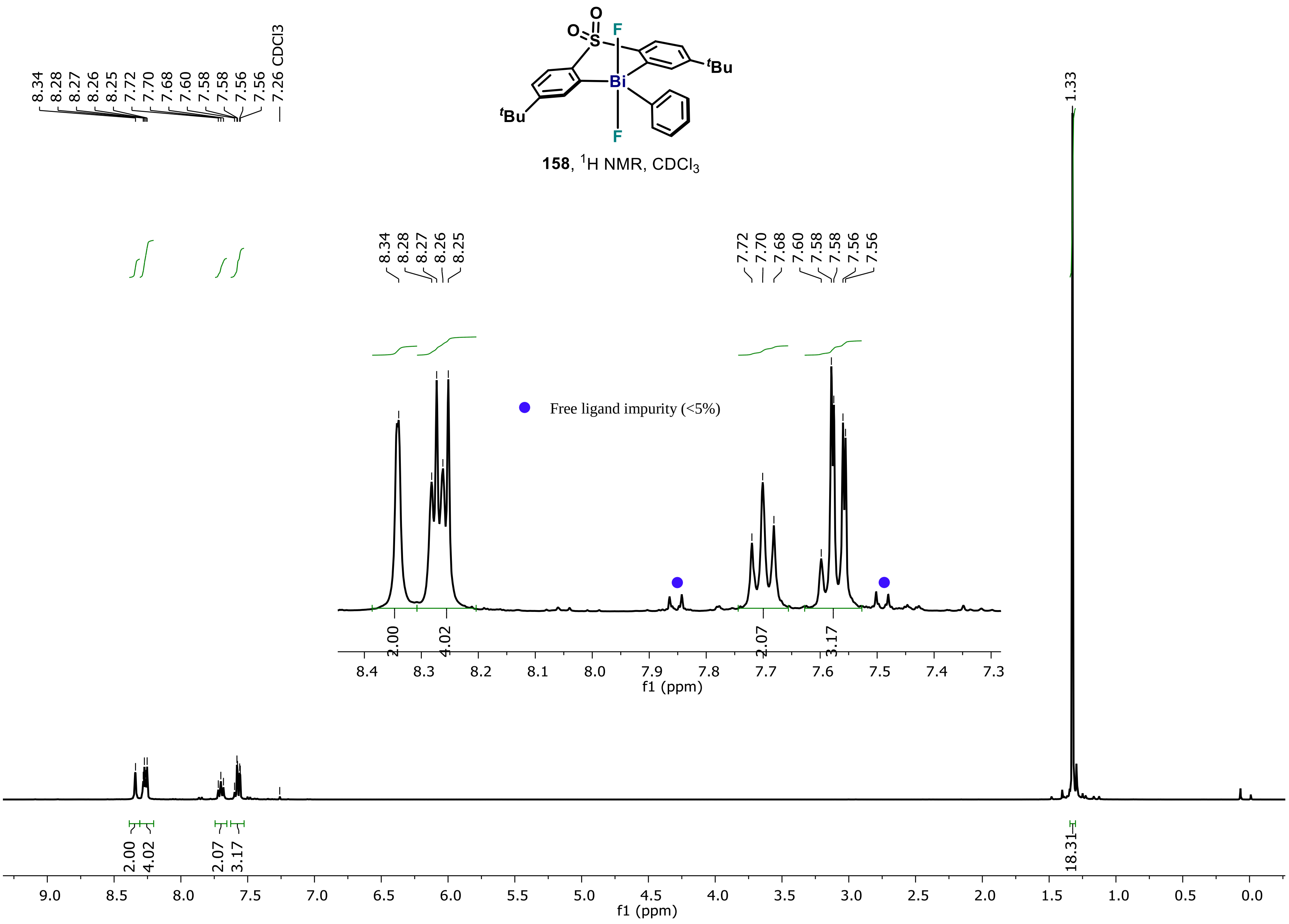

8.34
8.28
8.27
8.26
8.25
7.72
7.70
7.68
7.60
7.58
7.58
7.56
7.56
7.26 CDCl3
1.33
158, 1H NMR, CDCl3
tBu
tBu
Bi
F
F
S
O
O
Free ligand impurity (<5%)
2.00
4.02
2.07
3.17
18.31
f1 (ppm)

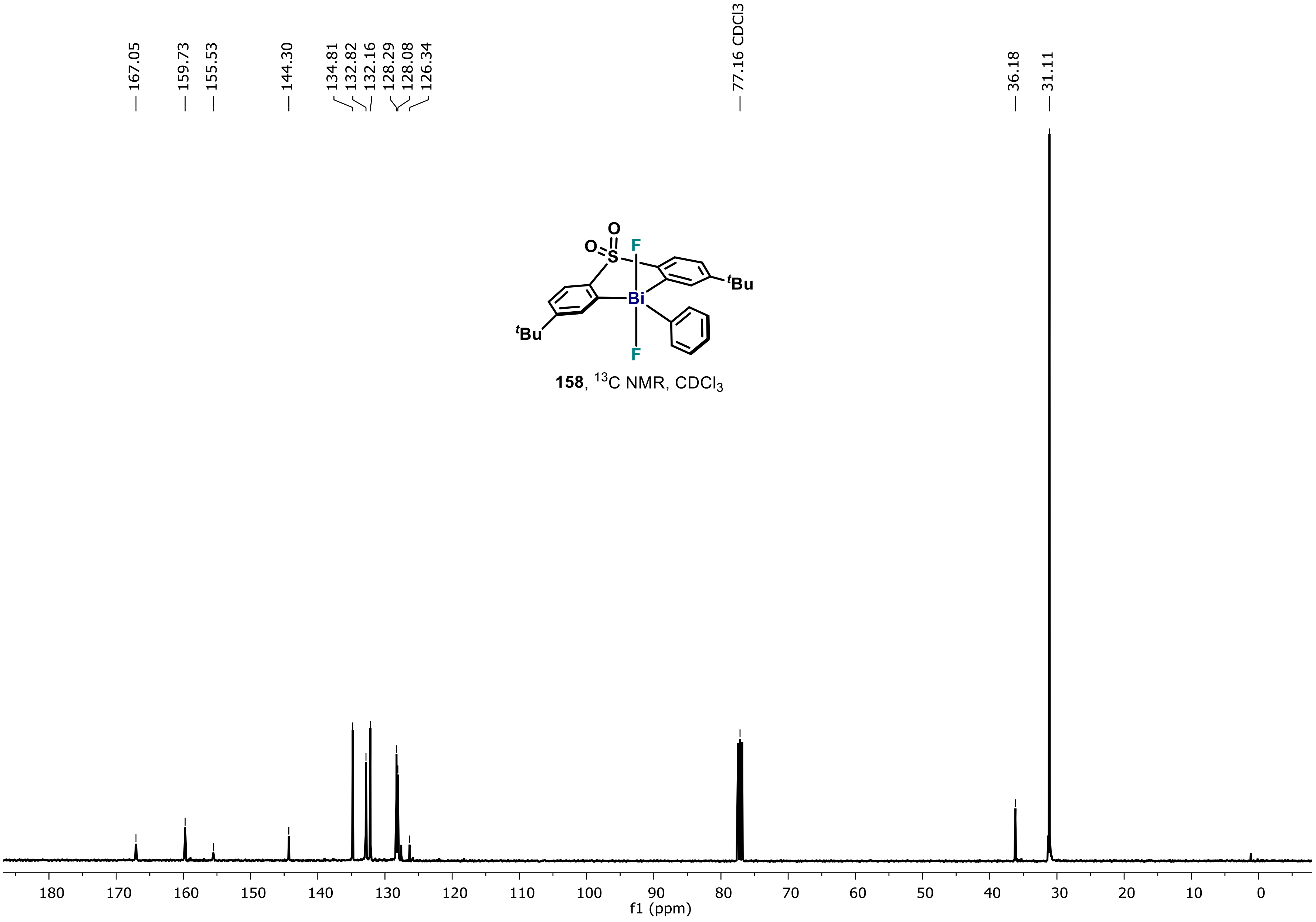

167.05
159.73
155.53
144.30
134.81
132.82
132.16
128.29
128.08
126.34
77.16 CDCl3
36.18
31.11
O
O
S
F
Bi
F
tBu
tBu
158, 13C NMR, CDCl3
180
170
160
150
140
130
120
110
100
90
80
70
60
50
40
30
20
10
0
f1 (ppm)

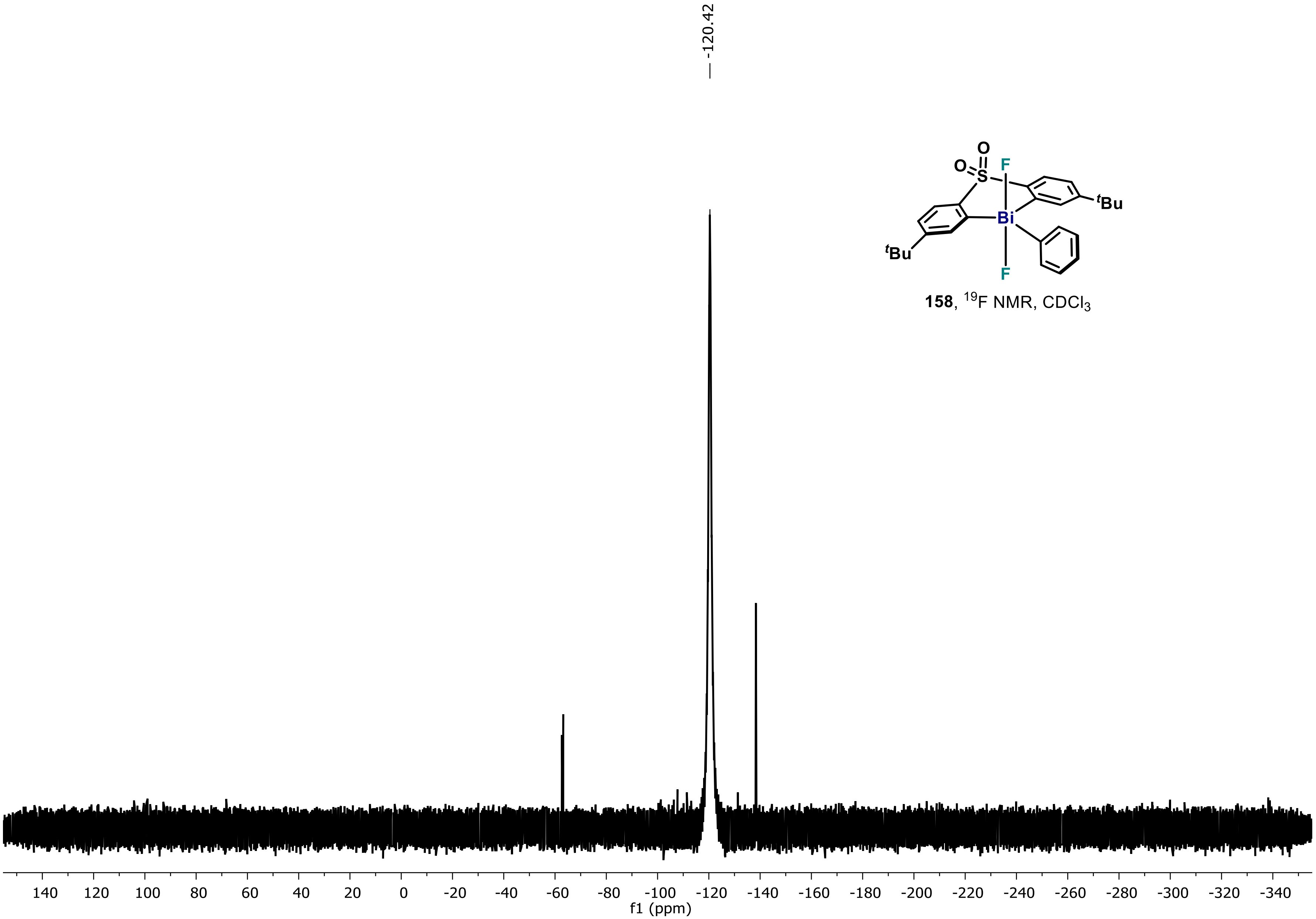
-120.42
O
O
S
F
tBu
Bi
tBu
F
158, ^{19}F NMR, $CDCl_3$
140 120 100 80 60 40 20 0 -20 -40 -60 -80 -100 -120 -140 -160 -180 -200 -220 -240 -260 -280 -300 -320 -340
f1 (ppm)

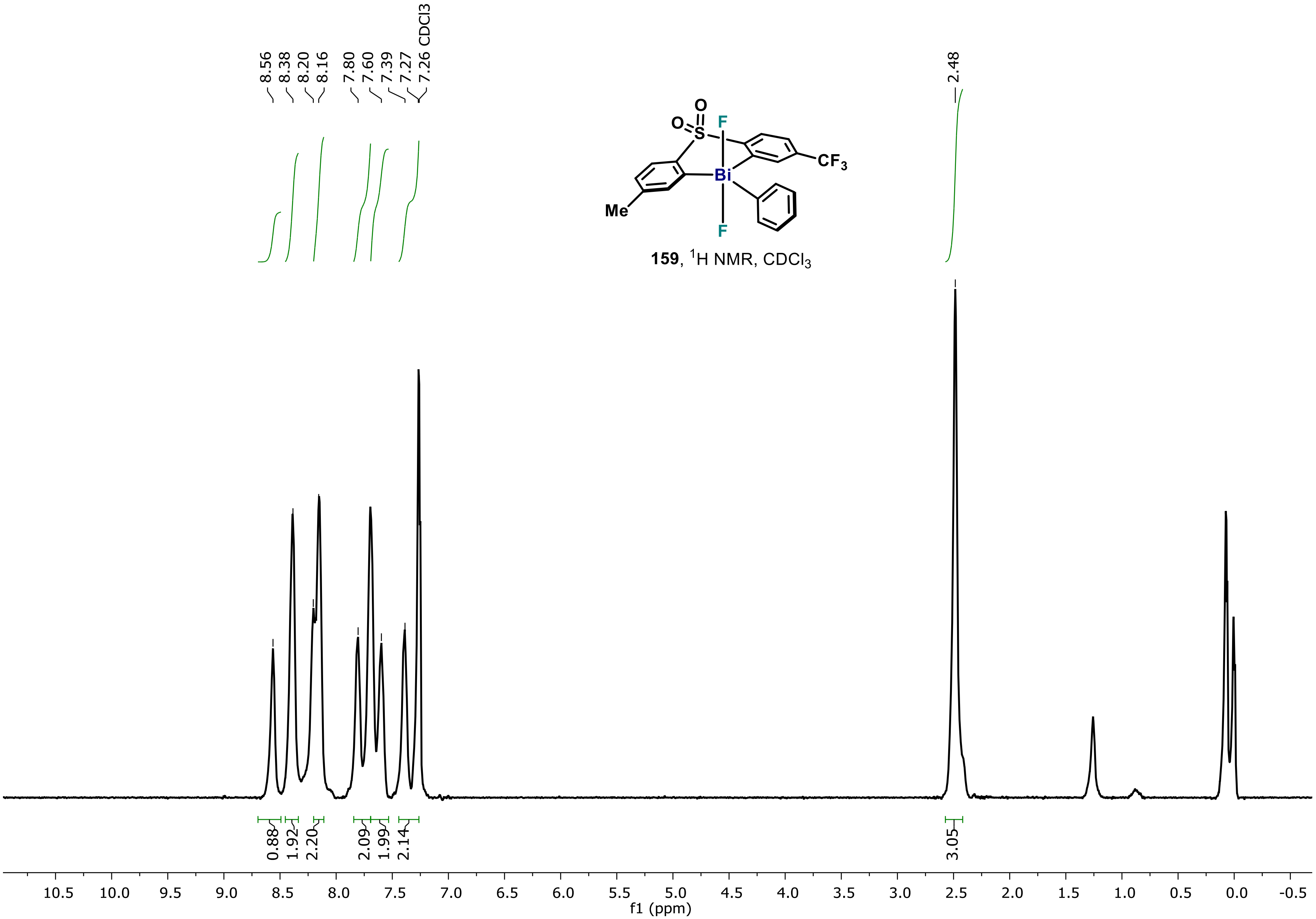
8.56
8.38
8.20
8.16
7.80
7.60
7.39
7.27
7.26 CDCl3
2.48
O
O
S
F
F
Bi
CF3
Me
159, 1H NMR, CDCl3
0.88
1.92
2.20
2.09
1.99
2.14
3.05
10.5
10.0
9.5
9.0
8.5
8.0
7.5
7.0
6.5
6.0
5.5
5.0
4.5
4.0
3.5
3.0
2.5
2.0
1.5
1.0
0.5
0.0
-0.5
f1 (ppm)

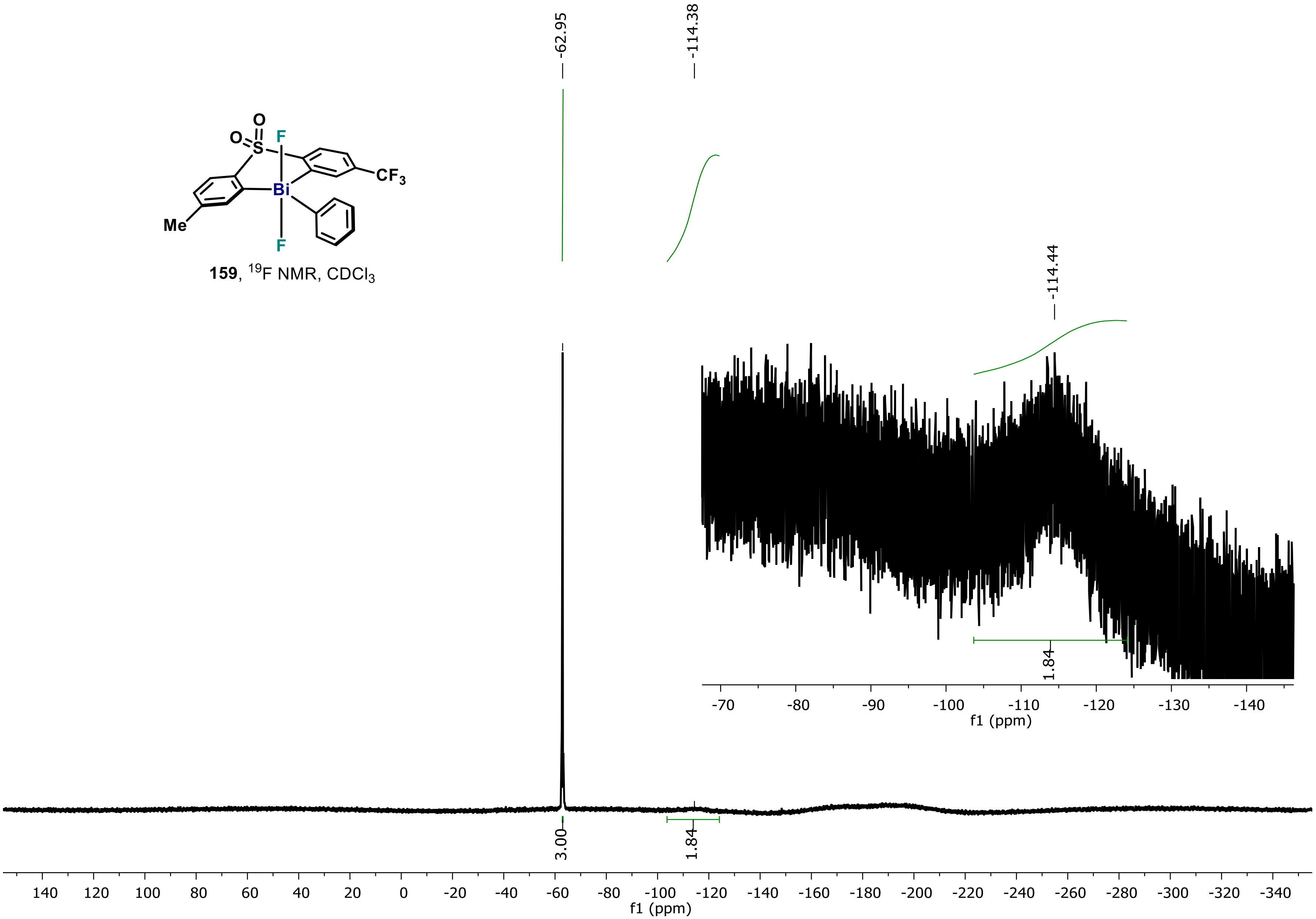

159, ^{19}F NMR, $CDCl_3$

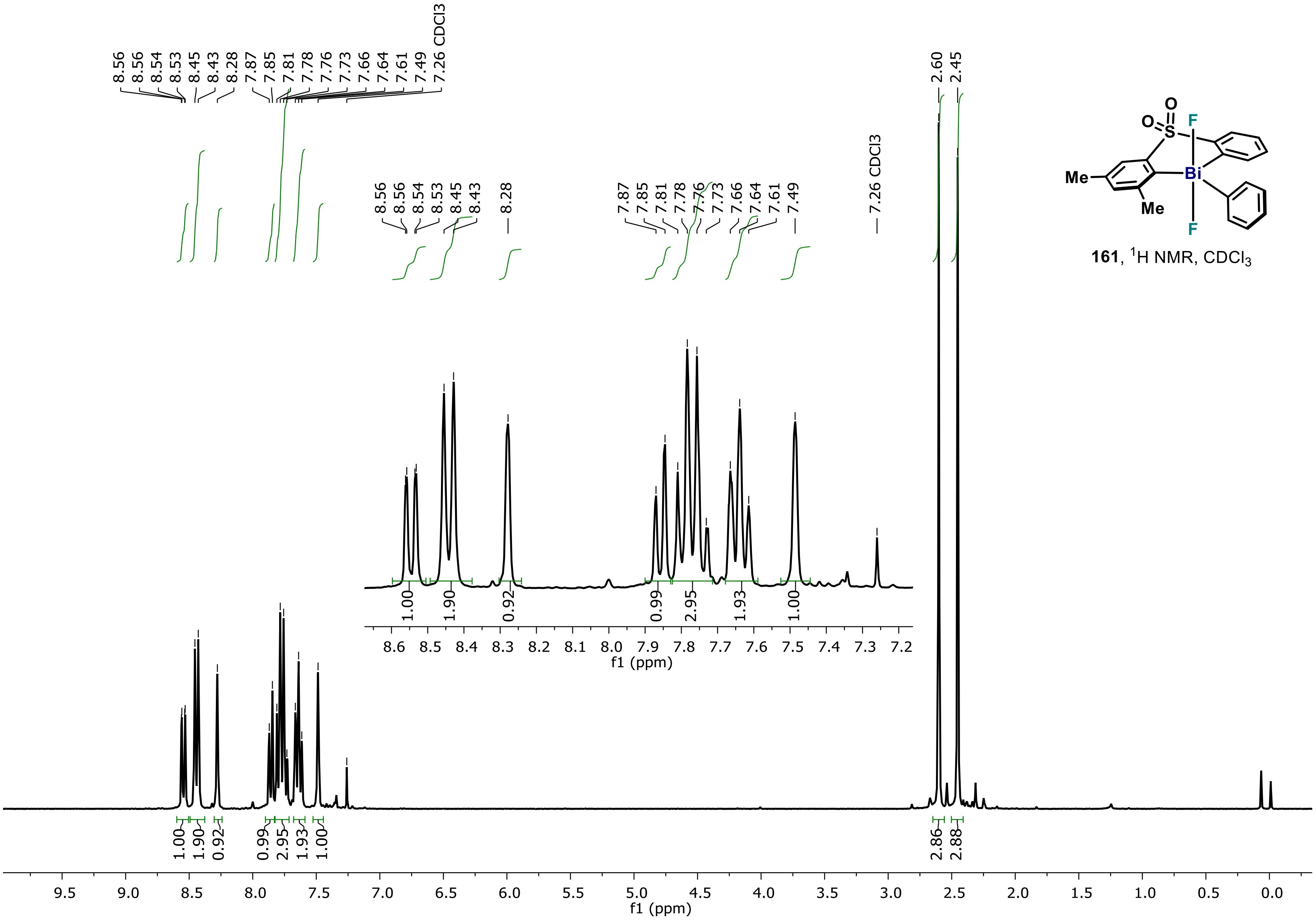
8.56 8.56 8.54 8.53 8.45 8.43 8.28 7.87 7.85 7.81 7.78 7.76 7.73 7.66 7.64 7.61 7.49 7.26 CDCl3
2.60 2.45
Me
Me
O
O
S
F
Bi
F
161, 1H NMR, CDCl3
1.00 1.90 0.92 0.99 2.95 1.93 1.00
2.86 2.88
f1 (ppm)

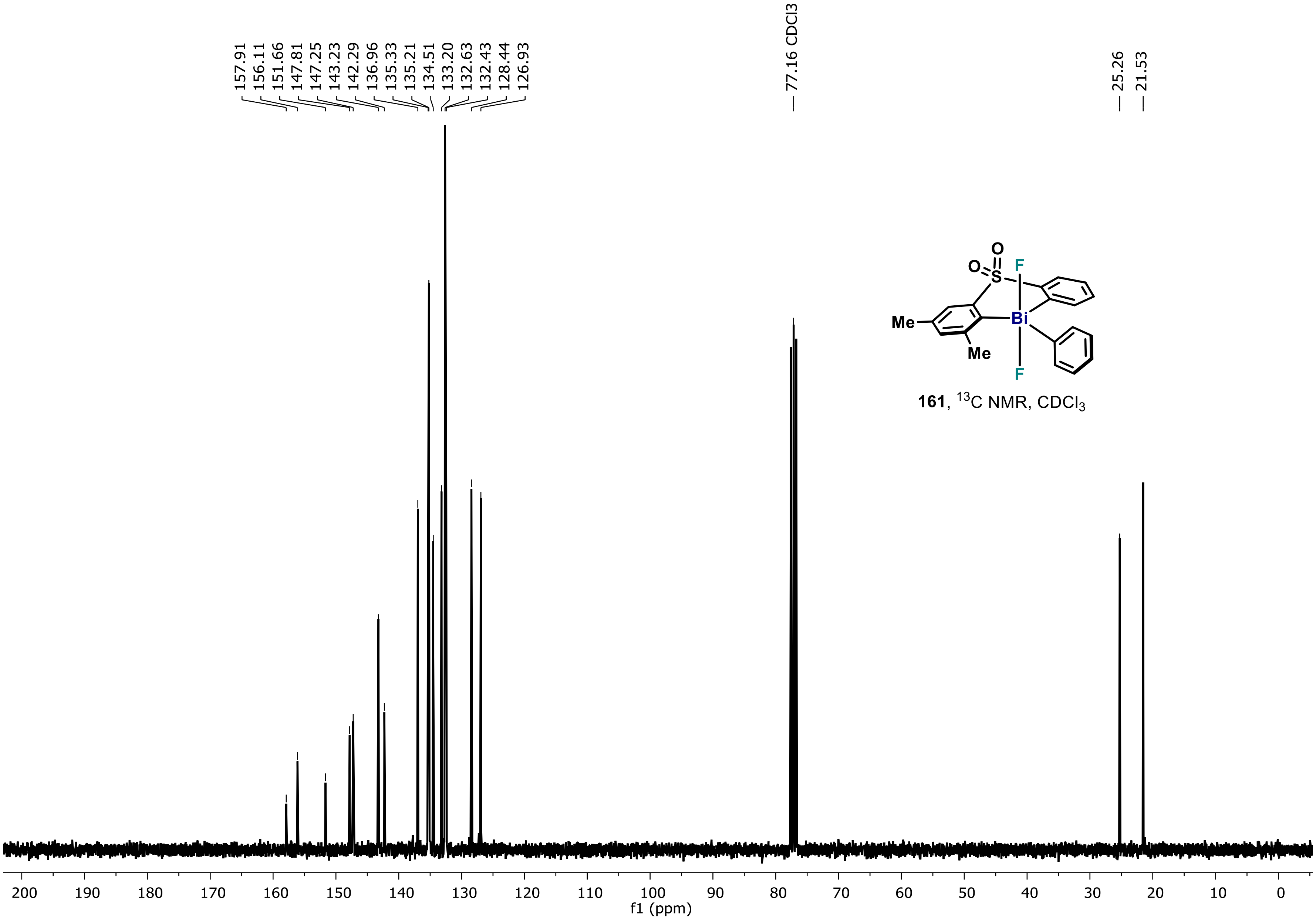

161, ^{13}C NMR, $CDCl_3$

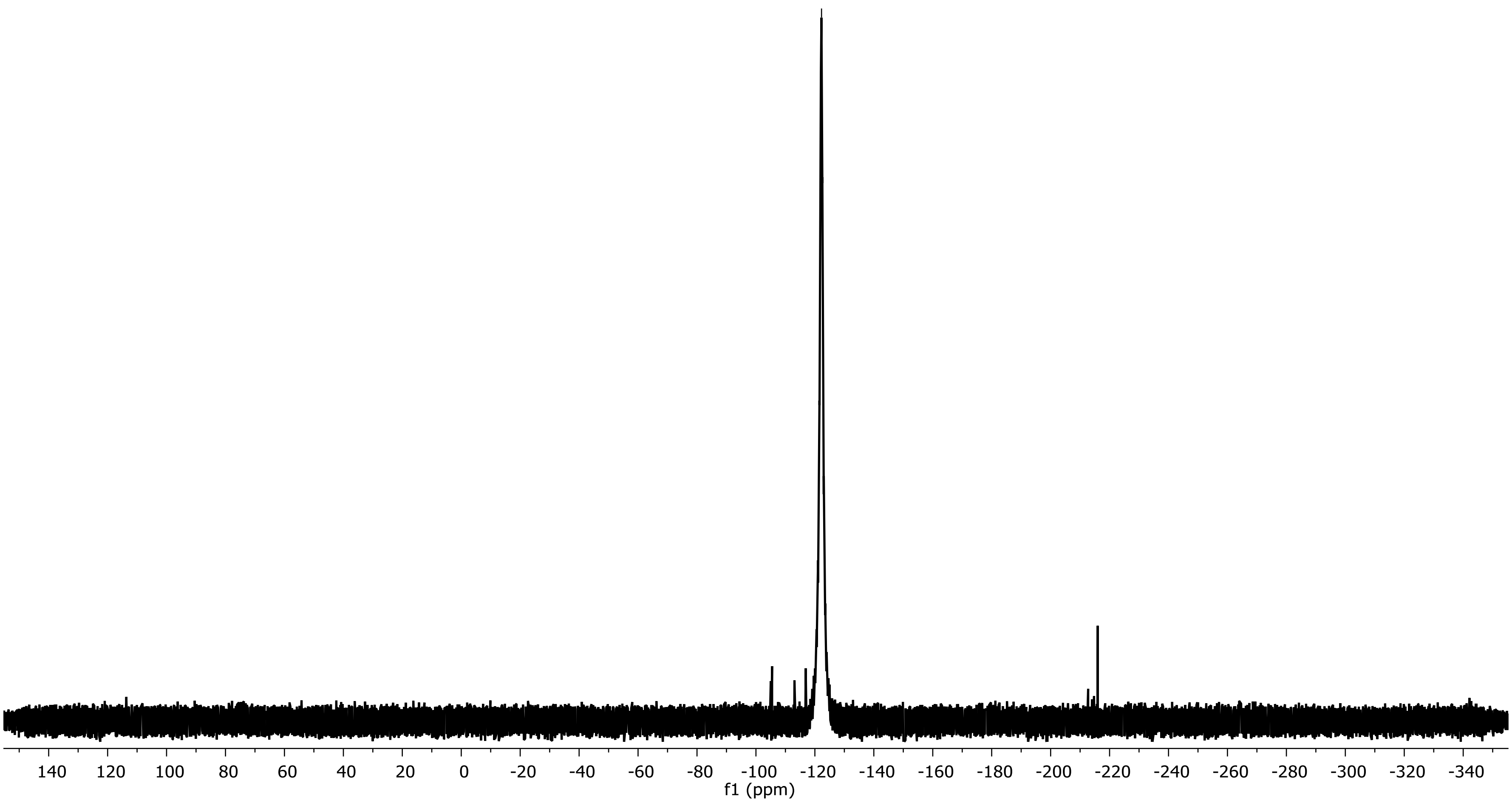

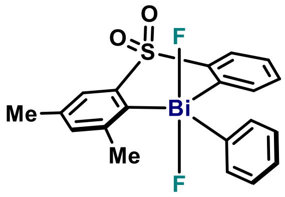

161, ^{19}F NMR, $CDCl_3$

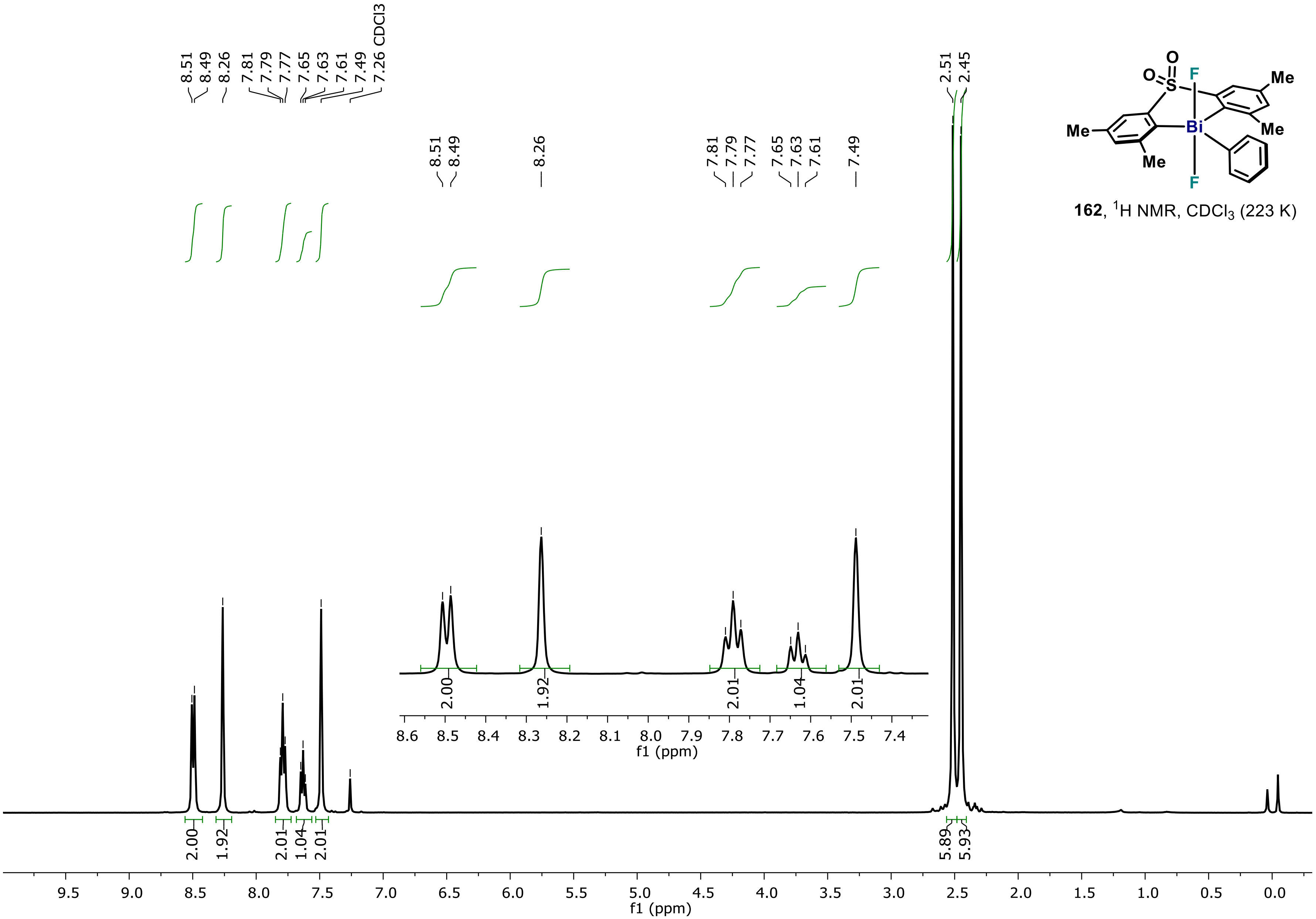

162, ^{1}H NMR, $CDCl_3$ (223 K)

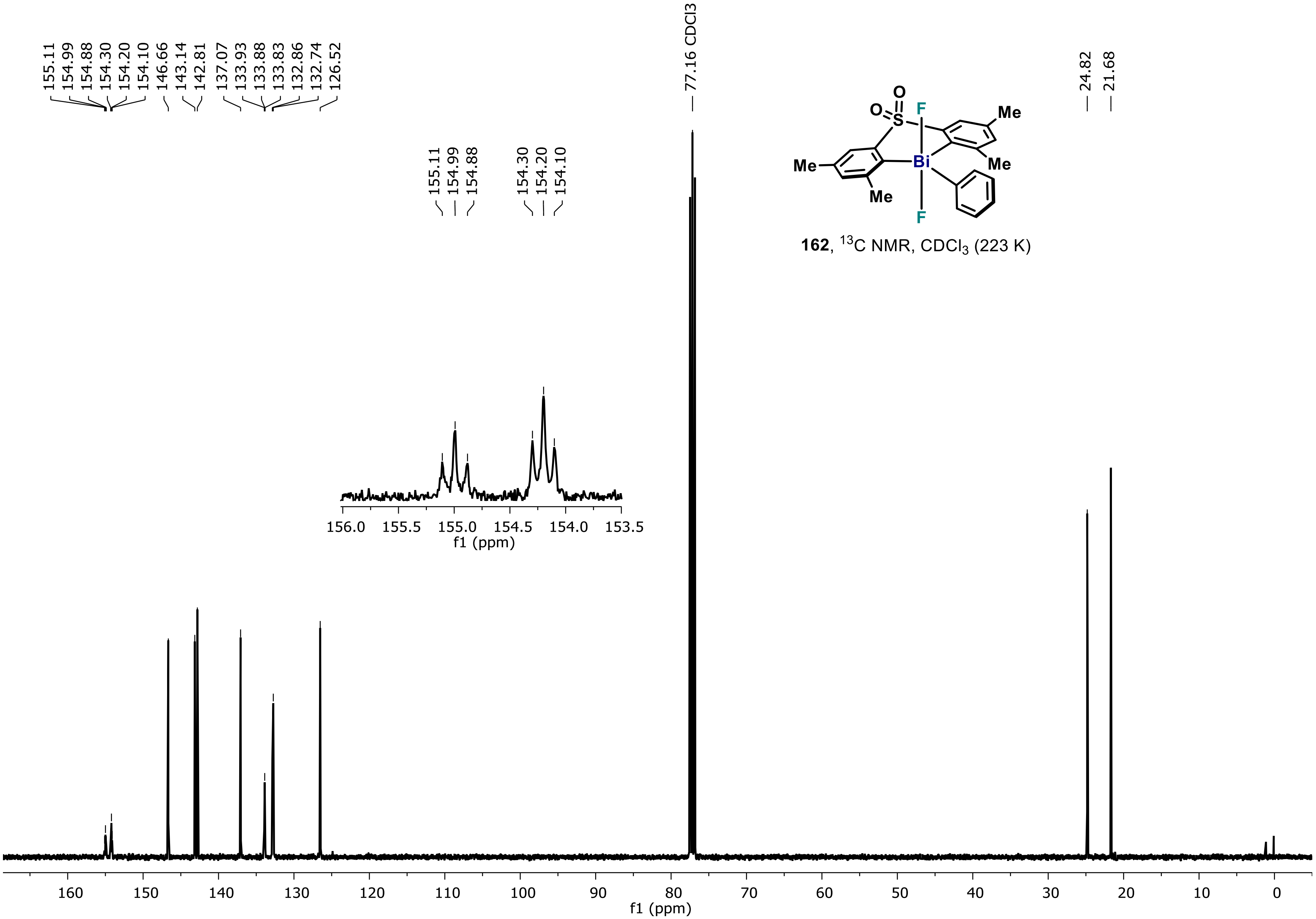

162, ^{13}C NMR, $CDCl_3$ (223 K)

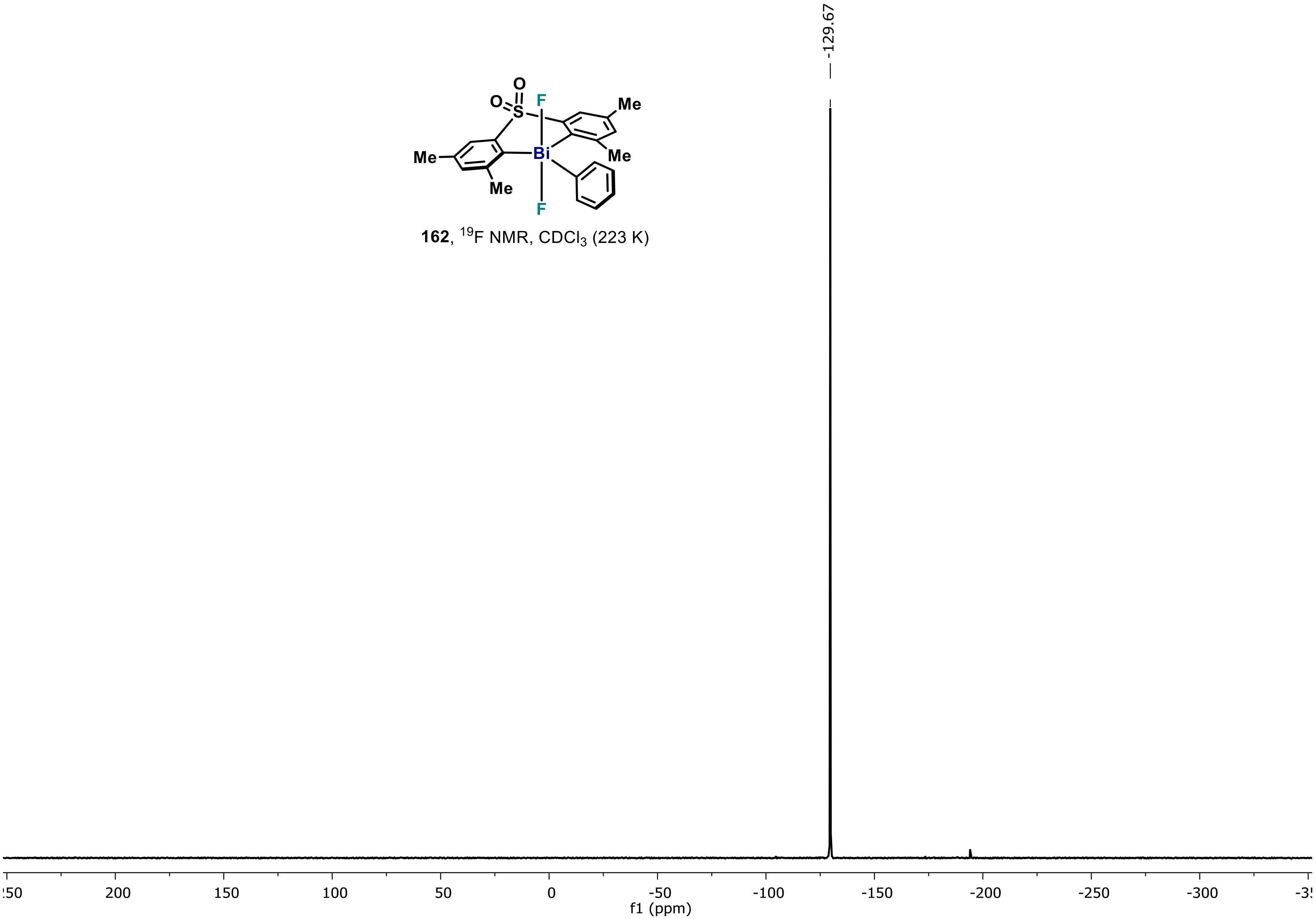

162, ^{19}F NMR, $CDCl_3$ (223 K)

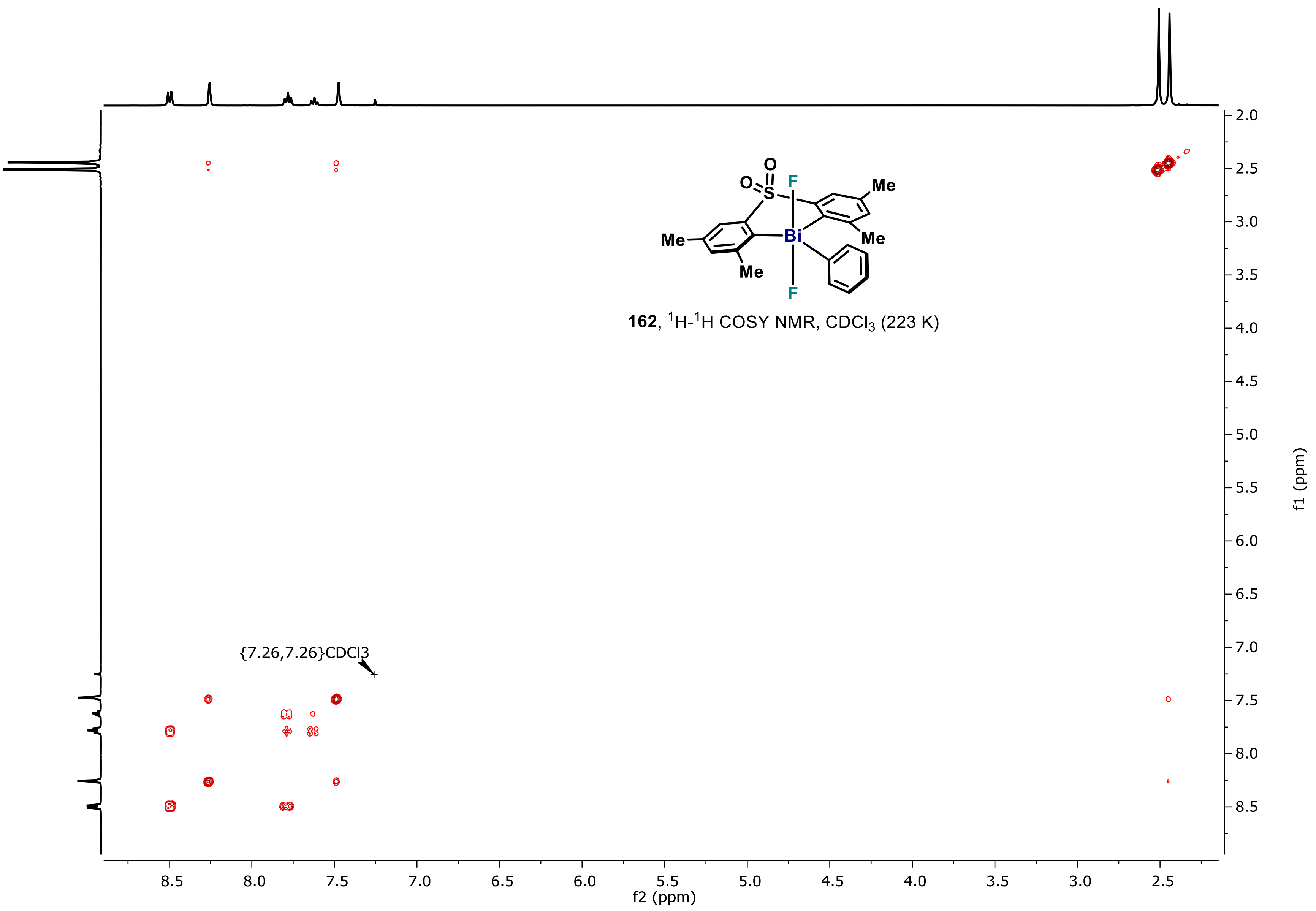
O
O
S
F
Me
Me
Bi
Me
Me
F
162, ^{1}H-^{1}H COSY NMR, CDCl$_3$ (223 K)
{7.26,7.26}CDCl3
f2 (ppm)
f1 (ppm)
8.5
8.0
7.5
7.0
6.5
6.0
5.5
5.0
4.5
4.0
3.5
3.0
2.5
2.0

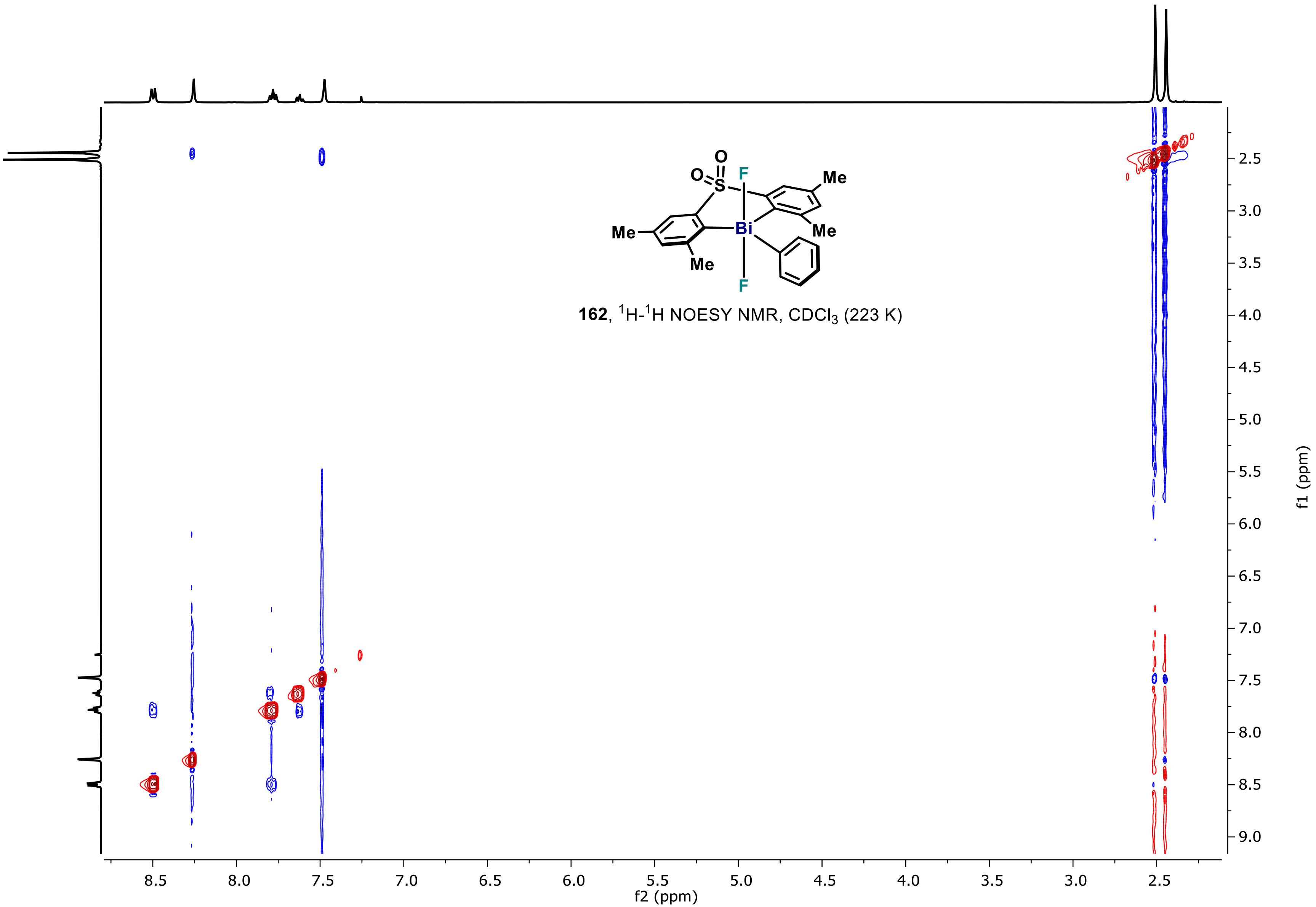
O
O
S
F
Me
Me
Bi
Me
Me
F
162, ¹H-¹H NOESY NMR, CDCl₃ (223 K)
f1 (ppm)
f2 (ppm)
8.5
8.0
7.5
7.0
6.5
6.0
5.5
5.0
4.5
4.0
3.5
3.0
2.5
9.0

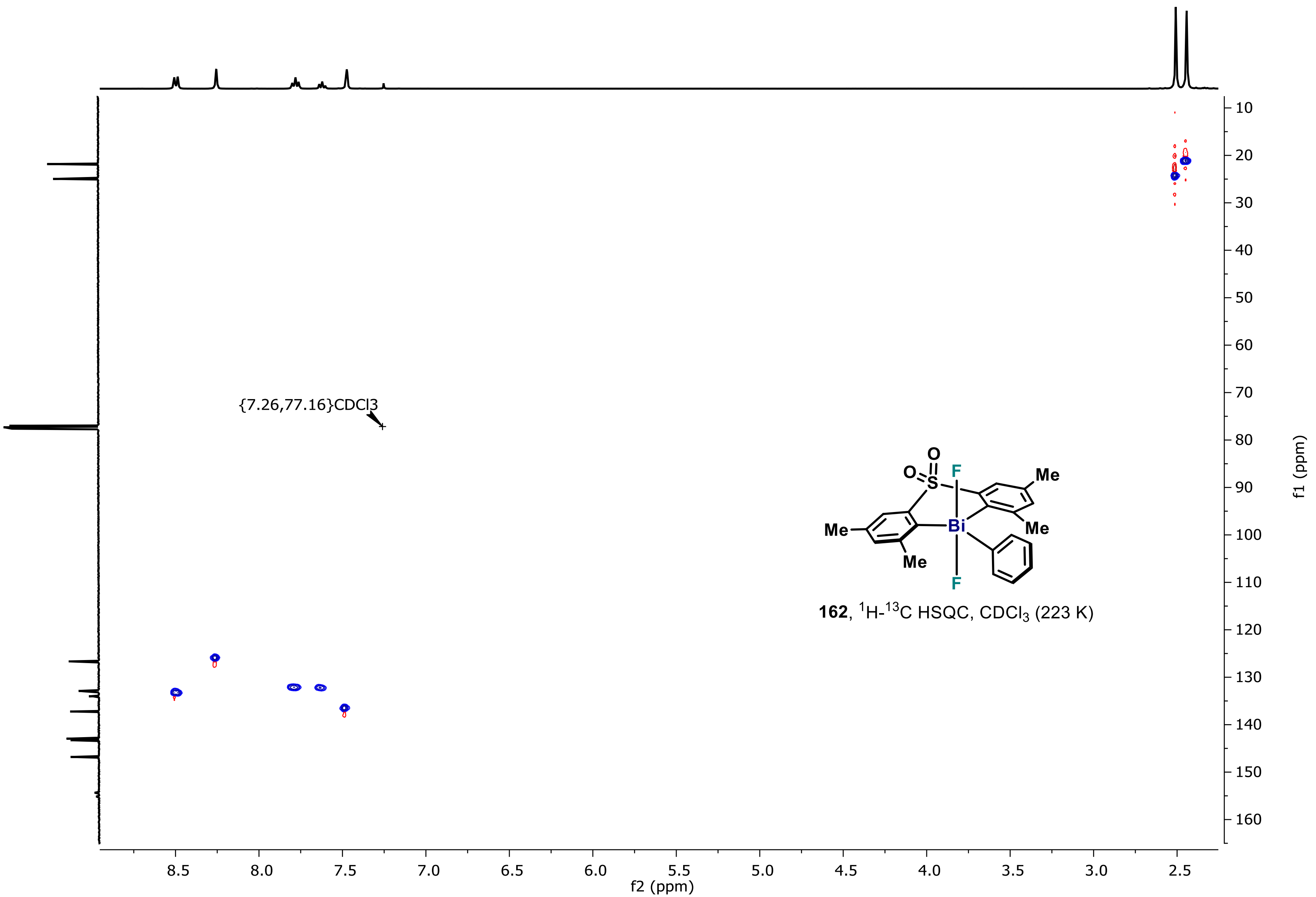

162, ^{1}H-^{13}C HSQC, $CDCl_3$ (223 K)

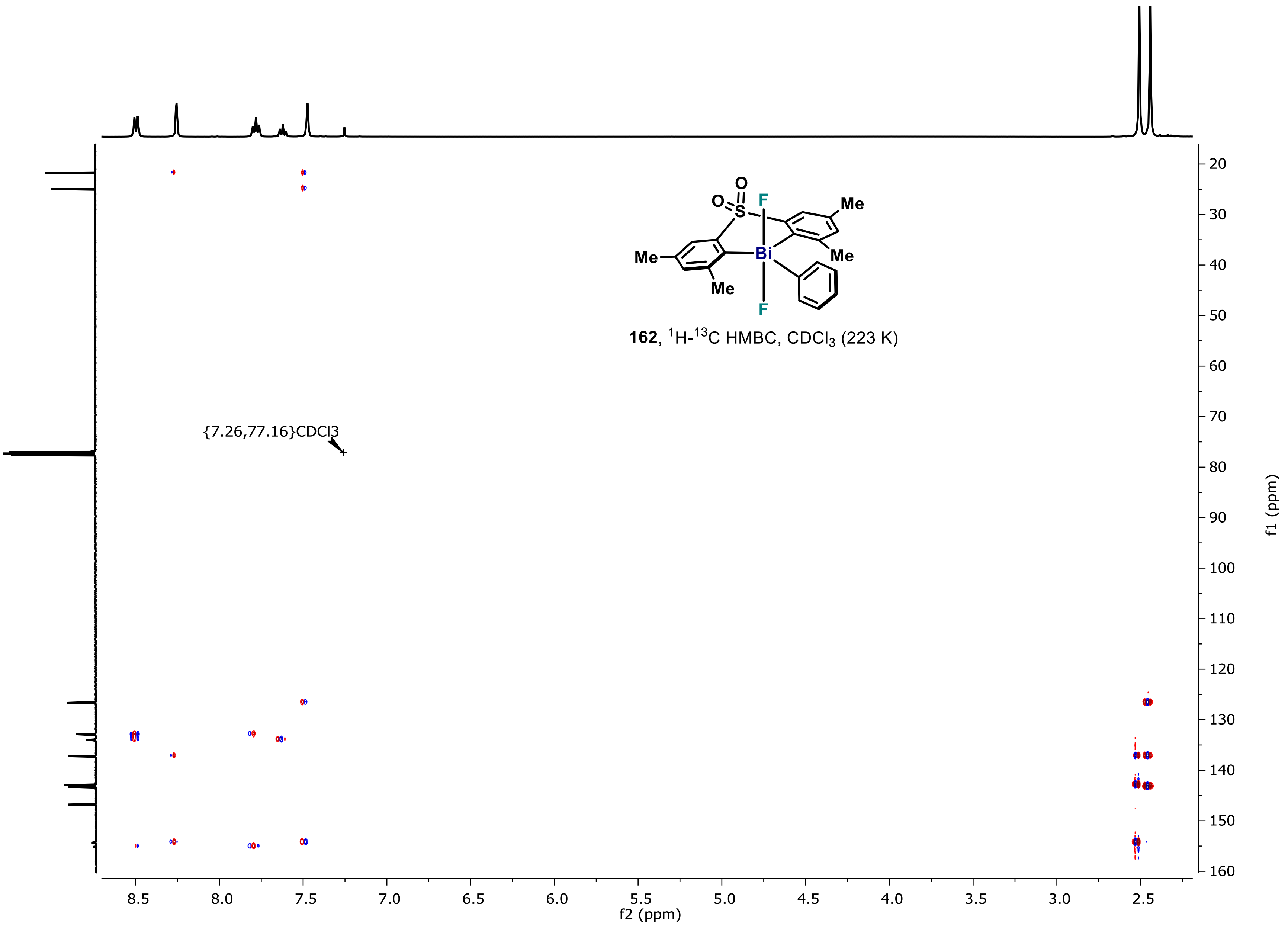

162, ^{1}H-^{13}C HMBC, $CDCl_3$ (223 K)

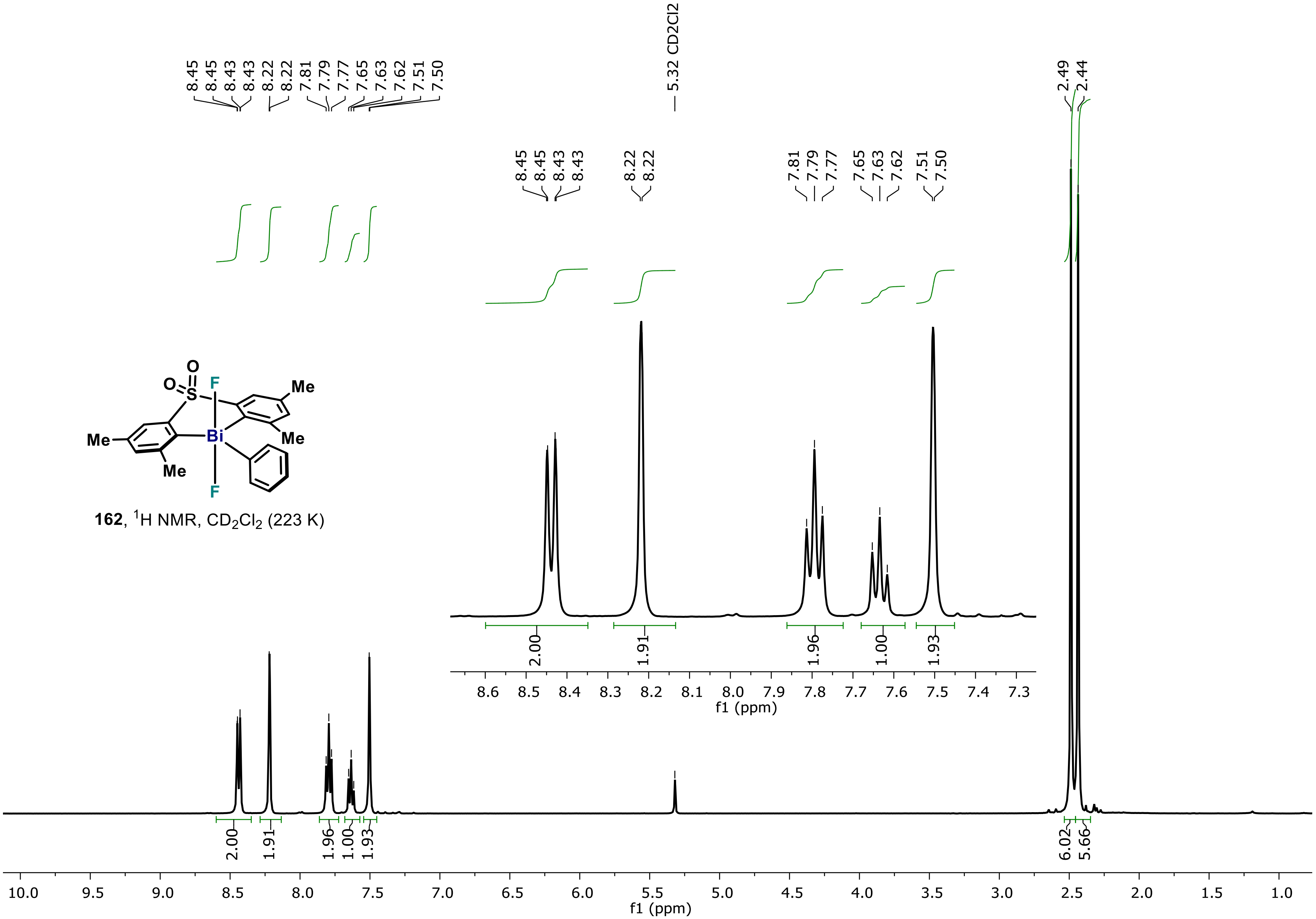

162, ^{1}H NMR, CD_2Cl_2 (223 K)

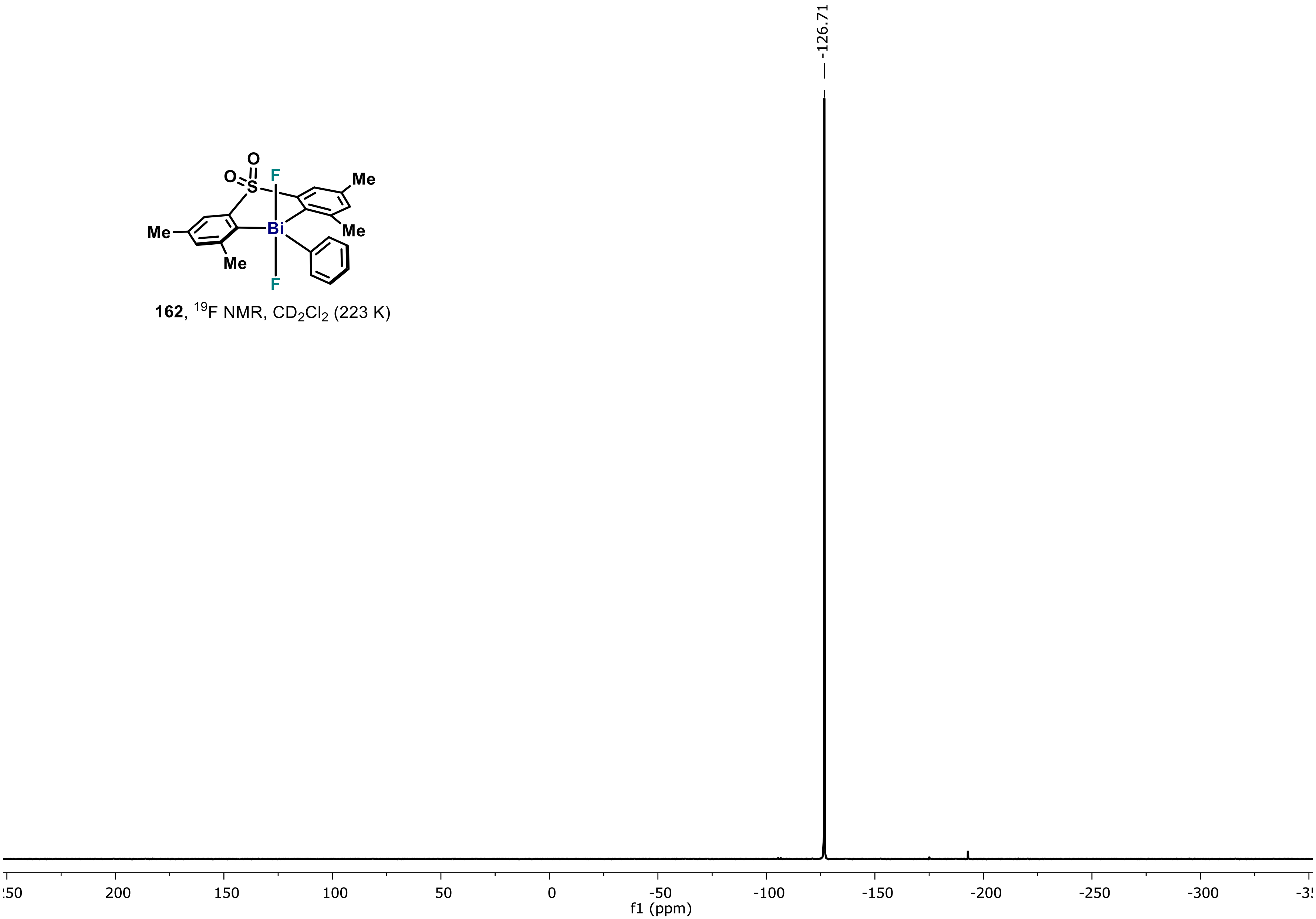

162, ^{19}F NMR, CD_2Cl_2 (223 K)

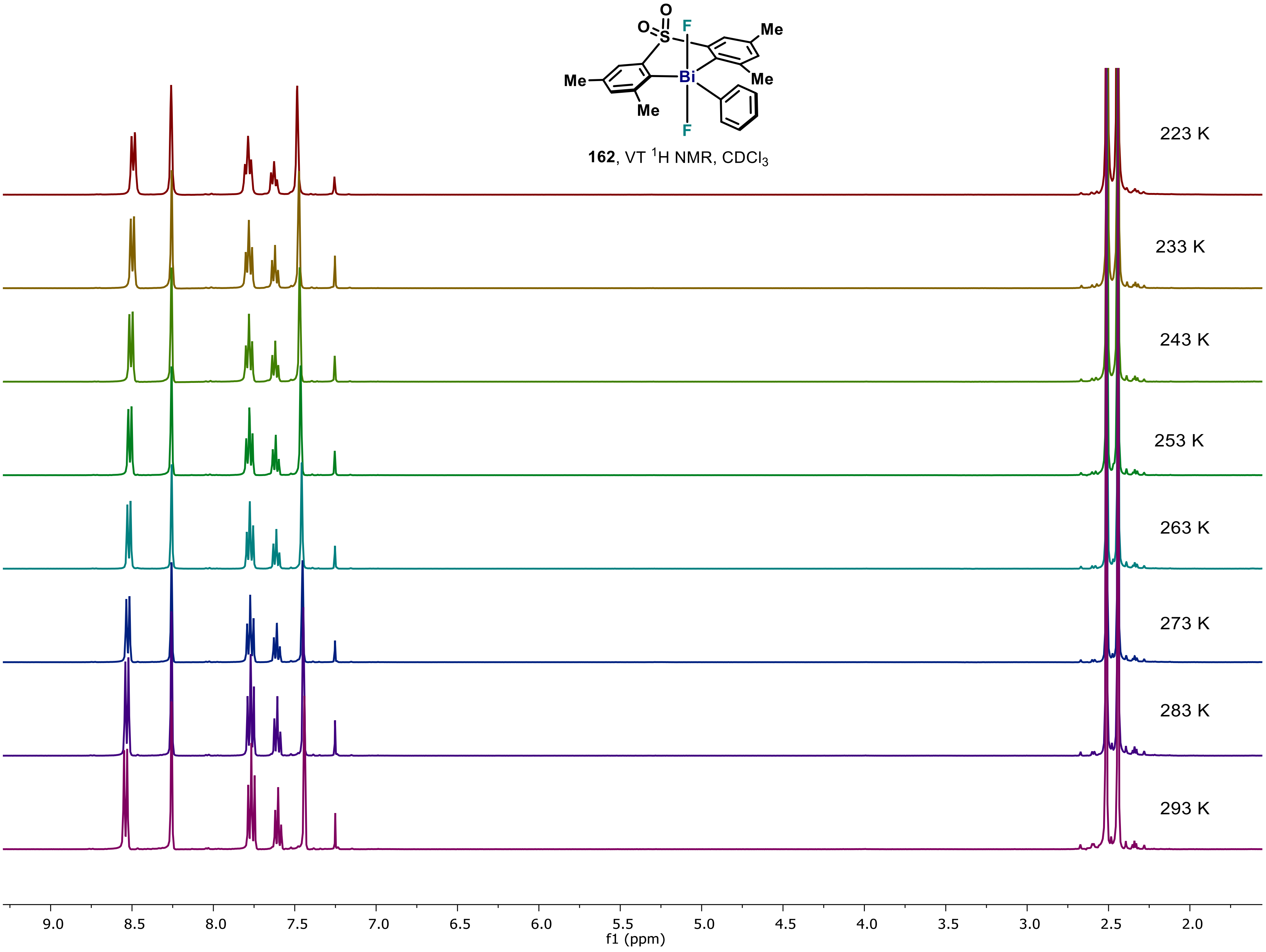

162, VT ^{1}H NMR, $CDCl_3$
223 K
233 K
243 K
253 K
263 K
273 K
283 K
293 K
9.0
8.5
8.0
7.5
7.0
6.5
6.0
5.5
5.0
4.5
4.0
3.5
3.0
2.5
2.0
f1 (ppm)

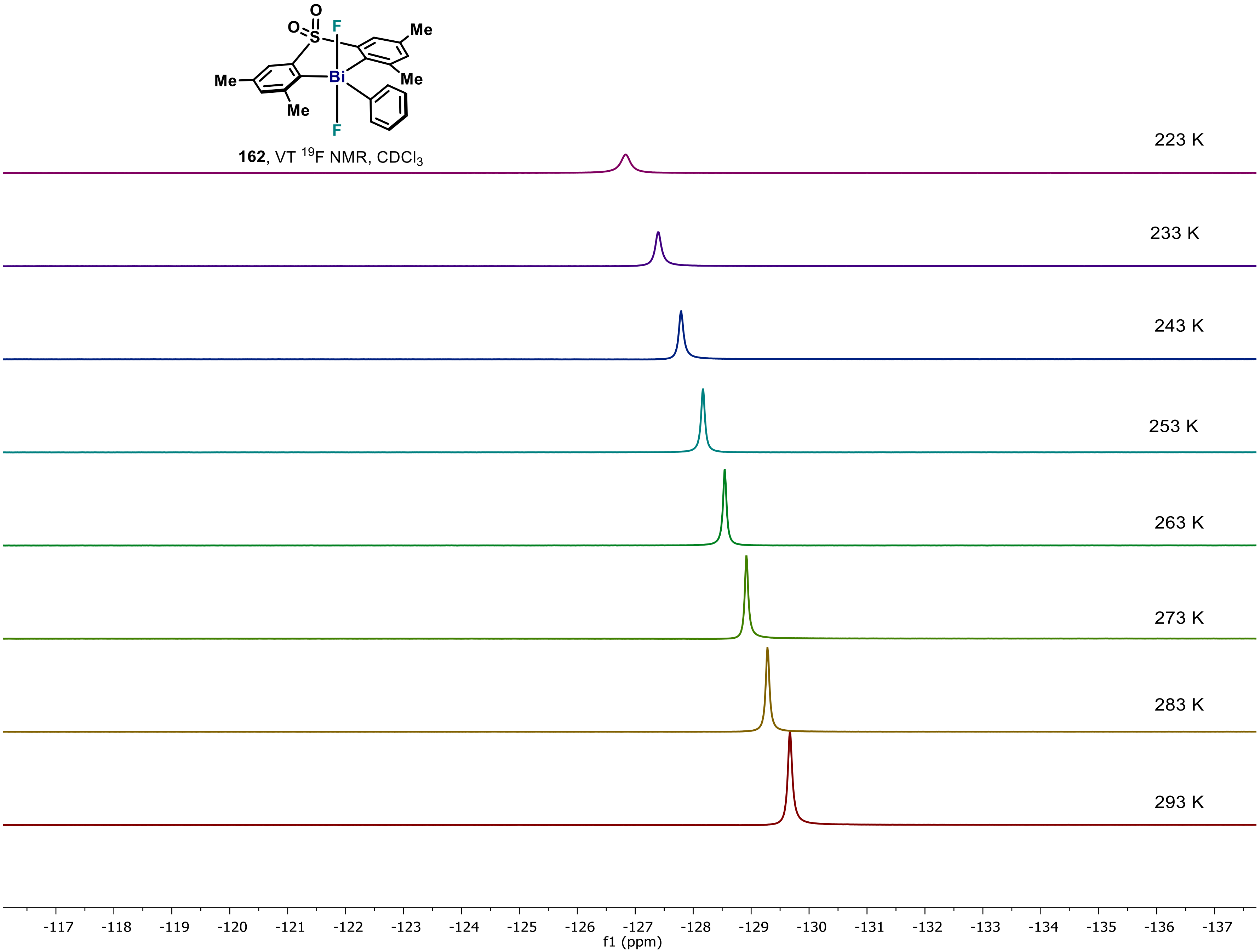

162, VT ^{19}F NMR, $CDCl_3$

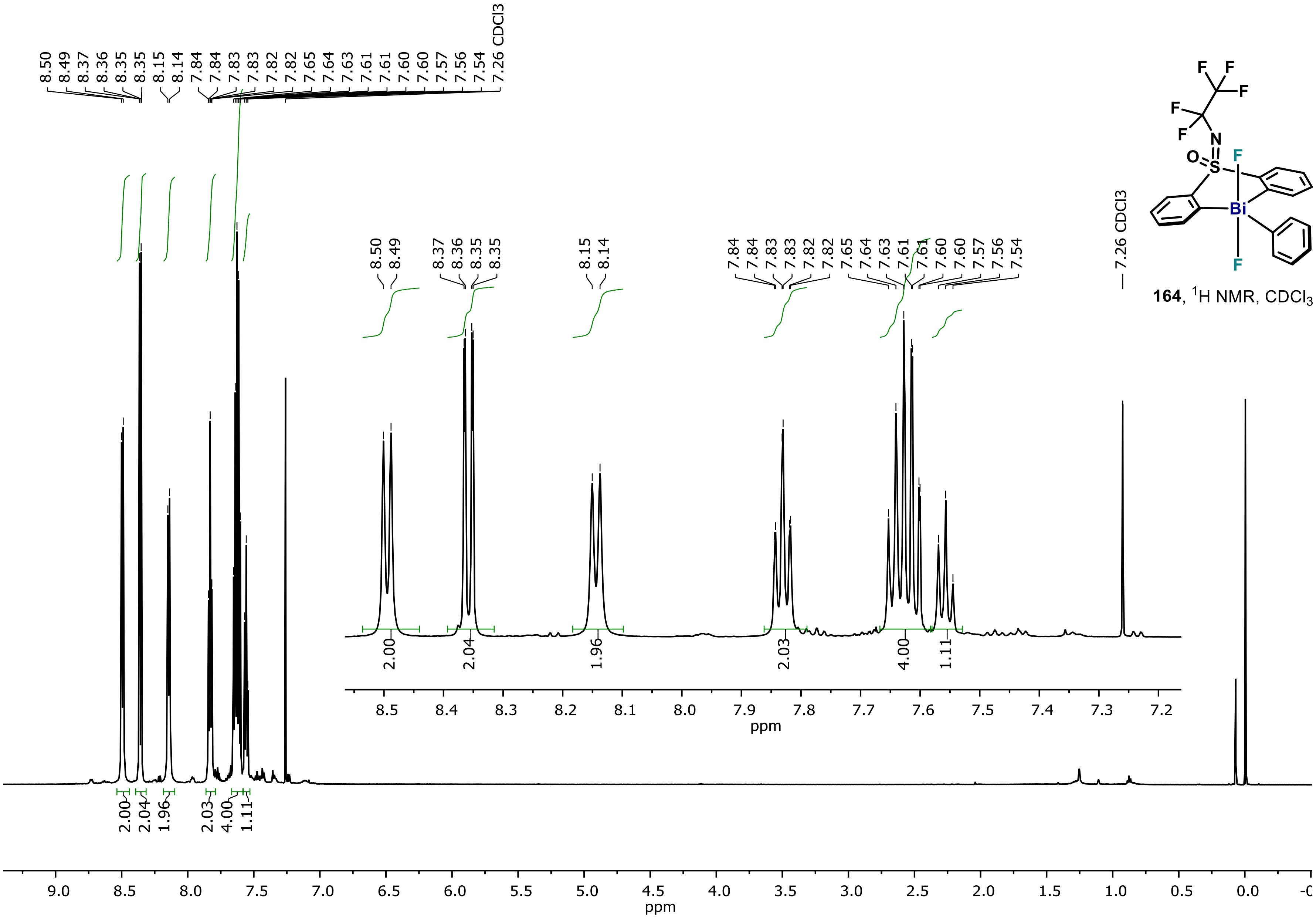

164, ^{1}H NMR, $CDCl_3$

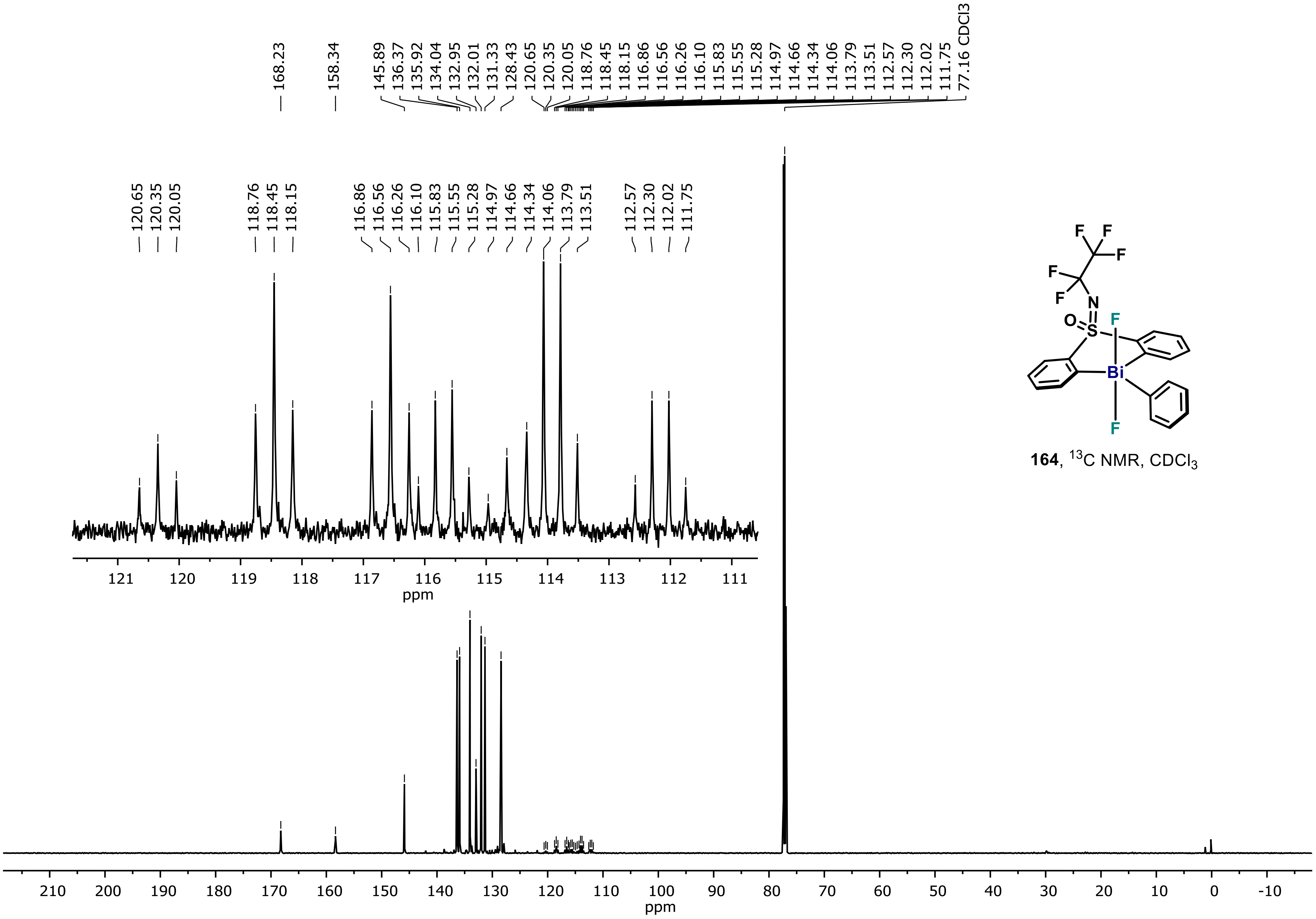

164, ^{13}C NMR, $CDCl_3$

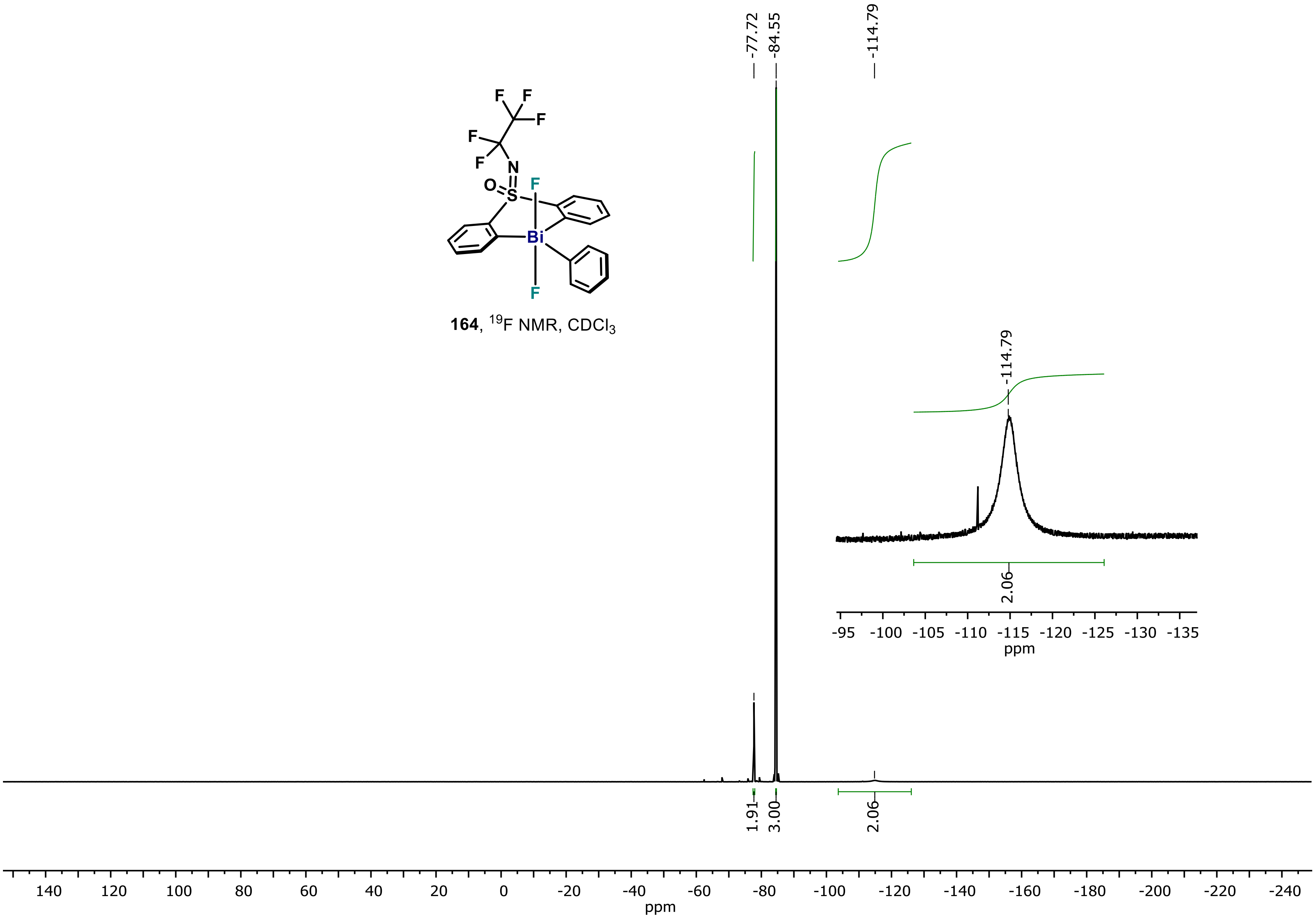

164, ^{19}F NMR, $CDCl_3$

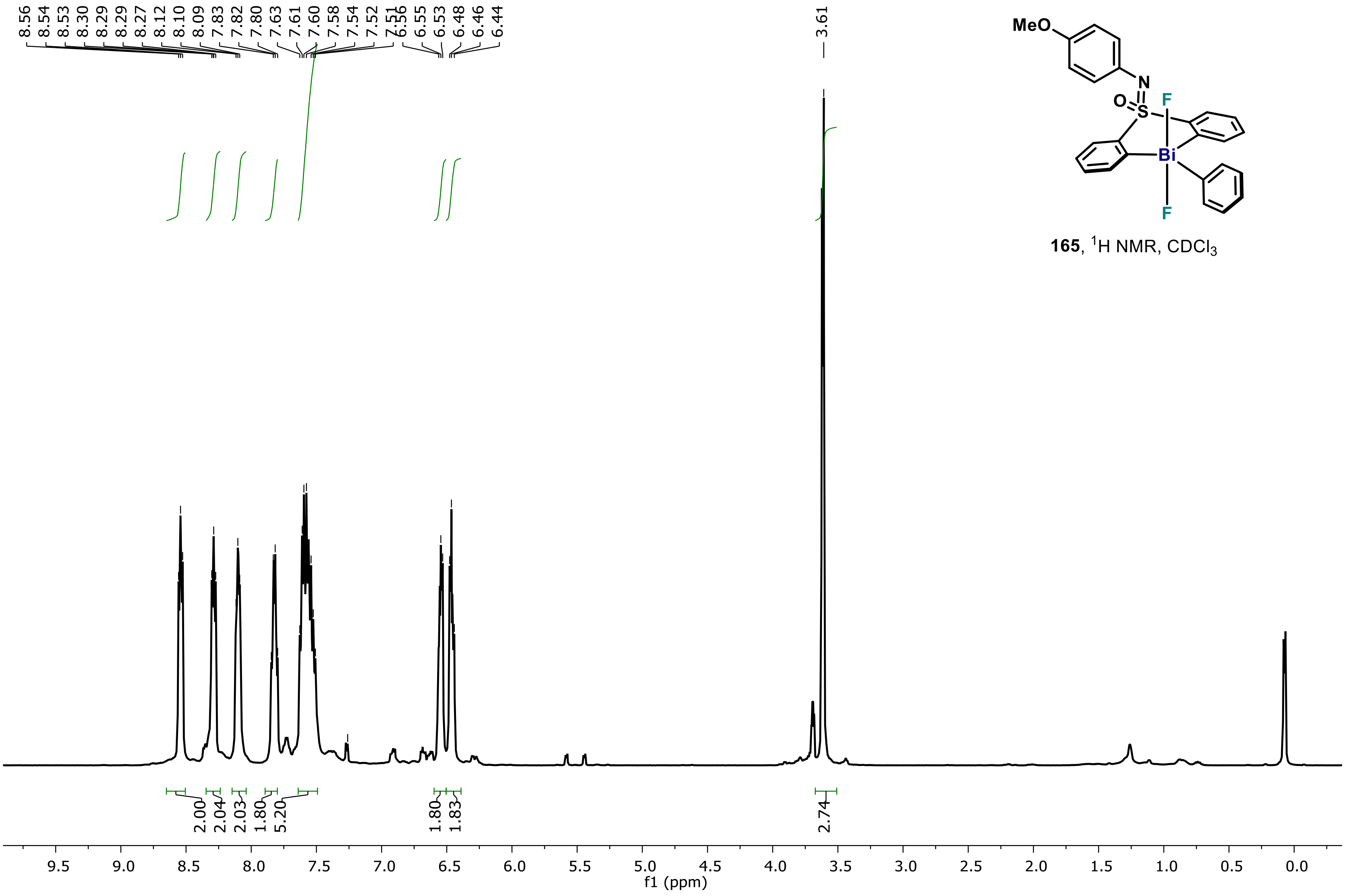

165, ^{1}H NMR, $CDCl_3$

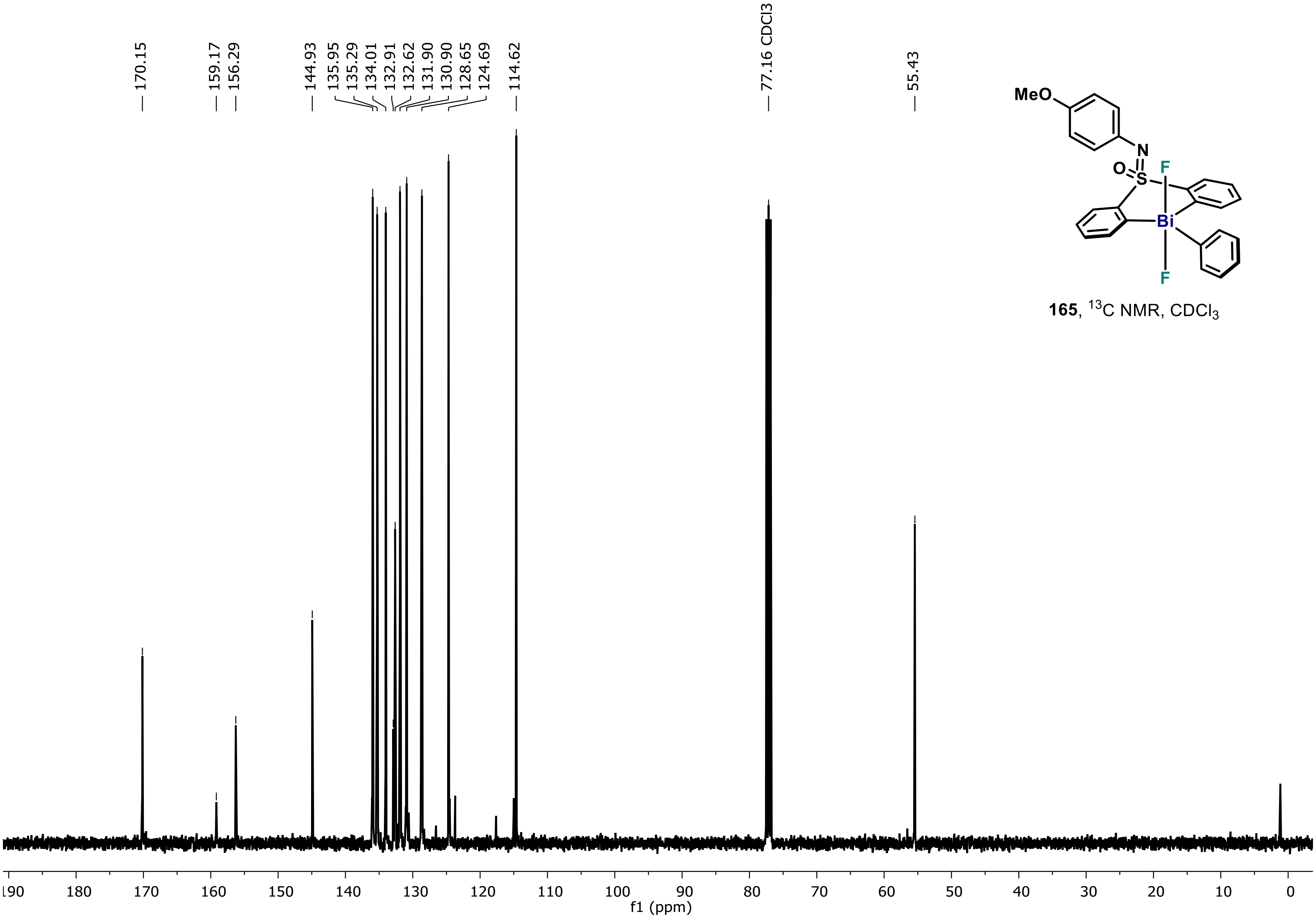

165, ^{13}C NMR, $CDCl_3$

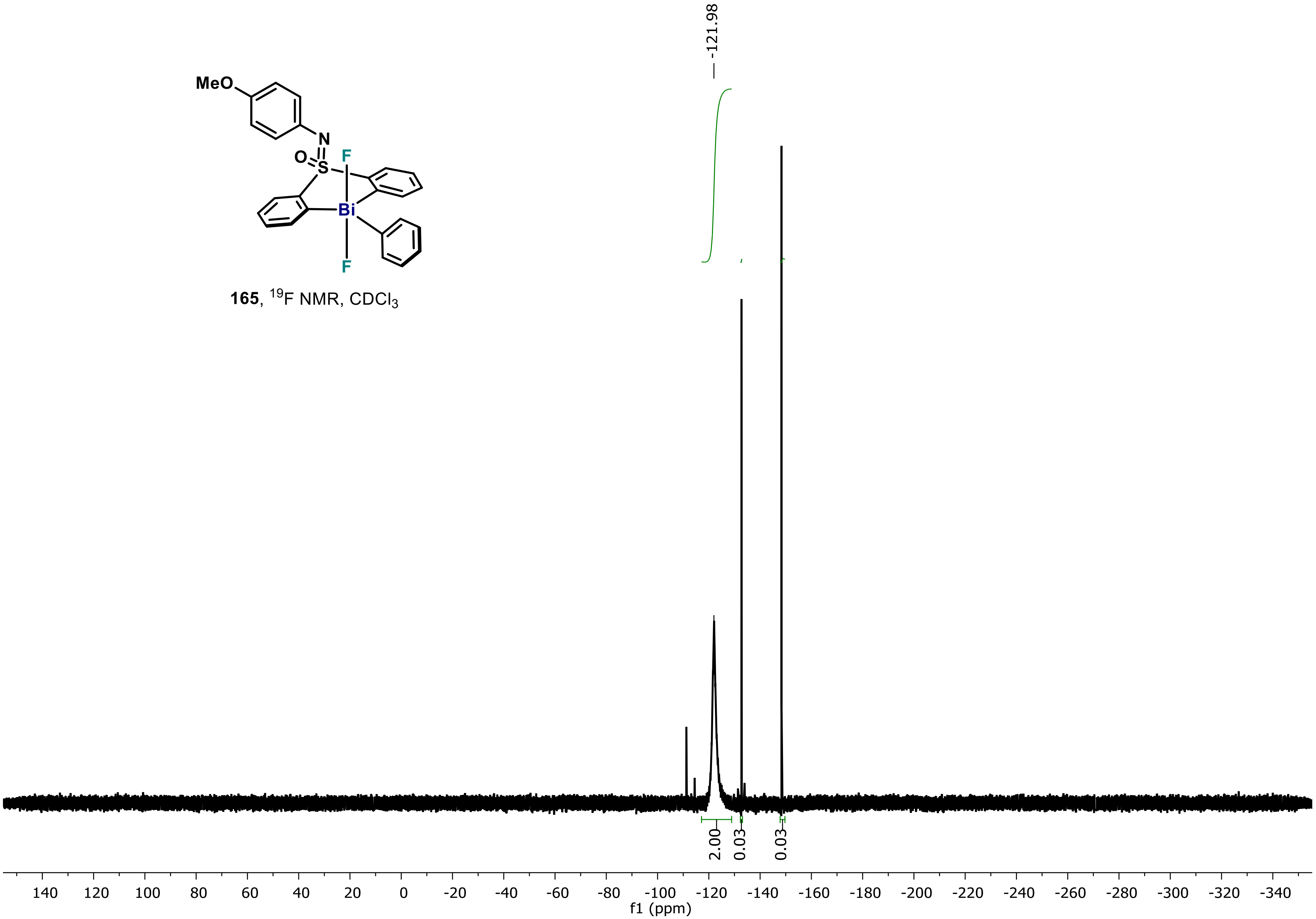

165, ^{19}F NMR, $CDCl_3$

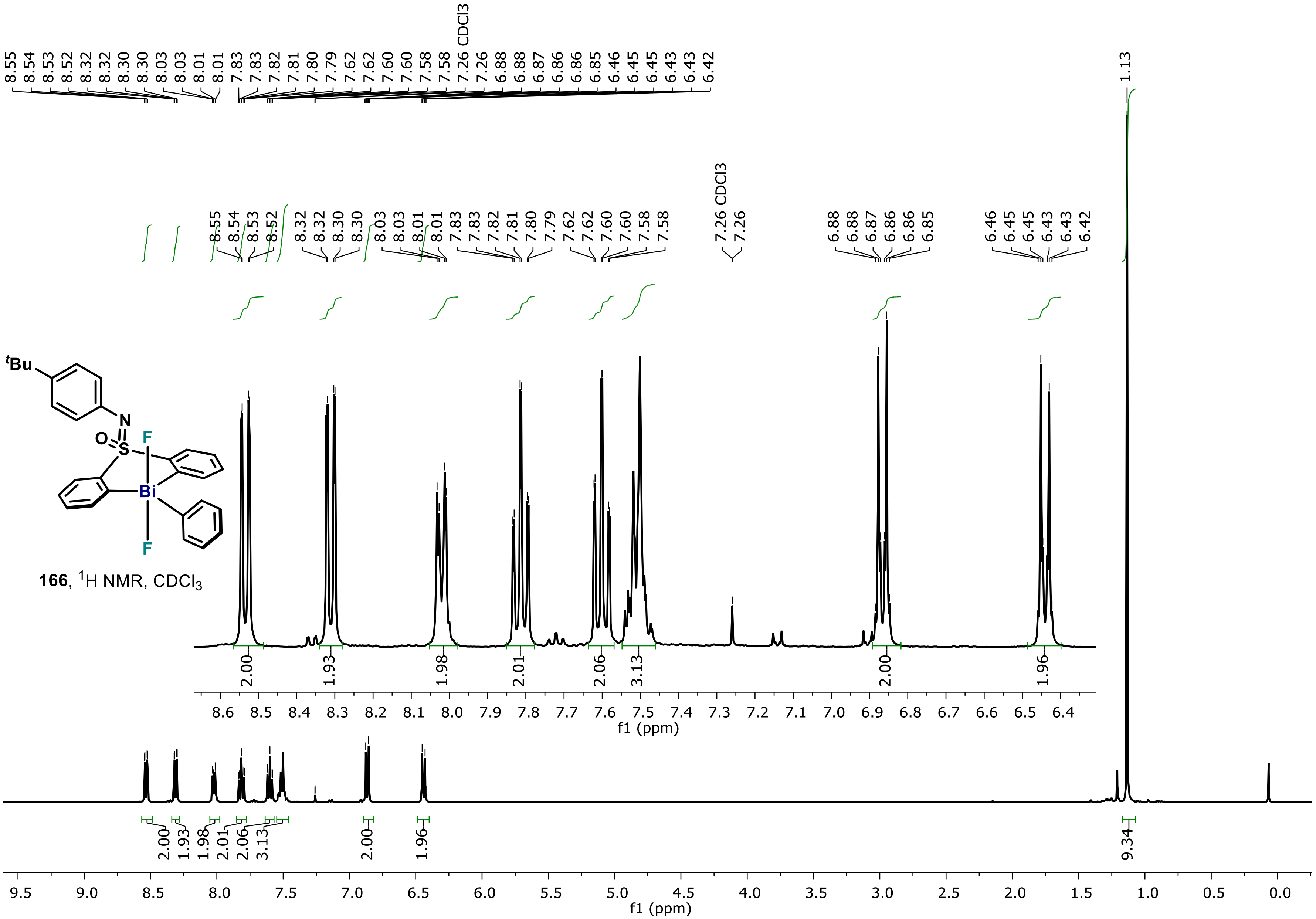

166, ^{1}H NMR, $CDCl_3$

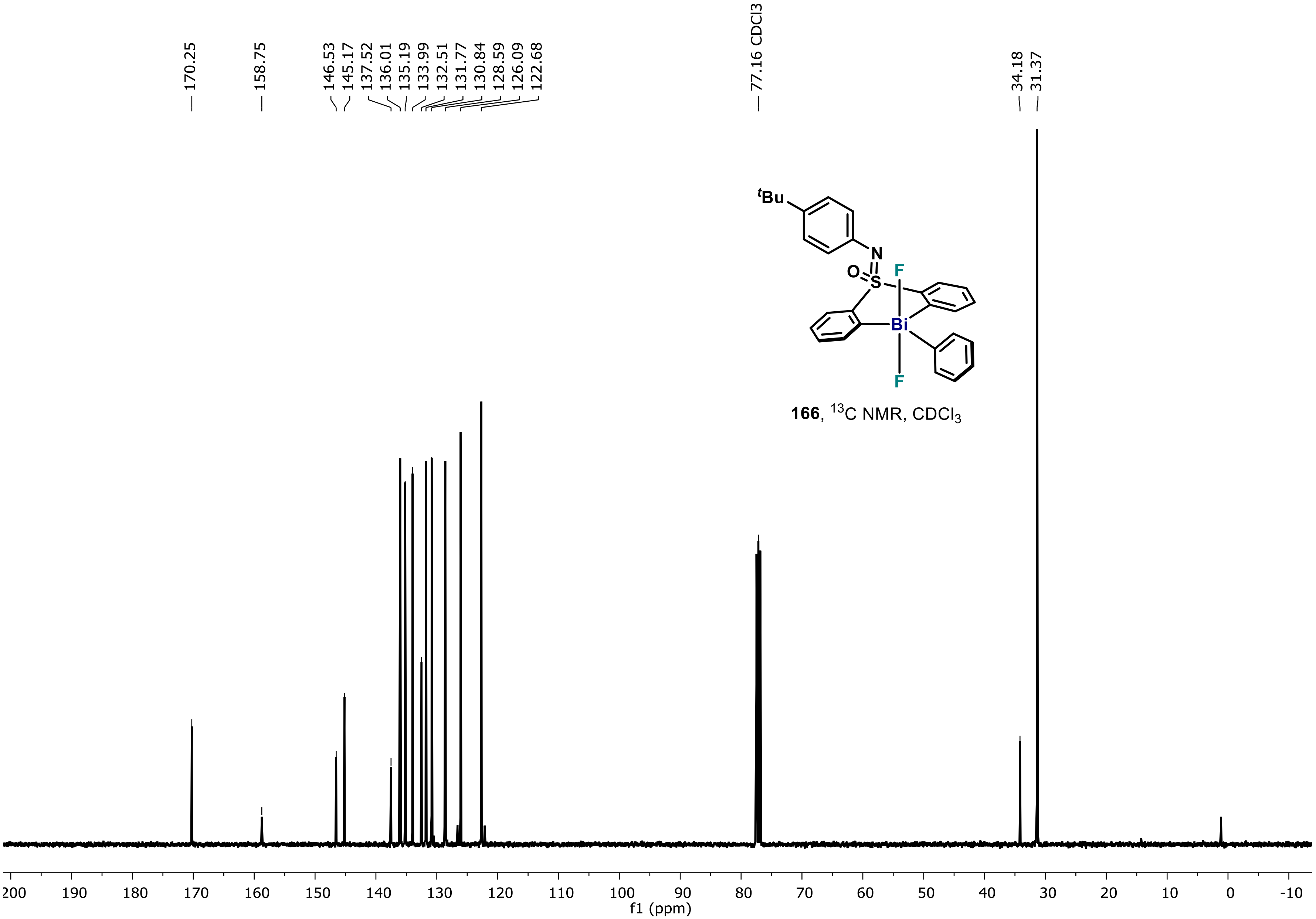
170.25
158.75
146.53
145.17
137.52
136.01
135.19
133.99
132.51
131.77
130.84
128.59
126.09
122.68
77.16 CDCl3
34.18
31.37
tBu
N
O
S
F
Bi
F
166, 13C NMR, CDCl3
200 190 180 170 160 150 140 130 120 110 100 90 80 70 60 50 40 30 20 10 0 -10
f1 (ppm)

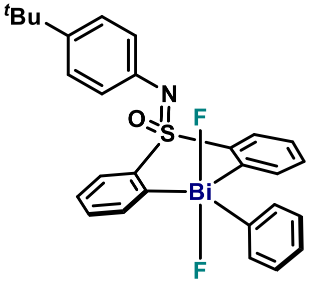

166, ^{13}C NMR, $CDCl_3$

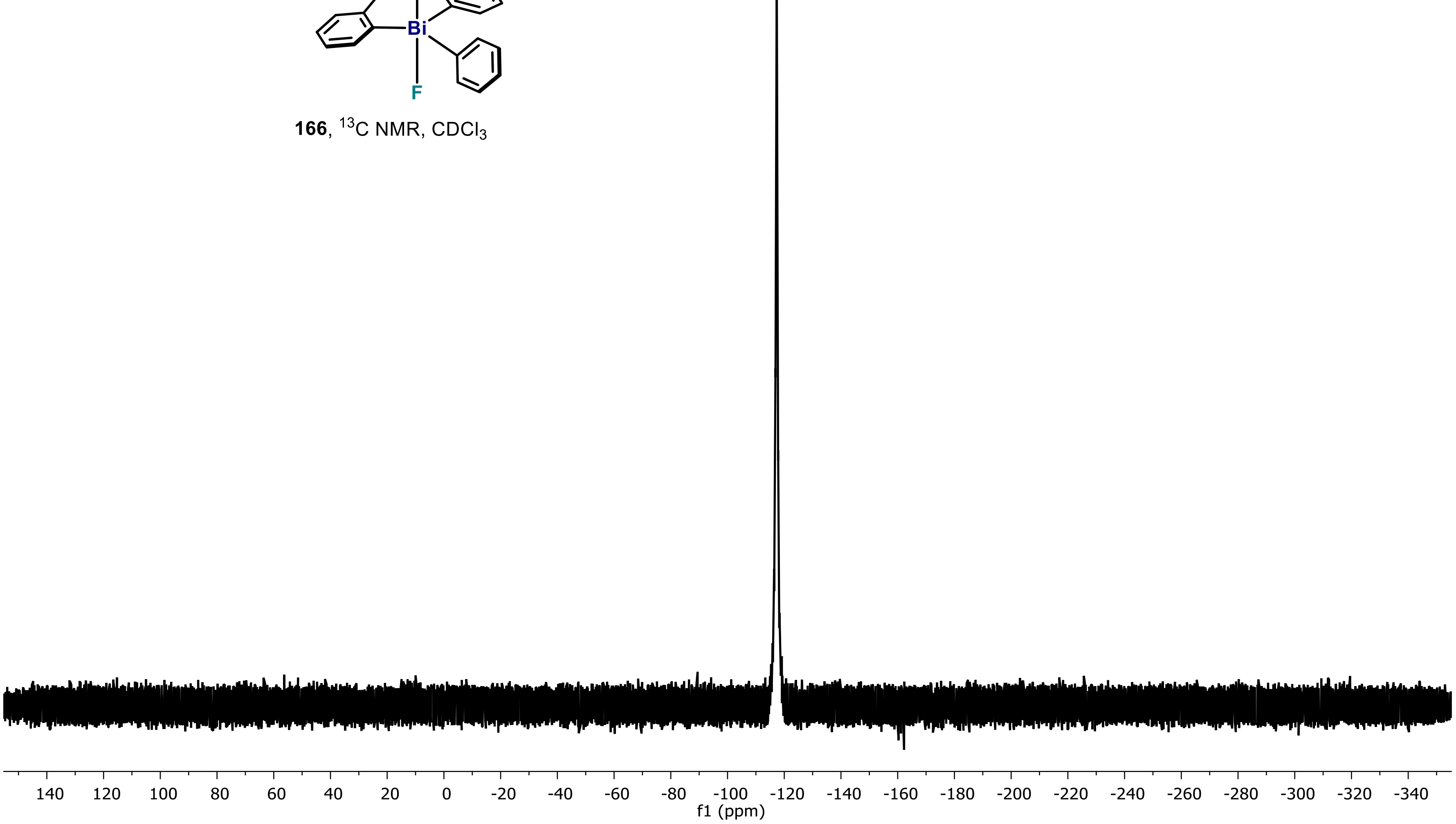

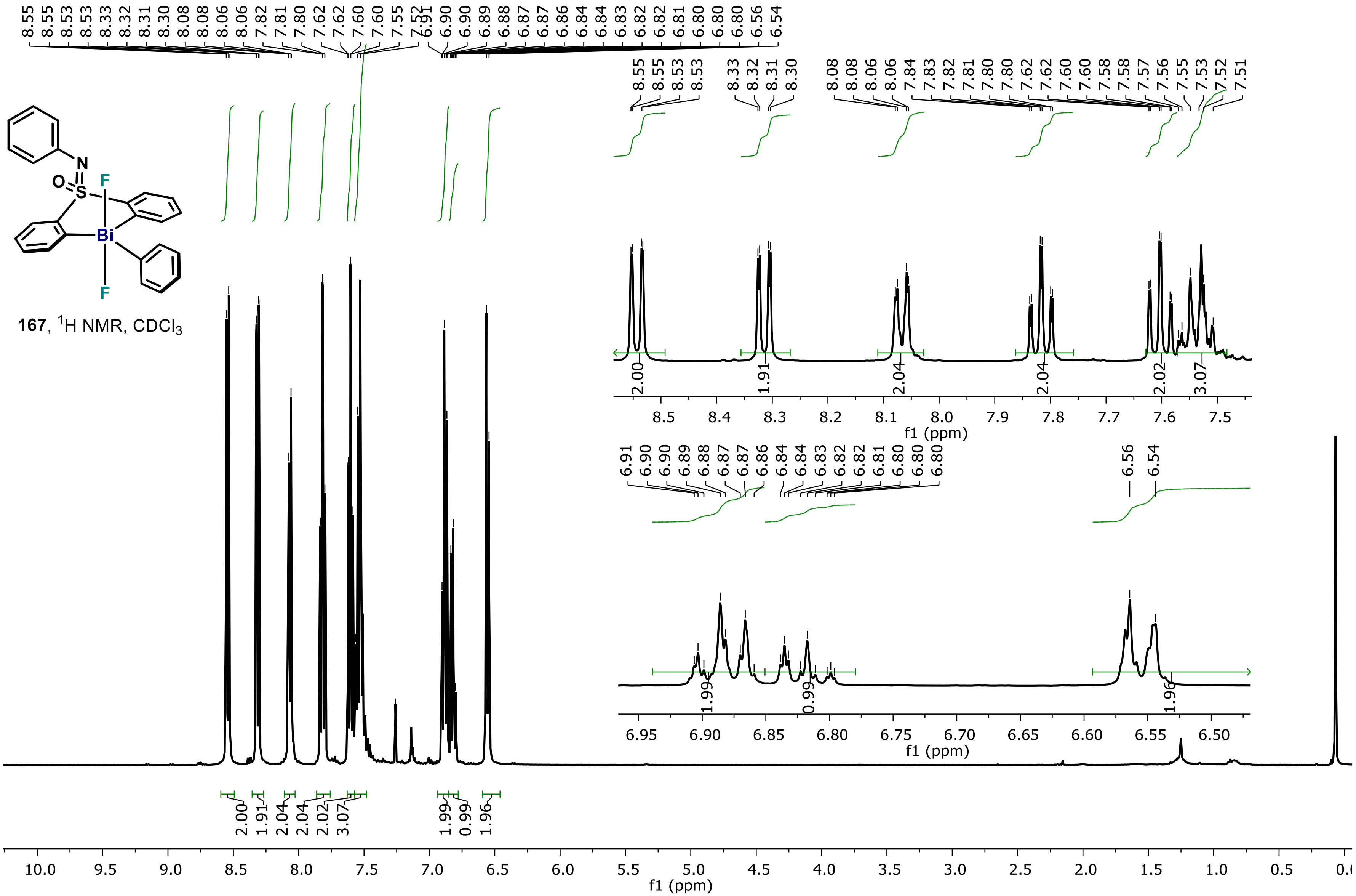

167, ^{1}H NMR, $CDCl_3$

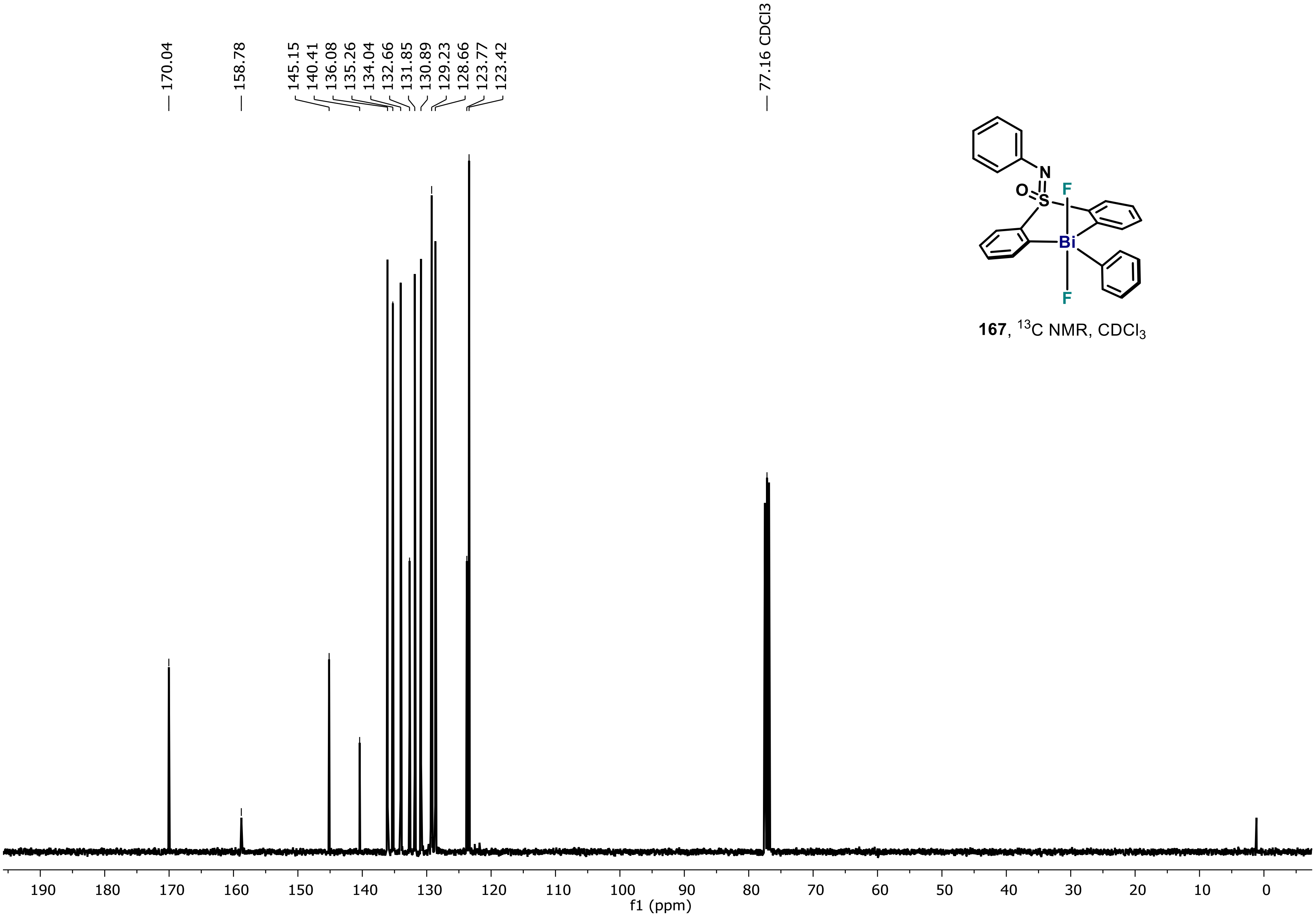
170.04
158.78
145.15
140.41
136.08
135.26
134.04
132.66
131.85
130.89
129.23
128.66
123.77
123.42
77.16 CDCl3
N
O
S
F
Bi
F
167, ^{13}C NMR, $CDCl_3$
190 180 170 160 150 140 130 120 110 100 90 80 70 60 50 40 30 20 10 0
f1 (ppm)

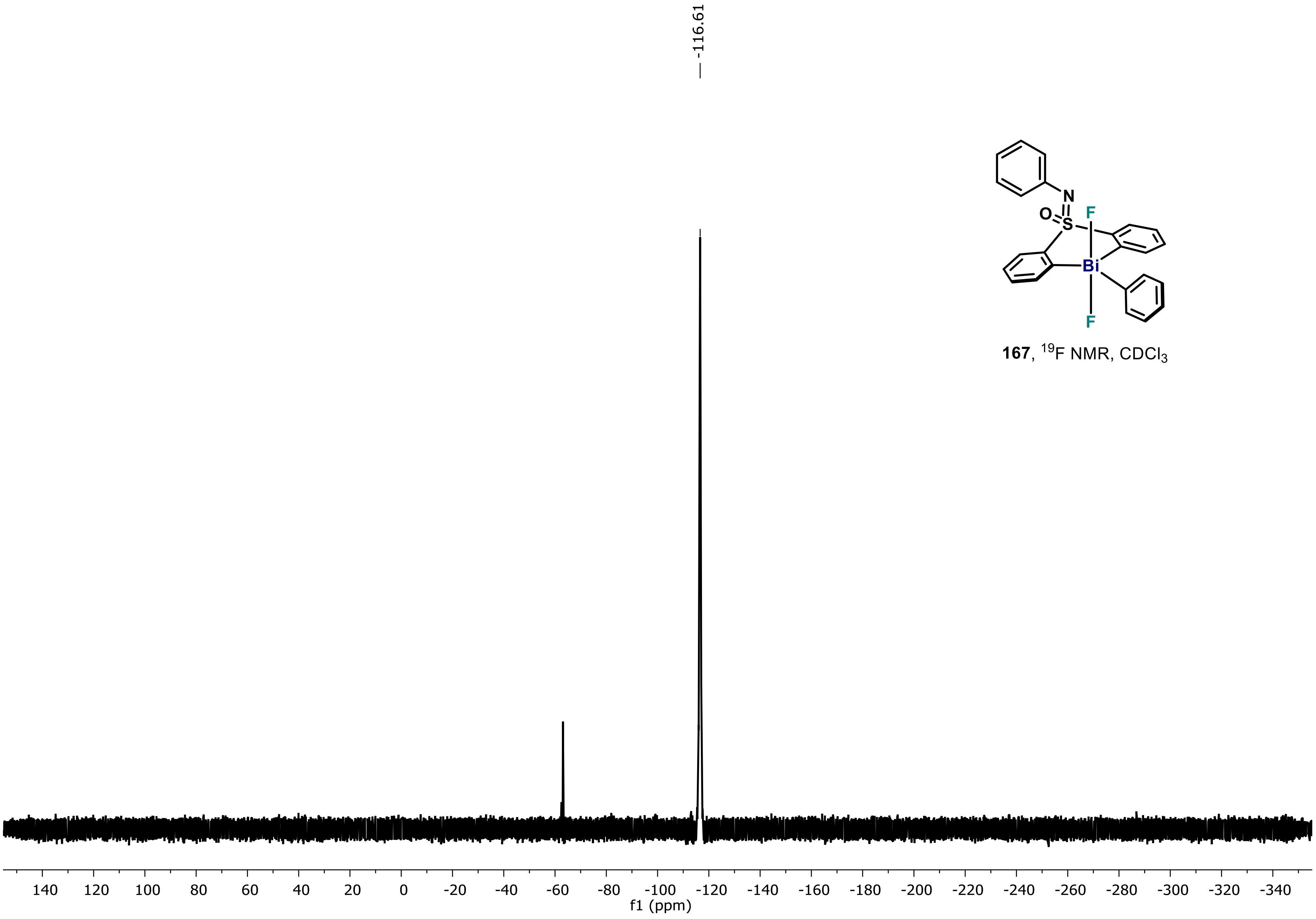
-116.61
N
O
S
F
Bi
F
167, ^{19}F NMR, $CDCl_3$
140 120 100 80 60 40 20 0 -20 -40 -60 -80 -100 -120 -140 -160 -180 -200 -220 -240 -260 -280 -300 -320 -340
f1 (ppm)

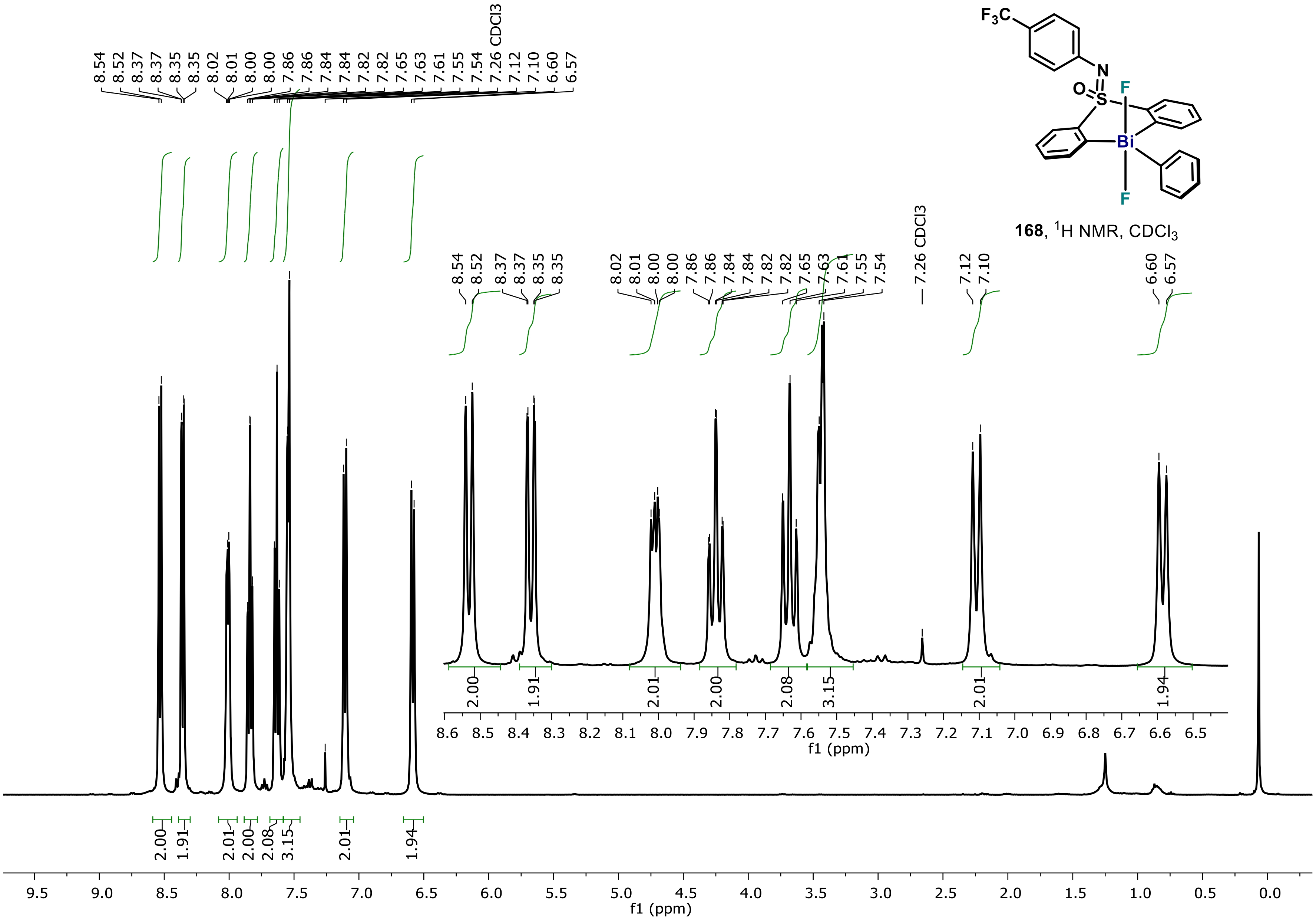

168, ^{1}H NMR, $CDCl_3$

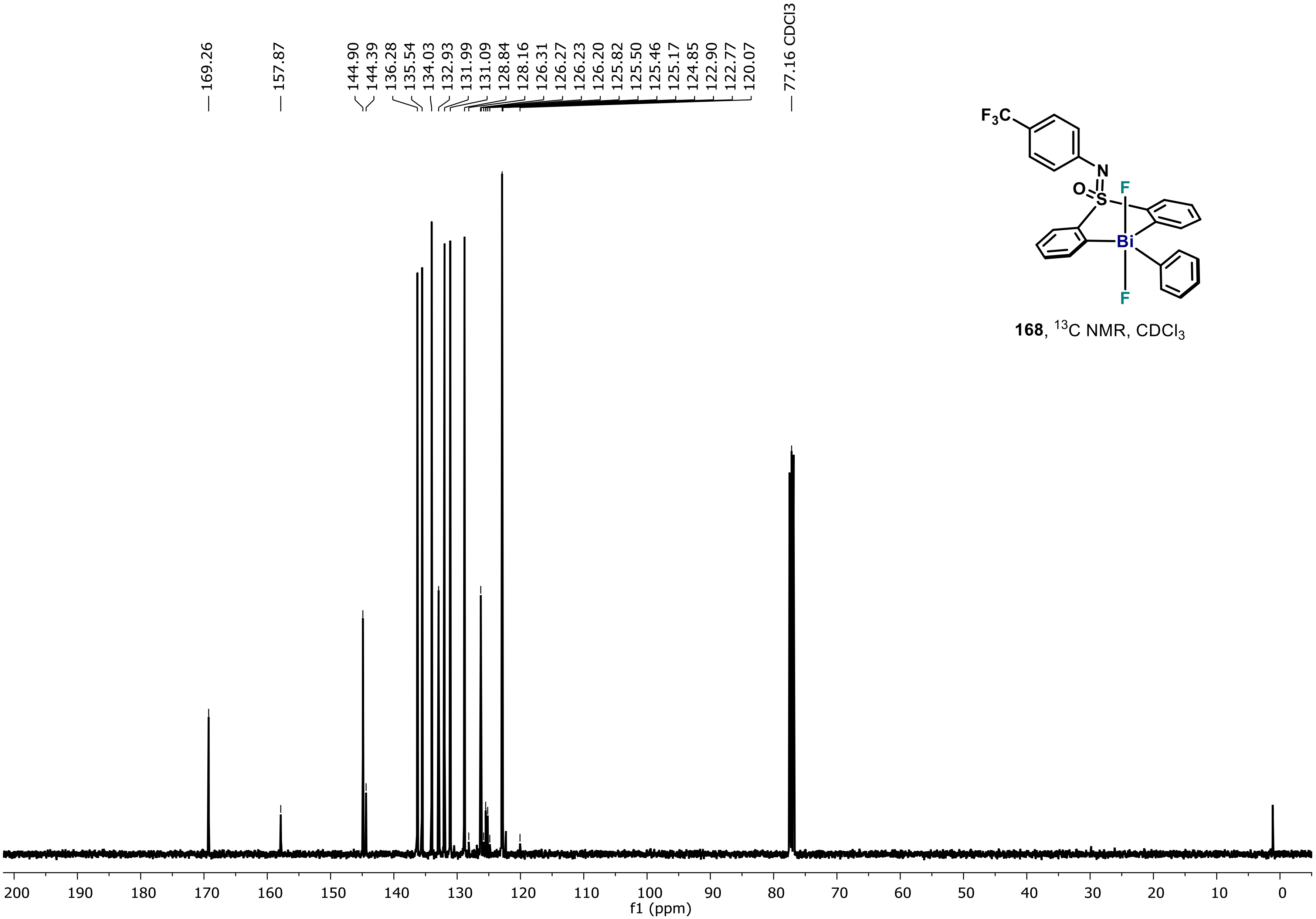

168, ^{13}C NMR, $CDCl_3$

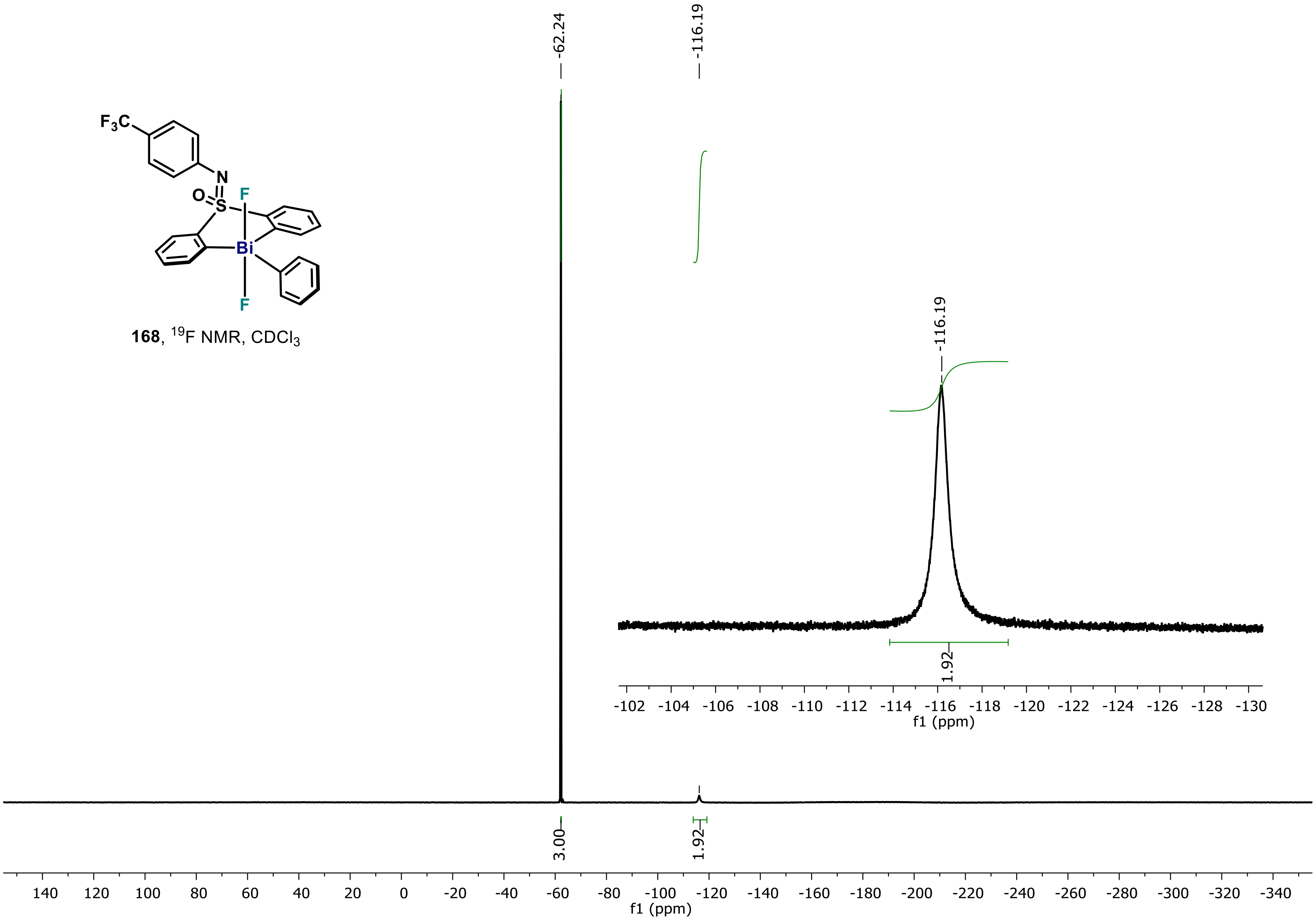

F3C
N
O
S
F
Bi
F
168, 19F NMR, CDCl3
-62.24
-116.19
-116.19
1.92
-102 -104 -106 -108 -110 -112 -114 -116 -118 -120 -122 -124 -126 -128 -130
f1 (ppm)
3.00
1.92
140 120 100 80 60 40 20 0 -20 -40 -60 -80 -100 -120 -140 -160 -180 -200 -220 -240 -260 -280 -300 -320 -340
f1 (ppm)

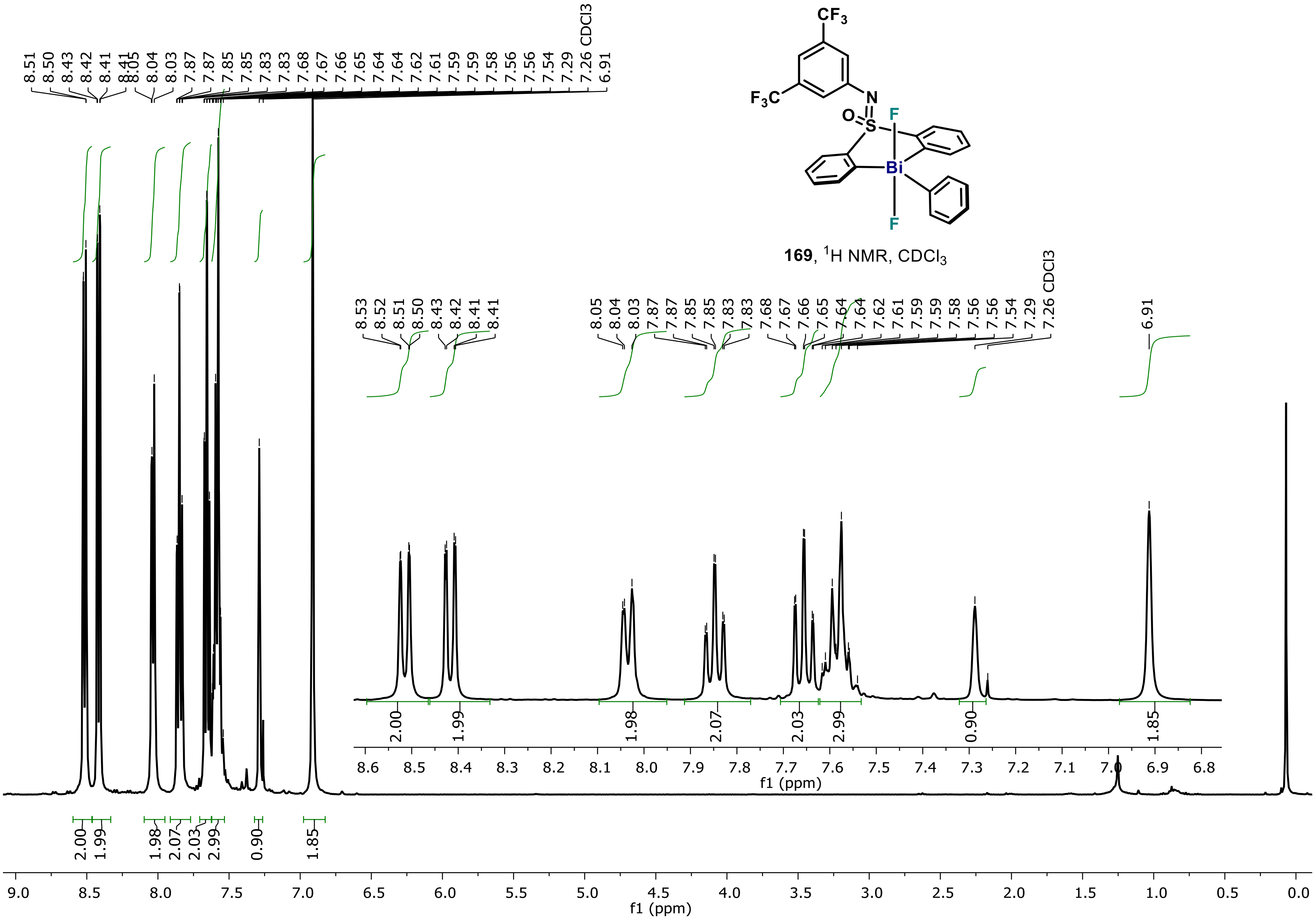

169, ^{1}H NMR, $CDCl_3$

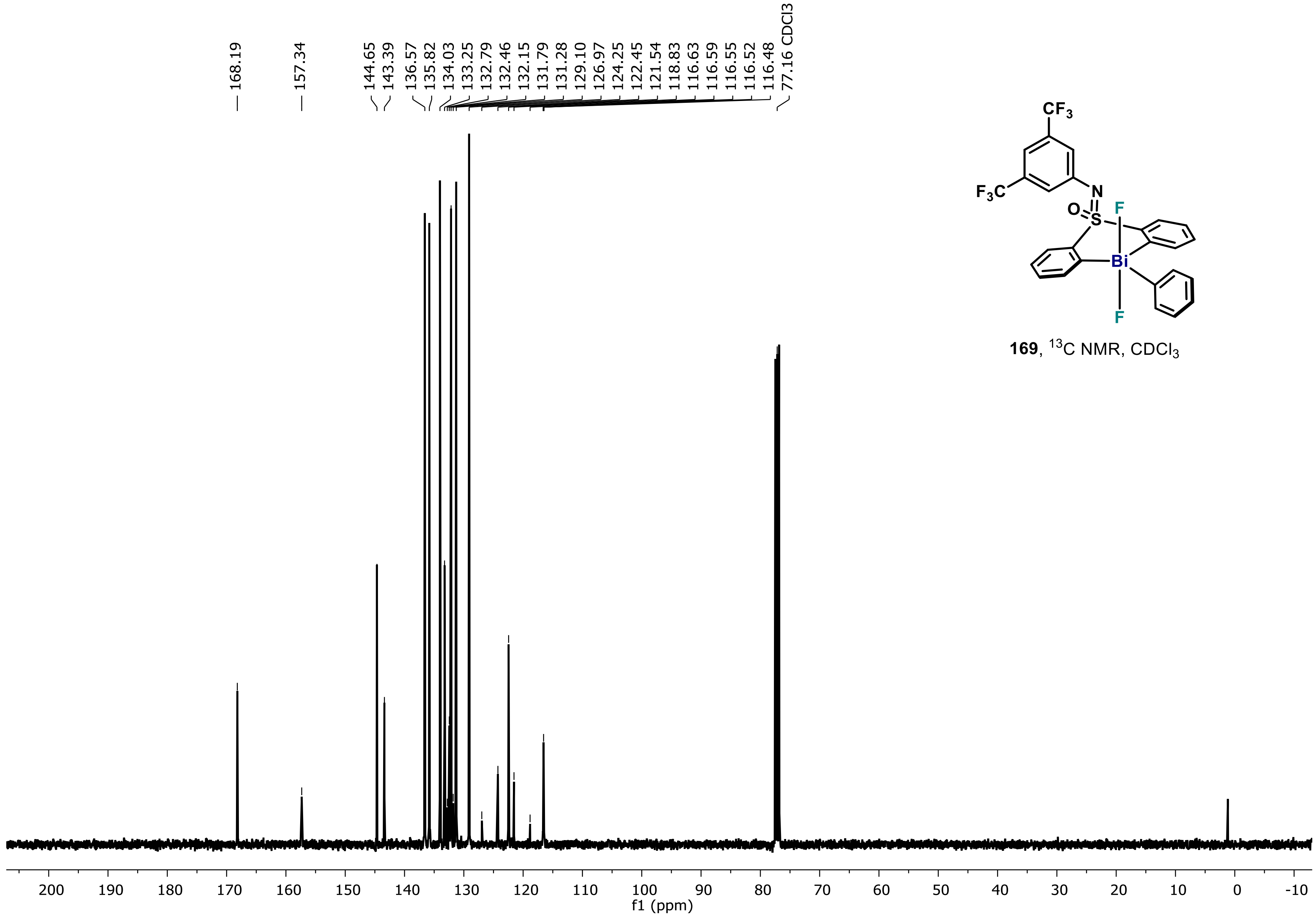

168.19
157.34
144.65
143.39
136.57
135.82
134.03
133.25
132.79
132.46
132.15
131.79
131.28
129.10
126.97
124.25
122.45
121.54
118.83
116.63
116.59
116.55
116.52
116.48
77.16 CDCl3
CF3
F3C
N
O
S
F
Bi
F
169, 13C NMR, CDCl3
200
190
180
170
160
150
140
130
120
110
100
90
80
70
60
50
40
30
20
10
0
-10
f1 (ppm)

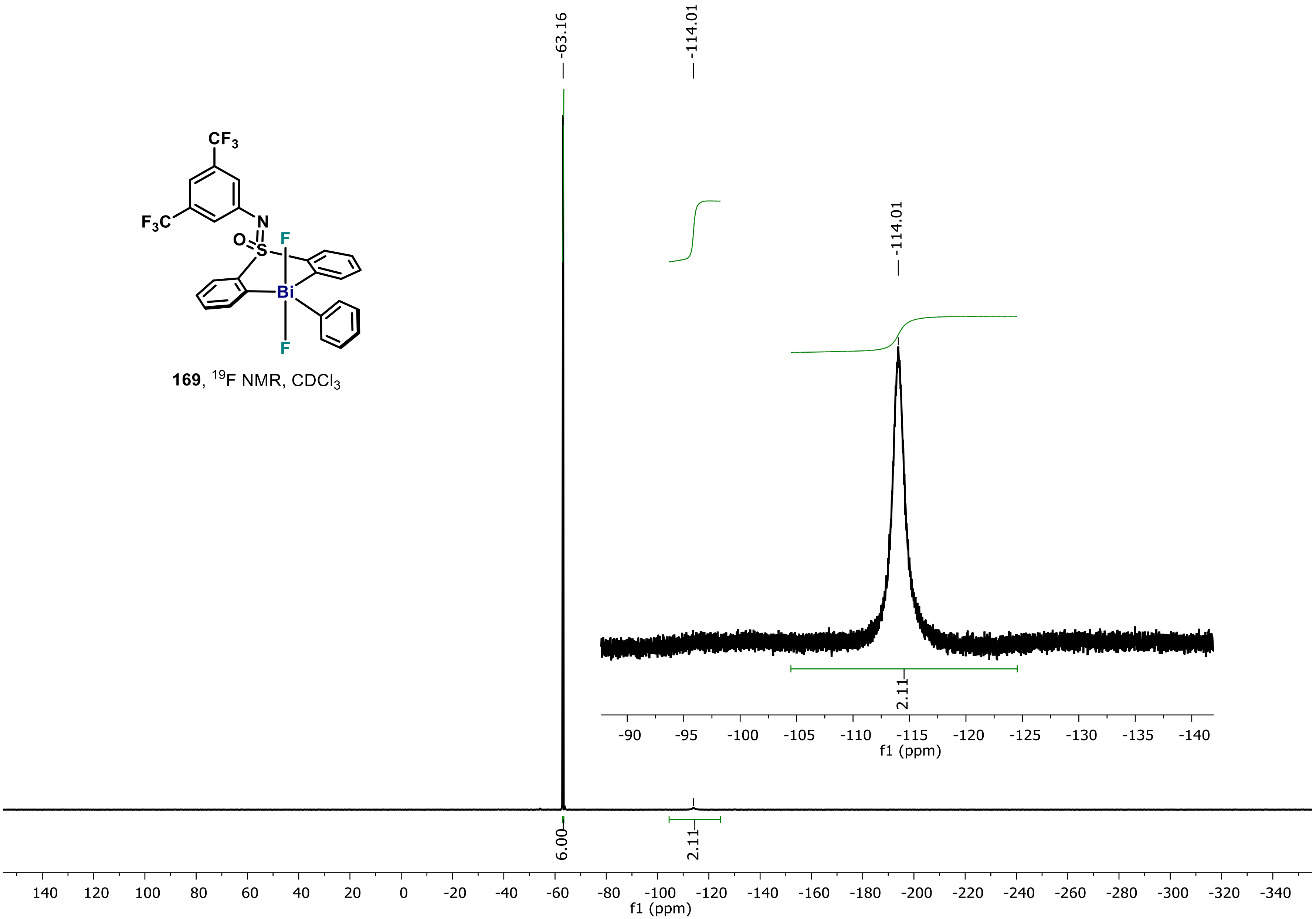

-63.16
-114.01
CF3
F3C
N
O
S
F
Bi
F
169, 19F NMR, CDCl3
-114.01
2.11
-90 -95 -100 -105 -110 -115 -120 -125 -130 -135 -140
f1 (ppm)
6.00
2.11
140 120 100 80 60 40 20 0 -20 -40 -60 -80 -100 -120 -140 -160 -180 -200 -220 -240 -260 -280 -300 -320 -340
f1 (ppm)

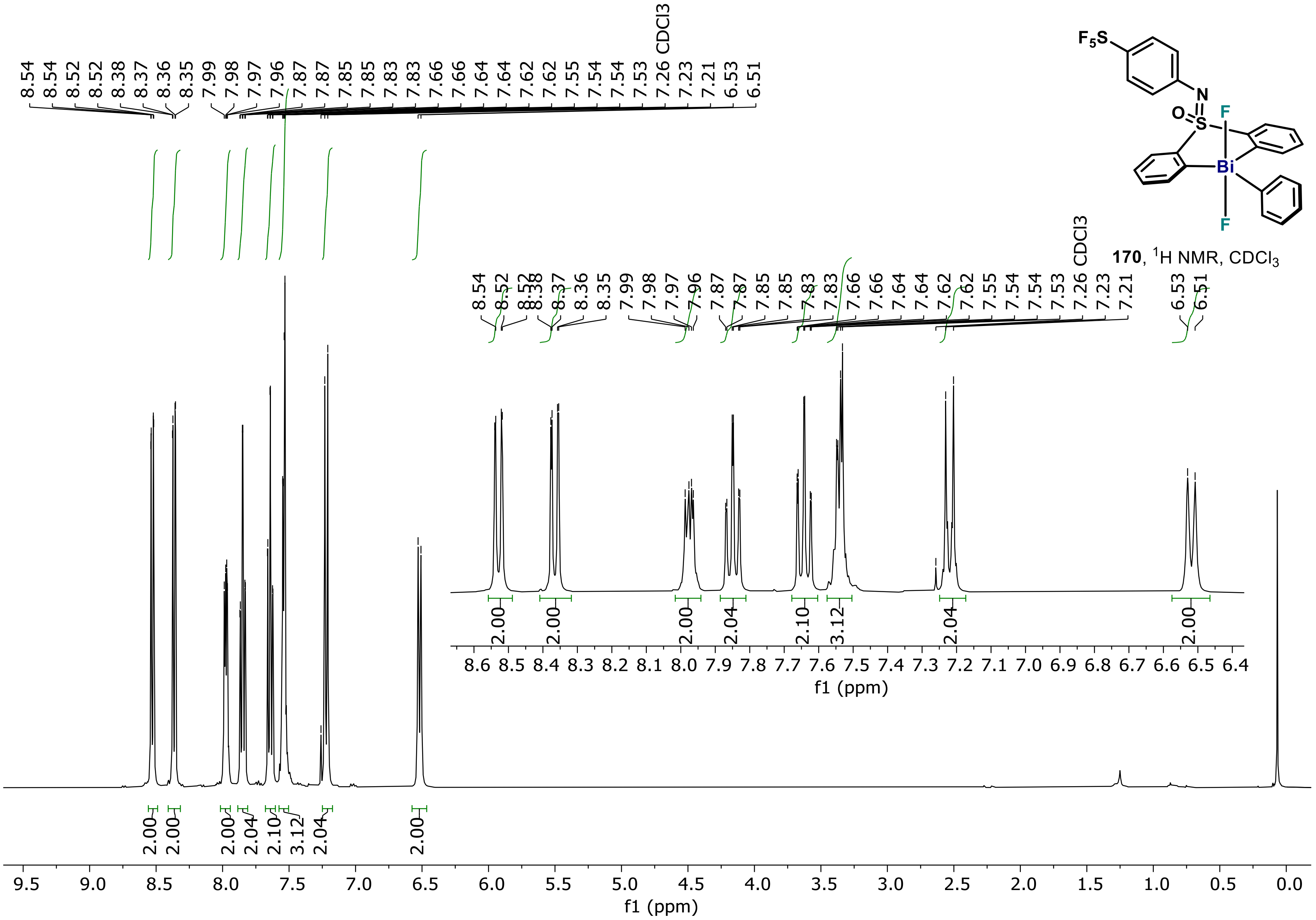

170, ^{1}H NMR, $CDCl_3$

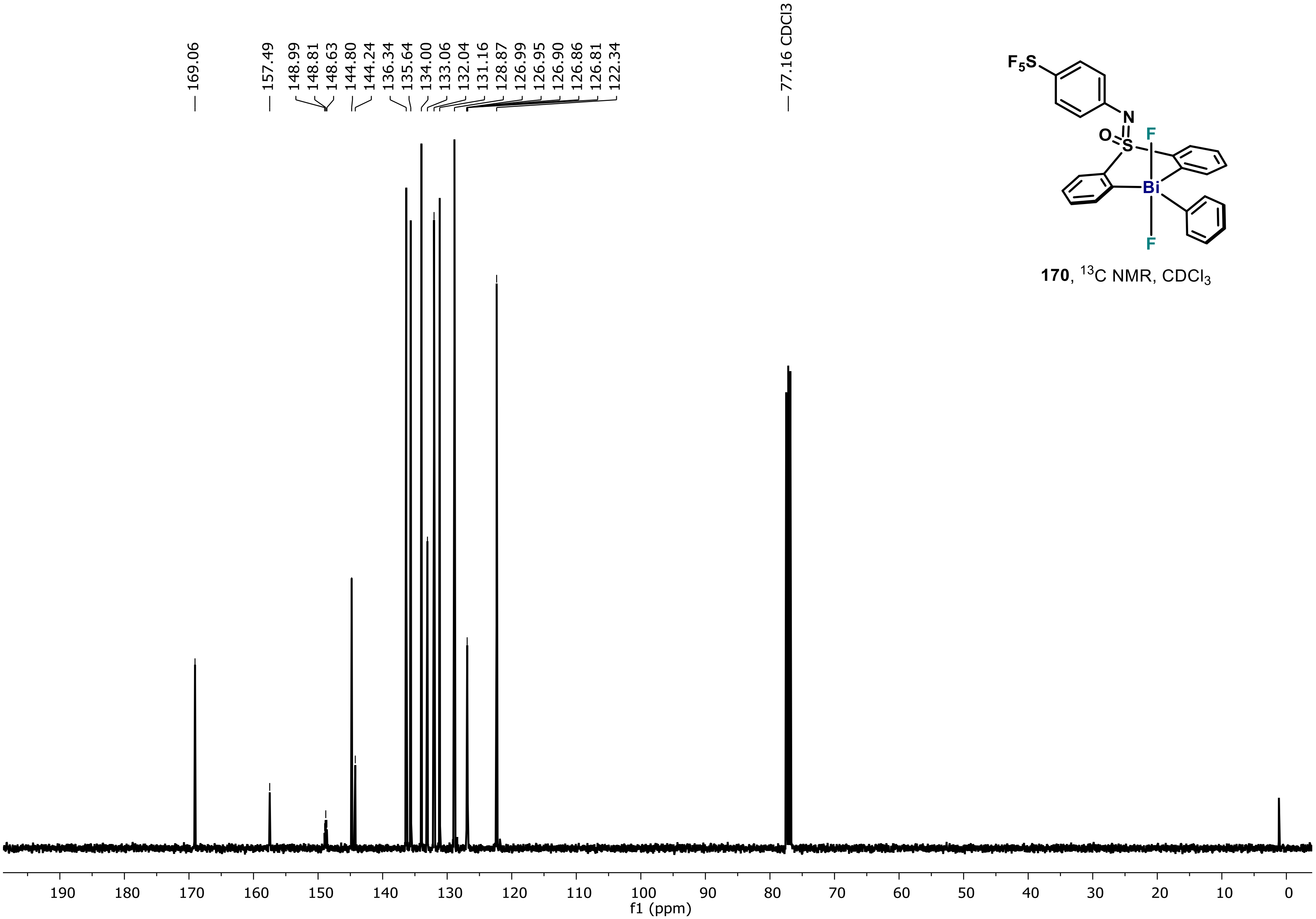

170, ^{13}C NMR, $CDCl_3$

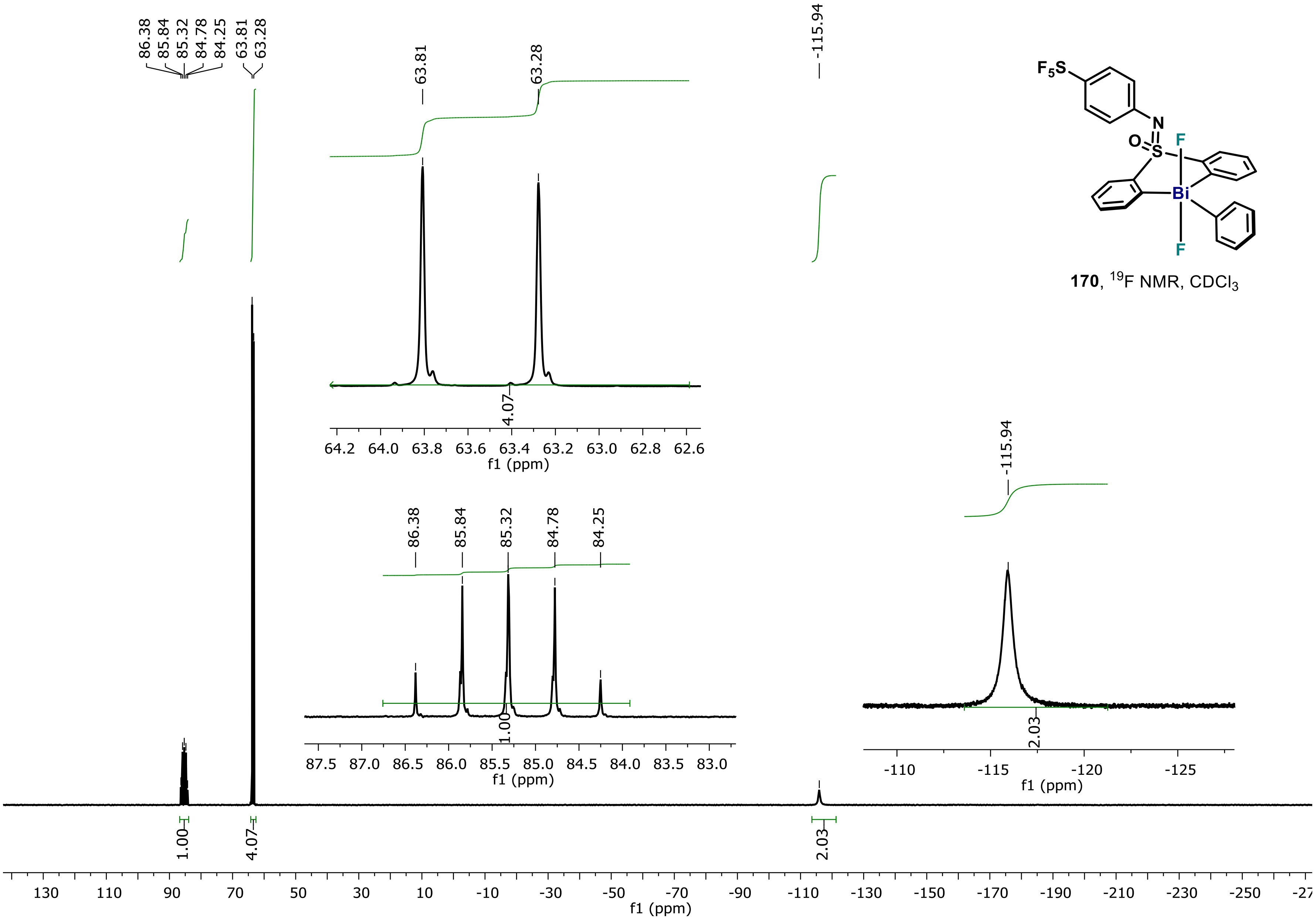
86.38
85.84
85.32
84.78
84.25
63.81
63.28
-115.94
4.07
64.2 64.0 63.8 63.6 63.4 63.2 63.0 62.8 62.6
f1 (ppm)
1.00
87.5 87.0 86.5 86.0 85.5 85.0 84.5 84.0 83.5 83.0
2.03
-110 -115 -120 -125
130 110 90 70 50 30 10 -10 -30 -50 -70 -90 -110 -130 -150 -170 -190 -210 -230 -250
F5S
N
O
S
F
Bi
F
170, 19F NMR, CDCl3

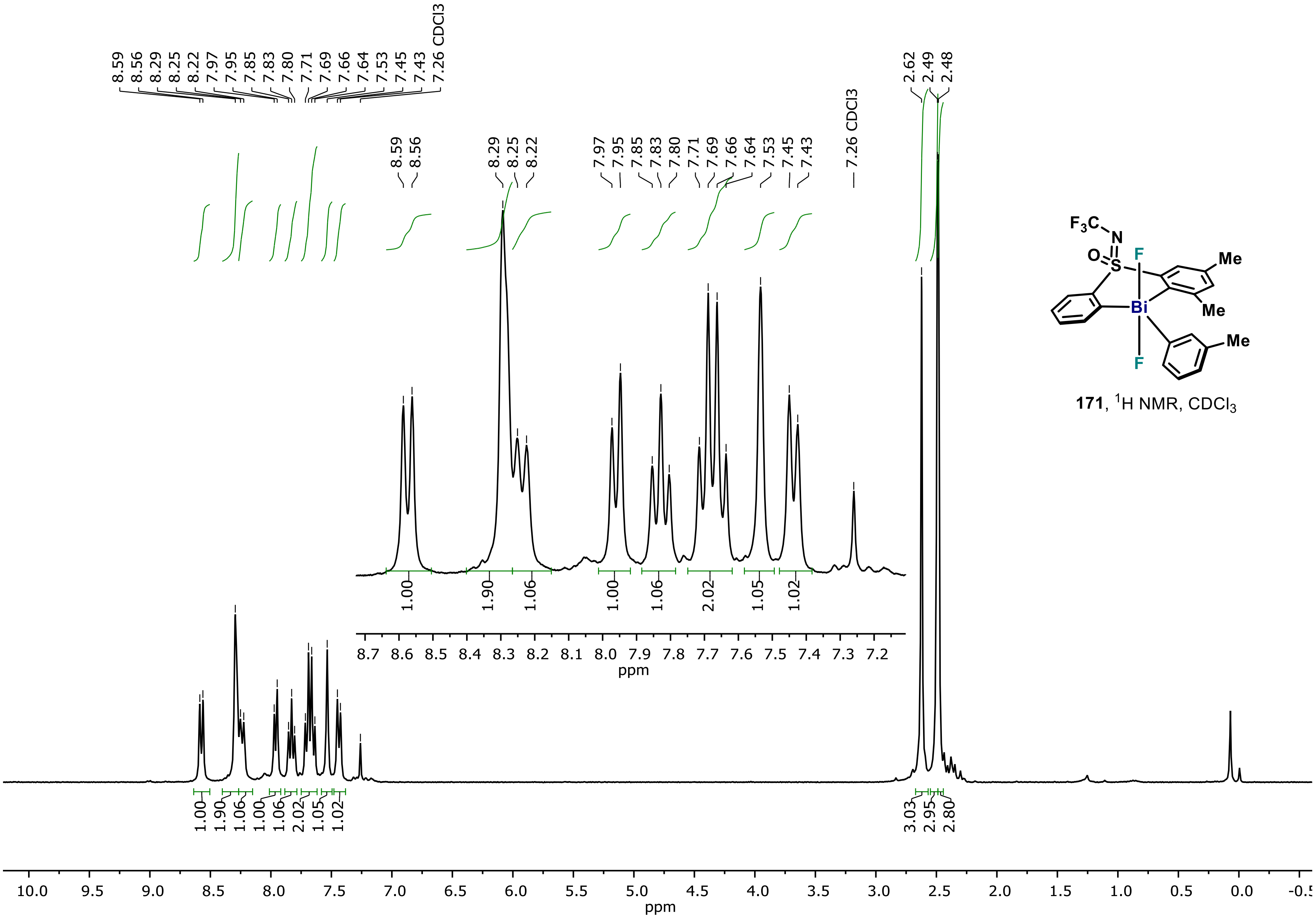
8.59 8.56 8.29 8.25 8.22 7.97 7.95 7.85 7.83 7.80 7.71 7.69 7.66 7.64 7.53 7.45 7.43 7.26 CDCl3
2.62 2.49 2.48
1.00 1.90 1.06 1.00 1.06 2.02 1.05 1.02
3.03 2.95 2.80
ppm
F3C N O S F Me Bi Me Me F
171, 1H NMR, CDCl3

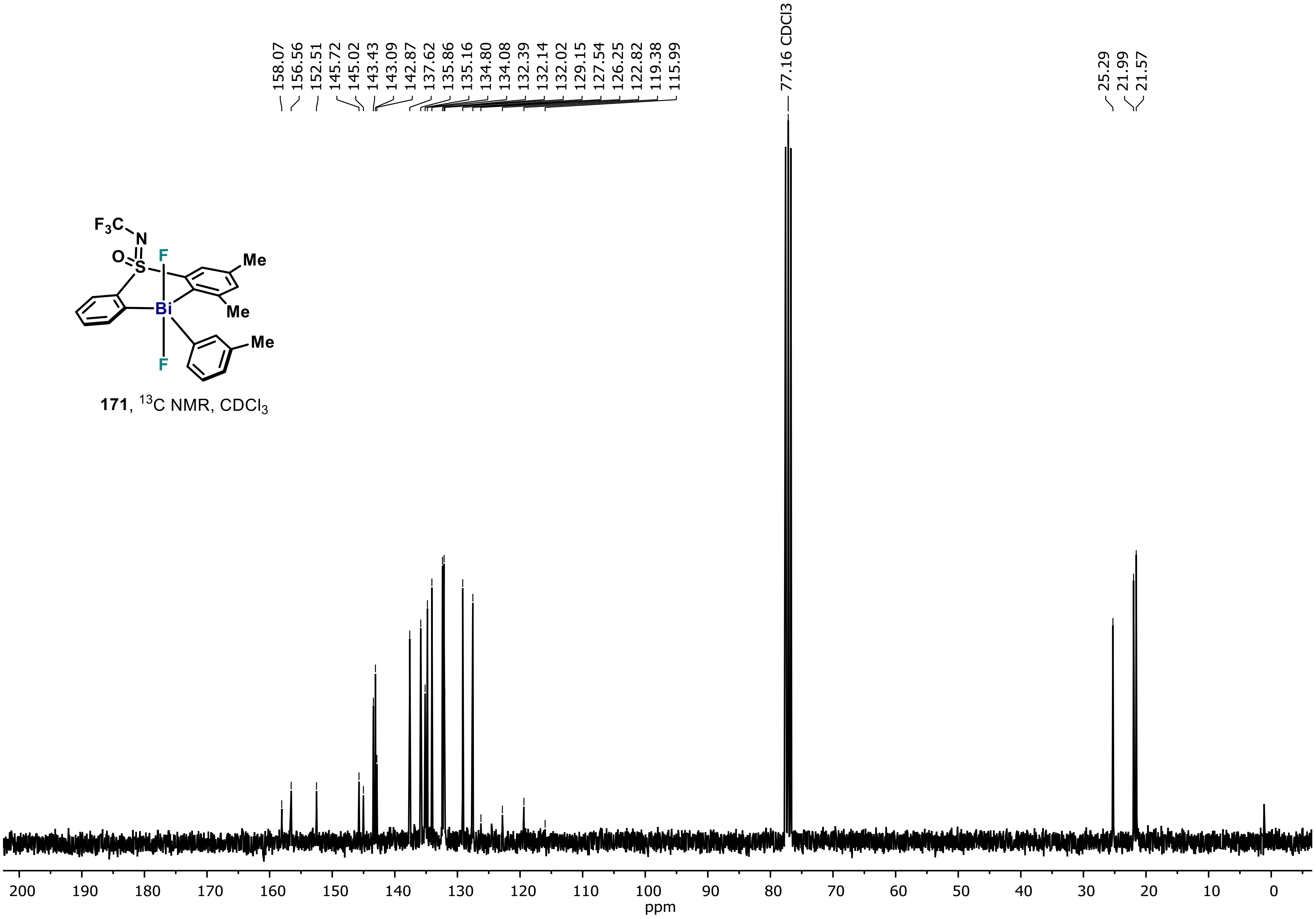
158.07
156.56
152.51
145.72
145.02
143.43
143.09
142.87
137.62
135.86
135.16
134.80
134.08
132.39
132.14
132.02
129.15
127.54
126.25
122.82
119.38
115.99
77.16 CDCl3
25.29
21.99
21.57
F3C
N
O
S
F
Me
Me
Bi
Me
F
171, 13C NMR, CDCl3
200
190
180
170
160
150
140
130
120
110
100
90
80
70
60
50
40
30
20
10
0
ppm

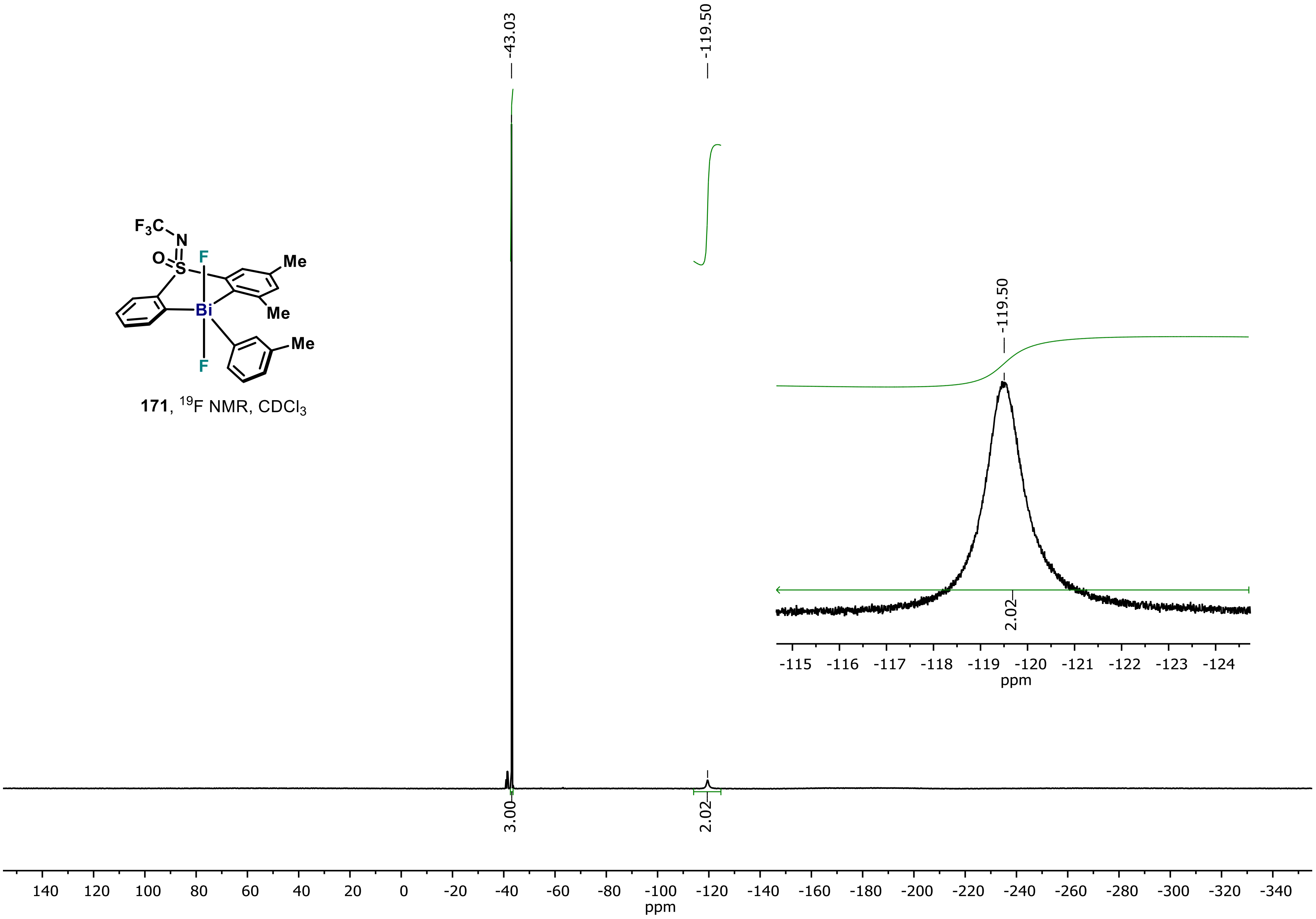

171, ^{19}F NMR, $CDCl_3$

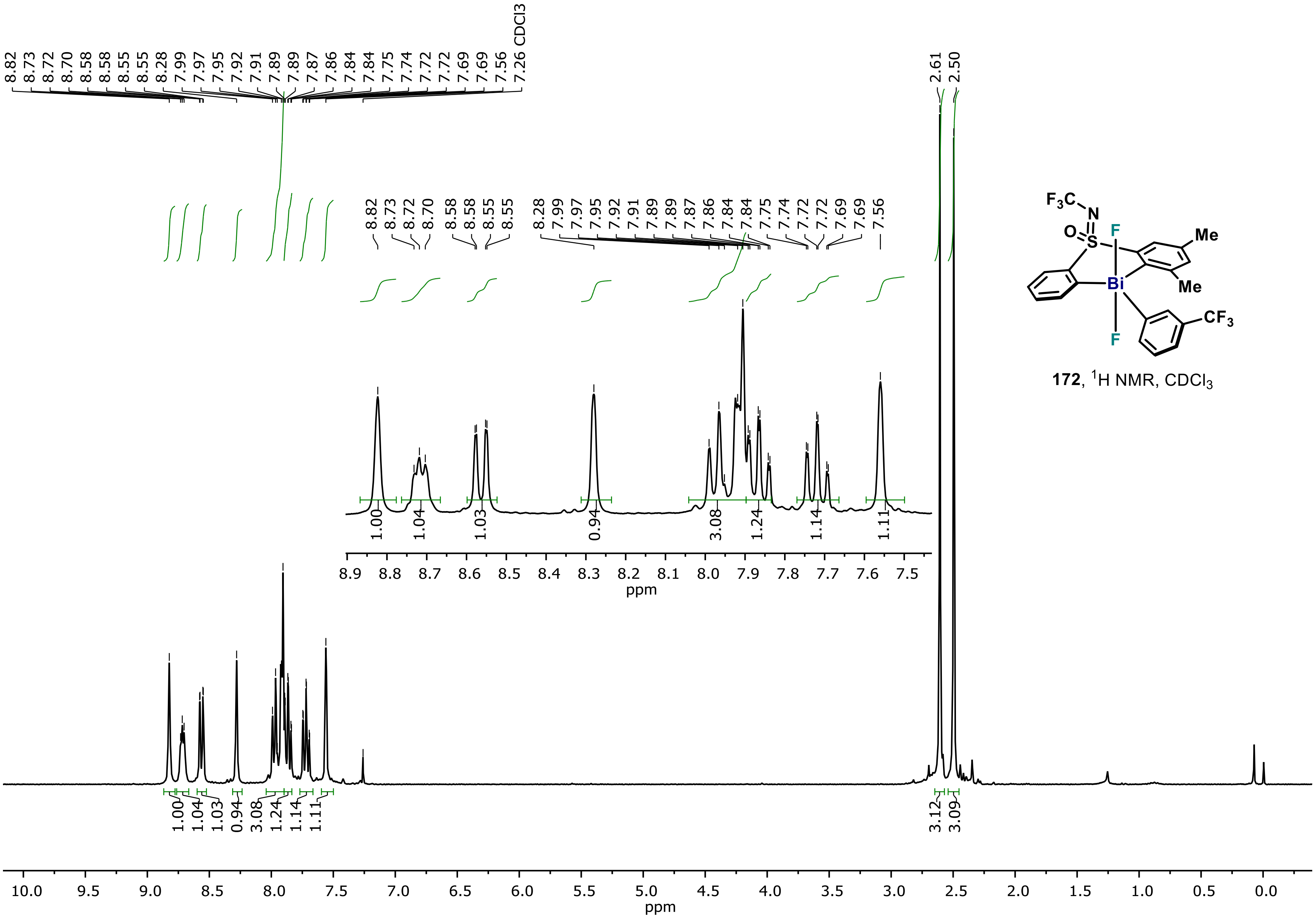
8.82 8.73 8.72 8.70 8.58 8.58 8.55 8.55 8.28 7.99 7.97 7.95 7.92 7.91 7.89 7.89 7.87 7.86 7.84 7.84 7.75 7.74 7.72 7.72 7.69 7.69 7.56 7.26 CDCl3
2.61 2.50
1.00 1.04 1.03 0.94 3.08 1.24 1.14 1.11
3.12 3.09
ppm
F3C N O S F Me Bi Me CF3 F
172, 1H NMR, CDCl3

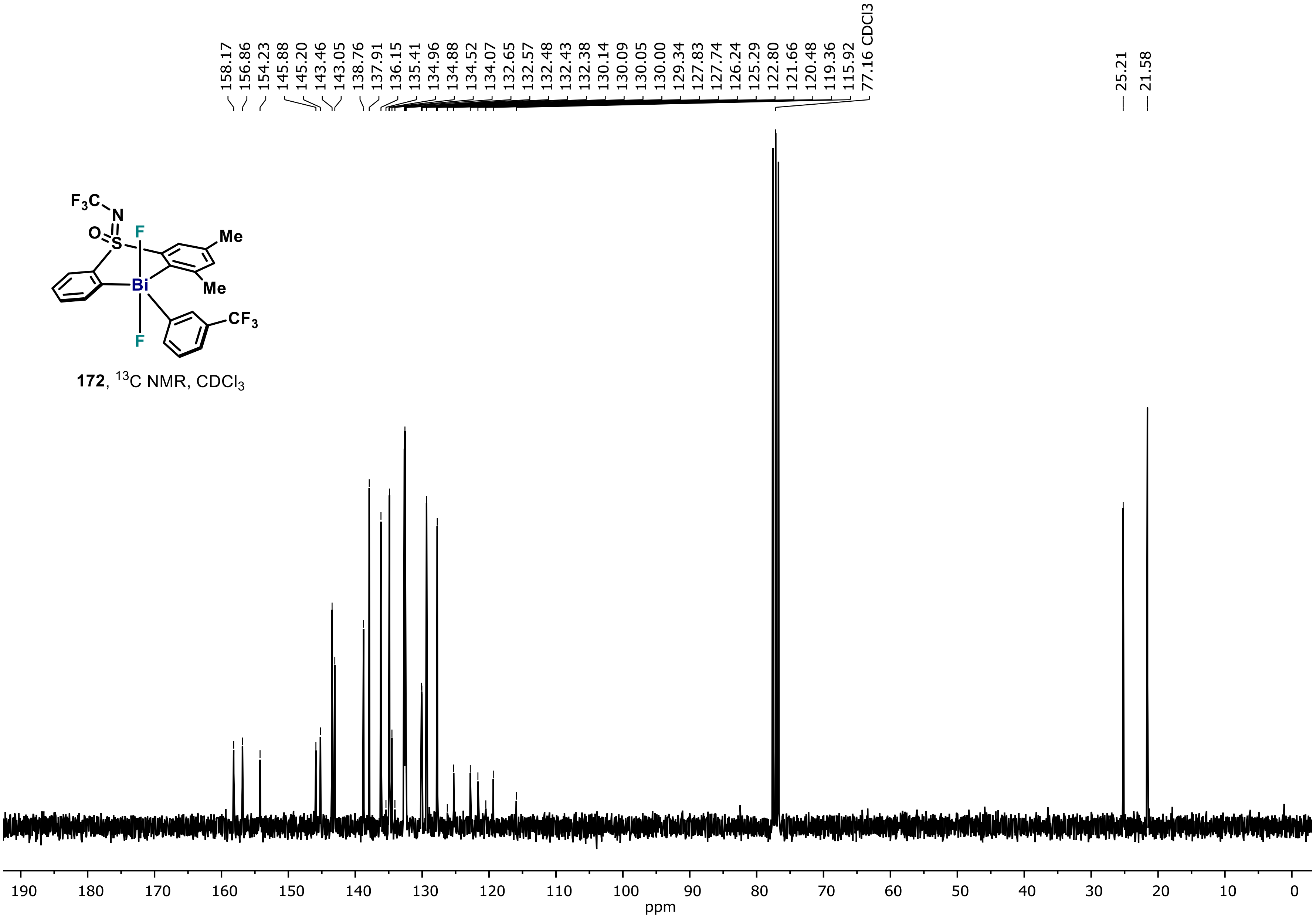

172, ^{13}C NMR, $CDCl_3$

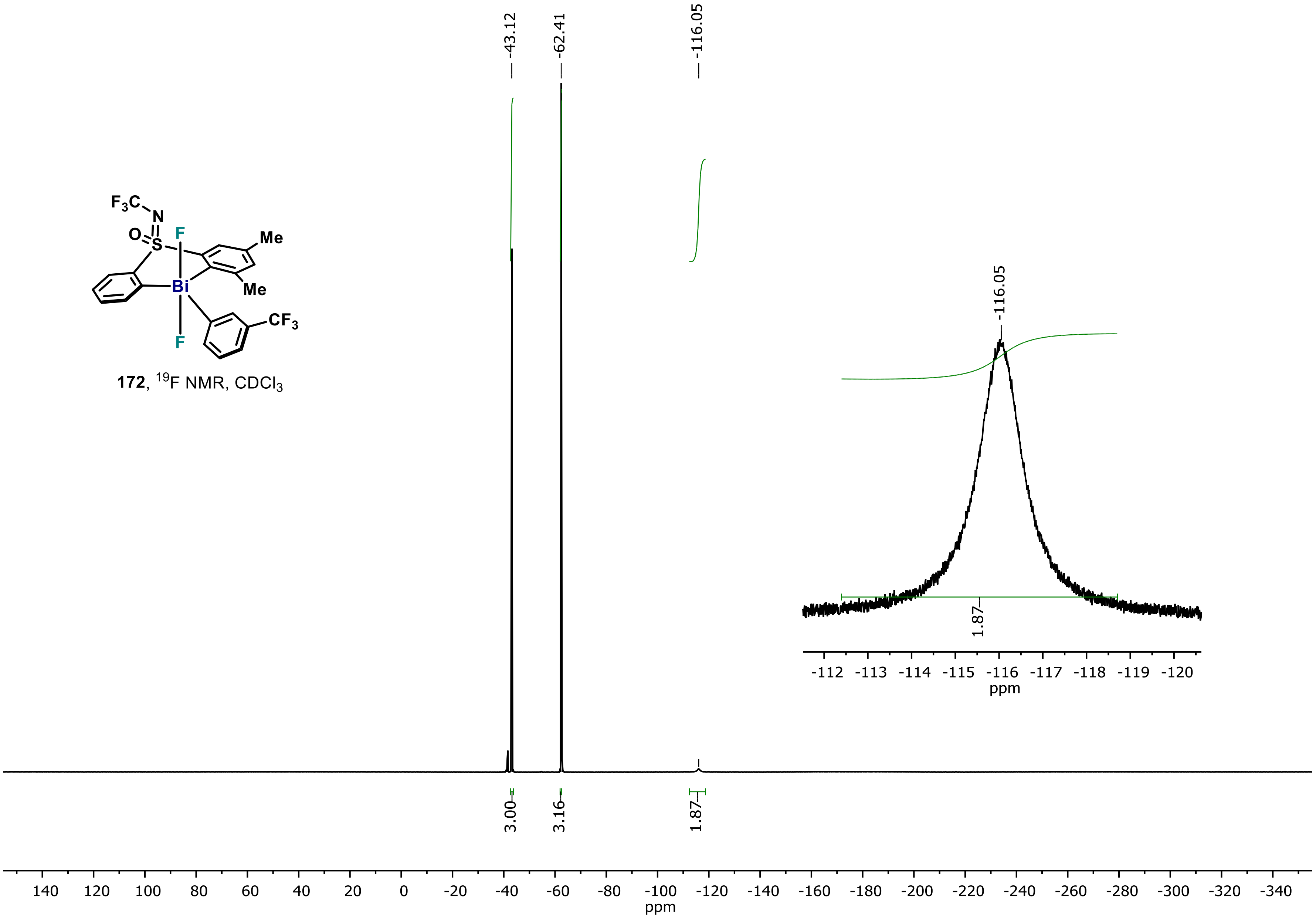

172, ^{19}F NMR, $CDCl_3$

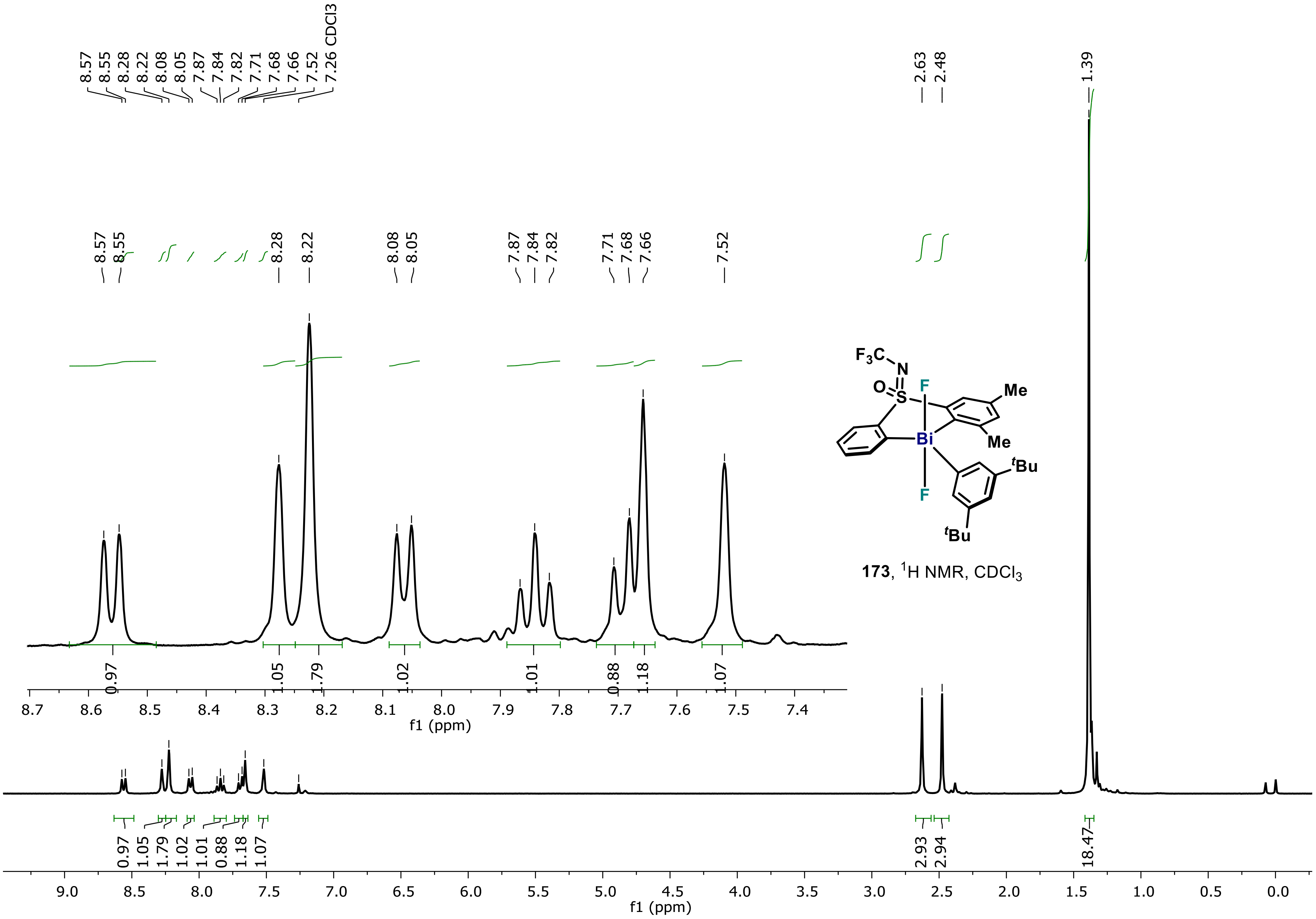
8.57
8.55
8.28
8.22
8.08
8.05
7.87
7.84
7.82
7.71
7.68
7.66
7.52
7.26 CDCl3
2.63
2.48
1.39
0.97
.05
.79
.02
.01
0.88
.18
.07
f1 (ppm)
0.97
1.05
1.79
1.02
1.01
0.88
1.18
1.07
2.93
2.94
18.47
f1 (ppm)
F3C
N
O
S
F
Me
Bi
Me
tBu
F
tBu
173, 1H NMR, CDCl3

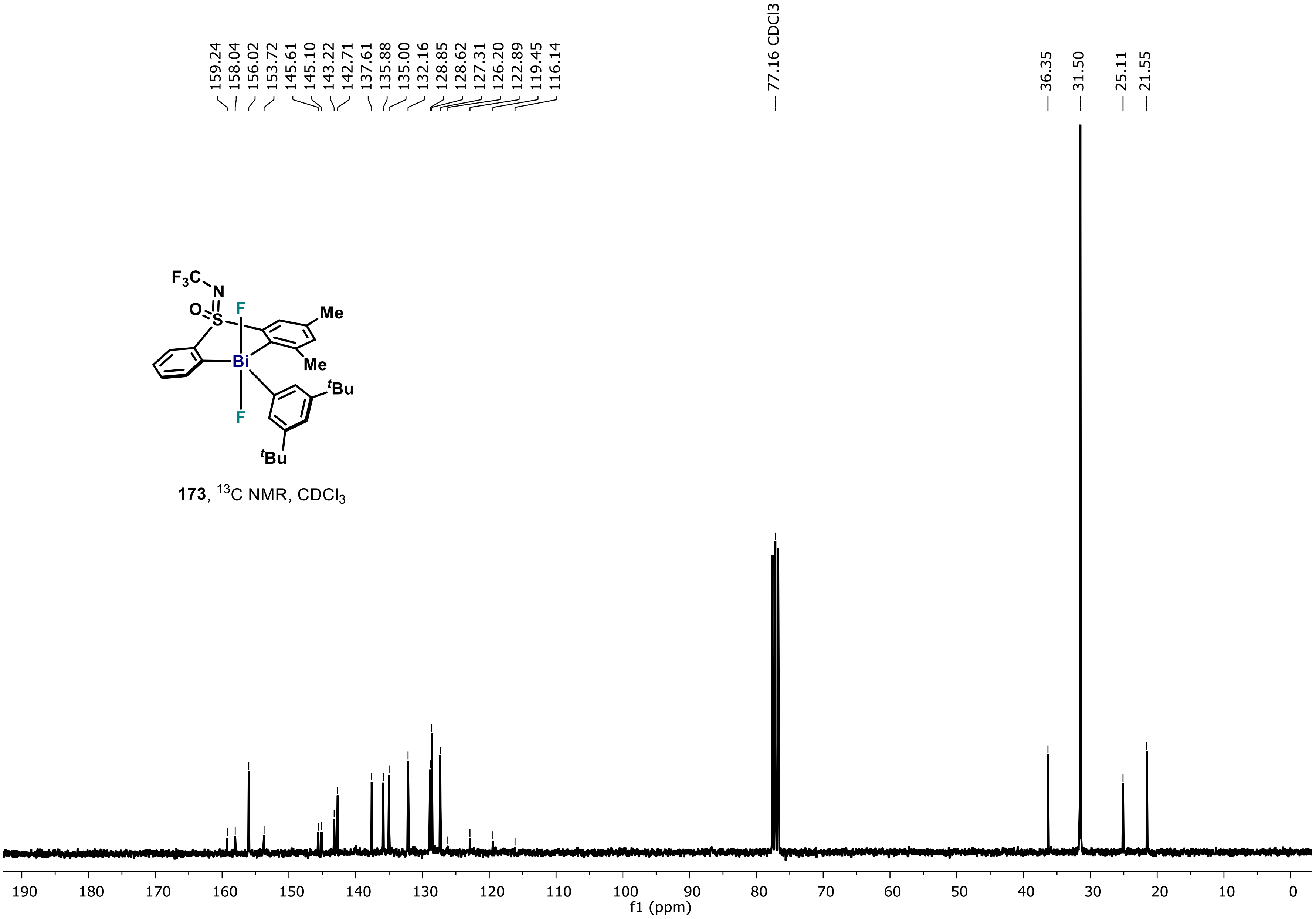

173, ^{13}C NMR, $CDCl_3$

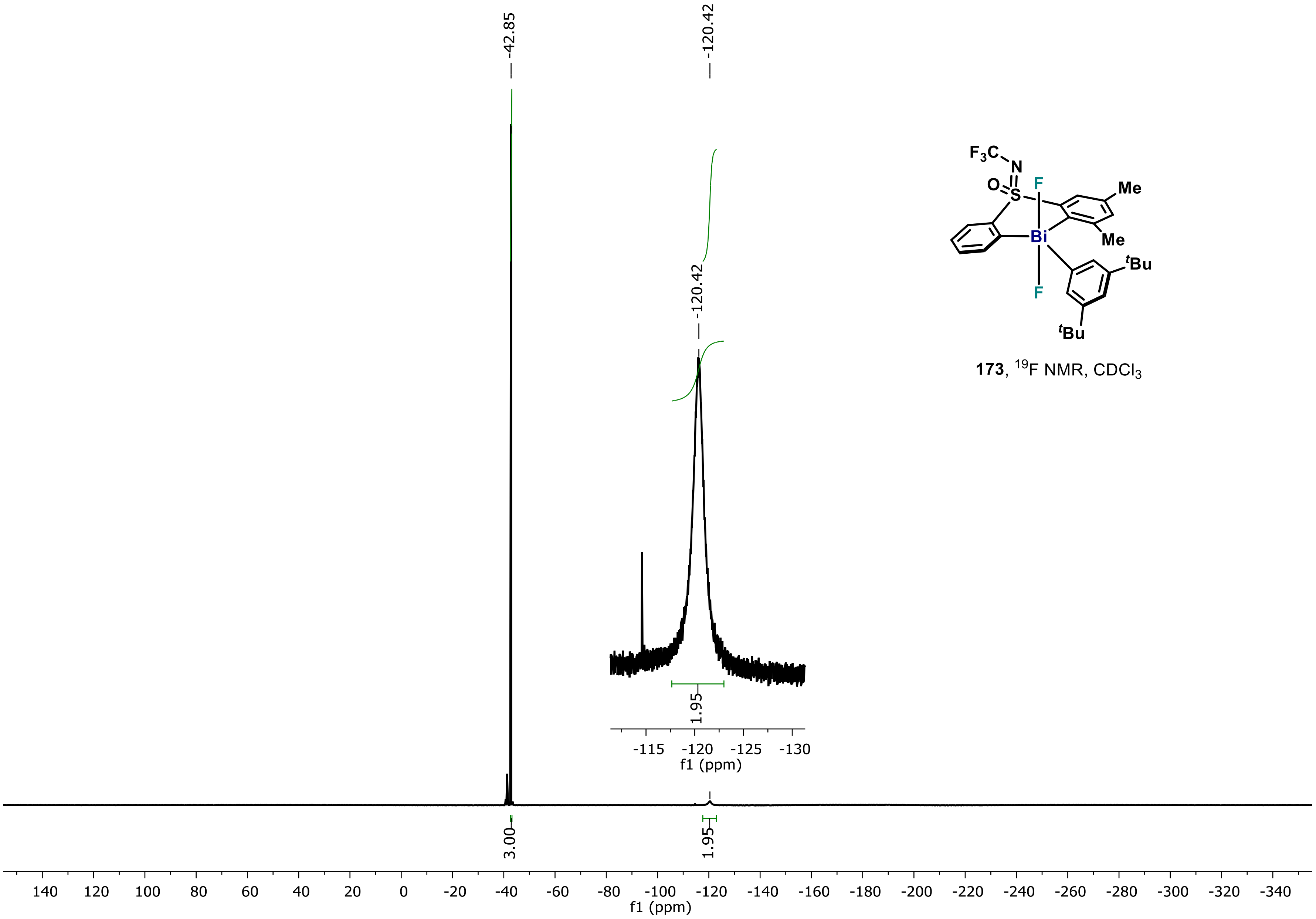

173, ^{19}F NMR, $CDCl_3$

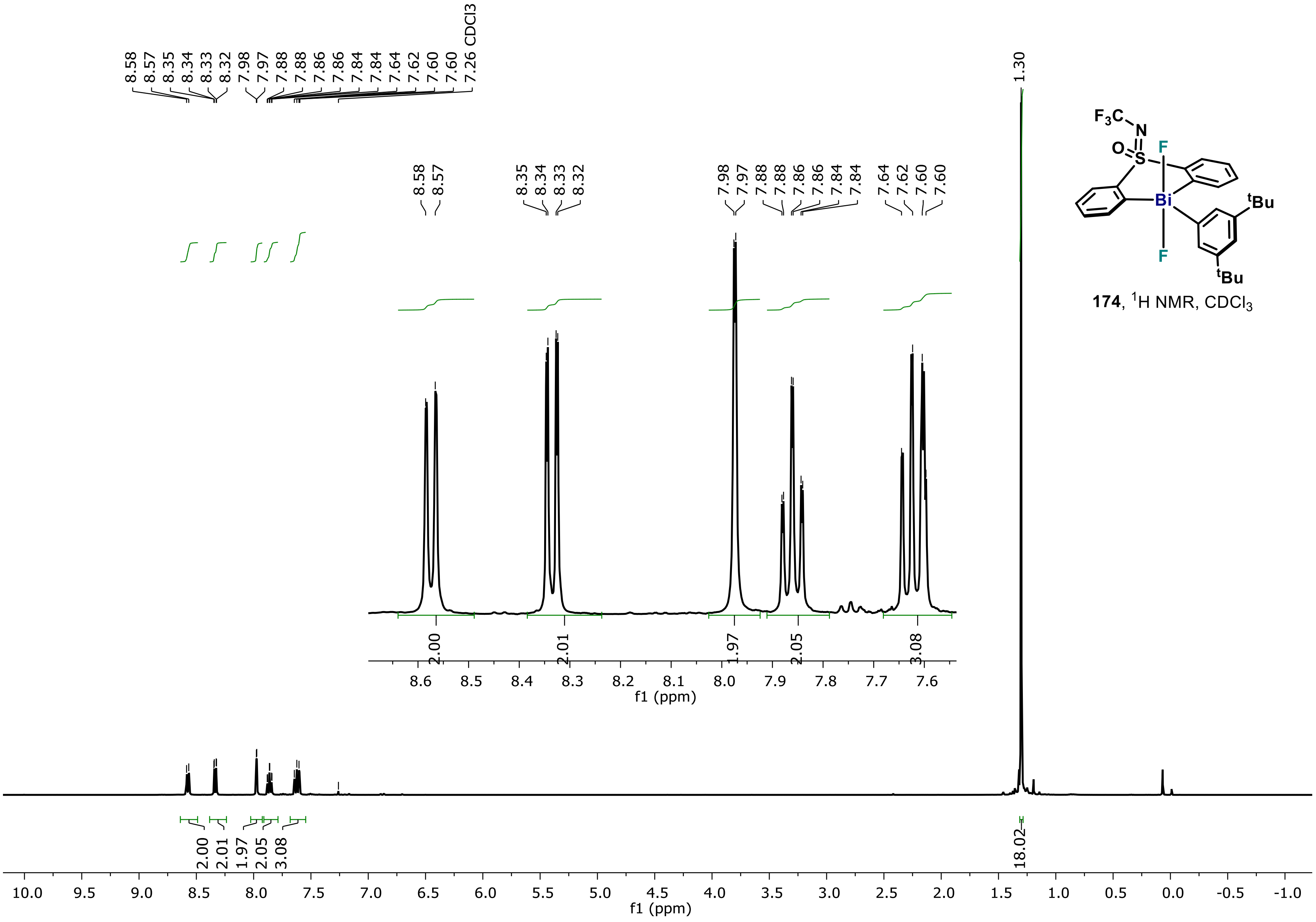

174, ^{1}H NMR, $CDCl_3$

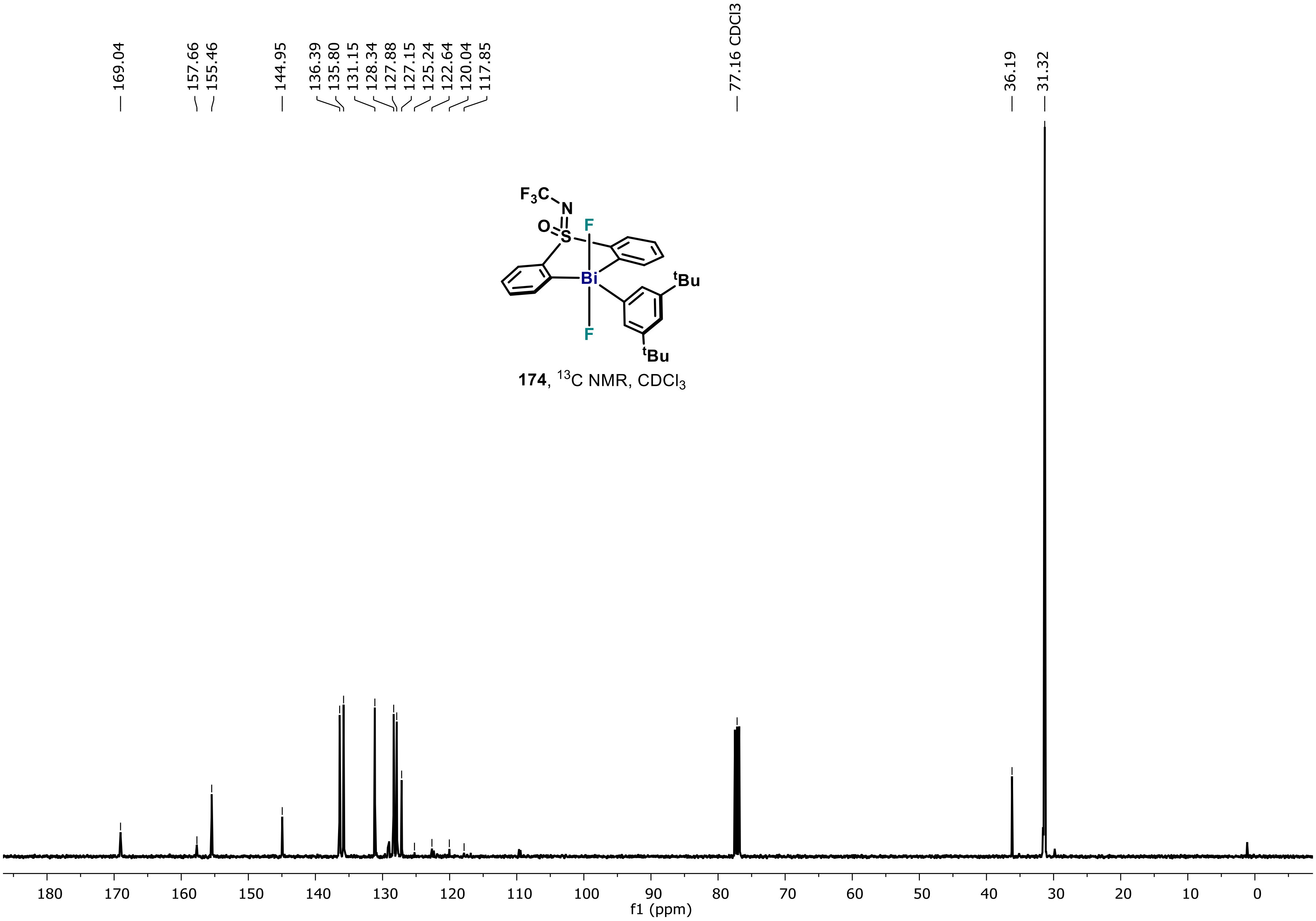

169.04
157.66
155.46
144.95
136.39
135.80
131.15
128.34
127.88
127.15
125.24
122.64
120.04
117.85
77.16 CDCl3
36.19
31.32
F3C
N
O
S
F
Bi
tBu
F
tBu
174, 13C NMR, CDCl3
180
170
160
150
140
130
120
110
100
90
80
70
60
50
40
30
20
10
0
f1 (ppm)

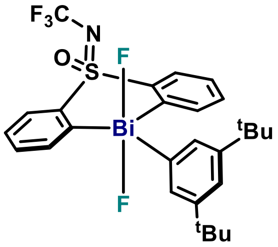

174, ^{19}F NMR, $CDCl_3$

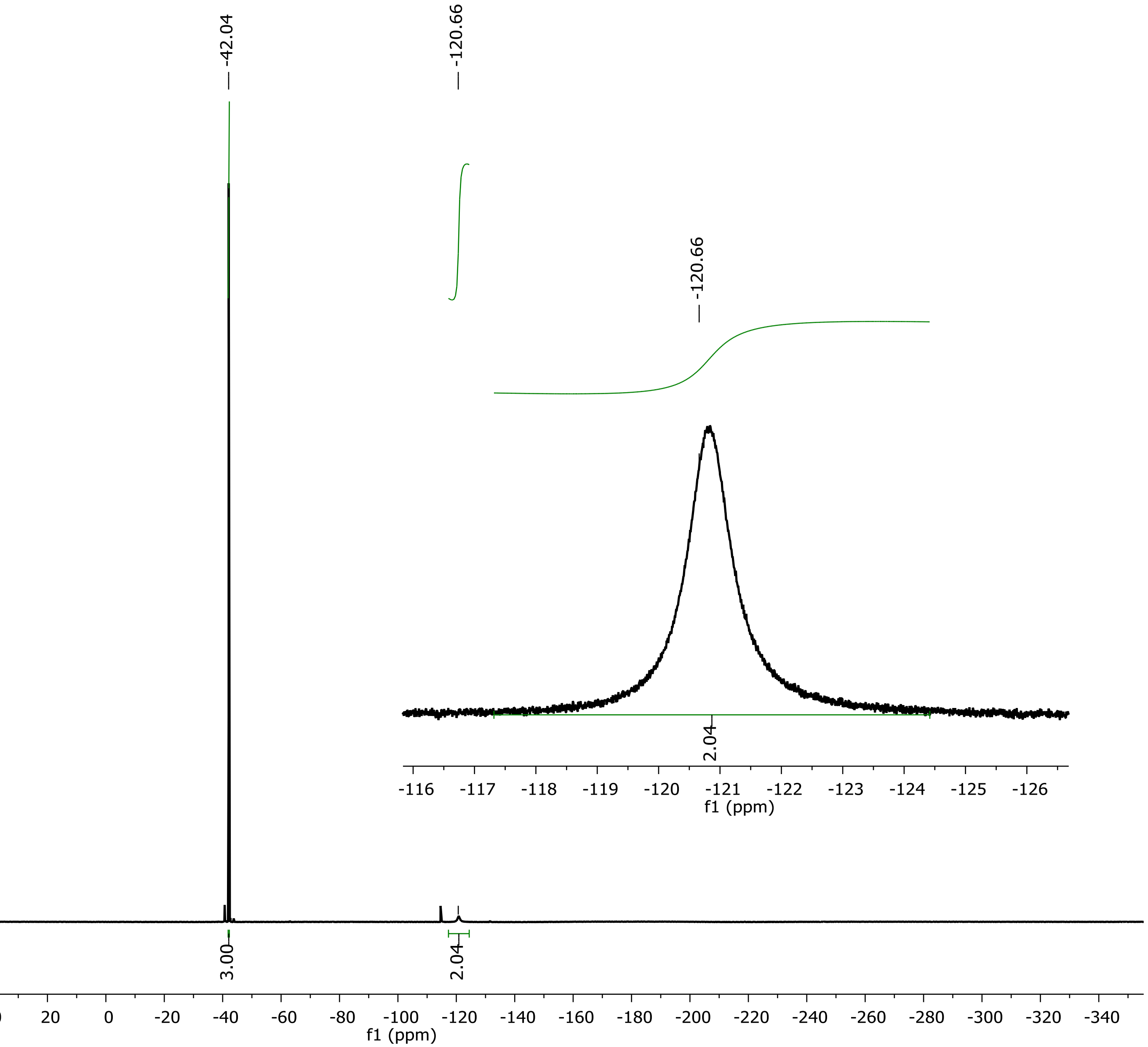

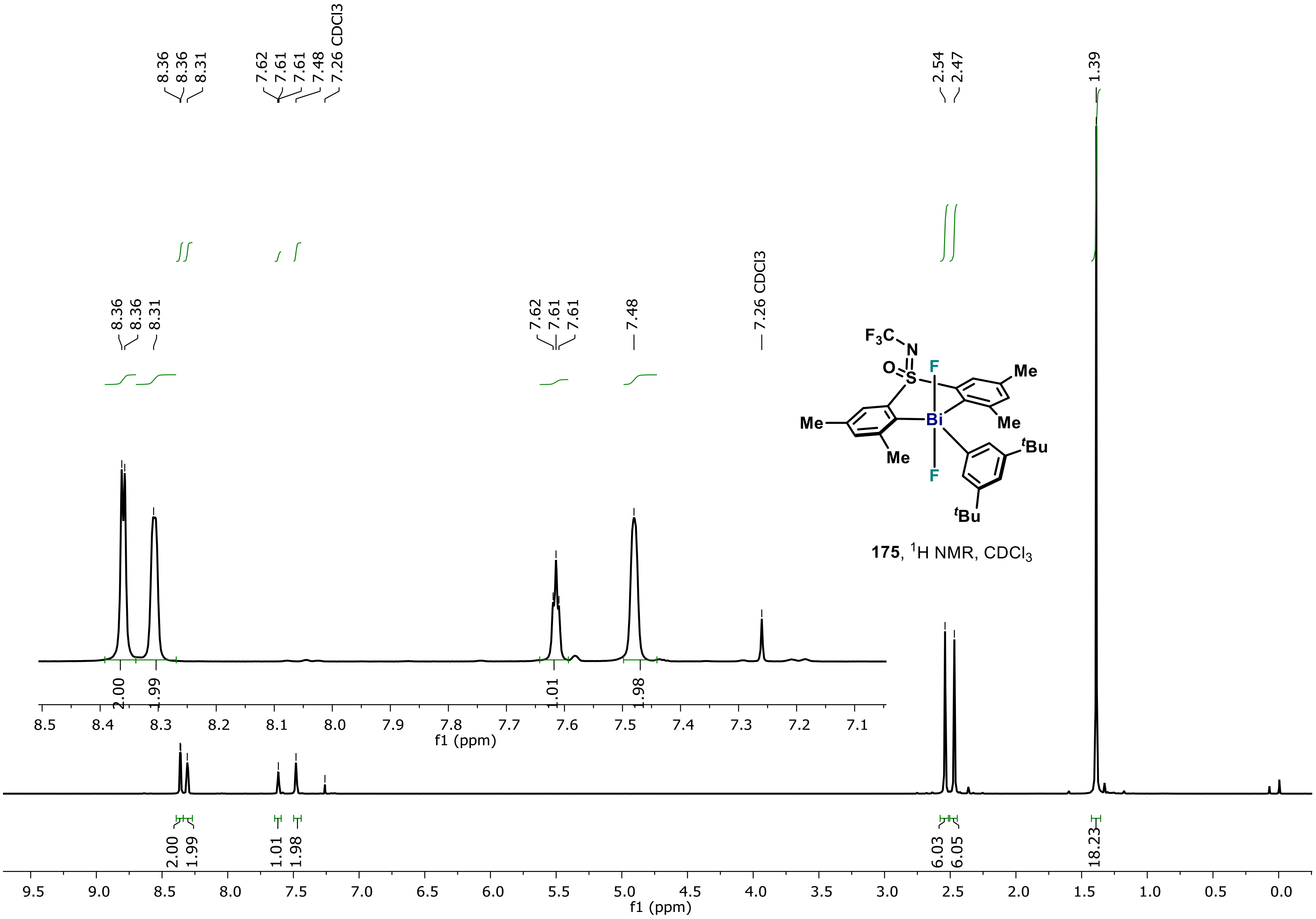

175, ^{1}H NMR, $CDCl_3$

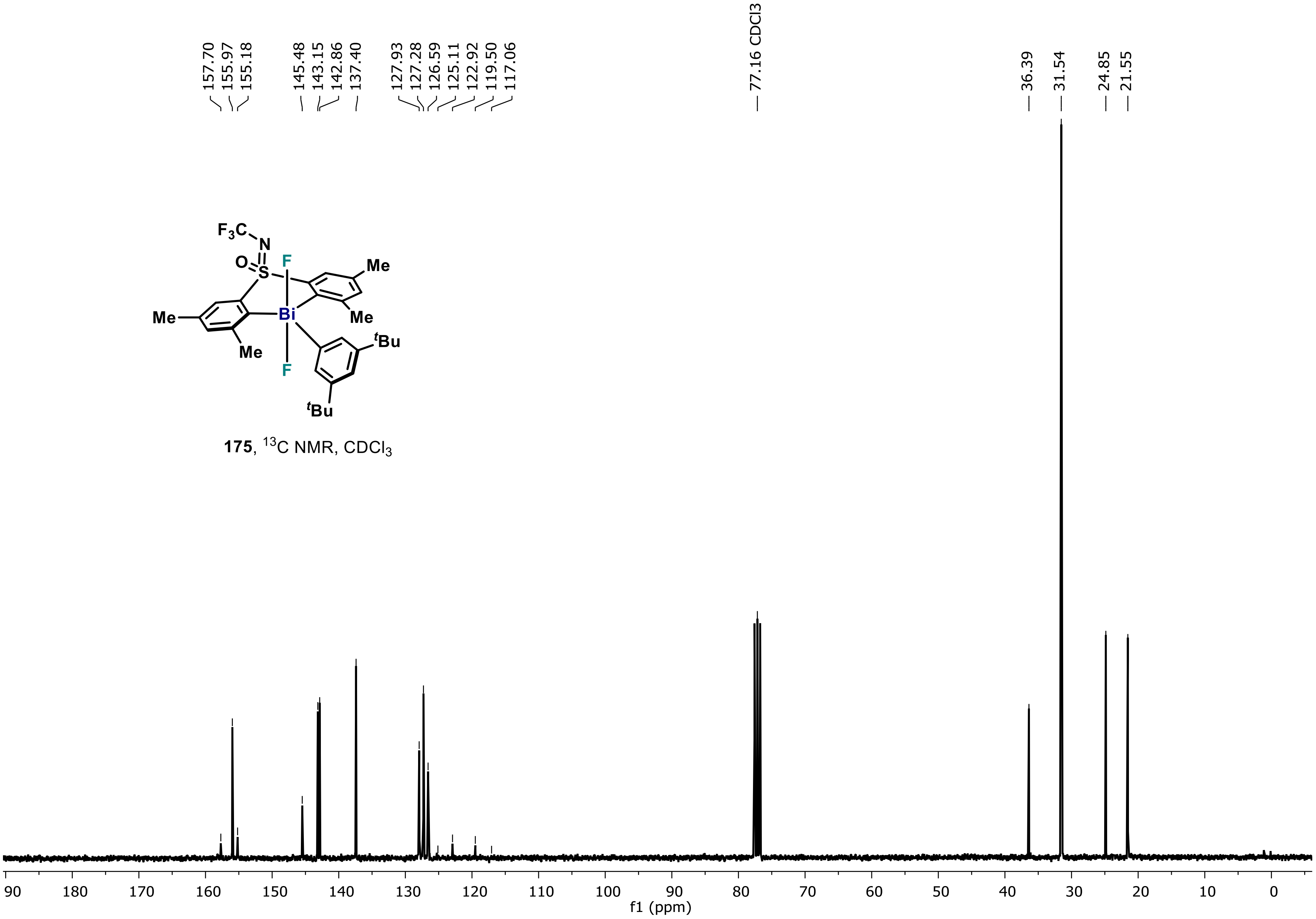

175, ^{13}C NMR, $CDCl_3$

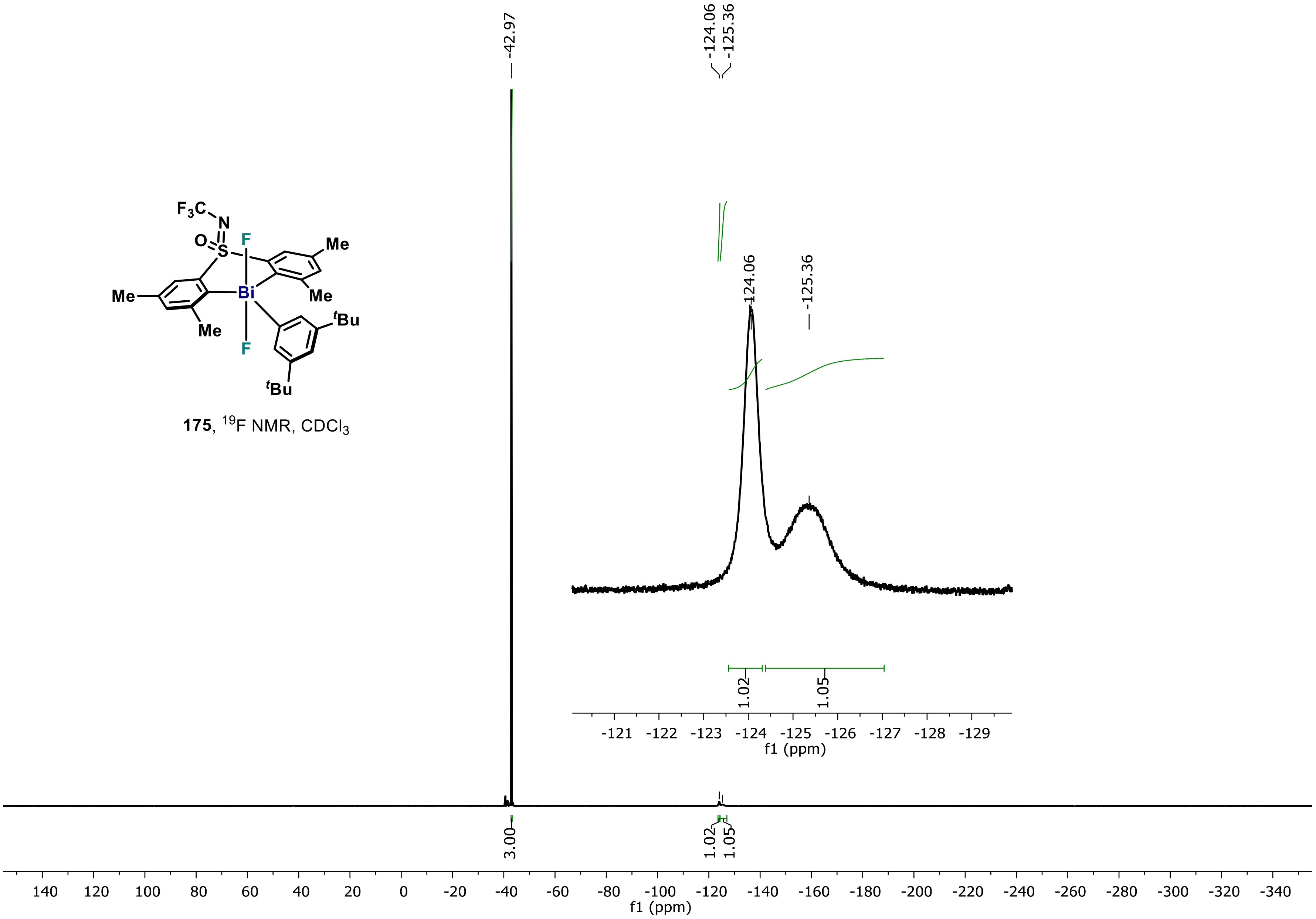

175, ^{19}F NMR, $CDCl_3$

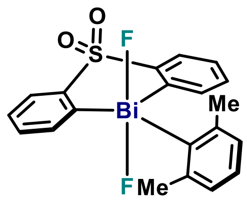

177, ^{1}H NMR, $CDCl_3$

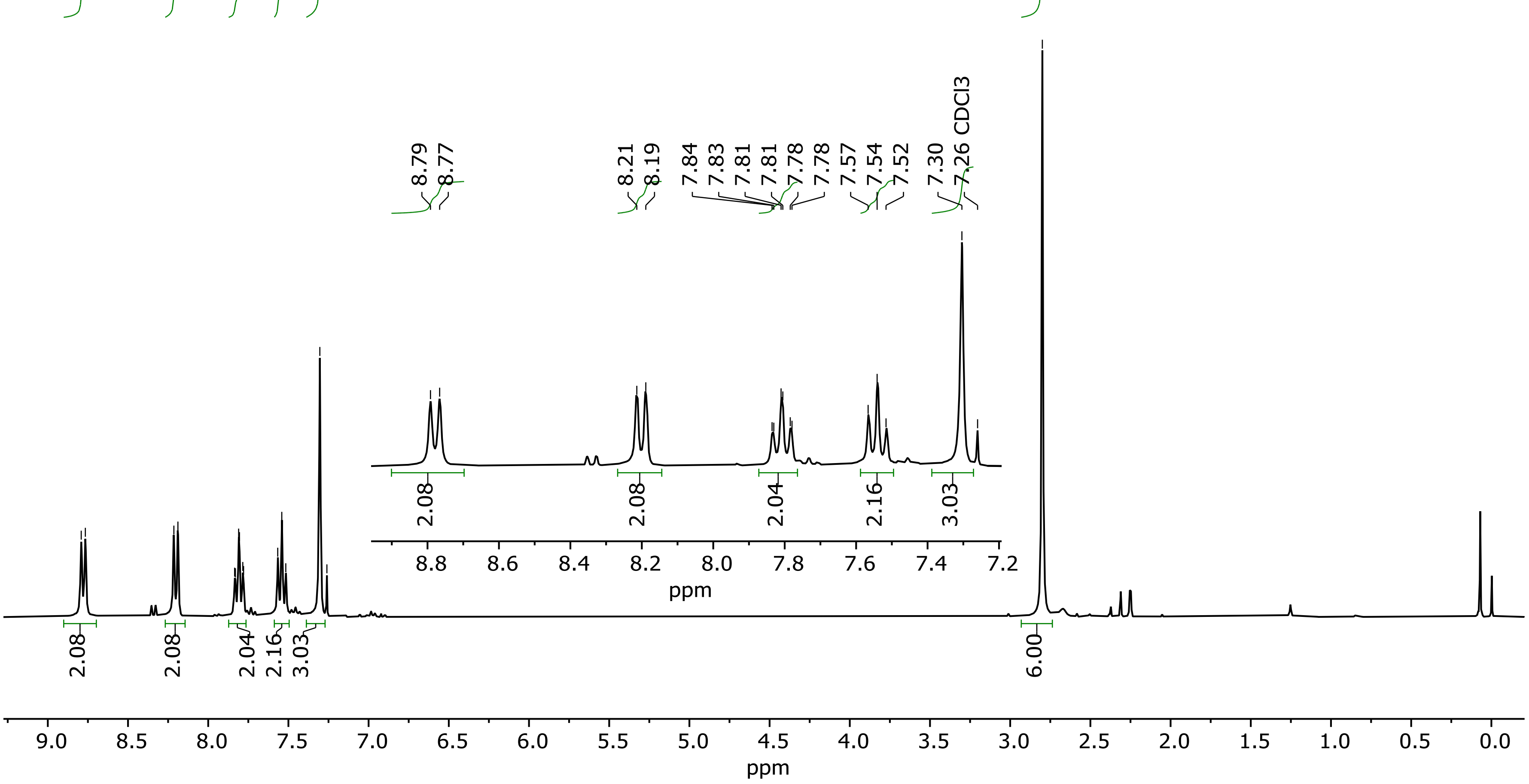

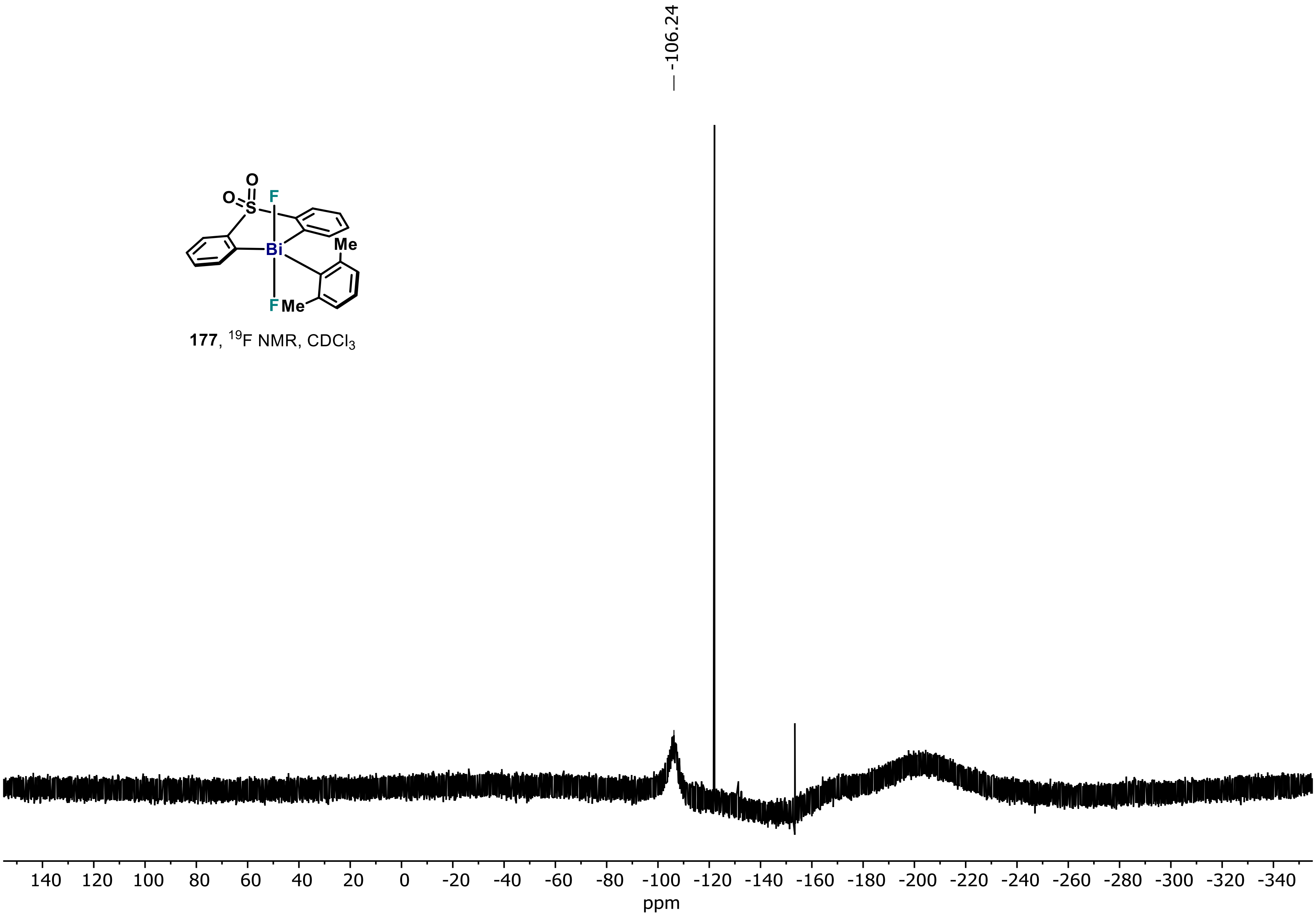

177, ^{19}F NMR, $CDCl_3$

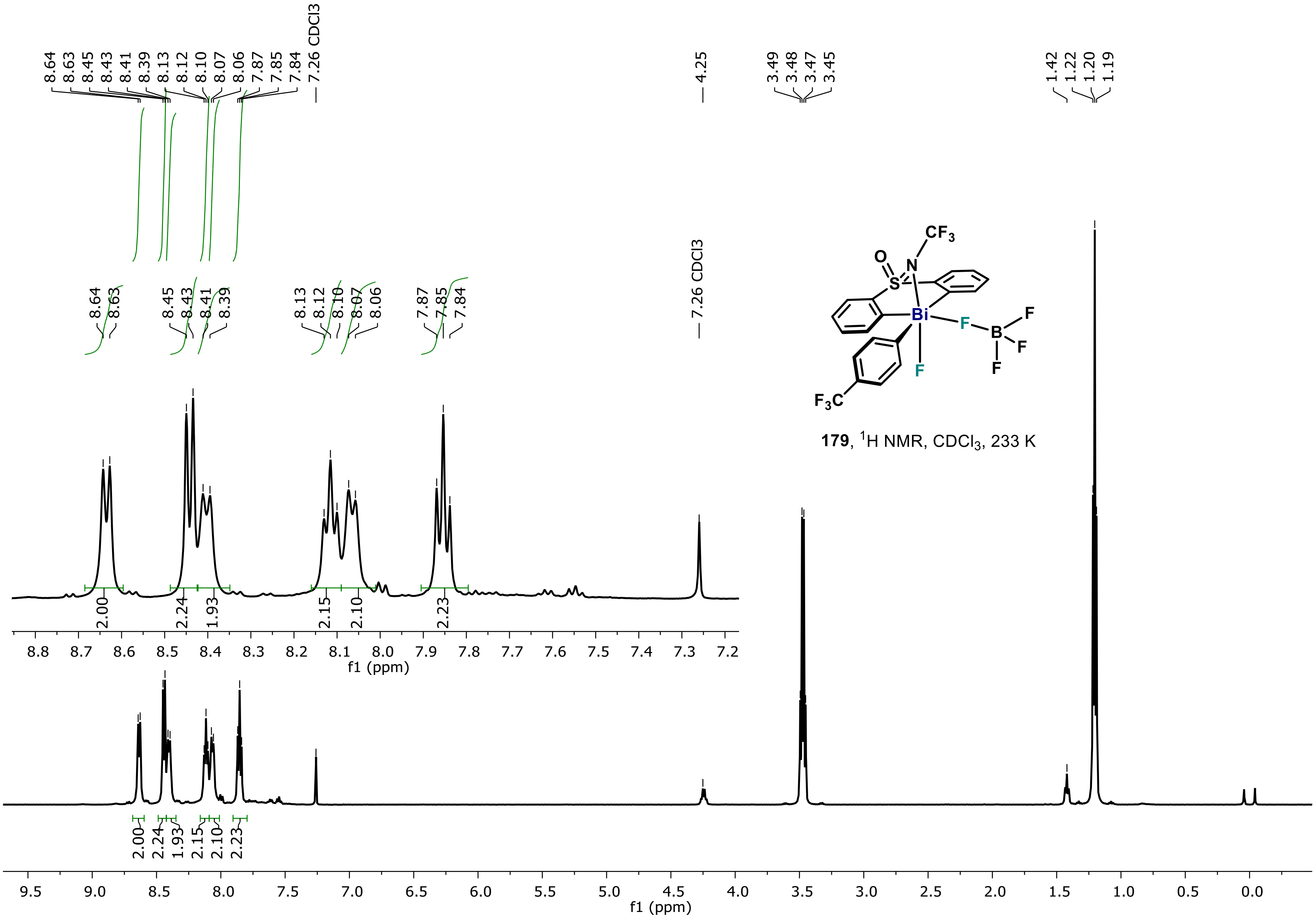
179, ^{1}H NMR, $CDCl_3$, 233 K
8.64 8.63 8.45 8.43 8.41 8.39 8.13 8.12 8.10 8.07 8.06 7.87 7.85 7.84
7.26 CDCl3
4.25
3.49 3.48 3.47 3.45
1.42 1.22 1.20 1.19
2.00 2.24 1.93 2.15 2.10 2.23
f1 (ppm)
CF3
O
S
N
Bi
F
B
F3C

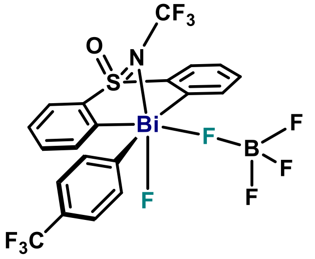

179, ^{11}B NMR, $CDCl_3$, 233 K

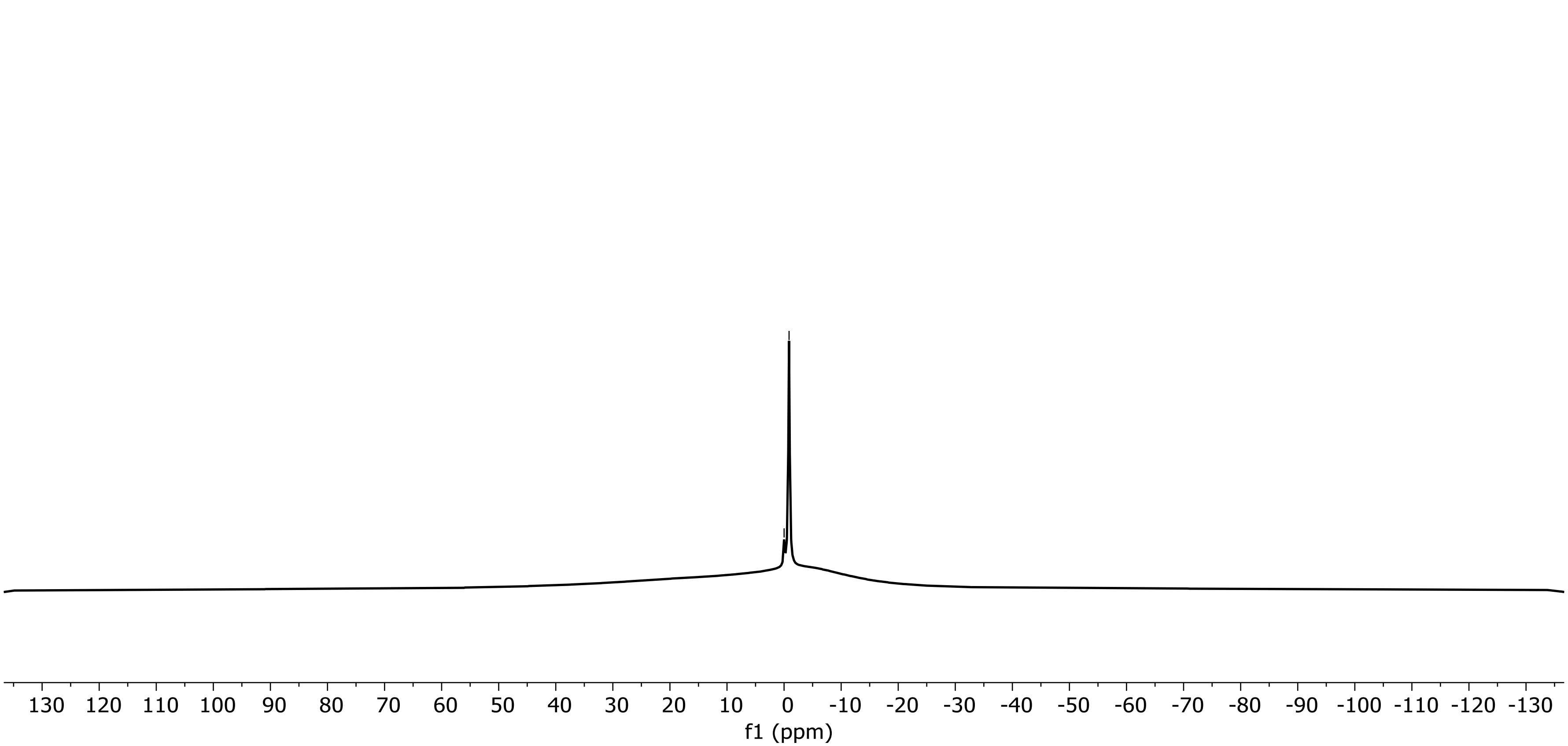

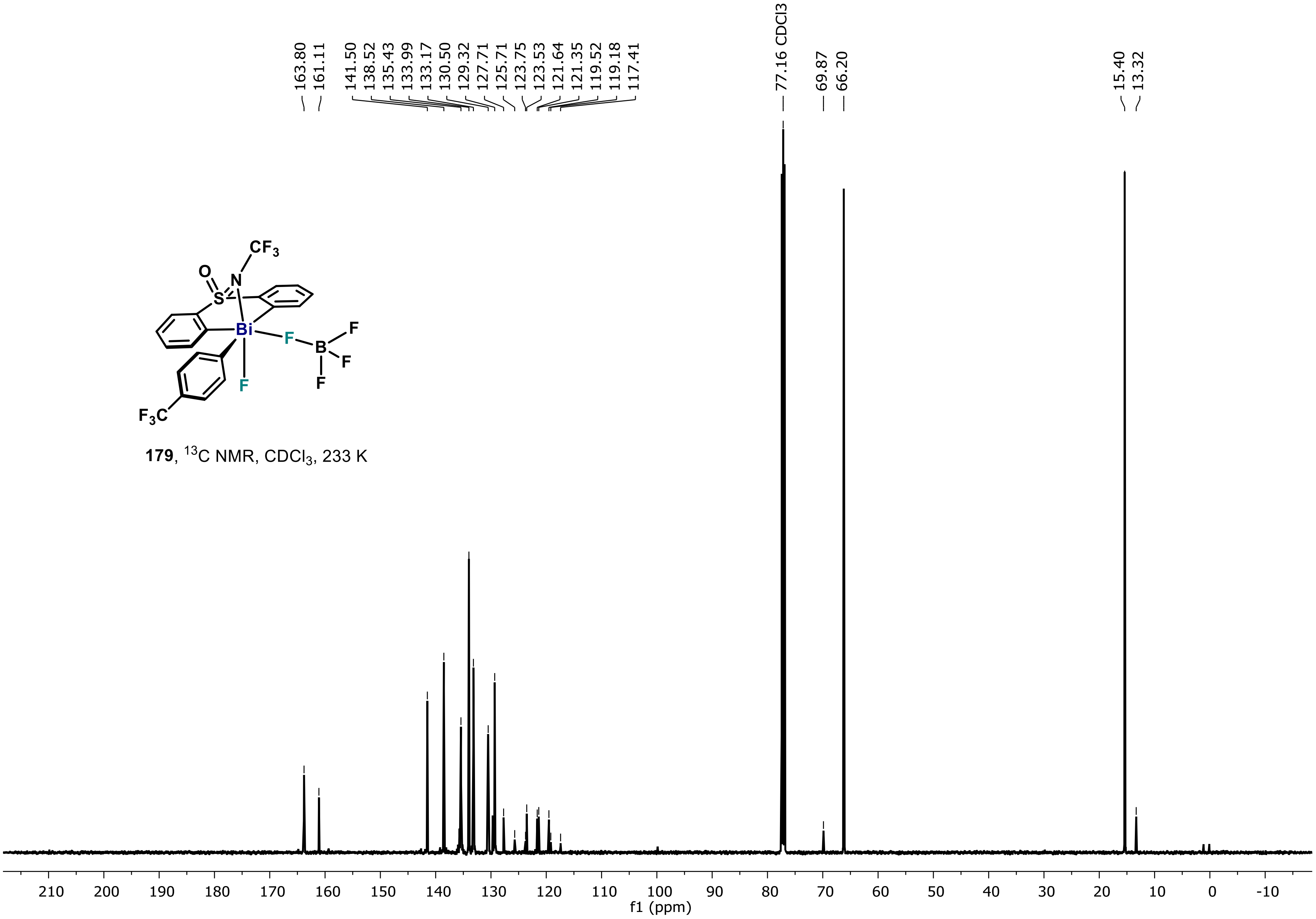

179, ^{13}C NMR, $CDCl_3$, 233 K

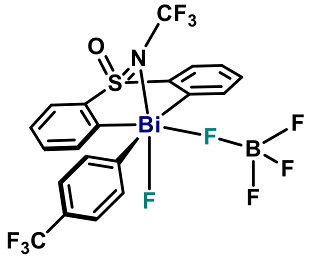

179, ^{19}F NMR, $CDCl_3$, 233 K

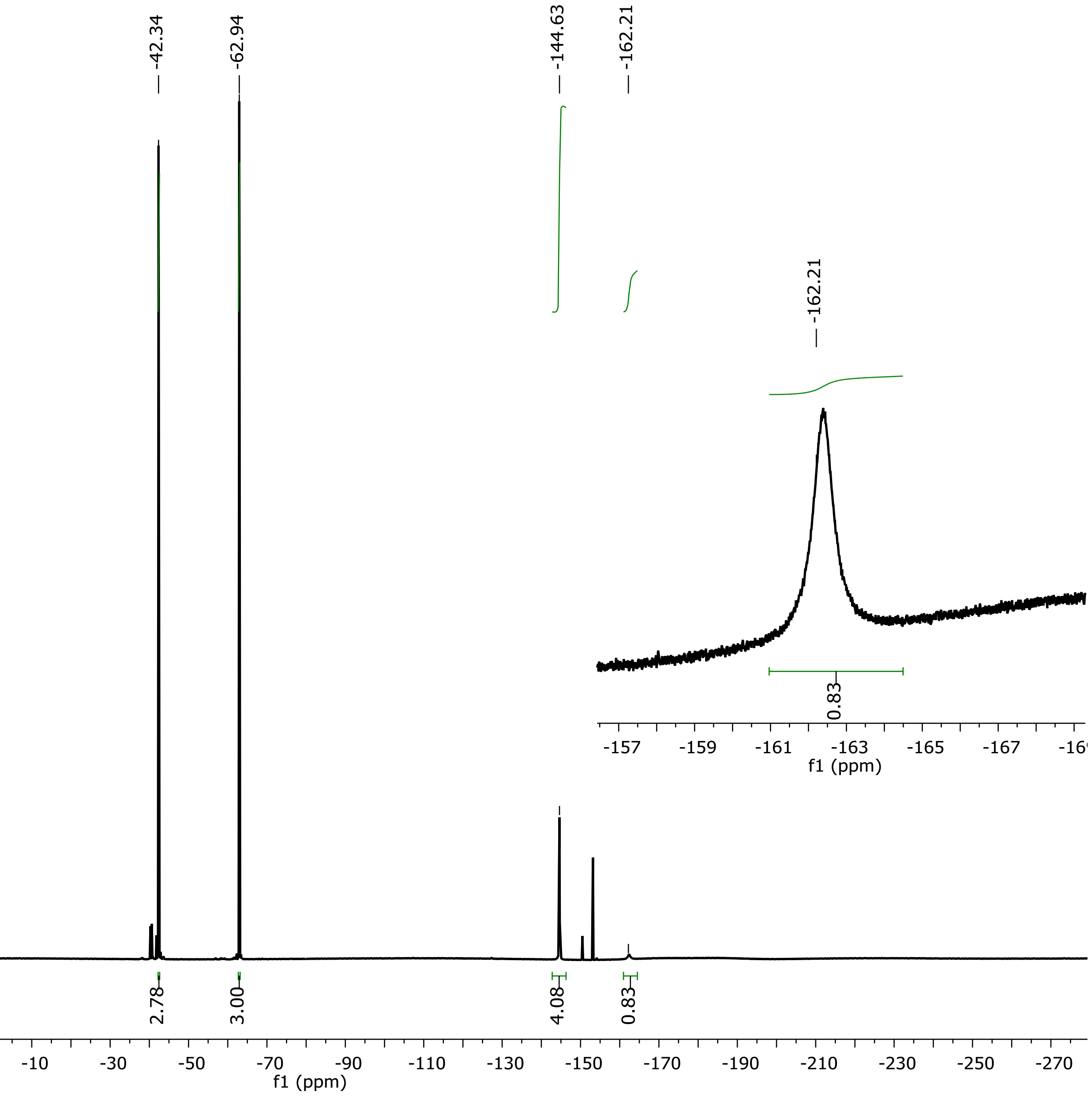

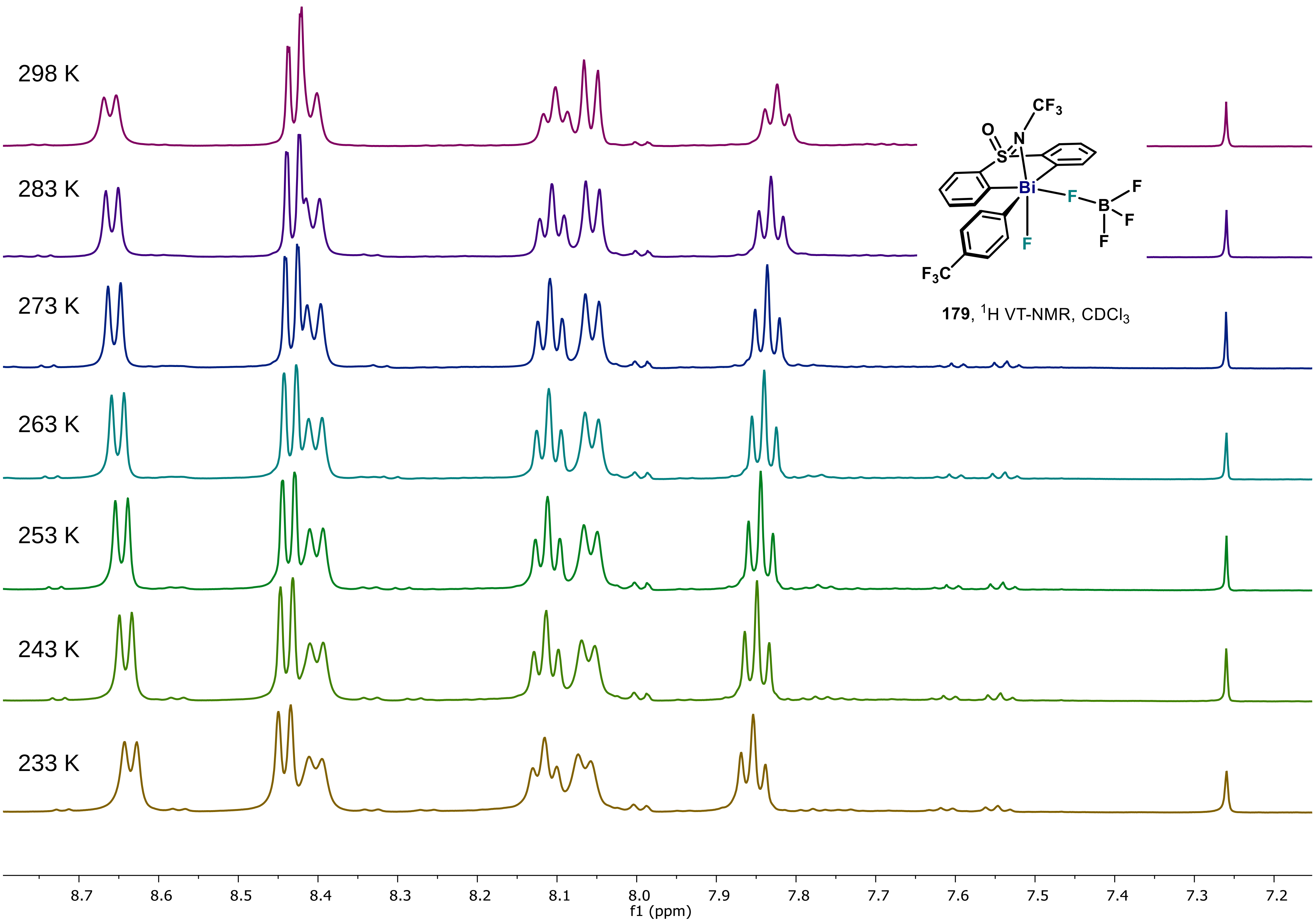
298 K
283 K
273 K
263 K
253 K
243 K
233 K
CF3
O
N
S
Bi
F
B
F
F
F
F
F3C
179, 1H VT-NMR, CDCl3
8.7
8.6
8.5
8.4
8.3
8.2
8.1
8.0
7.9
7.8
7.7
7.6
7.5
7.4
7.3
7.2
f1 (ppm)

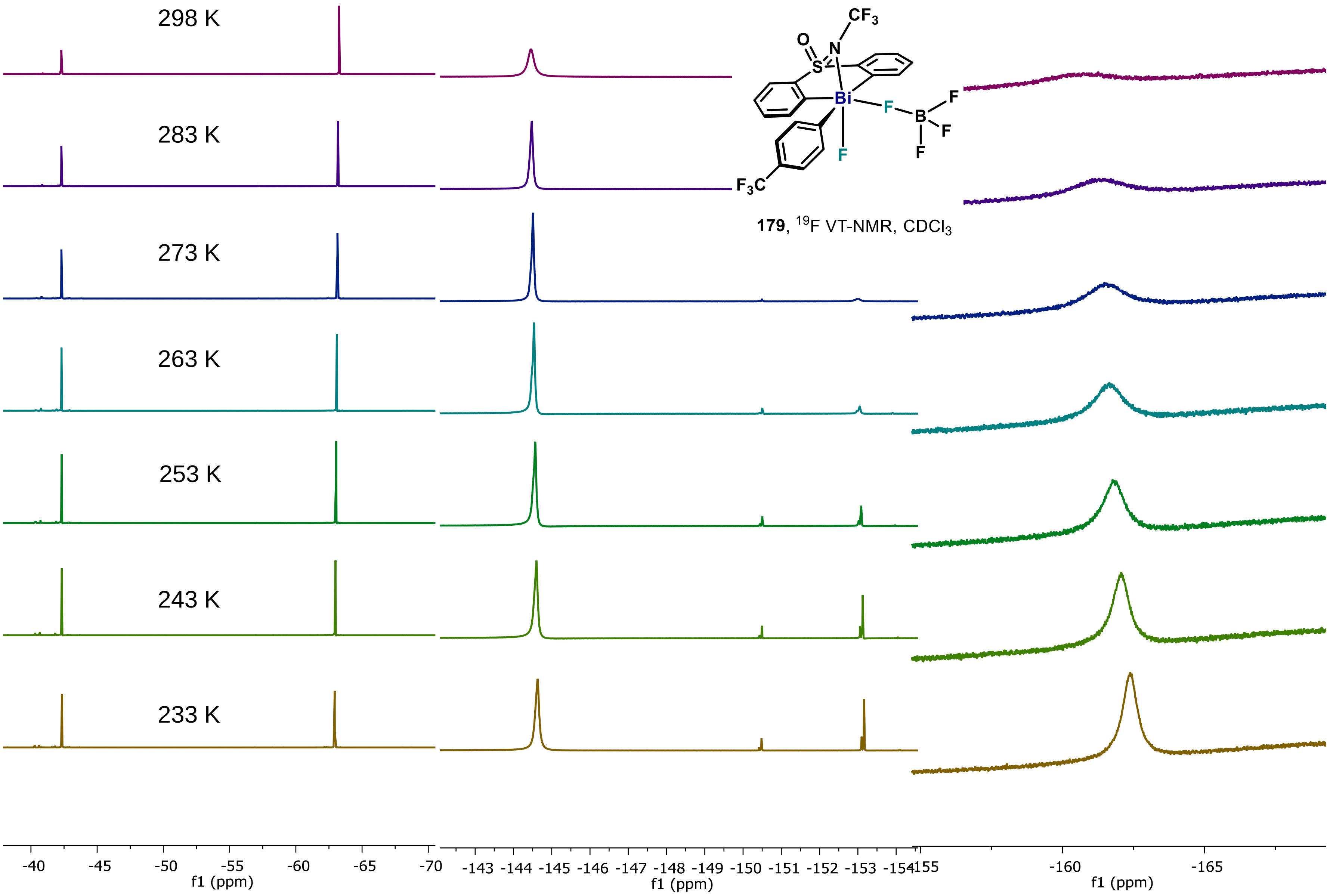
298 K
283 K
273 K
263 K
253 K
243 K
233 K
179, ¹⁹F VT-NMR, CDCl₃
-40 -45 -50 -55 -60 -65 -70
f1 (ppm)
-143 -144 -145 -146 -147 -148 -149 -150 -151 -152 -153 -154
f1 (ppm)
-155 -160 -165
f1 (ppm)

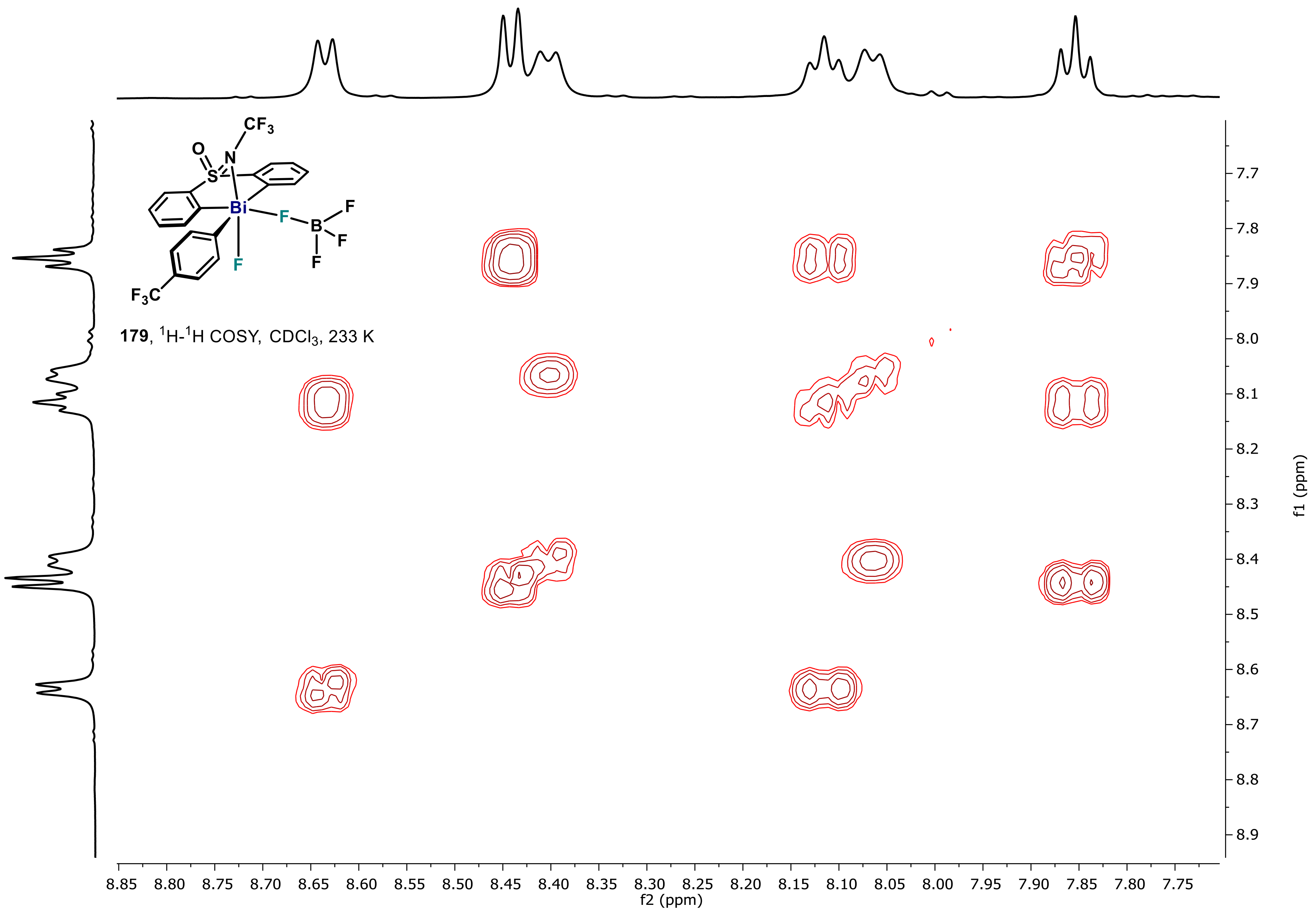

CF3
O
N
S
Bi
F
B
F
F
F
F
F3C
179, 1H-1H COSY, CDCl3, 233 K
7.7
7.8
7.9
8.0
8.1
8.2
8.3
8.4
8.5
8.6
8.7
8.8
8.9
f1 (ppm)
8.85 8.80 8.75 8.70 8.65 8.60 8.55 8.50 8.45 8.40 8.35 8.30 8.25 8.20 8.15 8.10 8.05 8.00 7.95 7.90 7.85 7.80 7.75
f2 (ppm)

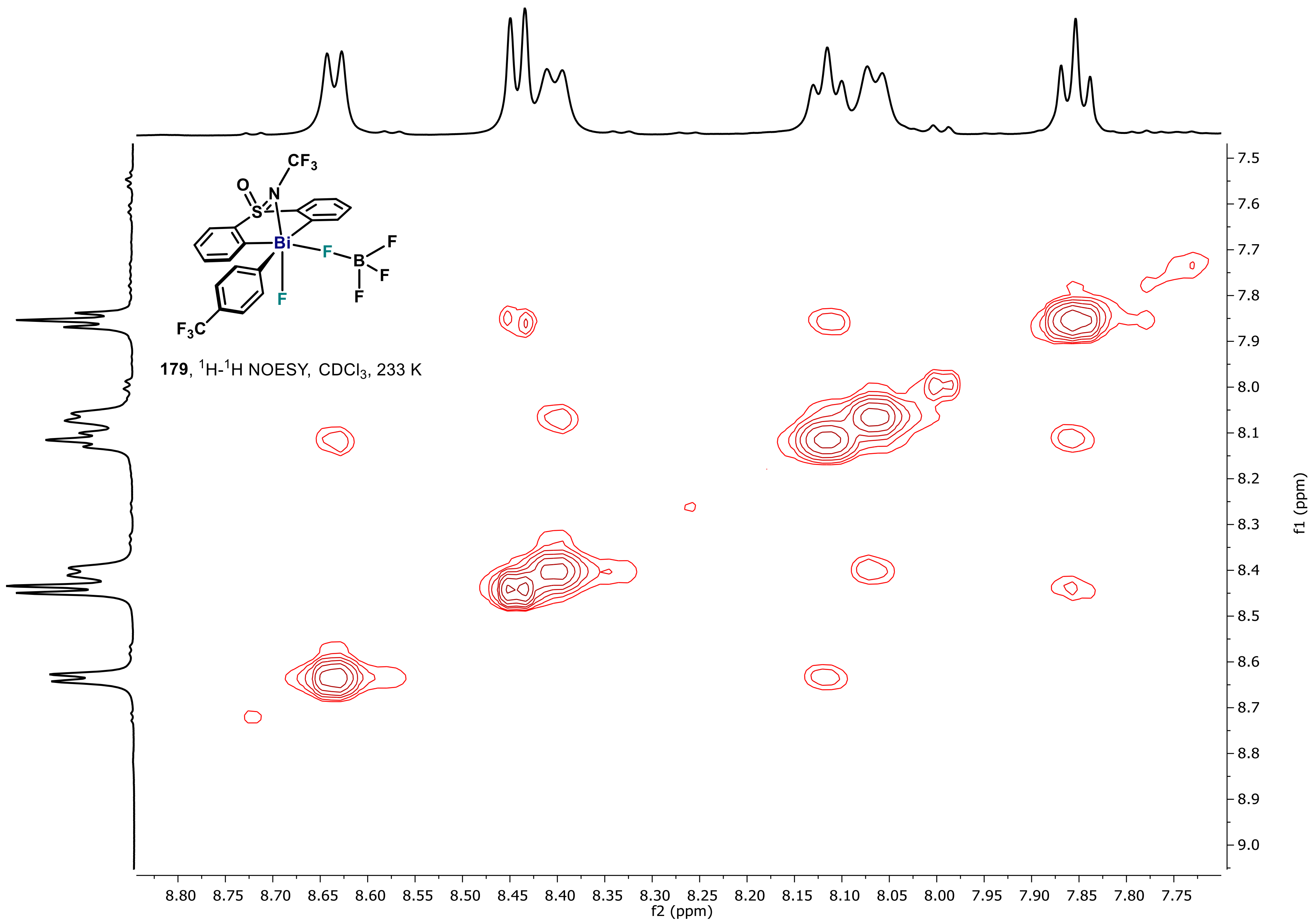
179, 1H-1H NOESY, CDCl3, 233 K
f2 (ppm)
f1 (ppm)

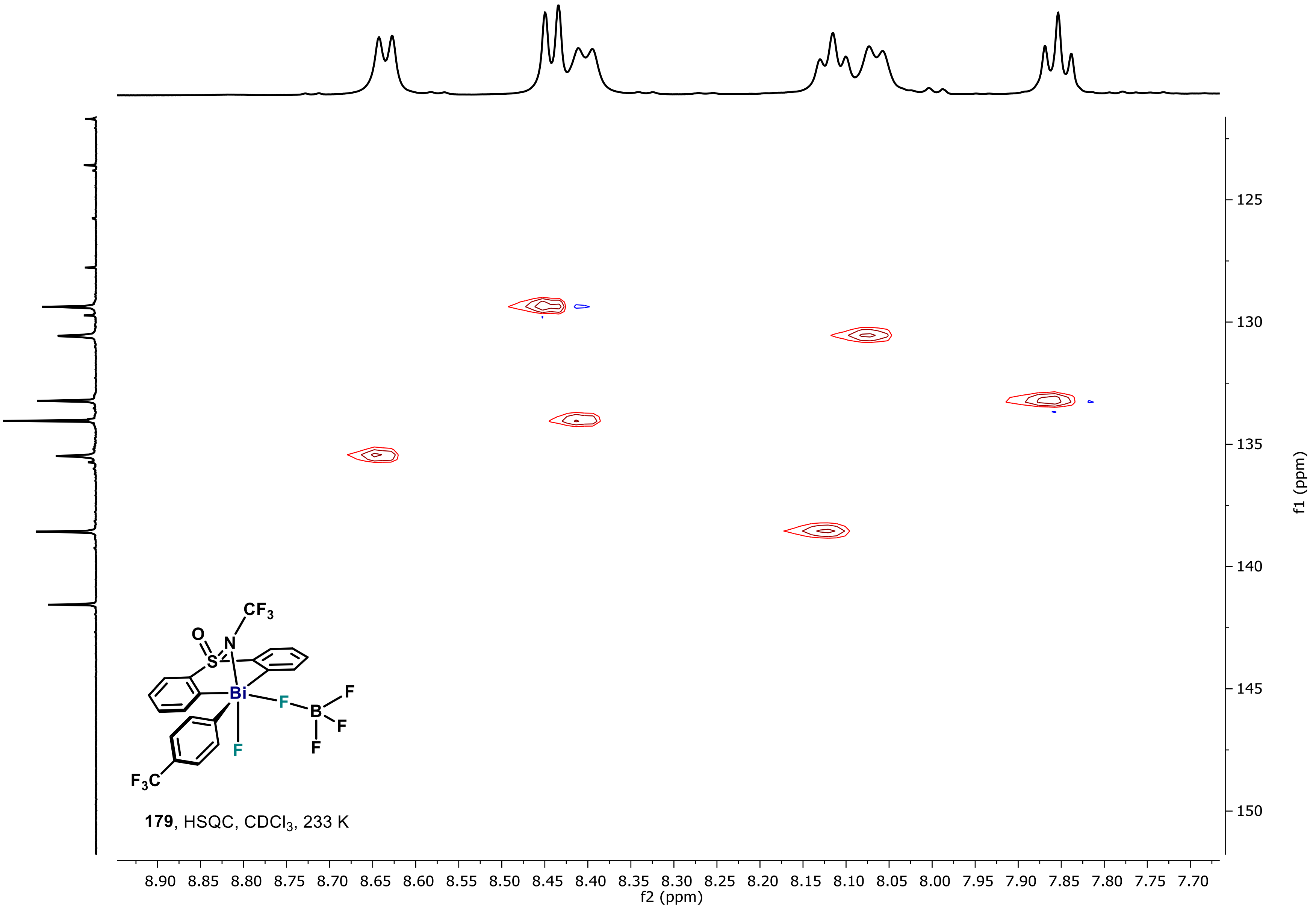

CF3
O
N
S
Bi
F
B
F
F
F
F
F3C
179, HSQC, CDCl3, 233 K
f1 (ppm)
125
130
135
140
145
150
8.90 8.85 8.80 8.75 8.70 8.65 8.60 8.55 8.50 8.45 8.40 8.35 8.30 8.25 8.20 8.15 8.10 8.05 8.00 7.95 7.90 7.85 7.80 7.75 7.70
f2 (ppm)

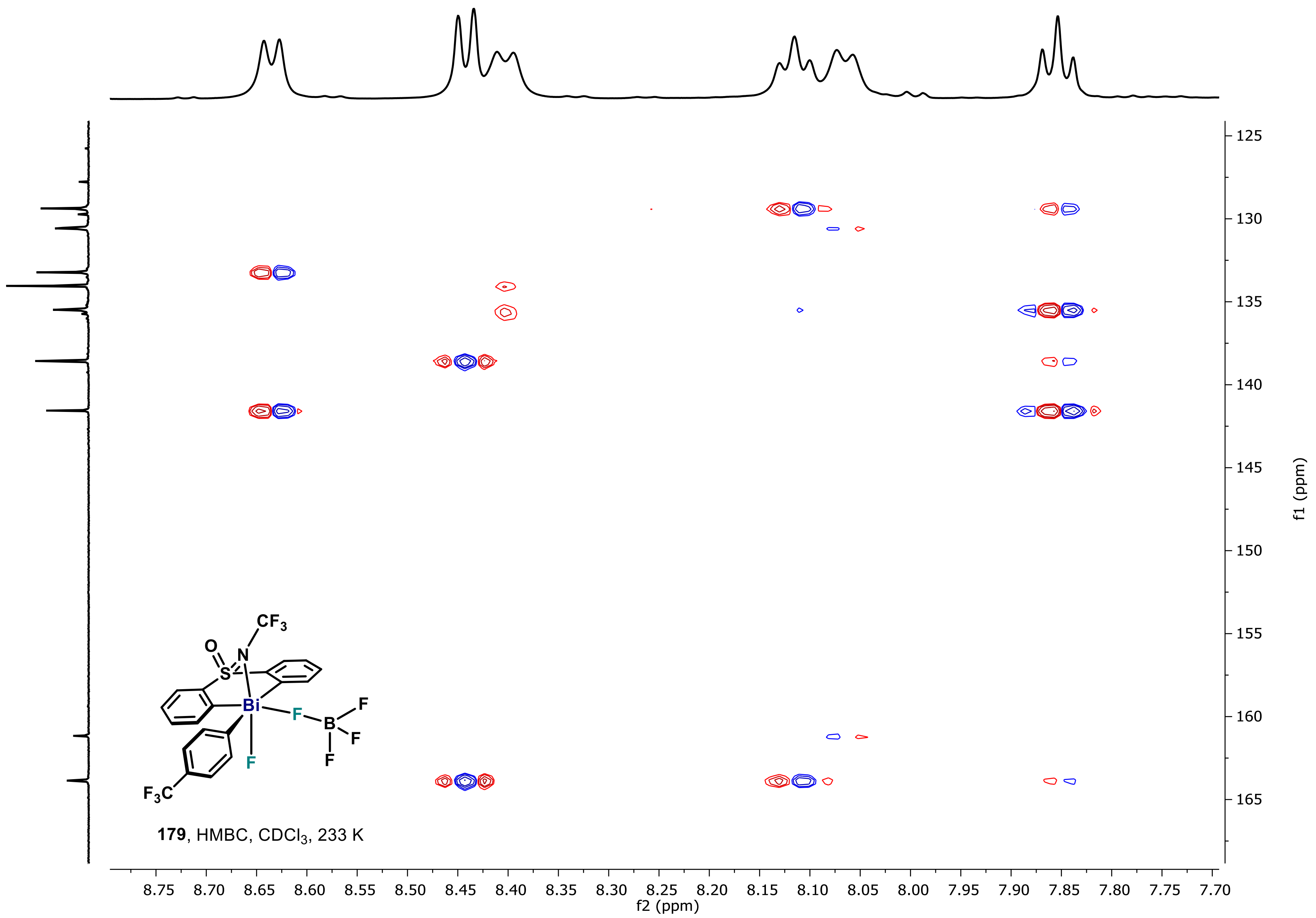

CF3
O
N
S
Bi
F
B
F
F
F
F
F3C
179, HMBC, CDCl3, 233 K
f2 (ppm)
f1 (ppm)

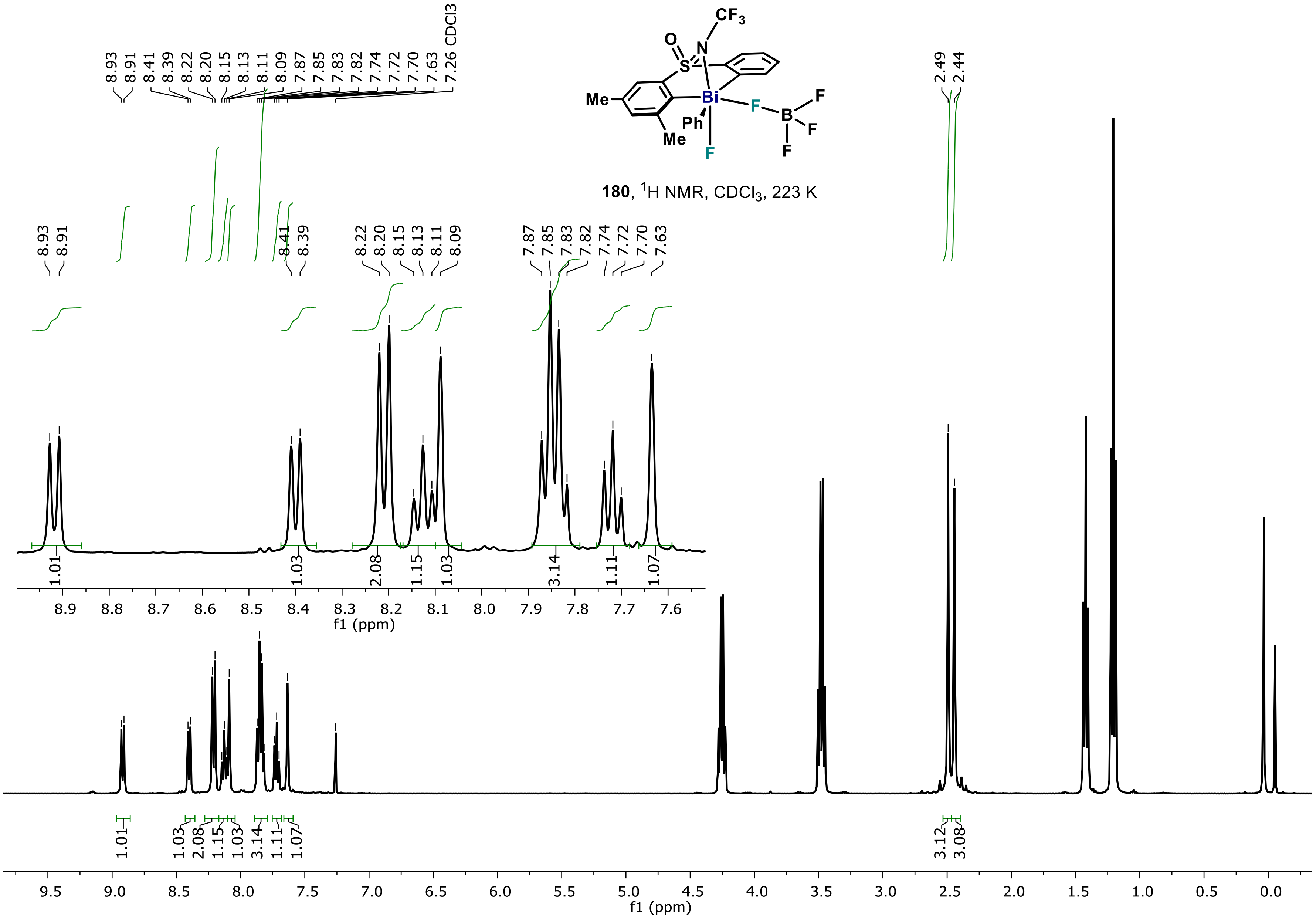
CF3
O
N
S
Me
Bi
F
B
F
F
Ph
Me
F
180, 1H NMR, CDCl3, 223 K
8.93
8.91
8.41
8.39
8.22
8.20
8.15
8.13
8.11
8.09
7.87
7.85
7.83
7.82
7.74
7.72
7.70
7.63
7.26 CDCl3
2.49
2.44
1.01
1.03
2.08
1.15
1.03
3.14
1.11
1.07
3.12
3.08
f1 (ppm)

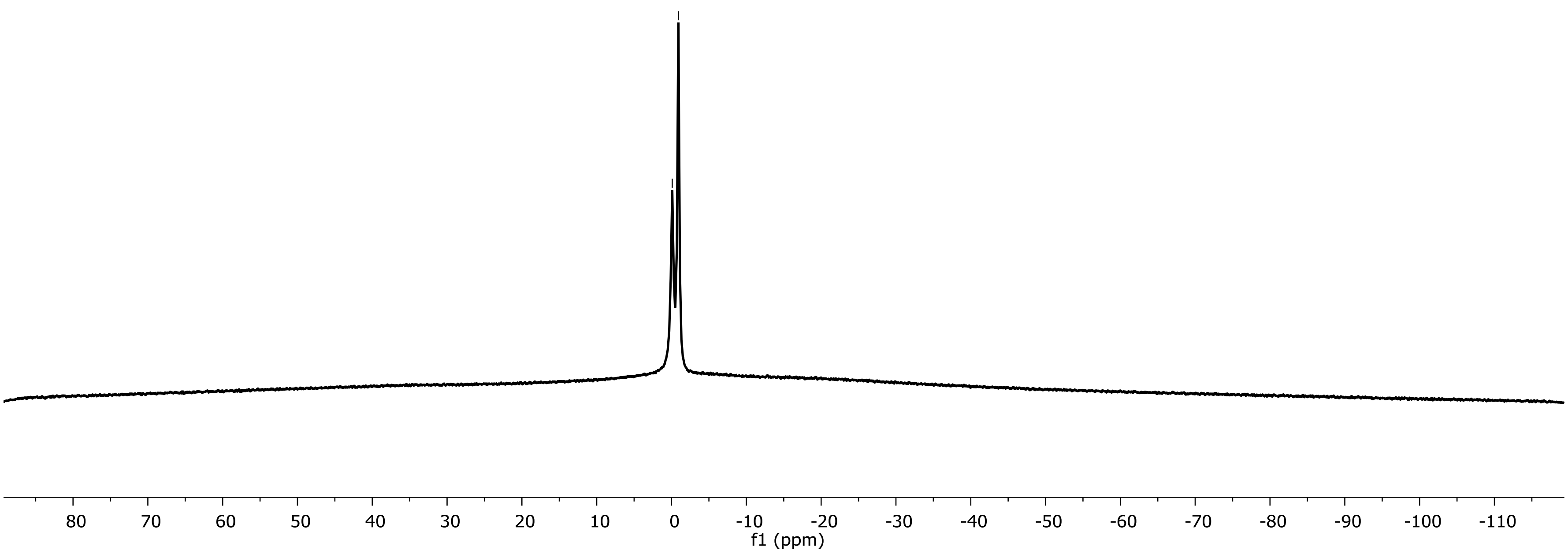

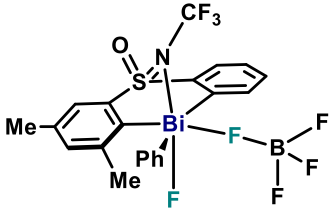

180, ^{11}B NMR, $CDCl_3$, 223 K

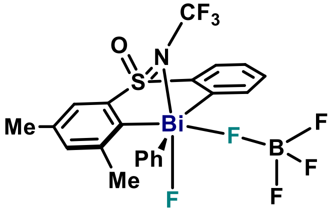

180, ^{13}C NMR, $CDCl_3$, 223 K

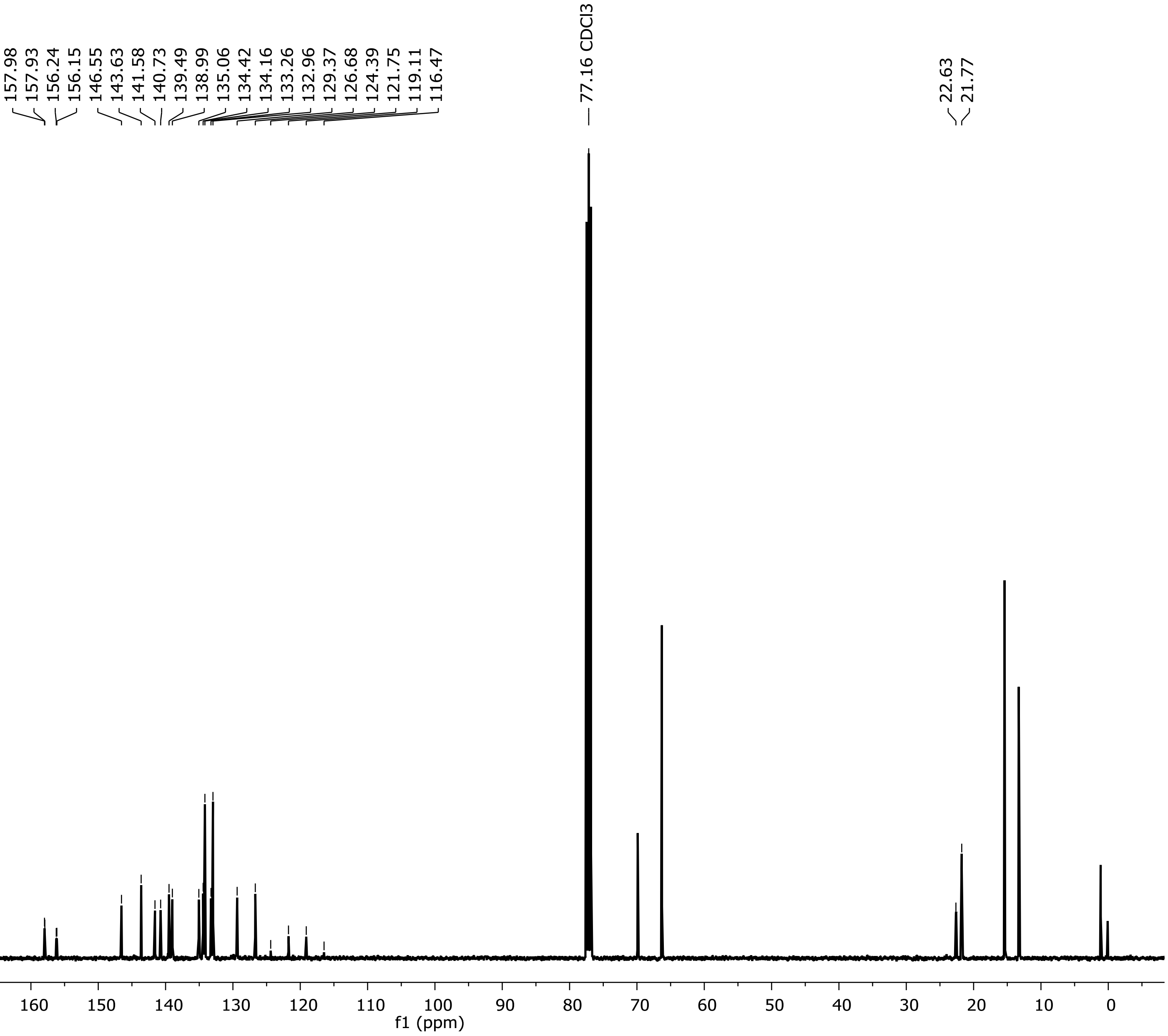

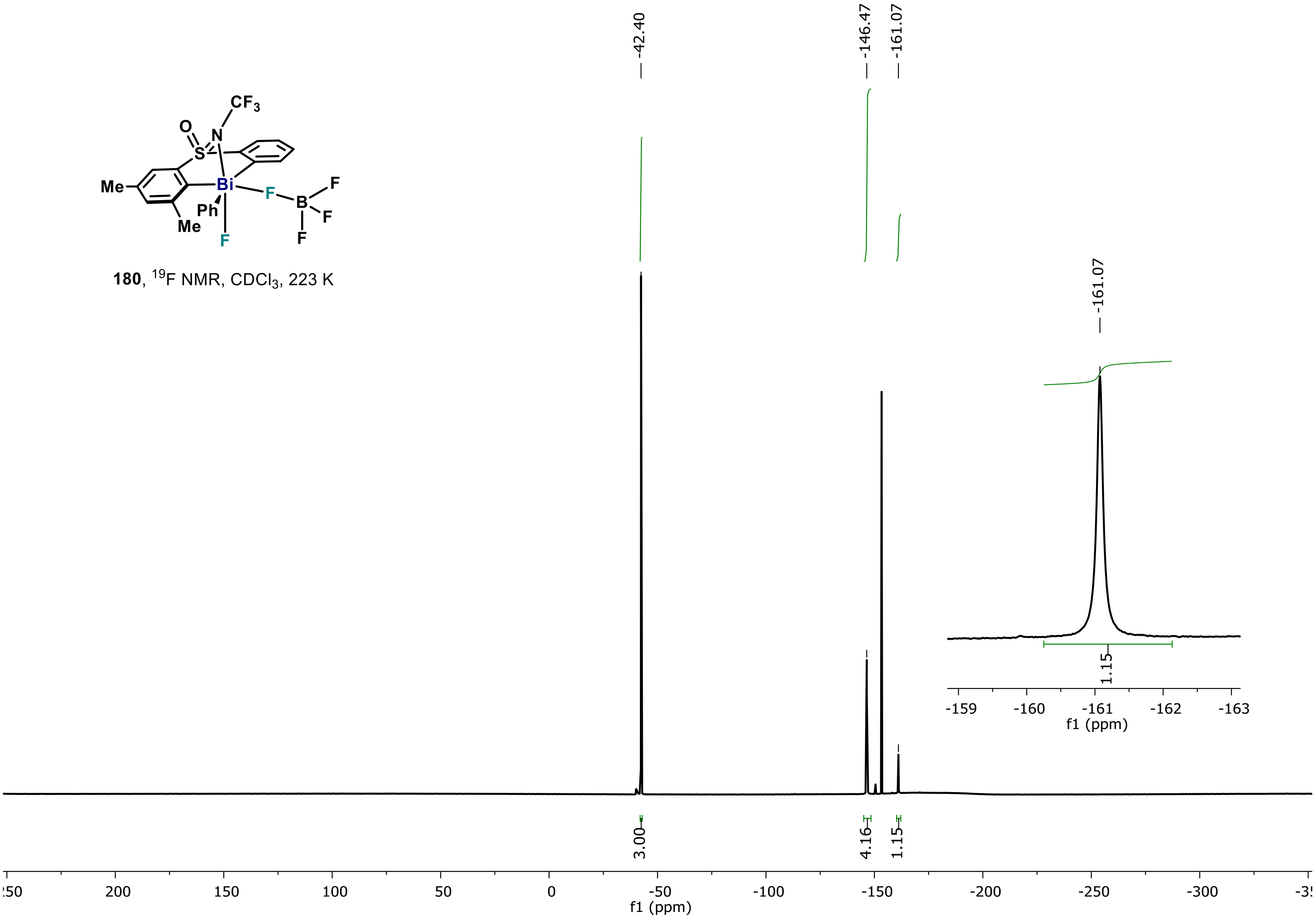

180, ^{19}F NMR, $CDCl_3$, 223 K

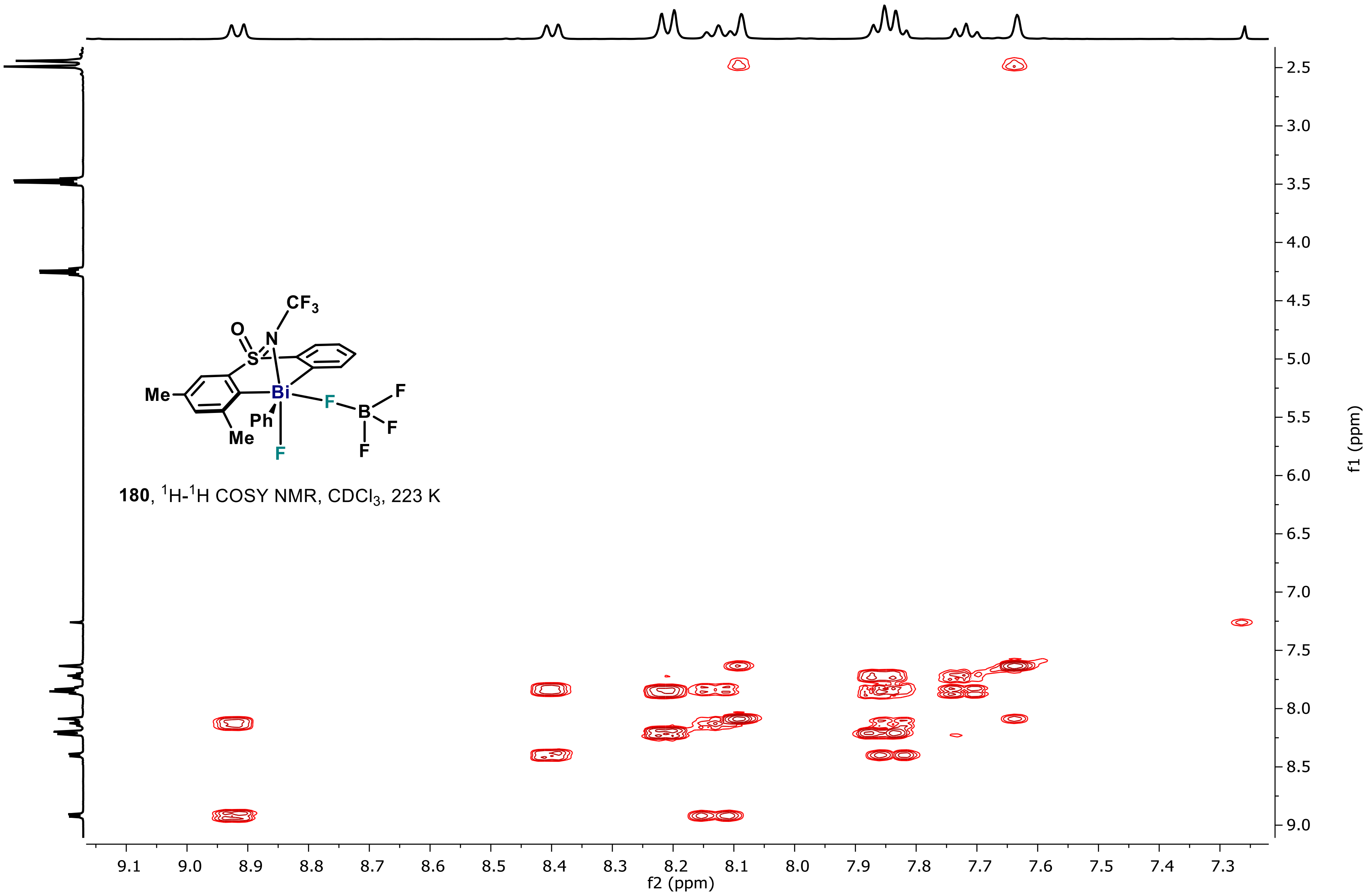

CF3
O
N
S
Me
Bi
F
B
F
F
Ph
Me
F
180, 1H-1H COSY NMR, CDCl3, 223 K
f2 (ppm)
f1 (ppm)

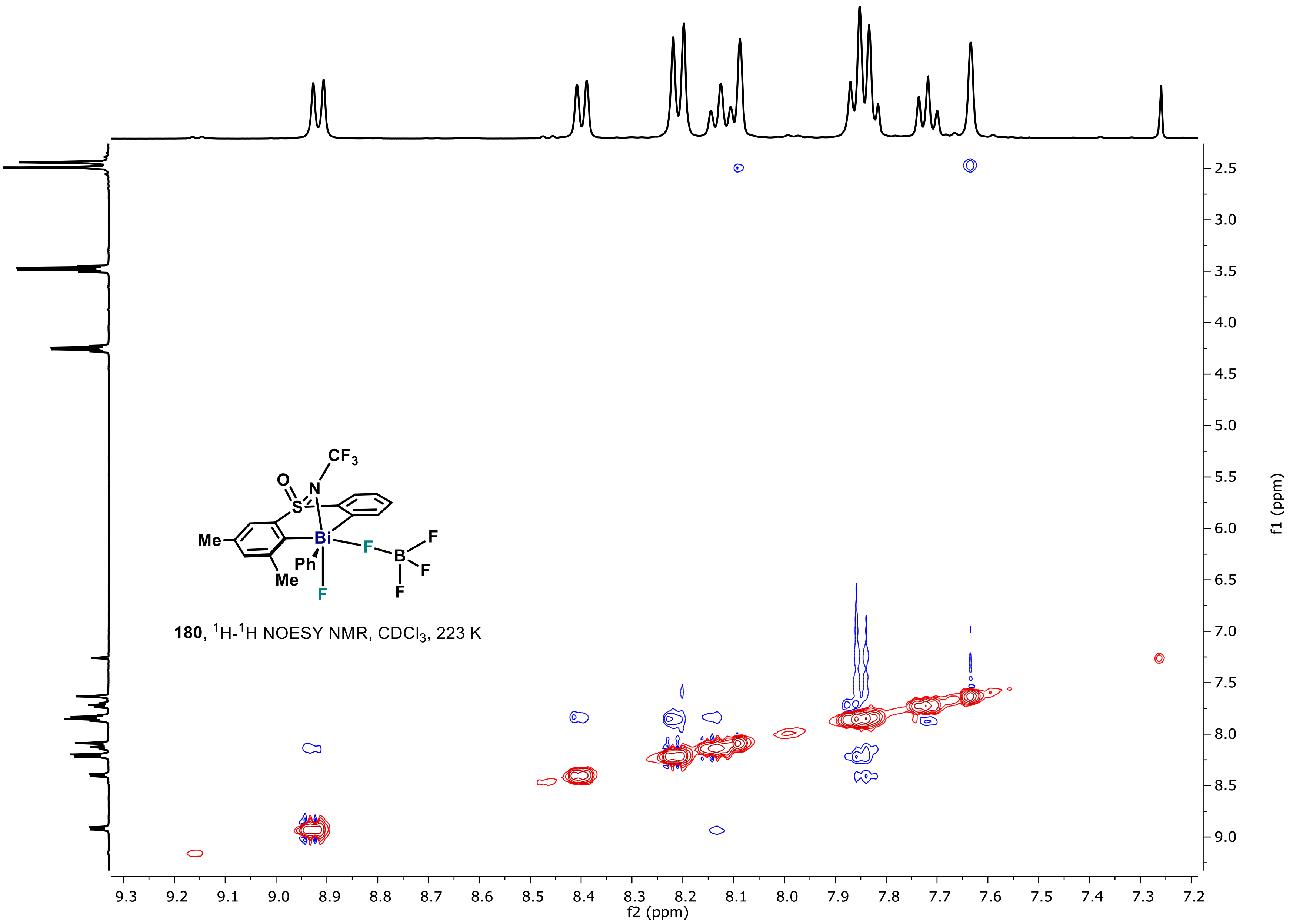

180, ^{1}H-^{1}H NOESY NMR, $CDCl_3$, 223 K

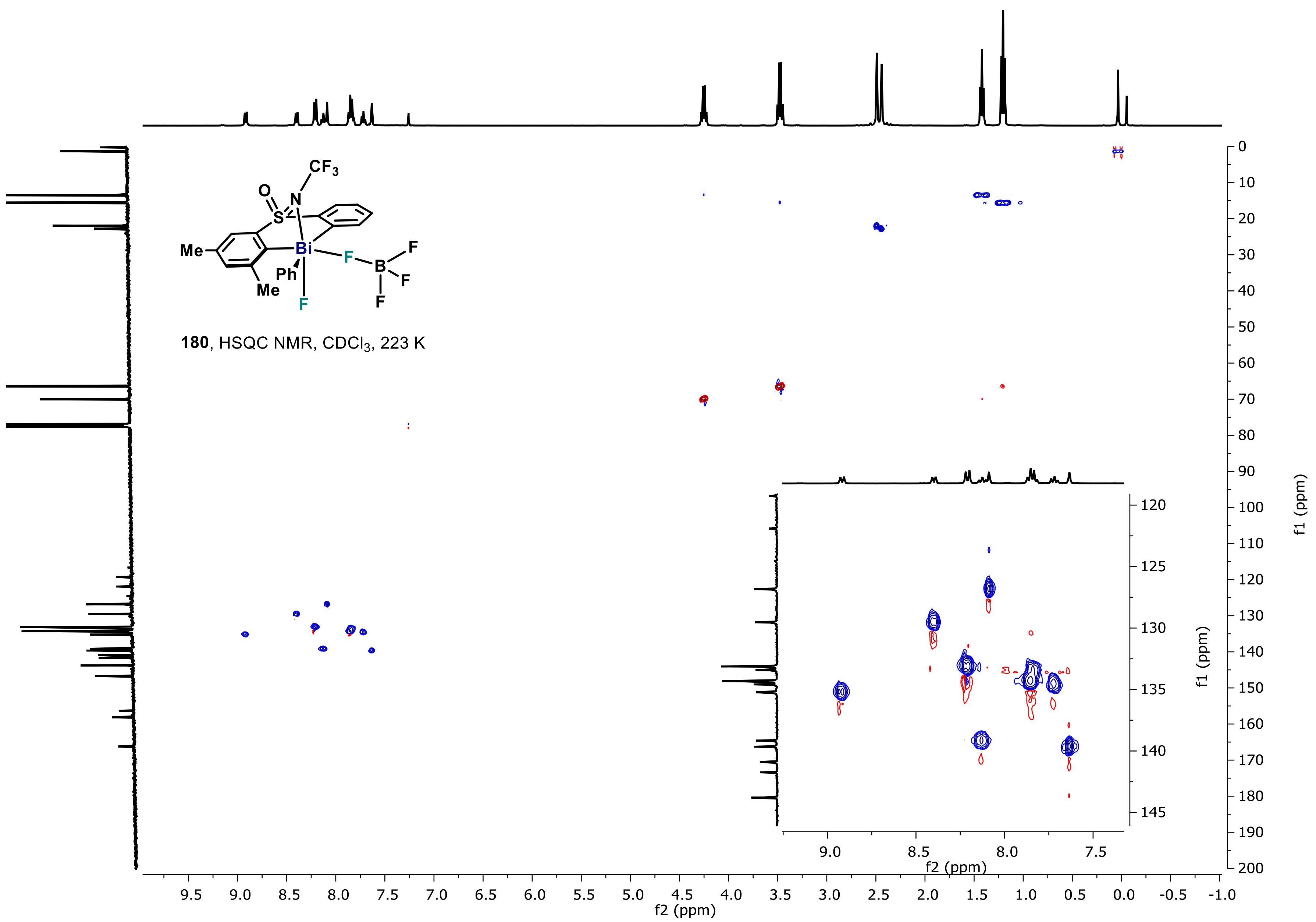

CF3
O
N
S
Me
Bi
F
B
F
Ph
F
Me
F
F
180, HSQC NMR, CDCl3, 223 K
f1 (ppm)
f2 (ppm)

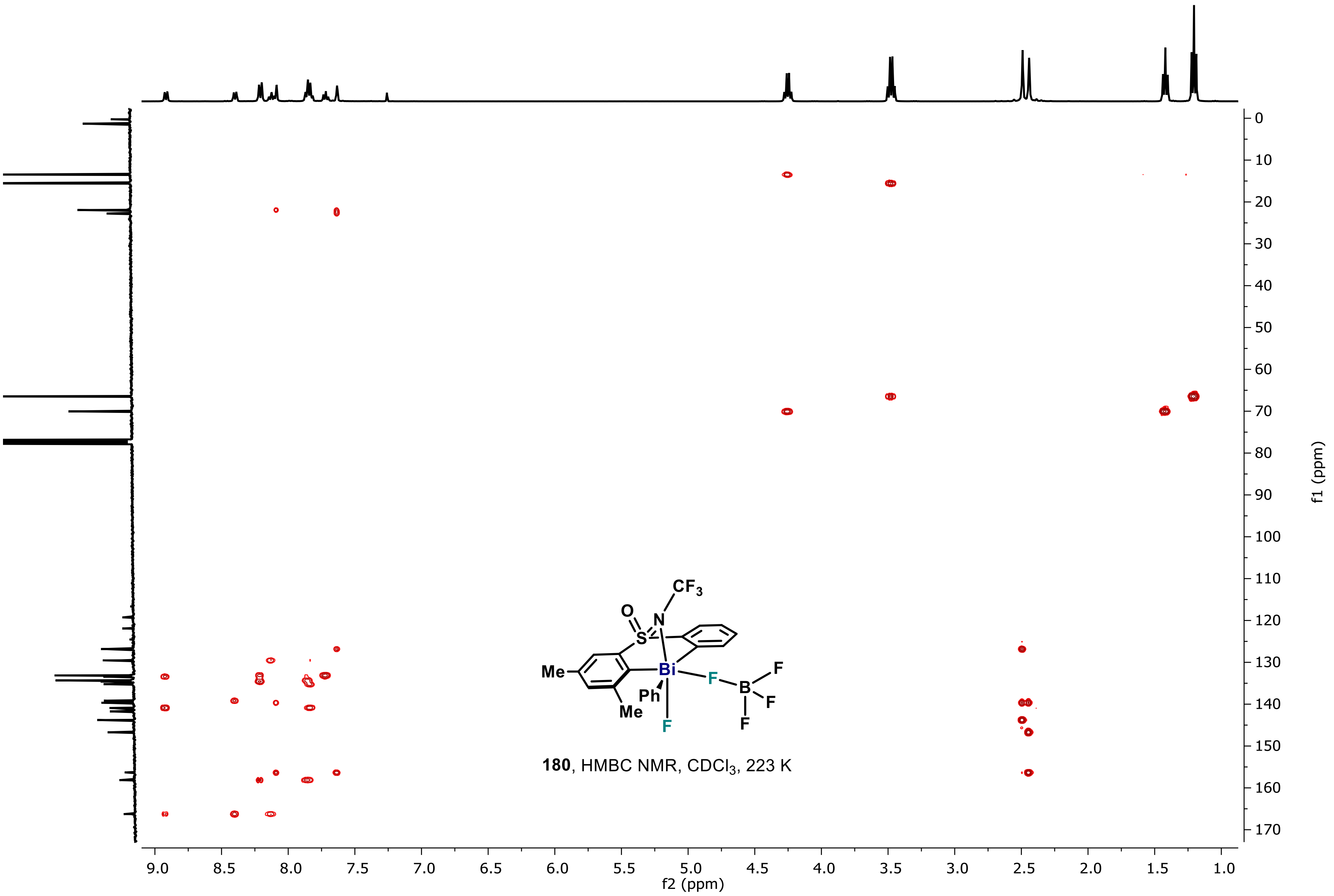

180, HMBC NMR, $CDCl_3$, 223 K

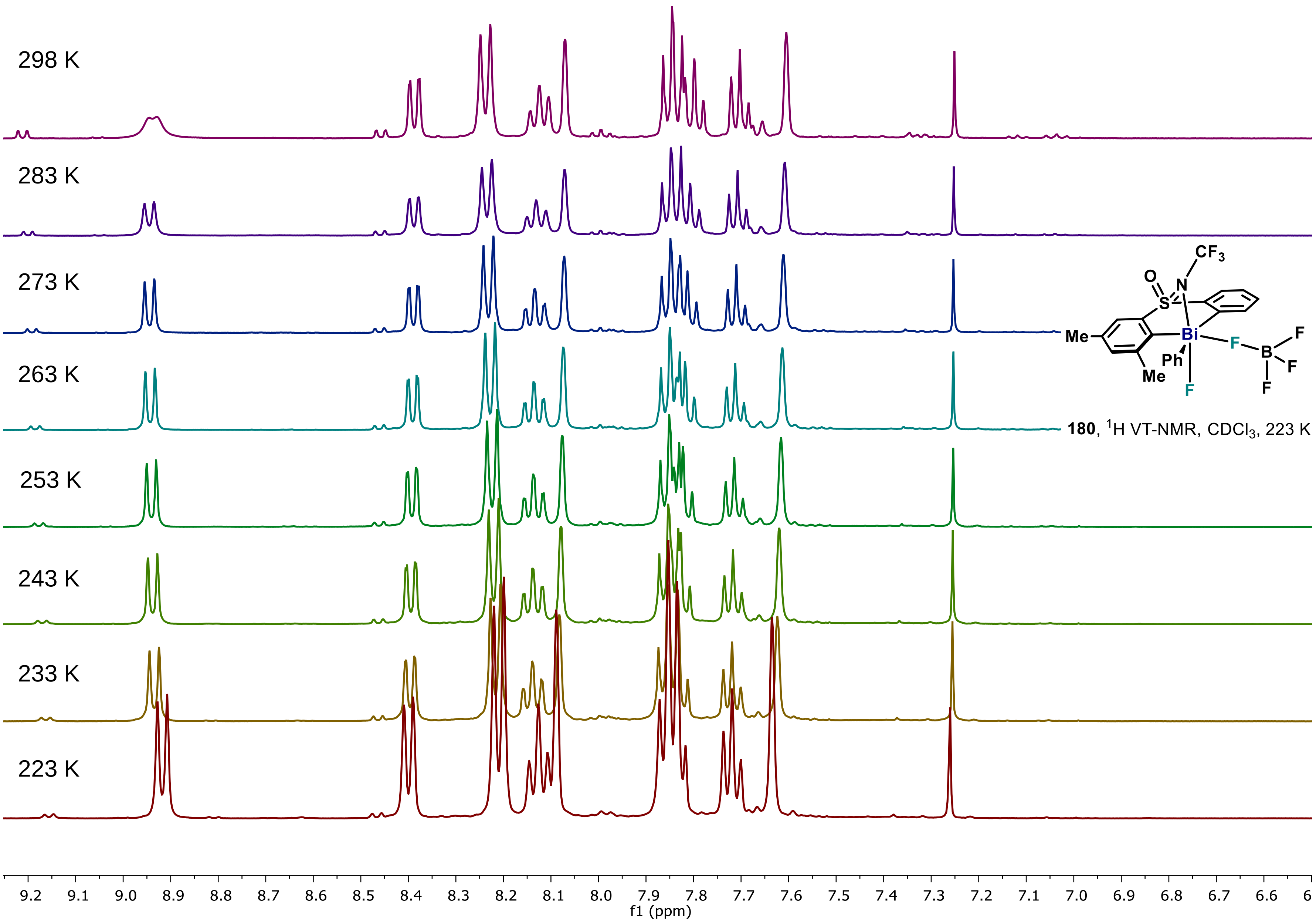
298 K
283 K
273 K
263 K
253 K
243 K
233 K
223 K
CF3
O
N
S
Me
Bi
F
B
F
F
Ph
Me
F
F
180, 1H VT-NMR, CDCl3, 223 K
9.2 9.1 9.0 8.9 8.8 8.7 8.6 8.5 8.4 8.3 8.2 8.1 8.0 7.9 7.8 7.7 7.6 7.5 7.4 7.3 7.2 7.1 7.0 6.9 6.8 6.7 6.6 6
f1 (ppm)

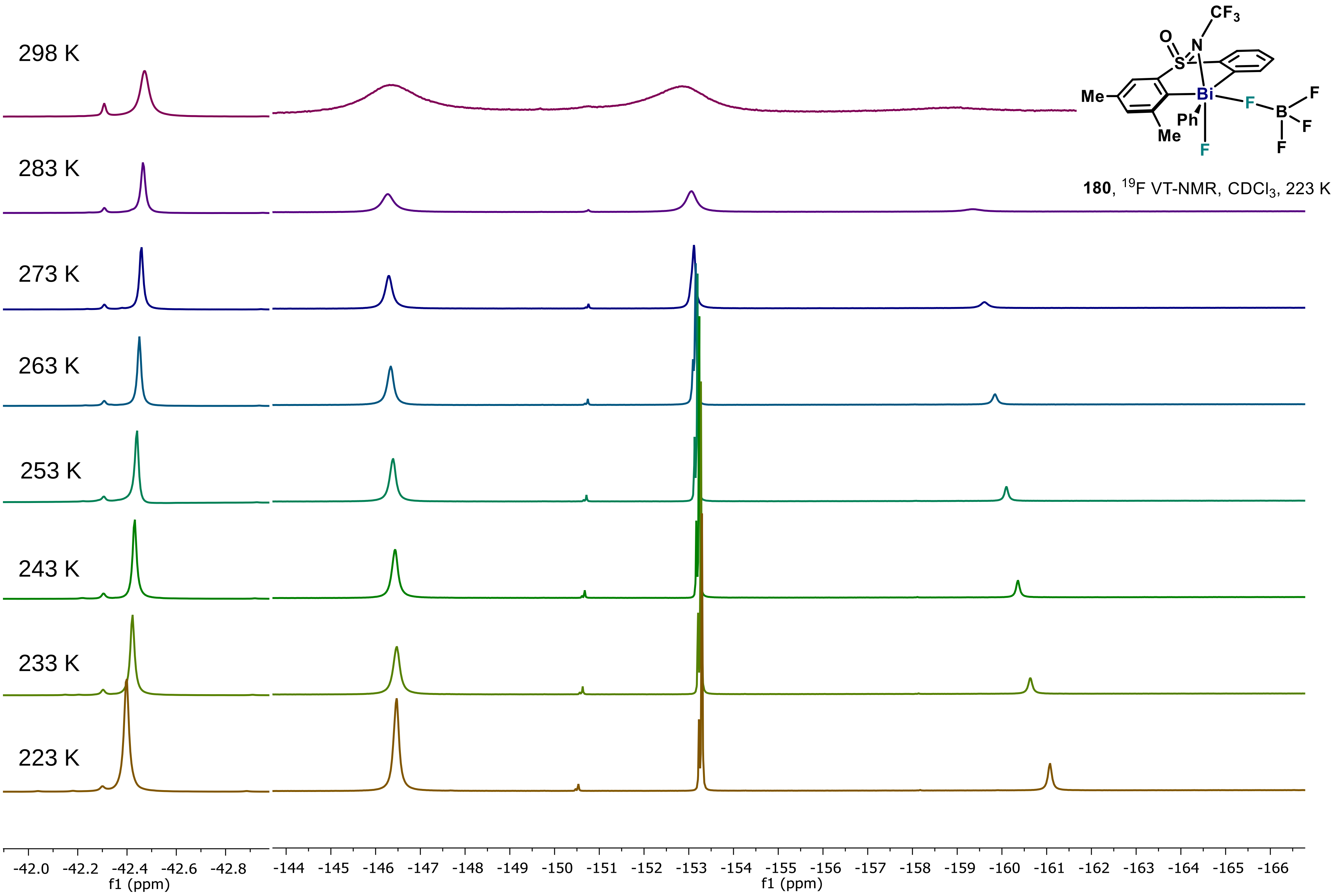
298 K
283 K
273 K
263 K
253 K
243 K
233 K
223 K
CF3
O
N
S
Me
Bi
F
B
F
F
F
Ph
Me
F
180, 19F VT-NMR, CDCl3, 223 K
-42.0 -42.2 -42.4 -42.6 -42.8
f1 (ppm)
-144 -145 -146 -147 -148 -149 -150 -151 -152 -153 -154 -155 -156 -157 -158 -159 -160 -161 -162 -163 -164 -165 -166
f1 (ppm)

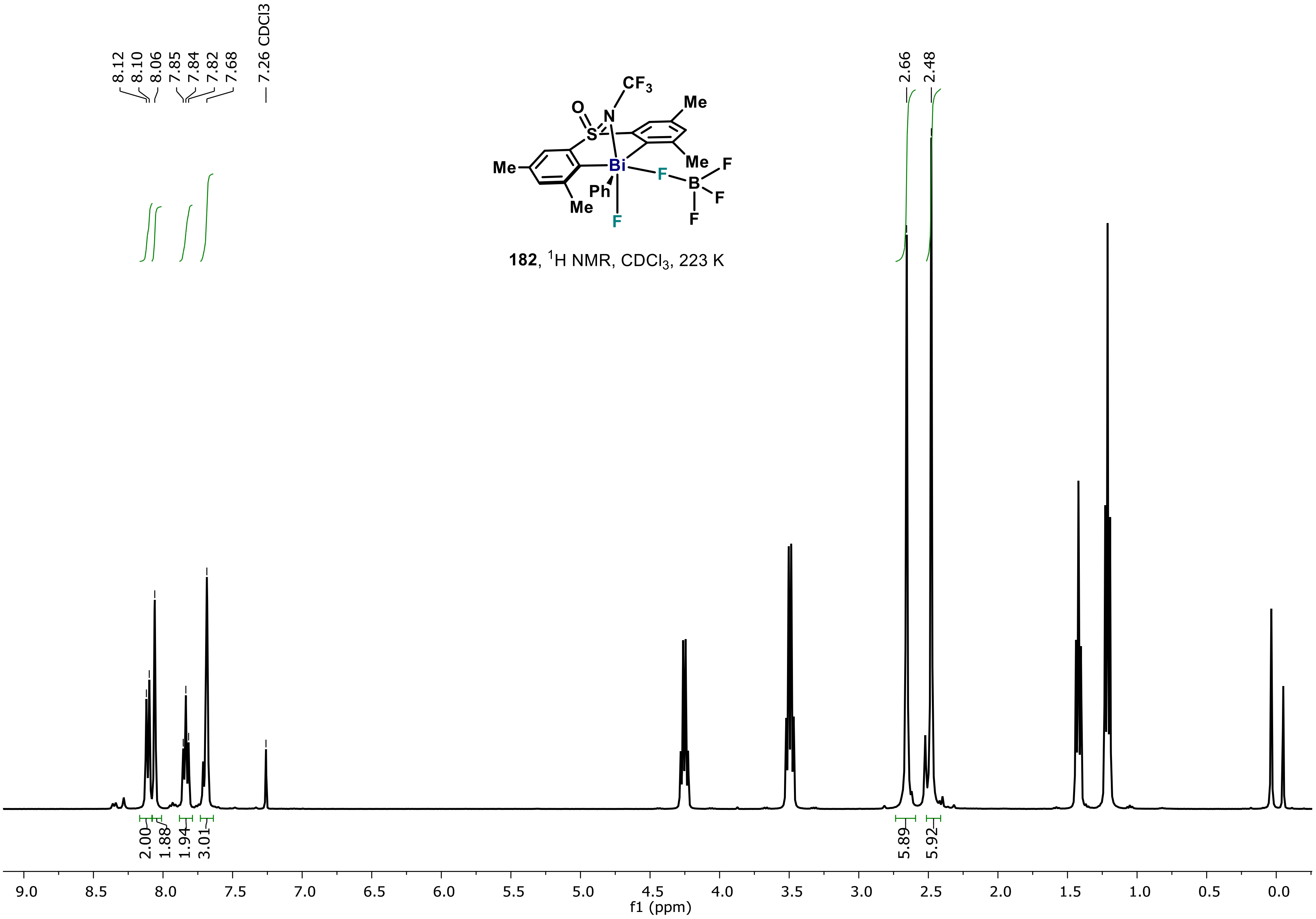

182, ^{1}H NMR, $CDCl_3$, 223 K

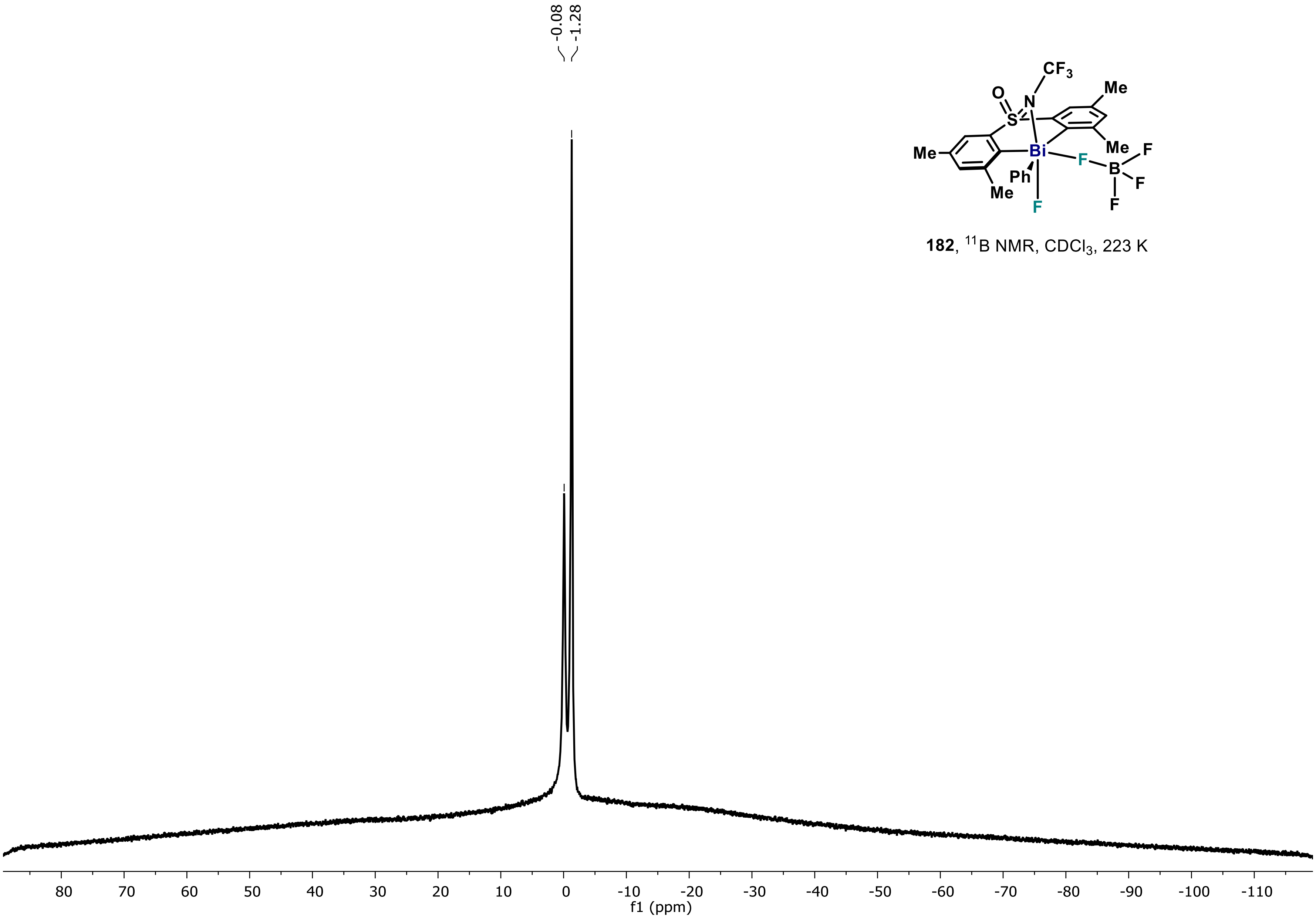

182, ^{11}B NMR, $CDCl_3$, 223 K

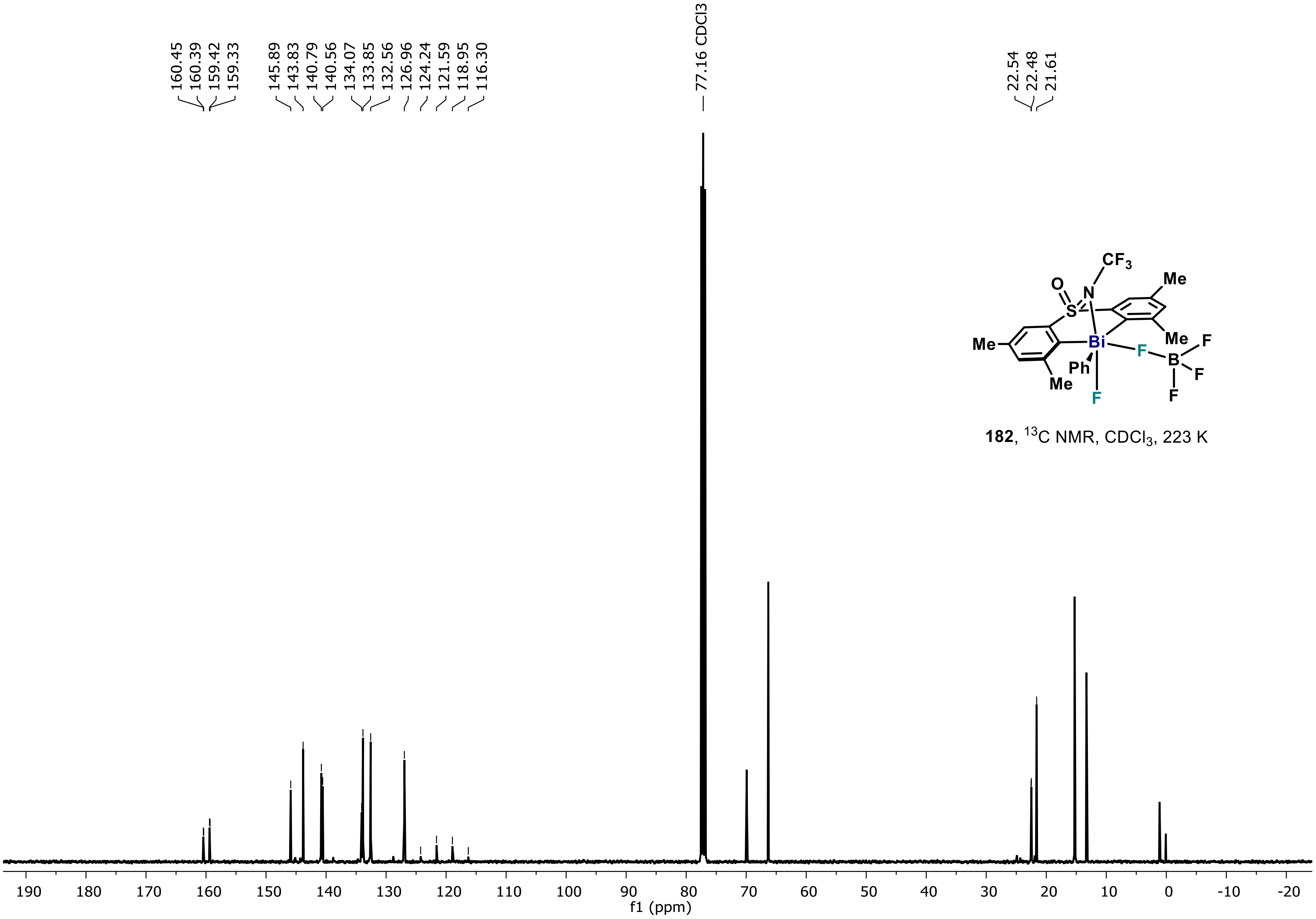

182, ^{13}C NMR, $CDCl_3$, 223 K

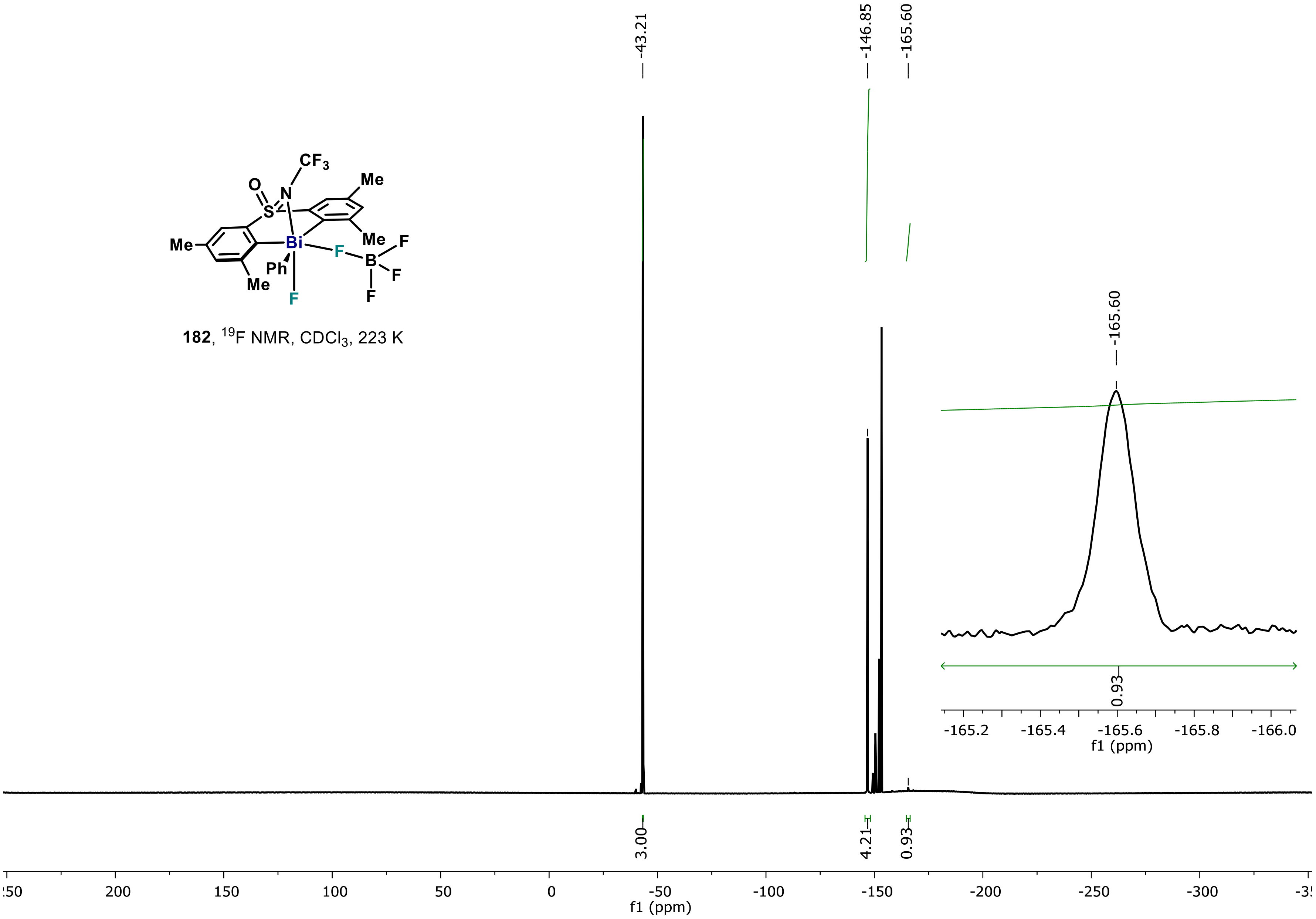

182, ^{19}F NMR, $CDCl_3$, 223 K

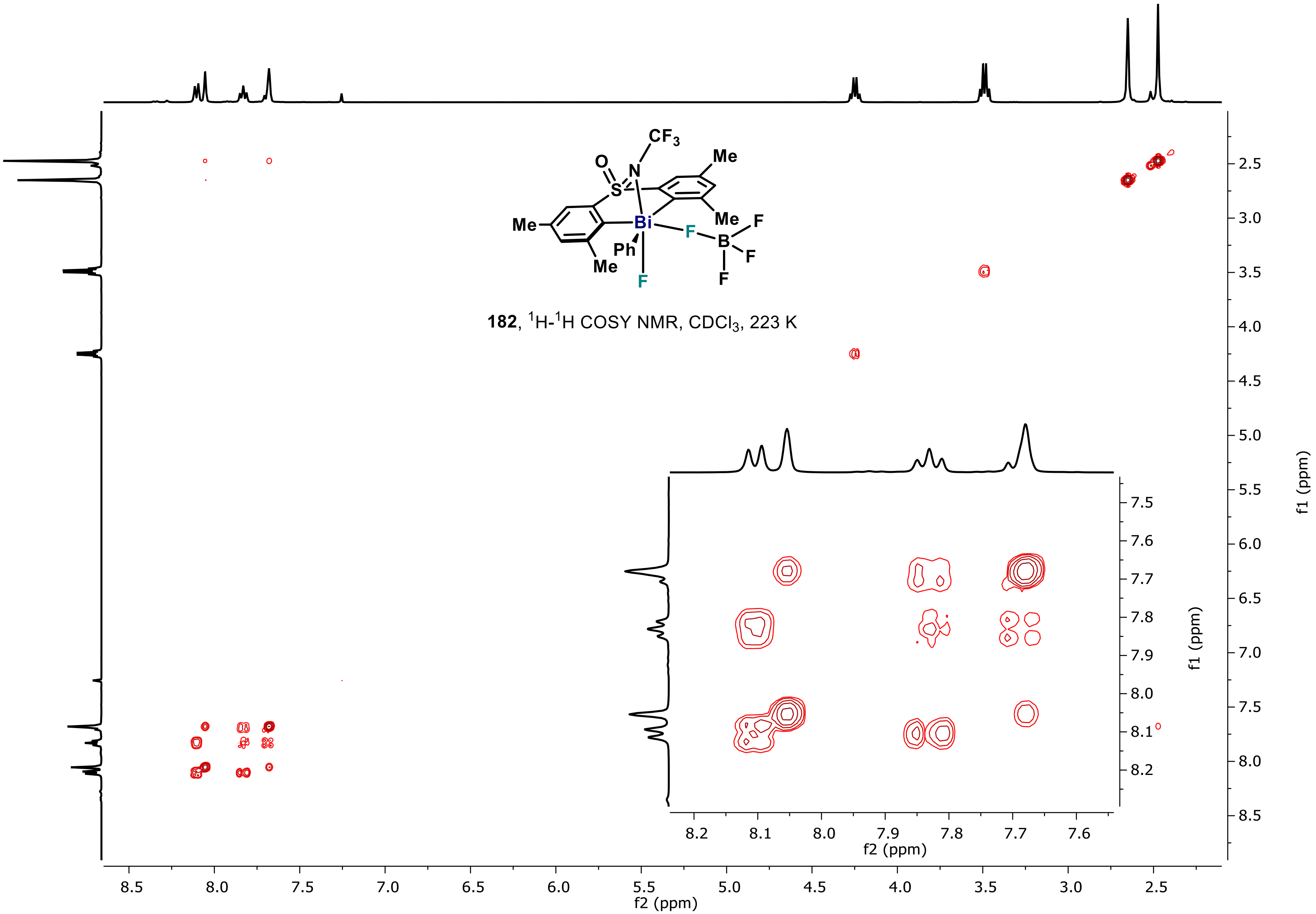
CF3
O
N
S
Me
Me
Bi
F
B
F
F
Ph
Me
F
F
Me
182, 1H-1H COSY NMR, CDCl3, 223 K
f2 (ppm)
f1 (ppm)

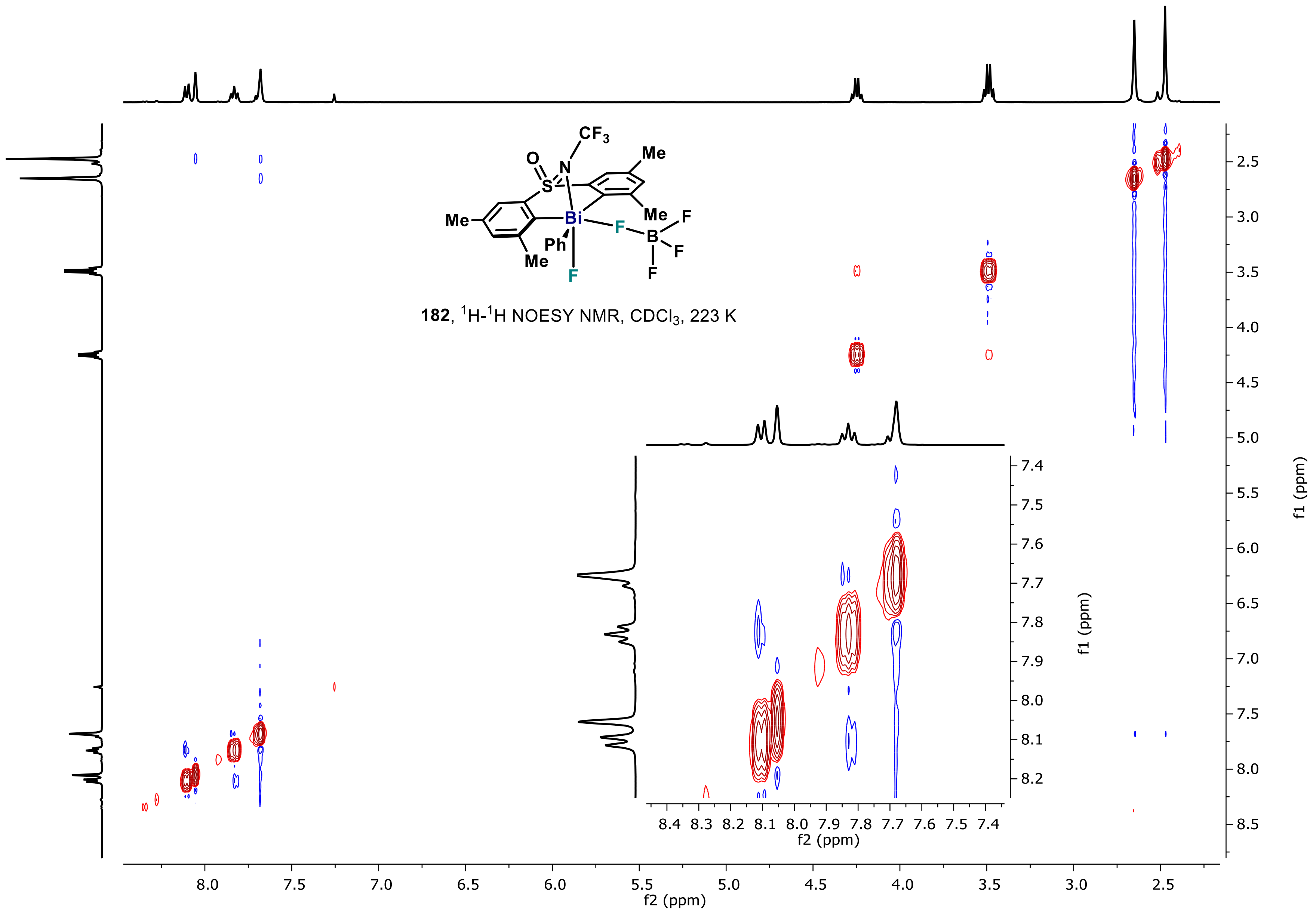
CF3
O
N
S
Me
Me
Bi
F
Me
F
B
F
Ph
Me
F
F
182, 1H-1H NOESY NMR, CDCl3, 223 K
f1 (ppm)
f2 (ppm)

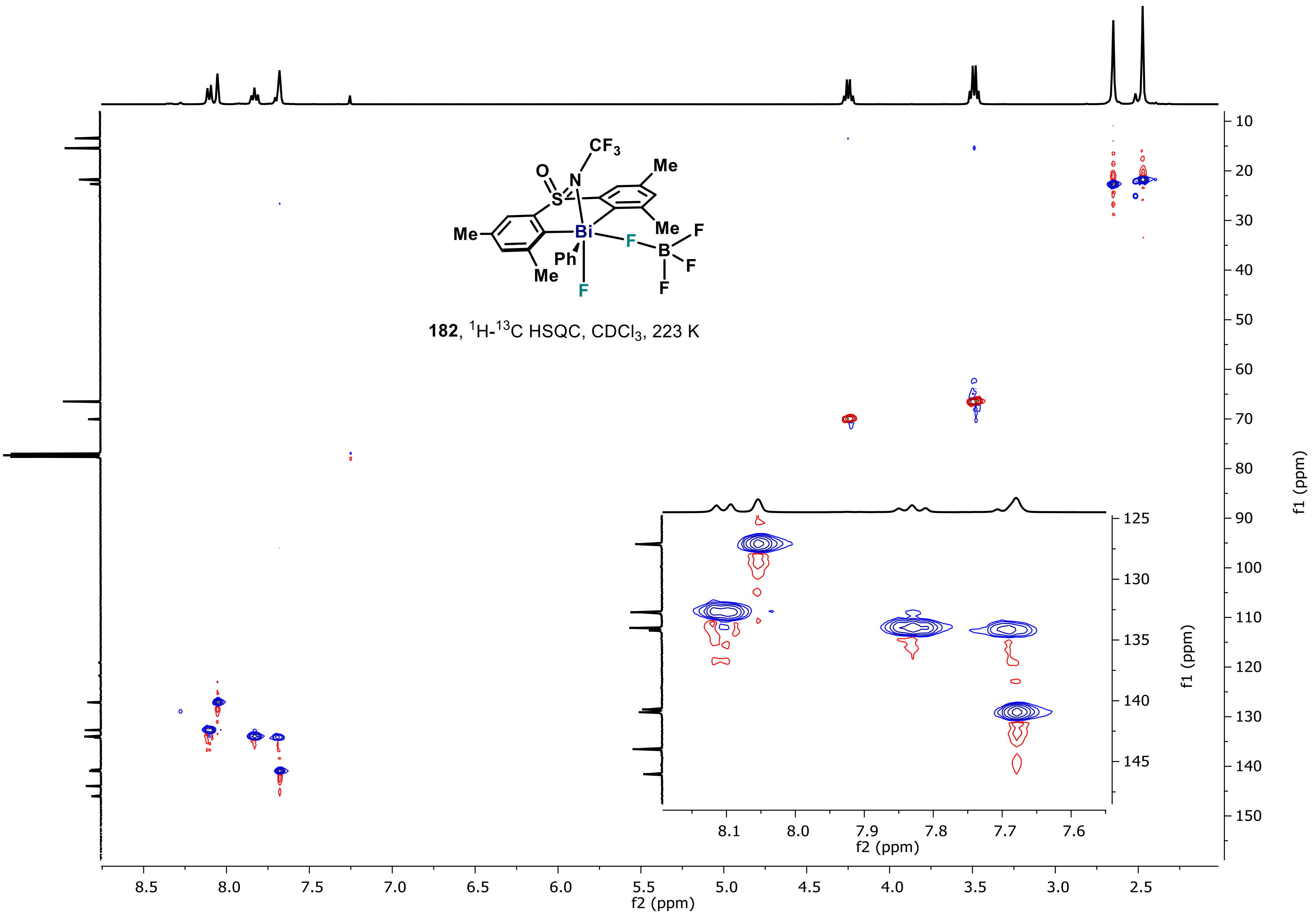

CF3
O
S
N
Me
Me
Bi
F
B
F
F
F
Ph
Me
Me
F
182, 1H-13C HSQC, CDCl3, 223 K
f2 (ppm)
f1 (ppm)

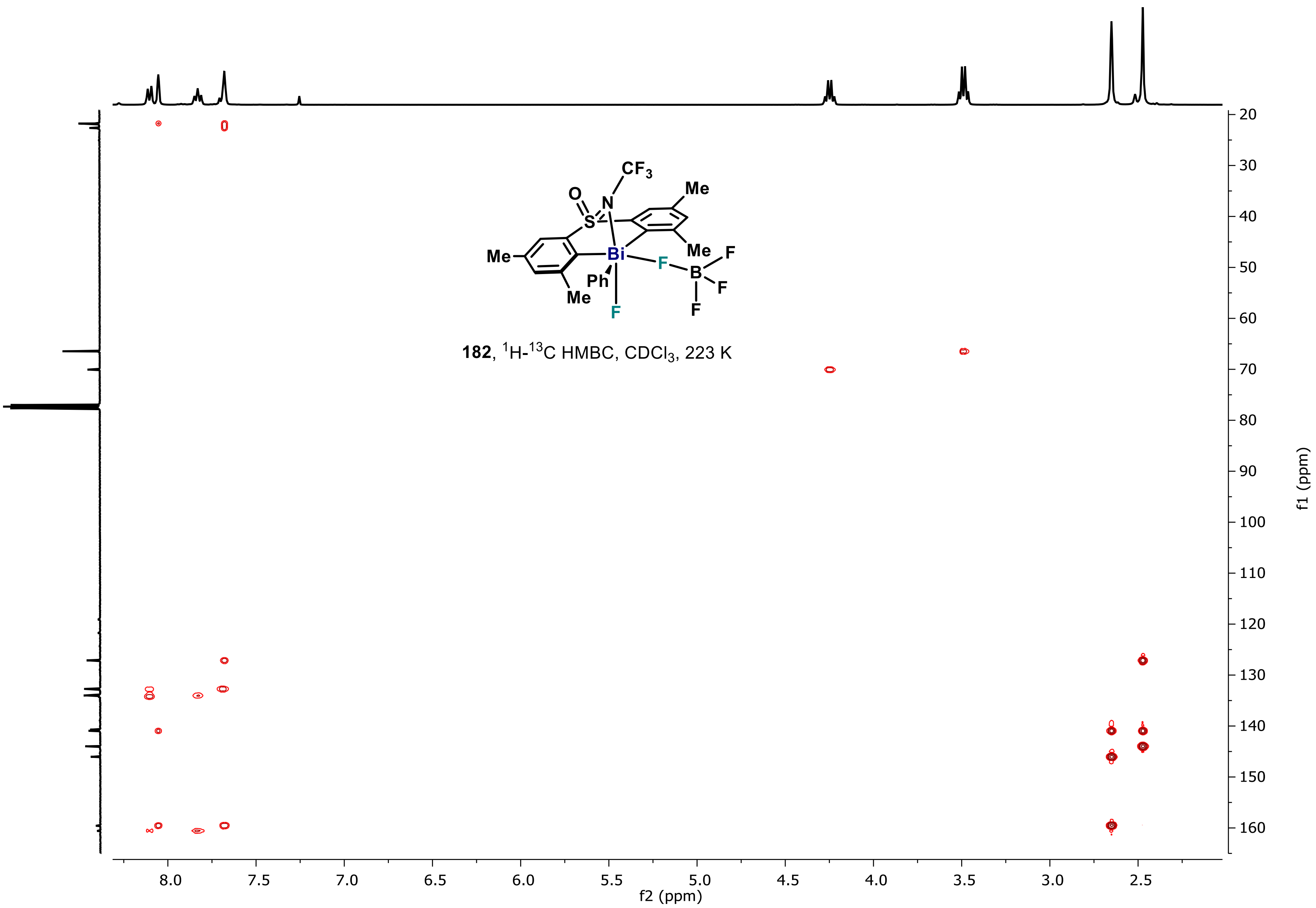
CF3
Me
O
N
S
Me
Bi
F
B
F
F
Ph
Me
F
F
182, 1H-13C HMBC, CDCl3, 223 K
f1 (ppm)
f2 (ppm)

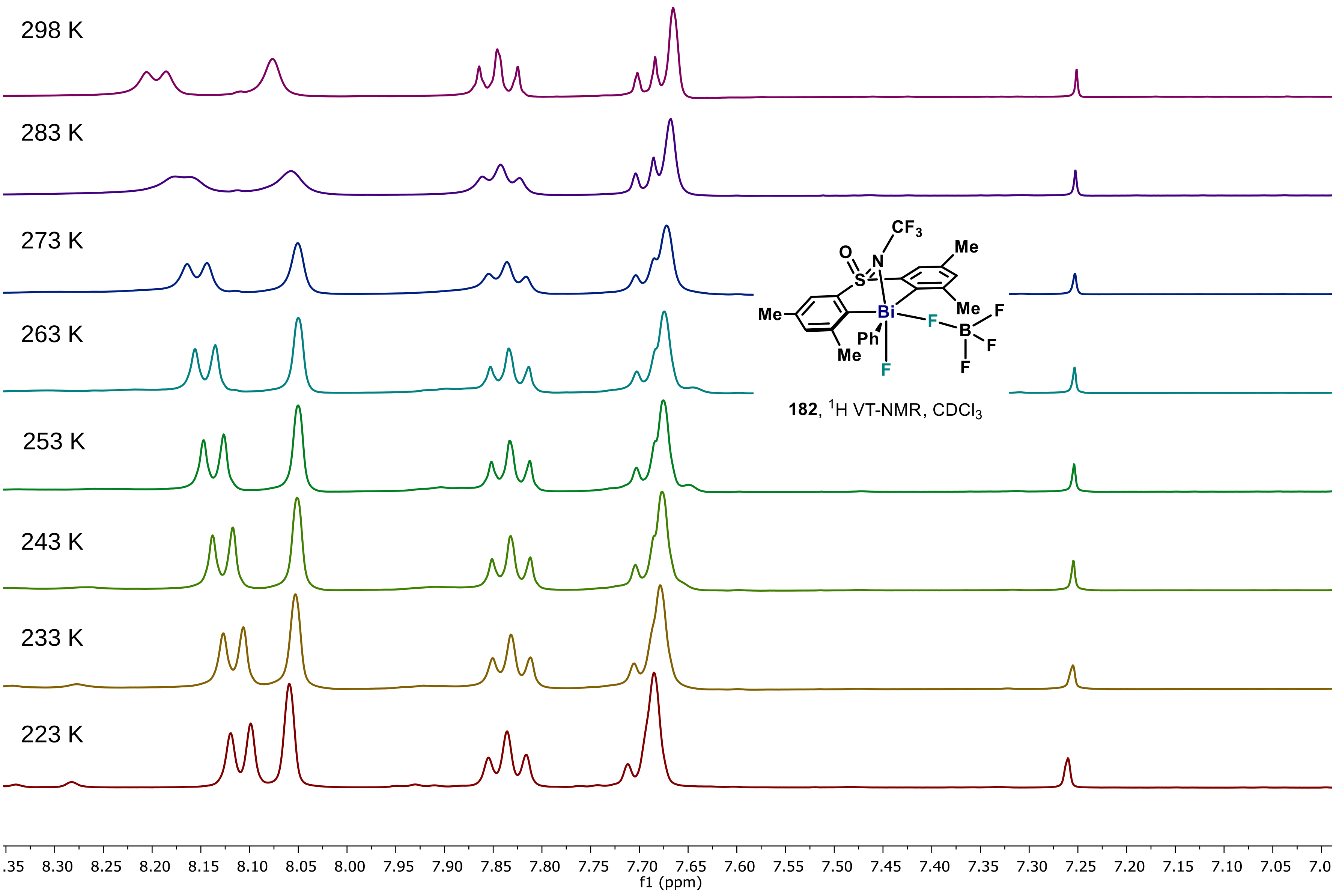
298 K
283 K
273 K
263 K
253 K
243 K
233 K
223 K
182, 1H VT-NMR, CDCl3
f1 (ppm)

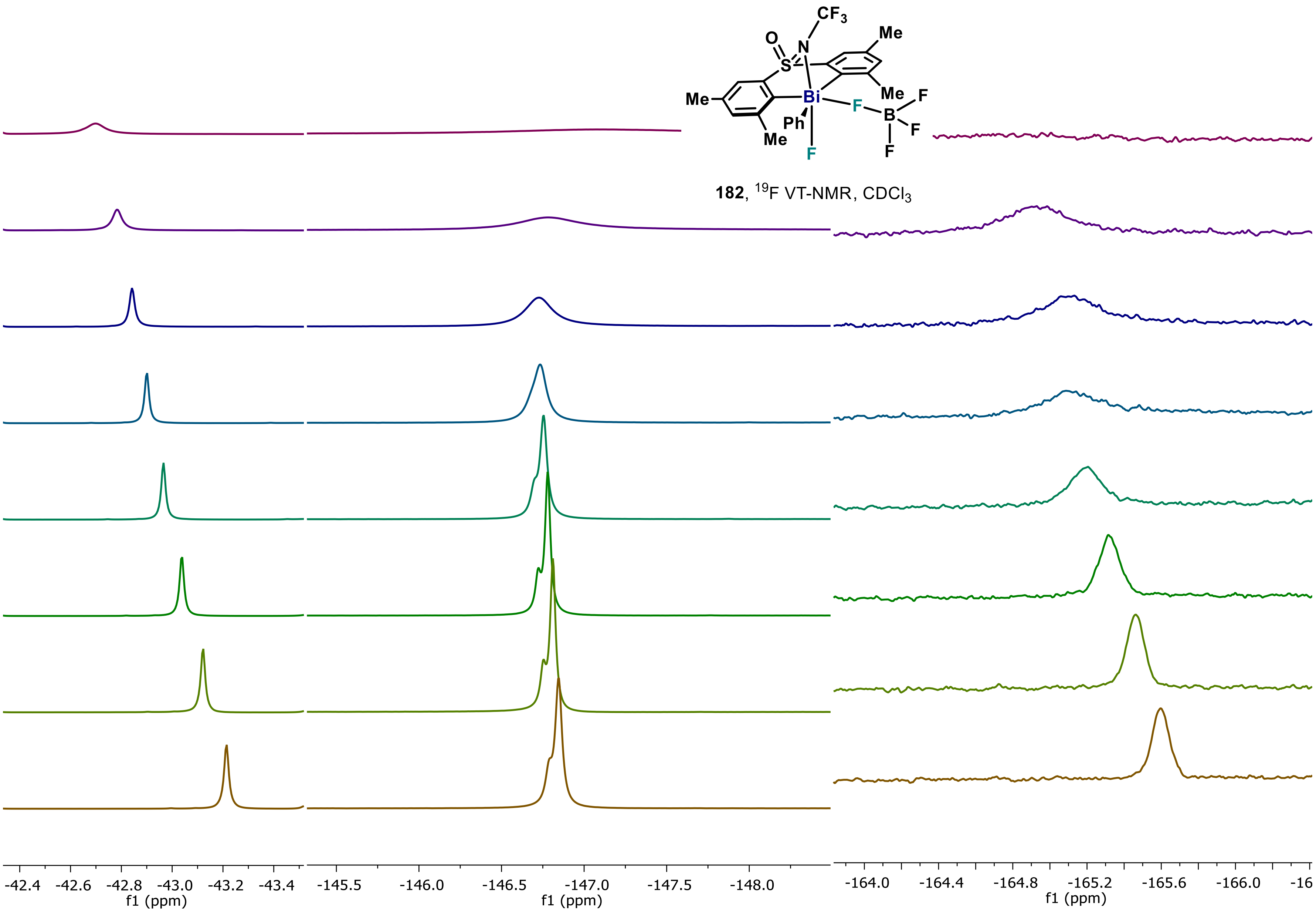
CF3
O
N
Me
S
Me
Bi
F
Me
Ph
F
B
F
Me
F
F
182, 19F VT-NMR, CDCl3
-42.4 -42.6 -42.8 -43.0 -43.2 -43.4
f1 (ppm)
-145.5 -146.0 -146.5 -147.0 -147.5 -148.0
f1 (ppm)
-164.0 -164.4 -164.8 -165.2 -165.6 -166.0 -16
f1 (ppm)

12. Crude ^{19}F NMR spectra for stoichiometric fluorination of Bi(III) compounds

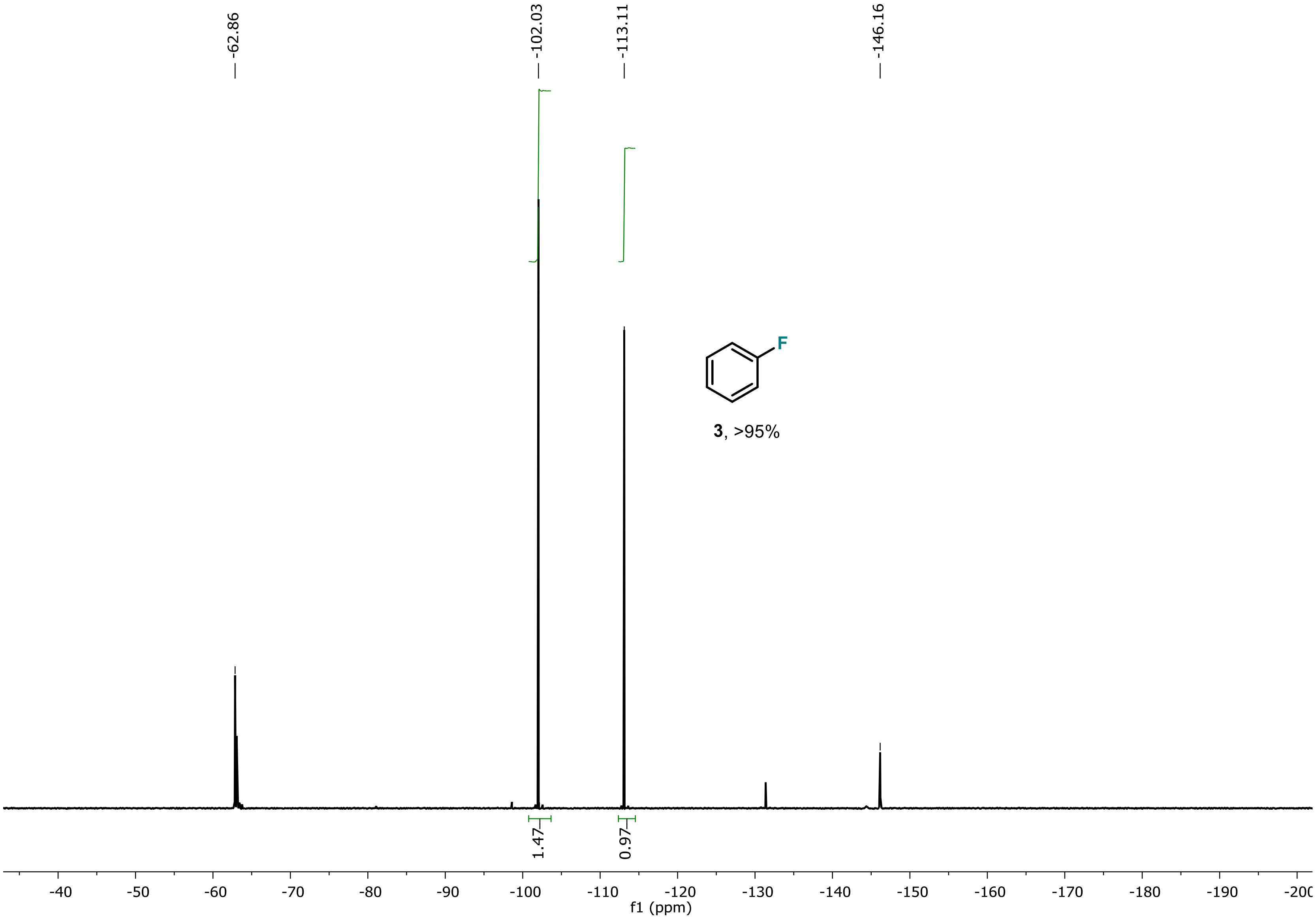

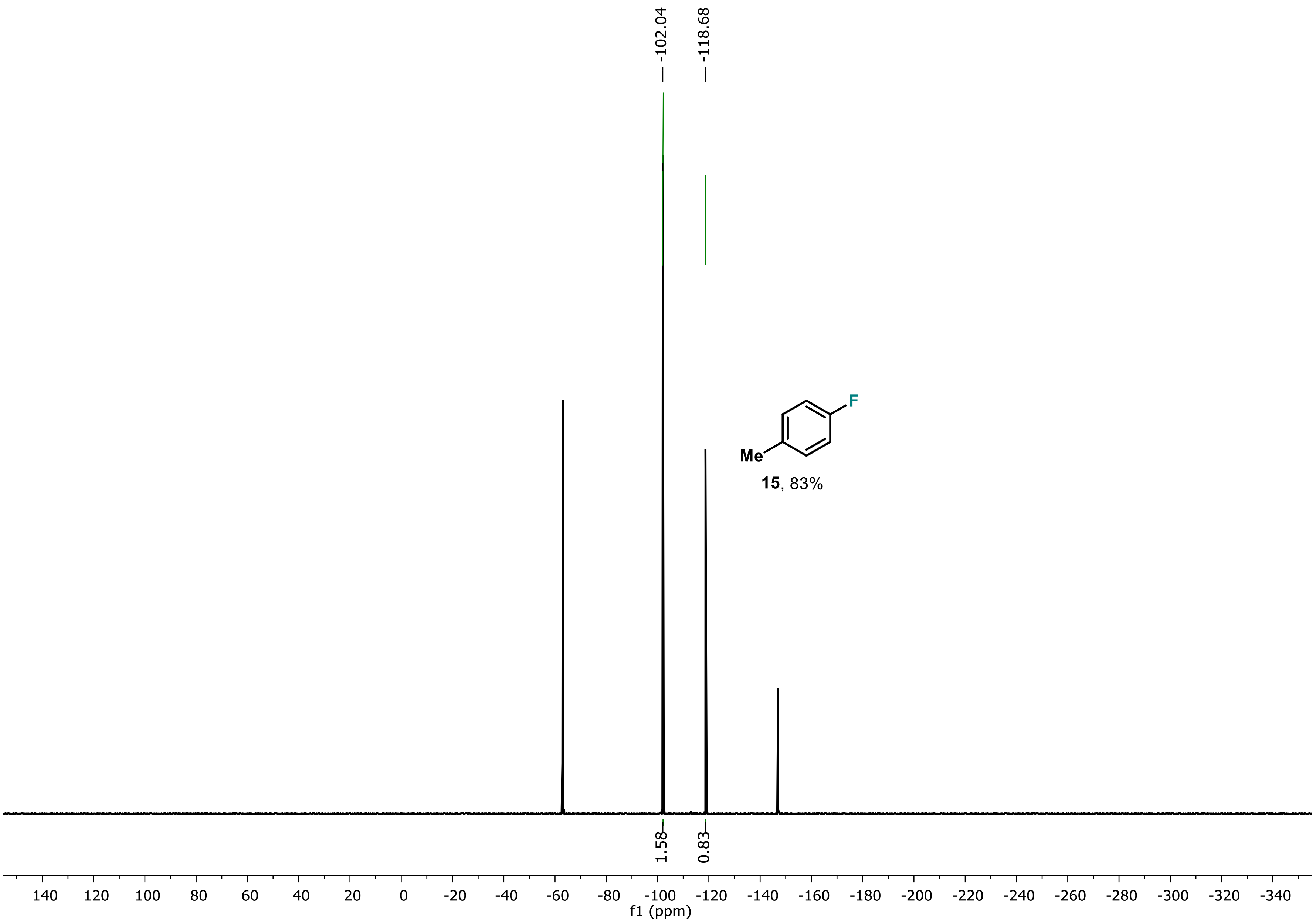
-102.04
-118.68
F
Me
15, 83%
1.58
0.83
140 120 100 80 60 40 20 0 -20 -40 -60 -80 -100 -120 -140 -160 -180 -200 -220 -240 -260 -280 -300 -320 -340
f1 (ppm)

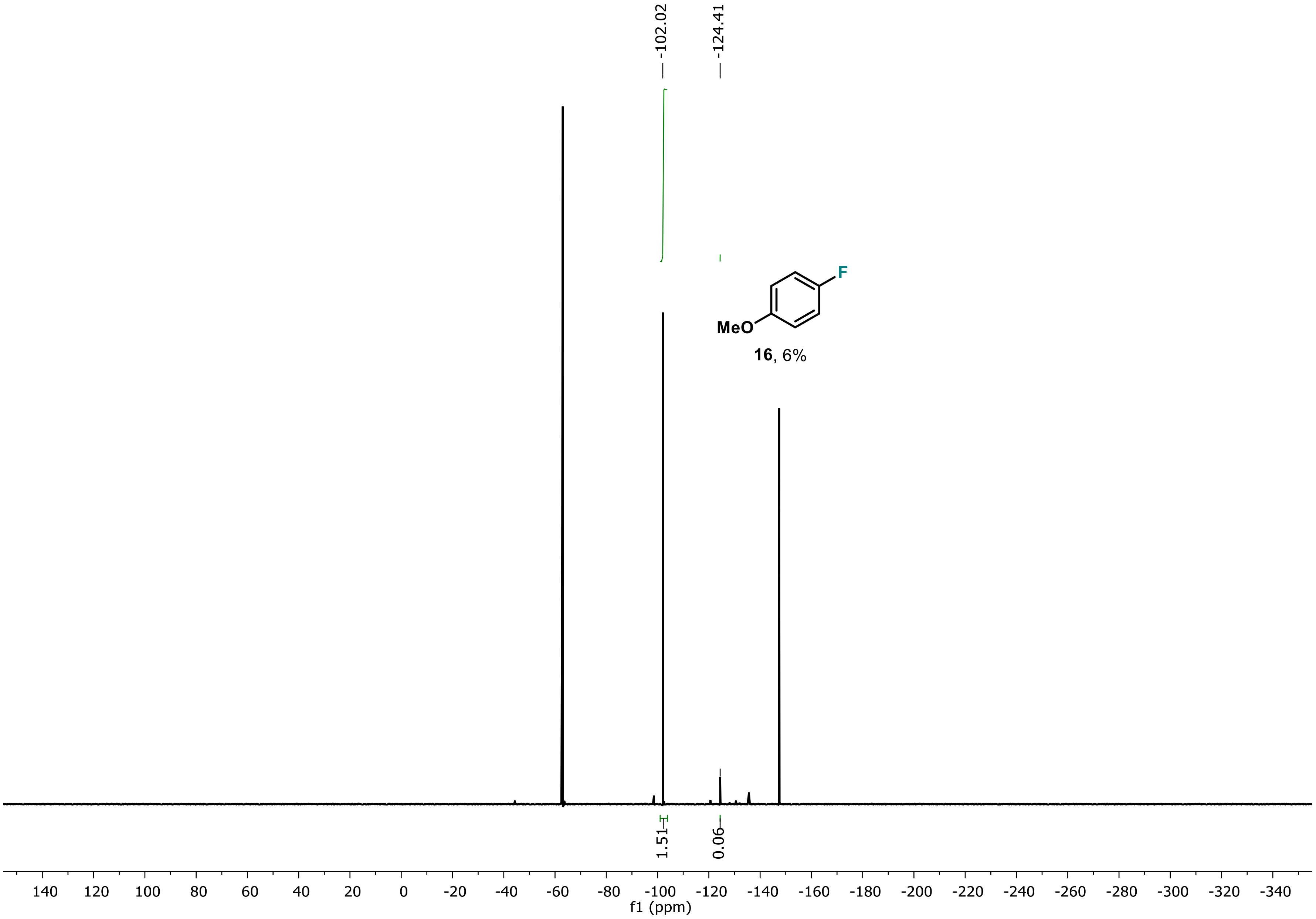
-102.02
-124.41
MeO
F
16, 6%
1.51
0.06
140 120 100 80 60 40 20 0 -20 -40 -60 -80 -100 -120 -140 -160 -180 -200 -220 -240 -260 -280 -300 -320 -340
f1 (ppm)

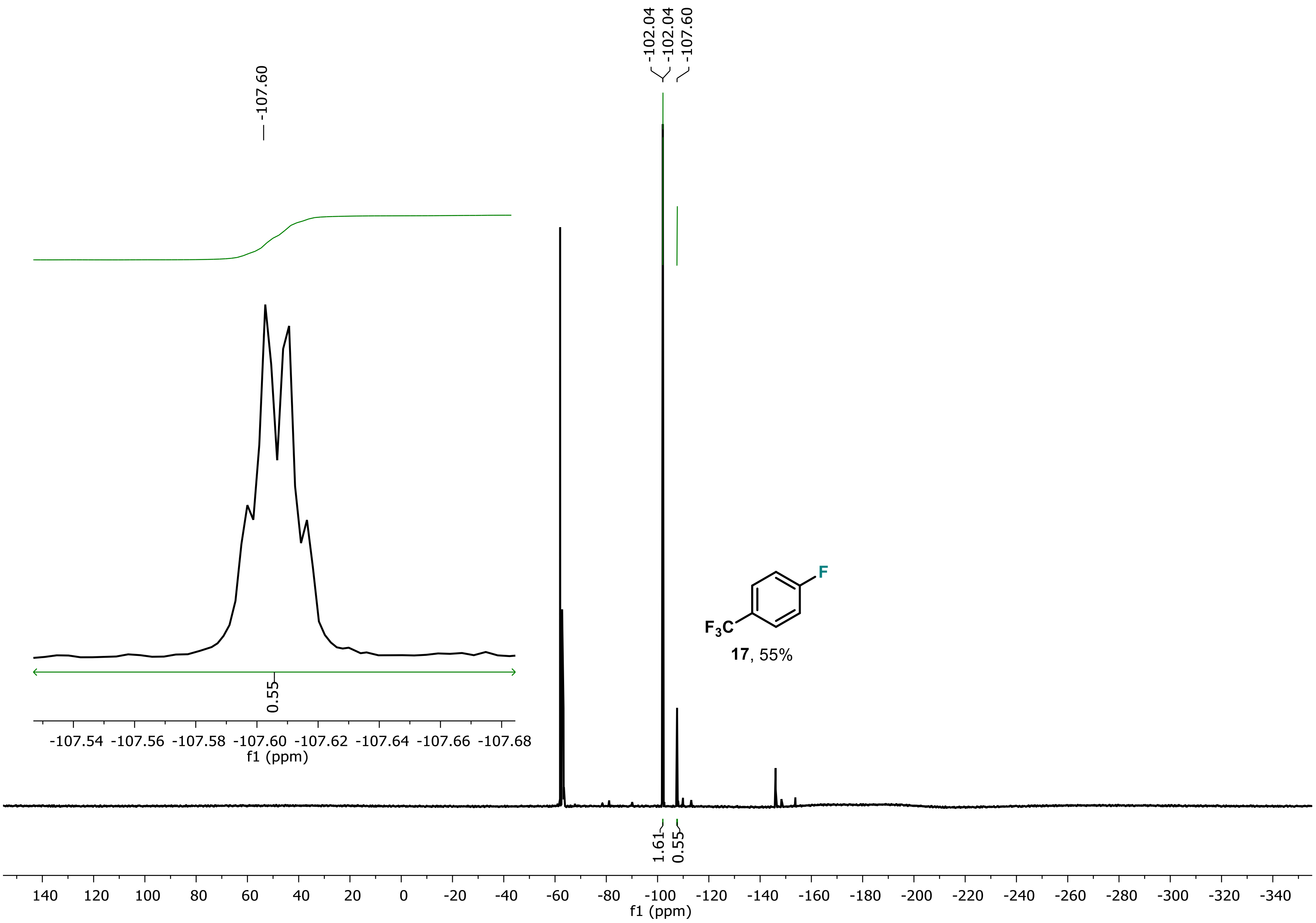

-102.04
-102.04
-107.60
-107.60
0.55
-107.54 -107.56 -107.58 -107.60 -107.62 -107.64 -107.66 -107.68
f1 (ppm)
F
F3C
17, 55%
1.61
0.55
140 120 100 80 60 40 20 0 -20 -40 -60 -80 -100 -120 -140 -160 -180 -200 -220 -240 -260 -280 -300 -320 -340
f1 (ppm)

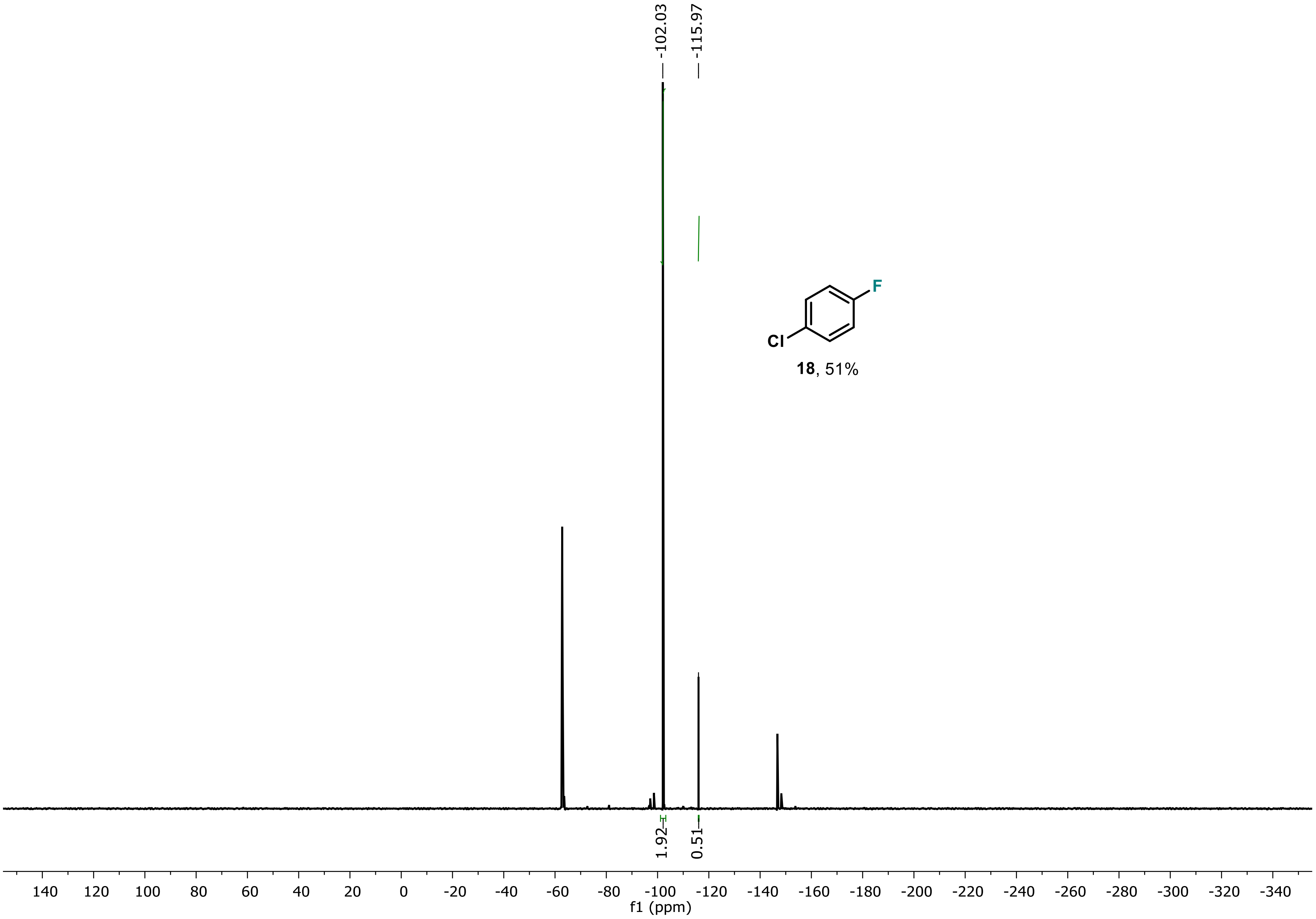

-102.03
-115.97
F
Cl
18, 51%
1.92
0.51
140 120 100 80 60 40 20 0 -20 -40 -60 -80 -100 -120 -140 -160 -180 -200 -220 -240 -260 -280 -300 -320 -340
f1 (ppm)

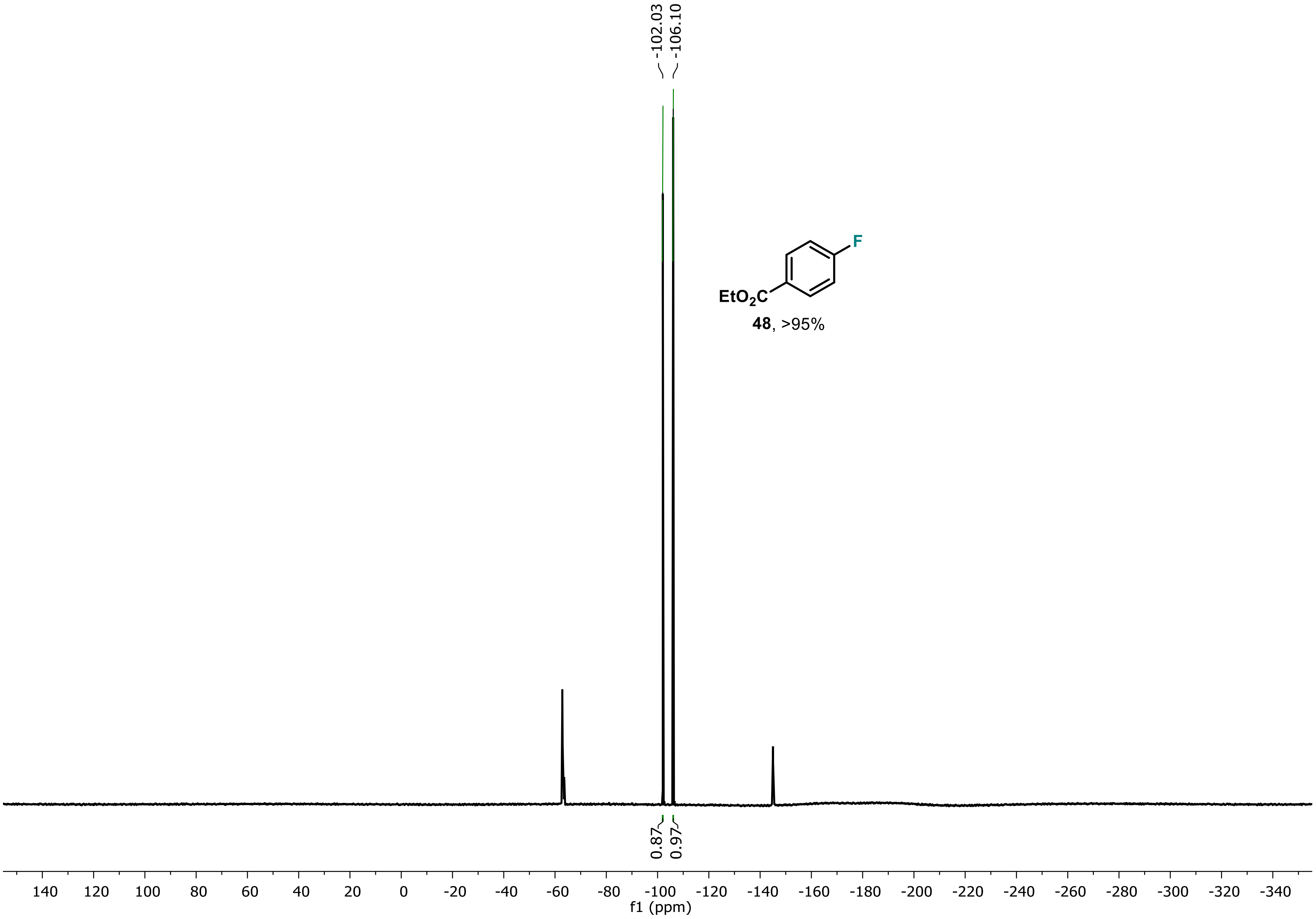

-102.03
-106.10
F
EtO2C
48, >95%
0.87
0.97
140
120
100
80
60
40
20
0
-20
-40
-60
-80
-100
-120
-140
-160
-180
-200
-220
-240
-260
-280
-300
-320
-340
f1 (ppm)

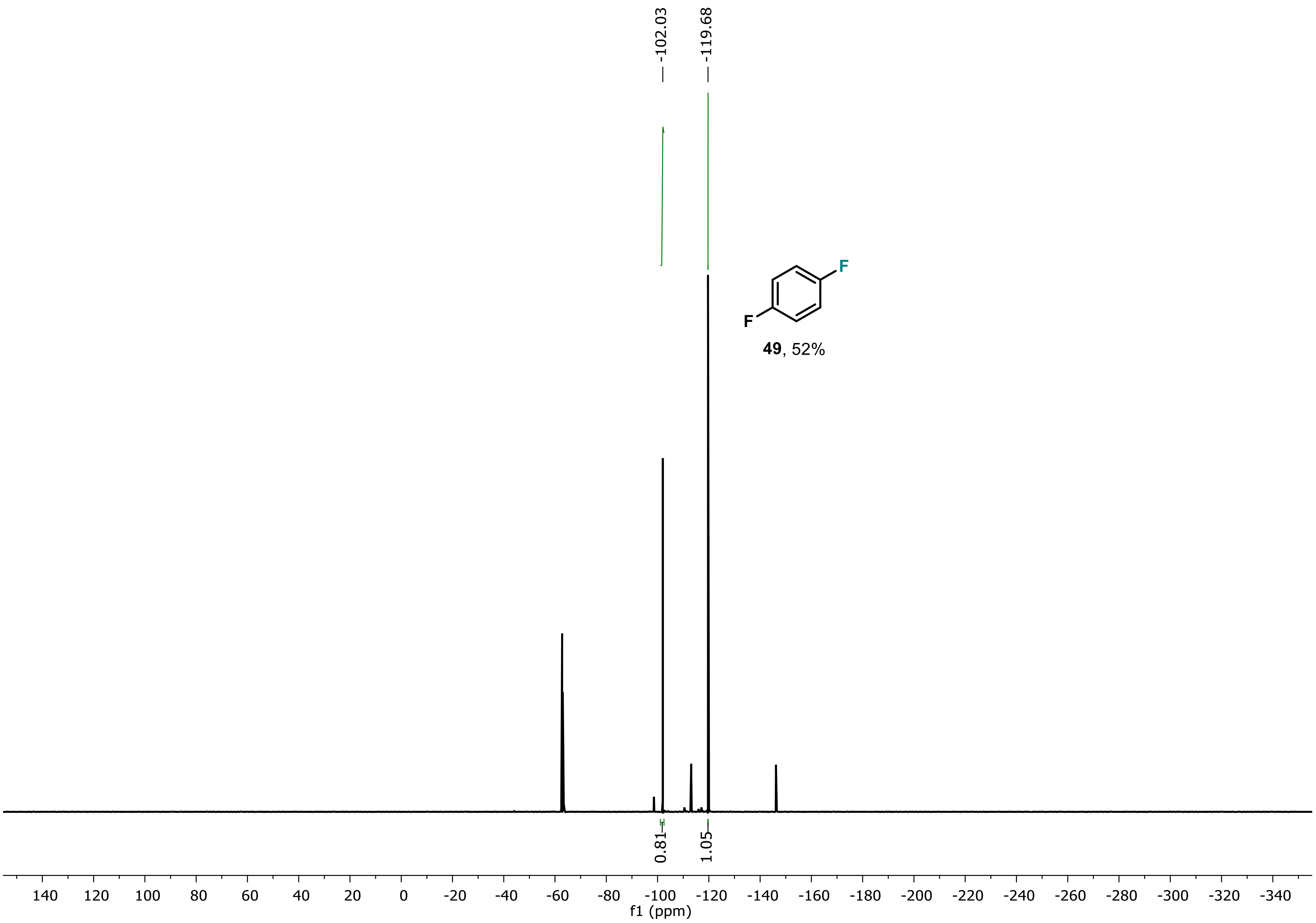

-102.03
-119.68
F
F
49, 52%
0.81
1.05
140
120
100
80
60
40
20
0
-20
-40
-60
-80
-100
-120
-140
-160
-180
-200
-220
-240
-260
-280
-300
-320
-340
f1 (ppm)

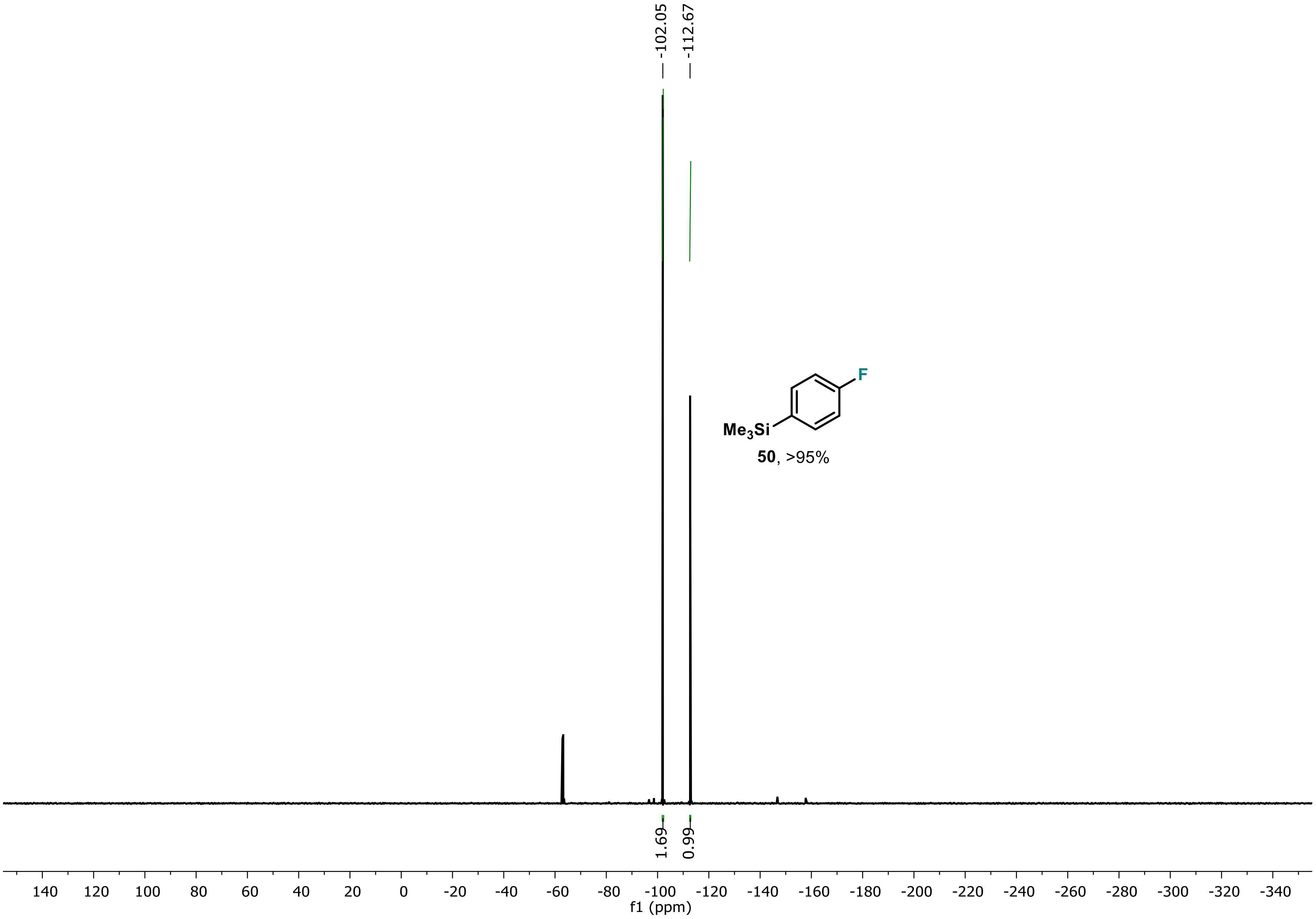

-102.05
-112.67
Me3Si
F
50, >95%
1.69
0.99
140 120 100 80 60 40 20 0 -20 -40 -60 -80 -100 -120 -140 -160 -180 -200 -220 -240 -260 -280 -300 -320 -340
f1 (ppm)

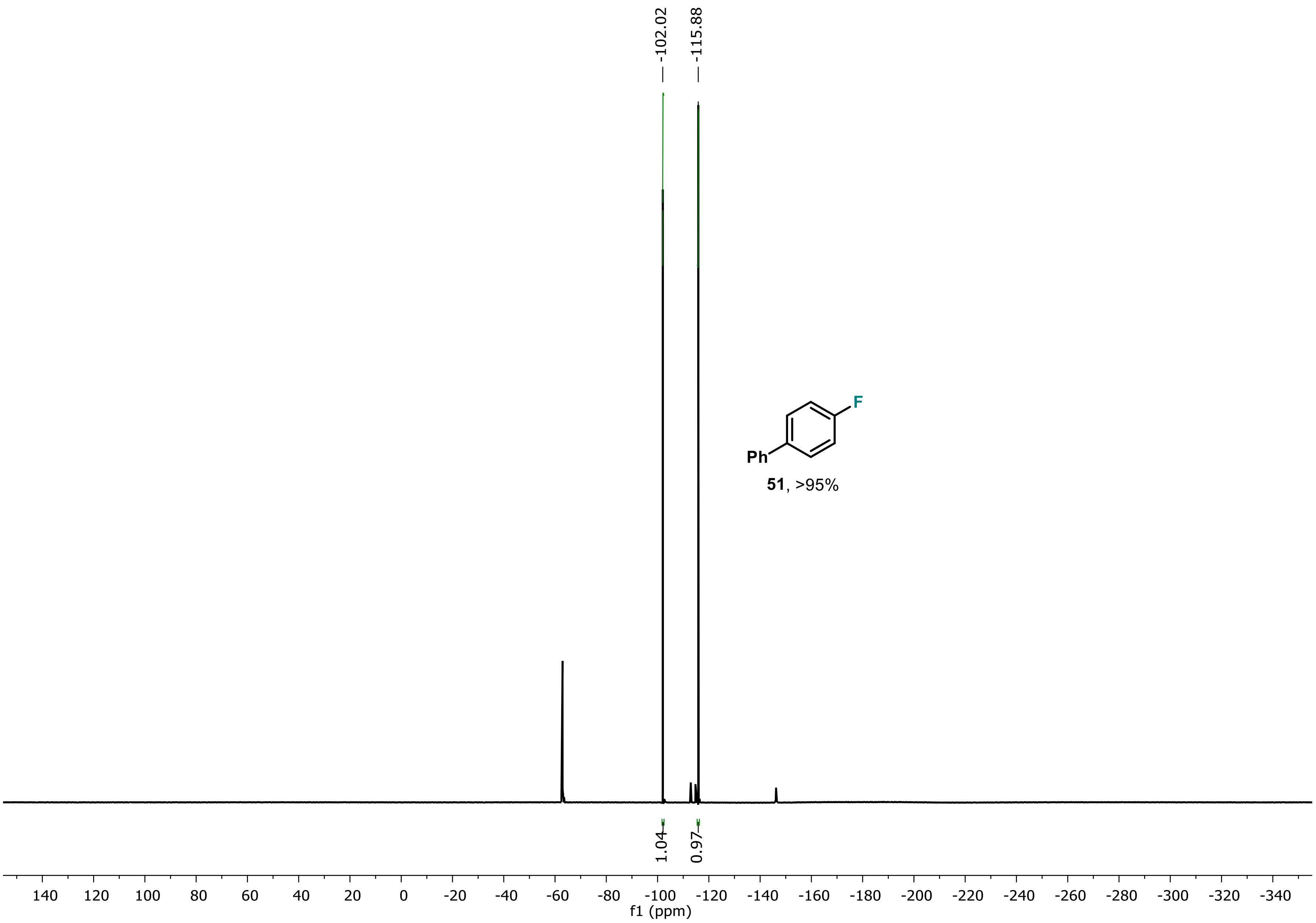

-102.02
-115.88
F
Ph
51, >95%
1.04
0.97
140
120
100
80
60
40
20
0
-20
-40
-60
-80
-100
-120
-140
-160
-180
-200
-220
-240
-260
-280
-300
-320
-340
f1 (ppm)

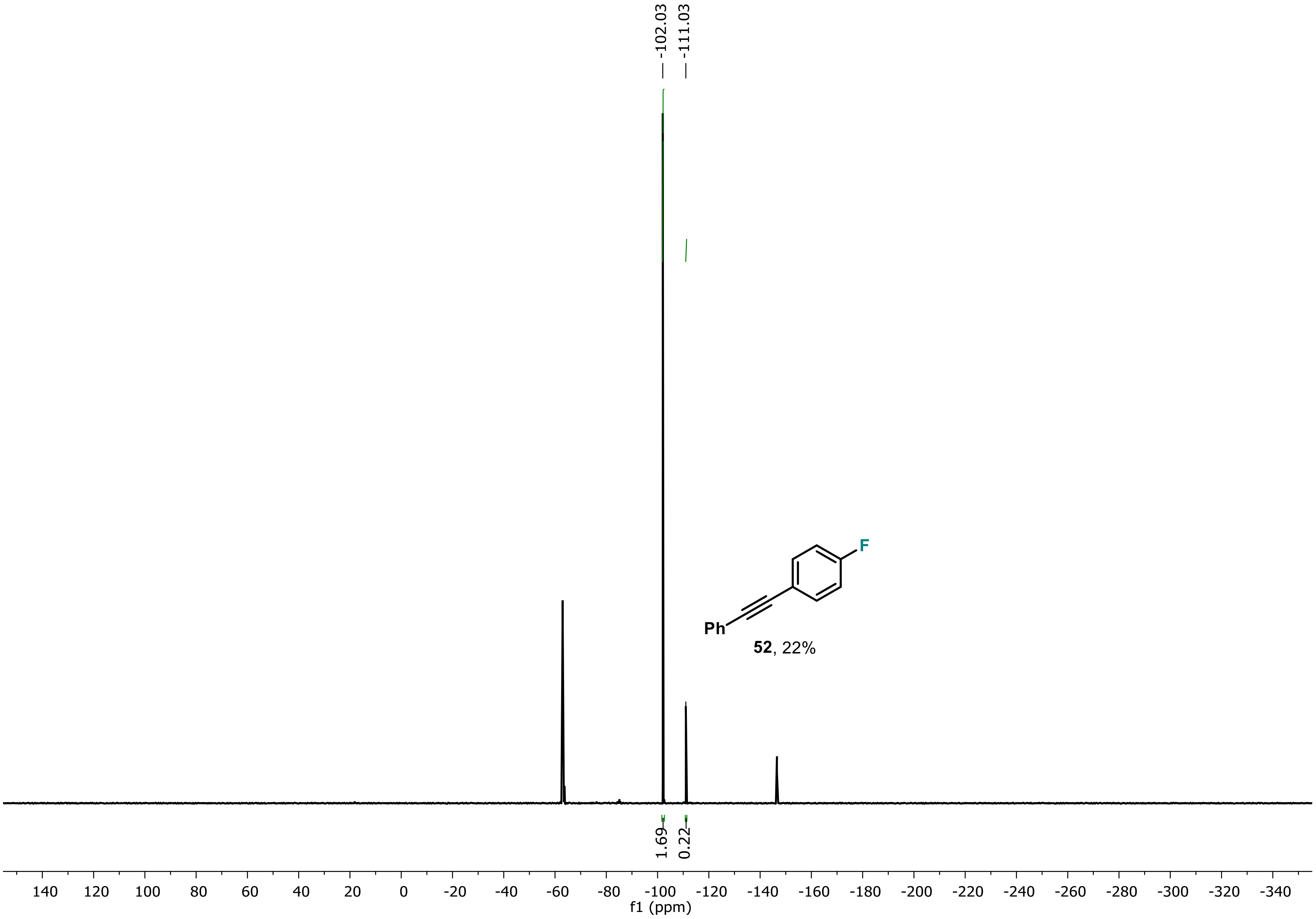

-102.03
-111.03
Ph
F
52, 22%
1.69
0.22
140 120 100 80 60 40 20 0 -20 -40 -60 -80 -100 -120 -140 -160 -180 -200 -220 -240 -260 -280 -300 -320 -340
f1 (ppm)

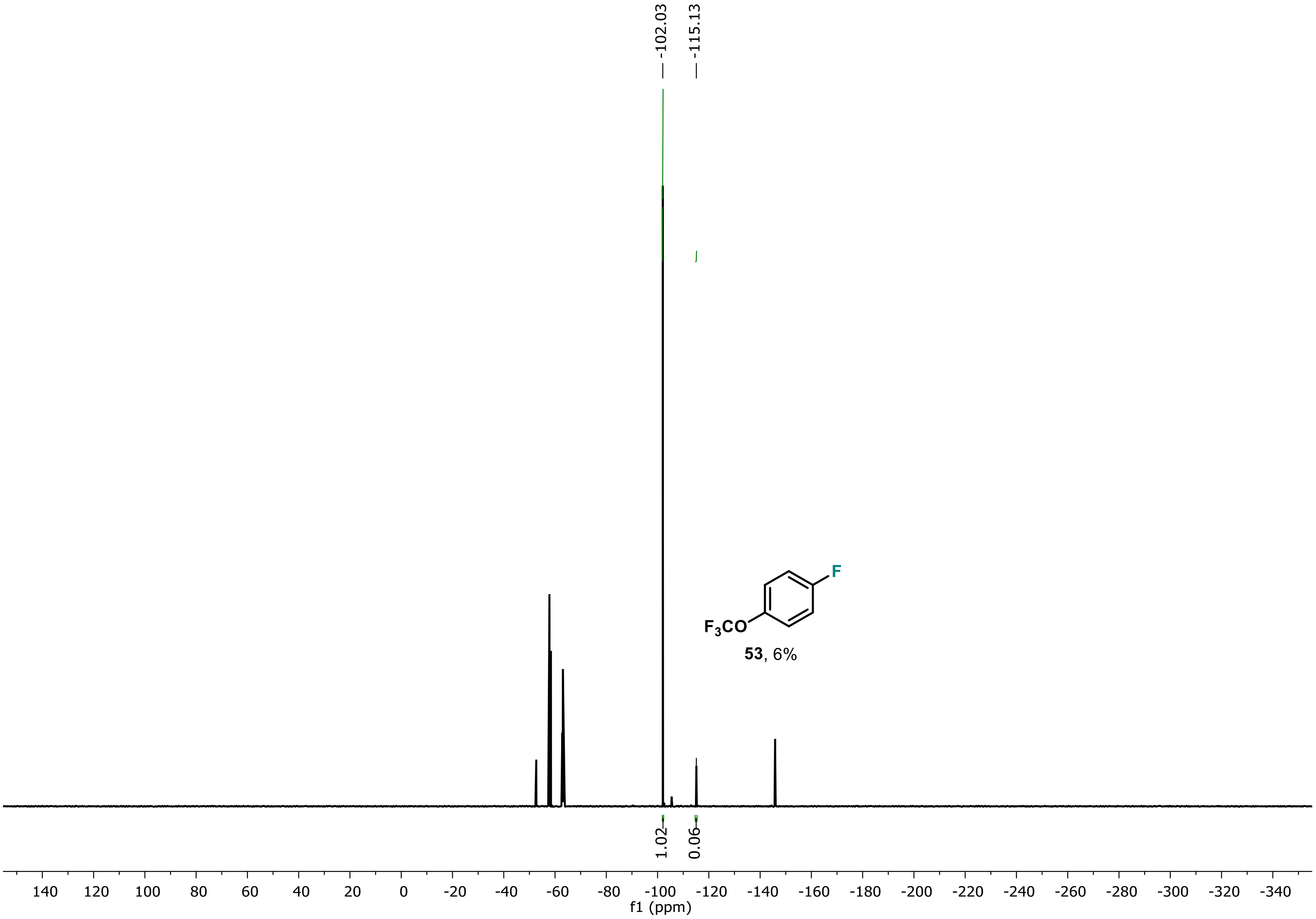

-102.03
-115.13
F3CO
F
53, 6%
1.02
0.06
140 120 100 80 60 40 20 0 -20 -40 -60 -80 -100 -120 -140 -160 -180 -200 -220 -240 -260 -280 -300 -320 -340
f1 (ppm)

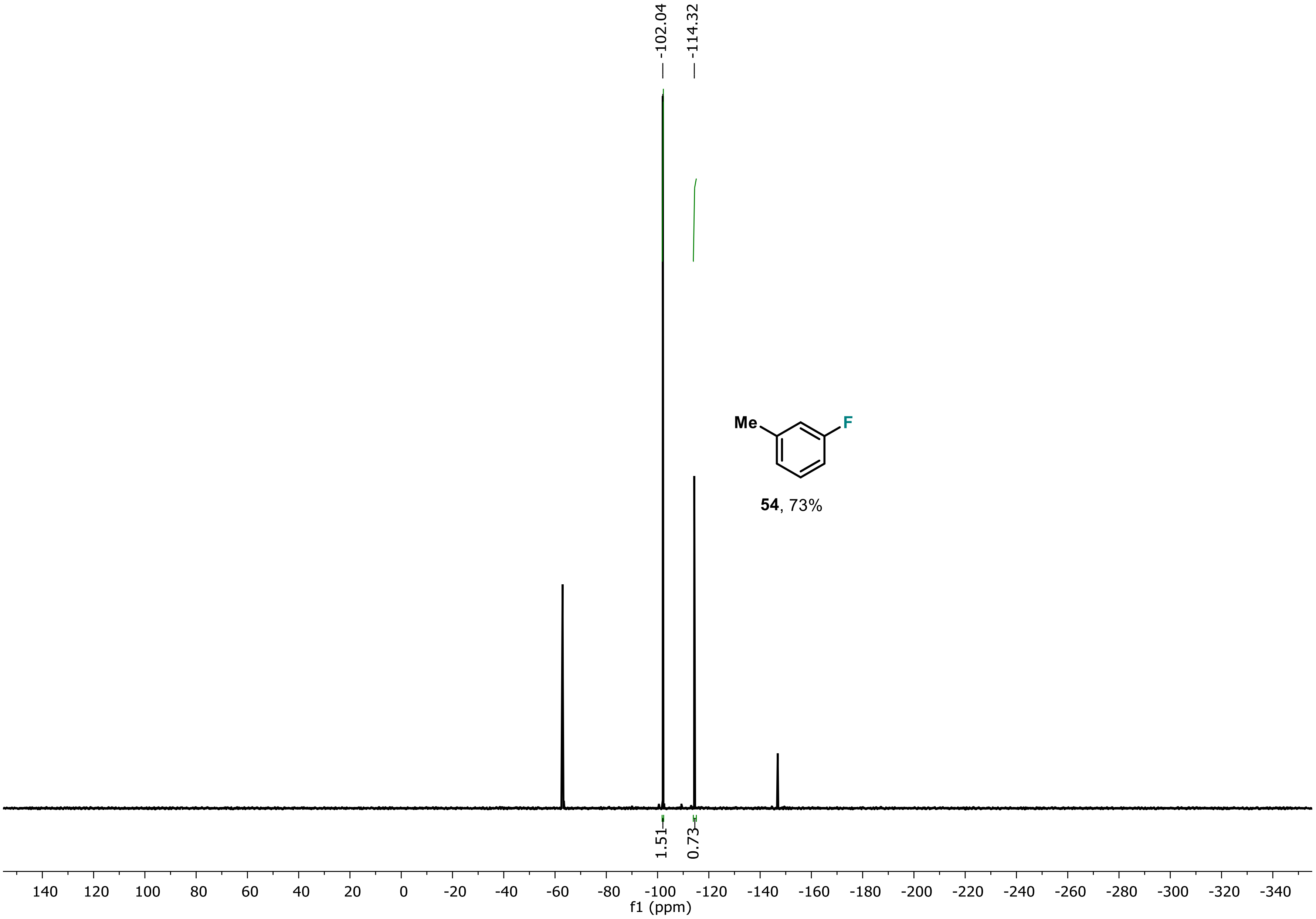
-102.04
-114.32
Me
F
54, 73%
1.51
0.73
140
120
100
80
60
40
20
0
-20
-40
-60
-80
-100
-120
-140
-160
-180
-200
-220
-240
-260
-280
-300
-320
-340
f1 (ppm)

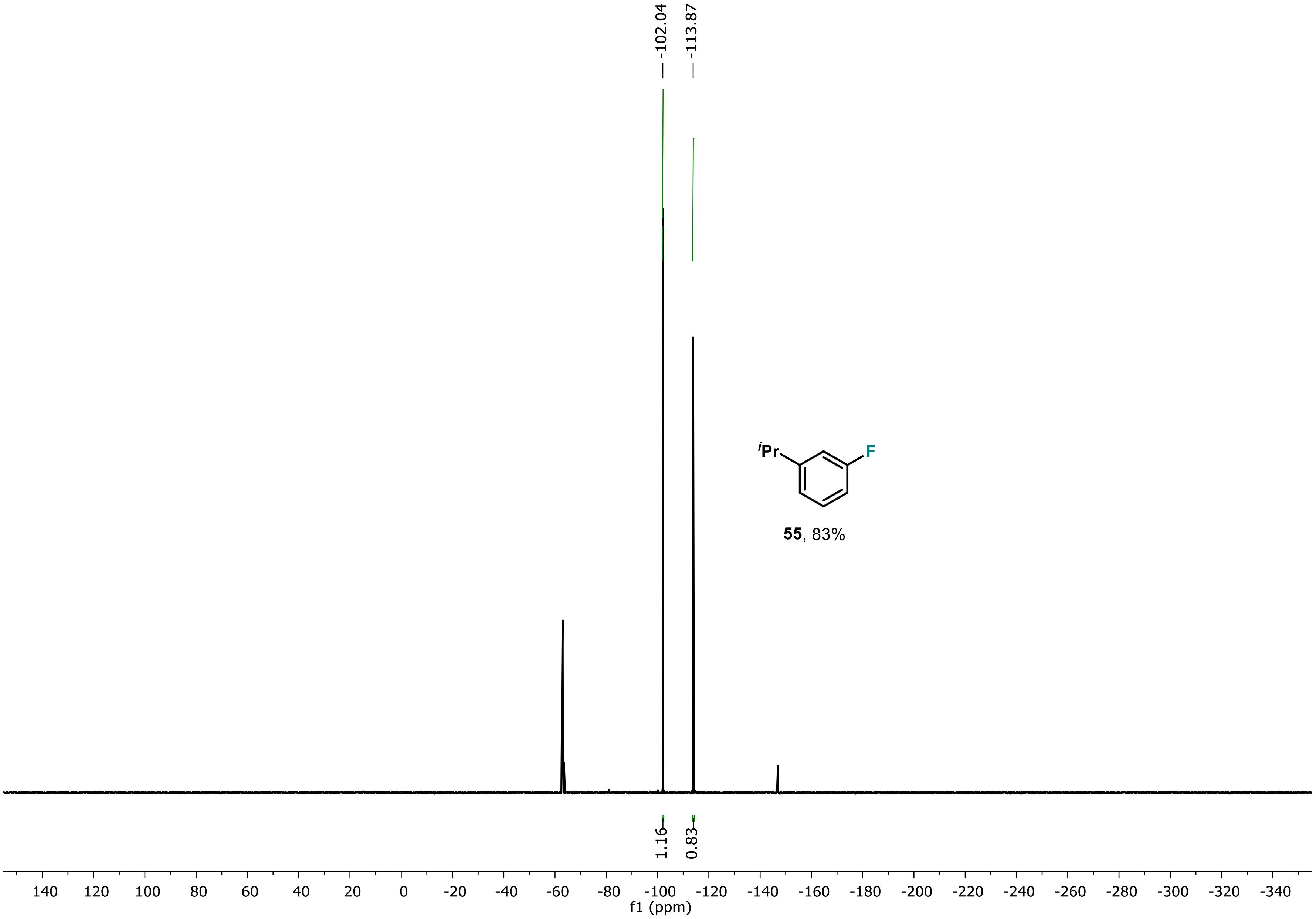
-102.04
-113.87
iPr
F
55, 83%
1.16
0.83
140 120 100 80 60 40 20 0 -20 -40 -60 -80 -100 -120 -140 -160 -180 -200 -220 -240 -260 -280 -300 -320 -340
f1 (ppm)

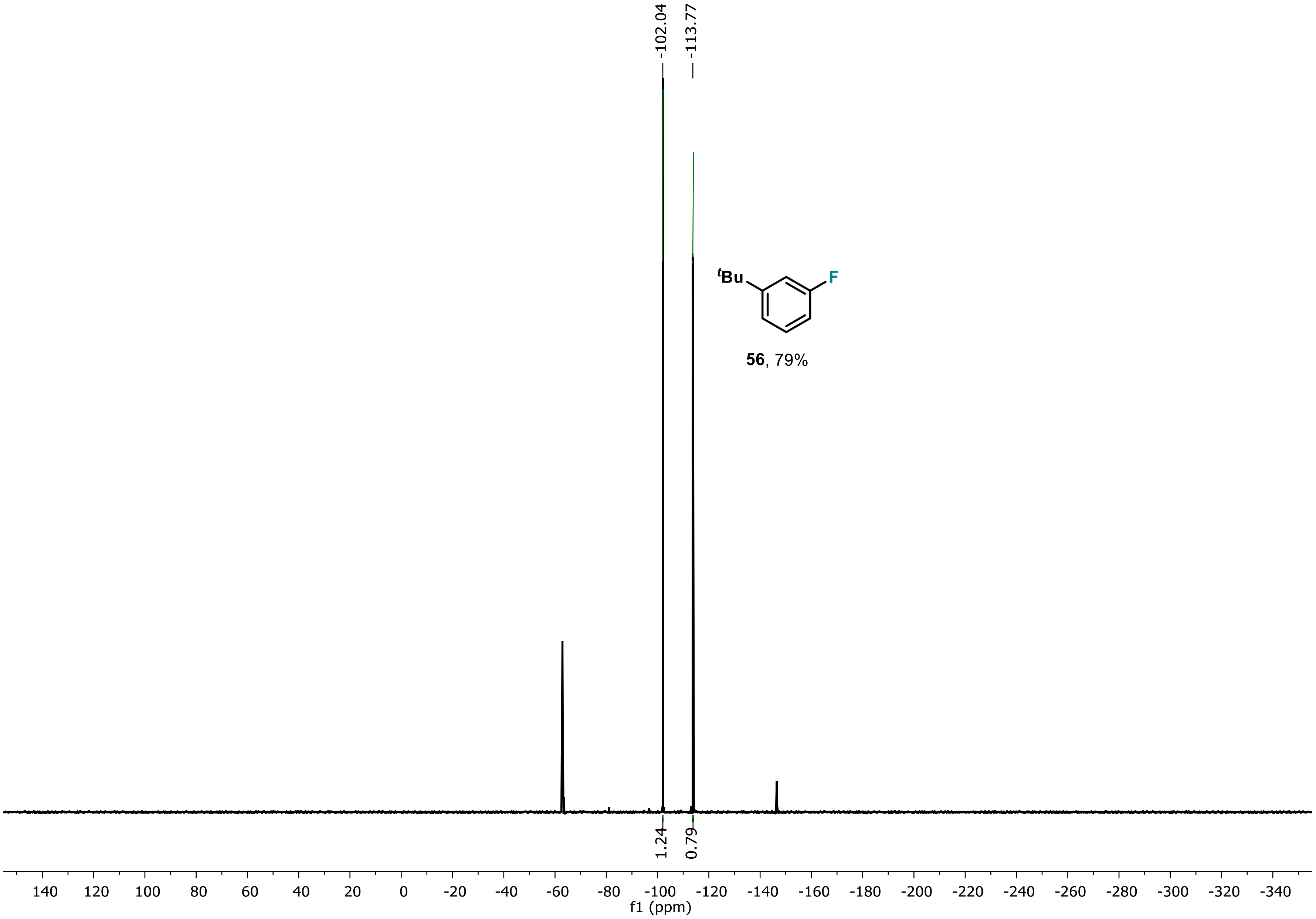

-102.04
-113.77
^{t}Bu
F
56, 79%
1.24
0.79
140 120 100 80 60 40 20 0 -20 -40 -60 -80 -100 -120 -140 -160 -180 -200 -220 -240 -260 -280 -300 -320 -340
f1 (ppm)

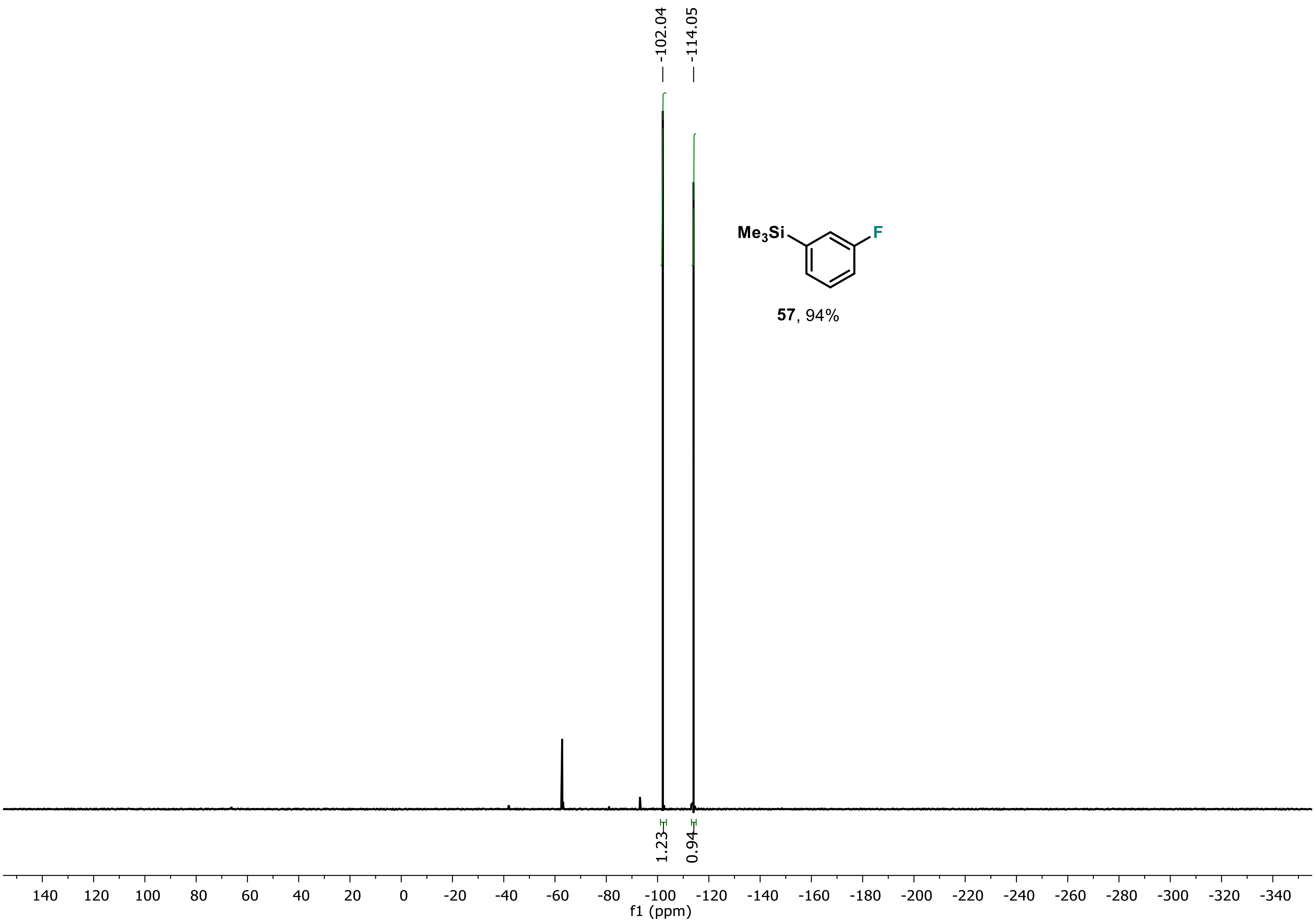

-102.04
-114.05
Me3Si
F
57, 94%
1.23
0.94
140 120 100 80 60 40 20 0 -20 -40 -60 -80 -100 -120 -140 -160 -180 -200 -220 -240 -260 -280 -300 -320 -340
f1 (ppm)

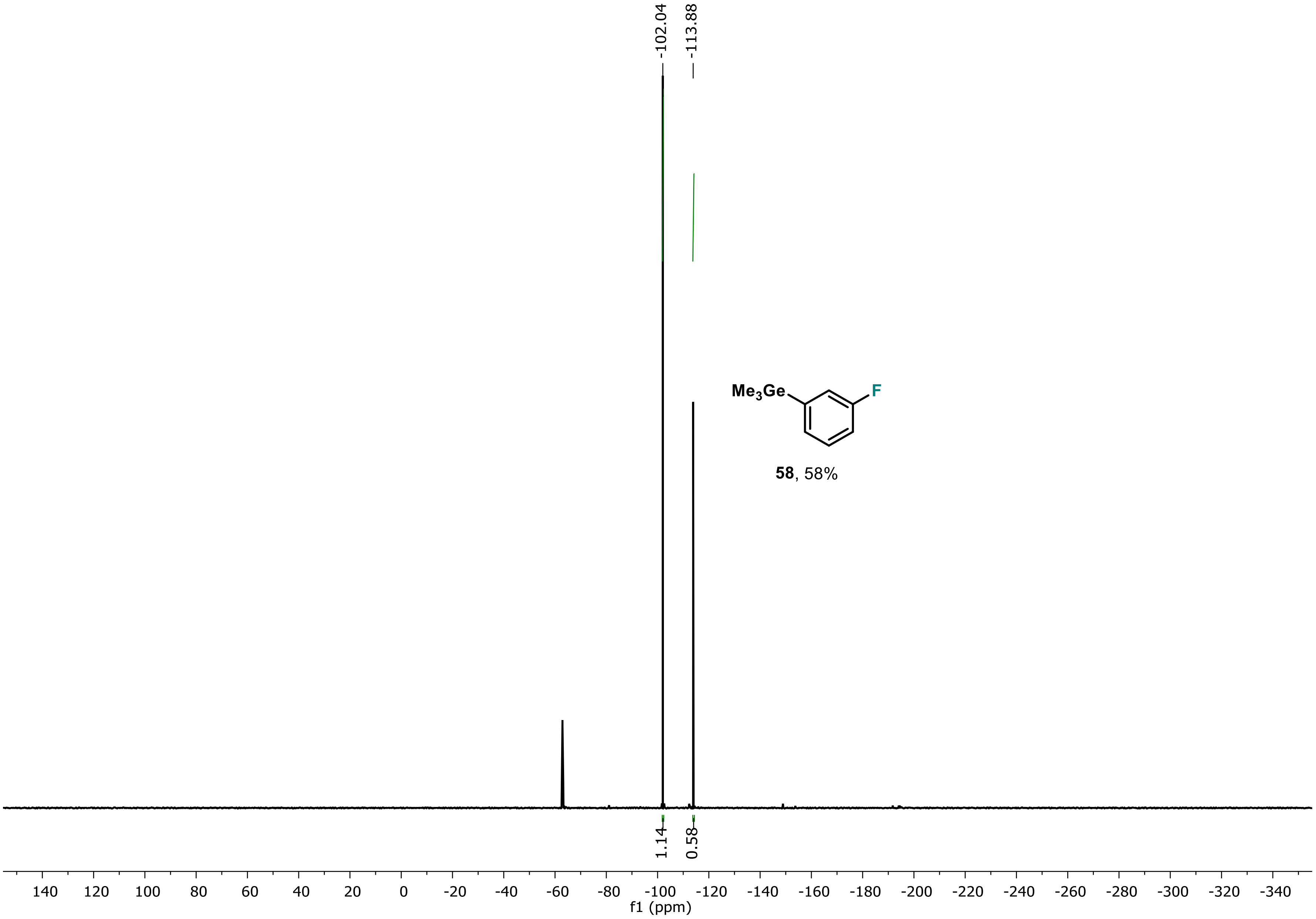

-102.04
-113.88
Me3Ge
F
1.14
0.58
140
120
100
80
60
40
20
0
-20
-40
-60
-80
-100
-120
-140
-160
-180
-200
-220
-240
-260
-280
-300
-320
-340
f1 (ppm)

58, 58%

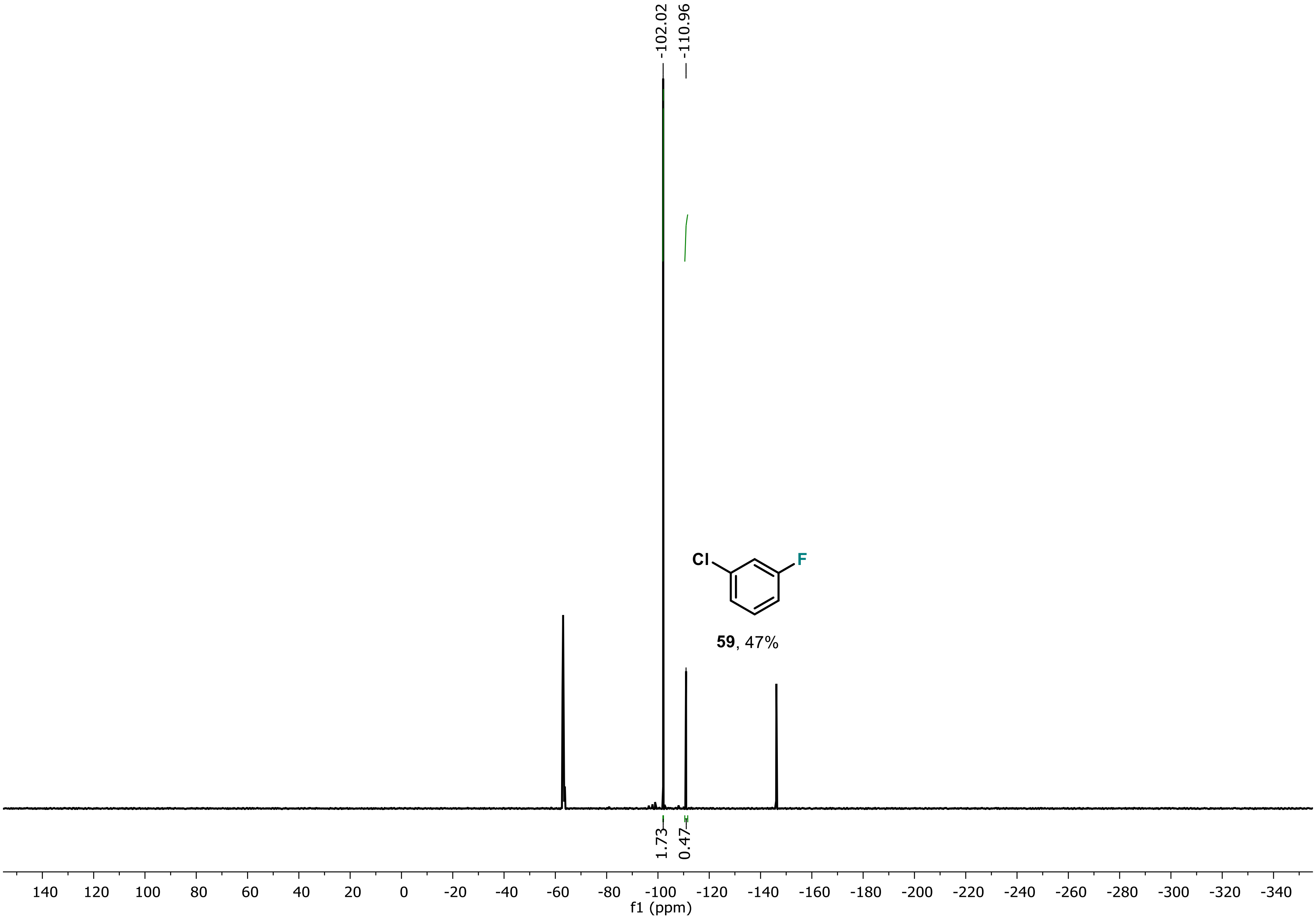
-102.02
-110.96
Cl
F
59, 47%
1.73
0.47
140 120 100 80 60 40 20 0 -20 -40 -60 -80 -100 -120 -140 -160 -180 -200 -220 -240 -260 -280 -300 -320 -340
f1 (ppm)

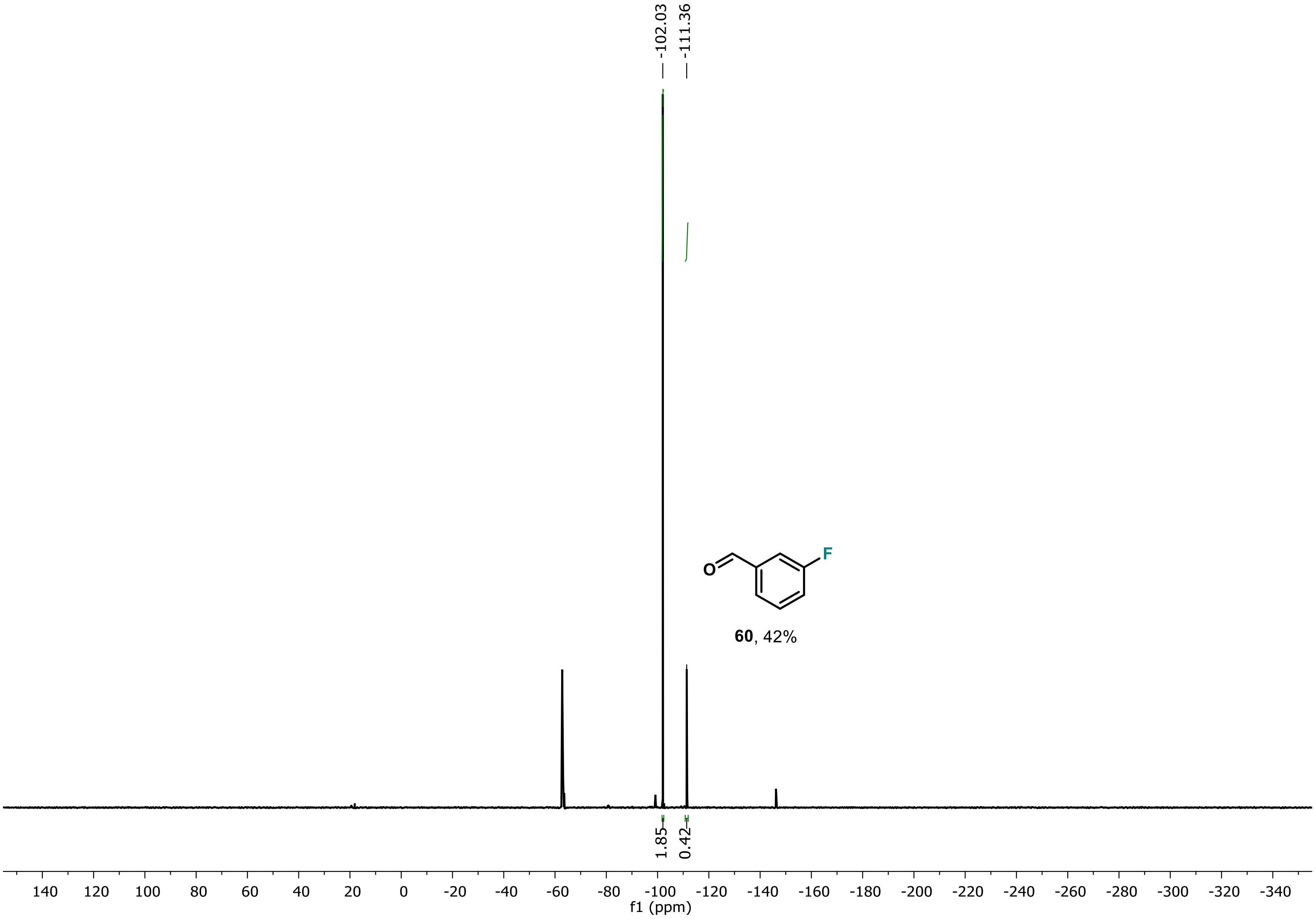
-102.03
-111.36
O
F
1.85
0.42
140 120 100 80 60 40 20 0 -20 -40 -60 -80 -100 -120 -140 -160 -180 -200 -220 -240 -260 -280 -300 -320 -340
f1 (ppm)

60, 42%

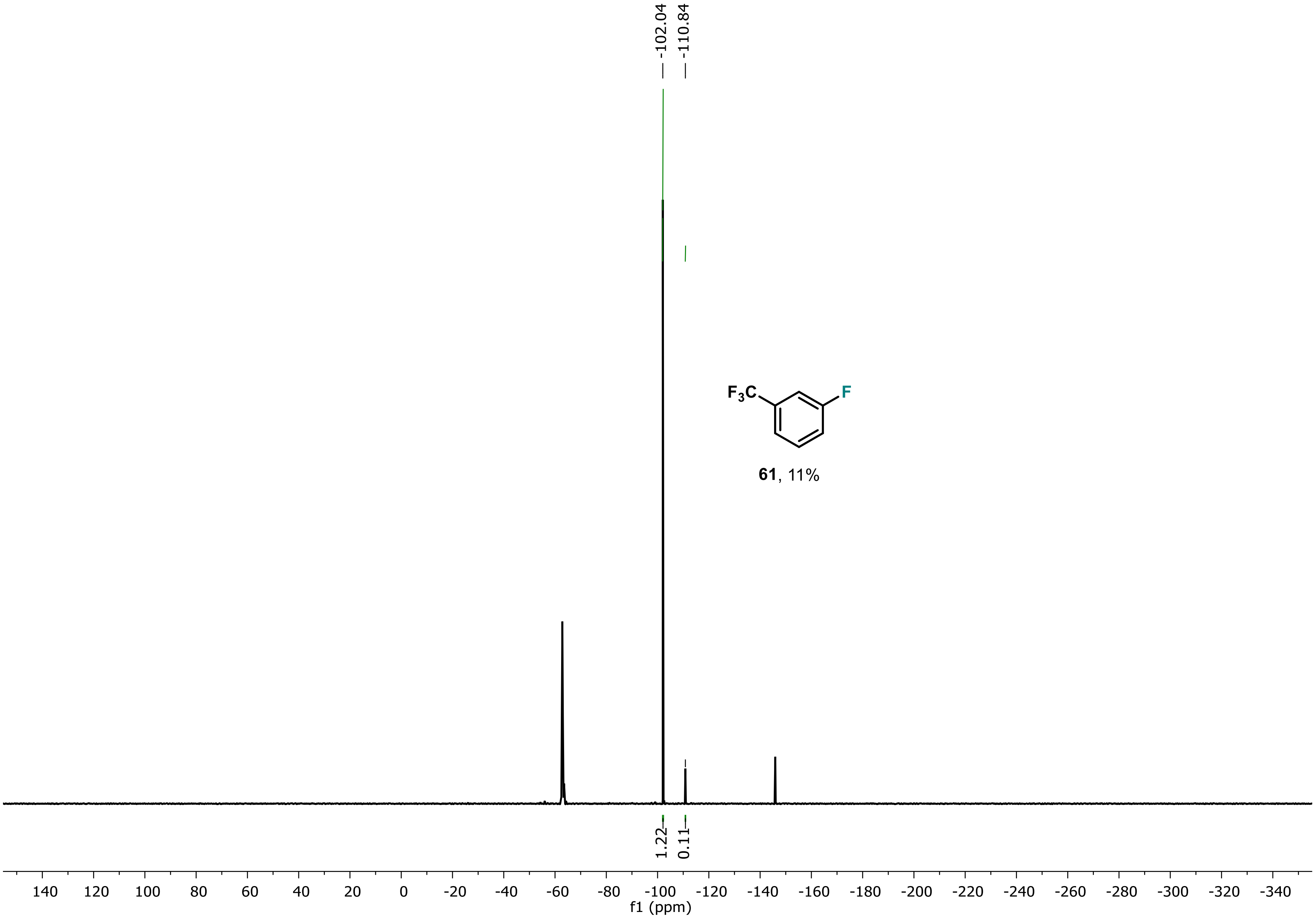
-102.04
-110.84
F3C
F
61, 11%
1.22
0.11
140 120 100 80 60 40 20 0 -20 -40 -60 -80 -100 -120 -140 -160 -180 -200 -220 -240 -260 -280 -300 -320 -340
f1 (ppm)

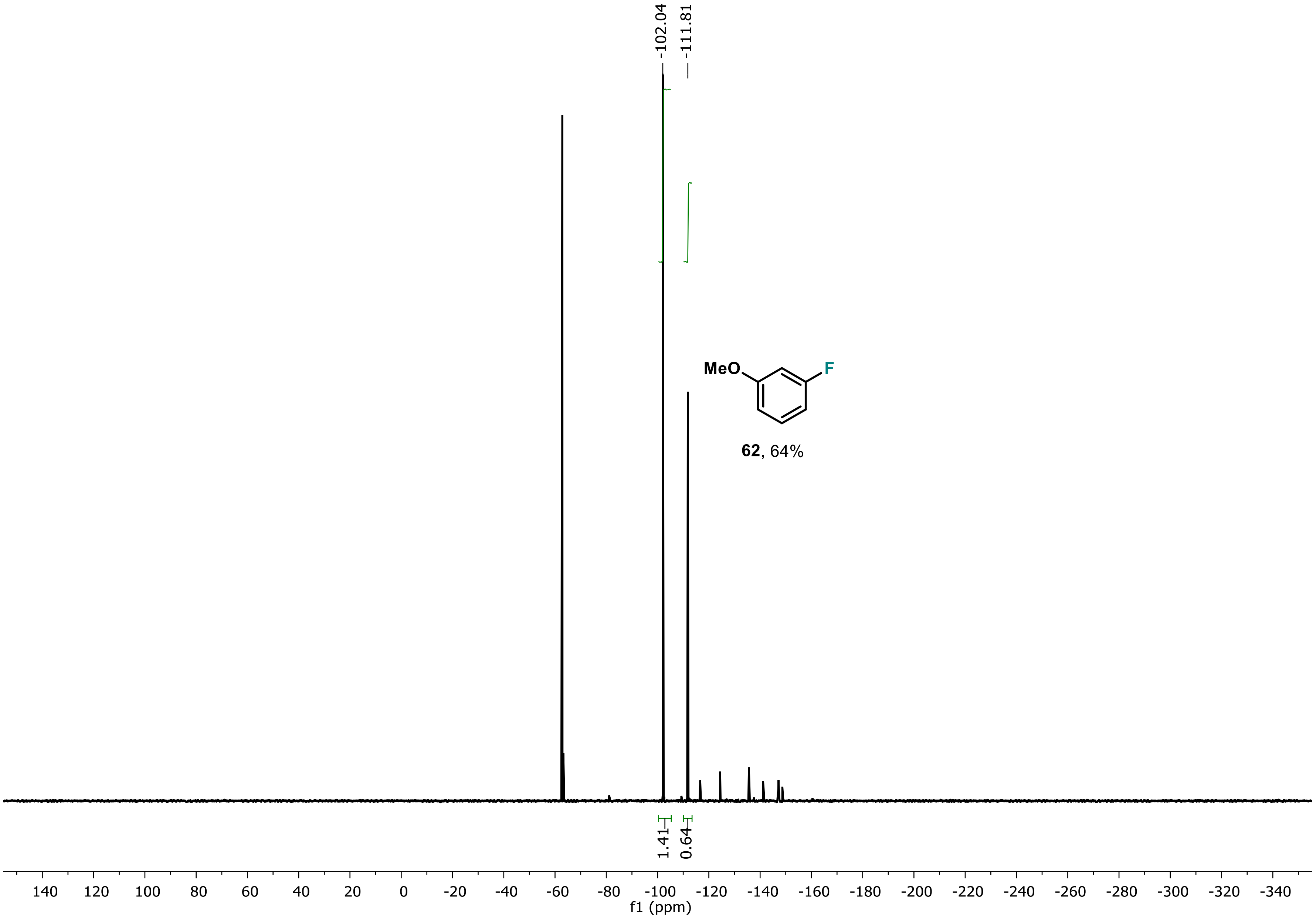

-102.04
-111.81
MeO
F
1.41
0.64
140
120
100
80
60
40
20
0
-20
-40
-60
-80
-100
-120
-140
-160
-180
-200
-220
-240
-260
-280
-300
-320
-340
f1 (ppm)

62, 64%

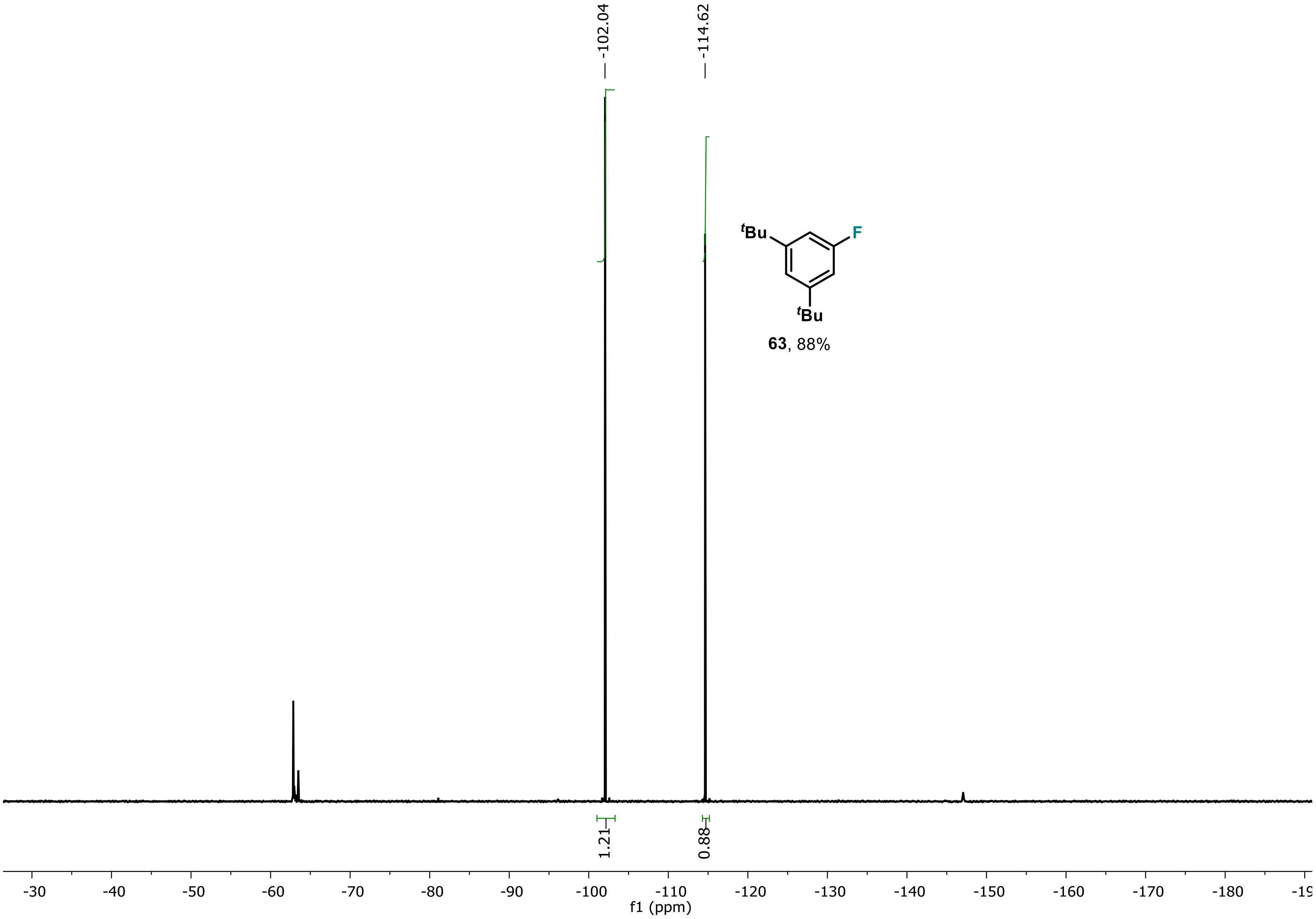
-102.04
-114.62
^{t}Bu
F
^{t}Bu
63, 88%
1.21
0.88
-30
-40
-50
-60
-70
-80
-90
-100
-110
-120
-130
-140
-150
-160
-170
-180
f1 (ppm)

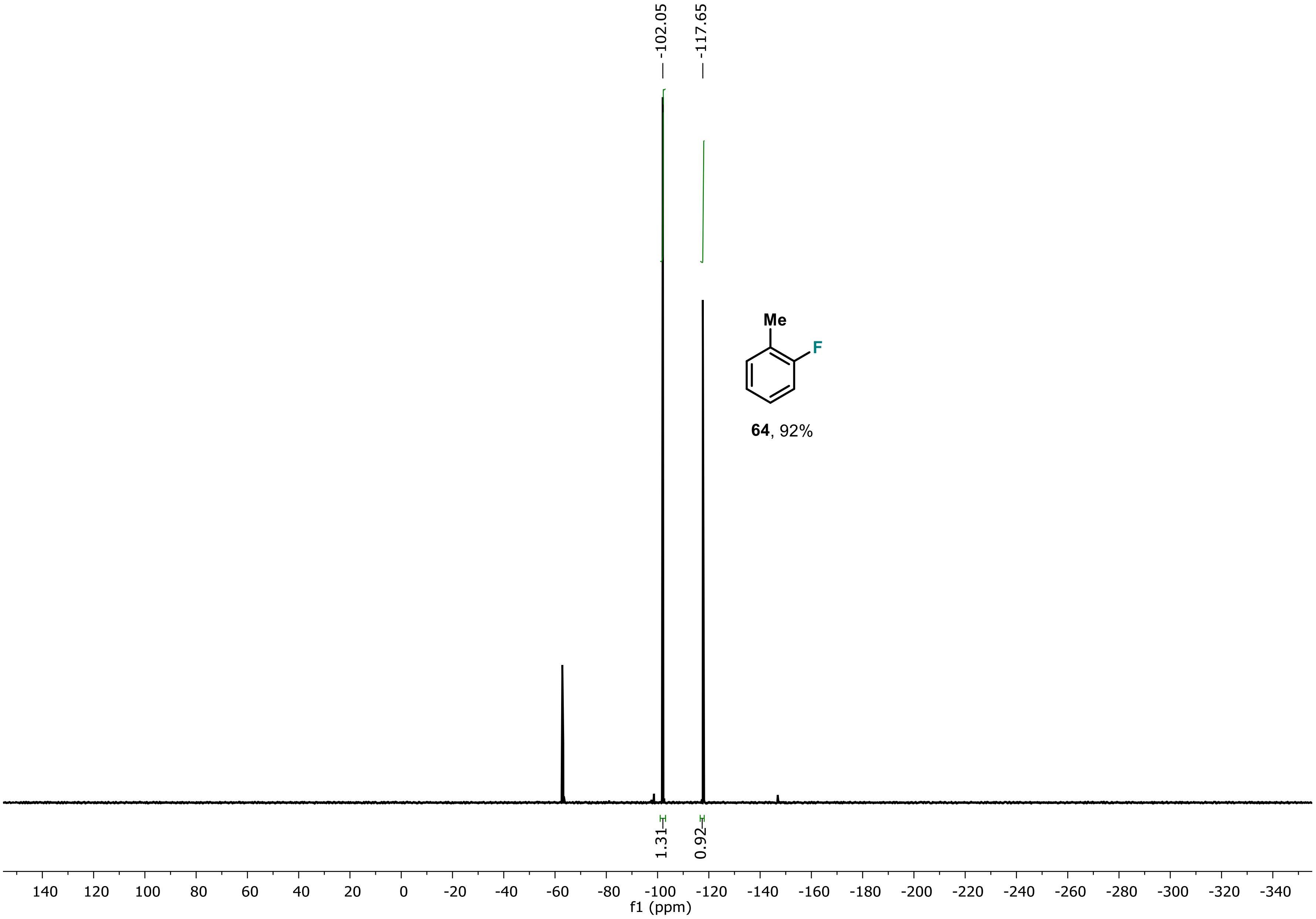
-102.05
-117.65
Me
F
64, 92%
1.31
0.92
140 120 100 80 60 40 20 0 -20 -40 -60 -80 -100 -120 -140 -160 -180 -200 -220 -240 -260 -280 -300 -320 -340
f1 (ppm)

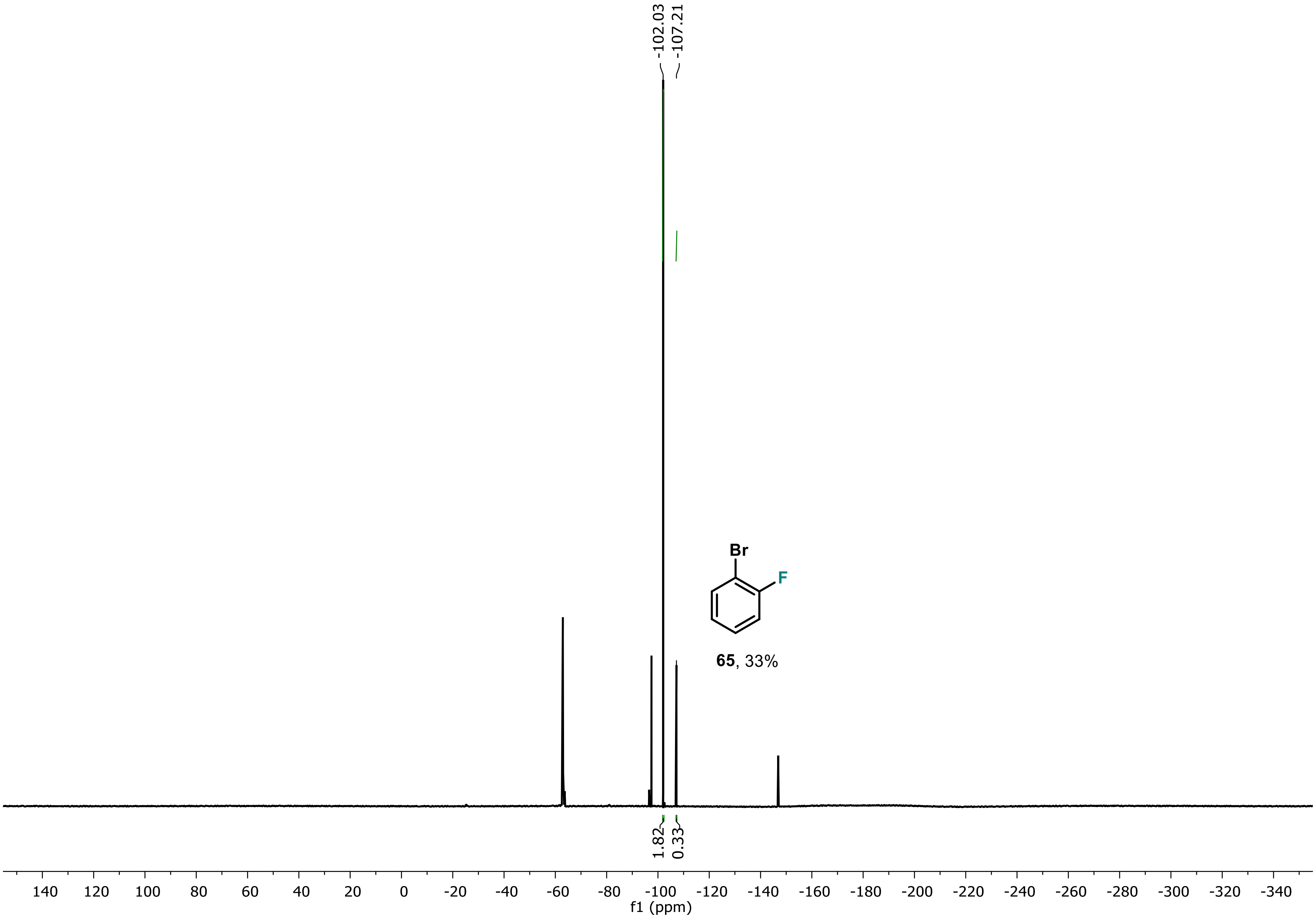
-102.03
-107.21
Br
F
65, 33%
1.82
0.33
140 120 100 80 60 40 20 0 -20 -40 -60 -80 -100 -120 -140 -160 -180 -200 -220 -240 -260 -280 -300 -320 -340
f1 (ppm)

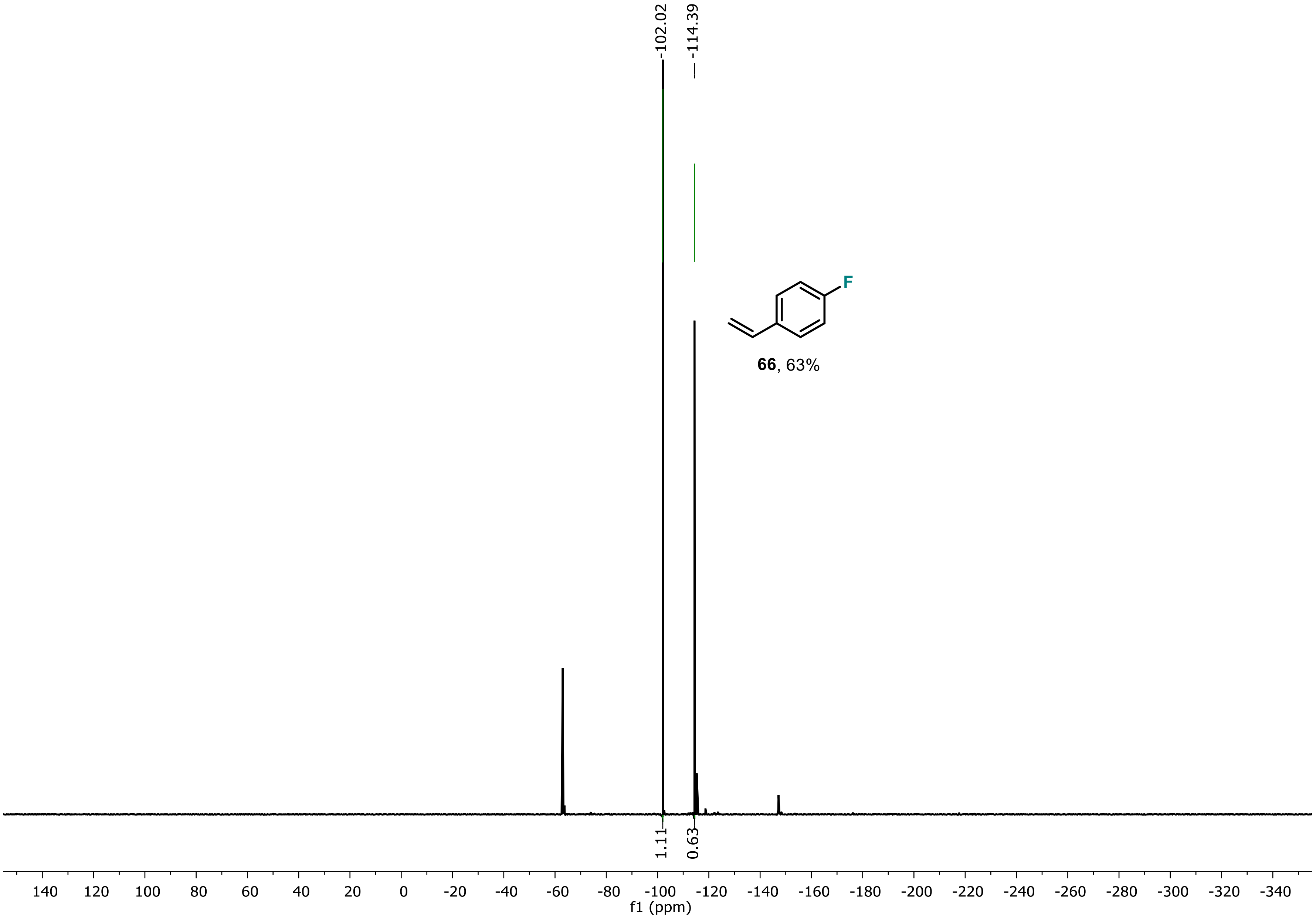
-102.02
-114.39
F
66, 63%
1.11
0.63
140 120 100 80 60 40 20 0 -20 -40 -60 -80 -100 -120 -140 -160 -180 -200 -220 -240 -260 -280 -300 -320 -340
f1 (ppm)

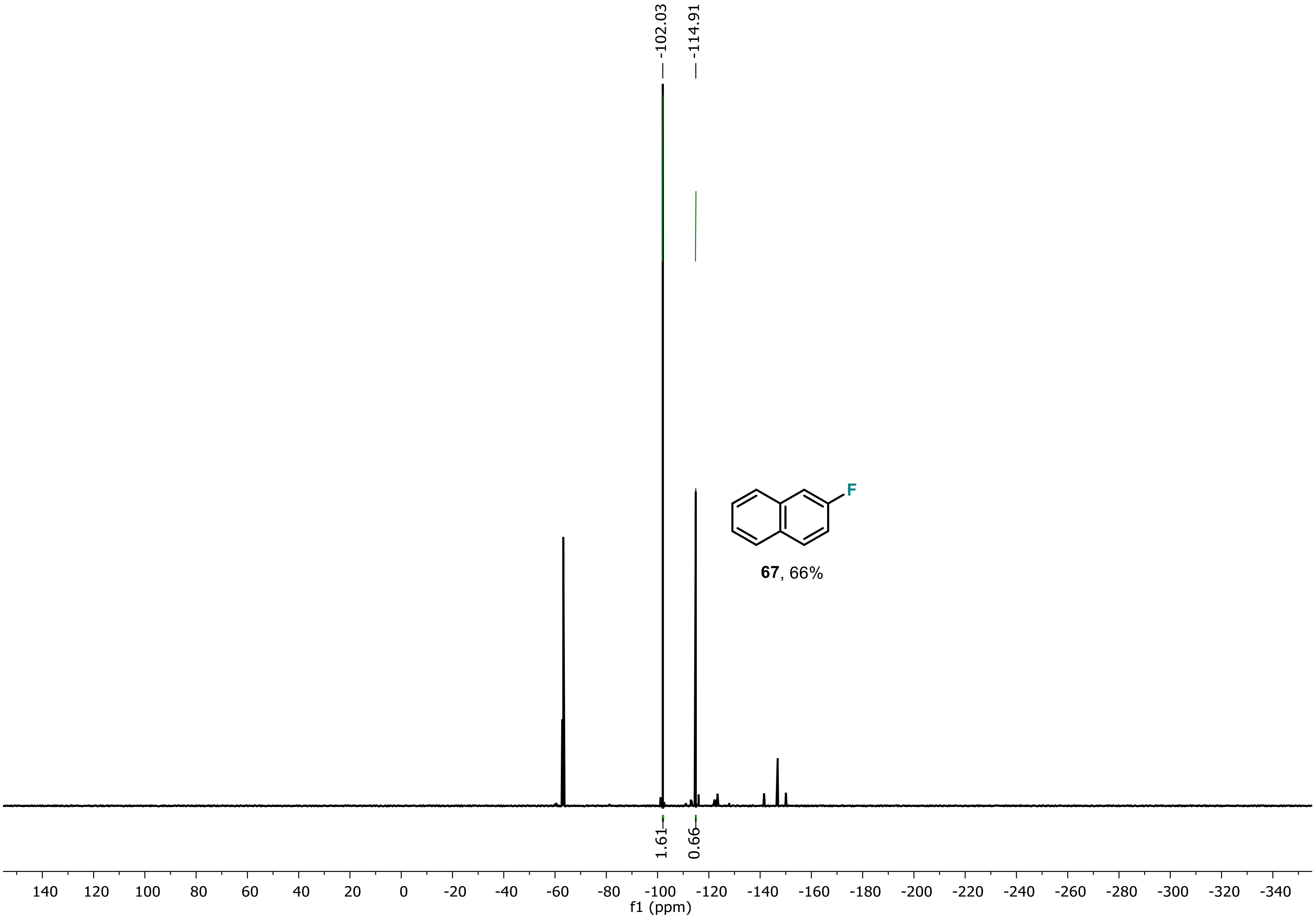

-102.03
-114.91
F
1.61
0.66
140
120
100
80
60
40
20
0
-20
-40
-60
-80
-100
-120
-140
-160
-180
-200
-220
-240
-260
-280
-300
-320
-340
f1 (ppm)

67, 66%

13. NMR spectra for isolated fluoroarenes from stoichiometric reactions

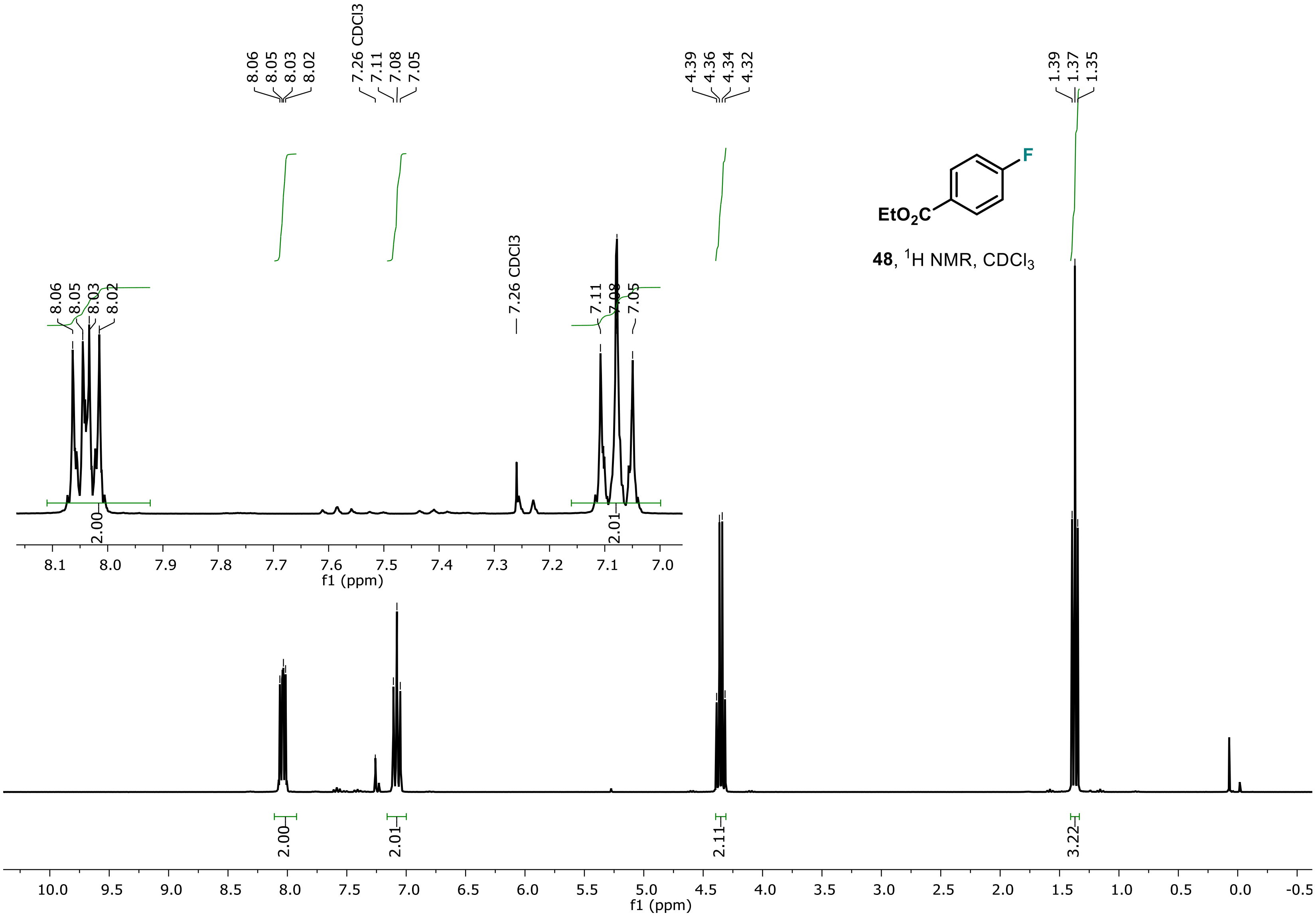

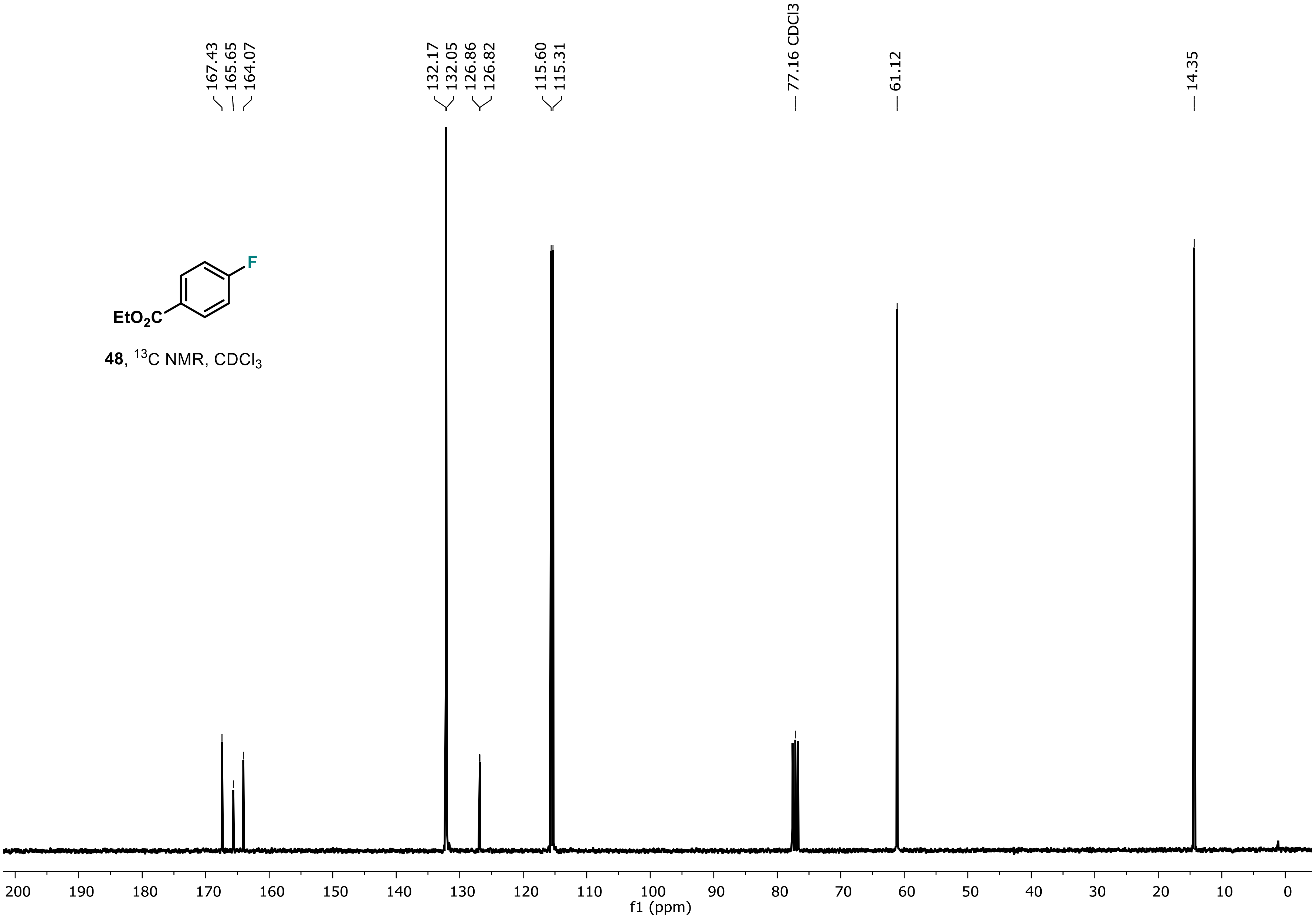

167.43
165.65
164.07
132.17
132.05
126.86
126.82
115.60
115.31
77.16 CDCl3
61.12
14.35
EtO2C
F
48, 13C NMR, CDCl3
200 190 180 170 160 150 140 130 120 110 100 90 80 70 60 50 40 30 20 10 0
f1 (ppm)

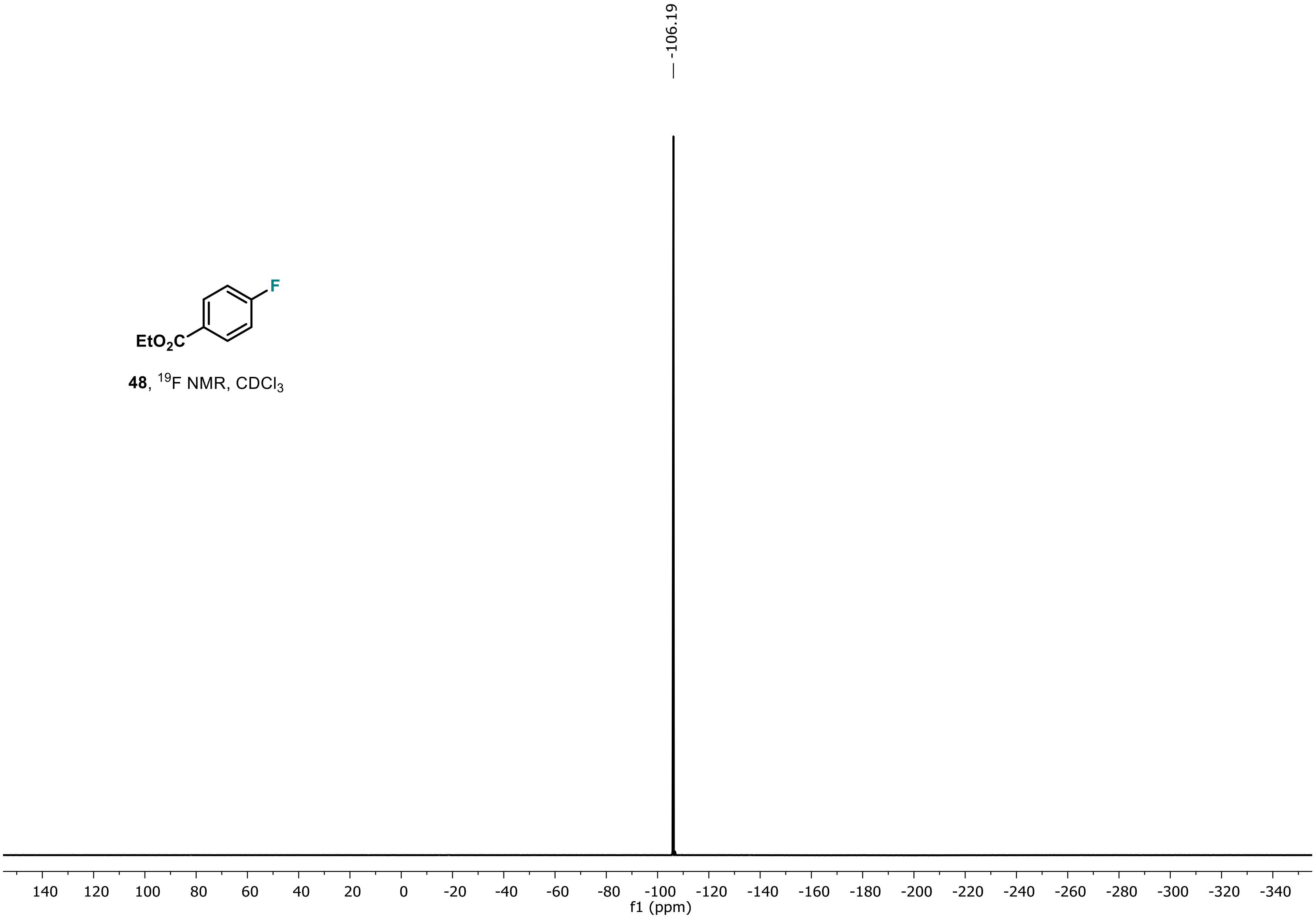
-106.19
EtO_2C
F
48, ^{19}F NMR, $CDCl_3$
140 120 100 80 60 40 20 0 -20 -40 -60 -80 -100 -120 -140 -160 -180 -200 -220 -240 -260 -280 -300 -320 -340
f1 (ppm)

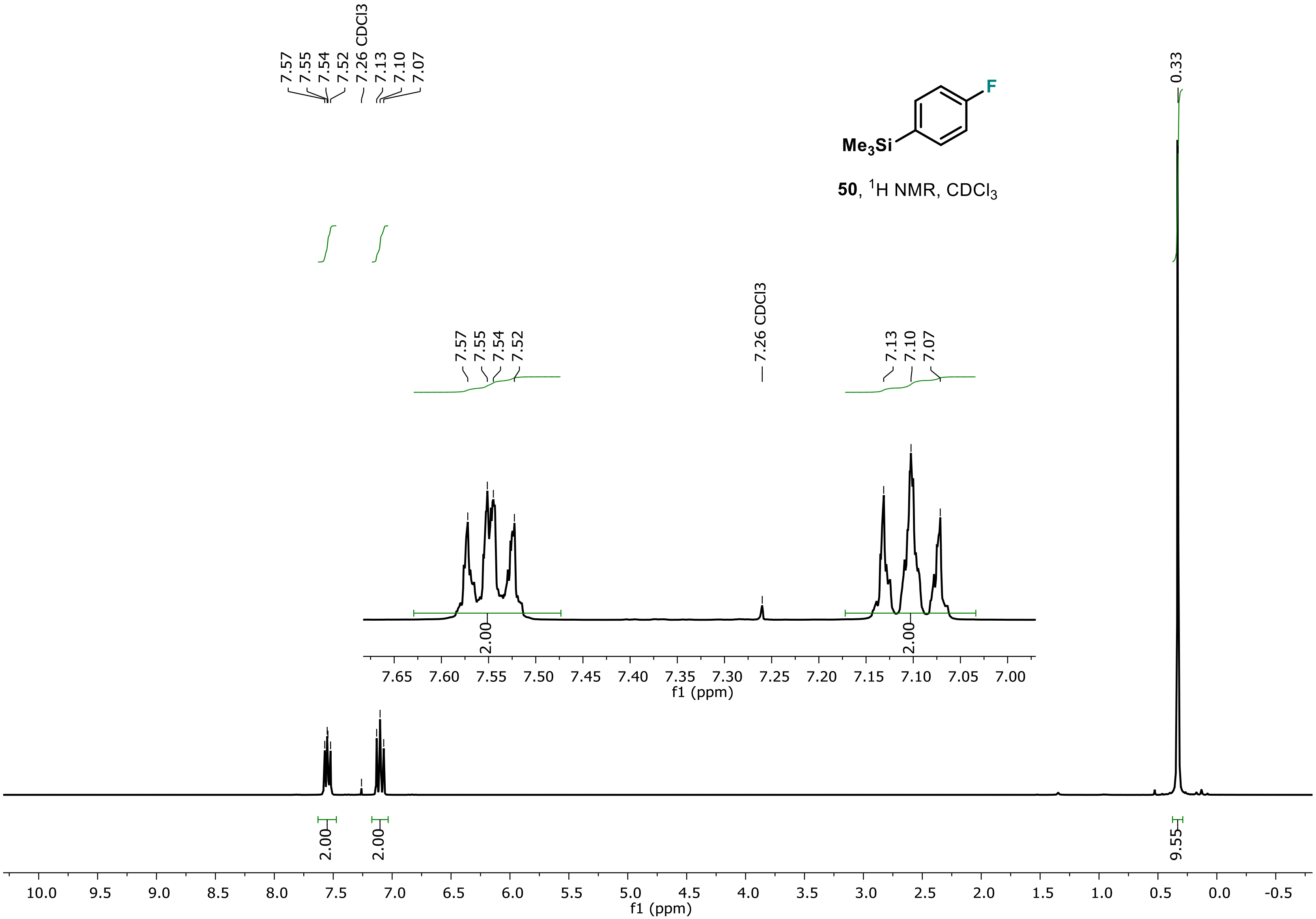
7.57
7.55
7.54
7.52
7.26 CDCl3
7.13
7.10
7.07
0.33
F
Me3Si
50, 1H NMR, CDCl3
2.00
2.00
9.55
f1 (ppm)

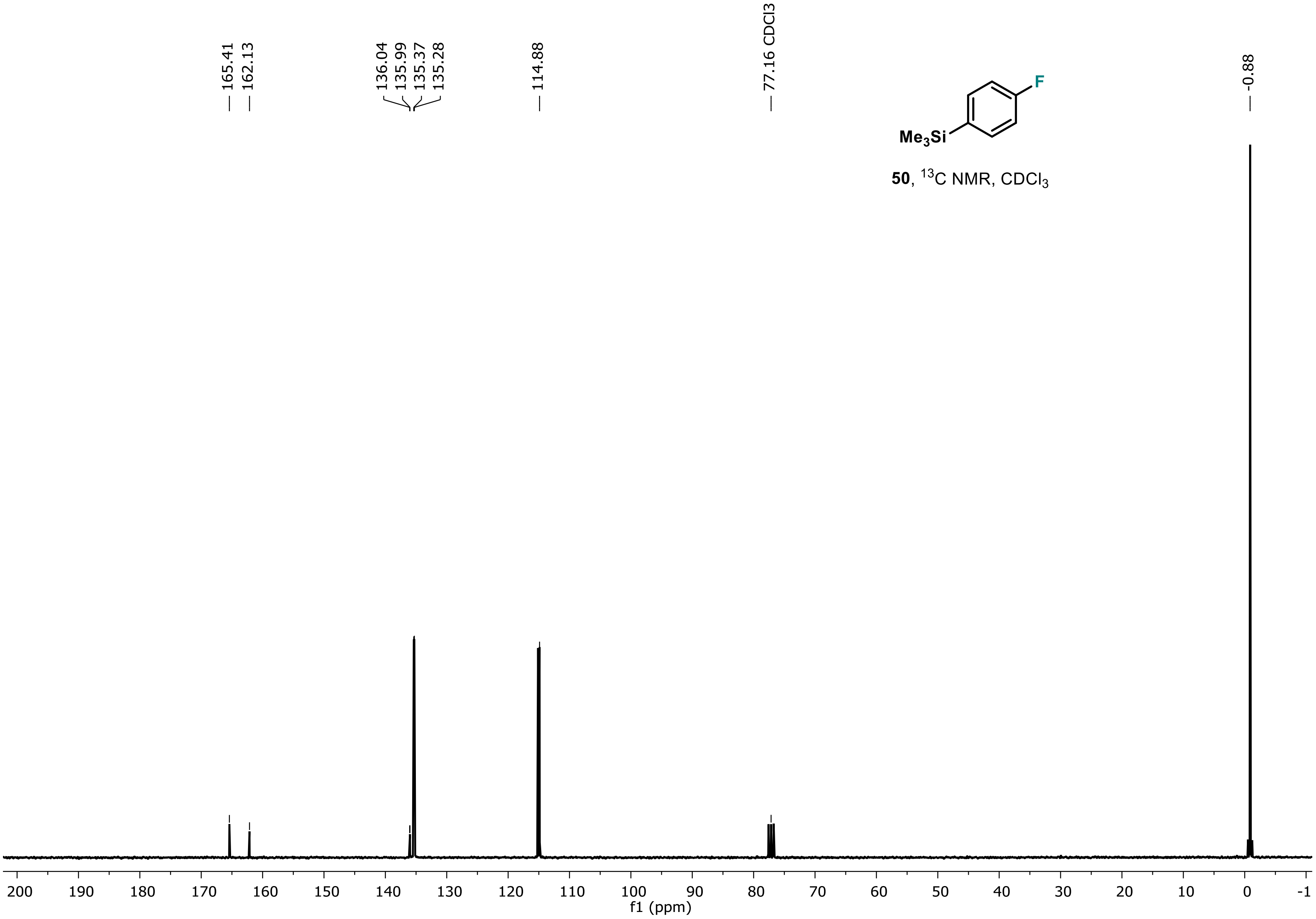

50, ^{13}C NMR, $CDCl_3$

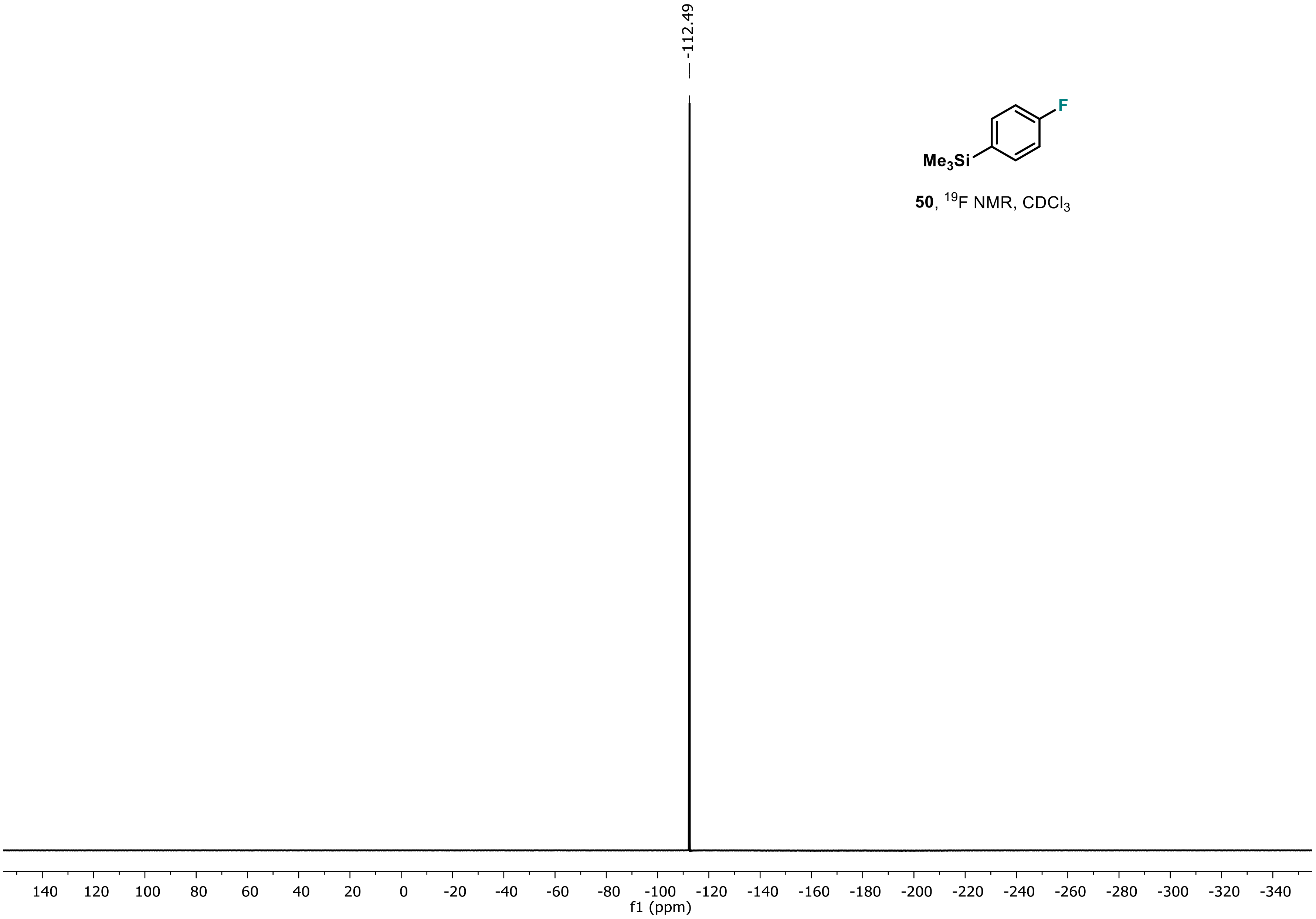
-112.49
F
Me3Si
50, 19F NMR, CDCl3
140 120 100 80 60 40 20 0 -20 -40 -60 -80 -100 -120 -140 -160 -180 -200 -220 -240 -260 -280 -300 -320 -340
f1 (ppm)

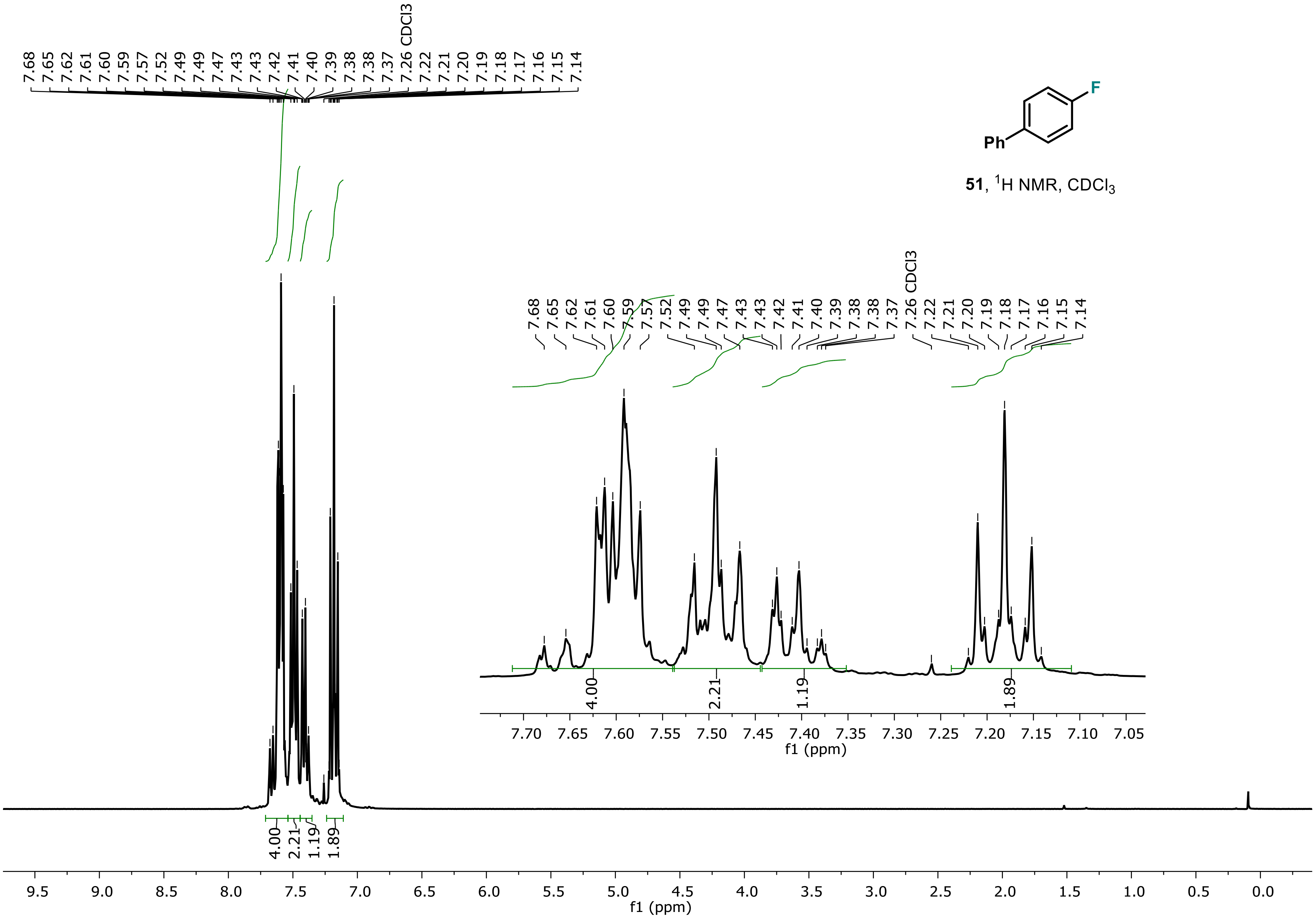

51, ^{1}H NMR, $CDCl_3$
Ph
F
7.68 7.65 7.62 7.61 7.60 7.59 7.57 7.52 7.49 7.49 7.47 7.43 7.43 7.42 7.41 7.40 7.39 7.38 7.38 7.37 7.26 CDCl3 7.22 7.21 7.20 7.19 7.18 7.17 7.16 7.15 7.14
4.00
2.21
1.19
1.89
f1 (ppm)

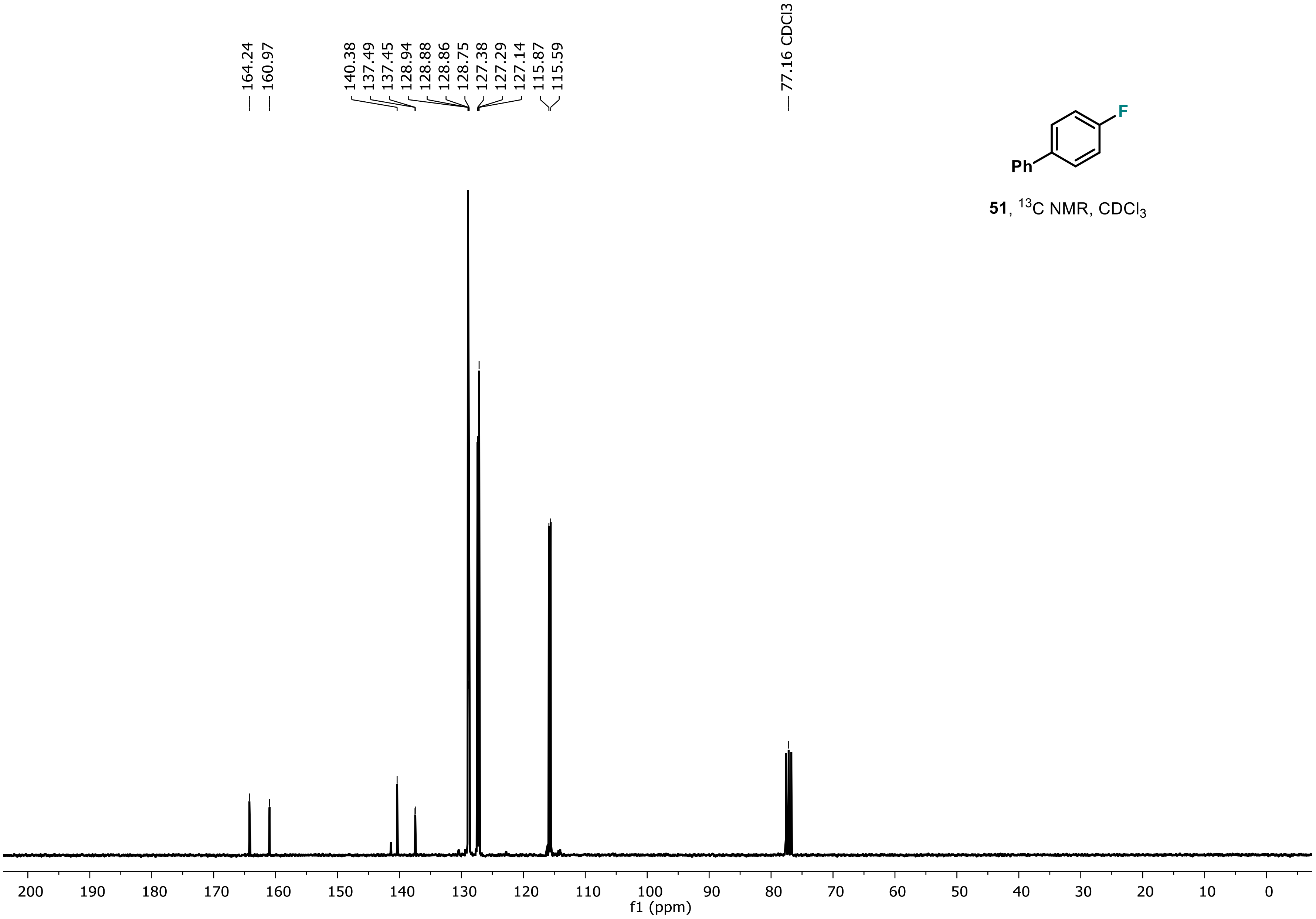

51, ^{13}C NMR, $CDCl_3$

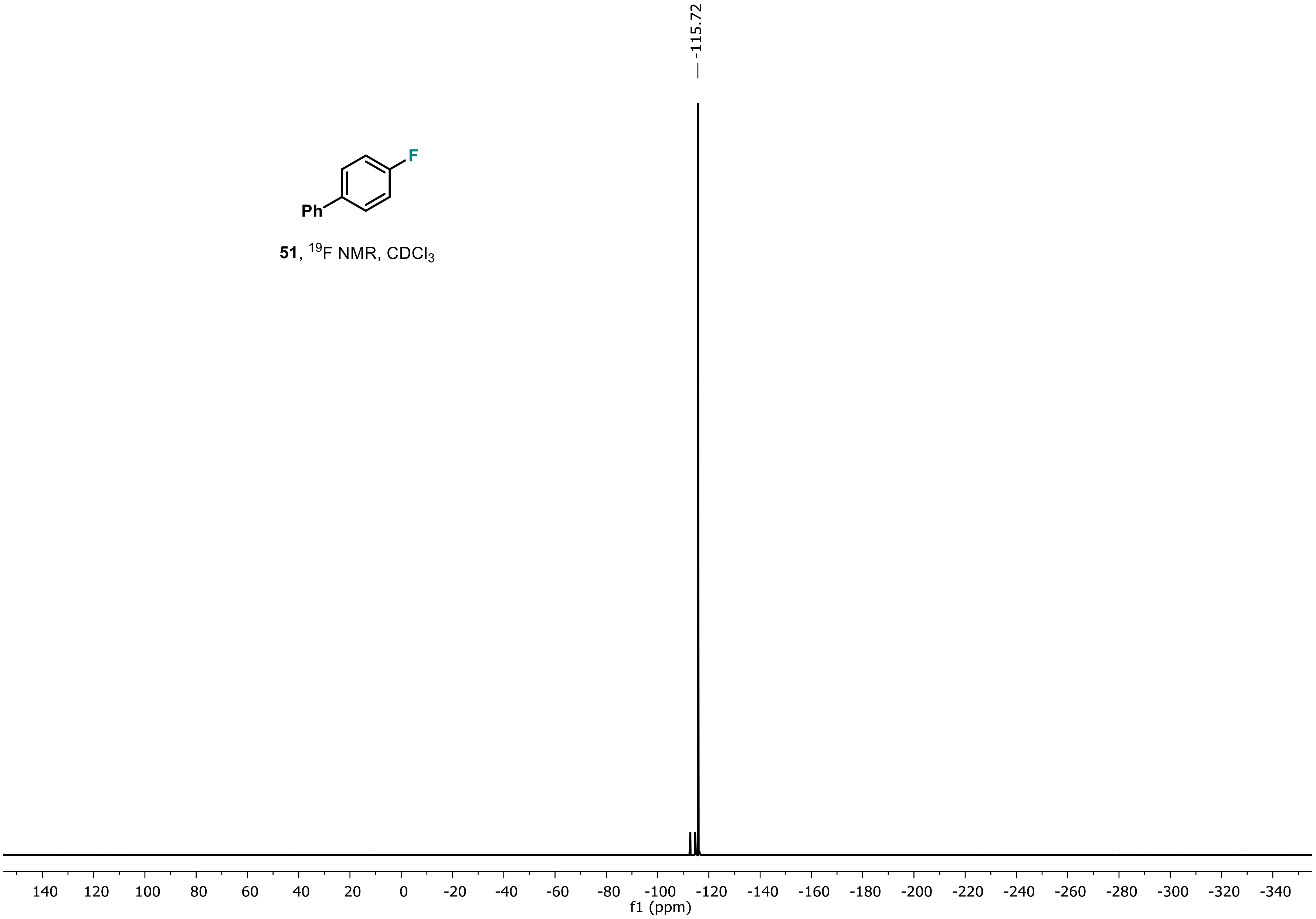

51, ^{19}F NMR, $CDCl_3$

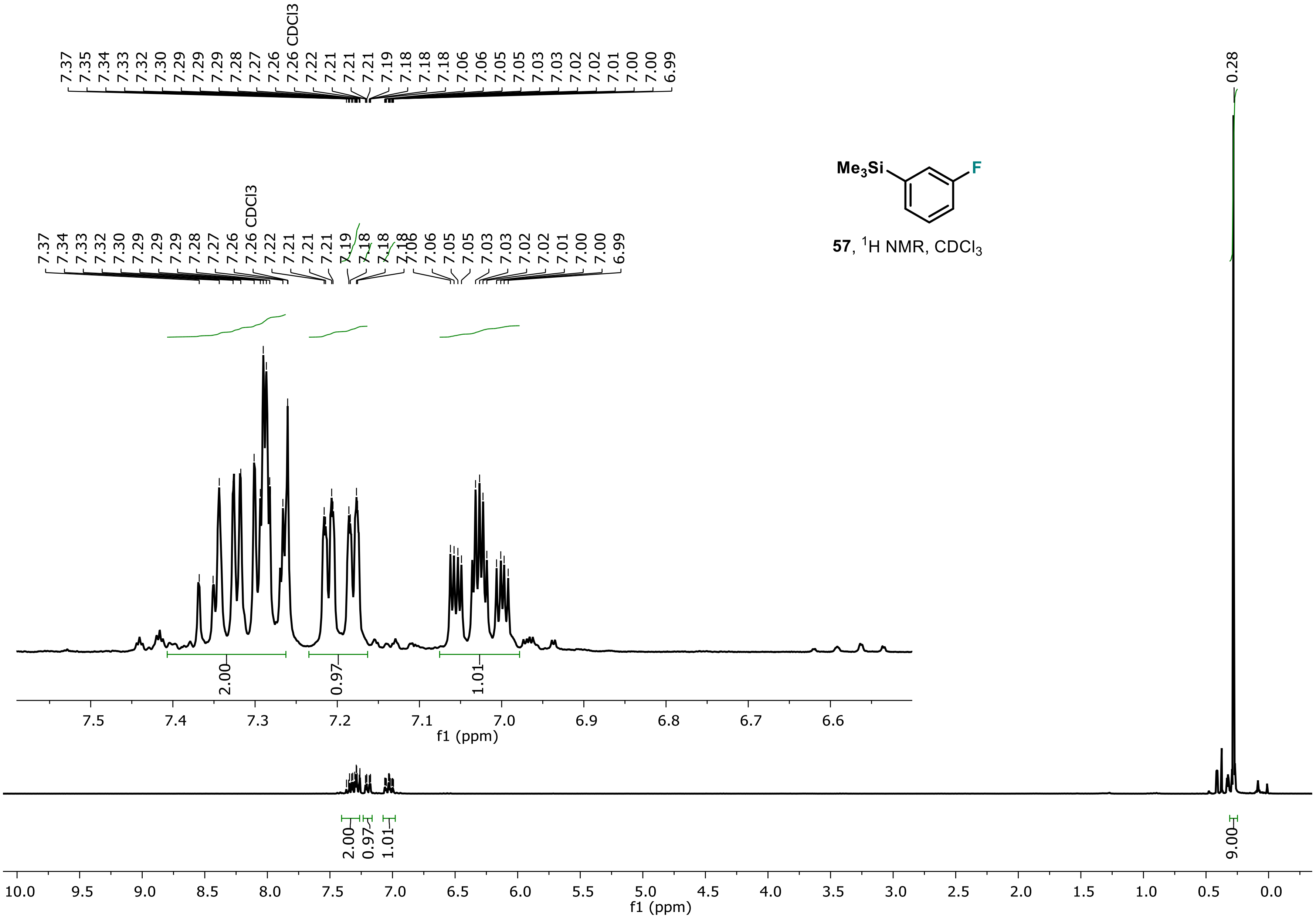

7.37 7.35 7.34 7.33 7.32 7.30 7.29 7.29 7.29 7.28 7.27 7.26 7.26 CDCl3 7.22 7.21 7.21 7.21 7.19 7.18 7.18 7.18 7.06 7.06 7.05 7.05 7.03 7.03 7.02 7.02 7.01 7.00 7.00 6.99
0.28
Me3Si
F
57, 1H NMR, CDCl3
2.00
0.97
1.01
9.00
f1 (ppm)

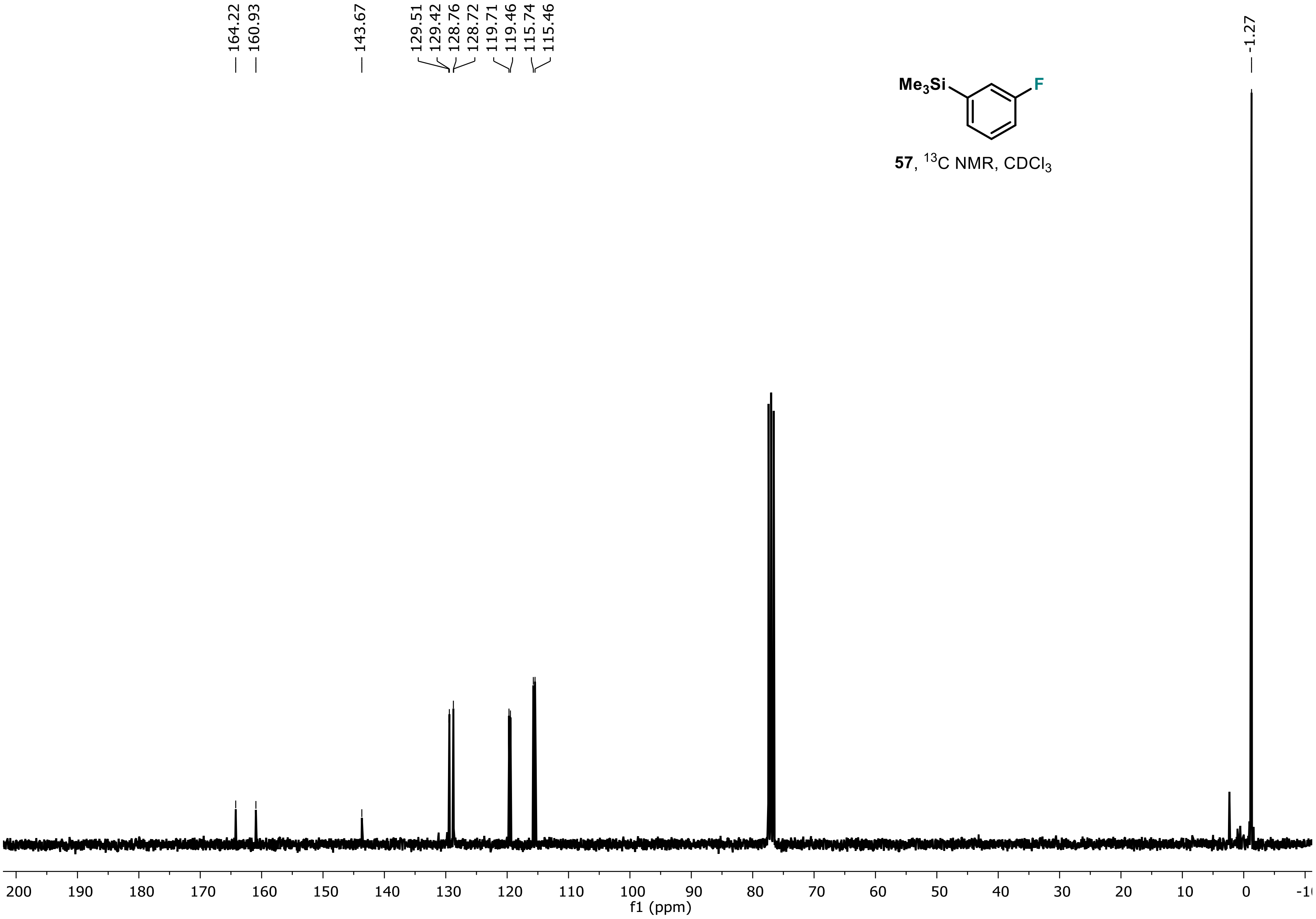

164.22
160.93
143.67
129.51
129.42
128.76
128.72
119.71
119.46
115.74
115.46
-1.27
Me3Si
F
57, 13C NMR, CDCl3
200 190 180 170 160 150 140 130 120 110 100 90 80 70 60 50 40 30 20 10 0
f1 (ppm)

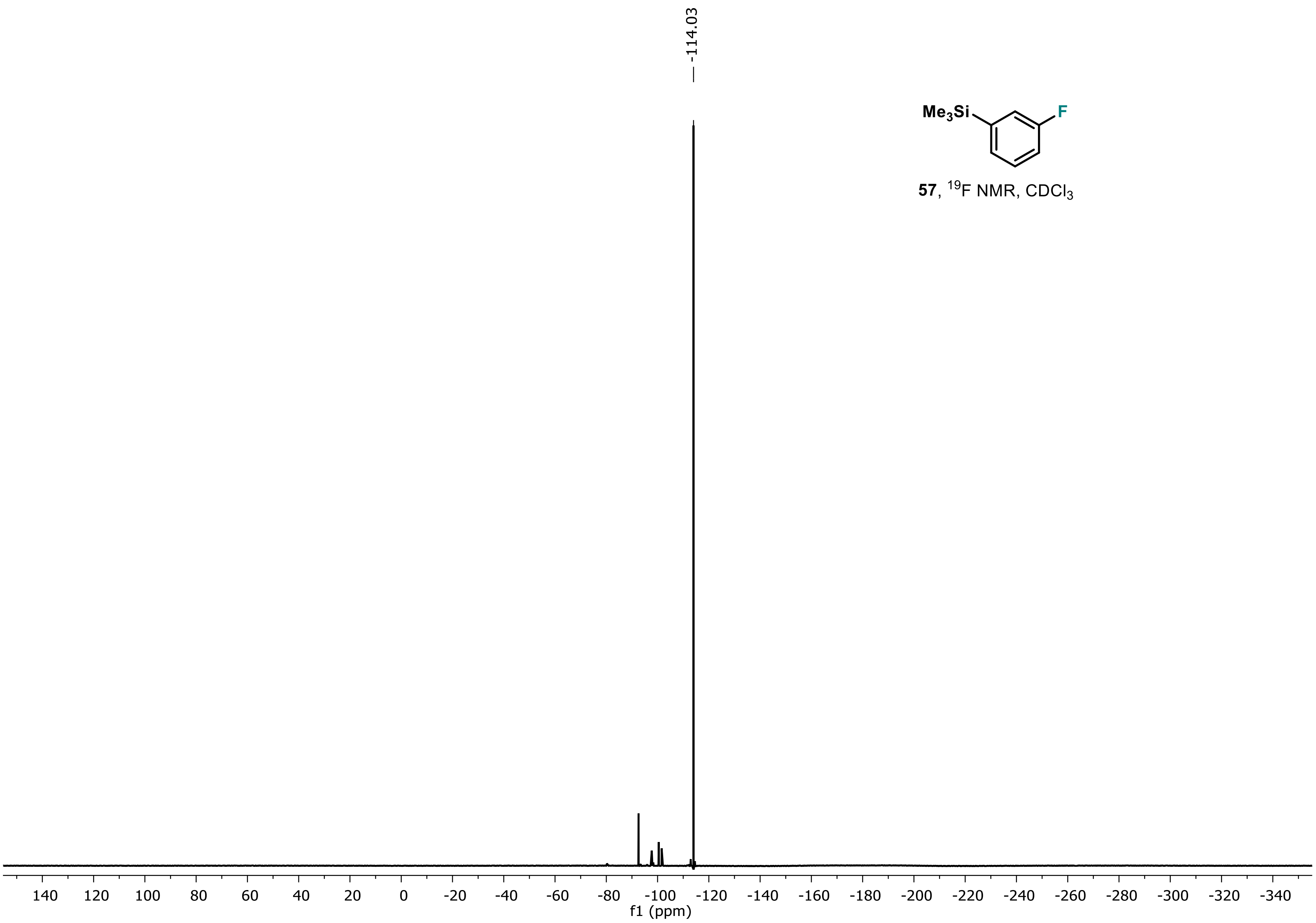
-114.03
Me3Si
F
57, 19F NMR, CDCl3
140 120 100 80 60 40 20 0 -20 -40 -60 -80 -100 -120 -140 -160 -180 -200 -220 -240 -260 -280 -300 -320 -340
f1 (ppm)

14. Crude ^{19}F NMR spectra for catalytic fluorination of arylboronic esters

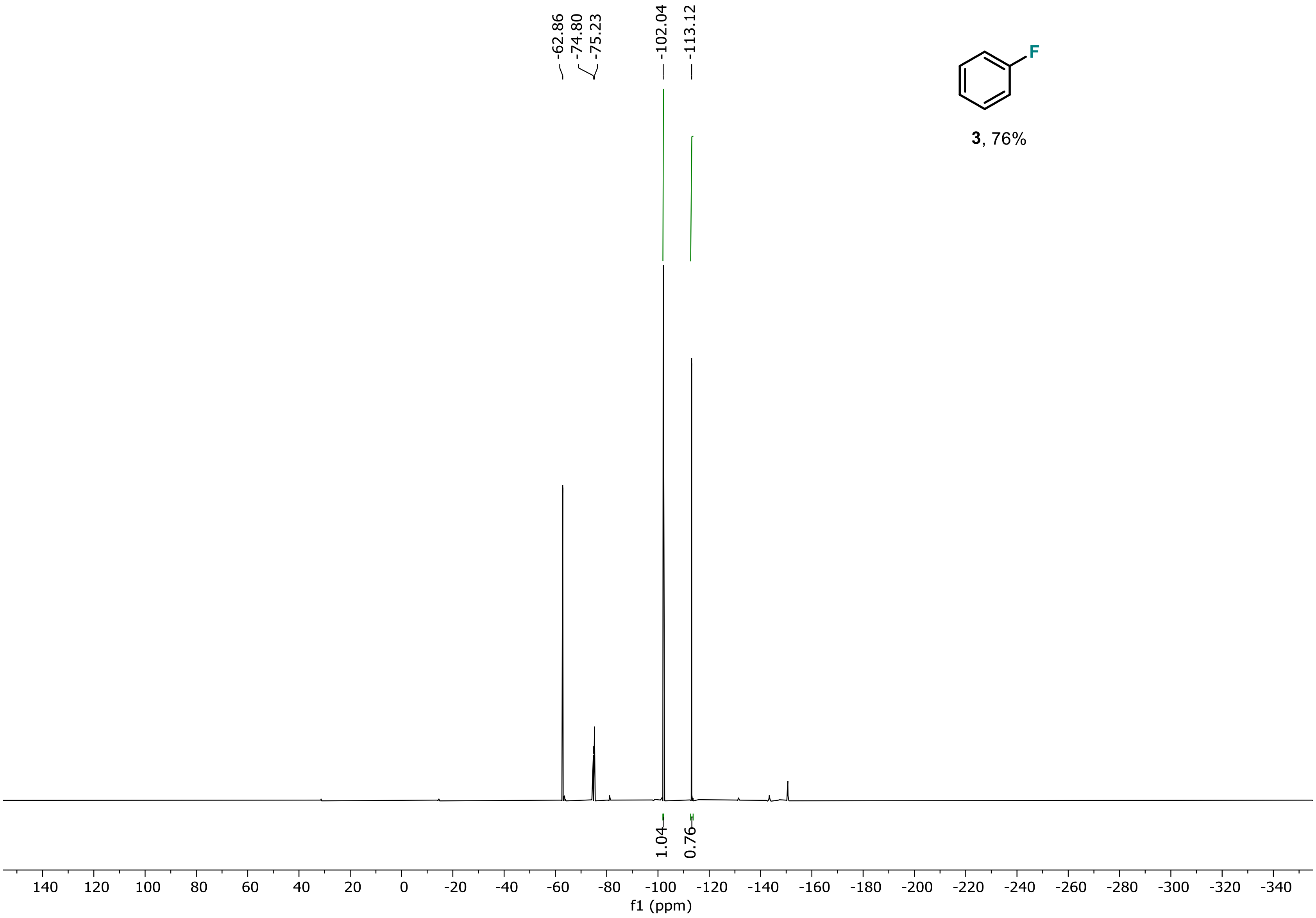

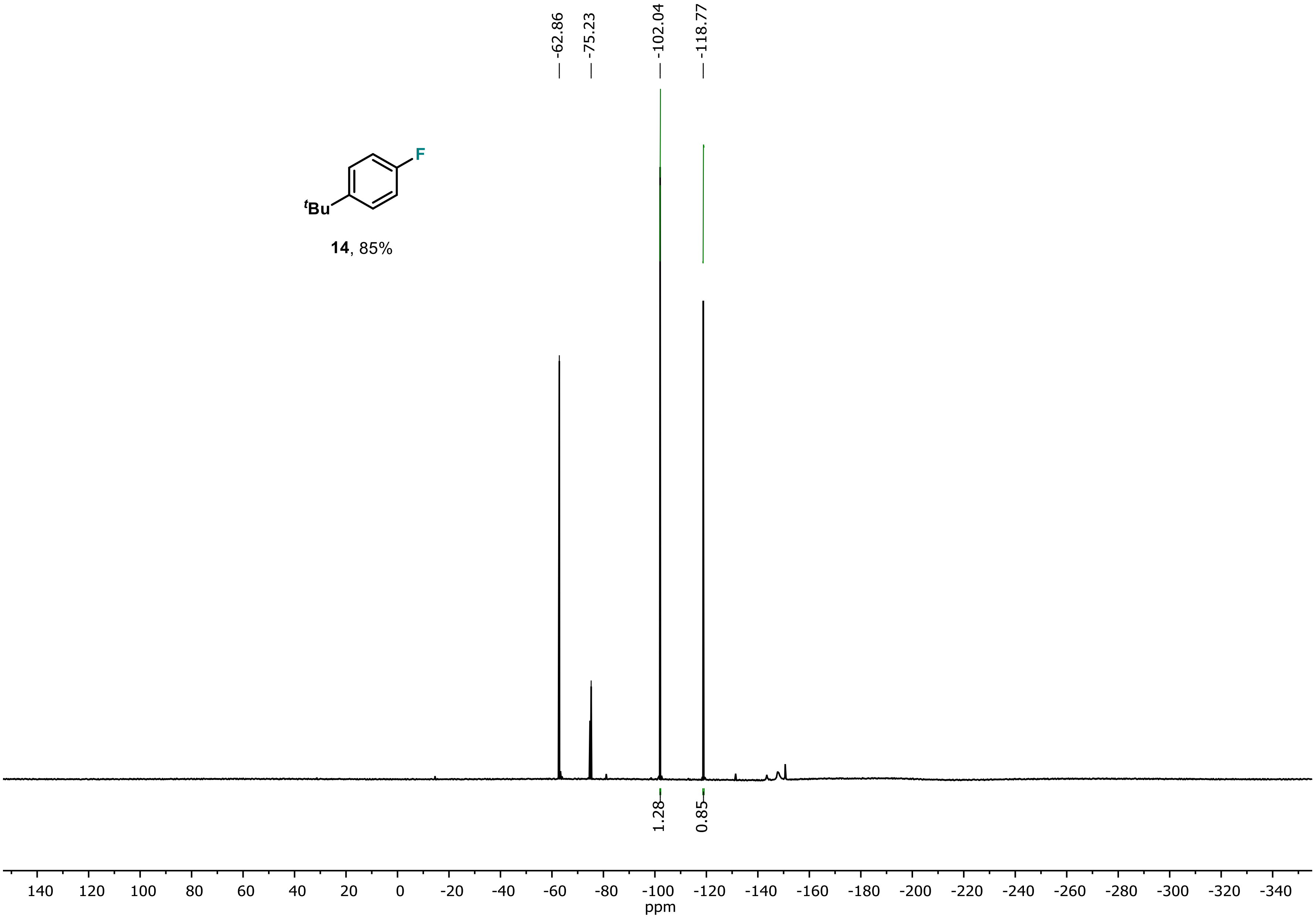

-62.86
-75.23
-102.04
-118.77
F
tBu
14, 85%
1.28
0.85
140 120 100 80 60 40 20 0 -20 -40 -60 -80 -100 -120 -140 -160 -180 -200 -220 -240 -260 -280 -300 -320 -340
ppm

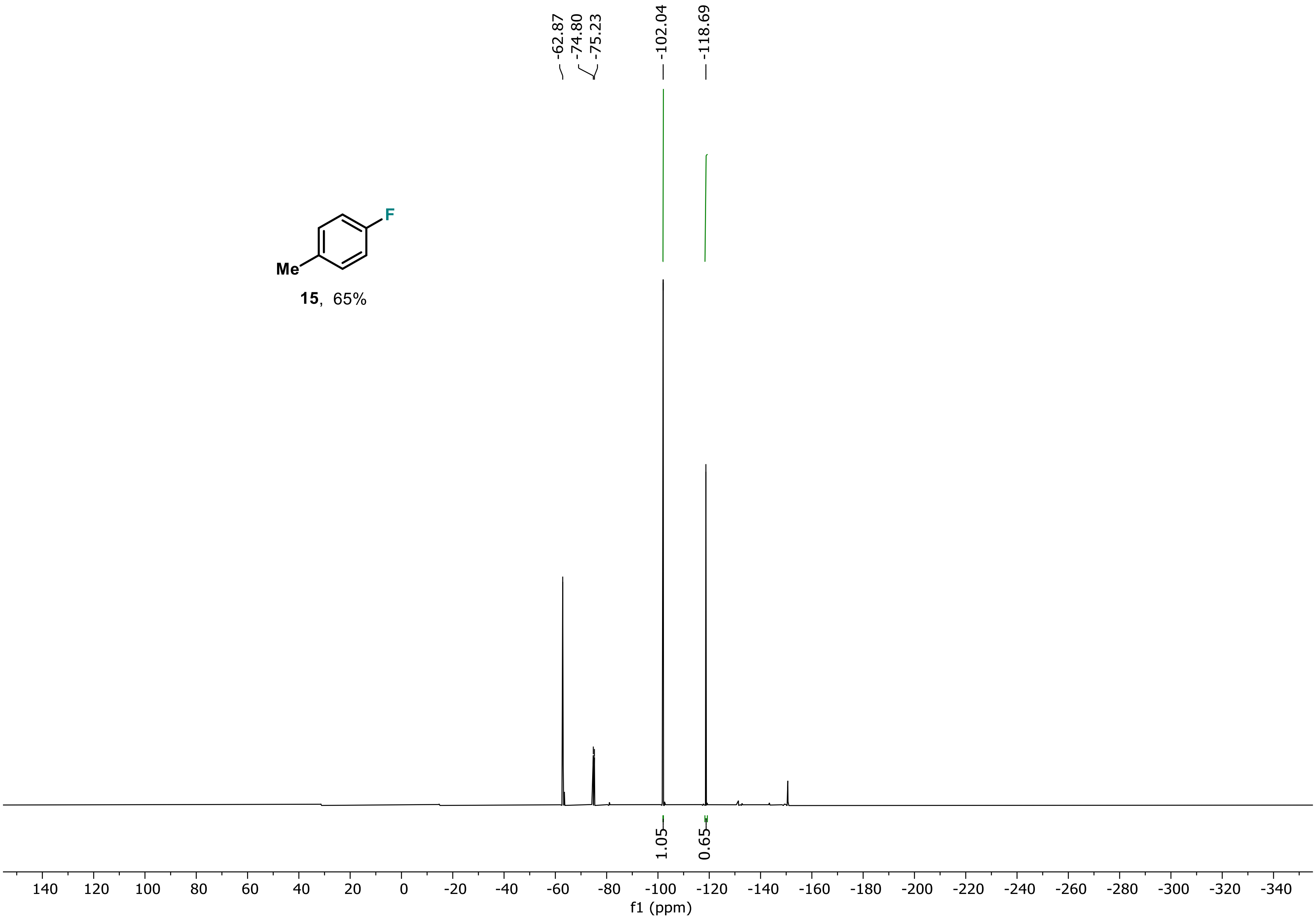
-62.87
-74.80
-75.23
-102.04
-118.69
Me
F
1.05
0.65
140 120 100 80 60 40 20 0 -20 -40 -60 -80 -100 -120 -140 -160 -180 -200 -220 -240 -260 -280 -300 -320 -340
f1 (ppm)

15, 65%

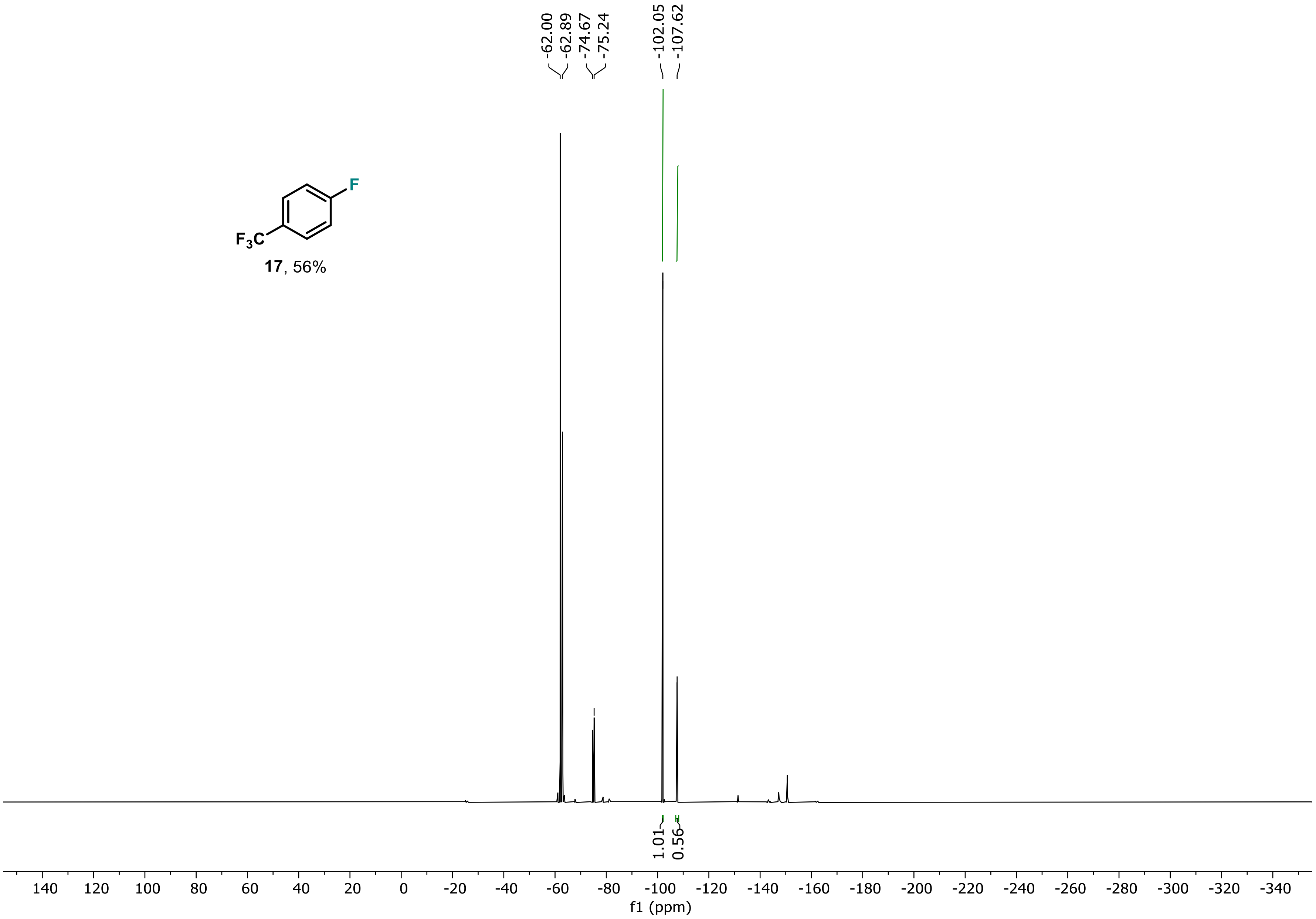
F
F3C
-62.00
-62.89
-74.67
-75.24
-102.05
-107.62
1.01
0.56
140 120 100 80 60 40 20 0 -20 -40 -60 -80 -100 -120 -140 -160 -180 -200 -220 -240 -260 -280 -300 -320 -340
f1 (ppm)
17, 56%

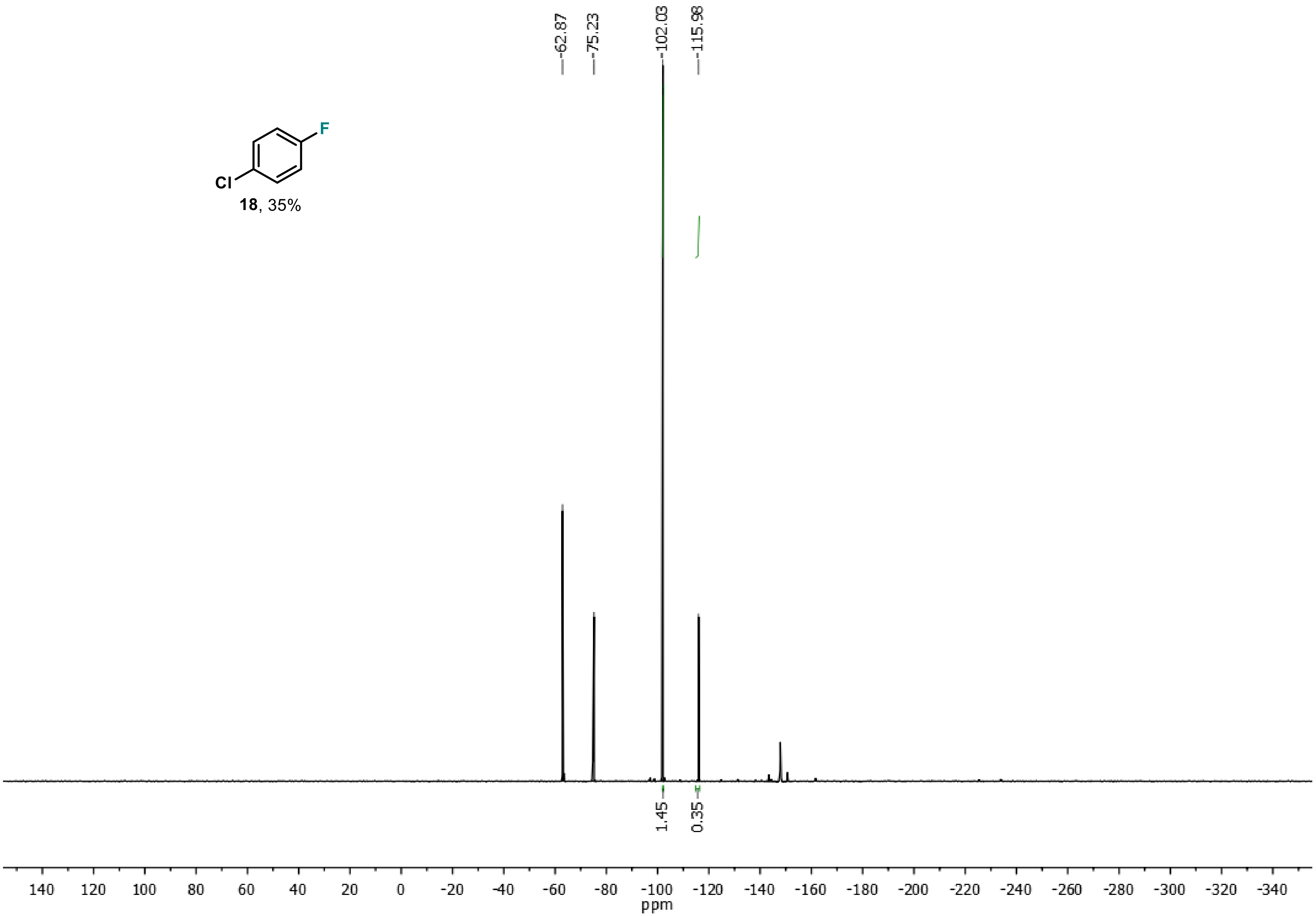

-62.87
-75.23
-102.03
-115.98
1.45
0.35
140 120 100 80 60 40 20 0 -20 -40 -60 -80 -100 -120 -140 -160 -180 -200 -220 -240 -260 -280 -300 -320 -340
ppm
F
Cl

18, 35%

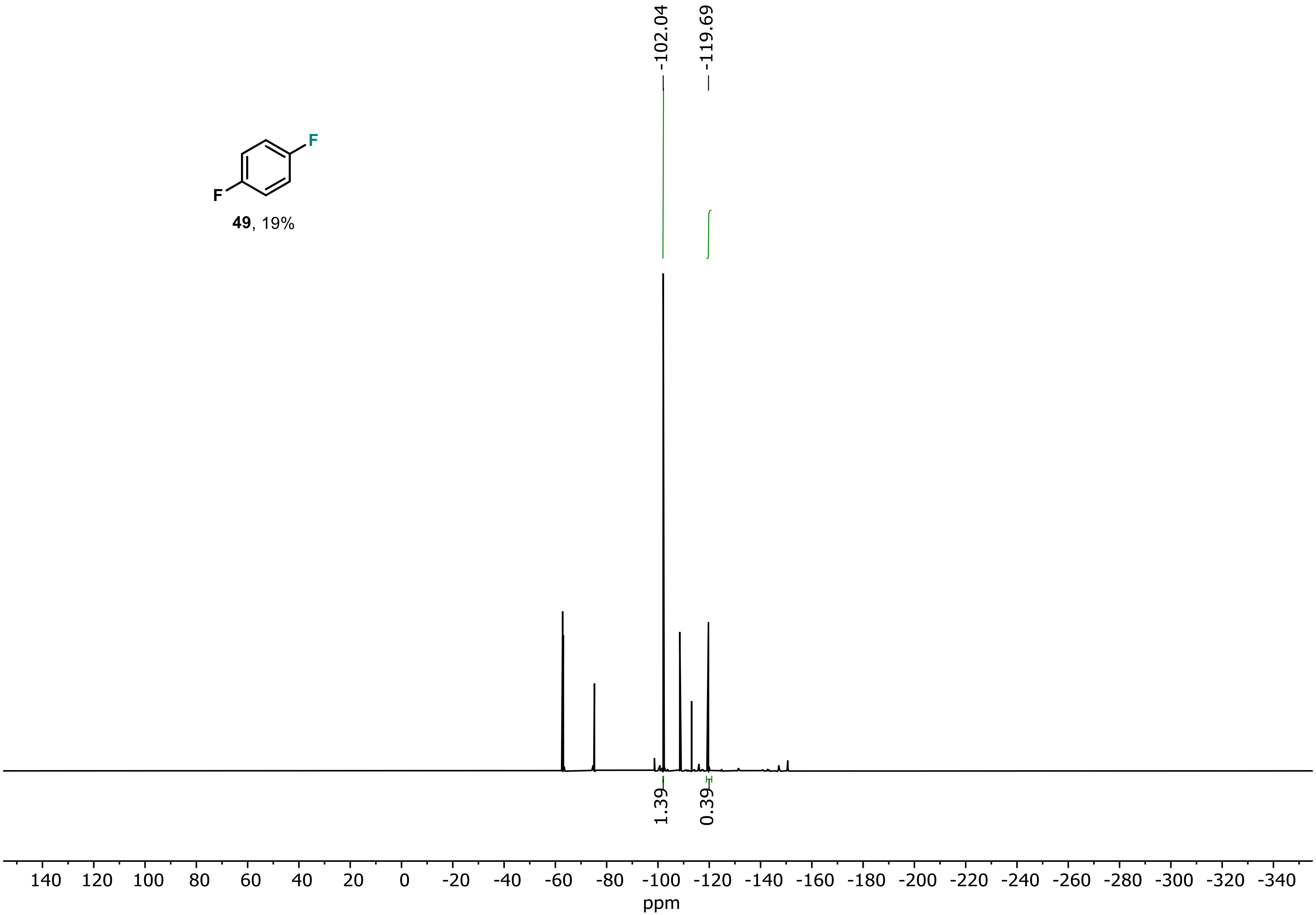

-102.04
-119.69
1.39
0.39
140 120 100 80 60 40 20 0 -20 -40 -60 -80 -100 -120 -140 -160 -180 -200 -220 -240 -260 -280 -300 -320 -340
ppm

49, 19%

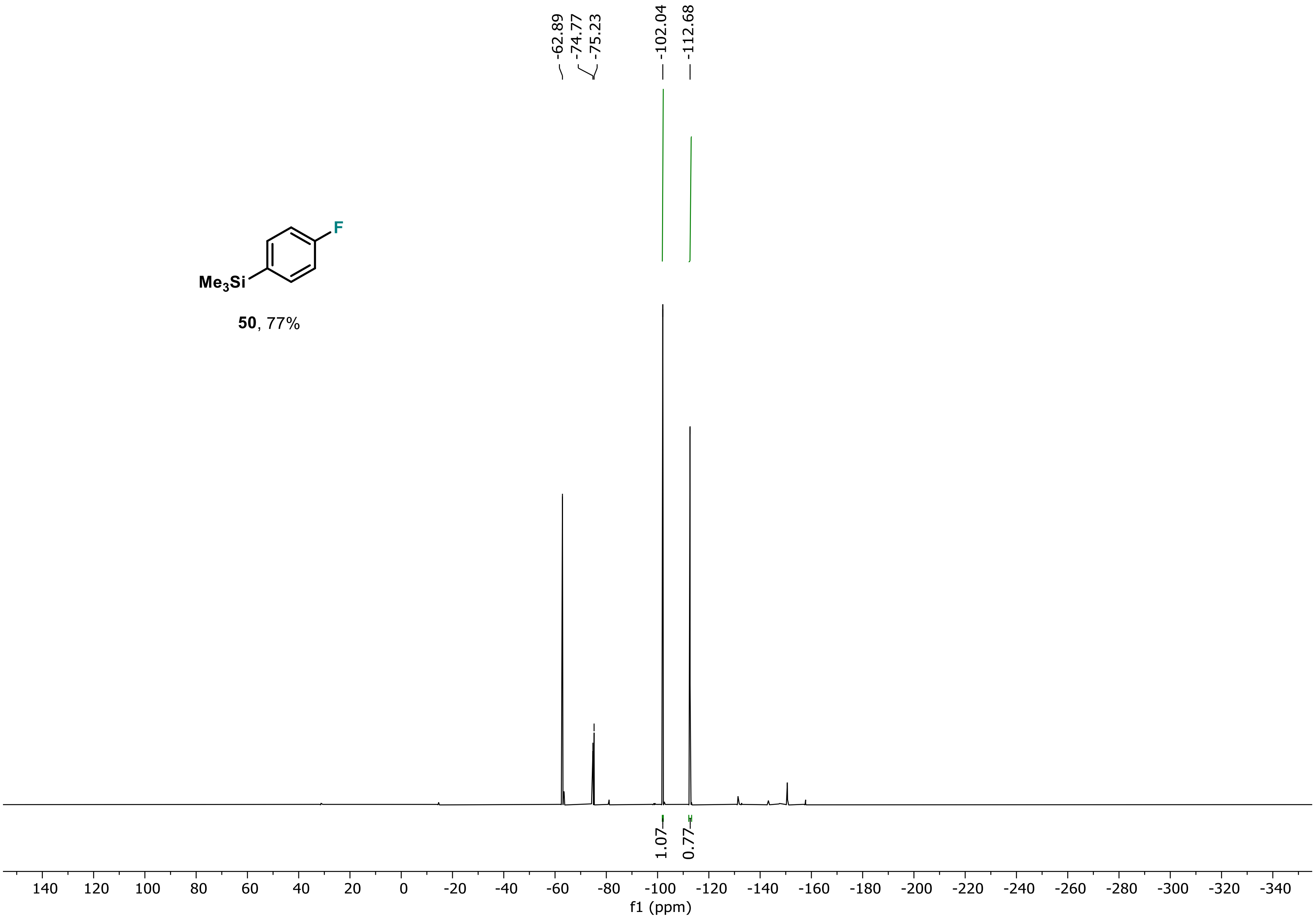
Me3Si
F
50, 77%
-62.89
-74.77
-75.23
-102.04
-112.68
1.07
0.77
140 120 100 80 60 40 20 0 -20 -40 -60 -80 -100 -120 -140 -160 -180 -200 -220 -240 -260 -280 -300 -320 -340
f1 (ppm)

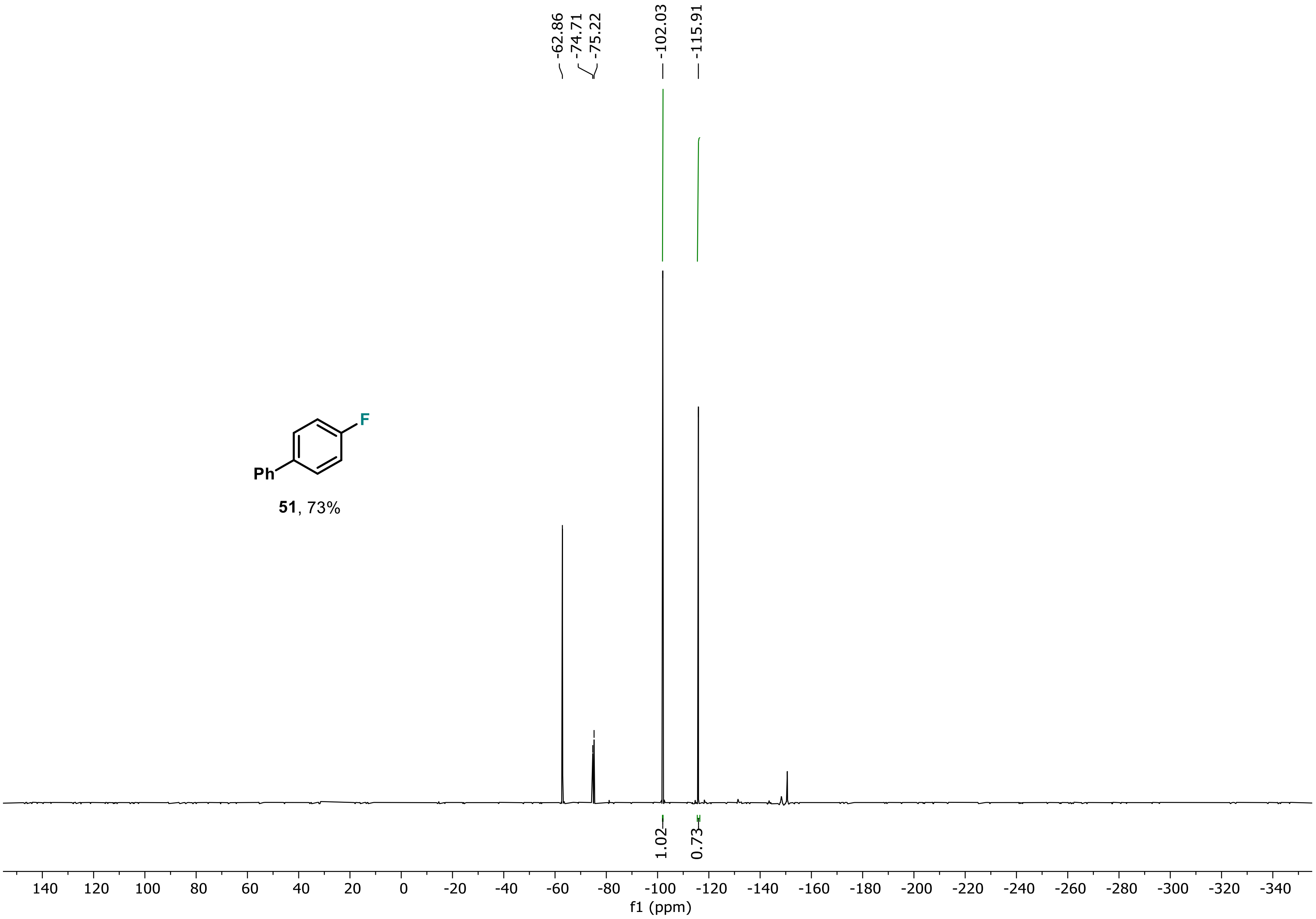

-62.86
-74.71
-75.22
-102.03
-115.91
1.02
0.73
140 120 100 80 60 40 20 0 -20 -40 -60 -80 -100 -120 -140 -160 -180 -200 -220 -240 -260 -280 -300 -320 -340
f1 (ppm)
F
Ph

51, 73%

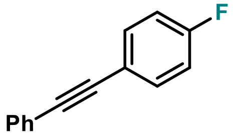
F
Ph

52, 49%

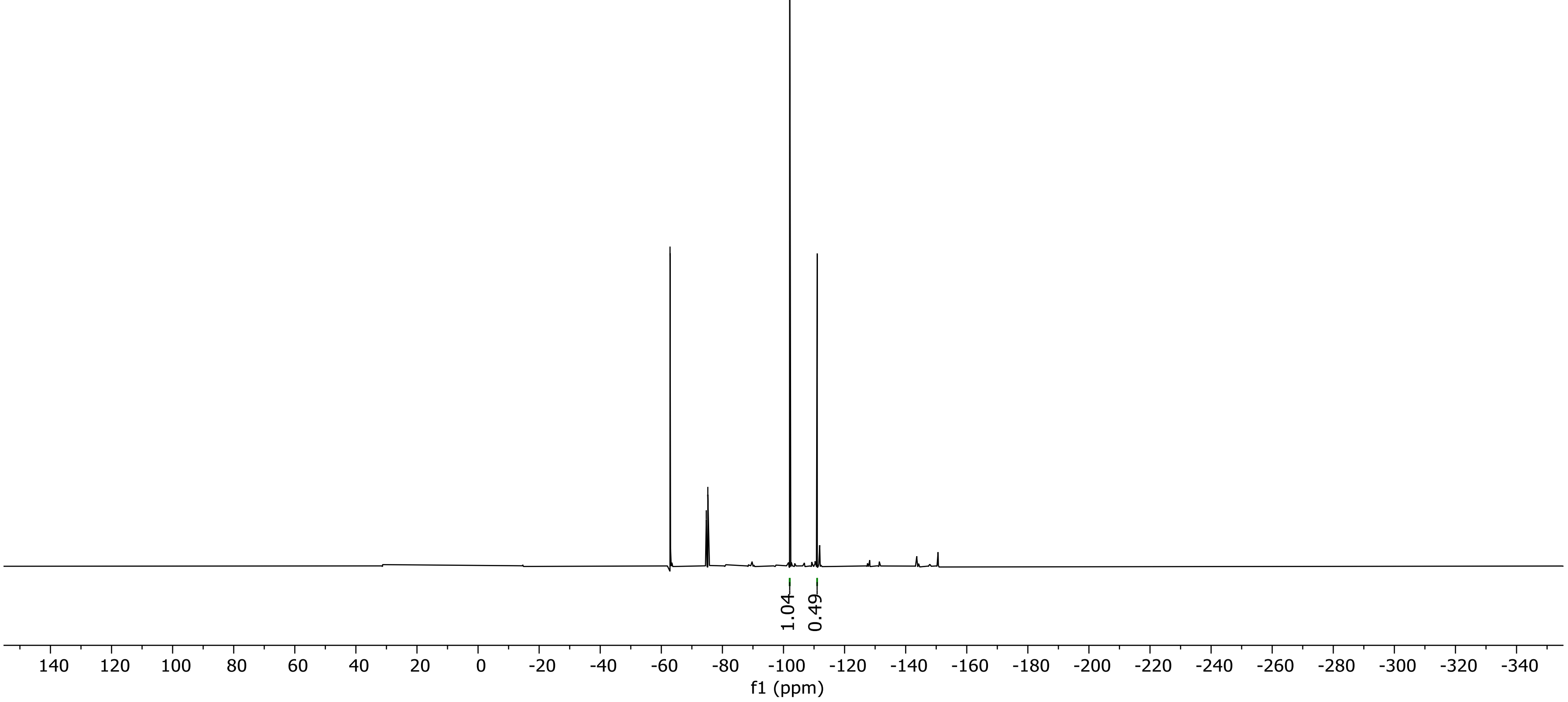
-62.85
-74.72
-75.22
-102.03
-111.03
1.04
0.49
140 120 100 80 60 40 20 0 -20 -40 -60 -80 -100 -120 -140 -160 -180 -200 -220 -240 -260 -280 -300 -320 -340
f1 (ppm)

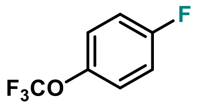
F
F3CO

53, 18%

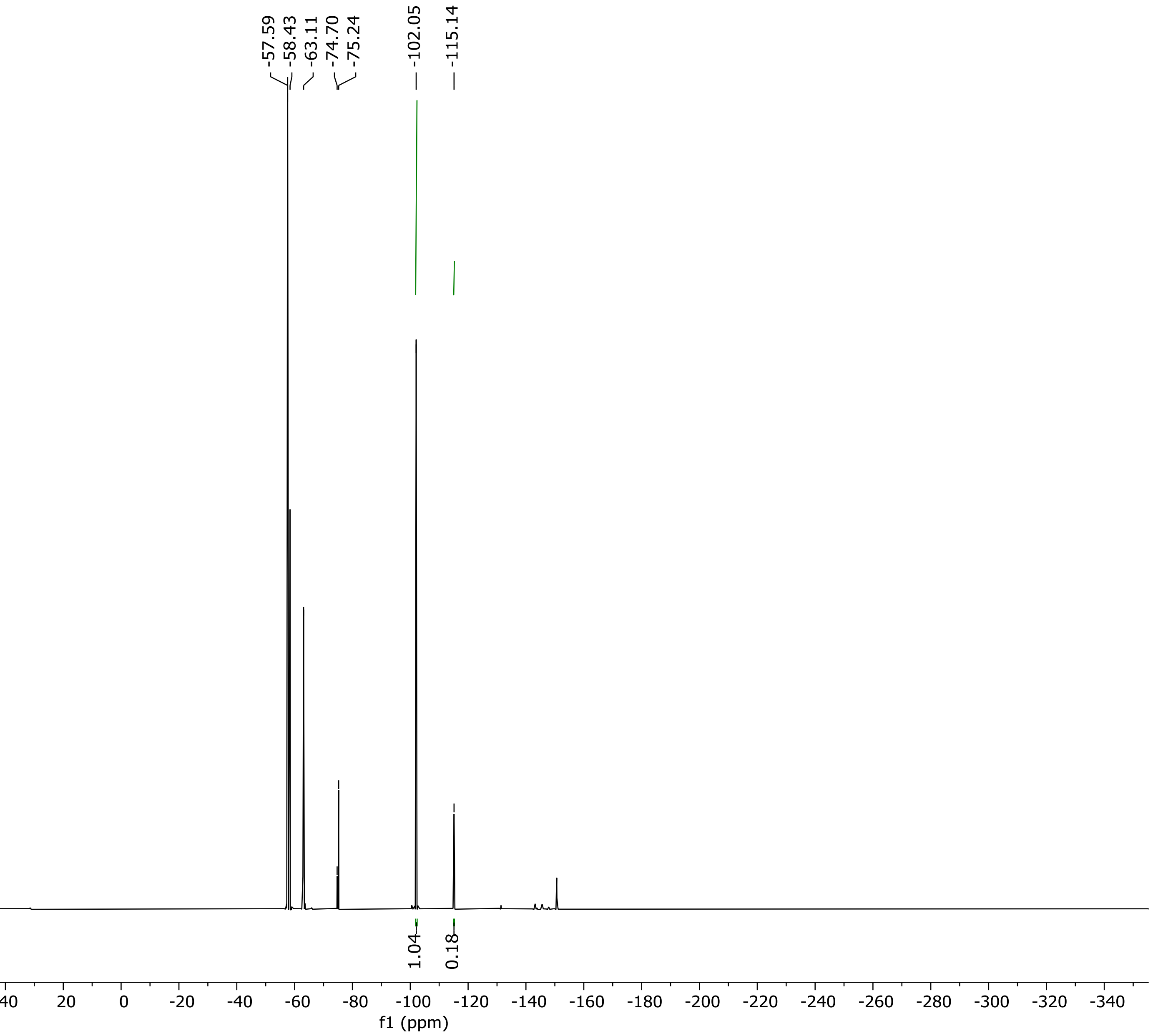
-57.59
-58.43
-63.11
-74.70
-75.24
-102.05
-115.14
1.04
0.18
140
120
100
80
60
40
20
0
-20
-40
-60
-80
-100
-120
-140
-160
-180
-200
-220
-240
-260
-280
-300
-320
-340
f1 (ppm)

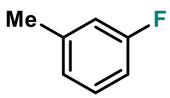

54, 72%

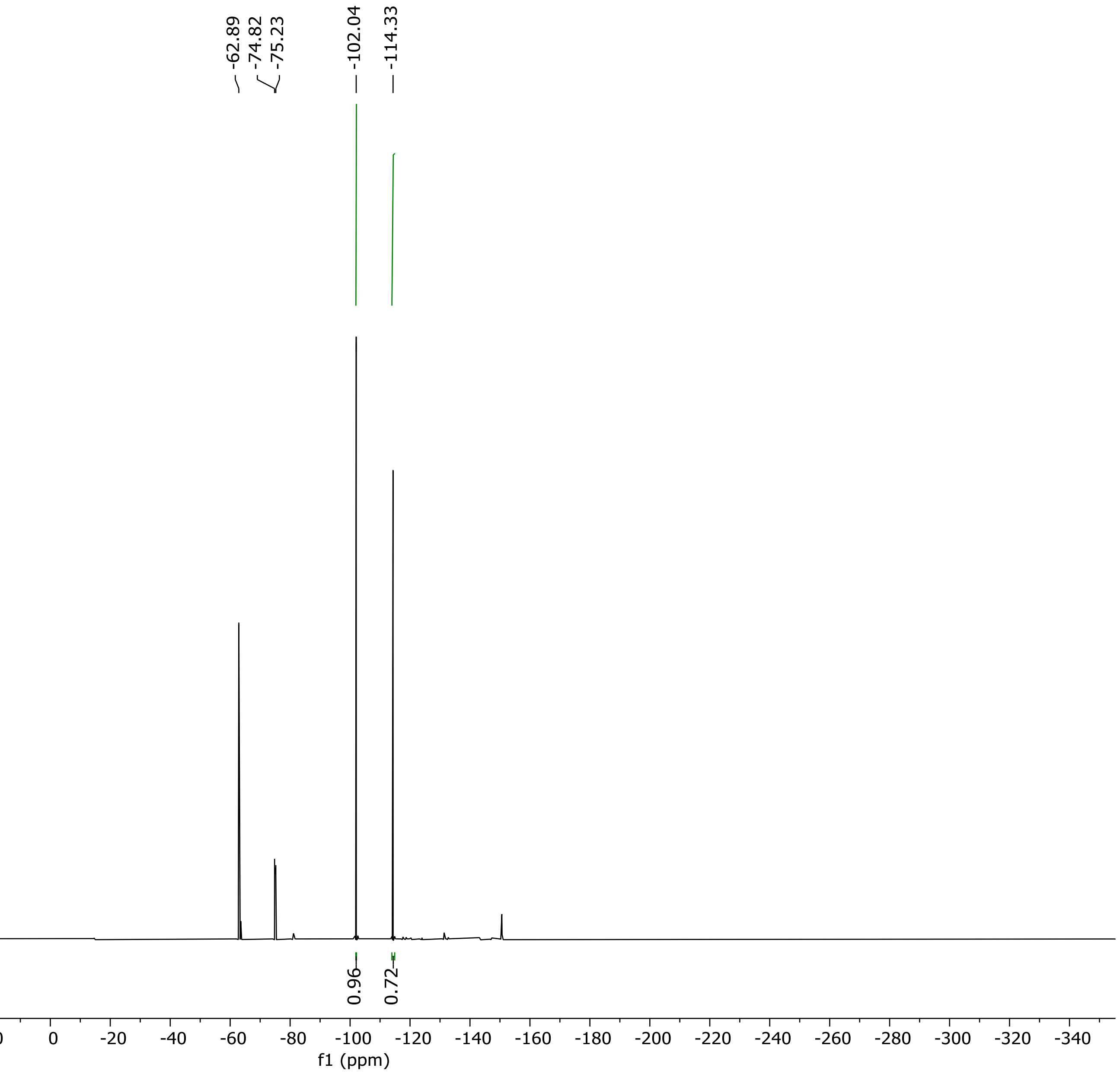

-62.89
-74.82
-75.23
-102.04
-114.33
0.96
0.72
140 120 100 80 60 40 20 0 -20 -40 -60 -80 -100 -120 -140 -160 -180 -200 -220 -240 -260 -280 -300 -320 -340
f1 (ppm)

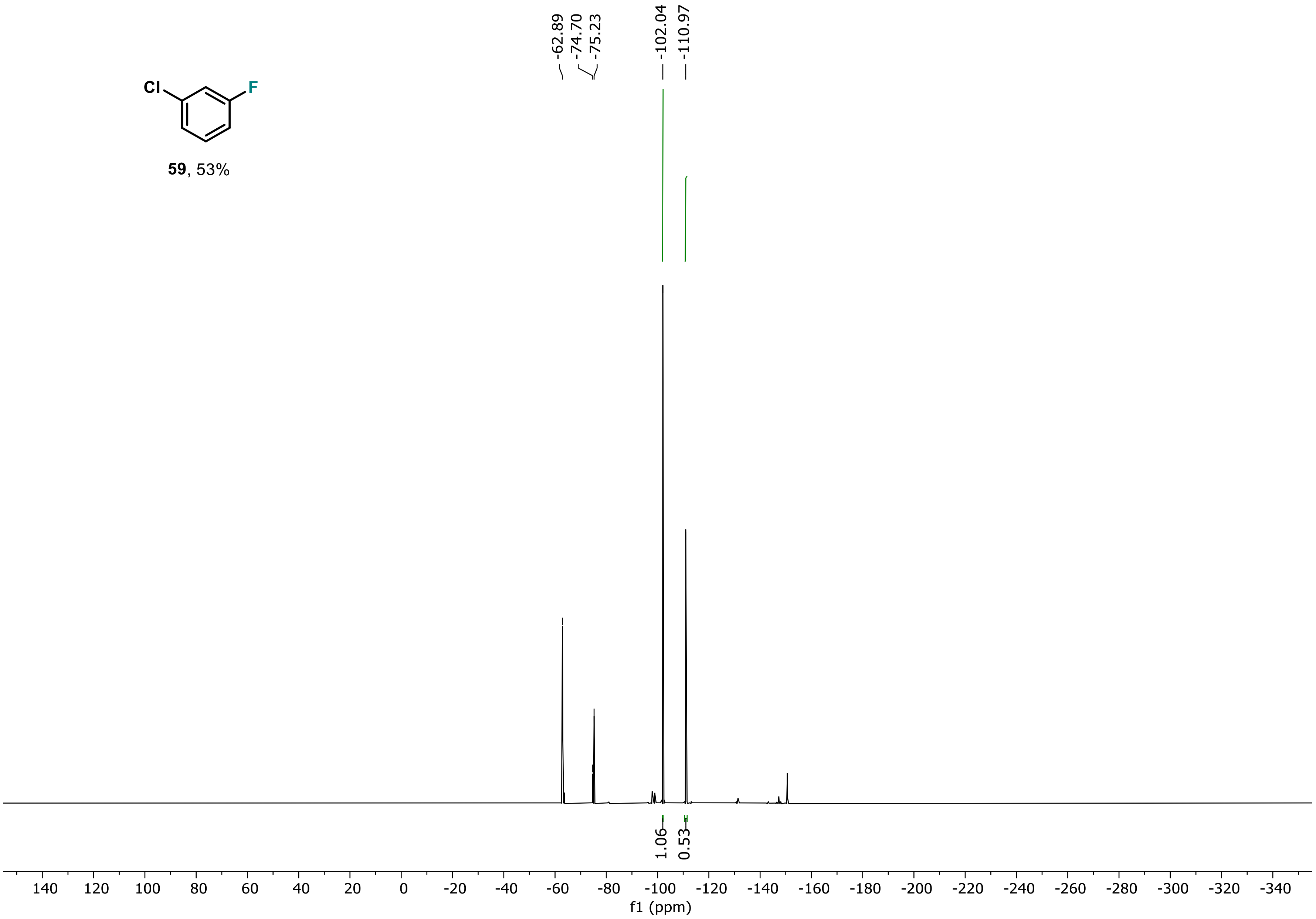

-62.89
-74.70
-75.23
-102.04
-110.97
1.06
0.53
140 120 100 80 60 40 20 0 -20 -40 -60 -80 -100 -120 -140 -160 -180 -200 -220 -240 -260 -280 -300 -320 -340
f1 (ppm)

59, 53%

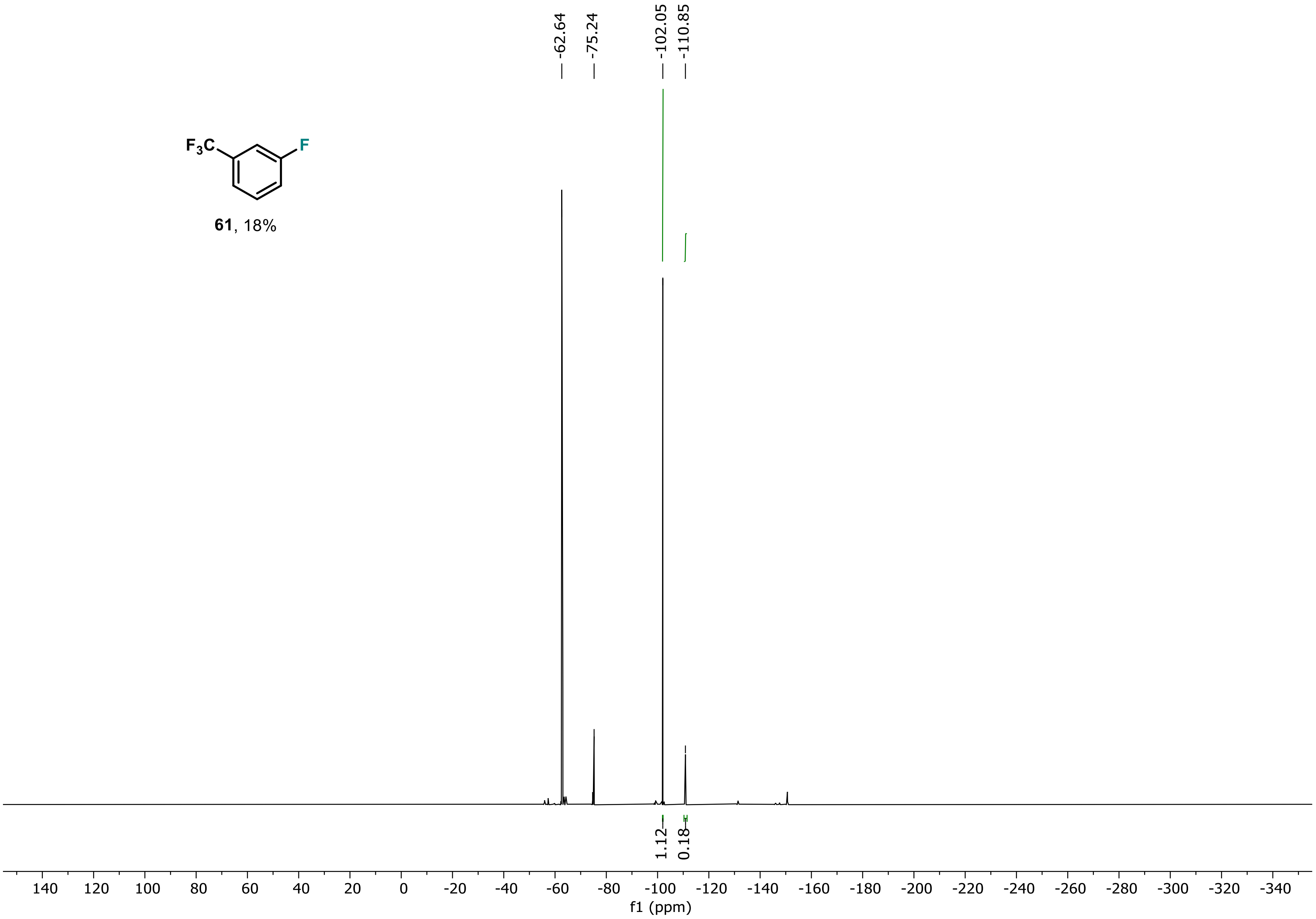
F3C
F
61, 18%
-62.64
-75.24
-102.05
-110.85
1.12
0.18
140 120 100 80 60 40 20 0 -20 -40 -60 -80 -100 -120 -140 -160 -180 -200 -220 -240 -260 -280 -300 -320 -340
f1 (ppm)

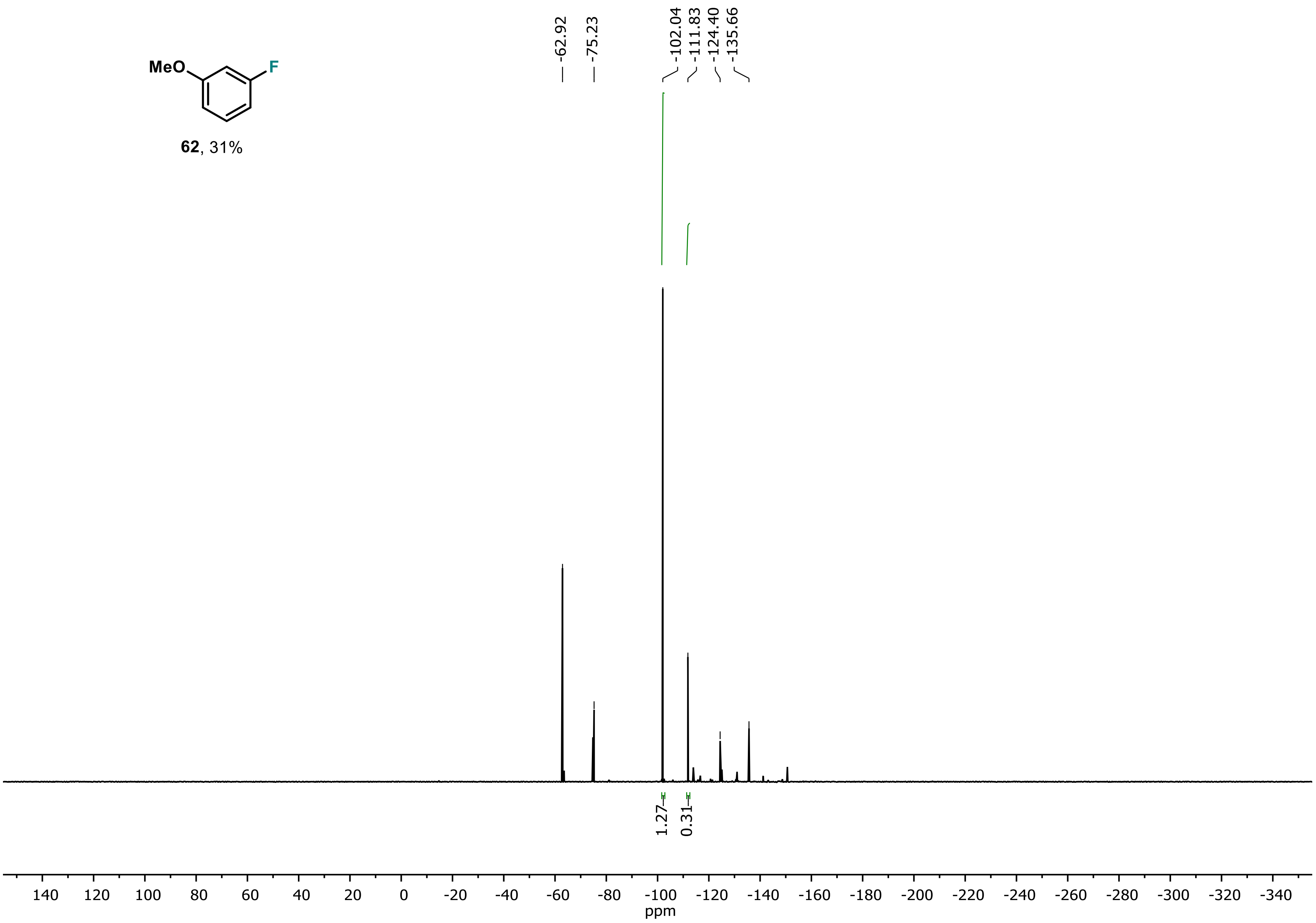

MeO
F
-62.92
-75.23
-102.04
-111.83
-124.40
-135.66
1.27
0.31
140 120 100 80 60 40 20 0 -20 -40 -60 -80 -100 -120 -140 -160 -180 -200 -220 -240 -260 -280 -300 -320 -340
ppm

62, 31%

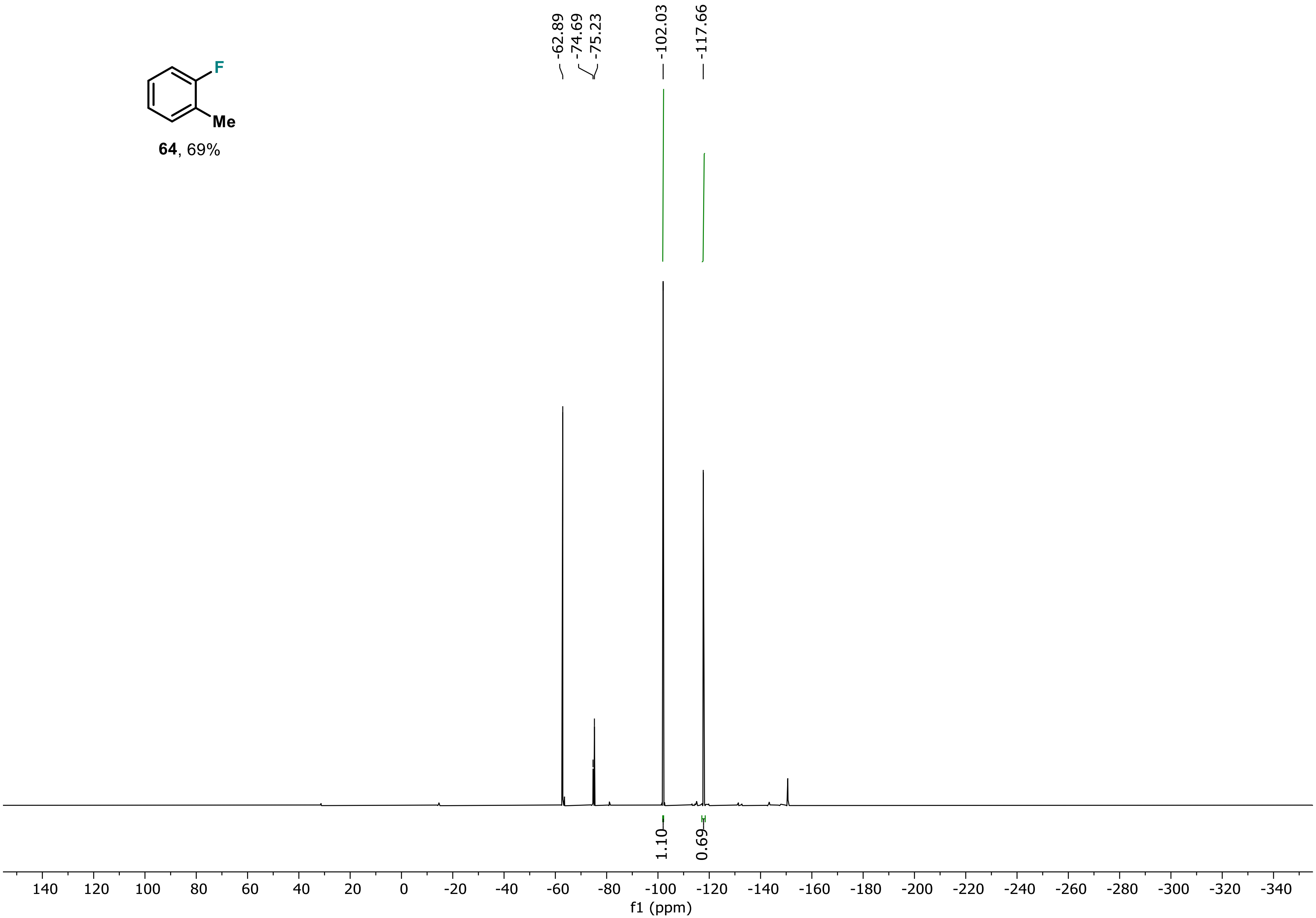

64, 69%
-62.89
-74.69
-75.23
-102.03
-117.66
1.10
0.69
140 120 100 80 60 40 20 0 -20 -40 -60 -80 -100 -120 -140 -160 -180 -200 -220 -240 -260 -280 -300 -320 -340
f1 (ppm)

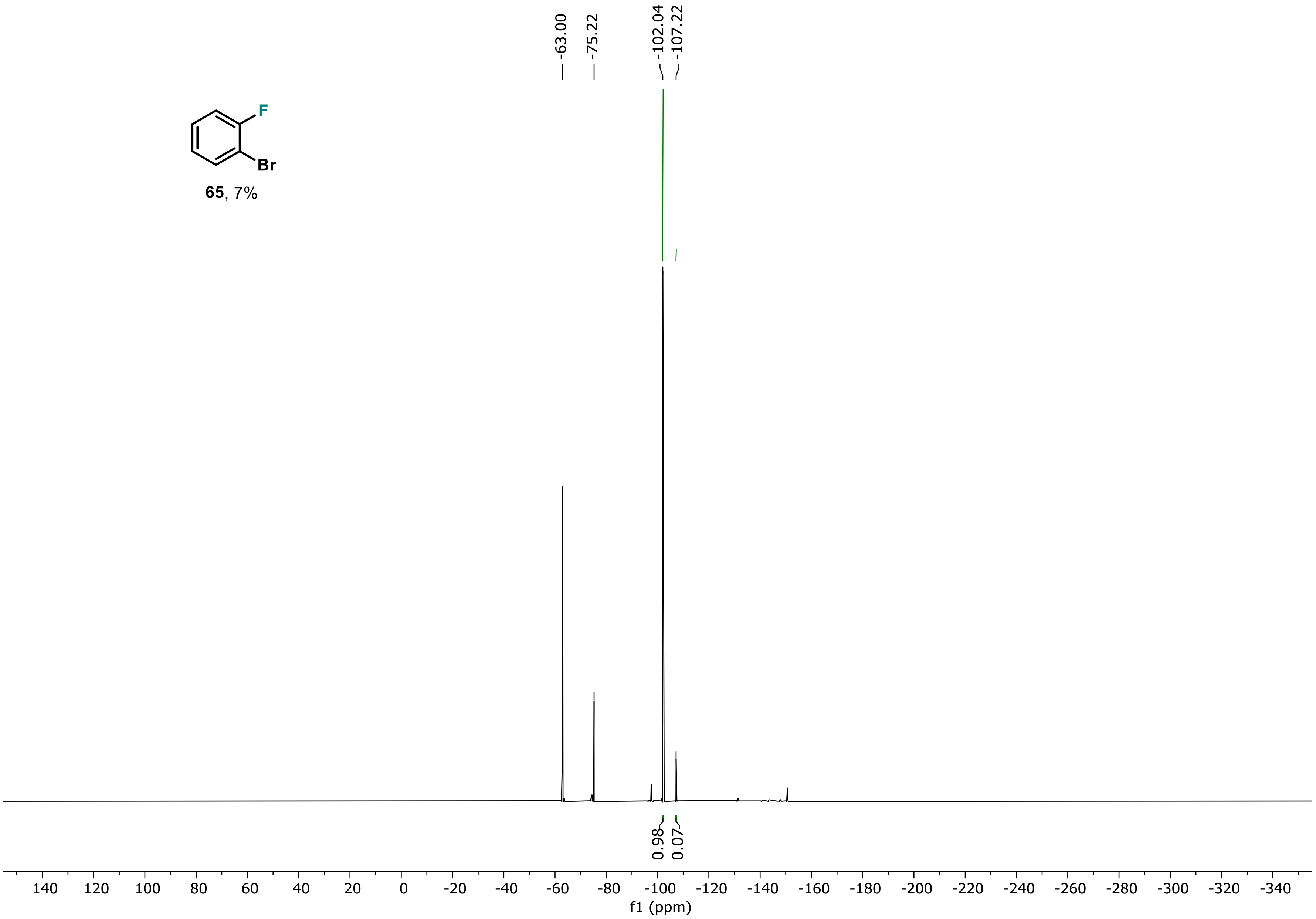

F
Br
65, 7%
-63.00
-75.22
-102.04
-107.22
0.98
0.07
140 120 100 80 60 40 20 0 -20 -40 -60 -80 -100 -120 -140 -160 -180 -200 -220 -240 -260 -280 -300 -320 -340
f1 (ppm)

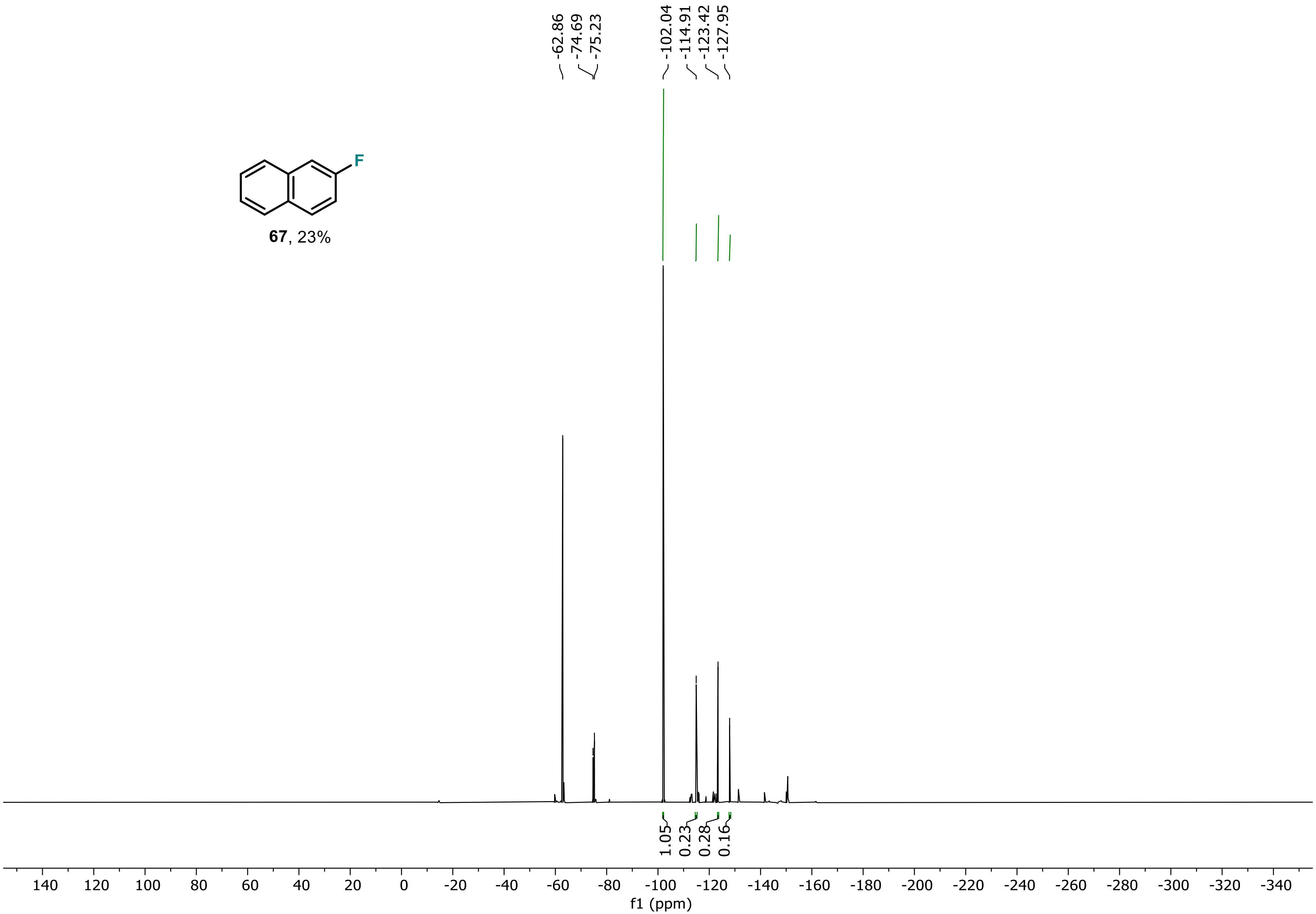
F
67, 23%
-62.86
-74.69
-75.23
-102.04
-114.91
-123.42
-127.95
1.05
0.23
0.28
0.16
140 120 100 80 60 40 20 0 -20 -40 -60 -80 -100 -120 -140 -160 -180 -200 -220 -240 -260 -280 -300 -320 -340
f1 (ppm)

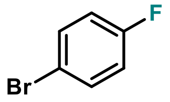

69, 51%

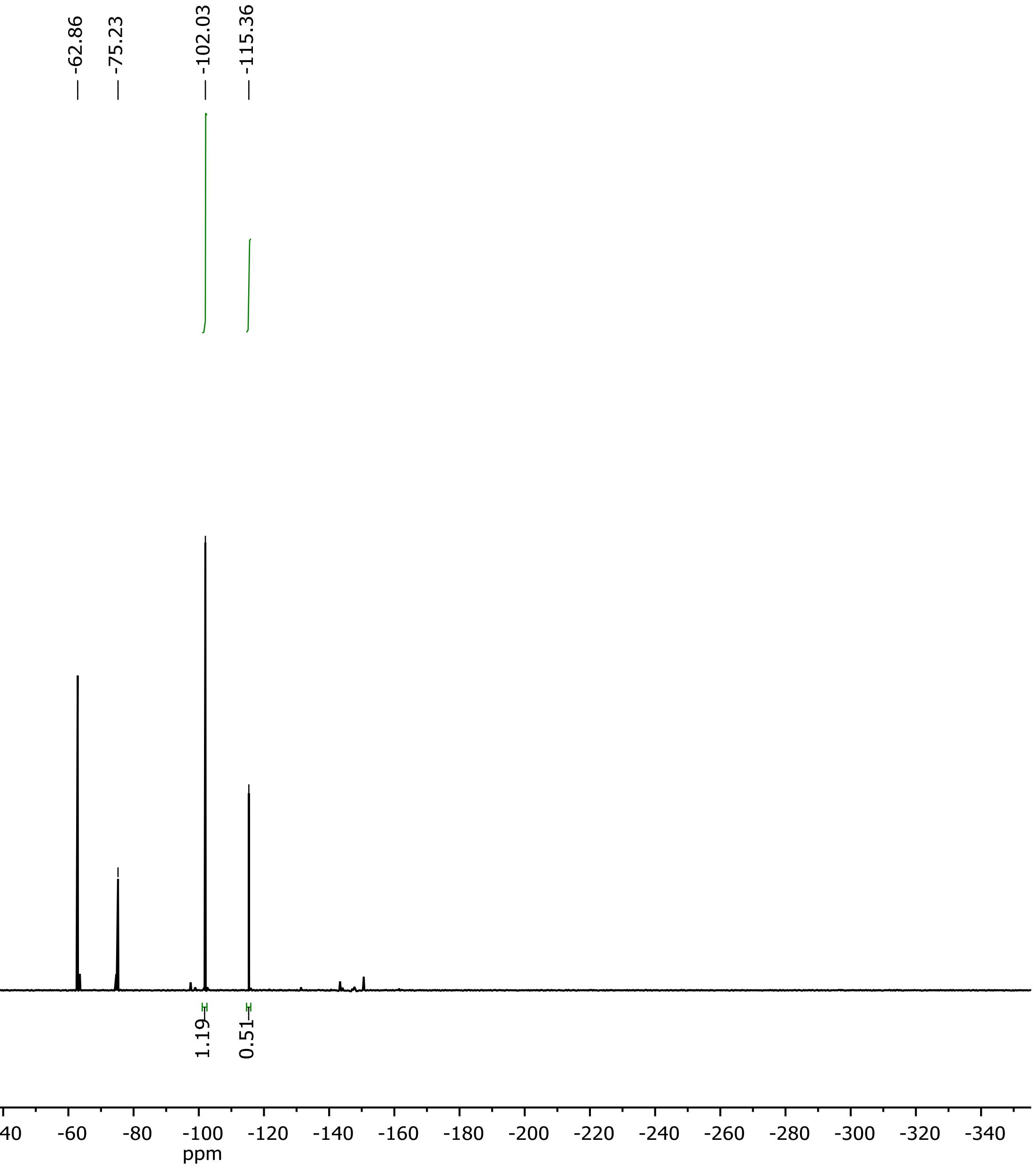

-62.86
-75.23
-102.03
-115.36
1.19
0.51
140 120 100 80 60 40 20 0 -20 -40 -60 -80 -100 -120 -140 -160 -180 -200 -220 -240 -260 -280 -300 -320 -340
ppm

70, 43%

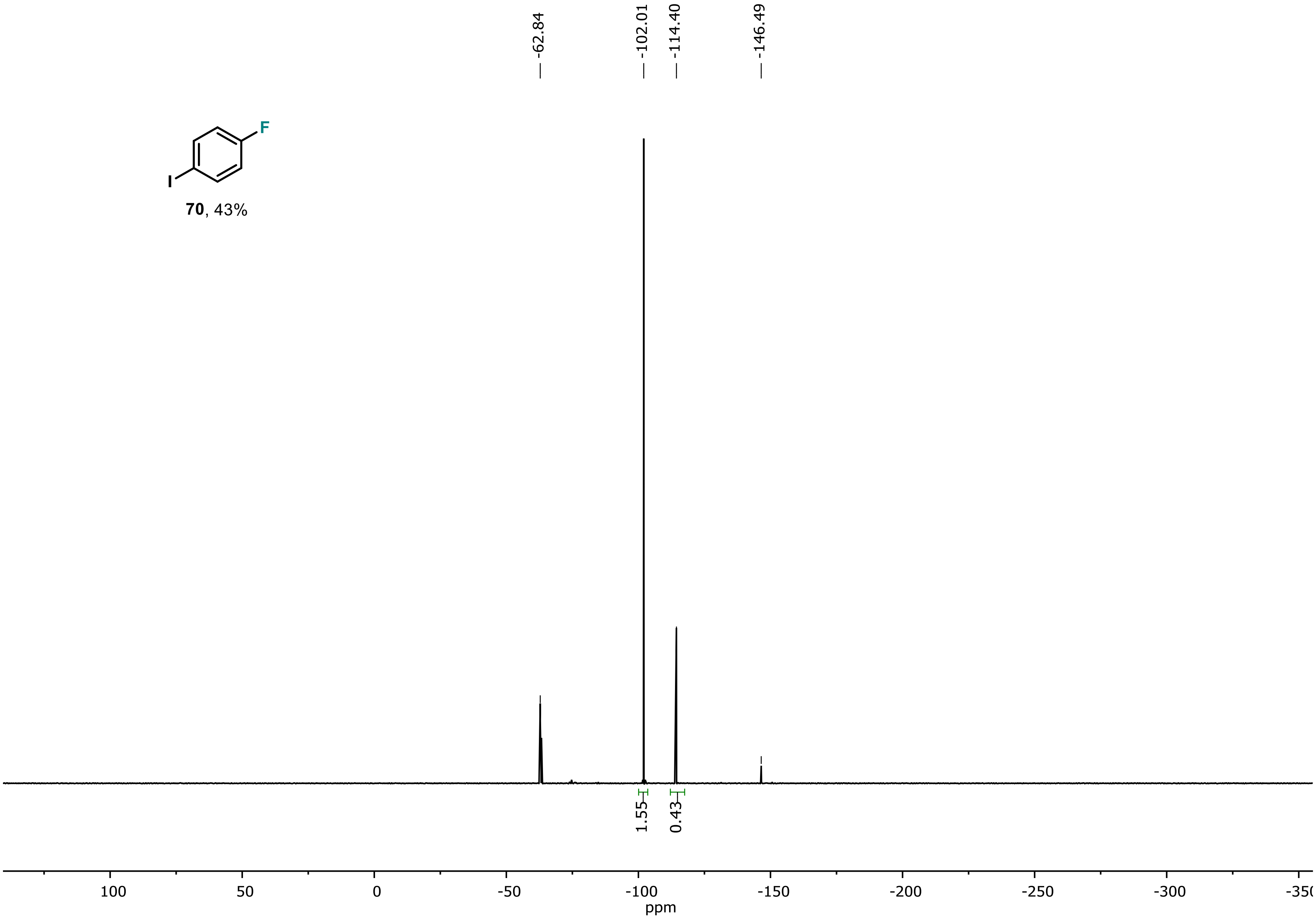
-62.84
-102.01
-114.40
-146.49
1.55
0.43
100
50
0
-50
-100
-150
-200
-250
-300
-350
ppm

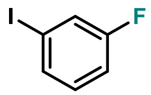

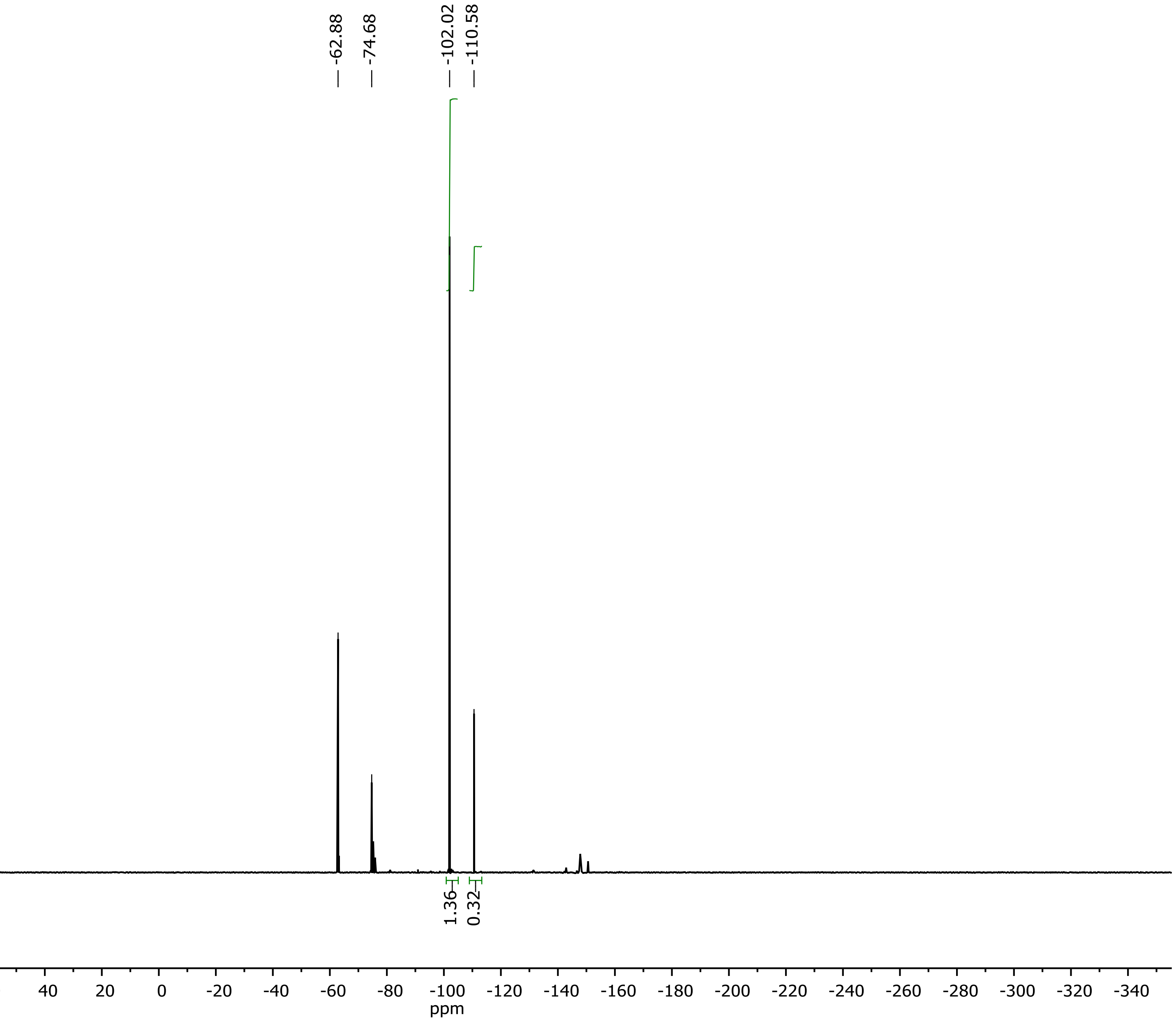

-62.88
-74.68
-102.02
-110.58
1.36
0.32
140
120
100
80
60
40
20
0
-20
-40
-60
-80
-100
-120
-140
-160
-180
-200
-220
-240
-260
-280
-300
-320
-340
ppm

71, 32%

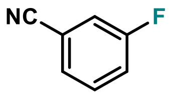

72, 10%

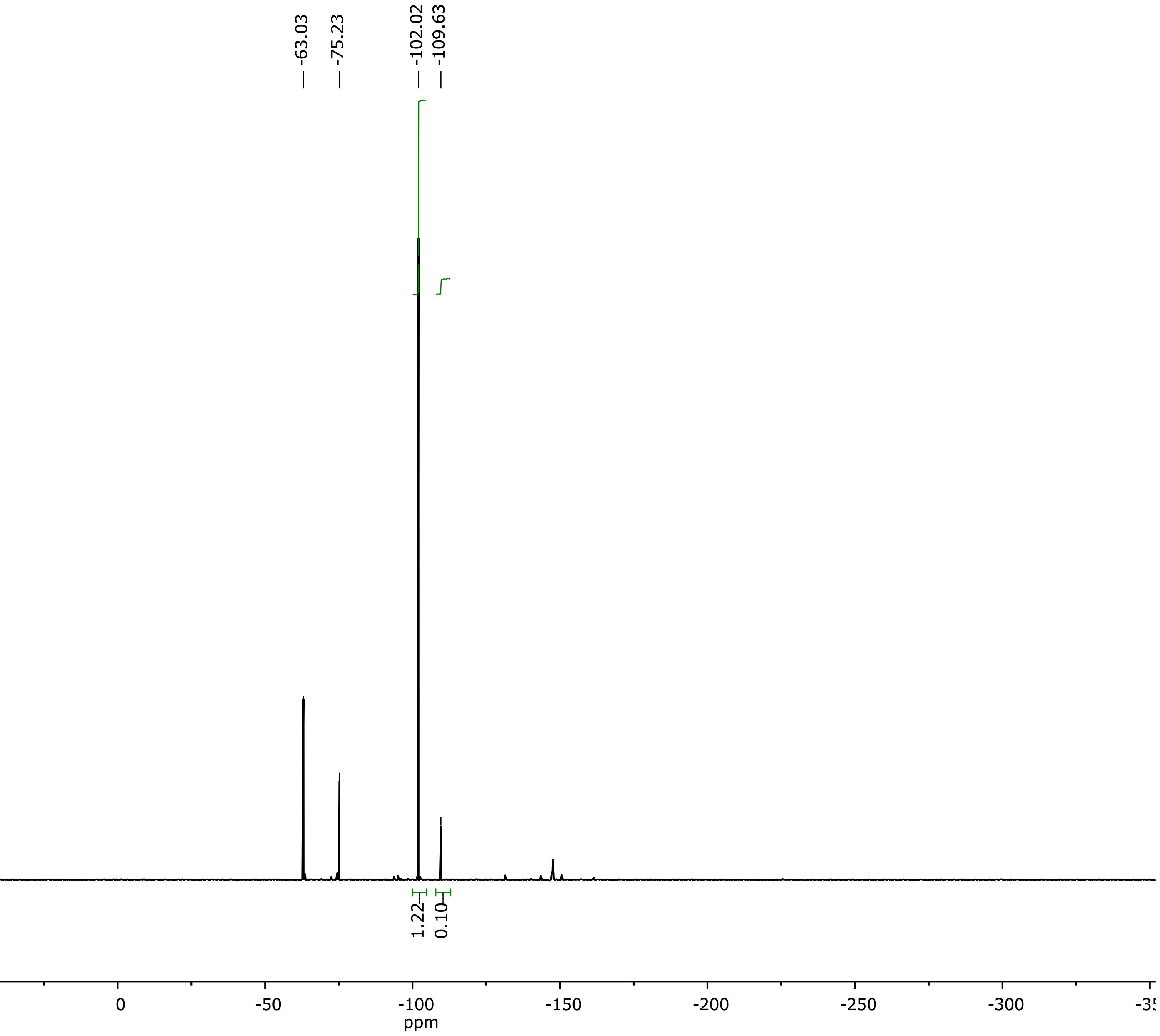

-63.03
-75.23
-102.02
-109.63
1.22
0.10
100
50
0
-50
-100
-150
-200
-250
-300
ppm

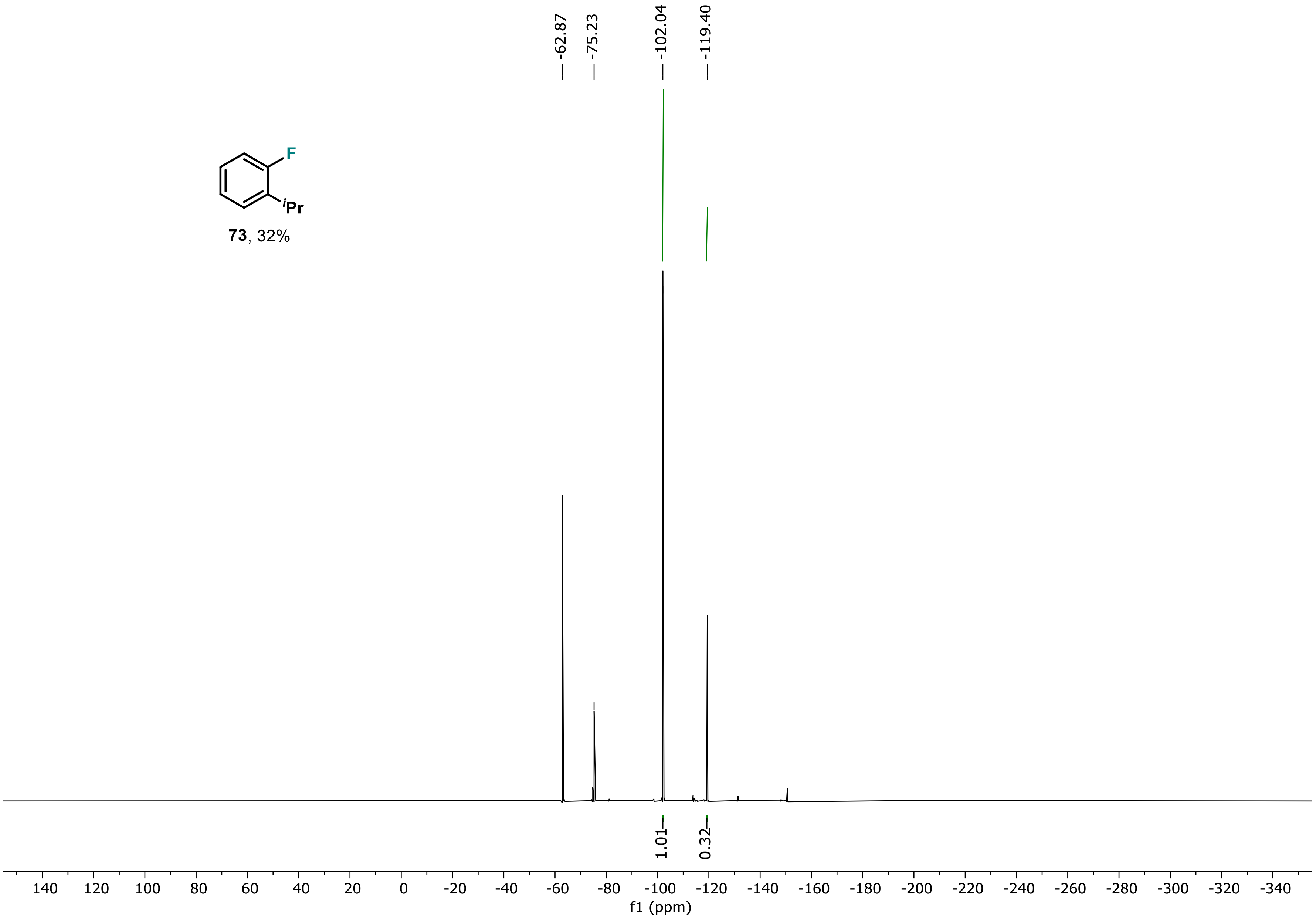
-62.87
-75.23
-102.04
-119.40
F
iPr
73, 32%
1.01
0.32
140 120 100 80 60 40 20 0 -20 -40 -60 -80 -100 -120 -140 -160 -180 -200 -220 -240 -260 -280 -300 -320 -340
f1 (ppm)

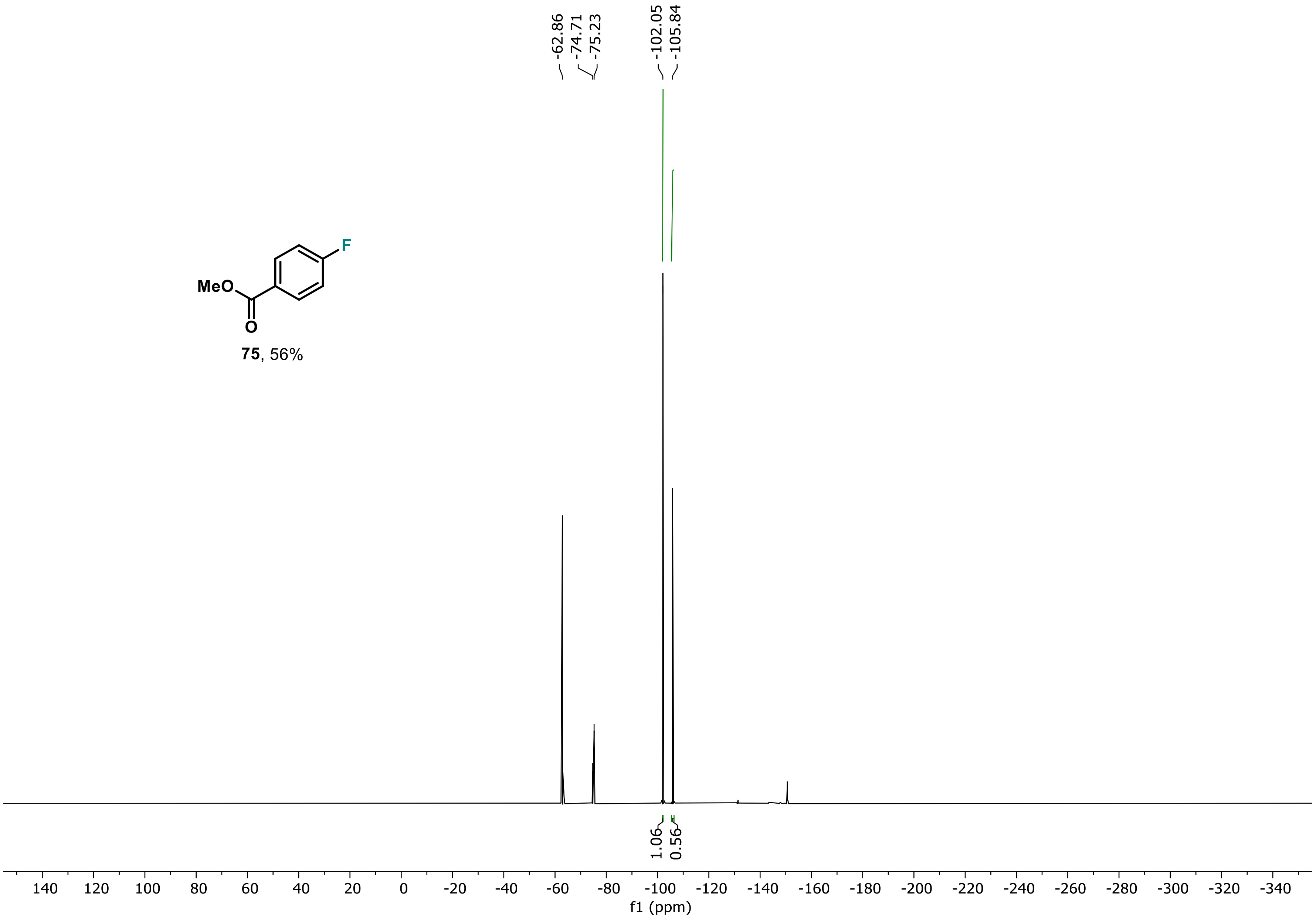

-62.86
-74.71
-75.23
-102.05
-105.84
1.06
0.56
140
120
100
80
60
40
20
0
-20
-40
-60
-80
-100
-120
-140
-160
-180
-200
-220
-240
-260
-280
-300
-320
-340
f1 (ppm)

75, 56%

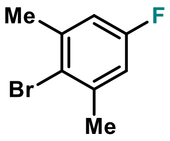

Me
F
Br
Me

76, 69%

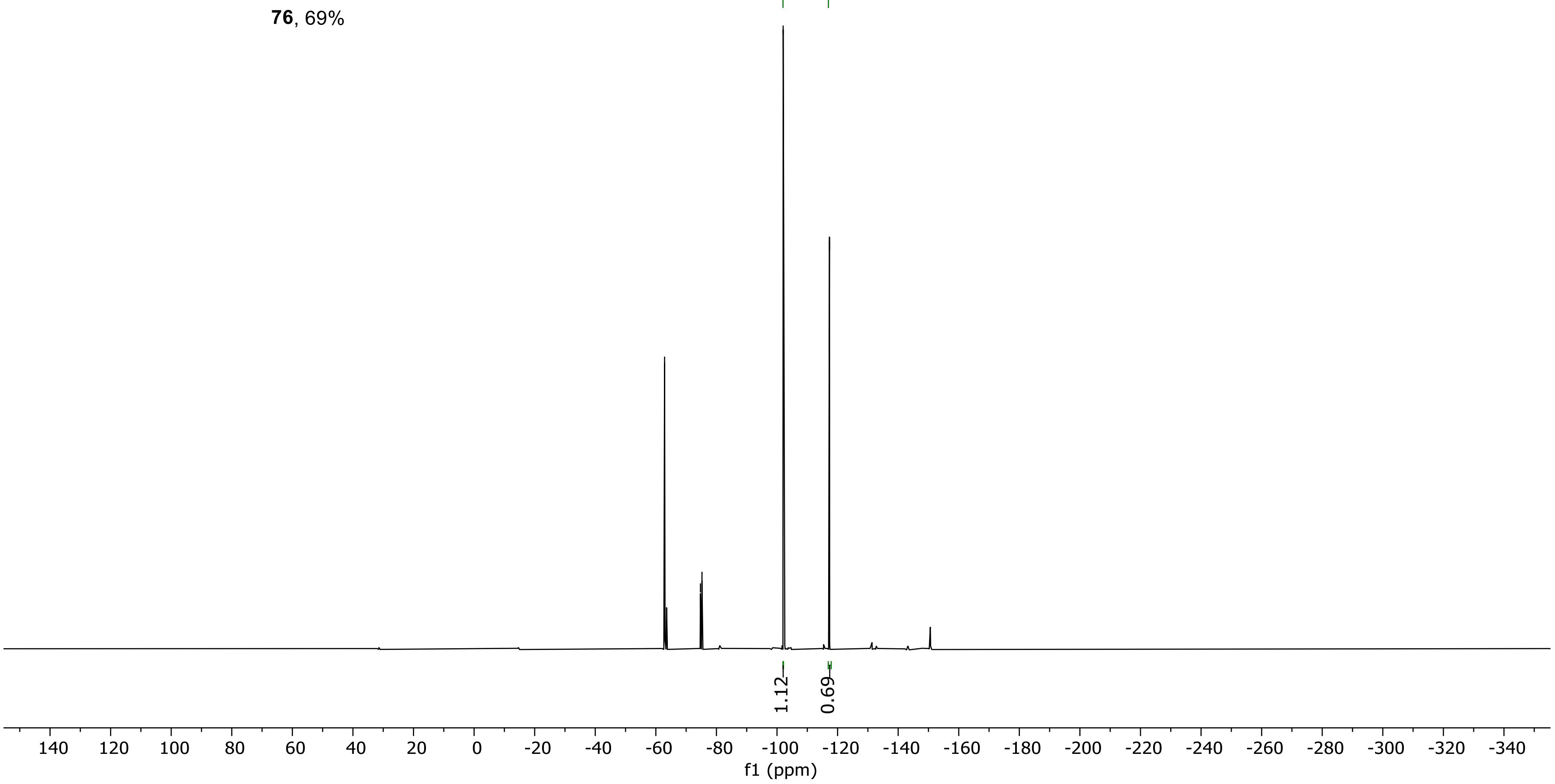

-62.89
-74.73
-75.23
-102.03
-117.32
1.12
0.69
140 120 100 80 60 40 20 0 -20 -40 -60 -80 -100 -120 -140 -160 -180 -200 -220 -240 -260 -280 -300 -320 -340
f1 (ppm)

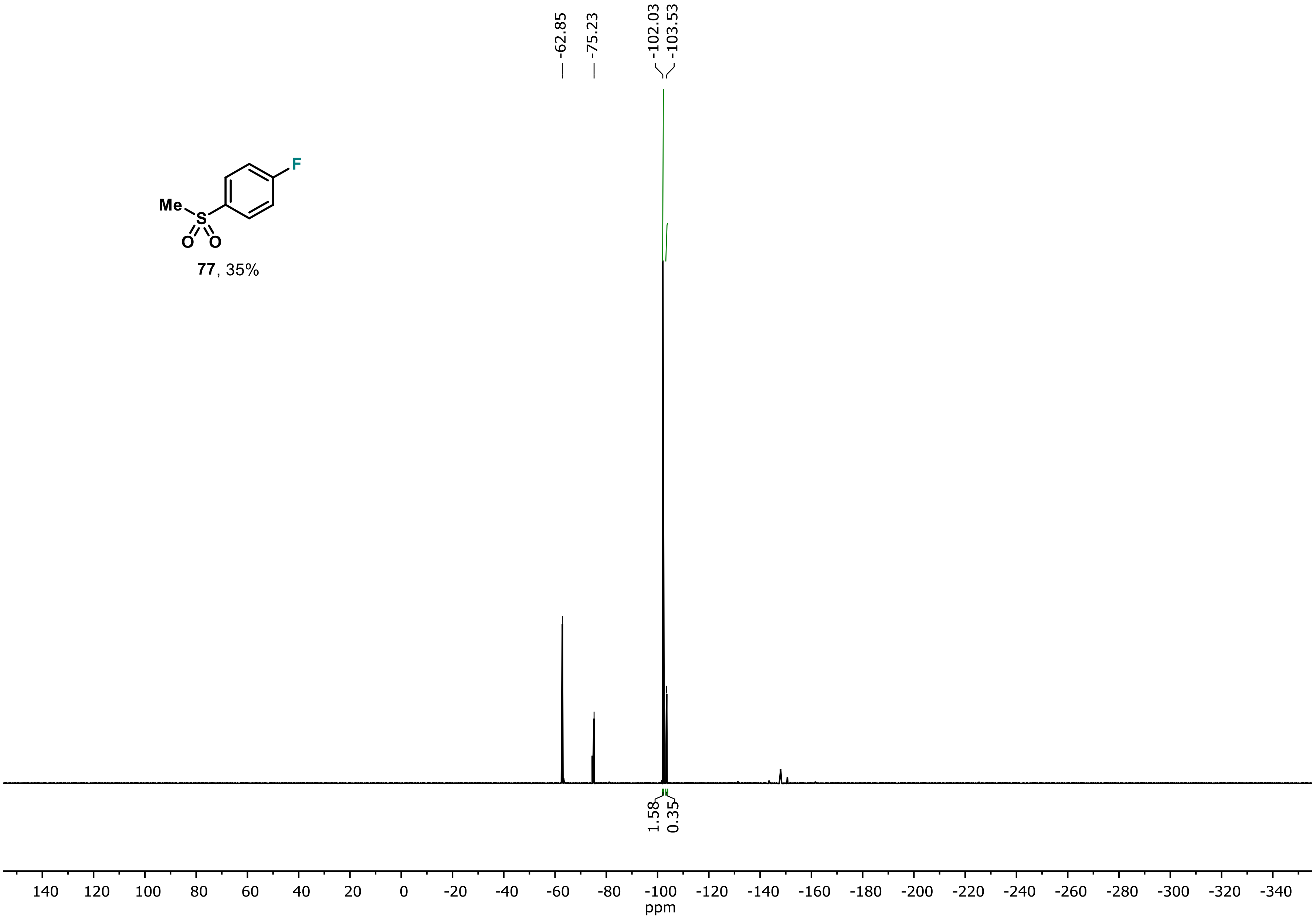
Me
S
O
O
F
-62.85
-75.23
-102.03
-103.53
1.58
0.35
140 120 100 80 60 40 20 0 -20 -40 -60 -80 -100 -120 -140 -160 -180 -200 -220 -240 -260 -280 -300 -320 -340
ppm

77, 35%

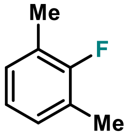

78, 63%

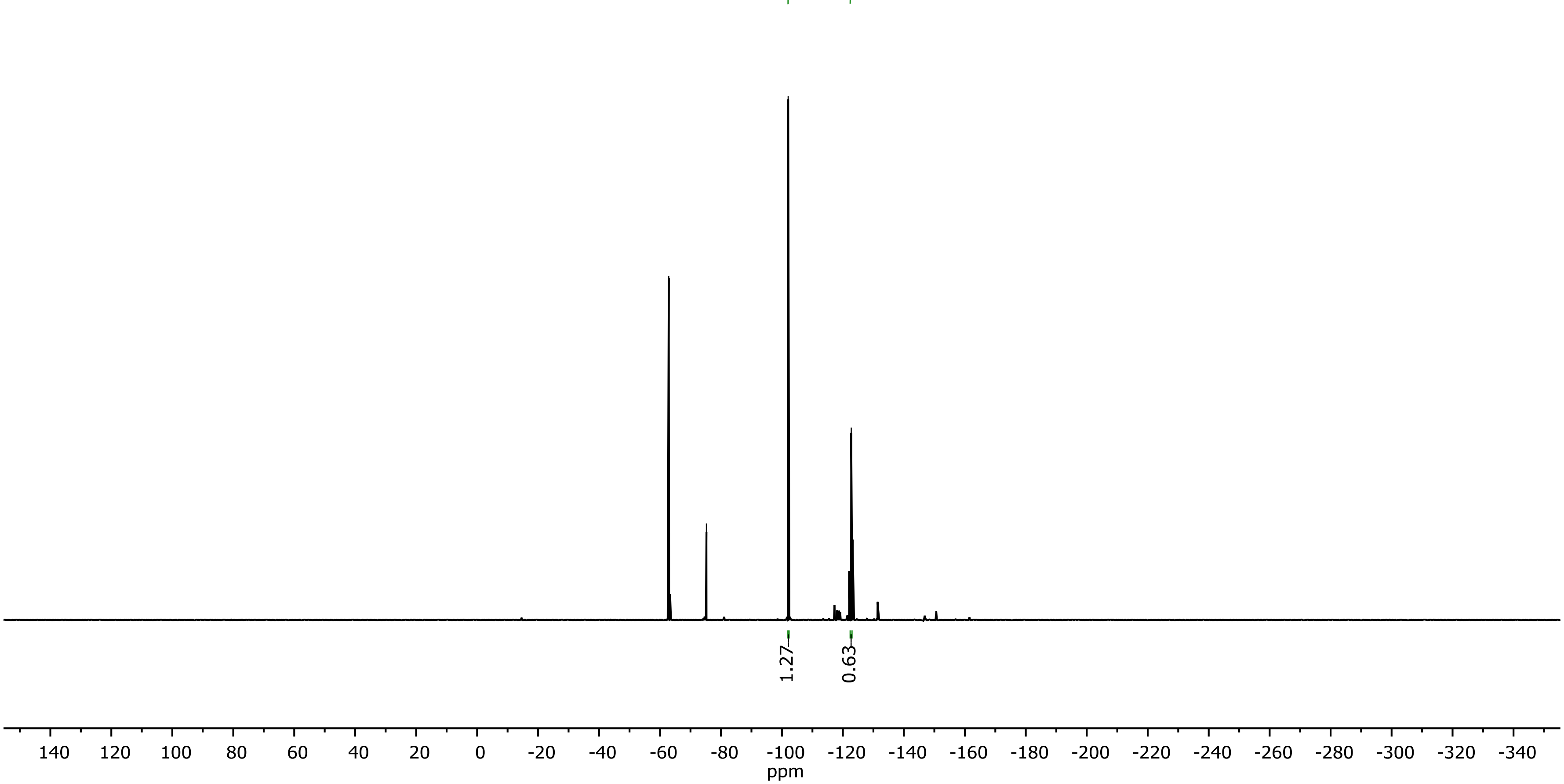

-62.87
-75.23
-102.04
-122.73
1.27
0.63
140
120
100
80
60
40
20
0
-20
-40
-60
-80
-100
-120
-140
-160
-180
-200
-220
-240
-260
-280
-300
-320
-340
ppm

15. NMR spectra for isolated fluoroarenes from catalytic reactions

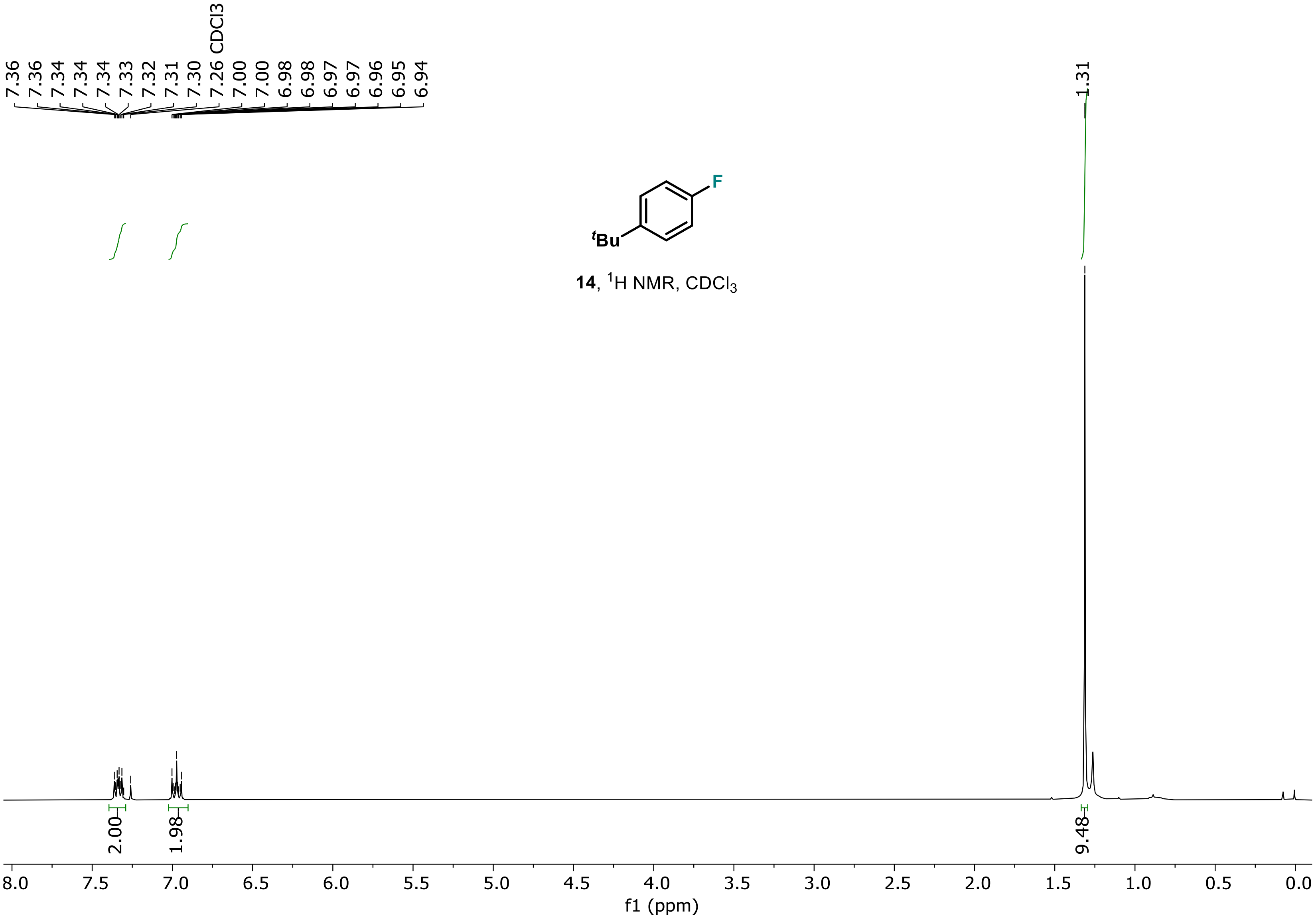

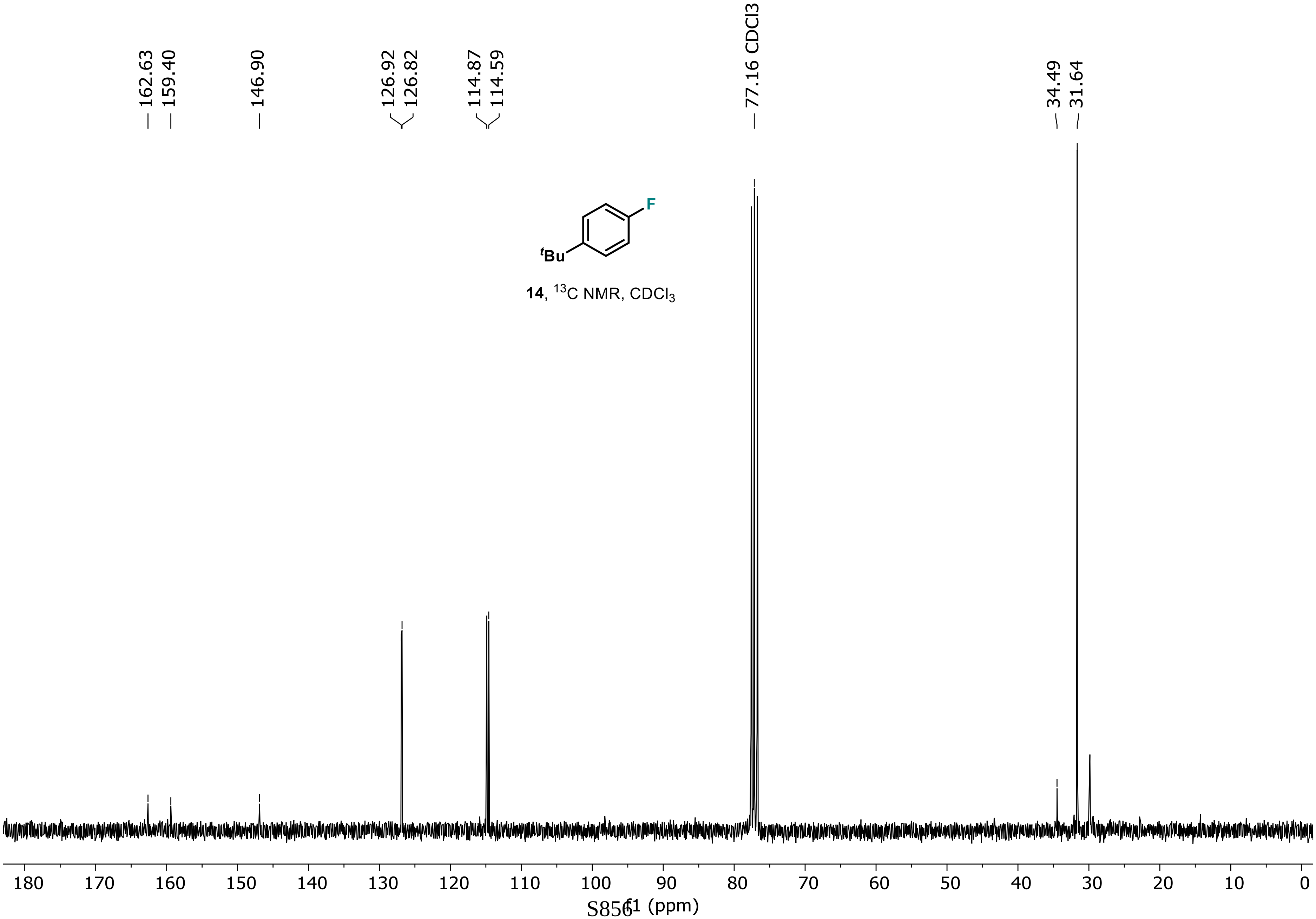

162.63
159.40
146.90
126.92
126.82
114.87
114.59
77.16 CDCl3
34.49
31.64
F
tBu
14, 13C NMR, CDCl3
180 170 160 150 140 130 120 110 100 90 80 70 60 50 40 30 20 10 0
f1 (ppm)

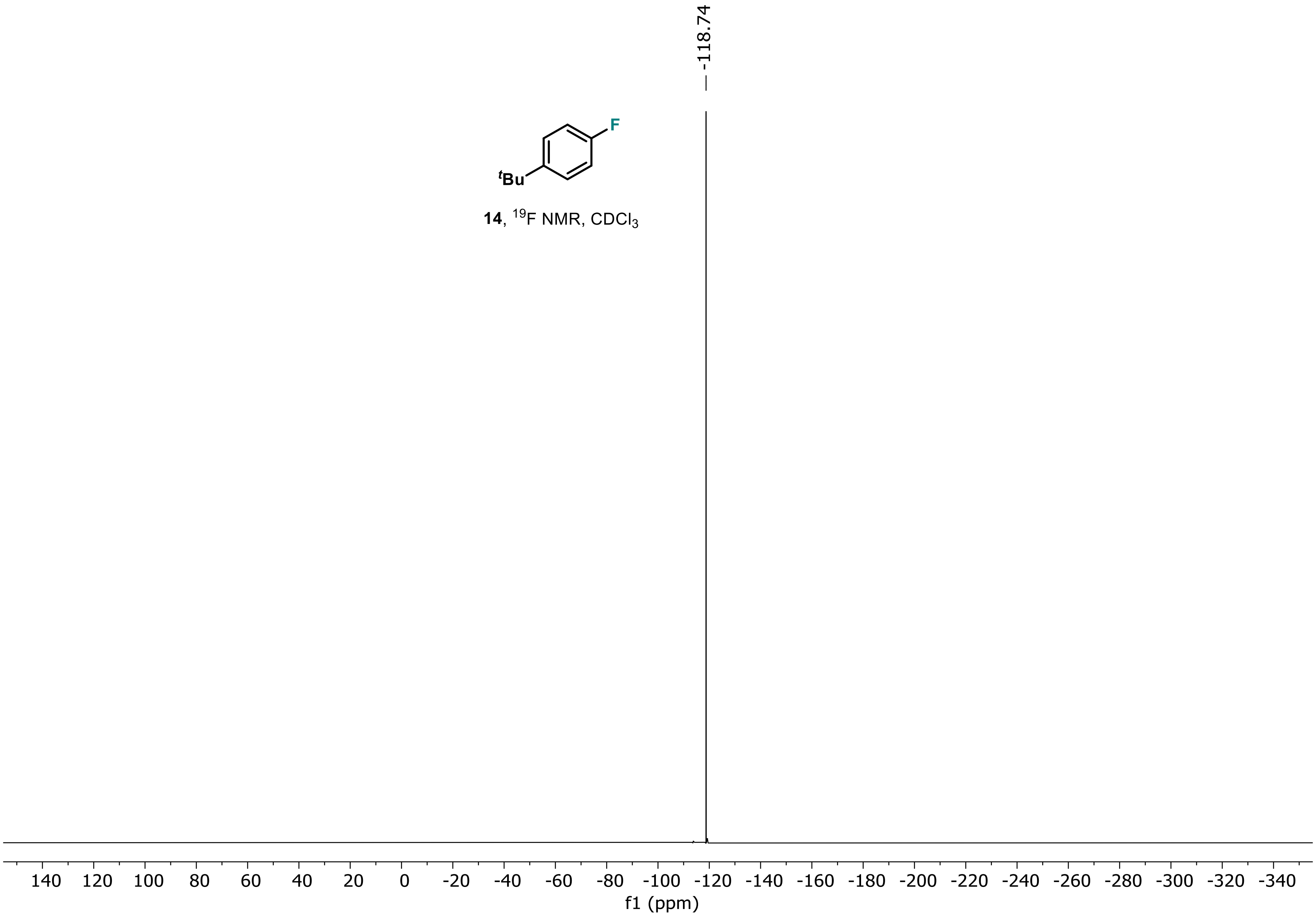

14, ^{19}F NMR, $CDCl_3$

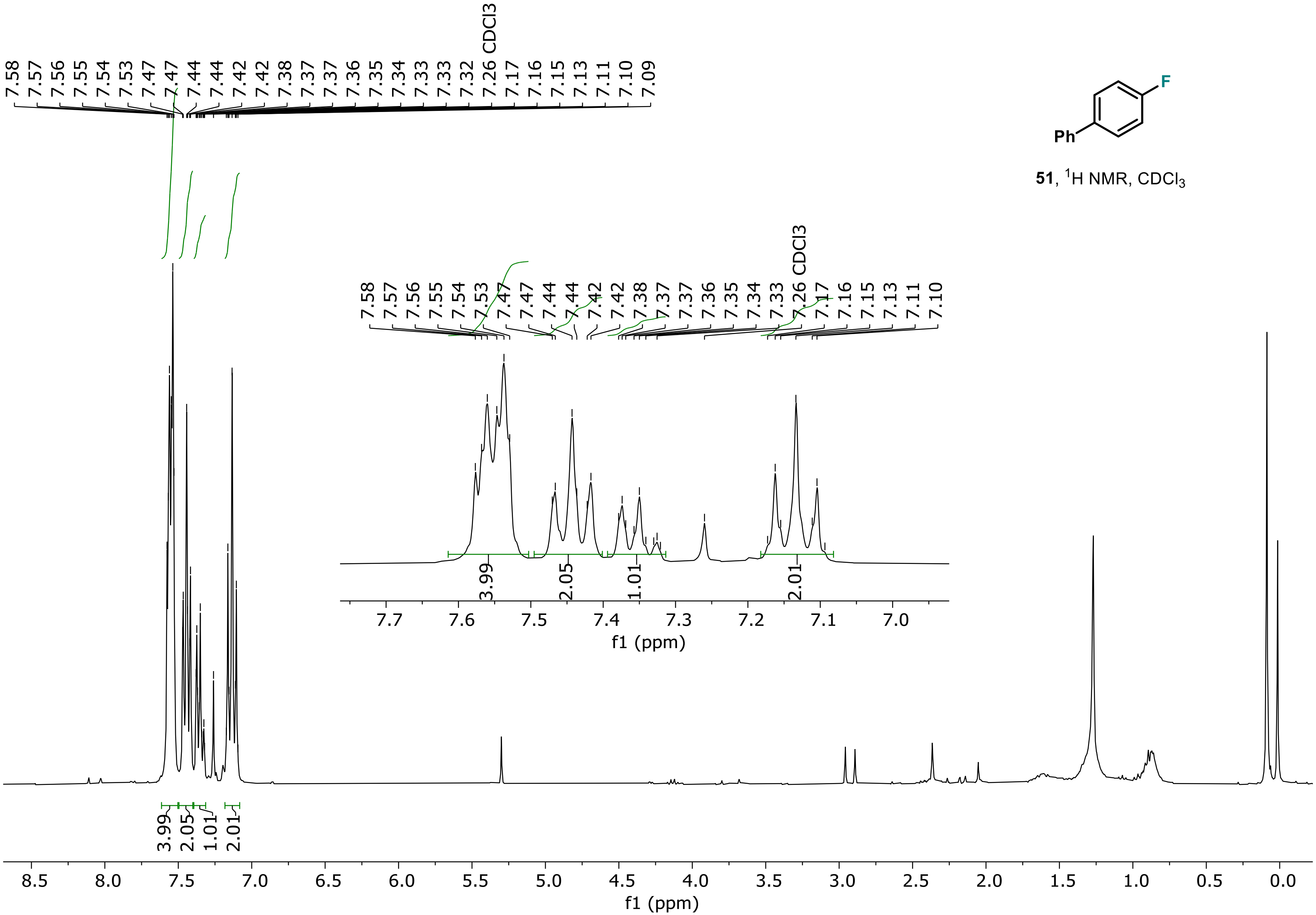

51, ^{1}H NMR, $CDCl_3$

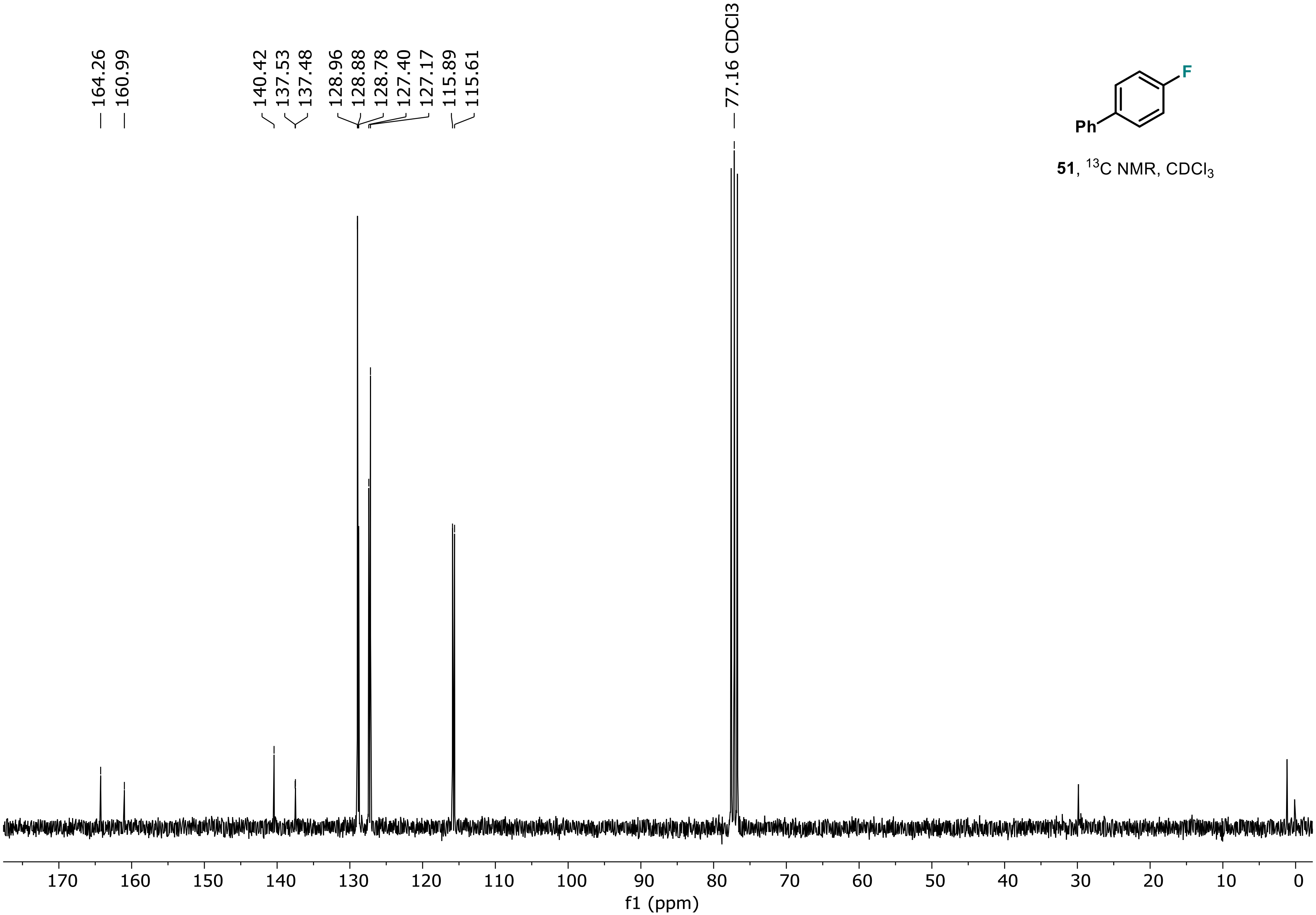

164.26
160.99
140.42
137.53
137.48
128.96
128.88
128.78
127.40
127.17
115.89
115.61
77.16 CDCl3
F
Ph
51, ^{13}C NMR, $CDCl_3$
170
160
150
140
130
120
110
100
90
80
70
60
50
40
30
20
10
0
f1 (ppm)

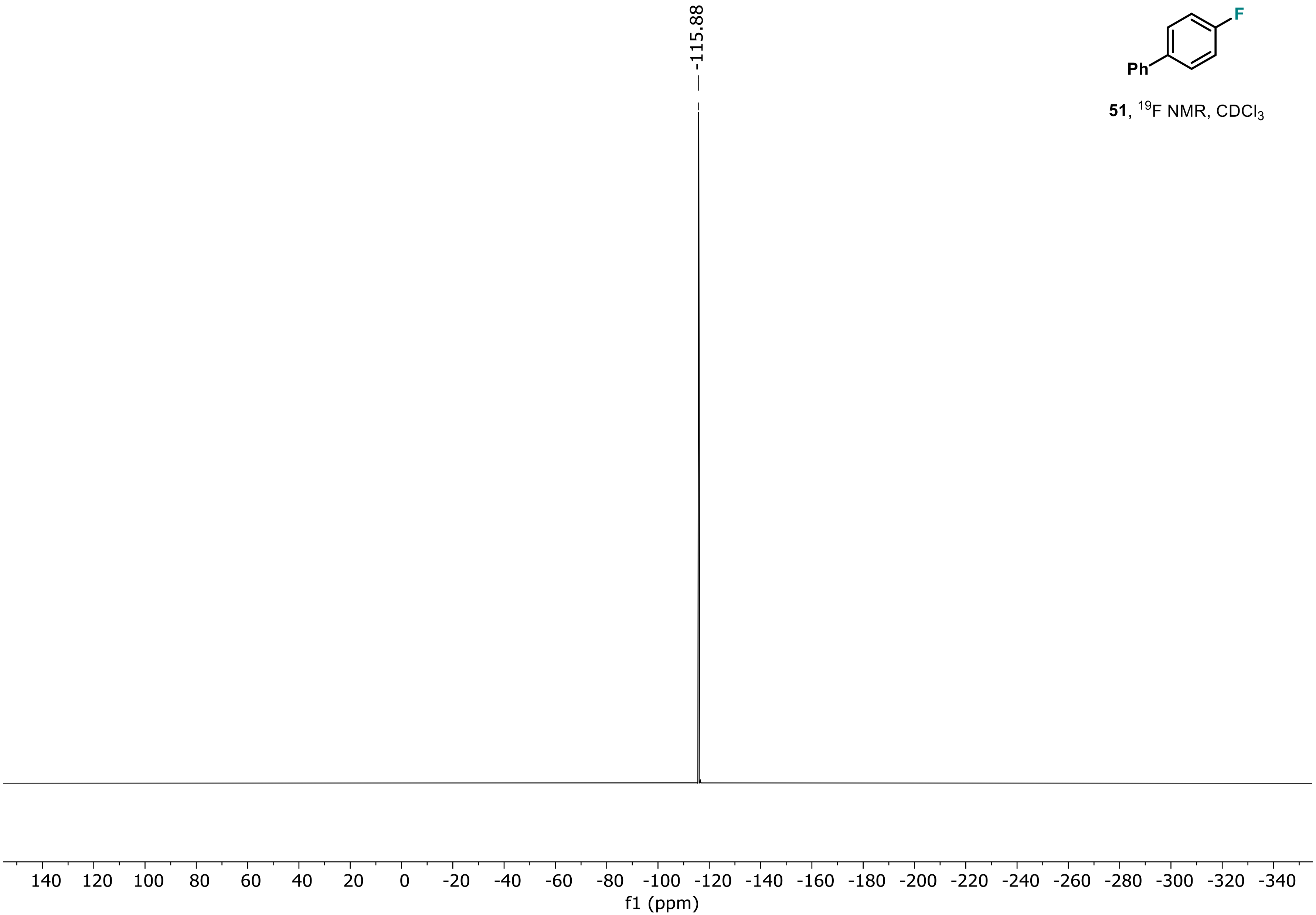

51, ^{19}F NMR, $CDCl_3$

16. References

1. Shen, X.; Ho, D. M.; Pascal, R. A., Synthesis of Polyphenylene Dendrimers Related to “Cubic Graphite”. *J. Am. Chem. Soc.* **2004**, *126*, 5798-5805.
2. Planas, O.; Wang, F.; Leutzsch, M.; Cornella, J., Fluorination of arylboronic esters enabled by bismuth redox catalysis. *Science* **2020**, *367*, 313-317.
3. Planas, O.; Peciukenas, V.; Cornella, J., Bismuth-Catalyzed Oxidative Coupling of Arylboronic Acids with Triflate and Nonaflate Salts. *J. Am. Chem. Soc.* **2020**, *142*, 11382-11387.
4. Rivero-Crespo, M. A.; Toupalas, G.; Morandi, B., Preparation of Recyclable and Versatile Porous Poly(aryl thioether)s by Reversible Pd-Catalyzed C–S/C–S Metathesis. *J. Am. Chem. Soc.* **2021**, *143*, 21331-21339.
5. Yang, Q.; Choy, P. Y.; Zhao, Q.; Leung, M. P.; Chan, H. S.; So, C. M.; Wong, W.-T.; Kwong, F. Y., Palladium-Catalyzed N-Arylation of Sulfoximines with Aryl Sulfonates. *J. Org. Chem.* **2018**, *83*, 11369-11376.
6. Vaddula, B.; Leazer, J.; Varma, R. S., Copper-Catalyzed Ultrasound-Expedited N-Arylation of Sulfoximines using Diaryliodonium Salts. *Adv. Synth. Catal.* **2012**, *354*, 986-990.
7. Gupta, S.; Baranwal, S.; Muniyappan, N.; Sabiah, S.; Kandasamy, J., Copper-Catalyzed N-Arylation of Sulfoximines with Arylboronic Acids under Mild Conditions. *Synthesis* **2019**, *51*, 2171-2182.
8. Teng, F.; Cheng, J.; Bolm, C., Silver-Mediated N-Trifluoromethylation of Sulfoximines. *Org. Lett.* **2015**, *17*, 3166-3169.
9. Sedelmeier, J.; Bolm, C., Efficient Copper-Catalyzed N-Arylation of Sulfoximines with Aryl Iodides and Aryl Bromides. *J. Org. Chem.* **2005**, *70*, 6904-6906.
10. Okauchi, T.; Kuramoto, K.; Kitamura, M., Facile Preparation of Aryl Sulfides Using Palladium Catalysis under Mild Conditions. *Synlett* **2010**, *2010*, 2891-2894.
11. Lamers, P.; Priebbenow, D. L.; Bolm, C., Iron-Catalyzed Acylative Dealkylation ofN-Alkylsulfoximines. *Eur. J. Org. Chem.* **2015**, *2015*, 5594-5602.
12. Fulmer, G. R.; Miller, A. J. M.; Sherden, N. H.; Gottlieb, H. E.; Nudelman, A.; Stoltz, B. M.; Bercaw, J. E.; Goldberg, K. I., NMR Chemical Shifts of Trace Impurities: Common Laboratory Solvents, Organics, and Gases in Deuterated Solvents Relevant to the Organometallic Chemist. *Organometallics* **2010**, *29*, 2176-2179.

13. Worrell, B. T.; Ellery, S. P.; Fokin, V. V., Copper(I)-Catalyzed Cycloaddition of Bismuth(III) Acetylides with Organic Azides: Synthesis of Stable Triazole Anion Equivalents. *Angew. Chem. Int. Ed.* **2013**, *52*, 13037-13041.
14. Jurrat, M.; Maggi, L.; Lewis, W.; Ball, L. T., Modular bismacycles for the selective C–H arylation of phenols and naphthols. *Nat. Chem.* **2020**, *12*, 260-269.
15. Altieri, A. S.; Hinton, D. P.; Byrd, R. A., Association of Biomolecular Systems via Pulsed Field Gradient NMR Self-Diffusion Measurements. *J. Am. Chem. Soc.* **1995**, *117*, 7566-7567.
16. Chen, A.; Wu, D.; Johnson, C. S., Determination of Molecular Weight Distributions for Polymers by Diffusion-Ordered NMR. *J. Am. Chem. Soc.* **1995**, *117*, 7965-7970.
17. Vrentas, J. S.; Duda, J. L., Diffusion in polymer–solvent systems. II. A predictive theory for the dependence of diffusion coefficients on temperature, concentration, and molecular weight. *J. Polym. Sci. Polym. Phys. Ed.* **1977**, *15*, 417-439.
18. Swain, C. G.; Unger, S. H.; Rosenquist, N. R.; Swain, M. S., Substituent effects on chemical reactivity. Improved evaluation of field and resonance components. *J. Am. Chem. Soc.* **1983**, *105*, 492-502.
19. Swain, C. G.; Lupton, E. C., Field and resonance components of substituent effects. *J. Am. Chem. Soc.* **1968**, *90*, 4328-4337.
20. Ehrenson, S.; Brownlee, R. T. C.; Taft, R. W., A Generalized Treatment of Substituent Effects in the Benzene Series. A Statistical Analysis by the Dual Substituent Parameter Equation (1). *Prog. Phys. Org. Chem.* **1973**, *10*, 1-80.
21. Wells, P. R.; Ehrenson, S.; Taft, R. W., Substituent Effects in the Naphthalene Series. An Analysis of Polar and pI Delocalization Effects. *Progr. Phys. Org. Chem.* **1968**, *6*, 147-322.
22. Mann, G.; Baranano, D.; Hartwig, J. F.; Rheingold, A. L.; Guzei, I. A., Carbon−Sulfur Bond-Forming Reductive Elimination Involving sp-, sp2-, and sp3-Hybridized Carbon. Mechanism, Steric Effects, and Electronic Effects on Sulfide Formation. *J. Am. Chem. Soc.* **1998**, *120*, 9205-9219.
23. Jiang, X.-K., Establishment and Successful Application of the $\sigma_{JJ}^{\bullet}$ Scale of Spin-Delocalization Substituent Constants. *Acc. Chem. Res.* **1997**, *30*, 283-289.
24. Ye, Y.; Sanford, M. S., Mild copper-mediated fluorination of aryl stannanes and aryl trifluoroborates. *J. Am. Chem. Soc.* **2013**, *135*, 4648-4651.

25. Dubbaka, S. R.; Narreddula, V. R.; Gadde, S.; Mathew, T., Silver-mediated fluorination of potassium aryltrifluoroborates with Selectfluor®. *Tetrahedron* **2014**, *70*, 9676-9681.
26. Ryan, S. J.; Schimler, S. D.; Bland, D. C.; Sanford, M. S., Acyl Azolium Fluorides for Room Temperature Nucleophilic Aromatic Fluorination of Chloro- and Nitroarenes. *Org. Lett.* **2015**, *17*, 1866-1869.
27. Zou, L.-H.; Johansson, A. J.; Zuidema, E.; Bolm, C., Mechanistic Insights into Copper-Catalyzed Sonogashira–Hagihara-Type Cross-Coupling Reactions: Sub-Mol % Catalyst Loadings and Ligand Effects. *Chem. Eur. J.* **2013**, *19*, 8144-8152.
28. Yang, Y.-M.; Yao, J.-F.; Yan, W.; Luo, Z.; Tang, Z.-Y., Silver-Mediated Trifluoromethoxylation of (Hetero)aryldiazonium Tetrafluoroborates. *Org. Lett.* **2019**, *21*, 8003-8007.
29. Schimler, S. D.; Froese, R. D. J.; Bland, D. C.; Sanford, M. S., Reactions of Arylsulfonate Electrophiles with NMe4F: Mechanistic Insight, Reactivity, and Scope. *J. Org. Chem.* **2018**, *83*, 11178-11190.
30. Milner, P. J.; Kinzel, T.; Zhang, Y.; Buchwald, S. L., Studying Regioisomer Formation in the Pd-Catalyzed Fluorination of Aryl Triflates by Deuterium Labeling. *J. Am. Chem. Soc.* **2014**, *136*, 15757-15766.
31. Möckel, R.; Hille, J.; Winterling, E.; Weidemüller, S.; Faber, T. M.; Hilt, G., Electrochemical Synthesis of Aryl Iodides by Anodic Iododesilylation. *Angew. Chem. Int. Ed.* **2018**, *57*, 442-445.
32. Dorian, A.; Landgreen, E. J.; Petras, H. R.; Shepherd, J. J.; Williams, F. J., Iron-Catalyzed Halogen Exchange of Trifluoromethyl Arenes**. *Chem. Eur. J.* **2021**, *27*, 10839-10843.
33. Coutinho, J. T.; Perfetti, M.; Baldoví, J. J.; Antunes, M. A.; Hallmen, P. P.; Bamberger, H.; Crassee, I.; Orlita, M.; Almeida, M.; van Slageren, J.; Pereira, L. C. J., Spectroscopic Determination of the Electronic Structure of a Uranium Single-Ion Magnet. *Chem. Eur. J.* **2019**, *25*, 1758-1766.
34. Ye, Y.; Sanford, M. S., Mild Copper-Mediated Fluorination of Aryl Stannanes and Aryl Trifluoroborates. *J. Am. Chem. Soc.* **2013**, *135*, 4648-4651.
35. Furuya, T.; Kaiser, H. M.; Ritter, T., Palladium-Mediated Fluorination of Arylboronic Acids. *Angew. Chem. Int. Ed.* **2008**, *47*, 5993-5996.

36. Iakobson, G.; Du, J.; Slawin, A. M. Z.; Beier, P., Pyridine-promoted dediazoniation of aryldiazonium tetrafluoroborates: Application to the synthesis of SF5-substituted phenylboronic esters and iodobenzenes. *Beilstein J. Org. Chem.* **2015**, *11*, 1494-1502.
37. Chénard, E.; Sutrisno, A.; Zhu, L.; Assary, R. S.; Kowalski, J. A.; Barton, J. L.; Bertke, J. A.; Gray, D. L.; Brushett, F. R.; Curtiss, L. A.; Moore, J. S., Synthesis of Pyridine– and Pyrazine–BF3 Complexes and Their Characterization in Solution and Solid State. *J. Phys. Chem. C* **2016**, *120*, 8461-8471.
38. Harper, M. J.; Emmett, E. J.; Bower, J. F.; Russell, C. A., Oxidative 1,2-Difunctionalization of Ethylene via Gold-Catalyzed Oxyarylation. *J. Am. Chem. Soc.* **2017**, *139*, 12386-12389.
39. Sather, A. C.; Lee, H. G.; De La Rosa, V. Y.; Yang, Y.; Muller, P.; Buchwald, S. L., A Fluorinated Ligand Enables Room-Temperature and Regioselective Pd-Catalyzed Fluorination of Aryl Triflates and Bromides. *J. Am. Chem. Soc.* **2015**, *137*, 13433-13438.
40. Scharf, A.; Goldberg, I.; Vigalok, A., Evidence for Metal–Ligand Cooperation in a Pd–PNF Pincer-Catalyzed Cross-Coupling. *J. Am. Chem. Soc.* **2013**, *135*, 967-970.
41. Konovalov, A. I.; Gorbacheva, E. O.; Miloserdov, F. M.; Grushin, V. V., Ruthenium-catalyzed nucleophilic fluorination of halobenzenes. *Chem. Commun.* **2015**, *51*, 13527-13530.
42. Furuya, T.; Kaiser, H. M.; Ritter, T., Palladium-Mediated Fluorination of Arylboronic Acids. *Angew. Chem. Int. Ed.* **2008**, *47*, 5993-5996.
43. Liu, W.; Yang, X.; Gao, Y.; Li, C.-J., Simple and Efficient Generation of Aryl Radicals from Aryl Triflates: Synthesis of Aryl Boronates and Aryl Iodides at Room Temperature. *J. Am. Chem. Soc.* **2017**, *139*, 8621-8627.
44. Mohy El Dine, T.; Sadek, O.; Gras, E.; Perrin, D. M., Expanding the Balz–Schiemann Reaction: Organotrifluoroborates Serve as Competent Sources of Fluoride Ion for Fluoro-Dediazoniation. *Chem. Eur. J.* **2018**, *24*, 14933-14937.
45. Xu, J.-D.; Su, X.-B.; Wang, C.; Yao, L.-W.; Liu, J.-H.; Hu, G.-Q., Mild Copper-Catalyzed Addition of Arylboronic Esters to Di-tert-butyl Dicarbonate: An Easy Access to Methyl Arylcarboxylates. *Synlett* **2021**, *32*, 833-837.
46. Romanato, P.; Duttwyler, S.; Linden, A.; Baldridge, K. K.; Siegel, J. S., Competition between π-Arene and Lone-Pair Halogen Coordination of Silylium Ions? *J. Am. Chem. Soc.* **2011**, *133*, 11844-11846.

47. Tang, P.; Furuya, T.; Ritter, T., Silver-catalyzed late-stage fluorination. *J. Am. Chem. Soc.* **2010**, *132*, 12150-12154.
48. Neese, F., The ORCA program system. *Wiley Interdiscip. Rev. Comput. Mol. Sci.* **2011**, *2*, 73-78.
49. Neese, F., Software update: the ORCA program system, version 4.0. *Wiley Interdiscip. Rev. Comput. Mol. Sci.* **2017**, *8*, e1327.
50. Becke, A. D., Density-functional thermochemistry. III. The role of exact exchange. *J. Chem. Phys.* **1993**, *98*, 5648-5652.
51. Lee, C.; Yang, W.; Parr, R. G., Development of the Colle-Salvetti correlation-energy formula into a functional of the electron density. *Phys. Rev. B* **1988**, *37*, 785-789.
52. Becke, A. D.; Johnson, E. R., A density-functional model of the dispersion interaction. *J. Chem. Phys.* **2005**, *123*, 154101.
53. Weigend, F.; Ahlrichs, R., Balanced basis sets of split valence, triple zeta valence and quadruple zeta valence quality for H to Rn: Design and assessment of accuracy. *Phys. Chem. Chem. Phys.* **2005**, *7*, 3297-305.
54. Weigend, F., Accurate Coulomb-fitting basis sets for H to Rn. *Phys. Chem. Chem. Phys.* **2006**, *8*, 1057-1065.
55. Metz, B.; Stoll, H.; Dolg, M., Small-core multiconfiguration-Dirac–Hartree–Fock-adjusted pseudopotentials for post-d main group elements: Application to PbH and PbO. *J. Chem. Phys.* **2000**, *113*, 2563-2569.
56. Neese, F.; Wennmohs, F.; Hansen, A.; Becker, U., Efficient, approximate and parallel Hartree–Fock and hybrid DFT calculations. A 'chain-of-spheres' algorithm for the Hartree–Fock exchange. *Chem. Phys.* **2009**, *356*, 98-109.
57. Reed, A. E.; Curtiss, L. A.; Weinhold, F., Intermolecular interactions from a natural bond orbital, donor-acceptor viewpoint. *Chem. Rev.* **2002**, *88*, 899-926.
58. *Chemcraft Version 1.8, h. w. c. c.* .
